# Net-Net Auto Machine Learning (AutoML) Prediction of Complex Ecosystems

**DOI:** 10.1038/s41598-018-30637-w

**Published:** 2018-08-17

**Authors:** Enrique Barreiro, Cristian R. Munteanu, Maykel Cruz-Monteagudo, Alejandro Pazos, Humbert González-Díaz

**Affiliations:** 1 Department of Computation, Computer Science Faculty, University of A Coruna (UDC), 15071 A Coruña, Spain; 2 Center for Computational Science (CCS), University of Miami (UM), Miami, 33136 FL USA; 3 West Coast University, Miami Campus, 33178 FL USA; 4 Biomedical Research Institute of A Coruña (INIBIC), University Hospital Complex of A Coruña (CHUAC), A Coruña, 15006 Spain; 5 Faculty of Science and Technology, University of the Basque Country (UPV/EHU), 48940 Biscay, Spain; 6 IKERBASQUE, Basque Foundation for Science, 48011 Bilbao, Biscay Spain

## Abstract

Biological Ecosystem Networks (BENs) are webs of biological species (nodes) establishing trophic relationships (links). Experimental confirmation of all possible links is difficult and generates a huge volume of information. Consequently, computational prediction becomes an important goal. Artificial Neural Networks (ANNs) are Machine Learning (ML) algorithms that may be used to predict BENs, using as input Shannon entropy information measures (Sh_k_) of known ecosystems to train them. However, it is difficult to select *a priori* which ANN topology will have a higher accuracy. Interestingly, Auto Machine Learning (AutoML) methods focus on the automatic selection of the more efficient ML algorithms for specific problems. In this work, a preliminary study of a new approach to AutoML selection of ANNs is proposed for the prediction of BENs. We call it the Net-Net AutoML approach, because it uses for the first time Sh_k_ values of both networks involving BENs (networks to be predicted) and ANN topologies (networks to be tested). Twelve types of classifiers have been tested for the Net-Net model including linear, Bayesian, trees-based methods, multilayer perceptrons and deep neuronal networks. The best Net-Net AutoML model for 338,050 outputs of 10 ANN topologies for links of 69 BENs was obtained with a deep fully connected neuronal network, characterized by a test accuracy of 0.866 and a test AUROC of 0.935. This work paves the way for the application of Net-Net AutoML to other systems or ML algorithms.

## Introduction

Many important molecular, living, economical, and other complex systems may be described as complex networks of *i* parts or nodes interconnected by links, edges, bonds, ties, or relationships^1–7^. The volume of information about all these collections of nodes and links is so large that it is impossible for a single person to remember and rationalize all possible connections in known networks. Consequently, it is even more difficult to assign/predict correct connections in new cases. This problem can be solved using Machine Learning (ML) models. In this area, ML models used as input variables are able to quantify structural information of the system. The process has been applied to multiple levels, ranging from the prediction of drug-target networks in molecules to the construction of complex biological networks^8–11^.

Specifically, a molecular or living complex system can be explained using numerical parameters that quantify information about the structure of the system. In information theory, Shannon entropy quantifies the information contained in a message, usually in bits. The concept was introduced by Claude E. Shannon in his 1948 paper “A Mathematical Theory of Communication”^12^. With the pass of time, the Shannon entropy information measures (Sh_k_) of different types and other related information measures have become commonly used indices in quantifying information of the system under study in ML modelling^13–29^.

In any case, developing ML models using as input Sh_k_ values involves, as in other ML problems, the application of data pre-processing variable selection and other techniques. Next, it is necessary to *a priori* select one or more ML algorithms and train/validate them to seek the final ML model. Consequently, non-experts in ML may encounter difficulties to accomplish this goal. Specifically, in the case of complex molecular and living systems, a non-expert may find it difficult to decide a priori which ML algorithms should be selected to develop the model. In this context, Automated Machine Learning (AutoML) may have an important role in automatically selecting ML algorithms during the development of practical ML applications by non-experts^30,31^.

This work proposes for the first time the use of Sh_k_ values to quantify both the structure of the complex biological system to be predicted and the structure of the ML algorithm to be selected for this task. To this end, a preliminary proof-of-concept experiment is carried out, focusing on a specific class of complex biological systems, and a specific type of ML algorithms. Biological Ecosystem Networks (BENs) have been selected to play the role of a complex biological system.

In addition, Artificial Neural Networks (ANN) have been selected to represent the ML algorithms. The current study uses the entropy values Sh_k_(A_i_) and Sh_k_(B_j_) as inputs for different pairs of species in the BENs, of the systems under study. The Sh_k_(ANN_j_) values calculated are also used as inputs for different ANN topologies. In fact, Ecosystems represent one of the most important examples of complex systems. They are a clear example of network-like structures with known procedures to calculate the Sh_k_ values^32–34^. In this sense, our group reported different ML models that evaluate the structure of parasite-host webs to predict the interactions between species in different networks^35–37^. In one of our previous works, special emphasis has been placed on the use of Sh_k_ information measures to codify structural information in this type of ML studies^38^. On the other hand, ANNs have been selected because of their more apparent network-like structure, and because they are a useful tool to solve this kind of ML problems. In fact, ANNs are powerful bio-inspired algorithms able to learn/infer large datasets^39–42^. ANNs are also able to learn topology patterns in large datasets of bio-systems and other complex networks^43^. This work proposes the calculation of Sh_k_ information indices in both sets of networks: BENs and ANNs. That is why, it has been called a Net-Net AutoML approach. Last, an AutoML linear model is sought using these indices as input. This Net-Net AutoML model could be employed to screen different ANN topologies in order to pre-select the one expected to correctly predict BEN structures before training it.

## Results

**Figure 1:**
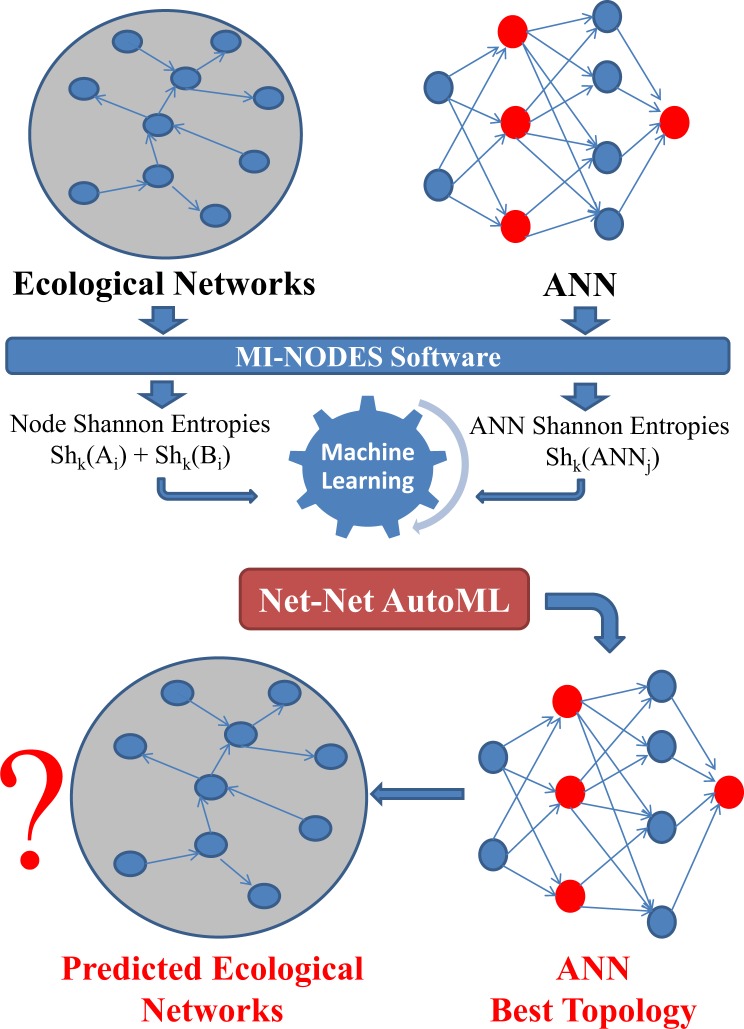
General workflow of the Net-Net AutoML methodology.

This work introduces for the first time a new type of algorithm to find the best ANNs that predict BENs. The main steps of the methodology are described in Fig. 1. This is the first report of a Net-Net AutoML model for ANN screening, with the subsequent saving of time and computational resources in the prediction of Complex Networks. The BEN node pairs and the ANN classifiers that were trained for the prediction of BEN node connectivity were turned into Sh_k_ descriptors that encoded information for the BEN nodes and the entire ANN topology. Sh_k_ were calculated for each node with the MI-NODES software^44^. In the case of ANN classifiers, the average of all the values of Sh_k_ for all the neurons in the ANN was used as input. For the MI-NODES descriptors, the Markov chains theory was applied and, therefore, they were calculated for each *k* values ranging between 0 and 5 (*k* = node distance of interaction) as Sh_k_^45^. These descriptors were linearly combined to find a model (AutoML) that was able to predict how a specific ANN topology would evaluate BEN node connectivity. Thus, AutoML could be used to screen which is the best ANN classifier topology for BEN node connectivity prediction. The AutoML methodology used for the prediction of BEN connectivity includes the following steps with their respective results.

First, Sh_k_ values were calculated for a large number of nodes in 69 BENs using the MI-NODES software. We created a dataset of biological systems (bsi dataset) using 33,805 pairs of nodes selected randomly from the 69 BENs. If we consider the adjacency matrix (**A**) as the mathematical representation of all pairs of A*i* vs. B*j* nodes in the BEN, the output variable of this dataset are the elements A_ij_ of this matrix. These values quantify the structure (connectivity) of the BEN with values A_ij_ = 1 for the pairs of nodes that are connected (interacting biological species) and A_ij_ = 0 otherwise (non-interacting biological species).

Next, the bsi dataset was expanded with node differences as input variables ∆Sh_kij_ = Sh_ki_ − Sh_kj_ for each pair, where Sh_ki_ is the Sh_k_ for the first node and Sh_kj_ the Sh_k_ for the second node (k = 0–5). As a result, there are ^bsi^N_var_ = 6·3 = 18 input features for the A_ij_ output for each pair of BEN nodes. The variables Sh_0_ quantify information for an isolated node, Sh_1_ refers to the nodes with direct link, Sh_2_ to nodes that have other nodes between them, and so on.

**Figure 2:**
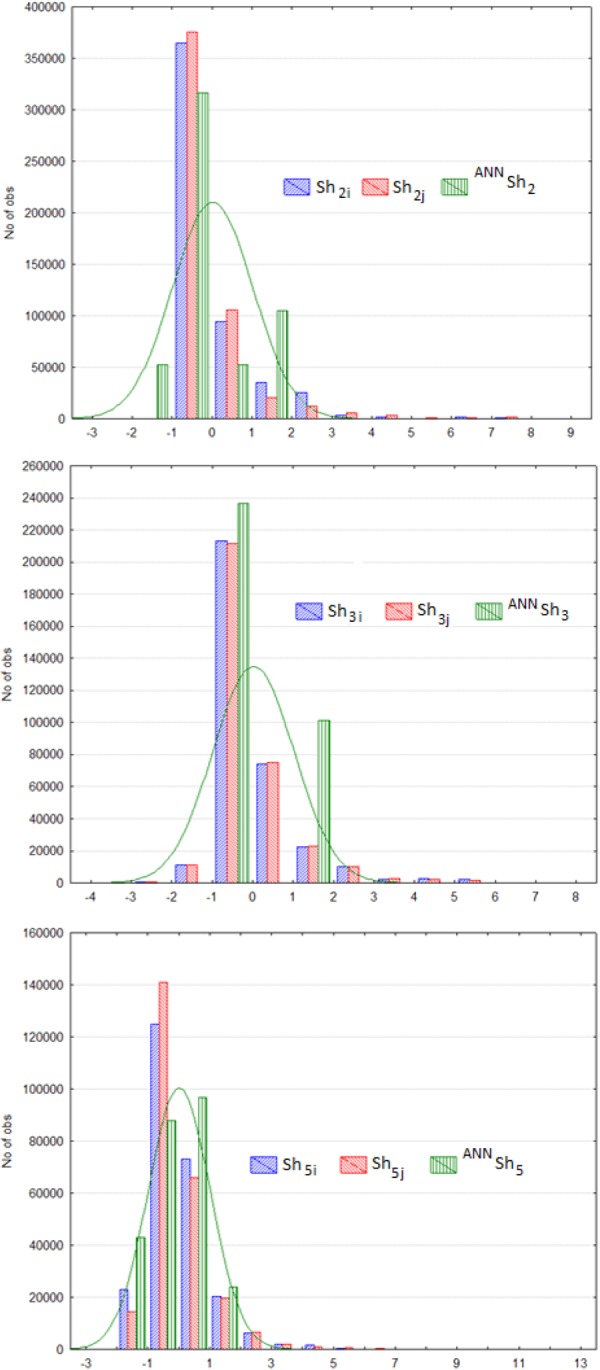
Distribution of Sh_k_ values (k = 2, 3, 5) for ANNs *vs*. BENs nodes.

Figure 2 illustrates the distribution of three Sh_k_ parameters (k = 2, 3, 5) for both BENs and ANN classifiers to predict them. This dataset was used to train 10 different ANNs. Next, the ANN screening model testing dataset (mt dataset) was made up. The output variable of the mt dataset represents the values of correct or incurred prediction of BEN connectivity A_ij_ by a specific ANN classifier topology, P(^ANN^A_ij_) = 1 when the ANN topology correctly predicts the observed BEN nodes connectivity A_ij_ (A_ij_ = 1 or 0 in the original bsi dataset). On the contrary, P(^ANN^A_ij_) = 0 when a specific ANN topology fails to correctly classify the observed A_ij_ of 1 or 0 from the original bsi dataset. The mt dataset contains the predictions of 338,050 node pairs from 69 BENs using 10 different trained ANN classifiers (different topologies). The input variables of the mt dataset are the original variables for each pair of nodes and the values of information indices ^ANN^Sh_k_ (average value of Sh of all ANN neurons): ^mt^N_var_ = 6*(Sh_ki_ + Sh_kj_ + ∆Sh_kij_ + ^ANN^Sh_k_) = 24. The last step consists of the dataset analysis to find the best linear AutoML model for ANN classifier screening (see previous Fig. 1).

## Discussion

There are at least two major problems if ANNs are used to predict node connectivity in complex networks. First, the information in complex systems should be turned into numerical input parameters to the future ANN classifiers for node connectivity. Secondly, many ANN classifiers with different topologies should be trained in order to find the best ANN topology that can learn the complex system structure patterns. The first problem can be solved by quantifying the structural information of the complex system (Brain, Ecological, Social, etc.) with Sh_k_ information measures^46^. The classical solution for the second problem is the training of different ANNs to find the best topology. This step involves the use of High Performance Computing (HPC) services if the aim is to test a high number of ANNs for many complex systems.

The current study proposes a new methodology to evaluate how a new ANN classifier could predict the BEN node connectivity, without the need for ANN training. Thus, two types of information descriptors were used: node descriptors for BEN complex networks and the average of ANN neuron descriptors. If ANNs are networks with nodes (neurons) and links (weights), the same mathematical processing as in the case of the BEN complex network could be applied. Therefore, it is possible to quantify topological (connectivity) information of both the BEN complex networks under study and a set of ANNs trained using Sh_k_ descriptors. Thus, each node of the complex networks encoded information into Sh_k_ descriptors and each ANN classifier was characterized as an entire network by the average of Sh_k_.

The new AutoML methodology proposed a screening of ANN classifier topologies for BEN node connectivity prediction. The current work applied the AutoML methodology to the Ecological systems. Consequently, the AutoML output P(^ANN^A_ij_) predicted the propensity of a specific ANN topology to predict the biological interaction A_ij_ between the species A_*i*_ and B_*j*_ from the ecological web (A_ij_ = 1 or not A_ij_ = 0). The best linear AutoML with maximum values of Ac, Sp, and Sn (training and external validation series) is described by Eq. 1. The best linear AutoML was made up of only 5 features: two Sh_k_ (*k* = 0, 3) for each node A_*i*_ and B_*j*_, and a Sh_k_ (*k* = 3) for the ANN classifier.

1 $$\begin{array}{rcl}{\rm{S}}({\rm{L}}={1,\mathrm{ANN}}_{{\rm{j}}})\, & = & -{\rm{42}}\mathrm{.38}\cdot S{h}_{0}({A}_{i})+15.69\cdot S{h}_{3}({A}_{i})\\  &  & -{\rm{44.95}}\cdot S{h}_{0}({B}_{j})+13.79\cdot S{h}_{3}({B}_{j})\\  &  & -{\rm{0.014}}\cdot S{h}_{3}(AN{N}_{j})-0.8263\\  &  & {\rm{n}}=235\,\,540\,{\chi }^{2}\,=\mathrm{56326}{\rm{.2}}\,p < 0{\rm{.001}}\end{array}$$

In each BEN, the connections (A_ij_ = 1) indicated the existence of a biological interaction between the organisms of *i* biological species with the organisms of *j* species. Table 1 shows the ANN topologies trained to predict BEN connectivity. Thus, the Net-Net AutoML model was able to predict whether a new ANN topology could correctly predict the connectivity between a pair of BEN nodes, prior to training. We introduced variability in the ANN topologies using ANNs without hidden layers (no. 8, 9, 10), with only one hidden layer (no. 1, 2, 7, 5) and with two hidden layers (no. 3, 4, 6). Future work should include different ANN topologies such as skip layer connections, drop out neurons, deep ANNs. These will enable a wider search in the space of possible networks.

**Table 1:** Information indices ^ANN^Sh_k_ of the ANNs used as inputs to train the AutoML model.

ANN	ANN	ANN	Sh_k_(ANN_j_)					
No.	Topology	AUROC	k = 0	k = 1	k = 2	k = 3	k = 4	k = 5
1	MLP14:14-10-1:1	0.6	1.367	1.561	1.428	1.428	1.428	1.428
2	MLP15:15-12-1:1	0.7	1.424	1.571	1.492	1.492	1.492	1.492
3	MLP18:18-8-13-1:1	0.8	1.518	2.074	1.509	1.704	1.704	1.704
4	MLP16:16-8-10-1:1	0.7	1.486	1.881	1.415	1.532	1.532	1.532
5	MLP18:18-8-1:1	0.8	1.564	1.759	1.423	1.481	1.481	1.481
6	MLP16:16-12-13-1:1	0.8	1.358	1.481	1.481	1.481	1.481	1.481
7	MLP11:11-10-1:1	0.7	1.394	1.806	1.395	1.395	1.395	1.395
8	LNN16:16-1:1	0.6	0.881	2.637	2.637	2.637	2.637	2.637
9	LNN17:17-1:1	0.6	0.895	2.788	2.788	2.788	2.788	2.788
10	LNN18:18-1:1	0.6	0.908	2.938	2.938	2.938	2.938	2.938

The LDA model showed significant goodness-of-fit, also illustrated by Accuracy (Ac), Sensitivity (Sn), and Specificity (Sp) classification values, both in training and external validation series (see Table 2**)**. The proof-of-concept AutoML model fit very well 338,050 outcomes predicted with 10 (previously trained) ANNs. These results were obtained after training the 10 ANNs to learn to discriminate between biological interactions (predation, parasitism, mutualism, *etc*.) which were connected (A_ij_ = 1) or not (A_ij_ = 0) in BENs of many ecosystems. The mission of the AutoML did not consist of the prediction of BEN connectivity, and, therefore, Sn referred to the number of times that the AutoML was able to evaluate whether a given ANN topology could correctly predict BEN nodes connectivity. The same analogy applied to Sp and Ac. Using Net-Net AutoML methodology, one could decide which ANN will receive more computing resources for training and which one can be used to predict different links (A_ij_ = 1 or 0). The parameter Sh_3i_ quantified the information related to the position of *i* organism and their neighbours (*k* = 3) placing a topological distance d ≤ 3 in the BEN. ^ANN^Sh_3_ is also similar but quantifies information for the neurons in a specific ANN topology and not for the organisms in the biological network. Figure 2 illustrates the distribution of the Sh_k_ values for all the BENs and ANN topologies studied herein.

**Table 2:** Statistics for the base line LDA Net-Net AutoML model.

Model	Training Series			
Param.	%	Class	A_ij_ = 0	A_ij_ = 1
Sp	74.2	A_ij_ = 0	**93933**	32745
Sn	70.5	A_ij_ = 1	37438	**89424**
Ac	72.3	Total		
**Model**	**Cross-Validation Series**			
Sp	76.0	A_ij_ = 0	**32163**	10179
Sn	70.4	A_ij_ = 1	12465	**29703**
Ac	73.2	Total		

Note: rows: Observed classifications; columns: Predicted classifications; A_ij_ = 1, calculation with high priority; A_ij_ = 0 otherwise.

The LDA model is a base line classifier that was compared to 11 complex classifiers obtained with 9 ML methods, such as Bayesian Nets, Naïve Bayes Nets, Logistic Regression, Decision Table, Multilayer Perceptron (MLP), Random Forest, Bagging, AdaBoost, and Deep Fully Connected (FC) Networks. All 18 descriptors were used as inputs. The test accuracy (ACC) and AUROC values are presented in Table 3.

**Table 3:** Accuracies of non-linear Net-Net classifiers.

ML Classifier	Test Accuracy	Test AUROC
Bayesian Nets	0.681	0.737
Naive Bayes Nets	0.586	0.636
Logistic Regression	0.618	0.668
Decision Table	0.516	0.552
MLP 1H	0.809	0.878
MLP 2H	0.827	0.902
Random Forest	0.832	0.914
Bagging REP	0.804	0.884
Bagging MLP	0.819	0.896
AdaBoostM1 MLP	0.821	0.884
Deep FC Nets	0.866	0.935

Note: please see Methods section for details on the classifier.

It should be observed that the Bayesian methods, Decision Table and Logistic Regression provided accuracies lower than the LDA model. With the MLP, by introducing hidden layers in Artificial Neural Networks, the accuracies and AUROC were improved, with values over 0.8, better than the LDA classifier. More complex models such as ensemble classifiers based on MLP with only one hidden layer (Bagging MLP and AdaBoostM1 MLP) could produce slightly better results. By introducing more hidden layers with MLP 2 H and Deep FC Nets, the test accuracy was increased up to 0.866 (test AUROC = 0.935). Random Forest was not the best model, but it was able to provide a test accuracy of 0.832 (AUROC = 0.914). The ensemble classifier based on simple REP trees, such as Bagging REP, had a performance similar to the MLP 1 H (only one hidden layer).

Therefore, Deep Nets provided the best results, starting with MLP 2H with only 18 neurons (=number of input features) in the first hidden layer, and 9 neurons in the second hidden layer (ACC = 0.827, AUROC = 0.902), leading to the more complex Deep FC Nets with 200, 400 and 200 neurons in the hidden layers 1, 2 and 3 (ACC = 0.866, AUROC = 0.935). An accuracy increase of 4% was obtained with more complex topology of the neural network from 18–9 to 200–400–200 neurons. The DL model was obtained using 10 different network topologies, from one to three hidden layers, with different optimization algorithms, dropout rates, and other hyperparameters. The best DL model had the hidden layer topology n-n*2-n, with activation functions = tanh, n = 200, dropout rate = 0.5, optimizer algorithm = Adam, initialization of weights = glorot_normal, batch size = 4096, epochs = 500, training AUROC = 0.963, training ACC = 0.897, test AUROC = 0.935 and test ACC = 0.866.

The current method used different applications such as MI-NODES for descriptors, STATISTICA, Weka, and Python/Keras for ML classifiers. If the user does not test deep learning classifiers for the final Net-Net model, there is no need for programming. In Weka it is possible to test a deeper MLP. Therefore, scientists without advanced knowledge of programming are able to implement this methodology for specific BENs. An optimal implementation of the method should be performed using a unique python code for all the Net-Net methodology steps. This is the next step for the future version of this method.

## Conclusions

The current study confirms that Markov chains are useful to calculate Sh_k_ information indices in order to quantify the connectivity patterns of both BENs and ANNs. The new Net-Net AutoML methodology demonstrated how to develop a linear AutoML model, able to select which ANN topology would correctly predict the connectivity of BEN nodes before training it. The best AutoML model demonstrated an accuracy over 86% in test subsets. In conclusion, Net-Net AutoMLs with Sh_k_ information indices could be used to screen ANN topologies that can predict the links in biological networks. This may lead to an optimization of computing resources with the prioritization of the training of the best ANN topologies.

## Methods

### Biological ecosystem networks dataset

A number of 69 Ecosystems or Food Webs were used. The network files in.net format were assembled by our group in a previous work^47^. The datasets were downloaded from the Interaction Web Database (IWDB): http://www.nceas.ucsb.edu/interactionweb/index.html.

### Computational model

#### Markov-Shannon Entropy Centralities from MI-NODES tool

In the present work, the classical Markov matrix (^1^**Π**) was constructed for each network (BEN complex networks and ANNs). In the case of BENs, the adjacency/connectivity matrix were downloaded from public resources as **A** (*n* by *n* matrix, where *n* is the number of nodes/vertices). Next, the Markov matrix **Π** was calculated. It contains the vertices probability (*p*_*ij*_) based on **A**. The probability matrix was raised to the power *k*, resulting (^1^**Π)**^k^, and it was multiplied by the vector of the initial probabilities (^0^*p*_*j*_). The resulting vectors ^k^P contained the absolute probabilities to reach the nodes moving throughout a walk of length *k* from node *j* (^*k*^*p*_*j*_) for each *k* (Eqs 2 and 3). The entropy of graph Sh_k_(G) could be calculated based on the entropy of each node Sh_kj_:

2 $$\,{}^{k}P=\,{}^{0}P\times {({}^{1}{\rm{\Pi }})}^{k}=[\,{}^{k}p_{1},\,{}^{k}p_{2},\,\ldots ,\,{}^{k}p_{j}]$$

3 $$S{h}_{k}(G)=\sum _{j\in G}S{h}_{kj}=-\,\sum _{j\in G}\,{}^{k}p_{j}\,\mathrm{log}{}^{k}p_{j}$$

### Net-Net AutoML models

Due to the dimension of the dataset and the complexity of the models, for the current calculations two systems were used:

- BioCAI cluster of RNASA-IMEDIR group (UDC) with 200 CPU cores;
- A desktop computer with processor i7 (3.60 GHz × 4 physical cores), 16 G RAM, and a GPU NVIDIA Titan Xp (Pascal architecture, dedicated memory of 12 G G5X, 3840 CUDA cores, boost clock 1,582 MHz).

The GPU was particularly useful for the Deep Learning calculations with Keras. All the calculations could be carried out with a desktop computer over a larger period a time, especially due to the Deep Learning calculations.

Once the Sh_k_ values of both the ANNs and BENs have been obtained, a Linear Discriminant Analysis (LDA) implemented in the STATISTICA software^48^ can be run. Let P(^ANN^A_ij_) be the output of a screening model used to predict the ability of a given ANN topology to correctly classify the BEN connectivity A_ij_ between two nodes *i* and *j* (A_ij_ = 1 or 0). Eq. 4 describes the general formula for the LDA model using the following coefficients: a_ki_ as coefficients of the Sh for the *i* node (Sh_ki_), b_kj_ as coefficients of the Sh for the *j* node (Sh_kj_), c_kij_ as coefficients of the differences between the Sh of the nodes *i* and *j* (∆Sh_kij_ = Sh_ki_ − Sh_kj_), ^ANN^d_k_ as coefficient of the average Sh for a specific ANN topology, and e_0_ as the free term coefficient. The *k* index indicates that this Sh_k_ value codifies information for all nodes placed at least at topological distance *k* from the reference node.

Different statistical parameters can be used to evaluate the statistical significance and validate the goodness-of-fit of LDA equation: n = number of cases, χ^2^ = Chi-square, p = error level, as well as the Accuracy (Ac), Specificity (Sp), and Sensitivity (Sn) of both training and external validation series^49^.

4 $$S(L=1,ANN)=\sum _{k=0}^{5}{a}_{ki}\cdot S{h}_{ki}+\sum _{k=0}^{5}{b}_{kj}\cdot S{h}_{kj}+\sum _{k=0}^{5}{c}_{kij}\cdot {\rm{\Delta }}S{h}_{kij}+\sum _{k=0}^{5}{}^{ANN}d_{k}\cdot {}^{ANN}S{h}_{k}+{e}_{0}$$

Several complex classifiers were tested (see Table 3):

- Bayesian Nets = weka.classifiers.bayes.BayesNet -D -Q weka.classifiers.bayes.net.search.local.K2–P 1 -S BAYES -E weka.classifiers.bayes.net.estimate.SimpleEstimator –A 0.
- Naive Bayes Nets = weka.classifiers.bayes.NaiveBayes
- Logistic Regression = weka.classifiers.functions.Logistic -R 1.0E-8 -M -1 -num-decimal-places 4
- Decision Table = weka.classifiers.rules.DecisionTable -X 1 -S “weka.attributeSelection.BestFirst -D 1 -N 5”
- MLP 1H (Multilayer Perceptron 1 hidden layer) = weka.classifiers.functions.MultilayerPerceptron -L 0.3 -M 0.2 -N 1000 -V 0 -S 0 -E 20 -H a
- MLP 2H (Multilayer Perceptron 2 hidden layers) = weka.classifiers.functions.MultilayerPerceptron -L 0.3 -M 0.2 -N 5000 -V 0 -S 0 -E 20 -H “18, 9” -batch-size 500
- Random Forest = weka.classifiers.trees.RandomForest -P 100 -I 500 -num-slots 1 -K 0 -M 1.0 -V 0.001 -S 1
- Bagging REP = weka.classifiers.meta.Bagging -P 100 -S 1 -num-slots 1 -I 10 -W weka.classifiers.trees.REPTree –M 2 -V 0.001 -N 3 -S 1 -L -1 -I 0.0
- Bagging MLP = weka.classifiers.meta.Bagging -P 100 -S 1 -num-slots 1 -I 10 -W weka.classifiers.functions.MultilayerPerceptron -batch-size 4000 –L 0.3 -M 0.2 -N 500 -V 0 -S 0 -E 20 -H a
- AdaBoostM1 MLP = weka.classifiers.meta.AdaBoostM1 -P 100 -S 1 -I 10 -W weka.classifiers.functions.MultilayerPerceptron -batch-size 4000 –L 0.3 -M 0.2 -N 500 -V 0 -S 0 -E 20 -H a
- Deep FC Nets (Deep Learning Fully Connected Networks) = n-n*2-n′ hidden layer topology (n = 200).

Deep Learning FC Nets were programmed in Python with Keras, and the other classifiers were obtained with the Weka tool. For the DL models, different hyperparameter values were tested:

- n = Number of neurons in a hidden layer: 10, 18, 50, 100, 200, 500.
- Network topologies: ‘n’,‘n-n’, ‘n-n-n’, ‘n-n*2’, ‘n-n*2-n’, ‘n*2’, ‘n*2-n’, ‘n-n:2’, ‘n:2’, ‘n-n:2-n:4’ (this notation does not include the input layer with 18 neurons = no. of features and the output layer with a neuron for the class).
- Neuron dropout rate: 0.0, 0.1, 0.2, 0.3, 0.4, 0.5, 0.6, 0.7, 0.8, 0.9.
- Optimizers: ‘RMSprop’, ‘Adagrad’, ‘Adadelta’, ‘Adam’, ‘Adamax’, ‘Nadam’.
- Weight initialization for hidden layer neurons: ‘uniform’, ‘lecun_uniform’, ‘normal’, ‘glorot_normal’, ‘glorot_uniform’, ‘he_normal’, ‘he_uniform’.
- Batch size for training = 1024, 2048, 4096.
- Training epochs = 20, 50, 100, 200, 300, 400, 500.
- Training cross validation: 3-folds (default value in Keras).

The Net-Net AutoML algorithm shown in Fig. 1 could be described as follows:

1. For each BEN:
  (1.1) Get the connectivity matrix.
  (1.2) Add weights for the BEN connections (if present).
  (1.3) For each node *A*:
    (1.3.1) Calculate node Shannon Entropies with MI-NODES: Sh_k_(A_i_).
    (1.3.2) Create pairs of entropies for all the other nodes *B*: Sh_k_(A_i_) − Sh_k_(B_j_).
2. Find different ANN classifiers to predict BEN node A_j_ − B_j_ connectivity:
  (2.1) For each ANN_j_ classifier:

(2.1.1) Calculate network Shannon Entropy: Sh_k_(ANN_j_).

(3) Merge BEN node descriptors with ANN descriptors into Net-Net dataset: Sh_k_(A_i_), Sh_k_(B_j_), Sh_k_(ANN_j_).
(4) Split Net-Net dataset into training and test subsets.
(5) Find the best Net-Net classifier to evaluate whether a specific ANN can predict the BEN connectivity:

(5.1) For each ML method (Bayesian, Trees, Artificial Neural Networks, etc.)

(5.1.1) For each set of model parameters (ex: topology, activation function, etc.)

(5.1.1.1) Use a Net-Net subset to train the classifier
(5.1.1.2) Evaluate the model with test subset calculating accuracy (ACC) and AUROC.

(6) Choose the best Net-Net classifier with the best ACC and AUROC.

Steps (5) and (6) used Weka and Python/Keras scripts. In the future version of the method, different classifiers will be tested for the BEN connectivity prediction (not only ANNs). This involves the adaptation of MI-NODES application. The main advantage of the Net-Net methodology is that it can build a Net-Net classifier able to screen ANN classifiers which predict BEN node connectivities.

## Electronic supplementary material

Supplementary Information

## Data Availability

All data generated or analyzed during this study were included in this article (along with its Supplementary Information files) and they are publicly available at Figshare repository with 10.6084/m9.figshare.6238424.
