# Supplementary material for: Net-Net Auto Machine Learning (AutoML) Prediction of Complex Ecosystems
Source: Sci Rep. 2018 Aug 17;8:12340. doi: 10.1038/s41598-018-30637-w (PMC6098100; doi:10.1038/s41598-018-30637-w)
Supplement: Supplementary file 1 — Supplementary Information [file 41598_2018_30637_MOESM1_ESM.pdf]

Net-Net Auto Machine Learning (AutoML) Prediction of Complex Ecosystems

Enrique Barreiro[1,2,3], Cristian R. Munteanu[1], Maykel Cruz-Monteagudo[2,3], Alejandro Pazos[1,4], and Humbert González-Díaz[5,6,*]

[1] Department of Computation, Computer Science Faculty, University of A Coruna (UDC), 15071, A Coruña, Spain.

[2] Center for Computational Science (CCS), University of Miami (UM), Miami, 33136, FL, USA.

[3] West Coast University, Miami Campus, 33178, FL, USA.

[4] Biomedical Research Institute of A Coruña (INIBIC), University Hospital Complex of A Coruña (CHUAC), A Coruña, 15006, Spain.

[5] Faculty of Science and Technology, University of the Basque Country (UPV/EHU), 48940, Biscay, Spain.

[6] IKERBASQUE, Basque Foundation for Science, 48011 Bilbao, Biscay, Spain.

*Corresponding authors: H.G.D., Faculty of Science and Technology, University of the Basque Country (UPV/EHU), 48940, Biscay, Spain, Email: humberto.gonzalezdiaz@ehu.es.

SM2

C(Lij) in	C(Lij) out	p1	Lij	cv	ANN	Node_A	Node_B	IW DB	Sh0(A)	Sh3(A)	Sh0(B)	Sh3(B)
1	1	0.89	1	t	1	Amphiprion bicinctus	Heteractis aurora	1	0.05	0.08	0.06	0.06
1	1	0.97	1	t	1	Amphiprion bicinctus	Stichodactyla mertensii	1	0.05	0.08	0.09	0.07
1	1	0.97	1	t	1	Amphiprion bicinctus	Entacmaea quadricolor	1	0.05	0.08	0.09	0.07
1	1	0.96	1	cv	1	Amphiprion bicinctus	Heteractis magnifica	1	0.05	0.08	0.08	0.06
1	1	0.97	1	t	1	Amphiprion bicinctus	Heteractis crispa	1	0.05	0.08	0.09	0.07
1	1	0.91	1	t	1	Amphiprion bicinctus	Stichodactyla gigantea	1	0.05	0.08	0.07	0.06
0	1	0.76	1	t	1	Cryptodendrum adhaesivum	Amphiprion clarkii	1	0.01	0.06	0.08	0.07
1	1	0.85	1	cv	1	Premnas biaculeatus	Entacmaea quadricolor	1	0.01	0.08	0.09	0.07
1	1	0.96	1	t	1	Stichodactyla haddoni	Amphiprion clarkii	1	0.06	0.06	0.08	0.07
0	1	0.92	1	t	1	Stichodactyla haddoni	Amphiprion akindynos	1	0.06	0.06	0.06	0.08
0	1	0.92	1	t	1	Stichodactyla haddoni	Amphiprion chrysopterus	1	0.06	0.06	0.06	0.08
0	1	0.76	1	cv	1	Stichodactyla haddoni	Amphiprion omanensis	1	0.06	0.06	0.03	0.08
0	1	0.82	1	t	1	Stichodactyla haddoni	Amphiprion chrysogaster	1	0.06	0.06	0.04	0.07
0	1	0.59	1	t	1	Stichodactyla haddoni	Amphiprion sebae	1	0.06	0.06	0.01	0.07
0	1	0.82	1	t	1	Stichodactyla haddoni	Amphiprion polymnus	1	0.06	0.06	0.04	0.07
1	1	0.90	1	cv	1	Amphiprion akallopisos	Stichodactyla mertensii	1	0.02	0.08	0.09	0.07
1	1	0.88	1	t	1	Amphiprion akallopisos	Heteractis magnifica	1	0.02	0.08	0.08	0.06
1	1	0.89	1	t	1	Amphiprion sandaracinos	Stichodactyla mertensii	1	0.02	0.08	0.09	0.07
1	1	0.91	1	t	1	Amphiprion sandaracinos	Heteractis crispa	1	0.02	0.08	0.09	0.07
1	1	0.77	1	cv	1	Amphiprion allardi	Heteractis aurora	1	0.03	0.08	0.06	0.06
1	1	0.92	1	t	1	Amphiprion allardi	Stichodactyla mertensii	1	0.03	0.08	0.09	0.07
1	1	0.92	1	t	1	Amphiprion allardi	Entacmaea quadricolor	1	0.03	0.08	0.09	0.07
1	1	0.91	1	t	1	Amphiprion nigripes	Heteractis magnifica	1	0.03	0.07	0.08	0.06
1	1	0.93	1	cv	1	Amphiprion perideraion	Heteractis magnifica	1	0.04	0.07	0.08	0.06

1	1	0.95	1	t	1	Amphiprion perideraion	Heteractis crispa	1	0.04	0.07	0.09	0.07
1	1	0.86	1	t	1	Amphiprion perideraion	Stichodactyla gigantea	1	0.04	0.07	0.07	0.06
1	1	0.66	1	t	1	Amphiprion perideraion	Macrodactyla doreensis	1	0.04	0.07	0.04	0.06
0	1	0.82	1	cv	1	Heteractis aurora	Amphiprion tricinctus	1	0.06	0.06	0.04	0.08
1	1	0.96	1	t	1	Heteractis aurora	Amphiprion clarkii	1	0.06	0.06	0.08	0.07
1	1	0.92	1	t	1	Heteractis aurora	Amphiprion akindynos	1	0.06	0.06	0.06	0.08
1	1	0.92	1	t	1	Heteractis aurora	Amphiprion chrysopterus	1	0.06	0.06	0.06	0.08
0	1	0.82	1	cv	1	Heteractis aurora	Amphiprion chrysogaster	1	0.06	0.06	0.04	0.07
1	1	0.85	1	t	1	Amphiprion mccullochi	Entacmaea quadricolor	1	0.01	0.08	0.09	0.07
1	1	0.94	1	t	1	Stichodactyla mertensii	Amphiprion tricinctus	1	0.09	0.07	0.04	0.08
0	1	0.82	1	t	1	Stichodactyla mertensii	Amphiprion leucokranos	1	0.09	0.07	0.01	0.08
0	1	0.82	1	cv	1	Stichodactyla mertensii	Amphiprion latifasciatus	1	0.09	0.07	0.01	0.08
1	1	0.99	1	t	1	Stichodactyla mertensii	Amphiprion clarkii	1	0.09	0.07	0.08	0.07
1	1	0.97	1	t	1	Stichodactyla mertensii	Amphiprion akindynos	1	0.09	0.07	0.06	0.08
1	1	0.97	1	t	1	Stichodactyla mertensii	Amphiprion chrysopterus	1	0.09	0.07	0.06	0.08
0	1	0.92	1	cv	1	Stichodactyla mertensii	Amphiprion ocellaris	1	0.09	0.07	0.03	0.08
0	1	0.82	1	t	1	Stichodactyla mertensii	Amphiprion fuscocaudatus	1	0.09	0.07	0.01	0.08
0	1	0.94	1	t	1	Stichodactyla mertensii	Amphiprion chrysogaster	1	0.09	0.07	0.04	0.07
1	1	0.89	1	t	1	Amphiprion ephippium	Entacmaea quadricolor	1	0.02	0.08	0.09	0.07
1	1	0.91	1	cv	1	Amphiprion ephippium	Heteractis crispa	1	0.02	0.08	0.09	0.07
1	1	0.87	1	t	1	Amphiprion latezonatus	Heteractis crispa	1	0.01	0.08	0.09	0.07
0	1	0.76	1	t	1	Heteractus malu	Amphiprion clarkii	1	0.01	0.06	0.08	0.07
1	1	0.94	1	t	1	Entacmaea quadricolor	Amphiprion tricinctus	1	0.09	0.07	0.04	0.08
0	1	0.88	1	cv	1	Entacmaea quadricolor	Amphiprion rubrocinctus	1	0.09	0.07	0.02	0.08
1	1	0.99	1	t	1	Entacmaea quadricolor	Amphiprion clarkii	1	0.09	0.07	0.08	0.07

1	1	0.97	1	t	1	Entacmaea quadricolor	Amphiprion akindynos	1	0.09	0.07	0.06	0.08
1	1	0.97	1	t	1	Entacmaea quadricolor	Amphiprion chrysopterus	1	0.09	0.07	0.06	0.08
0	1	0.82	1	cv	1	Entacmaea quadricolor	Amphiprion frenatus	1	0.09	0.07	0.01	0.08
0	1	0.92	1	t	1	Entacmaea quadricolor	Amphiprion omanensis	1	0.09	0.07	0.03	0.08
0	1	0.92	1	t	1	Entacmaea quadricolor	Amphiprion melanopus	1	0.09	0.07	0.03	0.08
1	1	0.95	1	t	1	Amphiprion tricinctus	Heteractis crispa	1	0.04	0.08	0.09	0.07
1	1	0.75	1	cv	1	Amphiprion rubrocinctus	Stichodactyla gigantea	1	0.02	0.08	0.07	0.06
1	1	0.82	1	t	1	Amphiprion leucokranos	Heteractis magnifica	1	0.01	0.08	0.08	0.06
1	1	0.87	1	t	1	Amphiprion leucokranos	Heteractis crispa	1	0.01	0.08	0.09	0.07
1	1	0.98	1	t	1	Amphiprion clarkii	Heteractis magnifica	1	0.08	0.07	0.08	0.06
1	1	0.99	1	cv	1	Amphiprion clarkii	Heteractis crispa	1	0.08	0.07	0.09	0.07
1	1	0.96	1	t	1	Amphiprion clarkii	Stichodactyla gigantea	1	0.08	0.07	0.07	0.06
1	1	0.90	1	t	1	Amphiprion clarkii	Macrodactyla doreensis	1	0.08	0.07	0.04	0.06
1	1	0.97	1	t	1	Heteractis magnifica	Amphiprion akindynos	1	0.08	0.06	0.06	0.08
1	1	0.97	1	cv	1	Heteractis magnifica	Amphiprion chrysopterus	1	0.08	0.06	0.06	0.08
0	1	0.90	1	t	1	Heteractis magnifica	Amphiprion percula	1	0.08	0.06	0.03	0.08
0	1	0.90	1	t	1	Heteractis magnifica	Amphiprion ocellaris	1	0.08	0.06	0.03	0.08
0	1	0.93	1	t	1	Heteractis magnifica	Amphiprion chrysogaster	1	0.08	0.06	0.04	0.07
0	1	0.90	1	cv	1	Heteractis magnifica	Amphiprion melanopus	1	0.08	0.06	0.03	0.08
1	1	0.98	1	t	1	Amphiprion akindynos	Heteractis crispa	1	0.06	0.08	0.09	0.07
1	1	0.93	1	t	1	Amphiprion akindynos	Stichodactyla gigantea	1	0.06	0.08	0.07	0.06
1	1	0.98	1	t	1	Heteractis crispa	Amphiprion chrysopterus	1	0.09	0.07	0.06	0.08
1	1	0.93	1	cv	1	Heteractis crispa	Amphiprion percula	1	0.09	0.07	0.03	0.08
0	1	0.93	1	t	1	Heteractis crispa	Amphiprion omanensis	1	0.09	0.07	0.03	0.08
1	1	0.93	1	t	1	Heteractis crispa	Amphiprion melanopus	1	0.09	0.07	0.03	0.08

0	1	0.95	1	t	1	Heteractis crispa	Amphiprion polymnus	1	0.09	0.07	0.04	0.07
1	1	0.82	1	cv	1	Amphiprion chrysopterus	Macrodactyla doreensis	1	0.06	0.08	0.04	0.06
1	1	0.81	1	t	1	Amphiprion percula	Stichodactyla gigantea	1	0.03	0.08	0.07	0.06
0	1	0.80	1	t	1	Stichodactyla gigantea	Amphiprion ocellaris	1	0.07	0.06	0.03	0.08
0	1	0.80	1	t	1	Stichodactyla gigantea	Amphiprion melanopus	1	0.07	0.06	0.03	0.08
0	1	0.67	1	cv	1	Macrodactyla doreensis	Amphiprion polymnus	1	0.04	0.06	0.04	0.07
0	0	0.20	0	t	1	Amphiprion akallopisos	Amphiprion latezonatus	1	0.02	0.08	0.01	0.08
0	0	0.39	0	t	1	Premnas biaculeatus	Macrodactyla doreensis	1	0.01	0.08	0.04	0.06
0	0	0.28	0	t	1	Amphiprion ocellaris	Amphiprion sebae	1	0.03	0.08	0.01	0.07
0	1	0.80	0	cv	1	Amphiprion tricinctus	Amphiprion chrysopterus	1	0.04	0.08	0.06	0.08
1	1	0.82	0	t	1	Amphiprion perideraion	Heteractis aurora	1	0.04	0.07	0.06	0.06
0	0	0.32	0	t	1	Heteractus malu	Amphiprion omanensis	1	0.01	0.06	0.03	0.08
0	1	0.76	0	t	1	Stichodactyla haddoni	Amphiprion melanopus	1	0.06	0.06	0.03	0.08
0	0	0.14	0	cv	1	Amphiprion mccullochi	Amphiprion frenatus	1	0.01	0.08	0.01	0.08
0	1	0.98	0	t	1	Heteractis aurora	Entacmaea quadricolor	1	0.06	0.06	0.09	0.07
0	1	0.54	0	t	1	Amphiprion chrysogaster	Amphiprion melanopus	1	0.04	0.07	0.03	0.08
0	1	0.73	0	t	1	Amphiprion chrysopterus	Amphiprion melanopus	1	0.06	0.08	0.03	0.08
0	0	0.28	0	cv	1	Amphiprion frenatus	Amphiprion ocellaris	1	0.01	0.08	0.03	0.08
0	1	0.73	0	t	1	Amphiprion chrysopterus	Amphiprion ocellaris	1	0.06	0.08	0.03	0.08
0	1	0.84	0	t	1	Stichodactyla haddoni	Macrodactyla doreensis	1	0.06	0.06	0.04	0.06
0	1	0.88	0	t	1	Stichodactyla mertensii	Amphiprion rubrocinctus	1	0.09	0.07	0.02	0.08
0	1	0.63	0	cv	1	Amphiprion perideraion	Amphiprion polymnus	1	0.04	0.07	0.04	0.07
0	0	0.37	0	t	1	Amphiprion sandaracinos	Amphiprion allardi	1	0.02	0.08	0.03	0.08
0	1	0.61	0	t	1	Heteractus malu	Amphiprion chrysopterus	1	0.01	0.06	0.06	0.08
0	0	0.28	0	t	1	Premnas biaculeatus	Amphiprion omanensis	1	0.01	0.08	0.03	0.08

0	1	0.57	0	cv	1	Premnas biaculeatus	Amphiprion akindynos	1	0.01	0.08	0.06	0.08
0	0	0.21	0	t	1	Amphiprion akallopisos	Amphiprion sebae	1	0.02	0.08	0.01	0.07
1	1	0.82	0	t	1	Amphiprion akindynos	Macrodactyla doreensis	1	0.06	0.08	0.04	0.06
0	1	0.80	0	t	1	Amphiprion rubrocinctus	Amphiprion clarkii	1	0.02	0.08	0.08	0.07
0	1	0.84	0	cv	1	Heteractis aurora	Macrodactyla doreensis	1	0.06	0.06	0.04	0.06
0	0	0.45	0	t	1	Amphiprion perideraion	Amphiprion rubrocinctus	1	0.04	0.07	0.02	0.08
0	0	0.40	0	t	1	Cryptodendrum adhaesivum	Amphiprion perideraion	1	0.01	0.06	0.04	0.07
0	0	0.14	0	t	1	Premnas biaculeatus	Amphiprion leucokranos	1	0.01	0.08	0.01	0.08
0	0	0.20	0	cv	1	Amphiprion ephippium	Amphiprion latezonatus	1	0.02	0.08	0.01	0.08
1	1	0.89	0	t	1	Amphiprion tricinctus	Amphiprion clarkii	1	0.04	0.08	0.08	0.07
0	1	0.75	0	t	1	Amphiprion nigripes	Amphiprion akindynos	1	0.03	0.07	0.06	0.08
0	1	0.80	0	t	1	Heteractis magnifica	Amphiprion frenatus	1	0.08	0.06	0.01	0.08
0	1	0.74	0	cv	1	Amphiprion allardi	Amphiprion chrysopterus	1	0.03	0.08	0.06	0.08
0	0	0.39	0	t	1	Amphiprion latezonatus	Macrodactyla doreensis	1	0.01	0.08	0.04	0.06
0	0	0.27	0	t	1	Amphiprion nigripes	Amphiprion latezonatus	1	0.03	0.07	0.01	0.08
0	0	0.20	0	t	1	Amphiprion sandaracinos	Amphiprion latifasciatus	1	0.02	0.08	0.01	0.08
0	1	0.54	0	cv	1	Amphiprion chrysopterus	Amphiprion fuscocaudatus	1	0.06	0.08	0.01	0.08
0	0	0.35	0	t	1	Amphiprion frenatus	Amphiprion chrysogaster	1	0.01	0.08	0.04	0.07
0	0	0.32	0	t	1	Cryptodendrum adhaesivum	Amphiprion melanopus	1	0.01	0.06	0.03	0.08
0	0	0.28	0	t	1	Premnas biaculeatus	Amphiprion melanopus	1	0.01	0.08	0.03	0.08
0	0	0.17	0	cv	1	Cryptodendrum adhaesivum	Amphiprion latezonatus	1	0.01	0.06	0.01	0.08
0	0	0.14	0	t	1	Amphiprion latezonatus	Amphiprion fuscocaudatus	1	0.01	0.08	0.01	0.08
0	0	0.28	0	t	1	Amphiprion mccullochi	Amphiprion percula	1	0.01	0.08	0.03	0.08
0	0	0.36	0	t	1	Amphiprion fuscocaudatus	Amphiprion chrysogaster	1	0.01	0.08	0.04	0.07

0	1	0.57	0	cv	1	Amphiprion mccullochi	Amphiprion akindynos	1	0.01	0.08	0.06	0.08
0	0	0.27	0	t	1	Amphiprion allardi	Amphiprion frenatus	1	0.03	0.08	0.01	0.08
0	0	0.37	0	t	1	Amphiprion ephippium	Amphiprion melanopus	1	0.02	0.08	0.03	0.08
1	1	0.89	0	t	1	Amphiprion akallopisos	Entacmaea quadricolor	1	0.02	0.08	0.09	0.07
0	1	0.65	0	cv	1	Stichodactyla gigantea	Amphiprion sebae	1	0.07	0.06	0.01	0.07
0	0	0.21	0	t	1	Premnas biaculeatus	Amphiprion rubrocinctus	1	0.01	0.08	0.02	0.08
0	0	0.36	0	t	1	Amphiprion fuscocaudatus	Amphiprion polymnus	1	0.01	0.08	0.04	0.07
0	1	0.93	0	t	1	Stichodactyla haddoni	Heteractis aurora	1	0.06	0.06	0.06	0.06
0	1	0.80	0	cv	1	Amphiprion perideraion	Amphiprion akindynos	1	0.04	0.07	0.06	0.08
1	1	0.91	0	t	1	Amphiprion akallopisos	Heteractis crispa	1	0.02	0.08	0.09	0.07
0	1	0.86	0	t	1	Amphiprion nigripes	Amphiprion clarkii	1	0.03	0.07	0.08	0.07
0	0	0.14	0	t	1	Amphiprion latezonatus	Amphiprion frenatus	1	0.01	0.08	0.01	0.08
0	1	0.73	0	cv	1	Amphiprion akindynos	Amphiprion omanensis	1	0.06	0.08	0.03	0.08
0	0	0.37	0	t	1	Amphiprion sebae	Amphiprion polymnus	1	0.01	0.07	0.04	0.07
0	1	0.54	0	t	1	Amphiprion perideraion	Amphiprion percula	1	0.04	0.07	0.03	0.08
0	0	0.14	0	t	1	Premnas biaculeatus	Amphiprion fuscocaudatus	1	0.01	0.08	0.01	0.08
0	1	0.80	0	cv	1	Amphiprion akallopisos	Amphiprion clarkii	1	0.02	0.08	0.08	0.07
0	0	0.37	0	t	1	Amphiprion ephippium	Amphiprion percula	1	0.02	0.08	0.03	0.08
0	0	0.20	0	t	1	Amphiprion akallopisos	Amphiprion fuscocaudatus	1	0.02	0.08	0.01	0.08
0	0	0.37	0	t	1	Amphiprion akallopisos	Amphiprion allardi	1	0.02	0.08	0.03	0.08
1	1	0.93	0	cv	1	Amphiprion nigripes	Stichodactyla mertensii	1	0.03	0.07	0.09	0.07
0	0	0.17	0	t	1	Cryptodendrum adhaesivum	Amphiprion mccullochi	1	0.01	0.06	0.01	0.08
1	1	0.93	0	t	1	Amphiprion chrysopterus	Stichodactyla gigantea	1	0.06	0.08	0.07	0.06
0	0	0.29	0	t	1	Amphiprion sandaracinos	Amphiprion rubrocinctus	1	0.02	0.08	0.02	0.08

1	1	0.91	0	cv	1	Amphiprion rubrocinctus	Heteractis crispa	1	0.02	0.08	0.09	0.07
0	1	0.54	0	t	1	Amphiprion perideraion	Amphiprion melanopus	1	0.04	0.07	0.03	0.08
0	0	0.37	0	t	1	Amphiprion akallopisos	Amphiprion ocellaris	1	0.02	0.08	0.03	0.08
1	1	0.88	0	t	1	Amphiprion ephippium	Heteractis magnifica	1	0.02	0.08	0.08	0.06
0	1	0.55	0	cv	1	Amphiprion omanensis	Amphiprion polymnus	1	0.03	0.08	0.04	0.07
1	1	0.59	0	t	1	Amphiprion percula	Macrodactyla doreensis	1	0.03	0.08	0.04	0.06
0	1	0.64	0	t	1	Stichodactyla gigantea	Amphiprion frenatus	1	0.07	0.06	0.01	0.08
0	0	0.14	0	t	1	Amphiprion mccullochi	Amphiprion leucokranos	1	0.01	0.08	0.01	0.08
0	0	0.47	0	cv	1	Amphiprion nigripes	Amphiprion omanensis	1	0.03	0.07	0.03	0.08
0	1	0.73	0	t	1	Amphiprion chrysopterus	Amphiprion percula	1	0.06	0.08	0.03	0.08
0	1	0.73	0	t	1	Amphiprion chrysopterus	Amphiprion omanensis	1	0.06	0.08	0.03	0.08
0	0	0.14	0	t	1	Premnas biaculeatus	Amphiprion latezonatus	1	0.01	0.08	0.01	0.08
0	0	0.23	0	cv	1	Amphiprion sandaracinos	Heteractus malu	1	0.02	0.08	0.01	0.06
1	1	0.95	1	t	1	Salvelinus namaycush	Eubothrium salvelini	2	0.09	0.07	0.04	0.08
1	1	0.97	1	t	1	Salvelinus namaycush	Neoechinorhynchus tumidus	2	0.09	0.07	0.05	0.08
1	1	0.96	1	t	1	Salvelinus namaycush	Cystidicola farionis	2	0.09	0.07	0.05	0.08
1	1	0.95	1	cv	1	Salvelinus namaycush	Triaenophorus crassus (L)	2	0.09	0.07	0.04	0.08
1	1	0.93	1	t	1	Salvelinus namaycush	Cyathocephalus truncatus	2	0.09	0.07	0.03	0.08
0	1	0.84	1	t	1	Salvelinus namaycush	Salmincola edwardsii	2	0.09	0.07	0.01	0.08
1	1	0.95	1	t	1	Salvelinus namaycush	Piscicola milneri	2	0.09	0.07	0.04	0.08
1	1	0.95	1	cv	1	Salvelinus namaycush	Diphyllobothrium sp. (L)	2	0.09	0.07	0.04	0.08
1	1	0.98	1	t	1	Salvelinus namaycush	Diplostomum spathaceum (L)	2	0.09	0.07	0.06	0.08
1	1	0.84	1	t	1	Salvelinus namaycush	Proteocephalus tumidocollus	2	0.09	0.07	0.01	0.08
1	1	0.93	1	t	1	Salvelinus namaycush	Capillaria salvelini	2	0.09	0.07	0.03	0.08

1	1	0.84	1	cv	1	Salvelinus namaycush	Discocotyle sagittata	2	0.09	0.07	0.01	0.08
1	1	0.97	1	t	1	Salvelinus namaycush	Crepidostomum farionis	2	0.09	0.07	0.05	0.08
1	1	0.95	1	t	1	Salvelinus namaycush	Tetracotyle sp. (L)	2	0.09	0.07	0.04	0.08
1	1	0.95	1	t	1	Eubothrium salvelini	Coregonus clupeaformis	2	0.04	0.08	0.09	0.07
1	1	0.95	1	cv	1	Eubothrium salvelini	Prosopium cylindraceum	2	0.04	0.08	0.09	0.07
1	1	0.96	1	t	1	Eubothrium salvelini	Thymallus arcticus	2	0.04	0.08	0.10	0.07
1	1	0.93	1	t	1	Esox lucius	Neoechinorhynchus tumidus	2	0.07	0.06	0.05	0.08
1	1	0.88	1	t	1	Esox lucius	Piscicola milneri	2	0.07	0.06	0.04	0.08
0	1	0.70	1	cv	1	Esox lucius	Tetraonchus monenteron	2	0.07	0.06	0.01	0.08
0	1	0.70	1	t	1	Esox lucius	Triaenophorus crassus	2	0.07	0.06	0.01	0.08
1	1	0.95	1	t	1	Esox lucius	Diplostomum spathaceum (L)	2	0.07	0.06	0.06	0.08
0	1	0.68	1	t	1	Esox lucius	Proteocephalus tumidocollus	2	0.07	0.06	0.01	0.08
1	1	0.93	1	cv	1	Esox lucius	Crepidostomum farionis	2	0.07	0.06	0.05	0.08
0	1	0.77	1	t	1	Esox lucius	Allocreadium isosporum	2	0.07	0.06	0.02	0.08
0	1	0.70	1	t	1	Esox lucius	Raphidascaris acus	2	0.07	0.06	0.01	0.08
1	1	0.97	1	t	1	Neoechinorhynchus tumidus	Coregonus clupeaformis	2	0.05	0.08	0.09	0.07
1	1	0.97	1	cv	1	Neoechinorhynchus tumidus	Prosopium cylindraceum	2	0.05	0.08	0.09	0.07
1	1	0.92	1	t	1	Neoechinorhynchus tumidus	Cottus cognatus	2	0.05	0.08	0.07	0.06
1	1	0.98	1	t	1	Neoechinorhynchus tumidus	Thymallus arcticus	2	0.05	0.08	0.10	0.07
1	1	0.96	1	t	1	Cystidicola farionis	Coregonus clupeaformis	2	0.05	0.08	0.09	0.07
1	1	0.96	1	cv	1	Cystidicola farionis	Prosopium cylindraceum	2	0.05	0.08	0.09	0.07
1	1	0.72	1	t	1	Cystidicola farionis	Catostomus catostomus	2	0.05	0.08	0.04	0.06
1	1	0.97	1	t	1	Cystidicola farionis	Thymallus arcticus	2	0.05	0.08	0.10	0.07
1	1	0.95	1	t	1	Coregonus clupeaformis	Triaenophorus crassus (L)	2	0.09	0.07	0.04	0.08

1	1	0.93	1	cv	1	Coregonus clupeaformis	Cyathocephalus truncatus	2	0.09	0.07	0.03	0.08
1	1	0.95	1	t	1	Coregonus clupeaformis	Piscicola milneri	2	0.09	0.07	0.04	0.08
1	1	0.95	1	t	1	Coregonus clupeaformis	Diphyllobothrium sp. (L)	2	0.09	0.07	0.04	0.08
1	1	0.98	1	t	1	Coregonus clupeaformis	Diplostomum spathaceum (L)	2	0.09	0.07	0.06	0.08
1	1	0.84	1	cv	1	Coregonus clupeaformis	Proteocephalus tumidocollus	2	0.09	0.07	0.01	0.08
0	1	0.84	1	t	1	Coregonus clupeaformis	Salmincola extensus	2	0.09	0.07	0.01	0.08
1	1	0.84	1	t	1	Coregonus clupeaformis	Discocotyle sagittata	2	0.09	0.07	0.01	0.08
1	1	0.97	1	t	1	Coregonus clupeaformis	Crepidostomum farionis	2	0.09	0.07	0.05	0.08
1	1	0.95	1	cv	1	Coregonus clupeaformis	Tetracotyle sp. (L)	2	0.09	0.07	0.04	0.08
1	1	0.89	1	t	1	Coregonus clupeaformis	Raphidascaris acus (L)	2	0.09	0.07	0.02	0.08
1	1	0.95	1	t	1	Triaenophorus crassus (L)	Prosopium cylindraceum	2	0.04	0.08	0.09	0.07
1	1	0.96	1	t	1	Triaenophorus crassus (L)	Thymallus arcticus	2	0.04	0.08	0.10	0.07
1	1	0.93	1	cv	1	Cyathocephalus truncatus	Prosopium cylindraceum	2	0.03	0.08	0.09	0.07
1	1	0.70	1	t	1	Dactylogyrus buddi	Cottus cognatus	2	0.01	0.07	0.07	0.06
1	1	0.95	1	t	1	Prosopium cylindraceum	Piscicola milneri	2	0.09	0.07	0.04	0.08
1	1	0.95	1	t	1	Prosopium cylindraceum	Diphyllobothrium sp. (L)	2	0.09	0.07	0.04	0.08
1	1	0.98	1	cv	1	Prosopium cylindraceum	Diplostomum spathaceum (L)	2	0.09	0.07	0.06	0.08
1	1	0.84	1	t	1	Prosopium cylindraceum	Proteocephalus tumidocollus	2	0.09	0.07	0.01	0.08
1	1	0.93	1	t	1	Prosopium cylindraceum	Capillaria salvelini	2	0.09	0.07	0.03	0.08
1	1	0.84	1	t	1	Prosopium cylindraceum	Discocotyle sagittata	2	0.09	0.07	0.01	0.08
1	1	0.97	1	cv	1	Prosopium cylindraceum	Crepidostomum farionis	2	0.09	0.07	0.05	0.08
1	1	0.95	1	t	1	Prosopium cylindraceum	Tetracotyle sp. (L)	2	0.09	0.07	0.04	0.08
0	1	0.84	1	t	1	Prosopium cylindraceum	Tetraonchus variabilis	2	0.09	0.07	0.01	0.08

1	1	0.70	1	t	1	Neascus sp. (L)	Cottus cognatus	2	0.01	0.07	0.07	0.06
0	0	0.43	1	cv	1	Anonchohaptor anomalus	Catostomus catostomus	2	0.01	0.07	0.04	0.06
1	1	0.88	1	t	1	Tetraonchus borealis	Thymallus arcticus	2	0.01	0.08	0.10	0.07
1	1	0.88	1	t	1	Salmincola thymalli	Thymallus arcticus	2	0.01	0.08	0.10	0.07
1	1	0.96	1	t	1	Diphyllobothrium sp. (L)	Thymallus arcticus	2	0.04	0.08	0.10	0.07
1	1	0.88	1	cv	1	Schistocephalus solidus (L)	Cottus cognatus	2	0.04	0.07	0.07	0.06
1	1	0.83	1	t	1	Catostomus catostomus	Diplostomum spathaceum (L)	2	0.04	0.06	0.06	0.08
0	0	0.41	1	t	1	Catostomus catostomus	Glaridacris catostomi	2	0.04	0.06	0.01	0.07
1	1	0.93	1	t	1	Diplostomum spathaceum	Cottus cognatus	2	0.06	0.08	0.07	0.06
1	1	0.98	1	cv	1	Diplostomum spathaceum	Thymallus arcticus	2	0.06	0.08	0.10	0.07
1	1	0.66	1	t	1	Proteocephalus tumidocollus	Cottus cognatus	2	0.01	0.08	0.07	0.06
1	1	0.88	1	t	1	Proteocephalus tumidocollus	Thymallus arcticus	2	0.01	0.08	0.10	0.07
1	1	0.94	1	t	1	Capillaria salvelini	Thymallus arcticus	2	0.03	0.08	0.10	0.07
1	1	0.88	1	cv	1	Discocotyle sagittata	Thymallus arcticus	2	0.01	0.08	0.10	0.07
1	1	0.92	1	t	1	Crepidostomum farionis	Cottus cognatus	2	0.05	0.08	0.07	0.06
1	1	0.98	1	t	1	Crepidostomum farionis	Thymallus arcticus	2	0.05	0.08	0.10	0.07
0	1	0.93	1	t	1	Cottus cognatus	Heterophyidmeta (L)	2	0.07	0.06	0.05	0.07
1	1	0.95	1	cv	1	Thymallus arcticus	Tetracotyle sp. (L)	2	0.10	0.07	0.04	0.08
1	1	0.91	1	t	1	Thymallus arcticus	Allocreadium isosporum	2	0.10	0.07	0.02	0.08
1	1	0.91	1	t	1	Thymallus arcticus	Raphidascaris acus (L)	2	0.10	0.07	0.02	0.08
0	0	0.21	0	t	1	Neascus sp. (L)	Allocreadium isosporum	2	0.01	0.07	0.02	0.08
0	0	0.15	0	cv	1	Dactylogyrus buddi	Triaenophorus crassus	2	0.01	0.07	0.01	0.08
0	0	0.41	0	t	1	Eubothrium salvelini	Allocreadium isosporum	2	0.04	0.08	0.02	0.08
1	1	0.66	0	t	1	Discocotyle sagittata	Cottus cognatus	2	0.01	0.08	0.07	0.06
0	0	0.12	0	t	1	Salmincola edwardsii	Salmincola extensus	2	0.01	0.08	0.01	0.08

0	0	0.41	0	cv	1	Cystidicola farionis	Anonchohaptor anomalus	2	0.05	0.08	0.01	0.07
0	0	0.37	0	t	1	Cystidicola farionis	Discocotyle sagittata	2	0.05	0.08	0.01	0.08
0	1	0.59	0	t	1	Triaenophorus crassus (L)	Piscicola milneri	2	0.04	0.08	0.04	0.08
0	0	0.13	0	t	1	Triaenophorus crassus	Tetraonchus variabilis	2	0.01	0.08	0.01	0.08
0	1	0.71	0	cv	1	Esox lucius	Dactylogyrus buddi	2	0.07	0.06	0.01	0.07
0	1	0.84	0	t	1	Salvelinus namaycush	Tetraonchus borealis	2	0.09	0.07	0.01	0.08
0	0	0.27	0	t	1	Cyathocephalus truncatus	Neascus sp. (L)	2	0.03	0.08	0.01	0.07
0	0	0.16	0	t	1	Anonchohaptor anomalus	Tetraonchus monenteron	2	0.01	0.07	0.01	0.08
0	0	0.30	0	cv	1	Eubothrium salvelini	Tetraonchus variabilis	2	0.04	0.08	0.01	0.08
0	1	0.68	0	t	1	Esox lucius	Salmincola thymalli	2	0.07	0.06	0.01	0.08
0	0	0.30	0	t	1	Triaenophorus crassus (L)	Salmincola extensus	2	0.04	0.08	0.01	0.08
0	0	0.39	0	t	1	Cystidicola farionis	Tetraonchus monenteron	2	0.05	0.08	0.01	0.08
0	1	0.51	0	cv	1	Salmincola extensus	Heterophyidmeta (L)	2	0.01	0.08	0.05	0.07
1	1	0.68	0	t	1	Tetraonchus monenteron	Cottus cognatus	2	0.01	0.08	0.07	0.06
1	0	0.48	0	t	1	Neoechinorhynchus tumidus	Glaridacris catostomi	2	0.05	0.08	0.01	0.07
0	0	0.13	0	t	1	Triaenophorus crassus	Proteocephalus tumidocollus	2	0.01	0.08	0.01	0.08
0	0	0.13	0	cv	1	Salmincola thymalli	Tetraonchus monenteron	2	0.01	0.08	0.01	0.08
0	0	0.12	0	t	1	Salmincola extensus	Discocotyle sagittata	2	0.01	0.08	0.01	0.08
0	0	0.18	0	t	1	Discocotyle sagittata	Raphidascaris acus (L)	2	0.01	0.08	0.02	0.08
0	0	0.18	0	t	1	Allocreadium isosporum	Tetraonchus variabilis	2	0.02	0.08	0.01	0.08
0	1	0.99	0	cv	1	Esox lucius	Thymallus arcticus	2	0.07	0.06	0.10	0.07
0	0	0.14	0	t	1	Dactylogyrus buddi	Tetraonchus borealis	2	0.01	0.07	0.01	0.08
0	1	0.54	0	t	1	Salmincola thymalli	Diplostomum spathaceum (L)	2	0.01	0.08	0.06	0.08
1	1	0.88	0	t	1	Salmincola extensus	Thymallus arcticus	2	0.01	0.08	0.10	0.07

0	0	0.14	0	cv	1	Dactylogyrus buddi	Proteocephalus tumidocollus	2	0.01	0.07	0.01	0.08
0	0	0.41	0	t	1	Cystidicola farionis	Neascus sp. (L)	2	0.05	0.08	0.01	0.07
0	0	0.32	0	t	1	Tetraonchus borealis	Tetracotyle sp. (L)	2	0.01	0.08	0.04	0.08
0	1	0.85	0	t	1	Salvelinus namaycush	Raphidascaris acus	2	0.09	0.07	0.01	0.08
0	0	0.13	0	cv	1	Salmincola thymalli	Triaenophorus crassus	2	0.01	0.08	0.01	0.08
0	0	0.32	0	t	1	Salmincola thymalli	Diphyllobothrium sp. (L)	2	0.01	0.08	0.04	0.08
0	1	0.66	0	t	1	Cystidicola farionis	Tetracotyle sp. (L)	2	0.05	0.08	0.04	0.08
0	0	0.20	0	t	1	Tetraonchus monenteron	Raphidascaris acus (L)	2	0.01	0.08	0.02	0.08
0	0	0.16	0	cv	1	Dactylogyrus buddi	Anonchohaptor anomalus	2	0.01	0.07	0.01	0.07
0	1	0.80	0	t	1	Schistocephalus solidus (L)	Heterophyid meta (L)	2	0.04	0.07	0.05	0.07
0	1	0.54	0	t	1	Raphidascaris acus	Heterophyid meta (L)	2	0.01	0.08	0.05	0.07
0	1	0.87	0	t	1	Diplostomum spathaceum	Crepidostomum farionis	2	0.06	0.08	0.05	0.08
0	1	0.78	0	cv	1	Piscicola milneri	Diplostomum spathaceum (L)	2	0.04	0.08	0.06	0.08
0	0	0.24	0	t	1	Cyathocephalus truncatus	Salmincola edwardsii	2	0.03	0.08	0.01	0.08
0	1	0.84	0	t	1	Prosopium cylindraceum	Salmincola extensus	2	0.09	0.07	0.01	0.08
1	1	0.81	0	t	1	Capillaria salvelini	Cottus cognatus	2	0.03	0.08	0.07	0.06
0	0	0.37	0	cv	1	Cystidicola farionis	Salmincola edwardsii	2	0.05	0.08	0.01	0.08
0	0	0.34	0	t	1	Eubothrium salvelini	Anonchohaptor anomalus	2	0.04	0.08	0.01	0.07
0	1	0.84	0	t	1	Prosopium cylindraceum	Tetraonchus borealis	2	0.09	0.07	0.01	0.08
1	0	0.48	0	t	1	Neoechinorhynchus tumidus	Anonchohaptor anomalus	2	0.05	0.08	0.01	0.07
0	0	0.32	0	cv	1	Proteocephalus tumidocollus	Tetracotyle sp. (L)	2	0.01	0.08	0.04	0.08
0	0	0.14	0	t	1	Glaridacris catostomi	Discocotyle sagittata	2	0.01	0.07	0.01	0.08
0	1	0.56	0	t	1	Crepidostomum farionis	Raphidascaris acus (L)	2	0.05	0.08	0.02	0.08
0	1	0.56	0	t	1	Dactylogyrus buddi	Heterophyid meta (L)	2	0.01	0.07	0.05	0.07

0	1	0.56	0	cv	1	Cyathocephalus truncatus	Schistocephalus solidus (L)	2	0.03	0.08	0.04	0.07
1	0	0.38	0	t	1	Salmincola edwardsii	Catostomus catostomus	2	0.01	0.08	0.04	0.06
0	0	0.19	0	t	1	Salmincola edwardsii	Allocreadium isosporum	2	0.01	0.08	0.02	0.08
0	0	0.44	0	t	1	Neoechinorhynchus tumidus	Tetraonchus variabilis	2	0.05	0.08	0.01	0.08
0	0	0.20	0	cv	1	Triaenophorus crassus	Raphidascaris acus (L)	2	0.01	0.08	0.02	0.08
0	1	0.54	0	t	1	Tetraonchus monenteron	Heterophyid meta (L)	2	0.01	0.08	0.05	0.07
0	0	0.22	0	t	1	Anonchohaptor anomalus	Allocreadium isosporum	2	0.01	0.07	0.02	0.08
0	1	0.52	0	t	1	Glaridacris catostomi	Crepidostomum farionis	2	0.01	0.07	0.05	0.08
0	0	0.15	0	cv	1	Neascus sp. (L)	Tetraonchus monenteron	2	0.01	0.07	0.01	0.08
0	0	0.36	0	t	1	Neascus sp. (L)	Piscicola milneri	2	0.01	0.07	0.04	0.08
0	1	0.66	0	t	1	Cystidicola farionis	Diphyllobothrium sp. (L)	2	0.05	0.08	0.04	0.08
0	0	0.15	0	t	1	Anonchohaptor anomalus	Salmincola extensus	2	0.01	0.07	0.01	0.08
0	0	0.14	0	cv	1	Neascus sp. (L)	Tetraonchus variabilis	2	0.01	0.07	0.01	0.08
0	1	0.86	0	t	1	Coregonus clupeaformis	Neascus sp. (L)	2	0.09	0.07	0.01	0.07
0	1	0.72	0	t	1	Neoechinorhynchus tumidus	Tetracotyle sp. (L)	2	0.05	0.08	0.04	0.08
0	0	0.33	0	t	1	Triaenophorus crassus (L)	Dactylogyrus buddi	2	0.04	0.08	0.01	0.07
0	0	0.30	0	cv	1	Eubothrium salvelini	Tetraonchus borealis	2	0.04	0.08	0.01	0.08
0	1	0.96	0	t	1	Salvelinus namaycush	Catostomus catostomus	2	0.09	0.07	0.04	0.06
0	0	0.36	0	t	1	Salmincola thymalli	Schistocephalus solidus (L)	2	0.01	0.08	0.04	0.07
0	0	0.42	0	t	1	Cyathocephalus truncatus	Capillaria salvelini	2	0.03	0.08	0.03	0.08
0	1	0.73	0	cv	1	Diphyllobothrium sp. (L)	Crepidostomum farionis	2	0.04	0.08	0.05	0.08
0	0	0.44	0	t	1	Neoechinorhynchus tumidus	Salmincola edwardsii	2	0.05	0.08	0.01	0.08
0	1	0.62	0	t	1	Diplostomum spathaceum	Raphidascaris acus (L)	2	0.06	0.08	0.02	0.08
0	0	0.41	0	t	1	Piscicola milneri	Raphidascaris acus (L)	2	0.04	0.08	0.02	0.08
0	1	0.61	0	cv	1	Heterophyid meta (L)	Raphidascaris acus (L)	2	0.05	0.07	0.02	0.08

0	0	0.48	1	t	1	Lota lota	Eubothrium rugosum	3	0.05	0.07	0.01	0.07
0	1	0.96	1	t	1	Lota lota	Haplonema hamulatum	3	0.05	0.07	0.09	0.07
0	0	0.50	1	t	1	Lota lota	Cyathocephalus truncatus	3	0.05	0.07	0.01	0.06
0	1	0.74	1	cv	1	Lota lota	Cystobranchus verrilli	3	0.05	0.07	0.04	0.06
0	1	0.93	1	t	1	Lota lota	Metechinorhynchus salmonis	3	0.05	0.07	0.07	0.06
0	1	0.75	1	t	1	Lota lota	Triaenophorus nodulosus (L)	3	0.05	0.07	0.04	0.06
0	1	0.76	1	t	1	Salvelinus namaycush	Eubothrium salvelini	3	0.06	0.05	0.02	0.05
0	1	0.66	1	cv	1	Salvelinus namaycush	Ergasilus auritus	3	0.06	0.05	0.01	0.06
0	1	0.67	1	t	1	Salvelinus namaycush	Pomphorhynchus bulbocolli	3	0.06	0.05	0.01	0.06
0	1	0.75	1	t	1	Salvelinus namaycush	Ergasilus nerkae	3	0.06	0.05	0.02	0.06
1	1	0.96	1	t	1	Salvelinus namaycush	Metechinorhynchus salmonis	3	0.06	0.05	0.07	0.06
0	1	0.85	1	cv	1	Salvelinus namaycush	Triaenophorus nodulosus (L)	3	0.06	0.05	0.04	0.06
0	1	0.82	1	t	1	Salvelinus namaycush	Diplostomum spathaceum (L)	3	0.06	0.05	0.03	0.05
0	1	0.76	1	t	1	Salvelinus namaycush	Raphidascaris acus (L)	3	0.06	0.05	0.02	0.05
0	0	0.30	1	t	1	Eubothrium salvelini	Oncorhynchus kisutch	3	0.02	0.05	0.01	0.05
0	0	0.31	1	cv	1	Bothriocephalus cuspidatus	Stizostedion vitreum	3	0.01	0.05	0.02	0.05
0	1	0.52	1	t	1	Cystidicola farionis	Coregonus clupeaformis	3	0.03	0.06	0.03	0.06
1	1	0.91	1	t	1	Cystidicola farionis	Coregonus artedii	3	0.03	0.06	0.08	0.06
0	0	0.35	1	t	1	Cystidicola farionis	Oncorhynchus kisutch	3	0.03	0.06	0.01	0.05
0	0	0.34	1	cv	1	Coregonus clupeaformis	Ergasilus auritus	3	0.03	0.06	0.01	0.06
0	0	0.33	1	t	1	Coregonus clupeaformis	Cyathocephalus truncatus	3	0.03	0.06	0.01	0.06
0	1	0.54	1	t	1	Coregonus clupeaformis	Proteocephalus exiguus	3	0.03	0.06	0.03	0.05

0	0	0.33	1	t	1	Coregonus clupeaformis	Diphyllobothrium sp. (L)	3	0.03	0.06	0.01	0.06
0	1	0.54	1	cv	1	Coregonus clupeaformis	Salmincola extumescens	3	0.03	0.06	0.03	0.05
0	1	0.92	1	t	1	Coregonus clupeaformis	Phyllodistomum coregoni	3	0.03	0.06	0.09	0.07
0	0	0.34	1	t	1	Coregonus clupeaformis	Pomphorhynchus bulbocolli	3	0.03	0.06	0.01	0.06
0	1	0.72	1	t	1	Coregonus clupeaformis	Salmincola extensus	3	0.03	0.06	0.05	0.06
0	0	0.43	1	cv	1	Coregonus clupeaformis	Ergasilus nerkae	3	0.03	0.06	0.02	0.06
0	1	0.52	1	t	1	Coregonus clupeaformis	Discocotyle sagittata	3	0.03	0.06	0.03	0.06
0	1	0.52	1	t	1	Coregonus clupeaformis	Crepidostomum farionis	3	0.03	0.06	0.03	0.06
0	1	0.87	1	t	1	Coregonus clupeaformis	Metechinorhynchus salmonis	3	0.03	0.06	0.07	0.06
0	1	0.52	1	cv	1	Coregonus clupeaformis	Cotylurus erraticus (L)	3	0.03	0.06	0.03	0.06
0	1	0.53	1	t	1	Coregonus clupeaformis	Diplostomum spathaceum (L)	3	0.03	0.06	0.03	0.05
0	0	0.21	1	t	1	Ergasilus auritus	Pungitius pungitius	3	0.01	0.06	0.01	0.05
1	1	0.83	1	t	1	Ergasilus auritus	Coregonus artedii	3	0.01	0.06	0.08	0.06
0	0	0.21	1	cv	1	Ergasilus auritus	Oncorhynchus kisutch	3	0.01	0.06	0.01	0.05
1	1	0.94	1	t	1	Triaenophorus crassus (L)	Coregonus artedii	3	0.04	0.06	0.08	0.06
0	0	0.25	1	t	1	Gyrodactylus sp.	Pungitius pungitius	3	0.01	0.04	0.01	0.05
1	1	0.82	1	t	1	Philonema agubernaculu	Coregonus artedii	3	0.01	0.06	0.08	0.06
0	0	0.21	1	cv	1	Philonema agubernaculu	Oncorhynchus kisutch	3	0.01	0.06	0.01	0.05
0	1	0.82	1	t	1	Cyathocephalus truncatus	Coregonus artedii	3	0.01	0.06	0.08	0.06
0	0	0.20	1	t	1	Cyathocephalus truncatus	Oncorhynchus kisutch	3	0.01	0.06	0.01	0.05
0	0	0.26	1	t	1	Ergasilus sp. A	Catostomus commersoni	3	0.01	0.05	0.01	0.05
0	0	0.34	1	cv	1	Ergasilus sp. A	Catostomus catostomus	3	0.01	0.05	0.02	0.05
0	0	0.29	1	t	1	Lissorchis attenuatum	Catostomus commersoni	3	0.01	0.04	0.01	0.05
0	0	0.42	1	t	1	Proteocephalus filicollis	Pungitius pungitius	3	0.04	0.06	0.01	0.05

1	1	0.93	1	t	1	Proteocephalus filicollis	Coregonus artedii	3	0.04	0.06	0.08	0.06
0	0	0.30	1	cv	1	Stizostedion vitreum	Proteocephalus sp. A	3	0.02	0.05	0.01	0.05
0	1	0.83	1	t	1	Stizostedion vitreum	Metechinorhynchus salmonis	3	0.02	0.05	0.07	0.06
0	1	0.54	1	t	1	Catostomus commersoni	Hunterella nodulosa	3	0.01	0.05	0.04	0.04
0	0	0.23	1	t	1	Catostomus commersoni	Pomphorhynchus bulbocolli	3	0.01	0.05	0.01	0.06
0	0	0.43	1	cv	1	Catostomus commersoni	Neoechinorhynchus strigosus	3	0.01	0.05	0.03	0.05
0	0	0.36	1	t	1	Catostomus commersoni	Rhabdochona cascadilla	3	0.01	0.05	0.02	0.04
0	1	0.79	1	t	1	Catostomus commersoni	Metechinorhynchus salmonis	3	0.01	0.05	0.07	0.06
0	0	0.36	1	t	1	Catostomus commersoni	Glaridacris sp.	3	0.01	0.05	0.02	0.04
0	1	0.68	1	cv	1	Apatemon gracilis (L)	Pungitius pungitius	3	0.06	0.05	0.01	0.05
1	1	0.82	1	t	1	Diphyllobothrium sp. (L)	Coregonus artedii	3	0.01	0.06	0.08	0.06
0	0	0.21	1	t	1	Diphyllobothrium sp. (L)	Oncorhynchus kisutch	3	0.01	0.06	0.01	0.05
0	0	0.20	1	t	1	Schistocephalus solidus (L)	Esox lucius	3	0.01	0.05	0.01	0.06
0	0	0.22	1	cv	1	Schistocephalus solidus (L)	Pungitius pungitius	3	0.01	0.05	0.01	0.05
0	0	0.22	1	t	1	Schistocephalus solidus (L)	Oncorhynchus kisutch	3	0.01	0.05	0.01	0.05
0	0	0.43	1	t	1	Catostomus catostomus	Rhabdochona cascadilla	3	0.02	0.05	0.02	0.04
0	1	0.84	1	t	1	Catostomus catostomus	Metechinorhynchus salmonis	3	0.02	0.05	0.07	0.06
0	0	0.43	1	cv	1	Catostomus catostomus	Glaridacris sp.	3	0.02	0.05	0.02	0.04
0	0	0.29	1	t	1	Proteocephalus pinguis	Esox lucius	3	0.02	0.05	0.01	0.06
0	1	0.62	1	t	1	Esox lucius	Triaenophorus crassus	3	0.01	0.06	0.05	0.05
0	0	0.37	1	t	1	Esox lucius	Neoechinorhynchus strigosus	3	0.01	0.06	0.03	0.05
0	1	0.82	1	cv	1	Esox lucius	Triaenophorus nodulosus	3	0.01	0.06	0.09	0.08

0	1	0.75	1	t	1	Esox lucius	Metechinorhynchus salmonis	3	0.01	0.06	0.07	0.06
0	0	0.20	1	t	1	Esox lucius	Raphidascaris acus	3	0.01	0.06	0.01	0.05
0	0	0.21	1	t	1	Pungitius pungitius	Pomphorhynchus bulbocolli	3	0.01	0.05	0.01	0.06
0	1	0.78	1	cv	1	Pungitius pungitius	Metechinorhynchus salmonis	3	0.01	0.05	0.07	0.06
0	0	0.44	1	t	1	Pungitius pungitius	Triaenophorus nodulosus (L)	3	0.01	0.05	0.04	0.06
0	0	0.36	1	t	1	Pungitius pungitius	Cotylurus erraticus (L)	3	0.01	0.05	0.03	0.06
0	0	0.38	1	t	1	Pungitius pungitius	Diplostomum spathaceum (L)	3	0.01	0.05	0.03	0.05
0	0	0.25	1	cv	1	Pungitius pungitius	Bunodera luciopercae	3	0.01	0.05	0.01	0.04
0	0	0.30	1	t	1	Pungitius pungitius	Raphidascaris acus (L)	3	0.01	0.05	0.02	0.05
0	1	0.81	1	t	1	Coregonus artedii	Pomphorhynchus bulbocolli	3	0.08	0.06	0.01	0.06
1	1	0.95	1	t	1	Coregonus artedii	Salmincola extensus	3	0.08	0.06	0.05	0.06
0	1	0.86	1	cv	1	Coregonus artedii	Ergasilus nerkae	3	0.08	0.06	0.02	0.06
0	1	0.90	1	t	1	Coregonus artedii	Discocotyle sagittata	3	0.08	0.06	0.03	0.06
0	1	0.90	1	t	1	Coregonus artedii	Crepidostomum farionis	3	0.08	0.06	0.03	0.06
1	1	0.98	1	t	1	Coregonus artedii	Metechinorhynchus salmonis	3	0.08	0.06	0.07	0.06
0	1	0.90	1	cv	1	Coregonus artedii	Cotylurus erraticus (L)	3	0.08	0.06	0.03	0.06
0	0	0.21	1	t	1	Pomphorhynchus bulbocolli	Oncorhynchus kisutch	3	0.01	0.06	0.01	0.05
0	0	0.28	1	t	1	Ergasilus nerkae	Oncorhynchus kisutch	3	0.02	0.06	0.01	0.05
0	0	0.40	1	t	1	Neoechinorhynchus	Oncorhynchus kisutch	3	0.03	0.05	0.01	0.05
0	0	0.35	1	cv	1	Discocotyle sagittata	Oncorhynchus kisutch	3	0.03	0.06	0.01	0.05
0	0	0.35	1	t	1	Crepidostomum farionis	Oncorhynchus kisutch	3	0.03	0.06	0.01	0.05

0	1	0.78	1	t	1	Oncorhynchus kisutch	Metechinorhynchus salmonis	3	0.01	0.05	0.07	0.06
0	0	0.45	1	t	1	Oncorhynchus kisutch	Triaenophorus nodulosus (L)	3	0.01	0.05	0.04	0.06
0	0	0.41	0	cv	1	Cystobranchus verrilli	Raphidascaris acus	3	0.04	0.06	0.01	0.05
0	1	0.57	0	t	1	Cystobranchus verrilli	Cotylurus erraticus (L)	3	0.04	0.06	0.03	0.06
0	1	0.89	0	t	1	Salvelinus namaycush	Hunterella nodulosa	3	0.06	0.05	0.04	0.04
0	1	0.97	0	t	1	Triaenophorus crassus	Triaenophorus nodulosus	3	0.05	0.05	0.09	0.08
0	0	0.41	0	cv	1	Philonema agubernaculu	Proteocephalus filicollis	3	0.01	0.06	0.04	0.06
0	1	0.73	0	t	1	Stizostedion vitreum	Triaenophorus crassus	3	0.02	0.05	0.05	0.05
0	1	0.65	0	t	1	Triaenophorus crassus (L)	Discocotyle sagittata	3	0.04	0.06	0.03	0.06
0	0	0.39	0	t	1	Ergasilus sp. A	Cotylurus erraticus (L)	3	0.01	0.05	0.03	0.06
0	1	0.89	0	cv	1	Triaenophorus crassus	Salmincola extensus	3	0.05	0.05	0.05	0.06
1	1	0.93	0	t	1	Apatemon gracilis (L)	Triaenophorus crassus	3	0.06	0.05	0.05	0.05
0	1	0.89	0	t	1	Neoechinorhynchus strigosus	Metechinorhynchus salmonis	3	0.03	0.05	0.07	0.06
0	0	0.30	0	t	1	Philonema agubernaculu	Proteocephalus pinguis	3	0.01	0.06	0.02	0.05
0	0	0.33	0	cv	1	Pungitius pungitius	Rhabdochona cascadilla	3	0.01	0.05	0.02	0.04
0	1	0.91	0	t	1	Proteocephalus pinguis	Phyllodistomum coregoni	3	0.02	0.05	0.09	0.07
0	1	0.68	0	t	1	Catostomus commersoni	Triaenophorus crassus	3	0.01	0.05	0.05	0.05
1	1	0.89	0	t	1	Catostomus catostomus	Coregonus artedii	3	0.02	0.05	0.08	0.06
0	0	0.36	0	cv	1	Diphyllobothrium sp. (L)	Salmincola extumescens	3	0.01	0.06	0.03	0.05
0	0	0.39	0	t	1	Lissorchis attenuatum	Rhabdochona cascadilla	3	0.01	0.04	0.02	0.04
0	1	0.84	0	t	1	Philonema agubernaculu	Phyllodistomum coregoni	3	0.01	0.06	0.09	0.07
0	0	0.33	0	t	1	Coregonus clupeaformis	Philonema agubernaculum	3	0.03	0.06	0.01	0.06
1	1	0.99	0	cv	1	Haplonema hamulatum	Phyllodistomum coregoni	3	0.09	0.07	0.09	0.07

0	0	0.19	0	t	1	Eubothrium rugosum	Raphidascaris acus	3	0.01	0.07	0.01	0.05
0	0	0.34	0	t	1	Bothriocephalus cuspidatus	Rhabdochona cascadilla	3	0.01	0.05	0.02	0.04
0	1	0.92	0	t	1	Phyllodistomum coregoni	Neoechinorhynchus strigosus	3	0.09	0.07	0.03	0.05
0	0	0.41	0	cv	1	Cystobranchus verrilli	Pungitius pungitius	3	0.04	0.06	0.01	0.05
0	0	0.31	0	t	1	Diphyllobothrium sp. (L)	Glaridacris sp.	3	0.01	0.06	0.02	0.04
0	1	0.95	0	t	1	Triaenophorus crassus (L)	Triaenophorus nodulosus	3	0.04	0.06	0.09	0.08
0	0	0.24	0	t	1	Gyrodactylus sp.	Pomphorhynchus bulbocolli	3	0.01	0.04	0.01	0.06
0	0	0.49	0	cv	1	Lota lota	Esox lucius	3	0.05	0.07	0.01	0.06
0	1	0.59	0	t	1	Triaenophorus nodulosus (L)	Cotylurus erraticus (L)	3	0.04	0.06	0.03	0.06
0	0	0.20	0	t	1	Cyathocephalus truncatus	Pungitius pungitius	3	0.01	0.06	0.01	0.05
0	0	0.33	0	t	1	Cyathocephalus truncatus	Crepidostomum farionis	3	0.01	0.06	0.03	0.06
0	1	0.55	0	cv	1	Salmincola extumescens	Diplostomum spathaceum (L)	3	0.03	0.05	0.03	0.05
0	1	0.84	0	t	1	Bothriocephalus cuspidatus	Coregonus artedii	3	0.01	0.05	0.08	0.06
1	1	0.98	0	t	1	Phyllodistomum coregoni	Metechinorhynchus salmonis	3	0.09	0.07	0.07	0.06
0	1	0.52	0	t	1	Proteocephalus sp. A	Hunterella nodulosa	3	0.01	0.05	0.04	0.04
0	1	0.51	0	cv	1	Triaenophorus crassus (L)	Catostomus commersoni	3	0.04	0.06	0.01	0.05
0	0	0.31	0	t	1	Proteocephalus pinguis	Pomphorhynchus bulbocolli	3	0.02	0.05	0.01	0.06
0	0	0.48	0	t	1	Cystidicola farionis	Glaridacris sp.	3	0.03	0.06	0.02	0.04
0	1	0.51	0	t	1	Hunterella nodulosa	Oncorhynchus kisutch	3	0.04	0.04	0.01	0.05
0	0	0.32	0	cv	1	Proteocephalus sp. A	Catostomus catostomus	3	0.01	0.05	0.02	0.05
0	0	0.41	0	t	1	Bothriocephalus cuspidatus	Neoechinorhynchus strigosus	3	0.01	0.05	0.03	0.05
0	0	0.31	0	t	1	Philonema agubernaculu	Glaridacris sp.	3	0.01	0.06	0.02	0.04
0	0	0.26	0	t	1	Eubothrium salvelini	Eubothrium rugosum	3	0.02	0.05	0.01	0.07

0	0	0.28	0	cv	1	Diphyllobothrium sp. (L)	Raphidascaris acus (L)	3	0.01	0.06	0.02	0.05
0	1	0.55	0	t	1	Philonema agubernaculu	Salmincola extensus	3	0.01	0.06	0.05	0.06
0	0	0.36	0	t	1	Schistocephalus solidus (L)	Discocotyle sagittata	3	0.01	0.05	0.03	0.06
0	1	0.78	0	t	1	Salvelinus namaycush	Rhabdochona cascadilla	3	0.06	0.05	0.02	0.04
1	1	0.81	0	cv	1	Cystidicola farionis	Apatemon gracilis (L)	3	0.03	0.06	0.06	0.05
0	1	0.58	0	t	1	Coregonus clupeaformis	Cystobranchus verrilli	3	0.03	0.06	0.04	0.06
0	0	0.27	0	t	1	Stizostedion vitreum	Esox lucius	3	0.02	0.05	0.01	0.06
0	1	0.59	0	t	1	Cystobranchus verrilli	Salmincola extumescens	3	0.04	0.06	0.03	0.05
0	0	0.30	0	cv	1	Esox lucius	Rhabdochona cascadilla	3	0.01	0.06	0.02	0.04
0	0	0.22	0	t	1	Schistocephalus solidus (L)	Raphidascaris acus	3	0.01	0.05	0.01	0.05
0	1	0.68	0	t	1	Salvelinus namaycush	Proteocephalus sp. A	3	0.06	0.05	0.01	0.05
0	0	0.38	0	t	1	Diphyllobothrium sp. (L)	Neoechinorhynchus strigosus	3	0.01	0.06	0.03	0.05
0	1	0.81	0	cv	1	Eubothrium rugosum	Triaenophorus nodulosus	3	0.01	0.07	0.09	0.08
0	0	0.28	0	t	1	Ergasilus auritus	Stizostedion vitreum	3	0.01	0.06	0.02	0.05
0	1	0.65	0	t	1	Cystobranchus verrilli	Triaenophorus nodulosus (L)	3	0.04	0.06	0.04	0.06
0	0	0.29	0	t	1	Diphyllobothrium sp. (L)	Catostomus catostomus	3	0.01	0.06	0.02	0.05
0	1	0.68	0	cv	1	Lota lota	Crepidostomum farionis	3	0.05	0.07	0.03	0.06
0	0	0.37	0	t	1	Cystobranchus verrilli	Esox lucius	3	0.04	0.06	0.01	0.06
0	1	0.81	0	t	1	Apatemon gracilis (L)	Diplostomum spathaceum (L)	3	0.06	0.05	0.03	0.05
0	1	0.88	0	t	1	Coregonus artedii	Rhabdochona cascadilla	3	0.08	0.06	0.02	0.04
0	1	0.74	0	cv	1	Triaenophorus crassus	Rhabdochona cascadilla	3	0.05	0.05	0.02	0.04
0	0	0.45	0	t	1	Eubothrium salvelini	Cystidicola farionis	3	0.02	0.05	0.03	0.06
0	0	0.40	0	t	1	Salmincola extumescens	Bunodera luciopercae	3	0.03	0.05	0.01	0.04

0	1	0.78	0	t	1	Proteocephalus sp. A	Metechinorhynchus salmonis	3	0.01	0.05	0.07	0.06
0	0	0.33	0	cv	1	Cystidicola farionis	Philonema agubernaculum	3	0.03	0.06	0.01	0.06
0	1	0.58	0	t	1	Glaridacris sp.	Triaenophorus nodulosus (L)	3	0.02	0.04	0.04	0.06
1	1	0.99	0	t	1	Haplonema hamulatum	Triaenophorus nodulosus	3	0.09	0.07	0.09	0.08
0	1	0.65	0	t	1	Schistocephalus solidus (L)	Triaenophorus crassus	3	0.01	0.05	0.05	0.05
0	1	0.57	0	cv	1	Cystobranchus verrilli	Discocotyle sagittata	3	0.04	0.06	0.03	0.06
0	1	0.91	0	t	1	Eubothrium salvelini	Haplonema hamulatum	3	0.02	0.05	0.09	0.07
0	0	0.23	0	t	1	Bothriocephalus cuspidatus	Schistocephalus solidus (L)	3	0.01	0.05	0.01	0.05
0	1	0.81	0	t	1	Triaenophorus nodulosus	Oncorhynchus kisutch	3	0.09	0.08	0.01	0.05
0	0	0.21	0	cv	1	Philonema agubernaculu	Proteocephalus sp. A	3	0.01	0.06	0.01	0.05
0	1	0.86	0	t	1	Ergasilus sp. A	Coregonus artedii	3	0.01	0.05	0.08	0.06
0	1	0.68	0	t	1	Salvelinus namaycush	Schistocephalus solidus (L)	3	0.06	0.05	0.01	0.05
1	1	0.91	0	t	1	Salvelinus namaycush	Salmincola extensus	3	0.06	0.05	0.05	0.06
0	1	0.76	0	cv	1	Salvelinus namaycush	Stizostedion vitreum	3	0.06	0.05	0.02	0.05
0	0	0.32	0	t	1	Pomphorhynchus bulbocolli	Glaridacris sp.	3	0.01	0.06	0.02	0.04
0	0	0.48	0	t	1	Proteocephalus exiguus	Catostomus catostomus	3	0.03	0.05	0.02	0.05
0	1	0.85	0	t	1	Schistocephalus solidus (L)	Phyllodistomum coregoni	3	0.01	0.05	0.09	0.07
1	1	0.91	0	cv	1	Apatemon gracilis (L)	Salmincola extensus	3	0.06	0.05	0.05	0.06
0	0	0.23	0	t	1	Gyrodactylus sp.	Cyathocephalus truncatus	3	0.01	0.04	0.01	0.06
0	1	0.59	0	t	1	Hunterella nodulosa	Ergasilus nerkae	3	0.04	0.04	0.02	0.06
0	0	0.43	0	t	1	Ergasilus nerkae	Cotylurus erraticus (L)	3	0.02	0.06	0.03	0.06
0	1	0.83	0	cv	1	Hunterella nodulosa	Salmincola extensus	3	0.04	0.04	0.05	0.06
0	0	0.23	0	t	1	Catostomus commersoni	Diphyllobothrium sp. (L)	3	0.01	0.05	0.01	0.06
0	1	0.60	0	t	1	Ergasilus sp. A	Salmincola extensus	3	0.01	0.05	0.05	0.06

0	0	0.45	1	t	1	Gyrodactylus macrochiri	Lepomis gibbosus	4	0.00	0.04	0.03	0.03
0	1	0.55	1	cv	1	Etheostoma exile	Gyrodactylus sp.	4	0.03	0.03	0.02	0.03
1	1	0.61	1	t	1	Etheostoma exile	Azygia angusticauda	4	0.03	0.03	0.02	0.03
1	1	0.63	1	t	1	Etheostoma exile	Contracaecum sp. (L)	4	0.03	0.03	0.02	0.04
1	1	0.61	1	t	1	Etheostoma exile	Camallanus oxycephalus	4	0.03	0.03	0.02	0.04
1	0	0.45	1	cv	1	Etheostoma exile	Argulus catostomi	4	0.03	0.03	0.01	0.04
1	1	0.70	1	t	1	Etheostoma exile	Leptorhynchoides thecatus (L)	4	0.03	0.03	0.03	0.03
0	0	0.40	1	t	1	Etheostoma exile	Neascus sp. (L)	4	0.03	0.03	0.00	0.04
0	1	0.57	1	t	1	Etheostoma exile	Rhabdochona cascadilla	4	0.03	0.03	0.02	0.03
1	1	0.77	1	cv	1	Etheostoma exile	Tetracotyle sp. (L)	4	0.03	0.03	0.04	0.04
0	0	0.47	1	t	1	Etheostoma exile	Apophallus itascensis (L)	4	0.03	0.03	0.01	0.03
1	1	0.83	1	t	1	Etheostoma exile	Diplostomulum sp. (L)	4	0.03	0.03	0.05	0.03
0	0	0.47	1	t	1	Etheostoma exile	Bothriocephalus formosus	4	0.03	0.03	0.01	0.03
0	0	0.31	1	cv	1	Alloglossidium corti	Noturus gyrinus	4	0.00	0.03	0.02	0.03
0	0	0.24	1	t	1	Crepidostomum isostomum	Percopsis omiscomaycus	4	0.01	0.03	0.01	0.03
0	0	0.47	1	t	1	Crepidostomum isostomum	Percina caprodes	4	0.01	0.03	0.03	0.03
0	0	0.24	1	t	1	Cleidodiscus aculeatus	Stizostedion canadense	4	0.01	0.03	0.00	0.03
0	0	0.26	1	cv	1	Cleidodiscus aculeatus	Stizostedion vitreum	4	0.01	0.03	0.01	0.03
0	0	0.48	1	t	1	Azygia longa	Esox lucius	4	0.00	0.04	0.04	0.04
1	1	0.76	1	t	1	Esox lucius	Leptorhynchoides thecatus	4	0.04	0.04	0.03	0.04
1	1	0.67	1	t	1	Esox lucius	Azygia angusticauda	4	0.04	0.04	0.02	0.03
0	0	0.49	1	cv	1	Esox lucius	Illinobdella sp.	4	0.04	0.04	0.01	0.04
0	0	0.49	1	t	1	Esox lucius	Uvulifer ambloplitis (L)	4	0.04	0.04	0.01	0.04
1	1	0.87	1	t	1	Esox lucius	Proteocephalus pinguis	4	0.04	0.04	0.05	0.04

0	0	0.46	1	t	1	Esox lucius	Triaenophorus crassus	4	0.04	0.04	0.00	0.04
1	1	0.78	1	cv	1	Esox lucius	Clinostomum marginatum (L)	4	0.04	0.04	0.04	0.03
0	0	0.49	1	t	1	Esox lucius	Tetraonchus monenteron	4	0.04	0.04	0.01	0.04
0	0	0.47	1	t	1	Esox lucius	Posthodiplostomum minimum (L)	4	0.04	0.04	0.00	0.03
0	0	0.46	1	t	1	Esox lucius	Triaenophorus nodulosus	4	0.04	0.04	0.00	0.04
1	1	0.67	1	cv	1	Esox lucius	Metechinorhynchus salmonis	4	0.04	0.04	0.02	0.03
1	1	0.81	1	t	1	Esox lucius	Tetracotyle sp. (L)	4	0.04	0.04	0.04	0.04
1	1	0.86	1	t	1	Esox lucius	Diplostomulum sp. (L)	4	0.04	0.04	0.05	0.03
0	1	0.53	1	t	1	Esox lucius	Neoechinorhynchus tenellum	4	0.04	0.04	0.01	0.04
0	1	0.61	1	cv	1	Esox lucius	Contracaecum brachyurum	4	0.04	0.04	0.02	0.03
0	0	0.24	1	t	1	Proteocephalus stizostethi	Stizostedion canadense	4	0.01	0.03	0.00	0.03
0	0	0.26	1	t	1	Proteocephalus stizostethi	Stizostedion vitreum	4	0.01	0.03	0.01	0.03
1	1	0.81	1	t	1	Leptorhynchoides thecatus	Micropterus dolomieui	4	0.03	0.04	0.04	0.03
1	1	0.81	1	cv	1	Leptorhynchoides thecatus	Pomoxis nigromaculatus	4	0.03	0.04	0.04	0.03
1	0	0.49	1	t	1	Leptorhynchoides thecatus	Moxostoma anisurum	4	0.03	0.04	0.01	0.03
1	0	0.46	1	t	1	Leptorhynchoides thecatus	Perca flavescens	4	0.03	0.04	0.00	0.03
1	0	0.46	1	t	1	Leptorhynchoides thecatus	Lota lota	4	0.03	0.04	0.00	0.03
1	1	0.79	1	cv	1	Leptorhynchoides thecatus	Ambloplites rupestris	4	0.03	0.04	0.04	0.03
1	1	0.79	1	t	1	Leptorhynchoides thecatus	Ictalurus nebulosus	4	0.03	0.04	0.04	0.03
1	1	0.57	1	t	1	Leptorhynchoides thecatus	Notropis anogenus	4	0.03	0.04	0.01	0.03
1	1	0.60	1	t	1	Leptorhynchoides thecatus	Noturus gyrinus	4	0.03	0.04	0.02	0.03
1	0	0.49	1	cv	1	Leptorhynchoides thecatus	Culaea inconstans	4	0.03	0.04	0.01	0.03

1	1	0.89	1	t	1	Leptorhynchoides thecatus	Catostomus commersoni	4	0.03	0.04	0.06	0.03
1	0	0.45	1	t	1	Leptorhynchoides thecatus	Etheostoma nigrum	4	0.03	0.04	0.00	0.03
1	1	0.73	1	t	1	Leptorhynchoides thecatus	Percina caprodes	4	0.03	0.04	0.03	0.03
1	1	0.74	1	cv	1	Leptorhynchoides thecatus	Lepomis gibbosus	4	0.03	0.04	0.03	0.03
0	1	0.56	1	t	1	Bothriocephalus sp.	Pomoxis nigromaculatus	4	0.00	0.04	0.04	0.03
1	0	0.47	1	t	1	Coregonus clupeaformis	Cystidicola stigmatura	4	0.03	0.03	0.01	0.03
1	1	0.50	1	t	1	Coregonus clupeaformis	Tetracotyle intermedia (L)	4	0.03	0.03	0.01	0.03
0	0	0.44	1	cv	1	Coregonus clupeaformis	Phyllodistomum coregoni	4	0.03	0.03	0.00	0.03
1	0	0.47	1	t	1	Coregonus clupeaformis	Neoechinorhynchus tumidum	4	0.03	0.03	0.01	0.03
1	1	0.50	1	t	1	Coregonus clupeaformis	Achtheres corpulentus	4	0.03	0.03	0.01	0.03
0	0	0.44	1	t	1	Coregonus clupeaformis	Proteocephalus exiguus	4	0.03	0.03	0.00	0.03
1	1	0.81	1	cv	1	Coregonus clupeaformis	Metechinorhynchus lateralis	4	0.03	0.03	0.04	0.04
0	0	0.44	1	t	1	Coregonus clupeaformis	Proteocephalus laruei	4	0.03	0.03	0.00	0.03
0	0	0.44	1	t	1	Coregonus clupeaformis	Cyathocephalus truncatus	4	0.03	0.03	0.00	0.03
0	0	0.44	1	t	1	Coregonus clupeaformis	Diphyllobothrium sp. (L)	4	0.03	0.03	0.00	0.03
1	0	0.47	1	cv	1	Coregonus clupeaformis	Discocotyle sagittata	4	0.03	0.03	0.01	0.03
1	1	0.64	1	t	1	Coregonus clupeaformis	Metechinorhynchus salmonis	4	0.03	0.03	0.02	0.03
1	1	0.53	1	t	1	Coregonus clupeaformis	Triaenophorus crassus (L)	4	0.03	0.03	0.01	0.03
1	1	0.84	1	t	1	Coregonus clupeaformis	Diplostomulum sp. (L)	4	0.03	0.03	0.05	0.03
1	0	0.33	1	cv	1	Allocreadium lobatum	Pimephales promelas	4	0.01	0.04	0.02	0.03
1	1	0.71	1	t	1	Allocreadium lobatum	Catostomus commersoni	4	0.01	0.04	0.06	0.03
0	0	0.32	1	t	1	Gyrodactylus sp.	Percopsis omiscomaycus	4	0.02	0.03	0.01	0.03

0	1	0.66	1	t	1	Gyrodactylus sp.	Ambloplites rupestris	4	0.02	0.03	0.04	0.03
0	0	0.40	1	cv	1	Gyrodactylus sp.	Notropis anogenus	4	0.02	0.03	0.01	0.03
0	0	0.43	1	t	1	Gyrodactylus sp.	Noturus gyrinus	4	0.02	0.03	0.02	0.03
0	0	0.30	1	t	1	Gyrodactylus sp.	Etheostoma nigrum	4	0.02	0.03	0.00	0.03
1	1	0.68	1	t	1	Plagioporus sinitsini	Catostomus commersoni	4	0.00	0.04	0.06	0.03
1	1	0.74	1	cv	1	Azygia angusticauda	Micropterus dolomieui	4	0.02	0.03	0.04	0.03
0	0	0.38	1	t	1	Azygia angusticauda	Stizostedion vitreum	4	0.02	0.03	0.01	0.03
0	0	0.35	1	t	1	Azygia angusticauda	Lota lota	4	0.02	0.03	0.00	0.03
1	1	0.71	1	t	1	Azygia angusticauda	Ambloplites rupestris	4	0.02	0.03	0.04	0.03
1	1	0.71	1	cv	1	Azygia angusticauda	Ictalurus nebulosus	4	0.02	0.03	0.04	0.03
0	0	0.35	1	t	1	Azygia angusticauda	Etheostoma nigrum	4	0.02	0.03	0.00	0.03
1	1	0.65	1	t	1	Azygia angusticauda	Lepomis gibbosus	4	0.02	0.03	0.03	0.03
0	0	0.32	1	t	1	Pimephales promelas	Dactylogyrus sp.	4	0.02	0.03	0.00	0.03
0	0	0.38	1	cv	1	Pimephales promelas	Biacetabulum sp.	4	0.02	0.03	0.01	0.03
0	0	0.31	1	t	1	Pimephales promelas	Neascus sp. (L)	4	0.02	0.03	0.00	0.04
0	0	0.31	1	t	1	Pimephales promelas	Posthodiplostomum minimum (L)	4	0.02	0.03	0.00	0.03
0	0	0.38	1	t	1	Pimephales promelas	Sanguinicola sp.	4	0.02	0.03	0.01	0.03
1	1	0.76	1	cv	1	Pimephales promelas	Diplostomulum sp. (L)	4	0.02	0.03	0.05	0.03
0	0	0.22	1	t	1	Cystidicola stigmatura	Coregonus artedii	4	0.01	0.03	0.00	0.03
0	0	0.21	1	t	1	Illinobdella sp.	Perca flavescens	4	0.01	0.04	0.00	0.03
1	0	0.25	1	t	1	Anonchohaptor anomalum	Moxostoma anisurum	4	0.01	0.04	0.01	0.03
1	0	0.31	1	cv	1	Anonchohaptor anomalum	Carpiodes cyprinus	4	0.01	0.04	0.01	0.03
1	1	0.74	1	t	1	Anonchohaptor anomalum	Catostomus commersoni	4	0.01	0.04	0.06	0.03
1	1	0.63	1	t	1	Contracaecum sp. (L)	Cottus bairdi	4	0.02	0.04	0.03	0.03
1	1	0.75	1	t	1	Contracaecum sp. (L)	Micropterus dolomieui	4	0.02	0.04	0.04	0.03

1	0	0.36	1	cv	1	Contracaecum sp. (L)	Perca flavescens	4	0.02	0.04	0.00	0.03
1	1	0.63	1	t	1	Contracaecum sp. (L)	Notropis hudsonius	4	0.02	0.04	0.03	0.03
1	1	0.73	1	t	1	Contracaecum sp. (L)	Ictalurus nebulosus	4	0.02	0.04	0.04	0.03
1	1	0.85	1	t	1	Contracaecum sp. (L)	Catostomus commersoni	4	0.02	0.04	0.06	0.03
0	0	0.36	1	cv	1	Contracaecum sp. (L)	Etheostoma nigrum	4	0.02	0.04	0.00	0.03
1	1	0.65	1	t	1	Contracaecum sp. (L)	Percina caprodes	4	0.02	0.04	0.03	0.03
1	1	0.66	1	t	1	Contracaecum sp. (L)	Lepomis gibbosus	4	0.02	0.04	0.03	0.03
1	0	0.26	1	t	1	Triganodistom um	Moxostoma anisurum	4	0.01	0.03	0.01	0.03
1	0	0.33	1	cv	1	Triganodistom um	Carpiodes cyprinus	4	0.01	0.03	0.01	0.03
0	0	0.22	1	t	1	Piscicola punctata	Lota lota	4	0.01	0.03	0.00	0.03
0	0	0.47	1	t	1	Piscicola punctata	Percina caprodes	4	0.01	0.03	0.03	0.03
0	0	0.26	1	t	1	Argulus versicolor	Stizostedion vitreum	4	0.01	0.03	0.01	0.03
0	0	0.25	1	cv	1	Argulus versicolor	Percopsis omiscomaycu s	4	0.01	0.03	0.01	0.03
1	1	0.59	1	t	1	Uvulifer ambloplitis (L)	Micropterus dolomieui	4	0.01	0.04	0.04	0.03
1	1	0.68	1	t	1	Neoechinorhy nchus	Catostomus commersoni	4	0.00	0.04	0.06	0.03
0	0	0.42	1	t	1	Dactylogyrus	Cottus bairdi	4	0.00	0.03	0.03	0.03
0	0	0.42	1	cv	1	Dactylogyrus sp.	Notropis hudsonius	4	0.00	0.03	0.03	0.03
0	0	0.24	1	t	1	Tetracotyle intermedia (L)	Coregonus artedii	4	0.01	0.03	0.00	0.03
1	1	0.71	1	t	1	Hunterella nodulosa	Catostomus commersoni	4	0.01	0.04	0.06	0.03
1	1	0.68	1	t	1	Neoechinorhy nchus crassus	Catostomus commersoni	4	0.00	0.04	0.06	0.03
1	1	0.74	1	cv	1	Proteocephalu s pearsei	Pomoxis nigromaculat us	4	0.02	0.03	0.04	0.03
0	0	0.35	1	t	1	Proteocephalu s pearsei	Perca flavescens	4	0.02	0.03	0.00	0.03
0	0	0.46	1	t	1	Proteocephalu s pearsei	Notropis atherinoides	4	0.02	0.03	0.01	0.03
1	0	0.47	1	t	1	Ligula intestinalis (L)	Notropis hudsonius	4	0.01	0.04	0.03	0.03
0	0	0.38	1	cv	1	Ligula intestinalis (L)	Pimephales notatus	4	0.01	0.04	0.02	0.03

1	1	0.74	1	t	1	Ligula intestinalis (L)	Catostomus commersoni	4	0.01	0.04	0.06	0.03
1	1	0.61	1	t	1	Cottus bairdi	Camallanus oxycephalus	4	0.03	0.03	0.02	0.04
0	0	0.41	1	t	1	Cottus bairdi	Gyrodactylus bairdi	4	0.03	0.03	0.00	0.03
0	1	0.57	1	cv	1	Cottus bairdi	Rhabdochona cascadilla	4	0.03	0.03	0.02	0.03
0	0	0.41	1	t	1	Cottus bairdi	Phyllodistom um undulans	4	0.03	0.03	0.00	0.03
0	1	0.61	1	t	1	Cottus bairdi	Metechinorh ynchus salmonis	4	0.03	0.03	0.02	0.03
1	1	0.77	1	t	1	Cottus bairdi	Tetracotyle sp. (L)	4	0.03	0.03	0.04	0.04
1	1	0.83	1	cv	1	Cottus bairdi	Diplostomulu m sp. (L)	4	0.03	0.03	0.05	0.03
0	0	0.44	1	t	1	Cottus bairdi	Neoechinorh ynchus rutili	4	0.03	0.03	0.01	0.03
0	0	0.40	1	t	1	Cottus bairdi	Proteocephal us ambloplitis (L)	4	0.03	0.03	0.00	0.03
0	0	0.41	1	t	1	Cottus bairdi	Schistocephal us solidus (L)	4	0.03	0.03	0.00	0.03
0	0	0.22	1	cv	1	Contracaecum brachyurum	Moxostoma anisurum	4	0.00	0.03	0.01	0.03
1	1	0.53	1	t	1	Contracaecum brachyurum	Ambloplites rupestris	4	0.00	0.03	0.04	0.03
0	0	0.23	1	t	1	Cystidicola cristivomeri	Salvelinus namaycush	4	0.00	0.03	0.01	0.03
1	0	0.24	1	t	1	Centrovarium lobotes	Stizostedion canadense	4	0.01	0.03	0.00	0.03
0	0	0.24	1	cv	1	Centrovarium lobotes	Perca flavescens	4	0.01	0.03	0.00	0.03
0	0	0.23	1	t	1	Triaenophorus stizostedionis (L)	Percopsis omiscomaycu s	4	0.00	0.03	0.01	0.03
0	0	0.22	1	t	1	Neoechinorhy nchus	Coregonus artedii	4	0.01	0.03	0.00	0.03
1	1	0.68	1	t	1	Glaridacris intermedius	Catostomus commersoni	4	0.00	0.04	0.06	0.03
1	1	0.73	1	cv	1	Micropterus dolomieui	Camallanus oxycephalus	4	0.04	0.03	0.02	0.04
1	1	0.62	1	t	1	Micropterus dolomieui	Crepidostom um cooperi	4	0.04	0.03	0.01	0.04
0	1	0.53	1	t	1	Micropterus dolomieui	Neochasmus umbellus	4	0.04	0.03	0.00	0.04
0	1	0.53	1	t	1	Micropterus dolomieui	Cleidodiscus banghami	4	0.04	0.03	0.00	0.04

1	1	0.83	1	cv	1	Micropterus dolomieui	Clinostomum marginatum (L)	4	0.04	0.03	0.04	0.03
0	1	0.53	1	t	1	Micropterus dolomieui	Microphallus opacus	4	0.04	0.03	0.00	0.04
1	1	0.56	1	t	1	Micropterus dolomieui	Ergasilus caeruleus	4	0.04	0.03	0.01	0.04
0	1	0.53	1	t	1	Micropterus dolomieui	Proteocephalus fluviatilis	4	0.04	0.03	0.00	0.04
1	1	0.85	1	cv	1	Micropterus dolomieui	Tetracotyle sp. (L)	4	0.04	0.03	0.04	0.04
1	1	0.60	1	t	1	Micropterus dolomieui	Ergasilus centrarchidarum	4	0.04	0.03	0.01	0.03
1	1	0.89	1	t	1	Micropterus dolomieui	Diplostomulum sp. (L)	4	0.04	0.03	0.05	0.03
1	1	0.57	1	t	1	Micropterus dolomieui	Neoechinorhynchus cylindratus	4	0.04	0.03	0.01	0.04
0	1	0.53	1	cv	1	Micropterus dolomieui	Actinocleidus fusiformis	4	0.04	0.03	0.00	0.04
0	1	0.53	1	t	1	Micropterus dolomieui	Proteocephalus ambloplitis	4	0.04	0.03	0.00	0.04
1	1	0.62	1	t	1	Micropterus dolomieui	Philometra sp.	4	0.04	0.03	0.01	0.04
0	1	0.53	1	t	1	Micropterus dolomieui	Caecincola parvus	4	0.04	0.03	0.00	0.04
1	0	0.34	1	cv	1	Camallanus oxycephalus	Stizostedion canadense	4	0.02	0.04	0.00	0.03
1	0	0.34	1	t	1	Camallanus oxycephalus	Perca flavescens	4	0.02	0.04	0.00	0.03
1	0	0.34	1	t	1	Camallanus oxycephalus	Lota lota	4	0.02	0.04	0.00	0.03
1	0	0.38	1	t	1	Camallanus oxycephalus	Culaea inconstans	4	0.02	0.04	0.01	0.03
1	1	0.83	1	cv	1	Camallanus oxycephalus	Catostomus commersoni	4	0.02	0.04	0.06	0.03
1	1	0.63	1	t	1	Camallanus oxycephalus	Percina caprodes	4	0.02	0.04	0.03	0.03
1	1	0.74	1	t	1	Argulus catostomi	Catostomus commersoni	4	0.01	0.04	0.06	0.03
0	0	0.22	1	t	1	Argulus catostomi	Etheostoma nigrum	4	0.01	0.04	0.00	0.03
0	0	0.26	1	cv	1	Triaenophorus nodulosus (L)	Stizostedion canadense	4	0.01	0.04	0.00	0.03
0	0	0.26	1	t	1	Triaenophorus nodulosus (L)	Perca flavescens	4	0.01	0.04	0.00	0.03
0	0	0.26	1	t	1	Triaenophorus nodulosus (L)	Lota lota	4	0.01	0.04	0.00	0.03

1	1	0.77	1	t	1	Triaenophorus nodulosus (L)	Catostomus commersoni	4	0.01	0.04	0.06	0.03
1	1	0.55	1	cv	1	Triaenophorus nodulosus (L)	Lepomis gibbosus	4	0.01	0.04	0.03	0.03
1	1	0.53	1	t	1	Ergasilus versicolor	Ictalurus nebulosus	4	0.00	0.03	0.04	0.03
1	1	0.76	1	t	1	Crepidostomum cooperi	Catostomus commersoni	4	0.01	0.04	0.06	0.03
1	1	0.53	1	t	1	Crepidostomum cooperi	Lepomis gibbosus	4	0.01	0.04	0.03	0.03
0	0	0.22	1	cv	1	Argulus canadensis	Stizostedion vitreum	4	0.00	0.03	0.01	0.03
0	0	0.20	1	t	1	Argulus canadensis	Perca flavescens	4	0.00	0.03	0.00	0.03
0	0	0.20	1	t	1	Argulus canadensis	Coregonus artedii	4	0.00	0.03	0.00	0.03
0	0	0.43	1	t	1	Argulus canadensis	Notropis hudsonius	4	0.00	0.03	0.03	0.03
1	0	0.39	1	cv	1	Gyrodactylus eucaliae	Culaea inconstans	4	0.02	0.02	0.01	0.03
1	0	0.26	1	t	1	Salvelinus namaycush	Eubothrium salvelini	4	0.01	0.03	0.01	0.03
0	0	0.23	1	t	1	Salvelinus namaycush	Proteocephalus parallacticus	4	0.01	0.03	0.00	0.03
1	0	0.39	1	t	1	Salvelinus namaycush	Metechinorhynchus salmonis	4	0.01	0.03	0.02	0.03
1	0	0.29	1	cv	1	Salvelinus namaycush	Triaenophorus crassus (L)	4	0.01	0.03	0.01	0.03
1	1	0.66	1	t	1	Salvelinus namaycush	Diplostomulum sp. (L)	4	0.01	0.03	0.05	0.03
0	0	0.23	1	t	1	Salvelinus namaycush	Salmincola siscowet	4	0.01	0.03	0.00	0.03
1	0	0.21	1	t	1	Gyrodactylus spathulatus	Moxostoma anisurum	4	0.00	0.04	0.01	0.03
1	1	0.68	1	cv	1	Gyrodactylus spathulatus	Catostomus commersoni	4	0.00	0.04	0.06	0.03
1	0	0.33	1	t	1	Octomacrum sp.	Couesius plumbeus	4	0.01	0.02	0.00	0.03
0	0	0.20	1	t	1	Bothriocephalus cuspidatus	Stizostedion canadense	4	0.00	0.03	0.00	0.03
0	0	0.22	1	t	1	Bothriocephalus cuspidatus	Stizostedion vitreum	4	0.00	0.03	0.01	0.03
0	0	0.20	1	cv	1	Bothriocephalus cuspidatus	Perca flavescens	4	0.00	0.03	0.00	0.03
0	1	0.53	1	t	1	Bothriocephalus cuspidatus	Ictalurus nebulosus	4	0.00	0.03	0.04	0.03
0	0	0.28	1	t	1	Bothriocephalus cuspidatus	Notropis atherinoides	4	0.00	0.03	0.01	0.03

1	0	0.43	1	t	1	Eubothrium rugosum	Lota lota	4	0.03	0.03	0.00	0.03
1	1	0.53	1	cv	1	Pomoxis nigromaculatu	Neascus sp. (L)	4	0.04	0.03	0.00	0.04
1	1	0.69	1	t	1	Pomoxis nigromaculatu	Spinitectus gracilis	4	0.04	0.03	0.02	0.03
1	1	0.56	1	t	1	Pomoxis nigromaculatu	Ergasilus caeruleus	4	0.04	0.03	0.01	0.04
0	1	0.54	1	t	1	Pomoxis nigromaculatu s	Posthodiplost omum minimum (L)	4	0.04	0.03	0.00	0.03
0	1	0.54	1	cv	1	Pomoxis nigromaculatu	Cleidodiscus capax	4	0.04	0.03	0.00	0.04
1	1	0.85	1	t	1	Pomoxis nigromaculatu	Tetracotyle sp. (L)	4	0.04	0.03	0.04	0.04
1	1	0.65	1	t	1	Pomoxis nigromaculatu	Diplostomum scheuringi	4	0.04	0.03	0.01	0.03
1	1	0.89	1	t	1	Pomoxis nigromaculatu	Diplostomulu m sp. (L)	4	0.04	0.03	0.05	0.03
1	1	0.54	1	cv	1	Pomoxis nigromaculatu s	Proteocephal us ambloplitis (L)	4	0.04	0.03	0.00	0.03
0	0	0.22	1	t	1	Stizostedion canadense	Argulus appendiculos us	4	0.00	0.03	0.00	0.03
0	0	0.21	1	t	1	Stizostedion canadense	Eustrongylide s sp. (L)	4	0.00	0.03	0.00	0.03
0	0	0.21	1	t	1	Stizostedion canadense	Illinobdella moorei	4	0.00	0.03	0.00	0.03
0	0	0.19	1	cv	1	Stizostedion canadense	Neascus sp. (L)	4	0.00	0.03	0.00	0.04
0	0	0.22	1	t	1	Stizostedion canadense	Bucephalus pusillus	4	0.00	0.03	0.00	0.03
0	0	0.50	1	t	1	Stizostedion canadense	Clinostomum marginatum (L)	4	0.00	0.03	0.04	0.03
0	0	0.21	1	t	1	Stizostedion canadense	Ergasilus caeruleus	4	0.00	0.03	0.01	0.04
0	0	0.36	1	cv	1	Stizostedion canadense	Metechinorh ynchus salmonis	4	0.00	0.03	0.02	0.03
0	1	0.55	1	t	1	Stizostedion canadense	Tetracotyle sp. (L)	4	0.00	0.03	0.04	0.04
0	1	0.63	1	t	1	Stizostedion canadense	Diplostomulu m sp. (L)	4	0.00	0.03	0.05	0.03
0	0	0.24	1	t	1	Stizostedion canadense	Neoechinorh ynchus tenellum	4	0.00	0.03	0.01	0.04

0	0	0.20	1	cv	1	Stizostedion canadense	Proteocephalus amblopIitis (L)	4	0.00	0.03	0.00	0.03
0	0	0.26	1	t	1	Stizostedion canadense	Phyllodistomum	4	0.00	0.03	0.01	0.03
0	0	0.30	1	t	1	Stizostedion canadense	Contracaecum brachyurum	4	0.00	0.03	0.02	0.03
0	0	0.37	1	t	1	Dactylogyrus bifurcatus	Pimephales notatus	4	0.01	0.03	0.02	0.03
1	0	0.21	1	cv	1	Diplostomum flexicaudum	Moxostoma anisurum	4	0.00	0.04	0.01	0.03
1	1	0.68	1	t	1	Diplostomum flexicaudum	Catostomus commersoni	4	0.00	0.04	0.06	0.03
0	0	0.20	1	t	1	Proteocephalus exiguus	Coregonus artedii	4	0.00	0.03	0.00	0.03
1	0	0.44	1	t	1	Triaenophorus stizostedionis	Stizostedion vitreum	4	0.02	0.02	0.01	0.03
0	0	0.23	1	cv	1	Eustrongylides sp. (L)	Stizostedion vitreum	4	0.00	0.03	0.01	0.03
0	0	0.21	1	t	1	Eustrongylides sp. (L)	Perca flavescens	4	0.00	0.03	0.00	0.03
0	0	0.23	1	t	1	Stizostedion vitreum	Illinobdella moorei	4	0.01	0.03	0.00	0.03
0	0	0.26	1	t	1	Stizostedion vitreum	Bunodera sacculata	4	0.01	0.03	0.01	0.03
0	1	0.53	1	cv	1	Stizostedion vitreum	Clinostomum marginatum (L)	4	0.01	0.03	0.04	0.03
0	0	0.24	1	t	1	Stizostedion vitreum	Ergasilus caeruleus	4	0.01	0.03	0.01	0.04
0	0	0.40	1	t	1	Stizostedion vitreum	Metechinorhynchus salmonis	4	0.01	0.03	0.02	0.03
0	1	0.58	1	t	1	Stizostedion vitreum	Tetracotyle sp. (L)	4	0.01	0.03	0.04	0.04
0	0	0.26	1	cv	1	Stizostedion vitreum	Ergasilus centrarchidarum	4	0.01	0.03	0.01	0.03
0	0	0.31	1	t	1	Stizostedion vitreum	Diplostomum scheuringi	4	0.01	0.03	0.01	0.03
1	1	0.66	1	t	1	Stizostedion vitreum	Diplostomulum sp. (L)	4	0.01	0.03	0.05	0.03
0	0	0.26	1	t	1	Stizostedion vitreum	Neoechinorhynchus tenellum	4	0.01	0.03	0.01	0.04
0	0	0.22	1	cv	1	Stizostedion vitreum	Proteocephalus amblopIitis (L)	4	0.01	0.03	0.00	0.03

0	0	0.28	1	t	1	Stizostedion vitreum	Phyllodistomum	4	0.01	0.03	0.01	0.03
0	0	0.33	1	t	1	Stizostedion vitreum	Contracaecum brachyurum	4	0.01	0.03	0.02	0.03
1	0	0.45	1	t	1	Ergasilus sp.	Coregonus artedii	4	0.03	0.03	0.00	0.03
1	1	0.71	1	cv	1	Ergasilus sp.	Notropis hudsonius	4	0.03	0.03	0.03	0.03
0	0	0.22	1	t	1	Moxostoma anisurum	Neascus sp. (L)	4	0.01	0.03	0.00	0.04
0	0	0.24	1	t	1	Moxostoma anisurum	Biacetabulum infrequens	4	0.01	0.03	0.00	0.03
0	0	0.35	1	t	1	Moxostoma anisurum	Spinitectus gracilis	4	0.01	0.03	0.02	0.03
0	0	0.24	1	cv	1	Moxostoma anisurum	Pseudomurraytrema sp.	4	0.01	0.03	0.00	0.03
0	0	0.24	1	t	1	Moxostoma anisurum	Ergasilus caeruleus	4	0.01	0.03	0.01	0.04
0	0	0.27	1	t	1	Moxostoma anisurum	Sanguinicola sp.	4	0.01	0.03	0.01	0.03
0	0	0.30	1	t	1	Moxostoma anisurum	Triaenophorus crassus (L)	4	0.01	0.03	0.01	0.03
0	0	0.23	1	cv	1	Moxostoma anisurum	Pseudomurraytrema copulatum	4	0.01	0.03	0.01	0.04
0	0	0.20	1	t	1	Neascus sp. (L)	Chrosomus neogaeus	4	0.00	0.04	0.00	0.03
0	0	0.41	1	t	1	Neascus sp. (L)	Notropis hudsonius	4	0.00	0.04	0.03	0.03
0	0	0.27	1	t	1	Neascus sp. (L)	Notropis anogenus	4	0.00	0.04	0.01	0.03
0	0	0.22	1	cv	1	Neascus sp. (L)	Culaea inconstans	4	0.00	0.04	0.01	0.03
1	1	0.70	1	t	1	Neascus sp. (L)	Catostomus commersoni	4	0.00	0.04	0.06	0.03
0	0	0.20	1	t	1	Neascus sp. (L)	Couesius plumbeus	4	0.00	0.04	0.00	0.03
1	0	0.43	1	t	1	Neascus sp. (L)	Percina caprodes	4	0.00	0.04	0.03	0.03
1	0	0.45	1	cv	1	Neascus sp. (L)	Lepomis gibbosus	4	0.00	0.04	0.03	0.03
0	0	0.22	1	t	1	Perca flavescens	Cleidodiscus adspectus	4	0.00	0.03	0.00	0.03
0	0	0.24	1	t	1	Perca flavescens	Bunodera sacculata	4	0.00	0.03	0.01	0.03
0	0	0.32	1	t	1	Perca flavescens	Spinitectus gracilis	4	0.00	0.03	0.02	0.03
0	0	0.50	1	cv	1	Perca flavescens	Clinostomum marginatum (L)	4	0.00	0.03	0.04	0.03

0	0	0.27	1	t	1	Perca flavescens	Dichelyne cotylophora	4	0.00	0.03	0.01	0.02
0	0	0.20	1	t	1	Perca flavescens	Cyathocephalus truncatus	4	0.00	0.03	0.00	0.03
0	0	0.21	1	t	1	Perca flavescens	Ergasilus caeruleus	4	0.00	0.03	0.01	0.04
0	1	0.55	1	cv	1	Perca flavescens	Tetracotyle sp. (L)	4	0.00	0.03	0.04	0.04
0	0	0.24	1	t	1	Perca flavescens	Apophallus itascensis (L)	4	0.00	0.03	0.01	0.03
0	0	0.28	1	t	1	Perca flavescens	Diplostomum scheuringi	4	0.00	0.03	0.01	0.03
0	0	0.22	1	t	1	Perca flavescens	Crassiphiala bulboglossa	4	0.00	0.03	0.00	0.03
0	1	0.63	1	cv	1	Perca flavescens	Diplostomulum sp. (L)	4	0.00	0.03	0.05	0.03
0	0	0.20	1	t	1	Perca flavescens	Proteocephalus ambloplitis (L)	4	0.00	0.03	0.00	0.03
0	0	0.26	1	t	1	Perca flavescens	Philometra sp.	4	0.00	0.03	0.01	0.04
0	0	0.22	1	t	1	Perca flavescens	Philometra cylindracea	4	0.00	0.03	0.00	0.03
0	0	0.26	1	cv	1	Perca flavescens	Phyllodistomum	4	0.00	0.03	0.01	0.03
0	0	0.47	1	t	1	Capillaria	Lota lota	4	0.03	0.03	0.00	0.03
0	0	0.32	1	t	1	Coregonus artedii	Spinitectus gracilis	4	0.00	0.03	0.02	0.03
0	0	0.20	1	t	1	Coregonus artedii	Proteocephalus laruei	4	0.00	0.03	0.00	0.03
0	0	0.20	1	cv	1	Coregonus artedii	Cyathocephalus truncatus	4	0.00	0.03	0.00	0.03
0	0	0.20	1	t	1	Coregonus artedii	Diphyllobothrium sp. (L)	4	0.00	0.03	0.00	0.03
0	0	0.22	1	t	1	Coregonus artedii	Discocotyle sagittata	4	0.00	0.03	0.01	0.03
0	0	0.36	1	t	1	Coregonus artedii	Metechinorhynchus salmonis	4	0.00	0.03	0.02	0.03
0	0	0.27	1	cv	1	Coregonus artedii	Triaenophorus crassus (L)	4	0.00	0.03	0.01	0.03
0	1	0.63	1	t	1	Coregonus artedii	Diplostomulum sp. (L)	4	0.00	0.03	0.05	0.03
0	0	0.22	1	t	1	Coregonus artedii	Phyllodistomum sp.	4	0.00	0.03	0.00	0.02
1	1	0.68	1	t	1	Glaridacris catostomi	Catostomus commersoni	4	0.00	0.04	0.06	0.03
1	1	0.53	1	cv	1	Crepidostomum ictaluri	Ictalurus nebulosus	4	0.00	0.03	0.04	0.03

1	1	0.84	1	t	1	Actinobdella triannulata	Catostomus commersoni	4	0.02	0.04	0.06	0.03
0	0	0.32	1	t	1	Rhabdochona cascadilla	Lota lota	4	0.02	0.03	0.00	0.03
0	1	0.58	1	t	1	Rhabdochona cascadilla	Notropis hudsonius	4	0.02	0.03	0.03	0.03
0	1	0.68	1	cv	1	Rhabdochona cascadilla	Ambloplites rupestris	4	0.02	0.03	0.04	0.03
0	0	0.42	1	t	1	Rhabdochona cascadilla	Notropis anogenus	4	0.02	0.03	0.01	0.03
0	0	0.31	1	t	1	Rhabdochona cascadilla	Etheostoma nigrum	4	0.02	0.03	0.00	0.03
1	1	0.56	1	t	1	Crepidostomum cornutum	Ambloplites rupestris	4	0.01	0.03	0.04	0.03
0	0	0.33	1	cv	1	Crepidostomum cornutum	Noturus gyrinus	4	0.01	0.03	0.02	0.03
0	0	0.31	1	t	1	Spinitectus gracilis	Lota lota	4	0.02	0.03	0.00	0.03
0	0	0.34	1	t	1	Spinitectus gracilis	Percopsis omiscomaycus	4	0.02	0.03	0.01	0.03
1	1	0.68	1	t	1	Spinitectus gracilis	Ictalurus nebulosus	4	0.02	0.03	0.04	0.03
1	1	0.53	1	cv	1	Cryptogonimus chilli	Ambloplites rupestris	4	0.00	0.03	0.04	0.03
1	0	0.33	1	t	1	Carpiodes cyprinus	Glaridacris confusus	4	0.01	0.03	0.01	0.02
0	0	0.39	1	t	1	Carpiodes cyprinus	Pomphorhynchus bulbocolli	4	0.01	0.03	0.02	0.04
1	1	0.53	1	t	1	Cleidodiscus pricei	Ictalurus nebulosus	4	0.00	0.03	0.04	0.03
1	1	0.75	1	cv	1	Capillaria catenata	Ambloplites rupestris	4	0.03	0.03	0.04	0.03
1	1	0.73	1	t	1	Clinostomum marginatum	Notropis hudsonius	4	0.04	0.03	0.03	0.03
1	1	0.81	1	t	1	Clinostomum marginatum	Ambloplites rupestris	4	0.04	0.03	0.04	0.03
1	1	0.81	1	t	1	Clinostomum marginatum	Ictalurus nebulosus	4	0.04	0.03	0.04	0.03
1	1	0.66	1	cv	1	Clinostomum marginatum	Pimephales notatus	4	0.04	0.03	0.02	0.03
0	0	0.47	1	t	1	Clinostomum marginatum	Etheostoma nigrum	4	0.04	0.03	0.00	0.03
1	1	0.76	1	t	1	Clinostomum marginatum	Lepomis gibbosus	4	0.04	0.03	0.03	0.03
0	0	0.27	1	t	1	Lota lota	Dichelyne cotylophora	4	0.00	0.03	0.01	0.02
0	0	0.36	1	cv	1	Lota lota	Metechinorhynchus salmonis	4	0.00	0.03	0.02	0.03

0	0	0.30	1	t	1	Lota lota	Pomphorhynchus bulbocolli	4	0.00	0.03	0.02	0.04
0	1	0.55	1	t	1	Lota lota	Tetracotyle sp. (L)	4	0.00	0.03	0.04	0.04
0	0	0.22	1	t	1	Lota lota	Ergasilus osburni	4	0.00	0.03	0.00	0.03
0	1	0.63	1	cv	1	Lota lota	Diplostomulum sp. (L)	4	0.00	0.03	0.05	0.03
0	0	0.30	1	t	1	Lota lota	Contracaecum brachyurum	4	0.00	0.03	0.02	0.03
1	1	0.73	1	t	1	Octospinifer macilentus	Catostomus commersoni	4	0.01	0.04	0.06	0.03
0	1	0.62	1	t	1	Neoechinorhynchus sp.	Notropis hudsonius	4	0.02	0.03	0.03	0.03
0	1	0.54	1	cv	1	Neoechinorhynchus sp.	Pimephales notatus	4	0.02	0.03	0.02	0.03
0	0	0.47	1	t	1	Neoechinorhynchus sp.	Notropis atherinoides	4	0.02	0.03	0.01	0.03
1	1	0.58	1	t	1	Percopsis omiscomaycus	Tetracotyle sp. (L)	4	0.01	0.03	0.04	0.04
1	1	0.66	1	t	1	Percopsis omiscomaycus	Diplostomulum sp. (L)	4	0.01	0.03	0.05	0.03
0	0	0.26	1	cv	1	Percopsis omiscomaycus	Bothriocephalus formosus	4	0.01	0.03	0.01	0.03
0	0	0.33	1	t	1	Percopsis omiscomaycus	Contracaecum brachyurum	4	0.01	0.03	0.02	0.03
1	1	0.73	1	t	1	Pellucidhaptor nasalis	Catostomus commersoni	4	0.01	0.04	0.06	0.03
1	1	0.71	1	t	1	Phyllodistomum staffordi	Ictalurus nebulosus	4	0.02	0.03	0.04	0.03
1	1	0.55	1	cv	1	Ergasilus caeruleus	Ambloplites rupestris	4	0.01	0.04	0.04	0.03
1	1	0.72	1	t	1	Ergasilus caeruleus	Catostomus commersoni	4	0.01	0.04	0.06	0.03
1	0	0.46	1	t	1	Ergasilus caeruleus	Percina caprodes	4	0.01	0.04	0.03	0.03
1	1	0.64	1	t	1	Bothriocephalus claviceps	Ambloplites rupestris	4	0.01	0.03	0.04	0.03
1	1	0.56	1	cv	1	Bothriocephalus claviceps	Lepomis gibbosus	4	0.01	0.03	0.03	0.03
1	0	0.40	1	t	1	Chrosomus neogaeus	Octomacrum semotili	4	0.00	0.03	0.02	0.02
0	0	0.20	1	t	1	Chrosomus neogaeus	Posthodiplostomum minimum (L)	4	0.00	0.03	0.00	0.03
0	0	0.22	1	t	1	Chrosomus neogaeus	Cleidodiscus brachus	4	0.00	0.03	0.00	0.03

0	1	0.56	1	cv	1	Chrosomus neogaeus	Tetracotyle sp. (L)	4	0.00	0.03	0.04	0.04
1	1	0.53	1	t	1	Spinitectus carolini	Ambloplites rupestris	4	0.00	0.03	0.04	0.03
1	0	0.44	1	t	1	Semotilus margarita	Octomacrum semotili	4	0.00	0.02	0.02	0.02
0	0	0.27	1	t	1	Bunoderina eucaliae	Culaea inconstans	4	0.01	0.03	0.01	0.03
0	0	0.42	1	cv	1	Posthodiplostomum minimum (L)	Notropis hudsonius	4	0.00	0.03	0.03	0.03
0	0	0.34	1	t	1	Posthodiplostomum minimum (L)	Pimephales notatus	4	0.00	0.03	0.02	0.03
0	0	0.44	1	t	1	Posthodiplostomum minimum (L)	Percina caprodes	4	0.00	0.03	0.03	0.03
0	0	0.45	1	t	1	Posthodiplostomum minimum (L)	Lepomis gibbosus	4	0.00	0.03	0.03	0.03
0	0	0.22	1	cv	1	Dactylogyrus mylocheilus	Couesius plumbeus	4	0.00	0.03	0.00	0.03
1	1	0.77	1	t	1	Notropis hudsonius	Tetracotyle sp. (L)	4	0.03	0.03	0.04	0.04
1	1	0.83	1	t	1	Notropis hudsonius	Diplostomulum sp. (L)	4	0.03	0.03	0.05	0.03
0	0	0.44	1	t	1	Cleidodiscus malleus	Percina caprodes	4	0.00	0.03	0.03	0.03
1	1	0.84	1	cv	1	Ambloplites rupestris	Tetracotyle sp. (L)	4	0.04	0.03	0.04	0.04
1	1	0.57	1	t	1	Ambloplites rupestris	Ergasilus centrarchidarum	4	0.04	0.03	0.01	0.03
1	1	0.88	1	t	1	Ambloplites rupestris	Diplostomulum sp. (L)	4	0.04	0.03	0.05	0.03
1	1	0.54	1	t	1	Ambloplites rupestris	Neoechinorhynchus cylindratus	4	0.04	0.03	0.01	0.04
0	1	0.51	1	cv	1	Ambloplites rupestris	Proteocephalus ambloplitis (L)	4	0.04	0.03	0.00	0.03
0	1	0.51	1	t	1	Ambloplites rupestris	Achtheres micropteri	4	0.04	0.03	0.00	0.03
1	1	0.68	1	t	1	Neoechinorhynchus	Catostomus commersoni	4	0.00	0.04	0.06	0.03
0	1	0.71	1	t	1	Metechinorhynchus	Ictalurus nebulosus	4	0.02	0.03	0.04	0.03
1	1	0.65	1	cv	1	Pomphorhynchus bulbocolli	Ictalurus nebulosus	4	0.02	0.04	0.04	0.03

1	0	0.42	1	t	1	Pomphorhynchus bulbocolli	Noturus gyrinus	4	0.02	0.04	0.02	0.03
1	1	0.80	1	t	1	Pomphorhynchus bulbocolli	Catostomus commersoni	4	0.02	0.04	0.06	0.03
1	1	0.57	1	t	1	Pomphorhynchus bulbocolli	Percina caprodes	4	0.02	0.04	0.03	0.03
1	1	0.83	1	cv	1	Tetracotyle sp. (L)	Ictalurus nebulosus	4	0.04	0.04	0.04	0.03
1	1	0.70	1	t	1	Tetracotyle sp. (L)	Pimephales notatus	4	0.04	0.04	0.02	0.03
1	1	0.56	1	t	1	Tetracotyle sp. (L)	Culaea inconstans	4	0.04	0.04	0.01	0.03
1	1	0.91	1	t	1	Tetracotyle sp. (L)	Catostomus commersoni	4	0.04	0.04	0.06	0.03
1	1	0.52	1	cv	1	Tetracotyle sp. (L)	Etheostoma nigrum	4	0.04	0.04	0.00	0.03
1	1	0.79	1	t	1	Tetracotyle sp. (L)	Lepomis gibbosus	4	0.04	0.04	0.03	0.03
0	0	0.24	1	t	1	Apophallus itascensis (L)	Etheostoma nigrum	4	0.01	0.03	0.00	0.03
1	1	0.88	1	t	1	Ictalurus nebulosus	Diplostomulum sp. (L)	4	0.04	0.03	0.05	0.03
1	1	0.54	1	cv	1	Ictalurus nebulosus	Corallobothrium fimbriatum	4	0.04	0.03	0.01	0.03
0	1	0.51	1	t	1	Ictalurus nebulosus	Proteocephalus ambloplitis (L)	4	0.04	0.03	0.00	0.03
0	1	0.54	1	t	1	Ictalurus nebulosus	Allocreadium ictaluri	4	0.04	0.03	0.01	0.03
0	1	0.51	1	t	1	Ictalurus nebulosus	Alloglossidium geminus	4	0.04	0.03	0.00	0.03
1	1	0.72	1	cv	1	Notropis anogenus	Diplostomulum sp. (L)	4	0.01	0.03	0.05	0.03
0	1	0.55	1	t	1	Diplostomum scheuringi	Percina caprodes	4	0.01	0.03	0.03	0.03
0	1	0.56	1	t	1	Diplostomum scheuringi	Lepomis gibbosus	4	0.01	0.03	0.03	0.03
1	1	0.68	1	t	1	Phyllodistomum lysteri	Catostomus commersoni	4	0.00	0.04	0.06	0.03
0	0	0.40	1	cv	1	Sanguinicola sp.	Pimephales notatus	4	0.01	0.03	0.02	0.03
1	1	0.76	1	t	1	Diplostomulum sp. (L)	Pimephales notatus	4	0.05	0.03	0.02	0.03
1	1	0.61	1	t	1	Diplostomulum sp. (L)	Couesius plumbeus	4	0.05	0.03	0.00	0.03
1	1	0.60	1	t	1	Diplostomulum sp. (L)	Etheostoma nigrum	4	0.05	0.03	0.00	0.03
1	1	0.83	1	cv	1	Diplostomulum sp. (L)	Percina caprodes	4	0.05	0.03	0.03	0.03

1	1	0.84	1	t	1	Diplostomulum sp. (L)	Lepomis gibbosus	4	0.05	0.03	0.03	0.03
1	1	0.71	1	t	1	Diplostomulum sp. (L)	Notropis atherinoides	4	0.05	0.03	0.01	0.03
1	1	0.68	1	t	1	Spiroxys sp (L)	Catostomus commersoni	4	0.00	0.04	0.06	0.03
0	0	0.33	1	cv	1	Corallobothrium fimbriatum	Noturus gyrinus	4	0.01	0.03	0.02	0.03
0	0	0.25	1	t	1	Neoechinorhynchus rutili	Culaea inconstans	4	0.01	0.03	0.01	0.03
0	0	0.22	1	t	1	Proteocephalus ambloplitis	Culaea inconstans	4	0.00	0.03	0.01	0.03
0	0	0.45	1	t	1	Proteocephalus ambloplitis	Lepomis gibbosus	4	0.00	0.03	0.03	0.03
0	0	0.23	1	cv	1	Culaea inconstans	Diplostomum baeri (L)	4	0.01	0.03	0.00	0.03
0	0	0.23	1	t	1	Culaea inconstans	Dactylogyrus eucaliae	4	0.01	0.03	0.00	0.03
1	1	0.65	1	t	1	Catostomus commersoni	Acolpenteron catostomi	4	0.06	0.03	0.00	0.04
1	1	0.65	1	t	1	Catostomus commersoni	Octomacrum lanceatum	4	0.06	0.03	0.00	0.04
1	1	0.69	1	cv	1	Catostomus commersoni	Pseudomurraytrema copulatum	4	0.06	0.03	0.01	0.04
0	1	0.50	1	t	1	Bothriocephalus formosus	Percina caprodes	4	0.01	0.03	0.03	0.03
0	0	0.40	1	t	1	Contracaecum brachyurum	Notropis atherinoides	4	0.02	0.03	0.01	0.03
0	0	0.25	0	t	1	Sanguinicola sp.	Salmincola siscowet	4	0.01	0.03	0.00	0.03
0	0	0.21	0	cv	1	Posthodiplostomum minimum (L)	Neoechinorhynchus cylindratus	4	0.00	0.03	0.01	0.04
0	0	0.39	0	t	1	Stizostedion canadense	Octomacrum semotili	4	0.00	0.03	0.02	0.02
0	0	0.37	0	t	1	Piscicola punctata	Pimephales notatus	4	0.01	0.03	0.02	0.03
0	0	0.35	0	t	1	Apophallus itascensis (L)	Contracaecum brachyurum	4	0.01	0.03	0.02	0.03
0	0	0.39	0	cv	1	Cleidodiscus adspectus	Phyllodistomum staffordi	4	0.00	0.03	0.02	0.03
0	0	0.19	0	t	1	Cleidodiscus banghami	Phyllodistomum undulans	4	0.00	0.04	0.00	0.03
1	0	0.40	0	t	1	Contracaecum sp. (L)	Moxostoma anisurum	4	0.02	0.04	0.01	0.03
0	0	0.34	0	t	1	Bothriocephalus sp.	Pimephales notatus	4	0.00	0.04	0.02	0.03
0	0	0.27	0	cv	1	Dactylogyrus bifurcatus	Sanguinicola sp.	4	0.01	0.03	0.01	0.03

0	0	0.24	0	t	1	Centrovarium lobotes	Pseudomurraytrema copulatum	4	0.01	0.03	0.01	0.04
1	0	0.23	0	t	1	Triganodistomum	Proteocephalus exiguus	4	0.01	0.03	0.00	0.03
0	0	0.35	0	t	1	Triaenophorus crassus	Neoechinorhynchus sp.	4	0.00	0.04	0.02	0.03
0	1	0.63	0	cv	1	Proteocephalus pinguis	Neoechinorhynchus cylindratus	4	0.05	0.04	0.01	0.04
0	0	0.20	0	t	1	Ergasilus versicolor	Illinobdella moorei	4	0.00	0.03	0.00	0.03
0	0	0.18	0	t	1	Glaridacris intermedius	Couesius plumbeus	4	0.00	0.04	0.00	0.03
0	0	0.25	0	t	1	Bunodera sacculata	Tetraonchus monenteron	4	0.01	0.03	0.01	0.04
1	0	0.35	0	cv	1	Gyrodactylus eucaliae	Cleidodiscus pricei	4	0.02	0.02	0.00	0.03
0	0	0.30	0	t	1	Gyrodactylus sp.	Eustrongylides sp. (L)	4	0.02	0.03	0.00	0.03
1	0	0.45	0	t	1	Argulus catostomi	Triaenophorus stizostedionis	4	0.01	0.04	0.02	0.02
0	0	0.19	0	t	1	Glaridacris catostomi	Ergasilus osburni	4	0.00	0.04	0.00	0.03
0	1	0.69	0	cv	1	Proteocephalus pinguis	Triaenophorus crassus (L)	4	0.05	0.04	0.01	0.03
0	0	0.21	0	t	1	Illinobdella sp.	Cleidodiscus malleus	4	0.01	0.04	0.00	0.03
0	0	0.21	0	t	1	Cleidodiscus brachus	Alloglossidium geminus	4	0.00	0.03	0.00	0.03
0	0	0.41	0	t	1	Pimephales notatus	Philometra sp.	4	0.02	0.03	0.01	0.04
0	0	0.23	0	cv	1	Pellucidhaptor nasalis	Diplostomum baeri (L)	4	0.01	0.04	0.00	0.03
1	0	0.38	0	t	1	Camallanus oxycephalus	Stizostedion vitreum	4	0.02	0.04	0.01	0.03
0	0	0.24	0	t	1	Dactylogyrus bifurcatus	Neoechinorhynchus cylindratus	4	0.01	0.03	0.01	0.04
0	0	0.27	0	t	1	Pomphorhynchus bulbocolli	Acolpenteron catostomi	4	0.02	0.04	0.00	0.04
0	0	0.22	0	cv	1	Dactylogyrus bifurcatus	Cryptogonimus chilli	4	0.01	0.03	0.00	0.03
0	0	0.23	0	t	1	Argulus canadensis	Culaea inconstans	4	0.00	0.03	0.01	0.03
0	0	0.22	0	t	1	Illinobdella moorei	Dactylogyrus mylocheilus	4	0.00	0.03	0.00	0.03
0	0	0.20	0	t	1	Proteocephalus ambloplitis	Diplostomum baeri (L)	4	0.00	0.03	0.00	0.03

0	0	0.23	0	cv	1	Cystidicola stigmatura	Diplostomum baeri (L)	4	0.01	0.03	0.00	0.03
1	1	0.55	0	t	1	Metechinorhynchus lateralis	Etheostoma nigrum	4	0.04	0.04	0.00	0.03
0	0	0.29	0	t	1	Illinobdella moorei	Carpiodes cyprinus	4	0.00	0.03	0.01	0.03
0	0	0.25	0	t	1	Triaenophorus stizostedionis (L)	Sanguinicola sp.	4	0.00	0.03	0.01	0.03
0	0	0.18	0	cv	1	Acolpenteron catostomi	Etheostoma nigrum	4	0.00	0.04	0.00	0.03
0	0	0.47	0	t	1	Leptorhynchoides thecatus	Pseudomurraytrema sp.	4	0.03	0.04	0.00	0.03
0	0	0.40	0	t	1	Proteocephalus pearsei	Eubothrium salvelini	4	0.02	0.03	0.01	0.03
0	0	0.50	0	t	1	Esox lucius	Argulus appendiculosus	4	0.04	0.04	0.00	0.03
0	0	0.23	0	cv	1	Salvelinus namaycush	Couesius plumbeus	4	0.01	0.03	0.00	0.03
0	0	0.35	0	t	1	Azygia angusticauda	Coregonus artedii	4	0.02	0.03	0.00	0.03
0	0	0.28	0	t	1	Bunoderina eucaliae	Apophallus itascensis (L)	4	0.01	0.03	0.01	0.03
0	0	0.35	0	t	1	Dactylogyrus bifurcatus	Spinitectus gracilis	4	0.01	0.03	0.02	0.03
1	1	0.76	0	cv	1	Notropis hudsonius	Ambloplites rupestris	4	0.03	0.03	0.04	0.03
0	0	0.28	0	t	1	Biacetabulum sp.	Bunodera sacculata	4	0.01	0.03	0.01	0.03
1	1	0.62	0	t	1	Actinobdella triannulata	Notropis hudsonius	4	0.02	0.04	0.03	0.03
0	0	0.23	0	t	1	Cleidodiscus capax	Ergasilus centrarchidarum	4	0.00	0.04	0.01	0.03
0	0	0.49	0	cv	1	Capillaria bakeri	Tetraonchus monenteron	4	0.03	0.03	0.01	0.04
1	0	0.26	0	t	1	Triganodistomum	Discocotyle sagittata	4	0.01	0.03	0.01	0.03
0	0	0.20	0	t	1	Triaenophorus crassus	Dactylogyrus eucaliae	4	0.00	0.04	0.00	0.03
0	0	0.42	0	t	1	Cottus bairdi	Eustrongylides sp. (L)	4	0.03	0.03	0.00	0.03
0	0	0.31	0	cv	1	Dactylogyrus eucaliae	Contracaecum brachyurum	4	0.00	0.03	0.02	0.03
1	0	0.31	0	t	1	Semotilus margarita	Triaenophorus crassus (L)	4	0.00	0.02	0.01	0.03
0	1	0.63	0	t	1	Proteocephalus pinguis	Philometra cylindracea	4	0.05	0.04	0.00	0.03

0	0	0.18	0	t	1	Bothriocephalus sp.	Octomacrum lanceatum	4	0.00	0.04	0.00	0.04
1	1	0.57	0	cv	1	Anonchohaptor anomalum	Ambloplites rupestris	4	0.01	0.04	0.04	0.03
0	0	0.22	0	t	1	Crassiphiala bulboglossa	Dactylogyrus eucaliae	4	0.00	0.03	0.00	0.03
0	0	0.20	0	t	1	Allocreadium lobatum	Microphallus opacus	4	0.01	0.04	0.00	0.04
1	0	0.25	0	t	1	Crepidostomum cooperi	Illinobdella moorei	4	0.01	0.04	0.00	0.03
1	0	0.26	0	cv	1	Dichelyne cotylophora	Phyllodistomum undulans	4	0.01	0.02	0.00	0.03
0	0	0.33	0	t	1	Spinitectus gracilis	Cleidodiscus brachus	4	0.02	0.03	0.00	0.03
0	0	0.21	0	t	1	Cystidicola cristivomeri	Achtheres micropteri	4	0.00	0.03	0.00	0.03
0	0	0.21	0	t	1	Dactylogyrus mylocheilus	Proteocephalus amploplitis	4	0.00	0.03	0.00	0.04
0	0	0.33	0	cv	1	Eustrongylides sp. (L)	Spinitectus gracilis	4	0.00	0.03	0.02	0.03
0	0	0.21	0	t	1	Moxostoma anisurum	Neoechinorhynchus cristatus	4	0.01	0.03	0.00	0.04
0	0	0.19	0	t	1	Bothriocephalus sp.	Neascus sp. (L)	4	0.00	0.04	0.00	0.04
0	1	0.68	0	t	1	Tetracotyle intermedia (L)	Diplostomulum sp. (L)	4	0.01	0.03	0.05	0.03
0	0	0.23	0	cv	1	Crepidostomum cornutum	Ergasilus caeruleus	4	0.01	0.03	0.01	0.04
0	0	0.20	0	t	1	Cleidodiscus banghami	Biacetabulum infrequens	4	0.00	0.04	0.00	0.03
1	0	0.19	0	t	1	Glaridacris catostomi	Salmincola siscowet	4	0.00	0.04	0.00	0.03
0	1	0.60	0	t	1	Biacetabulum sp.	Ambloplites rupestris	4	0.01	0.03	0.04	0.03
1	1	0.79	0	cv	1	Coregonus clupeaformis	Tetracotyle sp. (L)	4	0.03	0.03	0.04	0.04
0	0	0.41	0	t	1	Proteocephalus pearsei	Sanguinicola sp.	4	0.02	0.03	0.01	0.03
0	0	0.23	0	t	1	Percopsis omiscomaycus	Pseudomurraytrema copulatum	4	0.01	0.03	0.01	0.04
0	0	0.36	0	t	1	Neoechinorhynchus sp.	Etheostoma nigrum	4	0.02	0.03	0.00	0.03
0	0	0.43	0	cv	1	Cottus bairdi	Ergasilus osburni	4	0.03	0.03	0.00	0.03
0	0	0.31	0	t	1	Rhabdochona cascadilla	Proteocephalus laruei	4	0.02	0.03	0.00	0.03
0	0	0.22	0	t	1	Argulus catostomi	Cleidodiscus pricei	4	0.01	0.04	0.00	0.03

1	0	0.20	0	t	1	Hunterella nodulosa	Cyathocephalus truncatus	4	0.01	0.04	0.00	0.03
0	1	0.55	0	cv	1	Chrosomus neogaeus	Ambloplites rupestris	4	0.00	0.03	0.04	0.03
0	0	0.31	0	t	1	Noturus gyrinus	Diplostomum baeri (L)	4	0.02	0.03	0.00	0.03
0	1	0.70	0	t	1	Catostomus commersoni	Philometra cylindracea	4	0.06	0.03	0.00	0.03
0	0	0.48	0	t	1	Leptorhynchoides thecatus	Cleidodiscus adspectus	4	0.03	0.04	0.00	0.03
0	0	0.27	0	cv	1	Ergasilus centrarchidarum	Neoechinorhynchus tenellum	4	0.01	0.03	0.01	0.04
1	0	0.18	0	t	1	Neoechinorhynchus crassus	Schistocephalus solidus (L)	4	0.00	0.04	0.00	0.03
1	1	0.55	0	t	1	Diplostomum flexicaudum (L)	Metechinorhynchus lateralis	4	0.00	0.04	0.04	0.04
1	0	0.24	0	t	1	Hunterella nodulosa	Apophallus itascensis (L)	4	0.01	0.04	0.01	0.03
0	1	0.56	0	cv	1	Metechinorhynchus lateralis	Dactylogyrus eucaliae	4	0.04	0.04	0.00	0.03
0	1	0.65	0	t	1	Illinobdella moorei	Proteocephalus pinguis	4	0.00	0.03	0.05	0.04
1	1	0.51	0	t	1	Tetracotyle sp. (L)	Achtheres micropteri	4	0.04	0.04	0.00	0.03
0	0	0.19	0	t	1	Cystidicola cristivomeri	Diplostomum flexicaudum (L)	4	0.00	0.03	0.00	0.04
0	0	0.34	0	cv	1	Illinobdella sp.	Rhabdochona cascadilla	4	0.01	0.04	0.02	0.03
0	0	0.22	0	t	1	Bothriocephalus sp.	Moxostoma anisurum	4	0.00	0.04	0.01	0.03
1	0	0.26	0	t	1	Cleidodiscus aculeatus	Crepidostomum cornutum	4	0.01	0.03	0.01	0.03
1	0	0.33	0	t	1	Octospinifer macilentus	Noturus gyrinus	4	0.01	0.04	0.02	0.03
0	0	0.45	0	cv	1	Coregonus clupeaformis	Eustrongylides sp. (L)	4	0.03	0.03	0.00	0.03
0	0	0.19	0	t	1	Neoechinorhynchus crassus	Crassiphiala bulboglossa	4	0.00	0.04	0.00	0.03
0	0	0.20	0	t	1	Coregonus artedii	Pseudomurraytrema copulatum	4	0.00	0.03	0.01	0.04
0	0	0.22	0	t	1	Glaridacris catostomi	Glaridacris confusus	4	0.00	0.04	0.01	0.02
0	0	0.23	0	cv	1	Crepidostomum cornutum	Proteocephalus parallacticus	4	0.01	0.03	0.00	0.03
0	0	0.38	0	t	1	Camallanus oxycephalus	Pellucidhaptor nasalis	4	0.02	0.04	0.01	0.04

0	0	0.22	0	t	1	Actinocleidus fusiformis	Culaea inconstans	4	0.00	0.04	0.01	0.03
0	0	0.17	0	t	1	Azygia longa	Plagioporus sinitsini	4	0.00	0.04	0.00	0.04
1	1	0.68	0	cv	1	Spinitectus gracilis	Ambloplites rupestris	4	0.02	0.03	0.04	0.03
0	0	0.22	0	t	1	Argulus catostomi	Gyrodactylus bairdi	4	0.01	0.04	0.00	0.03
0	0	0.19	0	t	1	Bothriocephal us cuspidatus	Triaenophoru s crassus	4	0.00	0.03	0.00	0.04
0	0	0.39	0	t	1	Azygia longa	Capillaria catenata	4	0.00	0.04	0.03	0.03
0	0	0.43	0	cv	1	Alloglossidium corti	Cottus bairdi	4	0.00	0.03	0.03	0.03
0	0	0.49	0	t	1	Alloglossidium corti	Capillaria bakeri	4	0.00	0.03	0.03	0.03
0	0	0.20	0	t	1	Crepidostomu m isostomum	Octomacrum lanceatum	4	0.01	0.03	0.00	0.04
0	0	0.36	0	t	1	Achtheres corpulentus	Noturus gyrinus	4	0.01	0.03	0.02	0.03
0	0	0.25	0	cv	1	Proteocephalu s amblopIitis	Phyllodistom um	4	0.00	0.04	0.01	0.03
0	0	0.42	0	t	1	Leptorhynchoi des thecatus	Cleidodiscus capax	4	0.03	0.03	0.00	0.04
1	1	0.58	0	t	1	Pomoxis nigromaculatu s	Percopsis omiscomaycu s	4	0.04	0.03	0.01	0.03
0	0	0.25	0	t	1	Biacetabulum sp.	Lota lota	4	0.01	0.03	0.00	0.03
0	0	0.28	0	cv	1	Achtheres corpulentus	Bunoderina eucaliae	4	0.01	0.03	0.01	0.03
0	0	0.17	0	t	1	Neoechinorhy nchus crassus	Proteocephal us fluviatilis	4	0.00	0.04	0.00	0.04
0	0	0.28	0	t	1	Pellucidhaptor nasalis	Philometra sp.	4	0.01	0.04	0.01	0.04
0	0	0.25	0	t	1	Anonchohapto r anomalum	Salvelinus namaycush	4	0.01	0.04	0.01	0.03
0	1	0.56	0	cv	1	Cystidicola cristivomeri	Tetracotyle sp. (L)	4	0.00	0.03	0.04	0.04
0	0	0.25	0	t	1	Glaridacris intermedius	Triaenophoru s nodulosus (L)	4	0.00	0.04	0.01	0.04
0	0	0.22	0	t	1	Anonchohapto r anomalum	Contracaecu m brachyurum (L)	4	0.01	0.04	0.00	0.03
1	0	0.43	0	t	1	Leptorhynchoi des thecatus	Glaridacris catostomi	4	0.03	0.04	0.00	0.04
0	0	0.20	0	cv	1	Illinobdella moorei	Alloglossidiu m geminus	4	0.00	0.03	0.00	0.03

0	0	0.21	0	t	1	Gyrodactylus macrochiri	Dactylogyrus bifurcatus	4	0.00	0.04	0.01	0.03
1	0	0.37	0	t	1	Gyrodactylus eucaliae	Cleidodiscus brachus	4	0.02	0.02	0.00	0.03
1	0	0.24	0	t	1	Cleidodiscus pricei	Glaridacris confusus	4	0.00	0.03	0.01	0.02
0	0	0.20	0	cv	1	Triaenophorus nodulosus	Pseudomurraytrema copulatum	4	0.00	0.04	0.01	0.04
0	1	0.66	0	t	1	Uvulifer ambloplitis (L)	Proteocephalus pinguis	4	0.01	0.04	0.05	0.04
1	0	0.50	0	t	1	Argulus catostomi	Lepomis gibbosus	4	0.01	0.04	0.03	0.03
0	0	0.33	0	t	1	Bunoderina eucaliae	Notropis anogenus	4	0.01	0.03	0.01	0.03
0	0	0.25	0	cv	1	Couesius plumbeus	Bothriocephalus formosus	4	0.00	0.03	0.01	0.03
1	1	0.57	0	t	1	Leptorhynchoides thecatus (L)	Contracaecum brachyurum	4	0.03	0.03	0.02	0.03
0	0	0.33	0	t	1	Spinitectus gracilis	Ergasilus caeruleus	4	0.02	0.03	0.01	0.04
0	0	0.38	0	t	1	Contracaecum sp. (L)	Uvulifer ambloplitis (L)	4	0.02	0.04	0.01	0.04
0	0	0.22	0	cv	1	Argulus catostomi	Proteocephalus laruei	4	0.01	0.04	0.00	0.03
1	0	0.26	0	t	1	Dichelyne cotylophora	Achtheres micropteri	4	0.01	0.02	0.00	0.03
0	1	0.63	0	t	1	Proteocephalus exiguus	Diplostomulum sp. (L)	4	0.00	0.03	0.05	0.03
0	0	0.21	0	t	1	Argulus versicolor	Neoechinorhynchus cristatus	4	0.01	0.03	0.00	0.04
0	0	0.22	0	cv	1	Eustrongylides sp. (L)	Biacetabulum infrequens	4	0.00	0.03	0.00	0.03
0	1	0.52	0	t	1	Dactylogyrus bifurcatus	Capillaria bakeri	4	0.01	0.03	0.03	0.03
0	0	0.36	0	t	1	Chrosomus neogaeus	Pimephales notatus	4	0.00	0.03	0.02	0.03
1	1	0.57	0	t	1	Percopsis omiscomaycus	Ambloplites rupestris	4	0.01	0.03	0.04	0.03
0	0	0.24	0	cv	1	Biacetabulum sp.	Gyrodactylus bairdi	4	0.01	0.03	0.00	0.03
1	0	0.34	0	t	1	Camallanus oxycephalus	Gyrodactylus bairdi	4	0.02	0.04	0.00	0.03
0	0	0.35	0	t	1	Rhabdochona cascadilla	Culaea inconstans	4	0.02	0.03	0.01	0.03
1	1	0.53	0	t	1	Notropis hudsonius	Notropis atherinoides	4	0.03	0.03	0.01	0.03

1	0	0.33	0	cv	1	Octospinifer macilentus	Contracaecum brachyurum	4	0.01	0.04	0.02	0.03
0	0	0.26	0	t	1	Cleidodiscus aculeatus	Cystidicola stigmatura	4	0.01	0.03	0.01	0.03
1	0	0.46	0	t	1	Pomphorhynchus bulbocolli	Pimephales notatus	4	0.02	0.04	0.02	0.03
1	1	0.87	0	t	1	Pomoxis nigromaculatus	Metechinorhynchus lateralis	4	0.04	0.03	0.04	0.04
0	0	0.23	0	cv	1	Neoechinorhynchus tumidum	Proteocephalus parallacticus	4	0.01	0.03	0.00	0.03
0	0	0.18	0	t	1	Alloglossidium geminus	Octomacrum lanceatum	4	0.00	0.03	0.00	0.04
1	1	0.57	0	t	1	Hunterella nodulosa	Micropterus dolomieui	4	0.01	0.04	0.04	0.03
0	0	0.22	0	t	1	Contracaecum brachyurum	Salvelinus namaycush	4	0.00	0.03	0.01	0.03
0	0	0.18	0	cv	1	Octomacrum lanceatum	Etheostoma nigrum	4	0.00	0.04	0.00	0.03
1	0	0.44	0	t	1	Ergasilus sp.	Cleidodiscus pricei	4	0.03	0.03	0.00	0.03
0	0	0.20	0	t	1	Neochasmus umbellus	Diplostomum baeri (L)	4	0.00	0.04	0.00	0.03
1	0	0.23	0	t	1	Semotilus margarita	Cleidodiscus malleus	4	0.00	0.02	0.00	0.03
1	0	0.47	0	cv	1	Pseudomurraytrema	Lepomis gibbosus	4	0.01	0.04	0.03	0.03
0	0	0.20	0	t	1	Microphallus opacus	Salmincola siscowet	4	0.00	0.04	0.00	0.03
0	0	0.20	0	t	1	Allocreadium lobatum	Neochasmus umbellus	4	0.01	0.04	0.00	0.04
0	1	0.52	0	t	1	Bucephalus pusillus	Clinostomum marginatum (L)	4	0.00	0.03	0.04	0.03
0	1	0.54	0	cv	1	Micropterus dolomieui	Proteocephalus ambloplitis (L)	4	0.04	0.03	0.00	0.03
0	0	0.25	0	t	1	Triaenophorus nodulosus (L)	Caecincola parvus	4	0.01	0.04	0.00	0.04
0	0	0.22	0	t	1	Moxostoma anisurum	Proteocephalus ambloplitis	4	0.01	0.03	0.00	0.04
0	0	0.44	0	t	1	Tetraonchus monenteron	Notropis hudsonius	4	0.01	0.04	0.03	0.03
0	0	0.20	0	cv	1	Salvelinus namaycush	Acolpenteron catostomi	4	0.01	0.03	0.00	0.04
0	0	0.26	0	t	1	Uvulifer ambloplitis (L)	Crepidostomum cooperi	4	0.01	0.04	0.01	0.04

0	1	0.70	0	t	1	Gyrodactylus macrochiri	Catostomus commersoni	4	0.00	0.04	0.06	0.03
1	1	0.65	0	t	1	Diplostomulum sp. (L)	Neoechinorhynchus tenellum	4	0.05	0.03	0.01	0.04
0	0	0.48	0	cv	1	Clinostomum marginatum	Dactylogyrus eucaliae	4	0.04	0.03	0.00	0.03
0	0	0.22	0	t	1	Percopsis omiscomaycus	Diphyllobothrium sp. (L)	4	0.01	0.03	0.00	0.03
0	0	0.17	0	t	1	Neoechinorhynchus cristatus	Proteocephalus ambloplitis	4	0.00	0.04	0.00	0.04
0	0	0.26	0	t	1	Moxostoma anisurum	Semotilus margarita	4	0.01	0.03	0.00	0.02
0	0	0.26	0	cv	1	Proteocephalus exiguus	Philometra sp.	4	0.00	0.03	0.01	0.04
1	0	0.21	0	t	1	Cleidodiscus malleus	Crassiphiala bulboglossa	4	0.00	0.03	0.00	0.03
0	0	0.22	0	t	1	Bothriocephalus sp.	Stizostedion vitreum	4	0.00	0.04	0.01	0.03
1	0	0.27	0	t	1	Biacetabulum infrequens	Phyllodistomum	4	0.00	0.03	0.01	0.03
0	0	0.21	0	cv	1	Gyrodactylus macrochiri	Neoechinorhynchus cylindratus	4	0.00	0.04	0.01	0.04
0	0	0.32	0	t	1	Argulus canadensis	Octomacrum sp.	4	0.00	0.03	0.01	0.02
0	0	0.50	0	t	1	Capillaria bakeri	Discocotyle sagittata	4	0.03	0.03	0.01	0.03
0	1	0.65	0	t	1	Ambloplites rupestris	Noturus gyrinus	4	0.04	0.03	0.02	0.03
0	1	0.75	0	cv	1	Sanguinicola sp.	Catostomus commersoni	4	0.01	0.03	0.06	0.03
0	0	0.33	0	t	1	Metechinorhynchus	Acolpenteron catostomi	4	0.02	0.03	0.00	0.04
0	1	0.56	0	t	1	Micropterus dolomieui	Biacetabulum infrequens	4	0.04	0.03	0.00	0.03
0	0	0.22	0	t	1	Hunterella nodulosa	Bucephalus pusillus	4	0.01	0.04	0.00	0.03
0	0	0.28	0	cv	1	Achtheres corpulentus	Bunodera sacculata	4	0.01	0.03	0.01	0.03
0	0	0.26	0	t	1	Proteocephalus stizostethi	Moxostoma anisurum	4	0.01	0.03	0.01	0.03
0	1	0.61	0	t	1	Micropterus dolomieui	Biacetabulum sp.	4	0.04	0.03	0.01	0.03
0	0	0.19	0	t	1	Neascus sp. (L)	Proteocephalus ambloplitis (L)	4	0.00	0.04	0.00	0.03

1	0	0.44	0	cv	1	Ergasilus sp.	Posthodiplostomum minimum (L)	4	0.03	0.03	0.00	0.03
0	0	0.23	0	t	1	Sanguinicola sp.	Alloglossidium geminus	4	0.01	0.03	0.00	0.03
0	0	0.34	0	t	1	Bothriocephalus cuspidatus	Pimephales notatus	4	0.00	0.03	0.02	0.03
1	0	0.28	0	t	1	Crepidostomum cornutum	Phyllodistomum	4	0.01	0.03	0.01	0.03
1	0	0.46	0	cv	1	Azygia angusticauda	Notropis atherinoides	4	0.02	0.03	0.01	0.03
1	0	0.44	0	t	1	Coregonus clupeaformis	Cleidodiscus malleus	4	0.03	0.03	0.00	0.03
0	0	0.26	0	t	1	Cleidodiscus aculeatus	Pellucidhaptor nasalis	4	0.01	0.03	0.01	0.04
0	0	0.22	0	t	1	Culaea inconstans	Alloglossidium geminus	4	0.01	0.03	0.00	0.03
0	0	0.39	0	cv	1	Proteocephalus pearsei	Argulus catostomi	4	0.02	0.03	0.01	0.04
0	0	0.24	0	t	1	Perca flavescens	Neoechinorhynchus tenellum	4	0.00	0.03	0.01	0.04
1	1	0.85	0	t	1	Metechinorhynchus lateralis	Ambloplites rupestris	4	0.04	0.04	0.04	0.03
0	0	0.19	0	t	1	Cleidodiscus banghami	Cleidodiscus malleus	4	0.00	0.04	0.00	0.03
0	0	0.29	0	cv	1	Notropis anogenus	Salmincola siscowet	4	0.01	0.03	0.00	0.03
1	0	0.48	0	t	1	Eubothrium rugosum	Bunoderina eucaliae	4	0.03	0.03	0.01	0.03
1	0	0.38	0	t	1	Cottus bairdi	Glaridacris catostomi	4	0.03	0.03	0.00	0.04
0	0	0.22	0	t	1	Neoechinorhynchus	Dactylogyrus eucaliae	4	0.01	0.04	0.00	0.03
0	0	0.24	0	cv	1	Cystidicola cristivomeri	Semotilus margarita	4	0.00	0.03	0.00	0.02
0	0	0.23	0	t	1	Crepidostomum isostomum	Allocreadium ictaluri	4	0.01	0.03	0.01	0.03
0	0	0.20	0	t	1	Gyrodactylus spathulatus	Philometra cylindracea	4	0.00	0.04	0.00	0.03
0	0	0.35	0	t	1	Posthodiplostomum minimum (L)	Metechinorhynchus salmonis	4	0.00	0.03	0.02	0.03
0	0	0.26	0	cv	1	Crepidostomum cooperi	Ergasilus osburni	4	0.01	0.04	0.00	0.03
0	0	0.21	0	t	1	Azygia longa	Octospinifer macilentus	4	0.00	0.04	0.01	0.04
0	0	0.26	0	t	1	Dactylogyrus mylocheilus	Apophallus itascensis (L)	4	0.00	0.03	0.01	0.03
0	0	0.21	0	t	1	Dactylogyrus sp.	Chrosomus neogaeus	4	0.00	0.03	0.00	0.03

0	0	0.28	0	cv	1	Triaenophorus nodulosus (L)	Crepidostomum cornutum	4	0.01	0.04	0.01	0.03
1	0	0.23	0	t	1	Triganodistomum	Cryptogonimus chilli	4	0.01	0.03	0.00	0.03
1	0	0.22	0	t	1	Coregonus artedii	Cleidodiscus adspectus	4	0.00	0.03	0.00	0.03
0	0	0.24	0	t	1	Crepidostomum cooperi	Cryptogonimus chilli	4	0.01	0.04	0.00	0.03
0	0	0.29	0	cv	1	Gyrodactylus macrochiri	Pomphorhynchus bulbocolli	4	0.00	0.04	0.02	0.04
0	0	0.20	0	t	1	Neoechinorhynchus crassus	Bucephalus pusillus	4	0.00	0.04	0.00	0.03
0	0	0.23	0	t	1	Argulus versicolor	Etheostoma nigrum	4	0.01	0.03	0.00	0.03
0	0	0.22	0	t	1	Crepidostomum cornutum	Cyathocephalus truncatus	4	0.01	0.03	0.00	0.03
0	0	0.22	0	cv	1	Cleidodiscus aculeatus	Glaridacris catostomi	4	0.01	0.03	0.00	0.04
0	0	0.19	0	t	1	Triaenophorus crassus	Diphyllobothrium sp. (L)	4	0.00	0.04	0.00	0.03
0	0	0.18	0	t	1	Gyrodactylus macrochiri	Plagioporus sinitsini	4	0.00	0.04	0.00	0.04
0	0	0.21	0	t	1	Cleidodiscus adspectus	Proteocephalus ambloplitis	4	0.00	0.03	0.00	0.04
0	0	0.26	0	cv	1	Cystidicola stigmatura	Achtheres corpulentus	4	0.01	0.03	0.01	0.03
1	1	0.55	0	t	1	Azygia angusticauda	Metechinorhynchus salmonis	4	0.02	0.03	0.02	0.03
0	0	0.30	0	t	1	Crepidostomum isostomum	Carpiodes cyprinus	4	0.01	0.03	0.01	0.03
0	0	0.21	0	t	1	Argulus canadensis	Crassiphiala bulboglossa	4	0.00	0.03	0.00	0.03
0	1	0.63	0	cv	1	Proteocephalus stizostethi	Metechinorhynchus lateralis	4	0.01	0.03	0.04	0.04
0	0	0.21	0	t	1	Bucephalus pusillus	Actinocleidus fusiformis	4	0.00	0.03	0.00	0.04
1	0	0.42	0	t	1	Coregonus clupeaformis	Glaridacris intermedius	4	0.03	0.03	0.00	0.04
0	0	0.33	0	t	1	Bunoderina eucaliae	Notropis atherinoides	4	0.01	0.03	0.01	0.03
0	0	0.40	0	cv	1	Camallanus oxycephalus	Glaridacris confusus	4	0.02	0.04	0.01	0.02
0	0	0.18	0	t	1	Gyrodactylus spathulatus	Cleidodiscus pricei	4	0.00	0.04	0.00	0.03
0	0	0.19	0	t	1	Cyathocephalus truncatus	Caecincola parvus	4	0.00	0.03	0.00	0.04

0	0	0.20	0	t	1	Stizostedion canadense	Gyrodactylus bairdi	4	0.00	0.03	0.00	0.03
0	0	0.22	0	cv	1	Cryptogonimus chilli	Octospinifer macilentus	4	0.00	0.03	0.01	0.04
0	0	0.21	0	t	1	Argulus appendiculosu	Schistocephalus solidus (L)	4	0.00	0.03	0.00	0.03
1	0	0.20	0	t	1	Neoechinorhynchus	Neoechinorhynchus rutili	4	0.00	0.04	0.01	0.03
0	0	0.17	0	t	1	Neoechinorhynchus crassus	Cleidodiscus banghami	4	0.00	0.04	0.00	0.04
1	0	0.45	0	cv	1	Capillaria catenata	Apophallus itascensis (L)	4	0.03	0.03	0.01	0.03
0	0	0.23	0	t	1	Anonchohaptor anomalum	Biacetabulum infrequens	4	0.01	0.04	0.00	0.03
0	0	0.23	0	t	1	Piscicola punctata	Cleidodiscus adspectus	4	0.01	0.03	0.00	0.03
0	0	0.24	0	t	1	Argulus canadensis	Eubothrium salvelini	4	0.00	0.03	0.01	0.03
0	0	0.38	0	cv	1	Dichelyne cotylophora	Contracaecum brachyurum	4	0.01	0.02	0.02	0.03
0	0	0.19	0	t	1	Ergasilus versicolor	Phyllodistomum undulans	4	0.00	0.03	0.00	0.03
0	0	0.26	0	t	1	Alloglossidium corti	Phyllodistomum	4	0.00	0.03	0.01	0.03
0	1	0.51	0	t	1	Ictalurus nebulosus	Etheostoma nigrum	4	0.04	0.03	0.00	0.03
0	0	0.46	0	cv	1	Azygia angusticauda	Carpiodes cyprinus	4	0.02	0.03	0.01	0.03
0	0	0.31	0	t	1	Ergasilus versicolor	Spinitectus gracilis	4	0.00	0.03	0.02	0.03
0	1	0.85	0	t	1	Leptorhynchoides thecatus	Proteocephalus pinguis	4	0.03	0.03	0.05	0.04
0	0	0.21	0	t	1	Ligula intestinalis (L)	Acolpenteron catostomi	4	0.01	0.04	0.00	0.04
0	0	0.19	0	cv	1	Glaridacris catostomi	Crassiphiala bulboglossa	4	0.00	0.04	0.00	0.03
1	0	0.23	0	t	1	Triganodistomum	Alloglossidium geminus	4	0.01	0.03	0.00	0.03
0	0	0.44	0	t	1	Cottus bairdi	Dactylogyrus bifurcatus	4	0.03	0.03	0.01	0.03
0	0	0.21	0	t	1	Stizostedion vitreum	Glaridacris catostomi	4	0.01	0.03	0.00	0.04
0	0	0.18	0	cv	1	Proteocephalus exiguus	Glaridacris catostomi	4	0.00	0.03	0.00	0.04
0	0	0.22	0	t	1	Octospinifer macilentus	Achtheres micropteri	4	0.01	0.04	0.00	0.03
0	0	0.41	0	t	1	Proteocephalus pearsei	Apophallus itascensis (L)	4	0.02	0.03	0.01	0.03
0	0	0.23	0	t	1	Proteocephalus stizostethi	Caecincola parvus	4	0.01	0.03	0.00	0.04

0	0	0.20	0	cv	1	Triaenophorus crassus	Diplostomum baeri (L)	4	0.00	0.04	0.00	0.03
1	1	0.53	0	t	1	Spinitectus carolini	Ictalurus nebulosus	4	0.00	0.03	0.04	0.03
0	0	0.23	0	t	1	Bothriocephalus cuspidatus	Neoechinorhynchus tenellum	4	0.00	0.03	0.01	0.04
0	0	0.20	0	t	1	Cleidodiscus banghami	Ergasilus osburni	4	0.00	0.04	0.00	0.03
0	0	0.22	0	cv	1	Triaenophorus stizostedionis (L)	Crassiphiala bulboglossa	4	0.00	0.03	0.00	0.03
1	0	0.26	0	t	1	Glaridacris intermedius	Notropis anogenus	4	0.00	0.04	0.01	0.03
0	0	0.20	0	t	1	Spiroxys sp (L)	Corallobothrium fimbriatum	4	0.00	0.04	0.01	0.03
0	0	0.48	0	t	1	Neoechinorhynchus tumidum	Leptorhynchoides thecatus (L)	4	0.01	0.03	0.03	0.03
0	0	0.42	0	cv	1	Cleidodiscus pricei	Notropis hudsonius	4	0.00	0.03	0.03	0.03
1	0	0.28	0	t	1	Glaridacris catostomi	Contracaecum brachyurum	4	0.00	0.04	0.02	0.03
1	0	0.25	0	t	1	Triganodistomum	Cleidodiscus brachus	4	0.01	0.03	0.00	0.03
1	1	0.67	0	t	1	Ligula intestinalis (L)	Diplostomulum sp. (L)	4	0.01	0.04	0.05	0.03
0	0	0.49	0	cv	1	Azygia angusticauda	Noturus gyrinus	4	0.02	0.03	0.02	0.03
0	0	0.22	0	t	1	Stizostedion vitreum	Microphallus opacus	4	0.01	0.03	0.00	0.04
0	0	0.24	0	t	1	Argulus versicolor	Pseudomurraytrema copulatum	4	0.01	0.03	0.01	0.04
0	0	0.19	0	t	1	Cleidodiscus malleus	Proteocephalus ambloplitis (L)	4	0.00	0.03	0.00	0.03
0	1	0.60	0	cv	1	Pomoxis nigromaculatu	Eubothrium salvelini	4	0.04	0.03	0.01	0.03
1	1	0.51	0	t	1	Ergasilus sp.	Glaridacris confusus	4	0.03	0.03	0.01	0.02
0	0	0.20	0	t	1	Bucephalus pusillus	Phyllodistomum lysteri	4	0.00	0.03	0.00	0.04
0	0	0.24	0	t	1	Cryptogonimus chilli	Apophallus itascensis (L)	4	0.00	0.03	0.01	0.03
0	0	0.20	0	cv	1	Perca flavescens	Coregonus artedii	4	0.00	0.03	0.00	0.03

0	0	0.23	0	t	1	Cystidicola cristivomeri	Dactylogyrus bifurcatus	4	0.00	0.03	0.01	0.03
0	0	0.27	0	t	1	Neochasmus umbellus	Bothriocephalus claviceps	4	0.00	0.04	0.01	0.03
0	0	0.21	0	t	1	Cleidodiscus pricei	Phyllodistomum sp.	4	0.00	0.03	0.00	0.02
1	1	0.62	0	cv	1	Gyrodactylus macrochiri	Diplostomulum sp. (L)	4	0.00	0.04	0.05	0.03
0	0	0.22	0	t	1	Cystidicola stigmatura	Couesius plumbeus	4	0.01	0.03	0.00	0.03
1	1	0.62	0	t	1	Pomoxis nigromaculatu	Philometra sp.	4	0.04	0.03	0.01	0.04
0	0	0.22	0	t	1	Illinobdella moorei	Chrosomus neogaeus	4	0.00	0.03	0.00	0.03
1	0	0.38	0	cv	1	Gyrodactylus eucaliae	Allocreadium ictaluri	4	0.02	0.02	0.01	0.03
0	0	0.21	0	t	1	Bothriocephalus sp.	Cleidodiscus brachus	4	0.00	0.04	0.00	0.03
1	0	0.21	0	t	1	Biacetabulum infrequens	Phyllodistomum undulans	4	0.00	0.03	0.00	0.03
1	0	0.48	0	t	1	Leptorhynchoides thecatus	Neoechinorhynchus rutili	4	0.03	0.04	0.01	0.03
0	0	0.49	0	cv	1	Clinostomum marginatum (L)	Proteocephalus parallacticus	4	0.04	0.03	0.00	0.03
0	0	0.20	0	t	1	Proteocephalus parallacticus	Actinocleidus fusiformis	4	0.00	0.03	0.00	0.04
0	0	0.22	0	t	1	Perca flavescens	Cleidodiscus brachus	4	0.00	0.03	0.00	0.03
0	0	0.28	0	t	1	Uvulifer amploplitis (L)	Triaenophorus crassus (L)	4	0.01	0.04	0.01	0.03
0	0	0.31	0	cv	1	Percopsis omiscomaycus	Notropis anogenus	4	0.01	0.03	0.01	0.03
0	0	0.34	0	t	1	Actinobdella triannulata	Proteocephalus ambloplitis (L)	4	0.02	0.04	0.00	0.03
0	0	0.22	0	t	1	Bothriocephalus cuspidatus	Phyllodistomum sp.	4	0.00	0.03	0.00	0.02
0	1	0.79	0	t	1	Proteocephalus pearsei	Proteocephalus pinguis	4	0.02	0.03	0.05	0.04
0	0	0.40	0	cv	1	Gyrodactylus sp.	Notropis atherinoides	4	0.02	0.03	0.01	0.03
0	0	0.18	0	t	1	Diplostomum flexicaudum	Alloglossidium geminus	4	0.00	0.04	0.00	0.03
0	1	0.55	0	t	1	Proteocephalus parallacticus	Ictalurus nebulosus	4	0.00	0.03	0.04	0.03
0	0	0.24	0	t	1	Biacetabulum sp.	Coregonus artedii	4	0.01	0.03	0.00	0.03
0	0	0.23	0	cv	1	Allocreadium lobatum	Pellucidhaptor nasalis	4	0.01	0.04	0.01	0.04

0	0	0.27	0	t	1	Argulus versicolor	Bunoderina eucaliae	4	0.01	0.03	0.01	0.03
0	0	0.34	0	t	1	Gyrodactylus sp.	Eubothrium salvelini	4	0.02	0.03	0.01	0.03
1	0	0.21	0	t	1	Glaridacris catostomi	Neoechinorhynchus tenellum	4	0.00	0.04	0.01	0.04
0	0	0.24	0	cv	1	Crepidostomum isostomum	Pellucidhaptor nasalis	4	0.01	0.03	0.01	0.04
0	0	0.25	0	t	1	Proteocephalus stizostethi	Allocreadium ictaluri	4	0.01	0.03	0.01	0.03
0	0	0.21	0	t	1	Cyathocephalus truncatus	Philometra cylindracea	4	0.00	0.03	0.00	0.03
1	0	0.50	0	t	1	Camallanus oxycephalus	Spinitectus gracilis	4	0.02	0.04	0.02	0.03
1	0	0.27	0	cv	1	Dichelyne cotylophora	Chrosomus neogaeus	4	0.01	0.02	0.00	0.03
0	0	0.22	0	t	1	Stizostedion vitreum	Coregonus artedii	4	0.01	0.03	0.00	0.03
0	0	0.44	0	t	1	Chrosomus neogaeus	Notropis hudsonius	4	0.00	0.03	0.03	0.03
1	1	0.62	0	t	1	Cryptogonimus chilli	Diplostomulum sp. (L)	4	0.00	0.03	0.05	0.03
0	0	0.22	0	cv	1	Salvelinus namaycush	Proteocephalus exiguus	4	0.01	0.03	0.00	0.03
0	0	0.20	0	t	1	Azygia longa	Salmincola siscowet	4	0.00	0.04	0.00	0.03
0	1	0.60	0	t	1	Biacetabulum sp.	Ictalurus nebulosus	4	0.01	0.03	0.04	0.03
0	0	0.23	0	t	1	Ligula intestinalis (L)	Dactylogyrus eucaliae	4	0.01	0.04	0.00	0.03
0	0	0.35	0	cv	1	Neoechinorhynchus sp.	Schistocephalus solidus (L)	4	0.02	0.03	0.00	0.03
0	0	0.21	0	t	1	Allocreadium ictaluri	Etheostoma nigrum	4	0.01	0.03	0.00	0.03
0	0	0.24	0	t	1	Biacetabulum sp.	Neascus sp. (L)	4	0.01	0.03	0.00	0.04
0	0	0.17	0	t	1	Plagioporus sinitsini	Octomacrum lanceatum	4	0.00	0.04	0.00	0.04
0	0	0.26	0	cv	1	Centrovarium lobotes	Phyllodistomum sp.	4	0.01	0.03	0.00	0.02
1	0	0.38	0	t	1	Actinobdella triannulata	Neoechinorhynchus rutili	4	0.02	0.04	0.01	0.03
0	0	0.21	0	t	1	Argulus appendiculosu	Cleidodiscus capax	4	0.00	0.03	0.00	0.04
0	0	0.20	0	t	1	Spinitectus carolini	Couesius plumbeus	4	0.00	0.03	0.00	0.03
0	0	0.17	0	cv	1	Gyrodactylus spathulatus	Cleidodiscus banghami	4	0.00	0.04	0.00	0.04
1	1	0.62	0	t	1	Pimephales promelas	Ergasilus sp.	4	0.02	0.03	0.03	0.03

0	0	0.40	0	t	1	Capillaria catenata	Diplostomum baeri (L)	4	0.03	0.03	0.00	0.03
0	0	0.20	0	t	1	Actinocleidus fusiformis	Diplostomum baeri (L)	4	0.00	0.04	0.00	0.03
0	0	0.26	0	cv	1	Bothriocephalus sp.	Triaenophorus crassus (L)	4	0.00	0.04	0.01	0.03
0	0	0.23	0	t	1	Discocotyle sagittata	Dactylogyrus eucaliae	4	0.01	0.03	0.00	0.03
0	0	0.18	0	t	1	Bothriocephalus sp.	Phyllodistomum lysteri	4	0.00	0.04	0.00	0.04
0	0	0.24	0	t	1	Proteocephalus exiguus	Apophallus itascensis (L)	4	0.00	0.03	0.01	0.03
0	0	0.19	0	cv	1	Dactylogyrus sp.	Actinocleidus fusiformis	4	0.00	0.03	0.00	0.04
1	0	0.39	0	t	1	Triaenophorus stizostedionis	Cleidodiscus banghami	4	0.02	0.02	0.00	0.04
0	0	0.25	0	t	1	Cleidodiscus aculeatus	Illinobdella sp.	4	0.01	0.03	0.01	0.04
0	0	0.19	0	t	1	Dactylogyrus sp.	Proteocephalus fluviatilis	4	0.00	0.03	0.00	0.04
0	0	0.25	0	cv	1	Argulus appendiculosu	Moxostoma anisurum	4	0.00	0.03	0.01	0.03
1	1	0.63	0	t	1	Ambloplites rupestris	Notropis anogenus	4	0.04	0.03	0.01	0.03
0	0	0.45	0	t	1	Stizostedion canadense	Percina caprodes	4	0.00	0.03	0.03	0.03
0	0	0.21	0	t	1	Cystidicola cristivomeri	Cryptogonimus chilli	4	0.00	0.03	0.00	0.03
0	0	0.27	0	cv	1	Bunodera sacculata	Semotilus margarita	4	0.01	0.03	0.00	0.02
0	0	0.25	0	t	1	Cystidicola stigmatura	Stizostedion vitreum	4	0.01	0.03	0.01	0.03
0	0	0.28	0	t	1	Proteocephalus exiguus	Notropis atherinoides	4	0.00	0.03	0.01	0.03
0	1	0.78	0	t	1	Bothriocephalus claviceps	Catostomus commersoni	4	0.01	0.03	0.06	0.03
1	0	0.25	0	cv	1	Triganodistomum	Tetraonchus monenteron	4	0.01	0.03	0.01	0.04
1	0	0.36	0	t	1	Hunterella nodulosa	Proteocephalus pearsei	4	0.01	0.04	0.02	0.03
0	0	0.22	0	t	1	Allocreadium lobatum	Uvulifer amblopitis (L)	4	0.01	0.04	0.01	0.04
1	0	0.35	0	t	1	Octomacrum sp.	Stizostedion vitreum	4	0.01	0.02	0.01	0.03
0	0	0.20	0	cv	1	Triaenophorus stizostedionis (L)	Ergasilus versicolor	4	0.00	0.03	0.00	0.03
0	0	0.49	0	t	1	Esox lucius	Cystidicola cristivomeri	4	0.04	0.04	0.00	0.03

0	0	0.38	0	t	1	Triaenophorus crassus (L)	Contracaecum brachyurum	4	0.01	0.03	0.02	0.03
0	1	0.51	0	t	1	Centrovarium lobotes	Leptorhynchoides thecatus (L)	4	0.01	0.03	0.03	0.03
0	0	0.21	0	cv	1	Crepidostomum isostomum	Bothriocephalus cuspidatus	4	0.01	0.03	0.00	0.03
0	0	0.26	0	t	1	Proteocephalus stizostethi	Dactylogyrus bifurcatus	4	0.01	0.03	0.01	0.03
1	0	0.29	0	t	1	Plagioporus sinitsini	Spinitectus gracilis	4	0.00	0.04	0.02	0.03
0	0	0.21	0	t	1	Illinobdella sp.	Spinitectus carolini	4	0.01	0.04	0.00	0.03
0	0	0.24	0	cv	1	Moxostoma anisurum	Dactylogyrus mylocheilus	4	0.01	0.03	0.00	0.03
1	1	0.70	0	t	1	Metechinorhynchus lateralis	Rhabdochona cascadilla	4	0.04	0.04	0.02	0.03
0	0	0.23	0	t	1	Cleidodiscus banghami	Eubothrium salvelini	4	0.00	0.04	0.01	0.03
0	0	0.40	0	t	1	Ligula intestinalis (L)	Actinobdella triannulata	4	0.01	0.04	0.02	0.04
0	0	0.28	0	cv	1	Pomphorhynchus bulbocolli	Achtheres micropteri	4	0.02	0.04	0.00	0.03
0	1	0.74	0	t	1	Argulus versicolor	Catostomus commersoni	4	0.01	0.03	0.06	0.03
1	0	0.44	0	t	1	Neochasmus umbellus	Leptorhynchoides thecatus (L)	4	0.00	0.04	0.03	0.03
0	0	0.34	0	t	1	Centrovarium lobotes	Pomphorhynchus bulbocolli	4	0.01	0.03	0.02	0.04
0	0	0.23	0	cv	1	Eubothrium salvelini	Proteocephalus ambloplitis (L)	4	0.01	0.03	0.00	0.03
1	0	0.20	0	t	1	Alloglossidium corti	Argulus canadensis	4	0.00	0.03	0.00	0.03
0	0	0.43	0	t	1	Actinobdella triannulata	Phyllodistomum	4	0.02	0.04	0.01	0.03
0	0	0.22	0	t	1	Argulus appendiculosu	Lota lota	4	0.00	0.03	0.00	0.03
0	1	0.58	0	cv	1	Micropterus dolomieui	Moxostoma anisurum	4	0.04	0.03	0.01	0.03
0	0	0.22	0	t	1	Salvelinus namaycush	Diphyllobothrium sp. (L)	4	0.01	0.03	0.00	0.03
0	0	0.41	0	t	1	Bunodera sacculata	Phyllodistomum staffordi	4	0.01	0.03	0.02	0.03

0	0	0.28	0	t	1	Bothriocephalus claviceps	Pseudomurraytrema copulatum	4	0.01	0.03	0.01	0.04
0	0	0.34	0	cv	1	Azygia angusticauda	Spinitectus carolini	4	0.02	0.03	0.00	0.03
0	0	0.18	0	t	1	Acolpenteron catostomi	Alloglossidium geminus	4	0.00	0.04	0.00	0.03
0	0	0.19	0	t	1	Contracaecum brachyurum	Cleidodiscus capax	4	0.00	0.03	0.00	0.04
0	1	0.57	1	t	1	Pseudomurraytrema	Catostomus macrocheilus	5	0.02	0.03	0.03	0.04
0	0	0.36	1	cv	1	Pseudomurraytrema	Catostomus catostomus	5	0.02	0.03	0.01	0.04
0	0	0.39	1	t	1	Lota lota	Eustrongylides sp. (L)	5	0.03	0.05	0.01	0.05
0	1	0.72	1	t	1	Lota lota	Crepidostomum farionis	5	0.03	0.05	0.04	0.05
0	1	0.57	1	t	1	Lota lota	Haplonema hamatulum	5	0.03	0.05	0.02	0.05
0	0	0.44	1	cv	1	Lota lota	Hysterothylacium brachyurum	5	0.03	0.05	0.01	0.04
0	0	0.18	1	t	1	Philonema oncorhynchi	Oncorhynchus tshawytscha	5	0.01	0.05	0.01	0.06
1	1	0.95	1	t	1	Salvelinus namaycush	Eubothrium salvelini	5	0.05	0.06	0.08	0.06
1	1	0.90	1	t	1	Salvelinus namaycush	Salmincola edwardsii	5	0.05	0.06	0.06	0.06
0	1	0.50	1	cv	1	Salvelinus namaycush	Phyllodistomum sp.	5	0.05	0.06	0.01	0.05
0	1	0.50	1	t	1	Salvelinus namaycush	Diphyllobothrium sp. (L)	5	0.05	0.06	0.01	0.06
0	1	0.61	1	t	1	Salvelinus namaycush	Diplostomum sp.2 (L)	5	0.05	0.06	0.02	0.05
0	1	0.73	1	t	1	Salvelinus namaycush	Tetracotyle sp. (L)	5	0.05	0.06	0.03	0.05
0	1	0.76	1	cv	1	Salvelinus namaycush	Neoechinorhynchus rutili	5	0.05	0.06	0.04	0.06
0	0	0.48	1	t	1	Octospinifer macilentus	Catostomus macrocheilus	5	0.01	0.04	0.03	0.04
0	1	0.77	1	t	1	Eubothrium salvelini	Oncorhynchus tshawytscha	5	0.08	0.06	0.01	0.06
1	1	0.90	1	t	1	Eubothrium salvelini	Prosopium williamsoni	5	0.08	0.06	0.03	0.06
0	1	0.79	1	cv	1	Eubothrium salvelini	Salmo gairdneri	5	0.08	0.06	0.01	0.06
1	1	0.98	1	t	1	Eubothrium salvelini	Salvelinus malma	5	0.08	0.06	0.06	0.06

0	1	0.64	1	t	1	Ligula intestinalis (L)	Mylocheilus caurinus	5	0.04	0.03	0.02	0.04
0	0	0.23	1	t	1	Ptychocheilus oregonensis	Eustrongylides sp. (L)	5	0.01	0.04	0.01	0.05
0	0	0.39	1	cv	1	Ptychocheilus oregonensis	Proteocephalus ptychocheilus	5	0.01	0.04	0.02	0.02
1	0	0.31	1	t	1	Ptychocheilus oregonensis	Bothriocephalus opsariichthydis	5	0.01	0.04	0.01	0.02
0	0	0.44	1	t	1	Ptychocheilus oregonensis	Tetracotyle sp. (L)	5	0.01	0.04	0.03	0.05
0	0	0.48	1	t	1	Neoechinorhynchus	Catostomus macrocheilus	5	0.01	0.04	0.03	0.04
0	0	0.28	1	cv	1	Neoechinorhynchus	Catostomus catostomus	5	0.01	0.04	0.01	0.04
0	0	0.37	1	t	1	Mylocheilus caurinus	Proteocephalus sp.	5	0.02	0.04	0.02	0.05
0	1	0.54	1	t	1	Mylocheilus caurinus	Diplostomum sp.1 (L)	5	0.02	0.04	0.03	0.05
0	0	0.30	1	t	1	Mylocheilus caurinus	Ergasilus nerkae	5	0.02	0.04	0.01	0.05
0	0	0.37	1	cv	1	Mylocheilus caurinus	Octomacrum micronfibulum	5	0.02	0.04	0.01	0.03
1	0	0.44	1	t	1	Mylocheilus caurinus	Rhabdochona cascadilla	5	0.02	0.04	0.02	0.03
0	1	0.52	1	t	1	Mylocheilus caurinus	Tetracotyle sp. (L)	5	0.02	0.04	0.03	0.05
0	0	0.37	1	t	1	Mylocheilus caurinus	Allocreadium isosporum	5	0.02	0.04	0.01	0.03
1	1	0.93	1	cv	1	Cystidicola farionis	Salvelinus malma	5	0.05	0.06	0.06	0.06
0	1	0.84	1	t	1	Catostomus macrocheilus	Rhabdochona zacconis	5	0.03	0.04	0.05	0.04
0	0	0.47	1	t	1	Catostomus macrocheilus	Acolpenteron catostomi	5	0.03	0.04	0.01	0.04
0	1	0.50	1	t	1	Catostomus macrocheilus	Diplostomum sp.2 (L)	5	0.03	0.04	0.02	0.05
0	1	0.64	1	cv	1	Catostomus macrocheilus	Tetracotyle sp. (L)	5	0.03	0.04	0.03	0.05
0	1	0.65	1	t	1	Oncorhynchus nerka	Philonema sp.	5	0.05	0.06	0.02	0.06
0	1	0.97	1	t	1	Oncorhynchus nerka	Rhabdochona kisutchi	5	0.05	0.06	0.09	0.06
0	1	0.59	1	t	1	Oncorhynchus nerka	Proteocephalus sp.	5	0.05	0.06	0.02	0.05
0	0	0.49	1	cv	1	Oncorhynchus nerka	Diphyllobothrium sp. (L)	5	0.05	0.06	0.01	0.06

0	1	0.51	1	t	1	Oncorhynchus nerka	Ergasilus nerkae	5	0.05	0.06	0.01	0.05
0	1	0.80	1	t	1	Oncorhynchus nerka	Crepidostomum farionis	5	0.05	0.06	0.04	0.05
0	1	0.73	1	t	1	Oncorhynchus nerka	Tetracotyle sp. (L)	5	0.05	0.06	0.03	0.05
0	1	0.76	1	cv	1	Oncorhynchus nerka	Neoechinorhynchus rutili	5	0.05	0.06	0.04	0.06
0	0	0.37	1	t	1	Rhabdochona milleri	Prosopium williamsoni	5	0.01	0.05	0.03	0.06
0	0	0.25	1	t	1	Brachyphallus crenatus	Oncorhynchus tshawytscha	5	0.02	0.04	0.01	0.06
0	0	0.44	1	t	1	Oncorhynchus tshawytscha	Lampritrema miescheri	5	0.01	0.06	0.04	0.04
0	1	0.80	1	cv	1	Oncorhynchus tshawytscha	Octomacrum lanceatum	5	0.01	0.06	0.08	0.08
0	0	0.17	1	t	1	Oncorhynchus tshawytscha	Salmincola californiensis	5	0.01	0.06	0.01	0.05
0	0	0.25	1	t	1	Oncorhynchus tshawytscha	Genolinea oncorhynchi	5	0.01	0.06	0.02	0.04
0	0	0.45	1	t	1	Oncorhynchus tshawytscha	Crepidostomum farionis	5	0.01	0.06	0.04	0.05
0	0	0.18	1	cv	1	Oncorhynchus tshawytscha	Phyllobothrium sp. (L)	5	0.01	0.06	0.01	0.05
0	0	0.19	1	t	1	Oncorhynchus tshawytscha	Neascus sp. (L)	5	0.01	0.06	0.01	0.04
0	0	0.37	1	t	1	Eustrongylides sp. (L)	Prosopium williamsoni	5	0.01	0.05	0.03	0.06
0	0	0.19	1	t	1	Eustrongylides sp. (L)	Salmo gairdneri	5	0.01	0.05	0.01	0.06
0	1	0.71	1	cv	1	Eustrongylides sp. (L)	Salvelinus malma	5	0.01	0.05	0.06	0.06
0	1	0.81	1	t	1	Salmincola edwardsii	Prosopium williamsoni	5	0.06	0.06	0.03	0.06
1	1	0.95	1	t	1	Salmincola edwardsii	Salvelinus malma	5	0.06	0.06	0.06	0.06
0	0	0.50	1	t	1	Philonema sp.	Prosopium williamsoni	5	0.02	0.06	0.03	0.06
0	0	0.29	1	cv	1	Philonema sp.	Salmo gairdneri	5	0.02	0.06	0.01	0.06
0	1	0.81	1	t	1	Philonema sp.	Salvelinus malma	5	0.02	0.06	0.06	0.06
0	1	0.94	1	t	1	Prosopium williamsoni	Rhabdochona kisutchi	5	0.03	0.06	0.09	0.06
0	0	0.43	1	t	1	Prosopium williamsoni	Proteocephalus sp.	5	0.03	0.06	0.02	0.05
0	0	0.44	1	cv	1	Prosopium williamsoni	Salmincola thymalli	5	0.03	0.06	0.02	0.05
0	0	0.35	1	t	1	Prosopium williamsoni	Diphyllobothrium sp. (L)	5	0.03	0.06	0.01	0.06

0	1	0.60	1	t	1	Prosopium williamsoni	Diplostomum sp.1 (L)	5	0.03	0.06	0.03	0.05
0	1	0.81	1	t	1	Prosopium williamsoni	Philonema agubernaculum	5	0.03	0.06	0.06	0.05
0	0	0.35	1	cv	1	Prosopium williamsoni	Salmincola extensus	5	0.03	0.06	0.01	0.05
0	0	0.44	1	t	1	Prosopium williamsoni	Diplostomum sp.2 (L)	5	0.03	0.06	0.02	0.05
0	1	0.68	1	t	1	Prosopium williamsoni	Crepidostomum farionis	5	0.03	0.06	0.04	0.05
0	1	0.53	1	t	1	Prosopium williamsoni	Haplonema hamatulum	5	0.03	0.06	0.02	0.05
0	1	0.59	1	cv	1	Prosopium williamsoni	Tetracotyle sp. (L)	5	0.03	0.06	0.03	0.05
0	1	0.63	1	t	1	Prosopium williamsoni	Neoechinorhynchus rutili	5	0.03	0.06	0.04	0.06
0	0	0.35	1	t	1	Prosopium williamsoni	Tetraonchus variabilis	5	0.03	0.06	0.01	0.05
0	1	0.84	1	t	1	Rhabdochona kisutchi	Salmo gairdneri	5	0.09	0.06	0.01	0.06
1	1	0.98	1	cv	1	Rhabdochona kisutchi	Salvelinus malma	5	0.09	0.06	0.06	0.06
0	1	0.69	1	t	1	Rhabdochona canadensis	Salvelinus malma	5	0.01	0.06	0.06	0.06
1	0	0.33	1	t	1	Philometroides huronensis	Catostomus catostomus	5	0.01	0.02	0.01	0.04
0	0	0.28	1	t	1	Catostomus catostomus	Acolpenteron catostomi	5	0.01	0.04	0.01	0.04
1	0	0.32	1	cv	1	Catostomus catostomus	Glaridacris catostomi	5	0.01	0.04	0.01	0.02
0	0	0.32	1	t	1	Catostomus catostomus	Diplostomum sp.2 (L)	5	0.01	0.04	0.02	0.05
0	0	0.45	1	t	1	Catostomus catostomus	Tetracotyle sp. (L)	5	0.01	0.04	0.03	0.05
0	0	0.27	1	t	1	Catostomus catostomus	Neascus sp. (L)	5	0.01	0.04	0.01	0.04
1	0	0.32	1	cv	1	Catostomus catostomus	Anoncohaptor anomalum	5	0.01	0.04	0.01	0.02
0	0	0.19	1	t	1	Phyllodistomum sp.	Salmo gairdneri	5	0.01	0.05	0.01	0.06
0	0	0.43	1	t	1	Proteocephalus sp.	Cottus asper	5	0.02	0.05	0.02	0.04
0	1	0.77	1	t	1	Proteocephalus sp.	Salvelinus malma	5	0.02	0.05	0.06	0.06
0	0	0.18	1	cv	1	Diphyllobothrium sp. (L)	Salmo gairdneri	5	0.01	0.06	0.01	0.06
0	1	0.69	1	t	1	Proteocephalus	Salvelinus malma	5	0.01	0.06	0.06	0.06
0	0	0.38	1	t	1	Diplostomum sp.1 (L)	Salmo gairdneri	5	0.03	0.05	0.01	0.06

0	1	0.62	1	t	1	Philonema agubernaculu	Salmo gairdneri	5	0.06	0.05	0.01	0.06
0	0	0.19	1	cv	1	Salmo gairdneri	Salmincola californiensis	5	0.01	0.06	0.01	0.05
0	0	0.26	1	t	1	Salmo gairdneri	Diplostomum sp.2 (L)	5	0.01	0.06	0.02	0.05
0	0	0.48	1	t	1	Salmo gairdneri	Crepidostom um farionis	5	0.01	0.06	0.04	0.05
0	0	0.33	1	t	1	Salmo gairdneri	Haplonema hamatulum	5	0.01	0.06	0.02	0.05
0	0	0.23	1	cv	1	Salmo gairdneri	Cystidicoloide s tenuissima	5	0.01	0.06	0.02	0.06
0	0	0.38	1	t	1	Salmo gairdneri	Tetracotyle sp. (L)	5	0.01	0.06	0.03	0.05
0	0	0.42	1	t	1	Salmo gairdneri	Neoechinorh ynchus rutili	5	0.01	0.06	0.04	0.06
0	0	0.47	1	t	1	Salmo gairdneri	Proteocephal us tumidocollus	5	0.01	0.06	0.04	0.04
0	0	0.21	1	cv	1	Salmo gairdneri	Bunodera luciopercae	5	0.01	0.06	0.01	0.05
1	0	0.42	1	t	1	Rhabdochona cotti	Cottus cognatus	5	0.01	0.03	0.02	0.04
0	0	0.43	1	t	1	Diplostomum sp.2 (L)	Cottus cognatus	5	0.02	0.05	0.02	0.04
0	0	0.44	1	t	1	Diplostomum sp.2 (L)	Richardsonius balteatus	5	0.02	0.05	0.02	0.04
0	1	0.78	1	cv	1	Diplostomum sp.2 (L)	Salvelinus malma	5	0.02	0.05	0.06	0.06
0	1	0.90	1	t	1	Crepidostomu m farionis	Salvelinus malma	5	0.04	0.05	0.06	0.06
1	1	0.51	1	t	1	Rhabdochona cascadilla	Richardsonius balteatus	5	0.02	0.03	0.02	0.04
0	1	0.57	1	t	1	Cottus cognatus	Tetracotyle sp. (L)	5	0.02	0.04	0.03	0.05
0	1	0.58	1	cv	1	Richardsonius balteatus	Tetracotyle sp. (L)	5	0.02	0.04	0.03	0.05
0	1	0.76	1	t	1	Cystidicoloides tenuissima	Salvelinus malma	5	0.02	0.06	0.06	0.06
0	1	0.86	1	t	1	Tetracotyle sp. (L)	Salvelinus malma	5	0.03	0.05	0.06	0.06
0	1	0.87	1	t	1	Neoechinorhy nchus rutili	Salvelinus malma	5	0.04	0.06	0.06	0.06
1	1	0.93	1	cv	1	Tetraonchus alaskensis	Salvelinus malma	5	0.05	0.06	0.06	0.06
0	0	0.23	0	t	1	Diphyllobothri um sp. (L)	Rhabdochona cotti	5	0.01	0.06	0.01	0.03
0	0	0.46	0	t	1	Catostomus macrocheilus	Hysterothylac ium brachyurum	5	0.03	0.04	0.01	0.04

0	0	0.27	0	t	1	Philometroides huronensis	Diphyllobothrium sp. (L)	5	0.01	0.02	0.01	0.06
0	0	0.45	0	cv	1	Genolinea oncorhynchi	Haplonema hamatulum	5	0.02	0.04	0.02	0.05
0	1	0.78	0	t	1	Octomacrum micronfibulum	Salvelinus malma	5	0.01	0.03	0.06	0.06
0	0	0.18	0	t	1	Rhabdochona milleri	Proteocephalus salmonidicola	5	0.01	0.05	0.01	0.06
0	1	0.58	0	t	1	Lota lota	Cottus cognatus	5	0.03	0.05	0.02	0.04
0	0	0.44	0	cv	1	Bothriocephalus	Haplonema hamatulum	5	0.01	0.02	0.02	0.05
0	1	0.87	0	t	1	Philonema agubernaculu	Crepidostomum farionis	5	0.06	0.05	0.04	0.05
1	0	0.29	0	t	1	Acolpenteron catostomi	Rhabdochona cotti	5	0.01	0.04	0.01	0.03
0	1	0.77	0	t	1	Tetracotyle sp. (L)	Tetraonchus alaskensis	5	0.03	0.05	0.05	0.06
0	0	0.20	0	cv	1	Rhabdochona canadensis	Bunodera luciopercae	5	0.01	0.06	0.01	0.05
0	0	0.21	0	t	1	Tetraonchus variabilis	Bunodera luciopercae	5	0.01	0.05	0.01	0.05
0	0	0.21	0	t	1	Salmincola extensus	Bunodera luciopercae	5	0.01	0.05	0.01	0.05
0	0	0.24	0	t	1	Octospinifer macilentus	Phyllobothrium sp. (L)	5	0.01	0.04	0.01	0.05
0	0	0.28	0	cv	1	Glaridacris catostomi	Salmincola californiensis	5	0.01	0.02	0.01	0.05
0	0	0.46	0	t	1	Diplostomum sp.1 (L)	Rhabdochona cotti	5	0.03	0.05	0.01	0.03
1	1	0.60	0	t	1	Catostomus macrocheilus	Proteocephalus ptychocheilus	5	0.03	0.04	0.02	0.02
0	0	0.31	0	t	1	Pseudomurraytrema	Ergasilus nerkae	5	0.02	0.03	0.01	0.05
0	1	0.70	0	cv	1	Lampritrema miescheri	Diplostomum sp.1 (L)	5	0.04	0.04	0.03	0.05
0	1	0.65	0	t	1	Lampritrema miescheri	Cottus cognatus	5	0.04	0.04	0.02	0.04
0	1	0.62	0	t	1	Richardsonius balteatus	Neoechinorhynchus rutili	5	0.02	0.04	0.04	0.06
0	1	0.84	0	t	1	Rhabdochona kisutchi	Rhabdochona canadensis	5	0.09	0.06	0.01	0.06
0	0	0.43	0	cv	1	Lota lota	Ptychocheilus oregonensis	5	0.03	0.05	0.01	0.04
0	1	0.52	0	t	1	Catostomus macrocheilus	Genolinea oncorhynchi	5	0.03	0.04	0.02	0.04

0	0	0.17	0	t	1	Oncorhynchus tshawytscha	Diphyllobothrium sp. (L)	5	0.01	0.06	0.01	0.06
0	0	0.48	0	t	1	Lota lota	Salmincola thymalli	5	0.03	0.05	0.02	0.05
0	0	0.24	0	cv	1	Eustrongylides sp. (L)	Cystidicoloides tenuissima	5	0.01	0.05	0.02	0.06
0	1	0.85	0	t	1	Lota lota	Salmincola edwardsii	5	0.03	0.05	0.06	0.06
0	1	0.67	0	t	1	Octomacrum micronfibulum	Tetraonchus alaskensis	5	0.01	0.03	0.05	0.06
1	0	0.35	0	t	1	Allocreadium isosporum	Anoncohaptor anomalum	5	0.01	0.03	0.01	0.02
0	0	0.28	0	cv	1	Eustrongylides sp. (L)	Genolinea oncorhynchi	5	0.01	0.05	0.02	0.04
0	1	0.85	0	t	1	Rhabdochona kisutchi	Salmincola californiensis	5	0.09	0.06	0.01	0.05
0	0	0.32	0	t	1	Brachyphallus crenatus	Hysterothylacium brachyurum	5	0.02	0.04	0.01	0.04
0	0	0.34	0	t	1	Proteocephalus sp.	Glaridacris catostomi	5	0.02	0.05	0.01	0.02
0	1	0.79	0	cv	1	Salvelinus namaycush	Lampritrema miescheri	5	0.05	0.06	0.04	0.04
0	1	0.81	0	t	1	Rhabdochona zacconis	Tetracotyle sp. (L)	5	0.05	0.04	0.03	0.05
0	0	0.26	0	t	1	Rhabdochona milleri	Salmincola thymalli	5	0.01	0.05	0.02	0.05
0	1	0.54	0	t	1	Prosopium williamsoni	Proteocephalus ptychocheilus	5	0.03	0.06	0.02	0.02
0	1	0.55	0	cv	1	Crepidostomum farionis	Allocreadium isosporum	5	0.04	0.05	0.01	0.03
0	0	0.34	0	t	1	Haplonema hamatulum	Phyllobothrium sp. (L)	5	0.02	0.05	0.01	0.05
0	1	0.64	0	t	1	Neoechinorhynchus	Tetraonchus alaskensis	5	0.01	0.04	0.05	0.06
0	0	0.31	0	t	1	Phyllodistomum sp.	Rhabdochona cascadilla	5	0.01	0.05	0.02	0.03
0	1	0.68	0	cv	1	Rhabdochona milleri	Salmincola edwardsii	5	0.01	0.05	0.06	0.06
0	0	0.31	0	t	1	Pseudomurraytrema	Philonema oncorhynchi	5	0.02	0.03	0.01	0.05
0	0	0.48	0	t	1	Mylocheilus caurinus	Cottus cognatus	5	0.02	0.04	0.02	0.04
0	1	0.84	0	t	1	Rhabdochona kisutchi	Proteocephalus salmonidicola	5	0.09	0.06	0.01	0.06

0	0	0.36	0	cv	1	Pseudomurraytrema copulata	Neoechinorhynchus cristatus	5	0.02	0.03	0.01	0.04
0	1	0.97	0	t	1	Salmincola edwardsii	Octomacrum lanceatum	5	0.06	0.06	0.08	0.08
0	0	0.33	0	t	1	Salmincola thymalli	Diplostomum sp.2 (L)	5	0.02	0.05	0.02	0.05
0	0	0.37	0	t	1	Philonema oncorhynchi	Prosopium williamsoni	5	0.01	0.05	0.03	0.06
0	0	0.31	0	cv	1	Catostomus catostomus	Proteocephalus sp.	5	0.01	0.04	0.02	0.05
0	1	0.51	0	t	1	Rhabdochona cascadilla	Cottus cognatus	5	0.02	0.03	0.02	0.04
0	1	0.80	0	t	1	Neoechinorhynchus rutili	Tetraonchus alaskensis	5	0.04	0.06	0.05	0.06
1	0	0.46	0	t	1	Bothriocephalus	Richardsonius balteatus	5	0.01	0.02	0.02	0.04
0	0	0.23	0	cv	1	Oncorhynchus tshawytscha	Diplostomum sp.2 (L)	5	0.01	0.06	0.02	0.05
0	0	0.19	0	t	1	Salmincola extensus	Salmincola californiensis	5	0.01	0.05	0.01	0.05
0	0	0.34	0	t	1	Diphyllobothrium sp. (L)	Cottus cognatus	5	0.01	0.06	0.02	0.04
0	0	0.48	0	t	1	Tetracotyle sp. (L)	Anoncohaptor anomalum	5	0.03	0.05	0.01	0.02
0	0	0.18	0	cv	1	Proteocephalus	Tetraonchus variabilis	5	0.01	0.06	0.01	0.05
0	0	0.40	0	t	1	Catostomus catostomus	Proteocephalus ptychocheilus	5	0.01	0.04	0.02	0.02
0	0	0.37	0	t	1	Oncorhynchus tshawytscha	Diplostomum sp.1 (L)	5	0.01	0.06	0.03	0.05
0	0	0.45	0	t	1	Cystidicoloides tenuissima	Tetracotyle sp. (L)	5	0.02	0.06	0.03	0.05
0	1	0.60	0	cv	1	Glaridacris catostomi	Proteocephalus tumidocollus	5	0.01	0.02	0.04	0.04
0	1	0.73	0	t	1	Crepidostomum farionis	Neoechinorhynchus rutili	5	0.04	0.05	0.04	0.06
0	0	0.22	0	t	1	Oncorhynchus tshawytscha	Allocreadium isosporum	5	0.01	0.06	0.01	0.03
0	1	0.56	0	t	1	Neoechinorhynchus	Crepidostomum farionis	5	0.01	0.04	0.04	0.05
0	0	0.45	0	cv	1	Rhabdochona canadensis	Lampritrema miescheri	5	0.01	0.06	0.04	0.04
0	0	0.43	0	t	1	Neoechinorhynchus	Prosopium williamsoni	5	0.01	0.04	0.03	0.06
1	1	0.94	0	t	1	Eubothrium salvelini	Crepidostomum farionis	5	0.08	0.06	0.04	0.05

0	1	0.54	0	t	1	Oncorhynchus tshawytscha	Tetraonchus alaskensis	5	0.01	0.06	0.05	0.06
0	0	0.39	0	cv	1	Eustrongylides sp. (L)	Tetracotyle sp. (L)	5	0.01	0.05	0.03	0.05
0	0	0.28	0	t	1	Philometroides huronensis	Tetraonchus variabilis	5	0.01	0.02	0.01	0.05
0	0	0.23	0	t	1	Octospinifer macilentus	Salmo gairdneri	5	0.01	0.04	0.01	0.06
0	1	0.69	0	t	1	Genolinea oncorhynchi	Tetraonchus alaskensis	5	0.02	0.04	0.05	0.06
0	0	0.28	0	cv	1	Mylocheilus caurinus	Proteocephalus salmonidicola	5	0.02	0.04	0.01	0.06
0	1	0.54	0	t	1	Philonema oncorhynchi	Salvelinus namaycush	5	0.01	0.05	0.05	0.06
0	1	0.56	0	t	1	Ligula intestinalis (L)	Ptychocheilus oregonensis	5	0.04	0.03	0.01	0.04
0	1	0.52	0	t	1	Salvelinus namaycush	Phyllobothrium sp. (L)	5	0.05	0.06	0.01	0.05
0	1	0.54	0	cv	1	Cystidicola farionis	Salmo gairdneri	5	0.05	0.06	0.01	0.06
0	1	0.70	0	t	1	Salvelinus namaycush	Richardsonius balteatus	5	0.05	0.06	0.02	0.04
0	0	0.24	0	t	1	Ptychocheilus oregonensis	Phyllobothrium sp. (L)	5	0.01	0.04	0.01	0.05
0	0	0.31	0	t	1	Neoechinorhynchus	Proteocephalus sp.	5	0.01	0.04	0.02	0.05
0	0	0.28	0	cv	1	Philonema oncorhynchi	Genolinea oncorhynchi	5	0.01	0.05	0.02	0.04
0	1	0.64	0	t	1	Neoechinorhynchus	Cystidicola farionis	5	0.01	0.04	0.05	0.06
0	0	0.28	0	t	1	Octomacrum micronfibulum	Bunodera luciopercae	5	0.01	0.03	0.01	0.05
0	0	0.17	0	t	1	Rhabdochona canadensis	Proteocephalus salmonidicola	5	0.01	0.06	0.01	0.06
0	0	0.33	0	cv	1	Philonema sp.	Neascus sp. (L)	5	0.02	0.06	0.01	0.04
0	0	0.33	0	t	1	Pseudomurraytrema	Bunodera luciopercae	5	0.02	0.03	0.01	0.05
0	1	0.79	0	t	1	Oncorhynchus nerka	Lampritrema miescheri	5	0.05	0.06	0.04	0.04
0	1	0.61	0	t	1	Rhabdochona zacconis	Diphyllobothrium sp. (L)	5	0.05	0.04	0.01	0.06
0	0	0.39	0	cv	1	Lota lota	Phyllobothrium sp. (L)	5	0.03	0.05	0.01	0.05
0	0	0.25	0	t	1	Salmincola thymalli	Diphyllobothrium sp. (L)	5	0.02	0.05	0.01	0.06

1	1	0.97	0	t	1	Eubothrium salvelini	Salmincola edwardsii	5	0.08	0.06	0.06	0.06
0	1	0.80	0	t	1	Salvelinus namaycush	Proteocephalus tumidocollus	5	0.05	0.06	0.04	0.04
0	0	0.24	0	cv	1	Acolpenteron catostomi	Phyllobothrium sp. (L)	5	0.01	0.04	0.01	0.05
0	0	0.30	0	t	1	Proteocephalus	Rhabdochona cascadilla	5	0.01	0.06	0.02	0.03
0	0	0.41	0	t	1	Lota lota	Bunodera luciopercae	5	0.03	0.05	0.01	0.05
0	1	0.73	0	t	1	Ligula intestinalis (L)	Tetracotyle sp. (L)	5	0.04	0.03	0.03	0.05
0	0	0.35	0	cv	1	Philonema sp.	Acolpenteron catostomi	5	0.02	0.06	0.01	0.04
0	0	0.26	0	t	1	Salmo gairdneri	Anoncohaptor anomalum	5	0.01	0.06	0.01	0.02
0	0	0.40	0	t	1	Rhabdochona canadensis	Neoechinorhynchus rutili	5	0.01	0.06	0.04	0.06
1	0	0.32	0	t	1	Ptychocheilus oregonensis	Anoncohaptor anomalum	5	0.01	0.04	0.01	0.02
0	0	0.35	0	cv	1	Neoechinorhynchus	Mylocheilus caurinus	5	0.01	0.04	0.02	0.04
0	1	0.89	0	t	1	Rhabdochona kisutchi	Salmincola thymalli	5	0.09	0.06	0.02	0.05
0	1	0.65	0	t	1	Salmincola edwardsii	Phyllobothrium sp. (L)	5	0.06	0.06	0.01	0.05
0	0	0.37	0	t	1	Cottus cognatus	Neascus sp. (L)	5	0.02	0.04	0.01	0.04
0	0	0.35	0	cv	1	Richardsonius balteatus	Phyllobothrium sp. (L)	5	0.02	0.04	0.01	0.05
0	1	0.50	0	t	1	Catostomus macrocheilus	Bothriocephalus opsariichthydis	5	0.03	0.04	0.01	0.02
0	1	0.71	0	t	1	Oncorhynchus nerka	Prosopium williamsoni	5	0.05	0.06	0.03	0.06
1	1	0.61	0	t	1	Ligula intestinalis (L)	Anoncohaptor anomalum	5	0.04	0.03	0.01	0.02
0	0	0.45	0	cv	1	Lampritrema miescheri	Salmo gairdneri	5	0.04	0.04	0.01	0.06
0	0	0.35	0	t	1	Eustrongylides sp. (L)	Cottus asper	5	0.01	0.05	0.02	0.04
0	1	0.87	0	t	1	Eustrongylides sp. (L)	Rhabdochona kisutchi	5	0.01	0.05	0.09	0.06
0	1	0.69	1	t	1	Pseudomurraytrema	Catostomus macrocheilus	6	0.02	0.04	0.04	0.04
0	0	0.45	1	cv	1	Pseudomurraytrema	Catostomus catostomus	6	0.02	0.04	0.02	0.04
0	0	0.37	1	t	1	Pseudomurraytrema	Catostomus commersoni	6	0.02	0.04	0.01	0.04

0	1	0.78	1	t	1	Lota lota	Diplostomum sp.2 (L)	6	0.03	0.05	0.05	0.05
0	0	0.40	1	t	1	Lota lota	Neoechinorhynchus rutili	6	0.03	0.05	0.01	0.05
0	0	0.34	1	cv	1	Lota lota	Crepidostomum farionis	6	0.03	0.05	0.01	0.05
0	1	0.71	1	t	1	Lota lota	Haplonema hamatulum	6	0.03	0.05	0.04	0.05
0	0	0.33	1	t	1	Lota lota	Hysterothylacium brachyurum	6	0.03	0.05	0.01	0.06
0	1	0.70	1	t	1	Salvelinus namaycush	Eubothrium salvelini	6	0.04	0.05	0.03	0.05
0	1	0.50	1	cv	1	Salvelinus namaycush	Tetracotyle sp. (L)	6	0.04	0.05	0.01	0.05
0	1	0.61	1	t	1	Salvelinus namaycush	Cystidicola farionis	6	0.04	0.05	0.02	0.05
0	1	0.87	1	t	1	Salvelinus namaycush	Diplostomum sp.2 (L)	6	0.04	0.05	0.05	0.05
0	1	0.74	1	t	1	Salvelinus namaycush	Truttaedacnitis truttae	6	0.04	0.05	0.03	0.05
0	1	0.78	1	cv	1	Salvelinus namaycush	Diphyllobothrium sp. (L)	6	0.04	0.05	0.04	0.05
0	1	0.56	1	t	1	Salvelinus namaycush	Neoechinorhynchus rutili	6	0.04	0.05	0.01	0.05
0	1	0.93	1	t	1	Salvelinus namaycush	Salmincola edwardsii	6	0.04	0.05	0.07	0.05
0	1	0.79	1	t	1	Salvelinus namaycush	Salmincola californiensis	6	0.04	0.05	0.04	0.05
0	1	0.69	1	cv	1	Octospinifer macilentus	Catostomus macrocheilus	6	0.02	0.04	0.04	0.04
0	0	0.45	1	t	1	Octospinifer macilentus	Catostomus catostomus	6	0.02	0.04	0.02	0.04
0	0	0.37	1	t	1	Octospinifer macilentus	Catostomus commersoni	6	0.02	0.04	0.01	0.04
0	0	0.40	1	t	1	Eubothrium salvelini	Oncorhynchus nerka	6	0.03	0.05	0.01	0.05
0	0	0.34	1	cv	1	Eubothrium salvelini	Prosopium williamsoni	6	0.03	0.05	0.01	0.04
0	0	0.34	1	t	1	Eubothrium salvelini	Salmo gairdneri	6	0.03	0.05	0.01	0.04
1	1	0.88	1	t	1	Eubothrium salvelini	Salvelinus malma	6	0.03	0.05	0.07	0.05
0	0	0.23	1	t	1	Pellucidhaptor pellucidhaptor	Richardsonius balteatus	6	0.01	0.04	0.01	0.04
0	1	0.57	1	cv	1	Tetracotyle sp. (L)	Coregonus clupeaformis	6	0.01	0.05	0.05	0.05
0	1	0.55	1	t	1	Tetracotyle sp. (L)	Catostomus macrocheilus	6	0.01	0.05	0.04	0.04
0	0	0.23	1	t	1	Tetracotyle sp. (L)	Oncorhynchus nerka	6	0.01	0.05	0.01	0.05

0	0	0.19	1	t	1	Tetracotyle sp. (L)	Prosopium williamsoni	6	0.01	0.05	0.01	0.04
0	0	0.31	1	cv	1	Tetracotyle sp. (L)	Catostomus catostomus	6	0.01	0.05	0.02	0.04
0	0	0.19	1	t	1	Tetracotyle sp. (L)	Salmo gairdneri	6	0.01	0.05	0.01	0.04
0	0	0.24	1	t	1	Tetracotyle sp. (L)	Catostomus commersoni	6	0.01	0.05	0.01	0.04
0	0	0.28	1	t	1	Tetracotyle sp. (L)	Rhinichthys cataractae	6	0.01	0.05	0.01	0.03
0	0	0.20	1	cv	1	Tetracotyle sp. (L)	Thymallus arcticus	6	0.01	0.05	0.01	0.04
0	0	0.19	1	t	1	Tetracotyle sp. (L)	Richardsonius balteatus	6	0.01	0.05	0.01	0.04
0	1	0.70	1	t	1	Tetracotyle sp. (L)	Cottus cognatus	6	0.01	0.05	0.06	0.05
0	1	0.61	1	t	1	Tetracotyle sp. (L)	Ptychocheilus oregonensis	6	0.01	0.05	0.05	0.05
0	1	0.76	1	cv	1	Tetracotyle sp. (L)	Salvelinus malma	6	0.01	0.05	0.07	0.05
0	1	0.69	1	t	1	Neoechinorhy nchus	Catostomus macrocheilus	6	0.02	0.04	0.04	0.04
0	0	0.45	1	t	1	Neoechinorhy nchus	Catostomus catostomus	6	0.02	0.04	0.02	0.04
0	0	0.37	1	t	1	Neoechinorhy nchus	Catostomus commersoni	6	0.02	0.04	0.01	0.04
0	1	0.54	1	cv	1	Mylocheilus caurinus	Bothriocepha lus opsariichthyd is	6	0.03	0.05	0.02	0.05
0	1	0.77	1	t	1	Mylocheilus caurinus	Diplostomum sp.1 (L)	6	0.03	0.05	0.05	0.05
0	1	0.72	1	t	1	Mylocheilus caurinus	Ergasilus nerkae	6	0.03	0.05	0.04	0.05
0	0	0.43	1	t	1	Mylocheilus caurinus	Allocreadium isosporum	6	0.03	0.05	0.01	0.05
0	1	0.70	1	cv	1	Mylocheilus caurinus	Eustrongylide s sp. (L)	6	0.03	0.05	0.04	0.05
0	0	0.26	1	t	1	Cystidicola farionis	Prosopium williamsoni	6	0.02	0.05	0.01	0.04
1	1	0.82	1	t	1	Cystidicola farionis	Salvelinus malma	6	0.02	0.05	0.07	0.05
1	1	0.94	1	t	1	Coregonus clupeaformis	Pomphorhyn chus bulbocolli	6	0.05	0.05	0.06	0.05
1	1	0.89	1	cv	1	Coregonus clupeaformis	Diplostomum sp.2 (L)	6	0.05	0.05	0.05	0.05
0	1	0.60	1	t	1	Coregonus clupeaformis	Philonema sp.	6	0.05	0.05	0.01	0.05
0	1	0.54	1	t	1	Coregonus clupeaformis	Proteocephal us sp.	6	0.05	0.05	0.01	0.05

0	1	0.81	1	t	1	Coregonus clupeaformis	Diphyllobothrium sp. (L)	6	0.05	0.05	0.04	0.05
1	1	0.88	1	cv	1	Coregonus clupeaformis	Diplostomum sp.1 (L)	6	0.05	0.05	0.05	0.05
0	1	0.61	1	t	1	Coregonus clupeaformis	Neoechinorhynchus rutili	6	0.05	0.05	0.01	0.05
0	1	0.85	1	t	1	Coregonus clupeaformis	Ergasilus nerkae	6	0.05	0.05	0.04	0.05
0	1	0.54	1	t	1	Coregonus clupeaformis	Crepidostomum farionis	6	0.05	0.05	0.01	0.05
0	0	0.24	1	cv	1	Rhabdochona cascadilla	Richardsonius balteatus	6	0.01	0.05	0.01	0.04
0	1	0.68	1	t	1	Rhabdochona cascadilla	Ptychocheilus oregonensis	6	0.01	0.05	0.05	0.05
1	1	0.93	1	t	1	Pomphorhynchus bulbocolli	Catostomus macrocheilus	6	0.06	0.05	0.04	0.04
1	1	0.71	1	t	1	Pomphorhynchus bulbocolli	Prosopium williamsoni	6	0.06	0.05	0.01	0.04
1	1	0.71	1	cv	1	Pomphorhynchus bulbocolli	Salmo gairdneri	6	0.06	0.05	0.01	0.04
1	1	0.77	1	t	1	Pomphorhynchus bulbocolli	Catostomus commersoni	6	0.06	0.05	0.01	0.04
1	1	0.94	1	t	1	Pomphorhynchus bulbocolli	Ptychocheilus oregonensis	6	0.06	0.05	0.05	0.05
0	1	0.55	1	t	1	Catostomus macrocheilus	Rhabdochona zacconis	6	0.04	0.04	0.01	0.04
1	1	0.88	1	cv	1	Catostomus macrocheilus	Diplostomum sp.2 (L)	6	0.04	0.04	0.05	0.05
0	1	0.55	1	t	1	Catostomus macrocheilus	Hunterella nodulosa	6	0.04	0.04	0.01	0.04
0	1	0.62	1	t	1	Catostomus macrocheilus	Philometroides huronensis	6	0.04	0.04	0.01	0.04
0	1	0.55	1	t	1	Catostomus macrocheilus	Glaridacris vogei	6	0.04	0.04	0.01	0.04
1	1	0.86	1	cv	1	Catostomus macrocheilus	Octomacrum lanceatus	6	0.04	0.04	0.04	0.04
0	1	0.62	1	t	1	Catostomus macrocheilus	Acolpenteron catostomi	6	0.04	0.04	0.01	0.04
0	1	0.83	1	t	1	Catostomus macrocheilus	Ergasilus nerkae	6	0.04	0.04	0.04	0.05
1	1	0.79	1	t	1	Catostomus macrocheilus	Glaridacris sp.	6	0.04	0.04	0.03	0.04
0	1	0.68	1	cv	1	Catostomus macrocheilus	Anoncohaptor anomalum	6	0.04	0.04	0.02	0.04
0	1	0.67	1	t	1	Oncorhynchus nerka	Diplostomum sp.2 (L)	6	0.01	0.05	0.05	0.05
0	0	0.27	1	t	1	Oncorhynchus nerka	Philonema sp.	6	0.01	0.05	0.01	0.05
0	0	0.22	1	t	1	Oncorhynchus nerka	Proteocephalus sp.	6	0.01	0.05	0.01	0.05

0	0	0.30	1	cv	1	Oncorhynchus nerka	Phyllodistomum sp.	6	0.01	0.05	0.01	0.04
0	1	0.51	1	t	1	Oncorhynchus nerka	Diphyllobothrium sp. (L)	6	0.01	0.05	0.04	0.05
0	0	0.27	1	t	1	Oncorhynchus nerka	Neoechinorhynchus rutili	6	0.01	0.05	0.01	0.05
0	0	0.22	1	t	1	Oncorhynchus nerka	Crepidostomum farionis	6	0.01	0.05	0.01	0.05
1	0	0.38	1	cv	1	Rhabdochona milleri	Rhinichthys cataractae	6	0.01	0.02	0.01	0.03
1	1	0.58	1	t	1	Diplostomum sp.2 (L)	Prosopium williamsoni	6	0.05	0.05	0.01	0.04
0	1	0.59	1	t	1	Diplostomum sp.2 (L)	Salmo gairdneri	6	0.05	0.05	0.01	0.04
0	1	0.59	1	t	1	Diplostomum sp.2 (L)	Richardsonius balteatus	6	0.05	0.05	0.01	0.04
1	1	0.93	1	cv	1	Diplostomum sp.2 (L)	Cottus cognatus	6	0.05	0.05	0.06	0.05
1	1	0.91	1	t	1	Diplostomum sp.2 (L)	Ptychocheilus oregonensis	6	0.05	0.05	0.05	0.05
1	1	0.95	1	t	1	Diplostomum sp.2 (L)	Salvelinus malma	6	0.05	0.05	0.07	0.05
0	1	0.77	1	t	1	Bothriocephalus	Ptychocheilus oregonensis	6	0.02	0.05	0.05	0.05
0	0	0.22	1	cv	1	Philonema sp.	Prosopium williamsoni	6	0.01	0.05	0.01	0.04
0	0	0.22	1	t	1	Philonema sp.	Salmo gairdneri	6	0.01	0.05	0.01	0.04
0	0	0.23	1	t	1	Philonema sp.	Thymallus arcticus	6	0.01	0.05	0.01	0.04
1	1	0.80	1	t	1	Philonema sp.	Salvelinus malma	6	0.01	0.05	0.07	0.05
0	0	0.38	1	cv	1	Truttaedacnitis truttae	Prosopium williamsoni	6	0.03	0.05	0.01	0.04
0	0	0.38	1	t	1	Truttaedacnitis truttae	Salmo gairdneri	6	0.03	0.05	0.01	0.04
1	1	0.89	1	t	1	Truttaedacnitis truttae	Salvelinus malma	6	0.03	0.05	0.07	0.05
0	0	0.18	1	t	1	Prosopium williamsoni	Proteocephalus sp.	6	0.01	0.04	0.01	0.05
0	0	0.38	1	cv	1	Prosopium williamsoni	Salmincola thymalli	6	0.01	0.04	0.02	0.03
0	0	0.45	1	t	1	Prosopium williamsoni	Diphyllobothrium sp. (L)	6	0.01	0.04	0.04	0.05
0	0	0.23	1	t	1	Prosopium williamsoni	Neoechinorhynchus rutili	6	0.01	0.04	0.01	0.05
0	0	0.26	1	t	1	Prosopium williamsoni	Philonema agubernaculum	6	0.01	0.04	0.01	0.04
0	0	0.18	1	cv	1	Prosopium williamsoni	Crepidostomum farionis	6	0.01	0.04	0.01	0.05

0	0	0.49	1	t	1	Prosopium williamsoni	Eustrongylides sp. (L)	6	0.01	0.04	0.04	0.05
0	0	0.38	1	t	1	Prosopium williamsoni	Tetraonchus variabilis	6	0.01	0.04	0.02	0.03
1	1	0.97	1	t	1	Rhabdochona kisutchi	Salvelinus malma	6	0.06	0.06	0.07	0.05
0	0	0.26	1	cv	1	Hunterella nodulosa	Catostomus commersoni	6	0.01	0.04	0.01	0.04
0	1	0.68	1	t	1	Neascus sp (L)	Ptychocheilus oregonensis	6	0.01	0.05	0.05	0.05
1	1	0.75	1	t	1	Proteocephalus sp.	Salvelinus malma	6	0.01	0.05	0.07	0.05
0	0	0.40	1	t	1	Catostomus catostomus	Philometroides huronensis	6	0.02	0.04	0.01	0.04
0	0	0.40	1	cv	1	Catostomus catostomus	Acolpenteron catostomi	6	0.02	0.04	0.01	0.04
0	0	0.35	1	t	1	Catostomus catostomus	Monobothrium sp.	6	0.02	0.04	0.01	0.03
0	0	0.35	1	t	1	Catostomus catostomus	Glaridacris catostomi	6	0.02	0.04	0.01	0.03
0	0	0.45	1	t	1	Catostomus catostomus	Anoncohaptor anomalum	6	0.02	0.04	0.02	0.04
0	0	0.26	1	cv	1	Phyllodistomum sp.	Salmo gairdneri	6	0.01	0.04	0.01	0.04
1	0	0.32	1	t	1	Tetraonchus borealis	Thymallus arcticus	6	0.01	0.03	0.01	0.04
0	0	0.39	1	t	1	Salmincola thymalli	Thymallus arcticus	6	0.02	0.03	0.01	0.04
0	0	0.32	1	t	1	Philometroides huronensis	Catostomus commersoni	6	0.01	0.04	0.01	0.04
0	0	0.43	1	cv	1	Diphyllobothrium sp. (L)	Salmo gairdneri	6	0.04	0.05	0.01	0.04
0	0	0.45	1	t	1	Diphyllobothrium sp. (L)	Thymallus arcticus	6	0.04	0.05	0.01	0.04
1	1	0.91	1	t	1	Diphyllobothrium sp. (L)	Salvelinus malma	6	0.04	0.05	0.07	0.05
1	1	0.74	1	t	1	Proteocephalus	Salvelinus malma	6	0.01	0.06	0.07	0.05
0	0	0.39	1	cv	1	Neoechinorhynchus	Richardsonius balteatus	6	0.02	0.03	0.01	0.04
0	1	0.57	1	t	1	Diplostomum sp.1 (L)	Richardsonius balteatus	6	0.05	0.05	0.01	0.04
1	1	0.90	1	t	1	Diplostomum sp.1 (L)	Ptychocheilus oregonensis	6	0.05	0.05	0.05	0.05
0	0	0.23	1	t	1	Neoechinorhynchus rutili	Salmo gairdneri	6	0.01	0.05	0.01	0.04
0	0	0.24	1	cv	1	Neoechinorhynchus rutili	Thymallus arcticus	6	0.01	0.05	0.01	0.04
0	0	0.23	1	t	1	Neoechinorhynchus rutili	Richardsonius balteatus	6	0.01	0.05	0.01	0.04

0	1	0.80	1	t	1	Neoechinorhynchus rutili	Salvelinus malma	6	0.01	0.05	0.07	0.05
0	1	0.66	1	t	1	Neoechinorhynchus rutili	Prosopium coulteri	6	0.01	0.05	0.05	0.05
0	0	0.27	1	cv	1	Philonema agubernaculu	Salmo gairdneri	6	0.01	0.04	0.01	0.04
0	0	0.29	1	t	1	Monobothrium sp.	Catostomus commersoni	6	0.01	0.03	0.01	0.04
0	0	0.23	1	t	1	Ligula intestinalis (L)	Richardsonius balteatus	6	0.01	0.05	0.01	0.04
1	1	0.74	1	t	1	Ligula intestinalis (L)	Cottus cognatus	6	0.01	0.05	0.06	0.05
1	1	0.97	1	cv	1	Salmincola edwardsii	Salvelinus malma	6	0.07	0.05	0.07	0.05
0	0	0.47	1	t	1	Salmo gairdneri	Salmincola californiensis	6	0.01	0.04	0.04	0.05
0	1	0.52	1	t	1	Salmo gairdneri	Ergasilus nerkae	6	0.01	0.04	0.04	0.05
0	0	0.19	1	t	1	Salmo gairdneri	Crepidostomum farionis	6	0.01	0.04	0.01	0.05
0	0	0.28	1	cv	1	Salmo gairdneri	Cystidicoloides tenuissima	6	0.01	0.04	0.01	0.03
0	0	0.22	1	t	1	Salmo gairdneri	Proteocephalus tumidocollus	6	0.01	0.04	0.01	0.04
0	0	0.22	1	t	1	Salmo gairdneri	Bunodera luciopercae	6	0.01	0.04	0.01	0.04
1	1	0.86	1	t	1	Rhabdochona cotti	Cottus cognatus	6	0.03	0.05	0.06	0.05
0	0	0.29	1	cv	1	Glaridacris catostomi	Catostomus commersoni	6	0.01	0.03	0.01	0.04
1	1	0.79	1	t	1	Rhabdochona canadensis	Salvelinus malma	6	0.01	0.06	0.07	0.05
0	1	0.68	1	t	1	Proteocephalus	Ptychocheilus oregonensis	6	0.01	0.05	0.05	0.05
0	1	0.57	1	t	1	Ergasilus nerkae	Catostomus commersoni	6	0.04	0.05	0.01	0.04
0	1	0.51	1	cv	1	Ergasilus nerkae	Thymallus arcticus	6	0.04	0.05	0.01	0.04
1	1	0.87	1	t	1	Ergasilus nerkae	Ptychocheilus oregonensis	6	0.04	0.05	0.05	0.05
0	0	0.19	1	t	1	Crepidostomum farionis	Thymallus arcticus	6	0.01	0.05	0.01	0.04
0	1	0.69	1	t	1	Crepidostomum farionis	Cottus cognatus	6	0.01	0.05	0.06	0.05
0	1	0.75	1	cv	1	Crepidostomum farionis	Salvelinus malma	6	0.01	0.05	0.07	0.05
0	0	0.38	1	t	1	Catostomus commersoni	Anoncohaptor anomalum	6	0.01	0.04	0.02	0.04
1	1	0.93	1	t	1	Haplonema hamatulum	Salvelinus malma	6	0.04	0.05	0.07	0.05

0	0	0.29	1	t	1	Thymallus arcticus	Cystidicoloides tenuissima	6	0.01	0.04	0.01	0.03
1	1	0.89	1	cv	1	Cottus cognatus	Eustrongylides sp. (L)	6	0.06	0.05	0.04	0.05
0	1	0.66	1	t	1	Ptychocheilus oregonensis	Allocreadium isosporum	6	0.05	0.05	0.01	0.05
0	1	0.59	1	t	1	Ptychocheilus oregonensis	Raphidascaris acus	6	0.05	0.05	0.01	0.05
1	1	0.92	1	t	1	Eustrongylides sp. (L)	Salvelinus malma	6	0.04	0.05	0.07	0.05
1	1	0.74	1	cv	1	Hysterothylacium	Salvelinus malma	6	0.01	0.06	0.07	0.05
1	1	0.60	0	t	1	Salmincola thymalli	Tetraonchus variabilis	6	0.02	0.03	0.02	0.03
0	1	0.77	0	t	1	Acolpenteron catostomi	Cottus cognatus	6	0.01	0.04	0.06	0.05
0	1	0.81	0	t	1	Philonema agubernaculu	Salmincola edwardsii	6	0.01	0.04	0.07	0.05
0	1	0.50	0	cv	1	Haplonema hamatulum	Richardsonius balteatus	6	0.04	0.05	0.01	0.04
0	1	0.57	0	t	1	Catostomus catostomus	Rhabdochona cotti	6	0.02	0.04	0.03	0.05
0	1	0.74	0	t	1	Salmincola edwardsii	Glaridacris catostomi	6	0.07	0.05	0.01	0.03
0	0	0.36	0	t	1	Truttaedacnitis truttae	Crepidostomum farionis	6	0.03	0.05	0.01	0.05
0	1	0.59	0	cv	1	Catostomus catostomus	Glaridacris sp.	6	0.02	0.04	0.03	0.04
0	1	0.61	0	t	1	Diplostomum sp.1 (L)	Neoechinorhynchus rutili	6	0.05	0.05	0.01	0.05
0	1	0.78	0	t	1	Hunterella nodulosa	Salvelinus malma	6	0.01	0.04	0.07	0.05
0	1	0.81	0	t	1	Pseudomurraytrema	Rhabdochona kisutchi	6	0.02	0.04	0.06	0.06
0	0	0.29	0	cv	1	Phyllodistomum sp.	Monobothrium sp.	6	0.01	0.04	0.01	0.03
0	1	0.55	0	t	1	Salvelinus namaycush	Glaridacris catostomi	6	0.04	0.05	0.01	0.03
0	0	0.25	0	t	1	Neoechinorhynchus rutili	Rhabdochona canadensis	6	0.01	0.05	0.01	0.06
0	1	0.78	0	t	1	Diplostomum sp.1 (L)	Rhabdochona cotti	6	0.05	0.05	0.03	0.05
0	1	0.81	0	cv	1	Allocreadium isosporum	Salvelinus malma	6	0.01	0.05	0.07	0.05
0	0	0.19	0	t	1	Hysterothylacium brachyurum	Proteocephalus tumidocollus	6	0.01	0.06	0.01	0.04
0	0	0.25	0	t	1	Philonema sp.	Glaridacris catostomi	6	0.01	0.05	0.01	0.03
0	0	0.21	0	t	1	Rhabdochona zacconis	Glaridacris vogei	6	0.01	0.04	0.01	0.04

0	1	0.70	0	cv	1	Rhabdochona milleri	Diplostomum sp.2 (L)	6	0.01	0.02	0.05	0.05
0	1	0.60	0	t	1	Philonema agubernaculu	Ergasilus nerkae	6	0.01	0.04	0.04	0.05
0	0	0.20	0	t	1	Neoechinorhynchus rutili	Hysterothylacium brachyurum	6	0.01	0.05	0.01	0.06
0	0	0.36	0	t	1	Catostomus catostomus	Neoechinorhynchus rutili	6	0.02	0.04	0.01	0.05
0	1	0.77	0	cv	1	Catostomus commersoni	Cottus cognatus	6	0.01	0.04	0.06	0.05
0	0	0.21	0	t	1	Salmo gairdneri	Thymallus arcticus	6	0.01	0.04	0.01	0.04
1	1	0.74	0	t	1	Diplostomum sp.2 (L)	Bothriocephalus opsariichthydis	6	0.05	0.05	0.02	0.05
0	1	0.70	0	t	1	Cystidicola farionis	Prosopium coulteri	6	0.02	0.05	0.05	0.05
0	1	0.55	0	cv	1	Diplostomum sp.2 (L)	Proteocephalus salmonidicola	6	0.05	0.05	0.01	0.06
0	1	0.81	0	t	1	Neoechinorhynchus	Cottus cognatus	6	0.02	0.04	0.06	0.05
0	1	0.61	0	t	1	Coregonus clupeaformis	Ligula intestinalis (L)	6	0.05	0.05	0.01	0.05
0	0	0.26	0	t	1	Cystidicola farionis	Salmo gairdneri	6	0.02	0.05	0.01	0.04
0	0	0.34	0	cv	1	Eubothrium salvelini	Raphidascaris acus	6	0.03	0.05	0.01	0.05
0	0	0.21	0	t	1	Tetracotyle sp. (L)	Proteocephalus tumidocollus	6	0.01	0.05	0.01	0.04
0	0	0.33	0	t	1	Rhabdochona zacconis	Catostomus catostomus	6	0.01	0.04	0.02	0.04
0	0	0.40	0	t	1	Neoechinorhynchus	Cystidicoloides tenuissima	6	0.02	0.04	0.01	0.03
0	0	0.29	0	cv	1	Phyllodistomum sp.	Neoechinorhynchus rutili	6	0.01	0.04	0.01	0.05
0	1	0.53	0	t	1	Philometroides huronensis	Diphyllobothrium sp. (L)	6	0.01	0.04	0.04	0.05
0	0	0.24	0	t	1	Philonema sp.	Rhabdochona canadensis	6	0.01	0.05	0.01	0.06
0	1	0.82	0	t	1	Phyllodistomum sp.	Salvelinus malma	6	0.01	0.04	0.07	0.05
0	1	0.72	0	cv	1	Thymallus arcticus	Cottus cognatus	6	0.01	0.04	0.06	0.05
0	1	0.57	0	t	1	Tetracotyle sp. (L)	Diplostomum sp.1 (L)	6	0.01	0.05	0.05	0.05

0	0	0.22	0	t	1	Glaridacris vogei	Bunodera luciopercae	6	0.01	0.04	0.01	0.04
0	1	0.66	0	t	1	Lota lota	Salmincola californiensis	6	0.03	0.05	0.04	0.05
0	1	0.67	0	cv	1	Catostomus macrocheilus	Tetraonchus borealis	6	0.04	0.04	0.01	0.03
0	1	0.52	0	t	1	Phyllodistomum sp.	Glaridacris sp.	6	0.01	0.04	0.03	0.04
0	1	0.73	0	t	1	Salmincola edwardsii	Proteocephalus tumidocollus	6	0.07	0.05	0.01	0.04
1	1	0.72	0	t	1	Pomphorhynchus bulbocolli	Hunterella nodulosa	6	0.06	0.05	0.01	0.04
0	0	0.49	0	cv	1	Glaridacris sp.	Allocreadium isosporum	6	0.03	0.04	0.01	0.05
0	1	0.73	0	t	1	Eubothrium salvelini	Octomacrum lanceatus	6	0.03	0.05	0.04	0.04
0	0	0.22	0	t	1	Rhabdochona cascadilla	Proteocephalus sp.	6	0.01	0.05	0.01	0.05
0	0	0.22	0	t	1	Neascus sp (L)	Crepidostomum farionis	6	0.01	0.05	0.01	0.05
0	0	0.42	0	cv	1	Hunterella nodulosa	Rhabdochona cotti	6	0.01	0.04	0.03	0.05
0	0	0.29	0	t	1	Neoechinorhynchus rutili	Catostomus commersoni	6	0.01	0.05	0.01	0.04
0	0	0.33	0	t	1	Glaridacris catostomi	Rhinichthys cataractae	6	0.01	0.03	0.01	0.03
0	0	0.34	0	t	1	Mylocheilus caurinus	Proteocephalus sp.	6	0.03	0.05	0.01	0.05
0	1	0.65	0	cv	1	Thymallus arcticus	Ptychocheilus oregonensis	6	0.01	0.04	0.05	0.05
0	0	0.34	0	t	1	Neoechinorhynchus	Glaridacris catostomi	6	0.02	0.04	0.01	0.03
1	1	0.83	0	t	1	Diplostomum sp.2 (L)	Salmincola californiensis	6	0.05	0.05	0.04	0.05
0	1	0.66	0	t	1	Philonema sp.	Ptychocheilus oregonensis	6	0.01	0.05	0.05	0.05
0	0	0.46	0	cv	1	Proteocephalus sp.	Eustrongylides sp. (L)	6	0.01	0.05	0.04	0.05
0	1	0.67	0	t	1	Glaridacris catostomi	Prosopium coulteri	6	0.01	0.03	0.05	0.05
0	0	0.42	0	t	1	Rhabdochona cotti	Bunodera luciopercae	6	0.03	0.05	0.01	0.04
1	1	0.86	0	t	1	Eubothrium salvelini	Pomphorhynchus bulbocolli	6	0.03	0.05	0.06	0.05
0	0	0.29	0	cv	1	Acolpenteron catostomi	Monobothrium sp.	6	0.01	0.04	0.01	0.03
0	1	0.53	0	t	1	Rhabdochona canadensis	Ergasilus nerkae	6	0.01	0.06	0.04	0.05

0	1	0.60	0	t	1	Mylocheilus caurinus	Neoechinorhynchus cylindratus	6	0.03	0.05	0.02	0.03
0	1	0.67	0	t	1	Glaridacris catostomi	Ptychocheilus oregonensis	6	0.01	0.03	0.05	0.05
0	0	0.40	0	cv	1	Bothriocephalus	Catostomus commersoni	6	0.02	0.05	0.01	0.04
0	1	0.75	0	t	1	Octospinifer macilentus	Ptychocheilus oregonensis	6	0.02	0.04	0.05	0.05
0	1	0.67	0	t	1	Eubothrium salvelini	Eustrongylides sp. (L)	6	0.03	0.05	0.04	0.05
1	1	0.52	0	t	1	Neoechinorhynchus	Salmincola thymalli	6	0.02	0.04	0.02	0.03
0	1	0.52	0	cv	1	Lota lota	Catostomus catostomus	6	0.03	0.05	0.02	0.04
0	0	0.28	0	t	1	Proteocephalus	Allocreadium isosporum	6	0.01	0.05	0.01	0.05
1	1	0.91	0	t	1	Pomphorhynchus bulbocolli	Eustrongylides sp. (L)	6	0.06	0.05	0.04	0.05
0	0	0.21	0	t	1	Hunterella nodulosa	Glaridacris vogei	6	0.01	0.04	0.01	0.04
0	1	0.59	0	cv	1	Octospinifer macilentus	Diphyllobothrium sp. (L)	6	0.02	0.04	0.04	0.05
0	0	0.24	0	t	1	Acolpenteron catostomi	Crepidostomum farionis	6	0.01	0.04	0.01	0.05
0	0	0.41	0	t	1	Neoechinorhynchus cylindratus	Proteocephalus tumidocollus	6	0.02	0.03	0.01	0.04
0	0	0.38	0	t	1	Pseudomurraytrema	Philometroides huronensis	6	0.02	0.04	0.01	0.04
1	1	0.87	0	cv	1	Salmincola edwardsii	Rhabdochona cotti	6	0.07	0.05	0.03	0.05
0	1	0.89	0	t	1	Tetraonchus variabilis	Salvelinus malma	6	0.02	0.03	0.07	0.05
0	0	0.43	0	t	1	Rhinichthys cataractae	Anoncohaptor anomalum	6	0.01	0.03	0.02	0.04
0	0	0.29	0	t	1	Pellucidhaptor pellucidhaptor	Philonema agubernaculum	6	0.01	0.04	0.01	0.04
0	0	0.49	0	cv	1	Rhabdochona cascadilla	Glaridacris sp.	6	0.01	0.05	0.03	0.04
0	0	0.33	0	t	1	Bothriocephalus	Hunterella nodulosa	6	0.02	0.05	0.01	0.04
0	0	0.19	0	t	1	Glaridacris vogei	Crepidostomum farionis	6	0.01	0.04	0.01	0.05
0	0	0.20	0	t	1	Prosopium williamsoni	Hunterella nodulosa	6	0.01	0.04	0.01	0.04
0	0	0.28	0	cv	1	Phyllodistomum sp.	Bunodera luciopercae	6	0.01	0.04	0.01	0.04
0	0	0.31	0	t	1	Cystidicola farionis	Rhabdochona cascadilla	6	0.02	0.05	0.01	0.05

0	0	0.28	0	t	1	Glaridacris catostomi	Allocreadium isosporum	6	0.01	0.03	0.01	0.05
0	0	0.31	0	t	1	Neoechinorhy nchus	Richardsonius balteatus	6	0.02	0.04	0.01	0.04
0	0	0.28	0	cv	1	Philonema sp.	Catostomus commersoni	6	0.01	0.05	0.01	0.04
0	1	0.63	0	t	1	Salmincola thymalli	Rhabdochona cotti	6	0.02	0.03	0.03	0.05
0	0	0.48	0	t	1	Philonema sp.	Diphyllobothr ium sp. (L)	6	0.01	0.05	0.04	0.05
0	1	0.71	0	t	1	Prosopium williamsoni	Rhabdochona kisutchi	6	0.01	0.04	0.06	0.06
1	1	0.90	0	cv	1	Rhabdochona cotti	Salvelinus malma	6	0.03	0.05	0.07	0.05
0	0	0.16	0	t	1	Proteocephalu s	Crepidostom um farionis	6	0.01	0.06	0.01	0.05
0	0	0.19	0	t	1	Tetracotyle sp. (L)	Rhabdochona zacconis	6	0.01	0.05	0.01	0.04
0	0	0.42	0	t	1	Pellucidhaptor pellucidhaptor	Tetraonchus variabilis	6	0.01	0.04	0.02	0.03
0	0	0.37	0	cv	1	Lota lota	Thymallus arcticus	6	0.03	0.05	0.01	0.04
0	0	0.34	0	t	1	Catostomus commersoni	Cystidicoloide s tenuissima	6	0.01	0.04	0.01	0.03
0	0	0.26	0	t	1	Tetracotyle sp. (L)	Cystidicoloide s tenuissima	6	0.01	0.05	0.01	0.03
0	1	0.52	0	t	1	Octospinifer macilentus	Mylocheilus caurinus	6	0.02	0.04	0.03	0.05
0	1	0.80	0	cv	1	Mylocheilus caurinus	Ptychocheilus oregonensis	6	0.03	0.05	0.05	0.05
1	0	0.42	0	t	1	Neoechinorhy nchus	Tetraonchus borealis	6	0.02	0.04	0.01	0.03
0	0	0.31	0	t	1	Tetraonchus borealis	Glaridacris vogei	6	0.01	0.03	0.01	0.04
0	1	0.71	0	t	1	Prosopium williamsoni	Cottus cognatus	6	0.01	0.04	0.06	0.05
0	0	0.28	0	cv	1	Philometroide s huronensis	Rhabdochona canadensis	6	0.01	0.04	0.01	0.06
0	1	0.63	0	t	1	Diplostomum sp.2 (L)	Neoechinorh ynchus rutili	6	0.05	0.05	0.01	0.05
0	0	0.47	0	t	1	Neoechinorhy nchus cristatus	Bothriocepha lus opsariichthyd is	6	0.02	0.04	0.02	0.05
0	0	0.26	0	t	1	Rhabdochona zacconis	Philometroid es huronensis	6	0.01	0.04	0.01	0.04
0	0	0.23	0	cv	1	Pellucidhaptor pellucidhaptor	Glaridacris vogei	6	0.01	0.04	0.01	0.04
0	0	0.35	0	t	1	Oncorhynchus nerka	Tetraonchus borealis	6	0.01	0.05	0.01	0.03

0	0	0.50	0	t	1	Mylocheilus caurinus	Rhinichthys cataractae	6	0.03	0.05	0.01	0.03
0	0	0.36	0	t	1	Pseudomurraytrema copulata	Proteocephalus ptychocheilus	6	0.02	0.04	0.01	0.05
0	1	0.75	0	cv	1	Pomphorhynchus bulbocolli	Philonema sp.	6	0.06	0.05	0.01	0.05
0	1	0.76	0	t	1	Oncorhynchus nerka	Rhabdochona kisutchi	6	0.01	0.05	0.06	0.06
0	0	0.34	0	t	1	Octospinifer macilentus	Philonema sp.	6	0.02	0.04	0.01	0.05
0	1	0.54	0	t	1	Salvelinus namaycush	Rhabdochona canadensis	6	0.04	0.05	0.01	0.06
0	1	0.65	0	cv	1	Bothriocephalus	Eustrongylides sp. (L)	6	0.02	0.05	0.04	0.05
0	1	0.58	0	t	1	Salvelinus namaycush	Proteocephalus ptychocheilus	6	0.04	0.05	0.01	0.05
0	0	0.16	0	t	1	Proteocephalus sp.	Proteocephalus salmonidicola	6	0.01	0.05	0.01	0.06
0	1	0.56	0	t	1	Pellucidhaptor pellucidhaptor	Haplonema hamatulum	6	0.01	0.04	0.04	0.05
0	1	0.63	0	cv	1	Glaridacris vogei	Prosopium coulteri	6	0.01	0.04	0.05	0.05
0	1	0.63	0	t	1	Eubothrium salvelini	Diphyllobothrium sp. (L)	6	0.03	0.05	0.04	0.05
0	1	0.87	0	t	1	Coregonus clupeaformis	Catostomus macrocheilus	6	0.05	0.05	0.04	0.04
0	0	0.41	0	t	1	Tetraonchus variabilis	Bunodera luciopercae	6	0.02	0.03	0.01	0.04
0	0	0.31	0	cv	1	Philometroides huronensis	Proteocephalus ptychocheilus	6	0.01	0.04	0.01	0.05
0	0	0.34	0	t	1	Octospinifer macilentus	Monobothrium sp.	6	0.02	0.04	0.01	0.03
0	0	0.30	0	t	1	Raphidascaris acus	Anoncohaptor anomalum	6	0.01	0.05	0.02	0.04
0	0	0.37	0	t	1	Eubothrium salvelini	Bunodera luciopercae	6	0.03	0.05	0.01	0.04
0	0	0.32	0	cv	1	Rhinichthys cataractae	Proteocephalus tumidocollus	6	0.01	0.03	0.01	0.04
0	0	0.34	0	t	1	Proteocephalus	Tetraonchus variabilis	6	0.01	0.06	0.02	0.03
0	1	0.65	0	t	1	Diphyllobothrium sp. (L)	Tetraonchus variabilis	6	0.04	0.05	0.02	0.03

0	0	0.47	0	t	1	Mylocheilus caurinus	Cystidicoloides tenuissima	6	0.03	0.05	0.01	0.03
0	0	0.50	0	cv	1	Rhabdochona milleri	Truttaedacnitis truttae	6	0.01	0.02	0.03	0.05
0	0	0.24	0	t	1	Rhabdochona milleri	Proteocephalus salmonidicola	6	0.01	0.02	0.01	0.06
0	0	0.25	0	t	1	Neoechinorhynchus rutili	Proteocephalus tumidocollus	6	0.01	0.05	0.01	0.04
0	0	0.27	0	t	1	Neoechinorhynchus rutili	Allocreadium isosporum	6	0.01	0.05	0.01	0.05
0	1	0.58	0	cv	1	Neoechinorhynchus	Glaridacris sp.	6	0.02	0.04	0.03	0.04
0	0	0.46	0	t	1	Phyllodistomum sp.	Tetraonchus variabilis	6	0.01	0.04	0.02	0.03
0	0	0.29	0	t	1	Phyllodistomum sp.	Ligula intestinalis (L)	6	0.01	0.04	0.01	0.05
0	0	0.24	0	t	1	Pellucidhaptor pellucidhaptor	Rhabdochona canadensis	6	0.01	0.04	0.01	0.06
0	0	0.27	0	cv	1	Octospinifer macilentus	Proteocephalus salmonidicola	6	0.02	0.04	0.01	0.06
0	1	0.56	0	t	1	Octomacrum lanceatus	Thymallus arcticus	6	0.04	0.04	0.01	0.04
0	1	0.71	0	t	1	Philonema agubernaculu	Prosopium coulteri	6	0.01	0.04	0.05	0.05
0	1	0.60	0	t	1	Acolpenteron catostomi	Ergasilus nerkae	6	0.01	0.04	0.04	0.05
0	1	0.58	0	cv	1	Diplostomum sp.1 (L)	Thymallus arcticus	6	0.05	0.05	0.01	0.04
1	1	0.89	0	t	1	Diplostomum sp.2 (L)	Diplostomum sp.1 (L)	6	0.05	0.05	0.05	0.05
0	0	0.40	0	t	1	Salmincola thymalli	Rhabdochona canadensis	6	0.02	0.03	0.01	0.06
0	0	0.28	0	t	1	Philonema sp.	Phyllodistomum sp.	6	0.01	0.05	0.01	0.04
0	1	0.71	0	cv	1	Truttaedacnitis truttae	Eustrongylides sp. (L)	6	0.03	0.05	0.04	0.05
0	0	0.45	0	t	1	Salmincola thymalli	Catostomus commersoni	6	0.02	0.03	0.01	0.04
0	0	0.47	0	t	1	Diphyllobothrium sp. (L)	Glaridacris catostomi	6	0.04	0.05	0.01	0.03
0	1	0.85	0	t	1	Lota lota	Rhabdochona kisutchi	6	0.03	0.05	0.06	0.06
0	0	0.27	0	cv	1	Glaridacris vogei	Philonema agubernaculum	6	0.01	0.04	0.01	0.04

0	1	0.74	0	t	1	Neoechinorhynchus	Prosopium coulteri	6	0.02	0.04	0.05	0.05
0	0	0.32	0	t	1	Rhabdochona milleri	Allocreadium isosporum	6	0.01	0.02	0.01	0.05
0	0	0.47	1	t	1	Etheostoma exile	Azygia angusticauda	7	0.02	0.04	0.02	0.04
0	0	0.33	1	cv	1	Etheostoma exile	Illinobdella sp.	7	0.02	0.04	0.01	0.04
0	0	0.45	1	t	1	Etheostoma exile	Rhabdochona cascadilla	7	0.02	0.04	0.02	0.04
0	1	0.72	1	t	1	Etheostoma exile	Posthodiplostomum minimum (L)	7	0.02	0.04	0.05	0.04
0	0	0.47	1	t	1	Etheostoma exile	Tetracotyle sp. (L)	7	0.02	0.04	0.02	0.04
0	0	0.36	1	cv	1	Etheostoma exile	Bothriocephalus formosus	7	0.02	0.04	0.01	0.04
0	1	0.53	1	t	1	Crepidostomum isostomum	Percopsis omiscomaycus	7	0.01	0.04	0.03	0.04
0	1	0.53	1	t	1	Crepidostomum isostomum	Etheostoma nigrum	7	0.01	0.04	0.03	0.04
0	0	0.39	1	t	1	Azygia longa	Esox lucius	7	0.00	0.04	0.03	0.04
0	1	0.64	1	cv	1	Esox lucius	Leptorhynchoides thecatus	7	0.03	0.04	0.03	0.04
0	1	0.53	1	t	1	Esox lucius	Neoechinorhynchus cylindratus	7	0.03	0.04	0.02	0.04
0	1	0.66	1	t	1	Esox lucius	Neoechinorhynchus rutili	7	0.03	0.04	0.03	0.04
0	0	0.38	1	t	1	Esox lucius	Proteocephalus pinguis	7	0.03	0.04	0.00	0.04
0	0	0.38	1	cv	1	Esox lucius	Triaenophorus crassus	7	0.03	0.04	0.00	0.04
1	1	0.78	1	t	1	Esox lucius	Echinorhynchus salmonis	7	0.03	0.04	0.04	0.04
0	1	0.54	1	t	1	Esox lucius	Pomphorhynchus bulbocolli	7	0.03	0.04	0.02	0.04
1	1	0.87	1	t	1	Esox lucius	Diplostomulum sp. (L)	7	0.03	0.04	0.06	0.04
0	1	0.54	1	cv	1	Esox lucius	Contracaecum brachyurum	7	0.03	0.04	0.02	0.04
0	0	0.44	1	t	1	Proteocephalus stizostethi	Stizostedion vitreum	7	0.00	0.04	0.03	0.04
0	0	0.38	1	t	1	Leptorhynchoides thecatus	Catostomus catostomus	7	0.03	0.04	0.00	0.04
0	0	0.37	1	t	1	Leptorhynchoides thecatus	Micropterus dolomieui	7	0.03	0.04	0.00	0.04

1	1	0.68	1	cv	1	Leptorhynchoides thecatus	Stizostedion vitreum	7	0.03	0.04	0.03	0.04
1	1	0.86	1	t	1	Leptorhynchoides thecatus	Perca flavescens	7	0.03	0.04	0.06	0.04
1	1	0.68	1	t	1	Leptorhynchoides thecatus	Micropterus salmoides	7	0.03	0.04	0.03	0.04
0	0	0.41	1	t	1	Leptorhynchoides thecatus	Notropis hudsonius	7	0.03	0.04	0.01	0.04
1	1	0.84	1	cv	1	Leptorhynchoides thecatus	Ambloplites rupestris	7	0.03	0.04	0.05	0.04
1	1	0.70	1	t	1	Leptorhynchoides thecatus	Etheostoma nigrum	7	0.03	0.04	0.03	0.04
1	1	0.75	1	t	1	Leptorhynchoides thecatus	Lepomis gibbosus	7	0.03	0.04	0.04	0.04
1	1	0.69	1	t	1	Bucephalus elegans	Perca flavescens	7	0.00	0.04	0.06	0.04
1	1	0.72	1	cv	1	Bothriocephalus sp.	Perca flavescens	7	0.01	0.04	0.06	0.04
0	0	0.44	1	t	1	Bothriocephalus sp.	Lota lota	7	0.01	0.04	0.03	0.04
0	1	0.50	1	t	1	Coregonus clupeaformis	Cystidicola stigmatura	7	0.03	0.04	0.01	0.04
0	0	0.50	1	t	1	Coregonus clupeaformis	Spinitectus gracilis (L)	7	0.03	0.04	0.01	0.04
0	0	0.39	1	cv	1	Coregonus clupeaformis	Cyathocephalus americanus	7	0.03	0.04	0.00	0.04
0	0	0.40	1	t	1	Coregonus clupeaformis	Proteocephalus laruei	7	0.03	0.04	0.00	0.04
0	1	0.50	1	t	1	Coregonus clupeaformis	Philometra sp. (L)	7	0.03	0.04	0.01	0.04
0	1	0.50	1	t	1	Coregonus clupeaformis	Diphyllobothrium sp. (L)	7	0.03	0.04	0.01	0.04
1	1	0.85	1	cv	1	Coregonus clupeaformis	Achtheres corpulentus	7	0.03	0.04	0.05	0.04
1	1	0.79	1	t	1	Coregonus clupeaformis	Echinorhynchus salmonis	7	0.03	0.04	0.04	0.04
0	1	0.50	1	t	1	Coregonus clupeaformis	Triaenophorus crassus (L)	7	0.03	0.04	0.01	0.04
1	1	0.88	1	t	1	Coregonus clupeaformis	Diplostomulum sp. (L)	7	0.03	0.04	0.06	0.04
1	1	0.53	1	cv	1	Coregonus clupeaformis	Proteocephalus exiguus	7	0.03	0.04	0.02	0.04
0	0	0.41	1	t	1	Haplonema hamulatum	Lota lota	7	0.00	0.04	0.03	0.04
0	0	0.34	1	t	1	Allocreadium lobatum	Semotilus atromaculatus	7	0.01	0.04	0.02	0.04
0	0	0.26	1	t	1	Allocreadium lobatum	Notropis hudsonius	7	0.01	0.04	0.01	0.04

0	0	0.34	1	cv	1	Allocreadium lobatum	Notropis cornutus	7	0.01	0.04	0.02	0.04
0	0	0.20	1	t	1	Rhipidocotyle papillosa	Micropterus dolomieui	7	0.00	0.03	0.00	0.04
1	1	0.54	1	t	1	Plagioporus sinitsini	Catostomus commersoni	7	0.00	0.04	0.04	0.04
0	0	0.31	1	t	1	Azygia angusticauda	Micropterus dolomieui	7	0.02	0.04	0.00	0.04
1	1	0.82	1	cv	1	Azygia angusticauda	Perca flavescens	7	0.02	0.04	0.06	0.04
1	1	0.61	1	t	1	Azygia angusticauda	Micropterus salmoides	7	0.02	0.04	0.03	0.04
1	1	0.80	1	t	1	Azygia angusticauda	Ambloplites rupestris	7	0.02	0.04	0.05	0.04
0	0	0.31	1	t	1	Azygia angusticauda	Ictalurus nebulosus	7	0.02	0.04	0.00	0.04
1	1	0.69	1	cv	1	Azygia angusticauda	Lepomis gibbosus	7	0.02	0.04	0.04	0.04
0	0	0.50	1	t	1	Cystidicola stigmatura	Prosopium cylindraceum	7	0.01	0.04	0.03	0.04
0	0	0.50	1	t	1	Cystidicola stigmatura	Coregonus hoyi	7	0.01	0.04	0.03	0.04
0	1	0.59	1	t	1	Cystidicola stigmatura	Coregonus artedi	7	0.01	0.04	0.04	0.04
0	0	0.50	1	cv	1	Salmincola inermis	Coregonus artedi	7	0.00	0.04	0.04	0.04
0	0	0.21	1	t	1	Illinobdella sp.	Micropterus dolomieui	7	0.01	0.04	0.00	0.04
0	1	0.50	1	t	1	Illinobdella sp.	Etheostoma nigrum	7	0.01	0.04	0.03	0.04
1	1	0.54	1	t	1	Triganodistom um	Catostomus commersoni	7	0.00	0.04	0.04	0.04
0	0	0.48	1	cv	1	Achtheres micropteri	Micropterus dolomieui	7	0.04	0.04	0.00	0.04
0	0	0.37	1	t	1	Echinorhynchu s salmonis (L)	Osmerus mordax	7	0.01	0.04	0.02	0.04
0	1	0.56	1	t	1	Echinorhynchu s salmonis (L)	Coregonus artedi	7	0.01	0.04	0.04	0.04
0	0	0.21	1	t	1	Lebouria cooperi	Notropis hudsonius	7	0.00	0.04	0.01	0.04
0	0	0.28	1	cv	1	Lebouria cooperi	Notropis cornutus	7	0.00	0.04	0.02	0.04
0	0	0.47	1	t	1	Lebouria cooperi	Etheostoma nigrum	7	0.00	0.04	0.03	0.04
0	0	0.49	1	t	1	Eubothrium salvelini	Salvelinus namaycush	7	0.03	0.03	0.01	0.04
0	0	0.29	1	t	1	Neoechinorhy nchus	Micropterus dolomieui	7	0.02	0.04	0.00	0.04
1	1	0.59	1	cv	1	Neoechinorhy nchus	Stizostedion vitreum	7	0.02	0.04	0.03	0.04

1	1	0.81	1	t	1	Neoechinorhynchus	Perca flavescens	7	0.02	0.04	0.06	0.04
1	1	0.56	1	t	1	Neoechinorhynchus	Lota lota	7	0.02	0.04	0.03	0.04
1	1	0.59	1	t	1	Neoechinorhynchus	Micropterus salmoides	7	0.02	0.04	0.03	0.04
1	1	0.79	1	cv	1	Neoechinorhynchus	Ambloplites rupestris	7	0.02	0.04	0.05	0.04
1	1	0.61	1	t	1	Neoechinorhynchus	Etheostoma nigrum	7	0.02	0.04	0.03	0.04
1	1	0.67	1	t	1	Neoechinorhynchus	Lepomis gibbosus	7	0.02	0.04	0.04	0.04
0	0	0.39	1	t	1	Neoechinorhynchus rutili	Culaea inconstans	7	0.03	0.04	0.00	0.04
0	0	0.39	1	cv	1	Neoechinorhynchus rutili	Micropterus dolomieui	7	0.03	0.04	0.00	0.04
1	1	0.87	1	t	1	Neoechinorhynchus rutili	Perca flavescens	7	0.03	0.04	0.06	0.04
1	1	0.68	1	t	1	Neoechinorhynchus rutili	Lota lota	7	0.03	0.04	0.03	0.04
0	0	0.43	1	t	1	Neoechinorhynchus rutili	Notropis hudsonius	7	0.03	0.04	0.01	0.04
0	0	0.21	1	cv	1	Catostomus catostomus	Diplostomum flexicaudum (L)	7	0.00	0.04	0.01	0.04
0	0	0.20	1	t	1	Catostomus catostomus	Triganodistomum simeri	7	0.00	0.04	0.00	0.03
0	0	0.21	1	t	1	Catostomus catostomus	Actinobdella triannulata	7	0.00	0.04	0.01	0.04
0	0	0.35	1	t	1	Catostomus catostomus	Ergasilus caeruleus	7	0.00	0.04	0.02	0.04
0	1	0.59	1	cv	1	Catostomus catostomus	Posthodiplostomum minimum (L)	7	0.00	0.04	0.05	0.04
0	0	0.21	1	t	1	Catostomus catostomus	Neoechinorhynchus strigosus	7	0.00	0.04	0.01	0.04
0	1	0.57	1	t	1	Catostomus catostomus	Echinorhynchus salmonis	7	0.00	0.04	0.04	0.04
0	0	0.31	1	t	1	Catostomus catostomus	Echinorhynchus leidyi	7	0.00	0.04	0.02	0.04
0	0	0.26	1	cv	1	Catostomus catostomus	Triaenophorus crassus (L)	7	0.00	0.04	0.01	0.04
0	0	0.21	1	t	1	Catostomus catostomus	Neoechinorhynchus crassus	7	0.00	0.04	0.01	0.04
0	0	0.24	1	t	1	Dichelyne robusta	Ictalurus nebulosus	7	0.01	0.03	0.00	0.04
0	0	0.23	1	t	1	Ligula intestinalis (L)	Notropis hudsonius	7	0.01	0.04	0.01	0.04

1	1	0.58	1	cv	1	Ligula intestinalis (L)	Catostomus commersoni	7	0.01	0.04	0.04	0.04
0	1	0.56	1	t	1	Cottus bairdi	Tetracotyle sp. (L)	7	0.03	0.04	0.02	0.04
1	1	0.87	1	t	1	Cottus bairdi	Diplostomulum sp. (L)	7	0.03	0.04	0.06	0.04
0	0	0.20	1	t	1	Centrovarium lobotes	Ictalurus nebulosus	7	0.00	0.03	0.00	0.04
0	0	0.48	1	cv	1	Culaea inconstans	Neascus sp. (L)	7	0.00	0.04	0.04	0.04
0	0	0.19	1	t	1	Culaea inconstans	Bunoderina eucaliae	7	0.00	0.04	0.00	0.03
0	1	0.58	1	t	1	Culaea inconstans	Posthodiplostomum minimum (L)	7	0.00	0.04	0.05	0.04
1	1	0.71	1	t	1	Culaea inconstans	Diplostomulum sp. (L)	7	0.00	0.04	0.06	0.04
0	0	0.20	1	cv	1	Micropterus dolomieui	Camallanus oxycephalus	7	0.00	0.04	0.01	0.04
0	0	0.25	1	t	1	Micropterus dolomieui	Spinitectus gracilis (L)	7	0.00	0.04	0.01	0.04
0	0	0.48	1	t	1	Micropterus dolomieui	Neascus sp. (L)	7	0.00	0.04	0.04	0.04
0	0	0.18	1	t	1	Micropterus dolomieui	Crepidostomum cornutum	7	0.00	0.04	0.00	0.04
0	0	0.18	1	cv	1	Micropterus dolomieui	Crepidostomum cooperi	7	0.00	0.04	0.00	0.04
0	0	0.25	1	t	1	Micropterus dolomieui	Clinostomum marginatum (L)	7	0.00	0.04	0.01	0.04
0	0	0.20	1	t	1	Micropterus dolomieui	Cryptogonimus chyli	7	0.00	0.04	0.01	0.04
0	0	0.27	1	t	1	Micropterus dolomieui	Dichelyne cotylophora	7	0.00	0.04	0.02	0.04
0	0	0.25	1	cv	1	Micropterus dolomieui	Microphallus opacus	7	0.00	0.04	0.01	0.04
0	0	0.34	1	t	1	Micropterus dolomieui	Ergasilus caeruleus	7	0.00	0.04	0.02	0.04
0	1	0.58	1	t	1	Micropterus dolomieui	Posthodiplostomum minimum (L)	7	0.00	0.04	0.05	0.04
0	0	0.25	1	t	1	Micropterus dolomieui	Spinitectus carolini	7	0.00	0.04	0.01	0.04
0	1	0.57	1	cv	1	Micropterus dolomieui	Echinorhynchus salmonis	7	0.00	0.04	0.04	0.04
0	0	0.30	1	t	1	Micropterus dolomieui	Pomphorhynchus bulbocolli	7	0.00	0.04	0.02	0.04
0	1	0.71	1	t	1	Micropterus dolomieui	Diplostomulum sp. (L)	7	0.00	0.04	0.06	0.04

0	0	0.27	1	t	1	Micropterus dolomieui	Proteocephalus ambloplitis	7	0.00	0.04	0.02	0.04
0	0	0.30	1	cv	1	Micropterus dolomieui	Contracaecum brachyurum	7	0.00	0.04	0.02	0.04
0	0	0.40	1	t	1	Crepidostomum farionis	Prosopium cylindraceum	7	0.00	0.04	0.03	0.04
0	1	0.58	1	t	1	Rhinichthys cataractae	Posthodiplostomum minimum (L)	7	0.00	0.04	0.05	0.04
1	1	0.69	1	t	1	Camallanus oxycephalus	Ambloplites rupestris	7	0.01	0.04	0.05	0.04
0	0	0.25	1	cv	1	Centrovarium lobotes (L)	Notropis volucellus	7	0.01	0.04	0.01	0.04
0	0	0.30	1	t	1	Centrovarium lobotes (L)	Pimephales notatus	7	0.01	0.04	0.02	0.04
1	1	0.69	1	t	1	Illinobdella alba	Perca flavescens	7	0.00	0.04	0.06	0.04
0	1	0.58	1	t	1	Notropis heterolepis	Neascus sp. (L)	7	0.01	0.04	0.04	0.04
1	1	0.67	1	cv	1	Notropis heterolepis	Posthodiplostomum minimum (L)	7	0.01	0.04	0.05	0.04
0	0	0.30	1	t	1	Notropis heterolepis	Spiroxys sp. (L)	7	0.01	0.04	0.01	0.04
1	1	0.78	1	t	1	Notropis heterolepis	Diplostomulum sp. (L)	7	0.01	0.04	0.06	0.04
1	1	0.66	1	t	1	Salvelinus namaycush	Echinorhynchus salmonis	7	0.01	0.04	0.04	0.04
1	0	0.40	1	cv	1	Salvelinus namaycush	Echinorhynchus leidyi	7	0.01	0.04	0.02	0.04
1	1	0.79	1	t	1	Salvelinus namaycush	Diplostomulum sp. (L)	7	0.01	0.04	0.06	0.04
0	0	0.44	1	t	1	Bothriocephalus cuspidatus	Stizostedion vitreum	7	0.00	0.04	0.03	0.04
0	0	0.40	1	t	1	Neoechinorhynchus tumidus	Prosopium cylindraceum	7	0.00	0.04	0.03	0.04
1	1	0.58	1	cv	1	Diplostomum flexicaudum	Catostomus commersoni	7	0.01	0.04	0.04	0.04
0	1	0.54	1	t	1	Triaenophorus stizostedionis	Stizostedion vitreum	7	0.01	0.04	0.03	0.04
0	0	0.44	1	t	1	Salmincola wisconsinsensi	Coregonus hoyi	7	0.01	0.04	0.03	0.04
0	1	0.53	1	t	1	Salmincola wisconsinsensi	Coregonus artedi	7	0.01	0.04	0.04	0.04
0	0	0.39	1	cv	1	Spinitectus gracilis (L)	Osmerus mordax	7	0.01	0.04	0.02	0.04
0	0	0.28	1	t	1	Spinitectus gracilis (L)	Notropis hudsonius	7	0.01	0.04	0.01	0.04

0	0	0.49	1	t	1	Abothrium crassum	Lota lota	7	0.01	0.04	0.03	0.04
0	0	0.37	1	t	1	Abothrium crassum	Oncorhynchus mykiss	7	0.01	0.04	0.02	0.04
0	1	0.56	1	cv	1	Abothrium crassum	Coregonus artedi	7	0.01	0.04	0.04	0.04
1	1	0.75	1	t	1	Stizostedion vitreum	Neascus sp. (L)	7	0.03	0.04	0.04	0.04
0	0	0.42	1	t	1	Stizostedion vitreum	Bucephalopsis pusilla	7	0.03	0.04	0.00	0.04
0	1	0.55	1	t	1	Stizostedion vitreum	Dichelyne cotylophora	7	0.03	0.04	0.02	0.04
0	1	0.63	1	cv	1	Stizostedion vitreum	Ergasilus caeruleus	7	0.03	0.04	0.02	0.04
0	0	0.42	1	t	1	Stizostedion vitreum	Spinitectus sp.	7	0.03	0.04	0.00	0.04
1	1	0.89	1	t	1	Stizostedion vitreum	Diplostomulum sp. (L)	7	0.03	0.04	0.06	0.04
0	1	0.58	1	t	1	Stizostedion vitreum	Contracaecum brachyurum	7	0.03	0.04	0.02	0.04
1	1	0.72	1	cv	1	Illinobdella moorei	Perca flavescens	7	0.01	0.04	0.06	0.04
1	1	0.69	1	t	1	Illinobdella moorei	Ambloplites rupestris	7	0.01	0.04	0.05	0.04
0	0	0.43	1	t	1	Caecincola parvulus	Micropterus salmoides	7	0.00	0.04	0.03	0.04
1	1	0.79	1	t	1	Pomphorhynchus bulbocolli	Perca flavescens	7	0.02	0.04	0.06	0.04
0	1	0.58	1	cv	1	Pomphorhynchus bulbocolli (L)	Percopsis omiscomaycus	7	0.02	0.04	0.03	0.04
0	0	0.41	1	t	1	Pomphorhynchus bulbocolli	Osmerus mordax	7	0.02	0.04	0.02	0.04
0	0	0.30	1	t	1	Pomphorhynchus bulbocolli	Notropis hudsonius	7	0.02	0.04	0.01	0.04
0	0	0.27	1	t	1	Pomphorhynchus bulbocolli	Ictalurus nebulosus	7	0.02	0.04	0.00	0.04
1	1	0.90	1	cv	1	Neascus sp. (L)	Perca flavescens	7	0.04	0.04	0.06	0.04
0	1	0.59	1	t	1	Neascus sp. (L)	Semotilus atromaculatus	7	0.04	0.04	0.02	0.04
1	1	0.77	1	t	1	Neascus sp. (L)	Percopsis omiscomaycus	7	0.04	0.04	0.03	0.04
0	0	0.50	1	t	1	Neascus sp. (L)	Notropis hudsonius	7	0.04	0.04	0.01	0.04
1	1	0.89	1	cv	1	Neascus sp. (L)	Ambloplites rupestris	7	0.04	0.04	0.05	0.04

0	1	0.59	1	t	1	Neascus sp. (L)	Pimephales notatus	7	0.04	0.04	0.02	0.04
1	1	0.81	1	t	1	Neascus sp. (L)	Catostomus commersoni	7	0.04	0.04	0.04	0.04
1	1	0.60	1	t	1	Neascus sp. (L)	Notropis cornutus	7	0.04	0.04	0.02	0.04
1	1	0.77	1	cv	1	Neascus sp. (L)	Etheostoma nigrum	7	0.04	0.04	0.03	0.04
1	1	0.81	1	t	1	Neascus sp. (L)	Lepomis gibbosus	7	0.04	0.04	0.04	0.04
1	1	0.75	1	t	1	Perca flavescens	Proteocephalus pearsei	7	0.06	0.04	0.01	0.04
1	1	0.66	1	t	1	Perca flavescens	Bunodera sacculata	7	0.06	0.04	0.00	0.04
1	1	0.75	1	cv	1	Perca flavescens	Spinitectus gracilis	7	0.06	0.04	0.01	0.04
0	1	0.66	1	t	1	Perca flavescens	Crepidostomum cooperi	7	0.06	0.04	0.00	0.04
1	1	0.75	1	t	1	Perca flavescens	Clinostomum marginatum (L)	7	0.06	0.04	0.01	0.04
1	1	0.77	1	t	1	Perca flavescens	Dichelyne cotylophora	7	0.06	0.04	0.02	0.04
1	1	0.69	1	cv	1	Perca flavescens	Rhabdochona sp.	7	0.06	0.04	0.01	0.04
1	1	0.66	1	t	1	Perca flavescens	Philometra cylindracea (L)	7	0.06	0.04	0.00	0.04
1	1	0.83	1	t	1	Perca flavescens	Ergasilus caeruleus	7	0.06	0.04	0.02	0.04
1	1	0.93	1	t	1	Perca flavescens	Posthodiplostomum minimum (L)	7	0.06	0.04	0.05	0.04
1	1	0.92	1	cv	1	Perca flavescens	Echinorhynchus salmonis	7	0.06	0.04	0.04	0.04
1	1	0.79	1	t	1	Perca flavescens	Pomphorhynchus bulbocolli	7	0.06	0.04	0.02	0.04
0	1	0.81	1	t	1	Perca flavescens	Tetracotyle sp. (L)	7	0.06	0.04	0.02	0.04
1	1	0.73	1	t	1	Perca flavescens	Spiroxys sp. (L)	7	0.06	0.04	0.01	0.04
1	1	0.96	1	cv	1	Perca flavescens	Diplostomulum sp. (L)	7	0.06	0.04	0.06	0.04
1	1	0.77	1	t	1	Perca flavescens	Proteocephalus ambloplitis	7	0.06	0.04	0.02	0.04
1	1	0.64	1	t	1	Notropis volucellus	Posthodiplostomum minimum (L)	7	0.01	0.04	0.05	0.04

1	1	0.76	1	t	1	Notropis volucellus	Diplostomulum sp. (L)	7	0.01	0.04	0.06	0.04
1	0	0.42	1	cv	1	Semotilus atromaculatus	Rhabdochona cascadilla	7	0.02	0.04	0.02	0.04
1	1	0.70	1	t	1	Semotilus atromaculatus	Posthodiplostomum minimum (L)	7	0.02	0.04	0.05	0.04
1	1	0.80	1	t	1	Semotilus atromaculatus	Diplostomulum sp. (L)	7	0.02	0.04	0.06	0.04
1	1	0.51	1	t	1	Proteocephalus pearsei	Lota lota	7	0.01	0.04	0.03	0.04
1	1	0.74	1	cv	1	Proteocephalus pearsei	Ambloplites rupestris	7	0.01	0.04	0.05	0.04
1	1	0.61	1	t	1	Proteocephalus pearsei	Lepomis gibbosus	7	0.01	0.04	0.04	0.04
0	0	0.40	1	t	1	Proteocephalus laruei	Coregonus hoyi	7	0.00	0.04	0.03	0.04
0	0	0.49	1	t	1	Proteocephalus laruei	Coregonus artedi	7	0.00	0.04	0.04	0.04
0	0	0.20	1	cv	1	Crepidostomum ictaluri	Ictalurus nebulosus	7	0.00	0.03	0.00	0.04
1	1	0.58	1	t	1	Actinobdella triannulata	Catostomus commersoni	7	0.01	0.04	0.04	0.04
1	1	0.66	1	t	1	Achtheres ambloplitis	Ambloplites rupestris	7	0.00	0.04	0.05	0.04
0	0	0.33	1	t	1	Rhabdochona cascadilla	Notropis hudsonius	7	0.02	0.04	0.01	0.04
0	0	0.42	1	cv	1	Rhabdochona cascadilla	Pimephales notatus	7	0.02	0.04	0.02	0.04
0	0	0.42	1	t	1	Rhabdochona cascadilla	Notropis cornutus	7	0.02	0.04	0.02	0.04
0	1	0.62	1	t	1	Rhabdochona cascadilla	Etheostoma nigrum	7	0.02	0.04	0.03	0.04
0	0	0.43	1	t	1	Crepidostomum cornutum	Micropterus salmoides	7	0.00	0.04	0.03	0.04
1	1	0.66	1	cv	1	Crepidostomum cornutum	Ambloplites rupestris	7	0.00	0.04	0.05	0.04
0	1	0.51	1	t	1	Crepidostomum cornutum	Lepomis gibbosus	7	0.00	0.04	0.04	0.04
0	1	0.51	1	t	1	Spinitectus gracilis	Lota lota	7	0.01	0.04	0.03	0.04
0	1	0.55	1	t	1	Spinitectus gracilis	Percopsis omiscomaycus	7	0.01	0.04	0.03	0.04
0	0	0.24	1	cv	1	Spinitectus gracilis	Ictalurus nebulosus	7	0.01	0.04	0.00	0.04
1	1	0.64	1	t	1	Hepaticola bakeri	Catostomus commersoni	7	0.01	0.04	0.04	0.04
1	1	0.66	1	t	1	Crepidostomum cooperi	Ambloplites rupestris	7	0.00	0.04	0.05	0.04

1	1	0.51	1	t	1	Crepidostomum cooperi	Lepomis gibbosus	7	0.00	0.04	0.04	0.04
1	1	0.74	1	cv	1	Clinostomum marginatum	Ambloplites rupestris	7	0.01	0.04	0.05	0.04
1	1	0.61	1	t	1	Clinostomum marginatum	Lepomis gibbosus	7	0.01	0.04	0.04	0.04
1	1	0.69	1	t	1	Cryptogonimus chyli	Ambloplites rupestris	7	0.01	0.04	0.05	0.04
1	1	0.80	1	t	1	Lota lota	Echinorhynchus salmonis	7	0.03	0.04	0.04	0.04
0	1	0.56	1	cv	1	Lota lota	Pomphorhynchus bulbocolli	7	0.03	0.04	0.02	0.04
0	1	0.57	1	t	1	Lota lota	Echinorhynchus leidyi	7	0.03	0.04	0.02	0.04
1	1	0.88	1	t	1	Lota lota	Diplostomulum sp. (L)	7	0.03	0.04	0.06	0.04
1	1	0.54	1	t	1	Octospinifer macilentus	Catostomus commersoni	7	0.00	0.04	0.04	0.04
1	1	0.56	1	cv	1	Dichelyne cotylophora	Micropterus salmoides	7	0.02	0.04	0.03	0.04
1	1	0.77	1	t	1	Dichelyne cotylophora	Ambloplites rupestris	7	0.02	0.04	0.05	0.04
0	0	0.46	1	t	1	Neoechinorhynchus sp.	Percopsis omiscomaycus	7	0.00	0.04	0.03	0.04
0	0	0.50	1	t	1	Philometra sp. (L)	Prosopium cylindraceum	7	0.01	0.04	0.03	0.04
0	0	0.39	1	cv	1	Philometra sp. (L)	Osmerus mordax	7	0.01	0.04	0.02	0.04
0	1	0.59	1	t	1	Philometra sp. (L)	Coregonus artedi	7	0.01	0.04	0.04	0.04
0	1	0.65	1	t	1	Percopsis omiscomaycus	Ergasilus caeruleus	7	0.03	0.04	0.02	0.04
1	1	0.83	1	t	1	Percopsis omiscomaycus	Posthodiplostomum minimum (L)	7	0.03	0.04	0.05	0.04
1	1	0.82	1	cv	1	Percopsis omiscomaycus	Echinorhynchus salmonis	7	0.03	0.04	0.04	0.04
0	1	0.63	1	t	1	Percopsis omiscomaycus	Tetracotyle sp. (L)	7	0.03	0.04	0.02	0.04
1	1	0.90	1	t	1	Percopsis omiscomaycus	Diplostomulum sp. (L)	7	0.03	0.04	0.06	0.04
0	1	0.52	1	t	1	Percopsis omiscomaycus	Bothriocephalus formosus	7	0.03	0.04	0.01	0.04
0	1	0.60	1	cv	1	Percopsis omiscomaycus	Contracaecum brachyurum	7	0.03	0.04	0.02	0.04
0	0	0.48	1	t	1	Percopsis omiscomaycus	Argulus sp.	7	0.03	0.04	0.01	0.04

1	1	0.61	1	t	1	Glaridacris catostomi	Catostomus commersoni	7	0.01	0.04	0.04	0.04
1	1	0.53	1	t	1	Microphallus opacus	Micropterus salmoides	7	0.01	0.04	0.03	0.04
1	1	0.74	1	cv	1	Microphallus opacus	Ambloplites rupestris	7	0.01	0.04	0.05	0.04
0	0	0.24	1	t	1	Microphallus opacus	Ictalurus nebulosus	7	0.01	0.04	0.00	0.04
0	0	0.40	1	t	1	Salmincola sp.	Prosopium cylindraceum	7	0.00	0.04	0.03	0.04
1	1	0.82	1	t	1	Ergasilus caeruleus	Ambloplites rupestris	7	0.02	0.04	0.05	0.04
1	1	0.72	1	cv	1	Ergasilus caeruleus	Catostomus commersoni	7	0.02	0.04	0.04	0.04
1	1	0.71	1	t	1	Ergasilus caeruleus	Lepomis gibbosus	7	0.02	0.04	0.04	0.04
1	0	0.46	1	t	1	Prosopium cylindraceum	Discocotyle salmonis	7	0.03	0.04	0.01	0.04
1	1	0.78	1	t	1	Prosopium cylindraceum	Echinorhynchus salmonis	7	0.03	0.04	0.04	0.04
1	1	0.87	1	cv	1	Prosopium cylindraceum	Diplostomulum sp. (L)	7	0.03	0.04	0.06	0.04
1	1	0.52	1	t	1	Prosopium cylindraceum	Proteocephalus exiguus	7	0.03	0.04	0.02	0.04
1	0	0.49	1	t	1	Prosopium cylindraceum	Achtheres coregoni	7	0.03	0.04	0.01	0.04
1	1	0.66	1	t	1	Bothriocephalus claviceps	Ambloplites rupestris	7	0.00	0.04	0.05	0.04
1	1	0.51	1	cv	1	Bothriocephalus claviceps	Lepomis gibbosus	7	0.00	0.04	0.04	0.04
1	0	0.39	1	t	1	Oncorhynchus mykiss	Diphyllobothrium sp. (L)	7	0.02	0.04	0.01	0.04
1	1	0.71	1	t	1	Oncorhynchus mykiss	Echinorhynchus salmonis	7	0.02	0.04	0.04	0.04
1	0	0.45	1	t	1	Oncorhynchus mykiss	Echinorhynchus leidyi	7	0.02	0.04	0.02	0.04
1	1	0.82	1	cv	1	Oncorhynchus mykiss	Diplostomulum sp. (L)	7	0.02	0.04	0.06	0.04
0	0	0.33	1	t	1	Oncorhynchus mykiss	Metabronema salvelini	7	0.02	0.04	0.01	0.04
1	1	0.64	1	t	1	Salvelinus fontinalis	Posthodiplostomum minimum (L)	7	0.01	0.04	0.05	0.04
1	1	0.76	1	t	1	Salvelinus fontinalis	Diplostomulum sp. (L)	7	0.01	0.04	0.06	0.04
1	0	0.25	1	cv	1	Salvelinus fontinalis	Metabronema salvelini	7	0.01	0.04	0.01	0.04
0	0	0.50	1	t	1	Diphyllobothrium sp. (L)	Coregonus hoyi	7	0.01	0.04	0.03	0.04
0	1	0.59	1	t	1	Diphyllobothrium sp. (L)	Coregonus artedi	7	0.01	0.04	0.04	0.04

1	1	0.71	1	t	1	Osmerus mordax	Echinorhynchus salmonis	7	0.02	0.04	0.04	0.04
1	1	0.82	1	cv	1	Osmerus mordax	Diplostomulum sp. (L)	7	0.02	0.04	0.06	0.04
0	0	0.47	1	t	1	Discocotyle salmonis	Coregonus hoyi	7	0.01	0.04	0.03	0.04
0	1	0.56	1	t	1	Discocotyle salmonis	Coregonus artedi	7	0.01	0.04	0.04	0.04
0	1	0.52	1	t	1	Micropterus salmoides	Spinitectus carolini	7	0.03	0.04	0.01	0.04
1	1	0.81	1	cv	1	Micropterus salmoides	Echinorhynchus salmonis	7	0.03	0.04	0.04	0.04
1	1	0.89	1	t	1	Micropterus salmoides	Diplostomulum sp. (L)	7	0.03	0.04	0.06	0.04
0	1	0.55	1	t	1	Micropterus salmoides	Proteocephalus ambloplitis	7	0.03	0.04	0.02	0.04
0	1	0.58	1	t	1	Micropterus salmoides	Contracaecum brachyurum	7	0.03	0.04	0.02	0.04
0	1	0.62	1	cv	1	Posthodiplostomum minimum (L)	Chrosomus eos	7	0.05	0.04	0.01	0.04
0	1	0.59	1	t	1	Posthodiplostomum minimum (L)	Notropis hudsonius	7	0.05	0.04	0.01	0.04
1	1	0.92	1	t	1	Posthodiplostomum minimum (L)	Ambloplites rupestris	7	0.05	0.04	0.05	0.04
1	1	0.68	1	t	1	Posthodiplostomum minimum (L)	Pimephales notatus	7	0.05	0.04	0.02	0.04
1	1	0.68	1	cv	1	Posthodiplostomum minimum (L)	Notropis cornutus	7	0.05	0.04	0.02	0.04
1	1	0.83	1	t	1	Posthodiplostomum minimum (L)	Etheostoma nigrum	7	0.05	0.04	0.03	0.04
1	1	0.86	1	t	1	Posthodiplostomum minimum (L)	Lepomis gibbosus	7	0.05	0.04	0.04	0.04
0	1	0.55	1	t	1	Posthodiplostomum minimum (L)	Notropis atherinoides	7	0.05	0.04	0.00	0.04
1	1	0.74	1	cv	1	Spinitectus carolini	Ambloplites rupestris	7	0.01	0.04	0.05	0.04
1	1	0.61	1	t	1	Spinitectus carolini	Lepomis gibbosus	7	0.01	0.04	0.04	0.04
1	1	0.78	1	t	1	Coregonus hoyi	Echinorhynchus salmonis	7	0.03	0.04	0.04	0.04

1	1	0.55	1	t	1	Coregonus hoyi	Echinorhynchus leidyi	7	0.03	0.04	0.02	0.04
1	0	0.49	1	cv	1	Coregonus hoyi	Triaenophorus crassus (L)	7	0.03	0.04	0.01	0.04
1	1	0.87	1	t	1	Coregonus hoyi	Diplostomulum sp. (L)	7	0.03	0.04	0.06	0.04
1	1	0.52	1	t	1	Coregonus hoyi	Proteocephalus exiguus	7	0.03	0.04	0.02	0.04
0	0	0.38	1	t	1	Chrosomus eos	Tetracotyle sp. (L)	7	0.01	0.04	0.02	0.04
1	1	0.76	1	cv	1	Chrosomus eos	Diplostomulum sp. (L)	7	0.01	0.04	0.06	0.04
0	1	0.60	1	t	1	Notropis hudsonius	Echinorhynchus salmonis	7	0.01	0.04	0.04	0.04
0	0	0.35	1	t	1	Notropis hudsonius	Tetracotyle sp. (L)	7	0.01	0.04	0.02	0.04
1	1	0.74	1	t	1	Notropis hudsonius	Diplostomulum sp. (L)	7	0.01	0.04	0.06	0.04
0	0	0.21	1	cv	1	Notropis hudsonius	Bucephalus sp.	7	0.01	0.04	0.00	0.04
1	1	0.58	1	t	1	Neoechinorhynchus	Catostomus commersoni	7	0.01	0.04	0.04	0.04
1	1	0.91	1	t	1	Ambloplites rupestris	Echinorhynchus salmonis	7	0.05	0.04	0.04	0.04
1	1	0.77	1	t	1	Ambloplites rupestris	Pomphorhynchus bulbocolli	7	0.05	0.04	0.02	0.04
0	1	0.67	1	cv	1	Ambloplites rupestris	Protometra macrostoma	7	0.05	0.04	0.01	0.04
1	1	0.95	1	t	1	Ambloplites rupestris	Diplostomulum sp. (L)	7	0.05	0.04	0.06	0.04
1	1	0.75	1	t	1	Ambloplites rupestris	Proteocephalus ambloplitis	7	0.05	0.04	0.02	0.04
0	1	0.77	1	t	1	Ambloplites rupestris	Contracaecum brachyurum	7	0.05	0.04	0.02	0.04
1	1	0.86	1	cv	1	Echinorhynchus salmonis	Catostomus commersoni	7	0.04	0.04	0.04	0.04
1	1	0.83	1	t	1	Echinorhynchus salmonis	Coregonus artedi	7	0.04	0.04	0.04	0.04
1	1	0.85	1	t	1	Echinorhynchus salmonis	Lepomis gibbosus	7	0.04	0.04	0.04	0.04
1	1	0.68	1	t	1	Pomphorhynchus bulbocolli	Catostomus commersoni	7	0.02	0.04	0.04	0.04
0	1	0.64	1	cv	1	Tetracotyle sp. (L)	Etheostoma nigrum	7	0.02	0.04	0.03	0.04
0	0	0.20	1	t	1	Ictalurus nebulosus	Acetodextra amiuri	7	0.00	0.04	0.00	0.03
1	1	0.71	1	t	1	Ictalurus nebulosus	Diplostomulum sp. (L)	7	0.00	0.04	0.06	0.04

0	0	0.20	1	t	1	Ictalurus nebulosus	Corallobothrium fimbriatum	7	0.00	0.04	0.00	0.03
0	1	0.69	1	cv	1	Echinorhynchus leidyi	Catostomus commersoni	7	0.02	0.04	0.04	0.04
0	1	0.52	1	t	1	Spiroxys sp. (L)	Etheostoma nigrum	7	0.01	0.04	0.03	0.04
1	1	0.55	1	t	1	Protometra macrostoma	Lepomis gibbosus	7	0.01	0.04	0.04	0.04
0	0	0.46	1	t	1	Dichelyne sp.	Etheostoma nigrum	7	0.00	0.04	0.03	0.04
0	1	0.59	1	cv	1	Triaenophorus crassus (L)	Coregonus artedi	7	0.01	0.04	0.04	0.04
1	1	0.79	1	t	1	Diplostomulum sp. (L)	Pimephales notatus	7	0.06	0.04	0.02	0.04
1	1	0.89	1	t	1	Diplostomulum sp. (L)	Etheostoma nigrum	7	0.06	0.04	0.03	0.04
1	1	0.90	1	t	1	Diplostomulum sp. (L)	Coregonus artedi	7	0.06	0.04	0.04	0.04
1	1	0.91	1	cv	1	Diplostomulum sp. (L)	Lepomis gibbosus	7	0.06	0.04	0.04	0.04
1	1	0.64	1	t	1	Proteocephalus ambloplitis	Lepomis gibbosus	7	0.02	0.04	0.04	0.04
1	1	0.58	1	t	1	Neoechinorhynchus crassus	Catostomus commersoni	7	0.01	0.04	0.04	0.04
0	1	0.62	1	t	1	Proteocephalus exiguus	Coregonus artedi	7	0.02	0.04	0.04	0.04
1	1	0.52	1	cv	1	Catostomus commersoni	Octomacrum lanceatum	7	0.04	0.04	0.00	0.04
0	1	0.52	1	t	1	Etheostoma nigrum	Bothriocephalus formosus	7	0.03	0.04	0.01	0.04
1	1	0.51	1	t	1	Coregonus artedi	Argulus sp.	7	0.04	0.04	0.01	0.04
0	1	0.58	0	t	1	Achtheres micropteri	Notropis heterolepis	7	0.04	0.04	0.01	0.04
0	0	0.18	0	cv	1	Crepidostomum cornutum	Octospinifer macilentus	7	0.00	0.04	0.00	0.04
0	0	0.37	0	t	1	Salvelinus namaycush	Notropis cornutus	7	0.01	0.04	0.02	0.04
0	0	0.22	0	t	1	Crepidostomum isostomum	Spinitectus sp.	7	0.01	0.04	0.00	0.04
0	1	0.55	0	t	1	Neoechinorhynchus rutili	Contracaecum brachyurum	7	0.03	0.04	0.02	0.04
0	0	0.32	0	cv	1	Allocreadium lobatum	Salvelinus namaycush	7	0.01	0.04	0.01	0.04
0	0	0.38	0	t	1	Illinobdella alba	Prosopium cylindraceum	7	0.00	0.04	0.03	0.04
0	1	0.62	0	t	1	Triaenophorus stizostedionis	Lepomis gibbosus	7	0.01	0.04	0.04	0.04

0	0	0.19	0	t	1	Rhipidocotyle papillosa	Caecincola parvulus	7	0.00	0.03	0.00	0.04
0	0	0.45	0	cv	1	Leptorhynchoides thecatus	Glaridacris catostomi	7	0.03	0.04	0.01	0.04
0	0	0.27	0	t	1	Chrosomus eos	Spiroxys sp. (L)	7	0.01	0.04	0.01	0.04
0	0	0.40	0	t	1	Azygia angusticauda	Notropis heterolepis	7	0.02	0.04	0.01	0.04
0	0	0.30	0	t	1	Neoechinorhynchus tumidus	Pomphorhynchus bulbocolli	7	0.00	0.04	0.02	0.04
0	0	0.42	0	cv	1	Philometra sp. (L)	Tetracotyle sp. (L)	7	0.01	0.04	0.02	0.04
0	0	0.43	0	t	1	Notropis heterolepis	Ergasilus caeruleus	7	0.01	0.04	0.02	0.04
0	0	0.34	0	t	1	Dichelyne cotylophora	Spinitectus carolini	7	0.02	0.04	0.01	0.04
0	0	0.21	0	t	1	Bothriocephalus cuspidatus	Diplostomum flexicaudum (L)	7	0.00	0.04	0.01	0.04
0	0	0.47	0	cv	1	Lota lota	Bothriocephalus formosus	7	0.03	0.04	0.01	0.04
1	1	0.75	0	t	1	Spinitectus gracilis	Ambloplites rupestris	7	0.01	0.04	0.05	0.04
1	1	0.88	0	t	1	Achtheres corpulentus	Coregonus artedi	7	0.05	0.04	0.04	0.04
0	0	0.20	0	t	1	Centrovarium lobotes	Bucephalopsis pusilla	7	0.00	0.03	0.00	0.04
0	0	0.18	0	cv	1	Lebouria cooperi	Crepidostomum cornutum	7	0.00	0.04	0.00	0.04
0	0	0.26	0	t	1	Illinobdella sp.	Bothriocephalus formosus	7	0.01	0.04	0.01	0.04
0	0	0.48	0	t	1	Ergasilus caeruleus	Contracaecum brachyurum	7	0.02	0.04	0.02	0.04
0	0	0.19	0	t	1	Bothriocephalus sp.	Bothriocephalus claviceps	7	0.01	0.04	0.00	0.04
0	0	0.39	0	cv	1	Proteocephalus pinguis	Coregonus hoyi	7	0.00	0.04	0.03	0.04
0	0	0.20	0	t	1	Illinobdella sp.	Triaenophorus crassus	7	0.01	0.04	0.00	0.04
0	0	0.25	0	t	1	Bothriocephalus cuspidatus	Diphyllobothrium sp. (L)	7	0.00	0.04	0.01	0.04
0	1	0.56	0	t	1	Micropterus salmoides	Proteocephalus exiguus	7	0.03	0.04	0.02	0.04
0	0	0.38	0	cv	1	Neoechinorhynchus rutili	Illinobdella alba	7	0.03	0.04	0.00	0.04
0	1	0.50	0	t	1	Illinobdella sp.	Percopsis omiscomaycus	7	0.01	0.04	0.03	0.04

0	0	0.30	0	t	1	Pomphorhynchus bulbocolli	Metabronema salvelini	7	0.02	0.04	0.01	0.04
0	0	0.28	0	t	1	Lebouria cooperi	Pimephales notatus	7	0.00	0.04	0.02	0.04
0	0	0.20	0	cv	1	Spinitectus sp.	Metabronema salvelini	7	0.00	0.04	0.01	0.04
0	0	0.23	0	t	1	Crepidostomum ictaluri	Metabronema salvelini	7	0.00	0.03	0.01	0.04
0	1	0.78	0	t	1	Rhabdochona cascadilla	Achtheres corpulentus	7	0.02	0.04	0.05	0.04
0	0	0.25	0	t	1	Triaenophorus stizostedionis	Ictalurus nebulosus	7	0.01	0.04	0.00	0.04
0	0	0.31	0	cv	1	Semotilus atromaculatus	Neoechinorhynchus strigosus	7	0.02	0.04	0.01	0.04
0	1	0.65	0	t	1	Rhinichthys cataractae	Achtheres corpulentus	7	0.00	0.04	0.05	0.04
1	0	0.26	0	t	1	Glaridacris catostomi	Metabronema salvelini	7	0.01	0.04	0.01	0.04
1	1	0.52	0	t	1	Achtheres micropteri	Actinobdella triannulata	7	0.04	0.04	0.01	0.04
0	0	0.21	0	cv	1	Illinobdella sp.	Catostomus catostomus	7	0.01	0.04	0.00	0.04
0	0	0.18	0	t	1	Illinobdella alba	Octospinifer macilentus	7	0.00	0.04	0.00	0.04
0	0	0.30	0	t	1	Etheostoma exile	Catostomus catostomus	7	0.02	0.04	0.00	0.04
0	0	0.30	0	t	1	Rhipidocotyle papillosa	Notropis cornutus	7	0.00	0.03	0.02	0.04
1	0	0.25	0	cv	1	Glaridacris catostomi	Acetodextra amiuri	7	0.01	0.04	0.00	0.03
0	0	0.39	0	t	1	Diphyllobothrium sp. (L)	Pomphorhynchus bulbocolli	7	0.01	0.04	0.02	0.04
0	0	0.18	0	t	1	Caecincola parvulus	Salmincola sp.	7	0.00	0.04	0.00	0.04
0	0	0.39	0	t	1	Eubothrium salvelini	Bucephalus sp.	7	0.03	0.03	0.00	0.04
0	0	0.42	0	cv	1	Coregonus clupeaformis	Centrovarium lobotes	7	0.03	0.04	0.00	0.03
1	0	0.18	0	t	1	Philometra cylindracea (L)	Bunoderina eucaliae	7	0.00	0.04	0.00	0.03
0	0	0.41	0	t	1	Eubothrium salvelini	Cryptogonimus chyli	7	0.03	0.03	0.01	0.04
0	1	0.66	0	t	1	Micropterus dolomieui	Achtheres corpulentus	7	0.00	0.04	0.05	0.04
0	0	0.29	0	cv	1	Neoechinorhynchus	Bucephalus sp.	7	0.02	0.04	0.00	0.04
0	0	0.31	0	t	1	Philometra cylindracea (L)	Tetracotyle sp. (L)	7	0.00	0.04	0.02	0.04

0	1	0.54	0	t	1	Coregonus hoyi	Pomphorhynchus bulbocolli	7	0.03	0.04	0.02	0.04
0	0	0.27	0	t	1	Achtheres ambloplitis	Proteocephalus exiguus	7	0.00	0.04	0.02	0.04
0	0	0.25	0	cv	1	Triaenophorus stizostedionis	Proteocephalus laruei	7	0.01	0.04	0.00	0.04
0	0	0.18	0	t	1	Crepidostomum farionis	Philometra cylindracea (L)	7	0.00	0.04	0.00	0.04
0	0	0.24	0	t	1	Proteocephalus pearsei	Proteocephalus laruei	7	0.01	0.04	0.00	0.04
0	1	0.79	0	t	1	Eubothrium salvelini	Posthodiplostomum minimum (L)	7	0.03	0.03	0.05	0.04
0	1	0.53	0	cv	1	Leptorhynchoides thecatus	Neoechinorhynchus cylindratus	7	0.03	0.04	0.02	0.04
0	0	0.24	0	t	1	Proteocephalus pearsei	Ictalurus nebulosus	7	0.01	0.04	0.00	0.04
0	1	0.87	0	t	1	Stizostedion vitreum	Ambloplites rupestris	7	0.03	0.04	0.05	0.04
0	1	0.72	0	t	1	Stizostedion vitreum	Micropterus salmoides	7	0.03	0.04	0.03	0.04
0	0	0.30	0	cv	1	Salmincola sp.	Pomphorhynchus bulbocolli	7	0.00	0.04	0.02	0.04
0	0	0.31	0	t	1	Neoechinorhynchus tumidus	Oncorhynchus mykiss	7	0.00	0.04	0.02	0.04
1	1	0.80	0	t	1	Etheostoma nigrum	Lepomis gibbosus	7	0.03	0.04	0.04	0.04
0	1	0.55	0	t	1	Coregonus clupeaformis	Contracaecum brachyurum	7	0.03	0.04	0.02	0.04
1	1	0.66	0	cv	1	Bucephalus elegans	Ambloplites rupestris	7	0.00	0.04	0.05	0.04
0	0	0.43	0	t	1	Coregonus clupeaformis	Centrovarium lobotes (L)	7	0.03	0.04	0.01	0.04
0	0	0.29	0	t	1	Haplonema hamulatum	Contracaecum brachyurum	7	0.00	0.04	0.02	0.04
0	1	0.50	0	t	1	Neascus sp. (L)	Neoechinorhynchus strigosus	7	0.04	0.04	0.01	0.04
0	0	0.27	0	cv	1	Bothriocephalus cuspidatus	Semotilus atromaculatus	7	0.00	0.04	0.02	0.04
0	0	0.19	0	t	1	Micropterus dolomieui	Bunoderina eucaliae	7	0.00	0.04	0.00	0.03
1	1	0.62	0	t	1	Spinitectus carolini	Catostomus commersoni	7	0.01	0.04	0.04	0.04

0	1	0.80	0	t	1	Perca flavescens	Echinorhynchus leidyi	7	0.06	0.04	0.02	0.04
0	0	0.39	0	cv	1	Neoechinorhynchus	Achtheres coregoni	7	0.02	0.04	0.01	0.04
1	1	0.68	0	t	1	Stizostedion vitreum	Prosopium cylindraceum	7	0.03	0.04	0.03	0.04
0	0	0.33	0	t	1	Triaenophorus stizostedionis	Spinitectus gracilis	7	0.01	0.04	0.01	0.04
0	0	0.34	0	t	1	Triaenophorus crassus	Ergasilus caeruleus	7	0.00	0.04	0.02	0.04
0	1	0.53	0	cv	1	Micropterus salmoides	Triaenophorus crassus (L)	7	0.03	0.04	0.01	0.04
0	0	0.27	0	t	1	Clinostomum marginatum	Notropis hudsonius	7	0.01	0.04	0.01	0.04
1	1	0.55	0	t	1	Illinobdella moorei	Lepomis gibbosus	7	0.01	0.04	0.04	0.04
0	1	0.55	0	t	1	Echinorhynchus salmonis	Bucephalus sp.	7	0.04	0.04	0.00	0.04
0	0	0.19	0	cv	1	Achtheres ambloplitis	Protometra macrostoma	7	0.00	0.04	0.01	0.04
1	1	0.72	0	t	1	Azygia angusticauda	Echinorhynchus salmonis	7	0.02	0.04	0.04	0.04
0	0	0.31	0	t	1	Azygia angusticauda	Crepidostomum farionis	7	0.02	0.04	0.00	0.04
0	1	0.78	0	t	1	Triaenophorus stizostedionis	Perca flavescens	7	0.01	0.04	0.06	0.04
0	0	0.20	0	cv	1	Cyathocephalus americanus	Rhabdochona sp.	7	0.00	0.04	0.01	0.04
1	1	0.75	0	t	1	Perca flavescens	Microphallus opacus	7	0.06	0.04	0.01	0.04
0	0	0.33	0	t	1	Centrovarium lobotes (L)	Oncorhynchus mykiss	7	0.01	0.04	0.02	0.04
0	0	0.22	0	t	1	Rhinichthys cataractae	Salvelinus fontinalis	7	0.00	0.04	0.01	0.04
0	0	0.39	0	cv	1	Esox lucius	Salmincola sp.	7	0.03	0.04	0.00	0.04
0	0	0.30	0	t	1	Neoechinorhynchus sp.	Echinorhynchus leidyi	7	0.00	0.04	0.02	0.04
0	0	0.30	0	t	1	Pimephales notatus	Neoechinorhynchus crassus	7	0.02	0.04	0.01	0.04
0	1	0.51	0	t	1	Eubothrium salvelini	Pomphorhynchus bulbocolli (L)	7	0.03	0.03	0.02	0.04
0	0	0.38	0	cv	1	Coregonus hoyi	Ictalurus nebulosus	7	0.03	0.04	0.00	0.04
0	0	0.39	0	t	1	Notropis heterolepis	Osmerus mordax	7	0.01	0.04	0.02	0.04
0	0	0.23	0	t	1	Allocreadium lobatum	Cyathocephalus americanus	7	0.01	0.04	0.00	0.04

0	0	0.19	0	t	1	Triganodistomum simeri	Bunodera sacculata	7	0.00	0.03	0.00	0.04
1	1	0.63	0	cv	1	Hepaticola bakeri	Lepomis gibbosus	7	0.01	0.04	0.04	0.04
0	0	0.21	0	t	1	Micropterus dolomieui	Diplostomum flexicaudum (L)	7	0.00	0.04	0.01	0.04
0	0	0.17	0	t	1	Crepidostomum cornutum	Philometra cylindracea (L)	7	0.00	0.04	0.00	0.04
0	0	0.20	0	t	1	Rhipidocotyle papillosa	Ictalurus nebulosus	7	0.00	0.03	0.00	0.04
0	0	0.34	0	cv	1	Illinobdella moorei	Tetracotyle sp. (L)	7	0.01	0.04	0.02	0.04
0	0	0.18	0	t	1	Lebouria cooperi	Culaea inconstans	7	0.00	0.04	0.00	0.04
0	0	0.29	0	t	1	Protometra macrostoma	Proteocephalus ambloplitis	7	0.01	0.04	0.02	0.04
0	0	0.30	0	t	1	Bucephalopsis pusilla	Oncorhynchus mykiss	7	0.00	0.04	0.02	0.04
0	0	0.31	0	cv	1	Osmerus mordax	Corallobothrium fimbriatum	7	0.02	0.04	0.00	0.03
0	1	0.65	0	t	1	Echinorhynchus salmonis (L)	Posthodiplostomum minimum (L)	7	0.01	0.04	0.05	0.04
0	0	0.36	0	t	1	Rhabdochona cascadilla	Chrosomus eos	7	0.02	0.04	0.01	0.04
0	0	0.19	0	t	1	Neoechinorhynchus sp.	Corallobothrium fimbriatum	7	0.00	0.04	0.00	0.03
0	0	0.30	0	cv	1	Oncorhynchus mykiss	Bunoderina eucaliae	7	0.02	0.04	0.00	0.03
0	1	0.50	0	t	1	Neascus sp. (L)	Actinobdella triannulata	7	0.04	0.04	0.01	0.04
0	1	0.56	0	t	1	Triaenophorus stizostedionis	Etheostoma nigrum	7	0.01	0.04	0.03	0.04
0	1	0.61	0	t	1	Achtheres micropteri	Pomphorhynchus bulbocolli (L)	7	0.04	0.04	0.02	0.04
0	0	0.21	0	cv	1	Cyathocephalus americanus	Metabronema salvelini	7	0.00	0.04	0.01	0.04
0	0	0.25	0	t	1	Spinitectus gracilis (L)	Bucephalopsis pusilla	7	0.01	0.04	0.00	0.04
0	0	0.22	0	t	1	Glaridacris catostomi	Philometra cylindracea (L)	7	0.01	0.04	0.00	0.04
0	0	0.25	0	t	1	Illinobdella moorei	Glaridacris catostomi	7	0.01	0.04	0.01	0.04

1	0	0.45	0	cv	1	Philometra cylindracea (L)	Etheostoma nigrum	7	0.00	0.04	0.03	0.04
1	1	0.88	0	t	1	Percopsis omiscomaycus	Ambloplites rupestris	7	0.03	0.04	0.05	0.04
0	0	0.44	0	t	1	Neoechinorhynchus	Oncorhynchus mykiss	7	0.02	0.04	0.02	0.04
1	1	0.59	0	t	1	Spiroxys sp. (L)	Lepomis gibbosus	7	0.01	0.04	0.04	0.04
0	0	0.40	0	cv	1	Rhabdochona cascadilla	Triaenophorus crassus (L)	7	0.02	0.04	0.01	0.04
0	0	0.39	0	t	1	Lebouria cooperi	Eubothrium salvelini	7	0.00	0.04	0.03	0.03
0	0	0.21	0	t	1	Rhabdochona sp.	Acetodextra amiuri	7	0.01	0.04	0.00	0.03
0	0	0.20	0	t	1	Neoechinorhynchus sp.	Protometra macrostoma	7	0.00	0.04	0.01	0.04
0	0	0.29	0	cv	1	Allocreadium lobatum	Abothrium crassum	7	0.01	0.04	0.01	0.04
0	0	0.28	0	t	1	Rhipidocotyle papillosa	Cystidicola stigmatura	7	0.00	0.03	0.01	0.04
0	1	0.56	0	t	1	Azygia longa	Echinorhynchus salmonis	7	0.00	0.04	0.04	0.04
0	0	0.41	0	t	1	Stizostedion vitreum	Achtheres ambloplitis	7	0.03	0.04	0.00	0.04
0	0	0.32	0	cv	1	Centrovarium lobotes	Osmerus mordax	7	0.00	0.03	0.02	0.04
1	1	0.75	0	t	1	Neascus sp. (L)	Micropterus salmoides	7	0.04	0.04	0.03	0.04
0	0	0.24	0	t	1	Proteocephalus stizostethi	Clinostomum marginatum (L)	7	0.00	0.04	0.01	0.04
0	0	0.25	0	t	1	Culaea inconstans	Salvelinus namaycush	7	0.00	0.04	0.01	0.04
0	0	0.18	0	cv	1	Azygia longa	Rhinichthys cataractae	7	0.00	0.04	0.00	0.04
0	0	0.36	0	t	1	Etheostoma exile	Dichelyne robusta	7	0.02	0.04	0.01	0.03
0	1	0.57	0	t	1	Echinorhynchus salmonis	Protometra macrostoma	7	0.04	0.04	0.01	0.04
0	0	0.22	0	t	1	Notropis volucellus	Bothriocephalus claviceps	7	0.01	0.04	0.00	0.04
0	1	0.52	0	cv	1	Stizostedion vitreum	Clinostomum marginatum (L)	7	0.03	0.04	0.01	0.04
0	0	0.22	0	t	1	Allocreadium lobatum	Illinobdella alba	7	0.01	0.04	0.00	0.04
0	0	0.27	0	t	1	Proteocephalus pinguis	Proteocephalus ambloplitis	7	0.00	0.04	0.02	0.04
0	0	0.19	0	t	1	Proteocephalus laruei	Bunoderina eucaliae	7	0.00	0.04	0.00	0.03

0	0	0.26	0	cv	1	Crepidostomum ictaluri	Discocotyle salmonis	7	0.00	0.03	0.01	0.04
0	0	0.39	0	t	1	Cottus bairdi	Bunoderina eucaliae	7	0.03	0.04	0.00	0.03
0	0	0.39	0	t	1	Etheostoma exile	Cystidicola stigmatura	7	0.02	0.04	0.01	0.04
0	0	0.25	0	t	1	Bothriocephalus claviceps	Triaenophorus crassus (L)	7	0.00	0.04	0.01	0.04
0	0	0.45	0	cv	1	Prosopium cylindraceum	Chrosomus eos	7	0.03	0.04	0.01	0.04
0	0	0.46	0	t	1	Micropterus dolomieui	Etheostoma nigrum	7	0.00	0.04	0.03	0.04
1	1	0.72	0	t	1	Neoechinorhynchus rutili	Percopsis omiscomaycus	7	0.03	0.04	0.03	0.04
0	0	0.18	0	t	1	Micropterus dolomieui	Bunodera sacculata	7	0.00	0.04	0.00	0.04
0	0	0.48	0	cv	1	Rhinichthys cataractae	Neascus sp. (L)	7	0.00	0.04	0.04	0.04
0	0	0.29	0	t	1	Semotilus atromaculatus	Protometra macrostoma	7	0.02	0.04	0.01	0.04
1	0	0.22	0	t	1	Diplostomum flexicaudum (L)	Corallobothrium fimbriatum	7	0.01	0.04	0.00	0.03
0	0	0.21	0	t	1	Salmincola inermis	Centrovarium lobotes (L)	7	0.00	0.04	0.01	0.04
0	0	0.17	0	cv	1	Crepidostomum cooperi	Bucephalopsis pusilla	7	0.00	0.04	0.00	0.04
0	0	0.32	0	t	1	Camallanus oxycephalus	Pomphorhynchus bulbocolli	7	0.01	0.04	0.02	0.04
0	1	0.64	0	t	1	Ambloplites rupestris	Ictalurus nebulosus	7	0.05	0.04	0.00	0.04
0	0	0.42	0	t	1	Bothriocephalus sp.	Prosopium cylindraceum	7	0.01	0.04	0.03	0.04
0	0	0.19	0	cv	1	Illinobdella alba	Cryptogonimus chyli	7	0.00	0.04	0.01	0.04
0	0	0.20	0	t	1	Culaea inconstans	Camallanus oxycephalus	7	0.00	0.04	0.01	0.04
0	0	0.25	0	t	1	Lebouria cooperi	Clinostomum marginatum (L)	7	0.00	0.04	0.01	0.04
0	0	0.32	0	t	1	Rhabdochona cascadilla	Corallobothrium fimbriatum	7	0.02	0.04	0.00	0.03
0	0	0.25	0	cv	1	Rhipidocotyle papillosa	Abothrium crassum	7	0.00	0.03	0.01	0.04
0	0	0.46	0	t	1	Bucephalopsis pusilla	Etheostoma nigrum	7	0.00	0.04	0.03	0.04
0	0	0.23	0	t	1	Crepidostomum farionis	Spiroxys sp. (L)	7	0.00	0.04	0.01	0.04

0	0	0.34	0	t	1	Echinorhynchus salmonis (L)	Notropis cornutus	7	0.01	0.04	0.02	0.04
0	0	0.21	0	cv	1	Proteocephalus stizostethi	Actinobdella triannulata	7	0.00	0.04	0.01	0.04
1	1	0.52	0	t	1	Hepaticola bakeri	Lota lota	7	0.01	0.04	0.03	0.04
0	0	0.22	0	t	1	Bothriocephalus cuspidatus	Chrosomus eos	7	0.00	0.04	0.01	0.04
0	0	0.26	0	t	1	Pomphorhynchus bulbocolli	Bunodera sacculata	7	0.02	0.04	0.00	0.04
0	0	0.45	0	cv	1	Esox lucius	Bothriocephalus formosus	7	0.03	0.04	0.01	0.04
0	0	0.22	0	t	1	Bucephalus sp.	Neoechinorhynchus crassus	7	0.00	0.04	0.01	0.04
0	0	0.20	0	t	1	Triganodistomum simeri	Neoechinorhynchus sp.	7	0.00	0.03	0.00	0.04
0	1	0.56	0	t	1	Proteocephalus stizostethi	Echinorhynchus salmonis	7	0.00	0.04	0.04	0.04
0	0	0.40	0	cv	1	Leptorhynchoides thecatus	Camallanus oxycephalus	7	0.03	0.04	0.01	0.04
0	0	0.39	0	t	1	Coregonus clupeaformis	Micropterus dolomieui	7	0.03	0.04	0.00	0.04
0	0	0.32	0	t	1	Azygia angusticauda	Salmincola inermis	7	0.02	0.04	0.00	0.04
0	0	0.19	0	t	1	Bothriocephalus cuspidatus	Bucephalus sp.	7	0.00	0.04	0.00	0.04
0	0	0.27	0	cv	1	Rhipidocotyle papillosa	Microphallus opacus	7	0.00	0.03	0.01	0.04
0	0	0.37	0	t	1	Leptorhynchoides thecatus	Notropis atherinoides	7	0.03	0.04	0.00	0.04
1	1	0.55	0	t	1	Prosopium cylindraceum	Echinorhynchus leidyi	7	0.03	0.04	0.02	0.04
0	0	0.19	0	t	1	Lebouria cooperi	Ictalurus nebulosus	7	0.00	0.04	0.00	0.04
0	0	0.18	0	cv	1	Bucephalus elegans	Salmincola sp.	7	0.00	0.04	0.00	0.04
0	0	0.41	0	t	1	Oncorhynchus mykiss	Pimephales notatus	7	0.02	0.04	0.02	0.04
0	0	0.48	0	t	1	Neascus sp. (L)	Crepidostomum ictaluri	7	0.04	0.04	0.00	0.03
0	1	0.56	0	t	1	Dichelyne robusta	Neascus sp. (L)	7	0.01	0.03	0.04	0.04
0	0	0.21	0	cv	1	Triganodistomum	Actinobdella triannulata	7	0.00	0.04	0.01	0.04
0	0	0.21	0	t	1	Octospinifer macilentus	Neoechinorhynchus crassus	7	0.00	0.04	0.01	0.04
0	1	0.56	0	t	1	Azygia angusticauda	Cottus bairdi	7	0.02	0.04	0.03	0.04

0	1	0.55	0	t	1	Achtheres micropteri	Notropis volucellus	7	0.04	0.04	0.01	0.04
1	0	0.27	0	cv	1	Dichelyne robusta	Ligula intestinalis (L)	7	0.01	0.03	0.01	0.04
0	0	0.24	0	t	1	Illinobdella sp.	Actinobdella triannulata	7	0.01	0.04	0.01	0.04
1	1	0.56	0	t	1	Camallanus oxycephalus	Catostomus commersoni	7	0.01	0.04	0.04	0.04
0	0	0.22	0	t	1	Crepidostomum isostomum	Bucephalus elegans	7	0.01	0.04	0.00	0.04
0	0	0.49	0	cv	1	Coregonus clupeaformis	Clinostomum marginatum (L)	7	0.03	0.04	0.01	0.04
0	0	0.39	0	t	1	Bothriocephalus cuspidatus	Prosopium cylindraceum	7	0.00	0.04	0.03	0.04
0	0	0.26	0	t	1	Neoechinorhynchus tumidus	Hepaticola bakeri	7	0.00	0.04	0.01	0.04
0	0	0.24	0	t	1	Proteocephalus stizostethi	Microphallus opacus	7	0.00	0.04	0.01	0.04
0	1	0.71	0	cv	1	Spinitectus sp.	Diplostomulum sp. (L)	7	0.00	0.04	0.06	0.04
0	0	0.28	0	t	1	Culaea inconstans	Notropis cornutus	7	0.00	0.04	0.02	0.04
0	0	0.41	0	t	1	Neoechinorhynchus	Proteocephalus exiguus	7	0.02	0.04	0.02	0.04
1	1	0.59	0	t	1	Triganodistomum attenuatum	Posthodiplostomum minimum (L)	7	0.00	0.04	0.05	0.04
1	0	0.29	0	cv	1	Bucephalus elegans	Rhabdochona cascadilla	7	0.00	0.04	0.02	0.04
0	0	0.33	0	t	1	Metabronema salvelini	Contracaecum brachyurum	7	0.01	0.04	0.02	0.04
0	1	0.67	0	t	1	Etheostoma exile	Lepomis gibbosus	7	0.02	0.04	0.04	0.04
0	0	0.24	0	t	1	Crepidostomum farionis	Bothriocephalus formosus	7	0.00	0.04	0.01	0.04
0	0	0.29	0	cv	1	Neoechinorhynchus	Octospinifer macilentus	7	0.02	0.04	0.00	0.04
0	0	0.22	0	t	1	Centrovarium lobotes (L)	Illinobdella moorei	7	0.01	0.04	0.01	0.04
0	0	0.22	0	t	1	Bothriocephalus sp.	Cryptogonimus chyli	7	0.01	0.04	0.01	0.04
1	1	0.76	0	t	1	Achtheres corpulentus	Pomphorhynchus bulbocolli	7	0.05	0.04	0.02	0.04
0	0	0.20	0	cv	1	Actinobdella triannulata	Bothriocephalus claviceps	7	0.01	0.04	0.00	0.04

0	0	0.28	0	t	1	Lebouria cooperi	Semotilus atromaculatus	7	0.00	0.04	0.02	0.04
0	0	0.31	0	t	1	Allocreadium lobatum	Microphallus opacus	7	0.01	0.04	0.01	0.04
0	0	0.28	0	t	1	Illinobdella sp.	Notropis heterolepis	7	0.01	0.04	0.01	0.04
1	0	0.35	0	cv	1	Hepaticola bakeri	Diphyllobothrium sp. (L)	7	0.01	0.04	0.01	0.04
1	1	0.78	0	t	1	Diplostomulum sp. (L)	Proteocephalus ambloplitis	7	0.06	0.04	0.02	0.04
0	0	0.29	0	t	1	Philometra sp. (L)	Neoechinorhynchus crassus	7	0.01	0.04	0.01	0.04
0	0	0.29	0	t	1	Salmincola wisconsinsensi	Diphyllobothrium sp. (L)	7	0.01	0.04	0.01	0.04
0	0	0.28	0	cv	1	Proteocephalus laruei	Dichelyne cotylophora	7	0.00	0.04	0.02	0.04
0	1	0.51	0	t	1	Coregonus hoyi	Pimephales notatus	7	0.03	0.04	0.02	0.04
0	0	0.27	0	t	1	Salvelinus namaycush	Triganodistomum simeri	7	0.01	0.04	0.00	0.03
0	1	0.82	0	t	1	Rhabdochona cascadilla	Diplostomulum sp. (L)	7	0.02	0.04	0.06	0.04
0	0	0.25	0	cv	1	Neoechinorhynchus tumidus	Spinitectus carolini	7	0.00	0.04	0.01	0.04
1	1	0.53	0	t	1	Neoechinorhynchus	Coregonus artedi	7	0.01	0.04	0.04	0.04
0	1	0.74	0	t	1	Philometra sp. (L)	Achtheres corpulentus	7	0.01	0.04	0.05	0.04
0	0	0.17	0	t	1	Bothriocephalus cuspidatus	Philometra cylindracea (L)	7	0.00	0.04	0.00	0.04
0	1	0.59	0	cv	1	Esox lucius	Ergasilus caeruleus	7	0.03	0.04	0.02	0.04
0	0	0.42	0	t	1	Cottus bairdi	Argulus sp.	7	0.03	0.04	0.01	0.04
0	0	0.25	0	t	1	Salvelinus fontinalis	Argulus sp.	7	0.01	0.04	0.01	0.04
0	0	0.20	0	t	1	Octospinifer macilentus	Rhabdochona sp.	7	0.00	0.04	0.01	0.04
0	0	0.20	0	cv	1	Bothriocephalus sp.	Lebouria cooperi	7	0.01	0.04	0.00	0.04
1	1	0.70	0	t	1	Posthodiplostomum minimum (L)	Pomphorhynchus bulbocolli	7	0.05	0.04	0.02	0.04
0	0	0.33	0	t	1	Cystidicola stigmatura	Proteocephalus pearsei	7	0.01	0.04	0.01	0.04
0	0	0.21	0	t	1	Cyathocephalus americanus	Argulus sp.	7	0.00	0.04	0.01	0.04

0	0	0.19	0	cv	1	Micropterus dolomieui	Proteocephalus laruei	7	0.00	0.04	0.00	0.04
0	1	0.67	0	t	1	Bucephalopsis pusilla	Ambloplites rupestris	7	0.00	0.04	0.05	0.04
0	0	0.44	0	t	1	Neoechinorhynchus rutili	Diplostomum flexicaudum (L)	7	0.03	0.04	0.01	0.04
0	0	0.29	0	t	1	Pomphorhynchus bulbocolli	Rhabdochona sp.	7	0.02	0.04	0.01	0.04
0	0	0.47	0	cv	1	Leptorhynchoides thecatus	Clinostomum marginatum (L)	7	0.03	0.04	0.01	0.04
0	0	0.19	0	t	1	Rhipidocotyle papillosa	Bothriocephalus claviceps	7	0.00	0.03	0.00	0.04
0	1	0.57	0	t	1	Cystidicola stigmatura	Etheostoma nigrum	7	0.01	0.04	0.03	0.04
0	0	0.27	0	t	1	Centrovarium lobotes (L)	Clinostomum marginatum (L)	7	0.01	0.04	0.01	0.04
0	1	0.57	0	cv	1	Notropis hudsonius	Catostomus commersoni	7	0.01	0.04	0.04	0.04
0	1	0.65	0	t	1	Bothriocephalus cuspidatus	Achtheres corpulentus	7	0.00	0.04	0.05	0.04
0	0	0.29	0	t	1	Crepidostomum cornutum	Echinorhynchus leidyi	7	0.00	0.04	0.02	0.04
0	0	0.37	0	t	1	Prosopium cylindraceum	Bothriocephalus claviceps	7	0.03	0.04	0.00	0.04
0	0	0.48	0	cv	1	Azygia longa	Coregonus artedi	7	0.00	0.04	0.04	0.04
0	1	0.58	0	t	1	Triaenophorus stizostedionis	Neascus sp. (L)	7	0.01	0.04	0.04	0.04
0	0	0.22	0	t	1	Notropis hudsonius	Corallobothrium fimbriatum	7	0.01	0.04	0.00	0.03
0	0	0.17	0	t	1	Caecincola parvulus	Bunodera sacculata	7	0.00	0.04	0.00	0.04
0	0	0.17	0	cv	1	Culaea inconstans	Illinobdella alba	7	0.00	0.04	0.00	0.04
0	0	0.21	0	t	1	Illinobdella sp.	Triganodistomum attenuatum	7	0.01	0.04	0.00	0.04
0	1	0.51	0	t	1	Salmincola wisconsinsensis	Percopsis omiscomaycus	7	0.01	0.04	0.03	0.04
0	0	0.25	0	t	1	Salvelinus namaycush	Ictalurus nebulosus	7	0.01	0.04	0.00	0.04
0	0	0.21	0	cv	1	Triaenophorus crassus	Neoechinorhynchus crassus	7	0.00	0.04	0.01	0.04
0	0	0.21	0	t	1	Ligula intestinalis (L)	Crepidostomum farionis	7	0.01	0.04	0.00	0.04

0	0	0.43	0	t	1	Centrovarium lobotes (L)	Coregonus hoyi	7	0.01	0.04	0.03	0.04
0	0	0.30	0	t	1	Triaenophorus stizostedionis	Salvelinus fontinalis	7	0.01	0.04	0.01	0.04
0	0	0.29	0	cv	1	Neoechinorhynchus	Crepidostomum farionis	7	0.02	0.04	0.00	0.04
0	1	0.72	0	t	1	Salmincola inermis	Diplostomulum sp. (L)	7	0.00	0.04	0.06	0.04
0	0	0.42	0	t	1	Etheostoma exile	Pimephales notatus	7	0.02	0.04	0.02	0.04
0	0	0.20	0	t	1	Lebouria cooperi	Centrovarium lobotes	7	0.00	0.04	0.00	0.03
0	0	0.32	0	cv	1	Salmincola wisconsinsensi	Proteocephalus exiguus	7	0.01	0.04	0.02	0.04
0	0	0.22	0	t	1	Centrovarium lobotes	Notropis hudsonius	7	0.00	0.03	0.01	0.04
0	0	0.22	0	t	1	Rhipidocotyle papillosa	Triganodistomum simeri	7	0.00	0.03	0.00	0.03
0	0	0.30	0	t	1	Protometra macrostoma	Pimephales notatus	7	0.01	0.04	0.02	0.04
0	0	0.22	0	cv	1	Camallanus oxycephalus	Illinobdella moorei	7	0.01	0.04	0.01	0.04
0	1	0.56	0	t	1	Achtheres micropteri	Discocotyle salmonis	7	0.04	0.04	0.01	0.04
0	0	0.27	0	t	1	Bucephalus elegans	Semotilus atromaculatus	7	0.00	0.04	0.02	0.04
0	0	0.18	0	t	1	Rhinichthys cataractae	Proteocephalus pinguis	7	0.00	0.04	0.00	0.04
0	0	0.34	0	cv	1	Diplostomum flexicaudum	Osmerus mordax	7	0.01	0.04	0.02	0.04
0	1	0.68	0	t	1	Rhabdochona cascadilla	Lepomis gibbosus	7	0.02	0.04	0.04	0.04
0	0	0.27	0	t	1	Triganodistomum simeri	Microphallus opacus	7	0.00	0.03	0.01	0.04
0	0	0.18	0	t	1	Caecincola parvulus	Bucephalopsis pusilla	7	0.00	0.04	0.00	0.04
1	0	0.46	0	cv	1	Cryptogonimus chyli	Micropterus salmoides	7	0.01	0.04	0.03	0.04
0	0	0.22	0	t	1	Salvelinus fontinalis	Dichelyne sp.	7	0.01	0.04	0.00	0.04
0	0	0.18	0	t	1	Bucephalus elegans	Salmincola inermis	7	0.00	0.04	0.00	0.04
0	0	0.24	0	t	1	Proteocephalus stizostethi	Spinitectus carolini	7	0.00	0.04	0.01	0.04
0	1	0.56	0	cv	1	Posthodiplostomum minimum (L)	Ictalurus nebulosus	7	0.05	0.04	0.00	0.04
0	0	0.31	0	t	1	Discocotyle salmonis	Spinitectus carolini	7	0.01	0.04	0.01	0.04

0	0	0.40	0	t	1	Philometra sp. (L)	Echinorhynchus leidyi	7	0.01	0.04	0.02	0.04
0	0	0.17	0	t	1	Illinobdella alba	Neoechinorhynchus sp.	7	0.00	0.04	0.00	0.04
0	0	0.22	0	cv	1	Abothrium crassum	Crepidostomum cornutum	7	0.01	0.04	0.00	0.04
0	0	0.35	0	t	1	Etheostoma exile	Spiroxys sp. (L)	7	0.02	0.04	0.01	0.04
0	0	0.40	0	t	1	Prosopium cylindraceum	Corallobothrium fimbriatum	7	0.03	0.04	0.00	0.03
0	0	0.22	0	t	1	Notropis volucellus	Notropis atherinoides	7	0.01	0.04	0.00	0.04
0	0	0.20	0	cv	1	Salmincola sp.	Acetodextra amiuri	7	0.00	0.04	0.00	0.03
0	1	0.78	0	t	1	Spinitectus gracilis (L)	Perca flavescens	7	0.01	0.04	0.06	0.04
0	0	0.25	0	t	1	Allocreadium lobatum	Illinobdella moorei	7	0.01	0.04	0.01	0.04
0	0	0.17	0	t	1	Bucephalus elegans	Haplonema hamulatum	7	0.00	0.04	0.00	0.04
0	0	0.36	0	cv	1	Crepidostomum isostomum	Rhabdochona cascadilla	7	0.01	0.04	0.02	0.04
0	0	0.29	0	t	1	Osmerus mordax	Ictalurus nebulosus	7	0.02	0.04	0.00	0.04
0	0	0.17	0	t	1	Culaea inconstans	Achtheres ambloplitis	7	0.00	0.04	0.00	0.04
0	0	0.49	0	t	1	Neoechinorhynchus tumidus	Neascus sp. (L)	7	0.00	0.04	0.04	0.04
0	0	0.24	0	cv	1	Rhabdochona sp.	Chrosomus eos	7	0.01	0.04	0.01	0.04
0	1	0.53	0	t	1	Neoechinorhynchus rutili	Pimephales notatus	7	0.03	0.04	0.02	0.04
0	0	0.23	0	t	1	Triganodistomum simeri	Notropis hudsonius	7	0.00	0.03	0.01	0.04
0	0	0.30	0	t	1	Centrovarium lobotes	Semotilus atromaculatus	7	0.00	0.03	0.02	0.04
0	0	0.34	0	cv	1	Notropis volucellus	Proteocephalus exiguus	7	0.01	0.04	0.02	0.04
1	0	0.19	0	t	1	Octospinifer macilentus	Bucephalus sp.	7	0.00	0.04	0.00	0.04
0	0	0.21	0	t	1	Salmincola inermis	Rhabdochona sp.	7	0.00	0.04	0.01	0.04
0	0	0.19	0	t	1	Crepidostomum ictaluri	Philometra cylindracea (L)	7	0.00	0.03	0.00	0.04
0	0	0.45	0	cv	1	Bunodera sacculata	Percopsis omiscomaycus	7	0.00	0.04	0.03	0.04

0	0	0.30	0	t	1	Crepidostomum isostomum	Clinostomum marginatum (L)	7	0.01	0.04	0.01	0.04
0	0	0.29	0	t	1	Crepidostomum cooperi	Echinorhynchus leidyi	7	0.00	0.04	0.02	0.04
0	0	0.23	0	t	1	Echinorhynchus salmonis (L)	Bothriocephalus cuspidatus	7	0.01	0.04	0.00	0.04
0	1	0.69	0	cv	1	Perca flavescens	Crepidostomum ictaluri	7	0.06	0.04	0.00	0.03
0	0	0.20	0	t	1	Illinobdella sp.	Illinobdella alba	7	0.01	0.04	0.00	0.04
0	0	0.30	0	t	1	Proteocephalus pearsei	Salvelinus fontinalis	7	0.01	0.04	0.01	0.04
0	0	0.42	0	t	1	Cottus bairdi	Diplostomum flexicaudum (L)	7	0.03	0.04	0.01	0.04
0	0	0.33	0	cv	1	Philometra sp. (L)	Spinitectus carolini	7	0.01	0.04	0.01	0.04
0	0	0.17	0	t	1	Rhinichthys cataractae	Bothriocephalus claviceps	7	0.00	0.04	0.00	0.04
0	0	0.18	0	t	1	Bunodera sacculata	Octomacrum lanceatum	7	0.00	0.04	0.00	0.04
1	1	0.89	1	t	1	Phyllodistomum lachancei	Salmo salar	8	0.04	0.10	0.08	0.08
1	1	0.97	1	cv	1	Phyllodistomum lachancei	Salvelinus namaycush	8	0.04	0.10	0.11	0.08
1	1	0.97	1	t	1	Phyllodistomum lachancei	Salvelinus fontinalis	8	0.04	0.10	0.11	0.08
0	1	0.88	1	t	1	Salmo salar	Salmincola extumescens	8	0.08	0.08	0.04	0.09
1	1	0.95	1	t	1	Salmo salar	Eubothrium salvelini	8	0.08	0.08	0.06	0.09
1	1	0.92	1	cv	1	Salmo salar	Proteocephalus sp.2	8	0.08	0.08	0.05	0.10
1	1	0.92	1	t	1	Salmo salar	Diphyllobothrium sp. (L)	8	0.08	0.08	0.05	0.10
0	1	0.88	1	t	1	Salmo salar	Discocotyle sagittata	8	0.08	0.08	0.04	0.09
0	1	0.88	1	t	1	Salmo salar	Crepidostomum cooperi	8	0.08	0.08	0.04	0.09
1	1	0.99	1	cv	1	Salvelinus namaycush	Eubothrium salvelini	8	0.11	0.08	0.06	0.09
1	1	0.95	1	t	1	Salvelinus namaycush	Triaenophorus crassus (L)	8	0.11	0.08	0.03	0.10
1	1	0.97	1	t	1	Salvelinus namaycush	Proteocephalus sp.1	8	0.11	0.08	0.04	0.10
1	1	0.98	1	t	1	Salvelinus namaycush	Proteocephalus sp.2	8	0.11	0.08	0.05	0.10
1	1	0.98	1	cv	1	Salvelinus namaycush	Diphyllobothrium sp. (L)	8	0.11	0.08	0.05	0.10

1	1	0.97	1	t	1	Salvelinus namaycush	Raphidascaris sp.	8	0.11	0.08	0.04	0.10
1	1	0.95	1	t	1	Salvelinus namaycush	Tetraonchus monenteron	8	0.11	0.08	0.03	0.09
1	1	0.97	1	t	1	Salvelinus namaycush	Capillaria salvelini	8	0.11	0.08	0.04	0.10
1	1	0.97	1	cv	1	Salvelinus namaycush	Crepidostomum farionis	8	0.11	0.08	0.04	0.10
0	1	0.91	1	t	1	Salvelinus namaycush	Salmincola siscowet	8	0.11	0.08	0.02	0.10
1	1	0.97	1	t	1	Salvelinus namaycush	Proteocephalus tumidocollus	8	0.11	0.08	0.04	0.10
1	1	0.97	1	t	1	Salvelinus namaycush	Crepidostomum cooperi	8	0.11	0.08	0.04	0.09
1	1	0.97	1	cv	1	Salmincola extumescens	Coregonus clupeaformis	8	0.04	0.09	0.11	0.08
1	1	0.99	1	t	1	Eubothrium salvelini	Salvelinus fontinalis	8	0.06	0.09	0.11	0.08
0	1	0.51	1	t	1	Esox lucius	Raphidascaris canadensis	8	0.02	0.05	0.03	0.04
0	0	0.35	1	t	1	Esox lucius	Metechinorhynchus lateralis	8	0.02	0.05	0.03	0.09
0	0	0.35	1	cv	1	Esox lucius	Tetraonchus monenteron	8	0.02	0.05	0.03	0.09
0	0	0.35	1	t	1	Esox lucius	Proteocephalus pinguis	8	0.02	0.05	0.02	0.05
0	0	0.35	1	t	1	Esox lucius	Triaenophorus crassus	8	0.02	0.05	0.02	0.05
1	1	0.95	1	t	1	Coregonus clupeaformis	Triaenophorus crassus (L)	8	0.11	0.08	0.03	0.10
1	1	0.97	1	cv	1	Coregonus clupeaformis	Proteocephalus sp.1	8	0.11	0.08	0.04	0.10
1	1	0.98	1	t	1	Coregonus clupeaformis	Proteocephalus sp.2	8	0.11	0.08	0.05	0.10
1	1	0.98	1	t	1	Coregonus clupeaformis	Diphyllobothrium sp. (L)	8	0.11	0.08	0.05	0.10
1	1	0.97	1	t	1	Coregonus clupeaformis	Raphidascaris sp.	8	0.11	0.08	0.04	0.10
0	1	0.91	1	cv	1	Coregonus clupeaformis	Phyllodistomum coregoni	8	0.11	0.08	0.02	0.10
1	1	0.97	1	t	1	Coregonus clupeaformis	Capillaria salvelini	8	0.11	0.08	0.04	0.10
1	1	0.97	1	t	1	Coregonus clupeaformis	Discocotyle sagittata	8	0.11	0.08	0.04	0.09
1	1	0.97	1	t	1	Coregonus clupeaformis	Crepidostomum farionis	8	0.11	0.08	0.04	0.10
0	1	0.91	1	cv	1	Coregonus clupeaformis	Salmincola coregonorum	8	0.11	0.08	0.02	0.10

1	1	0.97	1	t	1	Coregonus clupeaformis	Proteocephalus tumidocollus	8	0.11	0.08	0.04	0.10
1	1	0.97	1	t	1	Coregonus clupeaformis	Crepidostomum cooperi	8	0.11	0.08	0.04	0.09
1	1	0.97	1	t	1	Proteocephalus sp.1	Salvelinus fontinalis	8	0.04	0.10	0.11	0.08
1	1	0.98	1	cv	1	Proteocephalus sp.2	Salvelinus fontinalis	8	0.05	0.10	0.11	0.08
1	1	0.92	1	t	1	Metabronema salvelini	Salvelinus fontinalis	8	0.02	0.10	0.11	0.08
1	1	0.92	1	t	1	Salmincola edwardsii	Salvelinus fontinalis	8	0.02	0.10	0.11	0.08
1	1	0.67	1	t	1	Ergasilus caeruleus	Catostomus catostomus	8	0.02	0.03	0.03	0.03
1	1	0.96	1	cv	1	Metechinorhynchus lateralis	Salvelinus fontinalis	8	0.03	0.09	0.11	0.08
1	1	0.67	1	t	1	Neoechinorhynchus crassus	Catostomus catostomus	8	0.02	0.03	0.03	0.03
1	1	0.98	1	t	1	Salvelinus fontinalis	Diphyllobothrium sp. (L)	8	0.11	0.08	0.05	0.10
1	1	0.97	1	t	1	Salvelinus fontinalis	Raphidascaris sp.	8	0.11	0.08	0.04	0.10
1	1	0.97	1	cv	1	Salvelinus fontinalis	Capillaria salvelini	8	0.11	0.08	0.04	0.10
1	1	0.97	1	t	1	Salvelinus fontinalis	Discocotyle sagittata	8	0.11	0.08	0.04	0.09
1	1	0.97	1	t	1	Salvelinus fontinalis	Crepidostomum farionis	8	0.11	0.08	0.04	0.10
1	1	0.97	1	t	1	Salvelinus fontinalis	Proteocephalus tumidocollus	8	0.11	0.08	0.04	0.10
0	0	0.31	0	cv	1	Metabronema salvelini	Crepidostomum cooperi	8	0.02	0.10	0.04	0.09
0	1	0.56	0	t	1	Raphidascaris sp.	Crepidostomum cooperi	8	0.04	0.10	0.04	0.09
0	1	0.75	0	t	1	Phyllodistomum lachancei	Eubothrium salvelini	8	0.04	0.10	0.06	0.09
0	1	1.00	0	t	1	Salmo salar	Coregonus clupeaformis	8	0.08	0.08	0.11	0.08
0	0	0.39	0	cv	1	Proteocephalus sp.2	Salmincola siscowet	8	0.05	0.10	0.02	0.10
0	1	0.65	0	t	1	Eubothrium salvelini	Proteocephalus pinguis	8	0.06	0.09	0.02	0.05
0	0	0.22	0	t	1	Esox lucius	Phyllodistomum coregoni	8	0.02	0.05	0.02	0.10
0	0	0.13	0	t	1	Salmincola edwardsii	Salmincola coregonorum	8	0.02	0.10	0.02	0.10
0	0	0.45	0	cv	1	Ergasilus caeruleus	Proteocephalus pinguis	8	0.02	0.03	0.02	0.05

0	1	0.66	0	t	1	Proteocephalus sp.2	Capillaria salvelini	8	0.05	0.10	0.04	0.10
0	0	0.49	0	t	1	Triaenophorus crassus	Discocotyle sagittata	8	0.02	0.05	0.04	0.09
1	1	0.95	0	t	1	Coregonus clupeaformis	Tetraonchus monenteron	8	0.11	0.08	0.03	0.09
1	1	0.97	0	cv	1	Salmincola extumescens	Salvelinus fontinalis	8	0.04	0.09	0.11	0.08
0	1	0.70	0	t	1	Catostomus catostomus	Crepidostomum cooperi	8	0.03	0.03	0.04	0.09
0	0	0.21	0	t	1	Metechinorhynchus lateralis	Salmincola siscowet	8	0.03	0.09	0.02	0.10
0	1	0.67	0	t	1	Phyllodistomum lachancei	Catostomus catostomus	8	0.04	0.10	0.03	0.03
0	0	0.22	0	cv	1	Metabronema salvelini	Triaenophorus crassus	8	0.02	0.10	0.02	0.05
0	1	0.75	0	t	1	Proteocephalus sp.2	Diphyllobothrium sp. (L)	8	0.05	0.10	0.05	0.10
0	0	0.22	0	t	1	Esox lucius	Salmincola edwardsii	8	0.02	0.05	0.02	0.10
0	0	0.44	0	t	1	Raphidascaris sp.	Proteocephalus pinguis	8	0.04	0.10	0.02	0.05
0	1	0.65	0	cv	1	Eubothrium salvelini	Triaenophorus crassus	8	0.06	0.09	0.02	0.05
0	1	0.75	0	t	1	Eubothrium salvelini	Crepidostomum cooperi	8	0.06	0.09	0.04	0.09
0	1	0.55	0	t	1	Triaenophorus crassus (L)	Diphyllobothrium sp. (L)	8	0.03	0.10	0.05	0.10
0	0	0.22	0	t	1	Salmincola edwardsii	Tetraonchus monenteron	8	0.02	0.10	0.03	0.09
0	0	0.22	0	cv	1	Salmincola edwardsii	Proteocephalus pinguis	8	0.02	0.10	0.02	0.05
0	0	0.24	0	t	1	Triaenophorus crassus	Salmincola siscowet	8	0.02	0.05	0.02	0.10
0	0	0.37	0	t	1	Raphidascaris canadensis	Salmincola edwardsii	8	0.03	0.04	0.02	0.10
0	1	0.70	0	t	1	Catostomus catostomus	Discocotyle sagittata	8	0.03	0.03	0.04	0.09
0	1	0.62	0	cv	1	Salmincola extumescens	Raphidascaris canadensis	8	0.04	0.09	0.03	0.04
0	1	0.66	0	t	1	Proteocephalus sp.2	Raphidascaris sp.	8	0.05	0.10	0.04	0.10
0	1	0.53	0	t	1	Salmincola extumescens	Neoechinorhynchus crassus	8	0.04	0.09	0.02	0.03
0	0	0.49	0	t	1	Eubothrium salvelini	Salmincola coregonorum	8	0.06	0.09	0.02	0.10
0	0	0.30	0	cv	1	Salmincola extumescens	Phyllodistomum coregoni	8	0.04	0.09	0.02	0.10
0	1	0.82	0	t	1	Eubothrium salvelini	Proteocephalus sp.2	8	0.06	0.09	0.05	0.10

0	0	0.13	0	t	1	Salmincola coregonorum	Salmincola siscowet	8	0.02	0.10	0.02	0.10
0	1	0.70	0	t	1	Catostomus catostomus	Crepidostom um farionis	8	0.03	0.03	0.04	0.10
0	1	0.64	0	cv	1	Raphidascaris canadensis	Discocotyle sagittata	8	0.03	0.04	0.04	0.09
0	1	0.91	0	t	1	Salvelinus namaycush	Metabronem a salvelini	8	0.11	0.08	0.02	0.10
0	1	0.74	0	t	1	Eubothrium salvelini	Proteocephal us sp.1	8	0.06	0.09	0.04	0.10
0	1	0.60	0	t	1	Raphidascaris canadensis	Ergasilus caeruleus	8	0.03	0.04	0.02	0.03
0	1	0.71	0	cv	1	Salmo salar	Phyllodistom um coregoni	8	0.08	0.08	0.02	0.10
0	0	0.44	0	t	1	Proteocephalu s sp.1	Proteocephal us pinguis	8	0.04	0.10	0.02	0.05
0	1	0.82	0	t	1	Salmo salar	Triaenophoru s crassus	8	0.08	0.08	0.02	0.05
0	0	0.44	0	t	1	Phyllodistomu m lachancei	Tetraonchus monenteron	8	0.04	0.10	0.03	0.09
0	1	0.56	0	cv	1	Proteocephalu s tumidocollus	Crepidostom um cooperi	8	0.04	0.10	0.04	0.09
0	0	0.21	0	t	1	Triaenophorus crassus (L)	Phyllodistom um coregoni	8	0.03	0.10	0.02	0.10
0	1	0.66	0	t	1	Proteocephalu s sp.2	Proteocephal us tumidocollus	8	0.05	0.10	0.04	0.10
0	1	0.57	0	t	1	Neoechinorhy nchus crassus	Proteocephal us tumidocollus	8	0.02	0.03	0.04	0.10
0	0	0.34	0	cv	1	Metechinorhy nchus lateralis	Triaenophoru s crassus	8	0.03	0.09	0.02	0.05
0	1	0.63	0	t	1	Eubothrium salvelini	Esox lucius	8	0.06	0.09	0.02	0.05
0	0	0.44	0	t	1	Phyllodistomu m lachancei	Triaenophoru s crassus (L)	8	0.04	0.10	0.03	0.10
0	0	0.45	0	t	1	Tetraonchus monenteron	Capillaria salvelini	8	0.03	0.09	0.04	0.10
0	1	0.96	0	cv	1	Esox lucius	Coregonus clupeaformis	8	0.02	0.05	0.11	0.08
0	0	0.49	1	t	1	Leptomyrmex unicolor	Normanbya normanbyi	9	0.03	0.04	0.02	0.04
1	1	0.67	1	t	1	Leptomyrmex unicolor	Cryptocarya murrayi	9	0.03	0.04	0.04	0.04
0	0	0.49	1	t	1	Leptomyrmex unicolor	Cardwellia sublimis	9	0.03	0.04	0.02	0.04
0	1	0.55	1	cv	1	Leptomyrmex unicolor	Licuala ramsayi	9	0.03	0.04	0.02	0.04
0	1	0.61	1	t	1	Leptomyrmex unicolor	Ichnocarpus frutescens	9	0.03	0.04	0.03	0.04

1	1	0.86	1	t	1	Leptomyrmex unicolor	Merremia peltata	9	0.03	0.04	0.06	0.04
1	1	0.83	1	t	1	Leptomyrmex unicolor	Homalanthus novoguineensis	9	0.03	0.04	0.05	0.04
0	0	0.35	1	cv	1	Leptomyrmex unicolor	Archontophoenix alexandrae	9	0.03	0.04	0.00	0.04
1	1	0.83	1	t	1	Leptomyrmex unicolor	Flagellaria indica	9	0.03	0.04	0.05	0.04
0	0	0.30	1	t	1	Camponotus sp.1 (macrocephalus gp.)	Clerodendrum tracyanum	9	0.01	0.05	0.02	0.04
0	0	0.24	1	t	1	Camponotus sp.1 (macrocephalus gp.)	Euodia sp.	9	0.01	0.05	0.01	0.04
1	1	0.74	1	cv	1	Camponotus sp.1 (macrocephalus gp.)	Macaranga involucrata subsp. mallotoides	9	0.01	0.05	0.06	0.04
0	0	0.44	1	t	1	Normanbya normanbyi	Polyrhachis (Cyrtomyrma) 'Cyrto 03'	9	0.02	0.04	0.02	0.04
1	1	0.79	1	t	1	Normanbya normanbyi	Crematogaster aff. fusca	9	0.02	0.04	0.06	0.04
0	1	0.64	1	t	1	Normanbya normanbyi	Polyrhachis (Myrma) foreli	9	0.02	0.04	0.04	0.04
1	1	0.80	1	cv	1	Normanbya normanbyi	Oecophylla smaragdina	9	0.02	0.04	0.06	0.04
0	1	0.63	1	t	1	Polyrhachis (Cyrtomyrma) 'Cyrto 03'	Cryptocarya murrayi	9	0.02	0.04	0.04	0.04
0	0	0.47	1	t	1	Polyrhachis (Cyrtomyrma) 'Cyrto 03'	Rockinghamia angustifolia	9	0.02	0.04	0.02	0.04
0	0	0.50	1	t	1	Polyrhachis (Cyrtomyrma) 'Cyrto 03'	Licuala ramsayi	9	0.02	0.04	0.02	0.04
0	1	0.69	1	cv	1	Polyrhachis (Cyrtomyrma) 'Cyrto 03'	Ipomoea indica	9	0.02	0.04	0.04	0.04
0	1	0.53	1	t	1	Polyrhachis (Cyrtomyrma) 'Cyrto 03'	Syzygium erythrocalyx	9	0.02	0.04	0.03	0.04
0	1	0.80	1	t	1	Polyrhachis (Cyrtomyrma) 'Cyrto 03'	Homalanthus novoguineensis	9	0.02	0.04	0.05	0.04

0	1	0.59	1	t	1	Cryptocarya murrayi	Turneria bidentata	9	0.04	0.04	0.02	0.05
0	1	0.75	1	cv	1	Cryptocarya murrayi	Monomorium floricola	9	0.04	0.04	0.04	0.05
0	1	0.75	1	t	1	Cryptocarya murrayi	Camponotus vitreus	9	0.04	0.04	0.04	0.05
1	1	0.89	1	t	1	Cryptocarya murrayi	Crematogast er aff. fusca	9	0.04	0.04	0.06	0.04
0	1	0.79	1	t	1	Cryptocarya murrayi	Polyrhachis (Myrma) foreli	9	0.04	0.04	0.04	0.04
1	1	0.80	1	cv	1	Cryptocarya murrayi	Crematogast er aff. pythia	9	0.04	0.04	0.04	0.05
0	1	0.89	1	t	1	Cryptocarya murrayi	Oecophylla smaragdina	9	0.04	0.04	0.06	0.04
0	1	0.59	1	t	1	Cryptocarya murrayi	Tetraponera nitida	9	0.04	0.04	0.02	0.05
0	1	0.77	1	t	1	Cryptocarya murrayi	Technomyrm ex albipes gp.	9	0.04	0.04	0.04	0.05
0	0	0.42	1	cv	1	Cryptocarya murrayi	Podomyrma sp.1	9	0.04	0.04	0.00	0.04
1	1	0.71	1	t	1	Trichosanthes pentaphylla	Crematogast er aff. fusca	9	0.01	0.04	0.06	0.04
1	1	0.73	1	t	1	Trichosanthes pentaphylla	Oecophylla smaragdina	9	0.01	0.04	0.06	0.04
0	0	0.41	1	t	1	Camponotus sp.6 (gasseri gp.)	Dysoxylum pettigrewian um	9	0.00	0.04	0.03	0.04
1	1	0.71	1	cv	1	Smilax cf. australis	Crematogast er aff. fusca	9	0.01	0.04	0.06	0.04
1	1	0.73	1	t	1	Smilax cf. australis	Oecophylla smaragdina	9	0.01	0.04	0.06	0.04
0	1	0.63	1	t	1	Syzygium gustavioides	Anonychomy rma gilberti	9	0.01	0.04	0.05	0.04
0	1	0.55	1	t	1	Syzygium gustavioides	Polyrhachis (Myrma) foreli	9	0.01	0.04	0.04	0.04
0	0	0.29	1	cv	1	Polyrhachis (Cyrtomyrma) 'Cyrto 08'	Elaeocarpus angustifolius	9	0.01	0.05	0.02	0.04
0	1	0.55	1	t	1	Polyrhachis (Cyrtomyrma) 'Cyrto 08'	Entada phaseoloides	9	0.01	0.05	0.04	0.04
0	1	0.71	1	t	1	Polyrhachis (Cyrtomyrma) 'Cyrto 08'	Flagellaria indica	9	0.01	0.05	0.05	0.04
1	1	0.63	1	t	1	Polyrhachis (Cyrtomyrma) yorkana	Dysoxylum pettigrewian um	9	0.03	0.05	0.03	0.04

1	1	0.58	1	cv	1	Polyrhachis (Cyrtomyrma) yorkana	Ichnocarpus frutescens	9	0.03	0.05	0.03	0.04
1	1	0.71	1	t	1	Polyrhachis (Cyrtomyrma) yorkana	Ipomoea indica	9	0.03	0.05	0.04	0.04
1	1	0.85	1	t	1	Polyrhachis (Cyrtomyrma) yorkana	Merremia peltata	9	0.03	0.05	0.06	0.04
0	0	0.36	1	t	1	Polyrhachis (Cyrtomyrma) yorkana	unidentified liana	9	0.03	0.05	0.01	0.04
1	1	0.82	1	cv	1	Polyrhachis (Cyrtomyrma) yorkana	Homalanthus novoguineen sis	9	0.03	0.05	0.05	0.04
1	1	0.67	1	t	1	Polyrhachis (Cyrtomyrma) yorkana	Ardisia pachyrrhachi s	9	0.03	0.05	0.04	0.04
1	1	0.82	1	t	1	Polyrhachis (Cyrtomyrma) yorkana	Flagellaria indica	9	0.03	0.05	0.05	0.04
0	0	0.46	1	t	1	Dysoxylum mollissimum subsp. molle	Monomorium floricola	9	0.00	0.04	0.04	0.05
0	1	0.63	1	cv	1	Rockinghamia angustifolia	Monomorium floricola	9	0.02	0.04	0.04	0.05
0	1	0.74	1	t	1	Rockinghamia angustifolia	Anonychomy rma gilberti	9	0.02	0.04	0.05	0.04
0	0	0.41	1	t	1	Rockinghamia angustifolia	Paratrechina minutula gp.	9	0.02	0.04	0.02	0.05
0	1	0.81	1	t	1	Rockinghamia angustifolia	Crematogast er aff. fusca	9	0.02	0.04	0.06	0.04
0	1	0.69	1	cv	1	Rockinghamia angustifolia	Crematogast er aff. pythia	9	0.02	0.04	0.04	0.05
0	1	0.57	1	t	1	Clerodendrum tracyanum	Camponotus vitreus	9	0.02	0.04	0.04	0.05
0	1	0.77	1	t	1	Clerodendrum tracyanum	Crematogast er aff. fusca	9	0.02	0.04	0.06	0.04
0	1	0.59	1	t	1	Clerodendrum tracyanum	Technomyrm ex albipes gp.	9	0.02	0.04	0.04	0.05
0	1	0.59	1	cv	1	Embelia caulialata	Anonychomy rma gilberti	9	0.00	0.04	0.05	0.04
1	1	0.68	1	t	1	Odontomachu s cephalotes	Macaranga involucrata subsp. mallotoides	9	0.00	0.05	0.06	0.04
0	1	0.71	1	t	1	Cardwellia sublimis	Anonychomy rma gilberti	9	0.02	0.04	0.05	0.04
0	1	0.66	1	t	1	Cardwellia sublimis	Crematogast er aff. pythia	9	0.02	0.04	0.04	0.05

0	1	0.80	1	cv	1	Cardwellia sublimis	Oecophylla smaragdina	9	0.02	0.04	0.06	0.04
0	1	0.62	1	t	1	Cardwellia sublimis	Technomyrmex albipes gp.	9	0.02	0.04	0.04	0.05
0	1	0.53	1	t	1	Syzygium sayeri	Camponotus vitreus	9	0.01	0.04	0.04	0.05
0	1	0.66	1	t	1	Syzygium sayeri	Anonychomyrma gilberti	9	0.01	0.04	0.05	0.04
0	0	0.41	1	cv	1	Syzygium sayeri	Polyrhachis (Cyrtomyrma) 'Cyrto NB5041	9	0.01	0.04	0.03	0.05
0	1	0.54	1	t	1	Archontophoenix alexandrae2	Polyrhachis (Myrma) foreli	9	0.01	0.04	0.04	0.04
1	1	0.72	1	t	1	Archontophoenix	Oecophylla smaragdina	9	0.01	0.04	0.06	0.04
1	1	0.69	1	t	1	Dysoxylum papuanum	Oecophylla smaragdina	9	0.00	0.05	0.06	0.04
1	1	0.74	1	cv	1	Tetramorium insolens	Macaranga involucrata subsp. mallotoides	9	0.01	0.05	0.06	0.04
1	1	0.58	1	t	1	Tetramorium insolens	Ipomoea indica	9	0.01	0.05	0.04	0.04
0	1	0.55	1	t	1	Tetramorium insolens	Entada phaseoloides	9	0.01	0.05	0.04	0.04
1	1	0.68	1	t	1	Strumigenys guttulata	Macaranga involucrata subsp. mallotoides	9	0.00	0.05	0.06	0.04
0	0	0.28	1	cv	1	Turneria bidentata	Neosepicaea jucunda	9	0.02	0.05	0.00	0.04
1	1	0.81	1	t	1	Turneria bidentata	Macaranga involucrata subsp. mallotoides	9	0.02	0.05	0.06	0.04
0	1	0.58	1	t	1	Turneria bidentata	Dysoxylum pettigrewianum	9	0.02	0.05	0.03	0.04
0	0	0.50	1	t	1	Turneria bidentata	Castanospermum australe	9	0.02	0.05	0.03	0.04
1	1	0.82	1	cv	1	Turneria bidentata	Merremia peltata	9	0.02	0.05	0.06	0.04
0	1	0.54	1	t	1	Rockinghamia angustifolia2	Polyrhachis (Myrma) foreli	9	0.01	0.04	0.04	0.04
1	1	0.72	1	t	1	Rockinghamia angustifolia2	Oecophylla smaragdina	9	0.01	0.04	0.06	0.04

1	1	0.84	1	t	1	Tetramorium validiusculum	Macaranga involucrata subsp. mallotoides	9	0.03	0.05	0.06	0.04
1	1	0.71	1	cv	1	Tetramorium validiusculum	Ipomoea indica	9	0.03	0.05	0.04	0.04
1	1	0.85	1	t	1	Tetramorium validiusculum	Merremia peltata	9	0.03	0.05	0.06	0.04
1	1	0.82	1	t	1	Tetramorium validiusculum	Homalanthus novoguineensis	9	0.03	0.05	0.05	0.04
1	1	0.67	1	t	1	Tetramorium validiusculum	Ardisia pachyrrhachis	9	0.03	0.05	0.04	0.04
1	1	0.69	1	cv	1	Tetramorium validiusculum	Entada phaseoloides	9	0.03	0.05	0.04	0.04
1	1	0.82	1	t	1	Tetramorium validiusculum	Flagellaria indica	9	0.03	0.05	0.05	0.04
1	1	0.53	1	t	1	Tetramorium validiusculum	Endospermum myrmecophilum	9	0.03	0.05	0.02	0.04
1	1	0.89	1	t	1	Monomorium floricola	Macaranga involucrata subsp. mallotoides	9	0.04	0.05	0.06	0.04
1	1	0.73	1	cv	1	Monomorium floricola	Dysoxylum pettigrewianum	9	0.04	0.05	0.03	0.04
1	1	0.80	1	t	1	Monomorium floricola	Ipomoea indica	9	0.04	0.05	0.04	0.04
1	1	0.90	1	t	1	Monomorium floricola	Merremia peltata	9	0.04	0.05	0.06	0.04
0	1	0.67	1	t	1	Monomorium floricola	Syzygium erythrocalyx	9	0.04	0.05	0.03	0.04
0	1	0.51	1	cv	1	Monomorium floricola	Adenia heterophylla	9	0.04	0.05	0.01	0.04
1	1	0.88	1	t	1	Monomorium floricola	Homalanthus novoguineensis	9	0.04	0.05	0.05	0.04
1	1	0.77	1	t	1	Monomorium floricola	Ardisia pachyrrhachis	9	0.04	0.05	0.04	0.04
1	1	0.79	1	t	1	Monomorium floricola	Entada phaseoloides	9	0.04	0.05	0.04	0.04
1	1	0.88	1	cv	1	Monomorium floricola	Flagellaria indica	9	0.04	0.05	0.05	0.04
0	1	0.60	1	t	1	Glochidion philippicum	Camponotus vitreus	9	0.02	0.04	0.04	0.05
0	1	0.72	1	t	1	Glochidion philippicum	Anonychomyrma gilberti	9	0.02	0.04	0.05	0.04

0	0	0.47	1	t	1	Glochidion philippicum	Polyrhachis (Cyrtomyrma) 'Cyrto NB5041	9	0.02	0.04	0.03	0.05
0	1	0.79	1	cv	1	Glochidion philippicum	Crematogast er aff. fusca	9	0.02	0.04	0.06	0.04
0	1	0.64	1	t	1	Glochidion philippicum	Polyrhachis (Myrma) foreli	9	0.02	0.04	0.04	0.04
0	0	0.46	1	t	1	Polyrhachis (Hagiomyrma) thusnelda	Dysoxylum pettigrewian um	9	0.01	0.04	0.03	0.04
0	0	0.21	1	t	1	Polyrhachis (Hagiomyrma) thusnelda	Pachygone longifolia	9	0.01	0.04	0.01	0.04
0	1	0.76	1	cv	1	Licuala ramsayi	Anonychomy rma gilberti	9	0.02	0.04	0.05	0.04
0	1	0.69	1	t	1	Licuala ramsayi	Polyrhachis (Myrma) foreli	9	0.02	0.04	0.04	0.04
0	1	0.71	1	t	1	Licuala ramsayi	Crematogast er aff. pythia	9	0.02	0.04	0.04	0.05
0	1	0.83	1	t	1	Licuala ramsayi	Oecophylla smaragdina	9	0.02	0.04	0.06	0.04
0	1	0.67	1	cv	1	Licuala ramsayi	Technomyrm ex albipes gp.	9	0.02	0.04	0.04	0.05
1	1	0.69	1	t	1	Syzygium cormiflorum	Oecophylla smaragdina	9	0.00	0.05	0.06	0.04
0	0	0.24	1	t	1	Euodia sp.	Monomorium cf. intrudens	9	0.01	0.04	0.01	0.05
0	0	0.32	1	t	1	Elaeocarpus angustifolius	Polyrhachis (Cyrtomyrma) 'Cyrto 06'	9	0.02	0.04	0.02	0.05
0	1	0.69	1	cv	1	Elaeocarpus angustifolius	Anonychomy rma gilberti	9	0.02	0.04	0.05	0.04
1	1	0.78	1	t	1	Elaeocarpus angustifolius	Oecophylla smaragdina	9	0.02	0.04	0.06	0.04
0	1	0.51	1	t	1	Mucuna gigantea	Paratrechina vaga gp.	9	0.01	0.04	0.04	0.05
0	1	0.61	1	t	1	Mucuna gigantea	Crematogast er aff. pythia	9	0.01	0.04	0.04	0.05
0	0	0.46	1	cv	1	Mucuna gigantea	Pheidole aff. platypus	9	0.01	0.04	0.03	0.05
1	1	0.88	1	t	1	Macaranga involucrata subsp. mallotoides	Paratrechina vaga gp.	9	0.06	0.04	0.04	0.05

1	1	0.83	1	t	1	Macaranga involucrata subsp. mallotoides	Tapinoma melanocephalum	9	0.06	0.04	0.03	0.05
1	1	0.89	1	t	1	Macaranga involucrata subsp. mallotoides	Camponotus vitreus	9	0.06	0.04	0.04	0.05
0	1	0.75	1	cv	1	Macaranga involucrata subsp. mallotoides	Polyrhachis (Cyrtomyrma) 'Cyrto 06'	9	0.06	0.04	0.02	0.05
1	1	0.93	1	t	1	Macaranga involucrata subsp. mallotoides	Anonychomyrma gilberti	9	0.06	0.04	0.05	0.04
1	1	0.83	1	t	1	Macaranga involucrata subsp. mallotoides	Polyrhachis (Cyrtomyrma) 'Cyrto NB5041	9	0.06	0.04	0.03	0.05
1	1	0.79	1	t	1	Macaranga involucrata subsp. mallotoides	Camponotus spp. 'nocturnal' (novae-hollandiae gp., macrocephalus)	9	0.06	0.04	0.02	0.05
1	1	0.79	1	cv	1	Macaranga involucrata subsp. mallotoides	Rhoptromyrmex wroughtonii	9	0.06	0.04	0.02	0.05
1	1	0.95	1	t	1	Macaranga involucrata subsp. mallotoides	Crematogaster aff. fusca	9	0.06	0.04	0.06	0.04
1	1	0.91	1	t	1	Macaranga involucrata subsp. mallotoides	Polyrhachis (Myrma) foreli	9	0.06	0.04	0.04	0.04
1	1	0.91	1	t	1	Macaranga involucrata subsp. mallotoides	Crematogaster aff. pythia	9	0.06	0.04	0.04	0.05
1	1	0.96	1	cv	1	Macaranga involucrata subsp. mallotoides	Oecophylla smaragdina	9	0.06	0.04	0.06	0.04

0	1	0.79	1	t	1	Macaranga involucrata subsp. mallotoides	Tetraponera nitida	9	0.06	0.04	0.02	0.05
1	1	0.90	1	t	1	Macaranga involucrata subsp. mallotoides	Technomyrmex albipes gp.	9	0.06	0.04	0.04	0.05
1	1	0.86	1	t	1	Macaranga involucrata subsp. mallotoides	Pheidole aff. platypus	9	0.06	0.04	0.03	0.05
0	1	0.74	1	cv	1	Macaranga involucrata subsp. mallotoides	Pheidole sp.1	9	0.06	0.04	0.02	0.05
0	1	0.69	1	t	1	Macaranga involucrata subsp. mallotoides	Monomorium fieldi var. laeve nigrius	9	0.06	0.04	0.01	0.05
1	1	0.75	1	t	1	Monomorium cf. intrudens	Merremia peltata	9	0.01	0.05	0.06	0.04
1	1	0.71	1	t	1	Monomorium cf. intrudens	Homalanthus novoguineensis	9	0.01	0.05	0.05	0.04
1	1	0.76	1	cv	1	Wrightia laevis subsp. millgar	Crematogaster aff. fusca	9	0.02	0.04	0.06	0.04
0	1	0.63	1	t	1	Wrightia laevis subsp. millgar	Crematogaster aff. pythia	9	0.02	0.04	0.04	0.05
1	1	0.78	1	t	1	Wrightia laevis subsp. millgar	Oecophylla smaragdina	9	0.02	0.04	0.06	0.04
0	0	0.38	1	t	1	Wrightia laevis subsp. millgar	Tetraponera nitida	9	0.02	0.04	0.02	0.05
0	1	0.72	1	cv	1	Dysoxylum pettigrewianu	Paratrechina vaga gp.	9	0.03	0.04	0.04	0.05
0	1	0.63	1	t	1	Dysoxylum pettigrewianum	Tapinoma melanocephalum	9	0.03	0.04	0.03	0.05
0	1	0.51	1	t	1	Dysoxylum pettigrewianum	Polyrhachis (Cyrtomyrma) 'Cyrto 06'	9	0.03	0.04	0.02	0.05
0	1	0.83	1	t	1	Dysoxylum pettigrewianu	Anonychomyrma gilberti	9	0.03	0.04	0.05	0.04
0	1	0.63	1	cv	1	Dysoxylum pettigrewianum	Polyrhachis (Cyrtomyrma) 'Cyrto NB5041	9	0.03	0.04	0.03	0.05
0	1	0.88	1	t	1	Dysoxylum pettigrewianu	Crematogaster aff. fusca	9	0.03	0.04	0.06	0.04
1	1	0.79	1	t	1	Paratrechina vaga gp.	Ipomoea indica	9	0.04	0.05	0.04	0.04

1	1	0.89	1	t	1	Paratrechina vaga gp.	Merremia peltata	9	0.04	0.05	0.06	0.04
1	1	0.87	1	cv	1	Paratrechina vaga gp.	Homalanthus novoguineensis	9	0.04	0.05	0.05	0.04
1	0	0.42	1	t	1	Paratrechina vaga gp.	Aleurites rockinghamensis	9	0.04	0.05	0.00	0.04
1	1	0.75	1	t	1	Paratrechina vaga gp.	Ardisia pachyrrhachis	9	0.04	0.05	0.04	0.04
1	1	0.56	1	t	1	Paratrechina vaga gp.	Mallotus mollissimus	9	0.04	0.05	0.02	0.04
1	1	0.87	1	cv	1	Paratrechina vaga gp.	Flagellaria indica	9	0.04	0.05	0.05	0.04
1	1	0.62	1	t	1	Paratrechina vaga gp.	Endospermum myrmecophilum	9	0.04	0.05	0.02	0.04
1	0	0.42	1	t	1	Paratrechina vaga gp.	Crotalaria sp.	9	0.04	0.05	0.00	0.04
1	1	0.71	1	t	1	Tapinoma melanocephalum	Ipomoea indica	9	0.03	0.05	0.04	0.04
1	1	0.85	1	cv	1	Tapinoma melanocephalum	Merremia peltata	9	0.03	0.05	0.06	0.04
1	1	0.82	1	t	1	Tapinoma melanocephalum	Homalanthus novoguineensis	9	0.03	0.05	0.05	0.04
1	1	0.67	1	t	1	Tapinoma melanocephalum	Ardisia pachyrrhachis	9	0.03	0.05	0.04	0.04
1	1	0.69	1	t	1	Tapinoma melanocephalum	Entada phaseoloides	9	0.03	0.05	0.04	0.04
1	1	0.82	1	cv	1	Tapinoma melanocephalum	Flagellaria indica	9	0.03	0.05	0.05	0.04
0	1	0.68	1	t	1	Ardisia pachyrrhachis	Crematogaster aff. fusca	9	0.00	0.04	0.06	0.04
1	1	0.69	1	t	1	Camponotus vitreus	Ichnocarpus frutescens	9	0.04	0.05	0.03	0.04
0	1	0.67	1	t	1	Camponotus vitreus	Castanospermum australe	9	0.04	0.05	0.03	0.04
1	1	0.90	1	cv	1	Camponotus vitreus	Merremia peltata	9	0.04	0.05	0.06	0.04
1	1	0.88	1	t	1	Camponotus vitreus	Homalanthus novoguineensis	9	0.04	0.05	0.05	0.04

0	1	0.58	1	t	1	Camponotus vitreus	Mallotus mollissimus	9	0.04	0.05	0.02	0.04
1	1	0.79	1	t	1	Camponotus vitreus	Entada phaseoloides	9	0.04	0.05	0.04	0.04
1	1	0.88	1	cv	1	Camponotus vitreus	Flagellaria indica	9	0.04	0.05	0.05	0.04
0	1	0.52	1	t	1	Camponotus vitreus	Macaranga tanarius	9	0.04	0.05	0.01	0.04
1	1	0.78	1	t	1	Polyrhachis (Cyrtomyrma) 'Cyrto 06'	Merremia peltata	9	0.02	0.05	0.06	0.04
0	1	0.80	1	t	1	Ichnocarpus frutescens	Anonychomy rma gilberti	9	0.03	0.04	0.05	0.04
0	1	0.58	1	cv	1	Ichnocarpus frutescens	Polyrhachis (Cyrtomyrma) 'Cyrto NB5041	9	0.03	0.04	0.03	0.05
0	1	0.85	1	t	1	Ichnocarpus frutescens	Crematogast er aff. fusca	9	0.03	0.04	0.06	0.04
0	1	0.74	1	t	1	Ichnocarpus frutescens	Polyrhachis (Myrma) foreli	9	0.03	0.04	0.04	0.04
0	1	0.52	1	t	1	Ichnocarpus frutescens	Tetraponera nitida	9	0.03	0.04	0.02	0.05
0	1	0.72	1	cv	1	Ichnocarpus frutescens	Technomyrm ex albipes gp.	9	0.03	0.04	0.04	0.05
0	1	0.53	1	t	1	Mallotus paniculatus	Crematogast er aff. pythia	9	0.00	0.04	0.04	0.05
0	0	0.37	1	t	1	Polyrhachis (Myrmhopla) mucronata	Castanosper mum australe	9	0.01	0.05	0.03	0.04
1	1	0.68	1	t	1	Polyrhachis (Myrmhopla) mucronata	Homalanthus novoguineen sis	9	0.01	0.05	0.05	0.04
0	1	0.60	1	cv	1	Passiflora sp. indet.	Crematogast er aff. pythia	9	0.01	0.04	0.04	0.05
1	1	0.75	1	t	1	Passiflora sp. indet.	Oecophylla smaragdina	9	0.01	0.04	0.06	0.04
0	0	0.24	1	t	1	Passiflora sp. indet.	Echinopla australis	9	0.01	0.04	0.01	0.05
1	1	0.69	1	t	1	Toona ciliata	Oecophylla smaragdina	9	0.00	0.05	0.06	0.04
0	0	0.36	1	cv	1	Castanosperm um australe	Polyrhachis (Hedomyrma) cupreata	9	0.03	0.04	0.01	0.04
1	1	0.78	1	t	1	Castanosperm um australe	Anonychomy rma gilberti	9	0.03	0.04	0.05	0.04
1	1	0.84	1	t	1	Castanosperm um australe	Crematogast er aff. fusca	9	0.03	0.04	0.06	0.04
1	1	0.85	1	t	1	Castanosperm um australe	Oecophylla smaragdina	9	0.03	0.04	0.06	0.04

0	1	0.69	1	cv	1	Castanospermum australe	Technomyrmex albipes gp.	9	0.03	0.04	0.04	0.05
0	1	0.63	1	t	1	Ipomoea indica	Paratrechina minutula gp.	9	0.04	0.04	0.02	0.05
0	1	0.66	1	t	1	Ipomoea indica	Camponotus spp. 'nocturnal' (novae-hollandiae gp., macrocephalus)	9	0.04	0.04	0.02	0.05
0	1	0.66	1	t	1	Ipomoea indica	Rhoptromyrmex wroughtonii	9	0.04	0.04	0.02	0.05
0	1	0.91	1	cv	1	Ipomoea indica	Crematogaster aff. fusca	9	0.04	0.04	0.06	0.04
0	1	0.83	1	t	1	Ipomoea indica	Polyrhachis (Myrma) foreli	9	0.04	0.04	0.04	0.04
0	1	0.84	1	t	1	Ipomoea indica	Crematogaster aff. pythia	9	0.04	0.04	0.04	0.05
0	1	0.82	1	t	1	Ipomoea indica	Technomyrmex albipes gp.	9	0.04	0.04	0.04	0.05
0	1	0.75	1	cv	1	Ipomoea indica	Pheidole aff. platypus	9	0.04	0.04	0.03	0.05
0	0	0.30	1	t	1	Polyrhachis (Hedomyrma) cupreata	Mallotus mollissimus	9	0.01	0.04	0.02	0.04
1	1	0.94	1	t	1	Anonychomyrma gilberti	Merremia peltata	9	0.05	0.04	0.06	0.04
1	1	0.77	1	t	1	Anonychomyrma gilberti	Syzygium erythrocalyx	9	0.05	0.04	0.03	0.04
0	1	0.64	1	cv	1	Anonychomyrma gilberti	Adenia heterophylla	9	0.05	0.04	0.01	0.04
1	1	0.92	1	t	1	Anonychomyrma gilberti	Homalanthus novoguineensis	9	0.05	0.04	0.05	0.04
1	1	0.85	1	t	1	Anonychomyrma gilberti	Ardisia pachyrrhachis	9	0.05	0.04	0.04	0.04
1	1	0.86	1	t	1	Anonychomyrma gilberti	Entada phaseoloides	9	0.05	0.04	0.04	0.04
1	1	0.92	1	cv	1	Anonychomyrma gilberti	Flagellaria indica	9	0.05	0.04	0.05	0.04
1	1	0.84	1	t	1	Merremia peltata	Polyrhachis (Cyrtomyrma) 'Cyrto NB5041	9	0.06	0.04	0.03	0.05
1	1	0.78	1	t	1	Merremia peltata	Paratrechina minutula gp.	9	0.06	0.04	0.02	0.05

1	1	0.80	1	t	1	Merremia peltata	Rhoptromyrmex wroughtonii	9	0.06	0.04	0.02	0.05
1	1	0.96	1	cv	1	Merremia peltata	Crematogaster aff. fusca	9	0.06	0.04	0.06	0.04
1	1	0.91	1	t	1	Merremia peltata	Polyrhachis (Myrma) foreli	9	0.06	0.04	0.04	0.04
0	1	0.73	1	t	1	Merremia peltata	Tapinoma minutum gp.	9	0.06	0.04	0.01	0.05
1	1	0.92	1	t	1	Merremia peltata	Crematogaster aff. pythia	9	0.06	0.04	0.04	0.05
1	1	0.96	1	cv	1	Merremia peltata	Oecophylla smaragdina	9	0.06	0.04	0.06	0.04
0	1	0.80	1	t	1	Merremia peltata	Tetraponera nitida	9	0.06	0.04	0.02	0.05
1	1	0.90	1	t	1	Merremia peltata	Technomyrmex albipes gp.	9	0.06	0.04	0.04	0.05
0	1	0.70	1	t	1	Merremia peltata	Rhytidoponera spoliata	9	0.06	0.04	0.01	0.05
1	1	0.86	1	cv	1	Merremia peltata	Pheidole aff. platypus	9	0.06	0.04	0.03	0.05
1	1	0.76	1	t	1	Merremia peltata	Pheidole sp.1	9	0.06	0.04	0.02	0.05
0	1	0.70	1	t	1	Merremia peltata	Monomorium fieldi var. laeve nigrius	9	0.06	0.04	0.01	0.05
1	1	0.68	1	t	1	Polyrhachis (Cyrtomyrma) 'Cyrto NB5041	Ardisia pachyrrhachis	9	0.03	0.05	0.04	0.04
1	1	0.69	1	cv	1	Polyrhachis (Cyrtomyrma) 'Cyrto NB5041	Entada phaseoloides	9	0.03	0.05	0.04	0.04
1	1	0.76	1	t	1	Paratrechina minutula gp.	Homalanthus novoguineensis	9	0.02	0.05	0.05	0.04
1	1	0.76	1	t	1	Paratrechina minutula gp.	Flagellaria indica	9	0.02	0.05	0.05	0.04
0	1	0.71	1	t	1	Ficus septica	Crematogaster aff. fusca	9	0.01	0.04	0.06	0.04
0	0	0.42	1	cv	1	Ficus septica	Pheidole aff. platypus	9	0.01	0.04	0.03	0.05
1	1	0.74	1	t	1	Jasminum didymum	Crematogaster aff. fusca	9	0.01	0.04	0.06	0.04
0	1	0.58	1	t	1	Jasminum didymum	Polyrhachis (Myrma) foreli	9	0.01	0.04	0.04	0.04
1	1	0.75	1	t	1	Jasminum didymum	Oecophylla smaragdina	9	0.01	0.04	0.06	0.04
1	1	0.84	1	cv	1	Syzygium erythrocalyx	Crematogaster aff. fusca	9	0.03	0.04	0.06	0.04

0	1	0.73	1	t	1	Syzygium erythrocalyx	Crematogaster aff. pythia	9	0.03	0.04	0.04	0.05
1	1	0.85	1	t	1	Syzygium erythrocalyx	Oecophylla smaragdina	9	0.03	0.04	0.06	0.04
0	0	0.40	1	t	1	Syzygium erythrocalyx	Pheidole aff. impressiceps	9	0.03	0.04	0.01	0.05
0	0	0.32	1	cv	1	Syzygium erythrocalyx	Crematogaster sp.3	9	0.03	0.04	0.00	0.05
1	1	0.78	1	t	1	Camponotus spp. 'nocturnal' (novae-hollandiae gp.,	Homalanthus novoguineensis	9	0.02	0.05	0.05	0.04
1	1	0.62	1	t	1	Camponotus spp. 'nocturnal' (novae-hollandiae gp.,	Ardisia pachyrrhachis	9	0.02	0.05	0.04	0.04
1	1	0.78	1	t	1	Camponotus spp. 'nocturnal' (novae-hollandiae gp.,	Flagellaria indica	9	0.02	0.05	0.05	0.04
0	0	0.47	1	cv	1	Camponotus spp. 'nocturnal' (novae-hollandiae gp.,	Endospermum myrmecophilum	9	0.02	0.05	0.02	0.04
1	1	0.78	1	t	1	Rhoptromyrmex	Flagellaria indica	9	0.02	0.05	0.05	0.04
0	0	0.47	1	t	1	Rhoptromyrmex wroughtonii	Endospermum myrmecophilum	9	0.02	0.05	0.02	0.04
0	0	0.35	1	t	1	Rhoptromyrmex	Macaranga tanarius	9	0.02	0.05	0.01	0.04
1	1	0.72	1	cv	1	unidentified liana	Oecophylla smaragdina	9	0.01	0.04	0.06	0.04
1	1	0.75	1	t	1	Adenia heterophylla	Oecophylla smaragdina	9	0.01	0.04	0.06	0.04
1	1	0.95	1	t	1	Homalanthus novoguineensi	Crematogaster aff. fusca	9	0.05	0.04	0.06	0.04
0	1	0.69	1	t	1	Homalanthus novoguineensi	Tapinoma minutum gp.	9	0.05	0.04	0.01	0.05
1	1	0.90	1	cv	1	Homalanthus novoguineensi	Crematogaster aff. pythia	9	0.05	0.04	0.04	0.05
1	1	0.89	1	t	1	Homalanthus novoguineensi	Technomyrmex albipes gp.	9	0.05	0.04	0.04	0.05
1	1	0.84	1	t	1	Homalanthus novoguineensi	Pheidole aff. platypus	9	0.05	0.04	0.03	0.05

0	1	0.72	1	t	1	Homalanthus novoguineensi	Pheidole sp.1	9	0.05	0.04	0.02	0.05
0	1	0.69	1	cv	1	Homalanthus novoguineensi	Pheidole aff. impressiceps	9	0.05	0.04	0.01	0.05
0	1	0.62	1	t	1	Homalanthus novoguineensi s	Polyrhachis (Myrmothrin ax) delicata	9	0.05	0.04	0.00	0.05
0	1	0.62	1	t	1	Homalanthus novoguineensi	Crematogast er sp.3	9	0.05	0.04	0.00	0.05
1	1	0.89	1	t	1	Crematogaster aff. fusca	Ardisia pachyrrhachi s	9	0.06	0.04	0.04	0.04
1	1	0.77	1	cv	1	Crematogaster aff. fusca	Mallotus mollissimus	9	0.06	0.04	0.02	0.04
1	1	0.90	1	t	1	Crematogaster aff. fusca	Entada phaseoloides	9	0.06	0.04	0.04	0.04
1	1	0.95	1	t	1	Crematogaster aff. fusca	Flagellaria indica	9	0.06	0.04	0.05	0.04
1	1	0.81	1	t	1	Crematogaster aff. fusca	Endospermu m myrmecophil um	9	0.06	0.04	0.02	0.04
0	1	0.52	1	cv	1	Ardisia pachyrrhachis	Tapinoma minutum gp.	9	0.04	0.04	0.01	0.05
0	1	0.82	1	t	1	Ardisia pachyrrhachis	Crematogast er aff. pythia	9	0.04	0.04	0.04	0.05
0	1	0.79	1	t	1	Ardisia pachyrrhachis	Technomyrm ex albipes gp.	9	0.04	0.04	0.04	0.05
0	1	0.72	1	t	1	Ardisia pachyrrhachis	Pheidole aff. platypus	9	0.04	0.04	0.03	0.05
0	1	0.52	1	cv	1	Mallotus mollissimus	Pheidole aff. platypus	9	0.02	0.04	0.03	0.05
1	1	0.81	1	t	1	Polyrhachis (Myrma) foreli	Entada phaseoloides	9	0.04	0.04	0.04	0.04
1	1	0.90	1	t	1	Polyrhachis (Myrma) foreli	Flagellaria indica	9	0.04	0.04	0.05	0.04
0	0	0.48	1	t	1	Polyrhachis (Myrma) foreli	Stephania japonica	9	0.04	0.04	0.00	0.04
1	1	0.91	1	cv	1	Entada phaseoloides	Oecophylla smaragdina	9	0.04	0.04	0.06	0.04
0	1	0.63	1	t	1	Entada phaseoloides	Tetraponera nitida	9	0.04	0.04	0.02	0.05
0	0	0.50	1	t	1	Entada phaseoloides	Rhytidoponer a spoliata	9	0.04	0.04	0.01	0.05
0	0	0.46	1	t	1	Entada phaseoloides	Crematogast er sp.3	9	0.04	0.04	0.00	0.05
1	1	0.90	1	cv	1	Crematogaster aff. pythia	Flagellaria indica	9	0.04	0.05	0.05	0.04
1	1	0.58	1	t	1	Crematogaster aff. pythia	Macaranga tanarius	9	0.04	0.05	0.01	0.04

1	1	0.95	1	t	1	Oecophylla smaragdina	Flagellaria indica	9	0.06	0.04	0.05	0.04
0	1	0.70	1	t	1	Oecophylla smaragdina	Pachygone longifolia	9	0.06	0.04	0.01	0.04
0	1	0.75	1	cv	1	Oecophylla smaragdina	Cryptocarya hypospodia	9	0.06	0.04	0.02	0.05
0	0	0.46	1	t	1	Technomyrmex albipes gp.	Urena lobata	9	0.04	0.05	0.00	0.04
1	1	0.89	1	t	1	Technomyrmex albipes gp.	Flagellaria indica	9	0.04	0.05	0.05	0.04
1	1	0.66	1	t	1	Technomyrmex albipes gp.	Endospermum myrmecophilum	9	0.04	0.05	0.02	0.04
0	1	0.66	1	cv	1	Flagellaria indica	Echinopla australis	9	0.05	0.04	0.01	0.05
1	1	0.84	1	t	1	Flagellaria indica	Pheidole aff. platypus	9	0.05	0.04	0.03	0.05
0	1	0.72	1	t	1	Flagellaria indica	Pheidole sp.1	9	0.05	0.04	0.02	0.05
0	1	0.69	1	t	1	Flagellaria indica	Pheidole aff. impressiceps	9	0.05	0.04	0.01	0.05
0	1	0.62	1	cv	1	Flagellaria indica	Crematogaster sp.3	9	0.05	0.04	0.00	0.05
0	1	0.58	1	t	1	Endospermum myrmecophilu	Pheidole aff. platypus	9	0.02	0.04	0.03	0.05
0	0	0.33	0	t	1	Syzygium cormiflorum	Polyrhachis (Cyrtomyrma) 'Cyrto NB5041	9	0.00	0.05	0.03	0.05
0	0	0.23	0	t	1	Tetramorium insolens	Pachygone longifolia	9	0.01	0.05	0.01	0.04
0	0	0.30	0	cv	1	Polyrhachis (Hagiomyrma) thusnelda	Mallotus mollissimus	9	0.01	0.04	0.02	0.04
0	0	0.40	0	t	1	Dysoxylum pettigrewianu	Toona ciliata	9	0.03	0.04	0.00	0.05
0	1	0.51	0	t	1	Euodia sp.	Ardisia pachyrrhachis	9	0.01	0.04	0.04	0.04
0	0	0.19	0	t	1	Smilax cf. australis	Ardisia pachyrrhachis2	9	0.01	0.04	0.00	0.04
0	1	0.87	0	cv	1	Macaranga involucrata subsp. mallotoides	Dysoxylum pettigrewianum	9	0.06	0.04	0.03	0.04
0	0	0.35	0	t	1	Licuala	Ficus septica	9	0.02	0.04	0.01	0.04
0	1	0.66	0	t	1	Mallotus mollissimus	Crematogaster aff. pythia	9	0.02	0.04	0.04	0.05

0	0	0.35	0	t	1	Polyrhachis (Cyrtomyrma) yorkana	Polyrhachis (Myrmhopla) mucronata	9	0.03	0.05	0.01	0.05
0	0	0.40	0	cv	1	Licuala	Pheidole sp.1	9	0.02	0.04	0.02	0.05
0	0	0.21	0	t	1	Strumigenys guttulata	Jasminum didymum	9	0.00	0.05	0.01	0.04
0	0	0.16	0	t	1	Strumigenys guttulata	Polyrhachis (Myrmothrin ax) delicata	9	0.00	0.05	0.00	0.05
0	0	0.33	0	t	1	Strumigenys guttulata	Castanosper mum australe	9	0.00	0.05	0.03	0.04
0	0	0.40	0	cv	1	Syzygium erythrocalyx	Tapinoma minutum gp.	9	0.03	0.04	0.01	0.05
0	0	0.29	0	t	1	Polyrhachis (Cyrtomyrma) 'Cyrto 03'	Strumigenys guttulata	9	0.02	0.04	0.00	0.05
0	0	0.36	0	t	1	Polyrhachis (Cyrtomyrma) yorkana	Rockinghami a angustifolia2	9	0.03	0.05	0.01	0.04
0	0	0.18	0	t	1	Toona ciliata	Pachygone longifolia	9	0.00	0.05	0.01	0.04
0	0	0.20	0	cv	1	Pheidole aff. impressiceps	Polyrhachis (Myrmothrin ax) delicata	9	0.01	0.05	0.00	0.05
0	1	0.80	0	t	1	Homalanthus novoguineensi s	Endospermu m myrmecophil um	9	0.05	0.04	0.02	0.04
0	1	0.69	0	t	1	Macaranga involucrata subsp. mallotoides	unidentified liana	9	0.06	0.04	0.01	0.04
0	0	0.26	0	t	1	Monomorium cf. intrudens	Tapinoma minutum gp.	9	0.01	0.05	0.01	0.05
0	0	0.34	0	cv	1	Neosepicaea jucunda	Tapinoma melanocepha lum	9	0.00	0.04	0.03	0.05
0	0	0.36	0	t	1	Rockinghamia angustifolia	Syzygium sayeri	9	0.02	0.04	0.01	0.04
0	1	0.62	0	t	1	Leptomyrmex unicolor	Pheidole aff. platypus	9	0.03	0.04	0.03	0.05
0	0	0.23	0	t	1	Monomorium cf. intrudens	Monomorium fieldi var. laeve nigrius	9	0.01	0.05	0.01	0.05
0	0	0.18	0	cv	1	Monomorium fieldi var. laeve nigrius	Crematogast er sp.3	9	0.01	0.05	0.00	0.05

1	1	0.53	0	t	1	Polyrhachis (Cyrtomyrma) yorkana	Endospermum myrmecophilum	9	0.03	0.05	0.02	0.04	
0	0	0.33	0	t	1	Camponotus sp.6 (gasseri gp.)	Polyrhachis (Cyrtomyrma) 'Cyrto NB5041	9	0.00	0.04	0.03	0.05	
0	0	0.22	0	t	1	Embelia caulialata	Jasminum didymum	9	0.00	0.04	0.01	0.04	
0	0	0.29	0	cv	1	Normanbya normanbyi	Archontophoenix alexandrae2	9	0.02	0.04	0.01	0.04	
0	0	0.21	0	t	1	Ardisia pachyrrhachis	Tapinoma minutum gp.	9	0.00	0.04	0.01	0.05	
0	0	0.21	0	t	1	Dysoxylum mollissimum subsp. molle	Monomorium cf. intrudens	9	0.00	0.04	0.01	0.05	
0	0	0.28	0	t	1	Paratrechina minutula gp.	Pachygone longifolia	9	0.02	0.05	0.01	0.04	
0	0	0.21	0	cv	1	Pachygone longifolia	Monomorium fieldi var. laeve nigrius	9	0.01	0.04	0.01	0.05	
0	0	0.48	0	t	1	Dysoxylum papuanum	Entada phaseoloides	9	0.00	0.05	0.04	0.04	
0	0	0.45	0	t	1	Paratrechina vaga gp.	Echinopla australis	9	0.04	0.05	0.01	0.05	
0	0	0.48	0	t	1	Dysoxylum pettigrewianu	Jasminum didymum	9	0.03	0.04	0.01	0.04	
0	0	0.17	0	cv	1	Camponotus sp.6 (gasseri gp.)	Embelia caulialata	9	0.00	0.04	0.00	0.04	
0	0	0.19	0	t	1	Dysoxylum papuanum	Polyrhachis (Hagiomyrma) thusnelda	9	0.00	0.05	0.01	0.04	
0	1	0.53	0	t	1	Ardisia pachyrrhachis	Macaranga tanarius	9	0.04	0.04	0.01	0.04	
0	1	0.70	0	t	1	Dysoxylum mollissimum subsp. molle	Oecophylla smaragdina	9	0.00	0.04	0.06	0.04	
0	0	0.27	0	cv	1	Rockinghamia angustifolia2	Elaeocarpus angustifolius	9	0.01	0.04	0.02	0.04	
0	0	0.22	0	t	1	Mallotus paniculatus	Macaranga tanarius	9	0.00	0.04	0.01	0.04	
0	0	0.20	0	t	1	Strumigenys guttulata	Tapinoma minutum gp.	9	0.00	0.05	0.01	0.05	
0	0	0.19	0	t	1	Embelia caulialata	Polyrhachis (Hagiomyrma) thusnelda	9	0.00	0.04	0.01	0.04	
0	0	0.22	0	cv	1	Archontophoenix alexandrae	Macaranga tanarius	9	0.00	0.04	0.01	0.04	

0	0	0.38	0	t	1	Licuala ramsayi	Macaranga tanarius	9	0.02	0.04	0.01	0.04
0	0	0.22	0	t	1	Polyrhachis (Hagiomyrma) thusnelda	Euodia sp.	9	0.01	0.04	0.01	0.04
0	1	0.53	0	t	1	Polyrhachis (Cyrtomyrma) 'Cyrto 03'	Castanospermum australe	9	0.02	0.04	0.03	0.04
0	1	0.75	0	cv	1	Clerodendrum tracyanum	Flagellaria indica	9	0.02	0.04	0.05	0.04
0	0	0.18	0	t	1	Odontomachus cephalotes	Polyrhachis (Myrmhopla) mucronata	9	0.00	0.05	0.01	0.05
0	0	0.17	0	t	1	Aleurites rockinghamensis	Urena lobata	9	0.00	0.04	0.00	0.04
0	0	0.47	0	t	1	Polyrhachis (Myrma) foreli	Crematogaster sp.3	9	0.04	0.04	0.00	0.05
0	0	0.37	0	cv	1	Trichosanthes pentaphylla	Castanospermum australe	9	0.01	0.04	0.03	0.04
0	0	0.23	0	t	1	Elaeocarpus angustifolius	Crematogaster sp.3	9	0.02	0.04	0.00	0.05
0	1	0.62	0	t	1	Polyrhachis (Cyrtomyrma) 'Cyrto 06'	Crematogaster aff. pythia	9	0.02	0.05	0.04	0.05
0	0	0.35	0	t	1	Polyrhachis (Hedomyrma) cupreata	Endospermum myrmecophilum	9	0.01	0.04	0.02	0.04
0	1	0.65	0	cv	1	Camponotus sp.6 (gasseri gp.)	Flagellaria indica	9	0.00	0.04	0.05	0.04
0	0	0.37	0	t	1	Neosepicaea jucunda	Ichnocarpus frutescens	9	0.00	0.04	0.03	0.04
0	0	0.22	0	t	1	Dysoxylum mollissimum subsp. molle	Passiflora sp. indet.	9	0.00	0.04	0.01	0.04
0	0	0.19	0	t	1	Archontophoenix alexandrae	Monomorium fieldi var. laeve nigrius	9	0.00	0.04	0.01	0.05
0	0	0.41	0	cv	1	Euodia sp.	Ichnocarpus frutescens	9	0.01	0.04	0.03	0.04
0	0	0.21	0	t	1	Strumigenys guttulata	Passiflora sp. indet.	9	0.00	0.05	0.01	0.04
0	1	0.83	0	t	1	Polyrhachis (Cyrtomyrma) 'Cyrto 03'	Oecophylla smaragdina	9	0.02	0.04	0.06	0.04
0	0	0.18	0	t	1	Smilax cf. australis	Strumigenys guttulata	9	0.01	0.04	0.00	0.05

1	0	0.45	0	cv	1	Pheidole aff. platypus	Macaranga tanarius	9	0.03	0.05	0.01	0.04
0	1	0.65	0	t	1	Macaranga involucrata subsp. mallotoides	Crematogaster sp.3	9	0.06	0.04	0.00	0.05
0	1	0.54	0	t	1	Camponotus spp. 'nocturnal' (novae-hollandiae gp.,	Pheidole aff. platypus	9	0.02	0.05	0.03	0.05
0	1	0.89	0	t	1	Entada phaseoloides	Flagellaria indica	9	0.04	0.04	0.05	0.04
0	1	0.72	0	cv	1	Macaranga involucrata subsp. mallotoides	Tapinoma minutum gp.	9	0.06	0.04	0.01	0.05
0	0	0.23	0	t	1	Clerodendrum tracyanum	Syzygium cormiflorum	9	0.02	0.04	0.00	0.05
0	0	0.21	0	t	1	Dysoxylum papuanum	Monomorium cf. intrudens	9	0.00	0.05	0.01	0.05
0	0	0.19	0	t	1	Ficus septica	Podomyrma sp.1	9	0.01	0.04	0.00	0.04
0	0	0.32	0	cv	1	Smilax cf. australis	Rockinghamia angustifolia	9	0.01	0.04	0.02	0.04
0	0	0.21	0	t	1	Tapinoma minutum gp.	Crotalaria sp.	9	0.01	0.05	0.00	0.04
0	0	0.19	0	t	1	Camponotus sp.6 (gasseri gp.)	Pachygone longifolia	9	0.00	0.04	0.01	0.04
0	0	0.30	0	t	1	Clerodendrum tracyanum	Adenia heterophylla	9	0.02	0.04	0.01	0.04
0	0	0.43	0	cv	1	Cryptocarya murrayi	Aleurites rockinghamensis	9	0.04	0.04	0.00	0.04
0	0	0.26	0	t	1	Tetramorium insolens	Jasminum didymum	9	0.01	0.05	0.01	0.04
0	0	0.31	0	t	1	Licuala ramsayi	Stephania japonica	9	0.02	0.04	0.00	0.04
0	0	0.23	0	t	1	Tetramorium insolens	Polyrhachis (Myrmhopla) mucronata	9	0.01	0.05	0.01	0.05
0	0	0.17	0	cv	1	Dysoxylum mollissimum subsp. molle	Urena lobata	9	0.00	0.04	0.00	0.04
0	0	0.24	0	t	1	Mucuna gigantea	Rhytidoponera spoliata	9	0.01	0.04	0.01	0.05

0	0	0.34	0	t	1	Tetramorium insolens	Camponotus spp. 'nocturnal' (novae-hollandiae gp., macrocephalus)	9	0.01	0.05	0.02	0.05
0	0	0.46	0	t	1	Tetramorium validiusculum	Mallotus mollissimus	9	0.03	0.05	0.02	0.04
0	1	0.60	0	cv	1	Cardwellia sublimis	Ardisia pachyrrhachis	9	0.02	0.04	0.04	0.04
0	0	0.41	0	t	1	Mucuna gigantea	Tapinoma melanocephalum	9	0.01	0.04	0.03	0.05
0	0	0.38	0	t	1	Licuala ramsayi	Adenia heterophylla	9	0.02	0.04	0.01	0.04
1	1	0.77	0	t	1	Camponotus vitreus	Ardisia pachyrrhachis	9	0.04	0.05	0.04	0.04
0	0	0.17	0	cv	1	Camponotus sp.6 (gasseri gp.)	Mallotus paniculatus	9	0.00	0.04	0.00	0.04
0	0	0.20	0	t	1	Euodia sp.	Stephania japonica	9	0.01	0.04	0.00	0.04
0	1	0.69	0	t	1	Ficus septica	Flagellaria indica	9	0.01	0.04	0.05	0.04
0	1	0.52	0	t	1	Dysoxylum mollissimum subsp. molle	Ipomoea indica	9	0.00	0.04	0.04	0.04
0	0	0.42	0	cv	1	Tetramorium validiusculum	Cryptocarya hypospodia	9	0.03	0.05	0.02	0.05
1	1	0.67	0	t	1	Toona ciliata	Crematogaster aff. fusca	9	0.00	0.05	0.06	0.04
0	0	0.38	0	t	1	Leptomyrmex unicolor	Monomorium fieldi var. laeve nigrius	9	0.03	0.04	0.01	0.05
0	0	0.16	0	t	1	Syzygium cormiflorum	Ardisia pachyrrhachis2	9	0.00	0.05	0.00	0.04
0	0	0.36	0	cv	1	Pheidole aff. platypus	Podomyrma sp.1	9	0.03	0.05	0.00	0.04
0	1	0.51	0	t	1	Crematogaster aff. pythia	Crotalaria sp.	9	0.04	0.05	0.00	0.04
0	1	0.80	0	t	1	Polyrhachis (Cyrtomyrma) 'Cyrto 03'	Flagellaria indica	9	0.02	0.04	0.05	0.04
0	0	0.29	0	t	1	Normanbya normanbyi	Echinopla australis	9	0.02	0.04	0.01	0.05

0	0	0.35	0	cv	1	Cardwellia sublimis	Cryptocarya hypospodia	9	0.02	0.04	0.02	0.05
0	0	0.39	0	t	1	Polyrhachis (Cyrtomyrma) 'Cyrto NB5041	Pheidole aff. impressiceps	9	0.03	0.05	0.01	0.05
0	0	0.44	0	t	1	Camponotus vitreus	Urena lobata	9	0.04	0.05	0.00	0.04
0	1	0.72	0	t	1	Mucuna gigantea	Flagellaria indica	9	0.01	0.04	0.05	0.04
0	0	0.35	0	cv	1	Tetramorium validiusculum	Pachygone longifolia	9	0.03	0.05	0.01	0.04
0	0	0.40	0	t	1	Castanospermum australe	Jasminum didymum	9	0.03	0.04	0.01	0.04
0	0	0.33	0	t	1	Odontomachus cephalotes	Syzygium erythrocalyx	9	0.00	0.05	0.03	0.04
0	0	0.19	0	t	1	Archontophoenix alexandrae2	Aleurites rockinghamensis	9	0.01	0.04	0.00	0.04
0	0	0.19	0	cv	1	Syzygium gustavioides	Stephania japonica	9	0.01	0.04	0.00	0.04
0	0	0.27	0	t	1	Passiflora sp. indet.	Adenia heterophylla	9	0.01	0.04	0.01	0.04
0	0	0.19	0	t	1	Camponotus sp.6 (gasseri gp.)	Ficus septica	9	0.00	0.04	0.01	0.04
0	0	0.38	0	t	1	Wrightia laevis subsp. millgar	Camponotus spp. 'nocturnal' (novae-hollandiae gp., macrocephalus)	9	0.02	0.04	0.02	0.05
0	0	0.34	0	cv	1	Leptomyrmex unicolor	Podomyrma sp.1	9	0.03	0.04	0.00	0.04
1	1	0.69	0	t	1	Trichosanthes pentaphylla	Homalanthus novoguineensis	9	0.01	0.04	0.05	0.04
0	0	0.38	0	t	1	Licuala ramsayi	Jasminum didymum	9	0.02	0.04	0.01	0.04
0	0	0.35	0	t	1	Leptomyrmex unicolor	Dysoxylum mollissimum subsp. molle	9	0.03	0.04	0.00	0.04
1	1	0.85	0	cv	1	Camponotus vitreus	Anonychomyrma gilberti	9	0.04	0.05	0.05	0.04
0	0	0.31	0	t	1	Odontomachus cephalotes	Licuala ramsayi	9	0.00	0.05	0.02	0.04
0	0	0.44	0	t	1	Camponotus vitreus	Crotalaria sp.	9	0.04	0.05	0.00	0.04
0	0	0.28	0	t	1	Paratrechina minutula gp.	Echinopla australis	9	0.02	0.05	0.01	0.05

0	0	0.26	0	cv	1	Wrightia laevis subsp. millgar	Polyrhachis (Hedomyrma) cupreata	9	0.02	0.04	0.01	0.04
0	0	0.37	0	t	1	Trichosanthes pentaphylla	Polyrhachis (Cyrtomyrma) yorkana	9	0.01	0.04	0.03	0.05
0	0	0.25	0	t	1	Toona ciliata	Paratrechina minutula gp.	9	0.00	0.05	0.02	0.05
0	1	0.72	0	t	1	Mucuna gigantea	Homalanthus novoguineen sis	9	0.01	0.04	0.05	0.04
0	0	0.39	0	cv	1	Jasminum didymum	Endospermu m myrmecophil um	9	0.01	0.04	0.02	0.04
0	0	0.29	0	t	1	Embelia caulialata	Rhoptromyr mex wroughtonii	9	0.00	0.04	0.02	0.05
0	0	0.16	0	t	1	Dysoxylum papuanum	Strumigenys guttulata	9	0.00	0.05	0.00	0.05
0	0	0.48	0	t	1	Glochidion philippicum	Syzygium erythrocalyx	9	0.02	0.04	0.03	0.04
0	1	0.53	0	cv	1	Ipomoea indica	unidentified liana	9	0.04	0.04	0.01	0.04
0	0	0.24	0	t	1	Rockinghamia angustifolia2	Pheidole aff. impressiceps	9	0.01	0.04	0.01	0.05
0	1	0.52	0	t	1	Tetramorium insolens	Camponotus vitreus	9	0.01	0.05	0.04	0.05
0	0	0.16	0	t	1	Podomyrma sp.1	Polyrhachis (Myrmothrin ax) delicata	9	0.00	0.04	0.00	0.05
0	0	0.41	0	cv	1	Mallotus mollissimus	Tetraponera nitida	9	0.02	0.04	0.02	0.05
0	1	0.66	0	t	1	Mallotus paniculatus	Homalanthus novoguineen sis	9	0.00	0.04	0.05	0.04
0	0	0.30	0	t	1	Polyrhachis (Cyrtomyrma) 'Cyrto 03'	Syzygium cormiflorum	9	0.02	0.04	0.00	0.05
0	0	0.20	0	t	1	Pheidole aff. impressiceps	Crematogast er sp.3	9	0.01	0.05	0.00	0.05
0	0	0.32	0	cv	1	Tetramorium insolens	Mallotus mollissimus	9	0.01	0.05	0.02	0.04
0	0	0.31	0	t	1	Licuala	Urena lobata	9	0.02	0.04	0.00	0.04
0	0	0.16	0	t	1	Toona ciliata	Archontopho enix alexandrae	9	0.00	0.05	0.00	0.04
0	0	0.43	0	t	1	Camponotus vitreus	Toona ciliata	9	0.04	0.05	0.00	0.05
0	0	0.29	0	cv	1	Elaeocarpus angustifolius	Jasminum didymum	9	0.02	0.04	0.01	0.04

0	0	0.29	0	t	1	Rockinghamia angustifolia	Archontophoenix alexandrae	9	0.02	0.04	0.00	0.04
0	0	0.39	0	t	1	Passiflora sp. indet.	Endospermum myrmecophilum	9	0.01	0.04	0.02	0.04
0	0	0.28	0	t	1	Odontomachus cephalotes	Tetraponera nitida	9	0.00	0.05	0.02	0.05
0	0	0.17	0	cv	1	Ardisia pachyrrhachis 2	Aleurites rockinghamensis	9	0.00	0.04	0.00	0.04
0	0	0.46	0	t	1	Cryptocarya murrayi	Syzygium gustavioides	9	0.04	0.04	0.01	0.04
0	1	0.60	0	t	1	Camponotus vitreus	Rhoptromyrmex wroughtonii	9	0.04	0.05	0.02	0.05
1	1	0.72	0	t	1	Archontophoenix alexandrae2	Macaranga involucrata subsp. mallotoides	9	0.01	0.04	0.06	0.04
0	1	0.65	0	cv	1	Cryptocarya murrayi	Tapinoma melanocephalum	9	0.04	0.04	0.03	0.05
1	1	0.78	0	t	1	Elaeocarpus angustifolius	Merremia peltata	9	0.02	0.04	0.06	0.04
0	0	0.39	0	t	1	Polyrhachis (Cyrtomyrma) 'Cyrto NB5041	Adenia heterophylla	9	0.03	0.05	0.01	0.04
0	1	0.65	0	t	1	Polyrhachis (Myrma) foreli	Tetraponera nitida	9	0.04	0.04	0.02	0.05
0	0	0.48	0	cv	1	Monomorium floricola	Ficus septica	9	0.04	0.05	0.01	0.04
0	0	0.44	0	t	1	Rockinghamia angustifolia	Tetraponera nitida	9	0.02	0.04	0.02	0.05
0	0	0.31	0	t	1	Archontophoenix	Turneria bidentata	9	0.01	0.04	0.02	0.05
0	0	0.21	0	t	1	Embelia caulialata	Pheidole aff. impressiceps	9	0.00	0.04	0.01	0.05
1	1	0.69	0	cv	1	Toona ciliata	Merremia peltata	9	0.00	0.05	0.06	0.04
0	1	0.53	0	t	1	Camponotus sp.1 (macrocephalus gp.)	Ardisia pachyrrhachis	9	0.01	0.05	0.04	0.04
0	0	0.43	0	t	1	Turneria bidentata	Rhoptromyrmex wroughtonii	9	0.02	0.05	0.02	0.05
0	0	0.46	0	t	1	Cryptocarya murrayi	Echinopla australis	9	0.04	0.04	0.01	0.05

0	0	0.31	0	cv	1	Tetramorium validiusculum	Toona ciliata	9	0.03	0.05	0.00	0.05
0	0	0.26	0	t	1	Cardwellia sublimis	Crotalaria sp.	9	0.02	0.04	0.00	0.04
0	0	0.26	0	t	1	Wrightia laevis subsp. millgar	Polyrhachis (Myrmhopla) mucronata	9	0.02	0.04	0.01	0.05
0	0	0.21	0	t	1	Monomorium cf. intrudens	Archontophoenix alexandrae	9	0.01	0.05	0.00	0.04
0	0	0.16	0	cv	1	Camponotus sp.6 (gasseri gp.)	Podomyrma sp.1	9	0.00	0.04	0.00	0.04
0	1	0.58	0	t	1	Polyrhachis (Cyrtomyrma) 'Cyrto 06'	Technomyrmex albipes gp.	9	0.02	0.05	0.04	0.05
0	0	0.19	0	t	1	Polyrhachis (Hagiomyrma) thusnelda	Aleurites rockinghamensis	9	0.01	0.04	0.00	0.04
0	0	0.26	0	t	1	Cardwellia sublimis	Archontophoenix alexandrae	9	0.02	0.04	0.00	0.04
0	0	0.21	0	cv	1	Polyrhachis (Myrmhopla) mucronata	Monomorium fieldi var. laeve nigrius	9	0.01	0.05	0.01	0.05
0	0	0.18	0	t	1	Archontophoenix	Syzygium cormiflorum	9	0.01	0.04	0.00	0.05
0	0	0.24	0	t	1	Monomorium cf. intrudens	Ficus septica	9	0.01	0.05	0.01	0.04
0	1	0.56	0	t	1	Ipomoea indica	Tapinoma minutum gp.	9	0.04	0.04	0.01	0.05
0	0	0.41	0	cv	1	Endospermum myrmecophilu	Cryptocarya hypospodia	9	0.02	0.04	0.02	0.05
1	1	0.81	0	t	1	Rhoptromyrmex	Oecophylla smaragdina	9	0.02	0.05	0.06	0.04
0	0	0.32	0	t	1	Normanbya normanbyi	Monomorium cf. intrudens	9	0.02	0.04	0.01	0.05
0	0	0.24	0	t	1	Trichosanthes pentaphylla	Tetramorium insolens	9	0.01	0.04	0.01	0.05
0	0	0.33	0	cv	1	Syzygium sayeri	Mallotus mollissimus	9	0.01	0.04	0.02	0.04
1	1	0.84	0	t	1	Polyrhachis (Cyrtomyrma) yorkana	Macaranga involucrata subsp. mallotoides	9	0.03	0.05	0.06	0.04
0	0	0.21	0	t	1	Podomyrma sp.1	Pheidole aff. impressiceps	9	0.00	0.04	0.01	0.05
0	0	0.33	0	t	1	Normanbya normanbyi	Macaranga tanarius	9	0.02	0.04	0.01	0.04

0	0	0.47	0	cv	1	Licuala ramsayi	Rhoptromyrmex wroughtonii	9	0.02	0.04	0.02	0.05
0	0	0.19	0	t	1	Trichosanthes pentaphylla	Mallotus paniculatus	9	0.01	0.04	0.00	0.04
1	0	0.26	0	t	1	Polyrhachis (Cyrtomyrma) 'Cyrto 06'	Polyrhachis (Hedomyrma) cupreata	9	0.02	0.05	0.01	0.04
0	0	0.28	0	t	1	Pheidole sp.1	Pheidole aff. impressiceps	9	0.02	0.05	0.01	0.05
1	1	0.76	0	cv	1	Polyrhachis (Cyrtomyrma) 'Cyrto 06'	Crematogaster aff. fusca	9	0.02	0.05	0.06	0.04
0	0	0.33	0	t	1	Tetramorium validiusculum	Aleurites rockinghamensis	9	0.03	0.05	0.00	0.04
0	0	0.38	0	t	1	Tetramorium insolens	Licuala ramsayi	9	0.01	0.05	0.02	0.04
1	1	0.67	0	t	1	Dysoxylum papuanum	Crematogaster aff. fusca	9	0.00	0.05	0.06	0.04
0	0	0.19	0	cv	1	Trichosanthes pentaphylla	Urena lobata	9	0.01	0.04	0.00	0.04
0	1	0.67	0	t	1	Oecophylla smaragdina	Archontophoenix alexandrae	9	0.06	0.04	0.00	0.04
0	0	0.37	0	t	1	Smilax cf. australis	Polyrhachis (Cyrtomyrma) 'Cyrto NB5041	9	0.01	0.04	0.03	0.05
0	0	0.19	0	t	1	unidentified liana	Aleurites rockinghamensis	9	0.01	0.04	0.00	0.04
0	0	0.37	0	cv	1	Tetraponera nitida	Cryptocarya hypospodia	9	0.02	0.05	0.02	0.05
0	0	0.47	0	t	1	Camponotus sp.6 (gasseri gp.)	Technomyrmex albipes gp.	9	0.00	0.04	0.04	0.05
1	1	0.91	0	t	1	Polyrhachis (Myrma) foreli	Oecophylla smaragdina	9	0.04	0.04	0.06	0.04
0	0	0.30	0	t	1	Glochidion philippicum	Ficus septica	9	0.02	0.04	0.01	0.04
0	0	0.27	0	cv	1	Monomorium cf. intrudens	Macaranga tanarius	9	0.01	0.05	0.01	0.04
0	0	0.49	0	t	1	Syzygium erythrocalyx	Rhoptromyrmex wroughtonii	9	0.03	0.04	0.02	0.05
0	0	0.18	0	t	1	Syzygium cormiflorum	unidentified liana	9	0.00	0.05	0.01	0.04
0	1	0.56	0	t	1	Mucuna gigantea	Technomyrmex albipes gp.	9	0.01	0.04	0.04	0.05

0	0	0.21	0	cv	1	Mucuna gigantea	Podomyrma sp.1	9	0.01	0.04	0.00	0.04
0	0	0.19	0	t	1	Rhytidoponera spoliata	Stephania japonica	9	0.01	0.05	0.00	0.04
0	0	0.20	0	t	1	Tetramorium insolens	Polyrhachis (Myrmothrinax) delicata	9	0.01	0.05	0.00	0.05
0	0	0.20	0	t	1	Camponotus sp.1 (macrocephalus gp.)	Toona ciliata	9	0.01	0.05	0.00	0.05
0	0	0.18	0	cv	1	Trichosanthes pentaphylla	Syzygium cormiflorum	9	0.01	0.04	0.00	0.05
0	0	0.20	0	t	1	Embelia caulialata	Euodia sp.	9	0.00	0.04	0.01	0.04
0	0	0.18	0	t	1	Rhytidoponera spoliata	Polyrhachis (Myrmothrinax) delicata	9	0.01	0.05	0.00	0.05
0	0	0.22	0	t	1	Urena lobata	Macaranga tanarius	9	0.00	0.04	0.01	0.04
0	0	0.19	0	cv	1	Embelia caulialata	Rhytidoponera spoliata	9	0.00	0.04	0.01	0.05
0	1	0.62	0	t	1	Smilax cf. australis	Anonychomyrma gilberti	9	0.01	0.04	0.05	0.04
0	0	0.22	0	t	1	Dysoxylum mollissimum subsp. molle	Jasminum didymum	9	0.00	0.04	0.01	0.04
0	0	0.41	0	t	1	Syzygium erythrocalyx	Macaranga tanarius	9	0.03	0.04	0.01	0.04
0	0	0.19	0	cv	1	Rockinghamia angustifolia2	Neosepicaea jucunda	9	0.01	0.04	0.00	0.04
0	0	0.32	0	t	1	Trichosanthes pentaphylla	Camponotus spp. 'nocturnal' (novae-hollandiae gp., macrocephalus)	9	0.01	0.04	0.02	0.05
0	0	0.47	0	t	1	Monomorium floricola	Monomorium fieldi var. laeve nigrius	9	0.04	0.05	0.01	0.05
0	1	0.54	0	t	1	Clerodendrum tracyanum	Paratrechina vaga gp.	9	0.02	0.04	0.04	0.05
0	0	0.46	0	cv	1	Trichosanthes pentaphylla	Dysoxylum pettigrewianum	9	0.01	0.04	0.03	0.04
0	0	0.47	0	t	1	Monomorium floricola	Polyrhachis (Hedomyrma) cupreata	9	0.04	0.05	0.01	0.04

0	1	0.70	0	t	1	Euodia sp.	Homalanthus novoguineensis	9	0.01	0.04	0.05	0.04
0	0	0.29	0	t	1	Glochidion philippicum	Polyrhachis (Myrmhopla) mucronata	9	0.02	0.04	0.01	0.05
0	0	0.47	0	cv	1	Dysoxylum pettigrewianu	Tapinoma minutum gp.	9	0.03	0.04	0.01	0.05
0	0	0.26	0	t	1	Elaeocarpus angustifolius	unidentified liana	9	0.02	0.04	0.01	0.04
0	1	0.74	0	t	1	Merremia peltata	Macaranga tanarius	9	0.06	0.04	0.01	0.04
0	1	0.69	0	t	1	Macaranga involucrata subsp. mallotoides	Polyrhachis (Myrmhopla) mucronata	9	0.06	0.04	0.01	0.05
0	1	0.62	0	cv	1	Archontophoenix	Anonychomyrma gilberti	9	0.01	0.04	0.05	0.04
0	1	0.66	0	t	1	Mallotus paniculatus	Flagellaria indica	9	0.00	0.04	0.05	0.04
1	0	0.41	0	t	1	Paratrechina vaga gp.	Archontophoenix alexandrae	9	0.04	0.05	0.00	0.04
0	0	0.23	0	t	1	Clerodendrum tracyanum	Strumigenys guttulata	9	0.02	0.04	0.00	0.05
0	0	0.21	0	cv	1	Archontophoenix alexandrae2	Monomorium fieldi var. laeve nigrius	9	0.01	0.04	0.01	0.05
0	1	0.62	0	t	1	Flagellaria indica	Stephania japonica	9	0.05	0.04	0.00	0.04
0	0	0.21	0	t	1	Camponotus sp.6 (gasseri gp.)	Monomorium cf. intrudens	9	0.00	0.04	0.01	0.05
0	0	0.42	0	t	1	Tetramorium validiusculum	Pheidole sp.1	9	0.03	0.05	0.02	0.05
1	1	0.78	0	cv	1	Turneria bidentata	Homalanthus novoguineensis	9	0.02	0.05	0.05	0.04
0	0	0.18	0	t	1	Smilax cf. australis	Crematogaster sp.3	9	0.01	0.04	0.00	0.05
0	0	0.22	0	t	1	Dysoxylum mollissimum subsp. molle	Macaranga tanarius	9	0.00	0.04	0.01	0.04
0	0	0.40	0	t	1	Polyrhachis (Cyrtomyrma) 'Cyrto 03'	Cryptocarya hypospodia	9	0.02	0.04	0.02	0.05
0	0	0.40	0	cv	1	Licuala ramsayi	Polyrhachis (Cyrtomyrma) 'Cyrto 06'	9	0.02	0.04	0.02	0.05
0	0	0.26	0	t	1	Wrightia laevis subsp. millgar	Echinopla australis	9	0.02	0.04	0.01	0.05

0	0	0.47	0	t	1	Cardwellia sublimis	Castanospermum australe	9	0.02	0.04	0.03	0.04
0	0	0.35	0	t	1	Syzygium gustavioides	Licuala ramsayi	9	0.01	0.04	0.02	0.04
0	0	0.29	0	cv	1	Embelia caulialata	Camponotus spp. 'nocturnal' (novae-hollandiae gp., macrocephalus)	9	0.00	0.04	0.02	0.05
0	0	0.18	0	t	1	Strumigenys guttulata	Polyrhachis (Myrmhopla) mucronata	9	0.00	0.05	0.01	0.05
0	0	0.35	0	t	1	Polyrhachis (Cyrtomyrma) 'Cyrto NB5041	Monomorium fieldi var. laeve nigrius	9	0.03	0.05	0.01	0.05
0	0	0.29	0	t	1	Polyrhachis (Cyrtomyrma) 'Cyrto 03'	Odontomachus cephalotes	9	0.02	0.04	0.00	0.05
0	0	0.21	0	cv	1	Dysoxylum papuanum	Macaranga tanarius	9	0.00	0.05	0.01	0.04
0	0	0.24	0	t	1	Clerodendrum tracyanum	Podomyrma sp.1	9	0.02	0.04	0.00	0.04
0	0	0.43	0	t	1	Tetramorium validiusculum	Wrightia laevis subsp. millgar	9	0.03	0.05	0.02	0.04
1	1	0.82	0	t	1	Polyrhachis (Cyrtomyrma) 'Cyrto 03'	Crematogaster aff. fusca	9	0.02	0.04	0.06	0.04
0	1	0.72	0	cv	1	Macaranga involucrata subsp. mallotoides	Adenia heterophylla	9	0.06	0.04	0.01	0.04
0	1	0.52	0	t	1	Dysoxylum papuanum	Crematogaster aff. pythia	9	0.00	0.05	0.04	0.05
0	0	0.25	0	t	1	Cardwellia sublimis	Strumigenys guttulata	9	0.02	0.04	0.00	0.05
0	0	0.38	0	t	1	Licuala ramsayi	Mucuna gigantea	9	0.02	0.04	0.01	0.04
0	0	0.41	0	cv	1	Cryptocarya murrayi	Dysoxylum papuanum	9	0.04	0.04	0.00	0.05
0	0	0.24	0	t	1	Trichosanthes pentaphylla	Tapinoma minutum gp.	9	0.01	0.04	0.01	0.05
0	0	0.38	0	t	1	Leptomyrmex unicolor	Archontophoenix alexandrae2	9	0.03	0.04	0.01	0.04
0	1	0.67	0	t	1	Merremia peltata	Podomyrma sp.1	9	0.06	0.04	0.00	0.04

0	0	0.17	0	cv	1	Crotalaria sp.	Stephania japonica	9	0.00	0.04	0.00	0.04
0	0	0.31	0	t	1	Rockinghamia angustifolia	Polyrhachis (Myrmhopla) mucronata	9	0.02	0.04	0.01	0.05
0	0	0.18	0	t	1	Monomorium fieldi var. laeve nigrius	Stephania japonica	9	0.01	0.05	0.00	0.04
0	0	0.41	0	t	1	Glochidion philippicum	Rhoptromyrmex wroughtonii	9	0.02	0.04	0.02	0.05
0	0	0.35	0	cv	1	Mallotus mollissimus	Pheidole sp.1	9	0.02	0.04	0.02	0.05
0	0	0.33	0	t	1	Polyrhachis (Cyrtomyrma) 'Cyrto 03'	Rhytidoponera spoliata	9	0.02	0.04	0.01	0.05
0	1	0.57	0	t	1	Crematogaster aff. pythia	Pheidole aff. impressiceps	9	0.04	0.05	0.01	0.05
0	0	0.40	0	t	1	Polyrhachis (Cyrtomyrma) 'Cyrto 03'	Polyrhachis (Cyrtomyrma) 'Cyrto 06'	9	0.02	0.04	0.02	0.05
0	0	0.23	0	cv	1	Elaeocarpus angustifolius	Stephania japonica	9	0.02	0.04	0.00	0.04
0	0	0.22	0	t	1	Ficus septica	unidentified liana	9	0.01	0.04	0.01	0.04
0	0	0.28	0	t	1	Odontomachus cephalotes	Camponotus spp. 'nocturnal' (novae-hollandiae gp., macrocephalus)	9	0.00	0.05	0.02	0.05
0	0	0.22	0	t	1	Jasminum didymum	Crotalaria sp.	9	0.01	0.04	0.00	0.04
0	0	0.49	0	cv	1	Tapinoma melanocephalum	Tetraponera nitida	9	0.03	0.05	0.02	0.05
0	0	0.42	0	t	1	Neosepicaea jucunda	Dysoxylum pettigrewianum	9	0.00	0.04	0.03	0.04
0	0	0.27	0	t	1	Syzygium gustavioides	Wrightia laevis subsp. millgar	9	0.01	0.04	0.02	0.04
0	0	0.26	0	t	1	Archontophoenix	Cryptocarya hypospodia	9	0.01	0.04	0.02	0.05
0	0	0.37	0	cv	1	Smilax cf. australis	Castanospermum australe	9	0.01	0.04	0.03	0.04
0	0	0.31	0	t	1	Archontophoenix	Tetraponera nitida	9	0.01	0.04	0.02	0.05

0	0	0.41	0	t	1	Cardwellia sublimis	Camponotus spp. 'nocturnal' (novae-hollandiae gp., macrocephalus)	9	0.02	0.04	0.02	0.05
0	0	0.17	0	t	1	Urena lobata	Crematogaster sp.3	9	0.00	0.04	0.00	0.05
0	0	0.30	0	cv	1	Polyrhachis (Cyrtomyrma) 'Cyrto 03'	Ardisia pachyrrhachis2	9	0.02	0.04	0.00	0.04
0	0	0.25	0	t	1	Ficus septica	Macaranga tanarius	9	0.01	0.04	0.01	0.04
0	0	0.32	0	t	1	Polyrhachis (Cyrtomyrma) 'Cyrto 08'	Cardwellia sublimis	9	0.01	0.05	0.02	0.04
0	0	0.19	0	t	1	Dysoxylum mollissimum subsp. molle	Monomorium fieldi var. laeve nigrius	9	0.00	0.04	0.01	0.05
0	0	0.19	0	cv	1	Trichosanthes pentaphylla	Crotalaria sp.	9	0.01	0.04	0.00	0.04
0	0	0.18	0	t	1	Camponotus sp.6 (gasseri gp.)	Monomorium fieldi var. laeve nigrius	9	0.00	0.04	0.01	0.05
0	0	0.20	0	t	1	Syzygium gustavioides	Aleurites rockinghamensis	9	0.01	0.04	0.00	0.04
1	1	0.90	1	t	1	Gnamptogenys pleurodon	Cordia nodosa	10	0.04	0.06	0.07	0.06
1	1	0.91	1	cv	1	Myrmelachista sp.	Pleurothyrium sp.	10	0.04	0.06	0.07	0.06
1	1	0.90	1	t	1	Aztec sp. 9	Cordia nodosa	10	0.04	0.06	0.07	0.06
1	1	0.94	1	t	1	Aztec sp. 4	Tococa sp.	10	0.04	0.07	0.09	0.07
1	1	0.91	1	t	1	Aztec sp. 7	Triplaris poeppigiana	10	0.04	0.06	0.07	0.06
1	1	0.98	1	cv	1	Aztec sp. 1	Tococa sp.	10	0.07	0.07	0.09	0.07
1	1	0.96	1	t	1	Aztec sp. 1	Cordia nodosa	10	0.07	0.07	0.07	0.06
1	1	0.94	1	t	1	Aztec sp. 3	Tococa sp.	10	0.04	0.07	0.09	0.07
1	1	0.90	1	t	1	Allomerus demararae	Cordia nodosa	10	0.04	0.06	0.07	0.06
1	1	0.91	1	cv	1	Pachychondyla luteola	Cecropia sp.	10	0.04	0.06	0.07	0.06
1	1	0.91	1	t	1	Aztec sp. 8	Pleurothyrium sp.	10	0.04	0.06	0.07	0.06
1	1	0.91	1	t	1	Cecropia sp.	Camponotus balzani	10	0.07	0.06	0.04	0.06

0	1	0.91	1	t	1	Clidemia heterophylla	Pheidole minutula	10	0.07	0.09	0.07	0.09
1	1	0.99	1	cv	1	Clidemia heterophylla	Crematogaster victima	10	0.07	0.09	0.12	0.10
0	1	0.91	1	t	1	Pheidole minutula	Maieta guianensis	10	0.07	0.09	0.07	0.09
0	1	0.90	1	t	1	Cordia nodosa	Crematogaster limata parabiotica	10	0.07	0.06	0.04	0.06
1	1	0.84	1	t	1	Pseudomyrmex dendroicus	Triplaris americana	10	0.04	0.04	0.04	0.04
1	1	0.99	1	cv	1	Maieta guianensis	Crematogaster victima	10	0.07	0.09	0.12	0.10
1	1	0.91	1	t	1	Aztec sp. 2	Triplaris poeppigiana	10	0.04	0.06	0.07	0.06
0	1	0.90	0	t	1	Cecropia sp.	Crematogaster limata parabiotica	10	0.07	0.06	0.04	0.06
0	1	0.95	0	t	1	Cecropia sp.	Pheidole minutula	10	0.07	0.06	0.07	0.09
0	1	0.79	0	cv	1	Gnamptogenys pleurodon	Pseudomyrmex dendroicus	10	0.04	0.06	0.04	0.04
0	1	0.76	0	t	1	Myrmelachista sp.	Aztec sp. 7	10	0.04	0.06	0.04	0.06
0	1	0.76	0	t	1	Aztec sp. 8	Aztec sp. 2	10	0.04	0.06	0.04	0.06
0	1	0.89	0	t	1	Aztec sp. 4	Triplaris poeppigiana	10	0.04	0.07	0.07	0.06
0	1	0.76	0	cv	1	Aztec sp. 7	Crematogaster limata parabiotica	10	0.04	0.06	0.04	0.06
0	1	0.76	0	t	1	Myrmelachista sp.	Pachychondyla luteola	10	0.04	0.06	0.04	0.06
0	1	0.72	0	t	1	Myrmelachista sp.	Aztec sp. 4	10	0.04	0.06	0.04	0.07
0	1	0.94	0	t	1	Pheidole minutula	Cordia nodosa	10	0.07	0.09	0.07	0.06
0	1	0.86	0	cv	1	Allomerus demararae	Pheidole minutula	10	0.04	0.06	0.07	0.09
0	1	0.94	0	t	1	Maieta guianensis	Triplaris poeppigiana	10	0.07	0.09	0.07	0.06
0	1	0.84	0	t	1	Clidemia heterophylla	Aztec sp. 2	10	0.07	0.09	0.04	0.06
0	1	0.86	0	t	1	Aztec sp. 8	Maieta guianensis	10	0.04	0.06	0.07	0.09
0	1	0.76	0	cv	1	Myrmelachista sp.	Aztec sp. 2	10	0.04	0.06	0.04	0.06
0	1	0.76	0	t	1	Aztec sp. 7	Aztec sp. 2	10	0.04	0.06	0.04	0.06
0	1	0.99	0	t	1	Pleurothyrium sp.	Crematogaster victima	10	0.07	0.06	0.12	0.10

0	1	0.99	0	t	1	Cordia nodosa	Crematogaster victima	10	0.07	0.06	0.12	0.10
0	1	0.87	0	cv	1	Clidemia heterophylla	Triplaris americana	10	0.07	0.09	0.04	0.04
0	1	0.99	1	t	1	Cecropia species A	Azteca xanthochroa	11	0.05	0.12	0.14	0.12
1	1	1.00	1	t	1	Camponotus balzani	Cecropia membranacea	11	0.13	0.11	0.11	0.12
1	1	0.99	1	t	1	Camponotus balzani	Cecropia tessmannii	11	0.13	0.11	0.09	0.11
1	1	0.99	1	cv	1	Camponotus balzani	Cecropia ficifolia	11	0.13	0.11	0.09	0.12
1	1	1.00	1	t	1	Camponotus balzani	Cecropia polystachya	11	0.13	0.11	0.11	0.12
1	1	1.00	1	t	1	Azteca xanthochroa	Cecropia membranacea	11	0.14	0.12	0.11	0.12
1	1	1.00	1	t	1	Azteca xanthochroa	Cecropia engleriana	11	0.14	0.12	0.09	0.12
1	1	1.00	1	cv	1	Azteca xanthochroa	Cecropia ficifolia	11	0.14	0.12	0.09	0.12
1	1	1.00	1	t	1	Azteca xanthochroa	Cecropia polystachya	11	0.14	0.12	0.11	0.12
1	1	1.00	1	t	1	Cecropia membranacea	Azteca ovaticeps	11	0.11	0.12	0.11	0.11
0	1	0.90	1	t	1	Pachycondyla luteola	Cecropia tessmannii	11	0.05	0.10	0.09	0.11
1	1	0.99	1	cv	1	Cecropia engleriana	Azteca ovaticeps	11	0.09	0.12	0.11	0.11
1	1	1.00	1	t	1	Cecropia polystachya	Azteca ovaticeps	11	0.11	0.12	0.11	0.11
0	1	0.89	0	t	1	Pachycondyla luteola	Cecropia engleriana	11	0.05	0.10	0.09	0.12
0	1	0.98	0	t	1	Camponotus balzani	Pachycondyla luteola	11	0.13	0.11	0.05	0.10
0	1	0.99	0	cv	1	Cecropia ficifolia	Cecropia polystachya	11	0.09	0.12	0.11	0.12
1	1	0.99	0	t	1	Camponotus balzani	Cecropia engleriana	11	0.13	0.11	0.09	0.12
0	1	0.96	0	t	1	Cecropia engleriana	Cecropia tessmannii	11	0.09	0.12	0.09	0.11
0	1	0.66	0	t	1	Cecropia species A	Pachycondyla luteola	11	0.05	0.12	0.05	0.10
0	1	1.00	0	cv	1	Azteca xanthochroa	Azteca ovaticeps	11	0.14	0.12	0.11	0.11
1	1	0.99	0	t	1	Cecropia membranacea	Cecropia polystachya	11	0.11	0.12	0.11	0.12
0	1	1.00	0	t	1	Camponotus balzani	Azteca xanthochroa	11	0.13	0.11	0.14	0.12

1	1	0.99	0	t	1	Cecropia ficifolia	Azteca ovaticeps	11	0.09	0.12	0.11	0.11
0	1	0.97	0	cv	1	Cecropia tessmannii	Cecropia ficifolia	11	0.09	0.11	0.09	0.12
0	1	0.99	0	t	1	Cecropia tessmannii	Cecropia polystachya	11	0.09	0.11	0.11	0.12
0	1	0.95	0	t	1	Cecropia species A	Azteca ovaticeps	11	0.05	0.12	0.11	0.11
0	1	0.58	1	t	1	Cecropia concolor	Azteca isthmica	12	0.02	0.04	0.04	0.05
0	1	0.83	1	cv	1	Tococa	Azteca TO	12	0.08	0.06	0.02	0.06
0	1	0.83	1	t	1	Tococa bullifera	Pheidole minutula	12	0.08	0.06	0.02	0.06
1	1	0.94	1	t	1	Tococa	Azteca G	12	0.08	0.06	0.05	0.06
0	1	0.83	1	t	1	Tococa bullifera	Crematogaster C	12	0.08	0.06	0.02	0.06
1	1	0.96	1	cv	1	Tococa bullifera	Crematogaster A	12	0.08	0.06	0.06	0.06
0	1	0.90	1	t	1	Tococa bullifera	Crematogaster D	12	0.08	0.06	0.04	0.06
1	1	0.79	1	t	1	Camponotus balzanii	Cecropia purpuracens	12	0.02	0.05	0.06	0.05
0	0	0.38	1	t	1	Crematogaster B	Cordia aff. Nodosa	12	0.02	0.04	0.02	0.06
0	1	0.62	1	cv	1	Crematogaster B	Cordia nodosa	12	0.02	0.04	0.04	0.04
1	1	0.80	1	t	1	Crematogaster B	Duroia saccifera	12	0.02	0.04	0.06	0.04
1	1	0.88	1	t	1	Azteca schummani	Tachigali polyphylla	12	0.05	0.05	0.05	0.05
1	1	0.81	1	t	1	Azteca schummani	Tachigali myrmecophila	12	0.05	0.05	0.04	0.04
1	1	0.87	1	cv	1	Azteca alfari	Cecropia purpuracens	12	0.04	0.05	0.06	0.05
1	1	0.92	1	t	1	Tachigali polyphylla	Pseudomyrmex concolor	12	0.05	0.05	0.06	0.05
1	1	0.81	1	t	1	Tachigali polyphylla	Pseudomyrmex nigrescens	12	0.05	0.05	0.04	0.05
0	1	0.71	1	t	1	Solenops A	Hirtella physophora	12	0.04	0.05	0.04	0.05
1	1	0.88	1	cv	1	Solenops A	Duroia saccifera	12	0.04	0.05	0.06	0.04
0	0	0.46	1	t	1	Allomerus aff. Octoarticulata	Hirtella myrmecophila	12	0.02	0.04	0.02	0.04
0	1	0.57	1	t	1	Allomerus aff. Octoarticulata	Hirtella physophora	12	0.02	0.04	0.04	0.05
1	1	0.80	1	t	1	Allomerus aff. Octoarticulata	Duroia saccifera	12	0.02	0.04	0.06	0.04

1	1	0.87	1	cv	1	Pseudomyrmex concolor	Tachigali myrmecophila	12	0.06	0.05	0.04	0.04
1	1	0.78	1	t	1	Azteca aff. Isthmica	Cecropia ficifolia	12	0.02	0.05	0.06	0.05
1	1	0.79	1	t	1	Azteca aff. Isthmica	Cecropia purpuracens	12	0.02	0.05	0.06	0.05
1	1	0.61	1	t	1	Pouruma heterophylla	Allomerus D	12	0.02	0.02	0.02	0.02
1	1	0.78	1	cv	1	Amaioua aff. Guianensis	Azteca Q	12	0.04	0.04	0.04	0.04
0	1	0.84	1	t	1	Azteca CO	Cordia aff. Nodosa	12	0.08	0.07	0.02	0.06
1	1	0.87	1	t	1	Azteca isthmica	Cecropia ficifolia	12	0.04	0.05	0.06	0.05
1	1	0.87	1	t	1	Azteca isthmica	Cecropia purpuracens	12	0.04	0.05	0.06	0.05
1	1	0.81	1	cv	1	Azteca isthmica	Cecropia distachya	12	0.04	0.05	0.05	0.05
0	0	0.36	1	t	1	Cordia aff. Nodosa	Allomerus auripunctata	12	0.02	0.06	0.02	0.05
0	1	0.61	1	t	1	Cordia aff. Nodosa	Azteca G	12	0.02	0.06	0.05	0.06
0	0	0.35	1	t	1	Cordia aff. Nodosa	Azteca HC	12	0.02	0.06	0.02	0.05
0	0	0.48	1	cv	1	Cordia aff. Nodosa	Crematogaster D	12	0.02	0.06	0.04	0.06
0	1	0.65	1	t	1	Cordia aff. Nodosa	Crematogaster E	12	0.02	0.06	0.05	0.05
0	1	0.79	1	t	1	Allomerus auripunctata	Duroia saccifera	12	0.02	0.05	0.06	0.04
1	1	0.80	1	t	1	Allomerus prancei	Hirtella physophora	12	0.05	0.05	0.04	0.05
0	1	0.84	1	cv	1	Pheidole minutula	Maieta poeppiggi	12	0.02	0.06	0.08	0.06
0	1	0.61	1	t	1	Pheidole minutula	Maieta guianensis	12	0.02	0.06	0.05	0.06
0	1	0.82	1	t	1	Maieta poeppiggi	Crematogaster C	12	0.08	0.06	0.02	0.06
1	1	0.96	1	t	1	Maieta poeppiggi	Crematogaster A	12	0.08	0.06	0.06	0.06
1	1	0.72	1	cv	1	Tachigali myrmecophila	Pseudomyrmex nigrescens	12	0.04	0.04	0.04	0.05
0	1	0.59	1	t	1	Tachigali myrmecophila	Azteca polymorpha	12	0.04	0.04	0.02	0.04
0	1	0.59	1	t	1	Tachigali myrmecophila	Azteca D	12	0.04	0.04	0.02	0.04
0	1	0.81	1	t	1	Tachigali myrmecophila	Crematogaster E	12	0.04	0.04	0.05	0.05
0	1	0.84	1	cv	1	Hirtella physophora	Crematogaster A	12	0.04	0.05	0.06	0.06

0	1	0.83	1	t	1	Maieta guianensis	Azteca G	12	0.05	0.06	0.05	0.06
0	1	0.89	1	t	1	Maieta guianensis	Crematogaster A	12	0.05	0.06	0.06	0.06
0	1	0.83	0	t	1	Tococa bullifera	Cordia aff. Nodosa	12	0.08	0.06	0.02	0.06
0	0	0.47	0	cv	1	Pouruma heterophylla	Cordia aff. Nodosa	12	0.02	0.02	0.02	0.06
0	1	0.75	0	t	1	Camponotus balzanii	Crematogaster A	12	0.02	0.05	0.06	0.06
0	1	0.77	0	t	1	Duroia	Azteca HC	12	0.06	0.04	0.02	0.05
0	1	0.71	0	t	1	Azteca alfari	Azteca isthmica	12	0.04	0.05	0.04	0.05
0	1	0.94	0	cv	1	Cecropia ficifolia	Crematogaster A	12	0.06	0.05	0.06	0.06
0	1	0.65	0	t	1	Azteca schummani	Azteca TO	12	0.05	0.05	0.02	0.06
0	1	0.95	0	t	1	Tococa bullifera	Cecropia distachya	12	0.08	0.06	0.05	0.05
0	1	0.64	0	t	1	Cecropia distachya	Crematogaster C	12	0.05	0.05	0.02	0.06
0	0	0.45	0	cv	1	Azteca TO	Allomerus D	12	0.02	0.06	0.02	0.02
0	1	0.94	0	t	1	Tococa bullifera	Maieta guianensis	12	0.08	0.06	0.05	0.06
1	1	0.79	0	t	1	Camponotus balzanii	Cecropia ficifolia	12	0.02	0.05	0.06	0.05
0	1	0.92	0	t	1	Tococa bullifera	Azteca isthmica	12	0.08	0.06	0.04	0.05
0	1	0.57	0	cv	1	Azteca aff. Isthmica	Azteca isthmica	12	0.02	0.05	0.04	0.05
1	1	0.58	0	t	1	Azteca isthmica	Azteca polymorpha	12	0.04	0.05	0.02	0.04
0	1	0.86	0	t	1	Tococa bullifera	Allomerus auripunctata	12	0.08	0.06	0.02	0.05
0	1	0.52	0	t	1	Solenops A	Pheidole minutula	12	0.04	0.05	0.02	0.06
0	1	0.62	0	cv	1	Cordia nodosa	Azteca D	12	0.04	0.04	0.02	0.04
0	1	0.74	0	t	1	Amaioua aff. Guianensis	Hirtella physophora	12	0.04	0.04	0.04	0.05
0	1	0.86	0	t	1	Tachigali polyphylla	Azteca G	12	0.05	0.05	0.05	0.06
0	1	0.73	0	t	1	Cecropia purpuracens	Crematogaster C	12	0.06	0.05	0.02	0.06
0	1	0.68	0	cv	1	Allomerus D	Pseudomyrmex nigrescens	12	0.02	0.02	0.04	0.05
1	1	0.79	0	t	1	Crematogaster B	Cecropia ficifolia	12	0.02	0.04	0.06	0.05
0	1	0.58	0	t	1	Allomerus aff. Octoarticulata	Azteca isthmica	12	0.02	0.04	0.04	0.05
0	1	0.97	0	t	1	Azteca CO	Cecropia purpuracens	12	0.08	0.07	0.06	0.05

0	1	0.88	0	cv	1	Azteca CO	Hirtella myrmecophila	12	0.08	0.07	0.02	0.04
0	1	0.53	0	t	1	Allomerus D	Azteca polymorpha	12	0.02	0.02	0.02	0.04
0	1	0.96	0	t	1	Azteca CO	Crematogaster E	12	0.08	0.07	0.05	0.05
0	1	0.51	0	t	1	Azteca HC	Crematogaster D	12	0.02	0.05	0.04	0.06
0	1	0.55	0	cv	1	Allomerus D	Hirtella myrmecophila	12	0.02	0.02	0.02	0.04
0	1	0.95	0	t	1	Allomerus prancei	Maieta poeppiggi	12	0.05	0.05	0.08	0.06
0	1	0.59	0	t	1	Azteca Q	Azteca HC	12	0.04	0.04	0.02	0.05
0	1	0.86	0	t	1	Tococa bullifera	Camponotus balzanii	12	0.08	0.06	0.02	0.05
0	0	0.45	0	cv	1	Cordia aff. Nodosa	Allomerus D	12	0.02	0.06	0.02	0.02
0	1	0.51	0	t	1	Azteca alfari	Pheidole minutula	12	0.04	0.05	0.02	0.06
1	1	0.81	0	t	1	Azteca schummani	Azteca alfari	12	0.05	0.05	0.04	0.05
0	1	0.81	0	t	1	Allomerus prancei	Tachigali myrmecophila	12	0.05	0.05	0.04	0.04
0	1	0.90	0	cv	1	Cecropia ficifolia	Azteca G	12	0.06	0.05	0.05	0.06
1	1	0.95	0	t	1	Cecropia ficifolia	Duroia saccifera	12	0.06	0.05	0.06	0.04
0	1	0.97	0	t	1	Azteca CO	Cecropia ficifolia	12	0.08	0.07	0.06	0.05
0	1	0.64	0	t	1	Tachigali polyphylla	Cordia aff. Nodosa	12	0.05	0.05	0.02	0.06
0	1	0.88	0	cv	1	Pseudomyrmex nigrescens	Duroia saccifera	12	0.04	0.05	0.06	0.04
1	1	0.70	0	t	1	Azteca isthmica	Hirtella physophora	12	0.04	0.05	0.04	0.05
1	1	0.81	0	t	1	Tachigali polyphylla	Tachigali myrmecophila	12	0.05	0.05	0.04	0.04
0	1	0.72	0	t	1	Azteca schummani	Hirtella myrmecophila	12	0.05	0.05	0.02	0.04
0	1	0.71	0	cv	1	Cecropia concolor	Allomerus prancei	12	0.02	0.04	0.05	0.05
0	1	0.74	0	t	1	Pseudomyrmex concolor	Cordia aff. Nodosa	12	0.06	0.05	0.02	0.06
0	1	0.87	0	t	1	Cecropia ficifolia	Pseudomyrmex nigrescens	12	0.06	0.05	0.04	0.05

0	1	0.89	1	t	1	Unknown Forb N	Melanoplus desultorius	13	0.08	0.06	0.03	0.05
1	1	0.71	1	cv	1	Buchloe dactyloides	Heliaula rufa	13	0.04	0.05	0.03	0.04
0	1	0.58	1	t	1	Buchloe dactyloides	Opeia obscura	13	0.04	0.05	0.02	0.04
1	1	0.91	1	t	1	Buchloe dactyloides	Psoloessa texana	13	0.04	0.05	0.06	0.05
0	1	0.63	1	t	1	Buchloe dactyloides	Phoetaliotes nebrascensis	13	0.04	0.05	0.02	0.04
1	1	0.82	1	cv	1	Buchloe dactyloides	Arphia aberrans	13	0.04	0.05	0.04	0.04
0	1	0.52	1	t	1	Buchloe dactyloides	Leprus cyaneus	13	0.04	0.05	0.01	0.04
0	1	0.58	1	t	1	Buchloe dactyloides	Eritettix variabilis	13	0.04	0.05	0.02	0.04
0	1	0.58	1	t	1	Buchloe dactyloides	Mermiria bivittata	13	0.04	0.05	0.02	0.04
1	1	0.95	1	cv	1	Trimerotropis pallidipennis	Croton pottsii	13	0.07	0.05	0.04	0.05
1	1	0.81	1	t	1	Trimerotropis pallidipennis	Xanthocephalum texanum	13	0.07	0.05	0.01	0.05
0	1	0.76	1	t	1	Trimerotropis pallidipennis	Bouteloua gracilis	13	0.07	0.05	0.01	0.05
1	1	0.84	1	t	1	Trimerotropis pallidipennis	Ergarostis intermedia	13	0.07	0.05	0.02	0.05
1	1	0.90	1	cv	1	Trimerotropis pallidipennis	Iva ambrosiaefolia	13	0.07	0.05	0.03	0.05
1	1	0.80	1	t	1	Trimerotropis pallidipennis	Hilaria mutica	13	0.07	0.05	0.01	0.05
1	1	0.76	1	t	1	Trimerotropis pallidipennis	Polygala longa	13	0.07	0.05	0.01	0.05
1	1	0.88	1	t	1	Trimerotropis pallidipennis	Unknown Forb J	13	0.07	0.05	0.03	0.05
1	1	0.76	1	cv	1	Trimerotropis pallidipennis	Unknown Forb C	13	0.07	0.05	0.01	0.05
1	1	0.86	1	t	1	Trimerotropis pallidipennis	Sporobolus sp.	13	0.07	0.05	0.02	0.05
1	1	0.76	1	t	1	Trimerotropis pallidipennis	Unknown Forb D	13	0.07	0.05	0.01	0.05
1	1	0.88	1	t	1	Trimerotropis pallidipennis	Unknown Forb I	13	0.07	0.05	0.03	0.05
1	1	0.96	1	cv	1	Trimerotropis pallidipennis	Unknown Forb K	13	0.07	0.05	0.05	0.05
1	1	0.80	1	t	1	Trimerotropis pallidipennis	Setaria leucophila	13	0.07	0.05	0.01	0.05
1	1	0.80	1	t	1	Trimerotropis pallidipennis	Lesquerella fendleri	13	0.07	0.05	0.01	0.05
0	1	0.76	1	t	1	Trimerotropis pallidipennis	Unidentified sp.	13	0.07	0.05	0.01	0.05

1	1	0.84	1	cv	1	Trimerotropis pallidipennis	Argythamnia neomexicana	13	0.07	0.05	0.02	0.05
1	1	0.88	1	t	1	Trimerotropis pallidipennis	Cryptantha mexicana	13	0.07	0.05	0.03	0.05
1	1	0.96	1	t	1	Trimerotropis pallidipennis	Bouteloua hirsuta	13	0.07	0.05	0.05	0.05
1	1	0.84	1	t	1	Trimerotropis pallidipennis	Gilia rigidula	13	0.07	0.05	0.02	0.05
0	1	0.55	1	cv	1	Hesperotettix viridis	Dalea greggii	13	0.03	0.03	0.01	0.04
0	1	0.52	1	t	1	Hesperotettix viridis	Xanthocephalum texanum	13	0.03	0.03	0.01	0.05
0	0	0.45	1	t	1	Hesperotettix viridis	Polygala longa	13	0.03	0.03	0.01	0.05
1	1	0.75	1	t	1	Hesperotettix viridis	Zinnia grandiflora	13	0.03	0.03	0.03	0.03
0	0	0.48	1	cv	1	Hesperotettix viridis	Allionia incarnata	13	0.03	0.03	0.01	0.04
1	1	0.75	1	t	1	Hesperotettix viridis	Unknown Forb H	13	0.03	0.03	0.03	0.03
1	1	0.75	1	t	1	Hesperotettix viridis	Dyssodia acerosa	13	0.03	0.03	0.03	0.03
0	0	0.40	1	t	1	Aristida writgii	Heliaula rufa	13	0.01	0.05	0.03	0.04
0	1	0.76	1	cv	1	Heliaula rufa	Croton pottsii	13	0.03	0.04	0.04	0.05
0	0	0.46	1	t	1	Heliaula rufa	Unknown Forb G	13	0.03	0.04	0.01	0.04
0	0	0.37	1	t	1	Heliaula rufa	Bouteloua gracilis	13	0.03	0.04	0.01	0.05
0	1	0.83	1	t	1	Heliaula rufa	Bouteloua hirsuta	13	0.03	0.04	0.05	0.05
0	0	0.21	1	cv	1	Trachyrachys aspera	Bouteloua gracilis	13	0.01	0.05	0.01	0.05
0	1	0.68	1	t	1	Trachyrachys aspera	Bouteloua hirsuta	13	0.01	0.05	0.05	0.05
1	1	0.93	1	t	1	Hadrotettix trifasciatus	Croton pottsii	13	0.06	0.04	0.04	0.05
1	1	0.71	1	t	1	Hadrotettix trifasciatus	Choenopodium sp.	13	0.06	0.04	0.01	0.05
0	1	0.71	1	cv	1	Hadrotettix trifasciatus	Unknown Forb B	13	0.06	0.04	0.01	0.05
1	1	0.76	1	t	1	Hadrotettix trifasciatus	Xanthocephalum texanum	13	0.06	0.04	0.01	0.05
0	1	0.71	1	t	1	Hadrotettix trifasciatus	Bouteloua gracilis	13	0.06	0.04	0.01	0.05
1	1	0.71	1	t	1	Hadrotettix trifasciatus	Polygala longa	13	0.06	0.04	0.01	0.05
1	1	0.85	1	cv	1	Hadrotettix trifasciatus	Unknown Forb J	13	0.06	0.04	0.03	0.05
1	1	0.71	1	t	1	Hadrotettix trifasciatus	Unknown Forb C	13	0.06	0.04	0.01	0.05

1	1	0.80	1	t	1	Hadrotettix trifasciatus	Calyophus hartwegii	13	0.06	0.04	0.02	0.05
1	1	0.76	1	t	1	Hadrotettix trifasciatus	Lesquerella fendleri	13	0.06	0.04	0.01	0.05
1	1	0.80	1	cv	1	Hadrotettix trifasciatus	Argythamnia neomexicana	13	0.06	0.04	0.02	0.05
1	1	0.80	1	t	1	Hadrotettix trifasciatus	Unknown Forb M	13	0.06	0.04	0.02	0.05
1	1	0.88	1	t	1	Hadrotettix trifasciatus	Verbena wrightii	13	0.06	0.04	0.03	0.05
1	1	0.85	1	t	1	Hadrotettix trifasciatus	Cryptantha mexicana	13	0.06	0.04	0.03	0.05
1	1	0.76	1	cv	1	Hadrotettix trifasciatus	Sida filicaulis	13	0.06	0.04	0.01	0.05
1	1	0.80	1	t	1	Hadrotettix trifasciatus	Gaillardia pinnatifida	13	0.06	0.04	0.02	0.05
1	1	0.80	1	t	1	Hadrotettix trifasciatus	Gilia rigidula	13	0.06	0.04	0.02	0.05
0	0	0.20	1	t	1	Unknown Forb F	Melanoplus arizonae	13	0.01	0.04	0.01	0.04
1	1	0.51	1	cv	1	Croton pottsii	Xanthippus corallipes	13	0.04	0.05	0.01	0.04
1	1	0.71	1	t	1	Croton pottsii	Melanoplus desultorius	13	0.04	0.05	0.03	0.05
1	1	0.68	1	t	1	Croton pottsii	Phoetaliotes nebrascensis	13	0.04	0.05	0.02	0.04
1	1	0.52	1	t	1	Croton pottsii	Melanoplus arizonae	13	0.04	0.05	0.01	0.04
1	1	0.85	1	cv	1	Croton pottsii	Arphia aberrans	13	0.04	0.05	0.04	0.04
1	1	0.77	1	t	1	Croton pottsii	Acrolophitus variegatus	13	0.04	0.05	0.03	0.04
1	1	0.52	1	t	1	Croton pottsii	Melanoplus gladstoni	13	0.04	0.05	0.01	0.04
0	0	0.42	1	t	1	Dalea greggii	Melanoplus desultorius	13	0.01	0.04	0.03	0.05
0	0	0.31	1	cv	1	Unknown Forb G	Pedioscirtete s maculipennis	13	0.01	0.04	0.01	0.03
0	0	0.49	1	t	1	Unknown Forb G	Acrolophitus variegatus	13	0.01	0.04	0.03	0.04
0	0	0.38	1	t	1	Unknown Forb G	Ageneotettix deorum	13	0.01	0.04	0.02	0.04
1	0	0.36	1	t	1	Choenopodiu m sp.	Cibolacris parviceps	13	0.01	0.05	0.03	0.04
0	0	0.27	1	cv	1	Opeia obscura	Bouteloua gracilis	13	0.02	0.04	0.01	0.05
0	0	0.26	1	t	1	Opeia obscura	Unknown Forb C	13	0.02	0.04	0.01	0.05
0	1	0.50	1	t	1	Leptaloma cognata	Acrolophitus variegatus	13	0.01	0.04	0.03	0.04

1	0	0.35	1	t	1	Unknown Forb B	Cibolacris parviceps	13	0.01	0.05	0.03	0.04
0	0	0.33	1	cv	1	Unknown Forb B	Melanoplus desultorius	13	0.01	0.05	0.03	0.05
0	0	0.18	1	t	1	Unknown Forb B	Melanoplus arizonae	13	0.01	0.05	0.01	0.04
0	0	0.18	1	t	1	Unknown Forb B	Melanoplus gladstoni	13	0.01	0.05	0.01	0.04
1	1	0.51	1	t	1	Asclepius sp.	Pedioscirtetes maculipennis	13	0.03	0.03	0.01	0.03
0	0	0.39	1	cv	1	Xanthocephalum texanum	Melanoplus desultorius	13	0.01	0.05	0.03	0.05
1	0	0.36	1	t	1	Xanthocephalum texanum	Phoetaliotes nebrascensis	13	0.01	0.05	0.02	0.04
0	0	0.22	1	t	1	Xanthocephalum texanum	Melanoplus arizonae	13	0.01	0.05	0.01	0.04
0	1	0.58	1	t	1	Machaeranthera scabrella	Melanoplus arizonae	13	0.05	0.05	0.01	0.04
0	0	0.18	1	cv	1	Xanthippus corallipes	Bouteloua gracilis	13	0.01	0.04	0.01	0.05
0	0	0.37	1	t	1	Xanthippus corallipes	Iva ambrosiaefolia	13	0.01	0.04	0.03	0.05
0	0	0.18	1	t	1	Xanthippus corallipes	Polygala longa	13	0.01	0.04	0.01	0.05
0	1	0.63	1	t	1	Xanthippus corallipes	Bouteloua hirsuta	13	0.01	0.04	0.05	0.05
0	1	0.64	1	cv	1	Cibolacris parviceps	Acacia constricta	13	0.03	0.04	0.03	0.04
0	1	0.56	1	t	1	Cibolacris parviceps	Unknown Forb J	13	0.03	0.04	0.03	0.05
0	0	0.35	1	t	1	Cibolacris parviceps	Unknown Forb C	13	0.03	0.04	0.01	0.05
0	1	0.79	1	t	1	Cibolacris parviceps	Unknown Forb K	13	0.03	0.04	0.05	0.05
0	0	0.47	1	cv	1	Cibolacris parviceps	Unknown Forb M	13	0.03	0.04	0.02	0.05
0	1	0.55	1	t	1	Cibolacris parviceps	Lepidum lasiocarpum	13	0.03	0.04	0.02	0.04
0	1	0.55	1	t	1	Cibolacris parviceps	Cryptantha mexicana	13	0.03	0.04	0.03	0.05
0	0	0.46	1	t	1	Cibolacris parviceps	Gaillardia pinnatifida	13	0.03	0.04	0.02	0.05
0	0	0.46	1	cv	1	Cibolacris parviceps	Gilia rigidula	13	0.03	0.04	0.02	0.05
0	0	0.41	1	t	1	Cibolacris parviceps	Tidestromia lanuginosa	13	0.03	0.04	0.01	0.05
0	1	0.70	1	t	1	Psoloessa texana	Bouteloua gracilis	13	0.06	0.05	0.01	0.05

0	1	0.79	1	t	1	Psoloessa texana	Ergarostis intermedia	13	0.06	0.05	0.02	0.05
0	1	0.75	1	cv	1	Psoloessa texana	Muhlenbergia sp.	13	0.06	0.05	0.01	0.05
1	1	0.95	1	t	1	Psoloessa texana	Bouteloua hirsuta	13	0.06	0.05	0.05	0.05
0	0	0.32	1	t	1	Melanoplus desultorius	Polygala longa	13	0.03	0.05	0.01	0.05
0	0	0.32	1	t	1	Melanoplus desultorius	Unidentified voucher No.28	13	0.03	0.05	0.01	0.05
0	1	0.75	1	cv	1	Melanoplus desultorius	Cassia roemeriana	13	0.03	0.05	0.05	0.05
0	1	0.53	1	t	1	Melanoplus desultorius	Unknown Forb J	13	0.03	0.05	0.03	0.05
0	0	0.32	1	t	1	Melanoplus desultorius	Unknown Forb C	13	0.03	0.05	0.01	0.05
0	0	0.34	1	t	1	Melanoplus desultorius	Allionia incarnata	13	0.03	0.05	0.01	0.04
0	1	0.76	1	cv	1	Melanoplus desultorius	Unknown Forb K	13	0.03	0.05	0.05	0.05
0	1	0.51	1	t	1	Melanoplus desultorius	Unknown Forb L	13	0.03	0.05	0.02	0.04
0	0	0.32	1	t	1	Melanoplus desultorius	Convolvulus equitans	13	0.03	0.05	0.01	0.05
0	0	0.31	1	t	1	Melanoplus desultorius	Unidentified sp.	13	0.03	0.05	0.01	0.05
0	0	0.38	1	cv	1	Melanoplus desultorius	Guara coccinea	13	0.03	0.05	0.01	0.05
0	1	0.57	1	t	1	Melanoplus desultorius	Verbena wrightii	13	0.03	0.05	0.03	0.05
0	0	0.32	1	t	1	Melanoplus desultorius	Castilleja sp.	13	0.03	0.05	0.01	0.05
0	1	0.55	1	t	1	Melanoplus desultorius	Buddleja scordioides	13	0.03	0.05	0.03	0.05
0	1	0.52	1	cv	1	Melanoplus desultorius	Cryptantha mexicana	13	0.03	0.05	0.03	0.05
0	1	0.79	1	t	1	Melanoplus desultorius	Bouteloua hirsuta	13	0.03	0.05	0.05	0.05
0	0	0.43	1	t	1	Melanoplus desultorius	Gaillardia pinnatifida	13	0.03	0.05	0.02	0.05
0	0	0.39	1	t	1	Melanoplus desultorius	Tidestromia lanuginosa	13	0.03	0.05	0.01	0.05
0	0	0.30	1	cv	1	Bouteloua gracilis	Aulocara femoratum	13	0.01	0.05	0.02	0.05
0	0	0.18	1	t	1	Bouteloua gracilis	Syrbula fuscovittata	13	0.01	0.05	0.01	0.05
1	1	0.54	1	t	1	Bouteloua gracilis	Arphia aberrans	13	0.01	0.05	0.04	0.04
0	0	0.18	1	t	1	Bouteloua gracilis	Melanoplus gladstoni	13	0.01	0.05	0.01	0.04

0	0	0.30	1	cv	1	Bouteloua gracilis	Ageneotettix deorum	13	0.01	0.05	0.02	0.04
0	0	0.21	1	t	1	Bouteloua gracilis	Arphia crassa	13	0.01	0.05	0.01	0.05
0	0	0.22	1	t	1	Bouteloua gracilis	Leprus cyaneus	13	0.01	0.05	0.01	0.04
0	0	0.18	1	t	1	Bouteloua gracilis	Parapomala wyomingensi s	13	0.01	0.05	0.01	0.05
0	0	0.26	1	cv	1	Bouteloua gracilis	Eritettix variabilis	13	0.01	0.05	0.02	0.04
0	0	0.26	1	t	1	Bouteloua gracilis	Mermiria bivittata	13	0.01	0.05	0.02	0.04
0	0	0.29	1	t	1	Aulocara femoratum	Unknown Forb C	13	0.02	0.05	0.01	0.05
0	0	0.44	1	t	1	Aulocara femoratum	Sporobolus sp.	13	0.02	0.05	0.02	0.05
0	0	0.28	1	cv	1	Aulocara femoratum	Unknown Forb D	13	0.02	0.05	0.01	0.05
0	1	0.76	1	t	1	Aulocara femoratum	Bouteloua hirsuta	13	0.02	0.05	0.05	0.05
0	0	0.44	1	t	1	Pedioscirtetes maculipennis	Unknown Forb J	13	0.01	0.03	0.03	0.05
0	0	0.25	1	t	1	Pedioscirtetes maculipennis	Unknown Forb C	13	0.01	0.03	0.01	0.05
0	0	0.24	1	cv	1	Pedioscirtetes maculipennis	Unknown Forb D	13	0.01	0.03	0.01	0.05
0	0	0.42	1	t	1	Pedioscirtetes maculipennis	Unknown Forb L	13	0.01	0.03	0.02	0.04
1	0	0.40	1	t	1	Ergarostis intermedia	Phoetaliotes nebrascensis	13	0.02	0.05	0.02	0.04
1	0	0.35	1	t	1	Ergarostis intermedia	Mermiria bivittata	13	0.02	0.05	0.02	0.04
0	0	0.38	1	cv	1	Scleropogon brevifolius	Ageneotettix deorum	13	0.01	0.04	0.02	0.04
0	0	0.29	1	t	1	Scleropogon brevifolius	Leprus cyaneus	13	0.01	0.04	0.01	0.04
1	0	0.36	1	t	1	Iva ambrosiaefolia	Melanoplus gladstoni	13	0.03	0.05	0.01	0.04
0	0	0.25	1	t	1	Hilaria mutica	Arphia crassa	13	0.01	0.05	0.01	0.05
0	0	0.18	1	cv	1	Polygala longa	Melanoplus gladstoni	13	0.01	0.05	0.01	0.04
0	0	0.18	1	t	1	Unidentified voucher No.28	Melanoplus arizonae	13	0.01	0.05	0.01	0.04
0	0	0.32	1	t	1	Bouteloua curtipendula	Ageneotettix deorum	13	0.01	0.04	0.02	0.04
0	0	0.24	1	t	1	Bouteloua curtipendula	Leprus cyaneus	13	0.01	0.04	0.01	0.04
0	0	0.33	1	cv	1	Unknown Forb J	Melanoplus arizonae	13	0.03	0.05	0.01	0.04

1	0	0.31	1	t	1	Unknown Forb C	Phoetaliotes nebrascensis	13	0.01	0.05	0.02	0.04
0	0	0.18	1	t	1	Unknown Forb C	Melanoplus arizonae	13	0.01	0.05	0.01	0.04
1	1	0.54	1	t	1	Unknown Forb C	Arphia aberrans	13	0.01	0.05	0.04	0.04
0	0	0.18	1	cv	1	Unknown Forb C	Melanoplus gladstoni	13	0.01	0.05	0.01	0.04
1	0	0.45	1	t	1	Sporobolus sp.	Ageneotettix deorum	13	0.02	0.05	0.02	0.04
0	0	0.32	1	t	1	Phoetaliotes nebrascensis	Unknown voucher No. 1	13	0.02	0.04	0.01	0.04
0	0	0.17	1	t	1	Unknown Forb D	Melanoplus arizonae	13	0.01	0.05	0.01	0.04
0	0	0.20	1	cv	1	Allionia incarnata	Melanoplus arizonae	13	0.01	0.04	0.01	0.04
0	0	0.20	1	t	1	Allionia incarnata	Melanoplus gladstoni	13	0.01	0.04	0.01	0.04
0	1	0.63	1	t	1	Syrbula fuscovittata	Bouteloua hirsuta	13	0.01	0.05	0.05	0.05
1	1	0.57	1	t	1	Unknown Forb K	Melanoplus arizonae	13	0.05	0.05	0.01	0.04
1	1	0.58	1	cv	1	Unknown Forb K	Melanoplus gladstoni	13	0.05	0.05	0.01	0.04
0	1	0.64	1	t	1	Melanoplus arizonae	Bouteloua hirsuta	13	0.01	0.04	0.05	0.05
0	0	0.45	1	t	1	Acrolophitus variegatus	Lesquerella fendleri	13	0.03	0.04	0.01	0.05
0	1	0.59	1	t	1	Acrolophitus variegatus	Lepidum lasiocarpum	13	0.03	0.04	0.02	0.04
0	0	0.22	1	cv	1	Melanoplus gladstoni	Lesquerella fendleri	13	0.01	0.04	0.01	0.05
0	0	0.34	1	t	1	Melanoplus gladstoni	Cryptantha mexicana	13	0.01	0.04	0.03	0.05
0	1	0.77	1	t	1	Ageneotettix deorum	Bouteloua hirsuta	13	0.02	0.04	0.05	0.05
0	0	0.31	1	t	1	Unknown Forb M	Leprus cyaneus	13	0.02	0.05	0.01	0.04
0	1	0.68	1	cv	1	Arphia crassa	Bouteloua hirsuta	13	0.01	0.05	0.05	0.05
0	0	0.31	1	t	1	Muhlenbergia sp.	Eritettix variabilis	13	0.01	0.05	0.02	0.04
0	1	0.69	1	t	1	Leprus cyaneus	Bouteloua hirsuta	13	0.01	0.04	0.05	0.05
0	1	0.63	1	t	1	Parapomala wyomingensis	Bouteloua hirsuta	13	0.01	0.05	0.05	0.05
0	0	0.39	0	cv	1	Dalea greggii	Phoetaliotes nebrascensis	13	0.01	0.04	0.02	0.04
0	0	0.32	0	t	1	Unknown Forb L	Melanoplus gladstoni	13	0.02	0.04	0.01	0.04

0	0	0.26	0	t	1	Bouteloua curtipendula	Argythamnia neomexicana	13	0.01	0.04	0.02	0.05
0	0	0.43	0	t	1	Zinnia grandiflora	Parapomala wyomingensis	13	0.03	0.03	0.01	0.05
0	0	0.46	0	cv	1	Cibolacris parviceps	Calyophus hartwegii	13	0.03	0.04	0.02	0.05
0	1	0.85	0	t	1	Hesperotettix viridis	Unknown Forb K	13	0.03	0.03	0.05	0.05
0	0	0.37	0	t	1	Sporobolus sp.	Argythamnia neomexicana	13	0.02	0.05	0.02	0.05
0	1	0.56	0	t	1	Bouteloua curtipendula	Arphia aberrans	13	0.01	0.04	0.04	0.04
1	1	0.72	0	cv	1	Calyophus hartwegii	Bouteloua hirsuta	13	0.02	0.05	0.05	0.05
0	0	0.37	0	t	1	Cibolacris parviceps	Melanoplus arizonae	13	0.03	0.04	0.01	0.04
0	1	0.61	0	t	1	Acacia constricta	Melanoplus desultorius	13	0.03	0.04	0.03	0.05
0	1	0.61	0	t	1	Allionia incarnata	Unknown Forb K	13	0.01	0.04	0.05	0.05
0	0	0.28	0	cv	1	Xanthippus corallipes	Eritettix variabilis	13	0.01	0.04	0.02	0.04
0	1	0.58	0	t	1	Acrolophitus variegatus	Ageneotettix deorum	13	0.03	0.04	0.02	0.04
0	0	0.20	0	t	1	Castilleja sp.	Sida filicaulis	13	0.01	0.05	0.01	0.05
0	0	0.33	0	t	1	Syrbula fuscovittata	Cryptantha mexicana	13	0.01	0.05	0.03	0.05
1	1	0.78	0	cv	1	Cryptantha mexicana	Bouteloua hirsuta	13	0.03	0.05	0.05	0.05
0	0	0.30	0	t	1	Unidentified voucher No.28	Phoetaliotes nebrascensis	13	0.01	0.05	0.02	0.04
0	1	0.57	0	t	1	Hesperotettix viridis	Gilia rigidula	13	0.03	0.03	0.02	0.05
0	0	0.20	0	t	1	Allionia incarnata	Unknown voucher No. 1	13	0.01	0.04	0.01	0.04
1	0	0.35	0	cv	1	Ergarostis intermedia	Eritettix variabilis	13	0.02	0.05	0.02	0.04
0	0	0.47	0	t	1	Acacia constricta	Arphia crassa	13	0.03	0.04	0.01	0.05
0	0	0.26	0	t	1	Syrbula fuscovittata	Calyophus hartwegii	13	0.01	0.05	0.02	0.05
0	1	0.70	0	t	1	Machaeranthera scabrella	Mermiria bivittata	13	0.05	0.05	0.02	0.04
0	1	0.52	0	cv	1	Unknown Forb L	Buddleja scordioides	13	0.02	0.04	0.03	0.05
0	0	0.44	0	t	1	Hesperotettix viridis	Unidentified sp.	13	0.03	0.03	0.01	0.05
0	1	0.68	0	t	1	Machaeranthera scabrella	Gaillardia pinnatifida	13	0.05	0.05	0.02	0.05

1	0	0.25	0	t	1	Sida filicaulis	Tidestromia lanuginosa	13	0.01	0.05	0.01	0.05
0	1	0.54	0	cv	1	Iva ambrosiaefolia	Lepidum lasiocarpum	13	0.03	0.05	0.02	0.04
1	0	0.29	0	t	1	Calyophus hartwegii	Guara coccinea	13	0.02	0.05	0.01	0.05
1	0	0.40	0	t	1	Unknown Forb B	Acrolophitus variegatus	13	0.01	0.05	0.03	0.04
0	0	0.41	0	t	1	Unknown Forb J	Argythamnia neomexicana	13	0.03	0.05	0.02	0.05
0	1	0.76	0	cv	1	Unknown Forb F	Psoloessa texana	13	0.01	0.04	0.06	0.05
0	0	0.22	0	t	1	Scleropogon brevifolius	Unknown Forb D	13	0.01	0.04	0.01	0.05
0	0	0.41	0	t	1	Ageneotettix deorum	Gaillardia pinnatifida	13	0.02	0.04	0.02	0.05
0	0	0.43	0	t	1	Allionia incarnata	Acrolophitus variegatus	13	0.01	0.04	0.03	0.04
0	0	0.30	0	cv	1	Phoetaliotes nebrascensis	Castilleja sp.	13	0.02	0.04	0.01	0.05
0	1	0.62	0	t	1	Heliaula rufa	Iva ambrosiaefoli a	13	0.03	0.04	0.03	0.05
0	0	0.34	0	t	1	Calyophus hartwegii	Unknown Forb M	13	0.02	0.05	0.02	0.05
0	1	0.60	0	t	1	Cibolacris parviceps	Verbena wrightii	13	0.03	0.04	0.03	0.05
0	0	0.18	0	cv	1	Unknown Forb C	Syrbula fuscovittata	13	0.01	0.05	0.01	0.05
1	1	0.51	0	t	1	Ergarostis intermedia	Acrolophitus variegatus	13	0.02	0.05	0.03	0.04
0	0	0.33	0	t	1	Xanthocephal um texanum	Sporobolus sp.	13	0.01	0.05	0.02	0.05
0	1	0.53	0	t	1	Ergarostis intermedia	Unknown Forb H	13	0.02	0.05	0.03	0.03
0	0	0.48	0	cv	1	Zinnia grandiflora	Sida filicaulis	13	0.03	0.03	0.01	0.05
0	0	0.50	0	t	1	Unknown Forb L	Cryptantha mexicana	13	0.02	0.04	0.03	0.05
1	0	0.17	0	t	1	Polygala longa	Convolvulus equitans	13	0.01	0.05	0.01	0.05
0	0	0.39	0	t	1	Allionia incarnata	Verbena wrightii	13	0.01	0.04	0.03	0.05
0	0	0.49	0	cv	1	Ageneotettix deorum	Lepidum lasiocarpum	13	0.02	0.04	0.02	0.04
0	0	0.36	0	t	1	Unknown Forb	Sida filicaulis	13	0.03	0.05	0.01	0.05
0	0	0.47	0	t	1	Trachyrachys aspera	Acrolophitus variegatus	13	0.01	0.05	0.03	0.04
0	0	0.31	0	t	1	Sporobolus sp.	Setaria leucophila	13	0.02	0.05	0.01	0.05

0	0	0.45	0	cv	1	Bouteloua curtipendula	Dyssodia acerosa	13	0.01	0.04	0.03	0.03
1	1	0.53	0	t	1	Iva ambrosiaefolia	Phoetaliotes nebrascensis	13	0.03	0.05	0.02	0.04
0	1	0.53	0	t	1	Hesperotettix viridis	Arphia crassa	13	0.03	0.03	0.01	0.05
0	0	0.29	0	t	1	Unknown Forb B	Aulocara femoratum	13	0.01	0.05	0.02	0.05
0	0	0.39	0	cv	1	Asclepius sp.	Unidentified voucher No.28	13	0.03	0.03	0.01	0.05
0	0	0.34	0	t	1	Unknown Forb G	Opeia obscura	13	0.01	0.04	0.02	0.04
0	1	0.71	0	t	1	Leptaloma cognata	Bouteloua hirsuta	13	0.01	0.04	0.05	0.05
0	1	0.54	0	t	1	Buchloe dactyloides	Leptaloma cognata	13	0.04	0.05	0.01	0.04
0	0	0.44	0	cv	1	Unknown Forb G	Cibolacris parviceps	13	0.01	0.04	0.03	0.04
0	1	0.81	0	t	1	Machaeranthera scabrella	Acrolophitus variegatus	13	0.05	0.05	0.03	0.04
0	1	0.54	0	t	1	Buddleja scordioides	Cryptantha mexicana	13	0.03	0.05	0.03	0.05
1	0	0.41	0	t	1	Leptaloma cognata	Lepidum lasiocarpum	13	0.01	0.04	0.02	0.04
0	0	0.18	0	cv	1	Unidentified voucher No.28	Melanoplus gladstoni	13	0.01	0.05	0.01	0.04
0	0	0.39	0	t	1	Acacia constricta	Bouteloua gracilis	13	0.03	0.04	0.01	0.05
0	0	0.22	0	t	1	Allionia incarnata	Setaria leucophila	13	0.01	0.04	0.01	0.05
0	1	0.57	0	t	1	Cassia roemeriana	Allionia incarnata	13	0.05	0.05	0.01	0.04
0	0	0.19	0	cv	1	Xanthippus corallipes	Bouteloua curtipendula	13	0.01	0.04	0.01	0.04
1	0	0.20	0	t	1	Xanthocephalum texanum	Castilleja sp.	13	0.01	0.05	0.01	0.05
0	0	0.42	0	t	1	Xanthocephalum texanum	Verbena wrightii	13	0.01	0.05	0.03	0.05
0	0	0.22	0	t	1	Unknown voucher No. 1	Sida filicaulis	13	0.01	0.04	0.01	0.05
0	1	0.66	0	cv	1	Cassia roemeriana	Gaillardia pinnatifida	13	0.05	0.05	0.02	0.05
0	0	0.42	0	t	1	Lesquerella fendleri	Verbena wrightii	13	0.01	0.05	0.03	0.05
1	1	0.87	0	t	1	Croton pottsii	Unknown Forb K	13	0.04	0.05	0.05	0.05
0	1	0.64	0	t	1	Machaeranthera scabrella	Arphia crassa	13	0.05	0.05	0.01	0.05
1	1	0.54	0	cv	1	Polygala longa	Arphia aberrans	13	0.01	0.05	0.04	0.04

0	0	0.35	0	t	1	Pedioscirtetes maculipennis	Unknown Forb M	13	0.01	0.03	0.02	0.05
0	0	0.40	0	t	1	Ergarostis intermedia	Unknown Forb L	13	0.02	0.05	0.02	0.04
0	0	0.17	0	t	1	Bouteloua gracilis	Polygala longa	13	0.01	0.05	0.01	0.05
1	1	0.91	0	cv	1	Unknown Forb K	Bouteloua hirsuta	13	0.05	0.05	0.05	0.05
0	0	0.29	0	t	1	Muhlenbergia sp.	Gilia rigidula	13	0.01	0.05	0.02	0.05
1	1	0.59	0	t	1	Acacia constricta	Phoetaliotes nebrascensis	13	0.03	0.04	0.02	0.04
0	1	0.56	0	t	1	Aristida writgii	Arphia aberrans	13	0.01	0.05	0.04	0.04
0	0	0.41	0	cv	1	Acrolophitus variegatus	Melanoplus gladstoni	13	0.03	0.04	0.01	0.04
0	1	0.78	0	t	1	Trimerotropis pallidipennis	Xanthippus corallipes	13	0.07	0.05	0.01	0.04
0	0	0.29	0	t	1	Unknown Forb M	Sida filicaulis	13	0.02	0.05	0.01	0.05
0	0	0.33	0	t	1	Trachyrachys aspera	Opeia obscura	13	0.01	0.05	0.02	0.04
0	0	0.43	0	cv	1	Heliaula rufa	Lesquerella fendleri	13	0.03	0.04	0.01	0.05
0	0	0.35	0	t	1	Leptaloma cognata	Mermiria bivittata	13	0.01	0.04	0.02	0.04
0	0	0.28	0	t	1	Bouteloua gracilis	Sporobolus sp.	13	0.01	0.05	0.02	0.05
0	1	0.80	0	t	1	Leptaloma cognata	Psoloessa texana	13	0.01	0.04	0.06	0.05
0	1	0.61	0	cv	1	Machaeranthera scabrella	Setaria leucophila	13	0.05	0.05	0.01	0.05
0	1	0.68	0	t	1	Machaeranthera scabrella	Pedioscirtetes maculipennis	13	0.05	0.05	0.01	0.03
0	0	0.25	0	t	1	Choenopodium sp.	Calyophus hartwegii	13	0.01	0.05	0.02	0.05
1	1	0.90	0	t	1	Machaeranthera scabrella	Unknown Forb K	13	0.05	0.05	0.05	0.05
0	0	0.33	0	cv	1	Opeia obscura	Arphia crassa	13	0.02	0.04	0.01	0.05
0	0	0.43	0	t	1	Buchloe dactyloides	Convolvulus equitans	13	0.04	0.05	0.01	0.05
0	1	0.92	0	t	1	Unknown Forb N	Dyssodia acerosa	13	0.08	0.06	0.03	0.03
0	0	0.40	0	t	1	Asclepius sp.	Polygala longa	13	0.03	0.03	0.01	0.05
0	0	0.30	0	cv	1	Unknown Forb J	Unknown Forb D	13	0.03	0.05	0.01	0.05
1	1	0.83	0	t	1	Hadrotettix trifasciatus	Sporobolus sp.	13	0.06	0.04	0.02	0.05

0	1	0.94	0	t	1	Hadrotettix trifasciatus	Cassia roemeriana	13	0.06	0.04	0.05	0.05
0	0	0.43	0	t	1	Buchloe dactyloides	Unknown Forb D	13	0.04	0.05	0.01	0.05
0	1	0.65	0	cv	1	Hesperotettix viridis	Unknown Forb I	13	0.03	0.03	0.03	0.05
1	0	0.26	0	t	1	Unknown Forb B	Mermiria bivittata	13	0.01	0.05	0.02	0.04
0	0	0.32	0	t	1	Opeia obscura	Tidestromia lanuginosa	13	0.02	0.04	0.01	0.05
0	0	0.43	0	t	1	Melanoplus desultorius	Gilia rigidula	13	0.03	0.05	0.02	0.05
0	1	0.51	0	cv	1	Acacia constricta	Pedioscirtetes maculipennis	13	0.03	0.04	0.01	0.03
0	1	0.60	0	t	1	Syrbula fuscovittata	Unknown Forb K	13	0.01	0.05	0.05	0.05
0	1	0.61	0	t	1	Phoetaliotes nebrascensis	Unknown Forb H	13	0.02	0.04	0.03	0.03
0	0	0.31	0	t	1	Unknown Forb F	Sporobolus sp.	13	0.01	0.04	0.02	0.05
0	0	0.20	0	cv	1	Bouteloua curtipendula	Melanoplus gladstoni	13	0.01	0.04	0.01	0.04
0	0	0.24	0	t	1	Polygala longa	Argythamnia neomexicana	13	0.01	0.05	0.02	0.05
0	0	0.25	0	t	1	Aristida writgii	Gilia rigidula	13	0.01	0.05	0.02	0.05
0	0	0.29	0	t	1	Aulocara femoratum	Convolvulus equitans	13	0.02	0.05	0.01	0.05
0	0	0.34	0	cv	1	Convolvulus equitans	Buddleja scordioides	13	0.01	0.05	0.03	0.05
0	0	0.42	0	t	1	Argythamnia neomexicana	Cryptantha mexicana	13	0.02	0.05	0.03	0.05
0	0	0.33	0	t	1	Iva ambrosiaefolia	Unidentified sp.	13	0.03	0.05	0.01	0.05
0	0	0.43	0	t	1	Melanoplus desultorius	Pedioscirtetes maculipennis	13	0.03	0.05	0.01	0.03
0	1	0.56	0	cv	1	Buchloe dactyloides	Unknown Forb M	13	0.04	0.05	0.02	0.05
0	0	0.32	0	t	1	Bouteloua gracilis	Cryptantha mexicana	13	0.01	0.05	0.03	0.05
0	0	0.37	0	t	1	Cibolacris parviceps	Unknown voucher No. 1	13	0.03	0.04	0.01	0.04
0	1	0.66	0	t	1	Heliaula rufa	Asclepius sp.	13	0.03	0.04	0.03	0.03
0	0	0.41	0	cv	1	Iva ambrosiaefolia	Arphia crassa	13	0.03	0.05	0.01	0.05
0	0	0.26	0	t	1	Unknown voucher No. 1	Gilia rigidula	13	0.01	0.04	0.02	0.05
0	0	0.19	0	t	1	Unknown Forb F	Unknown Forb C	13	0.01	0.04	0.01	0.05

0	0	0.46	0	t	1	Asclepius sp.	Sida filicaulis	13	0.03	0.03	0.01	0.05
0	0	0.35	0	cv	1	Ageneotettix deorum	Muhlenbergi a sp.	13	0.02	0.04	0.01	0.05
0	1	0.66	0	t	1	Unknown Forb K	Argythamnia neomexicana	13	0.05	0.05	0.02	0.05
0	1	0.89	0	t	1	Hadrotettix trifasciatus	Acacia constricta	13	0.06	0.04	0.03	0.04
1	0	0.38	0	t	1	Hilaria mutica	Buddleja scordioides	13	0.01	0.05	0.03	0.05
0	0	0.43	0	cv	1	Unknown Forb L	Mermiria bivittata	13	0.02	0.04	0.02	0.04
1	1	0.89	0	t	1	Machaeranthe ra scabrella	Cassia roemeriana	13	0.05	0.05	0.05	0.05
0	1	0.74	0	t	1	Machaeranthe ra scabrella	Phoetaliotes nebrascensis	13	0.05	0.05	0.02	0.04
0	0	0.43	0	t	1	Opeia obscura	Aulocara femoratum	13	0.02	0.04	0.02	0.05
0	0	0.35	0	cv	1	Unknown Forb I	Setaria leucophila	13	0.03	0.05	0.01	0.05
0	1	0.82	0	t	1	Trimerotropis pallidipennis	Unknown Forb G	13	0.07	0.05	0.01	0.04
1	0	0.26	0	t	1	Choenopodiu m sp.	Eritettix variabilis	13	0.01	0.05	0.02	0.04
0	0	0.34	0	t	1	Leprus cyaneus	Mermiria bivittata	13	0.01	0.04	0.02	0.04
0	0	0.39	0	cv	1	Unknown voucher No. 1	Verbena wrightii	13	0.01	0.04	0.03	0.05
0	1	0.61	0	t	1	Unidentified voucher No.28	Bouteloua hirsuta	13	0.01	0.05	0.05	0.05
0	1	0.59	0	t	1	Phoetaliotes nebrascensis	Acrolophitus variegatus	13	0.02	0.04	0.03	0.04
1	1	0.78	0	t	1	Croton pottsii	Dyssodia acerosa	13	0.04	0.05	0.03	0.03
0	1	0.56	0	cv	1	Croton pottsii	Muhlenbergi a sp.	13	0.04	0.05	0.01	0.05
1	1	0.64	0	t	1	Croton pottsii	Opeia obscura	13	0.04	0.05	0.02	0.04
0	1	0.61	0	t	1	Croton pottsii	Gilia rigidula	13	0.04	0.05	0.02	0.05
0	0	0.42	0	t	1	Cryptantha mexicana	Gaillardia pinnatifida	13	0.03	0.05	0.02	0.05
0	0	0.35	0	cv	1	Phoetaliotes nebrascensis	Lesquerella fendleri	13	0.02	0.04	0.01	0.05
0	1	0.79	0	t	1	Asclepius sp.	Arphia aberrans	13	0.03	0.03	0.04	0.04
1	0	0.17	0	t	1	Choenopodiu m sp.	Convolvulus equitans	13	0.01	0.05	0.01	0.05
1	1	0.76	0	t	1	Unknown Forb J	Unknown Forb K	13	0.03	0.05	0.05	0.05
0	0	0.19	0	cv	1	Xanthippus corallipes	Melanoplus arizonae	13	0.01	0.04	0.01	0.04

0	0	0.36	0	t	1	Lesquerella fendleri	Lepidum lasiocarpum	13	0.01	0.05	0.02	0.04
0	0	0.26	0	t	1	Xanthippus corallipes	Pedioscirtetes maculipennis	13	0.01	0.04	0.01	0.03
0	0	0.47	0	t	1	Aulocara femoratum	Unknown Forb L	13	0.02	0.05	0.02	0.04
1	1	0.77	0	cv	1	Iva ambrosiaefolia	Cassia roemeriana	13	0.03	0.05	0.05	0.05
1	1	0.56	0	t	1	Polygala longa	Cassia roemeriana	13	0.01	0.05	0.05	0.05
0	0	0.45	0	t	1	Melanoplus gladstoni	Dyssodia acerosa	13	0.01	0.04	0.03	0.03
0	0	0.36	0	t	1	Unknown voucher No. 1	Buddleja scordioides	13	0.01	0.04	0.03	0.05
0	1	0.85	0	cv	1	Unknown Forb N	Eritettix variabilis	13	0.08	0.06	0.02	0.04
0	0	0.34	0	t	1	Melanoplus desultorius	Melanoplus arizonae	13	0.03	0.05	0.01	0.04
0	0	0.41	0	t	1	Cibolacris parviceps	Sida filicaulis	13	0.03	0.04	0.01	0.05
0	0	0.34	0	t	1	Setaria leucophila	Unknown Forb L	13	0.01	0.05	0.02	0.04
0	0	0.27	0	cv	1	Sporobolus sp.	Unknown Forb D	13	0.02	0.05	0.01	0.05
0	1	0.53	0	t	1	Leptaloma cognata	Dyssodia acerosa	13	0.01	0.04	0.03	0.03
0	1	0.67	0	t	1	Heliaula rufa	Acacia constricta	13	0.03	0.04	0.03	0.04
0	0	0.40	0	t	1	Dalea greggii	Unknown Forb I	13	0.01	0.04	0.03	0.05
0	0	0.34	0	cv	1	Aulocara femoratum	Lesquerella fendleri	13	0.02	0.05	0.01	0.05
0	0	0.33	0	t	1	Melanoplus gladstoni	Lepidum lasiocarpum	13	0.01	0.04	0.02	0.04
0	0	0.23	0	t	1	Leptaloma cognata	Bouteloua gracilis	13	0.01	0.04	0.01	0.05
0	0	0.41	0	t	1	Heliaula rufa	Unknown Forb F	13	0.03	0.04	0.01	0.04
0	0	0.29	0	cv	1	Aulocara femoratum	Castilleja sp.	13	0.02	0.05	0.01	0.05
0	0	0.25	1	t	1	Hoffmanseggia glauca	Dactylotum variegatum	14	0.01	0.04	0.01	0.04
1	1	0.66	1	t	1	Hoffmanseggia glauca	Melanoplus glaucipes	14	0.01	0.04	0.05	0.04
1	1	0.94	1	t	1	Unknown Forb O	Trimerotropis pallidipennis	14	0.06	0.05	0.05	0.05
0	1	0.65	1	cv	1	Unknown Forb O	Dactylotum variegatum	14	0.06	0.05	0.01	0.04

0	1	0.86	1	t	1	Buchloe dactyloides	Trimerotropis pallidipennis	14	0.03	0.04	0.05	0.05
1	1	0.68	1	t	1	Buchloe dactyloides	Opeia obscura	14	0.03	0.04	0.03	0.04
0	0	0.42	1	t	1	Buchloe dactyloides	Trachyrachys kiowa	14	0.03	0.04	0.01	0.04
1	1	0.87	1	cv	1	Buchloe dactyloides	Syrbula fuscovittata	14	0.03	0.04	0.05	0.04
0	1	0.65	1	t	1	Buchloe dactyloides	Psoloessa texana	14	0.03	0.04	0.03	0.04
0	1	0.65	1	t	1	Buchloe dactyloides	Aulocara femoratum	14	0.03	0.04	0.03	0.04
0	0	0.45	1	t	1	Buchloe dactyloides	Cordillacris crenulata	14	0.03	0.04	0.01	0.04
0	0	0.45	1	cv	1	Buchloe dactyloides	Philbostroma quadrimacula tum	14	0.03	0.04	0.01	0.04
0	1	0.58	1	t	1	Buchloe dactyloides	Phoetaliotes nebrascensis	14	0.03	0.04	0.02	0.05
0	0	0.50	1	t	1	Buchloe dactyloides	Melanoplus arizonae	14	0.03	0.04	0.01	0.04
0	1	0.50	1	t	1	Buchloe dactyloides	Boopedon nubilum	14	0.03	0.04	0.01	0.04
0	0	0.44	1	cv	1	Buchloe dactyloides	Ageneotettix deorum	14	0.03	0.04	0.01	0.04
0	1	0.69	1	t	1	Buchloe dactyloides	Arphia crassa	14	0.03	0.04	0.03	0.04
0	1	0.52	1	t	1	Buchloe dactyloides	Leprus cyaneus	14	0.03	0.04	0.02	0.05
0	0	0.45	1	t	1	Buchloe dactyloides	Eritettix variabilis	14	0.03	0.04	0.01	0.04
1	1	0.91	1	cv	1	Trimerotropis pallidipennis	Croton pottsii	14	0.05	0.05	0.05	0.04
0	1	0.69	1	t	1	Trimerotropis pallidipennis	Leptaloma cognata	14	0.05	0.05	0.01	0.04
1	1	0.96	1	t	1	Trimerotropis pallidipennis	Unknown Forb E	14	0.05	0.05	0.07	0.05
1	1	0.85	1	t	1	Trimerotropis pallidipennis	Bouteloua gracilis	14	0.05	0.05	0.03	0.04
1	1	0.84	1	cv	1	Trimerotropis pallidipennis	Gaura coccinea	14	0.05	0.05	0.03	0.05
0	1	0.62	1	t	1	Trimerotropis pallidipennis	Zinnia grandiflora	14	0.05	0.05	0.01	0.05
0	1	0.62	1	t	1	Trimerotropis pallidipennis	Unknown Forb N	14	0.05	0.05	0.01	0.05
0	1	0.72	1	t	1	Trimerotropis pallidipennis	Unknown Forb S	14	0.05	0.05	0.02	0.05
1	1	0.82	1	cv	1	Trimerotropis pallidipennis	Unknown Forb D	14	0.05	0.05	0.03	0.04

1	1	0.82	1	t	1	Trimerotropis pallidipennis	Convolvulus equitans	14	0.05	0.05	0.03	0.04
0	1	0.63	1	t	1	Trimerotropis pallidipennis	Iva dealbata	14	0.05	0.05	0.01	0.04
0	1	0.76	1	t	1	Trimerotropis pallidipennis	Unknown Forb R	14	0.05	0.05	0.02	0.05
0	0	0.43	1	cv	1	Xanthocephalum texanum	Melanoplus arizonae	14	0.03	0.04	0.01	0.04
0	0	0.44	1	t	1	Xanthocephalum texanum	Campylacantha olivacea	14	0.03	0.04	0.01	0.03
0	1	0.71	1	t	1	Xanthocephalum texanum	Melanoplus gladstoni	14	0.03	0.04	0.04	0.04
0	0	0.36	1	t	1	Xanthocephalum texanum	Dactylotum variegatum	14	0.03	0.04	0.01	0.04
1	1	0.77	1	cv	1	Xanthocephalum texanum	Melanoplus glaucipes	14	0.03	0.04	0.05	0.04
0	1	0.86	1	t	1	Eragrostis intermedia	Phoetaliotes nebrascensis	14	0.07	0.05	0.02	0.05
0	1	0.81	1	t	1	Eragrostis intermedia	Melanoplus lakinus	14	0.07	0.05	0.01	0.04
0	1	0.83	1	t	1	Eragrostis intermedia	Leprus cyaneus	14	0.07	0.05	0.02	0.05
0	0	0.24	1	cv	1	Unknown Forb J	Melanoplus lakinus	14	0.01	0.04	0.01	0.04
0	0	0.35	1	t	1	Unknown Forb C	Melanoplus desultorius	14	0.02	0.04	0.01	0.04
0	0	0.42	1	t	1	Unknown Forb C	Phoetaliotes nebrascensis	14	0.02	0.04	0.02	0.05
0	0	0.34	1	t	1	Unknown Forb C	Melanoplus arizonae	14	0.02	0.04	0.01	0.04
0	0	0.33	1	cv	1	Unknown Forb C	Melanoplus lakinus	14	0.02	0.04	0.01	0.04
0	0	0.36	1	t	1	Unknown Forb C	Campylacantha olivacea	14	0.02	0.04	0.01	0.03
0	1	0.63	1	t	1	Unknown Forb C	Melanoplus gladstoni	14	0.02	0.04	0.04	0.04
0	0	0.28	1	t	1	Unknown Forb C	Dactylotum variegatum	14	0.02	0.04	0.01	0.04
1	1	0.70	1	cv	1	Unidentified voucher No.28	Melanoplus desultorius	14	0.05	0.04	0.01	0.04
1	1	0.69	1	t	1	Unidentified voucher No.28	Melanoplus arizonae	14	0.05	0.04	0.01	0.04
0	1	0.68	1	t	1	Unidentified voucher No.28	Melanoplus lakinus	14	0.05	0.04	0.01	0.04
1	1	0.88	1	t	1	Unidentified voucher No.28	Melanoplus gladstoni	14	0.05	0.04	0.04	0.04
1	1	0.91	1	cv	1	Unidentified voucher No.28	Melanoplus glaucipes	14	0.05	0.04	0.05	0.04
1	1	0.50	1	t	1	Unknown Forb F	Melanoplus desultorius	14	0.03	0.03	0.01	0.04

1	0	0.45	1	t	1	Unknown Forb F	Cordillacris crenulata	14	0.03	0.03	0.01	0.04
0	0	0.43	1	t	1	Unknown Forb F	Dactylotum variegatum	14	0.03	0.03	0.01	0.04
1	1	0.79	1	cv	1	Croton pottsii	Opeia obscura	14	0.05	0.04	0.03	0.04
0	1	0.64	1	t	1	Croton pottsii	Melanoplus desultorius	14	0.05	0.04	0.01	0.04
0	1	0.58	1	t	1	Croton pottsii	Cordillacris crenulata	14	0.05	0.04	0.01	0.04
0	1	0.70	1	t	1	Croton pottsii	Phoetaliotes nebrascensis	14	0.05	0.04	0.02	0.05
0	1	0.63	1	cv	1	Croton pottsii	Melanoplus arizonae	14	0.05	0.04	0.01	0.04
0	1	0.61	1	t	1	Croton pottsii	Melanoplus lakinus	14	0.05	0.04	0.01	0.04
1	1	0.85	1	t	1	Croton pottsii	Melanoplus gladstoni	14	0.05	0.04	0.04	0.04
0	1	0.57	1	t	1	Croton pottsii	Ageneotettix deorum	14	0.05	0.04	0.01	0.04
0	1	0.65	1	cv	1	Croton pottsii	Leprus cyaneus	14	0.05	0.04	0.02	0.05
0	1	0.56	1	t	1	Croton pottsii	Dactylotum variegatum	14	0.05	0.04	0.01	0.04
0	1	0.57	1	t	1	Croton pottsii	Tropidolophus formosus	14	0.05	0.04	0.01	0.04
1	1	0.88	1	t	1	Croton pottsii	Melanoplus glaucipes	14	0.05	0.04	0.05	0.04
0	0	0.47	1	cv	1	Opeia obscura	Leptaloma cognata	14	0.03	0.04	0.01	0.04
1	1	0.69	1	t	1	Opeia obscura	Bouteloua gracilis	14	0.03	0.04	0.03	0.04
0	1	0.66	1	t	1	Opeia obscura	Muhlenbergia sp.	14	0.03	0.04	0.03	0.04
0	0	0.43	1	t	1	Opeia obscura	Aristida hamulosa	14	0.03	0.04	0.01	0.03
0	0	0.24	1	cv	1	Leptaloma cognata	Trachyrachys kiowa	14	0.01	0.04	0.01	0.04
0	0	0.44	1	t	1	Leptaloma cognata	Psoloessa texana	14	0.01	0.04	0.03	0.04
0	0	0.44	1	t	1	Leptaloma cognata	Aulocara femoratum	14	0.01	0.04	0.03	0.04
0	0	0.26	1	t	1	Leptaloma cognata	Cordillacris crenulata	14	0.01	0.04	0.01	0.04
0	0	0.26	1	cv	1	Leptaloma cognata	Philbostroma quadrimaculatum	14	0.01	0.04	0.01	0.04
0	0	0.30	1	t	1	Leptaloma cognata	Boopedon nubilum	14	0.01	0.04	0.01	0.04
0	0	0.25	1	t	1	Leptaloma cognata	Ageneotettix deorum	14	0.01	0.04	0.01	0.04

0	0	0.48	1	t	1	Leptaloma cognata	Arphia crassa	14	0.01	0.04	0.03	0.04
0	0	0.32	1	cv	1	Leptaloma cognata	Leprus cyaneus	14	0.01	0.04	0.02	0.05
0	0	0.43	1	t	1	Unknown Forb B	Melanoplus arizonae	14	0.03	0.04	0.01	0.04
0	0	0.41	1	t	1	Unknown Forb B	Melanoplus lakinus	14	0.03	0.04	0.01	0.04
0	0	0.44	1	t	1	Unknown Forb B	Campylacantha olivacea	14	0.03	0.04	0.01	0.03
0	1	0.71	1	cv	1	Unknown Forb B	Melanoplus gladstoni	14	0.03	0.04	0.04	0.04
0	1	0.76	1	t	1	Trachyrachys kiowa	Unknown Forb E	14	0.01	0.04	0.07	0.05
0	0	0.44	1	t	1	Trachyrachys kiowa	Bouteloua gracilis	14	0.01	0.04	0.03	0.04
0	0	0.20	1	t	1	Trachyrachys kiowa	Scleropogon brevifolius	14	0.01	0.04	0.01	0.04
0	1	0.63	1	cv	1	Trachyrachys kiowa	Setaria leucophila	14	0.01	0.04	0.05	0.04
0	0	0.47	1	t	1	Machaeranthera scabrella	Melanoplus lakinus	14	0.03	0.04	0.01	0.04
0	0	0.41	1	t	1	Machaeranthera scabrella	Dactylotum variegatum	14	0.03	0.04	0.01	0.04
1	1	0.86	1	t	1	Syrbula fuscovittata	Bouteloua gracilis	14	0.05	0.04	0.03	0.04
1	1	0.67	1	cv	1	Syrbula fuscovittata	Scleropogon brevifolius	14	0.05	0.04	0.01	0.04
1	1	0.76	1	t	1	Syrbula fuscovittata	Unknown Forb Q	14	0.05	0.04	0.02	0.04
1	1	0.93	1	t	1	Unknown Forb E	Melanoplus gladstoni	14	0.07	0.05	0.04	0.04
0	1	0.74	1	t	1	Unknown Forb E	Dactylotum variegatum	14	0.07	0.05	0.01	0.04
0	1	0.66	1	cv	1	Psoloessa texana	Bouteloua gracilis	14	0.03	0.04	0.03	0.04
0	0	0.38	1	t	1	Psoloessa texana	Scleropogon brevifolius	14	0.03	0.04	0.01	0.04
1	1	0.57	1	t	1	Psoloessa texana	Unknown Forb A	14	0.03	0.04	0.02	0.03
0	1	0.63	1	t	1	Psoloessa texana	Sporobolus sp.	14	0.03	0.04	0.03	0.04
0	1	0.52	1	cv	1	Melanoplus desultorius	Bouteloua gracilis	14	0.01	0.04	0.03	0.04
0	1	0.52	1	t	1	Melanoplus desultorius	Polygala longa	14	0.01	0.04	0.03	0.03
0	0	0.38	1	t	1	Melanoplus desultorius	Unknown Forb H	14	0.01	0.04	0.02	0.03
0	0	0.25	1	t	1	Melanoplus desultorius	Zinnia grandiflora	14	0.01	0.04	0.01	0.05

0	0	0.26	1	cv	1	Melanoplus desultorius	Conyza coulteri	14	0.01	0.04	0.01	0.04
0	0	0.47	1	t	1	Melanoplus desultorius	Gilia rigida	14	0.01	0.04	0.03	0.03
0	0	0.26	1	t	1	Melanoplus desultorius	Ratibida columnaris	14	0.01	0.04	0.01	0.04
0	0	0.48	1	t	1	Melanoplus desultorius	Unknown Forb D	14	0.01	0.04	0.03	0.04
0	0	0.28	1	cv	1	Melanoplus desultorius	Unknown Forb M	14	0.01	0.04	0.01	0.03
0	0	0.47	1	t	1	Melanoplus desultorius	Convolvulus equitans	14	0.01	0.04	0.03	0.04
0	1	0.65	1	t	1	Bouteloua gracilis	Aulocara femoratum	14	0.03	0.04	0.03	0.04
0	0	0.45	1	t	1	Bouteloua gracilis	Cordillacris crenulata	14	0.03	0.04	0.01	0.04
0	0	0.45	1	cv	1	Bouteloua gracilis	Philbostroma quadrimaculatum	14	0.03	0.04	0.01	0.04
0	1	0.58	1	t	1	Bouteloua gracilis	Phoetaliotes nebrascensis	14	0.03	0.04	0.02	0.05
0	0	0.49	1	t	1	Bouteloua gracilis	Melanoplus lakinus	14	0.03	0.04	0.01	0.04
0	1	0.50	1	t	1	Bouteloua gracilis	Boopedon nubilum	14	0.03	0.04	0.01	0.04
1	1	0.77	1	cv	1	Bouteloua gracilis	Melanoplus gladstoni	14	0.03	0.04	0.04	0.04
0	0	0.44	1	t	1	Bouteloua gracilis	Ageneotettix deorum	14	0.03	0.04	0.01	0.04
0	1	0.69	1	t	1	Bouteloua gracilis	Arphia crassa	14	0.03	0.04	0.03	0.04
0	1	0.52	1	t	1	Bouteloua gracilis	Leprus cyaneus	14	0.03	0.04	0.02	0.05
0	0	0.43	1	cv	1	Bouteloua gracilis	Dactylotum variegatum	14	0.03	0.04	0.01	0.04
0	0	0.45	1	t	1	Bouteloua gracilis	Eritettix variabilis	14	0.03	0.04	0.01	0.04
1	1	0.82	1	t	1	Bouteloua gracilis	Melanoplus glaucipes	14	0.03	0.04	0.05	0.04
0	0	0.38	1	t	1	Aulocara femoratum	Scleropogon brevifolius	14	0.03	0.04	0.01	0.04
1	1	0.76	1	cv	1	Aulocara femoratum	Bouteloua hirsuta	14	0.03	0.04	0.04	0.03
0	0	0.41	1	t	1	Gaura coccinea	Dactylotum variegatum	14	0.03	0.05	0.01	0.04
0	0	0.35	1	t	1	Cordillacris crenulata	Linum rigidum	14	0.01	0.04	0.02	0.04
1	1	0.60	1	t	1	Cordillacris crenulata	Bouteloua hirsuta	14	0.01	0.04	0.04	0.03

0	0	0.22	1	cv	1	Philbostroma quadrimaculatum	Scleropogon brevifolius	14	0.01	0.04	0.01	0.04
0	0	0.44	1	t	1	Philbostroma quadrimaculatum	Muhlenbergia sp.	14	0.01	0.04	0.03	0.04
0	0	0.26	1	t	1	Scleropogon brevifolius	Boopedon nubilum	14	0.01	0.04	0.01	0.04
0	0	0.27	1	t	1	Scleropogon brevifolius	Leprus cyaneus	14	0.01	0.04	0.02	0.05
0	0	0.36	1	cv	1	Panicum obtusum	Phoetaliotes nebrascensis	14	0.01	0.04	0.02	0.05
0	0	0.31	1	t	1	Unknown Forb H	Dactylotum variegatum	14	0.02	0.03	0.01	0.04
0	1	0.52	1	t	1	Conyza coulteri	Melanoplus gladstoni	14	0.01	0.04	0.04	0.04
0	0	0.20	1	t	1	Conyza coulteri	Dactylotum variegatum	14	0.01	0.04	0.01	0.04
0	1	0.50	1	cv	1	Bouteloua curtipendula	Melanoplus lakinus	14	0.03	0.04	0.01	0.04
1	0	0.46	1	t	1	Xanthocephalum sp.	Campylacantha olivacea	14	0.02	0.02	0.01	0.03
0	1	0.74	1	t	1	Sporobolus sp.	Melanoplus gladstoni	14	0.03	0.04	0.04	0.04
0	0	0.40	1	t	1	Sporobolus sp.	Dactylotum variegatum	14	0.03	0.04	0.01	0.04
0	1	0.54	1	cv	1	Phoetaliotes nebrascensis	Unknown Forb D	14	0.02	0.05	0.03	0.04
0	0	0.47	1	t	1	Phoetaliotes nebrascensis	Linum rigidum	14	0.02	0.05	0.02	0.04
0	1	0.56	1	t	1	Phoetaliotes nebrascensis	Muhlenbergia sp.	14	0.02	0.05	0.03	0.04
0	0	0.30	1	t	1	Phoetaliotes nebrascensis	Iva dealbata	14	0.02	0.05	0.01	0.04
0	1	0.67	1	cv	1	Prosopis glandulosa	Melanoplus gladstoni	14	0.02	0.04	0.04	0.04
0	0	0.29	1	t	1	Unknown Forb G	Melanoplus lakinus	14	0.01	0.04	0.01	0.04
0	0	0.31	1	t	1	Unknown Forb G	Campylacantha olivacea	14	0.01	0.04	0.01	0.03
0	1	0.58	1	t	1	Unknown Forb G	Melanoplus gladstoni	14	0.01	0.04	0.04	0.04
0	0	0.25	1	cv	1	Unknown Forb G	Ageneotettix deorum	14	0.01	0.04	0.01	0.04
0	0	0.29	1	t	1	Ziziphus obtusifolius	Campylacantha olivacea	14	0.01	0.03	0.01	0.03
0	0	0.22	1	t	1	Ziziphus obtusifolius	Dactylotum variegatum	14	0.01	0.03	0.01	0.04
0	0	0.24	1	t	1	Ziziphus obtusifolius	Eritettix variabilis	14	0.01	0.03	0.01	0.04

0	1	0.59	1	cv	1	Oenothera sp.	Campylacantha olivacea	14	0.04	0.03	0.01	0.03
0	1	0.67	1	t	1	Setaria leucophila	Boopedon nubilum	14	0.05	0.04	0.01	0.04
0	0	0.46	1	t	1	Melanoplus arizonae	Convolvulus equitans	14	0.01	0.04	0.03	0.04
0	0	0.24	1	t	1	Melanoplus arizonae	Iva dealbata	14	0.01	0.04	0.01	0.04
0	0	0.45	1	cv	1	Melanoplus lakinus	Convolvulus equitans	14	0.01	0.04	0.03	0.04
0	0	0.23	1	t	1	Melanoplus lakinus	Iva dealbata	14	0.01	0.04	0.01	0.04
0	0	0.24	1	t	1	Melanoplus lakinus	Cryptantha mexicana	14	0.01	0.04	0.01	0.04
0	0	0.48	1	t	1	Campylacantha olivacea	Unknown Forb D	14	0.01	0.03	0.03	0.04
0	0	0.26	1	cv	1	Campylacantha olivacea	Iva dealbata	14	0.01	0.03	0.01	0.04
0	0	0.21	1	t	1	Calyophus hartwegii	Dactylotum variegatum	14	0.01	0.04	0.01	0.04
0	1	0.60	1	t	1	Calyophus hartwegii	Melanoplus glaucipes	14	0.01	0.04	0.05	0.04
0	1	0.54	1	t	1	Unknown Forb Q	Arphia crassa	14	0.02	0.04	0.03	0.04
0	1	0.61	1	cv	1	Unknown Forb P	Dactylotum variegatum	14	0.05	0.05	0.01	0.04
0	1	0.64	1	t	1	Boopedon nubilum	Bouteloua hirsuta	14	0.01	0.04	0.04	0.03
1	1	0.66	1	t	1	Melanoplus gladstoni	Eupatorium greggii	14	0.04	0.04	0.02	0.04
0	1	0.50	1	t	1	Melanoplus gladstoni	Ratibida columnaris	14	0.04	0.04	0.01	0.04
1	1	0.72	1	cv	1	Melanoplus gladstoni	Convolvulus equitans	14	0.04	0.04	0.03	0.04
0	0	0.39	1	t	1	Gilia rigida	Dactylotum variegatum	14	0.03	0.03	0.01	0.04
0	0	0.21	1	t	1	Ratibida columnaris	Dactylotum variegatum	14	0.01	0.04	0.01	0.04
0	1	0.60	1	t	1	Ratibida columnaris	Melanoplus glaucipes	14	0.01	0.04	0.05	0.04
0	0	0.39	1	cv	1	Unknown Forb D	Dactylotum variegatum	14	0.03	0.04	0.01	0.04
1	1	0.79	1	t	1	Unknown Forb D	Melanoplus glaucipes	14	0.03	0.04	0.05	0.04
0	0	0.35	1	t	1	Linum rigidum	Eritettix variabilis	14	0.02	0.04	0.01	0.04
1	1	0.79	1	t	1	Arphia crassa	Bouteloua hirsuta	14	0.03	0.04	0.04	0.03
0	1	0.53	1	cv	1	Arphia crassa	Condalia ericoides	14	0.03	0.04	0.02	0.04

1	1	0.70	1	t	1	Verbena wrightii	Melanoplus glaucipes	14	0.02	0.04	0.05	0.04
0	0	0.43	1	t	1	Muhlenbergia sp.	Eritettix variabilis	14	0.03	0.04	0.01	0.04
0	0	0.38	1	t	1	Convolvulus equitans	Dactylotum variegatum	14	0.03	0.04	0.01	0.04
0	0	0.20	1	cv	1	Dactylotum variegatum	Iva dealbata	14	0.01	0.04	0.01	0.04
0	0	0.29	1	t	1	Dactylotum variegatum	Condalia ericoides	14	0.01	0.04	0.02	0.04
0	0	0.21	1	t	1	Iva dealbata	Eritettix variabilis	14	0.01	0.04	0.01	0.04
1	1	0.66	1	t	1	Dychoriste linearis	Melanoplus glaucipes	14	0.01	0.04	0.05	0.04
1	1	0.70	1	cv	1	Condalia ericoides	Melanoplus glaucipes	14	0.02	0.04	0.05	0.04
0	1	0.58	0	t	1	Machaeranthera scabrella	Prosopis glandulosa	14	0.03	0.04	0.02	0.04
0	0	0.39	0	t	1	Zinnia grandiflora	Convolvulus equitans	14	0.01	0.05	0.03	0.04
0	0	0.35	0	t	1	Unknown Forb B	Unknown Forb N	14	0.03	0.04	0.01	0.05
0	1	0.56	0	cv	1	Machaeranthera scabrella	Unknown Forb R	14	0.03	0.04	0.02	0.05
0	1	0.62	0	t	1	Setaria leucophila	Cryptantha mexicana	14	0.05	0.04	0.01	0.04
0	0	0.41	0	t	1	Scleropogon brevifolius	Convolvulus equitans	14	0.01	0.04	0.03	0.04
0	1	0.63	0	t	1	Unknown Forb D	Convolvulus equitans	14	0.03	0.04	0.03	0.04
0	1	0.57	0	cv	1	Croton pottsii	Calyophus hartwegii	14	0.05	0.04	0.01	0.04
0	1	0.79	0	t	1	Unknown Forb O	Linum rigidum	14	0.06	0.05	0.02	0.04
0	0	0.35	0	t	1	Xanthocephalum texanum	Unknown Forb N	14	0.03	0.04	0.01	0.05
0	1	0.66	0	t	1	Calyophus hartwegii	Unknown Forb P	14	0.01	0.04	0.05	0.05
1	1	0.86	0	cv	1	Unknown Forb O	Buchloe dactyloides	14	0.06	0.05	0.03	0.04
0	1	0.66	0	t	1	Unidentified voucher No.28	Ziziphus obtusifolius	14	0.05	0.04	0.01	0.03
0	0	0.32	0	t	1	Conyza coulteri	Phoetaliotes nebrascensis	14	0.01	0.04	0.02	0.05
0	0	0.25	0	t	1	Unknown Forb G	Calyophus hartwegii	14	0.01	0.04	0.01	0.04
1	1	0.88	0	cv	1	Unidentified voucher No.28	Oenothera sp.	14	0.05	0.04	0.04	0.03
0	1	0.50	0	t	1	Polygala longa	Boopedon nubilum	14	0.03	0.03	0.01	0.04

0	0	0.46	0	t	1	Melanoplus arizonae	Gilia rigida	14	0.01	0.04	0.03	0.03
0	0	0.49	0	t	1	Prosopis glandulosa	Linum rigidum	14	0.02	0.04	0.02	0.04
0	0	0.31	0	cv	1	Unknown Forb J	Unknown Forb R	14	0.01	0.04	0.02	0.05
0	1	0.53	0	t	1	Oenothera sp.	Aristida hamulosa	14	0.04	0.03	0.01	0.03
0	1	0.75	0	t	1	Croton pottsii	Unknown Forb B	14	0.05	0.04	0.03	0.04
0	1	0.50	0	t	1	Unknown Forb A	Phoetaliotes nebrascensis	14	0.02	0.03	0.02	0.05
0	1	0.52	0	cv	1	Bouteloua curtipendula	Boopedon nubilum	14	0.03	0.04	0.01	0.04
0	1	0.66	0	t	1	Opeia obscura	Arphia crassa	14	0.03	0.04	0.03	0.04
1	1	0.83	0	t	1	Syrbula fuscovittata	Convolvulus equitans	14	0.05	0.04	0.03	0.04
0	0	0.30	0	t	1	Unknown Forb C	Aristida hamulosa	14	0.02	0.04	0.01	0.03
0	0	0.20	0	cv	1	Zinnia grandiflora	Ratibida columnaris	14	0.01	0.05	0.01	0.04
0	1	0.56	0	t	1	Unknown Forb C	Polygala longa	14	0.02	0.04	0.03	0.03
0	0	0.37	0	t	1	Verbena wrightii	Leprus cyaneus	14	0.02	0.04	0.02	0.05
0	0	0.29	0	t	1	Verbena wrightii	Tropidolophus formosus	14	0.02	0.04	0.01	0.04
0	1	0.57	0	cv	1	Unknown Forb H	Muhlenbergia sp.	14	0.02	0.03	0.03	0.04
0	1	0.51	0	t	1	Leptaloma cognata	Polygala longa	14	0.01	0.04	0.03	0.03
0	0	0.35	0	t	1	Philbostroma quadrimaculatum	Linum rigidum	14	0.01	0.04	0.02	0.04
0	1	0.58	0	t	1	Cryptantha mexicana	Bouteloua hirsuta	14	0.01	0.04	0.04	0.03
0	1	0.70	0	cv	1	Croton pottsii	Unknown Forb H	14	0.05	0.04	0.02	0.03
0	0	0.40	0	t	1	Unknown Forb B	Unknown Forb M	14	0.03	0.04	0.01	0.03
0	1	0.60	0	t	1	Tropidolophus formosus	Melanoplus glaucipes	14	0.01	0.04	0.05	0.04
0	0	0.26	0	t	1	Philbostroma quadrimaculatum	Dychoriste linearis	14	0.01	0.04	0.01	0.04
0	1	0.72	0	cv	1	Psoloessa texana	Oenothera sp.	14	0.03	0.04	0.04	0.03
0	0	0.31	0	t	1	Phoetaliotes nebrascensis	Cryptantha mexicana	14	0.02	0.05	0.01	0.04
0	1	0.91	0	t	1	Eragrostis intermedia	Opeia obscura	14	0.07	0.05	0.03	0.04

0	1	0.84	0	t	1	Bouteloua gracilis	Setaria leucophila	14	0.03	0.04	0.05	0.04
0	0	0.31	0	cv	1	Phoetaliotes nebrascensis	Ageneotettix deorum	14	0.02	0.05	0.01	0.04
0	1	0.56	0	t	1	Melanoplus lakinus	Melanoplus gladstoni	14	0.01	0.04	0.04	0.04
0	0	0.27	0	t	1	Philbostroma quadrimaculat um	Campylacant ha olivacea	14	0.01	0.04	0.01	0.03
0	0	0.32	0	t	1	Ageneotettix deorum	Unknown Forb R	14	0.01	0.04	0.02	0.05
0	1	0.80	0	cv	1	Unknown Forb E	Campylacant ha olivacea	14	0.07	0.05	0.01	0.03
0	1	0.85	0	t	1	Polygala longa	Unknown Forb P	14	0.03	0.03	0.05	0.05
0	0	0.44	0	t	1	Unknown Forb J	Unknown Forb F	14	0.01	0.04	0.03	0.03
0	1	0.58	0	t	1	Unknown Forb C	Bouteloua curtipendula	14	0.02	0.04	0.03	0.04
0	0	0.46	0	cv	1	Psoloessa texana	Leprus cyaneus	14	0.03	0.04	0.02	0.05
0	1	0.60	0	t	1	Campylacanth a olivacea	Melanoplus gladstoni	14	0.01	0.03	0.04	0.04
0	1	0.62	0	t	1	Xanthocephal um sp.	Unknown Forb D	14	0.02	0.02	0.03	0.04
0	0	0.25	0	t	1	Unknown Forb J	Melanoplus arizonae	14	0.01	0.04	0.01	0.04
0	0	0.36	0	cv	1	Unknown Forb J	Unknown Forb A	14	0.01	0.04	0.02	0.03
0	1	0.65	0	t	1	Unknown Forb O	Cryptantha mexicana	14	0.06	0.05	0.01	0.04
0	0	0.45	0	t	1	Xanthocephal um texanum	Leprus cyaneus	14	0.03	0.04	0.02	0.05
0	1	0.59	0	t	1	Melanoplus gladstoni	Unknown Forb S	14	0.04	0.04	0.02	0.05
1	1	0.69	0	cv	1	Unidentified voucher No.28	Boopedon nubilum	14	0.05	0.04	0.01	0.04
1	1	0.81	0	t	1	Polygala longa	Bouteloua hirsuta	14	0.03	0.03	0.04	0.03
1	0	0.48	0	t	1	Unknown Forb A	Condalia ericoides	14	0.02	0.03	0.02	0.04
0	1	0.84	0	t	1	Unknown Forb O	Opeia obscura	14	0.06	0.05	0.03	0.04
0	0	0.28	0	cv	1	Unknown Forb Q	Unknown Forb N	14	0.02	0.04	0.01	0.05
0	0	0.30	0	t	1	Verbena wrightii	Eritettix variabilis	14	0.02	0.04	0.01	0.04
0	0	0.23	0	t	1	Conyza coulteri	Unknown Forb M	14	0.01	0.04	0.01	0.03
0	0	0.47	0	t	1	Unknown Forb J	Bouteloua curtipendula	14	0.01	0.04	0.03	0.04

1	1	0.96	0	cv	1	Unidentified voucher No.28	Unknown Forb E	14	0.05	0.04	0.07	0.05
0	0	0.42	0	t	1	Muhlenbergia sp.	Cryptantha mexicana	14	0.03	0.04	0.01	0.04
0	1	0.93	0	t	1	Buchloe dactyloides	Eragrostis intermedia	14	0.03	0.04	0.07	0.05
0	0	0.37	0	t	1	Unknown Forb H	Dychoriste linearis	14	0.02	0.03	0.01	0.04
0	1	0.61	0	cv	1	Setaria leucophila	Iva dealbata	14	0.05	0.04	0.01	0.04
0	1	0.65	0	t	1	Croton pottsii	Unknown Forb S	14	0.05	0.04	0.02	0.05
0	1	0.64	0	t	1	Melanoplus arizonae	Bouteloua hirsuta	14	0.01	0.04	0.04	0.03
0	1	0.69	0	t	1	Bouteloua gracilis	Muhlenbergi a sp.	14	0.03	0.04	0.03	0.04
0	0	0.35	0	cv	1	Boopedon nubilum	Verbena wrightii	14	0.01	0.04	0.02	0.04
0	0	0.45	0	t	1	Panicum obtusum	Convolvulus equitans	14	0.01	0.04	0.03	0.04
0	0	0.48	0	t	1	Gaura coccinea	Dychoriste linearis	14	0.03	0.05	0.01	0.04
0	0	0.40	0	t	1	Campylacanth a olivacea	Eupatorium greggii	14	0.01	0.03	0.02	0.04
0	1	0.59	0	cv	1	Unknown Forb F	Prosopis glandulosa	14	0.03	0.03	0.02	0.04
0	0	0.19	0	t	1	Unknown Forb N	Tropidolophu s formosus	14	0.01	0.05	0.01	0.04
0	1	0.65	0	t	1	Unknown Forb D	Arphia crassa	14	0.03	0.04	0.03	0.04
1	1	0.69	0	t	1	Unidentified voucher No.28	Leptaloma cognata	14	0.05	0.04	0.01	0.04
0	0	0.39	0	cv	1	Psoloessa texana	Philbostroma quadrimacula tum	14	0.03	0.04	0.01	0.04
0	1	0.92	0	t	1	Eragrostis intermedia	Bouteloua curtipendula	14	0.07	0.05	0.03	0.04
0	1	0.95	0	t	1	Eragrostis intermedia	Bouteloua hirsuta	14	0.07	0.05	0.04	0.03
0	1	0.52	0	t	1	Unknown Forb Q	Unknown Forb D	14	0.02	0.04	0.03	0.04
0	1	0.71	0	cv	1	Melanoplus desultorius	Setaria leucophila	14	0.01	0.04	0.05	0.04
1	1	0.67	0	t	1	Opeia obscura	Machaeranth era scabrella	14	0.03	0.04	0.03	0.04
0	0	0.33	0	t	1	Melanoplus lakinus	Condalia ericoides	14	0.01	0.04	0.02	0.04
1	1	0.62	0	t	1	Oenothera sp.	Verbena wrightii	14	0.04	0.03	0.02	0.04
1	1	0.66	0	cv	1	Syrbula fuscovittata	Calyophus hartwegii	14	0.05	0.04	0.01	0.04

0	0	0.26	0	t	1	Hoffmanseggia glauca	Philbostroma quadrimaculatum	14	0.01	0.04	0.01	0.04
0	1	0.90	0	t	1	Croton pottsii	Unknown Forb P	14	0.05	0.04	0.05	0.05
0	0	0.37	0	t	1	Unknown Forb H	Unknown Forb G	14	0.02	0.03	0.01	0.04
0	1	0.81	0	cv	1	Trimerotropis pallidipennis	Aulocara femoratum	14	0.05	0.05	0.03	0.04
0	0	0.24	0	t	1	Melanoplus arizonae	Dactylotum variegatum	14	0.01	0.04	0.01	0.04
0	0	0.26	0	t	1	Leprus cyaneus	Dactylotum variegatum	14	0.02	0.05	0.01	0.04
0	0	0.49	0	t	1	Boopedon nubilum	Muhlenbergia sp.	14	0.01	0.04	0.03	0.04
1	1	0.96	0	cv	1	Unknown Forb E	Unknown Forb P	14	0.07	0.05	0.05	0.05
1	1	0.62	0	t	1	Opeia obscura	Xanthocephalum sp.	14	0.03	0.04	0.02	0.02
0	1	0.53	0	t	1	Sporobolus sp.	Unknown Forb Q	14	0.03	0.04	0.02	0.04
0	1	0.60	0	t	1	Unknown Forb B	Convolvulus equitans	14	0.03	0.04	0.03	0.04
0	1	0.66	0	cv	1	Syrbula fuscovittata	Conyza coulteri	14	0.05	0.04	0.01	0.04
0	1	0.87	0	t	1	Unknown Forb F	Syrbula fuscovittata	14	0.03	0.03	0.05	0.04
0	1	0.63	0	t	1	Unknown Forb C	Oenothera sp.	14	0.02	0.04	0.04	0.03
1	1	0.64	0	t	1	Unidentified voucher No.28	Scleropogon brevifolius	14	0.05	0.04	0.01	0.04
0	1	0.77	0	cv	1	Unknown Forb E	Unknown Forb M	14	0.07	0.05	0.01	0.03
0	0	0.23	0	t	1	Cordillacris crenulata	Aristida hamulosa	14	0.01	0.04	0.01	0.03
0	0	0.30	0	t	1	Unknown Forb G	Dychoriste linearis	14	0.01	0.04	0.01	0.04
0	1	0.53	0	t	1	Polygala longa	Unknown Forb S	14	0.03	0.03	0.02	0.05
0	0	0.37	0	cv	1	Unknown Forb B	Ageneotettix deorum	14	0.03	0.04	0.01	0.04
0	1	0.85	0	t	1	Unknown Forb O	Machaeranthera scabrella	14	0.06	0.05	0.03	0.04
0	0	0.37	0	t	1	Unknown Forb S	Verbena wrightii	14	0.02	0.05	0.02	0.04
0	1	0.61	0	t	1	Xanthocephalum texanum	Unknown Forb D	14	0.03	0.04	0.03	0.04
0	0	0.37	0	cv	1	Aulocara femoratum	Tropidolophus formosus	14	0.03	0.04	0.01	0.04
1	0	0.40	0	t	1	Xanthocephalum sp.	Aristida hamulosa	14	0.02	0.02	0.01	0.03

0	0	0.26	0	t	1	Unknown Forb J	Melanoplus desultorius	14	0.01	0.04	0.01	0.04
1	1	0.80	0	t	1	Unknown Forb F	Bouteloua hirsuta	14	0.03	0.03	0.04	0.03
0	1	0.58	0	cv	1	Xanthocephalum texanum	Psoloessa texana	14	0.03	0.04	0.03	0.04
0	0	0.35	0	t	1	Unknown Forb B	Trachyrachys kiowa	14	0.03	0.04	0.01	0.04
0	0	0.49	0	t	1	Aulocara femoratum	Verbena wrightii	14	0.03	0.04	0.02	0.04
0	1	0.54	0	t	1	Unknown Forb H	Gilia rigida	14	0.02	0.03	0.03	0.03
0	0	0.41	0	cv	1	Ratibida columnaris	Convolvulus equitans	14	0.01	0.04	0.03	0.04
0	0	0.21	0	t	1	Scleropogon brevifolius	Iva dealbata	14	0.01	0.04	0.01	0.04
0	0	0.20	0	t	1	Scleropogon brevifolius	Zinnia grandiflora	14	0.01	0.04	0.01	0.05
0	1	0.66	0	t	1	Scleropogon brevifolius	Unknown Forb P	14	0.01	0.04	0.05	0.05
1	1	0.83	0	cv	1	Unidentified voucher No.28	Sporobolus sp.	14	0.05	0.04	0.03	0.04
0	1	0.74	0	t	1	Trimerotropis pallidipennis	Unknown Forb Q	14	0.05	0.05	0.02	0.04
0	0	0.42	0	t	1	Machaeranthera scabrella	Cryptantha mexicana	14	0.03	0.04	0.01	0.04
0	1	0.90	0	t	1	Opeia obscura	Unknown Forb E	14	0.03	0.04	0.07	0.05
0	0	0.32	0	cv	1	Unknown Forb G	Leprus cyaneus	14	0.01	0.04	0.02	0.05
0	0	0.36	0	t	1	Panicum obtusum	Unknown Forb R	14	0.01	0.04	0.02	0.05
0	0	0.29	0	t	1	Unknown Forb J	Unknown Forb C	14	0.01	0.04	0.02	0.04
1	1	0.65	0	t	1	Melanoplus desultorius	Bouteloua hirsuta	14	0.01	0.04	0.04	0.03
0	0	0.31	0	cv	1	Philbostroma quadrimaculatum	Condalia ericoides	14	0.01	0.04	0.02	0.04
0	1	0.55	0	t	1	Eupatorium greggii	Convolvulus equitans	14	0.02	0.04	0.03	0.04
0	0	0.42	0	t	1	Unknown Forb C	Unknown Forb H	14	0.02	0.04	0.02	0.03
0	0	0.45	0	t	1	Machaeranthera scabrella	Unknown Forb M	14	0.03	0.04	0.01	0.03
0	1	0.54	0	cv	1	Unknown Forb H	Convolvulus equitans	14	0.02	0.03	0.03	0.04
0	0	0.39	0	t	1	Xanthocephalum texanum	Aristida hamulosa	14	0.03	0.04	0.01	0.03
1	1	0.87	0	t	1	Syrbula fuscovittata	Bouteloua curtipendula	14	0.05	0.04	0.03	0.04

0	0	0.28	0	t	1	Unknown Forb N	Condalia ericoides	14	0.01	0.05	0.02	0.04
1	1	0.84	0	cv	1	Unknown Forb E	Unknown Forb R	14	0.07	0.05	0.02	0.05
0	0	0.27	0	t	1	Unknown Forb J	Leprus cyaneus	14	0.01	0.04	0.02	0.05
0	1	0.72	0	t	1	Psoloessa texana	Melanoplus gladstoni	14	0.03	0.04	0.04	0.04
0	0	0.38	0	t	1	Xanthocephalum sp.	Tropidolophus formosus	14	0.02	0.02	0.01	0.04
0	1	0.79	0	cv	1	Gilia rigida	Melanoplus glaucipes	14	0.03	0.03	0.05	0.04
1	1	0.74	0	t	1	Opeia obscura	Melanoplus gladstoni	14	0.03	0.04	0.04	0.04
0	0	0.22	0	t	1	Ziziphus obtusifolius	Iva dealbata	14	0.01	0.03	0.01	0.04
0	1	0.70	0	t	1	Unknown Forb C	Melanoplus glaucipes	14	0.02	0.04	0.05	0.04
0	1	0.52	0	cv	1	Psoloessa texana	Unknown Forb R	14	0.03	0.04	0.02	0.05
0	1	0.82	0	t	1	Eragrostis intermedia	Unknown Forb G	14	0.07	0.05	0.01	0.04
0	0	0.44	0	t	1	Buchloe dactyloides	Tropidolophus formosus	14	0.03	0.04	0.01	0.04
0	0	0.38	0	t	1	Psoloessa texana	Ratibida columnaris	14	0.03	0.04	0.01	0.04
1	1	0.84	0	cv	1	Unknown Forb E	Phoetaliotes nebrascensis	14	0.07	0.05	0.02	0.05
0	1	0.61	0	t	1	Buchloe dactyloides	Linum rigidum	14	0.03	0.04	0.02	0.04
0	0	0.22	0	t	1	Unknown Forb J	Cordillacris crenulata	14	0.01	0.04	0.01	0.04
0	1	0.91	0	t	1	Eragrostis intermedia	Machaeranthera scabrella	14	0.07	0.05	0.03	0.04
0	0	0.44	0	cv	1	Buchloe dactyloides	Calyophus hartwegii	14	0.03	0.04	0.01	0.04
0	0	0.20	0	t	1	Trachyrachys kiowa	Calyophus hartwegii	14	0.01	0.04	0.01	0.04
0	0	0.23	0	t	1	Unknown Forb J	Unknown Forb M	14	0.01	0.04	0.01	0.03
0	0	0.40	0	t	1	Unknown Forb G	Linum rigidum	14	0.01	0.04	0.02	0.04
0	1	0.74	0	cv	1	Trimerotropis pallidipennis	Unknown Forb C	14	0.05	0.05	0.02	0.04
0	0	0.46	0	t	1	Sporobolus sp.	Melanoplus lakinus	14	0.03	0.04	0.01	0.04
0	1	0.54	0	t	1	Machaeranthera scabrella	Condalia ericoides	14	0.03	0.04	0.02	0.04
0	0	0.38	0	t	1	Psoloessa texana	Ageneotettix deorum	14	0.03	0.04	0.01	0.04

0	0	0.22	0	cv	1	Ageneotettix deorum	Eritettix variabilis	14	0.01	0.04	0.01	0.04
0	1	0.57	0	t	1	Opeia obscura	Eupatorium greggii	14	0.03	0.04	0.02	0.04
0	0	0.47	0	t	1	Melanoplus lakinus	Arphia crassa	14	0.01	0.04	0.03	0.04
0	0	0.31	0	t	1	Unknown Forb H	Tropidolophus formosus	14	0.02	0.03	0.01	0.04
0	1	0.60	0	cv	1	Ageneotettix deorum	Melanoplus glaucipes	14	0.01	0.04	0.05	0.04
0	1	0.63	0	t	1	Unidentified voucher No.28	Cryptantha mexicana	14	0.05	0.04	0.01	0.04
0	1	0.53	0	t	1	Unknown Forb C	Muhlenbergia sp.	14	0.02	0.04	0.03	0.04
0	0	0.35	0	t	1	Leptaloma cognata	Condalia ericoides	14	0.01	0.04	0.02	0.04
0	1	0.54	0	cv	1	Aulocara femoratum	Linum rigidum	14	0.03	0.04	0.02	0.04
0	0	0.50	0	t	1	Unknown Forb F	Boopedon nubilum	14	0.03	0.03	0.01	0.04
0	1	0.55	0	t	1	Unknown Forb H	Unknown Forb D	14	0.02	0.03	0.03	0.04
0	0	0.38	0	t	1	Melanoplus arizonae	Eupatorium greggii	14	0.01	0.04	0.02	0.04
0	1	0.52	0	cv	1	Panicum obtusum	Bouteloua curtipendula	14	0.01	0.04	0.03	0.04
0	1	0.66	0	t	1	Philbostroma quadrimaculatum	Unknown Forb P	14	0.01	0.04	0.05	0.05
0	1	0.56	0	t	1	Polygala longa	Verbena wrightii	14	0.03	0.03	0.02	0.04
0	1	0.63	0	t	1	Psoloessa texana	Muhlenbergia sp.	14	0.03	0.04	0.03	0.04
0	1	0.72	0	cv	1	Aulocara femoratum	Oenothera sp.	14	0.03	0.04	0.04	0.03
0	0	0.28	1	t	1	Prunus avium	Diloba caeruleocephala	15	0.04	0.07	0.01	0.10
0	0	0.27	1	t	1	Prunus avium	Electrophaes corylata	15	0.04	0.07	0.01	0.10
0	0	0.44	1	t	1	Prunus avium	Aporia crataegi	15	0.04	0.07	0.03	0.10
0	0	0.49	1	cv	1	Prunus avium	Bison betularia	15	0.04	0.07	0.03	0.08
0	1	0.57	1	t	1	Prunus avium	Acronicta psi	15	0.04	0.07	0.04	0.08
0	0	0.24	1	t	1	Agriopis aurantiaria	Prunus padus	15	0.01	0.12	0.06	0.13
0	0	0.24	1	t	1	Dasychira pudibunda	Prunus padus	15	0.01	0.12	0.06	0.13
0	0	0.24	1	cv	1	Strymonidia pruni	Prunus padus	15	0.01	0.12	0.06	0.13

0	0	0.24	1	t	1	Lycia pomonaria	Prunus padus	15	0.01	0.12	0.06	0.13
0	0	0.40	1	t	1	Abraxas grossulariata	Prunus padus	15	0.03	0.12	0.06	0.13
0	0	0.42	1	t	1	Lomographa temerata	Prunus padus	15	0.03	0.11	0.06	0.13
0	0	0.12	1	cv	1	Lomographa temerata	Prunus cerasus	15	0.03	0.11	0.01	0.09
0	0	0.25	1	t	1	Eriogaster lanestris	Prunus padus	15	0.01	0.12	0.06	0.13
0	0	0.06	1	t	1	Eriogaster lanestris	Prunus cerasus	15	0.01	0.12	0.01	0.09
0	0	0.32	1	t	1	Triphosa dubitata	Prunus padus	15	0.02	0.12	0.06	0.13
0	0	0.42	1	cv	1	Opisthograptis luteolata	Prunus padus	15	0.02	0.09	0.06	0.13
0	1	0.75	1	t	1	Opisthograptis luteolata	Prunus domestica	15	0.02	0.09	0.08	0.07
0	0	0.25	1	t	1	Orthosia	Prunus padus	15	0.01	0.12	0.06	0.13
0	0	0.06	1	t	1	Orthosia gracilis	Prunus cerasus	15	0.01	0.12	0.01	0.09
0	0	0.32	1	cv	1	Boarmia punctinalis	Prunus padus	15	0.02	0.12	0.06	0.13
0	0	0.32	1	t	1	Lasiocampa quercus	Prunus padus	15	0.02	0.12	0.06	0.13
0	0	0.24	1	t	1	Trichiura crataegi	Prunus padus	15	0.01	0.12	0.06	0.13
0	0	0.24	1	t	1	Chloroclysta siterata	Prunus padus	15	0.01	0.12	0.06	0.13
0	0	0.24	1	cv	1	Lycia hirtaria	Prunus padus	15	0.01	0.12	0.06	0.13
0	0	0.24	1	t	1	Acronicta strigosa	Prunus padus	15	0.01	0.12	0.06	0.13
0	0	0.24	1	t	1	Lomographa bimaculata	Prunus padus	15	0.01	0.12	0.06	0.13
0	0	0.32	1	t	1	Blepharita satura	Prunus padus	15	0.02	0.12	0.06	0.13
0	0	0.33	1	cv	1	Colocasia	Prunus padus	15	0.01	0.09	0.06	0.13
0	1	0.67	1	t	1	Colocasia coryli	Prunus domestica	15	0.01	0.09	0.08	0.07
0	0	0.48	1	t	1	Abraxas	Prunus padus	15	0.04	0.12	0.06	0.13
0	0	0.31	1	t	1	Diloba caeruleocephala	Prunus padus	15	0.01	0.10	0.06	0.13
0	0	0.08	1	cv	1	Diloba caeruleocephala	Prunus cerasus	15	0.01	0.10	0.01	0.09
0	0	0.08	1	t	1	Diloba caeruleocephala	Prunus spinosa	15	0.01	0.10	0.01	0.08
0	0	0.24	1	t	1	Eurois	Prunus padus	15	0.01	0.12	0.06	0.13

0	1	0.99	1	t	1	Apocheima pilosaria	Prunus padus	15	0.16	0.15	0.06	0.13
0	0	0.24	1	cv	1	Dasychira fascelina	Prunus padus	15	0.01	0.12	0.06	0.13
0	0	0.29	1	t	1	Prunus padus	Selenia dentaria	15	0.06	0.13	0.02	0.12
0	0	0.29	1	t	1	Prunus padus	Orthosia gothica	15	0.06	0.13	0.01	0.09
0	0	0.45	1	t	1	Prunus padus	Chloroclystis chloerata	15	0.06	0.13	0.04	0.12
0	1	0.53	1	cv	1	Prunus padus	Odontopera bidentata	15	0.06	0.13	0.04	0.11
0	0	0.30	1	t	1	Prunus padus	Allophyes oxycanthae	15	0.06	0.13	0.01	0.09
0	0	0.22	1	t	1	Prunus padus	Selenia tetralunaria	15	0.06	0.13	0.01	0.12
0	0	0.29	1	t	1	Prunus padus	Eupithecia exiguata	15	0.06	0.13	0.02	0.12
0	0	0.26	1	cv	1	Prunus padus	Electrophaes corylata	15	0.06	0.13	0.01	0.10
0	0	0.23	1	t	1	Prunus padus	Erannis defoliaria	15	0.06	0.13	0.01	0.12
0	0	0.22	1	t	1	Prunus padus	Angerona prunaria	15	0.06	0.13	0.01	0.12
0	0	0.45	1	t	1	Prunus padus	Xanthia togata	15	0.06	0.13	0.04	0.12
0	0	0.30	1	cv	1	Prunus padus	Cosmia trapezina	15	0.06	0.13	0.01	0.09
0	0	0.29	1	t	1	Prunus padus	Selenia lunalaria	15	0.06	0.13	0.02	0.12
0	0	0.29	1	t	1	Prunus padus	Epirrita autumnata	15	0.06	0.13	0.01	0.09
0	0	0.22	1	t	1	Prunus padus	Euproctis similis	15	0.06	0.13	0.01	0.12
0	0	0.38	1	cv	1	Prunus padus	Thecla betulae	15	0.06	0.13	0.02	0.09
0	0	0.22	1	t	1	Prunus padus	Plagodis pulveraria	15	0.06	0.13	0.01	0.12
0	0	0.22	1	t	1	Prunus padus	Chloroclysta truncata	15	0.06	0.13	0.01	0.12
0	0	0.22	1	t	1	Prunus padus	Plemyria rubiginata	15	0.06	0.13	0.01	0.12
0	0	0.22	1	cv	1	Prunus padus	Camptogramma bilineata	15	0.06	0.13	0.01	0.12
0	0	0.22	1	t	1	Prunus padus	Polia bombycina	15	0.06	0.13	0.01	0.12
0	0	0.22	1	t	1	Prunus padus	Lithophane ornitopus	15	0.06	0.13	0.01	0.12
0	0	0.22	1	t	1	Prunus padus	Smerinthus ocellata	15	0.06	0.13	0.01	0.12

0	0	0.22	1	cv	1	Prunus padus	Naenia typica	15	0.06	0.13	0.01	0.12
0	0	0.22	1	t	1	Prunus padus	Lithophane socia	15	0.06	0.13	0.01	0.12
0	0	0.44	1	t	1	Prunus padus	Aporia crataegi	15	0.06	0.13	0.03	0.10
0	0	0.22	1	t	1	Prunus padus	Epirrita christyi	15	0.06	0.13	0.01	0.12
0	0	0.22	1	cv	1	Prunus padus	Psyche betulina	15	0.06	0.13	0.01	0.12
0	0	0.29	1	t	1	Prunus padus	Crocallis elinguaria	15	0.06	0.13	0.02	0.12
0	0	0.29	1	t	1	Prunus padus	Operophtera brumata	15	0.06	0.13	0.01	0.09
0	0	0.38	1	t	1	Prunus padus	Arctia caja	15	0.06	0.13	0.02	0.09
0	0	0.48	1	cv	1	Prunus padus	Bison betularia	15	0.06	0.13	0.03	0.08
0	0	0.22	1	t	1	Prunus padus	Agrochola circellaris	15	0.06	0.13	0.01	0.12
0	0	0.22	1	t	1	Prunus padus	Colotois pennaria	15	0.06	0.13	0.01	0.12
0	0	0.22	1	t	1	Prunus padus	Nola cucullatella	15	0.06	0.13	0.01	0.12
0	0	0.22	1	cv	1	Prunus padus	Chloroclystis rectangulata	15	0.06	0.13	0.01	0.12
0	1	0.57	1	t	1	Prunus padus	Acronicta psi	15	0.06	0.13	0.04	0.08
0	0	0.22	1	t	1	Prunus padus	Conistra vaccinii	15	0.06	0.13	0.01	0.12
0	0	0.29	1	t	1	Prunus padus	Eupsilia transversa	15	0.06	0.13	0.01	0.09
0	0	0.22	1	cv	1	Prunus padus	Brachionycha nubeculosa	15	0.06	0.13	0.01	0.12
0	0	0.09	1	t	1	Prunus cerasus	Allophyes oxycanthae	15	0.01	0.09	0.01	0.09
0	0	0.09	1	t	1	Prunus cerasus	Cosmia trapezina	15	0.01	0.09	0.01	0.09
0	0	0.09	1	t	1	Prunus cerasus	Operophtera brumata	15	0.01	0.09	0.01	0.09
0	0	0.24	1	cv	1	Prunus	Acronicta psi	15	0.01	0.09	0.04	0.08
0	0	0.09	1	t	1	Prunus cerasus	Eupsilia transversa	15	0.01	0.09	0.01	0.09
0	1	0.67	1	t	1	Orthosia gothica	Prunus domestica	15	0.01	0.09	0.08	0.07
1	0	0.09	1	t	1	Allophyes oxycanthae	Prunus spinosa	15	0.01	0.09	0.01	0.08
0	1	0.68	1	cv	1	Allophyes oxycanthae	Prunus domestica	15	0.01	0.09	0.08	0.07
0	0	0.07	1	t	1	Prunus spinosa	Erannis defoliaria	15	0.01	0.08	0.01	0.12
0	0	0.09	1	t	1	Prunus spinosa	Cosmia trapezina	15	0.01	0.08	0.01	0.09

0	0	0.16	1	t	1	Prunus spinosa	Aporia crataegi	15	0.01	0.08	0.03	0.10
0	1	0.68	1	cv	1	Cosmia trapezina	Prunus domestica	15	0.01	0.09	0.08	0.07
0	1	0.67	1	t	1	Epirrita autumnata	Prunus domestica	15	0.01	0.09	0.08	0.07
0	1	0.75	1	t	1	Thecla betulae	Prunus domestica	15	0.02	0.09	0.08	0.07
0	1	0.68	1	t	1	Operophtera brumata	Prunus domestica	15	0.01	0.09	0.08	0.07
0	1	0.75	1	cv	1	Arctia caja	Prunus domestica	15	0.02	0.09	0.08	0.07
0	1	0.82	1	t	1	Bison betularia	Prunus domestica	15	0.03	0.08	0.08	0.07
0	1	0.85	1	t	1	Prunus domestica	Acronicta psi	15	0.08	0.07	0.04	0.08
0	1	0.65	1	t	1	Prunus domestica	Eupsilia transversa	15	0.08	0.07	0.01	0.09
0	1	0.92	0	cv	1	Apocheima pilosaria	Dasychira fascelina	15	0.16	0.15	0.01	0.12
0	0	0.04	0	t	1	Angerona prunaria	Chloroclystis rectangulata	15	0.01	0.12	0.01	0.12
0	0	0.08	0	t	1	Eupithecia exiguata	Selenia lunalaria	15	0.02	0.12	0.02	0.12
0	0	0.06	0	t	1	Smerinthus ocellata	Operophtera brumata	15	0.01	0.12	0.01	0.09
0	0	0.12	0	cv	1	Allophyes oxycanthae	Thecla betulae	15	0.01	0.09	0.02	0.09
0	0	0.07	0	t	1	Abraxas grossulariata	Nola cucullatella	15	0.03	0.12	0.01	0.12
0	0	0.05	0	t	1	Triphosa dubitata	Lithophane socia	15	0.02	0.12	0.01	0.12
0	0	0.09	0	t	1	Lasiocampa quercus	Prunus spinosa	15	0.02	0.12	0.01	0.08
0	0	0.05	0	cv	1	Selenia lunalaria	Epirrita christyi	15	0.02	0.12	0.01	0.12
0	0	0.04	0	t	1	Dasychira pudibunda	Agrochola circellaris	15	0.01	0.12	0.01	0.12
0	0	0.06	0	t	1	Smerinthus ocellata	Crocallis elinguaria	15	0.01	0.12	0.02	0.12
0	1	0.97	0	t	1	Apocheima pilosaria	Xanthia togata	15	0.16	0.15	0.04	0.12
0	0	0.06	0	cv	1	Lithophane socia	Operophtera brumata	15	0.01	0.12	0.01	0.09
0	0	0.11	0	t	1	Abraxas grossulariata	Operophtera brumata	15	0.03	0.12	0.01	0.09
0	0	0.04	0	t	1	Lomographa bimaculata	Chloroclysta truncata	15	0.01	0.12	0.01	0.12
0	0	0.06	0	t	1	Orthosia gothica	Polia bombycina	15	0.01	0.09	0.01	0.12

0	0	0.06	0	cv	1	Colocasia coryli	Chloroclystis rectangulata	15	0.01	0.09	0.01	0.12
0	0	0.04	0	t	1	Dasychira pudibunda	Erannis defoliaria	15	0.01	0.12	0.01	0.12
0	0	0.05	0	t	1	Blepharita satura	Dasychira fascelina	15	0.02	0.12	0.01	0.12
0	0	0.05	0	t	1	Electrophaes corylata	Camptogramma bilineata	15	0.01	0.10	0.01	0.12
0	0	0.07	0	cv	1	Abraxas grossulariata	Camptogramma bilineata	15	0.03	0.12	0.01	0.12
0	0	0.04	0	t	1	Dasychira fascelina	Selenia tetralunaria	15	0.01	0.12	0.01	0.12
0	0	0.04	0	t	1	Eriogaster lanestris	Lithophane ornitopus	15	0.01	0.12	0.01	0.12
0	0	0.12	0	t	1	Lycia hirtaria	Bison betularia	15	0.01	0.12	0.03	0.08
0	0	0.15	0	cv	1	Smerinthus ocellata	Acronicta psi	15	0.01	0.12	0.04	0.08
0	0	0.04	0	t	1	Dasychira fascelina	Polia bombycina	15	0.01	0.12	0.01	0.12
0	0	0.07	0	t	1	Abraxas grossulariata	Lomographa bimaculata	15	0.03	0.12	0.01	0.12
0	1	0.95	0	t	1	Apocheima pilosaria	Orthosia gothica	15	0.16	0.15	0.01	0.09
0	0	0.06	0	cv	1	Colocasia coryli	Eurois occulata	15	0.01	0.09	0.01	0.12
0	0	0.04	0	t	1	Angerona prunaria	Conistra vaccinii	15	0.01	0.12	0.01	0.12
0	0	0.06	0	t	1	Angerona prunaria	Eupsilia transversa	15	0.01	0.12	0.01	0.09
0	0	0.05	0	t	1	Boarmia punctinalis	Selenia tetralunaria	15	0.02	0.12	0.01	0.12
0	0	0.06	0	cv	1	Orthosia gracilis	Orthosia gothica	15	0.01	0.12	0.01	0.09
0	0	0.08	0	t	1	Lasiocampa quercus	Eupithecia exiguata	15	0.02	0.12	0.02	0.12
0	0	0.06	0	t	1	Lycia pomonaria	Allophyes oxycanthae	15	0.01	0.12	0.01	0.09
0	0	0.05	0	t	1	Lasiocampa quercus	Naenia typica	15	0.02	0.12	0.01	0.12
0	0	0.05	0	cv	1	Eurois occulata	Epirrita autumnata	15	0.01	0.12	0.01	0.09
0	0	0.04	0	t	1	Lycia pomonaria	Agrochola circellaris	15	0.01	0.12	0.01	0.12
0	0	0.04	0	t	1	Orthosia gracilis	Camptogramma bilineata	15	0.01	0.12	0.01	0.12
0	0	0.16	0	t	1	Acronicta psi	Conistra vaccinii	15	0.04	0.08	0.01	0.12
0	0	0.22	0	cv	1	Prunus avium	Chloroclysta siterata	15	0.04	0.07	0.01	0.12

0	0	0.12	0	t	1	Cosmia trapezina	Arctia caja	15	0.01	0.09	0.02	0.09
0	0	0.06	0	t	1	Dasychira pudibunda	Cosmia trapezina	15	0.01	0.12	0.01	0.09
0	0	0.05	0	t	1	Eupithecia exiguata	Conistra vaccinii	15	0.02	0.12	0.01	0.12
0	0	0.07	0	cv	1	Abraxas grossulariata	Lithophane ornitopus	15	0.03	0.12	0.01	0.12
0	0	0.04	0	t	1	Strymonidia pruni	Eurois occulata	15	0.01	0.12	0.01	0.12
0	0	0.18	0	t	1	Odontopera bidentata	Eupsilia transversa	15	0.04	0.11	0.01	0.09
0	0	0.04	0	t	1	Strymonidia pruni	Lomographa bimaculata	15	0.01	0.12	0.01	0.12
0	0	0.09	0	cv	1	Opisthograptis luteolata	Orthosia gracilis	15	0.02	0.09	0.01	0.12
0	0	0.04	0	t	1	Euproctis similis	Plagodis pulveraria	15	0.01	0.12	0.01	0.12
0	0	0.08	0	t	1	Erannis defoliaria	Thecla betulae	15	0.01	0.12	0.02	0.09
0	0	0.08	0	t	1	Agriopis aurantiaria	Lomographa temerata	15	0.01	0.12	0.03	0.11
0	0	0.05	0	cv	1	Triphosa dubitata	Nola cucullatella	15	0.02	0.12	0.01	0.12
0	0	0.05	0	t	1	Triphosa dubitata	Plemyria rubiginata	15	0.02	0.12	0.01	0.12
0	1	0.92	0	t	1	Apocheima pilosaria	Colotois pennaria	15	0.16	0.15	0.01	0.12
0	0	0.04	0	t	1	Euproctis similis	Colotois pennaria	15	0.01	0.12	0.01	0.12
0	0	0.05	0	cv	1	Electrophaes corylata	Epirrita christyi	15	0.01	0.10	0.01	0.12
0	0	0.08	0	t	1	Lycia pomonaria	Thecla betulae	15	0.01	0.12	0.02	0.09
0	0	0.05	0	t	1	Selenia lunalaria	Naenia typica	15	0.02	0.12	0.01	0.12
0	0	0.08	0	t	1	Lomographa temerata	Plagodis pulveraria	15	0.03	0.11	0.01	0.12
0	0	0.05	0	cv	1	Selenia dentaria	Camptogramma bilineata	15	0.02	0.12	0.01	0.12
0	0	0.10	0	t	1	Agriopis aurantiaria	Xanthia togata	15	0.01	0.12	0.04	0.12
0	0	0.10	0	t	1	Abraxas sylvata	Lithophane ornitopus	15	0.04	0.12	0.01	0.12
0	1	0.91	0	t	1	Prunus avium	Prunus domestica	15	0.04	0.07	0.08	0.07
0	0	0.06	0	cv	1	Dasychira fascelina	Crocallis elinguaria	15	0.01	0.12	0.02	0.12
0	0	0.13	0	t	1	Electrophaes corylata	Aporia crataegi	15	0.01	0.10	0.03	0.10

0	0	0.04	0	t	1	Selenia tetralunaria	Epirrita christyi	15	0.01	0.12	0.01	0.12
0	0	0.04	0	t	1	Dasychira fascelina	Nola cucullatella	15	0.01	0.12	0.01	0.12
0	0	0.11	0	cv	1	Orthosia gracilis	Chloroclystis chloerata	15	0.01	0.12	0.04	0.12
0	0	0.08	0	t	1	Boarmia punctinalis	Cosmia trapezina	15	0.02	0.12	0.01	0.09
0	0	0.05	0	t	1	Boarmia punctinalis	Conistra vaccinii	15	0.02	0.12	0.01	0.12
0	0	0.05	0	t	1	Electrophaes corylata	Naenia typica	15	0.01	0.10	0.01	0.12
0	0	0.08	0	cv	1	Boarmia punctinalis	Prunus cerasus	15	0.02	0.12	0.01	0.09
0	0	0.05	0	t	1	Boarmia punctinalis	Plemyria rubiginata	15	0.02	0.12	0.01	0.12
0	0	0.06	0	t	1	Orthosia gracilis	Selenia lunalaria	15	0.01	0.12	0.02	0.12
0	0	0.23	0	t	1	Cosmia trapezina	Acronicta psi	15	0.01	0.09	0.04	0.08
0	0	0.06	0	cv	1	Eurois occulata	Prunus cerasus	15	0.01	0.12	0.01	0.09
0	0	0.10	0	t	1	Xanthia togata	Plagodis pulveraria	15	0.04	0.12	0.01	0.12
0	0	0.07	0	t	1	Blepharita satura	Electrophaes corylata	15	0.02	0.12	0.01	0.10
0	0	0.21	0	t	1	Eupithecia exiguata	Acronicta psi	15	0.02	0.12	0.04	0.08
0	0	0.04	0	cv	1	Erannis defoliaria	Polia bombycina	15	0.01	0.12	0.01	0.12
0	0	0.04	0	t	1	Euproctis similis	Plemyria rubiginata	15	0.01	0.12	0.01	0.12
0	0	0.27	0	t	1	Abraxas sylvata	Bison betularia	15	0.04	0.12	0.03	0.08
0	0	0.14	0	t	1	Eurois occulata	Odontopera bidentata	15	0.01	0.12	0.04	0.11
0	0	0.30	0	cv	1	Prunus avium	Colocasia coryli	15	0.04	0.07	0.01	0.09
0	0	0.08	0	t	1	Diloba caeruleocephala	Allophyes oxycanthae	15	0.01	0.10	0.01	0.09
0	0	0.04	0	t	1	Lomographa bimaculata	Angerona prunaria	15	0.01	0.12	0.01	0.12
0	0	0.08	0	t	1	Acronicta strigosa	Thecla betulae	15	0.01	0.12	0.02	0.09
0	0	0.08	0	cv	1	Opisthograptis luteolata	Polia bombycina	15	0.02	0.09	0.01	0.12
0	0	0.15	0	t	1	Selenia tetralunaria	Acronicta psi	15	0.01	0.12	0.04	0.08
0	0	0.06	0	t	1	Prunus cerasus	Agrochola circellaris	15	0.01	0.09	0.01	0.12

0	0	0.06	0	t	1	Eriogaster lanestris	Eupsilia transversa	15	0.01	0.12	0.01	0.09
0	1	0.98	0	cv	1	Lomographa temerata	Apocheima pilosaria	15	0.03	0.11	0.16	0.15
0	0	0.24	0	t	1	Chloroclystis chloerata	Aporia crataegi	15	0.04	0.12	0.03	0.10
0	0	0.15	0	t	1	Abraxas grossulariata	Opisthograptis luteolata	15	0.03	0.12	0.02	0.09
1	1	0.97	1	t	1	Chloroclystis rectangulata	Prunus spinosa	16	0.02	0.14	0.16	0.13
0	0	0.19	1	cv	1	Chloroclystis rectangulata	Prunus avium	16	0.02	0.14	0.04	0.08
0	0	0.12	1	t	1	Chloroclystis rectangulata	Prunus cerasus	16	0.02	0.14	0.03	0.11
0	1	0.94	1	t	1	Orthosia incerta	Prunus spinosa	16	0.01	0.14	0.16	0.13
0	1	0.94	1	t	1	Eriogaster lanestris	Prunus spinosa	16	0.01	0.14	0.16	0.13
1	0	0.10	1	cv	1	Triphosa dubitata	Prunus padus	16	0.01	0.10	0.02	0.10
1	1	0.82	1	t	1	Acronicta tridens	Prunus domestica	16	0.02	0.14	0.12	0.11
1	1	0.96	1	t	1	Acronicta tridens	Prunus spinosa	16	0.02	0.14	0.16	0.13
1	1	0.80	1	t	1	Lithophane semibrunnea	Prunus domestica	16	0.01	0.13	0.12	0.11
1	1	0.82	1	cv	1	Abraxas grossulariata	Prunus domestica	16	0.02	0.14	0.12	0.11
1	1	0.96	1	t	1	Abraxas grossulariata	Prunus spinosa	16	0.02	0.14	0.16	0.13
0	1	0.94	1	t	1	Diarsia mendica	Prunus spinosa	16	0.01	0.14	0.16	0.13
1	1	0.82	1	t	1	Nola cucullatella	Prunus domestica	16	0.02	0.14	0.12	0.11
1	1	0.96	1	cv	1	Nola cucullatella	Prunus spinosa	16	0.02	0.14	0.16	0.13
1	1	0.82	1	t	1	Angerona prunaria	Prunus domestica	16	0.02	0.14	0.12	0.11
1	1	0.96	1	t	1	Angerona prunaria	Prunus spinosa	16	0.02	0.14	0.16	0.13
1	1	0.80	1	t	1	Orthosia munda	Prunus domestica	16	0.01	0.13	0.12	0.11
1	1	0.82	1	cv	1	Thecla betulae	Prunus domestica	16	0.02	0.14	0.12	0.11
1	1	0.96	1	t	1	Thecla betulae	Prunus spinosa	16	0.02	0.14	0.16	0.13
1	1	0.80	1	t	1	Acronicta aceris	Prunus domestica	16	0.01	0.13	0.12	0.11
0	1	0.94	1	t	1	Smerinthus ocellata	Prunus spinosa	16	0.01	0.14	0.16	0.13

0	1	0.94	1	cv	1	Perconia strigillaria	Prunus spinosa	16	0.01	0.14	0.16	0.13
0	1	0.94	1	t	1	Xestia triangulum	Prunus spinosa	16	0.01	0.14	0.16	0.13
0	1	0.94	1	t	1	Agriopis aurantiaria	Prunus spinosa	16	0.01	0.14	0.16	0.13
1	1	0.82	1	t	1	Strymonidia pruni	Prunus domestica	16	0.02	0.14	0.12	0.11
1	1	0.96	1	cv	1	Strymonidia pruni	Prunus spinosa	16	0.02	0.14	0.16	0.13
1	1	0.82	1	t	1	Dasycampa rubiginea	Prunus domestica	16	0.02	0.14	0.12	0.11
1	1	0.96	1	t	1	Dasycampa rubiginea	Prunus spinosa	16	0.02	0.14	0.16	0.13
1	0	0.05	1	t	1	Lomographa temerata	Prunus padus	16	0.01	0.14	0.02	0.10
1	1	0.76	1	cv	1	Lomographa temerata	Prunus domestica	16	0.01	0.14	0.12	0.11
1	1	0.94	1	t	1	Lomographa temerata	Prunus spinosa	16	0.01	0.14	0.16	0.13
1	1	0.82	1	t	1	Conistra ligula	Prunus domestica	16	0.02	0.14	0.12	0.11
1	1	0.96	1	t	1	Conistra ligula	Prunus spinosa	16	0.02	0.14	0.16	0.13
0	1	0.94	1	cv	1	Orgyia recens	Prunus spinosa	16	0.01	0.14	0.16	0.13
0	1	0.94	1	t	1	Stauropus fagi	Prunus spinosa	16	0.01	0.14	0.16	0.13
1	0	0.26	1	t	1	Lomographa bimaculata	Prunus avium	16	0.01	0.08	0.04	0.08
0	1	0.94	1	t	1	Gastropacha quercifolia	Prunus spinosa	16	0.01	0.14	0.16	0.13
0	0	0.07	1	cv	1	Prunus padus	Xestia baja	16	0.02	0.10	0.02	0.14
1	1	0.95	1	t	1	Allophyes oxycanthae	Prunus spinosa	16	0.02	0.14	0.16	0.13
0	0	0.09	1	t	1	Allophyes oxycanthae	Prunus cerasus	16	0.02	0.14	0.03	0.11
1	1	0.82	1	t	1	Prunus domestica	Phalera bucephala	16	0.12	0.11	0.02	0.13
0	1	0.78	1	cv	1	Prunus domestica	Selenia lunalaria	16	0.12	0.11	0.02	0.14
0	1	0.78	1	t	1	Prunus domestica	Ennomos autumnaria	16	0.12	0.11	0.02	0.14
0	1	0.78	1	t	1	Prunus domestica	Aporia crataegi	16	0.12	0.11	0.02	0.14
0	1	0.78	1	t	1	Prunus domestica	Theria rupicaprarian	16	0.12	0.11	0.02	0.14
0	1	0.78	1	cv	1	Prunus domestica	Acronicta psi	16	0.12	0.11	0.02	0.14

0	1	0.83	1	t	1	Prunus domestica	Diloba caeruleocephala	16	0.12	0.11	0.02	0.14
0	1	0.78	1	t	1	Prunus domestica	Odontopera bidentata	16	0.12	0.11	0.02	0.14
0	1	0.78	1	t	1	Prunus domestica	Epirrita dilutata	16	0.12	0.11	0.02	0.14
0	1	0.76	1	cv	1	Prunus domestica	Amphipyra pyramidea	16	0.12	0.11	0.01	0.13
0	1	0.78	1	t	1	Prunus domestica	Naenia typica	16	0.12	0.11	0.02	0.14
0	1	0.78	1	t	1	Prunus domestica	Cosmia pyralina	16	0.12	0.11	0.02	0.14
0	1	0.76	1	t	1	Prunus domestica	Lasiocampa trifolii	16	0.12	0.11	0.01	0.13
0	1	0.76	1	cv	1	Prunus domestica	Catocala nupta	16	0.12	0.11	0.01	0.13
0	1	0.76	1	t	1	Prunus domestica	Bison betularia	16	0.12	0.11	0.01	0.13
0	1	0.78	1	t	1	Prunus domestica	Callimorpha dominula	16	0.12	0.11	0.02	0.14
0	1	0.76	1	t	1	Prunus domestica	Dasychira fascelina	16	0.12	0.11	0.01	0.13
0	1	0.78	1	cv	1	Prunus domestica	Thalera fimbrialis	16	0.12	0.11	0.02	0.14
0	1	0.76	1	t	1	Prunus domestica	Lithophane socia	16	0.12	0.11	0.01	0.13
0	1	0.94	1	t	1	Ourapteryx sambucaria	Prunus spinosa	16	0.01	0.14	0.16	0.13
0	1	0.94	1	t	1	Brachionycha sphinx	Prunus spinosa	16	0.01	0.14	0.16	0.13
0	1	0.94	1	cv	1	Erannis defoliaria	Prunus spinosa	16	0.01	0.14	0.16	0.13
1	0	0.11	1	t	1	Phalera bucephala	Prunus cerasus	16	0.02	0.13	0.03	0.11
1	1	0.96	1	t	1	Selenia lunalaria	Prunus spinosa	16	0.02	0.14	0.16	0.13
1	0	0.11	1	t	1	Plagodis pulveraria	Prunus cerasus	16	0.01	0.11	0.03	0.11
1	1	0.96	1	cv	1	Ennomos autumnaria	Prunus spinosa	16	0.02	0.14	0.16	0.13
1	1	0.96	1	t	1	Aporia crataegi	Prunus spinosa	16	0.02	0.14	0.16	0.13
0	1	0.94	1	t	1	Cilix glaucata	Prunus spinosa	16	0.01	0.14	0.16	0.13
1	1	0.96	1	t	1	Theria rupicaprarian	Prunus spinosa	16	0.02	0.14	0.16	0.13
0	1	0.94	1	cv	1	Acronicta strigosa	Prunus spinosa	16	0.01	0.14	0.16	0.13
1	1	0.96	1	t	1	Acronicta psi	Prunus spinosa	16	0.02	0.14	0.16	0.13

0	1	0.94	1	t	1	Crocallis elinguaria	Prunus spinosa	16	0.01	0.14	0.16	0.13
0	1	0.94	1	t	1	Malacosoma neustria	Prunus spinosa	16	0.01	0.14	0.16	0.13
1	0	0.26	1	cv	1	Alsophila aescularia	Prunus avium	16	0.01	0.08	0.04	0.08
1	1	0.97	1	t	1	Orthosia gracilis	Prunus spinosa	16	0.02	0.14	0.16	0.13
0	1	0.94	1	t	1	Lasiocampa quercus	Prunus spinosa	16	0.01	0.14	0.16	0.13
1	1	0.97	1	t	1	Diloba caeruleocephala	Prunus spinosa	16	0.02	0.14	0.16	0.13
1	0	0.05	1	cv	1	Diloba caeruleocephala	Prunus laurocerasus	16	0.02	0.14	0.01	0.12
0	1	0.94	1	t	1	Plemyria rubiginata	Prunus spinosa	16	0.01	0.14	0.16	0.13
0	1	0.94	1	t	1	Selenia dentaria	Prunus spinosa	16	0.01	0.14	0.16	0.13
0	1	0.94	1	t	1	Euproctis chrysorrhoea	Prunus spinosa	16	0.01	0.14	0.16	0.13
1	1	0.96	1	cv	1	Odontopera bidentata	Prunus spinosa	16	0.02	0.14	0.16	0.13
0	1	0.94	1	t	1	Prunus spinosa	Epirrita dilutata	16	0.16	0.13	0.02	0.14
0	1	0.91	1	t	1	Prunus spinosa	Chloroclysta siterata	16	0.16	0.13	0.01	0.14
0	1	0.94	1	t	1	Prunus	Xestia baja	16	0.16	0.13	0.02	0.14
0	1	0.91	1	cv	1	Prunus spinosa	Colotois pennaria	16	0.16	0.13	0.01	0.14
0	1	0.94	1	t	1	Prunus spinosa	Naenia typica	16	0.16	0.13	0.02	0.14
0	1	0.91	1	t	1	Prunus spinosa	Campaea margaritata	16	0.16	0.13	0.01	0.14
0	1	0.91	1	t	1	Prunus spinosa	Blepharita satura	16	0.16	0.13	0.01	0.14
0	1	0.91	1	cv	1	Prunus spinosa	Epirrita christyi	16	0.16	0.13	0.01	0.14
0	1	0.94	1	t	1	Prunus spinosa	Cosmia pyralina	16	0.16	0.13	0.02	0.14
0	1	0.91	1	t	1	Prunus spinosa	Eurois occulata	16	0.16	0.13	0.01	0.14
0	1	0.91	1	t	1	Prunus spinosa	Chloroclystis chloerata	16	0.16	0.13	0.01	0.14
0	1	0.91	1	cv	1	Prunus spinosa	Semiothsia alternaria	16	0.16	0.13	0.01	0.14
0	1	0.91	1	t	1	Prunus spinosa	Eupsilia transversa	16	0.16	0.13	0.01	0.14
0	1	0.91	1	t	1	Prunus spinosa	Nola confusalis	16	0.16	0.13	0.01	0.14

0	1	0.91	1	t	1	Prunus spinosa	Orgyia antiqua	16	0.16	0.13	0.01	0.14
0	1	0.91	1	cv	1	Prunus spinosa	Opisthograptis luteolata	16	0.16	0.13	0.01	0.14
0	1	0.91	1	t	1	Prunus spinosa	Eupithecia exiguata	16	0.16	0.13	0.01	0.14
0	1	0.94	1	t	1	Prunus spinosa	Callimorpha dominula	16	0.16	0.13	0.02	0.14
0	1	0.91	1	t	1	Prunus spinosa	Colocasia coryli	16	0.16	0.13	0.01	0.14
0	1	0.91	1	cv	1	Prunus spinosa	Saturnia pavonia	16	0.16	0.13	0.01	0.14
0	1	0.91	1	t	1	Prunus spinosa	Orthosia gothica	16	0.16	0.13	0.01	0.14
0	1	0.91	1	t	1	Prunus spinosa	Agrochola lota	16	0.16	0.13	0.01	0.14
0	1	0.91	1	t	1	Prunus spinosa	Hydrelia sylvata	16	0.16	0.13	0.01	0.14
0	1	0.91	1	cv	1	Prunus spinosa	Electrophaes corylata	16	0.16	0.13	0.01	0.14
0	1	0.91	1	t	1	Prunus spinosa	Trichiura crataegi	16	0.16	0.13	0.01	0.14
0	1	0.91	1	t	1	Prunus spinosa	Agriopsis marginaria	16	0.16	0.13	0.01	0.14
0	1	0.91	1	t	1	Prunus spinosa	Apocheima pilosaria	16	0.16	0.13	0.01	0.14
0	1	0.94	1	cv	1	Prunus spinosa	Thalera fimbrialis	16	0.16	0.13	0.02	0.14
0	1	0.91	1	t	1	Prunus spinosa	Graphiphora augur	16	0.16	0.13	0.01	0.14
0	1	0.91	1	t	1	Prunus spinosa	Operophtera brumata	16	0.16	0.13	0.01	0.14
0	1	0.91	1	t	1	Prunus spinosa	Egira conspicillaris	16	0.16	0.13	0.01	0.14
0	1	0.91	1	cv	1	Prunus spinosa	Aleucis distincata	16	0.16	0.13	0.01	0.14
1	0	0.26	1	t	1	Nymphalis polychlorus	Prunus avium	16	0.01	0.08	0.04	0.08
1	0	0.24	1	t	1	Prunus avium	Selenia tetralunaria	16	0.04	0.08	0.01	0.08
0	0	0.03	0	t	1	Plagodis pulveraria	Eupithecia exiguata	16	0.01	0.11	0.01	0.14
0	0	0.04	0	cv	1	Thecla betulae	Orthosia gracilis	16	0.02	0.14	0.02	0.14
0	0	0.04	0	t	1	Thecla betulae	Strymonidia pruni	16	0.02	0.14	0.02	0.14
0	0	0.02	0	t	1	Eriogaster lanestris	Dasychira fascelina	16	0.01	0.14	0.01	0.13
0	0	0.04	0	t	1	Acronicta tridens	Phalera bucephala	16	0.02	0.14	0.02	0.13

0	0	0.02	0	cv	1	Epirrita christyi	Egira conspicillaris	16	0.01	0.14	0.01	0.14
0	0	0.04	0	t	1	Perconia strigillaria	Lomographa bimaculata	16	0.01	0.14	0.01	0.08
0	0	0.04	0	t	1	Lomographa bimaculata	Eupithecia exiguata	16	0.01	0.08	0.01	0.14
0	0	0.02	0	t	1	Selenia dentaria	Dasychira fascelina	16	0.01	0.14	0.01	0.13
0	0	0.04	0	cv	1	Chloroclystis rectangulata	Xestia baja	16	0.02	0.14	0.02	0.14
0	0	0.03	0	t	1	Aporia crataegi	Bison betularia	16	0.02	0.14	0.01	0.13
0	0	0.03	0	t	1	Orthosia gracilis	Eurois occulata	16	0.02	0.14	0.01	0.14
0	0	0.03	0	t	1	Triphosa dubitata	Diarsia mendica	16	0.01	0.10	0.01	0.14
0	0	0.02	0	cv	1	Orgyia antiqua	Thalera fimbrialis	16	0.01	0.14	0.02	0.14
0	0	0.03	0	t	1	Ennomos autumnaria	Catocala nupta	16	0.02	0.14	0.01	0.13
0	0	0.04	0	t	1	Xestia triangulum	Nymphalis polychlorus	16	0.01	0.14	0.01	0.08
0	0	0.03	0	t	1	Acronicta aceris	Aporia crataegi	16	0.01	0.13	0.02	0.14
0	0	0.02	0	cv	1	Ourapteryx sambucaria	Apocheima pilosaria	16	0.01	0.14	0.01	0.14
0	0	0.02	0	t	1	Epirrita dilutata	Nola confusalis	16	0.02	0.14	0.01	0.14
0	0	0.05	0	t	1	Prunus padus	Epirrita christyi	16	0.02	0.10	0.01	0.14
0	0	0.03	0	t	1	Orthosia gracilis	Orgyia antiqua	16	0.02	0.14	0.01	0.14
0	0	0.04	0	cv	1	Conistra ligula	Theria rupicaprarian	16	0.02	0.14	0.02	0.14
0	0	0.07	0	t	1	Phalera bucephala	Alsophila aescularia	16	0.02	0.13	0.01	0.08
0	0	0.04	0	t	1	Callimorpha dominula	Thalera fimbrialis	16	0.02	0.14	0.02	0.14
0	0	0.11	0	t	1	Erannis defoliaria	Prunus avium	16	0.01	0.14	0.04	0.08
0	0	0.16	0	cv	1	Ennomos autumnaria	Prunus avium	16	0.02	0.14	0.04	0.08
0	0	0.04	0	t	1	Theria rupicaprarian	Cosmia pyralina	16	0.02	0.14	0.02	0.14
0	0	0.03	0	t	1	Ennomos autumnaria	Lasiocampa trifolii	16	0.02	0.14	0.01	0.13
0	0	0.02	0	t	1	Eriogaster lanestris	Lasiocampa trifolii	16	0.01	0.14	0.01	0.13
0	0	0.03	0	cv	1	Triphosa dubitata	Acronicta strigosa	16	0.01	0.10	0.01	0.14

0	0	0.02	0	t	1	Aporia crataegi	Plemyria rubiginata	16	0.02	0.14	0.01	0.14
0	0	0.02	0	t	1	Abraxas grossulariata	Euproctis chrysorrhoea	16	0.02	0.14	0.01	0.14
0	0	0.02	0	t	1	Nola confusalis	Lithophane socia	16	0.01	0.14	0.01	0.13
0	0	0.02	0	cv	1	Orthosia munda	Opisthograptis luteolata	16	0.01	0.13	0.01	0.14
0	0	0.03	0	t	1	Orthosia gracilis	Graphiphora augur	16	0.02	0.14	0.01	0.14
0	0	0.04	0	t	1	Lomographa bimaculata	Agrochola lota	16	0.01	0.08	0.01	0.14
0	0	0.04	0	t	1	Abraxas grossulariata	Odontopera bidentata	16	0.02	0.14	0.02	0.14
0	0	0.04	0	cv	1	Orgyia recens	Alsophila aescularia	16	0.01	0.14	0.01	0.08
0	0	0.03	0	t	1	Naenia typica	Lasiocampa trifolii	16	0.02	0.14	0.01	0.13
0	0	0.02	0	t	1	Brachionycha sphinx	Nola confusalis	16	0.01	0.14	0.01	0.14
0	0	0.03	0	t	1	Diloba caeruleocephala	Nola confusalis	16	0.02	0.14	0.01	0.14
0	0	0.04	0	cv	1	Diloba caeruleocephala	Lasiocampa trifolii	16	0.02	0.14	0.01	0.13
0	0	0.04	0	t	1	Phalera bucephala	Selenia lunalaria	16	0.02	0.13	0.02	0.14
0	0	0.02	0	t	1	Agriopis aurantiaria	Agrochola lota	16	0.01	0.14	0.01	0.14
0	1	0.98	0	t	1	Prunus spinosa	Prunus cerasus	16	0.16	0.13	0.03	0.11
0	0	0.04	0	cv	1	Phalera bucephala	Xestia baja	16	0.02	0.13	0.02	0.14
1	0	0.03	0	t	1	Bison betularia	Prunus laurocerasus	16	0.01	0.13	0.01	0.12
0	0	0.02	0	t	1	Cilix glaucata	Campaea margaritata	16	0.01	0.14	0.01	0.14
0	0	0.03	0	t	1	Ourapteryx sambucaria	Phalera bucephala	16	0.01	0.14	0.02	0.13
0	0	0.02	0	cv	1	Ourapteryx sambucaria	Lasiocampa quercus	16	0.01	0.14	0.01	0.14
0	0	0.02	0	t	1	Malacosoma neustria	Eurois occulata	16	0.01	0.14	0.01	0.14
0	0	0.03	0	t	1	Plagodis pulveraria	Agriopsis marginaria	16	0.01	0.11	0.01	0.14
0	0	0.02	0	t	1	Selenia lunalaria	Hydrelia sylvata	16	0.02	0.14	0.01	0.14
0	0	0.02	0	cv	1	Acronicta psi	Plemyria rubiginata	16	0.02	0.14	0.01	0.14

0	0	0.06	0	t	1	Angerona prunaria	Alsophila aescularia	16	0.02	0.14	0.01	0.08
0	0	0.03	0	t	1	Acronicta psi	Xestia baja	16	0.02	0.14	0.02	0.14
0	0	0.02	0	t	1	Ourapteryx sambucaria	Orthosia gothica	16	0.01	0.14	0.01	0.14
0	0	0.02	0	cv	1	Saturnia pavonia	Aleucis distincata	16	0.01	0.14	0.01	0.14
0	0	0.02	0	t	1	Orthosia gothica	Graphiphora augur	16	0.01	0.14	0.01	0.14
0	0	0.02	0	t	1	Ennomos autumnaria	Lasiocampa quercus	16	0.02	0.14	0.01	0.14
0	0	0.04	0	t	1	Lomographa bimaculata	Eupsilia transversa	16	0.01	0.08	0.01	0.14
0	0	0.05	0	cv	1	Alsophila aescularia	Lasiocampa trifolii	16	0.01	0.08	0.01	0.13
0	0	0.02	0	t	1	Catocala nupta	Aleucis distincata	16	0.01	0.13	0.01	0.14
0	0	0.02	0	t	1	Epirrita dilutata	Epirrita christyi	16	0.02	0.14	0.01	0.14
0	0	0.02	0	t	1	Conistra ligula	Egira conspicillaris	16	0.02	0.14	0.01	0.14
0	0	0.02	0	cv	1	Diarsia mendica	Callimorpha dominula	16	0.01	0.14	0.02	0.14
0	0	0.03	0	t	1	Chloroclystis rectangulata	Xestia triangulum	16	0.02	0.14	0.01	0.14
0	0	0.02	0	t	1	Xestia triangulum	Dasycampa rubiginea	16	0.01	0.14	0.02	0.14
0	0	0.02	0	t	1	Aporia crataegi	Euproctis chrysorrhoea	16	0.02	0.14	0.01	0.14
0	0	0.11	0	cv	1	Eurois	Prunus avium	16	0.01	0.14	0.04	0.08
0	0	0.02	0	t	1	Cilix glaucata	Theria rupicaprarian	16	0.01	0.14	0.02	0.14
0	0	0.02	0	t	1	Callimorpha dominula	Agrochola lota	16	0.02	0.14	0.01	0.14
0	0	0.02	0	t	1	Allophyes oxycanthae	Apocheima pilosaria	16	0.02	0.14	0.01	0.14
0	1	0.71	0	cv	1	Prunus domestica	Colocasia coryli	16	0.12	0.11	0.01	0.14
0	0	0.04	0	t	1	Diarsia mendica	Lomographa bimaculata	16	0.01	0.14	0.01	0.08
0	0	0.02	0	t	1	Orthosia munda	Epirrita christyi	16	0.01	0.13	0.01	0.14
0	0	0.04	0	t	1	Cilix glaucata	Alsophila aescularia	16	0.01	0.14	0.01	0.08
0	0	0.06	0	cv	1	Lomographa bimaculata	Prunus laurocerasus	16	0.01	0.08	0.01	0.12
0	0	0.03	0	t	1	Amphipyra pyramidea	Lithophane socia	16	0.01	0.13	0.01	0.13
0	0	0.02	0	t	1	Lithophane semibrunnea	Opisthograpti s luteolata	16	0.01	0.13	0.01	0.14

0	0	0.02	0	t	1	Chloroclysta siterata	Agriopsis marginaria	16	0.01	0.14	0.01	0.14
0	0	0.02	0	cv	1	Diarsia mendica	Hydrelia sylvata	16	0.01	0.14	0.01	0.14
0	0	0.02	0	t	1	Orthosia munda	Eupithecia exiguata	16	0.01	0.13	0.01	0.14
0	0	0.03	0	t	1	Phalera bucephala	Trichiura crataegi	16	0.02	0.13	0.01	0.14
0	0	0.02	0	t	1	Naenia typica	Agriopsis marginaria	16	0.02	0.14	0.01	0.14
0	0	0.02	0	cv	1	Chloroclystis chloerata	Eupsilia transversa	16	0.01	0.14	0.01	0.14
0	0	0.06	0	t	1	Angerona prunaria	Selenia tetralunaria	16	0.02	0.14	0.01	0.08
0	0	0.02	0	t	1	Brachionycha sphinx	Aporia crataegi	16	0.01	0.14	0.02	0.14
0	0	0.02	0	t	1	Diarsia mendica	Selenia lunalaria	16	0.01	0.14	0.02	0.14
0	0	0.02	0	cv	1	Electrophaes corylata	Trichiura crataegi	16	0.01	0.14	0.01	0.14
0	0	0.04	0	t	1	Abraxas grossulariata	Strymonidia pruni	16	0.02	0.14	0.02	0.14
0	0	0.02	0	t	1	Stauropus fagi	Eurois occulata	16	0.01	0.14	0.01	0.14
0	0	0.04	0	t	1	Nola cucullatella	Thecla betulae	16	0.02	0.14	0.02	0.14
0	0	0.02	0	cv	1	Campaea margaritata	Cosmia pyralina	16	0.01	0.14	0.02	0.14
0	0	0.02	0	t	1	Xestia baja	Eupithecia exiguata	16	0.02	0.14	0.01	0.14
0	0	0.03	0	t	1	Diloba caeruleocepha la	Selenia dentaria	16	0.02	0.14	0.01	0.14
0	0	0.02	0	t	1	Malacosoma neustria	Epirrita dilutata	16	0.01	0.14	0.02	0.14
0	0	0.02	0	cv	1	Agriopis aurantiaria	Dasycampa rubiginea	16	0.01	0.14	0.02	0.14
0	0	0.02	0	t	1	Orgyia recens	Egira conspicillaris	16	0.01	0.14	0.01	0.14
0	0	0.03	0	t	1	Diloba caeruleocepha la	Egira conspicillaris	16	0.02	0.14	0.01	0.14
0	0	0.02	0	t	1	Agriopis aurantiaria	Plemyria rubiginata	16	0.01	0.14	0.01	0.14
0	1	0.97	0	cv	1	Phalera bucephala	Prunus spinosa	16	0.02	0.13	0.16	0.13
0	0	0.02	0	t	1	Crocallis elinguaria	Agriopsis marginaria	16	0.01	0.14	0.01	0.14
0	0	0.02	0	t	1	Bison betularia	Agriopsis marginaria	16	0.01	0.13	0.01	0.14

1	0	0.03	0	t	1	Aporia crataegi	Prunus laurocerasus	16	0.02	0.14	0.01	0.12
1	0	0.03	0	cv	1	Lasiocampa trifolii	Prunus laurocerasus	16	0.01	0.13	0.01	0.12
0	0	0.02	0	t	1	Angerona prunaria	Orgyia recens	16	0.02	0.14	0.01	0.14
0	0	0.02	0	t	1	Brachionycha sphinx	Orgyia antiqua	16	0.01	0.14	0.01	0.14
0	0	0.03	0	t	1	Plagodis pulveraria	Saturnia pavonia	16	0.01	0.11	0.01	0.14
0	0	0.04	0	cv	1	Lomographa bimaculata	Chloroclystis chloerata	16	0.01	0.08	0.01	0.14
0	0	0.02	0	t	1	Nola confusalis	Apocheima pilosaria	16	0.01	0.14	0.01	0.14
0	0	0.04	0	t	1	Dasycampa rubiginea	Conistra ligula	16	0.02	0.14	0.02	0.14
0	0	0.04	0	t	1	Agrochola lota	Selenia tetralunaria	16	0.01	0.14	0.01	0.08
0	0	0.02	0	cv	1	Diarsia mendica	Selenia dentaria	16	0.01	0.14	0.01	0.14
0	0	0.04	0	t	1	Saturnia pavonia	Selenia tetralunaria	16	0.01	0.14	0.01	0.08
0	0	0.03	0	t	1	Lithophane semibrunnea	Ennomos autumnaria	16	0.01	0.13	0.02	0.14
1	0	0.31	1	t	1	Caenohalictus sp. 3	Sisyrinchium arenarium	17	0.01	0.02	0.01	0.03
1	0	0.28	1	cv	1	Chilicola sp.	Adesmia conferta	17	0.01	0.03	0.01	0.04
0	0	0.29	1	t	1	Chilicola sp.	Senecio erucaeformis	17	0.01	0.03	0.01	0.03
0	1	0.61	1	t	1	Bibionidae sp.1	Cerastium arvense	17	0.00	0.04	0.05	0.04
1	1	0.72	1	t	1	Acaena pinnatifida	Scaeva melanostoma	17	0.00	0.04	0.06	0.03
1	0	0.26	1	cv	1	Mesonychium gayi	Adesmia conferta	17	0.00	0.03	0.01	0.04
1	0	0.29	1	t	1	Mesonychium gayi	Astragalus curvicaulis	17	0.00	0.03	0.01	0.03
1	0	0.35	1	t	1	Schizanthus hookeri	Villa verdensis	17	0.01	0.03	0.01	0.03
0	0	0.27	1	t	1	Schizanthus hookeri	Anthidium funereum	17	0.01	0.03	0.00	0.02
0	0	0.28	1	cv	1	Schizanthus hookeri	Centris cineraria	17	0.01	0.03	0.01	0.02
0	0	0.28	1	t	1	Schizanthus hookeri	Megachile semirufa	17	0.01	0.03	0.01	0.02
1	1	0.55	1	t	1	Trichocereus chiloensis	Liphanthus sp.2	17	0.02	0.02	0.02	0.02
1	0	0.45	1	t	1	Trichocereus chiloensis	Liphanthus sabulosus	17	0.02	0.02	0.01	0.02

1	1	0.81	1	cv	1	Scaeva melanostoma	Mustisia sinuata	17	0.06	0.03	0.02	0.04
1	1	0.70	1	t	1	Scaeva melanostoma	Berberis empetrifolia	17	0.06	0.03	0.00	0.04
1	1	0.75	1	t	1	Scaeva melanostoma	Haplopappus chysanthemifolius	17	0.06	0.03	0.01	0.04
1	1	0.80	1	t	1	Scaeva melanostoma	Calandrinia affinis	17	0.06	0.03	0.01	0.04
1	1	0.85	1	cv	1	Scaeva melanostoma	Barneoudia major	17	0.06	0.03	0.02	0.04
1	1	0.71	1	t	1	Scaeva melanostoma	Chuquiraga oppositifolia	17	0.06	0.03	0.00	0.03
1	1	0.78	1	t	1	Scaeva melanostoma	Adesmia brachysemon	17	0.06	0.03	0.01	0.04
1	1	0.69	1	t	1	Scaeva melanostoma	Erodium cicutarium	17	0.06	0.03	0.00	0.04
1	1	0.73	1	cv	1	Scaeva melanostoma	Mulinum spinosum	17	0.06	0.03	0.01	0.04
1	1	0.73	1	t	1	Scaeva melanostoma	Sisyrinchium philippii	17	0.06	0.03	0.01	0.04
1	1	0.70	1	t	1	Scaeva melanostoma	Valeriana graciIeps	17	0.06	0.03	0.00	0.04
1	1	0.76	1	t	1	Scaeva melanostoma	Adesmia conferta	17	0.06	0.03	0.01	0.04
1	1	0.69	1	cv	1	Scaeva melanostoma	Senecio lithostaurus	17	0.06	0.03	0.00	0.04
1	1	0.72	1	t	1	Scaeva melanostoma	Leuceria landbeckii	17	0.06	0.03	0.01	0.04
1	1	0.81	1	t	1	Scaeva melanostoma	Haplopappus sericeus	17	0.06	0.03	0.02	0.04
1	1	0.79	1	t	1	Scaeva melanostoma	Adesmia aconcaguensis	17	0.06	0.03	0.01	0.04
1	1	0.69	1	cv	1	Scaeva melanostoma	Bowlesia tropaeolifolia	17	0.06	0.03	0.00	0.04
1	1	0.79	1	t	1	Scaeva melanostoma	Calandrinia uspallatensis	17	0.06	0.03	0.01	0.04
1	1	0.78	1	t	1	Scaeva melanostoma	Stachys albicaulis	17	0.06	0.03	0.01	0.03
1	1	0.69	1	t	1	Scaeva melanostoma	Chaetanthera microphylla	17	0.06	0.03	0.00	0.04
1	1	0.74	1	cv	1	Scaeva melanostoma	Senecio bustillosianus	17	0.06	0.03	0.01	0.03
1	1	0.71	1	t	1	Scaeva melanostoma	Adesmia radicifolia	17	0.06	0.03	0.00	0.04
1	1	0.95	1	t	1	Scaeva melanostoma	Cerastium arvense	17	0.06	0.03	0.05	0.04
1	1	0.83	1	t	1	Scaeva melanostoma	Phacelia secunda	17	0.06	0.03	0.02	0.04

1	1	0.87	1	cv	1	Scaeva melanostoma	Euphorbia portulacoides	17	0.06	0.03	0.03	0.04
1	1	0.70	1	t	1	Scaeva melanostoma	Sanicula graveolens	17	0.06	0.03	0.00	0.04
1	1	0.70	1	t	1	Scaeva melanostoma	Nothoscordum andinum	17	0.06	0.03	0.00	0.04
1	1	0.84	1	t	1	Scaeva melanostoma	Chaetanthera euphrasioides	17	0.06	0.03	0.02	0.04
1	1	0.69	1	cv	1	Scaeva melanostoma	Tetraglochin alatum	17	0.06	0.03	0.00	0.04
1	1	0.73	1	t	1	Scaeva melanostoma	Hypochoeris sp.	17	0.06	0.03	0.01	0.04
1	1	0.77	1	t	1	Scaeva melanostoma	Quinchamalium chilensis	17	0.06	0.03	0.01	0.04
1	0	0.33	1	t	1	Scaptia atra	Phacelia secunda	17	0.00	0.03	0.02	0.04
1	0	0.36	1	cv	1	Mustisia sinuata	Bombus dahlbomii	17	0.02	0.04	0.01	0.03
1	0	0.29	1	t	1	Mustisia sinuata	Isepeolus niveiventris	17	0.02	0.04	0.00	0.03
1	0	0.47	1	t	1	Mustisia sinuata	Etcheverrius chilensis	17	0.02	0.04	0.02	0.02
1	0	0.42	1	t	1	Mustisia sinuata	Megachile sp. 1	17	0.02	0.04	0.01	0.03
1	0	0.34	1	cv	1	Mustisia sinuata	Megachile semirufa	17	0.02	0.04	0.01	0.02
1	0	0.38	1	t	1	Tropaeolum tricolor	Hymenoptera n.i.	17	0.01	0.02	0.00	0.02
1	0	0.32	1	t	1	Berberis empetrifolia	Sarcophagidae n.i.	17	0.00	0.04	0.02	0.03
1	0	0.26	1	t	1	Berberis empetrifolia	Tachinidae n.i.	17	0.00	0.04	0.01	0.03
1	1	0.59	1	cv	1	Berberis empetrifolia	Hypodynerus chiliotus	17	0.00	0.04	0.05	0.04
1	0	0.42	1	t	1	Berberis empetrifolia	Caenohalictus sp.	17	0.00	0.04	0.03	0.03
1	1	0.55	1	t	1	Vibrissomyia sp.	Nassauvia heterophylla	17	0.00	0.03	0.04	0.03
1	0	0.34	1	t	1	Bombyllidae n.i.	Senecio francisci	17	0.00	0.01	0.01	0.02
1	0	0.50	1	cv	1	Tabanidae n.i.	Euphorbia portulacoides	17	0.01	0.03	0.03	0.04
1	0	0.23	1	t	1	Alstromemeria pallida	Alloscirtetica sp.	17	0.00	0.03	0.00	0.03
1	0	0.25	1	t	1	Alstromemeria pallida	Anthidium funereum	17	0.00	0.03	0.00	0.02

1	0	0.36	1	t	1	Alstromemeria pallida	Alloscirtetica gayi	17	0.00	0.03	0.02	0.02
1	0	0.31	1	cv	1	Alstromemeria pallida	Panurgineae sp.	17	0.00	0.03	0.01	0.02
1	0	0.26	1	t	1	Alstromemeria pallida	Megachile semirufa	17	0.00	0.03	0.01	0.02
1	0	0.27	1	t	1	Clarkia tenella	Hymenoptera n.i.	17	0.00	0.02	0.00	0.02
1	0	0.31	1	t	1	Corynura sp.	Madia sativa	17	0.00	0.01	0.01	0.02
1	1	0.54	1	cv	1	Villa arenarius	Chaetanthera euphrasioides	17	0.02	0.03	0.02	0.04
1	0	0.32	1	t	1	Villa sp.	Mulinum spinosum	17	0.01	0.03	0.01	0.04
1	1	0.66	1	t	1	Villa sp.	Nassauvia heterophylla	17	0.01	0.03	0.04	0.03
1	0	0.39	1	t	1	Villa sp.	Adesmia aconcaguensis	17	0.01	0.03	0.01	0.04
1	0	0.39	1	cv	1	Villa sp.	Calandrinia uspallatensis	17	0.01	0.03	0.01	0.04
1	0	0.32	1	t	1	Villa sp.	Senecio bustillosianus	17	0.01	0.03	0.01	0.03
1	0	0.31	1	t	1	Villa sp.	Verbena scoparia	17	0.01	0.03	0.00	0.03
1	0	0.47	1	t	1	Villa sp.	Chaetanthera euphrasioides	17	0.01	0.03	0.02	0.04
1	0	0.31	1	cv	1	Villa sp.	Hypochoeris sp.	17	0.01	0.03	0.01	0.04
1	0	0.36	1	t	1	Astragalus sp.	Hymenoptera n.i.	17	0.02	0.03	0.00	0.02
1	1	0.76	1	t	1	Astragalus sp.	Anthidium gayi	17	0.02	0.03	0.05	0.03
1	0	0.35	1	t	1	Astragalus sp.	Alloscirtetica sp.	17	0.02	0.03	0.00	0.03
1	1	0.67	1	cv	1	Astragalus sp.	Megachile sp. 2	17	0.02	0.03	0.03	0.03
1	0	0.40	1	t	1	Astragalus sp.	Pseudolucia chilensis	17	0.02	0.03	0.01	0.03
0	0	0.46	1	t	1	Sarcophagidae n.i.	Sisyrinchium junceum	17	0.02	0.03	0.01	0.03
1	0	0.44	1	t	1	Sarcophagidae n.i.	Calandrinia affinis	17	0.02	0.03	0.01	0.04
1	1	0.52	1	cv	1	Sarcophagidae n.i.	Barneoudia major	17	0.02	0.03	0.02	0.04
1	0	0.31	1	t	1	Sarcophagidae n.i.	Bowlesia tropaeolifolia	17	0.02	0.03	0.00	0.04

1	0	0.36	1	t	1	Sarcophagidae n.i.	Senecio bustillosianus	17	0.02	0.03	0.01	0.03
1	0	0.43	1	t	1	Sarcophagidae n.i.	Astragalus curvicaulis	17	0.02	0.03	0.01	0.03
1	0	0.25	1	cv	1	Bombus dahlbomii	Chuquiraga oppositifolia	17	0.01	0.03	0.00	0.03
1	0	0.26	1	t	1	Bombus dahlbomii	Loasa heterophylla	17	0.01	0.03	0.00	0.03
1	0	0.33	1	t	1	Bombus dahlbomii	Stachys albicaulis	17	0.01	0.03	0.01	0.03
0	0	0.31	1	t	1	Bombus dahlbomii	Senecio erucaeformis	17	0.01	0.03	0.01	0.03
1	0	0.28	1	cv	1	Bombus dahlbomii	Senecio bustillosianus	17	0.01	0.03	0.01	0.03
0	0	0.40	1	t	1	Bombus dahlbomii	Phacelia secunda	17	0.01	0.03	0.02	0.04
1	0	0.39	1	t	1	Lycophlaeba lugubris	Verbena scoparia	17	0.02	0.03	0.00	0.03
1	1	0.56	1	t	1	Lycophlaeba lugubris	Chaetanthera euphrasioide s	17	0.02	0.03	0.02	0.04
1	0	0.44	1	cv	1	Lycophlaeba lugubris	Quinchamaliu m chilensis	17	0.02	0.03	0.01	0.04
0	0	0.25	1	t	1	Haplopappus chysanthemifo lius	Hymenoptera n.i.	17	0.01	0.04	0.00	0.02
0	0	0.32	1	t	1	Haplopappus chysanthemifo lius	Lepidoptera n.i.	17	0.01	0.04	0.01	0.02
1	0	0.39	1	t	1	Adesmia exilis	Xanthocollete s incahuasi	17	0.01	0.02	0.01	0.02
1	1	0.50	1	cv	1	Adesmia exilis	Xanthocollete s sicheli	17	0.01	0.02	0.03	0.03
1	0	0.31	1	t	1	Adesmia exilis	Xanthocollete s sp.	17	0.01	0.02	0.00	0.02
1	0	0.37	1	t	1	Centris sp.	Calceolaria biflora	17	0.00	0.00	0.00	0.00
1	0	0.25	1	t	1	Villa semifuscata	Chuquiraga oppositifolia	17	0.01	0.03	0.00	0.03
1	0	0.34	1	cv	1	Villa semifuscata	Adesmia aconcaguensi s	17	0.01	0.03	0.01	0.04
1	0	0.42	1	t	1	Villa semifuscata	Chaetanthera euphrasioide s	17	0.01	0.03	0.02	0.04
1	0	0.32	1	t	1	Diptera n.i.	Calandrinia affinis	17	0.01	0.03	0.01	0.04
0	0	0.40	1	t	1	Diptera n.i.	Barneoudia major	17	0.01	0.03	0.02	0.04

0	0	0.29	1	cv	1	Diptera n.i.	Madia sativa	17	0.01	0.03	0.01	0.02
0	0	0.26	1	t	1	Diptera n.i.	Viola atropurpurea	17	0.01	0.03	0.00	0.02
0	0	0.21	1	t	1	Diptera n.i.	Erodium cicutarium	17	0.01	0.03	0.00	0.04
0	0	0.25	1	t	1	Diptera n.i.	Mulinum spinosum	17	0.01	0.03	0.01	0.04
1	0	0.39	1	cv	1	Diptera n.i.	Nardophyllu m lanatum	17	0.01	0.03	0.02	0.02
0	0	0.31	1	t	1	Diptera n.i.	Tristagama sessile	17	0.01	0.03	0.01	0.02
0	0	0.22	1	t	1	Diptera n.i.	Valeriana graciléps	17	0.01	0.03	0.00	0.04
1	1	0.58	1	t	1	Diptera n.i.	Nassauvia heterophylla	17	0.01	0.03	0.04	0.03
1	0	0.32	1	cv	1	Diptera n.i.	Adesmia aconcaguensi s	17	0.01	0.03	0.01	0.04
0	0	0.22	1	t	1	Diptera n.i.	Bowlesia tropaeolifolia	17	0.01	0.03	0.00	0.04
1	0	0.41	1	t	1	Diptera n.i.	Cardamine nivalis	17	0.01	0.03	0.02	0.02
1	0	0.32	1	t	1	Diptera n.i.	Calandrinia uspallatensis	17	0.01	0.03	0.01	0.04
0	0	0.31	1	cv	1	Diptera n.i.	Berberis montana	17	0.01	0.03	0.01	0.02
1	0	0.29	1	t	1	Diptera n.i.	Senecio erucaeformis	17	0.01	0.03	0.01	0.03
1	0	0.25	1	t	1	Diptera n.i.	Senecio bustillosianus	17	0.01	0.03	0.01	0.03
0	1	0.68	1	t	1	Diptera n.i.	Cerastium arvense	17	0.01	0.03	0.05	0.04
0	0	0.44	1	cv	1	Diptera n.i.	Euphorbia portulacoides	17	0.01	0.03	0.03	0.04
0	0	0.22	1	t	1	Diptera n.i.	Sanicula graveolens	17	0.01	0.03	0.00	0.04
1	0	0.24	1	t	1	Diptera n.i.	Verbena scoparia	17	0.01	0.03	0.00	0.03
0	0	0.22	1	t	1	Diptera n.i.	Nothoscordu m andinum	17	0.01	0.03	0.00	0.04
1	0	0.36	1	cv	1	Diptera n.i.	Asteriscium aemocarpon	17	0.01	0.03	0.01	0.02
0	0	0.28	1	t	1	Diptera n.i.	Barneoudia chilensis	17	0.01	0.03	0.01	0.03
0	0	0.28	1	t	1	Diptera n.i.	Oxalis sp.2	17	0.01	0.03	0.01	0.04
1	0	0.27	1	t	1	Diptera n.i.	Tristagama bivalve	17	0.01	0.03	0.01	0.03
0	0	0.29	1	cv	1	Diptera n.i.	Quinchamaliu m chilensis	17	0.01	0.03	0.01	0.04

1	0	0.25	1	t	1	Lithurgomma wagenknechti	Adesmia conferta	17	0.00	0.03	0.01	0.04
0	0	0.33	1	t	1	Lithurgomma wagenknechti	Phacelia secunda	17	0.00	0.03	0.02	0.04
1	1	0.61	1	t	1	Sisyrinchium junceum	Elaphroptera relicta	17	0.01	0.03	0.03	0.03
1	0	0.38	1	cv	1	Sisyrinchium junceum	Tachinidae n.i.	17	0.01	0.03	0.01	0.03
0	0	0.41	1	t	1	Sisyrinchium junceum	Coleoptera n.i.	17	0.01	0.03	0.02	0.04
1	1	0.55	1	t	1	Sisyrinchium junceum	Caenohalictu s sp.	17	0.01	0.03	0.03	0.03
1	0	0.31	1	t	1	Adesmia montana	Liphanthus sabulosus	17	0.00	0.02	0.01	0.02
1	0	0.37	1	cv	1	Hippeastrum uniflorum	Troch n.i.	17	0.00	0.00	0.00	0.00
1	0	0.34	1	t	1	Leucocoryne pauciflora	Lepidoptera n.i.	17	0.00	0.02	0.01	0.02
1	0	0.28	1	t	1	Yramea lathonoides	Collomia biflora	17	0.01	0.03	0.00	0.03
1	0	0.38	1	t	1	Yramea lathonoides	Haplopappus sericeus	17	0.01	0.03	0.02	0.04
1	0	0.27	1	cv	1	Yramea lathonoides	Anarthrophyll um cumingii	17	0.01	0.03	0.00	0.03
1	0	0.32	1	t	1	Villa verdensis	Mulinum spinosum	17	0.01	0.03	0.01	0.04
1	1	0.66	1	t	1	Villa verdensis	Nassauvia heterophylla	17	0.01	0.03	0.04	0.03
1	0	0.33	1	t	1	Villa verdensis	Senecio bustillosianus	17	0.01	0.03	0.01	0.03
1	0	0.45	1	cv	1	Villa verdensis	Phacelia secunda	17	0.01	0.03	0.02	0.04
1	0	0.25	1	t	1	Coelioxys sp.	Scypanthus elegans	17	0.00	0.02	0.00	0.03
1	0	0.27	1	t	1	Tropaeolum sessilifolium	Hymenoptera n.i.	17	0.01	0.03	0.00	0.02
1	0	0.36	1	t	1	Tropaeolum sessilifolium	Megachile sp. 1	17	0.01	0.03	0.01	0.03
1	0	0.26	1	cv	1	Calceolaria montana	Hymenoptera n.i.	17	0.01	0.03	0.00	0.02
1	1	0.56	1	t	1	Calceolaria montana	Megachile sp. 2	17	0.01	0.03	0.03	0.03
1	1	0.52	1	t	1	Calceolaria montana	Centris nigerrima	17	0.01	0.03	0.03	0.03
1	0	0.32	1	t	1	Hymenoptera n.i.	Calandrinia affinis	17	0.00	0.02	0.01	0.04
0	0	0.45	1	cv	1	Hymenoptera n.i.	Solenomelus sisyrinchium	17	0.00	0.02	0.02	0.02
1	0	0.27	1	t	1	Hymenoptera n.i.	Sisyrinchium arenarium	17	0.00	0.02	0.01	0.03

0	0	0.29	1	t	1	Hymenoptera n.i.	Oxalis geminata	17	0.00	0.02	0.01	0.03
1	0	0.40	1	t	1	Hymenoptera n.i.	Lupinus microcarpus	17	0.00	0.02	0.02	0.02
1	0	0.27	1	cv	1	Hymenoptera n.i.	Salpiglossus sp.	17	0.00	0.02	0.00	0.02
1	0	0.38	1	t	1	Hymenoptera n.i.	Calceolaria sp.	17	0.00	0.02	0.01	0.02
0	0	0.30	1	t	1	Hymenoptera n.i.	Berberis montana	17	0.00	0.02	0.01	0.02
1	0	0.27	1	t	1	Hymenoptera n.i.	Solanum tomatillo	17	0.00	0.02	0.00	0.02
1	0	0.27	1	cv	1	Hymenoptera n.i.	Loasa sigmoidea	17	0.00	0.02	0.00	0.02
1	0	0.25	1	t	1	Hymenoptera n.i.	Perezia carthamoides	17	0.00	0.02	0.00	0.03
1	0	0.25	1	t	1	Hymenoptera n.i.	Senecio bustillosianus	17	0.00	0.02	0.01	0.03
0	0	0.36	1	t	1	Hymenoptera n.i.	Phacelia secunda	17	0.00	0.02	0.02	0.04
1	0	0.24	1	cv	1	Hymenoptera n.i.	Verbena scoparia	17	0.00	0.02	0.00	0.03
0	0	0.27	1	t	1	Hymenoptera n.i.	Barneoudia chilensis	17	0.00	0.02	0.01	0.03
0	0	0.27	1	t	1	Hymenoptera n.i.	Oxalis sp.2	17	0.00	0.02	0.01	0.04
1	0	0.33	1	t	1	Colletes araucariae	Phacelia secunda	17	0.00	0.03	0.02	0.04
1	0	0.32	1	cv	1	Prosopochaet a caliginosa	Calandrinia affinis	17	0.01	0.03	0.01	0.04
1	0	0.22	1	t	1	Prosopochaet a caliginosa	Sanicula graveolens	17	0.01	0.03	0.00	0.04
1	0	0.33	1	t	1	Hypodynerus sp.2	Calandrinia affinis	17	0.01	0.02	0.01	0.04
1	0	0.25	1	t	1	Hypodynerus sp.2	Anarthrophyll um cumingii	17	0.01	0.02	0.00	0.03
1	0	0.36	1	cv	1	Ruizantheda mutabilis	Phacelia secunda	17	0.01	0.03	0.02	0.04
1	0	0.24	1	t	1	Ruizantheda mutabilis	Hypochoeris sp.	17	0.01	0.03	0.01	0.04
1	0	0.26	1	t	1	Villa durvillei	Verbena scoparia	17	0.00	0.01	0.00	0.03
1	0	0.28	1	t	1	Andinus venustus	Senecio erucaeformis	17	0.00	0.02	0.01	0.03
1	1	0.53	1	cv	1	Calandrinia affinis	Anthomyiida e sp.2	17	0.01	0.04	0.03	0.03
1	0	0.29	1	t	1	Calandrinia affinis	Villa hypoxantha	17	0.01	0.04	0.00	0.03
1	0	0.29	1	t	1	Calandrinia affinis	Phulia nymphula	17	0.01	0.04	0.00	0.03

1	0	0.35	1	t	1	Calandrinia affinis	Agromyzidae sp.1	17	0.01	0.04	0.01	0.03
1	0	0.35	1	cv	1	Calandrinia affinis	Agromyzidae sp.2	17	0.01	0.04	0.01	0.03
0	0	0.26	1	t	1	Calandrinia affinis	Habromyia lipoflava	17	0.01	0.04	0.00	0.04
1	1	0.54	1	t	1	Calandrinia affinis	Caenohalictu s sp.	17	0.01	0.04	0.03	0.03
1	0	0.34	1	t	1	Barneoudia major	Dasybasis sp. 2	17	0.02	0.04	0.00	0.03
1	0	0.35	1	cv	1	Barneoudia major	Dasybasis sp. 1	17	0.02	0.04	0.00	0.03
1	0	0.31	1	t	1	Madia sativa	Dialictus sp.	17	0.01	0.02	0.00	0.01
1	0	0.46	1	t	1	Madia sativa	Hemicolletes ruizi	17	0.01	0.02	0.02	0.02
0	0	0.31	1	t	1	Madia sativa	Centris cineraria	17	0.01	0.02	0.01	0.02
1	1	0.65	1	cv	1	Anthidium gayi	Adesmia conferta	17	0.05	0.03	0.01	0.04
1	1	0.68	1	t	1	Anthidium gayi	Stachys albicaulis	17	0.05	0.03	0.01	0.03
1	1	0.74	1	t	1	Anthidium gayi	Phacelia secunda	17	0.05	0.03	0.02	0.04
1	1	0.69	1	t	1	Anthidium gayi	Astragalus curvicaulis	17	0.05	0.03	0.01	0.03
1	0	0.26	1	cv	1	Alloscirtetica sp.	Adesmia conferta	17	0.00	0.03	0.01	0.04
1	0	0.20	1	t	1	Alloscirtetica sp.	Senecio lithostaurus	17	0.00	0.03	0.00	0.04
1	0	0.28	1	t	1	Alloscirtetica sp.	Stachys albicaulis	17	0.00	0.03	0.01	0.03
1	0	0.22	1	t	1	Alloscirtetica sp.	Scypanthus elegans	17	0.00	0.03	0.00	0.03
1	0	0.21	1	cv	1	Alloscirtetica sp.	Adesmia radicifolia	17	0.00	0.03	0.00	0.04
0	0	0.34	1	t	1	Alloscirtetica sp.	Phacelia secunda	17	0.00	0.03	0.02	0.04
1	0	0.29	1	t	1	Alloscirtetica sp.	Astragalus curvicaulis	17	0.00	0.03	0.01	0.03
1	0	0.30	1	t	1	Xanthocolletes incahuasi	Adesmia radicifolia	17	0.01	0.02	0.00	0.04
1	0	0.27	1	cv	1	Sisyrinchium arenarium	Tapinotaspis herbsti	17	0.01	0.03	0.00	0.02
1	0	0.27	1	t	1	Sisyrinchium arenarium	Tapinotaspis caerulea	17	0.01	0.03	0.00	0.02
1	0	0.46	1	t	1	Sisyrinchium arenarium	Caenohalictu s sp. 2	17	0.01	0.03	0.02	0.02
1	1	0.56	1	t	1	Sisyrinchium arenarium	Megachile sp. 2	17	0.01	0.03	0.03	0.03
1	0	0.42	1	cv	1	Sisyrinchium arenarium	Anthidium chilensis	17	0.01	0.03	0.02	0.03

1	0	0.49	1	t	1	Sisyrinchium arenarium	Caenohalictus sp.	17	0.01	0.03	0.03	0.03
1	0	0.27	1	t	1	Schizanthus garhamii	Centris cineraria	17	0.00	0.03	0.01	0.02
1	0	0.40	1	t	1	Anthomyiidae sp.2	Sanicula graveolens	17	0.03	0.03	0.00	0.04
1	0	0.35	1	cv	1	Chuquiraga oppositifolia	Etcheverrius chilensis	17	0.00	0.03	0.02	0.02
1	0	0.32	1	t	1	Chuquiraga oppositifolia	Tatochila mercedis	17	0.00	0.03	0.02	0.03
1	0	0.23	1	t	1	Chuquiraga oppositifolia	Anthidium funereum	17	0.00	0.03	0.00	0.02
1	0	0.22	1	t	1	Chuquiraga oppositifolia	Tatochila blanchardii	17	0.00	0.03	0.00	0.02
1	0	0.25	1	cv	1	Chuquiraga oppositifolia	Faunula leucoglene	17	0.00	0.03	0.01	0.03
1	0	0.30	1	t	1	Chuquiraga oppositifolia	Megachile sp. 1	17	0.00	0.03	0.01	0.03
1	0	0.25	1	t	1	Chuquiraga oppositifolia	Auca coctei-pales	17	0.00	0.03	0.01	0.02
1	0	0.47	1	t	1	Chuquiraga oppositifolia	Centris nigerrima	17	0.00	0.03	0.03	0.03
1	1	0.57	1	cv	1	Adesmia brachysemon	Anthidium decaspilum	17	0.01	0.04	0.03	0.03
1	0	0.48	1	t	1	Adesmia brachysemon	Xanthocolletes sicheli	17	0.01	0.04	0.03	0.03
1	0	0.30	1	t	1	Adesmia brachysemon	Centris cineraria	17	0.01	0.04	0.01	0.02
1	0	0.30	1	t	1	Adesmia brachysemon	Megachile semirufa	17	0.01	0.04	0.01	0.02
1	0	0.31	1	cv	1	Viola atropurpurea	Chaetodemoticus chilensis	17	0.00	0.02	0.01	0.01
1	0	0.44	1	t	1	Tropaeolum polyphyllum	Megachile semirufa	17	0.02	0.02	0.01	0.02
1	0	0.31	1	t	1	Loasa heterophylla	Megachile sp. 1	17	0.00	0.03	0.01	0.03
1	0	0.38	1	t	1	Loasa heterophylla	Anthidium chilensis	17	0.00	0.03	0.02	0.03
1	0	0.25	1	cv	1	Loasa heterophylla	Megachile semirufa	17	0.00	0.03	0.01	0.02
1	1	0.54	1	t	1	Anthidium decaspilum	Adesmia conferta	17	0.03	0.03	0.01	0.04
1	0	0.47	1	t	1	Anthidium decaspilum	Adesmia radicifolia	17	0.03	0.03	0.00	0.04
1	1	0.64	1	t	1	Anthidium decaspilum	Phacelia secunda	17	0.03	0.03	0.02	0.04
1	0	0.23	1	cv	1	Mulinum spinosum	Villa hypoxantha	17	0.01	0.04	0.00	0.03
1	0	0.26	1	t	1	Mulinum spinosum	Auca coctei-pales	17	0.01	0.04	0.01	0.02

1	0	0.30	1	t	1	Mulinum spinosum	Villa guttapennis	17	0.01	0.04	0.01	0.02
1	0	0.30	1	t	1	Mulinum spinosum	Panurgineae sp.	17	0.01	0.04	0.01	0.02
1	0	0.43	1	cv	1	Tristagama nivale	Lepidoptera n.i.	17	0.01	0.01	0.01	0.02
1	0	0.32	1	t	1	Lepidium suffruticosum	Ruiziella luctuosa	17	0.00	0.01	0.01	0.02
1	0	0.42	1	t	1	Vespidae n.i.	Euphorbia portulacoides	17	0.01	0.03	0.03	0.04
1	0	0.41	1	t	1	Nardophyllum lanatum	Auca coctei-pales	17	0.02	0.02	0.01	0.02
1	0	0.36	1	cv	1	Tachinidae n.i.	Senecio francisci	17	0.01	0.03	0.01	0.02
0	0	0.50	1	t	1	Tachinidae n.i.	Euphorbia portulacoides	17	0.01	0.03	0.03	0.04
1	0	0.34	1	t	1	Lathyrus subandinus	Centris cineraria	17	0.01	0.02	0.01	0.02
1	0	0.23	1	t	1	Spathipalpus philippii	Senecio bustillosianus	17	0.00	0.03	0.01	0.03
1	0	0.36	1	cv	1	Spathipalpus philippii	Chaetanthera euphrasioide s	17	0.00	0.03	0.02	0.04
1	0	0.41	1	t	1	Lepidoptera n.i.	Oenothera acaulis	17	0.01	0.02	0.01	0.02
1	0	0.40	1	t	1	Lepidoptera n.i.	Mustisia acerosa	17	0.01	0.02	0.01	0.02
1	0	0.34	1	t	1	Lepidoptera n.i.	Leucocoryne ixioides	17	0.01	0.02	0.00	0.02
1	0	0.31	1	cv	1	Sisyrinchium philippii	Coleoptera n.i.	17	0.01	0.04	0.02	0.04
1	0	0.25	1	t	1	Sisyrinchium philippii	Centris cineraria	17	0.01	0.04	0.01	0.02
1	0	0.44	1	t	1	Sisyrinchium philippii	Caenohalictu s sp.	17	0.01	0.04	0.03	0.03
1	0	0.36	1	t	1	Etcheverrius chilensis	Mustisia subulata	17	0.02	0.02	0.00	0.03
1	0	0.48	1	cv	1	Etcheverrius chilensis	Haplopappus sericeus	17	0.02	0.02	0.02	0.04
0	0	0.41	1	t	1	Etcheverrius chilensis	Senecio erucaeformis	17	0.02	0.02	0.01	0.03
1	0	0.37	1	t	1	Etcheverrius chilensis	Perezia carthamoides	17	0.02	0.02	0.00	0.03
1	0	0.38	1	t	1	Etcheverrius chilensis	Senecio bustillosianus	17	0.02	0.02	0.01	0.03
1	0	0.47	1	cv	1	Etcheverrius chilensis	Mustisia acerosa	17	0.02	0.02	0.01	0.02

1	0	0.36	1	t	1	Etcheverrius chilensis	Anarthrophyllum cumingii	17	0.02	0.02	0.00	0.03
1	0	0.26	1	t	1	Mustisia subulata	Auca coctei-pales	17	0.00	0.03	0.01	0.02
1	0	0.38	1	t	1	Mustisia subulata	Anthidium chilensis	17	0.00	0.03	0.02	0.03
1	0	0.27	1	cv	1	Eurygostomyia philippii	Stachys albicaulis	17	0.00	0.03	0.01	0.03
1	0	0.19	1	t	1	Eurygostomyia philippii	Chaetanthera microphylla	17	0.00	0.03	0.00	0.04
1	0	0.49	1	t	1	Calceolaria arachnoides	Centris nigerrima	17	0.00	0.03	0.03	0.03
0	0	0.36	1	t	1	Oxalis geminata	Coleoptera n.i.	17	0.01	0.03	0.02	0.04
1	1	0.54	1	cv	1	Oxalis	Muscidae sp.	17	0.01	0.03	0.03	0.03
1	0	0.27	1	t	1	Valeriana graciIeps	Anthomyiidae sp.4	17	0.00	0.04	0.01	0.02
1	1	0.61	1	t	1	Svastrides melanura	Phacelia secunda	17	0.03	0.03	0.02	0.04
1	0	0.24	1	t	1	Caupolicana dimidiata	Adesmia radicifolia	17	0.01	0.02	0.00	0.04
1	1	0.56	1	cv	1	Adesmia conferta	Megachile sp. 2	17	0.01	0.04	0.03	0.03
1	0	0.28	1	t	1	Adesmia conferta	Centris cineraria	17	0.01	0.04	0.01	0.02
1	0	0.30	1	t	1	Adesmia conferta	Pseudolucia chilensis	17	0.01	0.04	0.01	0.03
1	0	0.42	1	t	1	Adesmia conferta	Anthidium chilensis	17	0.01	0.04	0.02	0.03
1	0	0.30	1	cv	1	Adesmia conferta	Alloscirtetica rufitarsis	17	0.01	0.04	0.01	0.03
1	0	0.32	1	t	1	Adesmia conferta	Cadeguala occidentalis	17	0.01	0.04	0.01	0.03
1	0	0.47	1	t	1	Villa gayi	Chaetanthera euphrasioides	17	0.01	0.03	0.02	0.04
1	0	0.23	1	t	1	Senecio lithostaurus	Auca coctei-pales	17	0.00	0.04	0.01	0.02
1	1	0.52	1	cv	1	Nassauvia heterophylla	Dasybasis sp. 1	17	0.04	0.03	0.00	0.03
1	0	0.37	1	t	1	Hypsochila wagenknecti	Senecio francisci	17	0.01	0.01	0.01	0.02
1	0	0.31	1	t	1	Leuceria landbeckii	Megachile sp. 1	17	0.01	0.04	0.01	0.03
1	0	0.34	1	t	1	Chilimelissa sp.	Adesmia aconcaguensis	17	0.01	0.03	0.01	0.04
1	0	0.39	1	cv	1	Tatochila mercedis	Senecio erucaeformis	17	0.02	0.03	0.01	0.03

1	0	0.35	1	t	1	Tatochila mercedis	Senecio bustillosianus	17	0.02	0.03	0.01	0.03
1	0	0.34	1	t	1	Tatochila mercedis	Hypochoeris sp.	17	0.02	0.03	0.01	0.04
1	0	0.35	1	t	1	Haplopappus sericeus	Auca coctei-pales	17	0.02	0.04	0.01	0.02
1	0	0.40	1	cv	1	Haplopappus sericeus	Panurgineae sp.	17	0.02	0.04	0.01	0.02
1	0	0.34	1	t	1	Haplopappus sericeus	Megachile semirufa	17	0.02	0.04	0.01	0.02
1	1	0.51	1	t	1	Adesmia aconcaguensis	Xanthocollete s sicheli	17	0.01	0.04	0.03	0.03
0	0	0.34	1	t	1	Villa hypoxantha	Asteriscium aemocarpon	17	0.00	0.03	0.01	0.02
1	0	0.37	1	cv	1	Villa hypoxantha	Chaetanthera euphrasioide s	17	0.00	0.03	0.02	0.04
1	0	0.27	1	t	1	Villa hypoxantha	Quinchamaliu m chilensis	17	0.00	0.03	0.01	0.04
1	0	0.33	1	t	1	Anthidium sp.	Phacelia secunda	17	0.00	0.03	0.02	0.04
1	0	0.30	1	t	1	Colletes sp.	Astragalus curvicaulis	17	0.00	0.02	0.01	0.03
1	0	0.24	1	cv	1	Anthidium funereum	Scypanthus elegans	17	0.00	0.02	0.00	0.03
0	0	0.29	1	t	1	Anthidium funereum	Senecio erucaeformis	17	0.00	0.02	0.01	0.03
1	0	0.26	1	t	1	Anthidium funereum	Senecio bustillosianus	17	0.00	0.02	0.01	0.03
1	0	0.31	1	t	1	Anthidium funereum	Senecio francisci	17	0.00	0.02	0.01	0.02
1	0	0.24	1	cv	1	Eristalis tenax	Senecio bustillosianus	17	0.00	0.03	0.01	0.03
1	0	0.35	1	t	1	Calandrinia uspallatensis	Liphanthus sabulosus	17	0.01	0.04	0.01	0.02
1	0	0.46	1	t	1	Calandrinia uspallatensis	Anthidium chilensis	17	0.01	0.04	0.02	0.03
1	0	0.32	1	t	1	Calandrinia uspallatensis	Megachile semirufa	17	0.01	0.04	0.01	0.02
1	0	0.30	1	cv	1	Stachys albicaulis	Anthophora sp.	17	0.01	0.03	0.01	0.03
1	0	0.27	1	t	1	Stachys albicaulis	Anthophora incerta	17	0.01	0.03	0.00	0.03
1	1	0.59	1	t	1	Stachys albicaulis	Megachile sp. 2	17	0.01	0.03	0.03	0.03
1	0	0.38	1	t	1	Stachys albicaulis	Megachile sp. 1	17	0.01	0.03	0.01	0.03
1	0	0.41	1	cv	1	Stachys albicaulis	Alloscirtetica gayi	17	0.01	0.03	0.02	0.02

1	0	0.31	1	t	1	Stachys albicaulis	Centris cineraria	17	0.01	0.03	0.01	0.02
1	0	0.31	1	t	1	Stachys albicaulis	Megachile semirufa	17	0.01	0.03	0.01	0.02
1	1	0.56	1	t	1	Stachys albicaulis	Centris nigerrima	17	0.01	0.03	0.03	0.03
1	0	0.32	1	cv	1	Stachys albicaulis	Alloscirtetica rufitarsis	17	0.01	0.03	0.01	0.03
1	0	0.35	1	t	1	Stachys albicaulis	Cadeguala occidentalis	17	0.01	0.03	0.01	0.03
0	1	0.74	1	t	1	Coleoptera n.i.	Cerastium arvense	17	0.02	0.04	0.05	0.04
0	0	0.30	1	t	1	Coleoptera n.i.	Hypochoeris sp.	17	0.02	0.04	0.01	0.04
0	0	0.34	1	cv	1	Coleoptera n.i.	Oxalis sp.2	17	0.02	0.04	0.01	0.04
0	0	0.36	1	t	1	Anthophora sp.	Phacelia secunda	17	0.01	0.03	0.02	0.04
1	1	0.52	1	t	1	Scypanthus elegans	Megachile sp. 2	17	0.00	0.03	0.03	0.03
1	0	0.32	1	t	1	Scypanthus elegans	Megachile sp. 1	17	0.00	0.03	0.01	0.03
1	0	0.38	1	cv	1	Scypanthus elegans	Anthidium chilensis	17	0.00	0.03	0.02	0.03
1	0	0.25	1	t	1	Scypanthus elegans	Megachile semirufa	17	0.00	0.03	0.01	0.02
1	0	0.29	1	t	1	Chaetodemoticus chilensis	Verbena scoparia	17	0.01	0.01	0.00	0.03
1	1	0.76	1	t	1	Anthidium adriani	Phacelia secunda	17	0.05	0.04	0.02	0.04
1	0	0.24	1	cv	1	Chaetanthera microphylla	Faunula leucoglene	17	0.00	0.04	0.01	0.03
0	1	0.67	1	t	1	Senecio erucaeformis	Hypodynerus chiliotus	17	0.01	0.03	0.05	0.04
1	0	0.37	1	t	1	Senecio erucaeformis	Megachile sp. 1	17	0.01	0.03	0.01	0.03
1	0	0.30	1	t	1	Senecio erucaeformis	Auca coctei-pales	17	0.01	0.03	0.01	0.02
1	0	0.34	1	cv	1	Senecio erucaeformis	Panurgineae sp.	17	0.01	0.03	0.01	0.02
1	0	0.26	1	t	1	Senecio erucaeformis	Hypodynerus sp. 3	17	0.01	0.03	0.00	0.03
1	0	0.29	1	t	1	Senecio erucaeformis	Megachile semirufa	17	0.01	0.03	0.01	0.02
1	0	0.50	1	t	1	Senecio erucaeformis	Caenohalictus sp.	17	0.01	0.03	0.03	0.03
1	1	0.70	1	cv	1	Bibionidae sp. 2	Cerastium arvense	17	0.01	0.04	0.05	0.04
1	1	0.61	1	t	1	Hypodynerus chiliotus	Senecio bustillosianus	17	0.05	0.04	0.01	0.03
1	0	0.35	1	t	1	Perezia carthamoides	Truphoecus bellus	17	0.00	0.03	0.02	0.03

1	0	0.27	1	t	1	Perezia carthamoides	Auca coctei-pales	17	0.00	0.03	0.01	0.02
1	0	0.28	1	cv	1	Perezia carthamoides	Ruiziella luctuosa	17	0.00	0.03	0.01	0.02
1	0	0.28	1	t	1	Senecio bustillosianus	Faunula leucoglene	17	0.01	0.03	0.01	0.03
1	0	0.23	1	t	1	Senecio bustillosianus	Anthophora incerta	17	0.01	0.03	0.00	0.03
1	0	0.33	1	t	1	Senecio bustillosianus	Megachile sp. 1	17	0.01	0.03	0.01	0.03
1	0	0.27	1	cv	1	Senecio bustillosianus	Auca coctei-pales	17	0.01	0.03	0.01	0.02
1	0	0.31	1	t	1	Senecio bustillosianus	Panurgineae sp.	17	0.01	0.03	0.01	0.02
1	0	0.26	1	t	1	Senecio bustillosianus	Centris cineraria	17	0.01	0.03	0.01	0.02
1	0	0.28	1	t	1	Senecio bustillosianus	Ruiziella luctuosa	17	0.01	0.03	0.01	0.02
1	0	0.39	1	cv	1	Senecio bustillosianus	Anthidium chilensis	17	0.01	0.03	0.02	0.03
1	0	0.26	1	t	1	Senecio bustillosianus	Megachile semirufa	17	0.01	0.03	0.01	0.02
1	0	0.40	1	t	1	Adesmia radicifolia	Xanthocollete s sicheli	17	0.00	0.04	0.03	0.03
1	0	0.24	1	t	1	Adesmia radicifolia	Centris cineraria	17	0.00	0.04	0.01	0.02
1	0	0.23	1	cv	1	Adesmia radicifolia	Megachile semirufa	17	0.00	0.04	0.01	0.02
1	0	0.27	1	t	1	Adesmia radicifolia	Cadeguala occidentalis	17	0.00	0.04	0.01	0.03
1	0	0.32	1	t	1	Rhodophiala montana	Megachile sp. 1	17	0.00	0.03	0.01	0.03
1	1	0.59	1	t	1	Cerastium arvense	Habromyia lipoflava	17	0.05	0.04	0.00	0.04
1	0	0.33	1	cv	1	Phacelia secunda	Anthophora incerta	17	0.02	0.04	0.00	0.03
1	0	0.47	1	t	1	Phacelia secunda	Truphoecus bellus	17	0.02	0.04	0.02	0.03
1	0	0.45	1	t	1	Phacelia secunda	Megachile sp. 1	17	0.02	0.04	0.01	0.03
1	0	0.37	1	t	1	Phacelia secunda	Centris cineraria	17	0.02	0.04	0.01	0.02
1	0	0.33	1	cv	1	Phacelia secunda	Hypodynerus sp. 3	17	0.02	0.04	0.00	0.03
1	1	0.52	1	t	1	Phacelia secunda	Anthidium chilensis	17	0.02	0.04	0.02	0.03
1	0	0.37	1	t	1	Phacelia secunda	Megachile semirufa	17	0.02	0.04	0.01	0.02
1	1	0.58	1	t	1	Phacelia secunda	Caenohalictu s sp.	17	0.02	0.04	0.03	0.03

1	0	0.38	1	cv	1	Phacelia secunda	Alloscirtetica rufitarsis	17	0.02	0.04	0.01	0.03
1	0	0.41	1	t	1	Phacelia secunda	Cadeguala occidentalis	17	0.02	0.04	0.01	0.03
1	0	0.36	1	t	1	Mustisia acerosa	Auca coctei-pales	17	0.01	0.02	0.01	0.02
1	0	0.33	1	t	1	Senecio francisci	Auca coctei-pales	17	0.01	0.02	0.01	0.02
1	0	0.37	1	cv	1	Senecio francisci	Panurgineae sp.	17	0.01	0.02	0.01	0.02
1	0	0.32	1	t	1	Senecio francisci	Megachile semirufa	17	0.01	0.02	0.01	0.02
1	0	0.34	1	t	1	Truphoecus bellus	Verbena scoparia	17	0.02	0.03	0.00	0.03
1	0	0.39	1	t	1	Megachile sp. 1	Astragalus curvicaulis	17	0.01	0.03	0.01	0.03
1	0	0.32	1	cv	1	Megachile sp. 1	Cynoglossum creticum	17	0.01	0.03	0.00	0.03
1	0	0.32	1	t	1	Astragalus curvicaulis	Centris cineraria	17	0.01	0.03	0.01	0.02
1	0	0.32	1	t	1	Astragalus curvicaulis	Megachile semirufa	17	0.01	0.03	0.01	0.02
1	1	0.53	1	t	1	Astragalus curvicaulis	Caenohalictus sp.	17	0.01	0.03	0.03	0.03
1	0	0.29	1	cv	1	Astragalus curvicaulis	Colletes fulvipes	17	0.01	0.03	0.00	0.02
1	0	0.26	1	t	1	Anarthrophyllum cumingii	Centris cineraria	17	0.00	0.03	0.01	0.02
1	0	0.41	1	t	1	Nothoscordum andinum	Caenohalictus sp.	17	0.00	0.04	0.03	0.03
1	0	0.27	1	t	1	Centris cineraria	Malesherbia linearifolius	17	0.01	0.02	0.00	0.03
0	0	0.26	1	cv	1	Centris cineraria	Hypochoeris sp.	17	0.01	0.02	0.01	0.04
1	1	0.61	1	t	1	Chaetanthera euphrasioides	Caenohalictus sp.	17	0.02	0.04	0.03	0.03
1	0	0.47	1	t	1	Caenohalictus sp.	Tristagama bivalve	17	0.03	0.03	0.01	0.03
0	0	0.36	0	t	1	Adesmia aconcaguensis	Chaetodemoticus chilensis	17	0.01	0.04	0.01	0.01
0	1	0.80	0	cv	1	Anthidium decaspilum	Megachile sp. 2	17	0.03	0.03	0.03	0.03
0	1	0.58	0	t	1	Liphanthus sabulosus	Centris nigerrima	17	0.01	0.02	0.03	0.03
0	1	0.77	0	t	1	Cardamine nivalis	Hypodynerus chiliotus	17	0.02	0.02	0.05	0.04
1	0	0.26	0	t	1	Colletes sp.	Solanum tomatillo	17	0.00	0.02	0.00	0.02
0	0	0.29	0	cv	1	Hymenoptera n.i.	Quinchamalium chilensis	17	0.00	0.02	0.01	0.04

0	1	0.85	0	t	1	Anthomyiidae sp.2	Anthidium adriani	17	0.03	0.03	0.05	0.04
0	0	0.45	0	t	1	Euphorbia portulacoides	Auca coctei-pales	17	0.03	0.04	0.01	0.02
0	1	0.55	0	t	1	Caenohalictus sp. 3	Svastrides melanura	17	0.01	0.02	0.03	0.03
0	0	0.40	0	cv	1	Scypanthus elegans	Euphorbia portulacoides	17	0.00	0.03	0.03	0.04
1	0	0.20	0	t	1	Acaena pinnatifida	Colletes fulvipes	17	0.00	0.04	0.00	0.02
0	0	0.23	0	t	1	Eurygostomyia philippii	Eristalis tenax	17	0.00	0.03	0.00	0.03
1	0	0.26	0	t	1	Dialictus sp.	Anarthrophyll um cumingii	17	0.00	0.01	0.00	0.03
0	1	0.65	0	cv	1	Dasybasis sp. 1	Cerastium arvense	17	0.00	0.03	0.05	0.04
0	0	0.39	0	t	1	Bombus dahlbomii	Asteriscium aemocarpon	17	0.01	0.03	0.01	0.02
1	0	0.20	0	t	1	Erodium cicutarium	Vespidae n.i.	17	0.00	0.04	0.01	0.03
0	0	0.27	0	t	1	Lithurgomma wagenknechti	Pseudolucia chilensis	17	0.00	0.03	0.01	0.03
0	1	0.68	0	cv	1	Bibionidae sp. 2	Hypodynerus chiliotus	17	0.01	0.04	0.05	0.04
0	0	0.44	0	t	1	Bibionidae sp.1	Svastrides melanura	17	0.00	0.04	0.03	0.03
0	0	0.28	0	t	1	Berberis empetrifolia	Oenothera acaulis	17	0.00	0.04	0.01	0.02
1	0	0.41	0	t	1	Cardamine nivalis	Leucocoryne ixioides	17	0.02	0.02	0.00	0.02
1	0	0.45	0	cv	1	Mulinum spinosum	Caenohalictu s sp.	17	0.01	0.04	0.03	0.03
1	0	0.32	0	t	1	Haplopappus chysanthemifo lius	Panurgineae sp.	17	0.01	0.04	0.01	0.02
1	0	0.33	0	t	1	Hypsochila wagenknecti	Barneoudia chilensis	17	0.01	0.01	0.01	0.03
0	0	0.24	0	t	1	Hypodynerus sp. 3	Colletes fulvipes	17	0.00	0.03	0.00	0.02
0	0	0.19	0	cv	1	Colletes araucariae	Senecio lithostaurus	17	0.00	0.03	0.00	0.04
1	0	0.32	0	t	1	Prosopochaet a caliginosa	Adesmia aconcaguensi s	17	0.01	0.03	0.01	0.04
1	0	0.24	0	t	1	Chuquiraga oppositifolia	Centris cineraria	17	0.00	0.03	0.01	0.02
0	1	0.64	0	t	1	Colletes araucariae	Cerastium arvense	17	0.00	0.03	0.05	0.04
0	0	0.33	0	cv	1	Hypodynerus sp.2	Cadeguala occidentalis	17	0.01	0.02	0.01	0.03

1	0	0.41	0	t	1	Trichocereus chiloensis	Lepidium suffruticosum	17	0.02	0.02	0.00	0.01
0	0	0.42	0	t	1	Clarkia tenella	Villa arenarius	17	0.00	0.02	0.02	0.03
0	0	0.32	0	t	1	Bombus dahlbomii	Caupolicana dimidiata	17	0.01	0.03	0.01	0.02
1	0	0.28	0	cv	1	Bowlesia tropaeolifolia	Megachile sp. 1	17	0.00	0.04	0.01	0.03
0	0	0.33	0	t	1	Tristagama sessile	Chilimelissa sp.	17	0.01	0.02	0.01	0.03
1	0	0.41	0	t	1	Barneoudia major	Alloscirtetica rufitarsis	17	0.02	0.04	0.01	0.03
0	0	0.34	0	t	1	Villa gayi	Tapinotaspis caerulea	17	0.01	0.03	0.00	0.02
1	0	0.31	0	cv	1	Astragalus curvicaulis	Xanthocollete s sp.	17	0.01	0.03	0.00	0.02
0	0	0.38	0	t	1	Lithurgomma wagenknechti	Cardamine nivalis	17	0.00	0.03	0.02	0.02
0	0	0.41	0	t	1	Villa arenarius	Coelioxys sp.	17	0.02	0.03	0.00	0.02
1	0	0.44	0	t	1	Tropaeolum tricolor	Hypsochila wagenknecti	17	0.01	0.02	0.01	0.01
1	0	0.24	0	cv	1	Haplopappus chysanthemifo lius	Hypodynerus sp. 3	17	0.01	0.04	0.00	0.03
0	0	0.28	0	t	1	Caupolicana dimidiata	Colletes fulvipes	17	0.01	0.02	0.00	0.02
1	0	0.21	0	t	1	Alstromemeria pallida	Chuquiraga oppositifolia	17	0.00	0.03	0.00	0.03
1	0	0.21	0	t	1	Sanicula graveolens	Tristagama bivalve	17	0.00	0.04	0.01	0.03
1	0	0.33	0	cv	1	Stachys albicaulis	Faunula leucoglene	17	0.01	0.03	0.01	0.03
0	0	0.27	0	t	1	Vespidae n.i.	Chilimelissa sp.	17	0.01	0.03	0.01	0.03
0	0	0.28	0	t	1	Alloscirtetica rufitarsis	Colletes fulvipes	17	0.01	0.03	0.00	0.02
1	0	0.27	0	t	1	Sisyrinchium junceum	Valeriana gracileps	17	0.01	0.03	0.00	0.04
1	0	0.21	0	cv	1	Loasa heterophylla	Rhodophiala montana	17	0.00	0.03	0.00	0.03
1	0	0.31	0	t	1	Clarkia tenella	Yramea lathonoides	17	0.00	0.02	0.01	0.03
1	0	0.33	0	t	1	Sisyrinchium junceum	Calceolaria montana	17	0.01	0.03	0.01	0.03
1	0	0.43	0	t	1	Sisyrinchium junceum	Tristagama nivale	17	0.01	0.03	0.01	0.01
0	1	0.66	0	cv	1	Andinus venustus	Anthidium gayi	17	0.00	0.02	0.05	0.03
0	1	0.56	0	t	1	Tapinotaspis herbsti	Megachile sp. 2	17	0.00	0.02	0.03	0.03

1	0	0.26	0	t	1	Clarkia tenella	Senecio bustillosianus	17	0.00	0.02	0.01	0.03
1	1	0.71	0	t	1	Adesmia exilis	Anthidium gayi	17	0.01	0.02	0.05	0.03
1	1	0.53	0	cv	1	Schizanthus garhamii	Anthidium decaspilum	17	0.00	0.03	0.03	0.03
0	0	0.40	0	t	1	Coelioxys sp.	Chaetanthera euphrasioides	17	0.00	0.02	0.02	0.04
1	0	0.34	0	t	1	Adesmia conferta	Anthomyiidae sp.4	17	0.01	0.04	0.01	0.02
0	0	0.29	0	t	1	Chilicola sp.	Megachile semirufa	17	0.01	0.03	0.01	0.02
1	0	0.33	0	cv	1	Madia sativa	Tristagama sessile	17	0.01	0.02	0.01	0.02
0	0	0.25	0	t	1	Bibionidae sp.1	Adesmia aconcaguensis	17	0.00	0.04	0.01	0.04
0	0	0.39	0	t	1	Barneoudia major	Sisyrinchium arenarium	17	0.02	0.04	0.01	0.03
1	0	0.43	0	t	1	Sarcophagidae n.i.	Centris sp.	17	0.02	0.03	0.00	0.00
1	0	0.27	0	cv	1	Mustisia sinuata	Sanicula graveolens	17	0.02	0.04	0.00	0.04
1	1	0.68	0	t	1	Elaphroptera relicta	Liphanthus sp.2	17	0.03	0.03	0.02	0.02
1	0	0.26	0	t	1	Scaptia atra	Bombyllidae n.i.	17	0.00	0.03	0.00	0.01
0	1	0.70	0	t	1	Caenohalictus sp. 3	Anthidium gayi	17	0.01	0.02	0.05	0.03
1	0	0.24	0	cv	1	Vibrissomyia sp.	Villa hypoxantha	17	0.00	0.03	0.00	0.03
1	0	0.42	0	t	1	Lycophlaeba lugubris	Leucocoryne pauciflora	17	0.02	0.03	0.00	0.02
0	0	0.31	0	t	1	Colletes araucariae	Villa gayi	17	0.00	0.03	0.01	0.03
0	0	0.38	0	t	1	Alloscirtetica sp.	Cardamine nivalis	17	0.00	0.03	0.02	0.02
1	0	0.49	0	cv	1	Stachys albicaulis	Caenohalictus sp. 2	17	0.01	0.03	0.02	0.02
0	0	0.32	0	t	1	Tabanidae n.i.	Diptera n.i.	17	0.01	0.03	0.01	0.03
1	1	0.92	0	t	1	Scaeva melanostoma	Nassauvia heterophylla	17	0.06	0.03	0.04	0.03
1	0	0.35	0	t	1	Lathyrus subandinus	Alloscirtetica rufitarsis	17	0.01	0.02	0.01	0.03
0	0	0.38	0	cv	1	Barneoudia major	Tapinotaspis herbsti	17	0.02	0.04	0.00	0.02
0	0	0.27	0	t	1	Oxalis geminata	Colletes fulvipes	17	0.01	0.03	0.00	0.02

0	0	0.33	0	t	1	Anthophora sp.	Villa guttapennis	17	0.01	0.03	0.01	0.02
0	1	0.50	0	t	1	Troch n.i.	Euphorbia portulacoides	17	0.00	0.00	0.03	0.04
1	0	0.46	0	cv	1	Senecio francisci	Anthidium chilensis	17	0.01	0.02	0.02	0.03
0	1	0.69	0	t	1	Mesonychium gayi	Anthidium adriani	17	0.00	0.03	0.05	0.04
0	1	0.52	0	t	1	Faunula leucoglene	Caenohalictu s sp.	17	0.01	0.03	0.03	0.03
0	0	0.27	0	t	1	Corynura sp.	Hypodynerus sp. 3	17	0.00	0.01	0.00	0.03
1	1	0.51	0	cv	1	Leuceria landbeckii	Megachile sp. 2	17	0.01	0.04	0.03	0.03
1	0	0.39	0	t	1	Tristagama nivale	Caupolicana dimidiata	17	0.01	0.01	0.01	0.02
0	0	0.32	0	t	1	Leuceria landbeckii	Asteriscium aemocarpon	17	0.01	0.04	0.01	0.02
0	0	0.47	0	t	1	Anthomyiidae sp.2	Loasa sigmoidea	17	0.03	0.03	0.00	0.02
1	0	0.24	0	cv	1	Alstromemeria pallida	Vespidae n.i.	17	0.00	0.03	0.01	0.03
0	1	0.60	0	t	1	Hypodynerus chiliotus	Verbena scoparia	17	0.05	0.04	0.00	0.03
0	0	0.28	0	t	1	Lithurgomma wagenknechti	Tristagama sessile	17	0.00	0.03	0.01	0.02
1	0	0.39	0	t	1	Mustisia sinuata	Tabanidae n.i.	17	0.02	0.04	0.01	0.03
0	1	0.74	0	cv	1	Scaeva melanostoma	Colletes sp.	17	0.06	0.03	0.00	0.02
0	0	0.47	0	t	1	Hypodynerus sp.2	Solenomelus sisyrinchium	17	0.01	0.02	0.02	0.02
1	0	0.43	0	t	1	Mulinum spinosum	Caenohalictu s sp. 2	17	0.01	0.04	0.02	0.02
0	0	0.31	0	t	1	Spathipalpus philippii	Villa guttapennis	17	0.00	0.03	0.01	0.02
0	0	0.49	0	cv	1	Caenohalictus sp. 2	Faunula leucoglene	17	0.02	0.02	0.01	0.03
1	0	0.32	0	t	1	Oenothera acaulis	Senecio bustillosianus	17	0.01	0.02	0.01	0.03
0	0	0.38	0	t	1	Villa sp.	Bibionidae sp. 2	17	0.01	0.03	0.01	0.04
0	0	0.32	0	t	1	Scaptia atra	Xanthocollete s incahuasi	17	0.00	0.03	0.01	0.02
1	0	0.27	0	cv	1	Caupolicana dimidiata	Malesherbia linearifolius	17	0.01	0.02	0.00	0.03
0	0	0.38	0	t	1	Vespidae n.i.	Lupinus microcarpus	17	0.01	0.03	0.02	0.02
1	0	0.31	0	t	1	Hypsochila wagenknecti	Colletes fulvipes	17	0.01	0.01	0.00	0.02

1	0	0.39	0	t	1	Trichocereus chiloensis	Hymenoptera n.i.	17	0.02	0.02	0.00	0.02
1	0	0.32	0	cv	1	Tatochila mercedis	Adesmia radicifolia	17	0.02	0.03	0.00	0.04
1	0	0.34	0	t	1	Astragalus sp.	Hypodynerus sp. 3	17	0.02	0.03	0.00	0.03
0	0	0.35	0	t	1	Colletes araucariae	Calceolaria sp.	17	0.00	0.03	0.01	0.02
0	1	0.65	0	t	1	Prosopochaet a caliginosa	Hypodynerus chiliotus	17	0.01	0.03	0.05	0.04
0	0	0.35	0	cv	1	Spathipalpus philippii	Tatochila mercedis	17	0.00	0.03	0.02	0.03
1	0	0.25	0	t	1	Tabanidae n.i.	Tetraglochin alatum	17	0.01	0.03	0.00	0.04
1	0	0.22	0	t	1	Mustisia subulata	Eurygostomyi a philippii	17	0.00	0.03	0.00	0.03
0	0	0.26	0	t	1	Leucocoryne pauciflora	Vespidae n.i.	17	0.00	0.02	0.01	0.03
1	0	0.32	0	cv	1	Phacelia secunda	Verbena scoparia	17	0.02	0.04	0.00	0.03
1	0	0.35	0	t	1	Calceolaria biflora	Ruiziella luctuosa	17	0.00	0.00	0.01	0.02
0	1	0.79	0	t	1	Scaeva melanostoma	Centris sp.	17	0.06	0.03	0.00	0.00
0	0	0.26	0	t	1	Eristalis tenax	Barneoudia chilensis	17	0.00	0.03	0.01	0.03
1	0	0.28	0	cv	1	Hymenoptera n.i.	Villa durvillei	17	0.00	0.02	0.00	0.01
0	1	0.54	0	t	1	Viola atropurpurea	Anthidium decaspilum	17	0.00	0.02	0.03	0.03
1	0	0.35	0	t	1	Viola atropurpurea	Asteriscium aemocarpon	17	0.00	0.02	0.01	0.02
0	1	0.59	0	t	1	Svastrides melanura	Megachile sp. 1	17	0.03	0.03	0.01	0.03
1	0	0.31	0	cv	1	Villa verdensis	Hypochoeris sp.	17	0.01	0.03	0.01	0.04
0	0	0.44	0	t	1	Anthomyiidae sp.2	Hypodynerus sp. 3	17	0.03	0.03	0.00	0.03
1	0	0.28	0	t	1	Tapinotaspis caerulea	Salpiglossus sp.	17	0.00	0.02	0.00	0.02
0	1	0.77	0	t	1	Scaeva melanostoma	Dialictus sp.	17	0.06	0.03	0.00	0.01
1	0	0.39	0	cv	1	Lycophlaeba lugubris	Rhodophiala montana	17	0.02	0.03	0.00	0.03
0	0	0.46	0	t	1	Xanthocolletes sicheli	Centris cineraria	17	0.03	0.03	0.01	0.02
1	0	0.28	0	t	1	Collomia biflora	Faunula leucoglene	17	0.00	0.03	0.01	0.03
0	0	0.26	0	t	1	Mesonychium gayi	Coelioxys sp.	17	0.00	0.03	0.00	0.02

0	1	0.55	0	cv	1	Caupolicana dimidiata	Centris nigerrima	17	0.01	0.02	0.03	0.03
1	0	0.34	0	t	1	Tristagama nivale	Malesherbia linearifolius	17	0.01	0.01	0.00	0.03
1	0	0.29	0	t	1	Adesmia conferta	Auca coctei-pales	17	0.01	0.04	0.01	0.02
1	0	0.38	0	t	1	Tristagama nivale	Centris cineraria	17	0.01	0.01	0.01	0.02
1	1	0.73	0	cv	1	Scaeva melanostoma	Cynoglossum creticum	17	0.06	0.03	0.00	0.03
0	1	0.66	0	t	1	Schizanthus garhamii	Cerastium arvense	17	0.00	0.03	0.05	0.04
1	0	0.28	0	t	1	Haplopappus chysanthemifo lius	Alloscirtetica rufitarsis	17	0.01	0.04	0.01	0.03
0	0	0.30	0	t	1	Anthidium funereum	Alloscirtetica rufitarsis	17	0.00	0.02	0.01	0.03
0	0	0.37	0	cv	1	Cardamine nivalis	Hypodynerus sp. 3	17	0.02	0.02	0.00	0.03
0	1	0.79	0	t	1	Scaeva melanostoma	Berberis montana	17	0.06	0.03	0.01	0.02
1	0	0.39	0	t	1	Anthomyiidae sp.2	Tetraglochin alatum	17	0.03	0.03	0.00	0.04
0	0	0.19	0	t	1	Mustisia subulata	Chaetanthera microphylla	17	0.00	0.03	0.00	0.04
1	0	0.31	0	cv	1	Calceolaria montana	Chaetodemot icus chilensis	17	0.01	0.03	0.01	0.01
1	0	0.28	0	t	1	Tapinotaspis herbsti	Adesmia conferta	17	0.00	0.02	0.01	0.04
0	0	0.34	0	t	1	Isepeolus niveiventris	Truphoecus bellus	17	0.00	0.03	0.02	0.03
1	0	0.28	0	t	1	Corynura sp.	Hymenoptera n.i.	17	0.00	0.01	0.00	0.02
1	0	0.25	0	cv	1	Mesonychium gayi	Haplopappus chysanthemif olius	17	0.00	0.03	0.01	0.04
1	0	0.38	0	t	1	Adesmia conferta	Tatochila mercedis	17	0.01	0.04	0.02	0.03
1	0	0.26	0	t	1	Xanthocolletes sp.	Hypochoeris sp.	17	0.00	0.02	0.01	0.04
1	0	0.39	0	t	1	Adesmia exilis	Megachile sp. 1	17	0.01	0.02	0.01	0.03
1	0	0.46	0	cv	1	Schizanthus hookeri	Caenohalictu s sp. 2	17	0.01	0.03	0.02	0.02
0	0	0.28	0	t	1	Scaptia atra	Calceolaria biflora	17	0.00	0.03	0.00	0.00
1	0	0.28	0	t	1	Loasa heterophylla	Liphanthus sabulosus	17	0.00	0.03	0.01	0.02
1	0	0.35	0	t	1	Bombyllidae n.i.	Lathyrus subandinus	17	0.00	0.01	0.01	0.02

1	0	0.30	0	cv	1	Calceolaria montana	Agromyzidae sp.2	17	0.01	0.03	0.01	0.03
1	0	0.38	0	t	1	Calandrinia uspallatensis	Mustisia acerosa	17	0.01	0.04	0.01	0.02
1	0	0.30	0	t	1	Anthidium funereum	Stachys albicaulis	17	0.00	0.02	0.01	0.03
0	0	0.43	0	t	1	Villa sp.	Megachile sp. 1	17	0.01	0.03	0.01	0.03
1	0	0.23	0	cv	1	Vibrissomyia sp.	Collomia biflora	17	0.00	0.03	0.00	0.03
0	0	0.31	0	t	1	Anthomyiidae sp.4	Anthophora incerta	17	0.01	0.02	0.00	0.03
1	0	0.32	0	t	1	Chilicola sp.	Astragalus curvicaulis	17	0.01	0.03	0.01	0.03
1	0	0.24	0	t	1	Collomia biflora	Malesherbia linearifolius	17	0.00	0.03	0.00	0.03
1	0	0.29	0	cv	1	Clarkia tenella	Villa durvillei	17	0.00	0.02	0.00	0.01
1	0	0.23	0	t	1	Sanicula graveolens	Centris cineraria	17	0.00	0.04	0.01	0.02
1	0	0.36	0	t	1	Calandrinia affinis	Calandrinia uspallatensis	17	0.01	0.04	0.01	0.04
0	0	0.28	0	t	1	Andinus venustus	Quinchamalium chilensis	17	0.00	0.02	0.01	0.04
1	1	0.65	0	cv	1	Barneoudia major	Muscidae sp.	17	0.02	0.04	0.03	0.03
0	1	0.52	0	t	1	Villa gayi	Euphorbia portulacoides	17	0.01	0.03	0.03	0.04
0	0	0.48	0	t	1	Villa arenarius	Anthomyiidae sp.4	17	0.02	0.03	0.01	0.02
0	0	0.28	0	t	1	Spathipalpus philippii	Agromyzidae sp.2	17	0.00	0.03	0.01	0.03
1	0	0.44	0	cv	1	Hypsochila wagenknecti	Tatochila mercedis	17	0.01	0.01	0.02	0.03
1	0	0.27	0	t	1	Stachys albicaulis	Rhodophiala montana	17	0.01	0.03	0.00	0.03
1	1	0.50	0	t	1	Tatochila mercedis	Chaetanthera euphrasioides	17	0.02	0.03	0.02	0.04
1	0	0.41	0	t	1	Lupinus microcarpus	Barneoudia chilensis	17	0.02	0.02	0.01	0.03
0	0	0.28	0	cv	1	Barneoudia chilensis	Xanthocolletes sp.	17	0.01	0.03	0.00	0.02
0	0	0.43	0	t	1	Solanum tomatillo	Anthidium chilensis	17	0.00	0.02	0.02	0.03
0	1	0.79	0	t	1	Scaeva melanostoma	Tristagama sessile	17	0.06	0.03	0.01	0.02
0	0	0.20	0	t	1	Bibionidae sp.1	Vibrissomyia sp.	17	0.00	0.04	0.00	0.03

1	0	0.24	0	cv	1	Sisyrinchium arenarium	Anthidium sp.	17	0.01	0.03	0.00	0.03
1	0	0.21	0	t	1	Villa hypoxantha	Nothoscordum andinum	17	0.00	0.03	0.00	0.04
0	0	0.29	0	t	1	Viola atropurpurea	Chilimelissa sp.	17	0.00	0.02	0.01	0.03
0	0	0.28	0	t	1	Bibionidae sp. 2	Perezia carthamoides	17	0.01	0.04	0.00	0.03
0	0	0.29	0	cv	1	Ruiziella luctuosa	Colletes fulvipes	17	0.01	0.02	0.00	0.02
1	0	0.29	0	t	1	Chilicola sp.	Lepidium suffruticosum	17	0.01	0.03	0.00	0.01
1	0	0.47	0	t	1	Trichocereus chiloensis	Sisyrinchium junceum	17	0.02	0.02	0.01	0.03
0	0	0.32	0	t	1	Nardophyllum lanatum	Bowlesia tropaeolifolia	17	0.02	0.02	0.00	0.04
0	0	0.19	0	cv	1	Chaetanthera microphylla	Rhodophiala montana	17	0.00	0.04	0.00	0.03
1	0	0.33	0	t	1	Stachys albicaulis	Agromyzidae sp.2	17	0.01	0.03	0.01	0.03
0	0	0.33	0	t	1	Bombus dahlbomii	Agromyzidae sp.2	17	0.01	0.03	0.01	0.03
0	0	0.24	0	t	1	Diptera n.i.	Lithurgomma wagenknechti	17	0.01	0.03	0.00	0.03
1	0	0.25	0	cv	1	Mulinum spinosum	Megachile semirufa	17	0.01	0.04	0.01	0.02
0	0	0.27	0	t	1	Sisyrinchium philippii	Senecio francisci	17	0.01	0.04	0.01	0.02
1	0	0.50	0	t	1	Senecio lithostaurus	Nassauvia heterophylla	17	0.00	0.04	0.04	0.03
1	0	0.29	0	t	1	Loasa sigmoidea	Centris cineraria	17	0.00	0.02	0.01	0.02
1	0	0.25	0	cv	1	Collomia biflora	Loasa sigmoidea	17	0.00	0.03	0.00	0.02
0	1	0.76	0	t	1	Scaeva melanostoma	Centris cineraria	17	0.06	0.03	0.01	0.02
0	0	0.43	0	t	1	Troch n.i.	Truphoecus bellus	17	0.00	0.00	0.02	0.03
0	0	0.39	0	t	1	Villa sp.	Liphanthus sabulosus	17	0.01	0.03	0.01	0.02
0	0	0.33	0	cv	1	Lepidoptera n.i.	Tapinotaspis caerulea	17	0.01	0.02	0.00	0.02
0	0	0.21	0	t	1	Senecio lithostaurus	Tapinotaspis caerulea	17	0.00	0.04	0.00	0.02
0	0	0.37	0	t	1	Villa arenarius	Colletes araucariae	17	0.02	0.03	0.00	0.03

1	0	0.44	0	t	1	Astragalus sp.	Panurgineae sp.	17	0.02	0.03	0.01	0.02
1	0	0.35	0	cv	1	Adesmia exilis	Tristagama sessile	17	0.01	0.02	0.01	0.02
1	0	0.21	0	t	1	Dasybasis sp. 2	Anarthrophyllum cumingii	17	0.00	0.03	0.00	0.03
0	1	0.52	0	t	1	Nassauvia heterophylla	Anthidium sp.	17	0.04	0.03	0.00	0.03
0	0	0.24	0	t	1	Viola atropurpurea	Spathipalpus philippii	17	0.00	0.02	0.00	0.03
0	0	0.41	0	cv	1	Villa verdensis	Villa guttapennis	17	0.01	0.03	0.01	0.02
1	0	0.34	0	t	1	Stachys albicaulis	Liphanthus sabulosus	17	0.01	0.03	0.01	0.02
1	0	0.39	0	t	1	Villa gayi	Hypsochila wagenknecti	17	0.01	0.03	0.01	0.01
1	0	0.26	0	t	1	Coelioxys sp.	Senecio bustillosianus	17	0.00	0.02	0.01	0.03
0	1	0.51	0	cv	1	Pseudolucia chilensis	Caenohalictus sp.	17	0.01	0.03	0.03	0.03
0	0	0.29	0	t	1	Schizanthus hookeri	Chilimelissa sp.	17	0.01	0.03	0.01	0.03
0	1	0.70	0	t	1	Tachinidae n.i.	Hypodynerus chiliotus	17	0.01	0.03	0.05	0.04
1	1	0.56	0	t	1	Calceolaria sp.	Hemicolletes ruizi	17	0.01	0.02	0.02	0.02
0	0	0.48	0	cv	1	Colletes araucariae	Svastrides melanura	17	0.00	0.03	0.03	0.03
1	0	0.26	0	t	1	Mulinum spinosum	Senecio erucaeformis	17	0.01	0.04	0.01	0.03
0	0	0.25	0	t	1	Colletes araucariae	Oxalis sp.2	17	0.00	0.03	0.01	0.04
1	0	0.50	0	t	1	Collomia biflora	Centris nigerrima	17	0.00	0.03	0.03	0.03
1	0	0.26	0	cv	1	Valeriana gracileps	Cadeguala occidentalis	17	0.00	0.04	0.01	0.03
0	0	0.29	0	t	1	Vibrissomyia sp.	Bibionidae sp. 2	17	0.00	0.03	0.01	0.04
1	0	0.30	0	t	1	Yramea lathonoides	Tristagama bivalve	17	0.01	0.03	0.01	0.03
0	1	0.70	0	t	1	Lycophlaeba lugubris	Elaphroptera relicta	17	0.02	0.03	0.03	0.03
1	0	0.25	0	cv	1	Sisyrinchium philippii	Adesmia conferta	17	0.01	0.04	0.01	0.04
1	1	0.70	0	t	1	Anthidium gayi	Lathyrus subandinus	17	0.05	0.03	0.01	0.02
0	0	0.31	0	t	1	Caupolicana dimidiata	Chilimelissa sp.	17	0.01	0.02	0.01	0.03
0	0	0.40	0	t	1	Hippeastrum uniflorum	Haplopappus sericeus	17	0.00	0.00	0.02	0.04

1	0	0.41	0	cv	1	Calandrinia uspallatensis	Phacelia secunda	17	0.01	0.04	0.02	0.04
0	0	0.28	0	t	1	Scaptia atra	Chaetodemoticus chilensis	17	0.00	0.03	0.01	0.01
1	0	0.32	0	t	1	Xanthocolletes incahuasi	Calceolaria arachnoides	17	0.01	0.02	0.00	0.03
0	0	0.42	0	t	1	Anthidium chilensis	Megachile semirufa	17	0.02	0.03	0.01	0.02
0	0	0.37	0	cv	1	Villa sp.	Chilimelissa sp.	17	0.01	0.03	0.01	0.03
1	0	0.32	0	t	1	Caenohalictus sp. 3	Corynura sp.	17	0.01	0.02	0.00	0.01
1	0	0.29	0	t	1	Vibrissomyia sp.	Adesmia exilis	17	0.00	0.03	0.01	0.02
1	0	0.29	0	t	1	Bombus dahlbomii	Haplopappus chysanthemifolius	17	0.01	0.03	0.01	0.04
0	0	0.31	0	cv	1	Tristagama nivale	Habromyia lipoflava	17	0.01	0.01	0.00	0.04
0	1	0.69	0	t	1	Anthidium gayi	Calceolaria biflora	17	0.05	0.03	0.00	0.00
0	0	0.38	0	t	1	Truphoecus bellus	Barneoudia chilensis	17	0.02	0.03	0.01	0.03
0	0	0.29	0	t	1	Erodium cicutarium	Tristagama nivale	17	0.00	0.04	0.01	0.01
1	0	0.22	0	cv	1	Nothoscordum andinum	Oxalis sp.2	17	0.00	0.04	0.01	0.04
0	0	0.46	0	t	1	Caenohalictus sp. 2	Oxalis sp.2	17	0.02	0.02	0.01	0.04
0	0	0.34	0	t	1	Villa verdensis	Anthidium funereum	17	0.01	0.03	0.00	0.02
1	0	0.39	0	t	1	Lepidium suffruticosum	Truphoecus bellus	17	0.00	0.01	0.02	0.03
0	0	0.29	0	cv	1	Xanthocolletes incahuasi	Nothoscordum andinum	17	0.01	0.02	0.00	0.04
0	1	0.58	0	t	1	Villa arenarius	Lycophlaeba lugubris	17	0.02	0.03	0.02	0.03
0	0	0.37	0	t	1	Lathyrus subandinus	Calandrinia uspallatensis	17	0.01	0.02	0.01	0.04
1	0	0.23	0	t	1	Perezia carthamoides	Cynoglossum creticum	17	0.00	0.03	0.00	0.03
0	0	0.39	0	cv	1	Eristalis tenax	Liphanthus sp.2	17	0.00	0.03	0.02	0.02
0	0	0.25	0	t	1	Phulia nymphula	Loasa sigmoidea	17	0.00	0.03	0.00	0.02
0	0	0.29	0	t	1	Phulia nymphula	Chaetodemoticus chilensis	17	0.00	0.03	0.01	0.01
0	0	0.40	0	t	1	Mesonychium gayi	Anthidium chilensis	17	0.00	0.03	0.02	0.03
1	1	0.56	0	cv	1	Villa arenarius	Cardamine nivalis	17	0.02	0.03	0.02	0.02

1	0	0.31	0	t	1	Villa guttapennis	Malesherbia linearifolius	17	0.01	0.02	0.00	0.03
0	0	0.33	0	t	1	Adesmia brachysemon	Chaetodemoticus chilensis	17	0.01	0.04	0.01	0.01
0	0	0.22	0	t	1	Berberis empetrifolia	Tapinotaspis caerulea	17	0.00	0.04	0.00	0.02
0	0	0.22	0	cv	1	Scaptia atra	Anthidium sp.	17	0.00	0.03	0.00	0.03
0	0	0.19	0	t	1	Verbena scoparia	Tetraglochin alatum	17	0.00	0.03	0.00	0.04
1	0	0.25	0	t	1	Acaena pinnatifida	Cadeguala occidentalis	17	0.00	0.04	0.01	0.03
0	0	0.38	0	t	1	Sarcophagidae n.i.	Ruizantheda mutabilis	17	0.02	0.03	0.01	0.03
1	0	0.24	0	cv	1	Sisyrinchium philippii	Anthophora sp.	17	0.01	0.04	0.01	0.03
1	0	0.21	0	t	1	Sisyrinchium philippii	Scypanthus elegans	17	0.01	0.04	0.00	0.03
1	0	0.31	0	t	1	Lathyrus subandinus	Senecio bustillosianus	17	0.01	0.02	0.01	0.03
1	0	0.23	0	t	1	Lithurgomma wagenknechti	Schizanthus garhamii	17	0.00	0.03	0.00	0.03
1	0	0.41	0	cv	1	Anthomyiidae sp.2	Adesmia radicifolia	17	0.03	0.03	0.00	0.04
1	0	0.29	0	t	1	Calandrinia affinis	Verbena scoparia	17	0.01	0.04	0.00	0.03
0	0	0.27	0	t	1	Mustisia acerosa	Tetraglochin alatum	17	0.01	0.02	0.00	0.04
1	0	0.22	0	t	1	Scaptia atra	Scypanthus elegans	17	0.00	0.03	0.00	0.03
1	1	0.56	0	cv	1	Lupinus microcarpus	Cardamine nivalis	17	0.02	0.02	0.02	0.02
1	0	0.43	0	t	1	Tristagama nivale	Panurgineae sp.	17	0.01	0.01	0.01	0.02
1	0	0.22	0	t	1	Adesmia conferta	Senecio lithostaurus	17	0.01	0.04	0.00	0.04
1	0	0.29	0	t	1	Villa verdensis	Sanicula graveolens	17	0.01	0.03	0.00	0.04
0	1	0.57	0	cv	1	Corynura sp.	Elaphroptera relicta	17	0.00	0.01	0.03	0.03
0	0	0.28	0	t	1	Hypodynerus sp.2	Loasa sigmoidea	17	0.01	0.02	0.00	0.02
1	0	0.41	0	t	1	Barneoudia major	Pseudolucia chilensis	17	0.02	0.04	0.01	0.03
0	0	0.36	0	t	1	Chilimelissa sp.	Coleoptera n.i.	17	0.01	0.03	0.02	0.04
1	0	0.24	0	cv	1	Auca coctei-pales	Nothoscordum andinum	17	0.01	0.02	0.00	0.04
0	1	0.68	0	t	1	Anthidium adriani	Xanthocolletes sp.	17	0.05	0.04	0.00	0.02
0	0	0.37	0	t	1	Hymenoptera n.i.	Truphoecus bellus	17	0.00	0.02	0.02	0.03

0	1	0.52	0	t	1	Tapinotaspis herbsti	Svastrides melanura	17	0.00	0.02	0.03	0.03
1	0	0.35	0	cv	1	Acaena pinnatifida	Lycophlaeba lugubris	17	0.00	0.04	0.02	0.03
1	0	0.48	0	t	1	Erodium cicutarium	Megachile sp. 2	17	0.00	0.04	0.03	0.03
1	1	0.64	0	t	1	Tropaeolum polyphyllum	Caenohalictus sp. 2	17	0.02	0.02	0.02	0.02
1	0	0.31	0	t	1	Tabanidae n.i.	Tristagama bivalve	17	0.01	0.03	0.01	0.03
0	0	0.40	0	cv	1	Lycophlaeba lugubris	Eristalis tenax	17	0.02	0.03	0.00	0.03
0	0	0.25	0	t	1	Eurygostomyia philippii	Barneoudia chilensis	17	0.00	0.03	0.01	0.03
0	0	0.49	0	t	1	Villa arenarius	Coleoptera n.i.	17	0.02	0.03	0.02	0.04
1	0	0.44	0	t	1	Tropaeolum tricolor	Hippeastrum uniflorum	17	0.01	0.02	0.00	0.00
0	0	0.35	0	cv	1	Anthophora sp.	Megachile sp. 1	17	0.01	0.03	0.01	0.03
0	0	0.35	0	t	1	Anthidium sp.	Alloscirtetica gayi	17	0.00	0.03	0.02	0.02
0	0	0.40	0	t	1	Isepeolus niveiventris	Hemicolletes ruizi	17	0.00	0.03	0.02	0.02
1	0	0.34	0	t	1	Rhodophiala montana	Truphoecus bellus	17	0.00	0.03	0.02	0.03
0	0	0.17	0	cv	1	Acaena pinnatifida	Valeriana graciIeps	17	0.00	0.04	0.00	0.04
1	0	0.33	0	t	1	Villa semifuscata	Astragalus curvicaulis	17	0.01	0.03	0.01	0.03
1	0	0.35	0	t	1	Sisyrinchium junceum	Senecio erucaeformis	17	0.01	0.03	0.01	0.03
1	0	0.26	0	t	1	Calceolaria arachnoides	Dialictus sp.	17	0.00	0.03	0.00	0.01
1	0	0.25	0	cv	1	Anthidium funereum	Hypochoeris sp.	17	0.00	0.02	0.01	0.04
1	0	0.37	0	t	1	Hippeastrum uniflorum	Chaetodemoticus chilensis	17	0.00	0.00	0.01	0.01
1	0	0.44	0	t	1	Tetraglochin alatum	Centris nigerrima	17	0.00	0.04	0.03	0.03
1	0	0.33	0	t	1	Tropaeolum tricolor	Adesmia radicifolia	17	0.01	0.02	0.00	0.04
1	1	0.57	0	cv	1	Cardamine nivalis	Anthidium chilensis	17	0.02	0.02	0.02	0.03
0	1	0.54	0	t	1	Alloscirtetica gayi	Anthidium chilensis	17	0.02	0.02	0.02	0.03
0	0	0.42	0	t	1	Bombus dahlbomii	Nardophyllum lanatum	17	0.01	0.03	0.02	0.02
1	0	0.25	0	t	1	Tropaeolum sessilifolium	Alloscirtetica sp.	17	0.01	0.03	0.00	0.03

0	0	0.26	0	cv	1	Berberis empetrifolia	Chaetodemoticus chilensis	17	0.00	0.04	0.01	0.01
1	0	0.39	0	t	1	Clarkia tenella	Tatochila mercedis	17	0.00	0.02	0.02	0.03
1	1	0.50	0	t	1	Solenomelus sisyrinchium	Liphanthus sabulosus	17	0.02	0.02	0.01	0.02
1	0	0.22	0	t	1	Loasa heterophylla	Vespidae n.i.	17	0.00	0.03	0.01	0.03
0	0	0.43	0	cv	1	Chilimelissa sp.	Liphanthus sp.2	17	0.01	0.03	0.02	0.02
1	0	0.26	0	t	1	Mesonychium gayi	Leucocoryne ixioides	17	0.00	0.03	0.00	0.02
1	0	0.31	0	t	1	Diptera n.i.	Chaetodemoticus chilensis	17	0.01	0.03	0.01	0.01
1	0	0.49	0	t	1	Trichocereus chiloensis	Xanthocolletes incahuasi	17	0.02	0.02	0.01	0.02
0	0	0.39	0	cv	1	Barneoudia major	Salpiglossus sp.	17	0.02	0.04	0.00	0.02
0	0	0.31	0	t	1	Calceolaria montana	Bibionidae sp. 2	17	0.01	0.03	0.01	0.04
0	0	0.22	0	t	1	Adesmia conferta	Habromyia lipoflava	17	0.01	0.04	0.00	0.04
1	0	0.31	0	t	1	Adesmia exilis	Salpiglossus sp.	17	0.01	0.02	0.00	0.02
1	0	0.48	0	cv	1	Berberis empetrifolia	Elaphroptera relicta	17	0.00	0.04	0.03	0.03
0	0	0.46	0	t	1	Hippeastrum uniflorum	Etcheverrius chilensis	17	0.00	0.00	0.02	0.02
1	0	0.24	0	t	1	Alstromemeria pallida	Senecio bustillosianus	17	0.00	0.03	0.01	0.03
1	0	0.30	0	t	1	Scypanthus elegans	Panurgineae sp.	17	0.00	0.03	0.01	0.02
1	0	0.22	0	cv	1	Scypanthus elegans	Hypochoeris sp.	17	0.00	0.03	0.01	0.04
1	0	0.40	0	t	1	Trichocereus chiloensis	Clarkia tenella	17	0.02	0.02	0.00	0.02
1	0	0.30	0	t	1	Mustisia sinuata	Anthophora incerta	17	0.02	0.04	0.00	0.03
1	0	0.27	0	t	1	Verbena scoparia	Agromyzidae sp.1	17	0.00	0.03	0.01	0.03
0	0	0.28	0	cv	1	Yramea lathonoides	Eristalis tenax	17	0.01	0.03	0.00	0.03
0	0	0.35	0	t	1	Sarcophagidae n.i.	Anthophora incerta	17	0.02	0.03	0.00	0.03
0	0	0.39	0	t	1	Adesmia montana	Barneoudia major	17	0.00	0.02	0.02	0.04
0	0	0.35	0	t	1	Tristagama sessile	Bibionidae sp. 2	17	0.01	0.02	0.01	0.04
0	1	0.63	0	cv	1	Alloscirtetica sp.	Hypodynerus chiliotus	17	0.00	0.03	0.05	0.04

1	0	0.42	0	t	1	Madia sativa	Calceolaria sp.	17	0.01	0.02	0.01	0.02
1	0	0.28	0	t	1	Adesmia aconcaguensis	Anarthrophyllum cumingii	17	0.01	0.04	0.00	0.03
1	0	0.29	0	t	1	Mesonychium gayi	Senecio francisci	17	0.00	0.03	0.01	0.02
0	0	0.33	0	cv	1	Clarkia tenella	Calandrinia affinis	17	0.00	0.02	0.01	0.04
1	0	0.30	0	t	1	Vibrissomyia sp.	Calandrinia affinis	17	0.00	0.03	0.01	0.04
1	0	0.42	0	t	1	Sisyrinchium philippii	Xanthocolletes sicheli	17	0.01	0.04	0.03	0.03
0	0	0.39	0	t	1	Chilicola sp.	Alloscirtetica gayi	17	0.01	0.03	0.02	0.02
1	0	0.42	0	cv	1	Astragalus sp.	Adesmia aconcaguensis	17	0.02	0.03	0.01	0.04
1	1	0.53	0	t	1	Trichocereus chiloensis	Tropaeolum tricolor	17	0.02	0.02	0.01	0.02
0	0	0.32	0	t	1	Tabanidae n.i.	Anthophora sp.	17	0.01	0.03	0.01	0.03
1	0	0.45	0	t	1	Villa arenarius	Astragalus curvicaulis	17	0.02	0.03	0.01	0.03
1	0	0.40	0	cv	1	Tropaeolum polyphyllum	Verbena scoparia	17	0.02	0.02	0.00	0.03
0	0	0.26	0	t	1	Isepeolus niveiventris	Alloscirtetica rufitarsis	17	0.00	0.03	0.01	0.03
0	0	0.28	0	t	1	Andinus venustus	Caupolicana dimidiata	17	0.00	0.02	0.01	0.02
1	0	0.42	0	t	1	Barneoudia major	Faunula leucoglene	17	0.02	0.04	0.01	0.03
1	0	0.29	0	cv	1	Adesmia conferta	Quinchamalium chilensis	17	0.01	0.04	0.01	0.04
1	0	0.27	0	t	1	Adesmia montana	Megachile semirufa	17	0.00	0.02	0.01	0.02
1	0	0.30	0	t	1	Chaetanthera microphylla	Phacelia secunda	17	0.00	0.04	0.02	0.04
1	0	0.30	0	t	1	Viola atropurpurea	Tristagama sessile	17	0.00	0.02	0.01	0.02
0	0	0.25	0	cv	1	Vibrissomyia sp.	Ruizantheda mutabilis	17	0.00	0.03	0.01	0.03
0	0	0.21	0	t	1	Erodium cicutarium	Salpiglossus sp.	17	0.00	0.04	0.00	0.02
0	0	0.43	0	t	1	Villa sp.	Villa verdensis	17	0.01	0.03	0.01	0.03
1	0	0.31	0	t	1	Ruizantheda mutabilis	Troch n.i.	17	0.01	0.03	0.00	0.00
1	0	0.29	0	cv	1	Lepidium suffruticosum	Loasa sigmoidea	17	0.00	0.01	0.00	0.02
0	0	0.24	0	t	1	Acaena pinnatifida	Senecio francisci	17	0.00	0.04	0.01	0.02

1	0	0.22	0	t	1	Dasybasis sp. 2	Malesherbia linearifolius	17	0.00	0.03	0.00	0.03
0	1	0.77	0	t	1	Scaeva melanostoma	Corynura sp.	17	0.06	0.03	0.00	0.01
1	0	0.34	0	cv	1	Anthomyiidae sp.4	Barneoudia chilensis	17	0.01	0.02	0.01	0.03
1	0	0.27	0	t	1	Mustisia subulata	Ruiziella luctuosa	17	0.00	0.03	0.01	0.02
1	0	0.26	0	t	1	Acaena pinnatifida	Tabanidae n.i.	17	0.00	0.04	0.01	0.03
1	0	0.34	0	t	1	Bombyllidae n.i.	Liphanthus sabulosus	17	0.00	0.01	0.01	0.02
1	0	0.25	0	cv	1	Hypodynerus sp.2	Mustisia subulata	17	0.01	0.02	0.00	0.03
1	0	0.34	0	t	1	Schizanthus garhamii	Megachile sp. 1	17	0.00	0.03	0.01	0.03
0	0	0.43	0	t	1	Centris cineraria	Anthidium chilensis	17	0.01	0.02	0.02	0.03
0	0	0.41	0	t	1	Tropaeolum tricolor	Alloscirtetica rufitarsis	17	0.01	0.02	0.01	0.03
1	1	0.55	0	cv	1	Anthidium decaspilum	Senecio erucaeformis	17	0.03	0.03	0.01	0.03
1	0	0.26	0	t	1	Schizanthus garhamii	Solanum tomatillo	17	0.00	0.03	0.00	0.02
0	0	0.40	0	t	1	Truphoecus bellus	Pseudolucia chilensis	17	0.02	0.03	0.01	0.03
1	0	0.25	0	t	1	Hypodynerus sp.2	Verbena scoparia	17	0.01	0.02	0.00	0.03
0	0	0.25	0	cv	1	Anthidium funereum	Dasybasis sp. 1	17	0.00	0.02	0.00	0.03
0	1	0.68	0	t	1	Bombus dahlbomii	Hypodynerus chiliotus	17	0.01	0.03	0.05	0.04
1	1	0.59	0	t	1	Nassauvia heterophylla	Stachys albicaulis	17	0.04	0.03	0.01	0.03
0	0	0.36	0	t	1	Trichocereus chiloensis	Anthidium sp.	17	0.02	0.02	0.00	0.03
0	0	0.24	0	cv	1	Bombyllidae n.i.	Sanicula graveolens	17	0.00	0.01	0.00	0.04
0	0	0.43	0	t	1	Salpiglossus sp.	Anthidium chilensis	17	0.00	0.02	0.02	0.03
1	0	0.35	0	t	1	Adesmia exilis	Calceolaria biflora	17	0.01	0.02	0.00	0.00
1	0	0.33	1	t	1	Stenodraba stenophylla	Anthomyiida e n.i.	18	0.01	0.02	0.01	0.01
0	0	0.26	1	cv	1	Stenodraba stenophylla	Diptera n.i.	18	0.01	0.02	0.01	0.04
0	0	0.45	1	t	1	Gayella reedi	Astragalus curvicaulis	18	0.01	0.03	0.02	0.04
1	0	0.38	1	t	1	Gayella reedi	Anarthrophyll um cumingii	18	0.01	0.03	0.01	0.02

1	1	0.81	1	t	1	Scaeva melanostoma	Berberis empetrifolia	18	0.05	0.04	0.02	0.04
1	1	0.71	1	cv	1	Scaeva melanostoma	Calandrinia sericea	18	0.05	0.04	0.01	0.04
1	1	0.71	1	t	1	Scaeva melanostoma	Acaena pinnatifida	18	0.05	0.04	0.01	0.04
1	1	0.84	1	t	1	Scaeva melanostoma	L. acaulis	18	0.05	0.04	0.03	0.04
1	1	0.91	1	t	1	Scaeva melanostoma	Senecio sp.	18	0.05	0.04	0.04	0.04
0	1	0.71	1	cv	1	Scaeva melanostoma	Chuquiraga oppositifolia	18	0.05	0.04	0.01	0.04
1	1	0.86	1	t	1	Scaeva melanostoma	Tropaeolum polyphyllum	18	0.05	0.04	0.03	0.04
0	1	0.83	1	t	1	Scaeva melanostoma	Senecio bustillosianus	18	0.05	0.04	0.03	0.04
1	1	0.86	1	t	1	Scaeva melanostoma	Senecio francisci	18	0.05	0.04	0.03	0.04
1	1	0.92	1	cv	1	Scaeva melanostoma	Euphorbia portulacoides	18	0.05	0.04	0.05	0.04
1	1	0.79	1	t	1	Scaeva melanostoma	Sanicula graveolens	18	0.05	0.04	0.02	0.04
1	1	0.71	1	t	1	Scaeva melanostoma	Nothoscordu m andinum	18	0.05	0.04	0.01	0.04
1	1	0.90	1	t	1	Scaeva melanostoma	Chaetanthera euphrasioide s	18	0.05	0.04	0.04	0.04
1	1	0.72	1	cv	1	Scaeva melanostoma	Calandrinia affinis	18	0.05	0.04	0.01	0.04
1	1	0.78	1	t	1	Scaeva melanostoma	Nassauvia axillaris	18	0.05	0.04	0.02	0.04
0	1	0.51	1	t	1	Mustisia sinuata	Bombus dahlbomii	18	0.01	0.04	0.03	0.04
0	0	0.23	1	t	1	Mustisia sinuata	Etcheverrius chilensis	18	0.01	0.04	0.01	0.04
1	1	0.77	1	cv	1	Berberis empetrifolia	Sarcophagida e n.i.	18	0.02	0.04	0.05	0.03
0	0	0.36	1	t	1	Berberis empetrifolia	Tachinidae n.i.	18	0.02	0.04	0.01	0.04
1	1	0.79	1	t	1	Berberis empetrifolia	Hypodynerus chiliotus	18	0.02	0.04	0.05	0.04
1	1	0.66	1	t	1	Berberis empetrifolia	Caenohalictu s sp.	18	0.02	0.04	0.03	0.04
1	1	0.67	1	cv	1	Calandrinia sericea	Sarcophagida e n.i.	18	0.01	0.04	0.05	0.03
0	0	0.47	1	t	1	Senecio crithmoides	Andinus venustus	18	0.02	0.04	0.02	0.05
0	0	0.33	1	t	1	Senecio crithmoides	Etcheverrius chilensis	18	0.02	0.04	0.01	0.04

0	1	0.68	1	t	1	Senecio crithmoides	Auca coctei-pales	18	0.02	0.04	0.04	0.05
0	0	0.46	1	cv	1	Senecio crithmoides	Panurgineae sp.	18	0.02	0.04	0.02	0.05
0	1	0.65	1	t	1	Senecio crithmoides	Caenohalictus sp.	18	0.02	0.04	0.03	0.04
0	0	0.40	1	t	1	Vibrissomyia sp.	Nassauvia heterophylla	18	0.01	0.03	0.02	0.03
0	1	0.53	1	t	1	Vibrissomyia sp.	Senecio francisci	18	0.01	0.03	0.03	0.04
0	1	0.60	1	cv	1	Tabanidae n.i.	Euphorbia portulacoides	18	0.01	0.04	0.05	0.04
0	1	0.56	1	t	1	Villa arenarius	Chaetanthera euphrasioides	18	0.01	0.04	0.04	0.04
0	0	0.24	1	t	1	Calandrinia dianthoides	Diptera n.i.	18	0.01	0.03	0.01	0.04
1	1	0.78	1	t	1	Sarcophagidae n.i.	Azorella monantha	18	0.05	0.03	0.02	0.03
1	1	0.80	1	cv	1	Sarcophagidae n.i.	L. acaulis	18	0.05	0.03	0.03	0.04
1	1	0.80	1	t	1	Sarcophagidae n.i.	Discaria nana	18	0.05	0.03	0.03	0.03
0	1	0.79	1	t	1	Sarcophagidae n.i.	Senecio bustillosianus	18	0.05	0.03	0.03	0.04
1	1	0.80	1	t	1	Sarcophagidae n.i.	Werneria pygmaea	18	0.05	0.03	0.03	0.04
0	1	0.74	1	cv	1	Sarcophagidae n.i.	Oxalis compacta	18	0.05	0.03	0.02	0.04
0	1	0.77	1	t	1	Sarcophagidae n.i.	Astragalus curvicaulis	18	0.05	0.03	0.02	0.04
1	1	0.66	1	t	1	Sarcophagidae n.i.	Calandrinia affinis	18	0.05	0.03	0.01	0.04
1	1	0.70	1	t	1	Sarcophagidae n.i.	Azorella bolacina	18	0.05	0.03	0.02	0.04
0	1	0.73	1	cv	1	Sarcophagidae n.i.	Nassauvia axillaris	18	0.05	0.03	0.02	0.04
1	1	0.80	1	t	1	Bombus dahlbomii	Senecio sp.	18	0.03	0.04	0.04	0.04
1	1	0.64	1	t	1	Bombus dahlbomii	Loasa caespitosa	18	0.03	0.04	0.02	0.04
0	0	0.49	1	t	1	Bombus dahlbomii	Chuquiraga oppositifolia	18	0.03	0.04	0.01	0.04
1	1	0.92	1	cv	1	Bombus dahlbomii	Senecio erucaeformis	18	0.03	0.04	0.07	0.05
1	1	0.67	1	t	1	Bombus dahlbomii	Senecio bustillosianus	18	0.03	0.04	0.03	0.04
0	0	0.44	1	t	1	Bombus dahlbomii	Cerastium arvense	18	0.03	0.04	0.01	0.04

1	1	0.61	1	t	1	Bombus dahlbomii	Oxalis compacta	18	0.03	0.04	0.02	0.04
0	1	0.56	1	cv	1	Lycophlaeba lugubris	Chaetanthera euphrasioides	18	0.01	0.04	0.04	0.04
1	0	0.42	1	t	1	Nastanthus spathulatus	Hymenoptera n.i.	18	0.01	0.02	0.02	0.03
0	1	0.56	1	t	1	Villa semifuscata	Chaetanthera euphrasioides	18	0.01	0.04	0.04	0.04
1	1	0.63	1	t	1	Azorella monantha	Prosopochaeta caliginosa	18	0.02	0.03	0.03	0.03
1	0	0.38	1	cv	1	Azorella monantha	Tachinidae n.i.	18	0.02	0.03	0.01	0.04
1	0	0.38	1	t	1	Azorella monantha	Diptera n.i.	18	0.02	0.03	0.01	0.04
1	1	0.59	1	t	1	Azorella monantha	Habromyia lipoflava	18	0.02	0.03	0.02	0.03
1	1	0.65	1	t	1	L. acaulis	Prosopochaeta caliginosa	18	0.03	0.04	0.03	0.03
1	0	0.41	1	cv	1	L. acaulis	Tachinidae n.i.	18	0.03	0.04	0.01	0.04
0	0	0.41	1	t	1	L. acaulis	Diptera n.i.	18	0.03	0.04	0.01	0.04
1	1	0.62	1	t	1	L. acaulis	Habromyia lipoflava	18	0.03	0.04	0.02	0.03
1	1	0.59	1	t	1	Lepidoptera	Discaria nana	18	0.02	0.03	0.03	0.03
1	0	0.44	1	cv	1	Lepidoptera n.i.	Tristagama nivale	18	0.02	0.03	0.01	0.03
0	1	0.62	1	t	1	Lepidoptera n.i.	Senecio francisci	18	0.02	0.03	0.03	0.04
0	1	0.51	1	t	1	Lepidoptera n.i.	Oxalis compacta	18	0.02	0.03	0.02	0.04
0	1	0.56	1	t	1	Senecio sp.	Tachinidae n.i.	18	0.04	0.04	0.01	0.04
0	1	0.67	1	cv	1	Senecio sp.	Villa sp.	18	0.04	0.04	0.02	0.04
0	1	0.53	1	t	1	Senecio sp.	Anthidium funereum	18	0.04	0.04	0.01	0.04
0	1	0.56	1	t	1	Senecio sp.	Diptera n.i.	18	0.04	0.04	0.01	0.04
1	1	0.81	1	t	1	Senecio sp.	Bibionidae sp. 1	18	0.04	0.04	0.03	0.04
0	1	0.54	1	cv	1	Senecio sp.	Etcheverrius chilensis	18	0.04	0.04	0.01	0.04
0	1	0.62	1	t	1	Senecio sp.	Faunula leucoglene	18	0.04	0.04	0.01	0.04
0	1	0.61	1	t	1	Senecio sp.	Bombylliidae n.i.	18	0.04	0.04	0.01	0.04
1	1	0.66	1	t	1	Senecio sp.	Pseudolucia chilensis	18	0.04	0.04	0.02	0.04
1	0	0.46	1	cv	1	Ateloglutus sp.	Discaria nana	18	0.01	0.03	0.03	0.03

0	0	0.36	1	t	1	Yramea lathonoides	Oxalis compacta	18	0.01	0.03	0.02	0.04
1	0	0.39	1	t	1	Villa verdensis	Nassauvia heterophylla	18	0.01	0.02	0.02	0.03
0	0	0.41	1	t	1	Hymenoptera n.i.	Anarthrophyll um gayanum	18	0.02	0.03	0.01	0.04
1	1	0.56	1	cv	1	Hymenoptera n.i.	Oxalis geminata	18	0.02	0.03	0.02	0.02
1	0	0.43	1	t	1	Hymenoptera n.i.	Calandrinia affinis	18	0.02	0.03	0.01	0.04
1	1	0.67	1	t	1	Prosopochaet a caliginosa	Discaria nana	18	0.03	0.03	0.03	0.03
0	1	0.58	1	t	1	Prosopochaet a caliginosa	Oxalis compacta	18	0.03	0.03	0.02	0.04
0	1	0.58	1	cv	1	Prosopochaet a caliginosa	Sanicula graveolens	18	0.03	0.03	0.02	0.04
1	0	0.49	1	t	1	Prosopochaet a caliginosa	Calandrinia affinis	18	0.03	0.03	0.01	0.04
1	0	0.41	1	t	1	Hypodynerus sp.2	Anarthrophyll um cumingii	18	0.01	0.02	0.01	0.02
1	0	0.36	1	t	1	Hypodynerus sp.2	Calandrinia affinis	18	0.01	0.02	0.01	0.04
1	0	0.42	1	cv	1	Discaria nana	Tachinidae n.i.	18	0.03	0.03	0.01	0.04
1	1	0.63	1	t	1	Discaria nana	Habromyia lipoflava	18	0.03	0.03	0.02	0.03
0	0	0.38	1	t	1	Loasa caespitosa	Megachile sp.4	18	0.02	0.04	0.01	0.03
0	1	0.58	1	t	1	Loasa caespitosa	Megachile sp.5	18	0.02	0.04	0.02	0.03
0	0	0.44	1	cv	1	Loasa caespitosa	Anthidium sp.	18	0.02	0.04	0.01	0.03
0	0	0.40	1	t	1	Loasa caespitosa	Megachile semirufa	18	0.02	0.04	0.01	0.04
0	0	0.34	1	t	1	Andinus venustus	Chuquiraga oppositifolia	18	0.02	0.05	0.01	0.04
1	1	0.86	1	t	1	Andinus venustus	Senecio erucaeformis	18	0.02	0.05	0.07	0.05
0	0	0.45	1	cv	1	Andinus venustus	Oxalis compacta	18	0.02	0.05	0.02	0.04
1	1	0.77	1	t	1	Calceolaria biflora	Centris nigerrima	18	0.05	0.04	0.02	0.04
0	1	0.60	1	t	1	Calceolaria biflora	Centris cana	18	0.05	0.04	0.01	0.04
0	0	0.30	1	t	1	Alloscirtetica sp.	Anarthrophyll um gayanum	18	0.01	0.04	0.01	0.04
0	0	0.42	1	cv	1	Alloscirtetica sp.	Astragalus curvicaulis	18	0.01	0.04	0.02	0.04
0	0	0.26	1	t	1	Anarthrophyll um gayanum	Centris cineraria	18	0.01	0.04	0.01	0.03

0	1	0.69	1	t	1	Anarthrophyllum gayanum	Hypodynerus chiliotus	18	0.01	0.04	0.05	0.04
0	0	0.33	1	t	1	Anarthrophyllum gayanum	Pseudolucia chilensis	18	0.01	0.04	0.02	0.04
0	0	0.38	1	cv	1	Ranunculus peduncularis	Diptera n.i.	18	0.02	0.03	0.01	0.04
1	1	0.68	1	t	1	Ranunculus peduncularis	Caenohalictus sp.	18	0.02	0.03	0.03	0.04
0	0	0.25	1	t	1	Chuquiraga oppositifolia	Diptera n.i.	18	0.01	0.04	0.01	0.04
0	0	0.23	1	t	1	Chuquiraga oppositifolia	Etcheverrius chilensis	18	0.01	0.04	0.01	0.04
0	0	0.29	1	cv	1	Chuquiraga oppositifolia	Faunula leucoglene	18	0.01	0.04	0.01	0.04
0	1	0.56	1	t	1	Chuquiraga oppositifolia	Auca coctei-pales	18	0.01	0.04	0.04	0.05
1	1	0.65	1	t	1	Tropaeolum polyphyllum	Megachile sp.5	18	0.03	0.04	0.02	0.03
1	1	0.51	1	t	1	Tropaeolum polyphyllum	Anthidium sp.	18	0.03	0.04	0.01	0.03
1	0	0.45	1	cv	1	Tropaeolum polyphyllum	Troch n.i.	18	0.03	0.04	0.01	0.03
1	1	0.61	1	t	1	Tropaeolum polyphyllum	Yramea modesta	18	0.03	0.04	0.02	0.03
0	1	0.75	1	t	1	Vespidae n.i.	Euphorbia portulacoides	18	0.02	0.04	0.05	0.04
1	1	0.76	1	t	1	Chilicola sp.	Senecio erucaeformis	18	0.01	0.05	0.07	0.05
0	0	0.41	1	cv	1	Tachinidae n.i.	Senecio bustillosianus	18	0.01	0.04	0.03	0.04
0	0	0.22	1	t	1	Tachinidae n.i.	Cerastium arvense	18	0.01	0.04	0.01	0.04
0	1	0.61	1	t	1	Tachinidae n.i.	Euphorbia portulacoides	18	0.01	0.04	0.05	0.04
0	0	0.30	1	t	1	Tachinidae n.i.	Azorella bolacina	18	0.01	0.04	0.02	0.04
0	1	0.56	1	cv	1	Spathipalpus philippii	Chaetanthera euphrasioides	18	0.01	0.04	0.04	0.04
1	1	0.53	1	t	1	Chaetanthera pusilla	Diptera n.i.	18	0.04	0.03	0.01	0.04
1	1	0.59	1	t	1	Chaetanthera pusilla	Faunula leucoglene	18	0.04	0.03	0.01	0.04
1	1	0.69	1	t	1	Chaetanthera pusilla	Yramea modesta	18	0.04	0.03	0.02	0.03
0	0	0.39	1	cv	1	Centris cineraria	Astragalus curvicaulis	18	0.01	0.03	0.02	0.04

0	0	0.32	1	t	1	Centris cineraria	Anarthrophyllum cumingii	18	0.01	0.03	0.01	0.02
0	0	0.43	1	t	1	Villa sp.	Nassauvia heterophylla	18	0.02	0.04	0.02	0.03
0	1	0.56	1	t	1	Villa sp.	Senecio francisci	18	0.02	0.04	0.03	0.04
1	1	0.66	1	cv	1	Villa sp.	Chaetanthera euphrasioides	18	0.02	0.04	0.04	0.04
0	0	0.47	1	t	1	Calceolaria arachnoides	Centris nigerrima	18	0.02	0.04	0.02	0.04
1	1	0.61	1	t	1	Oxalis geminata	Coleoptera sp.	18	0.02	0.02	0.02	0.02
1	0	0.38	1	t	1	Oxalis	Diptera n.i.	18	0.02	0.02	0.01	0.04
0	1	0.56	1	cv	1	Villa gayi	Chaetanthera euphrasioides	18	0.01	0.04	0.04	0.04
1	0	0.49	1	t	1	Nassauvia heterophylla	Dasybasis sp. 1	18	0.02	0.03	0.02	0.02
0	0	0.33	1	t	1	Nassauvia heterophylla	Diptera n.i.	18	0.02	0.03	0.01	0.04
0	0	0.24	1	t	1	Erigeron andicola	Diptera n.i.	18	0.01	0.03	0.01	0.04
0	0	0.41	1	cv	1	Coleoptera sp.	Cerastium arvense	18	0.02	0.02	0.01	0.04
0	1	0.54	1	t	1	Megachile sp.5	Oxalis compacta	18	0.02	0.03	0.02	0.04
1	0	0.39	1	t	1	Megachile sp.2	Anarthrophyllum cumingii	18	0.01	0.01	0.01	0.02
0	1	0.66	1	t	1	Villa hypoxantha	Chaetanthera euphrasioides	18	0.02	0.03	0.04	0.04
0	0	0.36	1	cv	1	Villa hypoxantha	Calandrinia affinis	18	0.02	0.03	0.01	0.04
0	0	0.37	1	t	1	Phulia nymphula	Oxalis compacta	18	0.01	0.03	0.02	0.04
0	0	0.28	1	t	1	Phulia nymphula	Calandrinia affinis	18	0.01	0.03	0.01	0.04
0	0	0.43	1	t	1	Colletes sp.	Astragalus curvicaulis	18	0.01	0.04	0.02	0.04
1	1	0.81	1	cv	1	Megachile semirufa	Senecio erucaeformis	18	0.01	0.04	0.07	0.05
0	0	0.43	1	t	1	Megachile semirufa	Senecio bustillosianus	18	0.01	0.04	0.03	0.04
0	0	0.39	1	t	1	Megachile semirufa	Astragalus curvicaulis	18	0.01	0.04	0.02	0.04
1	1	0.77	1	t	1	Anthidium funereum	Senecio erucaeformis	18	0.01	0.04	0.07	0.05

0	0	0.38	1	cv	1	Anthidium funereum	Senecio bustillosianus	18	0.01	0.04	0.03	0.04
1	1	0.77	1	t	1	Tatochila mercedis	Senecio erucaeformis	18	0.01	0.05	0.07	0.05
0	0	0.31	1	t	1	Tatochila mercedis	Oxalis compacta	18	0.01	0.05	0.02	0.04
1	1	0.76	1	t	1	Tatochila blanchardii	Senecio erucaeformis	18	0.01	0.05	0.07	0.05
0	1	0.79	1	cv	1	Diptera n.i.	Senecio erucaeformis	18	0.01	0.04	0.07	0.05
0	0	0.41	1	t	1	Diptera n.i.	Senecio bustillosianus	18	0.01	0.04	0.03	0.04
0	0	0.21	1	t	1	Diptera n.i.	Cerastium arvense	18	0.01	0.04	0.01	0.04
0	0	0.42	1	t	1	Diptera n.i.	Werneria pygmaea	18	0.01	0.04	0.03	0.04
0	1	0.61	1	cv	1	Diptera n.i.	Euphorbia portulacoides	18	0.01	0.04	0.05	0.04
0	0	0.34	1	t	1	Diptera n.i.	Sanicula graveolens	18	0.01	0.04	0.02	0.04
0	0	0.23	1	t	1	Diptera n.i.	Cerastium montioides	18	0.01	0.04	0.01	0.03
0	0	0.38	1	t	1	Diptera n.i.	Cardamine nivalis	18	0.01	0.04	0.02	0.03
0	0	0.27	1	cv	1	Diptera n.i.	Calandrinia affinis	18	0.01	0.04	0.01	0.04
0	0	0.30	1	t	1	Diptera n.i.	Azorella bolacina	18	0.01	0.04	0.02	0.04
1	1	0.96	1	t	1	Senecio erucaeformis	Hypodynerus chiliotus	18	0.07	0.05	0.05	0.04
0	1	0.75	1	t	1	Senecio erucaeformis	Etcheverrius chilensis	18	0.07	0.05	0.01	0.04
1	1	0.81	1	cv	1	Senecio erucaeformis	Megachile sp. 1	18	0.07	0.05	0.02	0.05
1	1	0.93	1	t	1	Senecio erucaeformis	Auca coctei-pales	18	0.07	0.05	0.04	0.05
1	1	0.84	1	t	1	Senecio erucaeformis	Panurgineae sp.	18	0.07	0.05	0.02	0.05
0	1	0.73	1	t	1	Senecio erucaeformis	Hypodynerus sp. 3	18	0.07	0.05	0.01	0.05
0	1	0.61	1	cv	1	Hypodynerus chiliotus	Cerastium arvense	18	0.05	0.04	0.01	0.04
1	1	0.78	1	t	1	Hypodynerus chiliotus	Astragalus curvicaulis	18	0.05	0.04	0.02	0.04
0	1	0.74	1	t	1	Hypodynerus chiliotus	Nassauvia axillaris	18	0.05	0.04	0.02	0.04
0	0	0.37	1	t	1	Senecio bustillosianus	Etcheverrius chilensis	18	0.03	0.04	0.01	0.04
0	0	0.47	1	cv	1	Senecio bustillosianus	Megachile sp. 1	18	0.03	0.04	0.02	0.05

0	1	0.72	1	t	1	Senecio bustillosianus	Auca coctei-pales	18	0.03	0.04	0.04	0.05
1	1	0.69	1	t	1	Anthomyiidae sp.2	Sanicula graveolens	18	0.04	0.03	0.02	0.04
1	1	0.61	1	t	1	Anthomyiidae sp.2	Calandrinia affinis	18	0.04	0.03	0.01	0.04
0	0	0.31	1	cv	1	Rhodophiala montana	Megachile sp. 1	18	0.01	0.04	0.02	0.05
0	0	0.25	1	t	1	Cerastium arvense	Bombylliidae n.i.	18	0.01	0.04	0.01	0.04
0	0	0.29	1	t	1	Cerastium arvense	Pseudolucia chilensis	18	0.01	0.04	0.02	0.04
0	0	0.49	1	t	1	Cerastium arvense	Caenohalictus sp.	18	0.01	0.04	0.03	0.04
0	0	0.43	1	cv	1	Etcheverrius chilensis	Senecio francisci	18	0.01	0.04	0.03	0.04
1	1	0.50	1	t	1	Senecio francisci	Faunula leucoglene	18	0.03	0.04	0.01	0.04
0	0	0.40	1	t	1	Faunula leucoglene	Oxalis compacta	18	0.01	0.04	0.02	0.04
0	0	0.37	1	t	1	Bombylliidae n.i.	Nassauvia axillaris	18	0.01	0.04	0.02	0.04
0	0	0.44	1	cv	1	Megachile sp. 1	Astragalus curvicaulis	18	0.02	0.05	0.02	0.04
0	1	0.50	1	t	1	Oxalis compacta	Yramea modesta	18	0.02	0.04	0.02	0.03
0	1	0.55	1	t	1	Oxalis compacta	Habromyia lipoflava	18	0.02	0.04	0.02	0.03
0	1	0.66	1	t	1	Astragalus curvicaulis	Caenohalictus sp.	18	0.02	0.04	0.03	0.04
0	0	0.36	1	cv	1	Astragalus curvicaulis	Colletes fulvipes	18	0.02	0.04	0.01	0.04
1	0	0.39	1	t	1	Anarthrophyllum cumingii	Alloscirtetica rufitarsis	18	0.01	0.02	0.01	0.01
0	0	0.37	1	t	1	Yramea modesta	Viola philippii	18	0.02	0.03	0.01	0.03
0	1	0.56	1	t	1	Villa flavicura	Chaetanthera euphrasioides	18	0.01	0.04	0.04	0.04
0	0	0.29	1	cv	1	Agromyzidae sp.1	Calandrinia affinis	18	0.01	0.03	0.01	0.04
1	1	0.54	1	t	1	Nothoscordum andinum	Caenohalictus sp.	18	0.01	0.04	0.03	0.04
0	0	0.29	1	t	1	Agromyzidae sp.2	Calandrinia affinis	18	0.01	0.03	0.01	0.04
1	0	0.45	1	t	1	Habromyia lipoflava	Calandrinia affinis	18	0.02	0.03	0.01	0.04
1	1	0.80	1	cv	1	Chaetanthera euphrasioides	Caenohalictus sp.	18	0.04	0.04	0.03	0.04

1	1	0.55	1	t	1	Calandrinia affinis	Caenohalictus sp.	18	0.01	0.04	0.03	0.04
0	1	0.73	0	t	1	Megachile sp.2	Hypodynerus chiliotus	18	0.01	0.01	0.05	0.04
0	0	0.22	0	t	1	Ateloglutus sp.	Anthidium funereum	18	0.01	0.03	0.01	0.04
0	1	0.55	0	cv	1	Hymenoptera n.i.	Astragalus curvicaulis	18	0.02	0.03	0.02	0.04
0	0	0.47	0	t	1	Lepidoptera n.i.	Andinus venustus	18	0.02	0.03	0.02	0.05
0	1	0.67	0	t	1	Phulia nymphula	Hypodynerus chiliotus	18	0.01	0.03	0.05	0.04
0	0	0.26	0	t	1	Alloscirtetica sp.	Villa gayi	18	0.01	0.04	0.01	0.04
1	0	0.43	0	cv	1	Discaria nana	Troch n.i.	18	0.03	0.03	0.01	0.03
0	0	0.35	0	t	1	Alloscirtetica sp.	Azorella bolacina	18	0.01	0.04	0.02	0.04
0	0	0.32	0	t	1	Megachile sp.4	Villa hypoxantha	18	0.01	0.03	0.02	0.03
0	1	0.59	0	t	1	Megachile sp.2	Caenohalictus sp.	18	0.01	0.01	0.03	0.04
0	1	0.66	0	cv	1	Alloscirtetica sp.	Euphorbia portulacoides	18	0.01	0.04	0.05	0.04
0	0	0.34	0	t	1	Hymenoptera n.i.	Tatochila mercedis	18	0.02	0.03	0.01	0.05
0	1	0.62	0	t	1	Anthidium sp.	Chaetanthera euphrasioides	18	0.01	0.03	0.04	0.04
0	0	0.34	0	t	1	Ranunculus peduncularis	Hypodynerus sp. 3	18	0.02	0.03	0.01	0.05
0	0	0.26	0	cv	1	Nastanthus spathulatus	Villa flavicura	18	0.01	0.02	0.01	0.04
1	0	0.46	0	t	1	L. acaulis	Alloscirtetica sp.	18	0.03	0.04	0.01	0.04
0	1	0.74	0	t	1	Scaeva melanostoma	Alloscirtetica rufitarsis	18	0.05	0.04	0.01	0.01
0	1	0.57	0	t	1	Lepidoptera n.i.	Senecio bustillosianus	18	0.02	0.03	0.03	0.04
0	0	0.26	0	cv	1	Acaena pinnatifida	Megachile sp.4	18	0.01	0.04	0.01	0.03
0	1	0.82	0	t	1	Chaetanthera pusilla	Auca coctei-pales	18	0.04	0.03	0.04	0.05
0	0	0.36	0	t	1	Spathipalpus philippii	Yramea modesta	18	0.01	0.04	0.02	0.03
1	1	0.61	0	t	1	Discaria nana	Loasa caespitosa	18	0.03	0.03	0.02	0.04
0	0	0.31	0	cv	1	Hypodynerus sp.2	Villa gayi	18	0.01	0.02	0.01	0.04

0	0	0.37	0	t	1	Diptera n.i.	Centris nigerrima	18	0.01	0.04	0.02	0.04
1	0	0.49	0	t	1	Villa verdensis	Discaria nana	18	0.01	0.02	0.03	0.03
1	0	0.42	0	t	1	L. acaulis	Phulia nymphula	18	0.03	0.04	0.01	0.03
1	1	0.75	0	cv	1	Calceolaria biflora	Oxalis compacta	18	0.05	0.04	0.02	0.04
0	0	0.50	0	t	1	Bombus dahlbomii	Anarthrophyllum gayanum	18	0.03	0.04	0.01	0.04
1	1	0.91	0	t	1	Scaeva melanostoma	Anthomyiidae sp.2	18	0.05	0.04	0.04	0.03
0	1	0.61	0	t	1	Villa verdensis	Chaetanthera euphrasioides	18	0.01	0.02	0.04	0.04
0	0	0.20	0	cv	1	Lycophlaeba lugubris	Tatochila mercedis	18	0.01	0.04	0.01	0.05
0	0	0.29	0	t	1	Yramea lathonoides	Megachile sp. 1	18	0.01	0.03	0.02	0.05
0	0	0.39	0	t	1	Anthomyiidae n.i.	Panurgineae sp.	18	0.01	0.01	0.02	0.05
0	0	0.19	0	t	1	Lycophlaeba lugubris	Tatochila blanchardii	18	0.01	0.04	0.01	0.05
1	1	0.81	0	cv	1	Hypodynerus chiliotus	Werneria pygmaea	18	0.05	0.04	0.03	0.04
0	0	0.39	0	t	1	Hymenoptera n.i.	Phulia nymphula	18	0.02	0.03	0.01	0.03
0	0	0.26	0	t	1	Calceolaria arachnoides	Tatochila blanchardii	18	0.02	0.04	0.01	0.05
0	0	0.41	0	t	1	Hypodynerus sp.2	Villa hypoxantha	18	0.01	0.02	0.02	0.03
0	1	0.60	0	cv	1	Euphorbia portulacoides	Cerastium montioides	18	0.05	0.04	0.01	0.03
0	0	0.35	0	t	1	Berberis empetrifolia	Tabanidae n.i.	18	0.02	0.04	0.01	0.04
0	0	0.46	0	t	1	Centris nigerrima	Pseudolucia chilensis	18	0.02	0.04	0.02	0.04
0	0	0.28	0	t	1	Ateloglutus sp.	Bombylliidae n.i.	18	0.01	0.03	0.01	0.04
0	0	0.29	0	cv	1	Megachile sp.4	Megachile sp. 1	18	0.01	0.03	0.02	0.05
0	0	0.20	0	t	1	Tabanidae n.i.	Centris cana	18	0.01	0.04	0.01	0.04
0	1	0.57	0	t	1	Yramea lathonoides	Chaetanthera pusilla	18	0.01	0.03	0.04	0.03
0	0	0.23	0	t	1	Cerastium arvense	Agromyzidae sp.1	18	0.01	0.04	0.01	0.03
0	0	0.30	0	cv	1	Calandrinia dianthoides	Tristagama nivale	18	0.01	0.03	0.01	0.03
0	0	0.18	0	t	1	Tatochila mercedis	Centris cana	18	0.01	0.05	0.01	0.04

0	0	0.44	0	t	1	Hypodynerus sp. 3	Caenohalictus sp.	18	0.01	0.05	0.03	0.04
1	0	0.36	0	t	1	Anthomyiidae n.i.	Megachile sp.2	18	0.01	0.01	0.01	0.01
0	1	0.55	0	cv	1	Hymenoptera n.i.	Centris nigerrima	18	0.02	0.03	0.02	0.04
0	1	0.58	0	t	1	Ateloglutus sp.	Chaetanthera pusilla	18	0.01	0.03	0.04	0.03
1	0	0.33	0	t	1	Anarthrophyllum cumingii	Viola philippii	18	0.01	0.02	0.01	0.03
0	0	0.30	0	t	1	Villa flavicura	Pseudolucia chilensis	18	0.01	0.04	0.02	0.04
0	0	0.32	0	cv	1	Megachile sp.4	Panurgineae sp.	18	0.01	0.03	0.02	0.05
0	1	0.72	0	t	1	Dasybasis sp. 1	Anthomyiidae sp.2	18	0.02	0.02	0.04	0.03
0	0	0.46	0	t	1	Mustisia sinuata	Coleoptera sp.	18	0.01	0.04	0.02	0.02
1	1	0.65	0	t	1	Calandrinia sericea	Euphorbia portulacoides	18	0.01	0.04	0.05	0.04
0	0	0.21	0	cv	1	Phulia nymphula	Tatochila mercedis	18	0.01	0.03	0.01	0.05
0	0	0.34	0	t	1	Yramea modesta	Centris cana	18	0.02	0.03	0.01	0.04
0	0	0.40	0	t	1	Mustisia sinuata	Lepidoptera n.i.	18	0.01	0.04	0.02	0.03
0	1	0.70	0	t	1	Sarcophagidae n.i.	Pseudolucia chilensis	18	0.05	0.03	0.02	0.04
0	0	0.26	0	cv	1	Tristagama nivale	Anthidium funereum	18	0.01	0.03	0.01	0.04
1	1	0.58	0	t	1	Azorella monantha	Ranunculus peduncularis	18	0.02	0.03	0.02	0.03
0	0	0.36	0	t	1	Villa verdensis	Panurgineae sp.	18	0.01	0.02	0.02	0.05
0	1	0.51	0	t	1	Nastanthus spathulatus	Senecio francisci	18	0.01	0.02	0.03	0.04
0	1	0.96	0	cv	1	Calceolaria biflora	Senecio erucaeformis	18	0.05	0.04	0.07	0.05
0	0	0.44	0	t	1	Astragalus curvicaulis	Alloscirtetica rufitarsis	18	0.02	0.04	0.01	0.01
1	0	0.42	0	t	1	Azorella monantha	Chuquiraga oppositifolia	18	0.02	0.03	0.01	0.04
1	1	0.56	0	t	1	Tropaeolum polyphyllum	Villa hypoxantha	18	0.03	0.04	0.02	0.03
0	0	0.45	0	cv	1	Tristagama nivale	Vespidae n.i.	18	0.01	0.03	0.02	0.04
0	0	0.23	0	t	1	Agromyzidae sp.2	Centris cana	18	0.01	0.03	0.01	0.04
1	1	0.55	0	t	1	Tropaeolum polyphyllum	Villa sp.	18	0.03	0.04	0.02	0.04

0	0	0.39	0	t	1	Villa semifuscata	Ranunculus peduncularis	18	0.01	0.04	0.02	0.03
1	0	0.41	0	cv	1	Dasybasis sp. 1	Faunula leucoglene	18	0.02	0.02	0.01	0.04
0	1	0.58	0	t	1	Tropaeolum polyphyllum	Nassauvia axillaris	18	0.03	0.04	0.02	0.04
1	1	0.89	0	t	1	Senecio sp.	Hypodynerus chiliotus	18	0.04	0.04	0.05	0.04
0	0	0.33	0	t	1	Loasa caespitosa	Hypodynerus sp. 3	18	0.02	0.04	0.01	0.05
1	0	0.35	0	cv	1	Tachinidae n.i.	Dasybasis sp. 1	18	0.01	0.04	0.02	0.02
0	0	0.36	0	t	1	Colletes sp.	Panurgineae sp.	18	0.01	0.04	0.02	0.05
1	1	0.60	0	t	1	Ranunculus peduncularis	Senecio bustillosianus	18	0.02	0.03	0.03	0.04
1	1	0.55	0	t	1	Azorella monantha	Lepidoptera n.i.	18	0.02	0.03	0.02	0.03
0	1	0.95	0	cv	1	Senecio sp.	Senecio erucaeformis	18	0.04	0.04	0.07	0.05
1	0	0.35	0	t	1	Sanicula graveolens	Agromyzidae sp.2	18	0.02	0.04	0.01	0.03
0	0	0.46	0	t	1	Anthomyiidae n.i.	Astragalus curvicaulis	18	0.01	0.01	0.02	0.04
0	0	0.27	0	t	1	Anthidium sp.	Villa flavicura	18	0.01	0.03	0.01	0.04
1	1	0.62	0	cv	1	Chaetanthera pusilla	Pseudolucia chilensis	18	0.04	0.03	0.02	0.04
0	1	0.50	0	t	1	Hypodynerus sp.2	Ranunculus peduncularis	18	0.01	0.02	0.02	0.03
0	1	0.73	0	t	1	Hypodynerus sp.2	Calceolaria biflora	18	0.01	0.02	0.05	0.04
1	1	0.60	0	t	1	Chaetanthera euphrasioides	Calandrinia affinis	18	0.04	0.04	0.01	0.04
0	0	0.23	0	cv	1	Calandrinia sericea	Anthidium funereum	18	0.01	0.04	0.01	0.04
0	0	0.39	0	t	1	Tachinidae n.i.	Megachile sp.5	18	0.01	0.04	0.02	0.03
0	0	0.28	0	t	1	Diptera n.i.	Megachile sp. 1	18	0.01	0.04	0.02	0.05
1	0	0.27	0	t	1	Calandrinia sericea	Phulia nymphula	18	0.01	0.04	0.01	0.03
0	1	0.52	0	cv	1	Senecio sp.	Hypodynerus sp. 3	18	0.04	0.04	0.01	0.05
0	0	0.45	0	t	1	Megachile semirufa	Werneria pygmaea	18	0.01	0.04	0.03	0.04
0	1	0.60	0	t	1	Lepidoptera n.i.	Prosopochaeta caliginosa	18	0.02	0.03	0.03	0.03
0	0	0.23	0	t	1	Faunula leucoglene	Hypodynerus sp. 3	18	0.01	0.04	0.01	0.05

0	0	0.17	0	cv	1	Chilicola sp.	Etcheverrius chilensis	18	0.01	0.05	0.01	0.04
1	0	0.29	0	t	1	Megachile sp.4	Megachile sp.2	18	0.01	0.03	0.01	0.01
0	0	0.49	0	t	1	Vespidae n.i.	Nassauvia heterophylla	18	0.02	0.04	0.02	0.03
0	0	0.29	0	t	1	Tabanidae n.i.	Hypodynerus sp.2	18	0.01	0.04	0.01	0.02
0	1	0.69	0	cv	1	Scaeva melanostoma	Agromyzidae sp.1	18	0.05	0.04	0.01	0.03
0	0	0.29	0	t	1	Lycophlaeba lugubris	Megachile sp.2	18	0.01	0.04	0.01	0.01
0	0	0.39	0	t	1	L. acaulis	Centris cana	18	0.03	0.04	0.01	0.04
1	0	0.29	0	t	1	Anthomyiidae n.i.	Troch n.i.	18	0.01	0.01	0.01	0.03
0	0	0.34	0	cv	1	Azorella monantha	Tatochila blanchardii	18	0.02	0.03	0.01	0.05
0	0	0.31	0	t	1	Alloscirtetica sp.	Calandrinia affinis	18	0.01	0.04	0.01	0.04
0	0	0.22	0	t	1	Villa semifuscata	Tachinidae n.i.	18	0.01	0.04	0.01	0.04
0	1	0.59	0	t	1	Vespidae n.i.	Coleoptera sp.	18	0.02	0.04	0.02	0.02
0	0	0.34	0	cv	1	Centris cineraria	Nassauvia axillaris	18	0.01	0.03	0.02	0.04
0	0	0.33	0	t	1	Tristagama nivale	Nothoscordum andinum	18	0.01	0.03	0.01	0.04
0	1	0.52	0	t	1	Calandrinia dianthoides	Caenohalictus sp.	18	0.01	0.03	0.03	0.04
0	1	0.54	0	t	1	Senecio crithmoides	Oxalis geminata	18	0.02	0.04	0.02	0.02
1	0	0.42	0	cv	1	Anthomyiidae n.i.	Nassauvia heterophylla	18	0.01	0.01	0.02	0.03
0	0	0.25	0	t	1	Phulia nymphula	Cerastium montioides	18	0.01	0.03	0.01	0.03
0	0	0.29	0	t	1	Chuquiraga oppositifolia	Calandrinia affinis	18	0.01	0.04	0.01	0.04
0	0	0.25	0	t	1	Lycophlaeba lugubris	Rhodophiala montana	18	0.01	0.04	0.01	0.04
0	0	0.47	0	cv	1	Pseudolucia chilensis	Cardamine nivalis	18	0.02	0.04	0.02	0.03
1	1	0.78	0	t	1	Calceolaria biflora	Ranunculus peduncularis	18	0.05	0.04	0.02	0.03
0	0	0.33	0	t	1	Lepidoptera n.i.	Anthidium funereum	18	0.02	0.03	0.01	0.04
0	1	0.63	0	t	1	Senecio sp.	Megachile sp. 1	18	0.04	0.04	0.02	0.05
0	1	0.60	0	cv	1	Senecio crithmoides	Prosopochaeta caliginosa	18	0.02	0.04	0.03	0.03
0	0	0.25	0	t	1	Acaena pinnatifida	Villa gayi	18	0.01	0.04	0.01	0.04

0	0	0.50	0	t	1	Spathipalpus philippii	Caenohalictus sp.	18	0.01	0.04	0.03	0.04
1	1	0.54	0	t	1	Chaetanthera pusilla	Troch n.i.	18	0.04	0.03	0.01	0.03
1	0	0.33	0	cv	1	Nastanthus spathulatus	Tristagama nivale	18	0.01	0.02	0.01	0.03
0	0	0.46	0	t	1	Bombus dahlbomii	Troch n.i.	18	0.03	0.04	0.01	0.03
0	0	0.25	0	t	1	Mustisia sinuata	Villa semifuscata	18	0.01	0.04	0.01	0.04
0	0	0.28	0	t	1	Gayella reedi	Diptera n.i.	18	0.01	0.03	0.01	0.04
0	0	0.19	0	cv	1	Villa semifuscata	Hypodynerus sp. 3	18	0.01	0.04	0.01	0.05
0	0	0.27	0	t	1	Tachinidae n.i.	Colletes sp.	18	0.01	0.04	0.01	0.04
1	1	0.65	0	t	1	L. acaulis	Discaria nana	18	0.03	0.04	0.03	0.03
0	0	0.23	0	t	1	Yramea lathonoides	Cerastium arvense	18	0.01	0.03	0.01	0.04
0	1	0.80	0	cv	1	Troch n.i.	Senecio erucaeformis	18	0.01	0.03	0.07	0.05
0	0	0.45	0	t	1	Vespidae n.i.	Pseudolucia chilensis	18	0.02	0.04	0.02	0.04
0	0	0.21	0	t	1	Tabanidae n.i.	Colletes fulvipes	18	0.01	0.04	0.01	0.04
1	1	0.51	0	t	1	Tropaeolum polyphyllum	Calandrinia affinis	18	0.03	0.04	0.01	0.04
0	1	0.62	0	cv	1	Senecio sp.	Colletes sp.	18	0.04	0.04	0.01	0.04
0	0	0.32	0	t	1	Calandrinia sericea	Megachile sp. 1	18	0.01	0.04	0.02	0.05
0	1	0.66	0	t	1	Senecio bustillosianus	Senecio francisci	18	0.03	0.04	0.03	0.04
0	0	0.30	0	t	1	Tachinidae n.i.	Calceolaria arachnoides	18	0.01	0.04	0.02	0.04
0	0	0.24	0	cv	1	Erigeron andicola	Megachile sp.4	18	0.01	0.03	0.01	0.03
0	0	0.34	0	t	1	Dasybasis sp.	Centris cana	18	0.02	0.02	0.01	0.04
0	0	0.24	0	t	1	Villa arenarius	Megachile semirufa	18	0.01	0.04	0.01	0.04
0	0	0.42	0	t	1	Anarthrophyllum gayanum	Oxalis geminata	18	0.01	0.04	0.02	0.02
0	0	0.43	0	cv	1	Andinus venustus	Nassauvia heterophylla	18	0.02	0.05	0.02	0.03
0	1	0.55	0	t	1	Phulia nymphula	Auca coctei-pales	18	0.01	0.03	0.04	0.05
0	1	0.89	0	t	1	Oxalis geminata	Senecio erucaeformis	18	0.02	0.02	0.07	0.05
0	0	0.37	0	t	1	Centris cineraria	Yramea modesta	18	0.01	0.03	0.02	0.03
0	0	0.28	0	cv	1	Mustisia sinuata	Nastanthus spathulatus	18	0.01	0.04	0.01	0.02
0	0	0.28	0	t	1	Gayella reedi	Tachinidae n.i.	18	0.01	0.03	0.01	0.04

0	0	0.41	0	t	1	Mustisia sinuata	Astragalus curvicaulis	18	0.01	0.04	0.02	0.04
0	0	0.35	0	t	1	Oxalis geminata	Anthidium funereum	18	0.02	0.02	0.01	0.04
0	0	0.41	0	cv	1	Villa hypoxantha	Anarthrophyll um cumingii	18	0.02	0.03	0.01	0.02
0	0	0.32	0	t	1	Centris cineraria	Azorella bolacina	18	0.01	0.03	0.02	0.04
0	0	0.30	0	t	1	Vibrissomyia sp.	Ateloglutus sp.	18	0.01	0.03	0.01	0.03
0	0	0.26	0	t	1	Alloscirtetica sp.	Spathipalpus philippii	18	0.01	0.04	0.01	0.04
0	0	0.19	0	cv	1	Tatochila blanchardii	Villa flavicura	18	0.01	0.05	0.01	0.04
0	0	0.46	0	t	1	Senecio francisci	Cerastium montioides	18	0.03	0.04	0.01	0.03
0	0	0.30	0	t	1	Acaena pinnatifida	Calandrinia affinis	18	0.01	0.04	0.01	0.04
1	0	0.40	0	t	1	Azorella monantha	Agromyzidae sp.1	18	0.02	0.03	0.01	0.03
1	1	0.65	0	cv	1	Bibionidae sp. 1	Centris nigerrima	18	0.03	0.04	0.02	0.04
0	0	0.36	0	t	1	Tabanidae n.i.	Cardamine nivalis	18	0.01	0.04	0.02	0.03
0	0	0.39	0	t	1	Megachile sp.2	Villa hypoxantha	18	0.01	0.01	0.02	0.03
0	0	0.22	0	t	1	Tabanidae n.i.	Troch n.i.	18	0.01	0.04	0.01	0.03
0	0	0.26	0	cv	1	Lycophlaeba lugubris	Alloscirtetica sp.	18	0.01	0.04	0.01	0.04
0	0	0.24	0	t	1	Agromyzidae sp.2	Colletes fulvipes	18	0.01	0.03	0.01	0.04
0	0	0.45	0	t	1	Oxalis compacta	Panurgineae sp.	18	0.02	0.04	0.02	0.05
1	0	0.42	0	t	1	Vibrissomyia sp.	Dasybasis sp. 1	18	0.01	0.03	0.02	0.02
0	0	0.19	0	cv	1	Spathipalpus philippii	Hypodynerus sp. 3	18	0.01	0.04	0.01	0.05
0	1	0.77	0	t	1	Oxalis geminata	Euphorbia portulacoides	18	0.02	0.02	0.05	0.04
0	1	0.51	0	t	1	Nastanthus spathulatus	Tropaeolum polyphyllum	18	0.01	0.02	0.03	0.04
0	1	0.57	0	t	1	Anthomyiidae sp.2	Agromyzidae sp.2	18	0.04	0.03	0.01	0.03
0	0	0.30	0	cv	1	Tatochila blanchardii	Sanicula graveolens	18	0.01	0.05	0.02	0.04
0	0	0.44	0	t	1	Senecio bustillosianus	Bombylliidae n.i.	18	0.03	0.04	0.01	0.04
0	0	0.35	0	t	1	Chilicola sp.	Megachile sp.5	18	0.01	0.05	0.02	0.03

0	1	0.69	0	t	1	Senecio sp.	Nassauvia heterophylla	18	0.04	0.04	0.02	0.03
0	0	0.40	0	cv	1	Megachile sp. 1	Sanicula graveolens	18	0.02	0.05	0.02	0.04
1	0	0.41	0	t	1	Villa verdensis	Dasybasis sp. 1	18	0.01	0.02	0.02	0.02
0	0	0.25	0	t	1	Chuquiraga oppositifolia	Troch n.i.	18	0.01	0.04	0.01	0.03
0	0	0.47	0	t	1	Panurgineae sp.	Centris nigerrima	18	0.02	0.05	0.02	0.04
0	0	0.37	0	cv	1	Anarthrophyllum gayanum	Nassauvia axillaris	18	0.01	0.04	0.02	0.04
0	1	0.69	0	t	1	Prosopochaeta caliginosa	Tropaeolum polyphyllum	18	0.03	0.03	0.03	0.04
0	1	0.71	0	t	1	Scaeva melanostoma	Nastanthus spathulatus	18	0.05	0.04	0.01	0.02
0	0	0.47	0	t	1	Berberis empetrifolia	Andinus venustus	18	0.02	0.04	0.02	0.05
1	1	0.93	0	cv	1	Scaeva melanostoma	Sarcophagidae n.i.	18	0.05	0.04	0.05	0.03
0	1	0.55	0	t	1	Tachinidae n.i.	Chaetanthera pusilla	18	0.01	0.04	0.04	0.03
0	1	0.66	0	t	1	Anthomyiidae sp.2	Anarthrophyllum cumingii	18	0.04	0.03	0.01	0.02
0	0	0.35	0	t	1	Bombylliidae n.i.	Panurgineae sp.	18	0.01	0.04	0.02	0.05
0	0	0.33	0	cv	1	Berberis empetrifolia	Anthidium funereum	18	0.02	0.04	0.01	0.04
0	0	0.34	0	t	1	Mustisia sinuata	Azorella bolacina	18	0.01	0.04	0.02	0.04
0	0	0.45	0	t	1	Yramea modesta	Pseudolucia chilensis	18	0.02	0.03	0.02	0.04
0	0	0.44	0	t	1	Hymenoptera n.i.	Megachile sp. 1	18	0.02	0.03	0.02	0.05
0	1	0.79	0	cv	1	Hymenoptera n.i.	Calceolaria biflora	18	0.02	0.03	0.05	0.04
0	1	0.58	0	t	1	Senecio bustillosianus	Cardamine nivalis	18	0.03	0.04	0.02	0.03
0	1	0.77	0	t	1	Sarcophagidae n.i.	Loasa caespitosa	18	0.05	0.03	0.02	0.04
0	0	0.26	0	t	1	Villa verdensis	Diptera n.i.	18	0.01	0.02	0.01	0.04
0	1	0.75	0	cv	1	Senecio sp.	Habromyia lipoflava	18	0.04	0.04	0.02	0.03
0	1	0.64	1	t	1	Andinus venustus	Oxalis compacta	19	0.05	0.05	0.01	0.05
0	0	0.39	1	t	1	Valeriana radicalis	Diptera n.i.	19	0.02	0.05	0.02	0.06
0	0	0.38	1	t	1	Faunula leucoglene	Chaetanthera flabellata	19	0.01	0.03	0.01	0.03
0	0	0.34	1	cv	1	Faunula leucoglene	Chaetanthera apiculata	19	0.01	0.03	0.01	0.04

1	1	0.62	1	t	1	Faunula leucoglene	Adesmia glomerula	19	0.01	0.03	0.04	0.03
0	0	0.34	1	t	1	Faunula leucoglene	Chaetanthera lycopodioides	19	0.01	0.03	0.01	0.04
0	0	0.39	1	t	1	Acaena pinnatifida	Scaeva melanostoma	19	0.04	0.05	0.01	0.07
1	1	0.68	1	cv	1	Calandrinia sericea	Sarcophagidae sp.	19	0.01	0.04	0.05	0.04
0	0	0.29	1	t	1	Nassauvia pinnigera	Diptera n.i.	19	0.01	0.05	0.02	0.06
0	0	0.38	1	t	1	Megachile sp. 5	Tropaeolum polyphyllum	19	0.01	0.04	0.02	0.04
0	0	0.31	1	t	1	Megachile sp. 5	Loasa caespitosa	19	0.01	0.04	0.01	0.03
0	0	0.41	1	cv	1	Megachile sp. 5	Phacelia secunda	19	0.01	0.04	0.03	0.06
0	0	0.48	1	t	1	Erigeron andicola	Diptera n.i.	19	0.03	0.05	0.02	0.06
0	0	0.29	1	t	1	Draba gilliesii	Phulia nymphula	19	0.01	0.05	0.01	0.04
0	0	0.29	1	t	1	Draba gilliesii	Sarcophagidae n.i.	19	0.01	0.05	0.01	0.04
0	0	0.20	1	cv	1	Draba gilliesii	Scaeva melanostoma	19	0.01	0.05	0.01	0.07
1	1	0.86	1	t	1	Oreopolus glacialis	Yramea modesta	19	0.03	0.04	0.05	0.04
1	0	0.48	1	t	1	Oreopolus glacialis	Phulia nymphula	19	0.03	0.04	0.01	0.04
1	1	0.67	1	t	1	Oreopolus glacialis	Pseudolucia chilensis	19	0.03	0.04	0.03	0.03
1	1	0.76	1	cv	1	Megachile semirufa	Phacelia secunda	19	0.05	0.05	0.03	0.06
0	0	0.25	1	t	1	Oxalis compacta	Cynthia terpsichore	19	0.01	0.05	0.01	0.04
0	0	0.25	1	t	1	Oxalis compacta	Tatochila mercedis	19	0.01	0.05	0.01	0.04
0	1	0.79	1	t	1	Oxalis compacta	Hypsochila wagenknecti	19	0.01	0.05	0.07	0.07
0	1	0.70	1	cv	1	Oxalis compacta	Yramea modesta	19	0.01	0.05	0.05	0.04
0	0	0.26	1	t	1	Oxalis compacta	Phulia nymphula	19	0.01	0.05	0.01	0.04
0	0	0.42	1	t	1	Oxalis compacta	Anthidium sp.	19	0.01	0.05	0.03	0.04
0	0	0.16	1	t	1	Calandrinia affinis	Scaeva melanostoma	19	0.01	0.06	0.01	0.07

0	1	0.68	1	cv	1	Troch n.i. 2	Caiophora coronata	19	0.05	0.05	0.02	0.06
1	1	0.60	1	t	1	Syrphidae n.i.	Draba tenuis	19	0.01	0.03	0.04	0.03
0	0	0.30	1	t	1	Azorella madreporica	Sarcophagidae n.i.	19	0.01	0.04	0.01	0.04
0	0	0.21	1	t	1	Azorella madreporica	Scaeva melanostoma	19	0.01	0.04	0.01	0.07
0	0	0.32	1	cv	1	Azorella madreporica	Dasybasis sp. 1	19	0.01	0.04	0.01	0.03
0	0	0.26	1	t	1	Azorella madreporica	Habromyia lipoflava	19	0.01	0.04	0.01	0.05
0	0	0.33	1	t	1	Azorella madreporica	Diptera n.i.	19	0.01	0.04	0.02	0.06
0	0	0.41	1	t	1	Azorella madreporica	Hymenoptera n.i.	19	0.01	0.04	0.02	0.03
1	0	0.33	1	cv	1	Bombyllidae n.i.	Cerastium arvense	19	0.01	0.03	0.01	0.04
0	0	0.41	1	t	1	Troch n.i.	Tropaeolum polyphyllum	19	0.01	0.03	0.02	0.04
1	1	0.74	1	t	1	Viola montagnei	Yramea modesta	19	0.01	0.04	0.05	0.04
0	0	0.48	1	t	1	Oxalis sp.1	Diptera n.i.	19	0.03	0.05	0.02	0.06
0	0	0.39	1	cv	1	Pozoa	Diptera n.i.	19	0.02	0.05	0.02	0.06
1	1	0.74	1	t	1	Sarcophagidae sp.	Nassauvia lagascae	19	0.05	0.04	0.02	0.04
0	1	0.84	1	t	1	Yramea modesta	Junellia uniflora	19	0.05	0.04	0.03	0.04
1	1	0.79	1	t	1	Yramea modesta	Tropaeolum polyphyllum	19	0.05	0.04	0.02	0.04
1	1	0.72	1	cv	1	Yramea modesta	Chaetanthera apiculata	19	0.05	0.04	0.01	0.04
0	1	0.71	1	t	1	Yramea modesta	Viola atropurpurea	19	0.05	0.04	0.01	0.04
0	1	0.79	1	t	1	Yramea modesta	Nassauvia lagascae	19	0.05	0.04	0.02	0.04
1	1	0.72	1	t	1	Yramea modesta	Chaetanthera lycopodioides	19	0.05	0.04	0.01	0.04
0	1	0.79	1	cv	1	Yramea modesta	Nototriche compacta	19	0.05	0.04	0.02	0.04
1	1	0.72	1	t	1	Yramea modesta	Viola philippii	19	0.05	0.04	0.01	0.04
0	0	0.47	1	t	1	Phulia nymphula	Junellia uniflora	19	0.01	0.04	0.03	0.04
0	0	0.39	1	t	1	Phulia nymphula	Nassauvia lagascae	19	0.01	0.04	0.02	0.04
0	0	0.39	1	cv	1	Phulia nymphula	Nototriche compacta	19	0.01	0.04	0.02	0.04

0	1	0.57	1	t	1	Anthidium sp.	Tropaeolum polyphyllum	19	0.03	0.04	0.02	0.04
1	0	0.49	1	t	1	Anthidium sp.	Loasa caespitosa	19	0.03	0.04	0.01	0.03
0	1	0.51	1	t	1	Anthidium sp.	Caiophora coronata	19	0.03	0.04	0.02	0.06
1	0	0.49	1	cv	1	Anthidium sp.	Adesmia capitellata	19	0.03	0.04	0.01	0.03
0	1	0.74	1	t	1	Anthidium sp.	Adesmia glomerula	19	0.03	0.04	0.04	0.03
0	1	0.60	1	t	1	Anthidium sp.	Phacelia secunda	19	0.03	0.04	0.03	0.06
0	0	0.49	1	t	1	Junellia	Diptera n.i.	19	0.03	0.04	0.02	0.06
0	0	0.40	1	cv	1	Sarcophagidae n.i.	Nototriche compacta	19	0.01	0.04	0.02	0.04
0	1	0.75	1	t	1	Villa sp.	L. acaulis	19	0.01	0.05	0.06	0.05
0	0	0.48	1	t	1	Cerastium montioides	Diptera n.i.	19	0.03	0.05	0.02	0.06
0	1	0.97	1	t	1	Bombus dahlbomii	Nassauvia pungens	19	0.04	0.08	0.11	0.08
0	0	0.48	1	cv	1	Bombus dahlbomii	Caiophora coronata	19	0.04	0.08	0.02	0.06
0	1	0.58	1	t	1	Bombus dahlbomii	Phacelia secunda	19	0.04	0.08	0.03	0.06
0	0	0.29	1	t	1	Tropaeolum polyphyllum	Scaeva melanostoma	19	0.02	0.04	0.01	0.07
1	0	0.48	1	t	1	Nastanthus aglomeratus	Hymenoptera n.i.	19	0.01	0.02	0.02	0.03
0	1	0.75	1	cv	1	Vibrissiomyia sp.	L. acaulis	19	0.01	0.05	0.06	0.05
1	0	0.48	1	t	1	Nastanthus spathulatus	Hymenoptera n.i.	19	0.01	0.02	0.02	0.03
0	1	0.87	1	t	1	Nassauvia pungens	Scaeva melanostoma	19	0.11	0.08	0.01	0.07
0	1	0.93	1	t	1	Nassauvia pungens	Diptera n.i.	19	0.11	0.08	0.02	0.06
0	1	0.60	1	cv	1	Draba tenuis	Diptera n.i.	19	0.04	0.03	0.02	0.06
1	1	0.58	1	t	1	Draba tenuis	Tachinidae n.i.	19	0.04	0.03	0.01	0.03
1	1	0.58	1	t	1	Draba tenuis	Coleoptera n.i.	19	0.04	0.03	0.01	0.03
1	1	0.68	1	t	1	Draba tenuis	Hymenoptera n.i.	19	0.04	0.03	0.02	0.03
0	1	0.72	1	cv	1	L. acaulis	Habromyia lipoflava	19	0.06	0.05	0.01	0.05
0	1	0.79	1	t	1	L. acaulis	Diptera n.i.	19	0.06	0.05	0.02	0.06
0	0	0.24	1	t	1	Scaeva melanostoma	Leuceria salina	19	0.01	0.07	0.02	0.05

0	0	0.31	1	t	1	Scaeva melanostoma	Phacelia secunda	19	0.01	0.07	0.03	0.06
0	0	0.16	1	cv	1	Scaeva melanostoma	Senecio francisci	19	0.01	0.07	0.01	0.06
0	0	0.29	1	t	1	Calandrinia caespitosa	Diptera n.i.	19	0.01	0.05	0.02	0.06
0	0	0.39	1	t	1	Calandrinia occulta	Diptera n.i.	19	0.02	0.05	0.02	0.06
0	1	0.62	1	t	1	Cardamine nivalis	Diptera n.i.	19	0.04	0.05	0.02	0.06
0	0	0.31	1	cv	1	Diptera n.i.	Viola atropurpurea	19	0.02	0.06	0.01	0.04
0	0	0.40	1	t	1	Diptera n.i.	Nassauvia lagascae	19	0.02	0.06	0.02	0.04
0	1	0.56	1	t	1	Diptera n.i.	Ranunculus peduncularis	19	0.02	0.06	0.04	0.05
0	0	0.41	1	t	1	Diptera n.i.	Nototriche compacta	19	0.02	0.06	0.02	0.04
0	1	0.56	1	cv	1	Diptera n.i.	Calandrinia picta	19	0.02	0.06	0.04	0.05
0	0	0.31	1	t	1	Diptera n.i.	Cerastium arvense	19	0.02	0.06	0.01	0.04
0	0	0.31	1	t	1	Adesmia capitellata	Stenanthidiu m espinosai	19	0.01	0.03	0.01	0.05
0	0	0.40	1	t	1	Stenanthidium espinosai	Phacelia secunda	19	0.01	0.05	0.03	0.06
0	1	0.58	0	cv	1	Chaetanthera flabellata	Ranunculus peduncularis	19	0.01	0.03	0.04	0.05
0	0	0.28	0	t	1	Troch n.i.	Habromyia lipoflava	19	0.01	0.03	0.01	0.05
0	1	0.77	0	t	1	Acaena pinnatifida	Adesmia glomerula	19	0.04	0.05	0.04	0.03
1	0	0.36	0	t	1	Faunula leucoglene	Dasybasis sp. 1	19	0.01	0.03	0.01	0.03
0	1	0.66	0	cv	1	Yramea modesta	Senecio francisci	19	0.05	0.04	0.01	0.06
0	1	0.68	0	t	1	Megachile semirufa	Bombyllidae n.i.	19	0.05	0.05	0.01	0.03
0	0	0.32	0	t	1	Chaetanthera apiculata	Tachinidae n.i.	19	0.01	0.04	0.01	0.03
0	1	0.66	0	t	1	Troch n.i. 2	Tachinidae n.i.	19	0.05	0.05	0.01	0.03
0	1	0.63	0	cv	1	Nastanthus spathulatus	Draba tenuis	19	0.01	0.02	0.04	0.03
0	1	0.94	0	t	1	Chaetanthera apiculata	Nassauvia pungens	19	0.01	0.04	0.11	0.08
0	0	0.23	0	t	1	Nassauvia pinnigera	Calandrinia caespitosa	19	0.01	0.05	0.01	0.05
1	0	0.35	0	t	1	Bombyllidae n.i.	Tachinidae n.i.	19	0.01	0.03	0.01	0.03

0	0	0.32	0	cv	1	Troch n.i.	Chaetanthera apiculata	19	0.01	0.03	0.01	0.04
0	0	0.23	0	t	1	Calandrinia affinis	Cerastium arvense	19	0.01	0.06	0.01	0.04
0	1	0.63	0	t	1	Chaetanthera flabellata	Adesmia glomerula	19	0.01	0.03	0.04	0.03
0	0	0.31	0	t	1	Pozoa coriacea	Vibrissiomyia sp.	19	0.02	0.05	0.01	0.05
0	1	0.69	0	cv	1	Megachile semirufa	Leuceria salina	19	0.05	0.05	0.02	0.05
0	1	0.80	0	t	1	L. acaulis	Leuceria salina	19	0.06	0.05	0.02	0.05
0	0	0.43	0	t	1	Cerastium montioides	Cerastium arvense	19	0.03	0.05	0.01	0.04
0	0	0.23	0	t	1	Nassauvia pinnigera	Habromyia lipoflava	19	0.01	0.05	0.01	0.05
0	0	0.49	0	cv	1	Acaena pinnatifida	Tatochila mercedis	19	0.04	0.05	0.01	0.04
1	1	0.76	0	t	1	Pseudolucia chilensis	Adesmia glomerula	19	0.03	0.03	0.04	0.03
0	0	0.45	0	t	1	Draba tenuis	Scaeva melanostoma	19	0.04	0.03	0.01	0.07
0	1	0.80	0	t	1	Erigeron andicola	Sarcophagidae sp.	19	0.03	0.05	0.05	0.04
0	0	0.25	0	cv	1	Calandrinia affinis	Troch n.i.	19	0.01	0.06	0.01	0.03
0	1	0.95	0	t	1	Faunula leucoglene	Nassauvia pungens	19	0.01	0.03	0.11	0.08
0	1	0.58	0	t	1	Draba gilliesii	Cardamine nivalis	19	0.01	0.05	0.04	0.05
0	0	0.34	0	t	1	Diptera n.i.	Adesmia capitellata	19	0.02	0.06	0.01	0.03
0	0	0.48	0	cv	1	Anthidium sp.	Sarcophagidae n.i.	19	0.03	0.04	0.01	0.04
1	0	0.35	0	t	1	Chaetanthera apiculata	Nastanthus aglomeratus	19	0.01	0.04	0.01	0.02
0	1	0.67	0	t	1	Andinus venustus	Cynthia terpsichore	19	0.05	0.05	0.01	0.04
0	0	0.40	0	t	1	Valeriana radicalis	Leuceria salina	19	0.02	0.05	0.02	0.05
0	0	0.34	0	cv	1	Chaetanthera flabellata	Megachile sp. 5	19	0.01	0.03	0.01	0.04
0	0	0.25	0	t	1	Megachile sp.	Villa sp.	19	0.01	0.04	0.01	0.05
0	0	0.40	0	t	1	Draba gilliesii	Hymenoptera n.i.	19	0.01	0.05	0.02	0.03
0	1	0.74	0	t	1	Andinus venustus	Pozoa coriacea	19	0.05	0.05	0.02	0.05
0	1	0.65	0	cv	1	Sarcophagidae sp.	Cerastium arvense	19	0.05	0.04	0.01	0.04

0	1	0.83	0	t	1	Acaena pinnatifida	Troch n.i. 2	19	0.04	0.05	0.05	0.05
0	0	0.31	0	t	1	Nastanthus aglomeratus	Vibrissiomyia sp.	19	0.01	0.02	0.01	0.05
0	0	0.38	0	t	1	Chaetanthera flabellata	Caiophora coronata	19	0.01	0.03	0.02	0.06
0	1	0.81	0	cv	1	Draba gilliesii	Hypsochila wagenknecti	19	0.01	0.05	0.07	0.07
1	1	0.91	0	t	1	Andinus venustus	Megachile semirufa	19	0.05	0.05	0.05	0.05
0	0	0.47	0	t	1	Oreopolus glacialis	Chaetanthera apiculata	19	0.03	0.04	0.01	0.04
0	0	0.32	0	t	1	Tatochila mercedis	Caiophora coronata	19	0.01	0.04	0.02	0.06
0	0	0.26	0	cv	1	Nassauvia pinnigera	Viola philippii	19	0.01	0.05	0.01	0.04
0	1	0.62	0	t	1	Chaetanthera flabellata	Draba tenuis	19	0.01	0.03	0.04	0.03
0	0	0.18	0	t	1	Vibrissiomyia sp.	Scaeva melanostoma	19	0.01	0.05	0.01	0.07
0	1	0.60	0	t	1	Nastanthus aglomeratus	Calandrinia picta	19	0.01	0.02	0.04	0.05
0	1	0.56	0	cv	1	Pozoa coriacea	Pseudolucia chilensis	19	0.02	0.05	0.03	0.03
0	0	0.25	0	t	1	Cynthia terpsichore	Habromyia lipoflava	19	0.01	0.04	0.01	0.05
0	0	0.30	0	t	1	Bombyllidae n.i.	Calandrinia caespitosa	19	0.01	0.03	0.01	0.05
0	0	0.44	0	t	1	Ranunculus peduncularis	Senecio francisci	19	0.04	0.05	0.01	0.06
0	0	0.44	0	cv	1	Nastanthus spathulatus	Nassauvia lagascae	19	0.01	0.02	0.02	0.04
0	0	0.28	0	t	1	Cynthia terpsichore	Cerastium arvense	19	0.01	0.04	0.01	0.04
0	1	0.95	0	t	1	Nastanthus aglomeratus	Nassauvia pungens	19	0.01	0.02	0.11	0.08
0	1	0.96	0	t	1	Yramea modesta	L. acaulis	19	0.05	0.04	0.06	0.05
0	0	0.45	0	cv	1	Calandrinia affinis	Ranunculus peduncularis	19	0.01	0.06	0.04	0.05
0	1	0.94	0	t	1	Calandrinia sericea	Nassauvia pungens	19	0.01	0.04	0.11	0.08
0	1	0.77	0	t	1	Hypsochila wagenknecti	Stenanthidium espinosai	19	0.07	0.07	0.01	0.05
0	0	0.41	0	t	1	Loasa caespitosa	Nassauvia lagascae	19	0.01	0.03	0.02	0.04
1	0	0.32	0	cv	1	Syrphidae n.i.	Phulia nymphula	19	0.01	0.03	0.01	0.04

0	0	0.33	0	t	1	Dasybasis sp. 1	Chaetanthera lycopodioides	19	0.01	0.03	0.01	0.04
0	0	0.43	0	t	1	Nassauvia pinnigera	Bombus dahlbomii	19	0.01	0.05	0.04	0.08
0	0	0.30	0	t	1	Calandrinia caespitosa	Leuceria salina	19	0.01	0.05	0.02	0.05
0	0	0.41	0	cv	1	Pozoa coriacea	Nastanthus aglomeratus	19	0.02	0.05	0.01	0.02
0	0	0.34	0	t	1	Valeriana radicalis	Tatochila mercedis	19	0.02	0.05	0.01	0.04
0	0	0.32	0	t	1	Cynthia terpsichore	Diptera n.i.	19	0.01	0.04	0.02	0.06
0	1	0.51	0	t	1	Bombus dahlbomii	Calandrinia occulta	19	0.04	0.08	0.02	0.05
0	1	0.67	0	cv	1	Andinus venustus	Cerastium arvense	19	0.05	0.05	0.01	0.04
1	0	0.49	0	t	1	Anthidium sp.	Coleoptera n.i.	19	0.03	0.04	0.01	0.03
0	0	0.27	0	t	1	Vibrissiomyia sp.	Tachinidae n.i.	19	0.01	0.05	0.01	0.03
0	0	0.35	0	t	1	Calandrinia occulta	Chaetanthera lycopodioides	19	0.02	0.05	0.01	0.04
0	1	0.76	0	cv	1	L. acaulis	Chaetanthera lycopodioides	19	0.06	0.05	0.01	0.04
1	0	0.38	0	t	1	Nastanthus spathulatus	Tachinidae n.i.	19	0.01	0.02	0.01	0.03
0	1	0.86	0	t	1	Megachile semirufa	Adesmia glomerula	19	0.05	0.05	0.04	0.03
0	0	0.37	0	t	1	Troch n.i.	Nastanthus aglomeratus	19	0.01	0.03	0.01	0.02
0	0	0.40	0	cv	1	Stenanthidium espinosai	Hymenoptera n.i.	19	0.01	0.05	0.02	0.03
0	0	0.26	0	t	1	Megachile sp. 5	Habromyia lipoflava	19	0.01	0.04	0.01	0.05
0	0	0.39	0	t	1	Calandrinia occulta	Caiophora coronata	19	0.02	0.05	0.02	0.06
0	1	0.68	0	t	1	Yramea modesta	Villa sp.	19	0.05	0.04	0.01	0.05
0	0	0.40	0	cv	1	Viola atropurpurea	Hymenoptera n.i.	19	0.01	0.04	0.02	0.03
0	0	0.37	0	t	1	Villa sp.	Phacelia secunda	19	0.01	0.05	0.03	0.06
0	0	0.48	0	t	1	Scaeva melanostoma	Cardamine nivalis	19	0.01	0.07	0.04	0.05

0	0	0.46	0	t	1	Bombyllidae n.i.	Phacelia secunda	19	0.01	0.03	0.03	0.06
0	0	0.24	0	cv	1	Calandrinia sericea	Calandrinia affinis	19	0.01	0.04	0.01	0.06
0	1	0.79	0	t	1	Sarcophagidae sp.	Cerastium montioides	19	0.05	0.04	0.03	0.05
0	0	0.50	0	t	1	Erigeron andicola	Nastanthus spathulatus	19	0.03	0.05	0.01	0.02
0	0	0.42	0	t	1	Erigeron andicola	Stenanthidium espinosai	19	0.03	0.05	0.01	0.05
0	1	0.84	0	cv	1	Tropaeolum polyphyllum	L. acaulis	19	0.02	0.04	0.06	0.05
0	1	0.70	0	t	1	Andinus venustus	Troch n.i.	19	0.05	0.05	0.01	0.03
0	0	0.31	0	t	1	Tatochila mercedis	Dasybasis sp. 1	19	0.01	0.04	0.01	0.03
0	0	0.08	1	t	1	Syritta rectus	Cornus canadensis	20	0.01	0.07	0.01	0.09
0	0	0.08	1	cv	1	Temnostoma barberi	Cornus canadensis	20	0.01	0.07	0.01	0.09
0	0	0.22	1	t	1	Cypripedium acaule	Eusphalerum sp.	20	0.01	0.04	0.01	0.05
0	0	0.22	1	t	1	Cypripedium acaule	Bombus vagans	20	0.01	0.04	0.01	0.06
0	0	0.08	1	t	1	Blera confusa	Cornus canadensis	20	0.01	0.07	0.01	0.09
0	0	0.08	1	cv	1	Tychius stephensi	Cornus canadensis	20	0.01	0.07	0.01	0.09
0	0	0.34	1	t	1	Bombus terricola	Clintonia borealis	20	0.02	0.04	0.01	0.04
0	0	0.21	1	t	1	Bombus terricola	Cornus canadensis	20	0.02	0.04	0.01	0.09
0	0	0.34	1	t	1	Bombus terricola	Aralia nudicaulis	20	0.02	0.04	0.01	0.04
1	0	0.36	1	cv	1	Bombus terricola	Chimaphila umbellata	20	0.02	0.04	0.01	0.03
1	1	0.97	1	t	1	Linnaea borealis	Dialictus sp 2.	20	0.08	0.05	0.06	0.05
0	1	0.75	1	t	1	Linnaea borealis	Zeraea americana	20	0.08	0.05	0.01	0.06
1	1	0.93	1	t	1	Linnaea borealis	Eupyses vestris	20	0.08	0.05	0.04	0.06
0	1	0.76	1	cv	1	Linnaea borealis	Sphaerophoria sp.	20	0.08	0.05	0.01	0.06
0	1	0.76	1	t	1	Linnaea borealis	Eusphalerum sp.	20	0.08	0.05	0.01	0.05
0	1	0.80	1	t	1	Linnaea	Osmia pumila	20	0.08	0.05	0.01	0.06
0	1	0.80	1	t	1	Linnaea borealis	Hylaeus ellipticus	20	0.08	0.05	0.01	0.06
0	1	0.76	1	cv	1	Linnaea borealis	Bombus vagans	20	0.08	0.05	0.01	0.06

0	1	0.75	1	t	1	Linnaea borealis	Evylaeus quebecensis	20	0.08	0.05	0.01	0.06
0	1	0.75	1	t	1	Linnaea borealis	Osmia proxima	20	0.08	0.05	0.01	0.06
0	1	0.75	1	t	1	Linnaea borealis	Pyrophaena rosarum	20	0.08	0.05	0.01	0.06
0	1	0.75	1	cv	1	Linnaea borealis	Eclimus harrisi	20	0.08	0.05	0.01	0.06
1	1	0.88	1	t	1	Linnaea borealis	Fannia ungulata	20	0.08	0.05	0.03	0.06
0	1	0.76	1	t	1	Linnaea borealis	Carposcalis confusus	20	0.08	0.05	0.01	0.06
0	1	0.76	1	t	1	Linnaea borealis	Dialictus cressoni	20	0.08	0.05	0.01	0.06
0	1	0.88	1	cv	1	Linnaea	Crabro sp.	20	0.08	0.05	0.03	0.06
0	1	0.76	1	t	1	Linnaea borealis	Sphaerophori a bifurcata	20	0.08	0.05	0.01	0.06
0	1	0.80	1	t	1	Linnaea borealis	Hylaeus stevensi	20	0.08	0.05	0.01	0.06
0	1	0.76	1	t	1	Linnaea borealis	Melagyna lasiophthalm a	20	0.08	0.05	0.01	0.06
0	1	0.75	1	cv	1	Linnaea borealis	Passaloecus sp.	20	0.08	0.05	0.01	0.06
0	1	0.79	1	t	1	Linnaea borealis	Phaonia serva	20	0.08	0.05	0.01	0.06
0	0	0.08	1	t	1	Ancistrocerus sp.	Cornus canadensis	20	0.01	0.07	0.01	0.09
0	0	0.08	1	t	1	Dalopius sp.	Cornus canadensis	20	0.01	0.07	0.01	0.09
0	0	0.32	1	cv	1	Evylaeus divergens	Cornus canadensis	20	0.04	0.05	0.01	0.09
0	1	0.63	1	t	1	Evylaeus divergens	Maianthemu m canadense	20	0.04	0.05	0.02	0.05
0	0	0.08	1	t	1	Andrena sigmundi	Cornus canadensis	20	0.01	0.07	0.01	0.09
0	0	0.13	1	t	1	Tropidia quadrata	Cornus canadensis	20	0.01	0.05	0.01	0.09
0	0	0.35	1	cv	1	Tropidia quadrata	Maianthemu m canadense	20	0.01	0.05	0.02	0.05
0	0	0.08	1	t	1	Pieris napi	Cornus canadensis	20	0.01	0.07	0.01	0.09
0	0	0.27	1	t	1	Parasyrphus relictus	Clintonia borealis	20	0.02	0.05	0.01	0.04
0	0	0.16	1	t	1	Parasyrphus relictus	Cornus canadensis	20	0.02	0.05	0.01	0.09
0	0	0.41	1	cv	1	Parasyrphus relictus	Maianthemu m canadense	20	0.02	0.05	0.02	0.05
0	0	0.24	1	t	1	Rhopalum sp.	Clintonia borealis	20	0.01	0.03	0.01	0.04

0	0	0.22	1	t	1	Pidonia ruficolis	Clintonia borealis	20	0.01	0.05	0.01	0.04
0	0	0.12	1	t	1	Pidonia ruficolis	Cornus canadensis	20	0.01	0.05	0.01	0.09
0	1	0.67	1	cv	1	Dialictus sp 2.	Clintonia borealis	20	0.06	0.05	0.01	0.04
0	1	0.51	1	t	1	Dialictus sp 2.	Cornus canadensis	20	0.06	0.05	0.01	0.09
1	1	0.70	1	t	1	Dialictus sp 2.	Trillium undulatum	20	0.06	0.05	0.01	0.05
0	1	0.67	1	t	1	Dialictus sp 2.	Aralia nudicaulis	20	0.06	0.05	0.01	0.04
0	1	0.65	1	cv	1	Dialictus sp 2.	Pyrola secunda	20	0.06	0.05	0.01	0.05
1	1	0.79	1	t	1	Dialictus sp 2.	Maianthemu m canadense	20	0.06	0.05	0.02	0.05
1	1	0.84	1	t	1	Dialictus sp 2.	Oxalis montana	20	0.06	0.05	0.03	0.05
0	0	0.23	1	t	1	Clintonia borealis	Osmia lignaria	20	0.01	0.04	0.01	0.03
0	0	0.29	1	cv	1	Clintonia borealis	Poanes hobomok	20	0.01	0.04	0.01	0.03
0	0	0.18	1	t	1	Clintonia borealis	Sphaerophori a sp.	20	0.01	0.04	0.01	0.06
0	0	0.18	1	t	1	Clintonia borealis	Bombus vagans	20	0.01	0.04	0.01	0.06
0	0	0.17	1	t	1	Clintonia borealis	Bombus ternarius	20	0.01	0.04	0.01	0.06
0	0	0.23	1	cv	1	Clintonia borealis	Andrena wilkella	20	0.01	0.04	0.01	0.03
0	0	0.18	1	t	1	Clintonia borealis	Carposcalis confusus	20	0.01	0.04	0.01	0.06
0	0	0.18	1	t	1	Clintonia borealis	Dialictus cressoni	20	0.01	0.04	0.01	0.06
0	0	0.28	1	t	1	Clintonia borealis	Dialictus imitatus	20	0.01	0.04	0.01	0.03
0	0	0.22	1	cv	1	Clintonia borealis	Sarcophaga nearctica	20	0.01	0.04	0.01	0.05
0	0	0.08	1	t	1	Chalcosyrphus memorum	Cornus canadensis	20	0.01	0.07	0.01	0.09
0	1	0.89	1	t	1	Nomada sayi	Cornus canadensis	20	0.14	0.14	0.01	0.09
0	0	0.08	1	t	1	Chalcosyrphus nigra	Cornus canadensis	20	0.01	0.07	0.01	0.09
0	0	0.08	1	cv	1	Cornus canadensis	Blera nigripes	20	0.01	0.09	0.01	0.07
0	0	0.08	1	t	1	Cornus canadensis	Evodinus monticola	20	0.01	0.09	0.01	0.07
0	0	0.09	1	t	1	Cornus canadensis	Zeraea americana	20	0.01	0.09	0.01	0.06

0	0	0.11	1	t	1	Cornus canadensis	Temnostoma balyras	20	0.01	0.09	0.01	0.06
0	0	0.12	1	cv	1	Cornus canadensis	Andrena miranda	20	0.01	0.09	0.01	0.05
0	0	0.09	1	t	1	Cornus canadensis	Bombylius major	20	0.01	0.09	0.01	0.05
0	0	0.08	1	t	1	Cornus canadensis	Lygus refidorsus	20	0.01	0.09	0.01	0.07
0	0	0.09	1	t	1	Cornus canadensis	Xylota hinei	20	0.01	0.09	0.01	0.05
0	0	0.11	1	cv	1	Cornus canadensis	Orsodacne atra	20	0.01	0.09	0.01	0.06
0	0	0.14	1	t	1	Cornus canadensis	Anthaxia expansa	20	0.01	0.09	0.02	0.05
0	0	0.22	1	t	1	Cornus canadensis	Chalcosyrphus curvarius	20	0.01	0.09	0.03	0.05
0	0	0.09	1	t	1	Cornus canadensis	Mallota posticata	20	0.01	0.09	0.01	0.06
0	0	0.15	1	cv	1	Cornus canadensis	Judolia montivagans	20	0.01	0.09	0.02	0.05
0	0	0.08	1	t	1	Cornus canadensis	Sphaerophoria longipilosa	20	0.01	0.09	0.01	0.07
0	0	0.11	1	t	1	Cornus canadensis	Andrena thaspii	20	0.01	0.09	0.01	0.06
0	0	0.12	1	t	1	Cornus canadensis	Andrena w-scripta	20	0.01	0.09	0.01	0.05
0	0	0.08	1	cv	1	Cornus canadensis	Chalcosyrphus inarmatus	20	0.01	0.09	0.01	0.07
0	0	0.08	1	t	1	Cornus canadensis	Eudasyphora cyanicolor	20	0.01	0.09	0.01	0.07
0	0	0.08	1	t	1	Cornus canadensis	Xylota bigelowi	20	0.01	0.09	0.01	0.07
0	0	0.13	1	t	1	Cornus canadensis	Paramyia nitens	20	0.01	0.09	0.01	0.05
0	0	0.12	1	cv	1	Cornus canadensis	Bombus perplexus	20	0.01	0.09	0.01	0.05
0	0	0.09	1	t	1	Cornus canadensis	Bombus vagans	20	0.01	0.09	0.01	0.06
0	0	0.09	1	t	1	Cornus canadensis	Bombus ternarius	20	0.01	0.09	0.01	0.06
0	0	0.08	1	t	1	Cornus canadensis	Acmaeopsoides rufula	20	0.01	0.09	0.01	0.07
0	0	0.08	1	cv	1	Cornus canadensis	Xylota sp.	20	0.01	0.09	0.01	0.07
0	0	0.12	1	t	1	Cornus canadensis	Orthoneura pulchella	20	0.01	0.09	0.01	0.05
0	0	0.09	1	t	1	Cornus canadensis	Andrena nivalis	20	0.01	0.09	0.01	0.05
0	0	0.08	1	t	1	Cornus canadensis	Psithyrus sp.	20	0.01	0.09	0.01	0.07

0	0	0.18	1	cv	1	Cornus canadensis	Andrena melanochroa	20	0.01	0.09	0.02	0.05
0	0	0.09	1	t	1	Cornus canadensis	Evylaeus quebecensis	20	0.01	0.09	0.01	0.06
0	0	0.09	1	t	1	Cornus canadensis	Osmia proxima	20	0.01	0.09	0.01	0.06
0	0	0.18	1	t	1	Cornus canadensis	Andrena rufosignata	20	0.01	0.09	0.02	0.05
0	0	0.08	1	cv	1	Cornus canadensis	Hylaeus basalis	20	0.01	0.09	0.01	0.07
0	0	0.15	1	t	1	Cornus canadensis	Anthonomus sp.	20	0.01	0.09	0.02	0.05
0	0	0.11	1	t	1	Cornus canadensis	Temnostoma alternans	20	0.01	0.09	0.01	0.06
0	0	0.08	1	t	1	Cornus canadensis	Syritta pipiens	20	0.01	0.09	0.01	0.07
0	0	0.09	1	cv	1	Cornus canadensis	Eclimus harrisi	20	0.01	0.09	0.01	0.06
0	0	0.08	1	t	1	Cornus canadensis	Cheilosia sialia	20	0.01	0.09	0.01	0.07
0	0	0.19	1	t	1	Cornus canadensis	Crabro sp.	20	0.01	0.09	0.03	0.06
0	0	0.09	1	t	1	Cornus canadensis	Sphaerophoria bifurcata	20	0.01	0.09	0.01	0.06
0	0	0.11	1	cv	1	Cornus canadensis	Celastrina argiolus	20	0.01	0.09	0.01	0.06
0	0	0.08	1	t	1	Cornus canadensis	Blera badia	20	0.01	0.09	0.01	0.07
0	0	0.08	1	t	1	Cornus canadensis	Halictus confusus	20	0.01	0.09	0.01	0.07
0	0	0.08	1	t	1	Cornus canadensis	Sphecodes sp.	20	0.01	0.09	0.01	0.07
0	0	0.11	1	cv	1	Cornus canadensis	Phaonia serva	20	0.01	0.09	0.01	0.06
0	0	0.08	1	t	1	Cornus canadensis	Cantharis sp.	20	0.01	0.09	0.01	0.07
0	0	0.12	1	t	1	Cornus canadensis	Sarcophaga nearctica	20	0.01	0.09	0.01	0.05
0	0	0.15	1	t	1	Cornus canadensis	Eusphalerum phothos	20	0.01	0.09	0.02	0.05
0	0	0.39	1	cv	1	Cheilosia pallipes	Oxalis montana	20	0.01	0.05	0.03	0.05
0	0	0.24	1	t	1	Andrena wheeleri	Aralia nudicaulis	20	0.01	0.03	0.01	0.04
0	0	0.35	1	t	1	Andrena miranda	Maianthemum canadense	20	0.01	0.05	0.02	0.05
0	0	0.29	1	t	1	Bombylius major	Maianthemum canadense	20	0.01	0.05	0.02	0.05
0	0	0.32	1	cv	1	Agiotes stabilis	Maianthemum canadense	20	0.01	0.04	0.02	0.05

0	0	0.30	1	t	1	Cheilosia tristis	Aralia nudicaulis	20	0.01	0.03	0.01	0.04
0	0	0.29	1	t	1	Xylota hinei	Maianthemum canadense	20	0.01	0.05	0.02	0.05
0	0	0.29	1	t	1	Anthaxia expansa	Trillium undulatum	20	0.02	0.05	0.01	0.05
0	0	0.28	1	cv	1	Sphaerophoria sp.	Maianthemum canadense	20	0.01	0.06	0.02	0.05
0	0	0.20	1	t	1	Eusphalerum convexum	Aralia nudicaulis	20	0.01	0.05	0.01	0.04
0	0	0.39	1	t	1	Eusphalerum convexum	Oxalis montana	20	0.01	0.05	0.03	0.05
0	0	0.18	1	t	1	Eusphalerum sp.	Aralia nudicaulis	20	0.01	0.05	0.01	0.04
0	0	0.29	1	cv	1	Eusphalerum sp.	Maianthemum canadense	20	0.01	0.05	0.02	0.05
0	0	0.36	1	t	1	Eusphalerum sp.	Oxalis montana	20	0.01	0.05	0.03	0.05
0	0	0.17	1	t	1	Mallota posticata	Aralia nudicaulis	20	0.01	0.06	0.01	0.04
1	0	0.40	1	t	1	Trientalis borealis	Carposcalis obscura	20	0.01	0.03	0.01	0.02
0	0	0.25	1	cv	1	Trientalis borealis	Carposcalis confusus	20	0.01	0.03	0.01	0.06
0	0	0.24	1	t	1	Trientalis borealis	Sphaerophoria bifurcata	20	0.01	0.03	0.01	0.06
0	0	0.22	1	t	1	Andrena w-scripta	Aralia nudicaulis	20	0.01	0.05	0.01	0.04
0	0	0.32	1	t	1	Bombylius pigmaeus	Maianthemum canadense	20	0.01	0.04	0.02	0.05
0	0	0.25	1	cv	1	Paramyia nitens	Chimaphila umbellata	20	0.01	0.05	0.01	0.03
0	0	0.22	1	t	1	Bombus perplexus	Aralia nudicaulis	20	0.01	0.05	0.01	0.04
0	0	0.18	1	t	1	Bombus vagans	Aralia nudicaulis	20	0.01	0.06	0.01	0.04
0	0	0.16	1	t	1	Bombus vagans	Pyrola secunda	20	0.01	0.06	0.01	0.05
0	0	0.19	1	cv	1	Bombus vagans	Chimaphila umbellata	20	0.01	0.06	0.01	0.03
0	0	0.28	1	t	1	Bombus vagans	Maianthemum canadense	20	0.01	0.06	0.02	0.05
0	0	0.32	1	t	1	Pegohylemyia fugax	Maianthemum canadense	20	0.01	0.04	0.02	0.05
0	0	0.24	1	t	1	Cateres pennatus	Aralia nudicaulis	20	0.01	0.03	0.01	0.04
0	0	0.50	1	cv	1	Phalaenophana pyramusalis	Maianthemum canadense	20	0.02	0.04	0.02	0.05
0	0	0.20	1	t	1	Trillium undulatum	Carposcalis confusus	20	0.01	0.05	0.01	0.06

0	0	0.20	1	t	1	Trillium undulatum	Sphaerophoria bifurcata	20	0.01	0.05	0.01	0.06
0	0	0.35	1	t	1	Orthoneura pulchella	Maianthemum canadense	20	0.01	0.05	0.02	0.05
0	0	0.29	1	cv	1	Andrena nivalis	Maianthemum canadense	20	0.01	0.05	0.02	0.05
0	0	0.17	1	t	1	Evylaeus quebecensis	Aralia nudicaulis	20	0.01	0.06	0.01	0.04
0	0	0.27	1	t	1	Evylaeus quebecensis	Maianthemum canadense	20	0.01	0.06	0.02	0.05
0	0	0.19	1	t	1	Vespula arenaria	Pyrola secunda	20	0.01	0.04	0.01	0.05
1	1	0.83	1	cv	1	Cheilosia slossonae	Maianthemum canadense	20	0.07	0.05	0.02	0.05
0	0	0.24	1	t	1	Aralia nudicaulis	Trachysida aspera	20	0.01	0.04	0.01	0.03
0	0	0.21	1	t	1	Aralia nudicaulis	Halictus rubicundus	20	0.01	0.04	0.01	0.04
0	0	0.18	1	t	1	Aralia nudicaulis	Dialictus cressoni	20	0.01	0.04	0.01	0.06
0	0	0.28	1	cv	1	Aralia nudicaulis	Dialictus imitatus	20	0.01	0.04	0.01	0.03
0	0	0.27	1	t	1	Aralia nudicaulis	Eusphalerum phothos	20	0.01	0.04	0.02	0.05
0	0	0.39	1	t	1	Melanostoma sp.	Oxalis montana	20	0.01	0.05	0.03	0.05
0	0	0.19	1	t	1	Pyrola secunda	Hoplitis producta	20	0.01	0.05	0.01	0.04
0	0	0.33	1	cv	1	Halictus rubicundus	Maianthemum canadense	20	0.01	0.04	0.02	0.05
0	0	0.32	1	t	1	Thamnotettix confinis	Maianthemum canadense	20	0.01	0.04	0.02	0.05
0	0	0.28	1	t	1	Carposcalis confusus	Maianthemum canadense	20	0.01	0.06	0.02	0.05
0	0	0.32	1	t	1	Nomada cuneata	Maianthemum canadense	20	0.01	0.04	0.02	0.05
0	1	0.74	1	cv	1	Medeola virginiana	Ephemerella sp.	20	0.09	0.08	0.01	0.08
0	0	0.28	1	t	1	Maianthemum canadense	Dialictus cressoni	20	0.02	0.05	0.01	0.06
0	0	0.27	1	t	1	Maianthemum canadense	Sphaerophoria bifurcata	20	0.02	0.05	0.01	0.06
0	0	0.27	1	t	1	Maianthemum canadense	Melagyna lasiophthalma	20	0.02	0.05	0.01	0.06
0	0	0.31	1	cv	1	Maianthemum canadense	Eremomyioides setosa	20	0.02	0.05	0.01	0.04
0	0	0.48	1	t	1	Oxalis montana	Eusphalerum phothos	20	0.03	0.05	0.02	0.05
0	0	0.40	0	t	1	Temnostoma balyras	Oxalis montana	20	0.01	0.06	0.03	0.05

0	0	0.27	0	t	1	Andrena wheeleri	Andrena wilkella	20	0.01	0.03	0.01	0.03
0	0	0.42	0	cv	1	Pidonia ruficolis	Oxalis montana	20	0.01	0.05	0.03	0.05
0	0	0.16	0	t	1	Tropidia quadrata	Sphaerophoria longipilosa	20	0.01	0.05	0.01	0.07
0	0	0.16	0	t	1	Bombus perplexus	Psithyrus sp.	20	0.01	0.05	0.01	0.07
0	0	0.14	0	t	1	Bombylius major	Ephemerella sp.	20	0.01	0.05	0.01	0.08
0	1	0.51	0	cv	1	Bombus terricola	Phalaenophana pyramusalis	20	0.02	0.04	0.02	0.04
0	0	0.18	0	t	1	Bombus ternarius	Sarcophaga nearctica	20	0.01	0.06	0.01	0.05
0	0	0.14	0	t	1	Pieris napi	Agiotes stabilis	20	0.01	0.07	0.01	0.04
0	1	0.56	0	t	1	Eupyses vestris	Carposcalis obscura	20	0.04	0.06	0.01	0.02
0	0	0.30	0	cv	1	Bombus terricola	Bombylius major	20	0.02	0.04	0.01	0.05
0	0	0.16	0	t	1	Zeraea americana	Eremomyioides setosa	20	0.01	0.06	0.01	0.04
0	0	0.20	0	t	1	Vespula arenaria	Thamnotettix confinis	20	0.01	0.04	0.01	0.04
0	0	0.23	0	t	1	Psithyrus sp.	Andrena melanochroa	20	0.01	0.07	0.02	0.05
0	0	0.18	0	cv	1	Andrena w-scripta	Passaloecus sp.	20	0.01	0.05	0.01	0.06
0	0	0.27	0	t	1	Chalcosyrphus curvarius	Sphecodes sp.	20	0.03	0.05	0.01	0.07
0	0	0.30	0	t	1	Clintonia borealis	Cheilosia tristis	20	0.01	0.04	0.01	0.03
0	0	0.23	0	t	1	Pieris napi	Andrena rufosignata	20	0.01	0.07	0.02	0.05
0	0	0.20	0	cv	1	Clintonia borealis	Vespula arenaria	20	0.01	0.04	0.01	0.04
0	0	0.20	0	t	1	Trillium undulatum	Andrena nivalis	20	0.01	0.05	0.01	0.05
0	0	0.20	0	t	1	Trachysida aspera	Melagyna lasiophthalma	20	0.01	0.03	0.01	0.06
0	0	0.22	0	t	1	Andrena miranda	Bombylius pigmaeus	20	0.01	0.05	0.01	0.04
0	0	0.20	0	cv	1	Rhopalum sp.	Bombus ternarius	20	0.01	0.03	0.01	0.06
0	0	0.33	0	t	1	Andrena melanochroa	Chimaphila umbellata	20	0.02	0.05	0.01	0.03
0	1	0.94	0	t	1	Nomada sayi	Hylaeus ellipticus	20	0.14	0.14	0.01	0.06

0	0	0.28	0	t	1	Cheilosia tristis	Pyrola secunda	20	0.01	0.03	0.01	0.05
0	0	0.14	0	cv	1	Sphaerophoria longipilosa	Eremomyioides setosa	20	0.01	0.07	0.01	0.04
0	0	0.29	0	t	1	Andrena miranda	Anthonomus sp.	20	0.01	0.05	0.02	0.05
0	0	0.32	0	t	1	Andrena	Crabro sp.	20	0.01	0.06	0.03	0.06
0	0	0.25	0	t	1	Trientalis borealis	Andrena nivalis	20	0.01	0.03	0.01	0.05
0	0	0.12	0	cv	1	Temnostoma barberi	Pyrophaena rosarum	20	0.01	0.07	0.01	0.06
0	0	0.11	0	t	1	Pieris napi	Eclimus harrisi	20	0.01	0.07	0.01	0.06
0	0	0.14	0	t	1	Sphaerophoria longipilosa	Temnostoma alternans	20	0.01	0.07	0.01	0.06
0	0	0.16	0	t	1	Cheilosia pallipes	Pyrophaena rosarum	20	0.01	0.05	0.01	0.06
0	0	0.44	0	cv	1	Chimaphila umbellata	Oxalis montana	20	0.01	0.03	0.03	0.05
0	0	0.26	0	t	1	Cheilosia pallipes	Dialictus imitatus	20	0.01	0.05	0.01	0.03
0	0	0.16	0	t	1	Osmia proxima	Thamnotettix confinis	20	0.01	0.06	0.01	0.04
0	0	0.39	0	t	1	Judolia montivagans	Maianthemum canadense	20	0.02	0.05	0.02	0.05
0	1	0.91	0	cv	1	Nomada sayi	Chalcosyrphus nigra	20	0.14	0.14	0.01	0.07
0	0	0.19	0	t	1	Bombylius major	Andrena w-scripta	20	0.01	0.05	0.01	0.05
0	1	0.78	0	t	1	Linnaea borealis	Cheilosia pallipes	20	0.08	0.05	0.01	0.05
0	0	0.48	0	t	1	Evylaeus divergens	Clintonia borealis	20	0.04	0.05	0.01	0.04
0	0	0.38	0	cv	1	Phalaenophana pyramusalis	Andrena wilkella	20	0.02	0.04	0.01	0.03
0	0	0.28	0	t	1	Cheilosia tristis	Eusphalerum convexum	20	0.01	0.03	0.01	0.05
0	0	0.31	0	t	1	Syritta rectus	Oxalis montana	20	0.01	0.07	0.03	0.05
0	0	0.38	0	t	1	Evylaeus divergens	Xylota sp.	20	0.04	0.05	0.01	0.07
0	0	0.14	0	cv	1	Chalcosyrphus inarmatus	Hylaeus stevensi	20	0.01	0.07	0.01	0.06
0	0	0.24	0	t	1	Pidonia ruficolis	Trachysida aspera	20	0.01	0.05	0.01	0.03
0	0	0.15	0	t	1	Pegohylemyia fugax	Psithyrus sp.	20	0.01	0.04	0.01	0.07
0	0	0.24	0	t	1	Blera nigripes	Crabro sp.	20	0.01	0.07	0.03	0.06
0	0	0.21	0	cv	1	Trientalis borealis	Halictus confusus	20	0.01	0.03	0.01	0.07

0	0	0.40	0	t	1	Judolia montivagans	Fannia ungulata	20	0.02	0.05	0.03	0.06
0	0	0.16	0	t	1	Tropidia quadrata	Syritta pipiens	20	0.01	0.05	0.01	0.07
0	0	0.23	0	t	1	Andrena miranda	Hylaeus ellipticus	20	0.01	0.05	0.01	0.06
0	0	0.12	0	cv	1	Andrena sigmundi	Andrena nivalis	20	0.01	0.07	0.01	0.05
0	0	0.26	0	t	1	Poanes hobomok	Bombylius major	20	0.01	0.03	0.01	0.05
0	0	0.19	0	t	1	Andrena sigmundi	Judolia montivagans	20	0.01	0.07	0.02	0.05
0	0	0.14	0	t	1	Evodinus monticola	Osmia pumila	20	0.01	0.07	0.01	0.06
0	0	0.19	0	cv	1	Evylaeus quebecensis	Trachysida aspera	20	0.01	0.06	0.01	0.03
0	0	0.20	0	t	1	Temnostoma alternans	Thamnotettix confinis	20	0.01	0.06	0.01	0.04
0	0	0.17	0	t	1	Andrena wheeleri	Blera badia	20	0.01	0.03	0.01	0.07
0	0	0.22	0	t	1	Blera nigripes	Carposcalis obscura	20	0.01	0.07	0.01	0.02
0	0	0.12	0	cv	1	Pieris napi	Bombus ternarius	20	0.01	0.07	0.01	0.06
0	0	0.22	0	t	1	Agiotes stabilis	Andrena w-scripta	20	0.01	0.04	0.01	0.05
0	0	0.16	0	t	1	Andrena w-scripta	Xylota bigelowi	20	0.01	0.05	0.01	0.07
0	0	0.16	0	t	1	Andrena w-scripta	Sphecodes sp.	20	0.01	0.05	0.01	0.07
0	0	0.12	0	cv	1	Chalcosyrphus inarmatus	Pyrophaena rosarum	20	0.01	0.07	0.01	0.06
0	0	0.48	0	t	1	Poanes hobomok	Chalcosyrphus curvarius	20	0.01	0.03	0.03	0.05
0	0	0.14	0	t	1	Syritta rectus	Halictus rubicundus	20	0.01	0.07	0.01	0.04
0	0	0.22	0	t	1	Zeraea americana	Dialictus imitatus	20	0.01	0.06	0.01	0.03
0	0	0.10	0	cv	1	Lygus	Blera badia	20	0.01	0.07	0.01	0.07
1	0	0.33	0	t	1	Cheilosia tristis	Andrena wilkella	20	0.01	0.03	0.01	0.03
0	0	0.33	0	t	1	Anthonomus sp.	Eusphalerum phothos	20	0.02	0.05	0.02	0.05
0	0	0.23	0	t	1	Andrena wilkella	Hoplitis producta	20	0.01	0.03	0.01	0.04
0	0	0.37	0	cv	1	Bombylius major	Oxalis montana	20	0.01	0.05	0.03	0.05
0	0	0.14	0	t	1	Chalcosyrphus memorum	Bombylius pigmaeus	20	0.01	0.07	0.01	0.04
0	0	0.15	0	t	1	Osmia pumila	Sphecodes sp.	20	0.01	0.06	0.01	0.07

0	0	0.19	0	t	1	Anthonomus	Cantharis sp.	20	0.02	0.05	0.01	0.07
0	1	0.57	0	cv	1	Evylaeus divergens	Eusphalerum phothos	20	0.04	0.05	0.02	0.05
0	0	0.25	0	t	1	Andrena wilkella	Sarcophaga nearctica	20	0.01	0.03	0.01	0.05
0	0	0.12	0	t	1	Andrena sigmundi	Mallota posticata	20	0.01	0.07	0.01	0.06
0	0	0.13	0	t	1	Eclimus harrisi	Melagyna lasiophthalma	20	0.01	0.06	0.01	0.06
0	0	0.14	0	cv	1	Andrena sigmundi	Celastrina argiolus	20	0.01	0.07	0.01	0.06
0	0	0.26	0	t	1	Pieris napi	Phalaenophana pyramusalis	20	0.01	0.07	0.02	0.04
0	0	0.10	0	t	1	Evodinus monticola	Psithyrus sp.	20	0.01	0.07	0.01	0.07
0	0	0.18	0	t	1	Aralia nudicaulis	Sphaerophoria bifurcata	20	0.01	0.04	0.01	0.06
0	0	0.15	0	cv	1	Bombus vagans	Dialictus cressoni	20	0.01	0.06	0.01	0.06
0	0	0.20	0	t	1	Andrena thaspii	Celastrina argiolus	20	0.01	0.06	0.01	0.06
0	0	0.17	0	t	1	Trillium undulatum	Syritta pipiens	20	0.01	0.05	0.01	0.07
0	0	0.12	0	t	1	Chalcosyrphus nigra	Xylota hinei	20	0.01	0.07	0.01	0.05
0	0	0.35	0	cv	1	Pegohylemyia fugax	Phalaenophana pyramusalis	20	0.01	0.04	0.02	0.04
0	0	0.15	0	t	1	Bombylius pigmaeus	Chalcosyrphus inarmatus	20	0.01	0.04	0.01	0.07
0	0	0.10	0	t	1	Acmaeopsoides rufula	Xylota sp.	20	0.01	0.07	0.01	0.07
0	0	0.17	0	t	1	Vespula arenaria	Dialictus cressoni	20	0.01	0.04	0.01	0.06
0	0	0.34	0	cv	1	Eclimus harrisi	Oxalis montana	20	0.01	0.06	0.03	0.05
0	0	0.19	0	t	1	Osmia lignaria	Passaloecus sp.	20	0.01	0.03	0.01	0.06
0	0	0.10	0	t	1	Tychius stephensi	Cheilosia sialia	20	0.01	0.07	0.01	0.07
0	0	0.21	0	t	1	Anthonomus sp.	Ephemerella sp.	20	0.02	0.05	0.01	0.08
0	0	0.23	0	cv	1	Chalcosyrphus inarmatus	Andrena rufosignata	20	0.01	0.07	0.02	0.05
0	0	0.30	0	t	1	Trachysida aspera	Anthonomus sp.	20	0.01	0.03	0.02	0.05
0	0	0.10	0	t	1	Chalcosyrphus memorum	Halictus confusus	20	0.01	0.07	0.01	0.07

0	0	0.10	0	t	1	Sphaerophoria longipilosa	Syritta pipiens	20	0.01	0.07	0.01	0.07
0	0	0.27	0	cv	1	Tychius stephensi	Chalcosyrphus curvarius	20	0.01	0.07	0.03	0.05
0	0	0.23	0	t	1	Rhopalum sp.	Thamnotettix confinis	20	0.01	0.03	0.01	0.04
0	1	0.67	0	t	1	Dialictus sp 2.	Andrena thaspii	20	0.06	0.05	0.01	0.06
0	1	0.57	0	t	1	Dialictus sp 2.	Blera nigripes	20	0.06	0.05	0.01	0.07
0	0	0.25	0	cv	1	Anthaxia expansa	Hoplitis producta	20	0.02	0.05	0.01	0.04
0	0	0.20	0	t	1	Bombus perplexus	Pyrola secunda	20	0.01	0.05	0.01	0.05
0	0	0.27	0	t	1	Temnostoma balyras	Trientalis borealis	20	0.01	0.06	0.01	0.03
0	0	0.10	0	t	1	Temnostoma barberi	Ancistrocerus sp.	20	0.01	0.07	0.01	0.07
0	0	0.10	0	cv	1	Dalopius sp.	Xylota sp.	20	0.01	0.07	0.01	0.07
0	0	0.12	0	t	1	Blera nigripes	Melagyna lasiophthalma	20	0.01	0.07	0.01	0.06
0	1	0.91	0	t	1	Nomada sayi	Chalcosyrphus inarmatus	20	0.14	0.14	0.01	0.07
0	0	0.18	0	t	1	Bombylius major	Halictus rubicundus	20	0.01	0.05	0.01	0.04
0	0	0.28	0	cv	1	Parasyrphus relictus	Hylaeus stevensi	20	0.02	0.05	0.01	0.06
0	0	0.17	0	t	1	Evylaeus quebecensis	Vespula arenaria	20	0.01	0.06	0.01	0.04
0	0	0.39	0	t	1	Andrena wheeleri	Phalaenophana pyramusalis	20	0.01	0.03	0.02	0.04
0	0	0.12	0	t	1	Temnostoma barberi	Evylaeus quebecensis	20	0.01	0.07	0.01	0.06
0	0	0.10	0	cv	1	Syritta rectus	Dalopius sp.	20	0.01	0.07	0.01	0.07
0	0	0.10	0	t	1	Chalcosyrphus nigra	Chalcosyrphus inarmatus	20	0.01	0.07	0.01	0.07
0	0	0.17	0	t	1	Nomada cuneata	Passaloecus sp.	20	0.01	0.04	0.01	0.06
0	0	0.27	0	t	1	Cypripedium acaule	Andrena miranda	20	0.01	0.04	0.01	0.05
0	0	0.31	0	cv	1	Andrena rufosignata	Temnostoma alternans	20	0.02	0.05	0.01	0.06
0	0	0.18	0	t	1	Temnostoma balyras	Andrena nivalis	20	0.01	0.06	0.01	0.05
0	0	0.22	0	t	1	Poanes hobomok	Chalcosyrphus inarmatus	20	0.01	0.03	0.01	0.07
0	0	0.37	0	t	1	Poanes hobomok	Anthaxia expansa	20	0.01	0.03	0.02	0.05
0	1	0.69	0	cv	1	Dialictus sp 2.	Bombus perplexus	20	0.06	0.05	0.01	0.05

0	0	0.38	0	t	1	Evylaeus divergens	Pieris napi	20	0.04	0.05	0.01	0.07
0	0	0.20	0	t	1	Cateres pennatus	Melagyna lasiophthalma	20	0.01	0.03	0.01	0.06
0	0	0.10	0	t	1	Acmaeopsoides rufula	Sphecodes sp.	20	0.01	0.07	0.01	0.07
0	1	0.83	0	cv	1	Andrena thaspii	Medeola virginiana	20	0.01	0.06	0.09	0.08
0	0	0.27	0	t	1	Bombus ternarius	Crabro sp.	20	0.01	0.06	0.03	0.06
0	0	0.14	0	t	1	Evodinus monticola	Bombylius pigmaeus	20	0.01	0.07	0.01	0.04
0	0	0.25	0	t	1	Bombus terricola	Dalopius sp.	20	0.02	0.04	0.01	0.07
0	0	0.37	0	cv	1	Osmia lignaria	Fannia ungulata	20	0.01	0.03	0.03	0.06
0	0	0.15	0	t	1	Xylota hinei	Bombus ternarius	20	0.01	0.05	0.01	0.06
0	0	0.21	0	t	1	Dialictus imitatus	Halictus confusus	20	0.01	0.03	0.01	0.07
0	0	0.21	0	t	1	Paramyia nitens	Pyrola secunda	20	0.01	0.05	0.01	0.05
0	0	0.11	0	cv	1	Blera nigripes	Zeraea americana	20	0.01	0.07	0.01	0.06
0	1	0.94	0	t	1	Nomada sayi	Bombylius pigmaeus	20	0.14	0.14	0.01	0.04
0	0	0.12	0	t	1	Syritta rectus	Dialictus cressoni	20	0.01	0.07	0.01	0.06
0	1	0.91	0	t	1	Nomada sayi	Hylaeus basalis	20	0.14	0.14	0.01	0.07
0	0	0.14	0	cv	1	Ancistrocerus sp.	Phaonia serva	20	0.01	0.07	0.01	0.06
0	0	0.34	0	t	1	Bombus terricola	Temnostoma balyras	20	0.02	0.04	0.01	0.06
0	0	0.26	0	t	1	Eusphalerum convexum	Eusphalerum phothos	20	0.01	0.05	0.02	0.05
0	0	0.15	0	t	1	Pegohylemyia fugax	Sphecodes sp.	20	0.01	0.04	0.01	0.07
0	0	0.19	0	cv	1	Judolia montivagans	Chalcosyrphus inarmatus	20	0.02	0.05	0.01	0.07
0	0	0.14	0	t	1	Lygus refidorsus	Pegohylemyia fugax	20	0.01	0.07	0.01	0.04
0	0	0.19	0	t	1	Chalcosyrphus nigra	Anthaxia expansa	20	0.01	0.07	0.02	0.05
0	0	0.12	0	t	1	Evylaeus quebecensis	Blera badia	20	0.01	0.06	0.01	0.07
0	0	0.14	0	cv	1	Evodinus monticola	Eremomyioides setosa	20	0.01	0.07	0.01	0.04
0	0	0.18	0	t	1	Andrena miranda	Bombus ternarius	20	0.01	0.05	0.01	0.06

0	1	0.96	0	t	1	Nomada sayi	Trientalis borealis	20	0.14	0.14	0.01	0.03
0	0	0.19	0	t	1	Pieris napi	Anthonomus sp.	20	0.01	0.07	0.02	0.05
0	0	0.24	0	cv	1	Fannia	Cantharis sp.	20	0.03	0.06	0.01	0.07
0	0	0.22	0	t	1	Tropidia quadrata	Hoplitis producta	20	0.01	0.05	0.01	0.04
0	0	0.10	0	t	1	Andrena sigmundi	Evodinus monticola	20	0.01	0.07	0.01	0.07
0	1	0.92	0	t	1	Nomada sayi	Zeraea americana	20	0.14	0.14	0.01	0.06
0	0	0.15	0	cv	1	Syritta pipiens	Sarcophaga nearctica	20	0.01	0.07	0.01	0.05
0	1	0.96	0	t	1	Nomada sayi	Carposcalis obscura	20	0.14	0.14	0.01	0.02
0	0	0.10	0	t	1	Lygus	Cantharis sp.	20	0.01	0.07	0.01	0.07
0	0	0.09	0	t	1	Cornus canadensis	Carposcalis confusus	20	0.01	0.09	0.01	0.06
0	0	0.32	0	cv	1	Maianthemum canadense	Hylaeus stevensi	20	0.02	0.05	0.01	0.06
0	1	0.66	0	t	1	Xylota bigelowi	Cheilosia slossonae	20	0.01	0.07	0.07	0.05
0	0	0.10	0	t	1	Andrena sigmundi	Pieris napi	20	0.01	0.07	0.01	0.07
0	0	0.17	0	t	1	Eusphalerum sp.	Vespula arenaria	20	0.01	0.05	0.01	0.04
0	0	0.14	0	cv	1	Temnostoma barberi	Eremomyioides setosa	20	0.01	0.07	0.01	0.04
1	0	0.44	1	t	1	Satyrus charon	Erigeron macranthus	21	0.00	0.03	0.02	0.02
1	0	0.24	1	t	1	Satyrus charon	Sedum stenopetalum	21	0.00	0.03	0.00	0.02
1	0	0.48	1	t	1	Satyrus charon	Carduus hookerianus	21	0.00	0.03	0.03	0.02
1	0	0.34	1	cv	1	Paragus bicolor	Sieversia turbinata	21	0.00	0.00	0.00	0.00
1	1	0.57	1	t	1	Andronicus sp.	Pentstemon gracilis	21	0.01	0.03	0.04	0.02
1	1	0.69	1	t	1	Andronicus sp.	Geranium caespitosum	21	0.01	0.03	0.05	0.02
1	0	0.47	1	t	1	Andronicus sp.	Carduus hookerianus	21	0.01	0.03	0.03	0.02
1	0	0.25	1	cv	1	Andronicus sp.	Rubus deliciosus	21	0.01	0.03	0.00	0.02
1	0	0.27	1	t	1	Asclepias halli	Syrphus americanus	21	0.01	0.02	0.00	0.03
1	0	0.30	1	t	1	Asclepias halli	Triepeolus helianthi grandior	21	0.01	0.02	0.00	0.02
1	0	0.29	1	t	1	Asclepias halli	Apis mellifica	21	0.01	0.02	0.00	0.03

1	0	0.37	1	cv	1	Asclepias halli	Pseudomelecta californica miranda	21	0.01	0.02	0.01	0.02
1	0	0.30	1	t	1	Asclepias halli	Bembex spinolae	21	0.01	0.02	0.00	0.02
1	0	0.37	1	t	1	Asclepias halli	Halictus chloralictus	21	0.01	0.02	0.01	0.03
1	0	0.30	1	t	1	Asclepias halli	Tetraopes sp.	21	0.01	0.02	0.00	0.02
1	0	0.35	1	cv	1	Clementsia rhodantha	Anthomyia sp.	21	0.00	0.00	0.00	0.00
1	0	0.30	1	t	1	Campanula rotundifolia	Chrysophanus rubidus	21	0.01	0.02	0.00	0.03
1	0	0.27	1	t	1	Campanula rotundifolia	bombus hunti	21	0.01	0.02	0.00	0.03
1	0	0.26	1	t	1	Campanula rotundifolia	bombus juxtus	21	0.01	0.02	0.00	0.03
1	0	0.29	1	cv	1	Campanula rotundifolia	Anthophora smithi	21	0.01	0.02	0.00	0.03
1	0	0.26	1	t	1	Campanula rotundifolia	Megachile wootoni	21	0.01	0.02	0.00	0.03
1	0	0.34	1	t	1	Campanula rotundifolia	bombus edwardsi	21	0.01	0.02	0.01	0.03
1	0	0.30	1	t	1	Campanula rotundifolia	Odynerus annulatus	21	0.01	0.02	0.00	0.02
1	0	0.48	1	cv	1	Erysimum asperum	bombus occidentalis	21	0.00	0.02	0.03	0.03
1	1	0.64	1	t	1	Syrphus amalopis	Geranium caespitosum	21	0.00	0.03	0.05	0.02
1	1	0.66	1	t	1	Melitaea sp.	Geranium caespitosum	21	0.00	0.03	0.05	0.02
1	0	0.44	1	t	1	Melitaea sp.	Carduus hookerianus	21	0.00	0.03	0.03	0.02
1	1	0.54	1	cv	1	Andrena washingtoni	Pentstemon gracilis	21	0.00	0.03	0.04	0.02
1	1	0.62	1	t	1	Andrena washingtoni	Rosa acicularis	21	0.00	0.03	0.04	0.02
1	0	0.25	1	t	1	Osmia melanotricha	Pentstemon glaber	21	0.01	0.03	0.00	0.02
1	0	0.31	1	t	1	Osmia melanotricha	Penstemon halli	21	0.01	0.03	0.01	0.02
1	1	0.59	1	cv	1	Osmia melanotricha	Pentstemon gracilis	21	0.01	0.03	0.04	0.02
1	0	0.36	1	t	1	Osmia melanotricha	Pentstemon glaucus	21	0.01	0.03	0.01	0.02
1	1	0.67	1	t	1	Osmia melanotricha	Rosa acicularis	21	0.01	0.03	0.04	0.02
1	0	0.36	1	t	1	Osmia melanotricha	Mertensia pratensis	21	0.01	0.03	0.01	0.02
1	0	0.46	1	cv	1	Psithyrus fernaldae	Carduus hookerianus	21	0.00	0.03	0.03	0.02

1	1	0.58	1	t	1	Osmia wardiana	Chamaenerium angustifolium	21	0.00	0.03	0.04	0.02
1	0	0.20	1	t	1	Elephantella groenlandica	Osmia bruneri	21	0.00	0.02	0.00	0.03
1	0	0.25	1	t	1	Elephantella groenlandica	bombus bifarius	21	0.00	0.02	0.01	0.03
1	0	0.29	1	cv	1	Ophion sp.	Caltha leptosepala	21	0.00	0.01	0.00	0.01
1	0	0.30	1	t	1	Eliphilus latifrons	Cleome serrulata	21	0.00	0.02	0.01	0.02
1	0	0.23	1	t	1	Colletes kincaidi	Prunus demissa	21	0.01	0.03	0.00	0.02
1	0	0.31	1	t	1	Colletes kincaidi	Petalostemon purpureus	21	0.01	0.03	0.01	0.02
1	1	0.56	1	cv	1	Colletes kincaidi	Pentstemon gracilis	21	0.01	0.03	0.04	0.02
1	1	0.69	1	t	1	Colletes kincaidi	Geranium caespitosum	21	0.01	0.03	0.05	0.02
1	1	0.56	1	t	1	Heracleum lanatum	bombus occidentalis	21	0.01	0.02	0.03	0.03
1	0	0.50	1	t	1	Heracleum lanatum	Xylota angustiventris	21	0.01	0.02	0.02	0.02
0	0	0.27	1	cv	1	Heracleum lanatum	Tenthredella flavomarginis	21	0.01	0.02	0.00	0.03
0	0	0.27	1	t	1	Heracleum lanatum	Anthrax hypomelas	21	0.01	0.02	0.00	0.03
0	0	0.27	1	t	1	Heracleum lanatum	Ectemius muricatus	21	0.01	0.02	0.00	0.03
1	0	0.28	1	t	1	Heracleum lanatum	Echinomyia algens	21	0.01	0.02	0.00	0.03
1	0	0.32	1	cv	1	Heracleum lanatum	Hammerschmidtia ferruginea	21	0.01	0.02	0.01	0.03
1	0	0.47	1	t	1	bombus occidentalis	Prunus demissa	21	0.03	0.03	0.00	0.02
1	0	0.47	1	t	1	bombus occidentalis	Pentstemon glaber	21	0.03	0.03	0.00	0.02
1	1	0.59	1	t	1	bombus occidentalis	Sieversia ciliata	21	0.03	0.03	0.01	0.02
1	1	0.57	1	cv	1	bombus occidentalis	Petalostemon purpureus	21	0.03	0.03	0.01	0.02
1	0	0.47	1	t	1	bombus occidentalis	Allium recurvatum	21	0.03	0.03	0.00	0.02
1	0	0.49	1	t	1	bombus occidentalis	Aquilegia coerulea	21	0.03	0.03	0.00	0.02
1	1	0.63	1	t	1	bombus occidentalis	Solidago missouriensis	21	0.03	0.03	0.02	0.02
1	1	0.64	1	cv	1	bombus occidentalis	Penstemon secundiflorus	21	0.03	0.03	0.02	0.02

1	1	0.57	1	t	1	bombus occidentalis	Calochortus gunnisoni	21	0.03	0.03	0.01	0.02
1	1	0.61	1	t	1	bombus occidentalis	Potentilla pulcherrima	21	0.03	0.03	0.02	0.02
1	1	0.59	1	t	1	bombus occidentalis	Jamesia americana	21	0.03	0.03	0.01	0.02
1	1	0.73	1	cv	1	bombus occidentalis	Rubus strigosus	21	0.03	0.03	0.03	0.02
1	1	0.52	1	t	1	bombus occidentalis	Linaria vulgaris	21	0.03	0.03	0.01	0.02
1	1	0.60	1	t	1	bombus occidentalis	Pentstemon glaucus	21	0.03	0.03	0.01	0.02
1	1	0.87	1	t	1	bombus occidentalis	Geranium caespitosum	21	0.03	0.03	0.05	0.02
1	0	0.46	1	cv	1	bombus occidentalis	Geranium richardsoni	21	0.03	0.03	0.00	0.02
1	1	0.51	1	t	1	bombus occidentalis	Monarda fistulosa	21	0.03	0.03	0.01	0.02
1	1	0.74	1	t	1	bombus occidentalis	Pedicularis racemosa	21	0.03	0.03	0.03	0.02
1	0	0.48	1	t	1	bombus occidentalis	Aconitum columbianum	21	0.03	0.03	0.00	0.02
1	1	0.77	1	cv	1	bombus occidentalis	Mentzelia multiflora	21	0.03	0.03	0.03	0.03
1	1	0.55	1	t	1	bombus occidentalis	Holodiscus dumosus	21	0.03	0.03	0.01	0.02
1	1	0.83	1	t	1	bombus occidentalis	Chamaeneriu m angustifolium	21	0.03	0.03	0.04	0.02
1	1	0.84	1	t	1	bombus occidentalis	Rosa acicularis	21	0.03	0.03	0.04	0.02
1	1	0.56	1	cv	1	bombus occidentalis	Capnoides aureum	21	0.03	0.03	0.01	0.02
1	1	0.74	1	t	1	bombus occidentalis	Mertensia sibirica	21	0.03	0.03	0.03	0.02
1	1	0.66	1	t	1	bombus occidentalis	Opulaster opulifolius	21	0.03	0.03	0.02	0.02
1	1	0.58	1	t	1	bombus occidentalis	Castilleia miniata	21	0.03	0.03	0.01	0.02
1	0	0.47	1	cv	1	bombus occidentalis	Erigeron uniflorus	21	0.03	0.03	0.00	0.02
1	1	0.73	1	t	1	bombus occidentalis	Carduus hookerianus	21	0.03	0.03	0.03	0.02
1	0	0.50	1	t	1	bombus occidentalis	Rubus deliciosus	21	0.03	0.03	0.00	0.02
1	0	0.40	1	t	1	Gnophaela vermiculata	Solidago missouriensis	21	0.01	0.03	0.02	0.02
1	0	0.32	1	cv	1	Gnophaela vermiculata	Polygonum bistorta	21	0.01	0.03	0.01	0.02
1	0	0.38	1	t	1	Gnophaela vermiculata	Potentilla pulcherrima	21	0.01	0.03	0.02	0.02

1	1	0.52	1	t	1	Gnophaela vermiculata	Rubus strigosus	21	0.01	0.03	0.03	0.02
1	0	0.28	1	t	1	Gnophaela vermiculata	Achillea millefolium	21	0.01	0.03	0.01	0.03
1	0	0.23	1	cv	1	Chrysophanus rubidus	Phacelia heterophylla	21	0.00	0.03	0.00	0.02
1	0	0.48	1	t	1	Chrysophanus rubidus	Carduus hookerianus	21	0.00	0.03	0.03	0.02
1	0	0.45	1	t	1	Andrena lewisi	Rubus strigosus	21	0.00	0.03	0.03	0.02
1	0	0.23	1	t	1	Caltha leptosepala	Irbisia brachycerus	21	0.00	0.01	0.00	0.03
1	0	0.21	1	cv	1	Prunus demissa	Halictus (Lasioglossu m) sp.	21	0.00	0.02	0.00	0.03
1	0	0.23	1	t	1	Prunus demissa	Andrena crataegi	21	0.00	0.02	0.00	0.03
1	0	0.24	1	t	1	Prunus demissa	Syritta pipiens	21	0.00	0.02	0.00	0.02
1	0	0.32	1	t	1	Prunus demissa	Calliphora vomitoria	21	0.00	0.02	0.01	0.01
0	0	0.27	1	cv	1	Prunus demissa	Andrena edwiniae	21	0.00	0.02	0.01	0.02
1	0	0.24	1	t	1	Prunus demissa	Eristalis dimidiatus	21	0.00	0.02	0.00	0.02
1	0	0.23	1	t	1	Prunus	Andrena sp.	21	0.00	0.02	0.00	0.03
1	0	0.35	1	t	1	Prunus demissa	Zodion pygmaeum	21	0.00	0.02	0.01	0.01
0	0	0.24	1	cv	1	Prunus demissa	Brenthis triclaris	21	0.00	0.02	0.00	0.03
1	0	0.24	1	t	1	Prunus demissa	Syrphus disgregus	21	0.00	0.02	0.00	0.02
1	0	0.35	1	t	1	Prunus demissa	Chilosia petulca	21	0.00	0.02	0.01	0.01
1	0	0.24	1	t	1	Prunus demissa	Agapostemon sp.	21	0.00	0.02	0.00	0.02
0	0	0.24	1	cv	1	Prunus demissa	Brachyopa notata	21	0.00	0.02	0.00	0.02
1	0	0.23	1	t	1	Prunus demissa	Eristalis arbustorum	21	0.00	0.02	0.00	0.03
1	0	0.24	1	t	1	Prunus demissa	Eristalis meigeni	21	0.00	0.02	0.00	0.02
1	0	0.24	1	t	1	Prunus demissa	Spegina infuscata	21	0.00	0.02	0.00	0.02
1	0	0.36	1	cv	1	Prunus demissa	bombus flavifrons	21	0.00	0.02	0.02	0.03
1	0	0.36	1	t	1	Prunus demissa	Halictus pulzenus	21	0.00	0.02	0.02	0.03
1	0	0.30	1	t	1	Prunus demissa	Halictus (Evylaeus) sp.	21	0.00	0.02	0.01	0.03

1	0	0.24	1	t	1	Prunus demissa	Theventimtia muricatus	21	0.00	0.02	0.00	0.02
1	0	0.24	1	cv	1	Prunus demissa	Erebia epipsodea	21	0.00	0.02	0.01	0.03
0	0	0.32	1	t	1	Prunus	Halictus sp.	21	0.00	0.02	0.01	0.03
0	0	0.22	1	t	1	Prunus demissa	Eristalis temporalis	21	0.00	0.02	0.00	0.03
1	0	0.21	1	t	1	Prunus demissa	Colletes oromontis	21	0.00	0.02	0.00	0.03
0	0	0.22	1	cv	1	Prunus demissa	Andrena prunorum	21	0.00	0.02	0.00	0.03
1	0	0.28	1	t	1	Prunus demissa	Specomyia vittata	21	0.00	0.02	0.00	0.01
1	0	0.21	1	t	1	Prunus demissa	Pseudomasar is vespoides	21	0.00	0.02	0.00	0.03
1	0	0.23	1	t	1	Prunus demissa	Acmaeops pratensis	21	0.00	0.02	0.00	0.03
1	0	0.24	1	cv	1	Prunus demissa	Chilosia tristis	21	0.00	0.02	0.00	0.02
1	0	0.24	1	t	1	Prunus demissa	Ptilodexia harpasa	21	0.00	0.02	0.00	0.02
1	0	0.30	1	t	1	Prunus demissa	Halictus chloralictus	21	0.00	0.02	0.01	0.03
1	0	0.30	1	t	1	Prunus demissa	Andrena vicina	21	0.00	0.02	0.01	0.03
1	0	0.34	1	cv	1	Prunus demissa	Andrena madronitens	21	0.00	0.02	0.02	0.03
1	0	0.24	1	t	1	Prunus demissa	Microdon cothurnatus	21	0.00	0.02	0.00	0.02
1	0	0.25	1	t	1	Prunus demissa	Coenonymph a pamphiloides	21	0.00	0.02	0.00	0.03
1	0	0.25	1	t	1	Prunus demissa	Acmaeops longicornis	21	0.00	0.02	0.01	0.03
0	0	0.24	1	cv	1	Prunus demissa	Andrena canadensis	21	0.00	0.02	0.00	0.02
1	1	0.77	1	t	1	Erizoa olivalis	Carduus hookerianus	21	0.04	0.03	0.03	0.02
1	0	0.36	1	t	1	Volucella satur	Stanleya pinnatifida	21	0.00	0.02	0.02	0.02
1	0	0.31	1	t	1	Andrena gardineri	Astragalus drummondi	21	0.00	0.03	0.01	0.02
1	0	0.20	1	cv	1	Osmia bruneri	Pentstemon glaber	21	0.00	0.03	0.00	0.02
1	0	0.33	1	t	1	Osmia bruneri	Penstemon secundiflorus	21	0.00	0.03	0.02	0.02
1	1	0.52	1	t	1	Osmia bruneri	Pentstemon gracilis	21	0.00	0.03	0.04	0.02
1	1	0.65	1	t	1	Osmia bruneri	Geranium caespitosum	21	0.00	0.03	0.05	0.02

1	1	0.60	1	cv	1	Osmia bruneri	Rosa acicularis	21	0.00	0.03	0.04	0.02
1	0	0.35	1	t	1	Osmia bruneri	Opulaster opulifolius	21	0.00	0.03	0.02	0.02
1	0	0.26	1	t	1	Agapostemon splendens	Pentstemon glaber	21	0.01	0.03	0.00	0.02
1	1	0.68	1	t	1	Agapostemon splendens	Rosa acicularis	21	0.01	0.03	0.04	0.02
1	0	0.26	1	cv	1	Agapostemon splendens	Taraxacum officinale	21	0.01	0.03	0.00	0.02
1	0	0.26	1	t	1	Agapostemon splendens	Malvastrum coccineum	21	0.01	0.03	0.00	0.02
1	0	0.23	1	t	1	Thanaos martialis	Pentstemon glaber	21	0.01	0.03	0.00	0.02
1	0	0.44	1	t	1	Thanaos martialis	Erigeron macranthus	21	0.01	0.03	0.02	0.02
1	1	0.69	1	cv	1	Thanaos martialis	Geranium caespitosum	21	0.01	0.03	0.05	0.02
1	0	0.23	1	t	1	Thanaos martialis	Geranium richardsoni	21	0.01	0.03	0.00	0.02
1	0	0.23	1	t	1	Thanaos martialis	Delphinium scopulorum	21	0.01	0.03	0.00	0.02
1	0	0.26	1	t	1	Thanaos martialis	Monarda fistulosa	21	0.01	0.03	0.01	0.02
1	1	0.62	1	cv	1	Thanaos martialis	Chamaeneriu m angustifolium	21	0.01	0.03	0.04	0.02
1	0	0.49	1	t	1	Thanaos martialis	Mertensia sibirica	21	0.01	0.03	0.03	0.02
1	0	0.28	1	t	1	Prosopis episcopalis	Pentstemon glaber	21	0.01	0.03	0.00	0.02
1	0	0.30	1	t	1	Prosopis episcopalis	Galium boreale	21	0.01	0.03	0.00	0.02
1	1	0.55	1	cv	1	Prosopis episcopalis	Rubus strigosus	21	0.01	0.03	0.03	0.02
1	0	0.24	1	t	1	Panurginus sp.	Pentstemon glaber	21	0.00	0.03	0.00	0.02
1	0	0.24	1	t	1	Panurginus sp.	Tradescantia virginiana	21	0.00	0.03	0.00	0.02
1	0	0.32	1	t	1	Panurginus sp.	Calochortus gunnisoni	21	0.00	0.03	0.01	0.02
1	0	0.45	1	cv	1	Panurginus sp.	Erigeron macranthus	21	0.00	0.03	0.02	0.02
1	0	0.26	1	t	1	Panurginus sp.	Rubus deliciosus	21	0.00	0.03	0.00	0.02
1	0	0.24	1	t	1	bombus bifarius	Pentstemon glaber	21	0.01	0.03	0.00	0.02
1	0	0.25	1	t	1	bombus bifarius	Dodocatheon meadia	21	0.01	0.03	0.00	0.02
1	0	0.39	1	cv	1	bombus bifarius	Penstemon secundiflorus	21	0.01	0.03	0.02	0.02

1	0	0.45	1	t	1	bombus bifarius	Erigeron macranthus	21	0.01	0.03	0.02	0.02
1	0	0.34	1	t	1	bombus bifarius	Jamesia americana	21	0.01	0.03	0.01	0.02
1	0	0.50	1	t	1	bombus bifarius	Rubus strigosus	21	0.01	0.03	0.03	0.02
1	1	0.59	1	cv	1	bombus bifarius	Pentstemon gracilis	21	0.01	0.03	0.04	0.02
1	0	0.35	1	t	1	bombus bifarius	Pentstemon glaucus	21	0.01	0.03	0.01	0.02
1	1	0.71	1	t	1	bombus bifarius	Geranium caespitosum	21	0.01	0.03	0.05	0.02
1	0	0.24	1	t	1	bombus bifarius	Geranium richardsoni	21	0.01	0.03	0.00	0.02
1	0	0.24	1	cv	1	bombus bifarius	Delphinium scopulorum	21	0.01	0.03	0.00	0.02
1	0	0.27	1	t	1	bombus bifarius	Monarda fistulosa	21	0.01	0.03	0.01	0.02
1	0	0.25	1	t	1	bombus bifarius	Aconitum columbianum	21	0.01	0.03	0.00	0.02
1	1	0.56	1	t	1	bombus bifarius	Mentzelia multiflora	21	0.01	0.03	0.03	0.03
1	0	0.31	1	cv	1	bombus bifarius	Holodiscus dumosus	21	0.01	0.03	0.01	0.02
1	1	0.64	1	t	1	bombus bifarius	Chamaeneriu m angustifolium	21	0.01	0.03	0.04	0.02
1	0	0.38	1	t	1	bombus bifarius	Stanleya pinnatifida	21	0.01	0.03	0.02	0.02
1	1	0.51	1	t	1	bombus bifarius	Mertensia sibirica	21	0.01	0.03	0.03	0.02
1	0	0.49	1	cv	1	bombus bifarius	Carduus hookerianus	21	0.01	0.03	0.03	0.02
1	0	0.27	1	t	1	bombus bifarius	Rubus deliciosus	21	0.01	0.03	0.00	0.02
0	0	0.41	1	t	1	Xylota angustiventris	Pentstemon glaber	21	0.02	0.02	0.00	0.02
0	0	0.30	1	t	1	Trichius affinis	Calochortus gunnisoni	21	0.00	0.03	0.01	0.02
1	1	0.58	1	cv	1	Psithyrus ashtoni	Chamaeneriu m angustifolium	21	0.00	0.03	0.04	0.02
0	0	0.21	1	t	1	Pentstemon glaber	Melissodes fremonti	21	0.00	0.02	0.00	0.03
1	0	0.21	1	t	1	Pentstemon glaber	Anthophora simillima	21	0.00	0.02	0.00	0.03
1	0	0.25	1	t	1	Pentstemon glaber	Celerio lineata	21	0.00	0.02	0.00	0.02
1	0	0.21	1	cv	1	Pentstemon glaber	bombus hunti	21	0.00	0.02	0.00	0.03

1	0	0.21	1	t	1	Pentstemon glaber	bombus juxtus	21	0.00	0.02	0.00	0.03
0	0	0.23	1	t	1	Pentstemon glaber	Tetrachrysis lauta	21	0.00	0.02	0.00	0.02
1	0	0.30	1	t	1	Pentstemon glaber	Prosopis varifrons	21	0.00	0.02	0.01	0.03
1	0	0.25	1	cv	1	Pentstemon glaber	bombus centralis	21	0.00	0.02	0.01	0.03
1	0	0.21	1	t	1	Pentstemon glaber	Vespa germanica	21	0.00	0.02	0.00	0.03
1	0	0.23	1	t	1	Pentstemon glaber	Bombomelecta fulvida	21	0.00	0.02	0.00	0.03
0	0	0.23	1	t	1	Pentstemon glaber	Anthophora bomboides	21	0.00	0.02	0.00	0.02
0	0	0.23	1	cv	1	Pentstemon glaber	Oxybelus sp.	21	0.00	0.02	0.00	0.02
1	0	0.28	1	t	1	Pentstemon glaber	Osmia densa	21	0.00	0.02	0.01	0.03
1	0	0.25	1	t	1	Pentstemon glaber	Megachile wootoni calogaster	21	0.00	0.02	0.01	0.03
1	0	0.36	1	t	1	Pentstemon glaber	Halictus pulzenus	21	0.00	0.02	0.02	0.03
1	0	0.31	1	cv	1	Pentstemon glaber	bombus rufocinctus	21	0.00	0.02	0.01	0.03
1	0	0.32	1	t	1	Pentstemon glaber	Monumetha albifrons	21	0.00	0.02	0.02	0.03
1	0	0.32	1	t	1	Pentstemon glaber	Prosopis wootoni	21	0.00	0.02	0.02	0.03
0	0	0.23	1	t	1	Pentstemon glaber	Gonochrysis densa	21	0.00	0.02	0.00	0.02
1	0	0.20	1	cv	1	Pentstemon glaber	Osmia phaceliae	21	0.00	0.02	0.00	0.03
1	0	0.26	1	t	1	Pentstemon glaber	Atrytone taxiles	21	0.00	0.02	0.01	0.03
1	0	0.40	1	t	1	Pentstemon glaber	bombus appositus	21	0.00	0.02	0.02	0.03
1	0	0.25	1	t	1	Pentstemon glaber	Tenthredella unicincta	21	0.00	0.02	0.00	0.02
1	0	0.21	1	cv	1	Pentstemon glaber	Megachile wootoni	21	0.00	0.02	0.00	0.03
0	0	0.22	1	t	1	Pentstemon glaber	Andrena prunorum	21	0.00	0.02	0.00	0.03
1	0	0.27	1	t	1	Pentstemon glaber	Pompiloides sp.	21	0.00	0.02	0.01	0.02
0	0	0.23	1	t	1	Pentstemon glaber	Omalus sp.	21	0.00	0.02	0.00	0.02
1	0	0.21	1	cv	1	Pentstemon glaber	Pseudomasaris vespoides	21	0.00	0.02	0.00	0.03
1	0	0.22	1	t	1	Pentstemon glaber	bombus morrisoni	21	0.00	0.02	0.00	0.03

1	0	0.27	1	t	1	Pentstemon glaber	bombus edwardsi	21	0.00	0.02	0.01	0.03
1	0	0.21	1	t	1	Pentstemon glaber	Clisodon terminalis	21	0.00	0.02	0.00	0.03
0	0	0.22	1	cv	1	Pentstemon glaber	bombus kirbyellus	21	0.00	0.02	0.00	0.03
1	0	0.30	1	t	1	Pentstemon glaber	Halictus chloralictus	21	0.00	0.02	0.01	0.03
1	0	0.30	1	t	1	Pentstemon glaber	Andrena vicina	21	0.00	0.02	0.01	0.03
1	0	0.33	1	t	1	Pentstemon glaber	Andrena madronitens	21	0.00	0.02	0.02	0.03
0	0	0.23	1	cv	1	Pentstemon glaber	Papilio rutulus	21	0.00	0.02	0.00	0.02
1	0	0.23	1	t	1	Pentstemon glaber	Heriades gracilior	21	0.00	0.02	0.00	0.03
1	0	0.22	1	t	1	Pentstemon glaber	Osmia coloradella	21	0.00	0.02	0.00	0.03
1	0	0.22	1	t	1	Pentstemon glaber	Titusella pronitens	21	0.00	0.02	0.00	0.03
1	0	0.32	1	cv	1	Pentstemon glaber	Prosopis elliptica	21	0.00	0.02	0.02	0.03
0	1	0.59	1	t	1	Agathis vulgaris	Achillea millefolium	21	0.04	0.04	0.01	0.03
1	0	0.23	1	t	1	Prunus virginiana	Andrena crataegi	21	0.00	0.02	0.00	0.03
1	0	0.22	1	t	1	Prunus virginiana	Andrena sp.	21	0.00	0.02	0.00	0.03
0	0	0.22	1	cv	1	Prunus virginiana	Andrena prunorum	21	0.00	0.02	0.00	0.03
1	0	0.30	1	t	1	Prunus virginiana	Andrena vicina	21	0.00	0.02	0.01	0.03
0	0	0.23	1	t	1	Prunus virginiana	Nomada sp.	21	0.00	0.02	0.00	0.02
1	0	0.27	1	t	1	Prunus virginiana	Sphecodes sophiae	21	0.00	0.02	0.01	0.03
1	0	0.29	1	cv	1	Silene halli	Osmia hypoleuca	21	0.01	0.02	0.00	0.03
1	0	0.44	1	t	1	Halictus (Lasioglossum) sp.	Rubus strigosus	21	0.00	0.03	0.03	0.02
1	1	0.65	1	t	1	Halictus (Lasioglossum) sp.	Geranium caespitosum	21	0.00	0.03	0.05	0.02
1	0	0.23	1	t	1	Halictus (Lasioglossum) sp.	Monarda fistulosa	21	0.00	0.03	0.01	0.02
1	1	0.58	1	cv	1	Halictus (Lasioglossum) sp.	Chamaenerium angustifolium	21	0.00	0.03	0.04	0.02

1	0	0.28	1	t	1	Halictus (Lasioglossum) sp.	Castilleia miniata	21	0.00	0.03	0.01	0.02
1	0	0.22	1	t	1	Halictus (Lasioglossum) sp.	Rubus deliciosus	21	0.00	0.03	0.00	0.02
1	0	0.23	1	t	1	Halictus (Lasioglossum) sp.	Asclepias speciosa	21	0.00	0.03	0.00	0.02
1	0	0.34	1	cv	1	Oreochrysum parryi	Chrysophanus helloides	21	0.01	0.01	0.00	0.02
1	0	0.41	1	t	1	Ibidium	Halictus sp.	21	0.01	0.02	0.01	0.03
1	0	0.28	1	t	1	Ibidium strictum	Panurginus cressoniellus	21	0.01	0.02	0.00	0.03
1	1	0.50	1	t	1	Ibidium strictum	bombus appositus	21	0.01	0.02	0.02	0.03
1	0	0.30	1	cv	1	Ibidium strictum	bombus morrisoni	21	0.01	0.02	0.00	0.03
0	0	0.30	1	t	1	Ibidium strictum	Lygus pratensis	21	0.01	0.02	0.00	0.03
1	0	0.36	1	t	1	Sambucus racemosa	Leptura chrysocoma	21	0.01	0.01	0.00	0.01
1	0	0.23	1	t	1	Halictus lerouxi	Frasera speciosa	21	0.00	0.02	0.00	0.02
0	0	0.24	1	cv	1	Anthrax nigra	Rubus deliciosus	21	0.00	0.03	0.00	0.02
0	0	0.24	1	t	1	Melissodes fremonti	Monarda fistulosa	21	0.00	0.03	0.01	0.02
1	0	0.22	1	t	1	Dodocatheon meadia	Anthophora simillima	21	0.00	0.02	0.00	0.03
1	0	0.36	1	t	1	Dodocatheon meadia	bombus flavifrons	21	0.00	0.02	0.02	0.03
1	0	0.28	1	cv	1	Dodocatheon meadia	bombus edwardsi	21	0.00	0.02	0.01	0.03
1	0	0.26	1	t	1	Ctenucha sp.	Monarda fistulosa	21	0.01	0.03	0.01	0.02
1	1	0.62	1	t	1	Ctenucha sp.	Chamaenerium angustifolium	21	0.01	0.03	0.04	0.02
1	0	0.36	1	t	1	Prosopis sp.	Solidago missouriensis	21	0.00	0.03	0.02	0.02
1	0	0.32	1	cv	1	Anthophora simillima	Aragalus lamberti	21	0.00	0.03	0.01	0.01
1	0	0.27	1	t	1	Anthophora simillima	Penstemon unilateralis	21	0.00	0.03	0.01	0.02
1	0	0.26	1	t	1	Anthophora simillima	Penstemon halli	21	0.00	0.03	0.01	0.02
1	0	0.34	1	t	1	Anthophora simillima	Penstemon secundiflorus	21	0.00	0.03	0.02	0.02
1	0	0.45	1	cv	1	Anthophora simillima	Rubus strigosus	21	0.00	0.03	0.03	0.02

1	1	0.53	1	t	1	Anthophora simillima	Pentstemon gracilis	21	0.00	0.03	0.04	0.02
1	0	0.21	1	t	1	Anthophora simillima	Lithospermu m canescens	21	0.00	0.03	0.00	0.02
1	1	0.61	1	t	1	Anthophora simillima	Rosa acicularis	21	0.00	0.03	0.04	0.02
1	0	0.28	1	cv	1	Anthophora simillima	Capnoides aureum	21	0.00	0.03	0.01	0.02
1	0	0.42	1	t	1	Sieversia	Halictus sp.	21	0.01	0.02	0.01	0.03
1	0	0.37	1	t	1	Sieversia ciliata	bombus edwardsi	21	0.01	0.02	0.01	0.03
1	0	0.22	1	t	1	Andrena crataegi	Frasera speciosa	21	0.00	0.03	0.00	0.02
1	0	0.47	1	cv	1	Andrena crataegi	Rubus strigosus	21	0.00	0.03	0.03	0.02
1	0	0.25	1	t	1	Andrena crataegi	Monarda fistulosa	21	0.00	0.03	0.01	0.02
1	0	0.29	1	t	1	Andrena crataegi	Holodiscus dumosus	21	0.00	0.03	0.01	0.02
1	1	0.64	1	t	1	Andrena crataegi	Rosa acicularis	21	0.00	0.03	0.04	0.02
1	0	0.39	1	cv	1	Andrena crataegi	Opulaster opulifolius	21	0.00	0.03	0.02	0.02
1	0	0.46	1	t	1	Andrena crataegi	Carduus hookerianus	21	0.00	0.03	0.03	0.02
1	0	0.28	1	t	1	Petalostemon purpureus	bombus hunti	21	0.01	0.02	0.00	0.03
1	0	0.35	1	t	1	Petalostemon purpureus	bombus fervidus	21	0.01	0.02	0.01	0.03
1	0	0.40	1	cv	1	Petalostemon purpureus	bombus rufocinctus	21	0.01	0.02	0.01	0.03
1	0	0.29	1	t	1	Petalostemon purpureus	Apis mellifica	21	0.01	0.02	0.00	0.03
1	0	0.49	1	t	1	Petalostemon purpureus	bombus appositus	21	0.01	0.02	0.02	0.03
1	0	0.30	1	t	1	Petalostemon purpureus	Megachile pugnata	21	0.01	0.02	0.00	0.03
1	0	0.35	1	cv	1	Petalostemon purpureus	bombus edwardsi	21	0.01	0.02	0.01	0.03
1	0	0.27	1	t	1	Petalostemon purpureus	Clisodon terminalis	21	0.01	0.02	0.00	0.03
0	0	0.30	1	t	1	Petalostemon purpureus	Megachile sp.	21	0.01	0.02	0.00	0.03
1	0	0.27	1	t	1	Lathyrus ornatus	bombus edwardsi	21	0.00	0.02	0.01	0.03
1	0	0.30	1	cv	1	Apocynum androsaemifol ium	Prosopis varifrons	21	0.00	0.02	0.01	0.03
1	0	0.29	1	t	1	Apocynum androsaemifol ium	Halictus (Evylaeus) sp.	21	0.00	0.02	0.01	0.03

1	0	0.32	1	t	1	Apocynum androsaemifolium	Monumetha albifrons	21	0.00	0.02	0.02	0.03
1	0	0.32	1	t	1	Apocynum androsaemifolium	Prosopis elliptica	21	0.00	0.02	0.02	0.03
0	0	0.32	1	cv	1	Phorbia fusciceps	Holodiscus dumosus	21	0.01	0.03	0.01	0.02
1	0	0.21	1	t	1	Frasera speciosa	bombus hunti	21	0.00	0.02	0.00	0.03
1	0	0.21	1	t	1	Frasera speciosa	bombus juxtus	21	0.00	0.02	0.00	0.03
1	0	0.26	1	t	1	Frasera speciosa	Thyreocoris anthracina	21	0.00	0.02	0.00	0.02
1	0	0.21	1	cv	1	Frasera speciosa	Syrphus americanus	21	0.00	0.02	0.00	0.03
1	0	0.35	1	t	1	Frasera speciosa	bombus flavifrons	21	0.00	0.02	0.02	0.03
1	0	0.36	1	t	1	Frasera speciosa	Halictus pulzenus	21	0.00	0.02	0.02	0.03
1	0	0.32	1	t	1	Frasera speciosa	Monumetha albifrons	21	0.00	0.02	0.02	0.03
0	0	0.32	1	cv	1	Frasera speciosa	Halictus sp.	21	0.00	0.02	0.01	0.03
1	0	0.22	1	t	1	Frasera speciosa	Apis mellifica	21	0.00	0.02	0.00	0.03
1	0	0.27	1	t	1	Frasera speciosa	bombus edwardsi	21	0.00	0.02	0.01	0.03
1	0	0.21	1	t	1	Frasera speciosa	Clisodon terminalis	21	0.00	0.02	0.00	0.03
1	0	0.33	1	cv	1	Frasera speciosa	Andrena madronitens	21	0.00	0.02	0.02	0.03
1	1	0.52	1	t	1	Agapostemon coloradensis	Stanleya pinnatifida	21	0.02	0.02	0.02	0.02
1	0	0.37	1	t	1	Agapostemon coloradensis	Taraxacum officinale	21	0.02	0.02	0.00	0.02
1	1	0.66	1	t	1	Foenus perplexus	Geranium caespitosum	21	0.00	0.03	0.05	0.02
1	0	0.29	1	cv	1	Celerio lineata	Pachylophus caespitosus	21	0.00	0.02	0.00	0.00
0	0	0.29	1	t	1	Colias keewaydina	Castilleia miniata	21	0.00	0.03	0.01	0.02
1	0	0.21	1	t	1	Psithyrus insularis	Aquilegia coerulea	21	0.00	0.03	0.00	0.02
1	0	0.25	1	t	1	Psithyrus insularis	Dasyphora fruticosa	21	0.00	0.03	0.01	0.02
1	1	0.65	1	cv	1	Psithyrus insularis	Geranium caespitosum	21	0.00	0.03	0.05	0.02
1	0	0.20	1	t	1	Psithyrus insularis	Gentiana parryi	21	0.00	0.03	0.00	0.02

1	1	0.58	1	t	1	Psithyrus insularis	Chamaenerium angustifolium	21	0.00	0.03	0.04	0.02
1	0	0.43	1	t	1	Psithyrus insularis	Carduus hookerianus	21	0.00	0.03	0.03	0.02
1	0	0.22	1	cv	1	Thermopsis montana	bombus hunti	21	0.00	0.02	0.00	0.03
1	0	0.22	1	t	1	Thermopsis montana	bombus juxtus	21	0.00	0.02	0.00	0.03
1	0	0.26	1	t	1	Thermopsis montana	bombus americanorum	21	0.00	0.02	0.00	0.02
1	0	0.25	1	t	1	Thermopsis montana	Osmia sp.	21	0.00	0.02	0.01	0.03
1	0	0.22	1	cv	1	Thermopsis montana	Megachile wootoni	21	0.00	0.02	0.00	0.03
1	0	0.37	1	t	1	Andrena edwiniae	Jamesia americana	21	0.01	0.02	0.01	0.02
0	0	0.22	1	t	1	Allium recurvatum	Parnassius smintheus	21	0.00	0.02	0.00	0.04
1	0	0.27	1	t	1	Allium recurvatum	bombus edwardsi	21	0.00	0.02	0.01	0.03
1	0	0.22	1	cv	1	Aquilegia coerulea	bombus hunti	21	0.00	0.02	0.00	0.03
1	0	0.22	1	t	1	Aquilegia coerulea	Clisodon terminalis	21	0.00	0.02	0.00	0.03
1	0	0.49	1	t	1	Colias scudderi	Carduus hookerianus	21	0.01	0.03	0.03	0.02
1	0	0.24	1	t	1	bombus hunti	Pedicularis parryi	21	0.00	0.03	0.00	0.01
1	0	0.33	1	cv	1	bombus hunti	Solidago missouriensis	21	0.00	0.03	0.02	0.02
1	0	0.21	1	t	1	bombus hunti	Aquilegia brevistyla	21	0.00	0.03	0.00	0.02
1	0	0.40	1	t	1	bombus hunti	Erigeron macranthus	21	0.00	0.03	0.02	0.02
1	0	0.30	1	t	1	bombus hunti	Jamesia americana	21	0.00	0.03	0.01	0.02
1	0	0.26	1	cv	1	bombus hunti	Petalostemon candidus	21	0.00	0.03	0.01	0.02
1	0	0.45	1	t	1	bombus hunti	Rubus strigosus	21	0.00	0.03	0.03	0.02
1	1	0.53	1	t	1	bombus hunti	Pentstemon gracilis	21	0.00	0.03	0.04	0.02
1	0	0.21	1	t	1	bombus hunti	Sedum stenopetalum	21	0.00	0.03	0.00	0.02
1	0	0.30	1	cv	1	bombus hunti	Astragalus drummondi	21	0.00	0.03	0.01	0.02
1	0	0.21	1	t	1	bombus hunti	Helianthus petiolaris	21	0.00	0.03	0.00	0.02

1	0	0.23	1	t	1	bombus hunti	Trifolium dasyphyllum	21	0.00	0.03	0.00	0.02
1	1	0.66	1	t	1	bombus hunti	Geranium caespitosum	21	0.00	0.03	0.05	0.02
1	0	0.20	1	cv	1	bombus hunti	Geranium richardsoni	21	0.00	0.03	0.00	0.02
1	0	0.23	1	t	1	bombus hunti	Monarda fistulosa	21	0.00	0.03	0.01	0.02
1	0	0.27	1	t	1	bombus hunti	Holodiscus dumosus	21	0.00	0.03	0.01	0.02
1	1	0.59	1	t	1	bombus hunti	Chamaeneriu m angustifolium	21	0.00	0.03	0.04	0.02
1	1	0.61	1	cv	1	bombus hunti	Rosa acicularis	21	0.00	0.03	0.04	0.02
1	0	0.33	1	t	1	bombus hunti	Stanleya pinnatifida	21	0.00	0.03	0.02	0.02
1	0	0.28	1	t	1	bombus hunti	Rydbergia grandilfora	21	0.00	0.03	0.01	0.02
1	0	0.29	1	t	1	bombus hunti	Castilleia miniata	21	0.00	0.03	0.01	0.02
1	0	0.21	1	cv	1	bombus hunti	Erigeron uniflorus	21	0.00	0.03	0.00	0.02
1	0	0.44	1	t	1	bombus hunti	Carduus hookerianus	21	0.00	0.03	0.03	0.02
1	0	0.23	1	t	1	bombus hunti	Rubus deliciosus	21	0.00	0.03	0.00	0.02
0	1	0.51	1	t	1	Chrysophanus sirius	Monarda fistulosa	21	0.03	0.03	0.01	0.02
1	0	0.20	1	cv	1	bombus juxtus	Potentilla arguta	21	0.00	0.03	0.00	0.02
1	0	0.22	1	t	1	bombus juxtus	Onagra biennis	21	0.00	0.03	0.00	0.02
1	0	0.27	1	t	1	bombus juxtus	Penstemon unilateralis	21	0.00	0.03	0.01	0.02
1	0	0.34	1	t	1	bombus juxtus	Penstemon secundiflorus	21	0.00	0.03	0.02	0.02
1	0	0.30	1	cv	1	bombus juxtus	Jamesia americana	21	0.00	0.03	0.01	0.02
1	0	0.44	1	t	1	bombus juxtus	Rubus strigosus	21	0.00	0.03	0.03	0.02
1	1	0.53	1	t	1	bombus juxtus	Pentstemon gracilis	21	0.00	0.03	0.04	0.02
1	0	0.30	1	t	1	bombus juxtus	Astragalus drummondi	21	0.00	0.03	0.01	0.02
1	0	0.30	1	cv	1	bombus juxtus	Pentstemon glaucus	21	0.00	0.03	0.01	0.02
1	1	0.66	1	t	1	bombus juxtus	Geranium caespitosum	21	0.00	0.03	0.05	0.02
1	0	0.22	1	t	1	bombus juxtus	Potentilla gracilis	21	0.00	0.03	0.00	0.02

1	0	0.20	1	t	1	bombus juxtus	Delphinium scopulorum	21	0.00	0.03	0.00	0.02
1	0	0.20	1	cv	1	bombus juxtus	Chrysopsis villosa	21	0.00	0.03	0.00	0.02
1	0	0.21	1	t	1	bombus juxtus	Aconitum columbianum	21	0.00	0.03	0.00	0.02
1	0	0.50	1	t	1	bombus juxtus	Mentzelia multiflora	21	0.00	0.03	0.03	0.03
1	0	0.27	1	t	1	bombus juxtus	Holodiscus dumosus	21	0.00	0.03	0.01	0.02
1	0	0.20	1	cv	1	bombus juxtus	Gentiana parryi	21	0.00	0.03	0.00	0.02
1	1	0.58	1	t	1	bombus juxtus	Chamaeneriu m angustifolium	21	0.00	0.03	0.04	0.02
1	1	0.61	1	t	1	bombus juxtus	Rosa acicularis	21	0.00	0.03	0.04	0.02
1	0	0.27	1	t	1	bombus juxtus	Capnoides aureum	21	0.00	0.03	0.01	0.02
1	0	0.45	1	cv	1	bombus juxtus	Mertensia sibirica	21	0.00	0.03	0.03	0.02
1	0	0.44	1	t	1	bombus juxtus	Carduus hookerianus	21	0.00	0.03	0.03	0.02
1	0	0.23	1	t	1	bombus juxtus	Rubus deliciosus	21	0.00	0.03	0.00	0.02
1	1	0.58	1	t	1	Andrena imitatrix	Astragalus drummondi	21	0.03	0.02	0.01	0.02
1	0	0.21	1	cv	1	Tradescantia virginiana	Syrphus americanus	21	0.00	0.02	0.00	0.03
1	0	0.26	1	t	1	Tradescantia virginiana	Anthrax sinuosa	21	0.00	0.02	0.00	0.02
1	0	0.30	1	t	1	Tradescantia virginiana	Halictus (Evylaeus) sp.	21	0.00	0.02	0.01	0.03
1	0	0.23	1	t	1	Tradescantia virginiana	Apis mellifica	21	0.00	0.02	0.00	0.03
1	0	0.22	1	cv	1	Tradescantia virginiana	Phormia regina	21	0.00	0.02	0.00	0.03
1	0	0.29	1	t	1	Xylota flavitibia	Holodiscus dumosus	21	0.00	0.03	0.01	0.02
1	1	0.63	1	t	1	Xylota flavitibia	Rosa acicularis	21	0.00	0.03	0.04	0.02
1	0	0.24	1	t	1	Xylota flavitibia	Rubus deliciosus	21	0.00	0.03	0.00	0.02
1	1	0.64	1	cv	1	Epeolus helianthi	Geranium caespitosum	21	0.00	0.03	0.05	0.02
1	0	0.37	1	t	1	Prosopis varifrons	Penstemon unilateralis	21	0.01	0.03	0.01	0.02
1	1	0.56	1	t	1	Prosopis varifrons	Rubus strigosus	21	0.01	0.03	0.03	0.02
1	1	0.64	1	t	1	Prosopis varifrons	Pentstemon gracilis	21	0.01	0.03	0.04	0.02

1	1	0.75	1	cv	1	Prosopis varifrons	Geranium caespitosum	21	0.01	0.03	0.05	0.02
1	0	0.29	1	t	1	Prosopis varifrons	Geranium richardsoni	21	0.01	0.03	0.00	0.02
1	0	0.37	1	t	1	Prosopis varifrons	Holodiscus dumosus	21	0.01	0.03	0.01	0.02
1	1	0.72	1	t	1	Prosopis varifrons	Rosa acicularis	21	0.01	0.03	0.04	0.02
1	0	0.47	1	cv	1	Prosopis varifrons	Opulaster opulifolius	21	0.01	0.03	0.02	0.02
1	0	0.32	1	t	1	Prosopis varifrons	Rubus deliciosus	21	0.01	0.03	0.00	0.02
0	0	0.22	1	t	1	Potentilla arguta	bombus kirbyellus	21	0.00	0.02	0.00	0.03
1	0	0.29	1	t	1	Potentilla arguta	Andrena vicina	21	0.00	0.02	0.01	0.03
1	0	0.27	1	cv	1	Potentilla arguta	Sphecodes sophiae	21	0.00	0.02	0.01	0.03
0	0	0.33	1	t	1	Selasphorus platycercus	Penstemon secundiflorus	21	0.00	0.04	0.02	0.02
0	1	0.52	1	t	1	Selasphorus platycercus	Pentstemon gracilis	21	0.00	0.04	0.04	0.02
0	0	0.23	1	t	1	Selasphorus platycercus	Monarda fistulosa	21	0.00	0.04	0.01	0.02
0	1	0.60	1	cv	1	Selasphorus platycercus	Iris missouriensis	21	0.00	0.04	0.05	0.04
0	0	0.44	1	t	1	Selasphorus platycercus	Mertensia sibirica	21	0.00	0.04	0.03	0.02
0	0	0.28	1	t	1	Selasphorus platycercus	Castilleia miniata	21	0.00	0.04	0.01	0.02
0	0	0.28	1	t	1	Melitaea whitneyi	Rydbergia grandilfora	21	0.00	0.03	0.01	0.02
1	0	0.27	1	cv	1	Thalictrum fendleri	Halictus fendleri	21	0.00	0.02	0.00	0.02
1	0	0.35	1	t	1	Thalictrum fendleri	Halictus sp.	21	0.00	0.02	0.01	0.03
1	0	0.25	1	t	1	Thalictrum fendleri	Apis mellifica	21	0.00	0.02	0.00	0.03
1	0	0.33	1	t	1	Nathalis iole	Potentilla pulcherrima	21	0.00	0.03	0.02	0.02
1	0	0.33	1	cv	1	Osmia rohweri	Mertensia pratensis	21	0.00	0.02	0.01	0.02
0	1	0.56	1	t	1	Osmia bella	Iris missouriensis	21	0.00	0.04	0.05	0.04
1	1	0.58	1	t	1	bombus centralis	Pentstemon gracilis	21	0.01	0.03	0.04	0.02
1	0	0.24	1	t	1	bombus centralis	Delphinium scopulorum	21	0.01	0.03	0.00	0.02
1	0	0.27	1	cv	1	bombus centralis	Monarda fistulosa	21	0.01	0.03	0.01	0.02

1	1	0.64	1	t	1	bombus centralis	Chamaenerium angustifolium	21	0.01	0.03	0.04	0.02
1	1	0.63	1	t	1	Andrena sp.	Rosa acicularis	21	0.00	0.03	0.04	0.02
1	0	0.28	1	t	1	Thyreocoris anthracina	Physaria didymocarpa	21	0.00	0.02	0.00	0.02
1	0	0.26	1	cv	1	Pedicularis parryi	bombus kirbyellus	21	0.00	0.01	0.00	0.03
1	0	0.41	1	t	1	Andrena apacheorum	Erigeron macranthus	21	0.00	0.03	0.02	0.02
1	1	0.67	1	t	1	Andrena apacheorum	Geranium caespitosum	21	0.00	0.03	0.05	0.02
1	0	0.33	1	t	1	Osmia brevis	Mertensia pratensis	21	0.00	0.02	0.01	0.02
1	0	0.37	1	cv	1	Solidago missouriensis	Epalpus bicolor	21	0.02	0.02	0.00	0.03
1	0	0.34	1	t	1	Solidago missouriensis	Colletes americanus	21	0.02	0.02	0.00	0.03
1	0	0.34	1	t	1	Solidago missouriensis	Anthrax halycon	21	0.02	0.02	0.00	0.03
1	1	0.50	1	t	1	Solidago missouriensis	bombus flavifrons	21	0.02	0.02	0.02	0.03
1	0	0.46	1	cv	1	Solidago missouriensis	bombus rufocinctus	21	0.02	0.02	0.01	0.03
1	0	0.34	1	t	1	Solidago missouriensis	Mordella melaena	21	0.02	0.02	0.00	0.03
1	0	0.46	1	t	1	Solidago missouriensis	Halictus sp.	21	0.02	0.02	0.01	0.03
1	0	0.35	1	t	1	Solidago missouriensis	Apis mellifica	21	0.02	0.02	0.00	0.03
1	0	0.33	1	cv	1	Solidago missouriensis	Colletes oromontis	21	0.02	0.02	0.00	0.03
0	0	0.35	1	t	1	Solidago missouriensis	Psithyrus consultus	21	0.02	0.02	0.00	0.03
1	0	0.41	1	t	1	Solidago missouriensis	bombus edwardsi	21	0.02	0.02	0.01	0.03
1	0	0.30	1	t	1	Epalpus bicolor	Polygonum bistorta	21	0.00	0.03	0.01	0.02
1	0	0.48	1	cv	1	Epalpus bicolor	Carduus hookerianus	21	0.00	0.03	0.03	0.02
1	0	0.28	1	t	1	Brenthis triclaris	Dasyphora fruticosa	21	0.00	0.03	0.01	0.02
1	1	0.58	1	t	1	Monumetha argentifrons	Chamaenerium angustifolium	21	0.00	0.03	0.04	0.02
1	1	0.60	1	t	1	Mallota flavoterminata	Rosa acicularis	21	0.00	0.03	0.04	0.02
1	1	0.60	1	cv	1	Bombylius atriceps	Rosa acicularis	21	0.00	0.03	0.04	0.02

0	0	0.24	1	t	1	Onagra biennis	Megachile sp.	21	0.00	0.02	0.00	0.03
1	0	0.35	1	t	1	Onagra biennis	Andrena madronitens	21	0.00	0.02	0.02	0.03
0	0	0.24	1	t	1	Pipiza vanduzeei	Rubus deliciosus	21	0.00	0.03	0.00	0.02
1	0	0.32	1	cv	1	Chrysotoxum upsilon	Polygonum bistorta	21	0.00	0.01	0.01	0.02
1	0	0.26	1	t	1	Galium boreale	Lycaena glaucon	21	0.00	0.02	0.00	0.02
1	0	0.27	1	t	1	Vespa germanica	Penstemon unilateralis	21	0.00	0.03	0.01	0.02
1	0	0.30	1	t	1	Vespa germanica	Jamesia americana	21	0.00	0.03	0.01	0.02
1	0	0.23	1	cv	1	Vespa germanica	Monarda fistulosa	21	0.00	0.03	0.01	0.02
1	1	0.59	1	t	1	Vespa germanica	Chamaeneriu m angustifolium	21	0.00	0.03	0.04	0.02
1	0	0.45	1	t	1	Vespa germanica	Mertensia sibirica	21	0.00	0.03	0.03	0.02
1	0	0.36	1	t	1	Vespa germanica	Opulaster opulifolius	21	0.00	0.03	0.02	0.02
1	0	0.44	1	cv	1	Syrphus americanus	Rubus strigosus	21	0.00	0.03	0.03	0.02
1	1	0.52	1	t	1	Syrphus americanus	Pentstemon gracilis	21	0.00	0.03	0.04	0.02
1	1	0.65	1	t	1	Syrphus americanus	Geranium caespitosum	21	0.00	0.03	0.05	0.02
1	1	0.58	1	t	1	Syrphus americanus	Chamaeneriu m angustifolium	21	0.00	0.03	0.04	0.02
1	1	0.61	1	cv	1	Syrphus americanus	Rosa acicularis	21	0.00	0.03	0.04	0.02
1	0	0.22	1	t	1	Syrphus americanus	Rubus deliciosus	21	0.00	0.03	0.00	0.02
1	0	0.37	1	t	1	Agapostemon sp.	Stanleya pinnatifida	21	0.00	0.02	0.02	0.02
0	0	0.26	1	t	1	Brachyopa notata	Rubus deliciosus	21	0.00	0.02	0.00	0.02
1	0	0.29	1	cv	1	Dasyllis fernaldi	Calochortus gunnisoni	21	0.00	0.03	0.01	0.02
1	1	0.60	1	t	1	Dasyllis fernaldi	Chamaeneriu m angustifolium	21	0.00	0.03	0.04	0.02
0	0	0.24	1	t	1	Anthrax hypomelas	Potentilla gracilis	21	0.00	0.03	0.00	0.02
1	0	0.27	1	t	1	Prunus pennsylvanica	Sphecodes sophiae	21	0.00	0.02	0.01	0.03
0	0	0.22	1	cv	1	Valeriana	Epalpus rufus	21	0.00	0.02	0.00	0.03

1	0	0.26	1	t	1	Valeriana edulis	Sphecodes sophiae	21	0.00	0.02	0.01	0.03
1	0	0.26	1	t	1	Syrphus nitens	Rubus deliciosus	21	0.00	0.02	0.00	0.02
1	0	0.22	1	t	1	Arctostaphylus uva-ursi	Apis mellifica	21	0.00	0.02	0.00	0.03
1	0	0.27	1	cv	1	Arctostaphylus uva-ursi	bombus edwardsi	21	0.00	0.02	0.01	0.03
0	0	0.26	1	t	1	Halicyoides mamus	Achillea millefolium	21	0.01	0.03	0.01	0.03
1	0	0.50	1	t	1	Colias edwardsi	Carduus hookerianus	21	0.01	0.03	0.03	0.02
1	0	0.32	1	t	1	Halictus medionitens	Potentilla pulcherrima	21	0.00	0.03	0.02	0.02
1	1	0.66	1	cv	1	Halictus medionitens	Geranium caespitosum	21	0.00	0.03	0.05	0.02
1	0	0.27	1	t	1	Halictus medionitens	Holodiscus dumosus	21	0.00	0.03	0.01	0.02
1	1	0.62	1	t	1	Halictus medionitens	Rosa acicularis	21	0.00	0.03	0.04	0.02
1	0	0.23	1	t	1	Halictus medionitens	Rubus deliciosus	21	0.00	0.03	0.00	0.02
1	0	0.29	1	cv	1	Chrysotoxum integrum	Holodiscus dumosus	21	0.00	0.03	0.01	0.02
1	1	0.63	1	t	1	Chrysotoxum integrum	Rosa acicularis	21	0.00	0.03	0.04	0.02
1	0	0.24	1	t	1	Halictus manitonellus	Monarda fistulosa	21	0.00	0.03	0.01	0.02
1	1	0.62	1	t	1	Halictus manitonellus	Rosa acicularis	21	0.00	0.03	0.04	0.02
1	1	0.56	1	cv	1	Bombomelecta fulvida	Pentstemon gracilis	21	0.00	0.03	0.04	0.02
1	0	0.48	1	t	1	Bombomelecta fulvida	Mertensia sibirica	21	0.00	0.03	0.03	0.02
0	0	0.34	1	t	1	Penstemon unilateralis	bombus fervidus	21	0.01	0.02	0.01	0.03
1	0	0.32	1	t	1	Penstemon unilateralis	bombus americanorum	21	0.01	0.02	0.00	0.02
1	0	0.28	1	cv	1	Penstemon unilateralis	Apis mellifica	21	0.01	0.02	0.00	0.03
1	0	0.22	1	t	1	Lemonias nubigena wheeleri	Helianthus petiolaris	21	0.00	0.03	0.00	0.02
1	1	0.67	1	t	1	Lemonias nubigena wheeleri	Geranium caespitosum	21	0.00	0.03	0.05	0.02
1	0	0.29	1	t	1	Lemonias nubigena wheeleri	Rydbergia grandilfora	21	0.00	0.03	0.01	0.02

0	0	0.24	1	cv	1	Prosopis cressoni	Rubus deliciosus	21	0.00	0.03	0.00	0.02
1	0	0.39	1	t	1	Osmia pentstemonis	Penstemon secundiflorus	21	0.01	0.03	0.02	0.02
1	1	0.59	1	t	1	Osmia pentstemonis	Pentstemon gracilis	21	0.01	0.03	0.04	0.02
1	0	0.36	1	t	1	Osmia pentstemonis	Pentstemon glaucus	21	0.01	0.03	0.01	0.02
1	1	0.51	1	cv	1	Osmia pentstemonis	Mertensia sibirica	21	0.01	0.03	0.03	0.02
1	0	0.35	1	t	1	Osmia pentstemonis	Mertensia pratensis	21	0.01	0.03	0.01	0.02
1	0	0.47	1	t	1	Eristalis arbustorum	Rubus strigosus	21	0.00	0.03	0.03	0.02
1	0	0.33	1	t	1	bombus fervidus	Petalostemon candidus	21	0.01	0.03	0.01	0.02
1	0	0.37	1	cv	1	bombus fervidus	Astragalus drummondi	21	0.01	0.03	0.01	0.02
1	0	0.30	1	t	1	bombus fervidus	Monarda fistulosa	21	0.01	0.03	0.01	0.02
0	0	0.29	1	t	1	bombus fervidus	Rubus deliciosus	21	0.01	0.03	0.00	0.02
1	0	0.39	1	t	1	Syrphus opinator	Penstemon secundiflorus	21	0.01	0.03	0.02	0.02
1	1	0.59	1	cv	1	Syrphus opinator	Pentstemon gracilis	21	0.01	0.03	0.04	0.02
1	1	0.71	1	t	1	Syrphus opinator	Geranium caespitosum	21	0.01	0.03	0.05	0.02
1	0	0.24	1	t	1	Syrphus opinator	Gentiana parryi	21	0.01	0.03	0.00	0.02
1	1	0.67	1	t	1	Syrphus opinator	Rosa acicularis	21	0.01	0.03	0.04	0.02
1	1	0.64	1	cv	1	Protothyreopus dilectus	Geranium caespitosum	21	0.00	0.03	0.05	0.02
1	1	0.62	1	t	1	Colletes americanus	Rosa acicularis	21	0.00	0.03	0.04	0.02
1	1	0.58	1	t	1	Solenius sp.	Chamaenerium angustifolium	21	0.00	0.03	0.04	0.02
1	1	0.64	1	t	1	Apanteles sp.	Geranium caespitosum	21	0.00	0.03	0.05	0.02
1	0	0.33	1	cv	1	Cleome serrulata	bombus edwardsi	21	0.01	0.02	0.01	0.03
1	0	0.35	1	t	1	Penstemon	Osmia densa	21	0.01	0.02	0.01	0.03
1	0	0.43	1	t	1	Penstemon halli	Halictus pulzenus	21	0.01	0.02	0.02	0.03
1	0	0.39	1	t	1	Penstemon halli	Monumetha albifrons	21	0.01	0.02	0.02	0.03
1	0	0.25	1	cv	1	Penstemon halli	Osmia phaceliae	21	0.01	0.02	0.00	0.03

1	0	0.25	1	t	1	Penstemon halli	Prosopis basalis	21	0.01	0.02	0.00	0.03
1	0	0.44	1	t	1	Vespa occidentalis	Erigeron macranthus	21	0.00	0.03	0.02	0.02
1	0	0.27	1	t	1	Vespa occidentalis	Linaria vulgaris	21	0.00	0.03	0.01	0.02
1	0	0.42	1	cv	1	Anthrax tegminipennis	Erigeron macranthus	21	0.00	0.02	0.02	0.02
1	0	0.45	1	t	1	Scaba opinator	Mertensia sibirica	21	0.00	0.03	0.03	0.02
1	0	0.24	1	t	1	bombus nevadensis	Delphinium scopulorum	21	0.01	0.03	0.00	0.02
1	0	0.24	1	t	1	bombus nevadensis	Gentiana parryi	21	0.01	0.03	0.00	0.02
1	1	0.64	1	cv	1	bombus nevadensis	Chamaeneriu m angustifolium	21	0.01	0.03	0.04	0.02
1	0	0.41	1	t	1	bombus nevadensis	Opulaster opulifolius	21	0.01	0.03	0.02	0.02
1	0	0.49	1	t	1	bombus nevadensis	Carduus hookerianus	21	0.01	0.03	0.03	0.02
1	1	0.51	1	t	1	Penstemon secundiflorus	Halictus pulzenus	21	0.02	0.02	0.02	0.03
1	0	0.38	1	cv	1	Penstemon secundiflorus	Argynnis atlantis	21	0.02	0.02	0.01	0.03
0	0	0.34	1	t	1	Penstemon secundiflorus	Parnassius smintheus	21	0.02	0.02	0.00	0.04
1	0	0.33	1	t	1	Penstemon secundiflorus	Prosopis basalis	21	0.02	0.02	0.00	0.03
1	0	0.41	1	t	1	Penstemon secundiflorus	bombus edwardsi	21	0.02	0.02	0.01	0.03
1	0	0.33	1	cv	1	Penstemon secundiflorus	Clisodon terminalis	21	0.02	0.02	0.00	0.03
1	0	0.48	1	t	1	Penstemon secundiflorus	Andrena madronitens	21	0.02	0.02	0.02	0.03
1	0	0.47	1	t	1	Penstemon secundiflorus	Prosopis elliptica	21	0.02	0.02	0.02	0.03
1	0	0.34	1	t	1	Silene acaulis	Exoprosopa volucris	21	0.00	0.00	0.00	0.00
1	0	0.36	1	cv	1	Aster foliaceus	bombus flavifrons	21	0.00	0.02	0.02	0.03
1	0	0.28	1	t	1	Aster foliaceus	bombus edwardsi	21	0.00	0.02	0.01	0.03
1	0	0.39	1	t	1	Archytas sp.	Geum rivale	21	0.00	0.01	0.01	0.01
1	0	0.44	1	t	1	Calochortus gunnisoni	bombus flavifrons	21	0.01	0.02	0.02	0.03
1	0	0.40	1	cv	1	Calochortus gunnisoni	bombus rufocinctus	21	0.01	0.02	0.01	0.03
1	0	0.27	1	t	1	Calochortus gunnisoni	Pseudomasar is vespoides	21	0.01	0.02	0.00	0.03

1	0	0.35	1	t	1	Calochortus gunnisoni	bombus edwardsi	21	0.01	0.02	0.01	0.03
0	0	0.28	1	t	1	Calochortus gunnisoni	Curtipogon leucozona	21	0.01	0.02	0.00	0.03
1	1	0.72	1	cv	1	Lycaena pseudargiolus	Geranium caespitosum	21	0.01	0.03	0.05	0.02
0	0	0.22	1	t	1	Drymocallis fissa	Parnassius smintheus	21	0.00	0.02	0.00	0.04
1	0	0.21	1	t	1	Drymocallis fissa	Irbisia brachycerus	21	0.00	0.02	0.00	0.03
1	0	0.28	1	t	1	Drymocallis fissa	bombus edwardsi	21	0.00	0.02	0.01	0.03
1	0	0.41	1	cv	1	Erigeron macranthus	Peleteria robusta	21	0.02	0.02	0.00	0.02
1	0	0.44	1	t	1	Erigeron macranthus	Argynnis atlantis	21	0.02	0.02	0.01	0.03
1	0	0.44	1	t	1	Erigeron macranthus	Chrysophanus helloides	21	0.02	0.02	0.00	0.02
1	0	0.41	1	t	1	Erigeron macranthus	Bracon helena	21	0.02	0.02	0.00	0.02
1	0	0.42	1	cv	1	Erigeron macranthus	Panzeria radicum	21	0.02	0.02	0.00	0.03
1	0	0.39	1	t	1	Erigeron macranthus	Panurginus cressoniellus	21	0.02	0.02	0.00	0.03
1	0	0.44	1	t	1	Erigeron macranthus	Tenthredella unicincta	21	0.02	0.02	0.00	0.02
1	0	0.42	1	t	1	Erigeron macranthus	Megachile pugnata	21	0.02	0.02	0.00	0.03
1	0	0.40	1	cv	1	Erigeron macranthus	Parnassius clodius	21	0.02	0.02	0.00	0.03
1	0	0.47	1	t	1	Erigeron macranthus	bombus edwardsi	21	0.02	0.02	0.01	0.03
1	0	0.43	1	t	1	Erigeron macranthus	Brenthis helena	21	0.02	0.02	0.00	0.02
1	0	0.41	1	t	1	Erigeron macranthus	Anthrax fulviana	21	0.02	0.02	0.00	0.02
1	0	0.44	1	cv	1	Erigeron macranthus	Coenonympha pamphiloides	21	0.02	0.02	0.00	0.03
1	1	0.54	1	t	1	Erigeron macranthus	Prosopis elliptica	21	0.02	0.02	0.02	0.03
1	0	0.40	1	t	1	bombus flavifrons	Dasyphora fruticosa	21	0.02	0.03	0.01	0.02
1	1	0.61	1	t	1	bombus flavifrons	Rubus strigosus	21	0.02	0.03	0.03	0.02
1	0	0.34	1	cv	1	bombus flavifrons	Delphinium scopulorum	21	0.02	0.03	0.00	0.02
1	0	0.38	1	t	1	bombus flavifrons	Monarda fistulosa	21	0.02	0.03	0.01	0.02
1	0	0.35	1	t	1	bombus flavifrons	Aconitum columbianum	21	0.02	0.03	0.00	0.02

1	1	0.74	1	t	1	bombus flavifrons	Chamaenerium angustifolium	21	0.02	0.03	0.04	0.02
1	1	0.76	1	cv	1	bombus flavifrons	Rosa acicularis	21	0.02	0.03	0.04	0.02
1	1	0.62	1	t	1	bombus flavifrons	Mertensia sibirica	21	0.02	0.03	0.03	0.02
1	0	0.43	1	t	1	bombus flavifrons	Rydbergia grandilfora	21	0.02	0.03	0.01	0.02
1	0	0.37	1	t	1	bombus flavifrons	Rubus deliciosus	21	0.02	0.03	0.00	0.02
1	1	0.62	1	cv	1	Osmia densa	Pentstemon gracilis	21	0.01	0.03	0.04	0.02
1	1	0.74	1	t	1	Osmia densa	Geranium caespitosum	21	0.01	0.03	0.05	0.02
1	1	0.70	1	t	1	Osmia densa	Rosa acicularis	21	0.01	0.03	0.04	0.02
1	0	0.36	1	t	1	Osmia densa	Capnoides aureum	21	0.01	0.03	0.01	0.02
1	1	0.55	1	cv	1	Osmia densa	Mertensia sibirica	21	0.01	0.03	0.03	0.02
1	0	0.39	1	t	1	Osmia densa	Mertensia pratensis	21	0.01	0.03	0.01	0.02
1	0	0.45	1	t	1	Osmia densa	Opulaster opulifolius	21	0.01	0.03	0.02	0.02
1	0	0.48	1	t	1	Volucella rufomaculata	Rubus strigosus	21	0.00	0.03	0.03	0.02
1	0	0.32	1	cv	1	Volucella rufomaculata	Castilleia miniata	21	0.00	0.03	0.01	0.02
1	0	0.25	1	t	1	Volucella rufomaculata	Rubus deliciosus	21	0.00	0.03	0.00	0.02
1	0	0.32	1	t	1	Potentilla pulcherrima	Colias alexandra	21	0.02	0.02	0.00	0.03
1	0	0.32	1	t	1	Potentilla pulcherrima	Chrysotoxum ventricosum	21	0.02	0.02	0.00	0.03
1	0	0.34	1	cv	1	Potentilla pulcherrima	Erebia epipsodea	21	0.02	0.02	0.01	0.03
1	0	0.44	1	t	1	Potentilla pulcherrima	Halictus sp.	21	0.02	0.02	0.01	0.03
0	0	0.29	1	t	1	Potentilla pulcherrima	Chionobis macouni	21	0.02	0.02	0.00	0.04
1	0	0.37	1	t	1	Potentilla pulcherrima	Mesogramma marginata	21	0.02	0.02	0.01	0.02
0	0	0.31	1	cv	1	Potentilla pulcherrima	Parnassius smintheus	21	0.02	0.02	0.00	0.04
1	0	0.32	1	t	1	Potentilla pulcherrima	bombus kirbyellus	21	0.02	0.02	0.00	0.03
1	0	0.32	1	t	1	Potentilla pulcherrima	Geocoris bullatus	21	0.02	0.02	0.00	0.03
1	0	0.28	1	t	1	Dasyphora fruticosa	Erebia epipsodea	21	0.01	0.02	0.01	0.03

1	0	0.29	1	cv	1	Dasyphora fruticosa	Coenonympha pamphiloides	21	0.01	0.02	0.00	0.03
1	0	0.38	1	t	1	Dasyphora fruticosa	Prosopis elliptica	21	0.01	0.02	0.02	0.03
1	0	0.25	1	t	1	Anthophora smithi	Monarda fistulosa	21	0.00	0.03	0.01	0.02
1	1	0.61	1	t	1	Anthophora smithi	Chamaenerium angustifolium	21	0.00	0.03	0.04	0.02
1	0	0.34	1	cv	1	Jamesia americana	Ancistrocerus sp 2.	21	0.01	0.02	0.01	0.03
0	0	0.34	1	t	1	Jamesia americana	Eristalis tenax	21	0.01	0.02	0.00	0.03
1	0	0.31	1	t	1	Jamesia americana	Apis mellifica	21	0.01	0.02	0.00	0.03
1	0	0.29	1	t	1	Jamesia americana	Irbisia brachycerus	21	0.01	0.02	0.00	0.03
1	0	0.37	1	cv	1	Jamesia americana	bombus edwardsi	21	0.01	0.02	0.01	0.03
1	0	0.34	1	t	1	Petalostemon candidus	Sterictophora sp.	21	0.01	0.02	0.01	0.02
1	0	0.38	1	t	1	Petalostemon candidus	bombus rufocinctus	21	0.01	0.02	0.01	0.03
1	0	0.28	1	t	1	Petalostemon candidus	Apis mellifica	21	0.01	0.02	0.00	0.03
1	0	0.48	1	cv	1	Petalostemon candidus	bombus appositus	21	0.01	0.02	0.02	0.03
1	0	0.33	1	t	1	Petalostemon candidus	bombus edwardsi	21	0.01	0.02	0.01	0.03
1	1	0.62	1	t	1	Rubus strigosus	Halictus pulzenus	21	0.03	0.02	0.02	0.03
1	0	0.49	1	t	1	Rubus strigosus	Ancistrocerus sp 2.	21	0.03	0.02	0.01	0.03
1	1	0.58	1	cv	1	Rubus	Halictus sp.	21	0.03	0.02	0.01	0.03
1	0	0.45	1	t	1	Rubus	Apis mellifica	21	0.03	0.02	0.00	0.03
1	0	0.43	1	t	1	Rubus strigosus	Colletes oromontis	21	0.03	0.02	0.00	0.03
1	0	0.43	1	t	1	Rubus strigosus	Megachile wootoni	21	0.03	0.02	0.00	0.03
1	0	0.43	1	cv	1	Rubus strigosus	Pseudomasaris vespoides	21	0.03	0.02	0.00	0.03
1	0	0.45	1	t	1	Rubus strigosus	bombus morrisoni	21	0.03	0.02	0.00	0.03
1	1	0.52	1	t	1	Rubus strigosus	bombus edwardsi	21	0.03	0.02	0.01	0.03
1	0	0.47	1	t	1	Rubus strigosus	Osmia propinqua	21	0.03	0.02	0.01	0.03
1	0	0.43	1	cv	1	Rubus strigosus	Clisodon terminalis	21	0.03	0.02	0.00	0.03

1	1	0.55	1	t	1	Rubus strigosus	Andrena vicina	21	0.03	0.02	0.01	0.03
1	1	0.59	1	t	1	Rubus strigosus	Andrena madronitens	21	0.03	0.02	0.02	0.03
1	1	0.58	1	t	1	Megachile wootoni calogaster	Pentstemon gracilis	21	0.01	0.03	0.04	0.02
1	1	0.63	1	cv	1	Megachile wootoni calogaster	Chamaeneriu m angustifolium	21	0.01	0.03	0.04	0.02
1	1	0.66	1	t	1	Megachile wootoni calogaster	Rosa acicularis	21	0.01	0.03	0.04	0.02
1	0	0.26	1	t	1	Megachile wootoni calogaster	Rubus deliciosus	21	0.01	0.03	0.00	0.02
1	0	0.27	1	t	1	bombus americanorum	Rubus deliciosus	21	0.00	0.02	0.00	0.02
1	0	0.26	1	cv	1	Anthrax sinuosa	Sedum stenopetalum	21	0.00	0.02	0.00	0.02
1	1	0.64	1	t	1	Cenis uhleri	Geranium caespitosum	21	0.00	0.03	0.05	0.02
1	1	0.69	1	t	1	Pentstemon gracilis	Halictus pulzenus	21	0.04	0.02	0.02	0.03
1	1	0.63	1	t	1	Pentstemon gracilis	Halictus (Evylaeus) sp.	21	0.04	0.02	0.01	0.03
1	1	0.65	1	cv	1	Pentstemon gracilis	bombus rufocinctus	21	0.04	0.02	0.01	0.03
1	1	0.66	1	t	1	Pentstemon gracilis	Monumetha albifrons	21	0.04	0.02	0.02	0.03
1	1	0.53	1	t	1	Pentstemon gracilis	Anthophora neomexicana	21	0.04	0.02	0.00	0.03
1	1	0.51	1	t	1	Pentstemon gracilis	Osmia phaceliae	21	0.04	0.02	0.00	0.03
1	1	0.73	1	cv	1	Pentstemon gracilis	bombus appositus	21	0.04	0.02	0.02	0.03
1	1	0.65	1	t	1	Pentstemon gracilis	Cerastomia lignaria	21	0.04	0.02	0.01	0.03
1	1	0.51	1	t	1	Pentstemon gracilis	Pseudomasar is vespoides	21	0.04	0.02	0.00	0.03
1	1	0.53	1	t	1	Pentstemon gracilis	bombus morrisoni	21	0.04	0.02	0.00	0.03
1	1	0.51	1	cv	1	Pentstemon gracilis	Prosopis basalis	21	0.04	0.02	0.00	0.03
1	1	0.60	1	t	1	Pentstemon gracilis	bombus edwardsi	21	0.04	0.02	0.01	0.03
1	1	0.51	1	t	1	Pentstemon gracilis	Clisodon terminalis	21	0.04	0.02	0.00	0.03
1	1	0.53	1	t	1	Pentstemon gracilis	Phormia regina	21	0.04	0.02	0.00	0.03

1	1	0.63	1	cv	1	Pentstemon gracilis	Halictus chloralictus	21	0.04	0.02	0.01	0.03
1	1	0.54	1	t	1	Pentstemon gracilis	Heriades gracilior	21	0.04	0.02	0.00	0.03
1	1	0.54	1	t	1	Pentstemon gracilis	Systoechus vulgaris	21	0.04	0.02	0.00	0.03
1	1	0.55	1	t	1	Pentstemon gracilis	Osmia hypoleuca	21	0.04	0.02	0.00	0.03
1	1	0.53	1	cv	1	Pentstemon gracilis	Titusella pronitens	21	0.04	0.02	0.00	0.03
1	1	0.66	1	t	1	Pentstemon gracilis	Prosopis elliptica	21	0.04	0.02	0.02	0.03
1	1	0.55	1	t	1	Pentstemon gracilis	Ceratina sp.	21	0.04	0.02	0.00	0.03
1	0	0.24	1	t	1	Pieris protodice	Sedum stenopetalum	21	0.00	0.02	0.00	0.02
1	0	0.24	1	cv	1	Anthidium tenuiflorae	Sedum stenopetalum	21	0.00	0.02	0.00	0.02
0	0	0.25	1	t	1	Linaria vulgaris	Basilarchia weidemeyeri	21	0.01	0.02	0.00	0.03
1	0	0.28	1	t	1	Linaria vulgaris	Pyrameis atlanta	21	0.01	0.02	0.01	0.03
1	0	0.25	1	t	1	Linaria	Apis mellifica	21	0.01	0.02	0.00	0.03
0	0	0.24	1	cv	1	Exoprosopa caliptera	Rubus deliciosus	21	0.00	0.03	0.00	0.02
1	1	0.60	1	t	1	Sphaerophoria cylindrica	Rosa acicularis	21	0.00	0.03	0.04	0.02
1	1	0.64	1	t	1	Prosopis tridentula	Geranium caespitosum	21	0.00	0.03	0.05	0.02
1	0	0.26	1	t	1	Lappula floribunda	Sphecodes sophiae	21	0.00	0.02	0.01	0.03
1	1	0.80	1	cv	1	Halictus pulzenus	Geranium caespitosum	21	0.02	0.03	0.05	0.02
1	0	0.34	1	t	1	Halictus pulzenus	Geranium richardsoni	21	0.02	0.03	0.00	0.02
1	0	0.38	1	t	1	Halictus pulzenus	Monarda fistulosa	21	0.02	0.03	0.01	0.02
1	0	0.38	1	t	1	Halictus pulzenus	Gilia aggregata	21	0.02	0.03	0.01	0.02
1	1	0.74	1	cv	1	Halictus pulzenus	Chamaeneriu m angustifolium	21	0.02	0.03	0.04	0.02
1	1	0.76	1	t	1	Halictus pulzenus	Rosa acicularis	21	0.02	0.03	0.04	0.02
1	1	0.62	1	t	1	Halictus pulzenus	Mertensia sibirica	21	0.02	0.03	0.03	0.02
1	1	0.53	1	t	1	Halictus pulzenus	Opulaster opulifolius	21	0.02	0.03	0.02	0.02

1	0	0.45	1	cv	1	Halictus pulzenus	Castilleia miniata	21	0.02	0.03	0.01	0.02
1	0	0.37	1	t	1	Halictus pulzenus	Rubus deliciosus	21	0.02	0.03	0.00	0.02
1	0	0.26	1	t	1	Achillea millefolium	Chrysophanus helloides	21	0.01	0.03	0.00	0.02
0	0	0.22	1	t	1	Achillea millefolium	Trypeta occidentalis	21	0.01	0.03	0.00	0.03
1	0	0.24	1	cv	1	Achillea millefolium	Panzeria radicum	21	0.01	0.03	0.00	0.03
0	0	0.22	1	t	1	Achillea millefolium	Synanthedon albicornis	21	0.01	0.03	0.00	0.03
0	0	0.24	1	t	1	Anthrax lateralis	Rubus deliciosus	21	0.00	0.03	0.00	0.02
0	0	0.28	1	t	1	Osmia megacephala	Rydbergia grandilfora	21	0.00	0.03	0.01	0.02
1	0	0.24	1	cv	1	Sedum stenopetalum	Argynnis eurynome	21	0.00	0.02	0.01	0.03
1	0	0.27	1	t	1	Sedum stenopetalum	bombus edwardsi	21	0.00	0.02	0.01	0.03
1	0	0.34	1	t	1	Sedum stenopetalum	Andrena madronitens	21	0.00	0.02	0.02	0.03
0	0	0.24	1	t	1	Sedum stenopetalum	Phormia camillus	21	0.00	0.02	0.00	0.02
1	0	0.24	1	cv	1	Sedum stenopetalum	Exoprosopa divisa	21	0.00	0.02	0.00	0.02
1	0	0.40	1	t	1	Halictus (Evylaeus) sp.	Astragalus drummondi	21	0.01	0.03	0.01	0.02
1	1	0.69	1	t	1	Halictus (Evylaeus) sp.	Chamaenerium angustifolium	21	0.01	0.03	0.04	0.02
1	1	0.71	1	t	1	Halictus (Evylaeus) sp.	Rosa acicularis	21	0.01	0.03	0.04	0.02
1	1	0.56	1	cv	1	Halictus (Evylaeus) sp.	Mertensia sibirica	21	0.01	0.03	0.03	0.02
1	0	0.46	1	t	1	Halictus (Evylaeus) sp.	Opulaster opulifolius	21	0.01	0.03	0.02	0.02
1	0	0.38	1	t	1	Halictus (Evylaeus) sp.	Castilleia miniata	21	0.01	0.03	0.01	0.02
1	0	0.43	1	t	1	Astragalus drummondi	Monumetha albifrons	21	0.01	0.02	0.02	0.03
1	0	0.30	1	cv	1	Astragalus drummondi	Megachile wootoni	21	0.01	0.02	0.00	0.03
1	0	0.31	1	t	1	Astragalus drummondi	bombus morrisoni	21	0.01	0.02	0.00	0.03
1	0	0.37	1	t	1	Astragalus drummondi	bombus edwardsi	21	0.01	0.02	0.01	0.03
1	0	0.40	1	t	1	Astragalus drummondi	Andrena vicina	21	0.01	0.02	0.01	0.03
1	0	0.23	1	cv	1	Helianthus petiolaris	Argynnis eurynome	21	0.00	0.02	0.01	0.03

1	0	0.27	1	t	1	Argynnis atlantis	Monarda fistulosa	21	0.01	0.03	0.01	0.02
1	1	0.63	1	t	1	Argynnis atlantis	Chamaenerium angustifolium	21	0.01	0.03	0.04	0.02
1	0	0.22	1	t	1	Echinomyia algens	Chrysopsis villosa	21	0.00	0.03	0.00	0.02
1	1	0.61	1	cv	1	Echinomyia algens	Chamaenerium angustifolium	21	0.00	0.03	0.04	0.02
1	1	0.70	1	t	1	Ancistrocerus sp 2.	Geranium caespitosum	21	0.01	0.03	0.05	0.02
1	0	0.24	1	t	1	Ancistrocerus sp 2.	Geranium richardsoni	21	0.01	0.03	0.00	0.02
1	1	0.50	1	t	1	Ancistrocerus sp 2.	Mertensia sibirica	21	0.01	0.03	0.03	0.02
0	0	0.24	1	cv	1	Heringia salix	Rubus deliciosus	21	0.00	0.03	0.00	0.02
1	0	0.24	1	t	1	Trifolium dasyphyllum	bombus kirbyellus	21	0.00	0.02	0.00	0.03
1	1	0.76	1	t	1	bombus rufocinctus	Geranium caespitosum	21	0.01	0.03	0.05	0.02
1	0	0.31	1	t	1	bombus rufocinctus	Aconitum columbianum	21	0.01	0.03	0.00	0.02
1	1	0.70	1	cv	1	bombus rufocinctus	Chamaenerium angustifolium	21	0.01	0.03	0.04	0.02
1	1	0.72	1	t	1	bombus rufocinctus	Rosa acicularis	21	0.01	0.03	0.04	0.02
1	0	0.45	1	t	1	bombus rufocinctus	Stanleya pinnatifida	21	0.01	0.03	0.02	0.02
1	0	0.33	1	t	1	bombus rufocinctus	Rubus deliciosus	21	0.01	0.03	0.00	0.02
1	0	0.44	1	cv	1	Pentstemon glaucus	Monumetha albifrons	21	0.01	0.02	0.02	0.03
1	0	0.30	1	t	1	Pentstemon glaucus	Megachile wootoni	21	0.01	0.02	0.00	0.03
1	0	0.30	1	t	1	Pentstemon glaucus	Pseudomasaris vespoides	21	0.01	0.02	0.00	0.03
1	0	0.32	1	t	1	Pentstemon glaucus	bombus morrisoni	21	0.01	0.02	0.00	0.03
1	0	0.38	1	cv	1	Pentstemon glaucus	bombus edwardsi	21	0.01	0.02	0.01	0.03
1	0	0.30	1	t	1	Pentstemon glaucus	Clisodon terminalis	21	0.01	0.02	0.00	0.03
1	0	0.44	1	t	1	Pentstemon glaucus	Prosopis elliptica	21	0.01	0.02	0.02	0.03
1	1	0.58	1	t	1	Megachile gemula albula	Chamaenerium angustifolium	21	0.00	0.03	0.04	0.02

1	0	0.34	1	cv	1	Hyloicus separatus	Pachylophus caespitosus	21	0.00	0.00	0.00	0.00
0	0	0.24	1	t	1	Myopa clausa	Rubus deliciosus	21	0.00	0.03	0.00	0.02
0	0	0.24	1	t	1	Temnostoma aequale	Rubus deliciosus	21	0.00	0.03	0.00	0.02
1	1	0.69	1	t	1	Megachile relativa	Geranium caespitosum	21	0.01	0.03	0.05	0.02
1	0	0.22	1	cv	1	Megachile relativa	Geranium richardsoni	21	0.01	0.03	0.00	0.02
1	1	0.62	1	t	1	Megachile relativa	Chamaenerium angustifolium	21	0.01	0.03	0.04	0.02
1	1	0.64	1	t	1	Megachile relativa	Rosa acicularis	21	0.01	0.03	0.04	0.02
1	1	0.77	1	t	1	Monumetha albifrons	Geranium caespitosum	21	0.02	0.03	0.05	0.02
1	1	0.71	1	cv	1	Monumetha albifrons	Chamaenerium angustifolium	21	0.02	0.03	0.04	0.02
1	1	0.73	1	t	1	Monumetha albifrons	Rosa acicularis	21	0.02	0.03	0.04	0.02
1	1	0.59	1	t	1	Monumetha albifrons	Mertensia sibirica	21	0.02	0.03	0.03	0.02
1	1	0.57	1	t	1	Monumetha albifrons	Carduus hookerianus	21	0.02	0.03	0.03	0.02
1	0	0.34	1	cv	1	Monumetha albifrons	Rubus deliciosus	21	0.02	0.03	0.00	0.02
1	1	0.61	1	t	1	Dejeania vexatrix	Chamaenerium angustifolium	21	0.00	0.03	0.04	0.02
1	0	0.48	1	t	1	Dejeania vexatrix	Mertensia sibirica	21	0.00	0.03	0.03	0.02
1	0	0.39	1	t	1	Dejeania vexatrix	Opulaster opulifolius	21	0.00	0.03	0.02	0.02
1	1	0.69	1	cv	1	Erebia epipsodea	Geranium caespitosum	21	0.01	0.03	0.05	0.02
1	0	0.33	1	t	1	Halictus sp.	Physaria didymocarpa	21	0.01	0.03	0.00	0.02
1	0	0.49	1	t	1	Halictus sp.	Opulaster opulifolius	21	0.01	0.03	0.02	0.02
1	0	0.34	1	t	1	Halictus sp.	Rubus deliciosus	21	0.01	0.03	0.00	0.02
1	1	0.69	1	cv	1	Argynnis eurynome	Geranium caespitosum	21	0.01	0.03	0.05	0.02
1	0	0.30	1	t	1	Argynnis eurynome	Rydbergia grandilfora	21	0.01	0.03	0.01	0.02
1	0	0.46	1	t	1	Epalpus rufus	Carduus hookerianus	21	0.00	0.03	0.03	0.02
0	0	0.28	1	t	1	bombus dorsalis	Capnoides aureum	21	0.00	0.03	0.01	0.02

1	1	0.75	1	cv	1	Geranium caespitosum	Prosopis wootoni	21	0.05	0.02	0.02	0.03
1	1	0.63	1	t	1	Geranium caespitosum	Osmia phaceliae	21	0.05	0.02	0.00	0.03
1	1	0.68	1	t	1	Geranium caespitosum	Osmia sp.	21	0.05	0.02	0.01	0.03
1	1	0.63	1	t	1	Geranium caespitosum	Panurginus cressoniellus	21	0.05	0.02	0.00	0.03
0	1	0.64	1	cv	1	Geranium caespitosum	Parnassius smintheus	21	0.05	0.02	0.00	0.04
1	1	0.82	1	t	1	Geranium caespitosum	bombus appositus	21	0.05	0.02	0.02	0.03
1	1	0.63	1	t	1	Geranium caespitosum	Irbisia brachycerus	21	0.05	0.02	0.00	0.03
1	1	0.63	1	t	1	Geranium caespitosum	Colletes oromontis	21	0.05	0.02	0.00	0.03
1	1	0.65	1	cv	1	Geranium caespitosum	Psammophila violacepennis	21	0.05	0.02	0.00	0.03
1	1	0.66	1	t	1	Geranium caespitosum	Megachile pugnata	21	0.05	0.02	0.00	0.03
1	1	0.70	1	t	1	Geranium caespitosum	Hammersch midtia ferruginea	21	0.05	0.02	0.01	0.03
1	1	0.63	1	t	1	Geranium caespitosum	Pseudomasar is vespoides	21	0.05	0.02	0.00	0.03
1	1	0.63	1	cv	1	Geranium caespitosum	Prosopis basalis	21	0.05	0.02	0.00	0.03
1	1	0.71	1	t	1	Geranium caespitosum	bombus edwardsi	21	0.05	0.02	0.01	0.03
1	1	0.67	1	t	1	Geranium caespitosum	Osmia propinqua	21	0.05	0.02	0.01	0.03
1	1	0.63	1	t	1	Geranium caespitosum	Clisodon terminalis	21	0.05	0.02	0.00	0.03
1	1	0.78	1	cv	1	Geranium caespitosum	Andrena braccata	21	0.05	0.02	0.02	0.03
1	1	0.62	1	t	1	Geranium caespitosum	Colletes sieverti	21	0.05	0.02	0.00	0.03
1	1	0.62	1	t	1	Geranium caespitosum	Glypta sp.	21	0.05	0.02	0.00	0.03
1	1	0.62	1	t	1	Geranium caespitosum	Sphex vulgaris	21	0.05	0.02	0.00	0.03
1	1	0.73	1	cv	1	Geranium caespitosum	Halictus chloralictus	21	0.05	0.02	0.01	0.03
1	1	0.73	1	t	1	Geranium caespitosum	Andrena vicina	21	0.05	0.02	0.01	0.03
1	1	0.77	1	t	1	Geranium caespitosum	Andrena madronitens	21	0.05	0.02	0.02	0.03
1	1	0.62	1	t	1	Geranium caespitosum	Tabanus rhombicus	21	0.05	0.02	0.00	0.03
1	1	0.76	1	cv	1	Geranium caespitosum	Prosopis elliptica	21	0.05	0.02	0.02	0.03

1	1	0.64	1	t	1	Geranium caespitosum	Coelioxys moesta	21	0.05	0.02	0.00	0.03
1	1	0.58	1	t	1	Megachile texana	Chamaenerium angustifolium	21	0.00	0.03	0.04	0.02
1	1	0.60	1	t	1	Megachile texana	Rosa acicularis	21	0.00	0.03	0.04	0.02
0	1	0.59	1	cv	1	Chionobis macouni	Iris missouriensis	21	0.00	0.04	0.05	0.04
0	0	0.15	1	t	1	Chionobis macouni	Draba aurea	21	0.00	0.04	0.00	0.04
0	0	0.24	1	t	1	Potentilla gracilis	bombus kirbyellus	21	0.00	0.02	0.00	0.03
1	0	0.29	1	t	1	Potentilla gracilis	Sphecodes sophiae	21	0.00	0.02	0.01	0.03
1	0	0.23	1	cv	1	Dolichovespula arctica	Delphinium scopulorum	21	0.01	0.03	0.00	0.02
1	1	0.62	1	t	1	Dolichovespula arctica	Chamaenerium angustifolium	21	0.01	0.03	0.04	0.02
1	0	0.45	1	t	1	Colletes sp. 2	Mertensia sibirica	21	0.00	0.03	0.03	0.02
0	0	0.24	1	t	1	Trichopticus septentrionali	Rubus deliciosus	21	0.00	0.03	0.00	0.02
1	0	0.22	1	cv	1	Geranium richardsoni	Apis mellifica	21	0.00	0.02	0.00	0.03
1	0	0.24	1	t	1	Geranium richardsoni	Osmia fulgida	21	0.00	0.02	0.01	0.03
1	0	0.33	1	t	1	Geranium richardsoni	Andrena madronitens	21	0.00	0.02	0.02	0.03
0	0	0.22	1	t	1	Geranium richardsoni	Ectemius montanus	21	0.00	0.02	0.00	0.02
1	0	0.32	1	cv	1	Geranium richardsoni	Prosopis elliptica	21	0.00	0.02	0.02	0.03
1	1	0.73	1	t	1	Prosopis wootoni	Rosa acicularis	21	0.02	0.03	0.04	0.02
1	0	0.33	1	t	1	Prosopis wootoni	Rubus deliciosus	21	0.02	0.03	0.00	0.02
1	0	0.40	1	t	1	Delphinium scopulorum	bombus appositus	21	0.00	0.02	0.02	0.03
0	0	0.23	1	cv	1	Delphinium scopulorum	Psithyrus consultus	21	0.00	0.02	0.00	0.03
1	0	0.22	1	t	1	Delphinium scopulorum	bombus morrisoni	21	0.00	0.02	0.00	0.03
1	0	0.27	1	t	1	Delphinium scopulorum	bombus edwardsi	21	0.00	0.02	0.01	0.03
1	1	0.72	1	t	1	Xylota nigra	Rosa acicularis	21	0.01	0.03	0.04	0.02
1	0	0.44	1	cv	1	Osmia phaceliae	Mertensia sibirica	21	0.00	0.03	0.03	0.02

1	0	0.26	1	t	1	Osmia sp.	Monarda fistulosa	21	0.01	0.03	0.01	0.02
1	1	0.61	1	t	1	Panurginus cressoniellus	Rosa acicularis	21	0.00	0.03	0.04	0.02
1	0	0.22	1	t	1	Panurginus cressoniellus	Rubus deliciosus	21	0.00	0.03	0.00	0.02
1	1	0.64	1	cv	1	Megachile vidua	Chamaeneriu m angustifolium	21	0.01	0.03	0.04	0.02
0	0	0.28	1	t	1	Eristalis temporalis	Rydbergia grandilfora	21	0.00	0.03	0.01	0.02
0	0	0.23	1	t	1	Eristalis temporalis	Rubus deliciosus	21	0.00	0.03	0.00	0.02
0	1	0.61	1	t	1	Parnassius smintheus	Iris missouriensis	21	0.00	0.04	0.05	0.04
1	0	0.28	1	cv	1	Atrytone taxiles	Monarda fistulosa	21	0.01	0.03	0.01	0.02
1	1	0.65	1	t	1	Atrytone taxiles	Chamaeneriu m angustifolium	21	0.01	0.03	0.04	0.02
1	0	0.33	1	t	1	Atrytone taxiles	Capnoides aureum	21	0.01	0.03	0.01	0.02
1	0	0.49	1	t	1	Pyrameis atlanta	Carduus hookerianus	21	0.01	0.03	0.03	0.02
1	1	0.52	1	cv	1	Apis mellifica	Mentzelia multiflora	21	0.00	0.03	0.03	0.03
1	0	0.28	1	t	1	Apis mellifica	Holodiscus dumosus	21	0.00	0.03	0.01	0.02
1	1	0.60	1	t	1	Apis mellifica	Chamaeneriu m angustifolium	21	0.00	0.03	0.04	0.02
1	1	0.63	1	t	1	Apis mellifica	Rosa acicularis	21	0.00	0.03	0.04	0.02
1	0	0.35	1	cv	1	Apis mellifica	Stanleya pinnatifida	21	0.00	0.03	0.02	0.02
1	0	0.22	1	t	1	Apis mellifica	Taraxacum officinale	21	0.00	0.03	0.00	0.02
1	0	0.38	1	t	1	Apis mellifica	Opulaster opulifolius	21	0.00	0.03	0.02	0.02
1	0	0.24	1	t	1	Apis mellifica	Rubus deliciosus	21	0.00	0.03	0.00	0.02
1	0	0.25	1	cv	1	Apis mellifica	Asclepias speciosa	21	0.00	0.03	0.00	0.02
1	0	0.42	1	t	1	bombus appositus	Monarda fistulosa	21	0.02	0.03	0.01	0.02
1	0	0.39	1	t	1	bombus appositus	Aconitum columbianum	21	0.02	0.03	0.00	0.02
1	0	0.38	1	t	1	bombus appositus	Gentiana parryi	21	0.02	0.03	0.00	0.02

1	1	0.77	1	cv	1	bombus appositus	Chamaenerium angustifolium	21	0.02	0.03	0.04	0.02
1	1	0.79	1	t	1	bombus appositus	Rosa acicularis	21	0.02	0.03	0.04	0.02
1	0	0.48	1	t	1	bombus appositus	Rydbergia grandilfora	21	0.02	0.03	0.01	0.02
1	1	0.65	1	t	1	bombus appositus	Carduus hookerianus	21	0.02	0.03	0.03	0.02
1	0	0.41	1	cv	1	bombus appositus	Rubus deliciosus	21	0.02	0.03	0.00	0.02
1	0	0.41	1	t	1	Andrena placida	Rubus deliciosus	21	0.02	0.02	0.00	0.02
0	0	0.27	1	t	1	Anthrax alternata	Holodiscus dumosus	21	0.00	0.03	0.01	0.02
1	0	0.20	1	t	1	Irbisia brachycerus	Malvastrum coccineum	21	0.00	0.03	0.00	0.02
1	0	0.23	1	cv	1	Colletes oromontis	Monarda fistulosa	21	0.00	0.03	0.01	0.02
1	0	0.27	1	t	1	Colletes oromontis	Holodiscus dumosus	21	0.00	0.03	0.01	0.02
1	0	0.28	1	t	1	Colletes oromontis	Capnoides aureum	21	0.00	0.03	0.01	0.02
1	0	0.45	1	t	1	Colletes oromontis	Mertensia sibirica	21	0.00	0.03	0.03	0.02
1	0	0.30	1	cv	1	Colletes oromontis	Mertensia pratensis	21	0.00	0.03	0.01	0.02
1	0	0.23	1	t	1	Colletes oromontis	Rubus deliciosus	21	0.00	0.03	0.00	0.02
1	0	0.27	1	t	1	Chrysopsis villosa	bombus edwardsi	21	0.00	0.02	0.01	0.03
1	0	0.26	1	t	1	Chrysogsater parva	Rubus deliciosus	21	0.00	0.02	0.00	0.02
1	0	0.23	1	cv	1	Megachile wootoni	Monarda fistulosa	21	0.00	0.03	0.01	0.02
1	1	0.59	1	t	1	Megachile wootoni	Chamaenerium angustifolium	21	0.00	0.03	0.04	0.02
1	1	0.61	1	t	1	Megachile wootoni	Rosa acicularis	21	0.00	0.03	0.04	0.02
1	0	0.36	1	t	1	Megachile wootoni	Opulaster opulifolius	21	0.00	0.03	0.02	0.02
1	0	0.44	1	cv	1	Megachile wootoni	Carduus hookerianus	21	0.00	0.03	0.03	0.02
1	0	0.23	1	t	1	Megachile wootoni	Rubus deliciosus	21	0.00	0.03	0.00	0.02
1	0	0.36	1	t	1	Bracon vulgaris	Stanleya pinnatifida	21	0.00	0.02	0.02	0.02
1	0	0.34	1	t	1	Pachylophus caespitosus	Protoparce quinquemaculatus	21	0.00	0.00	0.00	0.00

0	0	0.24	1	cv	1	Andrena prunorum	Rubus deliciosus	21	0.00	0.03	0.00	0.02
0	0	0.25	1	t	1	Monarda fistulosa	Psithyrus consultus	21	0.01	0.02	0.00	0.03
1	0	0.23	1	t	1	Monarda fistulosa	Pseudomasaris vespoides	21	0.01	0.02	0.00	0.03
1	0	0.25	1	t	1	Monarda fistulosa	bombus morrisoni	21	0.01	0.02	0.00	0.03
1	0	0.23	1	cv	1	Monarda fistulosa	Clisodon terminalis	21	0.01	0.02	0.00	0.03
0	0	0.24	1	t	1	Monarda fistulosa	bombus pennsylvanicus	21	0.01	0.02	0.00	0.03
1	0	0.33	1	t	1	Monarda fistulosa	Halictus chloralictus	21	0.01	0.02	0.01	0.03
1	0	0.24	1	t	1	Monarda fistulosa	Erynnis leonardus snowi	21	0.01	0.02	0.00	0.03
1	0	0.35	1	cv	1	Monarda fistulosa	Prosopis elliptica	21	0.01	0.02	0.02	0.03
1	1	0.53	1	t	1	Pedicularis racemosa	bombus edwardsi	21	0.03	0.02	0.01	0.03
1	1	0.61	1	t	1	Megachile pugnata	Chamaenerium angustifolium	21	0.00	0.03	0.04	0.02
1	0	0.46	1	t	1	Megachile pugnata	Carduus hookerianus	21	0.00	0.03	0.03	0.02
1	0	0.42	1	cv	1	Pompiloides sp.	Stanleya pinnatifida	21	0.01	0.02	0.02	0.02
1	0	0.28	1	t	1	Aconitum columbianum	bombus edwardsi	21	0.00	0.02	0.01	0.03
1	0	0.33	1	t	1	Hammerschmidtia ferruginea	Holodiscus dumosus	21	0.01	0.03	0.01	0.02
1	0	0.45	1	t	1	Pseudomasaris vespoides	Mertensia sibirica	21	0.00	0.03	0.03	0.02
1	0	0.36	1	cv	1	Pseudomasaris vespoides	Opulaster opulifolius	21	0.00	0.03	0.02	0.02
1	0	0.22	1	t	1	Pseudomasaris vespoides	Rubus deliciosus	21	0.00	0.03	0.00	0.02
1	0	0.45	1	t	1	Parnassius clodius	Carduus hookerianus	21	0.00	0.03	0.03	0.02
1	0	0.27	1	t	1	bombus morrisoni	Scutellaria resinosa	21	0.00	0.03	0.01	0.01
1	1	0.63	1	cv	1	bombus morrisoni	Rosa acicularis	21	0.00	0.03	0.04	0.02
1	0	0.35	1	t	1	bombus morrisoni	Stanleya pinnatifida	21	0.00	0.03	0.02	0.02
1	0	0.24	1	t	1	bombus morrisoni	Rubus deliciosus	21	0.00	0.03	0.00	0.02
1	1	0.58	1	t	1	Pieris sisymbri	Carduus hookerianus	21	0.01	0.03	0.03	0.02

0	0	0.24	1	cv	1	Arctophila flagrans	Rubus deliciosus	21	0.00	0.03	0.00	0.02
1	1	0.58	1	t	1	Prosopis basalis	Chamaenerium angustifolium	21	0.00	0.03	0.04	0.02
1	1	0.60	1	t	1	Prosopis basalis	Rosa acicularis	21	0.00	0.03	0.04	0.02
1	0	0.22	1	t	1	Prosopis basalis	Rubus deliciosus	21	0.00	0.03	0.00	0.02
1	0	0.34	1	cv	1	bombus edwardsi	Holodiscus dumosus	21	0.01	0.03	0.01	0.02
1	0	0.26	1	t	1	bombus edwardsi	Fragaria vesca	21	0.01	0.03	0.00	0.02
1	1	0.66	1	t	1	bombus edwardsi	Chamaenerium angustifolium	21	0.01	0.03	0.04	0.02
1	1	0.68	1	t	1	bombus edwardsi	Rosa acicularis	21	0.01	0.03	0.04	0.02
1	1	0.53	1	cv	1	bombus edwardsi	Mertensia sibirica	21	0.01	0.03	0.03	0.02
1	0	0.44	1	t	1	bombus edwardsi	Opulaster opulifolius	21	0.01	0.03	0.02	0.02
1	0	0.35	1	t	1	bombus edwardsi	Rydbergia grandilfora	21	0.01	0.03	0.01	0.02
1	1	0.52	1	t	1	bombus edwardsi	Carduus hookerianus	21	0.01	0.03	0.03	0.02
1	0	0.29	1	cv	1	bombus edwardsi	Rubus deliciosus	21	0.01	0.03	0.00	0.02
1	1	0.64	1	t	1	Osmia propinqua	Rosa acicularis	21	0.01	0.03	0.04	0.02
1	0	0.23	1	t	1	Osmia propinqua	Taraxacum officinale	21	0.01	0.03	0.00	0.02
1	0	0.25	1	t	1	Osmia propinqua	Rubus deliciosus	21	0.01	0.03	0.00	0.02
0	1	0.57	1	cv	1	Osmia abnormis	Iris missouriensis	21	0.00	0.04	0.05	0.04
1	0	0.27	1	t	1	Clisodon terminalis	Holodiscus dumosus	21	0.00	0.03	0.01	0.02
1	1	0.58	1	t	1	Clisodon terminalis	Chamaenerium angustifolium	21	0.00	0.03	0.04	0.02
1	1	0.61	1	t	1	Clisodon terminalis	Rosa acicularis	21	0.00	0.03	0.04	0.02
1	0	0.30	1	cv	1	Clisodon terminalis	Mertensia pratensis	21	0.00	0.03	0.01	0.02
1	0	0.43	1	t	1	Clisodon terminalis	Carduus hookerianus	21	0.00	0.03	0.03	0.02
1	1	0.75	1	t	1	Prosopis coloradensis	Rosa acicularis	21	0.02	0.03	0.04	0.02
1	0	0.30	1	t	1	bombus kirbyellus	Castilleia miniata	21	0.00	0.03	0.01	0.02

0	0	0.24	1	cv	1	bombus kirbyellus	Rubus deliciosus	21	0.00	0.03	0.00	0.02
1	0	0.41	1	t	1	Holodiscus dumosus	Andrena madronitens	21	0.01	0.02	0.02	0.03
1	0	0.24	1	t	1	Fragaria vesca	Osmia fulgida	21	0.00	0.02	0.01	0.03
1	0	0.23	1	t	1	Fragaria vesca	Heriades gracilior	21	0.00	0.02	0.00	0.03
1	1	0.66	1	cv	1	Osmia fulgida	Rosa acicularis	21	0.01	0.03	0.04	0.02
1	0	0.26	1	t	1	Osmia fulgida	Rubus deliciosus	21	0.01	0.03	0.00	0.02
1	0	0.33	1	t	1	Gilia aggregata	Halictus chloralictus	21	0.01	0.02	0.01	0.03
1	0	0.44	1	t	1	Grapta hylas	Carduus hookerianus	21	0.00	0.03	0.03	0.02
1	1	0.63	1	cv	1	Acmaeops pratensis	Rosa acicularis	21	0.00	0.03	0.04	0.02
1	0	0.24	1	t	1	Acmaeops pratensis	Rubus deliciosus	21	0.00	0.03	0.00	0.02
1	1	0.56	1	t	1	Chamaeneriu m	Anthophora occidentalis	21	0.04	0.02	0.00	0.03
1	1	0.58	1	t	1	Chamaeneriu m angustifolium	Erynnis leonardus snowi	21	0.04	0.02	0.00	0.03
1	1	0.56	1	cv	1	Chamaeneriu m	Melissodes aurigenia	21	0.04	0.02	0.00	0.03
1	1	0.70	1	t	1	Chamaeneriu m	Prosopis elliptica	21	0.04	0.02	0.02	0.03
1	1	0.65	1	t	1	Chamaeneriu m	Sphecodes sophiae	21	0.04	0.02	0.01	0.03
1	1	0.57	1	t	1	Chamaeneriu m	Coelioxys moesta	21	0.04	0.02	0.00	0.03
1	1	0.56	1	cv	1	Chamaeneriu m	Dolichovespu la diabolica	21	0.04	0.02	0.00	0.03
1	1	0.70	1	t	1	Rosa acicularis	Andrena vicina	21	0.04	0.02	0.01	0.03
1	1	0.73	1	t	1	Rosa acicularis	Andrena madronitens	21	0.04	0.02	0.02	0.03
1	1	0.62	1	t	1	Rosa acicularis	Eristalis latifrons	21	0.04	0.02	0.00	0.03
1	1	0.61	1	cv	1	Rosa acicularis	Systoechus vulgaris	21	0.04	0.02	0.00	0.03
1	1	0.58	1	t	1	Rosa acicularis	Pipiza sp.	21	0.04	0.02	0.00	0.03
1	1	0.72	1	t	1	Rosa acicularis	Prosopis elliptica	21	0.04	0.02	0.02	0.03
1	1	0.64	1	t	1	Rosa acicularis	Acmaeops longicornis	21	0.04	0.02	0.01	0.03
0	0	0.28	1	cv	1	Thanaos propertius	Capnoides aureum	21	0.00	0.03	0.01	0.02
1	0	0.28	1	t	1	Capnoides aureum	bombus fulvida	21	0.01	0.02	0.00	0.03

1	0	0.35	1	t	1	Stanleya pinnatifida	Lithargus apicalis opuntiae	21	0.02	0.02	0.00	0.02
1	0	0.44	1	t	1	Stanleya pinnatifida	Andrena vicina	21	0.02	0.02	0.01	0.03
1	0	0.37	1	cv	1	Stanleya pinnatifida	Odynerus annulatus	21	0.02	0.02	0.00	0.02
1	0	0.36	1	t	1	Stanleya pinnatifida	Ceratina sp.	21	0.02	0.02	0.00	0.03
0	0	0.24	1	t	1	Melissodes sp.	Rubus deliciosus	21	0.00	0.03	0.00	0.02
1	1	0.56	1	t	1	Halictus chloralictus	Mertensia sibirica	21	0.01	0.03	0.03	0.02
1	0	0.46	1	cv	1	Halictus chloralictus	Opulaster opulifolius	21	0.01	0.03	0.02	0.02
1	0	0.38	1	t	1	Halictus chloralictus	Castilleia miniata	21	0.01	0.03	0.01	0.02
0	0	0.24	1	t	1	Trichodes ornatus	Rubus deliciosus	21	0.00	0.03	0.00	0.02
1	0	0.28	1	t	1	Echinomyia decias	Asclepias speciosa	21	0.00	0.01	0.00	0.02
1	0	0.31	1	cv	1	Andrena vicina	Rubus deliciosus	21	0.01	0.03	0.00	0.02
1	1	0.60	1	t	1	Andrena madronitens	Mertensia sibirica	21	0.02	0.03	0.03	0.02
1	1	0.50	1	t	1	Andrena madronitens	Opulaster opulifolius	21	0.02	0.03	0.02	0.02
1	0	0.35	1	t	1	Andrena madronitens	Rubus deliciosus	21	0.02	0.03	0.00	0.02
1	0	0.26	1	cv	1	Nomada sp.	Rubus deliciosus	21	0.00	0.02	0.00	0.02
1	0	0.48	1	t	1	Osmia nigrifrons	Mertensia sibirica	21	0.00	0.03	0.03	0.02
1	0	0.32	1	t	1	Osmia nigrifrons	Mertensia pratensis	21	0.00	0.03	0.01	0.02
1	0	0.45	1	t	1	Clisodon sp.	Mertensia sibirica	21	0.00	0.03	0.03	0.02
1	0	0.26	1	cv	1	Phormia camillus	Rubus deliciosus	21	0.00	0.02	0.00	0.02
1	0	0.46	1	t	1	Mertensia sibirica	Osmia coloradella	21	0.03	0.02	0.00	0.03
1	0	0.44	1	t	1	Mertensia sibirica	bombus fulvida	21	0.03	0.02	0.00	0.03
1	0	0.50	1	t	1	Mertensia sibirica	Acmaeops longicornis	21	0.03	0.02	0.01	0.03
1	0	0.47	1	cv	1	Mertensia sibirica	Ceratina sp.	21	0.03	0.02	0.00	0.03
1	0	0.33	1	t	1	Mertensia pratensis	Osmia hypoleuca	21	0.01	0.02	0.00	0.03
1	0	0.32	1	t	1	Mertensia pratensis	Osmia albolateralis	21	0.01	0.02	0.00	0.02

1	0	0.32	1	t	1	Mertensia pratensis	Osmia pusilla	21	0.01	0.02	0.00	0.02
1	0	0.38	1	cv	1	Opulaster opulifolius	Eristalis latifrons	21	0.02	0.02	0.00	0.03
1	0	0.24	1	t	1	Eristalis latifrons	Rubus deliciosus	21	0.00	0.03	0.00	0.02
1	0	0.24	1	t	1	Systoechus vulgaris	Rubus deliciosus	21	0.00	0.03	0.00	0.02
1	0	0.34	1	t	1	Prosopis elliptica	Rubus deliciosus	21	0.02	0.03	0.00	0.02
1	0	0.48	1	cv	1	Carduus hookerianus	Acmaeops longicornis	21	0.03	0.02	0.01	0.03
1	0	0.27	1	t	1	Rubus deliciosus	Acmaeops longicornis	21	0.00	0.02	0.01	0.03
1	0	0.30	1	t	1	Rubus deliciosus	Eristalis flavipes	21	0.00	0.02	0.01	0.02
0	0	0.26	1	t	1	Rubus deliciosus	Andrena canadensis	21	0.00	0.02	0.00	0.02
1	0	0.31	0	cv	1	Brenthis helena	Rydbergia grandilfora	21	0.00	0.02	0.01	0.02
1	0	0.24	0	t	1	Galium boreale	Anthrax lateralis	21	0.00	0.02	0.00	0.03
1	0	0.35	0	t	1	Sieversia ciliata	Syrphus opinator	21	0.01	0.02	0.01	0.03
1	0	0.23	0	t	1	Dasyllis fernaldi	Syrphus opinator	21	0.00	0.03	0.01	0.03
0	0	0.18	0	cv	1	Trypeta occidentalis	Thanaos propertius	21	0.00	0.03	0.00	0.03
1	0	0.39	0	t	1	Agapostemon coloradensis	Rubus deliciosus	21	0.02	0.02	0.00	0.02
1	0	0.22	0	t	1	Theventimtia muricatus	bombus kirbyellus	21	0.00	0.02	0.00	0.03
1	0	0.32	0	t	1	Caltha leptosepala	Pseudomelecta californica miranda	21	0.00	0.01	0.01	0.02
1	0	0.22	0	cv	1	Malvastrum coccineum	Osmia pusilla	21	0.00	0.02	0.00	0.02
1	0	0.20	0	t	1	Dejeania vexatrix	bombus fulvida	21	0.00	0.03	0.00	0.03
1	0	0.24	0	t	1	Apocynum androsaemifolium	Spegina infuscata	21	0.00	0.02	0.00	0.02
1	0	0.34	0	t	1	Penstemon secundiflorus	Grapta hylas	21	0.02	0.02	0.00	0.03
0	0	0.31	0	cv	1	Heracleum lanatum	Galium boreale	21	0.01	0.02	0.00	0.02
1	0	0.32	0	t	1	Prosopis coloradensis	Lithargus apicalis opuntiae	21	0.02	0.03	0.00	0.02
0	0	0.27	0	t	1	Penstemon halli	Melissodes sp.	21	0.01	0.02	0.00	0.03

0	0	0.31	0	t	1	Pedicularis parryi	Monarda fistulosa	21	0.00	0.01	0.01	0.02
1	0	0.23	0	cv	1	Halictus lerouxi	Phacelia heterophylla	21	0.00	0.02	0.00	0.02
0	0	0.16	0	t	1	Osmia bella	Pipiza vanduzeei	21	0.00	0.04	0.00	0.03
1	0	0.19	0	t	1	Andrena lewisi	Coelioxys moesta	21	0.00	0.03	0.00	0.03
1	0	0.23	0	t	1	Frasera speciosa	Vespa occidentalis	21	0.00	0.02	0.00	0.03
1	0	0.21	0	cv	1	Bembex spinolae	Melissodes aurigenia	21	0.00	0.02	0.00	0.03
1	0	0.32	0	t	1	Syrphus americanus	Geum rivale	21	0.00	0.03	0.01	0.01
1	0	0.26	0	t	1	Achillea millefolium	Eristalis tenax	21	0.01	0.03	0.00	0.03
1	0	0.25	0	t	1	Cenis uhleri	Andrena vicina	21	0.00	0.03	0.01	0.03
1	0	0.25	0	cv	1	Syrphus disgregus	bombus nevadensis	21	0.00	0.02	0.01	0.03
1	0	0.20	0	t	1	Erebia epipsodea	Trypeta occidentalis	21	0.01	0.03	0.00	0.03
1	0	0.23	0	t	1	Syritta pipiens	Osmia rohweri	21	0.00	0.02	0.00	0.02
1	0	0.49	0	t	1	Megachile texana	Mentzelia multiflora	21	0.00	0.03	0.03	0.03
0	0	0.30	0	cv	1	Silene halli	Taraxacum officinale	21	0.01	0.02	0.00	0.02
1	0	0.26	0	t	1	Onagra biennis	Megachile wootoni calogaster	21	0.00	0.02	0.01	0.03
1	0	0.25	0	t	1	Physaria didymocarpa	Nomada sp.	21	0.00	0.02	0.00	0.02
1	0	0.37	0	t	1	bombus appositus	Ectemius montanus	21	0.02	0.03	0.00	0.02
0	0	0.30	0	cv	1	Halictus lerouxi	Holodiscus dumosus	21	0.00	0.02	0.01	0.02
1	0	0.21	0	t	1	Colletes kincaidi	Basilarchia weidemeyeri	21	0.01	0.03	0.00	0.03
0	0	0.17	0	t	1	Osmia abnormis	Lithargus apicalis opuntiae	21	0.00	0.04	0.00	0.02
1	0	0.30	0	t	1	Silene halli	Osmia fulgida	21	0.01	0.02	0.01	0.03
1	0	0.25	0	cv	1	bombus bifarius	Epalpus bicolor	21	0.01	0.03	0.00	0.03
1	0	0.27	0	t	1	Clisodon terminalis	Rydbergia grandilfora	21	0.00	0.03	0.01	0.02
0	0	0.37	0	t	1	Penstemon secundiflorus	Erigeron uniflorus	21	0.02	0.02	0.00	0.02

0	0	0.22	0	t	1	Apocynum androsaemifolium	Geocoris bullatus	21	0.00	0.02	0.00	0.03
1	0	0.26	0	cv	1	Prosopis sp.	Hammerschmidtia ferruginea	21	0.00	0.03	0.01	0.03
1	0	0.24	0	t	1	Osmia densa	Clisodon terminalis	21	0.01	0.03	0.00	0.03
1	0	0.21	0	t	1	Xylota flavitibia	Melissodes sp.	21	0.00	0.03	0.00	0.03
1	0	0.32	0	t	1	Ibidium strictum	Syrphus nitens	21	0.01	0.02	0.00	0.02
1	0	0.23	0	cv	1	Tenthredella flavomarginis	Osmia pentstemonis	21	0.00	0.03	0.01	0.03
0	0	0.45	0	t	1	Melissodes fremonti	Rubus strigosus	21	0.00	0.03	0.03	0.02
1	0	0.32	0	t	1	Andrena crataegi	Andrena madronitens	21	0.00	0.03	0.02	0.03
0	1	0.72	0	t	1	Geranium caespitosum	Protoparce quinquemaculatus	21	0.05	0.02	0.00	0.00
1	0	0.28	0	cv	1	Caltha leptosepala	Echinomyia decias	21	0.00	0.01	0.00	0.01
1	0	0.23	0	t	1	Osmia rohweri	Coenonympha pamphiloides	21	0.00	0.02	0.00	0.03
1	0	0.26	0	t	1	Prunus demissa	Osmia pentstemonis	21	0.00	0.02	0.01	0.03
1	0	0.22	0	t	1	Epeolus helianthi	Rubus deliciosus	21	0.00	0.03	0.00	0.02
1	0	0.25	0	cv	1	Paragus bicolor	Osmia bruneri	21	0.00	0.00	0.00	0.03
1	0	0.22	0	t	1	Osmia megacephala	Echinomyia decias	21	0.00	0.03	0.00	0.01
1	1	0.65	0	t	1	Agapostemon sp.	Rosa acicularis	21	0.00	0.02	0.04	0.02
1	0	0.25	0	t	1	Anthrax sinuosa	Osmia hypoleuca	21	0.00	0.02	0.00	0.03
1	0	0.20	0	cv	1	Dejeania vexatrix	Thanaos propertius	21	0.00	0.03	0.00	0.03
0	0	0.40	0	t	1	Holodiscus dumosus	Castilleia miniata	21	0.01	0.02	0.01	0.02
0	0	0.36	0	t	1	Frasera speciosa	Geum rivale	21	0.00	0.02	0.01	0.01
1	0	0.23	0	t	1	Psithyrus fernaldae	Ancistrocerus sp 2.	21	0.00	0.03	0.01	0.03
1	0	0.30	0	cv	1	Megachile relativa	Rydbergia grandilfora	21	0.01	0.03	0.01	0.02
1	0	0.28	0	t	1	Hyloicus separatus	Gonochrysis densa	21	0.00	0.00	0.00	0.02

1	0	0.22	0	t	1	Galium boreale	Prosopis basalis	21	0.00	0.02	0.00	0.03
1	0	0.21	0	t	1	Epalpus bicolor	bombus dorsalis	21	0.00	0.03	0.00	0.03
1	0	0.23	0	cv	1	Triepeolus helianthi grandior	Eristalis latifrons	21	0.00	0.02	0.00	0.03
0	0	0.22	0	t	1	Phacelia heterophylla	Thanaos propertius	21	0.00	0.02	0.00	0.03
1	0	0.18	0	t	1	Epeolus helianthi	Anthrax alternata	21	0.00	0.03	0.00	0.03
0	0	0.28	0	t	1	Penstemon unilateralis	Arctophila flagrans	21	0.01	0.02	0.00	0.03
1	0	0.23	0	cv	1	Oxybelus sp.	Malvastrum coccineum	21	0.00	0.02	0.00	0.02
1	0	0.25	0	t	1	bombus fervidus	Osmia nigrifrons	21	0.01	0.03	0.00	0.03
1	0	0.24	0	t	1	Gilia aggregata	Clisodon sp.	21	0.01	0.02	0.00	0.03
1	0	0.22	0	t	1	Eristalis tenax	Eristalis temporalis	21	0.00	0.03	0.00	0.03
1	0	0.24	0	cv	1	Gnophaela vermiculata	Prosopis cressoni	21	0.01	0.03	0.00	0.03
1	0	0.27	0	t	1	Agapostemon sp.	bombus fervidus	21	0.00	0.02	0.01	0.03
0	0	0.25	0	t	1	Penstemon halli	Trypeta occidentalis	21	0.01	0.02	0.00	0.03
0	0	0.31	0	t	1	Aquilegia coerulea	Penstemon halli	21	0.00	0.02	0.01	0.02
1	0	0.27	0	cv	1	Celerio lineata	Trifolium dasyphyllum	21	0.00	0.02	0.00	0.02
1	0	0.18	0	t	1	Bombylius atriceps	Basilarchia weidemeyeri	21	0.00	0.03	0.00	0.03
0	0	0.25	0	t	1	Aster foliaceus	Eristalis tenax	21	0.00	0.02	0.00	0.03
1	0	0.28	0	t	1	bombus juxtus	Calochortus gunnisoni	21	0.00	0.03	0.01	0.02
1	0	0.27	0	cv	1	Specomyia vittata	Omalus sp.	21	0.00	0.01	0.00	0.02
1	0	0.37	0	t	1	Bombomelect a fulvida	Penstemon secundiflorus	21	0.00	0.03	0.02	0.02
1	0	0.34	0	t	1	Aragalus lamberti	Anthophora bomboides	21	0.01	0.01	0.00	0.02
1	0	0.28	0	t	1	Prosopis varifrons	Heringia salix	21	0.01	0.03	0.00	0.03
1	0	0.31	0	cv	1	Ibidium strictum	Gonochrysis densa	21	0.01	0.02	0.00	0.02
1	0	0.19	0	t	1	Andrena lewisi	Clisodon sp.	21	0.00	0.03	0.00	0.03
1	0	0.19	0	t	1	bombus morrisoni	Synanthedon albicornis	21	0.00	0.03	0.00	0.03
1	0	0.21	0	t	1	Myopa clausa	Panzeria radicum	21	0.00	0.03	0.00	0.03

1	0	0.28	0	cv	1	Hyloicus separatus	Megachile pugnata	21	0.00	0.00	0.00	0.03
1	0	0.38	0	t	1	Osmia fulgida	Stanleya pinnatifida	21	0.01	0.03	0.02	0.02
1	1	0.58	0	t	1	bombus occidentalis	Pieris sisymbri	21	0.03	0.03	0.01	0.03
1	0	0.22	0	t	1	Ectemius muricatus	Andrena canadensis	21	0.00	0.03	0.00	0.02
1	0	0.22	0	cv	1	Eristalis dimidiatus	Apis mellifica	21	0.00	0.02	0.00	0.03
0	0	0.19	0	t	1	Allium recurvatum	Osmia abnormis	21	0.00	0.02	0.00	0.04
1	0	0.20	0	t	1	Trypeta occidentalis	Osmia hypoleuca	21	0.00	0.03	0.00	0.03
1	0	0.19	0	t	1	Halictus manitonellus	Parnassius smintheus	21	0.00	0.03	0.00	0.04
1	0	0.40	0	cv	1	Penstemon secundiflorus	Atrytone taxiles	21	0.02	0.02	0.01	0.03
1	0	0.22	0	t	1	bombus bifarius	Eristalis temporalis	21	0.01	0.03	0.00	0.03
1	0	0.20	0	t	1	Solenius sp.	Helianthus petiolaris	21	0.00	0.03	0.00	0.02
1	0	0.20	0	t	1	Thanaos martialis	Apanteles sp.	21	0.01	0.03	0.00	0.03
1	0	0.18	0	cv	1	Osmia bruneri	bombus pennsylvanicus	21	0.00	0.03	0.00	0.03
1	0	0.34	0	t	1	bombus appositus	Pipiza sp.	21	0.02	0.03	0.00	0.03
0	0	0.43	0	t	1	Jamesia americana	Pieris sisymbri	21	0.01	0.02	0.01	0.03
0	0	0.27	0	t	1	Anthrax nigra	Dasyphora fruticosa	21	0.00	0.03	0.01	0.02
1	0	0.27	0	cv	1	bombus nevadensis	Achillea millefolium	21	0.01	0.03	0.01	0.03
1	0	0.23	0	t	1	Megachile texana	Eristalis flavipes	21	0.00	0.03	0.01	0.02
1	0	0.22	0	t	1	Brenthis triclaris	Bombomelecta fulvida	21	0.00	0.03	0.00	0.03
1	0	0.24	0	t	1	Caltha leptosepala	bombus hunti	21	0.00	0.01	0.00	0.03
1	0	0.23	0	cv	1	Elephantella groenlandica	Eristalis dimidiatus	21	0.00	0.02	0.00	0.02
1	0	0.17	0	t	1	Psithyrus insularis	Irbisia brachycerus	21	0.00	0.03	0.00	0.03
1	0	0.23	0	t	1	Megachile wootoni calogaster	Megachile pugnata	21	0.01	0.03	0.00	0.03
1	0	0.38	0	t	1	Prosopis episcopalis	Castilleia miniata	21	0.01	0.03	0.01	0.02

1	0	0.18	0	cv	1	Apanteles sp.	Arctophila flagrans	21	0.00	0.03	0.00	0.03
1	0	0.23	0	t	1	Anthrax	Osmia fulgida	21	0.00	0.03	0.01	0.03
1	0	0.45	0	t	1	bombus occidentalis	Colias alexandra	21	0.03	0.03	0.00	0.03
1	0	0.19	0	t	1	Halictus medionitens	Thanaos propertius	21	0.00	0.03	0.00	0.03
1	0	0.22	0	cv	1	bombus hunti	Megachile vidua	21	0.00	0.03	0.01	0.03
1	0	0.20	0	t	1	Tabanus rhombicus	Osmia hypoleuca	21	0.00	0.03	0.00	0.03
1	0	0.22	0	t	1	Scaba opinator	bombus nevadensis	21	0.00	0.03	0.01	0.03
0	0	0.20	0	t	1	Celerio lineata	Draba aurea	21	0.00	0.02	0.00	0.04
0	0	0.20	0	cv	1	Lithospermum canescens	Draba aurea	21	0.00	0.02	0.00	0.04
1	0	0.22	0	t	1	Vespa occidentalis	Trichodes ornatus	21	0.00	0.03	0.00	0.03
1	0	0.20	0	t	1	Pipiza vanduzeei	Parnassius clodius	21	0.00	0.03	0.00	0.03
1	0	0.31	0	t	1	Anthophora smithi	Prosopis elliptica	21	0.00	0.03	0.02	0.03
1	0	0.28	0	cv	1	Peleteria robusta	Halictus chloralictus	21	0.00	0.02	0.01	0.03
1	0	0.20	0	t	1	Anthophora bomboides	Colletes sieverti	21	0.00	0.02	0.00	0.03
1	0	0.47	0	t	1	Volucella rufomaculata	Carduus hookerianus	21	0.00	0.03	0.03	0.02
1	0	0.33	0	t	1	Osmia melanotricha	Calochortus gunnisoni	21	0.01	0.03	0.01	0.02
0	0	0.21	0	cv	1	Colias keewaydina	Chrysopsis villosa	21	0.00	0.03	0.00	0.02
1	0	0.23	0	t	1	Celerio lineata	Osmia coloradella	21	0.00	0.02	0.00	0.03
0	0	0.31	0	t	1	Colias scudderi	Penstemon unilateralis	21	0.01	0.03	0.01	0.02
0	0	0.17	0	t	1	Bombylius atriceps	Tabanus rhombicus	21	0.00	0.03	0.00	0.03
1	0	0.17	0	cv	1	Colletes sieverti	Synanthedon albicornis	21	0.00	0.03	0.00	0.03
0	0	0.23	0	t	1	Potentilla arguta	Andrena canadensis	21	0.00	0.02	0.00	0.02
1	0	0.37	0	t	1	Penstemon secundiflorus	Anthidium tenuiflorae	21	0.02	0.02	0.00	0.02
1	0	0.22	0	t	1	Erysimum asperum	Osmia nigrifrons	21	0.00	0.02	0.00	0.03
1	0	0.23	0	cv	1	Ophion sp.	Clisodon terminalis	21	0.00	0.01	0.00	0.03
1	0	0.28	0	t	1	Leptura chrysocoma	Coenonympha pamphiloides	21	0.00	0.01	0.00	0.03

1	0	0.29	0	t	1	Silene halli	Chrysogsater parva	21	0.01	0.02	0.00	0.02
0	0	0.43	0	t	1	Solidago missouriensis	Dasyphora fruticosa	21	0.02	0.02	0.01	0.02
1	0	0.28	0	cv	1	Colletes oromontis	Prosopis elliptica	21	0.00	0.03	0.02	0.03
1	0	0.22	0	t	1	Galium boreale	Irbisia brachycerus	21	0.00	0.02	0.00	0.03
1	0	0.23	0	t	1	Thanaos martialis	Tetrachrysis lauta	21	0.01	0.03	0.00	0.02
1	0	0.20	0	t	1	Andrena lewisi	Heringia salix	21	0.00	0.03	0.00	0.03
1	0	0.29	0	cv	1	Silene halli	Syritta pipiens	21	0.01	0.02	0.00	0.02
1	0	0.16	0	t	1	Anthophora occidentalis	Draba aurea	21	0.00	0.03	0.00	0.04
1	0	0.27	0	t	1	Pedicularis parryi	Osmia propinqua	21	0.00	0.01	0.01	0.03
1	0	0.43	0	t	1	bombus occidentalis	Grapta hylas	21	0.03	0.03	0.00	0.03
1	0	0.17	0	cv	1	Tabanus rhombicus	Clisodon sp.	21	0.00	0.03	0.00	0.03
0	0	0.32	0	t	1	Lappula floribunda	Castilleia miniata	21	0.00	0.02	0.01	0.02
1	0	0.18	0	t	1	bombus kirbyellus	Tabanus rhombicus	21	0.00	0.03	0.00	0.03
1	0	0.19	0	t	1	Apanteles sp.	Panzeria radicum	21	0.00	0.03	0.00	0.03
0	0	0.46	0	cv	1	Capnoides aureum	Stanleya pinnatifida	21	0.01	0.02	0.02	0.02
1	0	0.32	0	t	1	Pedicularis parryi	Protoparce quinquemaculatus	21	0.00	0.01	0.00	0.00
1	0	0.27	0	t	1	Silene halli	Titusella pronitens	21	0.01	0.02	0.00	0.03
1	0	0.24	0	t	1	Vespa germanica	bombus fervidus	21	0.00	0.03	0.01	0.03
1	0	0.30	0	cv	1	Andrena madronitens	Geocoris bullatus	21	0.02	0.03	0.00	0.03
1	0	0.25	0	t	1	Prosopis sp.	Physaria didymocarpa	21	0.00	0.03	0.00	0.02
1	0	0.38	0	t	1	Chilosia petulca	Sterictophora sp.	21	0.01	0.01	0.01	0.02
1	0	0.19	0	t	1	Colias alexandra	Colletes sieverti	21	0.00	0.03	0.00	0.03
0	0	0.22	0	cv	1	Anthrax hypomelas	Arctostaphylus uva-ursi	21	0.00	0.03	0.00	0.02
0	1	0.57	0	t	1	Onagra biennis	Mentzelia multiflora	21	0.00	0.02	0.03	0.03
1	0	0.29	0	t	1	Prosopis episcopalis	Chilosia tristis	21	0.01	0.03	0.00	0.02
0	1	0.56	0	t	1	Agathis	Nomada sp.	21	0.04	0.04	0.00	0.02

1	0	0.23	0	cv	1	Osmia brevis	Phormia camillus	21	0.00	0.02	0.00	0.02
1	0	0.23	0	t	1	Exoprosopa caliptera	Echinomyia decias	21	0.00	0.03	0.00	0.01
1	0	0.21	0	t	1	Gonochrysis densa	Grapta hylas	21	0.00	0.02	0.00	0.03
1	0	0.34	0	t	1	bombus fervidus	Holodiscus dumosus	21	0.01	0.03	0.01	0.02
1	0	0.41	0	cv	1	Halictus (Evylaeus) sp.	Andrena braccata	21	0.01	0.03	0.02	0.03
1	1	0.58	0	t	1	Agapostemon sp.	Pentstemon gracilis	21	0.00	0.02	0.04	0.02
1	0	0.24	0	t	1	Anthrax sinuosa	Trichodes ornatus	21	0.00	0.02	0.00	0.03
1	0	0.29	0	t	1	Bombylius atriceps	Prosopis coloradensis	21	0.00	0.03	0.02	0.03
1	0	0.21	0	cv	1	bombus juxtus	Agapostemon sp.	21	0.00	0.03	0.00	0.02
1	0	0.44	0	t	1	Chrysophanus sirius	Anthrax halycon	21	0.03	0.03	0.00	0.03
1	0	0.20	0	t	1	Osmia wardiana	Gentiana parryi	21	0.00	0.03	0.00	0.02
1	0	0.21	0	t	1	Panurginus sp.	Megachile texana	21	0.00	0.03	0.00	0.03
1	1	0.89	0	cv	1	Geranium caespitosum	Mentzelia multiflora	21	0.05	0.02	0.03	0.03
1	0	0.19	0	t	1	Tenthredella flavomarginis	Psithyrus insularis	21	0.00	0.03	0.00	0.03
1	0	0.27	0	t	1	Syrphus opinator	Achillea millefolium	21	0.01	0.03	0.01	0.03
1	0	0.21	0	t	1	Tenthredella unicincta	Dolichovespu la diabolica	21	0.00	0.02	0.00	0.03
1	0	0.45	0	cv	1	Agapostemon coloradensis	Holodiscus dumosus	21	0.02	0.02	0.01	0.02
1	0	0.20	0	t	1	Monumetha argentifrons	Geranium richardsoni	21	0.00	0.03	0.00	0.02
1	0	0.24	0	t	1	Eristalis dimidiatus	Brachyopa notata	21	0.00	0.02	0.00	0.02
1	0	0.23	0	t	1	Agapostemon sp.	Heriades gracilior	21	0.00	0.02	0.00	0.03
1	0	0.32	0	cv	1	Prosopis wootoni	Pyrameis atlanta	21	0.02	0.03	0.01	0.03
1	0	0.20	0	t	1	Osmia phaceliae	Osmia hypoleuca	21	0.00	0.03	0.00	0.03
0	0	0.25	0	t	1	Achillea millefolium	Andrena canadensis	21	0.01	0.03	0.00	0.02
1	0	0.23	0	t	1	Andrena edwiniae	Bombylius atriceps	21	0.01	0.02	0.00	0.03
1	0	0.21	0	cv	1	Trichius affinis	Curtipogon leucozona	21	0.00	0.03	0.00	0.03

1	1	0.62	0	t	1	Scaba opinator	Rosa acicularis	21	0.00	0.03	0.04	0.02
1	0	0.19	0	t	1	Syrphus amalopis	Panzeria radicum	21	0.00	0.03	0.00	0.03
1	0	0.21	0	t	1	Pipiza	Oxybelus sp.	21	0.00	0.03	0.00	0.02
1	0	0.29	0	cv	1	Penstemon unilateralis	Papilio rutulus	21	0.01	0.02	0.00	0.02
0	0	0.47	0	t	1	Trichius affinis	Rubus strigosus	21	0.00	0.03	0.03	0.02
1	0	0.29	0	t	1	Halictus (Evylaeus) sp.	Erigeron uniflorus	21	0.01	0.03	0.00	0.02
1	0	0.27	0	t	1	bombus nevadensis	Gilia aggregata	21	0.01	0.03	0.01	0.02
1	0	0.33	0	cv	1	Chilosia petulca	Chrysotoxum integrum	21	0.01	0.01	0.00	0.03
1	0	0.28	0	t	1	Caltha leptosepala	Colias edwardsi	21	0.00	0.01	0.01	0.03
1	0	0.20	0	t	1	Pipiza vanduzeei	Trichopticus septentrional is	21	0.00	0.03	0.00	0.03
1	0	0.25	0	t	1	Thyreocoris anthracina	Systoechus vulgaris	21	0.00	0.02	0.00	0.03
1	0	0.32	0	cv	1	Aragalus lamberti	bombus hunti	21	0.01	0.01	0.00	0.03
0	0	0.25	0	t	1	Colias edwardsi	Taraxacum officinale	21	0.01	0.03	0.00	0.02
1	0	0.30	0	t	1	Halictus pulzenus	Prosopis basalis	21	0.02	0.03	0.00	0.03
1	0	0.25	0	t	1	Paragus bicolor	Irbisia brachycerus	21	0.00	0.00	0.00	0.03
1	0	0.22	0	cv	1	Fragaria vesca	Eristalis latifrons	21	0.00	0.02	0.00	0.03
1	0	0.24	0	t	1	Celerio lineata	Dejeania vexatrix	21	0.00	0.02	0.00	0.03
1	0	0.32	0	t	1	Agapostemon sp.	Halictus sp.	21	0.00	0.02	0.01	0.03
1	0	0.23	0	t	1	Elephantella groenlandica	Theventimtia muricatus	21	0.00	0.02	0.00	0.02
0	0	0.33	0	cv	1	Spegina infuscata	Castilleia miniata	21	0.00	0.02	0.01	0.02
0	0	0.50	0	t	1	Prunus virginiana	Pedicularis racemosa	21	0.00	0.02	0.03	0.02
1	0	0.22	0	t	1	Thalictrum fendleri	Prosopis tridentula	21	0.00	0.02	0.00	0.03
0	0	0.19	0	t	1	Anthrax nigra	Synanthedon albicornis	21	0.00	0.03	0.00	0.03
1	0	0.26	0	cv	1	Colias	Archytas sp.	21	0.01	0.03	0.00	0.01
1	0	0.23	0	t	1	Psithyrus insularis	Gilia aggregata	21	0.00	0.03	0.01	0.02
1	0	0.23	0	t	1	Halictus lerouxi	Arctostaphyl us uva-ursi	21	0.00	0.02	0.00	0.02

1	0	0.23	0	t	1	Prunus pennsylvanica	Megachile pugnata	21	0.00	0.02	0.00	0.03
1	0	0.20	0	cv	1	Andrena crataegi	Panurginus cressoniellus	21	0.00	0.03	0.00	0.03
1	1	0.58	0	t	1	Pentstemon gracilis	Atrytone taxiles	21	0.04	0.02	0.01	0.03
1	0	0.21	0	t	1	Pyrameis atlanta	Pipiza sp.	21	0.01	0.03	0.00	0.03
0	0	0.28	0	t	1	Clementsia rhodantha	Elephantella groenlandica	21	0.00	0.00	0.00	0.02
1	0	0.27	0	cv	1	Cenis uhleri	Castilleia miniata	21	0.00	0.03	0.01	0.02
1	0	0.22	0	t	1	Lappula floribunda	Heriades gracilior	21	0.00	0.02	0.00	0.03
1	0	0.26	0	t	1	Protoparce quinquemacul atus	Anthophora occidentalis	21	0.00	0.00	0.00	0.03
1	0	0.29	0	t	1	Dasyllis fernaldi	Cerastomia lignaria	21	0.00	0.03	0.01	0.03
1	0	0.27	0	cv	1	bombus centralis	Lycaena pseudargiolu s	21	0.01	0.03	0.01	0.03
1	0	0.16	0	t	1	Chionobis macouni	Pipiza sp.	21	0.00	0.04	0.00	0.03
1	0	0.25	0	t	1	Trifolium dasyphyllum	Ectemius montanus	21	0.00	0.02	0.00	0.02
1	0	0.23	0	t	1	Tetrachrysis lauta	Spegina infuscata	21	0.00	0.02	0.00	0.02
1	0	0.20	0	cv	1	Psithyrus ashtoni	Echinomyia algens	21	0.00	0.03	0.00	0.03
1	0	0.20	0	t	1	Osmia fulgida	Glypta sp.	21	0.01	0.03	0.00	0.03
1	0	0.20	0	t	1	Pseudomasari s vespoides	Ceratina sp.	21	0.00	0.03	0.00	0.03
1	0	0.18	0	t	1	Osmia	Draba aurea	21	0.00	0.03	0.00	0.04
1	0	0.20	0	cv	1	Anthrax lateralis	Osmia megacephala	21	0.00	0.03	0.00	0.03
1	0	0.22	0	t	1	Thanaos martialis	Arctophila flagrans	21	0.01	0.03	0.00	0.03
1	0	0.21	0	t	1	Andrena washingtoni	Osmia hypoleuca	21	0.00	0.03	0.00	0.03
1	0	0.23	0	t	1	Halictus lerouxi	Megachile relativa	21	0.00	0.02	0.01	0.03
1	0	0.27	0	cv	1	Andrena edwiniae	Epalpus bicolor	21	0.01	0.02	0.00	0.03
0	0	0.31	0	t	1	Elephantella groenlandica	Capnoides aureum	21	0.00	0.02	0.01	0.02
0	0	0.30	0	t	1	Sterictophora sp.	Achillea millefolium	21	0.01	0.02	0.01	0.03
0	0	0.27	0	t	1	Selasphorus platycercus	Capnoides aureum	21	0.00	0.04	0.01	0.02

1	0	0.22	0	cv	1	Valeriana edulis	Osmia nigrifrons	21	0.00	0.02	0.00	0.03
1	0	0.27	0	t	1	Silene halli	Prosopis cressoni	21	0.01	0.02	0.00	0.03
1	0	0.26	0	t	1	Clementsia rhodantha	Colletes oromontis	21	0.00	0.00	0.00	0.03
1	0	0.23	0	t	1	bombus	Heringia salix	21	0.01	0.03	0.00	0.03
0	0	0.26	0	cv	1	Eristalis dimidiatus	Achillea millefolium	21	0.00	0.02	0.01	0.03
1	0	0.23	0	t	1	Drymocallis fissa	Phormia regina	21	0.00	0.02	0.00	0.03
1	0	0.26	0	t	1	bombus nevadensis	bombus americanorum	21	0.01	0.03	0.00	0.02
1	0	0.20	0	t	1	Anthrax nigra	Temnostoma aequale	21	0.00	0.03	0.00	0.03
1	0	0.21	0	cv	1	Osmia coloradella	Systoechus vulgaris	21	0.00	0.03	0.00	0.03
1	0	0.24	0	t	1	Syrphus disgregus	Halicyoides mamus	21	0.00	0.02	0.01	0.03
1	0	0.24	0	t	1	Helianthus petiolaris	Tenthredella unicincta	21	0.00	0.02	0.00	0.02
1	0	0.24	0	t	1	Aquilegia brevistyla	Nomada sp.	21	0.00	0.02	0.00	0.02
0	0	0.28	0	cv	1	Prunus demissa	Linaria vulgaris	21	0.00	0.02	0.01	0.02
1	0	0.22	0	t	1	Malvastrum coccineum	Osmia coloradella	21	0.00	0.02	0.00	0.03
1	0	0.28	0	t	1	Xylota flavitibia	Andrena vicina	21	0.00	0.03	0.01	0.03
0	0	0.34	0	t	1	Penstemon secundiflorus	Osmia megacephala	21	0.02	0.02	0.00	0.03
1	0	0.48	0	cv	1	Halictus pulzenus	Andrena braccata	21	0.02	0.03	0.02	0.03
0	0	0.22	0	t	1	Potentilla arguta	Prosopis cressoni	21	0.00	0.02	0.00	0.03
1	0	0.22	0	t	1	Satyrus charon	Anthrax fulviana	21	0.00	0.03	0.00	0.02
1	0	0.18	0	t	1	Epeolus helianthi	Nathalis iole	21	0.00	0.03	0.00	0.03
0	0	0.24	0	cv	1	Penstemon halli	Osmia abnormis	21	0.01	0.02	0.00	0.04
0	0	0.18	0	t	1	Colias alexandra	Chionobis macouni	21	0.00	0.03	0.00	0.04
0	1	0.57	0	t	1	Agathis vulgaris	Halicyoides mamus	21	0.04	0.04	0.01	0.03
1	0	0.23	0	t	1	Megachile pugnata	Coenonympha pamphiloides	21	0.00	0.03	0.00	0.03
1	0	0.22	0	cv	1	Lathyrus ornatus	Chrysotoxum integrum	21	0.00	0.02	0.00	0.03

1	0	0.29	0	t	1	Calochortus gunnisoni	Bracon helena	21	0.01	0.02	0.00	0.02
1	0	0.31	0	t	1	Osmia phaceliae	Andrena braccata	21	0.00	0.03	0.02	0.03
1	0	0.25	0	t	1	Lycaena pseudargiolus	Ptilodexia harpasa	21	0.01	0.03	0.00	0.02
1	0	0.31	0	cv	1	Pompiloides sp.	Sphecodes sophiae	21	0.01	0.02	0.01	0.03
1	0	0.20	0	t	1	Anthophora bomboides	Prosopis tridentula	21	0.00	0.02	0.00	0.03
1	0	0.20	0	t	1	Arctostaphylus uva-ursi	Tabanus rhombicus	21	0.00	0.02	0.00	0.03
1	0	0.34	0	t	1	Phormia camillus	Mertensia pratensis	21	0.00	0.02	0.01	0.02
1	0	0.29	0	cv	1	Pieris protodice	Pseudomelecta californica miranda	21	0.00	0.02	0.01	0.02
1	0	0.18	0	t	1	Colletes americanus	Glypta sp.	21	0.00	0.03	0.00	0.03
1	0	0.32	0	t	1	bombus flavifrons	Chrysotoxum ventricosum	21	0.02	0.03	0.00	0.03
1	0	0.20	0	t	1	Heringia salix	Basilarchia weidemeyeri	21	0.00	0.03	0.00	0.03
1	0	0.18	0	cv	1	Melitaea sp.	Apanteles sp.	21	0.00	0.03	0.00	0.03
1	0	0.20	0	t	1	Prunus virginiana	Tabanus rhombicus	21	0.00	0.02	0.00	0.03
1	0	0.25	0	t	1	Parnassius clodius	Eristalis flavipes	21	0.00	0.03	0.01	0.02
1	0	0.42	0	t	1	Onagra biennis	bombus appositus	21	0.00	0.02	0.02	0.03
1	0	0.46	0	cv	1	Halictus manitonellus	Mertensia sibirica	21	0.00	0.03	0.03	0.02
1	0	0.22	0	t	1	Galium boreale	Dolichovespula diabolica	21	0.00	0.02	0.00	0.03
1	0	0.24	0	t	1	bombus americanorum	Ceratina sp.	21	0.00	0.02	0.00	0.03
1	0	0.23	0	t	1	Osmia pentstemonis	bombus morrisoni	21	0.01	0.03	0.00	0.03
1	0	0.29	0	cv	1	Clisodon sp.	Castilleia miniata	21	0.00	0.03	0.01	0.02
1	0	0.21	0	t	1	Prosopis cressoni	Oxybelus sp.	21	0.00	0.03	0.00	0.02
1	0	0.22	0	t	1	Argynnis eurynome	Osmia nigrifrons	21	0.01	0.03	0.00	0.03
1	0	0.36	0	t	1	Strictophora sp.	Castilleia miniata	21	0.01	0.02	0.01	0.02
1	0	0.20	0	cv	1	Psithyrus ashtoni	Theventimtia muricatus	21	0.00	0.03	0.00	0.02
0	0	0.22	0	t	1	Pipiza vanduzeei	Fragaria vesca	21	0.00	0.03	0.00	0.02

1	0	0.49	0	t	1	Bombomelecta fulvida	Pedicularis racemosa	21	0.00	0.03	0.03	0.02
1	0	0.28	0	t	1	Prunus pennsylvanica	Pachylophus caespitosus	21	0.00	0.02	0.00	0.00
0	0	0.24	0	cv	1	Helianthus petiolaris	Eristalis tenax	21	0.00	0.02	0.00	0.03
1	0	0.27	0	t	1	Panurginus cressoniellus	Capnoides aureum	21	0.00	0.03	0.01	0.02
1	0	0.24	0	t	1	Panurginus sp.	Halictus lerouxi	21	0.00	0.03	0.00	0.02
1	0	0.36	0	t	1	bombus appositus	Arctophila flagrans	21	0.02	0.03	0.00	0.03
1	0	0.20	0	cv	1	Pipiza vanduzeei	Halictus medionitens	21	0.00	0.03	0.00	0.03
1	0	0.21	0	t	1	Andrena sp.	Omalus sp.	21	0.00	0.03	0.00	0.02
1	0	0.25	0	t	1	Xylota flavitibia	Monarda fistulosa	21	0.00	0.03	0.01	0.02
0	0	0.23	0	t	1	Pipiza vanduzeei	Aster foliaceus	21	0.00	0.03	0.00	0.02
1	0	0.22	0	cv	1	Foenus perplexus	Megachile vidua	21	0.00	0.03	0.01	0.03
1	0	0.24	0	t	1	Pedicularis parryi	Clisodon terminalis	21	0.00	0.01	0.00	0.03
0	0	0.24	0	t	1	Silene halli	Chionobis macouni	21	0.01	0.02	0.00	0.04
1	0	0.24	0	t	1	Pieris protodice	Chrysopsis villosa	21	0.00	0.02	0.00	0.02
1	0	0.20	0	cv	1	Phormia camillus	Pipiza sp.	21	0.00	0.02	0.00	0.03
1	0	0.21	0	t	1	Halictus manitonellus	Bembex spinolae	21	0.00	0.03	0.00	0.02
1	0	0.47	0	t	1	Chrysophanus sirius	Chrysogsater parva	21	0.03	0.03	0.00	0.02
1	0	0.20	0	t	1	Protothyreopus dilectus	Theventimtia muricatus	21	0.00	0.03	0.00	0.02
1	0	0.25	0	cv	1	Linaria	Heringia salix	21	0.01	0.02	0.00	0.03
1	0	0.25	0	t	1	Syrphus disgregus	Chrysophanus helloides	21	0.00	0.02	0.00	0.02
1	0	0.47	0	t	1	Erizoa olivalis	Glypta sp.	21	0.04	0.03	0.00	0.03
1	0	0.24	0	t	1	Syritta pipiens	Syrphus nitens	21	0.00	0.02	0.00	0.02
1	0	0.16	0	cv	1	Mallota flavoterminata	Draba aurea	21	0.00	0.03	0.00	0.04
1	0	0.23	0	t	1	Megachile wootoni calogaster	Psithyrus consultus	21	0.01	0.03	0.00	0.03
1	0	0.21	0	t	1	Melitaea sp.	Lithospermum canescens	21	0.00	0.03	0.00	0.02
1	0	0.23	0	t	1	Osmia brevis	Argynnis atlantis	21	0.00	0.02	0.01	0.03

1	0	0.25	0	cv	1	Psithyrus fernaldae	bombus fervidus	21	0.00	0.03	0.01	0.03
1	0	0.22	0	t	1	Osmia rohweri	Ceratina sp.	21	0.00	0.02	0.00	0.03
1	0	0.19	0	t	1	Osmia bruneri	Chrysotoxum ventricosum	21	0.00	0.03	0.00	0.03
1	0	0.38	0	t	1	Apanteles sp.	Erigeron macranthus	21	0.00	0.03	0.02	0.02
1	0	0.22	0	cv	1	Lycaena pseudargiolus	Sphaerophoria cylindrica	21	0.01	0.03	0.00	0.03
1	0	0.22	0	t	1	Peleteria robusta	Chrysopsis villosa	21	0.00	0.02	0.00	0.02
1	0	0.43	0	t	1	Syrphus amalopis	Rubus strigosus	21	0.00	0.03	0.03	0.02
1	0	0.20	0	t	1	Eristalis temporalis	Papilio rutulus	21	0.00	0.03	0.00	0.02
1	0	0.44	0	cv	1	Caltha leptosepala	bombus appositus	21	0.00	0.01	0.02	0.03
1	0	0.26	0	t	1	Monarda fistulosa	Osmia propinqua	21	0.01	0.02	0.01	0.03
1	0	0.40	0	t	1	Xylota angustiventris	Eristalis latifrons	21	0.02	0.02	0.00	0.03
1	0	0.25	0	t	1	Andrena prunorum	Specomyia vittata	21	0.00	0.03	0.00	0.01
1	0	0.30	0	cv	1	Polygonum bistorta	Chrysogsater parva	21	0.01	0.02	0.00	0.02
1	0	0.23	0	t	1	Brenthis triclaris	Syrphus nitens	21	0.00	0.03	0.00	0.02
1	0	0.21	0	t	1	Eristalis tenax	Megachile wootoni	21	0.00	0.03	0.00	0.03
1	0	0.25	0	t	1	bombus nevadensis	Coenonympha pamphiloides	21	0.01	0.03	0.00	0.03
1	0	0.28	0	cv	1	Halicyoides mamus	Exoprosopa volucris	21	0.01	0.03	0.00	0.00
1	0	0.24	0	t	1	Celerio lineata	Gonochrysis densa	21	0.00	0.02	0.00	0.02
1	0	0.21	0	t	1	Volucella rufomaculata	Parnassius smintheus	21	0.00	0.03	0.00	0.04
1	0	0.25	0	t	1	Panurginus sp.	Colias scudderi	21	0.00	0.03	0.01	0.03
1	0	0.18	0	cv	1	Prosopis tridentula	Parnassius clodius	21	0.00	0.03	0.00	0.03
0	0	0.28	0	t	1	Potentilla pulcherrima	Osmia abnormis	21	0.02	0.02	0.00	0.04
1	0	0.37	0	t	1	Echinomyia algens	Andrena placida	21	0.00	0.03	0.02	0.02
0	1	0.85	0	t	1	Rubus strigosus	Rosa acicularis	21	0.03	0.02	0.04	0.02
0	0	0.33	0	cv	1	Tradescantia virginiana	Castilleia miniata	21	0.00	0.02	0.01	0.02

1	1	0.60	0	t	1	Iris missouriensis	Systoechus vulgaris	21	0.05	0.04	0.00	0.03
1	0	0.44	0	t	1	Cerastomia lignaria	Andrena braccata	21	0.01	0.03	0.02	0.03
1	0	0.25	0	t	1	Anthidium tenuiflorae	Coenonymph a pamphiloides	21	0.00	0.02	0.00	0.03
1	1	0.60	0	cv	1	Erizoa olivalis	Andrena vicina	21	0.04	0.03	0.01	0.03
1	0	0.22	0	t	1	bombus nevadensis	Grapta hylas	21	0.01	0.03	0.00	0.03
1	0	0.25	0	t	1	Prosopis episcopalis	Pipiza sp.	21	0.01	0.03	0.00	0.03
1	0	0.38	0	t	1	Geum rivale	Acmaeops longicornis	21	0.01	0.01	0.01	0.03
1	0	0.24	0	cv	1	Monarda fistulosa	bombus fulvida	21	0.01	0.02	0.00	0.03
1	0	0.22	0	t	1	Thermopsis montana	Anthophora occidentalis	21	0.00	0.02	0.00	0.03
1	0	0.27	0	t	1	Clementsia rhodantha	Nathalis iole	21	0.00	0.00	0.00	0.03
1	0	0.24	0	t	1	Frasera speciosa	Syrphus disgregus	21	0.00	0.02	0.00	0.02
1	0	0.27	0	cv	1	Chilosia tristis	Sphecodes sophiae	21	0.00	0.02	0.01	0.03
1	0	0.27	0	t	1	Pyrameis atlanta	bombus edwardsi	21	0.01	0.03	0.01	0.03
0	0	0.26	0	t	1	Calliphora vomitoria	Chionobis macouni	21	0.01	0.01	0.00	0.04
1	0	0.22	0	t	1	Anthophora simillima	Megachile wootoni calogaster	21	0.00	0.03	0.01	0.03
1	0	0.42	0	cv	1	Colias edwardsi	Opulaster opulifolius	21	0.01	0.03	0.02	0.02
1	0	0.28	0	t	1	Panurginus sp.	Hyloicus separatus	21	0.00	0.03	0.00	0.00
1	0	0.30	0	t	1	Agapostemon sp.	Halictus chloralictus	21	0.00	0.02	0.01	0.03
1	0	0.33	0	t	1	Bombomelect a fulvida	Mertensia pratensis	21	0.00	0.03	0.01	0.02
1	0	0.24	0	cv	1	bombus fervidus	Coelioxys moesta	21	0.01	0.03	0.00	0.03
1	0	0.30	0	t	1	Andrena gardineri	Ibidium strictum	21	0.00	0.03	0.01	0.02
1	0	0.30	0	t	1	Xylota nigra	Tenthredella unicincta	21	0.01	0.03	0.00	0.02
1	0	0.29	0	t	1	Argynnis eurynome	Halictus chloralictus	21	0.01	0.03	0.01	0.03
1	0	0.36	0	cv	1	Osmia pentstemonis	Potentilla pulcherrima	21	0.01	0.03	0.02	0.02

1	0	0.18	0	t	1	bombus hunti	bombus juxtus	21	0.00	0.03	0.00	0.03
1	0	0.24	0	t	1	Anthrax sinuosa	Osmia nigrifrons	21	0.00	0.02	0.00	0.03
1	0	0.24	0	t	1	Prosopis sp.	Osmia sp.	21	0.00	0.03	0.01	0.03
1	0	0.41	0	cv	1	Andrena madronitens	Rydbergia grandilfora	21	0.02	0.03	0.01	0.02
1	0	0.29	0	t	1	Helianthus petiolaris	Pseudomelecta californica miranda	21	0.00	0.02	0.01	0.02
1	0	0.24	0	t	1	Halictus manitonellus	bombus fervidus	21	0.00	0.03	0.01	0.03
1	0	0.34	0	t	1	Prosopis varifrons	Linaria vulgaris	21	0.01	0.03	0.01	0.02
1	0	0.28	0	cv	1	Chrysophanus rubidus	Exoprosopa volucris	21	0.00	0.03	0.00	0.00
0	0	0.22	0	t	1	Drymocallis fissa	bombus dorsalis	21	0.00	0.02	0.00	0.03
0	0	0.21	0	t	1	Pentstemon glaber	Thanaos propertius	21	0.00	0.02	0.00	0.03
1	0	0.33	0	t	1	bombus edwardsi	Andrena vicina	21	0.01	0.03	0.01	0.03
1	0	0.23	0	cv	1	Osmia pentstemonis	Geocoris bullatus	21	0.01	0.03	0.00	0.03
1	0	0.22	0	t	1	Megachile wootoni calogaster	Thanaos propertius	21	0.01	0.03	0.00	0.03
1	0	0.20	0	t	1	Colias scudderi	Prosopis tridentula	21	0.01	0.03	0.00	0.03
1	0	0.23	0	t	1	Phorbia fusciceps	Phormia regina	21	0.01	0.03	0.00	0.03
1	0	0.20	0	cv	1	Syrphus americanus	Ptilodexia harpasa	21	0.00	0.03	0.00	0.02
1	0	0.22	0	t	1	Ctenucha sp.	Andrena sp.	21	0.01	0.03	0.00	0.03
1	0	0.21	0	t	1	Foenus perplexus	Sedum stenopetalum	21	0.00	0.03	0.00	0.02
0	0	0.24	0	t	1	Mallota flavoterminata	Silene acaulis	21	0.00	0.03	0.00	0.00
1	0	0.23	0	cv	1	Anthrax	Clisodon sp.	21	0.00	0.02	0.00	0.03
1	0	0.35	0	t	1	Andrena washingtoni	Andrena placida	21	0.00	0.03	0.02	0.02
1	0	0.22	0	t	1	Syritta pipiens	Heringia salix	21	0.00	0.02	0.00	0.03
1	0	0.30	0	t	1	Petalostemon purpureus	Anthophora bomboides	21	0.01	0.02	0.00	0.02
1	0	0.22	0	cv	1	Panzeria radicum	Geranium richardsoni	21	0.00	0.03	0.00	0.02
0	0	0.28	0	t	1	Heracleum lanatum	Lappula floribunda	21	0.01	0.02	0.00	0.02
1	0	0.29	0	t	1	Leptura chrysocoma	Anthrax sinuosa	21	0.00	0.01	0.00	0.02

0	0	0.30	0	t	1	Apocynum androsaemifolium	Holodiscus dumosus	21	0.00	0.02	0.01	0.02
1	0	0.20	0	cv	1	Bombylius atriceps	Tetraopes sp.	21	0.00	0.03	0.00	0.02
0	0	0.23	0	t	1	Potentilla gracilis	Thanaos propertius	21	0.00	0.02	0.00	0.03
1	0	0.23	0	t	1	Andrena edwiniae	bombus juxtus	21	0.01	0.02	0.00	0.03
0	0	0.30	0	t	1	Campanula rotundifolia	Prunus pennsylvanica	21	0.01	0.02	0.00	0.02
1	0	0.33	0	cv	1	Paragus bicolor	Pompiloides sp.	21	0.00	0.00	0.01	0.02
1	0	0.21	0	t	1	Chrysophanus helloides	Panurginus cressoniellus	21	0.00	0.02	0.00	0.03
0	0	0.22	0	t	1	Colias alexandra	Taraxacum officinale	21	0.00	0.03	0.00	0.02
0	0	0.21	0	t	1	Osmia melanotricha	Chionobis macouni	21	0.01	0.03	0.00	0.04
1	0	0.29	0	cv	1	Brenthis triclaris	Polygonum bistorta	21	0.00	0.03	0.01	0.02
1	0	0.31	0	t	1	Megachile wootoni calogaster	Holodiscus dumosus	21	0.01	0.03	0.01	0.02
1	0	0.20	0	t	1	Melitaea sp.	Acmaeops pratensis	21	0.00	0.03	0.00	0.03
1	0	0.23	0	t	1	Trifolium dasyphyllum	Megachile wootoni	21	0.00	0.02	0.00	0.03
1	0	0.29	0	cv	1	Andrena madronitens	Clisodon sp.	21	0.02	0.03	0.00	0.03
1	0	0.20	0	t	1	Omalus sp.	Glypta sp.	21	0.00	0.02	0.00	0.03
1	0	0.21	0	t	1	Dolichovespula arctica	Erynnis leonardus snowi	21	0.01	0.03	0.00	0.03
0	0	0.23	0	t	1	Potentilla arguta	Brachyopa notata	21	0.00	0.02	0.00	0.02
1	0	0.26	0	cv	1	Andrena washingtoni	Osmia densa	21	0.00	0.03	0.01	0.03
1	0	0.30	0	t	1	Andrena gardineri	Pieris sisymbri	21	0.00	0.03	0.01	0.03
1	0	0.19	0	t	1	Chrysotoxum integrum	Dolichovespula diabolica	21	0.00	0.03	0.00	0.03
1	0	0.19	0	t	1	Syrphus amalopis	Acmaeops pratensis	21	0.00	0.03	0.00	0.03
1	0	0.20	0	cv	1	Prosopis tridentula	Dolichovespula arctica	21	0.00	0.03	0.01	0.03
1	0	0.30	0	t	1	Panurginus sp.	Halictus (Evylaeus) sp.	21	0.00	0.03	0.01	0.03
1	0	0.25	0	t	1	Lycaena pseudargiolus	Ceratina sp.	21	0.01	0.03	0.00	0.03

1	0	0.24	0	t	1	Megachile wootoni	Sphecodes sophiae	21	0.00	0.03	0.01	0.03
1	0	0.46	0	cv	1	bombus occidentalis	Andrena crataegi	21	0.03	0.03	0.00	0.03
1	0	0.22	0	t	1	Volucella satur	Vespa occidentalis	21	0.00	0.02	0.00	0.03
0	0	0.47	0	t	1	Chrysophanus sirius	Malvastrum coccineum	21	0.03	0.03	0.00	0.02
1	0	0.23	0	t	1	Ctenucha sp.	Brachyopa notata	21	0.01	0.03	0.00	0.02
1	0	0.24	0	cv	1	Lycaena glaucon	Megachile sp.	21	0.00	0.02	0.00	0.03
1	0	0.22	0	t	1	Colias alexandra	Megachile relativa	21	0.00	0.03	0.01	0.03
1	0	0.25	0	t	1	Potentilla arguta	Echinomyia decias	21	0.00	0.02	0.00	0.01
1	0	0.23	0	t	1	Osmia brevis	Anthidium tenuiflorae	21	0.00	0.02	0.00	0.02
0	1	0.54	0	cv	1	Monarda fistulosa	Pedicularis racemosa	21	0.01	0.02	0.03	0.02
1	0	0.34	0	t	1	Campanula rotundifolia	Specomyia vittata	21	0.01	0.02	0.00	0.01
0	0	0.38	0	t	1	Allium recurvatum	Stanleya pinnatifida	21	0.00	0.02	0.02	0.02
0	0	0.24	0	t	1	Aquilegia brevistyla	Erigeron uniflorus	21	0.00	0.02	0.00	0.02
0	0	0.34	0	cv	1	Solidago missouriensis	bombus pennsylvanic us	21	0.02	0.02	0.00	0.03
1	0	0.47	0	t	1	Dasyllis fernaldi	Pedicularis racemosa	21	0.00	0.03	0.03	0.02
1	0	0.23	0	t	1	Thalictrum fendleri	Pipiza sp.	21	0.00	0.02	0.00	0.03
1	0	0.31	0	t	1	bombus flavifrons	Colletes sp. 2	21	0.02	0.03	0.00	0.03
1	0	0.24	0	cv	1	Helianthus petiolaris	Odynerus annulatus	21	0.00	0.02	0.00	0.02
1	0	0.24	0	t	1	Thanaos martialis	Halicyoides mamus	21	0.01	0.03	0.01	0.03
1	0	0.18	0	t	1	Syrphus amalopis	Eristalis temporalis	21	0.00	0.03	0.00	0.03
1	0	0.35	0	t	1	Vespa occidentalis	bombus flavifrons	21	0.00	0.03	0.02	0.03
1	0	0.23	0	cv	1	Volucella satur	Eristalis tenax	21	0.00	0.02	0.00	0.03
0	0	0.23	0	t	1	Phacelia heterophylla	Lappula floribunda	21	0.00	0.02	0.00	0.02
1	0	0.34	0	t	1	Chilosia petulca	Osmia propinqua	21	0.01	0.01	0.01	0.03
0	1	0.63	0	t	1	Chrysotoxum ventricosum	Rosa acicularis	21	0.00	0.03	0.04	0.02

1	0	0.31	0	cv	1	Phorbia fusciceps	Anthomyia sp.	21	0.01	0.03	0.00	0.00
1	0	0.31	0	t	1	bombus flavifrons	Colletes oromontis	21	0.02	0.03	0.00	0.03
1	0	0.37	0	t	1	Dolichovespula arctica	Stanleya pinnatifida	21	0.01	0.03	0.02	0.02
1	0	0.22	0	t	1	Prosopis tridentula	Gilia aggregata	21	0.00	0.03	0.01	0.02
1	0	0.20	0	cv	1	Prosopis cressoni	Trichopticus septentrionalis	21	0.00	0.03	0.00	0.03
1	0	0.25	0	t	1	Penstemon	Glypta sp.	21	0.01	0.02	0.00	0.03
1	0	0.20	0	t	1	Scaba opinator	Ectemius montanus	21	0.00	0.03	0.00	0.02
1	0	0.28	0	t	1	Osmia pentstemonis	Eristalis flavipes	21	0.01	0.03	0.01	0.02
1	0	0.21	0	cv	1	Vespa occidentalis	bombus fulvida	21	0.00	0.03	0.00	0.03
1	0	0.20	0	t	1	Psithyrus insularis	Potentilla arguta	21	0.00	0.03	0.00	0.02
1	0	0.23	0	t	1	Syrphus disgregus	Osmia pusilla	21	0.00	0.02	0.00	0.02
1	0	0.17	0	t	1	Pseudomasaris vespoides	Draba aurea	21	0.00	0.03	0.00	0.04
0	0	0.22	0	cv	1	Potentilla gracilis	Synanthedon albicornis	21	0.00	0.02	0.00	0.03
1	0	0.23	0	t	1	Caltha leptosepala	Protothyreopus dilectus	21	0.00	0.01	0.00	0.03
1	0	0.25	0	t	1	Petalostemon candidus	Tabanus rhombicus	21	0.01	0.02	0.00	0.03
1	1	0.58	0	t	1	Pseudomasaris vespoides	Chamaenerium angustifolium	21	0.00	0.03	0.04	0.02
1	0	0.20	0	cv	1	bombus dorsalis	Melissodes sp.	21	0.00	0.03	0.00	0.03
1	0	0.28	0	t	1	Anthomyia sp.	Trichopticus septentrionalis	21	0.00	0.00	0.00	0.03
1	0	0.23	0	t	1	Temnostoma aequale	Pyrameis atlanta	21	0.00	0.03	0.01	0.03
1	0	0.22	0	t	1	Tetrachrysis lauta	Panzeria radicum	21	0.00	0.02	0.00	0.03
1	0	0.18	0	cv	1	Osmia wardiana	bombus dorsalis	21	0.00	0.03	0.00	0.03
1	0	0.34	0	t	1	Andrena crataegi	Halictus pulzenus	21	0.00	0.03	0.02	0.03
1	0	0.23	0	t	1	Celerio lineata	Bracon helena	21	0.00	0.02	0.00	0.02
1	0	0.20	0	t	1	Melitaea sp.	Osmia rohweri	21	0.00	0.03	0.00	0.02

0	0	0.23	0	cv	1	Onagra biennis	Andrena prunorum	21	0.00	0.02	0.00	0.03
0	0	0.26	0	t	1	Gilia aggregata	Andrena canadensis	21	0.01	0.02	0.00	0.02
1	0	0.20	0	t	1	Syritta pipiens	Glypta sp.	21	0.00	0.02	0.00	0.03
1	0	0.21	0	t	1	Andrena crataegi	Basilarchia weidemeyeri	21	0.00	0.03	0.00	0.03
1	0	0.25	0	cv	1	Monumetha argentifrons	Pseudomelecta californica miranda	21	0.00	0.03	0.01	0.02
1	0	0.24	0	t	1	Megachile wootoni calogaster	Argynnis eurynome	21	0.01	0.03	0.01	0.03
1	0	0.34	0	t	1	Asclepias halli	bombus edwardsi	21	0.01	0.02	0.01	0.03
1	0	0.24	0	t	1	Syrphus opinator	Lappula floribunda	21	0.01	0.03	0.00	0.02
1	0	0.24	0	cv	1	Osmia fulgida	Odynerus annulatus	21	0.01	0.03	0.00	0.02
0	0	0.24	0	t	1	Andrena gardineri	Rubus deliciosus	21	0.00	0.03	0.00	0.02
1	0	0.21	0	t	1	Andrena lewisi	Nomada sp.	21	0.00	0.03	0.00	0.02
1	0	0.19	0	t	1	Anthrax lateralis	Melissodes aurigenia	21	0.00	0.03	0.00	0.03
1	0	0.27	0	cv	1	Polygonum bistorta	bombus dorsalis	21	0.01	0.02	0.00	0.03
1	0	0.29	0	t	1	Colletes kincaidi	Halictus chloralictus	21	0.01	0.03	0.01	0.03
1	0	0.23	0	t	1	Colletes kincaidi	Andrena canadensis	21	0.01	0.03	0.00	0.02
1	0	0.21	0	t	1	Achillea millefolium	Tabanus rhombicus	21	0.01	0.03	0.00	0.03
1	0	0.31	0	cv	1	Pompiloides sp.	bombus edwardsi	21	0.01	0.02	0.01	0.03
1	0	0.27	0	t	1	Osmia wardiana	Rydbergia grandilfora	21	0.00	0.03	0.01	0.02
1	0	0.18	0	t	1	bombus hunti	Melitaea whitneyi	21	0.00	0.03	0.00	0.03
1	0	0.22	0	t	1	Melissodes fremonti	Chrysotoxum upsilon	21	0.00	0.03	0.00	0.01
1	0	0.21	0	cv	1	Colletes kincaidi	Prosopis cressoni	21	0.01	0.03	0.00	0.03
1	0	0.22	0	t	1	Melitaea sp.	Tenthredella unicincta	21	0.00	0.03	0.00	0.02
0	1	0.51	0	t	1	Melitaea whitneyi	Mentzelia multiflora	21	0.00	0.03	0.03	0.03
1	0	0.17	0	t	1	Prosopis	Glypta sp.	21	0.00	0.03	0.00	0.03
0	0	0.39	0	cv	1	Leptura chrysocoma	Mertensia pratensis	21	0.00	0.01	0.01	0.02
1	0	0.18	0	t	1	Osmia wardiana	Anthrax alternata	21	0.00	0.03	0.00	0.03

0	0	0.37	0	t	1	Thalictrum fendleri	Mertensia pratensis	21	0.00	0.02	0.01	0.02
1	0	0.22	0	t	1	Epalpus	Heringia salix	21	0.00	0.03	0.00	0.03
1	0	0.20	0	cv	1	Andrena crataegi	Coelioxys moesta	21	0.00	0.03	0.00	0.03
1	0	0.26	0	t	1	Megachile wootoni	Andrena vicina	21	0.00	0.03	0.01	0.03
1	0	0.27	0	t	1	Calliphora vomitoria	Colletes sieverti	21	0.01	0.01	0.00	0.03
1	0	0.33	0	t	1	Oreochrysum parryi	Aquilegia brevistyla	21	0.01	0.01	0.00	0.02
1	0	0.23	0	cv	1	Colletes kincaidi	Erebia epipsodea	21	0.01	0.03	0.01	0.03
1	0	0.18	0	t	1	Epeolus helianthi	Dasyllis fernaldi	21	0.00	0.03	0.00	0.03
0	0	0.19	0	t	1	Colias alexandra	Trypeta occidentalis	21	0.00	0.03	0.00	0.03
1	0	0.38	0	t	1	bombus rufocinctus	Holodiscus dumosus	21	0.01	0.03	0.01	0.02
1	0	0.30	0	cv	1	Ctenucha sp.	Holodiscus dumosus	21	0.01	0.03	0.01	0.02
0	0	0.28	0	t	1	Penstemon unilateralis	Heringia salix	21	0.01	0.02	0.00	0.03
1	0	0.21	0	t	1	Bracon helena	Lithargus apicalis opuntiae	21	0.00	0.02	0.00	0.02
0	0	0.30	0	t	1	Campanula rotundifolia	Taraxacum officinale	21	0.01	0.02	0.00	0.02
1	0	0.25	0	cv	1	Gnophaela vermiculata	Tetrachrysis lauta	21	0.01	0.03	0.00	0.02
1	0	0.24	0	t	1	Megachile wootoni calogaster	Chrysopsis villosa	21	0.01	0.03	0.00	0.02
1	0	0.22	0	t	1	Andrena gardineri	Panurginus sp.	21	0.00	0.03	0.00	0.03
1	0	0.21	0	t	1	Volucella satur	Nathalis iole	21	0.00	0.02	0.00	0.03
1	0	0.23	0	cv	1	Microdon cothurnatus	Eristalis latifrons	21	0.00	0.02	0.00	0.03
1	0	0.31	0	t	1	Holodiscus dumosus	Echinomyia decias	21	0.01	0.02	0.00	0.01
1	0	0.35	0	t	1	bombus rufocinctus	Protoparce quinquemacu latus	21	0.01	0.03	0.00	0.00
1	0	0.31	0	t	1	Eristalis flavipes	Sphecodes sophiae	21	0.01	0.02	0.01	0.03
1	0	0.35	0	cv	1	Brachyopa notata	Potentilla pulcherrima	21	0.00	0.02	0.02	0.02
1	0	0.25	0	t	1	bombus centralis	Tenthredella unicincta	21	0.01	0.03	0.00	0.02

1	0	0.33	0	t	1	Linaria vulgaris	Pseudomelecta californica miranda	21	0.01	0.02	0.01	0.02
1	0	0.21	0	t	1	Triepeolus helianthi grandior	Pseudomasaris vespoides	21	0.00	0.02	0.00	0.03
0	0	0.25	0	cv	1	Psithyrus insularis	Anthomyia sp.	21	0.00	0.03	0.00	0.00
1	0	0.21	0	t	1	bombus juxtus	Taraxacum officinale	21	0.00	0.03	0.00	0.02
1	0	0.28	0	t	1	Heracleum lanatum	Bracon vulgaris	21	0.01	0.02	0.00	0.02
1	0	0.29	0	t	1	Polygonum bistorta	Oxybelus sp.	21	0.01	0.02	0.00	0.02
1	0	0.22	0	cv	1	Colletes sp. 2	Acmaeops longicornis	21	0.00	0.03	0.01	0.03
1	0	0.23	0	t	1	Myopa clausa	Echinomyia decias	21	0.00	0.03	0.00	0.01
1	0	0.39	0	t	1	Specomyia vittata	Mertensia pratensis	21	0.00	0.01	0.01	0.02
1	0	0.29	0	t	1	Lappula floribunda	Andrena vicina	21	0.00	0.02	0.01	0.03
0	0	0.31	0	cv	1	Gentiana parryi	Rydbergia grandilfora	21	0.00	0.02	0.01	0.02
0	1	0.64	0	t	1	Megachile pugnata	Iris missouriensis	21	0.00	0.03	0.05	0.04
1	0	0.22	0	t	1	bombus nevadensis	Coelioxys moesta	21	0.01	0.03	0.00	0.03
1	0	0.23	0	t	1	Peleteria robusta	Osmia sp.	21	0.00	0.02	0.01	0.03
1	0	0.18	0	cv	1	Prosopis cressoni	Prosopis tridentula	21	0.00	0.03	0.00	0.03
1	0	0.42	0	t	1	Xylota angustiventris	Eristalis tenax	21	0.02	0.02	0.00	0.03
1	0	0.20	0	t	1	Colletes sieverti	Coenonympha pamphiloides	21	0.00	0.03	0.00	0.03
0	1	0.71	0	t	1	Heracleum lanatum	Rosa acicularis	21	0.01	0.02	0.04	0.02
0	0	0.22	0	cv	1	Colias alexandra	Geranium richardsoni	21	0.00	0.03	0.00	0.02
1	0	0.23	0	t	1	Agapostemon sp.	Chrysopsis villosa	21	0.00	0.02	0.00	0.02
1	0	0.23	0	t	1	Andronicus sp.	Agapostemon sp.	21	0.01	0.03	0.00	0.02
1	0	0.22	0	t	1	Prunus virginiana	Parnassius clodius	21	0.00	0.02	0.00	0.03
1	0	0.36	0	cv	1	Andrena sp.	Penstemon secundiflorus	21	0.00	0.03	0.02	0.02
0	0	0.26	0	t	1	Potentilla gracilis	Pyrameis atlanta	21	0.00	0.02	0.01	0.03

1	1	0.50	0	t	1	Erizoa olivalis	Anthrax lateralis	21	0.04	0.03	0.00	0.03
1	0	0.21	0	t	1	Xylota	Nathalis iole	21	0.00	0.03	0.00	0.03
1	0	0.36	0	cv	1	Aragalus lamberti	Pyrameis atlanta	21	0.01	0.01	0.01	0.03
1	0	0.29	0	t	1	Ctenucha sp.	Scutellaria resinosa	21	0.01	0.03	0.01	0.01
1	0	0.25	0	t	1	Ophion sp.	Valeriana edulis	21	0.00	0.01	0.00	0.02
1	0	0.34	0	t	1	Halictus fendleri	Monumetha albifrons	21	0.00	0.02	0.02	0.03
1	0	0.27	0	cv	1	Paragus bicolor	Titusella pronitens	21	0.00	0.00	0.00	0.03
1	0	0.27	0	t	1	Ctenucha sp.	Pompiloides sp.	21	0.01	0.03	0.01	0.02
1	0	0.20	0	t	1	Andrena washingtoni	Volucella satur	21	0.00	0.03	0.00	0.02
1	0	0.23	0	t	1	Eristalis arbustorum	Megachile wootoni calogaster	21	0.00	0.03	0.01	0.03
1	0	0.25	0	cv	1	Prosopis wootoni	Draba aurea	21	0.02	0.03	0.00	0.04
1	0	0.36	0	t	1	Silene halli	Polygonum bistorta	21	0.01	0.02	0.01	0.02
1	0	0.21	0	t	1	Apocynum androsaemifolium	Solenius sp.	21	0.00	0.02	0.00	0.03
0	0	0.35	0	t	1	Zodion pygmaeum	Malvastrum coccineum	21	0.01	0.01	0.00	0.02
1	0	0.31	0	cv	1	Halictus sp.	Chrysogsater parva	21	0.01	0.03	0.00	0.02
1	0	0.25	0	t	1	Ancistrocerus sp 2.	Taraxacum officinale	21	0.01	0.03	0.00	0.02
1	0	0.24	0	t	1	Ancistrocerus sp 2.	Tetraopes sp.	21	0.01	0.03	0.00	0.02
1	0	0.20	0	t	1	Monumetha argentifrons	Brachyopa notata	21	0.00	0.03	0.00	0.02
0	1	0.69	0	cv	1	Silene acaulis	Chamaenerium angustifolium	21	0.00	0.00	0.04	0.02
1	0	0.36	0	t	1	Nathalis iole	Andrena placida	21	0.00	0.03	0.02	0.02
1	0	0.26	0	t	1	Syrphus americanus	Penstemon halli	21	0.00	0.03	0.01	0.02
1	0	0.22	0	t	1	Halictus lerouxi	Anthrax fulviana	21	0.00	0.02	0.00	0.02
1	0	0.37	0	cv	1	Andrena edwiniae	Astragalus drummondi	21	0.01	0.02	0.01	0.02
1	0	0.31	0	t	1	Ibidium strictum	Echinomyia algens	21	0.01	0.02	0.00	0.03

1	0	0.38	0	t	1	Stanleya pinnatifida	Acmaeops longicornis	21	0.02	0.02	0.01	0.03
1	0	0.25	0	t	1	bombus nevadensis	Microdon cothurnatus	21	0.01	0.03	0.00	0.02
0	0	0.25	0	cv	1	Thermopsis montana	Colias scudderi	21	0.00	0.02	0.01	0.03
1	0	0.21	0	t	1	Anthophora simillima	Osmia propinqua	21	0.00	0.03	0.01	0.03
1	0	0.35	0	t	1	Prosopis episcopalis	Andrena vicina	21	0.01	0.03	0.01	0.03
1	0	0.34	0	t	1	Penstemon	Silene acaulis	21	0.01	0.02	0.00	0.00
0	0	0.25	0	cv	1	Echinomyia decias	Erigeron uniflorus	21	0.00	0.01	0.00	0.02
1	0	0.27	0	t	1	bombus dorsalis	Andrena vicina	21	0.00	0.03	0.01	0.03
1	0	0.24	0	t	1	Brachyopa notata	Phacelia heterophylla	21	0.00	0.02	0.00	0.02
1	0	0.21	0	t	1	Trichius affinis	Anthrax nigra	21	0.00	0.03	0.00	0.03
0	0	0.48	0	cv	1	Chrysogsater parva	Carduus hookerianus	21	0.00	0.02	0.03	0.02
1	0	0.20	0	t	1	Halictus (Lasioglossum) sp.	Chrysogsater parva	21	0.00	0.03	0.00	0.02
0	1	0.51	0	t	1	bombus dorsalis	Mentzelia multiflora	21	0.00	0.03	0.03	0.03
1	0	0.39	0	t	1	Agapostemon coloradensis	Colias edwardsi	21	0.02	0.02	0.01	0.03
0	0	0.20	0	cv	1	Halicyoides mamus	Trypeta occidentalis	21	0.01	0.03	0.00	0.03
1	0	0.26	0	t	1	Leptura chrysocoma	Osmia coloradella	21	0.00	0.01	0.00	0.03
1	0	0.20	0	t	1	Sphaerophoria cylindrica	Exoprosopa divisa	21	0.00	0.03	0.00	0.02
1	0	0.35	0	t	1	Pentstemon glaucus	Coenonymph a pamphiloides	21	0.01	0.02	0.00	0.03
1	0	0.22	0	cv	1	Volucella rufomaculata	Trichodes ornatus	21	0.00	0.03	0.00	0.03
1	0	0.19	0	t	1	Psithyrus ashtoni	Trichopticus septentrional is	21	0.00	0.03	0.00	0.03
1	0	0.21	0	t	1	Osmia phaceliae	Tenthredella unicincta	21	0.00	0.03	0.00	0.02
1	0	0.36	0	t	1	Prunus pennsylvanica	Halictus pulzenus	21	0.00	0.02	0.02	0.03
1	0	0.20	0	cv	1	Prosopis sp.	Monumetha argentifrons	21	0.00	0.03	0.00	0.03
1	0	0.22	0	t	1	Syrphus americanus	Mesogramma marginata	21	0.00	0.03	0.01	0.02
1	1	0.66	0	t	1	Agathis vulgaris	Monumetha albifrons	21	0.04	0.04	0.02	0.03

1	0	0.21	0	t	1	Ctenucha sp.	Halictus medionitens	21	0.01	0.03	0.00	0.03
1	0	0.26	0	cv	1	Anthophora simillima	Polygonum bistorta	21	0.00	0.03	0.01	0.02
1	0	0.21	0	t	1	Halictus manitonellus	Vespa occidentalis	21	0.00	0.03	0.00	0.03
1	0	0.22	0	t	1	Peleteria robusta	Megachile relativa	21	0.00	0.02	0.01	0.03
1	0	0.20	0	t	1	Osmia brevis	Coelioxys moesta	21	0.00	0.02	0.00	0.03
1	0	0.19	0	cv	1	Psithyrus insularis	Phormia regina	21	0.00	0.03	0.00	0.03
0	1	0.55	0	t	1	Pentstemon gracilis	Helianthus petiolaris	21	0.04	0.02	0.00	0.02
1	0	0.43	0	t	1	Halictus (Lasioglossum) sp.	Carduus hookerianus	21	0.00	0.03	0.03	0.02
1	0	0.23	0	t	1	Brachyopa notata	Anthophora bomboides	21	0.00	0.02	0.00	0.02
1	0	0.22	0	cv	1	Gonochrysis densa	Megachile pugnata	21	0.00	0.02	0.00	0.03
1	0	0.27	0	t	1	Heriades gracilior	Sieversia turbinata	21	0.00	0.03	0.00	0.00
0	0	0.28	0	t	1	Erebia epipsodea	Protoparce quinquemaculatus	21	0.01	0.03	0.00	0.00
1	0	0.37	0	t	1	Asclepias halli	Andrena vicina	21	0.01	0.02	0.01	0.03
0	0	0.49	0	cv	1	Chrysophanus sirius	Potentilla gracilis	21	0.03	0.03	0.00	0.02
1	0	0.24	0	t	1	Chrysophanus helloides	Nomada sp.	21	0.00	0.02	0.00	0.02
1	0	0.23	0	t	1	Satyrus charon	Omalus sp.	21	0.00	0.03	0.00	0.02
1	0	0.27	0	t	1	Linaria	Ceratina sp.	21	0.01	0.02	0.00	0.03
1	0	0.22	0	cv	1	Bombylius atriceps	Onagra biennis	21	0.00	0.03	0.00	0.02
1	0	0.24	0	t	1	Linaria vulgaris	Megachile gemula albula	21	0.01	0.02	0.00	0.03
1	0	0.32	0	t	1	Andrena braccata	Osmia nigrifrons	21	0.02	0.03	0.00	0.03
1	0	0.23	0	t	1	Melitaea whitneyi	Physaria didymocarpa	21	0.00	0.03	0.00	0.02
1	0	0.21	0	cv	1	Eristalis dimidiatus	Vespa germanica	21	0.00	0.02	0.00	0.03
1	0	0.22	0	t	1	Prunus demissa	Anthophora neomexicana	21	0.00	0.02	0.00	0.03
1	0	0.20	0	t	1	Tradescantia virginiana	Sphex vulgaris	21	0.00	0.02	0.00	0.03
1	1	0.51	0	t	1	Mentzelia multiflora	Eristalis latifrons	21	0.03	0.03	0.00	0.03

1	0	0.33	0	cv	1	Syrphus opinator	Prosopis wootoni	21	0.01	0.03	0.02	0.03
1	0	0.21	0	t	1	Eristalis meigeni	Panurginus cressoniellus	21	0.00	0.02	0.00	0.03
1	0	0.21	0	t	1	Triepeolus helianthi grandior	Melissodes aurigenia	21	0.00	0.02	0.00	0.03
0	0	0.27	0	t	1	Aster foliaceus	Achillea millefolium	21	0.00	0.02	0.01	0.03
1	0	0.17	0	cv	1	bombus hunti	Sphex vulgaris	21	0.00	0.03	0.00	0.03
0	0	0.29	0	t	1	Thanaos propertius	Rydbergia grandilfora	21	0.00	0.03	0.01	0.02
1	0	0.34	0	t	1	Halictus sp.	Hammersch midtia ferruginea	21	0.01	0.03	0.01	0.03
1	0	0.26	0	t	1	Pompiloides sp.	Heriades gracilior	21	0.01	0.02	0.00	0.03
1	0	0.29	0	cv	1	Volucella rufomaculata	Scutellaria resinosa	21	0.00	0.03	0.01	0.01
1	0	0.20	0	t	1	Ectemius muricatus	Chrysotoxum ventricosum	21	0.00	0.03	0.00	0.03
1	0	0.21	0	t	1	Helianthus petiolaris	Pseudomasar is vespoides	21	0.00	0.02	0.00	0.03
1	1	0.52	0	t	1	Erizoa olivalis	Argynnis eurynome	21	0.04	0.03	0.01	0.03
1	0	0.24	0	cv	1	Panurginus sp.	Theventimtia muricatus	21	0.00	0.03	0.00	0.02
1	0	0.45	0	t	1	bombus occidentalis	Osmia coloradella	21	0.03	0.03	0.00	0.03
1	0	0.31	0	t	1	Archytas sp.	Pseudomelec ta californica miranda	21	0.00	0.01	0.01	0.02
1	0	0.21	0	t	1	Brenthis helena	bombus fulvida	21	0.00	0.02	0.00	0.03
1	0	0.37	0	cv	1	Penstemon unilateralis	Andrena vicina	21	0.01	0.02	0.01	0.03
0	0	0.17	0	t	1	Chionobis macouni	Anthrax alternata	21	0.00	0.04	0.00	0.03
1	0	0.20	0	t	1	Osmia fulgida	Colletes sieverti	21	0.01	0.03	0.00	0.03
1	0	0.21	0	t	1	Volucella satur	Anthrax hypomelas	21	0.00	0.02	0.00	0.03
1	0	0.29	0	cv	1	Apocynum androsaemifol ium	Halictus chloralictus	21	0.00	0.02	0.01	0.03
1	0	0.22	0	t	1	Osmia sp.	Lygus pratensis	21	0.01	0.03	0.00	0.03
1	0	0.18	0	t	1	Sphaerophoria cylindrica	Andrena prunorum	21	0.00	0.03	0.00	0.03

1	0	0.23	0	t	1	Monumetha argentifrons	Gilia aggregata	21	0.00	0.03	0.01	0.02
1	0	0.21	0	cv	1	Andrena apacheorum	Volucella rufomaculata	21	0.00	0.03	0.00	0.03
1	0	0.20	0	t	1	Megachile gemula albula	Brenthis helena	21	0.00	0.03	0.00	0.02
1	0	0.32	0	t	1	Ectemius muricatus	Prosopis coloradensis	21	0.00	0.03	0.02	0.03
1	0	0.20	0	t	1	Eristalis temporalis	Psithyrus consultus	21	0.00	0.03	0.00	0.03
1	0	0.26	0	cv	1	bombus centralis	Potentilla gracilis	21	0.01	0.03	0.00	0.02
1	0	0.21	0	t	1	Scaba opinator	Osmia hypoleuca	21	0.00	0.03	0.00	0.03
1	0	0.23	0	t	1	Eristalis arbustorum	Tenthredella unicincta	21	0.00	0.03	0.00	0.02
1	0	0.28	0	t	1	Anthophora bomboides	Exoprosopa volucris	21	0.00	0.02	0.00	0.00
0	0	0.32	0	cv	1	Syrphus disgregus	Calochortus gunnisoni	21	0.00	0.02	0.01	0.02
1	0	0.32	0	t	1	Chrysophanus rubidus	Pieris sisymbri	21	0.00	0.03	0.01	0.03
1	0	0.24	0	t	1	Chrysotoxum upsilon	Acmaeops pratensis	21	0.00	0.01	0.00	0.03
1	0	0.23	0	t	1	Oxybelus sp.	Phacelia heterophylla	21	0.00	0.02	0.00	0.02
1	0	0.25	0	cv	1	Anthrax sinuosa	Exoprosopa divisa	21	0.00	0.02	0.00	0.02
1	0	0.26	0	t	1	Vespa germanica	Penstemon halli	21	0.00	0.03	0.01	0.02
0	0	0.21	0	t	1	Colias keewaydina	Halicyoides mamus	21	0.00	0.03	0.01	0.03
1	0	0.21	0	t	1	Tenthredella unicincta	Tabanus rhombicus	21	0.00	0.02	0.00	0.03
1	0	0.26	0	cv	1	Mesogramma marginata	Ptilodexia harpasa	21	0.01	0.02	0.00	0.02
1	0	0.35	0	t	1	Theventimtia muricatus	Mertensia pratensis	21	0.00	0.02	0.01	0.02
1	0	0.31	0	t	1	Nomada sp.	Rydbergia grandilfora	21	0.00	0.02	0.01	0.02
0	0	0.22	0	t	1	Apocynum androsaemifol ium	Ectemius montanus	21	0.00	0.02	0.00	0.02
1	0	0.40	0	cv	1	Halictus sp.	Rydbergia grandilfora	21	0.01	0.03	0.01	0.02
1	0	0.23	0	t	1	Anthrax hypomelas	Acmaeops longicornis	21	0.00	0.03	0.01	0.03
1	0	0.33	0	t	1	Sieversia ciliata	Osmia propinqua	21	0.01	0.02	0.01	0.03
1	0	0.22	0	t	1	Thanaos martialis	Nathalis iole	21	0.01	0.03	0.00	0.03

1	0	0.23	0	cv	1	Colias scudderi	Xylota flavitibia	21	0.01	0.03	0.00	0.03
1	0	0.26	0	t	1	Prosopis sp.	Monarda fistulosa	21	0.00	0.03	0.01	0.02
1	0	0.41	0	t	1	Prosopis wootoni	Andrena madronitens	21	0.02	0.03	0.02	0.03
1	0	0.26	0	t	1	Colias edwardsi	Aconitum columbianum	21	0.01	0.03	0.00	0.02
0	0	0.21	0	cv	1	Halictus lerouxi	Parnassius smintheus	21	0.00	0.02	0.00	0.04
1	0	0.23	0	t	1	Ctenucha sp.	Osmia propinqua	21	0.01	0.03	0.01	0.03
0	0	0.16	0	t	1	Osmia bella	Lygus pratensis	21	0.00	0.04	0.00	0.03
1	0	0.22	0	t	1	bombus nevadensis	Curtipogon leucozona	21	0.01	0.03	0.00	0.03
0	0	0.30	0	cv	1	Agapostemon splendens	Pachylophus caespitosus	21	0.01	0.03	0.00	0.00
1	0	0.21	0	t	1	Psithyrus fernaldae	Osmia rohweri	21	0.00	0.03	0.00	0.02
1	0	0.19	0	t	1	Osmia bruneri	Psithyrus consultus	21	0.00	0.03	0.00	0.03
1	0	0.28	0	t	1	bombus centralis	bombus fervidus	21	0.01	0.03	0.01	0.03
1	0	0.40	0	cv	1	Xylota angustiventris	Ectemius montanus	21	0.02	0.02	0.00	0.02
1	0	0.30	0	t	1	Prosopis sp.	Calliphora vomitoria	21	0.00	0.03	0.01	0.01
1	0	0.33	0	t	1	Oreochrysum parryi	Argynnis eurynome	21	0.01	0.01	0.01	0.03
1	0	0.20	0	t	1	Osmia brevis	Syrphus americanus	21	0.00	0.02	0.00	0.03
1	0	0.23	0	cv	1	Sedum stenopetalum	Osmia coloradella	21	0.00	0.02	0.00	0.03
1	0	0.25	0	t	1	Epalpus bicolor	Halictus fendleri	21	0.00	0.03	0.00	0.02
1	0	0.21	0	t	1	Dasyllis fernaldi	Anthophora smithi	21	0.00	0.03	0.00	0.03
1	0	0.27	0	t	1	Dasyphora fruticosa	Apis mellifica	21	0.01	0.02	0.00	0.03
1	0	0.34	0	cv	1	Taraxacum officinale	Andrena madronitens	21	0.00	0.02	0.02	0.03
1	0	0.26	0	t	1	Polygonum bistorta	Prosopis basalis	21	0.01	0.02	0.00	0.03
1	0	0.24	0	t	1	Elephantella groenlandica	Ancistrocerus sp 2.	21	0.00	0.02	0.01	0.03
1	1	0.51	0	t	1	Erizoa olivalis	Psithyrus consultus	21	0.04	0.03	0.00	0.03
0	0	0.24	0	cv	1	Monumetha argentifrons	Pachylophus caespitosus	21	0.00	0.03	0.00	0.00
1	0	0.29	0	t	1	Syritta pipiens	Osmia densa	21	0.00	0.02	0.01	0.03

1	0	0.35	0	t	1	Melitaea whitneyi	Andrena placida	21	0.00	0.03	0.02	0.02
1	0	0.36	0	t	1	Petalostemon purpureus	Specomyia vittata	21	0.01	0.02	0.00	0.01
1	0	0.24	0	cv	1	Eliphilus latifrons	Phormia camillus	21	0.00	0.02	0.00	0.02
0	1	0.51	0	t	1	Cleome serrulata	Erigeron macranthus	21	0.01	0.02	0.02	0.02
1	0	0.21	0	t	1	Brenthis triclaris	bombus morrisoni	21	0.00	0.03	0.00	0.03
0	0	0.23	0	t	1	Celerio lineata	Psammophila violacepennis	21	0.00	0.02	0.00	0.03
1	0	0.20	0	cv	1	Colletes kincaidi	Monumetha argentifrons	21	0.01	0.03	0.00	0.03
0	0	0.23	0	t	1	Prosopis sp.	Sedum stenopetalum	21	0.00	0.03	0.00	0.02
1	0	0.21	0	t	1	Lathyrus ornatus	Vespa germanica	21	0.00	0.02	0.00	0.03
1	0	0.26	0	t	1	Oxybelus sp.	Sphecodes sophiae	21	0.00	0.02	0.01	0.03
1	0	0.27	0	cv	1	Caltha leptosepala	Ctenucha sp.	21	0.00	0.01	0.01	0.03
1	0	0.26	0	t	1	Ectemius muricatus	Hyloicus separatus	21	0.00	0.03	0.00	0.00
0	0	0.27	0	t	1	Aquilegia coerulea	Potentilla gracilis	21	0.00	0.02	0.00	0.02
1	0	0.21	0	t	1	Argynnis eurynome	bombus pennsylvanic us	21	0.01	0.03	0.00	0.03
1	0	0.20	0	cv	1	Solenius sp.	Megachile pugnata	21	0.00	0.03	0.00	0.03
1	0	0.23	0	t	1	Ancistrocerus sp 2.	Lithargus apicalis opuntiae	21	0.01	0.03	0.00	0.02
1	0	0.25	0	t	1	Andronicus sp.	Syrphus opinator	21	0.01	0.03	0.01	0.03
1	0	0.25	0	t	1	Erigeron uniflorus	Acmaeops longicornis	21	0.00	0.02	0.01	0.03
0	0	0.32	0	cv	1	Caltha leptosepala	Cleome serrulata	21	0.00	0.01	0.01	0.02
0	0	0.27	0	t	1	Parnassius smintheus	Holodiscus dumosus	21	0.00	0.04	0.01	0.02
1	0	0.24	0	t	1	Dolichovespul a arctica	Microdon cothurnatus	21	0.01	0.03	0.00	0.02
1	0	0.23	0	t	1	Syrphus opinator	bombus morrisoni	21	0.01	0.03	0.00	0.03
0	1	0.52	0	cv	1	Galium boreale	Pedicularis racemosa	21	0.00	0.02	0.03	0.02
1	0	0.27	0	t	1	Penstemon unilateralis	Scaba opinator	21	0.01	0.02	0.00	0.03

1	0	0.24	0	t	1	Drymocallis fissa	Megachile relativa	21	0.00	0.02	0.01	0.03
1	0	0.22	0	t	1	Volucella rufomaculata	Epalpus rufus	21	0.00	0.03	0.00	0.03
1	0	0.22	0	cv	1	bombus juxtus	Galium boreale	21	0.00	0.03	0.00	0.02
0	0	0.33	0	t	1	Erysimum asperum	Mertensia pratensis	21	0.00	0.02	0.01	0.02
1	0	0.31	0	t	1	Prosopis varifrons	bombus centralis	21	0.01	0.03	0.01	0.03
1	0	0.27	0	t	1	Xylota nigra	Titusella pronitens	21	0.01	0.03	0.00	0.03
0	0	0.40	0	cv	1	Erigeron macranthus	bombus pennsylvanicus	21	0.02	0.02	0.00	0.03
1	0	0.19	0	t	1	Prosopis basalis	bombus kirbyellus	21	0.00	0.03	0.00	0.03
1	0	0.18	0	t	1	bombus juxtus	Melitaea whitneyi	21	0.00	0.03	0.00	0.03
1	0	0.22	0	t	1	Bracon	Osmia pusilla	21	0.00	0.02	0.00	0.02
0	0	0.48	0	cv	1	Chrysotoxum ventricosum	Pedicularis racemosa	21	0.00	0.03	0.03	0.02
1	0	0.21	0	t	1	Psithyrus fernaldae	Systoechus vulgaris	21	0.00	0.03	0.00	0.03
0	0	0.25	0	t	1	Holodiscus dumosus	Draba aurea	21	0.01	0.02	0.00	0.04
1	0	0.25	0	t	1	Chrysophanus rubidus	Echinomyia decias	21	0.00	0.03	0.00	0.01
1	0	0.21	0	cv	1	Anthrax tegminipennis	Apis mellifica	21	0.00	0.02	0.00	0.03
1	0	0.21	0	t	1	Andrena crataegi	Mordella melaena	21	0.00	0.03	0.00	0.03
1	0	0.23	0	t	1	Tetrachrysis lauta	Osmia hypoleuca	21	0.00	0.02	0.00	0.03
1	0	0.24	0	t	1	Vespa germanica	Sphecodes sophiae	21	0.00	0.03	0.01	0.03
1	0	0.21	0	cv	1	Psithyrus fernaldae	Trichius affinis	21	0.00	0.03	0.00	0.03
1	0	0.23	0	t	1	Dasyllis fernaldi	Acmaeops longicornis	21	0.00	0.03	0.01	0.03
1	0	0.21	0	t	1	Syritta pipiens	Monumetha argentifrons	21	0.00	0.02	0.00	0.03
1	0	0.30	0	t	1	Anthrax alternata	Andrena madronitens	21	0.00	0.03	0.02	0.03
1	0	0.20	0	cv	1	Curtipogon leucozona	Osmia nigrifrons	21	0.00	0.03	0.00	0.03
1	0	0.21	0	t	1	Andrena lewisi	Bombomelecta fulvida	21	0.00	0.03	0.00	0.03
1	0	0.21	0	t	1	Ancistrocerus sp 2.	Osmia phaceliae	21	0.01	0.03	0.00	0.03

0	0	0.29	0	t	1	Monarda fistulosa	Rubus deliciosus	21	0.01	0.02	0.00	0.02
1	0	0.22	0	cv	1	Microdon cothurnatus	Titusella pronitens	21	0.00	0.02	0.00	0.03
1	0	0.27	0	t	1	Psithyrus fernaldae	Silene halli	21	0.00	0.03	0.01	0.02
0	1	0.51	0	t	1	Pentstemon gracilis	Synanthedon albicornis	21	0.04	0.02	0.00	0.03
1	0	0.31	0	t	1	Celerio lineata	Halictus chloralictus	21	0.00	0.02	0.01	0.03
1	0	0.16	0	cv	1	Cenis uhleri	Draba aurea	21	0.00	0.03	0.00	0.04
1	0	0.41	0	t	1	Leptura chrysocoma	Geum rivale	21	0.00	0.01	0.01	0.01
1	0	0.37	0	t	1	Silene halli	Xylota nigra	21	0.01	0.02	0.01	0.03
1	0	0.19	0	t	1	Basilarchia weidemeyeri	Dolichovespu la diabolica	21	0.00	0.03	0.00	0.03
1	0	0.23	0	cv	1	Chrysotoxum integrum	Coenonymph a pamphiloides	21	0.00	0.03	0.00	0.03
1	0	0.24	0	t	1	Linaria vulgaris	Anthophora occidentalis	21	0.01	0.02	0.00	0.03
0	1	0.66	0	t	1	Eristalis tenax	Iris missouriensis	21	0.00	0.03	0.05	0.04
1	0	0.23	0	t	1	Colletes kincaidi	Syrphus nitens	21	0.01	0.03	0.00	0.02
0	0	0.47	0	cv	1	Solidago missouriensis	Pieris sisymbri	21	0.02	0.02	0.01	0.03
1	0	0.25	0	t	1	Scutellaria resinosa	Colletes sieverti	21	0.01	0.01	0.00	0.03
1	0	0.18	0	t	1	Halictus (Lasioglossum) sp.	Scaba opinator	21	0.00	0.03	0.00	0.03
1	0	0.21	0	t	1	Melitaea sp.	Prunus virginiana	21	0.00	0.03	0.00	0.02
0	0	0.50	0	cv	1	Microdon cothurnatus	Mertensia sibirica	21	0.00	0.02	0.03	0.02
0	0	0.24	0	t	1	Irbisia brachycerus	Protoparce quinquemacu latus	21	0.00	0.03	0.00	0.00
1	0	0.20	0	t	1	Myopa clausa	Arctophila flagrans	21	0.00	0.03	0.00	0.03
0	0	0.29	0	t	1	Paragus bicolor	Helianthus petiolaris	21	0.00	0.00	0.00	0.02
1	0	0.35	0	cv	1	Chilosia petulca	Chrysophanu s helloides	21	0.01	0.01	0.00	0.02
1	0	0.22	0	t	1	Eristalis meigeni	Erynnis leonardus snowi	21	0.00	0.02	0.00	0.03
1	0	0.24	0	t	1	Prosopis sp.	Osmia fulgida	21	0.00	0.03	0.01	0.03
1	0	0.49	0	t	1	Rubus strigosus	Echinomyia decias	21	0.03	0.02	0.00	0.01

1	0	0.23	0	cv	1	Colias edwardsi	Lygus pratensis	21	0.01	0.03	0.00	0.03
0	0	0.18	0	t	1	Protothyreopus dilectus	Psammophila violacepennis	21	0.00	0.03	0.00	0.03
1	0	0.30	0	t	1	Prosopis varifrons	Argynnis atlantis	21	0.01	0.03	0.01	0.03
0	1	0.51	0	t	1	Aquilegia coerulea	Rubus strigosus	21	0.00	0.02	0.03	0.02
1	0	0.30	0	cv	1	Volucella satur	Capnoides aureum	21	0.00	0.02	0.01	0.02
1	0	0.26	0	t	1	Prosopis varifrons	Megachile texana	21	0.01	0.03	0.00	0.03
1	0	0.27	0	t	1	Tradescantia virginiana	Lycaena pseudargiolus	21	0.00	0.02	0.01	0.03
1	0	0.35	0	t	1	Aragalus lamberti	Triepeolus helianthi grandior	21	0.01	0.01	0.00	0.02
1	0	0.23	0	cv	1	Andrena lewisi	Osmia pentstemonis	21	0.00	0.03	0.01	0.03
1	0	0.34	0	t	1	Aquilegia coerulea	Pieris sisymbri	21	0.00	0.02	0.01	0.03
0	0	0.29	0	t	1	Clementsia rhodantha	Pentstemon glaber	21	0.00	0.00	0.00	0.02
1	0	0.19	0	t	1	Panurginus cressoniellus	Ectemius montanus	21	0.00	0.03	0.00	0.02
1	0	0.29	0	cv	1	Scutellaria resinosa	Bembex spinolae	21	0.01	0.01	0.00	0.02
0	0	0.44	0	t	1	Eristalis temporalis	Carduus hookerianus	21	0.00	0.03	0.03	0.02
1	0	0.35	0	t	1	Pachylophus caespitosus	Scutellaria resinosa	21	0.00	0.00	0.01	0.01
1	0	0.22	0	t	1	Ectemius muricatus	Anthidium tenuiflorae	21	0.00	0.03	0.00	0.02
1	0	0.23	0	cv	1	Atrytone taxiles	Thanaos propertius	21	0.01	0.03	0.00	0.03
1	0	0.34	0	t	1	Halictus lerouxi	Chilosia petulca	21	0.00	0.02	0.01	0.01
0	0	0.36	0	t	1	Clementsia rhodantha	Penstemon unilateralis	21	0.00	0.00	0.01	0.02
1	0	0.32	0	t	1	Anthrax nigra	Prosopis coloradensis	21	0.00	0.03	0.02	0.03
1	0	0.41	0	cv	1	Halictus medionitens	Erigeron macranthus	21	0.00	0.03	0.02	0.02
1	0	0.25	0	t	1	bombus bifarius	Pyrameis atlanta	21	0.01	0.03	0.01	0.03
1	0	0.27	0	t	1	Pachylophus caespitosus	Ectemius montanus	21	0.00	0.00	0.00	0.02
1	0	0.21	0	t	1	Psithyrus ashtoni	Tenthredella unicincta	21	0.00	0.03	0.00	0.02

1	0	0.32	0	cv	1	Allium recurvatum	Prosopis elliptica	21	0.00	0.02	0.02	0.03
1	0	0.23	0	t	1	Lathyrus ornatus	Bembex spinolae	21	0.00	0.02	0.00	0.02
1	0	0.26	0	t	1	Caltha leptosepala	Bombomelecta fulvida	21	0.00	0.01	0.00	0.03
1	0	0.27	0	t	1	Colletes sp. 2	Xylota nigra	21	0.00	0.03	0.01	0.03
1	0	0.22	0	cv	1	Osmia rohweri	Psithyrus consultus	21	0.00	0.02	0.00	0.03
1	0	0.35	0	t	1	Silene acaulis	Pseudomelecta californica miranda	21	0.00	0.00	0.01	0.02
1	0	0.20	0	t	1	Frasera speciosa	Sphaerophoria cylindrica	21	0.00	0.02	0.00	0.03
1	0	0.19	0	t	1	Mallota flavoterminata	Oxybelus sp.	21	0.00	0.03	0.00	0.02
1	0	0.20	0	cv	1	Osmia megacephala	bombus kirbyellus	21	0.00	0.03	0.00	0.03
1	0	0.24	0	t	1	Aquilegia brevistyla	Brenthis helena	21	0.00	0.02	0.00	0.02
0	0	0.28	0	t	1	Erysimum asperum	Cleome serrulata	21	0.00	0.02	0.01	0.02
1	0	0.25	0	t	1	Phorbia fusciceps	Erebia epipsodea	21	0.01	0.03	0.01	0.03
1	0	0.30	0	cv	1	Silene acaulis	Ancistrocerus sp 2.	21	0.00	0.00	0.01	0.03
1	0	0.22	0	t	1	Tetrachrysis lauta	Lemonias nubigena wheeleri	21	0.00	0.02	0.00	0.03
1	0	0.23	0	t	1	Pipiza vanduzeei	Megachile vidua	21	0.00	0.03	0.01	0.03
1	0	0.25	0	t	1	Thalictrum fendleri	Osmia coloradella	21	0.00	0.02	0.00	0.03
1	0	0.18	0	cv	1	Anthophora simillima	Psithyrus insularis	21	0.00	0.03	0.00	0.03
1	0	0.32	0	t	1	Solidago missouriensis	Tabanus rhombicus	21	0.02	0.02	0.00	0.03
1	0	0.22	0	t	1	Megachile wootoni calogaster	Andrena prunorum	21	0.01	0.03	0.00	0.03
1	1	0.59	0	t	1	Agapostemon coloradensis	Erigeron macranthus	21	0.02	0.02	0.02	0.02
1	0	0.22	0	cv	1	Panurginus sp.	Colias alexandra	21	0.00	0.03	0.00	0.03
1	0	0.22	0	t	1	Osmia pentstemonis	Scaba opinator	21	0.01	0.03	0.00	0.03
1	0	0.32	0	t	1	Osmia phaceliae	Geum rivale	21	0.00	0.03	0.01	0.01
1	0	0.49	0	t	1	Rubus strigosus	Megachile wootoni calogaster	21	0.03	0.02	0.01	0.03

1	0	0.24	0	cv	1	Thanaos martialis	Celerio lineata	21	0.01	0.03	0.00	0.02
1	0	0.25	0	t	1	Arctostaphylus uva-ursi	Chrysophanus helloides	21	0.00	0.02	0.00	0.02
1	0	0.25	0	t	1	Drymocallis fissa	Osmia sp.	21	0.00	0.02	0.01	0.03
0	0	0.43	0	t	1	Penstemon secundiflorus	Sieversia turbinata	21	0.02	0.02	0.00	0.00
0	0	0.32	0	cv	1	Silene acaulis	Lycaena pseudargiolus	21	0.00	0.00	0.01	0.03
0	0	0.32	0	t	1	Prunus demissa	Calochortus gunnisoni	21	0.00	0.02	0.01	0.02
1	0	0.22	0	t	1	Volucella satur	Fragaria vesca	21	0.00	0.02	0.00	0.02
1	0	0.26	0	t	1	Colias edwardsi	Megachile wootoni calogaster	21	0.01	0.03	0.01	0.03
1	0	0.21	0	cv	1	Lycaena glaucon	Tabanus rhombicus	21	0.00	0.02	0.00	0.03
0	0	0.32	0	t	1	Ibidium strictum	Phacelia heterophylla	21	0.01	0.02	0.00	0.02
0	1	0.53	0	t	1	bombus fervidus	Rubus strigosus	21	0.01	0.03	0.03	0.02
1	0	0.31	0	t	1	Melitaea whitneyi	Pentstemon glaucus	21	0.00	0.03	0.01	0.02
1	0	0.25	0	cv	1	Lathyrus ornatus	Syrphus opinator	21	0.00	0.02	0.01	0.03
1	0	0.20	0	t	1	Trichodes ornatus	Titusella pronitens	21	0.00	0.03	0.00	0.03
1	0	0.26	0	t	1	Prosopis cressoni	Specomyia vittata	21	0.00	0.03	0.00	0.01
1	0	0.19	0	t	1	Sphaerophoria cylindrica	Anthrax fulviana	21	0.00	0.03	0.00	0.02
1	0	0.22	0	cv	1	Apocynum androsaemifolium	Xylota flavitibia	21	0.00	0.02	0.00	0.03
1	0	0.34	0	t	1	Ctenucha sp.	Chilosia petulca	21	0.01	0.03	0.01	0.01
1	0	0.30	0	t	1	Osmia rohweri	Rydbergia grandilfora	21	0.00	0.02	0.01	0.02
1	0	0.43	0	t	1	Penstemon	Geum rivale	21	0.01	0.02	0.01	0.01
0	0	0.27	0	cv	1	Paragus bicolor	Lemonias nubigena wheeleri	21	0.00	0.00	0.00	0.03
1	0	0.23	0	t	1	Anthrax nigra	Megachile vidua	21	0.00	0.03	0.01	0.03
1	0	0.31	0	t	1	Nathalis iole	Andrena madronitens	21	0.00	0.03	0.02	0.03
1	0	0.23	0	t	1	Trichius affinis	Eristalis tenax	21	0.00	0.03	0.00	0.03

1	0	0.37	0	cv	1	Solidago missouriensis	Anthidium tenuiflorae	21	0.02	0.02	0.00	0.02
1	0	0.27	0	t	1	Chrysotoxum upsilon	bombus americanorum	21	0.00	0.01	0.00	0.02
1	0	0.22	0	t	1	Anthrax	Tetraopes sp.	21	0.00	0.03	0.00	0.02
1	0	0.23	0	t	1	bombus centralis	Apis mellifica	21	0.01	0.03	0.00	0.03
1	0	0.22	0	cv	1	Foenus perplexus	Acmaeops longicornis	21	0.00	0.03	0.01	0.03
0	1	0.56	0	t	1	Cleome serrulata	Rubus strigosus	21	0.01	0.02	0.03	0.02
1	0	0.23	0	t	1	Ectemius montanus	Odynerus annulatus	21	0.00	0.02	0.00	0.02
1	0	0.18	0	t	1	Protothyreopus dilectus	Anthophora neomexicana	21	0.00	0.03	0.00	0.03
1	0	0.47	0	cv	1	Silene halli	bombus appositus	21	0.01	0.02	0.02	0.03
1	0	0.25	0	t	1	Aquilegia coerulea	Brachyopa notata	21	0.00	0.02	0.00	0.02
1	0	0.35	0	t	1	Anthidium tenuiflorae	Mertensia pratensis	21	0.00	0.02	0.01	0.02
1	0	0.30	0	t	1	Lithospermum canescens	Halictus chloralictus	21	0.00	0.02	0.01	0.03
1	0	0.22	0	cv	1	Basilarchia weidemeyeri	Brenthis helena	21	0.00	0.03	0.00	0.02
1	0	0.20	0	t	1	Parnassius smintheus	Heriades gracilior	21	0.00	0.04	0.00	0.03
1	0	0.34	0	t	1	Potentilla gracilis	Prosopis elliptica	21	0.00	0.02	0.02	0.03
1	0	0.23	0	t	1	Prosopis sp.	Chrysogsater parva	21	0.00	0.03	0.00	0.02
1	0	0.23	0	cv	1	Ptilodexia harpasa	Osmia pusilla	21	0.00	0.02	0.00	0.02
1	0	0.34	0	t	1	Halictus (Evylaeus) sp.	Exoprosopa volucris	21	0.01	0.03	0.00	0.00
1	0	0.23	0	t	1	Andronicus sp.	Allium recurvatum	21	0.01	0.03	0.00	0.02
1	0	0.31	0	t	1	Petalostemon purpureus	Chrysogsater parva	21	0.01	0.02	0.00	0.02
0	0	0.22	0	cv	1	Drymocallis fissa	Curtipogon leucozona	21	0.00	0.02	0.00	0.03
1	0	0.23	0	t	1	Colias keewaydina	Trifolium dasyphyllum	21	0.00	0.03	0.00	0.02
1	0	0.27	0	t	1	Caltha leptosepala	Dolichovespula arctica	21	0.00	0.01	0.01	0.03
0	0	0.35	0	t	1	Drymocallis fissa	Astragalus drummondi	21	0.00	0.02	0.01	0.02
1	0	0.22	0	cv	1	Andrena lewisi	Celerio lineata	21	0.00	0.03	0.00	0.02

0	0	0.22	0	t	1	Colias keewaydina	Aconitum columbianum	21	0.00	0.03	0.00	0.02
1	0	0.21	0	t	1	Megachile relativa	Psammophila violacepennis	21	0.01	0.03	0.00	0.03
1	0	0.48	0	t	1	Chrysophanus sirius	Megachile wootoni calogaster	21	0.03	0.03	0.01	0.03
1	0	0.30	0	cv	1	Campanula rotundifolia	Dolichovespu la arctica	21	0.01	0.02	0.01	0.03
0	1	0.74	0	t	1	Geranium caespitosum	Holodiscus dumosus	21	0.05	0.02	0.01	0.02
1	0	0.28	0	t	1	Calliphora vomitoria	Vespa germanica	21	0.01	0.01	0.00	0.03
1	0	0.31	0	t	1	Onagra biennis	Pseudomelec ta californica miranda	21	0.00	0.02	0.01	0.02
0	0	0.20	0	cv	1	Elephantella groenlandica	Selasphorus platycercus	21	0.00	0.02	0.00	0.04
0	0	0.28	0	t	1	Asclepias halli	Melissodes fremonti	21	0.01	0.02	0.00	0.03
1	0	0.47	0	t	1	Lemonias nubigena wheeleri	Pedicularis racemosa	21	0.00	0.03	0.03	0.02
1	0	0.20	0	t	1	Chrysotoxum ventricosum	Titusella pronitens	21	0.00	0.03	0.00	0.03
1	0	0.22	0	cv	1	bombus kirbyellus	Microdon cothurnatus	21	0.00	0.03	0.00	0.02
0	0	0.25	0	t	1	Pyrameis atlanta	Aconitum columbianum	21	0.01	0.03	0.00	0.02
1	0	0.48	0	t	1	Chrysophanus sirius	Pyrameis atlanta	21	0.03	0.03	0.01	0.03
1	0	0.21	0	t	1	Psithyrus fernaldae	Tetrachrysis lauta	21	0.00	0.03	0.00	0.02
0	0	0.48	0	cv	1	Erysimum asperum	Chrysophanu s sirius	21	0.00	0.02	0.03	0.03
0	0	0.23	0	t	1	Fragaria vesca	Nomada sp.	21	0.00	0.02	0.00	0.02
1	0	0.30	0	t	1	Ibidium strictum	Anthrax lateralis	21	0.01	0.02	0.00	0.03
1	0	0.21	0	t	1	Tenthredella flavomarginis	Osmia brevis	21	0.00	0.03	0.00	0.02
1	0	0.27	0	cv	1	Thermopsis montana	Lycaena pseudargiolu s	21	0.00	0.02	0.01	0.03
1	0	0.18	0	t	1	Coelioxys moesta	Dolichovespu la diabolica	21	0.00	0.03	0.00	0.03
1	0	0.35	0	t	1	Panurginus sp.	Potentilla pulcherrima	21	0.00	0.03	0.02	0.02
1	0	0.36	0	t	1	Andronicus sp.	Agapostemon coloradensis	21	0.01	0.03	0.02	0.02

1	0	0.28	0	cv	1	Oreochrysum parryi	Cenis uhleri	21	0.01	0.01	0.00	0.03
0	0	0.25	0	t	1	Paragus bicolor	Psithyrus insularis	21	0.00	0.00	0.00	0.03
1	0	0.40	0	t	1	Asclepias halli	Sambucus racemosa	21	0.01	0.02	0.01	0.01
1	0	0.30	0	t	1	Tetrachrysis lauta	Xylota nigra	21	0.00	0.02	0.01	0.03
1	0	0.42	0	cv	1	Chrysophanus sirius	Epeolus helianthi	21	0.03	0.03	0.00	0.03
1	0	0.22	0	t	1	Gonochrysis densa	Osmia albolateralis	21	0.00	0.02	0.00	0.02
1	0	0.23	0	t	1	Galium boreale	Parnassius clodius	21	0.00	0.02	0.00	0.03
1	0	0.42	0	t	1	Calochortus gunnisoni	Andrena madronitens	21	0.01	0.02	0.02	0.03
1	0	0.50	0	cv	1	Mentzelia multiflora	bombus kirbyellus	21	0.03	0.03	0.00	0.03
1	0	0.18	0	t	1	Syrphus amalopis	Parnassius smintheus	21	0.00	0.03	0.00	0.04
0	0	0.19	0	t	1	Eristalis temporalis	Parnassius smintheus	21	0.00	0.03	0.00	0.04
1	1	0.53	0	t	1	Volucella satur	Mentzelia multiflora	21	0.00	0.02	0.03	0.03
1	0	0.34	0	cv	1	Lycaena pseudargiolus	Cerastomia lignaria	21	0.01	0.03	0.01	0.03
0	1	0.67	0	t	1	Halictus fendleri	Rosa acicularis	21	0.00	0.02	0.04	0.02
1	0	0.30	0	t	1	Pentstemon glaber	Halictus (Evylaeus) sp.	21	0.00	0.02	0.01	0.03
1	0	0.20	0	t	1	Mordella melaena	Geocoris bullatus	21	0.00	0.03	0.00	0.03
1	0	0.25	0	cv	1	Galium boreale	Microdon cothurnatus	21	0.00	0.02	0.00	0.02
0	1	0.72	0	t	1	Anthomyia sp.	Rosa acicularis	21	0.00	0.00	0.04	0.02
1	0	0.35	0	t	1	Dodocatheon meadia	Chilosia petulca	21	0.00	0.02	0.01	0.01
1	0	0.32	0	t	1	Pieris protodice	Rydbergia grandilfora	21	0.00	0.02	0.01	0.02
1	0	0.19	0	cv	1	Monumetha argentifrons	Lemonias nubigena wheeleri	21	0.00	0.03	0.00	0.03
1	0	0.28	0	t	1	Pseudomelecta californica miranda	Megachile sp.	21	0.01	0.02	0.00	0.03
1	0	0.23	0	t	1	Prunus pennsylvanica	Anthophora bomboides	21	0.00	0.02	0.00	0.02
1	0	0.20	0	t	1	Pyrameis atlanta	Glypta sp.	21	0.01	0.03	0.00	0.03

1	0	0.21	0	cv	1	Clisodon sp.	Osmia hypoleuca	21	0.00	0.03	0.00	0.03
1	0	0.24	0	t	1	bombus edwardsi	Clisodon sp.	21	0.01	0.03	0.00	0.03
0	0	0.38	0	t	1	Heracleum lanatum	Petalostemon purpureus	21	0.01	0.02	0.01	0.02
0	0	0.27	0	t	1	Dodocatheon meadia	Gilia aggregata	21	0.00	0.02	0.01	0.02
1	0	0.21	0	cv	1	Melitaea sp.	Halictus lerouxi	21	0.00	0.03	0.00	0.02
1	0	0.19	0	t	1	bombus juxtus	Trichopticus septentrionalis	21	0.00	0.03	0.00	0.03
1	0	0.30	0	t	1	Sambucus racemosa	Phormia regina	21	0.01	0.01	0.00	0.03
0	1	0.63	0	t	1	Lathyrus ornatus	Chamaenerium angustifolium	21	0.00	0.02	0.04	0.02
1	0	0.35	0	cv	1	Sieversia ciliata	Atrytone taxiles	21	0.01	0.02	0.01	0.03
1	0	0.36	0	t	1	Arctostaphylus uva-ursi	Halictus pulzenus	21	0.00	0.02	0.02	0.03
0	0	0.23	0	t	1	Brachyopa notata	Delphinium scopulorum	21	0.00	0.02	0.00	0.02
1	1	0.89	0	t	1	Agathis vulgaris	Iris missouriensis	21	0.04	0.04	0.05	0.04
1	0	0.24	0	cv	1	Caltha leptosepala	Parnassius clodius	21	0.00	0.01	0.00	0.03
1	0	0.20	0	t	1	Andrena sp.	Colletes americanus	21	0.00	0.03	0.00	0.03
1	0	0.39	0	t	1	Andronicus sp.	Opulaster opulifolius	21	0.01	0.03	0.02	0.02
0	0	0.32	0	t	1	Pentstemon glaber	Pieris sisymbri	21	0.00	0.02	0.01	0.03
1	0	0.29	0	cv	1	Osmia densa	Ancistrocerus sp 2.	21	0.01	0.03	0.01	0.03
1	0	0.43	0	t	1	Pedicularis racemosa	Colletes sieverti	21	0.03	0.02	0.00	0.03
1	0	0.22	0	t	1	Andrena prunorum	Exoprosopa divisa	21	0.00	0.03	0.00	0.02
1	1	0.93	1	t	1	Adenocarpus viscosus	Osmia canariensis	22	0.06	0.05	0.05	0.05
0	1	0.90	1	cv	1	Adenocarpus viscosus	Geron hesperidon	22	0.06	0.05	0.04	0.06
0	1	0.83	1	t	1	Gasteruption sp.	Pimpinella cumbrae	22	0.02	0.07	0.08	0.07
0	0	0.36	1	t	1	Gasteruption sp.	Pterocephalus lasiospermus	22	0.02	0.07	0.03	0.05
0	1	0.84	1	t	1	Anthrax anthrax	Pimpinella cumbrae	22	0.02	0.06	0.08	0.07

0	0	0.37	1	cv	1	Anthrax anthrax	Pterocephalus lasiospermus	22	0.02	0.06	0.03	0.05
0	1	0.51	1	t	1	Anthrax anthrax	Echium wildpretii	22	0.02	0.06	0.04	0.05
0	1	0.59	1	t	1	Anthrax anthrax	Scrophularia glabrata	22	0.02	0.06	0.05	0.06
0	1	0.90	1	t	1	Cephalodromia sp.	Pimpinella cumbrae	22	0.03	0.06	0.08	0.07
0	1	0.71	1	cv	1	Cephalodromia sp.	Argyranthemum teneriffae	22	0.03	0.06	0.04	0.05
0	0	0.43	1	t	1	Cephalodromia sp.	Tolpis webbii	22	0.03	0.06	0.02	0.06
0	1	0.90	1	t	1	Lasioglossum viride	Pimpinella cumbrae	22	0.03	0.06	0.08	0.07
0	1	0.51	1	t	1	Lasioglossum viride	Pterocephalus lasiospermus	22	0.03	0.06	0.03	0.05
0	1	0.65	1	cv	1	Lasioglossum viride	Echium wildpretii	22	0.03	0.06	0.04	0.05
0	1	0.72	1	t	1	Lasioglossum viride	Scrophularia glabrata	22	0.03	0.06	0.05	0.06
0	1	0.77	1	t	1	Leptochilus eatoni	Pimpinella cumbrae	22	0.01	0.07	0.08	0.07
0	1	0.76	1	t	1	Pimpinella cumbrae	Linnaemyia soror	22	0.08	0.07	0.01	0.06
1	1	0.98	1	cv	1	Pimpinella cumbrae	Eristalis tenax	22	0.08	0.07	0.08	0.06
1	1	0.99	1	t	1	Pimpinella cumbrae	Lasioglossum actifrons	22	0.08	0.07	0.09	0.07
0	1	0.83	1	t	1	Pimpinella cumbrae	Apis mellifera	22	0.08	0.07	0.02	0.06
1	1	0.98	1	t	1	Pimpinella cumbrae	Dermasothes gracile	22	0.08	0.07	0.07	0.07
0	1	0.75	1	cv	1	Pimpinella cumbrae	Hyleaus canariensis	22	0.08	0.07	0.01	0.06
0	1	0.75	1	t	1	Pimpinella cumbrae	Euodynerus reflexus	22	0.08	0.07	0.01	0.06
0	1	0.94	1	t	1	Pimpinella cumbrae	Geron hesperidon	22	0.08	0.07	0.04	0.06
0	1	0.74	1	t	1	Pimpinella cumbrae	Nyctia lugubris	22	0.08	0.07	0.01	0.07
0	1	0.65	1	cv	1	Anastoechus latifrons	Nepeta teydea	22	0.04	0.05	0.02	0.04
0	1	0.69	1	t	1	Anastoechus latifrons	Pterocephalus lasiospermus	22	0.04	0.05	0.03	0.05
0	1	0.79	1	t	1	Anastoechus latifrons	Echium wildpretii	22	0.04	0.05	0.04	0.05

0	1	0.56	1	t	1	Anastoechus latifrons	Spartocytisus supranubius	22	0.04	0.05	0.01	0.04
0	1	0.61	1	cv	1	Anastoechus latifrons	Tolpis webbii	22	0.04	0.05	0.02	0.06
1	1	0.89	1	t	1	Anastoechus latifrons	Erysimum scoparium	22	0.04	0.05	0.05	0.05
0	1	0.59	1	t	1	Nepeta teydea	Anthidium manicatum	22	0.02	0.04	0.03	0.03
0	0	0.31	1	t	1	Nepeta teydea	Eucera gracilipes	22	0.02	0.04	0.01	0.05
0	0	0.27	1	cv	1	Nepeta teydea	Euodynerus reflexus	22	0.02	0.04	0.01	0.06
0	1	0.65	1	t	1	Nepeta teydea	Anthophora alluaudi	22	0.02	0.04	0.04	0.05
0	1	0.57	1	t	1	Serinus canarius	Echium wildpretii	22	0.02	0.05	0.04	0.05
0	0	0.44	1	t	1	Linnaemyia soror	Echium wildpretii	22	0.01	0.06	0.04	0.05
0	1	0.51	1	cv	1	Linnaemyia soror	Argyranthem um teneriffae	22	0.01	0.06	0.04	0.05
0	0	0.18	1	t	1	Linnaemyia soror	Mentha longifolia	22	0.01	0.06	0.01	0.06
0	1	0.72	1	t	1	Osmia canariensis	Pterocephalu s lasiospermus	22	0.05	0.05	0.03	0.05
1	1	0.91	1	t	1	Osmia canariensis	Erysimum scoparium	22	0.05	0.05	0.05	0.05
0	1	0.51	1	cv	1	Cyclyrius webbianus	Pterocephalu s lasiospermus	22	0.03	0.05	0.03	0.05
0	0	0.33	1	t	1	Cyclyrius webbianus	Mentha longifolia	22	0.03	0.05	0.01	0.06
0	1	0.79	1	t	1	Cyclyrius webbianus	Erysimum scoparium	22	0.03	0.05	0.05	0.05
0	0	0.36	1	t	1	Bibio elmoi	Mentha longifolia	22	0.03	0.04	0.01	0.06
0	0	0.48	1	cv	1	Lasioglossum chalcodes	Echium wildpretii	22	0.01	0.05	0.04	0.05
0	0	0.35	1	t	1	Lucilia sericata	Pterocephalu s lasiospermus	22	0.01	0.05	0.03	0.05
0	1	0.55	1	t	1	Lucilia sericata	Argyranthem um teneriffae	22	0.01	0.05	0.04	0.05
0	0	0.42	1	t	1	Estheria simonyi	Pterocephalu s lasiospermus	22	0.02	0.05	0.03	0.05
0	1	0.56	1	cv	1	Estheria simonyi	Echium wildpretii	22	0.02	0.05	0.04	0.05

0	0	0.26	1	t	1	Estheria simonyi	Mentha longifolia	22	0.02	0.05	0.01	0.06
0	0	0.39	1	t	1	Andrena wollestoni	Spartocytisus supranubius	22	0.03	0.04	0.01	0.04
0	0	0.44	1	t	1	Andrena wollestoni	Tolpis webbii	22	0.03	0.04	0.02	0.06
0	1	0.89	1	cv	1	Pterocephalus lasiospermus	Eristalis tenax	22	0.03	0.05	0.08	0.06
0	1	0.60	1	t	1	Pterocephalus lasiospermus	Scaeva albomaculata	22	0.03	0.05	0.04	0.06
0	0	0.42	1	t	1	Pterocephalus lasiospermus	Megachile canariensis	22	0.03	0.05	0.02	0.05
0	0	0.40	1	t	1	Pterocephalus lasiospermus	Apis mellifera	22	0.03	0.05	0.02	0.06
0	1	0.66	1	cv	1	Pterocephalus lasiospermus	Geron hesperidon	22	0.03	0.05	0.04	0.06
0	1	0.50	1	t	1	Pterocephalus lasiospermus	Melecta curvispina	22	0.03	0.05	0.03	0.05
1	1	0.93	1	t	1	Eristalis tenax	Echium wildpretii	22	0.08	0.06	0.04	0.05
0	1	0.78	1	t	1	Eristalis tenax	Mentha longifolia	22	0.08	0.06	0.01	0.06
0	1	0.84	1	cv	1	Eristalis tenax	Tolpis webbii	22	0.08	0.06	0.02	0.06
0	1	0.78	1	t	1	Scaeva albomaculata	Scrophularia glabrata	22	0.04	0.06	0.05	0.06
0	1	0.57	1	t	1	Megachile canariensis	Echium wildpretii	22	0.02	0.05	0.04	0.05
0	0	0.30	1	t	1	Megachile canariensis	Spartocytisus supranubius	22	0.02	0.05	0.01	0.04
0	0	0.35	1	cv	1	Megachile canariensis	Tolpis webbii	22	0.02	0.05	0.02	0.06
0	1	0.74	1	t	1	Megachile canariensis	Erysimum scoparium	22	0.02	0.05	0.05	0.05
0	0	0.47	1	t	1	Echium wildpretii	Macroglossu m stellatarum	22	0.04	0.05	0.01	0.05
0	1	0.56	1	t	1	Echium wildpretii	Tachinidae sp.	22	0.04	0.05	0.02	0.05
0	1	0.53	1	cv	1	Echium wildpretii	Apis mellifera	22	0.04	0.05	0.02	0.06
0	0	0.47	1	t	1	Echium wildpretii	Eucera gracilipes	22	0.04	0.05	0.01	0.05
0	0	0.46	1	t	1	Echium wildpretii	Phylloscopus collybita	22	0.04	0.05	0.01	0.05
0	0	0.42	1	t	1	Echium wildpretii	Hyleaus canariensis	22	0.04	0.05	0.01	0.06
0	1	0.88	1	cv	1	Echium wildpretii	Stomorhina lunata	22	0.04	0.05	0.06	0.05
0	1	0.63	1	t	1	Echium wildpretii	Melecta curvispina	22	0.04	0.05	0.03	0.05

0	1	0.79	1	t	1	Echium wildpretii	Anthophora alluaudi	22	0.04	0.05	0.04	0.05
0	0	0.42	1	t	1	Peleteria ruficornis	Mentha longifolia	22	0.03	0.04	0.01	0.06
0	1	0.85	1	cv	1	Lasioglossum actifrons	Mentha longifolia	22	0.09	0.07	0.01	0.06
1	1	0.97	1	t	1	Lasioglossum actifrons	Scrophularia glabrata	22	0.09	0.07	0.05	0.06
0	1	0.67	1	t	1	Macroglossum stellatarum	Erysimum scoparium	22	0.01	0.05	0.05	0.05
0	1	0.64	1	t	1	Tachinidae sp.	Argyranthemum teneriffae	22	0.02	0.05	0.04	0.05
0	1	0.61	1	cv	1	Apis mellifera	Argyranthemum teneriffae	22	0.02	0.06	0.04	0.05
0	0	0.28	1	t	1	Apis mellifera	Spartocytisus supranubius	22	0.02	0.06	0.01	0.04
0	1	0.71	1	t	1	Apis mellifera	Erysimum scoparium	22	0.02	0.06	0.05	0.05
0	0	0.24	1	t	1	Eucera gracilipes	Spartocytisus supranubius	22	0.01	0.05	0.01	0.04
0	1	0.67	1	cv	1	Eucera gracilipes	Erysimum scoparium	22	0.01	0.05	0.05	0.05
0	1	0.76	1	t	1	Argyranthemum teneriffae	Drosophila sp.	22	0.04	0.05	0.03	0.04
0	1	0.82	1	t	1	Argyranthemum teneriffae	Geron hesperidon	22	0.04	0.05	0.04	0.06
0	1	0.67	1	t	1	Tachysphex unicolor	Scrophularia glabrata	22	0.03	0.06	0.05	0.06
0	0	0.17	1	cv	1	Mentha longifolia	Euodynerus reflexus	22	0.01	0.06	0.01	0.06
0	1	0.54	1	t	1	Mentha longifolia	Tachina canariensis	22	0.01	0.06	0.04	0.05
0	1	0.68	1	t	1	Mentha longifolia	Stomorhina lunata	22	0.01	0.06	0.06	0.05
0	0	0.50	1	t	1	Mentha longifolia	Colletes dimidiatus	22	0.01	0.06	0.04	0.04
0	1	0.52	1	cv	1	Mentha longifolia	Anthophora alluaudi	22	0.01	0.06	0.04	0.05
0	0	0.20	1	t	1	Hyleaus canariensis	Spartocytisus supranubius	22	0.01	0.06	0.01	0.04
0	0	0.23	1	t	1	Hyleaus canariensis	Tolpis webbii	22	0.01	0.06	0.02	0.06
0	0	0.20	1	t	1	Euodynerus reflexus	Spartocytisus supranubius	22	0.01	0.06	0.01	0.04
0	0	0.23	1	cv	1	Euodynerus reflexus	Tolpis webbii	22	0.01	0.06	0.02	0.06
0	1	0.51	1	t	1	Euodynerus reflexus	Scrophularia glabrata	22	0.01	0.06	0.05	0.06

0	1	0.57	1	t	1	Geron hesperidon	Tolpis webbii	22	0.04	0.06	0.02	0.06
0	1	0.88	1	t	1	Geron hesperidon	Erysimum scoparium	22	0.04	0.06	0.05	0.05
0	1	0.54	1	cv	1	Spartocytisus supranubius	Colletes dimidiatus	22	0.01	0.04	0.04	0.04
0	1	0.56	1	t	1	Spartocytisus supranubius	Anthophora alluaudi	22	0.01	0.04	0.04	0.05
0	1	0.83	1	t	1	Scrophularia glabrata	Anthophora alluaudi	22	0.05	0.06	0.04	0.05
0	1	0.79	1	t	1	Melecta curvispina	Erysimum scoparium	22	0.03	0.05	0.05	0.05
0	1	0.88	1	cv	1	Erysimum scoparium	Anthophora alluaudi	22	0.05	0.05	0.04	0.05
0	1	0.54	0	t	1	Mentha longifolia	Scrophularia glabrata	22	0.01	0.06	0.05	0.06
0	1	0.76	0	t	1	Tachysphex unicolor	Erysimum scoparium	22	0.03	0.06	0.05	0.05
0	0	0.29	0	t	1	Lucilia sericata	Tachinidae sp.	22	0.01	0.05	0.02	0.05
0	1	0.74	0	cv	1	Bibio elmoi	Tachina canariensis	22	0.03	0.04	0.04	0.05
0	0	0.15	0	t	1	Linnaemyia soror	Nyctia lugubris	22	0.01	0.06	0.01	0.07
0	0	0.45	0	t	1	Bibio elmoi	Tolpis webbii	22	0.03	0.04	0.02	0.06
0	1	0.79	0	t	1	Pimpinella cumbrae	Macroglossu m stellatarum	22	0.08	0.07	0.01	0.05
0	0	0.37	0	cv	1	Tachysphex unicolor	Tolpis webbii	22	0.03	0.06	0.02	0.06
0	1	0.87	0	t	1	Scaeva albomaculata	Stomorhina lunata	22	0.04	0.06	0.06	0.05
0	0	0.25	0	t	1	Leptochilus eatoni	Nepeta teydea	22	0.01	0.07	0.02	0.04
0	0	0.45	0	t	1	Lasioglossum viride	Tachinidae sp.	22	0.03	0.06	0.02	0.05
0	1	0.70	0	cv	1	Cyclyrius webbianus	Anthophora alluaudi	22	0.03	0.05	0.04	0.05
0	1	0.60	0	t	1	Estheria simonyi	Geron hesperidon	22	0.02	0.05	0.04	0.06
0	1	0.72	0	t	1	Anastoechus latifrons	Andrena wollestoni	22	0.04	0.05	0.03	0.04
0	1	0.80	0	t	1	Anthidium manicatum	Scrophularia glabrata	22	0.03	0.03	0.05	0.06
0	0	0.39	0	cv	1	Hyleaus canariensis	Drosophila sp.	22	0.01	0.06	0.03	0.04
0	1	0.71	0	t	1	Bibio elmoi	Geron hesperidon	22	0.03	0.04	0.04	0.06
0	1	0.59	0	t	1	Echium wildpretii	Tachysphex unicolor	22	0.04	0.05	0.03	0.06

0	1	0.62	0	t	1	Pterocephalus lasiospermus	Echium wildpretii	22	0.03	0.05	0.04	0.05
0	0	0.41	0	cv	1	Anthidium manicatum	Nyctia lugubris	22	0.03	0.03	0.01	0.07
0	1	0.53	0	t	1	Lasioglossum viride	Cyclyrius webbianus	22	0.03	0.06	0.03	0.05
1	1	0.97	0	t	1	Eristalis tenax	Stomorhina lunata	22	0.08	0.06	0.06	0.05
0	1	0.83	0	t	1	Tachysphex unicolor	Dermasothes gracile	22	0.03	0.06	0.07	0.07
0	1	0.92	0	cv	1	Megachile canariensis	Lasioglossum actifrons	22	0.02	0.05	0.09	0.07
0	0	0.48	0	t	1	Lucilia sericata	Echium wildpretii	22	0.01	0.05	0.04	0.05
0	0	0.25	0	t	1	Mentha longifolia	Tolpis webbii	22	0.01	0.06	0.02	0.06
0	1	0.63	0	t	1	Tachinidae sp.	Anthophora alluaudi	22	0.02	0.05	0.04	0.05
0	0	0.38	0	cv	1	Lasioglossum viride	Spartocytisus supranubius	22	0.03	0.06	0.01	0.04
0	1	0.54	0	t	1	Anastoechus latifrons	Macroglossu m stellatarum	22	0.04	0.05	0.01	0.05
0	1	0.53	0	t	1	Megachile canariensis	Drosophila sp.	22	0.02	0.05	0.03	0.04
0	0	0.40	0	t	1	Pterocephalus lasiospermus	Tolpis webbii	22	0.03	0.05	0.02	0.06
0	1	0.52	0	cv	1	Peleteria ruficornis	Tolpis webbii	22	0.03	0.04	0.02	0.06
0	1	0.54	0	t	1	Anastoechus latifrons	Phylloscopus collybita	22	0.04	0.05	0.01	0.05
0	0	0.32	0	t	1	Cephalodromi a sp.	Euodynerus reflexus	22	0.03	0.06	0.01	0.06
0	1	0.84	0	t	1	Gasteruption sp.	Eristalis tenax	22	0.02	0.07	0.08	0.06
0	0	0.45	0	cv	1	Cephalodromi a sp.	Megachile canariensis	22	0.03	0.06	0.02	0.05
0	1	0.91	0	t	1	Tachina canariensis	Stomorhina lunata	22	0.04	0.05	0.06	0.05
0	0	0.28	0	t	1	Lucilia sericata	Tolpis webbii	22	0.01	0.05	0.02	0.06
0	1	0.92	0	t	1	Serinus canarius	Lasioglossum actifrons	22	0.02	0.05	0.09	0.07
0	0	0.23	0	cv	1	Leptochilus eatoni	Megachile canariensis	22	0.01	0.07	0.02	0.05
0	1	0.97	0	t	1	Lasioglossum actifrons	Tachina canariensis	22	0.09	0.07	0.04	0.05
0	1	0.65	0	t	1	Osmia canariensis	Tolpis webbii	22	0.05	0.05	0.02	0.06
0	0	0.34	0	t	1	Mentha longifolia	Melecta curvispina	22	0.01	0.06	0.03	0.05

0	1	0.70	0	cv	1	Melecta curvispina	Anthophora alluaudi	22	0.03	0.05	0.04	0.05
0	0	0.29	0	t	1	Lasioglossum chalcodes	Megachile canariensis	22	0.01	0.05	0.02	0.05
0	0	0.48	0	t	1	Lucilia sericata	Anthidium manicatum	22	0.01	0.05	0.03	0.03
0	1	0.91	0	t	1	Estheria simonyi	Lasioglossum actifrons	22	0.02	0.05	0.09	0.07
0	0	0.29	0	cv	1	Melecta curvispina	Nyctia lugubris	22	0.03	0.05	0.01	0.07
0	0	0.25	0	t	1	Tachinidae sp.	Euodynerus reflexus	22	0.02	0.05	0.01	0.06
0	1	0.66	0	t	1	Anthidium manicatum	Andrena wollestoni	22	0.03	0.03	0.03	0.04
0	0	0.14	0	t	1	Leptochilus eatoni	Nyctia lugubris	22	0.01	0.07	0.01	0.07
0	1	0.56	0	cv	1	Cephalodromia sp.	Bibio elmoi	22	0.03	0.06	0.03	0.04
1	1	0.73	0	t	1	Osmia canariensis	Cyclyrius webbianus	22	0.05	0.05	0.03	0.05
0	0	0.42	0	t	1	Anthidium manicatum	Euodynerus reflexus	22	0.03	0.03	0.01	0.06
0	1	0.52	0	t	1	Anastoechus latifrons	Mentha longifolia	22	0.04	0.05	0.01	0.06
0	1	0.82	0	cv	1	Anastoechus latifrons	Colletes dimidiatus	22	0.04	0.05	0.04	0.04
0	1	0.96	0	t	1	Echium wildpretii	Lasioglossum actifrons	22	0.04	0.05	0.09	0.07
0	0	0.16	0	t	1	Hyleaus canariensis	Euodynerus reflexus	22	0.01	0.06	0.01	0.06
0	0	0.29	0	t	1	Estheria simonyi	Eucera gracilipes	22	0.02	0.05	0.01	0.05
0	1	0.57	0	cv	1	Lucilia sericata	Tachina canariensis	22	0.01	0.05	0.04	0.05
0	0	0.36	0	t	1	Cyclyrius webbianus	Eucera gracilipes	22	0.03	0.05	0.01	0.05
0	1	0.73	0	t	1	Dermasothes gracile	Spartocytisus supranubius	22	0.07	0.07	0.01	0.04
0	1	0.96	0	t	1	Anthidium manicatum	Lasioglossum actifrons	22	0.03	0.03	0.09	0.07
0	0	0.27	0	cv	1	Apis mellifera	Eucera gracilipes	22	0.02	0.06	0.01	0.05
0	0	0.46	0	t	1	Scrophularia glabrata	Nyctia lugubris	22	0.05	0.06	0.01	0.07
0	1	0.91	0	t	1	Lasioglossum viride	Eristalis tenax	22	0.03	0.06	0.08	0.06
1	1	0.95	0	t	1	Eristalis tenax	Scrophularia glabrata	22	0.08	0.06	0.05	0.06
0	1	0.76	0	cv	1	Adenocarpus viscosus	Tolpis webbii	22	0.06	0.05	0.02	0.06

0	1	0.63	0	t	1	Anthrax anthrax	Osmia canariensis	22	0.02	0.06	0.05	0.05
1	1	0.77	0	t	1	Anastoechus latifrons	Drosophila sp.	22	0.04	0.05	0.03	0.04
0	1	0.84	0	t	1	Eristalis tenax	Apis mellifera	22	0.08	0.06	0.02	0.06
0	1	0.85	0	cv	1	Eristalis tenax	Megachile canariensis	22	0.08	0.06	0.02	0.05
0	1	0.53	0	t	1	Macroglossum stellatarum	Colletes dimidiatus	22	0.01	0.05	0.04	0.04
0	0	0.39	0	t	1	Estheria simonyi	Tachysphex unicolor	22	0.02	0.05	0.03	0.06
0	0	0.46	0	t	1	Andrena wollestoni	Tachinidae sp.	22	0.03	0.04	0.02	0.05
0	1	0.71	0	cv	1	Spartocytisus supranubius	Stomorhina lunata	22	0.01	0.04	0.06	0.05
0	1	0.84	0	t	1	Pimpinella cumbrae	Serinus canarius	22	0.08	0.07	0.02	0.05
0	0	0.47	0	t	1	Hyleaus canariensis	Colletes dimidiatus	22	0.01	0.06	0.04	0.04
0	1	0.94	0	t	1	Osmia canariensis	Dermasothes gracile	22	0.05	0.05	0.07	0.07
0	0	0.47	0	cv	1	Anthidium manicatum	Phylloscopus collybita	22	0.03	0.03	0.01	0.05
0	0	0.32	0	t	1	Linnaemyia soror	Cyclyrius webbianus	22	0.01	0.06	0.03	0.05
0	0	0.47	0	t	1	Leptochilus eatoni	Argyranthem um teneriffae	22	0.01	0.07	0.04	0.05
0	1	0.54	0	t	1	Anastoechus latifrons	Lucilia sericata	22	0.04	0.05	0.01	0.05
0	1	0.64	0	cv	1	Estheria simonyi	Tachina canariensis	22	0.02	0.05	0.04	0.05
0	1	0.69	0	t	1	Cephalodromi a sp.	Geron hesperidon	22	0.03	0.06	0.04	0.06
0	0	0.37	0	t	1	Megachile canariensis	Tachinidae sp.	22	0.02	0.05	0.02	0.05
0	0	0.49	0	t	1	Cephalodromi a sp.	Tachysphex unicolor	22	0.03	0.06	0.03	0.06
0	1	0.52	0	cv	1	Geron hesperidon	Spartocytisus supranubius	22	0.04	0.06	0.01	0.04
0	0	0.20	0	t	1	Eucera gracilipes	Hyleaus canariensis	22	0.01	0.05	0.01	0.06
0	1	0.83	0	t	1	Bibio elmoi	Stomorhina lunata	22	0.03	0.04	0.06	0.05
0	0	0.24	0	t	1	Estheria simonyi	Hyleaus canariensis	22	0.02	0.05	0.01	0.06
0	1	0.63	0	cv	1	Cyclyrius webbianus	Scaeva albomaculata	22	0.03	0.05	0.04	0.06
0	1	0.92	0	t	1	Dermasothes gracile	Tachina canariensis	22	0.07	0.07	0.04	0.05

0	0	0.44	0	t	1	Lasioglossum chalcodes	Drosophila sp.	22	0.01	0.05	0.03	0.04
0	1	0.86	0	t	1	Osmia canariensis	Argyranthemum teneriffae	22	0.05	0.05	0.04	0.05
0	0	0.40	0	cv	1	Peleteria ruficornis	Hyleaus canariensis	22	0.03	0.04	0.01	0.06
0	1	0.65	0	t	1	Serinus canarius	Scrophularia glabrata	22	0.02	0.05	0.05	0.06
0	1	0.62	0	t	1	Linnaemyia soror	Erysimum scoparium	22	0.01	0.06	0.05	0.05
0	1	0.71	0	t	1	Adenocarpus viscosus	Eucera gracilipes	22	0.06	0.05	0.01	0.05
0	1	0.84	0	cv	1	Tachina canariensis	Anthophora alluaudi	22	0.04	0.05	0.04	0.05
0	1	0.65	0	t	1	Serinus canarius	Tachina canariensis	22	0.02	0.05	0.04	0.05
0	1	0.89	0	t	1	Adenocarpus viscosus	Anthidium manicatum	22	0.06	0.05	0.03	0.03
0	1	0.57	0	t	1	Scaeva albomaculata	Tachysphex unicolor	22	0.04	0.06	0.03	0.06
0	1	0.52	0	cv	1	Estheria simonyi	Drosophila sp.	22	0.02	0.05	0.03	0.04
0	1	0.68	0	t	1	Gasteruption sp.	Erysimum scoparium	22	0.02	0.07	0.05	0.05
0	1	0.65	0	t	1	Euodynerus reflexus	Stomorhina lunata	22	0.01	0.06	0.06	0.05
1	1	0.73	1	t	1	Spilogona triangulifera	Diapensia lapponica	23	0.01	0.05	0.07	0.05
1	0	0.31	1	cv	1	Bartsia alpina	Bombus hyperboreus	23	0.01	0.02	0.01	0.03
1	1	0.66	1	t	1	Coenosia atritibia	Pinguicula alpina	23	0.03	0.03	0.02	0.03
1	1	0.75	1	t	1	Coenosia atritibia	Viola biflora	23	0.03	0.03	0.03	0.03
1	0	0.46	1	t	1	Coenosia atritibia	Potentilla crantzii	23	0.03	0.03	0.01	0.03
1	1	0.75	1	cv	1	Coenosia atritibia	Polygonum viviparum	23	0.03	0.03	0.03	0.03
1	0	0.47	1	t	1	Coenosia atritibia	Silene acaulis	23	0.03	0.03	0.01	0.03
1	1	0.74	1	t	1	Coenosia atritibia	Salix reticulata	23	0.03	0.03	0.03	0.04
1	1	0.60	1	t	1	Coenosia atritibia	Cerastium alpinun	23	0.03	0.03	0.02	0.03
1	0	0.50	1	cv	1	Coenosia atritibia	Cassiope hypniodes	23	0.03	0.03	0.01	0.03
1	0	0.45	1	t	1	Coenosia atritibia	Saxifraga aizoides	23	0.03	0.03	0.01	0.04
0	0	0.32	1	t	1	Phygadenon sp.	Cerastium alpinun	23	0.01	0.03	0.02	0.03

0	0	0.23	1	t	1	Megaselia coci	Dryas octopetala	23	0.01	0.03	0.01	0.03
1	1	0.73	1	cv	1	Corynoptera sp. 1	Diapensia lapponica	23	0.01	0.05	0.07	0.05
1	1	0.85	1	t	1	Diapensia lapponica	Zaphne barbiventris	23	0.07	0.05	0.02	0.05
1	1	0.78	1	t	1	Diapensia lapponica	Aclastus sp. 1	23	0.07	0.05	0.01	0.05
1	1	0.81	1	t	1	Diapensia lapponica	Megaselia digitalis	23	0.07	0.05	0.02	0.05
1	1	0.78	1	cv	1	Diapensia lapponica	Spilogona megastoma	23	0.07	0.05	0.01	0.05
1	1	0.93	1	t	1	Diapensia lapponica	Micropsectra sp. 2	23	0.07	0.05	0.04	0.05
1	1	0.81	1	t	1	Diapensia lapponica	Limnophilus sp.	23	0.07	0.05	0.02	0.05
1	1	0.72	1	t	1	Diapensia lapponica	Xestommaste r sp.	23	0.07	0.05	0.01	0.05
1	1	0.88	1	cv	1	Diapensia lapponica	Thysan. sp.	23	0.07	0.05	0.03	0.05
1	1	0.72	1	t	1	Diapensia lapponica	Arpedium sp.	23	0.07	0.05	0.01	0.05
1	1	0.72	1	t	1	Diapensia lapponica	Smittia aterrima	23	0.07	0.05	0.01	0.05
1	1	0.94	1	t	1	Diapensia lapponica	Corynoneura scutella	23	0.07	0.05	0.05	0.05
1	1	0.76	1	cv	1	Diapensia lapponica	Smittia betuletorum	23	0.07	0.05	0.01	0.05
1	1	0.71	1	t	1	Diapensia lapponica	Lycoriella curvispina	23	0.07	0.05	0.01	0.05
1	1	0.78	1	t	1	Diapensia lapponica	Dipt. sp. 1	23	0.07	0.05	0.01	0.05
1	1	0.71	1	t	1	Diapensia lapponica	Megaselia groenlandica	23	0.07	0.05	0.01	0.05
1	1	0.71	1	cv	1	Diapensia lapponica	Megaselia girandii	23	0.07	0.05	0.01	0.05
1	1	0.72	1	t	1	Diapensia lapponica	Rhamphomyi a obscuripennis	23	0.07	0.05	0.01	0.05
1	1	0.75	1	t	1	Diapensia lapponica	Pseudosmitti a oxoniana	23	0.07	0.05	0.01	0.05
1	1	0.71	1	t	1	Diapensia lapponica	Ichn. sp. 3	23	0.07	0.05	0.01	0.05
1	1	0.76	1	cv	1	Diapensia lapponica	Ichn. sp. 1	23	0.07	0.05	0.01	0.05
1	1	0.71	1	t	1	Diapensia lapponica	Ichn. sp. 4	23	0.07	0.05	0.01	0.05
1	1	0.83	1	t	1	Diapensia lapponica	Corynoptera sp. 2	23	0.07	0.05	0.02	0.05

1	1	0.71	1	t	1	Diapensia lapponica	Lycoriella sp. 1	23	0.07	0.05	0.01	0.05
0	0	0.33	1	cv	1	Alysiinae sp. 3	Saxifraga aizoides	23	0.02	0.02	0.01	0.04
1	0	0.27	1	t	1	Delai piliventris	Parnassia palustris	23	0.01	0.02	0.01	0.03
1	0	0.32	1	t	1	Zaphne barbiventris	Dryas octopetala	23	0.02	0.05	0.01	0.03
0	0	0.32	1	t	1	Zaphne barbiventris	Potentilla crantzii	23	0.02	0.05	0.01	0.03
1	1	0.60	1	cv	1	Zaphne barbiventris	Salix reticulata	23	0.02	0.05	0.03	0.04
1	0	0.45	1	t	1	Zaphne barbiventris	Cerastium alpinun	23	0.02	0.05	0.02	0.03
1	1	0.88	1	t	1	Zaphne barbiventris	Saxifraga oppositifolia	23	0.02	0.05	0.07	0.05
0	0	0.21	1	t	1	Rhaphium crassipes	Saxifraga aizoides	23	0.01	0.03	0.01	0.04
1	0	0.44	1	cv	1	Bombus alpinus	Rhododendro n lapponicum	23	0.02	0.05	0.02	0.04
0	0	0.30	1	t	1	Bombus alpinus	Potentilla crantzii	23	0.02	0.05	0.01	0.03
1	0	0.40	1	t	1	Bombus alpinus	Astragalus alpinus	23	0.02	0.05	0.02	0.04
1	1	0.87	1	t	1	Bombus alpinus	Saxifraga oppositifolia	23	0.02	0.05	0.07	0.05
0	1	0.54	1	cv	1	Bombus	Salix lanata	23	0.02	0.05	0.03	0.03
1	0	0.35	1	t	1	Heterotrissocl adius	Cassiope tetragona	23	0.01	0.01	0.01	0.01
1	0	0.24	1	t	1	Aclastus sp. 2	Silene acaulis	23	0.01	0.03	0.01	0.03
1	0	0.35	1	t	1	Aclastus sp. 1	Cerastium alpinun	23	0.01	0.05	0.02	0.03
0	0	0.22	1	cv	1	Aclastus sp. 1	Saxifraga aizoides	23	0.01	0.05	0.01	0.04
1	0	0.35	1	t	1	Bombus hyperboreus	Astragalus alpinus	23	0.01	0.03	0.02	0.04
0	0	0.21	1	t	1	Smittia sp. 2	Saxifraga aizoides	23	0.01	0.03	0.01	0.04
0	0	0.23	1	t	1	Empis lucida	Dryas octopetala	23	0.01	0.03	0.01	0.03
1	0	0.38	1	cv	1	Spilogona nitidicauda	Dryas octopetala	23	0.02	0.03	0.01	0.03
1	1	0.68	1	t	1	Spilogona nitidicauda	Viola biflora	23	0.02	0.03	0.03	0.03
1	0	0.40	1	t	1	Spilogona nitidicauda	Parnassia palustris	23	0.02	0.03	0.01	0.03
1	0	0.38	1	t	1	Spilogona nitidicauda	Potentilla crantzii	23	0.02	0.03	0.01	0.03
1	1	0.68	1	cv	1	Spilogona nitidicauda	Polygonum viviparum	23	0.02	0.03	0.03	0.03

1	1	0.52	1	t	1	Spilogona nitidicauda	Cerastium alpinun	23	0.02	0.03	0.02	0.03
1	0	0.26	1	t	1	Megaselia digitalis	Dryas octopetala	23	0.02	0.05	0.01	0.03
0	0	0.28	1	t	1	Megaselia digitalis	Parnassia palustris	23	0.02	0.05	0.01	0.03
1	0	0.27	1	cv	1	Megaselia digitalis	Silene acaulis	23	0.02	0.05	0.01	0.03
0	0	0.47	1	t	1	Pinguicula alpina	Rhamphomyia aethiops	23	0.02	0.03	0.01	0.03
0	1	0.65	1	t	1	Pinguicula alpina	Rhamphomyia morio	23	0.02	0.03	0.03	0.04
1	0	0.38	1	t	1	Pinguicula alpina	Chamaepsila morio	23	0.02	0.03	0.01	0.03
0	0	0.33	1	cv	1	Pinguicula alpina	Rhamphomyia obscuripennis	23	0.02	0.03	0.01	0.05
0	1	0.58	1	t	1	Pinguicula alpina	Thricops hirtulus	23	0.02	0.03	0.03	0.04
1	0	0.27	1	t	1	Acamptocladius	Parnassia palustris	23	0.01	0.02	0.01	0.03
0	0	0.29	1	t	1	Thricops furcatus	Potentilla crantzii	23	0.01	0.03	0.01	0.03
1	1	0.58	1	cv	1	Thricops furcatus	Polygonum viviparum	23	0.01	0.03	0.03	0.03
0	0	0.27	1	t	1	Thricops furcatus	Saxifraga aizoides	23	0.01	0.03	0.01	0.04
0	0	0.21	1	t	1	Limnophyes natalensis	Saxifraga aizoides	23	0.01	0.03	0.01	0.04
0	0	0.46	1	t	1	Dryas octopetala	Rhamphomyia morio	23	0.01	0.03	0.03	0.04
0	0	0.29	1	cv	1	Dryas octopetala	Anarta sp.	23	0.01	0.03	0.01	0.03
0	0	0.28	1	t	1	Dryas octopetala	Thricops rostratus	23	0.01	0.03	0.02	0.05
0	0	0.24	1	t	1	Dryas octopetala	Spilogona megastoma	23	0.01	0.03	0.01	0.05
0	0	0.23	1	t	1	Dryas octopetala	Bradysia sp. 3	23	0.01	0.03	0.01	0.03
0	0	0.32	1	cv	1	Dryas octopetala	Porizontini sp.	23	0.01	0.03	0.01	0.02
0	0	0.40	1	t	1	Dryas octopetala	Thysan. sp.	23	0.01	0.03	0.03	0.05
0	1	0.62	1	t	1	Dryas octopetala	Megaselia diversa	23	0.01	0.03	0.05	0.04
0	0	0.23	1	t	1	Dryas octopetala	Botanophilia bidens	23	0.01	0.03	0.01	0.03
0	0	0.19	1	cv	1	Dryas octopetala	Smittia aterrima	23	0.01	0.03	0.01	0.05

0	0	0.23	1	t	1	Dryas octopetala	Megaselia sordida	23	0.01	0.03	0.01	0.03
0	0	0.20	1	t	1	Dryas octopetala	Podabrus sp.	23	0.01	0.03	0.01	0.04
0	0	0.21	1	t	1	Dryas octopetala	Spilogona alpica	23	0.01	0.03	0.01	0.04
0	0	0.23	1	cv	1	Dryas octopetala	Chloropinae sp.	23	0.01	0.03	0.01	0.03
0	0	0.18	1	t	1	Dryas octopetala	Rhamphomyi a obscuripennis	23	0.01	0.03	0.01	0.05
0	0	0.23	1	t	1	Dryas octopetala	Phaonia lugubris	23	0.01	0.03	0.01	0.03
0	0	0.33	1	t	1	Dryas octopetala	Phaonia subfuscinerei s	23	0.01	0.03	0.02	0.03
0	0	0.23	1	cv	1	Dryas octopetala	Alysiinae sp. 2	23	0.01	0.03	0.01	0.03
0	0	0.21	1	t	1	Dryas octopetala	Pseudosmitti a oxoniana	23	0.01	0.03	0.01	0.05
0	0	0.26	1	t	1	Dryas octopetala	Ichn. sp. 2	23	0.01	0.03	0.01	0.03
0	0	0.25	1	t	1	Dryas octopetala	Rhamphomyi a reflexa	23	0.01	0.03	0.01	0.03
0	0	0.29	1	cv	1	Dryas octopetala	Micropsectra radialis	23	0.01	0.03	0.01	0.02
0	0	0.38	1	t	1	Dryas octopetala	Thricops hirtulus	23	0.01	0.03	0.03	0.04
1	0	0.27	1	t	1	Megaselia pleuralis	Parnassia palustris	23	0.01	0.02	0.01	0.03
1	1	0.73	1	t	1	Rhamphomyia morio	Viola biflora	23	0.03	0.04	0.03	0.03
0	1	0.90	1	cv	1	Rhamphomyia morio	Arabis alpina	23	0.03	0.04	0.06	0.05
0	0	0.44	1	t	1	Rhamphomyia morio	Potentilla crantzii	23	0.03	0.04	0.01	0.03
0	0	0.45	1	t	1	Rhamphomyia morio	Taraxacum sp.	23	0.03	0.04	0.01	0.03
1	1	0.73	1	t	1	Rhamphomyia morio	Polygonum viviparum	23	0.03	0.04	0.03	0.03
0	0	0.45	1	cv	1	Rhamphomyia morio	Silene acaulis	23	0.03	0.04	0.01	0.03
0	1	0.58	1	t	1	Rhamphomyia morio	Cerastium alpinun	23	0.03	0.04	0.02	0.03
0	0	0.23	1	t	1	Phytomyza aquilonia	Potentilla crantzii	23	0.01	0.03	0.01	0.03
0	0	0.21	1	t	1	Alliopsis glacialis	Saxifraga aizoides	23	0.01	0.03	0.01	0.04
1	0	0.25	1	cv	1	Micropsectra sp.	Taraxacum sp.	23	0.01	0.03	0.01	0.03

1	0	0.48	1	t	1	Viola biflora	Lycoriella freyi	23	0.03	0.03	0.01	0.03
1	0	0.48	1	t	1	Viola biflora	Bradysia sp. 2	23	0.03	0.03	0.01	0.03
1	0	0.48	1	t	1	Viola biflora	Chamaepsila morio	23	0.03	0.03	0.01	0.03
1	0	0.48	1	cv	1	Viola biflora	Stenomacrus sp.	23	0.03	0.03	0.01	0.03
1	0	0.48	1	t	1	Viola biflora	Megaselia sp.	23	0.03	0.03	0.01	0.03
0	0	0.44	1	t	1	Viola biflora	Coenosia octopunctata	23	0.03	0.03	0.01	0.05
1	0	0.25	1	t	1	Alysiinae sp. 5	Taraxacum sp.	23	0.01	0.03	0.01	0.03
1	1	0.58	1	cv	1	Anarta sp.	Polygonum viviparum	23	0.01	0.03	0.03	0.03
1	0	0.29	1	t	1	Anarta sp.	Silene acaulis	23	0.01	0.03	0.01	0.03
1	0	0.24	1	t	1	Alysiinae sp. 4	Silene acaulis	23	0.01	0.03	0.01	0.03
0	0	0.29	1	t	1	Thricops rostratus	Parnassia palustris	23	0.02	0.05	0.01	0.03
1	1	0.81	1	cv	1	Thricops rostratus	Arabis alpina	23	0.02	0.05	0.06	0.05
0	0	0.27	1	t	1	Thricops rostratus	Potentilla crantzii	23	0.02	0.05	0.01	0.03
1	0	0.35	1	t	1	Parapiophila vulgaris	Potentilla crantzii	23	0.02	0.02	0.01	0.03
1	0	0.45	1	t	1	Egle sp.	Salix lanata	23	0.01	0.03	0.03	0.03
0	1	0.51	1	cv	1	Rhodiola rosea	Xestommaster sp.	23	0.04	0.04	0.01	0.05
1	1	0.60	1	t	1	Rhodiola rosea	Gimnomera dorsala	23	0.04	0.04	0.01	0.04
1	1	0.55	1	t	1	Rhodiola rosea	Urbicola sp.	23	0.04	0.04	0.01	0.04
0	1	0.56	1	t	1	Rhodiola rosea	Smittia betuletorum	23	0.04	0.04	0.01	0.05
1	1	0.59	1	cv	1	Rhodiola rosea	Limnophyes ninae	23	0.04	0.04	0.01	0.04
1	1	0.54	1	t	1	Rhodiola rosea	Trichosia truncata	23	0.04	0.04	0.01	0.04
0	1	0.74	1	t	1	Rhodiola rosea	Thricops hirtulus	23	0.04	0.04	0.03	0.04
1	0	0.43	1	t	1	Phaonia alpicola	Taraxacum sp.	23	0.03	0.03	0.01	0.03
1	1	0.71	1	cv	1	Phaonia alpicola	Polygonum viviparum	23	0.03	0.03	0.03	0.03
1	0	0.44	1	t	1	Corynoptera refrigerata	Salix polaris	23	0.01	0.03	0.02	0.03
1	0	0.50	1	t	1	Corynoptera refrigerata	Salix lanata	23	0.01	0.03	0.03	0.03
1	0	0.37	1	t	1	Salix polaris	Chamaepsila morio	23	0.02	0.03	0.01	0.03
1	0	0.39	1	cv	1	Salix polaris	Lycoriella sp. 2	23	0.02	0.03	0.01	0.03

0	0	0.36	1	t	1	Salix polaris	Ichn. sp. 1	23	0.02	0.03	0.01	0.05
0	0	0.33	1	t	1	Salix polaris	Coenosia octopunctata	23	0.02	0.03	0.01	0.05
1	1	0.77	1	t	1	Allophorocera lapponica	Saxifraga oppositifolia	23	0.01	0.05	0.07	0.05
1	0	0.24	1	cv	1	Spilogona megastoma	Silene acaulis	23	0.01	0.05	0.01	0.03
0	0	0.21	1	t	1	Orthocentrinae sp.	Saxifraga aizoides	23	0.01	0.03	0.01	0.04
1	1	0.64	1	t	1	Pegomya haemorrhoum	Cerastium alpinun	23	0.04	0.03	0.02	0.03
0	0	0.48	1	t	1	Pegomya haemorrhoum	Saxifraga aizoides	23	0.04	0.03	0.01	0.04
0	0	0.26	1	cv	1	Paradelia sp.	Saxifraga aizoides	23	0.01	0.03	0.01	0.04
1	0	0.33	1	t	1	Rhododendron lapponicum	Bellardia pubicornis	23	0.02	0.04	0.01	0.04
0	0	0.41	1	t	1	Rhododendron lapponicum	Limnophilus sp.	23	0.02	0.04	0.02	0.05
0	0	0.31	1	t	1	Rhododendron lapponicum	Podabrus sp.	23	0.02	0.04	0.01	0.04
0	1	0.53	1	cv	1	Rhododendron lapponicum	Thricops hirtulus	23	0.02	0.04	0.03	0.04
1	0	0.24	1	t	1	Bradysia sp. 1	Silene acaulis	23	0.01	0.03	0.01	0.03
0	0	0.29	1	t	1	Phyllolabis macroura	Parnassia palustris	23	0.01	0.03	0.01	0.03
0	0	0.26	1	t	1	Phyllolabis macroura	Saxifraga aizoides	23	0.01	0.03	0.01	0.04
1	0	0.22	1	cv	1	Bellardia pubicornis	Silene acaulis	23	0.01	0.04	0.01	0.03
1	0	0.43	1	t	1	Bellardia pubicornis	Salix lanata	23	0.01	0.04	0.03	0.03
1	0	0.32	1	t	1	Parnassia palustris	Oliveridia tricornis	23	0.01	0.03	0.01	0.02
0	0	0.39	1	t	1	Parnassia palustris	Limnophyes brachytomus	23	0.01	0.03	0.02	0.02
0	0	0.20	1	cv	1	Parnassia palustris	Smittia aterrima	23	0.01	0.03	0.01	0.05
0	0	0.23	1	t	1	Parnassia palustris	Spilogona alpica	23	0.01	0.03	0.01	0.04
0	0	0.20	1	t	1	Parnassia palustris	Megaselia groenlandica	23	0.01	0.03	0.01	0.05
1	0	0.32	1	t	1	Parnassia palustris	Boletina sp.	23	0.01	0.03	0.01	0.02
1	1	0.54	1	cv	1	Limnophilus sp.	Salix reticulata	23	0.02	0.05	0.03	0.04
0	1	0.69	1	t	1	Arabis alpina	Smittia aterrima	23	0.06	0.05	0.01	0.05
0	1	0.68	1	t	1	Arabis alpina	Pegoplata aestiva	23	0.06	0.05	0.01	0.05

0	0	0.23	1	t	1	Potentilla crantzii	Cotesia sp. 6	23	0.01	0.03	0.01	0.03
0	0	0.40	1	cv	1	Potentilla crantzii	Thysan. sp.	23	0.01	0.03	0.03	0.05
0	0	0.22	1	t	1	Potentilla crantzii	Chamaepsila morio	23	0.01	0.03	0.01	0.03
0	0	0.28	1	t	1	Potentilla crantzii	Tetrastichinae sp.	23	0.01	0.03	0.01	0.02
0	1	0.62	1	t	1	Potentilla crantzii	Megaselia diversa	23	0.01	0.03	0.05	0.04
0	0	0.19	1	cv	1	Potentilla crantzii	Arpedium sp.	23	0.01	0.03	0.01	0.05
0	0	0.19	1	t	1	Potentilla crantzii	Smittia aterrima	23	0.01	0.03	0.01	0.05
0	0	0.25	1	t	1	Potentilla crantzii	Limnophyes schnelli	23	0.01	0.03	0.01	0.02
0	0	0.44	1	t	1	Potentilla crantzii	Thricops cunctans	23	0.01	0.03	0.03	0.03
0	0	0.22	1	cv	1	Potentilla crantzii	Spilogona alpica	23	0.01	0.03	0.01	0.04
0	0	0.18	1	t	1	Potentilla crantzii	Rhamphomyia obscuripennis	23	0.01	0.03	0.01	0.05
0	0	0.34	1	t	1	Potentilla crantzii	Phaonia subfuscinereis	23	0.01	0.03	0.02	0.03
0	0	0.23	1	t	1	Potentilla crantzii	Zaphne sp.	23	0.01	0.03	0.01	0.03
0	0	0.39	1	cv	1	Potentilla crantzii	Thricops hirtulus	23	0.01	0.03	0.03	0.04
0	0	0.17	1	t	1	Xestommaster sp.	Saxifraga aizoides	23	0.01	0.05	0.01	0.04
0	0	0.21	1	t	1	Mycomya fuscata	Saxifraga aizoides	23	0.01	0.03	0.01	0.04
0	0	0.32	1	t	1	Dasineura sp.	Cerastium alpinun	23	0.01	0.03	0.02	0.03
1	1	0.77	1	cv	1	Zaphne sp. 2	Saxifraga oppositifolia	23	0.01	0.05	0.07	0.05
0	0	0.21	1	t	1	Pegomya sp.	Saxifraga aizoides	23	0.01	0.03	0.01	0.04
1	0	0.50	1	t	1	Dolichopus plumipes	Cerastium alpinun	23	0.02	0.03	0.02	0.03
1	0	0.49	1	t	1	Thysan. sp.	Astragalus alpinus	23	0.03	0.05	0.02	0.04
1	0	0.36	1	cv	1	Thysan. sp.	Saxifraga aizoides	23	0.03	0.05	0.01	0.04
1	0	0.22	1	t	1	Chamaepsila morio	Silene acaulis	23	0.01	0.03	0.01	0.03
1	0	0.48	1	t	1	Chamaepsila morio	Salix reticulata	23	0.01	0.03	0.03	0.04

1	0	0.43	1	t	1	Chamaepsila morio	Salix lanata	23	0.01	0.03	0.03	0.03
1	0	0.27	1	cv	1	Chamaepsila morio	Cassiope tetragona	23	0.01	0.03	0.01	0.01
1	0	0.29	1	t	1	Gimnomera dorsala	Trollius europaeus	23	0.01	0.04	0.01	0.03
1	0	0.21	1	t	1	Urbicola sp.	Silene acaulis	23	0.01	0.04	0.01	0.03
0	0	0.25	1	t	1	Taraxacum sp.	Pteromalidae sp. 1	23	0.01	0.03	0.01	0.03
0	0	0.43	1	cv	1	Polygonum viviparum	Arpedium sp.	23	0.03	0.03	0.01	0.05
0	0	0.43	1	t	1	Polygonum viviparum	Smittia aterrima	23	0.03	0.03	0.01	0.05
1	0	0.48	1	t	1	Polygonum viviparum	Stenomacrus sp.	23	0.03	0.03	0.01	0.03
0	0	0.43	1	t	1	Polygonum viviparum	Rhamphomyia obscuripennis	23	0.03	0.03	0.01	0.05
1	1	0.61	1	cv	1	Polygonum viviparum	Smittia sp.	23	0.03	0.03	0.02	0.03
1	1	0.53	1	t	1	Polygonum viviparum	Rhamphomyia reflexa	23	0.03	0.03	0.01	0.03
0	1	0.67	1	t	1	Polygonum viviparum	Thricops hirtulus	23	0.03	0.03	0.03	0.04
0	0	0.33	1	t	1	Silene acaulis	Cidaria sp.	23	0.01	0.03	0.01	0.02
0	0	0.23	1	cv	1	Silene acaulis	Stenomacrus sp.	23	0.01	0.03	0.01	0.03
0	0	0.24	1	t	1	Silene acaulis	Alysiinae sp. 1	23	0.01	0.03	0.01	0.03
0	0	0.40	1	t	1	Silene acaulis	Thricops hirtulus	23	0.01	0.03	0.03	0.04
0	0	0.29	1	t	1	Astragalus alpinus	Bradysia girandii	23	0.02	0.04	0.01	0.04
1	0	0.44	1	cv	1	Salix reticulata	Podabrus sp.	23	0.03	0.04	0.01	0.04
0	0	0.41	1	t	1	Salix reticulata	Rhamphomyia obscuripennis	23	0.03	0.04	0.01	0.05
0	0	0.42	1	t	1	Salix reticulata	Coenosia octopunctata	23	0.03	0.04	0.01	0.05
1	0	0.28	1	t	1	Arpedium sp.	Cerastium alpinun	23	0.01	0.05	0.02	0.03
0	0	0.28	1	cv	1	Smittia aterrima	Cerastium alpinun	23	0.01	0.05	0.02	0.03
0	0	0.22	1	t	1	Smittia aterrima	Trollius europaeus	23	0.01	0.05	0.01	0.03
0	0	0.17	1	t	1	Smittia aterrima	Saxifraga aizoides	23	0.01	0.05	0.01	0.04
1	0	0.33	1	t	1	Egle minuta	Trollius europaeus	23	0.01	0.03	0.01	0.03

0	0	0.46	1	cv	1	Cerastium alpinun	Platygaster sp.	23	0.02	0.03	0.02	0.03
0	0	0.27	1	t	1	Cerastium alpinun	Megaselia groenlandica	23	0.02	0.03	0.01	0.05
0	1	0.52	1	t	1	Cerastium alpinun	Thricops hirtulus	23	0.02	0.03	0.03	0.04
1	0	0.25	1	t	1	Podabrus sp.	Trollius europaeus	23	0.01	0.04	0.01	0.03
1	0	0.28	1	cv	1	Fannia mollissima	Trollius europaeus	23	0.01	0.03	0.01	0.03
1	0	0.31	1	t	1	Limnophyes schnelli	Cassiope tetragona	23	0.01	0.02	0.01	0.01
1	0	0.27	1	t	1	Trollius europaeus	Thricops nigritellus	23	0.01	0.03	0.01	0.03
0	0	0.44	1	t	1	Trollius europaeus	Thricops hirtulus	23	0.01	0.03	0.03	0.04
0	0	0.20	1	cv	1	Spilogona alpica	Saxifraga aizoides	23	0.01	0.04	0.01	0.04
1	1	0.77	1	t	1	Dipt. sp. 3	Saxifraga oppositifolia	23	0.01	0.05	0.07	0.05
1	0	0.37	1	t	1	Cassiope hypniodes	Phaonia subfuscinerei s	23	0.01	0.03	0.02	0.03
1	0	0.45	1	t	1	Dipt. sp. 2	Salix lanata	23	0.01	0.03	0.03	0.03
0	0	0.17	1	cv	1	Megaselia groenlandica	Saxifraga aizoides	23	0.01	0.05	0.01	0.04
0	0	0.41	1	t	1	Zaphne frontata	Saxifraga aizoides	23	0.03	0.03	0.01	0.04
0	1	0.78	1	t	1	Rhamphomyia obscuripennis	Saxifraga oppositifolia	23	0.01	0.05	0.07	0.05
0	1	0.69	1	t	1	Platypalpus nigritarsis	Saxifraga aizoides	23	0.07	0.06	0.01	0.04
0	0	0.32	1	cv	1	Saxifraga aizoides	Phaonia subfuscinerei s	23	0.01	0.04	0.02	0.03
0	0	0.21	1	t	1	Saxifraga aizoides	Megaselia cirriventris	23	0.01	0.04	0.01	0.03
0	0	0.23	1	t	1	Saxifraga aizoides	Limnophyes ninae	23	0.01	0.04	0.01	0.04
0	0	0.24	1	t	1	Saxifraga aizoides	Ichn. sp. 2	23	0.01	0.04	0.01	0.03
0	1	0.76	1	cv	1	Saxifraga oppositifolia	Coenosia octopunctata	23	0.07	0.05	0.01	0.05
0	1	0.74	1	t	1	Saxifraga oppositifolia	Pteromalidae sp. 2	23	0.07	0.05	0.01	0.05
1	1	0.89	1	t	1	Saxifraga oppositifolia	Thricops hirtulus	23	0.07	0.05	0.03	0.04
1	0	0.43	1	t	1	Salix lanata	Egle parva	23	0.03	0.03	0.01	0.03
1	0	0.36	1	cv	1	Micropsectra radialis	Cassiope tetragona	23	0.01	0.02	0.01	0.01

0	0	0.27	0	t	1	Zaphne barbiventris	Pegoplata aestiva	23	0.02	0.05	0.01	0.05		
1	0	0.25	0	t	1	Delai piliventris	Spilogona alpica	23	0.01	0.02	0.01	0.04		
1	0	0.23	0	t	1	Silene acaulis	Egle parva	23	0.01	0.03	0.01	0.03		
0	0	0.20	0	cv	1	Pteromalidae sp. 1	Ichn. sp. 4	23	0.01	0.03	0.01	0.05		
0	0	0.36	0	t	1	Thysan. sp.	Urbicola sp.	23	0.03	0.05	0.01	0.04		
1	0	0.21	0	t	1	Phygadenon sp.	Bellardia pubicornis	23	0.01	0.03	0.01	0.04		
0	0	0.20	0	t	1	Delai	Zaphne sp. 2	23	0.01	0.02	0.01	0.05		
0	0	0.38	0	cv	1	Thysan. sp.	Megaselia cirriventris	23	0.03	0.05	0.01	0.03		
1	0	0.32	0	t	1	Oliveridia tricornis	Fannia mollissima	23	0.01	0.02	0.01	0.03		
1	0	0.22	0	t	1	Chloropinae sp.	Trichosia truncata	23	0.01	0.03	0.01	0.04		
1	0	0.28	0	t	1	Delai piliventris	Limnophyes schnelli	23	0.01	0.02	0.01	0.02		
0	1	0.76	0	cv	1	Bradysia sp. 3	Platypalpus nigritarsis	23	0.01	0.03	0.07	0.06		
0	0	0.39	0	t	1	Thricops cunctans	Coenosia octopunctata	23	0.03	0.03	0.01	0.05		
1	0	0.42	0	t	1	Heterotrissocl adius	Smittia sp.	23	0.01	0.01	0.02	0.03		
0	0	0.18	0	t	1	Bradysia sp. 2	Lycoriella sp. 1	23	0.01	0.03	0.01	0.05		
0	0	0.39	0	cv	1	Zaphne frontata	Coenosia octopunctata	23	0.03	0.03	0.01	0.05		
0	0	0.19	0	t	1	Alysiinae sp. 4	Lycoriella sp. 1	23	0.01	0.03	0.01	0.05		
1	1	0.57	0	t	1	Thricops cunctans	Smittia sp.	23	0.03	0.03	0.02	0.03		
0	0	0.34	0	t	1	Pegomya sp.	Platygaster sp.	23	0.01	0.03	0.02	0.03		
0	0	0.43	0	cv	1	Rhododendro n lapponicum	Oliveridia tricornis	23	0.02	0.04	0.01	0.02		
0	0	0.22	0	t	1	Bartsia alpina	Ichn. sp. 3	23	0.01	0.02	0.01	0.05		
0	0	0.19	0	t	1	Saxifraga aizoides	Pseudosmitti a oxoniana	23	0.01	0.04	0.01	0.05		
1	0	0.27	0	t	1	Ichn. sp. 2	Thricops nigritellus	23	0.01	0.03	0.01	0.03		
1	0	0.23	0	cv	1	Dryas octopetala	Lycoriella sp. 2	23	0.01	0.03	0.01	0.03		
0	0	0.19	0	t	1	Zaphne sp. 2	Cassiope hypniodes	23	0.01	0.05	0.01	0.03		
0	1	0.76	0	t	1	Platypalpus nigritarsis	Cassiope tetragona	23	0.07	0.06	0.01	0.01		
0	0	0.20	0	t	1	Delai piliventris	Megaselia girandii	23	0.01	0.02	0.01	0.05		

0	0	0.42	0	cv	1	Salix polaris	Trollius europaeus	23	0.02	0.03	0.01	0.03
0	0	0.27	0	t	1	Paradelia sp.	Smittia betuletorum	23	0.01	0.03	0.01	0.05
0	0	0.39	0	t	1	Thysan. sp.	Taraxacum sp.	23	0.03	0.05	0.01	0.03
0	0	0.18	0	t	1	Corynoptera sp. 1	Dipt. sp. 1	23	0.01	0.05	0.01	0.05
1	0	0.26	0	cv	1	Phyllolabis macroura	Podabrus sp.	23	0.01	0.03	0.01	0.04
0	1	0.86	0	t	1	Phyllolabis macroura	Saxifraga oppositifolia	23	0.01	0.03	0.07	0.05
0	0	0.24	0	t	1	Aclastus sp. 1	Mycomya fuscata	23	0.01	0.05	0.01	0.03
1	0	0.27	0	t	1	Bartsia alpina	Chamaepsila morio	23	0.01	0.02	0.01	0.03
0	0	0.20	0	cv	1	Egle parva	Coenosia octopunctata	23	0.01	0.03	0.01	0.05
0	1	0.64	0	t	1	Thysan. sp.	Thricops cunctans	23	0.03	0.05	0.03	0.03
0	1	0.62	0	t	1	Rhamphomyia morio	Dolichopus plumipes	23	0.03	0.04	0.02	0.03
1	0	0.24	0	t	1	Megaselia coci	Lycoriella freyi	23	0.01	0.03	0.01	0.03
0	0	0.45	0	cv	1	Thricops rostratus	Thricops hirtulus	23	0.02	0.05	0.03	0.04
0	0	0.16	0	t	1	Allophorocera lapponica	Urbicola sp.	23	0.01	0.05	0.01	0.04
0	1	0.54	0	t	1	Viola biflora	Trollius europaeus	23	0.03	0.03	0.01	0.03
0	1	0.61	0	t	1	Salix reticulata	Phaonia subfuscinereis	23	0.03	0.04	0.02	0.03
0	0	0.19	0	cv	1	Gimnomera dorsala	Lycoriella curvispina	23	0.01	0.04	0.01	0.05
0	0	0.48	0	t	1	Micropsectra sp. 2	Podabrus sp.	23	0.04	0.05	0.01	0.04
0	0	0.22	0	t	1	Megaselia digitalis	Xestommaster sp.	23	0.02	0.05	0.01	0.05
0	0	0.23	0	t	1	Smittia sp. 2	Chloropinae sp.	23	0.01	0.03	0.01	0.03
0	0	0.20	0	cv	1	Micropsectra sp.	Zaphne sp. 2	23	0.01	0.03	0.01	0.05
1	0	0.29	0	t	1	Egle minuta	Fannia mollissima	23	0.01	0.03	0.01	0.03
1	0	0.23	0	t	1	Megaselia sp.	Egle parva	23	0.01	0.03	0.01	0.03
1	0	0.24	0	t	1	Orthocentrinae sp.	Fannia mollissima	23	0.01	0.03	0.01	0.03
0	0	0.27	0	cv	1	Thricops rostratus	Stenomacrus sp.	23	0.02	0.05	0.01	0.03

1	0	0.23	0	t	1	Empis lucida	Spilogona alpica	23	0.01	0.03	0.01	0.04
0	0	0.22	0	t	1	Phygadenon sp.	Mycomya fuscata	23	0.01	0.03	0.01	0.03
0	0	0.32	0	t	1	Corynoptera sp. 1	Spilogona nitidicauda	23	0.01	0.05	0.02	0.03
0	1	0.77	0	cv	1	Diapensia lapponica	Fannia mollissima	23	0.07	0.05	0.01	0.03
0	0	0.34	0	t	1	Smittia sp. 2	Platygaster sp.	23	0.01	0.03	0.02	0.03
0	0	0.28	0	t	1	Botanophilia bidens	Cassiope tetragona	23	0.01	0.03	0.01	0.01
0	0	0.47	0	t	1	Phyllolabis macroura	Thysan. sp.	23	0.01	0.03	0.03	0.05
0	0	0.19	0	cv	1	Bradysia sp. 3	Pteromalidae sp. 2	23	0.01	0.03	0.01	0.05
0	1	0.57	0	t	1	Pegomya haemorrhoum	Cassiope tetragona	23	0.04	0.03	0.01	0.01
0	0	0.37	0	t	1	Bombus alpinus	Tetrastichinae sp.	23	0.02	0.05	0.01	0.02
0	0	0.19	0	t	1	Spilogona megastoma	Pegoplata aestiva	23	0.01	0.05	0.01	0.05
0	0	0.34	0	cv	1	Corynoptera sp. 2	Cassiope tetragona	23	0.02	0.05	0.01	0.01
0	1	0.56	0	t	1	Dasineura sp.	Corynoneura scutella	23	0.01	0.03	0.05	0.05
0	1	0.63	0	t	1	Acamptocladius	Rhodiola rosea	23	0.01	0.02	0.04	0.04
1	0	0.45	0	t	1	Zaphne frontata	Alysiinae sp. 1	23	0.03	0.03	0.01	0.03
0	0	0.27	0	cv	1	Spilogona triangulifera	Platygaster sp.	23	0.01	0.05	0.02	0.03
0	0	0.21	0	t	1	Potentilla crantzii	Urbicola sp.	23	0.01	0.03	0.01	0.04
0	0	0.23	0	t	1	Rhaphium crassipes	Orthocentrinae sp.	23	0.01	0.03	0.01	0.03
1	0	0.22	0	t	1	Urbicola sp.	Alysiinae sp. 2	23	0.01	0.04	0.01	0.03
1	0	0.35	0	cv	1	Bradysia sp. 3	Platygaster sp.	23	0.01	0.03	0.02	0.03
0	0	0.22	0	t	1	Smittia betuletorum	Lycoriella sp. 2	23	0.01	0.05	0.01	0.03
0	0	0.13	0	t	1	Zaphne sp. 2	Megaselia girandii	23	0.01	0.05	0.01	0.05
0	0	0.24	0	t	1	Bombus	Dipt. sp. 3	23	0.02	0.05	0.01	0.05
1	0	0.43	0	cv	1	Phaonia alpicola	Dipt. sp. 2	23	0.03	0.03	0.01	0.03
0	0	0.37	0	t	1	Paradelia sp.	Astragalus alpinus	23	0.01	0.03	0.02	0.04
1	0	0.31	0	t	1	Porizontini sp.	Bradysia girandii	23	0.01	0.02	0.01	0.04

0	0	0.26	0	t	1	Corynoptera refrigerata	Smittia betuletorum	23	0.01	0.03	0.01	0.05
0	1	0.60	0	cv	1	Megaselia diversa	Silene acaulis	23	0.05	0.04	0.01	0.03
1	1	0.67	0	t	1	Dolichopus plumipes	Polygonum viviparum	23	0.02	0.03	0.03	0.03
0	0	0.34	0	t	1	Rhamphomyia reflexa	Corynoptera sp. 2	23	0.01	0.03	0.02	0.05
0	0	0.18	0	t	1	Lycoriella freyi	Lycoriella sp. 1	23	0.01	0.03	0.01	0.05
0	0	0.13	0	cv	1	Spilogona triangulifera	Ichn. sp. 3	23	0.01	0.05	0.01	0.05
1	0	0.21	0	t	1	Limnophyes natalensis	Urbicola sp.	23	0.01	0.03	0.01	0.04
0	1	0.84	0	t	1	Rhododendro n lapponicum	Platypalpus nigritarsis	23	0.02	0.04	0.07	0.06
0	0	0.22	0	t	1	Megaselia coci	Saxifraga aizoides	23	0.01	0.03	0.01	0.04
0	0	0.19	0	cv	1	Dipt. sp. 2	Pegoplata aestiva	23	0.01	0.03	0.01	0.05
0	0	0.28	0	t	1	Heterotrissocl adius	Trichosia truncata	23	0.01	0.01	0.01	0.04
0	0	0.17	0	t	1	Corynoptera sp. 1	Mycomya fuscata	23	0.01	0.05	0.01	0.03
0	0	0.43	0	t	1	Thysan. sp.	Ichn. sp. 2	23	0.03	0.05	0.01	0.03
0	0	0.37	0	cv	1	Salix polaris	Lycoriella freyi	23	0.02	0.03	0.01	0.03
1	1	0.57	0	t	1	Coenosia atritibia	Oliveridia tricornis	23	0.03	0.03	0.01	0.02
0	0	0.23	0	t	1	Smittia betuletorum	Cassiope hypniodes	23	0.01	0.05	0.01	0.03
1	0	0.26	0	t	1	Bradysia sp. 1	Limnophyes ninae	23	0.01	0.03	0.01	0.04
0	0	0.21	0	cv	1	Saxifraga aizoides	Phaonia lugubris	23	0.01	0.04	0.01	0.03
0	0	0.22	0	t	1	Spilogona triangulifera	Tetrastichina e sp.	23	0.01	0.05	0.01	0.02
1	0	0.46	0	t	1	Limnophyes ninae	Salix lanata	23	0.01	0.04	0.03	0.03
0	0	0.17	0	t	1	Chamaepsila morio	Dipt. sp. 3	23	0.01	0.03	0.01	0.05
0	0	0.18	0	cv	1	Pegomya sp.	Ichn. sp. 4	23	0.01	0.03	0.01	0.05
0	0	0.46	0	t	1	Corynoneura scutella	Dipt. sp. 3	23	0.05	0.05	0.01	0.05
0	0	0.18	0	t	1	Allophorocera lapponica	Dipt. sp. 2	23	0.01	0.05	0.01	0.03
1	0	0.27	0	t	1	Thricops nigritellus	Rhamphomyi a reflexa	23	0.01	0.03	0.01	0.03
1	1	0.53	0	cv	1	Rhamphomyia aethiops	Zaphne frontata	23	0.01	0.03	0.03	0.03

0	0	0.20	0	t	1	Smittia sp. 2	Coenosia octopunctata	23	0.01	0.03	0.01	0.05
0	0	0.14	0	t	1	Xestommaster sp.	Ichn. sp. 4	23	0.01	0.05	0.01	0.05
0	0	0.20	0	t	1	Parnassia palustris	Rhamphomyia obscuripennis	23	0.01	0.03	0.01	0.05
1	0	0.29	0	cv	1	Tetrastichinae sp.	Chloropinae sp.	23	0.01	0.02	0.01	0.03
1	0	0.27	0	t	1	Stenomacrus sp.	Cassiope tetragona	23	0.01	0.03	0.01	0.01
1	0	0.23	0	t	1	Bradysia sp. 2	Egle parva	23	0.01	0.03	0.01	0.03
0	0	0.31	0	t	1	Zaphne barbiventris	Ichn. sp. 1	23	0.02	0.05	0.01	0.05
0	0	0.30	0	cv	1	Dolichopus plumipes	Ichn. sp. 4	23	0.02	0.03	0.01	0.05
1	0	0.35	0	t	1	Heterotrissocladius	Corynoptera refrigerata	23	0.01	0.01	0.01	0.03
1	0	0.24	0	t	1	Micropsectra sp.	Chamaepsila morio	23	0.01	0.03	0.01	0.03
0	1	0.51	0	t	1	Viola biflora	Pteromalidae sp. 1	23	0.03	0.03	0.01	0.03
0	0	0.41	0	cv	1	Thysan. sp.	Pteromalidae sp. 1	23	0.03	0.05	0.01	0.03
1	0	0.33	0	t	1	Tetrastichinae sp.	Rhamphomyia reflexa	23	0.01	0.02	0.01	0.03
0	1	0.82	0	t	1	Diapensia lapponica	Anarta sp.	23	0.07	0.05	0.01	0.03
1	0	0.44	0	t	1	Phaonia alpicola	Fannia mollissima	23	0.03	0.03	0.01	0.03
0	0	0.18	0	cv	1	Dipt. sp. 2	Megaselia girandii	23	0.01	0.03	0.01	0.05
0	0	0.25	0	t	1	Aclastus sp. 1	Lycoriella sp. 2	23	0.01	0.05	0.01	0.03
0	1	0.94	0	t	1	Polygonum viviparum	Saxifraga oppositifolia	23	0.03	0.03	0.07	0.05
1	1	0.55	0	t	1	Phaonia alpicola	Porizontini sp.	23	0.03	0.03	0.01	0.02
0	0	0.25	0	cv	1	Anarta sp.	Pegoplata aestiva	23	0.01	0.03	0.01	0.05
1	0	0.26	0	t	1	Delai	Megaselia sp.	23	0.01	0.02	0.01	0.03
0	0	0.33	0	t	1	Mycomya fuscata	Cerastium alpinun	23	0.01	0.03	0.02	0.03
1	1	0.52	0	t	1	Spilogona nitidicauda	Smittia sp.	23	0.02	0.03	0.02	0.03
0	1	0.54	0	cv	1	Corynoneura scutella	Chloropinae sp.	23	0.05	0.05	0.01	0.03
0	0	0.24	0	t	1	Megaselia coci	Orthocentrinae sp.	23	0.01	0.03	0.01	0.03

1	0	0.37	0	t	1	Zaphne barbiventris	Trollius europaeus	23	0.02	0.05	0.01	0.03
0	1	0.83	0	t	1	Diapensia lapponica	Oliveridia tricornis	23	0.07	0.05	0.01	0.02
1	0	0.32	0	cv	1	Phaonia subfuscinereis	Trichosia truncata	23	0.02	0.03	0.01	0.04
0	0	0.21	0	t	1	Arpedium sp.	Limnophyes schnelli	23	0.01	0.05	0.01	0.02
0	0	0.24	0	t	1	Limnophyes natalensis	Dipt. sp. 1	23	0.01	0.03	0.01	0.05
0	1	0.52	0	t	1	Rhodiola rosea	Coenosia octopunctata	23	0.04	0.04	0.01	0.05
1	1	0.56	0	cv	1	Bartsia alpina	Polygonum viviparum	23	0.01	0.02	0.03	0.03
1	0	0.26	0	t	1	Alysiinae sp. 5	Bradysia sp. 1	23	0.01	0.03	0.01	0.03
0	1	0.51	0	t	1	Salix reticulata	Rhamphomyia reflexa	23	0.03	0.04	0.01	0.03
0	0	0.24	0	t	1	Parnassia palustris	Potentilla crantzii	23	0.01	0.03	0.01	0.03
0	0	0.46	0	cv	1	Alysiinae sp. 5	Phaonia alpicola	23	0.01	0.03	0.03	0.03
1	0	0.41	0	t	1	Rhamphomyia aethiops	Smittia sp.	23	0.01	0.03	0.02	0.03
1	0	0.38	0	t	1	Dolichopus plumipes	Alysiinae sp. 2	23	0.02	0.03	0.01	0.03
1	0	0.31	0	t	1	Parnassia palustris	Micropsectra radialis	23	0.01	0.03	0.01	0.02
0	0	0.23	0	cv	1	Aclastus sp. 1	Bellardia pubicornis	23	0.01	0.05	0.01	0.04
0	0	0.23	0	t	1	Megaselia coci	Ichn. sp. 1	23	0.01	0.03	0.01	0.05
1	0	0.26	0	t	1	Megaselia pleuralis	Egle parva	23	0.01	0.02	0.01	0.03
1	0	0.48	0	t	1	Pegomya haemorrhoum	Bradysia girandii	23	0.04	0.03	0.01	0.04
1	0	0.23	0	cv	1	Podabrus sp.	Limnophyes ninae	23	0.01	0.04	0.01	0.04
0	1	0.68	0	t	1	Micropsectra sp. 2	Limnophyes brachytomus	23	0.04	0.05	0.02	0.02
0	0	0.19	0	t	1	Phytomyza aquilonia	Zaphne sp. 2	23	0.01	0.03	0.01	0.05
1	0	0.23	0	t	1	Potentilla crantzii	Egle parva	23	0.01	0.03	0.01	0.03
1	0	0.33	0	cv	1	Porizontini sp.	Silene acaulis	23	0.01	0.02	0.01	0.03
1	1	0.54	0	t	1	Anarta sp.	Thricops cunctans	23	0.01	0.03	0.03	0.03
0	1	0.51	0	t	1	Bradysia sp. 3	Polygonum viviparum	23	0.01	0.03	0.03	0.03
1	0	0.37	0	t	1	Parapiophila vulgaris	Cotesia sp. 6	23	0.02	0.02	0.01	0.03

0	1	0.82	0	cv	1	Orthocentrinae sp.	Saxifraga oppositifolia	23	0.01	0.03	0.07	0.05
0	0	0.22	0	t	1	Dasineura sp.	Megaselia cirriventris	23	0.01	0.03	0.01	0.03
0	1	0.70	0	t	1	Anarta sp.	Megaselia diversa	23	0.01	0.03	0.05	0.04
0	0	0.18	0	t	1	Lycoriella curvispina	Zaphne sp.	23	0.01	0.05	0.01	0.03
1	0	0.38	0	cv	1	Alysiinae sp. 4	Parapiophila vulgaris	23	0.01	0.03	0.02	0.02
1	1	0.83	0	t	1	Dipt. sp. 1	Saxifraga oppositifolia	23	0.01	0.05	0.07	0.05
0	0	0.15	0	t	1	Smittia	Ichn. sp. 3	23	0.01	0.05	0.01	0.05
1	0	0.44	0	t	1	Bombus hyperboreus	Pinguicula alpina	23	0.01	0.03	0.02	0.03
0	0	0.28	0	cv	1	Alliopsis glacialis	Tetrastichinae sp.	23	0.01	0.03	0.01	0.02
0	0	0.38	0	t	1	Pegoplata aestiva	Zaphne frontata	23	0.01	0.05	0.03	0.03
1	0	0.33	0	t	1	Cassiope hypniodes	Boletina sp.	23	0.01	0.03	0.01	0.02
0	1	0.53	0	t	1	Limnophyes natalensis	Pegomya haemorrhoum	23	0.01	0.03	0.04	0.03
0	0	0.42	0	cv	1	Gimnomera dorsala	Thricops hirtulus	23	0.01	0.04	0.03	0.04
0	1	0.78	0	t	1	Parnassia palustris	Arabis alpina	23	0.01	0.03	0.06	0.05
0	0	0.29	0	t	1	Rhamphomyia aethiops	Orthocentrinae sp.	23	0.01	0.03	0.01	0.03
0	0	0.29	0	t	1	Anarta sp.	Pegomya sp.	23	0.01	0.03	0.01	0.03
0	0	0.35	0	cv	1	Aclastus sp. 1	Platygaster sp.	23	0.01	0.05	0.02	0.03
0	1	0.83	0	t	1	Bradysia sp. 3	Saxifraga oppositifolia	23	0.01	0.03	0.07	0.05
0	1	0.83	0	t	1	Bradysia sp. 1	Saxifraga oppositifolia	23	0.01	0.03	0.07	0.05
0	0	0.22	0	t	1	Thricops rostratus	Ichn. sp. 4	23	0.02	0.05	0.01	0.05
0	0	0.17	0	cv	1	Smittia aterrima	Trichosia truncata	23	0.01	0.05	0.01	0.04
0	0	0.20	0	t	1	Pegoplata aestiva	Pteromalidae sp. 1	23	0.01	0.05	0.01	0.03
0	0	0.20	0	t	1	Phytomyza aquilonia	Xestommaster sp.	23	0.01	0.03	0.01	0.05
1	0	0.47	0	t	1	Gimnomera dorsala	Zaphne frontata	23	0.01	0.04	0.03	0.03
0	0	0.19	0	cv	1	Lycoriella sp. 2	Pteromalidae sp. 2	23	0.01	0.03	0.01	0.05
0	0	0.18	0	t	1	Corynoptera sp. 1	Bradysia sp. 1	23	0.01	0.05	0.01	0.03

1	0	0.30	0	t	1	Rhamphomyia aethiops	Botanophilia bidens	23	0.01	0.03	0.01	0.03
0	1	0.60	0	t	1	Bombus alpinus	Polygonum viviparum	23	0.02	0.05	0.03	0.03
0	0	0.33	0	cv	1	Dryas octopetala	Platygaster sp.	23	0.01	0.03	0.02	0.03
0	1	0.74	0	t	1	Rhododendron lapponicum	Megaselia diversa	23	0.02	0.04	0.05	0.04
0	0	0.19	0	t	1	Phaonia lugubris	Pteromalidae sp. 2	23	0.01	0.03	0.01	0.05
0	0	0.34	0	t	1	Bombus alpinus	Rhamphomyia reflexa	23	0.02	0.05	0.01	0.03
1	1	0.51	0	cv	1	Pegomya haemorrhoum	Stenomacrus sp.	23	0.04	0.03	0.01	0.03
0	0	0.23	0	t	1	Rhaphium crassipes	Alliopsis glacialis	23	0.01	0.03	0.01	0.03
0	0	0.14	0	t	1	Dipt. sp. 3	Rhamphomyia obscuripennis	23	0.01	0.05	0.01	0.05
0	0	0.45	0	t	1	Smittia sp. 2	Zaphne frontata	23	0.01	0.03	0.03	0.03
0	1	0.63	0	cv	1	Orthocentrinae sp.	Megaselia diversa	23	0.01	0.03	0.05	0.04
1	0	0.28	0	t	1	Gimnomera dorsala	Rhamphomyia reflexa	23	0.01	0.04	0.01	0.03
0	1	0.61	0	t	1	Coenosia atritibia	Zaphne barbiventris	23	0.03	0.03	0.02	0.05
0	1	0.94	0	t	1	Rhodiola rosea	Arabis alpina	23	0.04	0.04	0.06	0.05
1	0	0.26	0	cv	1	Bartsia alpina	Podabrus sp.	23	0.01	0.02	0.01	0.04
0	0	0.30	0	t	1	Parnassia palustris	Tetrastichinae sp.	23	0.01	0.03	0.01	0.02
0	0	0.47	0	t	1	Rhamphomyia morio	Dipt. sp. 1	23	0.03	0.04	0.01	0.05
1	0	0.27	0	t	1	Corynoptera refrigerata	Orthocentrinae sp.	23	0.01	0.03	0.01	0.03
0	0	0.31	0	cv	1	Bombus alpinus	Phaonia lugubris	23	0.02	0.05	0.01	0.03
0	1	0.59	0	t	1	Alysiinae sp. 4	Corynoneura scutella	23	0.01	0.03	0.05	0.05
0	0	0.36	0	t	1	Dipt. sp. 3	Salix lanata	23	0.01	0.05	0.03	0.03
0	0	0.26	0	t	1	Porizontini sp.	Lycoriella curvispina	23	0.01	0.02	0.01	0.05
0	0	0.21	0	cv	1	Trollius europaeus	Megaselia girandii	23	0.01	0.03	0.01	0.05
1	0	0.40	0	t	1	Dolichopus plumipes	Limnophyes ninae	23	0.02	0.03	0.01	0.04
1	0	0.32	0	t	1	Acamptocladius	Tetrastichinae sp.	23	0.01	0.02	0.01	0.02
0	0	0.24	0	t	1	Orthocentrinae sp.	Pteromalidae sp. 1	23	0.01	0.03	0.01	0.03

1	0	0.24	0	cv	1	Lycoriella freyi	Botanophilia bidens	23	0.01	0.03	0.01	0.03
0	0	0.37	0	t	1	Spilogona triangulifera	Thricops cunctans	23	0.01	0.05	0.03	0.03
1	0	0.22	0	t	1	Bradysia girandii	Phaonia lugubris	23	0.01	0.04	0.01	0.03
0	0	0.49	0	t	1	Astragalus alpinus	Thricops hirtulus	23	0.02	0.04	0.03	0.04
1	0	0.28	0	cv	1	Paradelia sp.	Bradysia sp. 2	23	0.01	0.03	0.01	0.03
0	1	0.77	0	t	1	Empis lucida	Arabis alpina	23	0.01	0.03	0.06	0.05
1	0	0.34	0	t	1	Chamaepsila morio	Phaonia subfuscinereis	23	0.01	0.03	0.02	0.03
1	0	0.45	0	t	1	Zaphne frontata	Thricops nigritellus	23	0.03	0.03	0.01	0.03
0	0	0.22	0	cv	1	Rhamphomyia reflexa	Coenosia octopunctata	23	0.01	0.03	0.01	0.05
1	0	0.29	0	t	1	Oliveridia tricornis	Bradysia girandii	23	0.01	0.02	0.01	0.04
0	0	0.22	0	t	1	Lycoriella freyi	Ichn. sp. 1	23	0.01	0.03	0.01	0.05
1	0	0.26	0	t	1	Acamptocladius	Limnophyes natalensis	23	0.01	0.02	0.01	0.03
0	0	0.23	0	cv	1	Rhamphomyia aethiops	Megaselia girandii	23	0.01	0.03	0.01	0.05
0	1	0.66	0	t	1	Bombus alpinus	Corynoneura scutella	23	0.02	0.05	0.05	0.05
1	0	0.45	0	t	1	Zaphne frontata	Lycoriella sp. 2	23	0.03	0.03	0.01	0.03
0	0	0.23	0	t	1	Bradysia sp. 2	Taraxacum sp.	23	0.01	0.03	0.01	0.03
0	0	0.39	0	cv	1	Dolichopus plumipes	Dipt. sp. 1	23	0.02	0.03	0.01	0.05
0	0	0.43	0	t	1	Limnophilus sp.	Limnophyes brachytomus	23	0.02	0.05	0.02	0.02
0	1	0.63	0	t	1	Rhodiola rosea	Paradelia sp.	23	0.04	0.04	0.01	0.03
0	1	0.77	0	t	1	Diapensia lapponica	Chloropinae sp.	23	0.07	0.05	0.01	0.03
0	0	0.23	0	cv	1	Rhamphomyia aethiops	Lycoriella sp. 1	23	0.01	0.03	0.01	0.05
0	0	0.19	0	t	1	Megaselia groenlandica	Thricops nigritellus	23	0.01	0.05	0.01	0.03
0	0	0.48	0	t	1	Viola biflora	Potentilla crantzii	23	0.03	0.03	0.01	0.03
0	0	0.24	0	t	1	Megaselia sordida	Megaselia cirriventris	23	0.01	0.03	0.01	0.03
0	0	0.21	0	cv	1	Megaselia digitalis	Pteromalidae sp. 2	23	0.02	0.05	0.01	0.05
0	0	0.36	0	t	1	Pinguicula alpina	Pseudosmittia oxoniana	23	0.02	0.03	0.01	0.05

0	0	0.33	0	t	1	Orthocentrinae sp.	Cerastium alpinun	23	0.01	0.03	0.02	0.03
0	0	0.35	0	t	1	Phygadenon sp.	Alysiinae sp. 3	23	0.01	0.03	0.02	0.02
0	0	0.20	0	cv	1	Megaselia pleuralis	Allophorocera lapponica	23	0.01	0.02	0.01	0.05
1	0	0.26	0	t	1	Bombus hyperboreus	Orthocentrinae sp.	23	0.01	0.03	0.01	0.03
0	0	0.21	0	t	1	Spilogona triangulifera	Paradelia sp.	23	0.01	0.05	0.01	0.03
1	0	0.18	1	t	1	Gynometriocnemus sp.	Salix arctica	24	0.01	0.05	0.01	0.05
1	1	0.58	1	cv	1	Pegomyia sp.	Potentilla sp.	24	0.01	0.06	0.05	0.05
1	0	0.20	1	t	1	Pegomyia sp. 2	Taraxacum arctogenum	24	0.01	0.06	0.01	0.04
1	0	0.18	1	t	1	Bradysia sp.	Salix arctica	24	0.01	0.05	0.01	0.05
1	0	0.37	1	t	1	Pogonomyioides segnis	Papaver radicatum	24	0.02	0.08	0.04	0.06
1	0	0.16	1	cv	1	Pogonomyioides segnis	Salix arctica	24	0.02	0.08	0.01	0.05
1	1	0.93	1	t	1	Pogonomyioides segnis	Dryas integrifolia	24	0.02	0.08	0.11	0.07
0	1	0.62	1	t	1	Erysimum pallasii	Crymodes exulis	24	0.02	0.05	0.04	0.04
0	1	0.55	1	t	1	Erysimum pallasii	Smittia extrema	24	0.02	0.05	0.04	0.07
0	0	0.34	1	cv	1	Erysimum pallasii	Paraphaenocladius despectus	24	0.02	0.05	0.01	0.05
1	0	0.28	1	t	1	Erysimum pallasii	Spilogona sp.	24	0.02	0.05	0.01	0.05
0	0	0.16	1	t	1	Chironomus sp. 3	Saxifraga oppositifolia	24	0.01	0.05	0.01	0.05
1	1	0.68	1	t	1	Megabombus polaris	Papaver radicatum	24	0.05	0.08	0.04	0.06
1	0	0.39	1	cv	1	Megabombus polaris	Cassiope tetragona	24	0.05	0.08	0.01	0.05
1	0	0.41	1	t	1	Megabombus polaris	Salix arctica	24	0.05	0.08	0.01	0.05
1	1	0.68	1	t	1	Megabombus polaris	Stellaria sp.	24	0.05	0.08	0.03	0.05
1	0	0.39	1	t	1	Megabombus polaris	Pedicularis capitata	24	0.05	0.08	0.01	0.05
1	1	0.98	1	cv	1	Megabombus polaris	Dryas integrifolia	24	0.05	0.08	0.11	0.07
1	0	0.38	1	t	1	Megabombus polaris	Saxifraga oppositifolia	24	0.05	0.08	0.01	0.05
1	0	0.44	1	t	1	Megabombus polaris	Pedicularis arctica	24	0.05	0.08	0.01	0.06
1	0	0.44	1	t	1	Megabombus polaris	Silene acaulis	24	0.05	0.08	0.01	0.06

1	1	0.58	1	cv	1	Megabombus polaris	Epilobium latifolium	24	0.05	0.08	0.03	0.06
1	0	0.39	1	t	1	Megabombus polaris	Pedicularis hirsuta	24	0.05	0.08	0.01	0.05
0	1	0.86	1	t	1	Heterotrissocladius	Lychnis triflora	24	0.01	0.08	0.09	0.07
0	0	0.11	1	t	1	Heterotrissocladius	Polygonum viviparum	24	0.01	0.08	0.01	0.07
1	0	0.31	1	cv	1	Ichneumonidae	Saxifraga tricuspidata	24	0.01	0.05	0.02	0.05
1	0	0.37	1	t	1	Ichneumonidae	Lesquerella arctica	24	0.01	0.05	0.03	0.05
1	0	0.13	1	t	1	Plebeius	Salix arctica	24	0.01	0.08	0.01	0.05
1	1	0.91	1	t	1	Plebeius aquilo	Dryas integrifolia	24	0.01	0.08	0.11	0.07
1	1	0.94	1	cv	1	Spilogona deflorata	Dryas integrifolia	24	0.02	0.08	0.11	0.07
0	0	0.27	1	t	1	Papaver radicatum	Smittia sp. 3	24	0.04	0.06	0.01	0.08
1	0	0.32	1	t	1	Papaver radicatum	Spilogona dorsata	24	0.04	0.06	0.01	0.08
1	0	0.37	1	t	1	Papaver radicatum	Xysticus deichmanni	24	0.04	0.06	0.02	0.08
1	0	0.27	1	cv	1	Papaver radicatum	Spilogona sanctipauli	24	0.04	0.06	0.01	0.08
0	0	0.37	1	t	1	Papaver radicatum	Limnophyes sp.	24	0.04	0.06	0.02	0.08
1	0	0.47	1	t	1	Papaver radicatum	Carposcalis carinata	24	0.04	0.06	0.03	0.08
1	0	0.13	1	t	1	Smittia sp. 3	Draba bellii	24	0.01	0.08	0.02	0.06
1	1	0.89	1	cv	1	Smittia sp. 3	Dryas integrifolia	24	0.01	0.08	0.11	0.07
1	0	0.10	1	t	1	Smittia sp. 3	Saxifraga oppositifolia	24	0.01	0.08	0.01	0.05
1	0	0.11	1	t	1	Smittia sp. 3	Cerastium alpinum	24	0.01	0.08	0.01	0.06
1	0	0.33	1	t	1	Saxifraga tricuspidata	Rhamphomyia sp.	24	0.02	0.05	0.02	0.06
0	0	0.34	1	cv	1	Saxifraga tricuspidata	Anarta richardsoni	24	0.02	0.05	0.03	0.08
0	0	0.34	1	t	1	Saxifraga tricuspidata	Carposcalis carinata	24	0.02	0.05	0.03	0.08
1	0	0.11	1	t	1	Petinarctia stylata	Salix arctica	24	0.01	0.08	0.01	0.05
1	1	0.89	1	t	1	Petinarctia stylata	Dryas integrifolia	24	0.01	0.08	0.11	0.07
1	0	0.20	1	cv	1	Petinarctia stylata	Epilobium latifolium	24	0.01	0.08	0.03	0.06
1	1	0.89	1	t	1	Tipula arctica	Dryas integrifolia	24	0.01	0.09	0.11	0.07

1	0	0.18	1	t	1	Cecidomyiidae sp.	Salix arctica	24	0.01	0.05	0.01	0.05
0	0	0.16	1	t	1	Einfeldia sp. 1	Saxifraga oppositifolia	24	0.01	0.05	0.01	0.05
0	0	0.16	1	cv	1	Cassiope tetragona	Protophormia terraenovae	24	0.01	0.05	0.02	0.08
0	0	0.19	1	t	1	Cassiope tetragona	Corynoneura scutellata	24	0.01	0.05	0.02	0.08
0	0	0.22	1	t	1	Cassiope tetragona	Apis mellifera	24	0.01	0.05	0.02	0.05
0	0	0.33	1	t	1	Metriocnemus obscuripes	Salix arctica	24	0.02	0.03	0.01	0.05
0	0	0.19	1	cv	1	Chironomidae	Arnica alpina	24	0.01	0.07	0.02	0.05
0	1	0.83	1	t	1	Chironomidae	Lychnis triflora	24	0.01	0.07	0.09	0.07
0	0	0.11	1	t	1	Chironomidae	Saxifraga oppositifolia	24	0.01	0.07	0.01	0.05
0	0	0.13	1	t	1	Chironomidae	Taraxacum arctogenum	24	0.01	0.07	0.01	0.04
0	0	0.33	1	cv	1	Rhyncaenus	Salix arctica	24	0.02	0.03	0.01	0.05
0	0	0.16	1	t	1	Cricotopus lestralis	Saxifraga oppositifolia	24	0.01	0.05	0.01	0.05
1	0	0.18	1	t	1	Erigeon compositus	Rhamphomyia nigrita	24	0.01	0.06	0.02	0.08
0	1	0.83	1	t	1	Cricotopus sp.	Lychnis triflora	24	0.01	0.08	0.09	0.07
0	0	0.39	1	cv	1	Lesquerella arctica	Orthocladiinae	24	0.03	0.05	0.02	0.07
1	0	0.34	1	t	1	Lesquerella arctica	Brevicoryne arctica	24	0.03	0.05	0.01	0.04
0	0	0.23	1	t	1	Lesquerella arctica	Lasiestra leucocycla	24	0.03	0.05	0.01	0.08
0	0	0.36	1	t	1	Lesquerella arctica	Peleteriopsis aenea	24	0.03	0.05	0.01	0.05
1	0	0.12	1	cv	1	Boloria sp.	Salix arctica	24	0.01	0.08	0.01	0.05
1	1	0.90	1	t	1	Boloria sp.	Dryas integrifolia	24	0.01	0.08	0.11	0.07
1	0	0.44	1	t	1	Boloria sp.	Potentilla sp.	24	0.01	0.08	0.05	0.05
1	0	0.21	1	t	1	Boloria sp.	Epilobium latifolium	24	0.01	0.08	0.03	0.06
1	0	0.36	1	cv	1	Dolichopus dasyops	Stellaria sp.	24	0.01	0.06	0.03	0.05
0	0	0.25	1	t	1	Draba bellii	Orthocladiinae	24	0.02	0.06	0.02	0.07
0	0	0.44	1	t	1	Draba bellii	Smittia extrema	24	0.02	0.06	0.04	0.07
1	1	0.91	1	t	1	Spilogona dorsata	Dryas integrifolia	24	0.01	0.08	0.11	0.07
1	1	0.52	1	cv	1	Dictyna	Potentilla sp.	24	0.01	0.06	0.05	0.05

1	1	0.93	1	t	1	Rhamphomyia nigrita	Dryas integrifolia	24	0.02	0.08	0.11	0.07
1	1	0.55	1	t	1	Rhamphomyia nigrita	Potentilla sp.	24	0.02	0.08	0.05	0.05
1	1	0.63	1	t	1	Rhamphomyia sp.	Potentilla sp.	24	0.02	0.06	0.05	0.05
1	0	0.23	1	cv	1	Rhamphomyia sp.	Taraxacum arctogenum	24	0.02	0.06	0.01	0.04
0	0	0.34	1	t	1	Saxifraga cernua	Smittia extrema	24	0.01	0.06	0.04	0.07
0	0	0.25	1	t	1	Arnica alpina	Xysticus deichmanni	24	0.02	0.05	0.02	0.08
1	0	0.23	1	t	1	Arnica alpina	Aedes nigripes	24	0.02	0.05	0.01	0.08
0	0	0.26	1	cv	1	Arnica alpina	Spilogona sp.	24	0.02	0.05	0.01	0.05
1	0	0.13	1	t	1	Smittia polaris	Salix arctica	24	0.01	0.08	0.01	0.05
1	1	0.91	1	t	1	Smittia polaris	Dryas integrifolia	24	0.01	0.08	0.11	0.07
1	0	0.16	1	t	1	Aedes impiger	Salix arctica	24	0.02	0.08	0.01	0.05
1	0	0.37	1	cv	1	Aedes impiger	Stellaria sp.	24	0.02	0.08	0.03	0.05
1	1	0.93	1	t	1	Aedes impiger	Dryas integrifolia	24	0.02	0.08	0.11	0.07
0	0	0.19	1	t	1	Saxifraga hirculus	Spilogona latilamina	24	0.02	0.05	0.01	0.08
1	0	0.28	1	t	1	Saxifraga hirculus	Isotoma viridis	24	0.02	0.05	0.01	0.05
1	0	0.24	1	cv	1	Saxifraga hirculus	Spilogona melanosoma	24	0.02	0.05	0.02	0.08
1	1	0.90	1	t	1	Orthocladiinae	Lychnis triflora	24	0.02	0.07	0.09	0.07
0	0	0.21	1	t	1	Orthocladiinae	Cerastium alpinum	24	0.02	0.07	0.01	0.06
1	0	0.16	1	t	1	Protophormia terraenovae	Salix arctica	24	0.02	0.08	0.01	0.05
1	1	0.92	1	cv	1	Protophormia terraenovae	Dryas integrifolia	24	0.02	0.08	0.11	0.07
1	0	0.20	1	t	1	Aedes sp.	Salix arctica	24	0.02	0.08	0.01	0.05
1	1	0.94	1	t	1	Aedes sp.	Dryas integrifolia	24	0.02	0.08	0.11	0.07
1	1	0.59	1	t	1	Aedes sp.	Potentilla sp.	24	0.02	0.08	0.05	0.05
1	0	0.33	1	cv	1	Aedes sp.	Epilobium latifolium	24	0.02	0.08	0.03	0.06
1	0	0.11	1	t	1	Helophilus borealis	Salix arctica	24	0.01	0.09	0.01	0.05
1	1	0.89	1	t	1	Helophilus borealis	Dryas integrifolia	24	0.01	0.09	0.11	0.07
1	1	0.91	1	t	1	Spilogona latilamina	Dryas integrifolia	24	0.01	0.08	0.11	0.07
1	0	0.18	1	cv	1	Micropsectra sp. 3	Salix arctica	24	0.01	0.05	0.01	0.05
1	0	0.18	1	t	1	Acarina	Salix arctica	24	0.01	0.05	0.01	0.05

1	0	0.13	1	t	1	Allopiophila arctica	Salix arctica	24	0.01	0.08	0.01	0.05
1	1	0.91	1	t	1	Allopiophila arctica	Dryas integrifolia	24	0.01	0.08	0.11	0.07
0	1	0.95	1	cv	1	Lychnis triflora	Smittia extrema	24	0.09	0.07	0.04	0.07
0	1	0.80	1	t	1	Lychnis triflora	Chalcidae	24	0.09	0.07	0.01	0.07
0	1	0.87	1	t	1	Lychnis triflora	Corynoneura scutellata	24	0.09	0.07	0.02	0.08
0	1	0.85	1	t	1	Lychnis triflora	Limnophyes sp.	24	0.09	0.07	0.02	0.08
1	1	0.94	1	cv	1	Rhamphomyia filicauda	Dryas integrifolia	24	0.02	0.08	0.11	0.07
1	0	0.44	1	t	1	Collembola sp.	Cerastium arcticum	24	0.01	0.01	0.01	0.01
0	0	0.34	1	t	1	Draba oblongata	Smittia extrema	24	0.01	0.06	0.04	0.07
0	0	0.18	1	t	1	Salix arctica	Limnophyes sp. 3	24	0.01	0.05	0.01	0.05
0	0	0.40	1	cv	1	Salix arctica	Smittia extrema	24	0.01	0.05	0.04	0.07
0	0	0.21	1	t	1	Salix arctica	Smittia polymorpha	24	0.01	0.05	0.01	0.05
0	0	0.22	1	t	1	Salix arctica	Paraphaenocl adius despectus	24	0.01	0.05	0.01	0.05
0	0	0.11	1	t	1	Salix arctica	Eupogonomyi a groenlandica	24	0.01	0.05	0.01	0.09
0	0	0.12	1	cv	1	Salix arctica	Spilogona sanctipauli	24	0.01	0.05	0.01	0.08
0	0	0.24	1	t	1	Salix arctica	Amauronema tus sp.	24	0.01	0.05	0.01	0.04
0	0	0.21	1	t	1	Salix arctica	Corynoneura scutellata	24	0.01	0.05	0.02	0.08
0	0	0.15	1	t	1	Salix arctica	Aedes nigripes	24	0.01	0.05	0.01	0.08
0	0	0.18	1	cv	1	Salix arctica	Limnophyes globifer	24	0.01	0.05	0.01	0.05
0	0	0.20	1	t	1	Salix arctica	Hyposoter luctus	24	0.01	0.05	0.01	0.03
0	0	0.18	1	t	1	Salix arctica	Limnophyes sp.	24	0.01	0.05	0.02	0.08
0	0	0.24	1	t	1	Salix arctica	Apis mellifera	24	0.01	0.05	0.02	0.05
0	0	0.24	1	cv	1	Salix arctica	Carposcalis carinata	24	0.01	0.05	0.03	0.08
0	0	0.25	1	t	1	Salix arctica	Cryptus arcticus	24	0.01	0.05	0.02	0.05
0	0	0.34	1	t	1	Draba cinerea	Smittia extrema	24	0.01	0.06	0.04	0.07

0	0	0.34	1	t	1	Stellaria sp.	Aedes nigripes	24	0.03	0.05	0.01	0.08
0	0	0.34	1	cv	1	Stellaria sp.	Braconidae	24	0.03	0.05	0.01	0.06
0	0	0.42	1	t	1	Stellaria sp.	Peleteriopsis aenea	24	0.03	0.05	0.01	0.05
0	0	0.48	1	t	1	Stellaria sp.	Cryptus arcticus	24	0.03	0.05	0.02	0.05
1	1	0.93	1	t	1	Xysticus deichmanni	Dryas integrifolia	24	0.02	0.08	0.11	0.07
0	0	0.34	1	cv	1	Smittia extrema	Saxifraga oppositifolia	24	0.04	0.07	0.01	0.05
0	0	0.37	1	t	1	Smittia extrema	Cerastium alpinum	24	0.04	0.07	0.01	0.06
1	1	0.86	1	t	1	Dryas integrifolia	Sympistris labradoris	24	0.11	0.07	0.01	0.09
1	1	0.86	1	t	1	Dryas integrifolia	Olethreutes inquietana	24	0.11	0.07	0.01	0.09
1	1	0.89	1	cv	1	Dryas integrifolia	Boreellus atriceps	24	0.11	0.07	0.01	0.08
1	1	0.86	1	t	1	Dryas integrifolia	Phalacrodira nigropilosa	24	0.11	0.07	0.01	0.09
1	1	0.86	1	t	1	Dryas integrifolia	Eupogonomyi a groenlandica	24	0.11	0.07	0.01	0.09
1	1	0.91	1	t	1	Dryas integrifolia	Spilogona tundrae	24	0.11	0.07	0.02	0.08
1	1	0.87	1	cv	1	Dryas integrifolia	Spilogona sanctipauli	24	0.11	0.07	0.01	0.08
1	1	0.92	1	t	1	Dryas integrifolia	Pseudodiame sa arctica	24	0.11	0.07	0.02	0.08
1	1	0.93	1	t	1	Dryas integrifolia	Corynoneura scutellata	24	0.11	0.07	0.02	0.08
1	1	0.94	1	t	1	Dryas integrifolia	Anarta richardsoni	24	0.11	0.07	0.03	0.08
1	1	0.90	1	cv	1	Dryas integrifolia	Aedes nigripes	24	0.11	0.07	0.01	0.08
1	1	0.87	1	t	1	Dryas integrifolia	Olethreutes mengelana	24	0.11	0.07	0.01	0.08
1	1	0.93	1	t	1	Dryas integrifolia	Prosmittia nanseni	24	0.11	0.07	0.02	0.08
1	1	0.91	1	t	1	Dryas integrifolia	Amauronema tus amentorum	24	0.11	0.07	0.02	0.08
1	1	0.91	1	cv	1	Dryas integrifolia	Limnophyes sp.	24	0.11	0.07	0.02	0.08
1	1	0.86	1	t	1	Dryas integrifolia	Colias hecla	24	0.11	0.07	0.01	0.08
1	1	0.91	1	t	1	Dryas integrifolia	Chironomus sp.	24	0.11	0.07	0.02	0.08
1	1	0.92	1	t	1	Dryas integrifolia	Spilogona melanosoma	24	0.11	0.07	0.02	0.08

1	1	0.89	1	cv	1	Dryas integrifolia	Rhamphomyia lamelliseta	24	0.11	0.07	0.01	0.08
1	1	0.87	1	t	1	Dryas integrifolia	Lasiestra leucocycla	24	0.11	0.07	0.01	0.08
1	1	0.86	1	t	1	Dryas integrifolia	Spilogona almquisti	24	0.11	0.07	0.01	0.09
1	1	0.94	1	t	1	Dryas integrifolia	Carposcalis carinata	24	0.11	0.07	0.03	0.08
1	0	0.12	1	cv	1	Boreellus atriceps	Saxifraga oppositifolia	24	0.01	0.08	0.01	0.05
1	1	0.52	1	t	1	Orthocladius consobrinus	Potentilla sp.	24	0.01	0.06	0.05	0.05
0	0	0.19	1	t	1	Saxifraga oppositifolia	Smittia polymorpha	24	0.01	0.05	0.01	0.05
0	0	0.34	1	t	1	Saxifraga oppositifolia	Psectrocladius barbatimanus	24	0.01	0.05	0.03	0.04
0	0	0.15	1	cv	1	Saxifraga oppositifolia	Tanytarsus niger	24	0.01	0.05	0.01	0.05
0	0	0.18	1	t	1	Saxifraga oppositifolia	Prosmittia nanseni	24	0.01	0.05	0.02	0.08
0	0	0.15	1	t	1	Saxifraga oppositifolia	Procladius sp.	24	0.01	0.05	0.01	0.05
0	0	0.15	1	t	1	Saxifraga oppositifolia	Amauronematus amentorum	24	0.01	0.05	0.02	0.08
0	0	0.15	1	cv	1	Saxifraga oppositifolia	Scatophaga nigripalpis	24	0.01	0.05	0.01	0.05
0	0	0.21	1	t	1	Saxifraga oppositifolia	Fucellia pictipennis	24	0.01	0.05	0.01	0.04
0	0	0.15	1	t	1	Saxifraga oppositifolia	Chironomus sp.	24	0.01	0.05	0.02	0.08
0	0	0.22	1	t	1	Saxifraga oppositifolia	Apis mellifera	24	0.01	0.05	0.02	0.05
0	0	0.13	1	cv	1	Saxifraga oppositifolia	Rhamphomyia lamelliseta	24	0.01	0.05	0.01	0.08
0	1	0.73	1	t	1	Saxifraga oppositifolia	Orthcladius sp.	24	0.01	0.05	0.07	0.07
0	0	0.22	1	t	1	Saxifraga oppositifolia	Carposcalis carinata	24	0.01	0.05	0.03	0.08
0	0	0.21	1	t	1	Saxifraga oppositifolia	Pardosa glacialis	24	0.01	0.05	0.01	0.04
0	0	0.24	1	cv	1	Megabombus hyperboreus	Epilobium latifolium	24	0.01	0.07	0.03	0.06
0	0	0.45	1	t	1	Potentilla sp.	Chalcidae	24	0.05	0.05	0.01	0.07
1	0	0.50	1	t	1	Potentilla sp.	Aedes nigripes	24	0.05	0.05	0.01	0.08
1	0	0.43	1	t	1	Potentilla sp.	Olethreutes mengelana	24	0.05	0.05	0.01	0.08

1	1	0.54	1	cv	1	Potentilla sp.	Spilogona melanosoma	24	0.05	0.05	0.02	0.08
1	0	0.49	1	t	1	Potentilla sp.	Braconidae	24	0.05	0.05	0.01	0.06
1	0	0.32	1	t	1	Braya humilis	Hyposoter luctus	24	0.01	0.02	0.01	0.03
1	0	0.37	1	t	1	Braya humilis	Cryptus arcticus	24	0.01	0.02	0.02	0.05
0	0	0.14	1	cv	1	Cerastium alpinum	Rhamphomyia lamelliseta	24	0.01	0.06	0.01	0.08
0	0	0.12	1	t	1	Taraxacum arctogenum	Colias hecla	24	0.01	0.04	0.01	0.08
1	0	0.30	1	t	1	Saxifraga flagellaris	Spilogona sp.	24	0.01	0.02	0.01	0.05
1	0	0.14	0	t	1	Amauronematus amentorum	Pedicularis hirsuta	24	0.02	0.08	0.01	0.05
0	0	0.26	0	cv	1	Ichneumonidae	Carposcalis carinata	24	0.01	0.05	0.03	0.08
1	0	0.12	0	t	1	Smittia polaris	Saxifraga oppositifolia	24	0.01	0.08	0.01	0.05
0	0	0.10	0	t	1	Limnophyes sp.	Spilogona almquisti	24	0.02	0.08	0.01	0.09
0	0	0.32	0	t	1	Rhamphomyia filicauda	Cerastium arcticum	24	0.02	0.08	0.01	0.01
0	0	0.12	0	cv	1	Prosmittia nanseni	Colias hecla	24	0.02	0.08	0.01	0.08
0	0	0.15	0	t	1	Anarta richardsoni	Olethreutes mengelana	24	0.03	0.08	0.01	0.08
0	0	0.11	0	t	1	Cricotopus lestralis	Boloria sp.	24	0.01	0.05	0.01	0.08
0	1	0.95	0	t	1	Lychnis triflora	Stellaria sp.	24	0.09	0.07	0.03	0.05
0	0	0.17	0	cv	1	Acarina	Limnophyes sp.	24	0.01	0.05	0.02	0.08
0	1	0.92	0	t	1	Saxifraga hirculus	Lychnis triflora	24	0.02	0.05	0.09	0.07
0	0	0.23	0	t	1	Acarina	Anarta richardsoni	24	0.01	0.05	0.03	0.08
0	0	0.13	0	t	1	Polygonum viviparum	Spilogona sp.	24	0.01	0.07	0.01	0.05
0	0	0.28	0	cv	1	Saxifraga tricuspidata	Erigeon compositus	24	0.02	0.05	0.01	0.06
1	0	0.49	0	t	1	Rhyncaenus sp.	Saxifraga flagellaris	24	0.02	0.03	0.01	0.02
0	0	0.13	0	t	1	Ichneumonidae	Spilogona almquisti	24	0.01	0.05	0.01	0.09
0	0	0.07	0	t	1	Cricotopus sp.	Eupogonomyia groenlandica	24	0.01	0.08	0.01	0.09
0	0	0.17	0	cv	1	Erigeon compositus	Pedicularis hirsuta	24	0.01	0.06	0.01	0.05
0	0	0.42	0	t	1	Rhyncaenus sp.	Saxifraga hirculus	24	0.02	0.03	0.02	0.05

0	0	0.09	0	t	1	Braconidae	Spilogona almquisti	24	0.01	0.06	0.01	0.09
0	0	0.16	0	t	1	Heterotrissocladius	Prosmittia nanseni	24	0.01	0.08	0.02	0.08
0	0	0.13	0	cv	1	Pedicularis arctica	Lasiestra leucocycla	24	0.01	0.06	0.01	0.08
0	0	0.29	0	t	1	Cricotopus sp.	Stellaria sp.	24	0.01	0.08	0.03	0.05
0	0	0.31	0	t	1	Micropsectra sp. 3	Cerastium arcticum	24	0.01	0.05	0.01	0.01
1	0	0.46	0	t	1	Crymodes exulis	Limnophyes sp. 3	24	0.04	0.04	0.01	0.05
0	0	0.17	0	cv	1	Chironomus sp. 3	Rhamphomyia nigrita	24	0.01	0.05	0.02	0.08
0	0	0.10	0	t	1	Xysticus deichmanni	Phalacrodira nigropilosa	24	0.02	0.08	0.01	0.09
0	0	0.42	0	t	1	Cerastium arcticum	Cryptus arcticus	24	0.01	0.01	0.02	0.05
1	0	0.23	0	t	1	Bradysia sp.	Amauronematus sp.	24	0.01	0.05	0.01	0.04
1	0	0.12	0	cv	1	Sympistris labradoris	Pedicularis arctica	24	0.01	0.09	0.01	0.06
0	0	0.17	0	t	1	Limnophyes sp. 3	Chironomus sp.	24	0.01	0.05	0.02	0.08
0	0	0.16	0	t	1	Tanytarsus niger	Scatophaga nigripalpis	24	0.01	0.05	0.01	0.05
0	0	0.20	0	t	1	Draba oblongata	Cryptus arcticus	24	0.01	0.06	0.02	0.05
0	0	0.14	0	cv	1	Phalacrodira nigropilosa	Anarta richardsoni	24	0.01	0.09	0.03	0.08
0	0	0.07	0	t	1	Eupogonomyia groenlandica	Spilogona almquisti	24	0.01	0.09	0.01	0.09
0	0	0.11	0	t	1	Megabombus hyperboreus	Rhamphomyia lamelliseta	24	0.01	0.07	0.01	0.08
1	0	0.23	0	t	1	Acarina	Fucellia pictipennis	24	0.01	0.05	0.01	0.04
1	0	0.37	0	cv	1	Amauronematus sp.	Saxifraga flagellaris	24	0.01	0.04	0.01	0.02
1	0	0.43	0	t	1	Papaver radicatum	Isotoma viridis	24	0.04	0.06	0.01	0.05
0	0	0.19	0	t	1	Cerastium alpinum	Taraxacum arctogenum	24	0.01	0.06	0.01	0.04
1	1	0.63	0	t	1	Phalacrodira nigropilosa	Orthcladius sp.	24	0.01	0.09	0.07	0.07
0	0	0.20	0	cv	1	Micropsectra sp. 3	Pseudodiamesa arctica	24	0.01	0.05	0.02	0.08
0	0	0.09	0	t	1	Eupogonomyia groenlandica	Braconidae	24	0.01	0.09	0.01	0.06
0	0	0.13	0	t	1	Corynoneura scutellata	Olethreutes mengelana	24	0.02	0.08	0.01	0.08
1	0	0.17	0	t	1	Spilogona tundrae	Pedicularis arctica	24	0.02	0.08	0.01	0.06

0	0	0.16	0	cv	1	Boloria sp.	Apis mellifera	24	0.01	0.08	0.02	0.05
0	0	0.14	0	t	1	Micropsectra sp. 3	Draba cinerea	24	0.01	0.05	0.01	0.06
0	0	0.09	0	t	1	Phalacrodira nigropilosa	Braconidae	24	0.01	0.09	0.01	0.06
1	0	0.27	0	t	1	Paraphaenocladius despectus	Fucellia pictipennis	24	0.01	0.05	0.01	0.04
1	0	0.15	0	cv	1	Cricotopus lestralis	Orthocladius consobrinus	24	0.01	0.05	0.01	0.06
1	0	0.13	0	t	1	Spilogona dorsata	Salix arctica	24	0.01	0.08	0.01	0.05
0	0	0.14	0	t	1	Anarta richardsoni	Colias hecla	24	0.03	0.08	0.01	0.08
0	0	0.32	0	t	1	Rhyncaenus sp.	Chironomus sp.	24	0.02	0.03	0.02	0.08
1	0	0.23	0	cv	1	Arnica alpina	Orthocladius consobrinus	24	0.02	0.05	0.01	0.06
1	0	0.19	0	t	1	Brevicoryne arctica	Spilogona sp.	24	0.01	0.04	0.01	0.05
0	0	0.07	0	t	1	Sympistris labradoris	Colias hecla	24	0.01	0.09	0.01	0.08
0	1	0.96	0	t	1	Saxifraga tricuspidata	Dryas integrifolia	24	0.02	0.05	0.11	0.07
0	0	0.15	0	cv	1	Erigeon compositus	Boreellus atriceps	24	0.01	0.06	0.01	0.08
1	0	0.18	0	t	1	Einfeldia sp. 1	Brevicoryne arctica	24	0.01	0.05	0.01	0.04
0	0	0.09	0	t	1	Draba cinerea	Olethreutes inquietana	24	0.01	0.06	0.01	0.09
0	0	0.07	0	t	1	Boloria sp.	Helophilus borealis	24	0.01	0.08	0.01	0.09
0	0	0.12	0	cv	1	Rhamphomyia filicauda	Olethreutes mengelana	24	0.02	0.08	0.01	0.08
1	1	0.68	0	t	1	Spilogona latilamina	Orthcladius sp.	24	0.01	0.08	0.07	0.07
0	0	0.20	0	t	1	Smittia polaris	Collembola sp.	24	0.01	0.08	0.01	0.01
0	1	0.94	0	t	1	Erigeon compositus	Dryas integrifolia	24	0.01	0.06	0.11	0.07
1	1	0.55	0	cv	1	Megabombus polaris	Saxifraga tricuspidata	24	0.05	0.08	0.02	0.05
0	0	0.14	0	t	1	Draba cinerea	Spilogona melanosoma	24	0.01	0.06	0.02	0.08
1	0	0.14	0	t	1	Smittia polaris	Taraxacum arctogenum	24	0.01	0.08	0.01	0.04
0	0	0.12	0	t	1	Polygonum viviparum	Saxifraga oppositifolia	24	0.01	0.07	0.01	0.05
0	0	0.29	0	cv	1	Braya humilis	Spilogona melanosoma	24	0.01	0.02	0.02	0.08
0	0	0.12	0	t	1	Salix arctica	Lasiestra leucocycla	24	0.01	0.05	0.01	0.08

0	0	0.19	0	t	1	Aedes impiger	Smittia polymorpha	24	0.02	0.08	0.01	0.05
0	0	0.26	0	t	1	Protophormia terraenovae	Saxifraga flagellaris	24	0.02	0.08	0.01	0.02
0	0	0.11	0	cv	1	Scatophaga nigripalpis	Spilogona almquisti	24	0.01	0.05	0.01	0.09
0	0	0.09	0	t	1	Petinarctia stylata	Spilogona dorsata	24	0.01	0.08	0.01	0.08
0	0	0.07	0	t	1	Spilogona sanctipauli	Spilogona almquisti	24	0.01	0.08	0.01	0.09
0	1	0.58	0	t	1	Papaver radicatum	Metriocnemus obscuripes	24	0.04	0.06	0.02	0.03
0	1	0.50	0	cv	1	Saxifraga cernua	Potentilla sp.	24	0.01	0.06	0.05	0.05
0	0	0.10	0	t	1	Smittia sp. 3	Protophormia terraenovae	24	0.01	0.08	0.02	0.08
0	0	0.11	0	t	1	Salix arctica	Spilogona almquisti	24	0.01	0.05	0.01	0.09
0	0	0.12	0	t	1	Boloria sp.	Brevicoryne arctica	24	0.01	0.08	0.01	0.04
1	0	0.11	0	cv	1	Spilogona latilamina	Draba oblongata	24	0.01	0.08	0.01	0.06
0	0	0.08	0	t	1	Spilogona sanctipauli	Chalcidae	24	0.01	0.08	0.01	0.07
0	0	0.27	0	t	1	Pedicularis capitata	Saxifraga flagellaris	24	0.01	0.05	0.01	0.02
0	0	0.12	0	t	1	Polygonum viviparum	Spilogona tundrae	24	0.01	0.07	0.02	0.08
0	0	0.24	0	cv	1	Corynoneura scutellata	Pardosa glacialis	24	0.02	0.08	0.01	0.04
0	0	0.22	0	t	1	Dictyna borealis	Collembola sp.	24	0.01	0.06	0.01	0.01
0	0	0.27	0	t	1	Draba bellii	Amauronematus sp.	24	0.02	0.06	0.01	0.04
0	0	0.23	0	t	1	Chironomus sp. 3	Saxifraga hirculus	24	0.01	0.05	0.02	0.05
1	0	0.24	0	cv	1	Isotoma viridis	Peleteriopsis aenea	24	0.01	0.05	0.01	0.05
0	0	0.21	0	t	1	Pegomyia sp. 2	Pseudodiamesa arctica	24	0.01	0.06	0.02	0.08
0	0	0.13	0	t	1	Phalacrodira nigropilosa	Isotoma viridis	24	0.01	0.09	0.01	0.05
0	0	0.39	0	t	1	Psectrocladius barbatimanus	Prosmittia nanseni	24	0.03	0.04	0.02	0.08
1	0	0.42	0	cv	1	Aedes sp.	Stellaria sp.	24	0.02	0.08	0.03	0.05
0	0	0.16	0	t	1	Rhamphomyia nigrita	Tanytarsus niger	24	0.02	0.08	0.01	0.05
0	0	0.15	0	t	1	Polygonum viviparum	Smittia polymorpha	24	0.01	0.07	0.01	0.05

1	0	0.15	0	t	1	Chironomus sp. 3	Orthocladius consobrinus	24	0.01	0.05	0.01	0.06
0	0	0.11	0	cv	1	Petinarctia stylata	Acarina	24	0.01	0.08	0.01	0.05
0	0	0.16	0	t	1	Smittia polaris	Paraphaenocladius despectus	24	0.01	0.08	0.01	0.05
1	0	0.29	0	t	1	Bradysia sp.	Erysimum pallasii	24	0.01	0.05	0.02	0.05
0	1	0.93	0	t	1	Einfeldia sp. 1	Dryas integrifolia	24	0.01	0.05	0.11	0.07
0	0	0.19	0	cv	1	Cassiope tetragona	Smittia polymorpha	24	0.01	0.05	0.01	0.05
0	0	0.12	0	t	1	Boreellus atriceps	Scatophaga nigripalpis	24	0.01	0.08	0.01	0.05
0	0	0.09	0	t	1	Smittia sp. 3	Spilogona latilamina	24	0.01	0.08	0.01	0.08
0	0	0.12	0	t	1	Erigeon compositus	Spilogona sanctipauli	24	0.01	0.06	0.01	0.08
0	0	0.18	0	cv	1	Amauronematus amentorum	Hyposoter luctus	24	0.02	0.08	0.01	0.03
1	0	0.18	0	t	1	Brevicoryne arctica	Procladius sp.	24	0.01	0.04	0.01	0.05
0	0	0.11	0	t	1	Acarina	Phalacrodira nigropilosa	24	0.01	0.05	0.01	0.09
0	0	0.25	0	t	1	Aedes sp.	Pardosa glacialis	24	0.02	0.08	0.01	0.04
1	0	0.20	0	cv	1	Bradysia sp.	Peleteriopsis aenea	24	0.01	0.05	0.01	0.05
0	0	0.11	0	t	1	Chironomus sp. 3	Tipula arctica	24	0.01	0.05	0.01	0.09
0	0	0.15	0	t	1	Pogonomyoides segnis	Protophormia terraenovae	24	0.02	0.08	0.02	0.08
1	0	0.13	0	t	1	Spilogona dorsata	Cerastium alpinum	24	0.01	0.08	0.01	0.06
0	0	0.22	0	cv	1	Silene acaulis	Hyposoter luctus	24	0.01	0.06	0.01	0.03
0	0	0.27	0	t	1	Braya humilis	Tanytarsus niger	24	0.01	0.02	0.01	0.05
0	0	0.26	0	t	1	Draba bellii	Anarta richardsoni	24	0.02	0.06	0.03	0.08
1	0	0.33	0	t	1	Cecidomyiidae sp.	Metriocnemus obscuripes	24	0.01	0.05	0.02	0.03
0	0	0.21	0	cv	1	Einfeldia sp. 1	Paraphaenocladius despectus	24	0.01	0.05	0.01	0.05
0	0	0.18	0	t	1	Chalcidae	Apis mellifera	24	0.01	0.07	0.02	0.05
0	0	0.49	0	t	1	Megabombus hyperboreus	Potentilla sp.	24	0.01	0.07	0.05	0.05

1	0	0.12	0	t	1	Plebeius aquilo	Pedicularis capitata	24	0.01	0.08	0.01	0.05
0	0	0.11	0	cv	1	Bradysia sp.	Spilogona almquisti	24	0.01	0.05	0.01	0.09
1	0	0.18	0	t	1	Rhamphomyia nigrita	Taraxacum arctogenum	24	0.02	0.08	0.01	0.04
0	0	0.22	0	t	1	Smittia polymorpha	Taraxacum arctogenum	24	0.01	0.05	0.01	0.04
0	0	0.11	0	t	1	Saxifraga cernua	Boreellus atriceps	24	0.01	0.06	0.01	0.08
0	0	0.18	0	cv	1	Erigeon compositus	Spilogona sp.	24	0.01	0.06	0.01	0.05
0	0	0.24	0	t	1	Pedicularis arctica	Pardosa glacialis	24	0.01	0.06	0.01	0.04
1	0	0.13	0	t	1	Boloria sp.	Pedicularis arctica	24	0.01	0.08	0.01	0.06
0	0	0.36	0	t	1	Metriocnemus obscuripes	Pseudodiame sa arctica	24	0.02	0.03	0.02	0.08
0	0	0.15	0	cv	1	Brevicoryne arctica	Rhamphomyi a lamelliseta	24	0.01	0.04	0.01	0.08
0	0	0.28	0	t	1	Anarta richardsoni	Carposcalis carinata	24	0.03	0.08	0.03	0.08
0	0	0.29	0	t	1	Paraphaenocla dius despectus	Apis mellifera	24	0.01	0.05	0.02	0.05
0	0	0.10	0	t	1	Pedicularis capitata	Phalacrodira nigropilosa	24	0.01	0.05	0.01	0.09
0	0	0.08	0	cv	1	Helophilus borealis	Boreellus atriceps	24	0.01	0.09	0.01	0.08
0	0	0.25	0	t	1	Arnica alpina	Amauronema tus amentorum	24	0.02	0.05	0.02	0.08
1	1	0.68	0	t	1	Boreellus atriceps	Orthcladius sp.	24	0.01	0.08	0.07	0.07
0	0	0.27	0	t	1	Saxifraga hirculus	Hyposoter luctus	24	0.02	0.05	0.01	0.03
0	0	0.23	0	cv	1	Erysimum pallasii	Allopiophila arctica	24	0.02	0.05	0.01	0.08
0	1	0.92	0	t	1	Dolichopus dasyops	Dryas integrifolia	24	0.01	0.06	0.11	0.07
0	0	0.28	0	t	1	Heterotrissocl adius	Rhyncaenus sp.	24	0.01	0.08	0.02	0.03
0	0	0.30	0	t	1	Pogonomyoid es segnis	Rhyncaenus sp.	24	0.02	0.08	0.02	0.03
0	0	0.07	0	cv	1	Boloria sp.	Olethreutes inquietana	24	0.01	0.08	0.01	0.09
0	0	0.37	0	t	1	Fucellia pictipennis	Epilobium latifolium	24	0.01	0.04	0.03	0.06
1	0	0.13	0	t	1	Protophormia terraenovae	Draba oblongata	24	0.02	0.08	0.01	0.06
0	0	0.27	0	t	1	Lesquerella arctica	Boreellus atriceps	24	0.03	0.05	0.01	0.08

0	0	0.35	0	cv	1	Smittia extrema	Scatophaga nigripalpis	24	0.04	0.07	0.01	0.05
0	0	0.27	0	t	1	Saxifraga tricuspidata	Limnophyes sp.	24	0.02	0.05	0.02	0.08
0	0	0.16	0	t	1	Aedes impiger	Limnophyes globifer	24	0.02	0.08	0.01	0.05
0	0	0.10	0	t	1	Pedicularis capitata	Spilogona almquisti	24	0.01	0.05	0.01	0.09
0	0	0.17	0	cv	1	Rhamphomyia filicauda	Procladius sp.	24	0.02	0.08	0.01	0.05
0	0	0.10	0	t	1	Protophormia terraenovae	Lasiestra leucocycla	24	0.02	0.08	0.01	0.08
0	0	0.17	0	t	1	Cassiope tetragona	Spilogona melanosoma	24	0.01	0.05	0.02	0.08
0	0	0.35	0	t	1	Metriocnemus obscuripes	Taraxacum arctogenum	24	0.02	0.03	0.01	0.04
0	0	0.23	0	cv	1	Lesquerella arctica	Olethreutes mengelana	24	0.03	0.05	0.01	0.08
1	0	0.17	0	t	1	Cricotopus lestralis	Spilogona sp.	24	0.01	0.05	0.01	0.05
0	1	0.98	0	t	1	Papaver radicatum	Dryas integrifolia	24	0.04	0.06	0.11	0.07
0	0	0.22	0	t	1	Saxifraga tricuspidata	Megabombus hyperboreus	24	0.02	0.05	0.01	0.07
0	1	0.52	0	cv	1	Draba bellii	Crymodes exulis	24	0.02	0.06	0.04	0.04
0	0	0.11	0	t	1	Cassiope tetragona	Olethreutes mengelana	24	0.01	0.05	0.01	0.08
1	0	0.21	0	t	1	Ichneumonidae	Erigeon compositus	24	0.01	0.05	0.01	0.06
1	0	0.09	0	t	1	Tipula arctica	Draba oblongata	24	0.01	0.09	0.01	0.06
0	0	0.11	0	cv	1	Chironomus sp. 3	Olethreutes inquietana	24	0.01	0.05	0.01	0.09
0	0	0.36	0	t	1	Papaver radicatum	Pedicularis capitata	24	0.04	0.06	0.01	0.05
0	0	0.27	0	t	1	Metriocnemus obscuripes	Boreellus atriceps	24	0.02	0.03	0.01	0.08
0	1	0.64	0	t	1	Potentilla sp.	Cryptus arcticus	24	0.05	0.05	0.02	0.05
0	0	0.27	0	cv	1	Erysimum pallasii	Tanytarsus niger	24	0.02	0.05	0.01	0.05
0	0	0.11	0	t	1	Petinarctia stylata	Rhamphomyia nigrita	24	0.01	0.08	0.02	0.08
0	0	0.10	0	t	1	Smittia polaris	Chalcidae	24	0.01	0.08	0.01	0.07
1	0	0.17	0	t	1	Cecidomyiidae sp.	Acarina	24	0.01	0.05	0.01	0.05
0	0	0.14	0	cv	1	Spilogona melanosoma	Braconidae	24	0.02	0.08	0.01	0.06
0	0	0.16	0	t	1	Cassiope tetragona	Rhamphomyia nigrita	24	0.01	0.05	0.02	0.08

0	0	0.19	0	t	1	Pogonomyoides segnis	Aedes sp.	24	0.02	0.08	0.02	0.08
0	0	0.21	0	t	1	Pogonomyoides segnis	Anarta richardsoni	24	0.02	0.08	0.03	0.08
0	0	0.19	0	cv	1	Chironomus sp. 3	Spilogona deflorata	24	0.01	0.05	0.02	0.08
0	0	0.39	0	t	1	Crymodes exulis	Boreellus atriceps	24	0.04	0.04	0.01	0.08
0	0	0.09	0	t	1	Draba cinerea	Sympistris labradoris	24	0.01	0.06	0.01	0.09
0	0	0.10	0	t	1	Phalacrodira nigropilosa	Procladius sp.	24	0.01	0.09	0.01	0.05
0	0	0.13	0	cv	1	Cricotopus lestralis	Polygonum viviparum	24	0.01	0.05	0.01	0.07
0	0	0.20	0	t	1	Orthocladius consobrinus	Carposcalis carinata	24	0.01	0.06	0.03	0.08
0	0	0.32	0	t	1	Rhyncaenus sp.	Tanytarsus niger	24	0.02	0.03	0.01	0.05
0	0	0.16	0	t	1	Saxifraga hirculus	Olethreutes inquietana	24	0.02	0.05	0.01	0.09
0	0	0.17	0	cv	1	Aedes nigripes	Peleteriopsis aenea	24	0.01	0.08	0.01	0.05
0	1	0.91	0	t	1	Dryas integrifolia	Pedicularis hirsuta	24	0.11	0.07	0.01	0.05
1	1	0.75	1	t	1	Helichrysum scorpioides	Incurviseta sp. D	27	0.01	0.04	0.06	0.04
0	0	0.27	1	t	1	Helichrysum scorpioides	Tephritis sp. B	27	0.01	0.04	0.01	0.04
1	0	0.40	1	cv	1	Helichrysum scorpioides	Oreixenica orichora	27	0.01	0.04	0.02	0.04
0	0	0.27	1	t	1	Leioproctus sp.2	Microseris lanceolata	27	0.00	0.04	0.02	0.04
0	0	0.34	1	t	1	Stylidium graminifolium	Lasioglossum sp. 4	27	0.01	0.03	0.01	0.04
1	1	0.54	1	t	1	Stylidium graminifolium	Megachile sp.	27	0.01	0.03	0.03	0.02
0	0	0.44	1	cv	1	Stylidium graminifolium	Oreixenica orichora	27	0.01	0.03	0.02	0.04
1	0	0.29	1	t	1	Asperula gunnii	Coenosia sp. A	27	0.01	0.03	0.00	0.02
0	0	0.30	1	t	1	Asperula gunnii	Poecilohetaerus sp.	27	0.01	0.03	0.01	0.04
0	0	0.26	1	t	1	Olearia phlogopappa	Prosena sp. C	27	0.01	0.03	0.01	0.04
0	0	0.29	1	cv	1	Olearia phlogopappa	Poecilohetaerus sp.	27	0.01	0.03	0.01	0.04
1	1	0.71	1	t	1	Calliphora sp.	Orites lancifolia	27	0.00	0.05	0.06	0.04
0	0	0.36	1	t	1	Unidentified Family	Baeckia gunniana	27	0.02	0.03	0.01	0.03

0	0	0.32	1	t	1	Unidentified Family	Epacris petrophila	27	0.02	0.03	0.01	0.03
0	0	0.28	1	cv	1	Unidentified Family	Aciphylla glacialis	27	0.02	0.03	0.00	0.04
0	1	0.61	1	t	1	Unidentified Family	Hypochoeris radicata	27	0.02	0.03	0.04	0.04
1	1	0.88	1	t	1	Lasioglossum sp. 3	Orites lancifolia	27	0.03	0.04	0.06	0.04
1	0	0.48	1	t	1	Lasioglossum sp. 3	Prostanthera cuneata	27	0.03	0.04	0.01	0.04
1	1	0.71	1	cv	1	Syrphus sp.	Orites lancifolia	27	0.00	0.05	0.06	0.04
1	1	0.71	1	t	1	Rutilia sp.	Orites lancifolia	27	0.00	0.05	0.06	0.04
1	0	0.37	1	t	1	Neopaxia australasica	Scaptomyza australis	27	0.01	0.02	0.01	0.02
1	0	0.33	1	t	1	Neopaxia australasica	Coenosia sp. A	27	0.01	0.02	0.00	0.02
0	0	0.35	1	cv	1	Neopaxia australasica	Poecilohetaer us sp.	27	0.01	0.02	0.01	0.04
1	0	0.39	1	t	1	Brachycome scapiformis	Empis sp. C	27	0.01	0.03	0.02	0.03
1	1	0.63	1	t	1	Limnophora	Craspedia sp.	27	0.00	0.04	0.05	0.04
0	1	0.67	1	t	1	Musca vetustissima (males)	Gentianella diemensis	27	0.03	0.03	0.03	0.04
0	0	0.47	1	cv	1	Musca vetustissima (males)	Baeckia gunniana	27	0.03	0.03	0.01	0.03
0	1	0.51	1	t	1	Musca vetustissima (males)	Helichrysum alpinum	27	0.03	0.03	0.02	0.04
0	0	0.44	1	t	1	Musca vetustissima (males)	Helipterum albicans	27	0.03	0.03	0.01	0.03
0	0	0.44	1	t	1	Musca vetustissima (males)	Epacris petrophila	27	0.03	0.03	0.01	0.03
1	1	0.67	1	cv	1	Musca vetustissima (males)	Epilobium gunnianum	27	0.03	0.03	0.03	0.03
0	0	0.39	1	t	1	Musca vetustissima (males)	Leucopogon montanus	27	0.03	0.03	0.00	0.04
0	1	0.72	1	t	1	Musca vetustissima (males)	Hypochoeris radicata	27	0.03	0.03	0.04	0.04
0	0	0.31	1	t	1	Calliphora fulvicoxa	Phebalium ovatifolium	27	0.00	0.04	0.02	0.04
1	1	0.72	1	cv	1	Senecio pectinatus	Incurviseta sp. D	27	0.01	0.04	0.06	0.04

1	0	0.37	1	t	1	Senecio pectinatus	Oreixenica orichora	27	0.01	0.04	0.02	0.04
1	1	0.72	1	t	1	Orites lancifolia	Unidentified no. 2	27	0.06	0.04	0.01	0.04
1	1	0.96	1	t	1	Orites lancifolia	Incurviseta sp. D	27	0.06	0.04	0.06	0.04
1	1	0.91	1	cv	1	Orites lancifolia	Lasioglossum sp. 5	27	0.06	0.04	0.04	0.04
0	1	0.73	1	t	1	Orites lancifolia	Calliphora sp. A	27	0.06	0.04	0.01	0.04
0	1	0.68	1	t	1	Orites lancifolia	Calliphora sp. B	27	0.06	0.04	0.00	0.04
1	1	0.72	1	t	1	Orites lancifolia	Euryglossa sp. 2	27	0.06	0.04	0.01	0.04
1	1	0.68	1	cv	1	Orites lancifolia	Comptosia sylvana	27	0.06	0.04	0.00	0.04
1	1	0.68	1	t	1	Orites lancifolia	Unidentified Family Dolichopodidae	27	0.06	0.04	0.00	0.05
1	1	0.71	1	t	1	Orites lancifolia	Hyphesma sp.	27	0.06	0.04	0.01	0.05
0	1	0.70	1	t	1	Orites lancifolia	Leioproctus sp. 1 (female)	27	0.06	0.04	0.00	0.04
1	1	0.73	1	cv	1	Orites lancifolia	Exoneura sp. (female)	27	0.06	0.04	0.01	0.04
0	1	0.69	1	t	1	Orites lancifolia	Euryinae sp. 1	27	0.06	0.04	0.00	0.04
1	1	0.68	1	t	1	Orites lancifolia	Exoneura sp. (male)	27	0.06	0.04	0.00	0.05
1	1	0.71	1	t	1	Orites lancifolia	Calliphora stygia	27	0.06	0.04	0.01	0.05
1	1	0.82	1	cv	1	Orites	Helina sp. A	27	0.06	0.04	0.02	0.04
0	1	0.73	1	t	1	Orites lancifolia	Musca vetustissima (females)	27	0.06	0.04	0.01	0.04
1	1	0.90	1	t	1	Orites lancifolia	Melangyna sp.	27	0.06	0.04	0.04	0.04
1	1	0.89	1	t	1	Orites lancifolia	Leioproctus sp. 3 (male)	27	0.06	0.04	0.04	0.04
0	1	0.68	1	cv	1	Orites lancifolia	Leioproctus sp. 3 (female)	27	0.06	0.04	0.00	0.04
1	1	0.83	1	t	1	Orites lancifolia	Prohardya carinata	27	0.06	0.04	0.02	0.04
0	1	0.76	1	t	1	Orites lancifolia	Poecilohetaerus sp.	27	0.06	0.04	0.01	0.04
0	0	0.26	1	t	1	Chaetophthalamus dorsalis	Prostanthera cuneata	27	0.00	0.04	0.01	0.04
0	0	0.36	1	cv	1	Unidentified no. 1	Baeckia gunniana	27	0.02	0.03	0.01	0.03

0	0	0.35	1	t	1	Unidentified no. 1	Senecio lautus	27	0.02	0.03	0.01	0.04
1	1	0.66	1	t	1	Unidentified no. 1	Pentachondr a pumila	27	0.02	0.03	0.04	0.03
0	0	0.33	1	t	1	Unidentified no. 1	Helipterum albicans	27	0.02	0.03	0.01	0.03
1	1	0.67	1	cv	1	Unidentified no. 3	Craspedia sp.	27	0.01	0.04	0.05	0.04
0	0	0.21	1	t	1	Unidentified no. 3	Brachycome scapigera	27	0.01	0.04	0.00	0.04
0	0	0.27	1	t	1	Unidentified no. 2	Baeckia gunniana	27	0.01	0.04	0.01	0.03
0	0	0.31	1	t	1	Unidentified no. 4	Baeckia gunniana	27	0.01	0.03	0.01	0.03
1	0	0.26	1	cv	1	Unidentified no. 4	Epacris microphylla	27	0.01	0.03	0.00	0.03
0	0	0.22	1	t	1	Unidentified Family Therevidae	Aciphylla glacialis	27	0.00	0.03	0.00	0.04
0	0	0.27	1	t	1	Tapeigaster nigricornis	Baeckia gunniana	27	0.00	0.03	0.01	0.03
0	0	0.28	1	t	1	Empis sp. D	Helipterum albicans	27	0.01	0.03	0.01	0.03
0	0	0.24	1	cv	1	Empis sp. D	Brachycome scapigera	27	0.01	0.03	0.00	0.04
0	0	0.27	1	t	1	Scaptomyza australis	Aciphylla glacialis	27	0.01	0.02	0.00	0.04
1	1	0.94	1	t	1	Craspedia sp.	Incurviseta sp. D	27	0.05	0.04	0.06	0.04
1	1	0.87	1	t	1	Craspedia sp.	Lasioglossum sp. 5	27	0.05	0.04	0.04	0.04
1	1	0.69	1	cv	1	Craspedia sp.	Lasioglossum sp. 4	27	0.05	0.04	0.01	0.04
1	1	0.71	1	t	1	Craspedia sp.	Tephritis sp. A	27	0.05	0.04	0.02	0.04
0	1	0.65	1	t	1	Craspedia sp.	Tephritis sp. B	27	0.05	0.04	0.01	0.04
0	1	0.61	1	t	1	Craspedia sp.	Leioproctus sp. 1 (female)	27	0.05	0.04	0.00	0.04
1	1	0.78	1	cv	1	Craspedia sp.	Empis ap. B	27	0.05	0.04	0.02	0.04
0	1	0.69	1	t	1	Craspedia sp.	Lasioglossum sp.	27	0.05	0.04	0.01	0.04
1	1	0.76	1	t	1	Craspedia sp.	Sapromyza sp.	27	0.05	0.04	0.02	0.04
0	1	0.61	1	t	1	Craspedia sp.	Euryinae sp. 1	27	0.05	0.04	0.00	0.04
0	1	0.65	1	cv	1	Craspedia sp.	Eristalis tenax	27	0.05	0.04	0.01	0.04
1	1	0.68	1	t	1	Craspedia sp.	Alophera sp.	27	0.05	0.04	0.01	0.04
1	1	0.81	1	t	1	Craspedia sp.	Senostoma sp. B	27	0.05	0.04	0.03	0.04

1	1	0.87	1	t	1	Craspedia sp.	Leioproctus sp. 1 (male)	27	0.05	0.04	0.04	0.04
0	1	0.61	1	cv	1	Craspedia sp.	Lasioglossum (Parasphecodes) sp. 1 (female)	27	0.05	0.04	0.00	0.04
0	1	0.65	1	t	1	Craspedia sp.	Senostoma sp. A	27	0.05	0.04	0.01	0.04
0	1	0.65	1	t	1	Craspedia sp.	Prosena sp. C	27	0.05	0.04	0.01	0.04
1	1	0.87	1	t	1	Incurviseta sp. D	Gentianella diemensis	27	0.06	0.04	0.03	0.04
1	1	0.74	1	cv	1	Incurviseta sp. D	Baeckia gunniana	27	0.06	0.04	0.01	0.03
1	1	0.70	1	t	1	Incurviseta sp. D	Senecio gunnii	27	0.06	0.04	0.01	0.04
1	1	0.76	1	t	1	Incurviseta sp. D	Helichrysum alpinum	27	0.06	0.04	0.02	0.04
1	1	0.73	1	t	1	Incurviseta sp. D	Senecio lautus	27	0.06	0.04	0.01	0.04
1	1	0.66	1	cv	1	Incurviseta sp. D	Leptorhynchos squamatus	27	0.06	0.04	0.00	0.04
1	1	0.84	1	t	1	Incurviseta sp. D	Celmisia longifolia	27	0.06	0.04	0.03	0.04
1	1	0.89	1	t	1	Incurviseta sp. D	Euphrasia collina diversicolor	27	0.06	0.04	0.04	0.04
1	1	0.82	1	t	1	Incurviseta sp. D	Wahlenbergia ceracea	27	0.06	0.04	0.02	0.04
1	1	0.66	1	cv	1	Incurviseta sp. D	Brachycome scapigera	27	0.06	0.04	0.00	0.04
1	1	0.76	1	t	1	Incurviseta sp. D	Microseris lanceolata	27	0.06	0.04	0.02	0.04
1	1	0.78	1	t	1	Incurviseta sp. D	Phebalium ovatifolium	27	0.06	0.04	0.02	0.04
1	1	0.66	1	t	1	Incurviseta sp. D	Pemelea ligustrina	27	0.06	0.04	0.00	0.04
1	1	0.67	1	cv	1	Incurviseta sp. D	Prasophyllum alpinum	27	0.06	0.04	0.00	0.03
1	1	0.67	1	t	1	Incurviseta sp. D	Aciphylla glacialis	27	0.06	0.04	0.00	0.04
1	1	0.66	1	t	1	Incurviseta sp. D	Leucopogon montanus	27	0.06	0.04	0.00	0.04
0	1	0.66	1	t	1	Incurviseta sp. D	Brachycome stolonifera	27	0.06	0.04	0.00	0.04
1	1	0.89	1	cv	1	Incurviseta sp. D	Hypochoeris radicata	27	0.06	0.04	0.04	0.04
0	0	0.25	1	t	1	Euryglossa sp.	Achillea millefolium	27	0.00	0.03	0.01	0.03
0	0	0.25	1	t	1	Incurviseta sp. K	Epacris petrophila	27	0.00	0.03	0.01	0.03

0	0	0.46	1	t	1	Gentianella diemensis	Calliphora sp. A	27	0.03	0.04	0.01	0.04
0	0	0.48	1	cv	1	Gentianella diemensis	Lasioglossum sp. 2	27	0.03	0.04	0.01	0.03
1	1	0.77	1	t	1	Gentianella diemensis	Dilophus sp.	27	0.03	0.04	0.04	0.04
1	0	0.48	1	t	1	Gentianella diemensis	Lasioglossum (Parasphecodes) sp. 2 (male)	27	0.03	0.04	0.01	0.03
1	1	0.51	1	t	1	Gentianella diemensis	Lasioglossum sp.	27	0.03	0.04	0.01	0.04
0	0	0.46	1	cv	1	Gentianella diemensis	Musca vetustissima (females)	27	0.03	0.04	0.01	0.04
0	1	0.50	1	t	1	Gentianella diemensis	Poecilohetaerus sp.	27	0.03	0.04	0.01	0.04
1	1	0.56	1	t	1	Gentianella diemensis	Empis sp. C	27	0.03	0.04	0.02	0.03
1	0	0.23	1	t	1	Lasioglossum sp. 6	Oxylobium ellipticum	27	0.00	0.03	0.00	0.03
0	0	0.50	1	cv	1	Lasioglossum sp. 5	Oxylobium ellipticum	27	0.04	0.04	0.00	0.03
1	1	0.57	1	t	1	Lasioglossum sp. 5	Prostanthera cuneata	27	0.04	0.04	0.01	0.04
1	1	0.71	1	t	1	Lasioglossum sp. 5	Celmisia longifolia	27	0.04	0.04	0.03	0.04
1	1	0.53	1	t	1	Lasioglossum sp. 5	Epacris petrophila	27	0.04	0.04	0.01	0.03
1	1	0.79	1	cv	1	Lasioglossum sp. 5	Euphrasia collina diversicolor	27	0.04	0.04	0.04	0.04
1	1	0.69	1	t	1	Lasioglossum sp. 5	Wahlenbergia ceracea	27	0.04	0.04	0.02	0.04
1	1	0.60	1	t	1	Lasioglossum sp. 5	Microseris lanceolata	27	0.04	0.04	0.02	0.04
1	1	0.75	1	t	1	Lasioglossum sp. 5	Kunzea muelleri	27	0.04	0.04	0.03	0.04
0	0	0.48	1	cv	1	Lasioglossum sp. 5	Leucopogon montanus	27	0.04	0.04	0.00	0.04
1	1	0.54	1	t	1	Lasioglossum sp. 5	Achillea millefolium	27	0.04	0.04	0.01	0.03
1	1	0.79	1	t	1	Lasioglossum sp. 5	Hypochoeris radicata	27	0.04	0.04	0.04	0.04
0	0	0.43	1	t	1	Lasioglossum sp. 4	Wahlenbergia ceracea	27	0.01	0.04	0.02	0.04
0	0	0.28	1	cv	1	Calliphora sp. A	Baeckia gunniana	27	0.01	0.04	0.01	0.03
0	0	0.28	1	t	1	Calliphora sp. A	Senecio lautus	27	0.01	0.04	0.01	0.04

0	0	0.42	1	t	1	Calliphora sp. A	Richea continentis	27	0.01	0.04	0.02	0.03
0	0	0.26	1	t	1	Calliphora sp. A	Helipterum albicans	27	0.01	0.04	0.01	0.03
0	0	0.25	1	cv	1	Calliphora sp. A	Epacris petrophila	27	0.01	0.04	0.01	0.03
0	0	0.22	1	t	1	Calliphora sp. A	Brachycome scapigera	27	0.01	0.04	0.00	0.04
0	0	0.34	1	t	1	Calliphora sp. A	Phebalium ovatifolium	27	0.01	0.04	0.02	0.04
0	0	0.22	1	t	1	Calliphora sp. A	Prasophyllum alpinum	27	0.01	0.04	0.00	0.03
0	0	0.22	1	cv	1	Calliphora sp. A	Leucopogon montanus	27	0.01	0.04	0.00	0.04
0	0	0.21	1	t	1	Calliphora sp. A	Brachycome stolonifera	27	0.01	0.04	0.00	0.04
0	0	0.24	1	t	1	Lasioglossum sp. 2	Oxylobium ellipticum	27	0.01	0.03	0.00	0.03
0	0	0.25	1	t	1	Lasioglossum sp. 1	Achillea millefolium	27	0.00	0.03	0.01	0.03
0	0	0.23	1	cv	1	Calliphora sp. B	Prostanthera cuneata	27	0.00	0.04	0.01	0.04
0	0	0.29	1	t	1	Calliphora sp. B	Phebalium ovatifolium	27	0.00	0.04	0.02	0.04
0	1	0.53	1	t	1	Apis mellifera	Pemelea ligustrina	27	0.04	0.04	0.00	0.04
1	1	0.60	1	t	1	Baeckia gunniana	Dilophus sp.	27	0.01	0.03	0.04	0.04
0	0	0.31	1	cv	1	Baeckia gunniana	Coenosia sp. B	27	0.01	0.03	0.01	0.03
0	0	0.35	1	t	1	Baeckia gunniana	Hylaeus sp.	27	0.01	0.03	0.01	0.03
0	0	0.26	1	t	1	Baeckia gunniana	Rivellia sp. F	27	0.01	0.03	0.00	0.04
0	0	0.42	1	t	1	Baeckia gunniana	Helina sp. B	27	0.01	0.03	0.02	0.03
1	1	0.63	1	cv	1	Baeckia gunniana	Tabanus froggatti	27	0.01	0.03	0.04	0.03
0	0	0.32	1	t	1	Baeckia gunniana	Poecilohetaerus sp.	27	0.01	0.03	0.01	0.04
0	0	0.36	1	t	1	Baeckia gunniana	Empis sp. C	27	0.01	0.03	0.02	0.03
0	0	0.37	1	t	1	Senecio gunnii	Chloromerus sp.	27	0.01	0.04	0.02	0.03
0	0	0.28	1	cv	1	Senecio gunnii	Poecilohetaerus sp.	27	0.01	0.04	0.01	0.04
0	0	0.21	1	t	1	Euryglossa sp. 2	Oxylobium ellipticum	27	0.01	0.04	0.00	0.03
1	0	0.34	1	t	1	Euryglossa sp. 1	Oxylobium ellipticum	27	0.02	0.03	0.00	0.03

1	0	0.40	1	t	1	Euryglossa sp. 1	Prostanthera cuneata	27	0.02	0.03	0.01	0.04
0	0	0.30	1	cv	1	Helichrysum alpinum	Musca vetustissima (females)	27	0.02	0.04	0.01	0.04
1	1	0.59	1	t	1	Helichrysum alpinum	Phytoliriomyza sp.	27	0.02	0.04	0.03	0.03
0	0	0.34	1	t	1	Helichrysum alpinum	Poecilohetaerus sp.	27	0.02	0.04	0.01	0.04
1	1	0.70	1	t	1	Dilophus sp.	Wahlenbergia ceracea	27	0.04	0.04	0.02	0.04
1	1	0.80	1	cv	1	Dilophus sp.	Hypochoeris radicata	27	0.04	0.04	0.04	0.04
0	0	0.24	1	t	1	Senecio lautus	Leioproctus sp. 1 (female)	27	0.01	0.04	0.00	0.04
0	0	0.31	1	t	1	Senecio lautus	Lasioglossum sp.	27	0.01	0.04	0.01	0.04
0	0	0.27	1	t	1	Senecio lautus	Exoneura sp. (female)	27	0.01	0.04	0.01	0.04
0	1	0.59	1	cv	1	Senecio lautus	Leioproctus sp. 1 (male)	27	0.01	0.04	0.04	0.04
0	1	0.55	1	t	1	Senecio lautus	Melangyna sp.	27	0.01	0.04	0.04	0.04
0	0	0.38	1	t	1	Senecio lautus	Lasioglossum (Parasphecodes) sp. 1 (male)	27	0.01	0.04	0.02	0.04
0	0	0.28	1	t	1	Senecio lautus	Senostoma sp. A	27	0.01	0.04	0.01	0.04
0	0	0.28	1	cv	1	Senecio lautus	Prosena sp. C	27	0.01	0.04	0.01	0.04
0	0	0.41	1	t	1	Senecio lautus	Oreixenica orichora	27	0.01	0.04	0.02	0.04
0	0	0.31	1	t	1	Senecio lautus	Poecilohetaerus sp.	27	0.01	0.04	0.01	0.04
0	0	0.31	1	t	1	Tephritis sp. A	Helipterum albicans	27	0.02	0.04	0.01	0.03
0	0	0.27	1	cv	1	Tephritis sp. A	Aciphylla glacialis	27	0.02	0.04	0.00	0.04
0	0	0.20	1	t	1	Oxylobium ellipticum	Leioproctus sp. 1 (female)	27	0.00	0.03	0.00	0.04
0	0	0.23	1	t	1	Oxylobium ellipticum	Exoneura sp. (female)	27	0.00	0.03	0.01	0.04
0	0	0.20	1	t	1	Oxylobium ellipticum	Euryinae sp. 1	27	0.00	0.03	0.00	0.04
0	0	0.20	1	cv	1	Oxylobium ellipticum	Lasioglossum (Parasphecodes) sp. 1 (female)	27	0.00	0.03	0.00	0.04
0	0	0.33	1	t	1	Oxylobium ellipticum	Pelecorhychus rubidus	27	0.00	0.03	0.02	0.03

1	0	0.26	1	t	1	Scaptia alpina	Helipterum albicans	27	0.00	0.03	0.01	0.03
0	0	0.22	1	t	1	Tephritis sp. B	Leptorhyncho s squamatus	27	0.01	0.04	0.00	0.04
0	1	0.53	1	cv	1	Tephritis sp. B	Hypochoeris radicata	27	0.01	0.04	0.04	0.04
0	0	0.25	1	t	1	Stylidium graminifolium	Poecilohetaer us sp.	27	0.00	0.03	0.01	0.04
0	0	0.18	1	t	1	Comptosia sylvana	Aciphylla glacialis	27	0.00	0.04	0.00	0.04
1	0	0.33	1	t	1	Diostracus sp. 1	Prasophyllum alpinum	27	0.02	0.02	0.00	0.03
0	0	0.37	1	cv	1	Richea continentis	Leioproctus sp. 1 (female)	27	0.02	0.03	0.00	0.04
1	1	0.54	1	t	1	Richea continentis	Pelecorhychu s rubidus	27	0.02	0.03	0.02	0.03
1	0	0.42	1	t	1	Richea continentis	Lasioglossum (Parasphecod es) sp. 3	27	0.02	0.03	0.00	0.02
0	0	0.27	1	t	1	Lasioglossum (Parasphecode s) sp. 2 (male)	Achillea millefolium	27	0.01	0.03	0.01	0.03
1	0	0.49	1	cv	1	Senostoma sp. D	Epacris microphylla	27	0.03	0.03	0.00	0.03
1	1	0.61	1	t	1	Senostoma sp. D	Phebalium ovatifolium	27	0.03	0.03	0.02	0.04
0	0	0.47	1	t	1	Senostoma sp. D	Prasophyllum alpinum	27	0.03	0.03	0.00	0.03
1	1	0.60	1	t	1	Villa sp.	Pentachondr a pumila	27	0.01	0.03	0.04	0.03
0	0	0.25	1	cv	1	Villa sp.	Helipterum anthemoides	27	0.01	0.03	0.00	0.03
0	0	0.25	1	t	1	Leioproctus sp. 1 (female)	Prostanthera cuneata	27	0.00	0.04	0.01	0.04
0	0	0.22	1	t	1	Leioproctus sp. 1 (female)	Epacris petrophila	27	0.00	0.04	0.01	0.03
0	0	0.35	1	t	1	Leioproctus sp. 1 (female)	Wahlenbergi a ceracea	27	0.00	0.04	0.02	0.04
0	0	0.27	1	cv	1	Leioproctus sp. 1 (female)	Microseris lanceolata	27	0.00	0.04	0.02	0.04
0	0	0.43	1	t	1	Leioproctus sp. 1 (female)	Kunzea muelleri	27	0.00	0.04	0.03	0.04
0	0	0.49	1	t	1	Leioproctus sp. 1 (female)	Hypochoeris radicata	27	0.00	0.04	0.04	0.04
0	0	0.37	1	t	1	Rivellia sp. F	Wahlenbergi a ceracea	27	0.00	0.04	0.02	0.04
1	0	0.40	1	cv	1	Aciphylla simplicifolia	Sphenella marginata	27	0.01	0.01	0.00	0.01
1	1	0.74	1	t	1	Pentachondra pumila	Senostoma sp. B	27	0.04	0.03	0.03	0.04

1	1	0.70	1	t	1	Pentachondra pumila	Helina sp. B	27	0.04	0.03	0.02	0.03
1	1	0.85	1	t	1	Pentachondra pumila	Tabanus froggatti	27	0.04	0.03	0.04	0.03
0	0	0.40	1	cv	1	Prostanthera cuneata	Sapromyza sp.	27	0.01	0.04	0.02	0.04
0	0	0.27	1	t	1	Prostanthera cuneata	Exoneura sp. (female)	27	0.01	0.04	0.01	0.04
0	0	0.24	1	t	1	Prostanthera cuneata	Euryinae sp. 1	27	0.01	0.04	0.00	0.04
0	1	0.59	1	t	1	Prostanthera cuneata	Leioproctus sp. 1 (male)	27	0.01	0.04	0.04	0.04
0	1	0.56	1	cv	1	Prostanthera cuneata	Melangyna sp.	27	0.01	0.04	0.04	0.04
0	0	0.25	1	t	1	Prostanthera cuneata	Lasioglossum (Parasphecodes) sp. 1 (female)	27	0.01	0.04	0.00	0.04
0	0	0.28	1	t	1	Prostanthera cuneata	Prosena sp. C	27	0.01	0.04	0.01	0.04
0	0	0.35	1	t	1	Prostanthera cuneata	Empis sp. E	27	0.01	0.04	0.02	0.03
0	1	0.56	1	cv	1	Lasioglossum sp.	Euphrasia collina diversicolor	27	0.01	0.04	0.04	0.04
0	0	0.43	1	t	1	Lasioglossum sp.	Wahlenbergia ceracea	27	0.01	0.04	0.02	0.04
0	0	0.34	1	t	1	Lasioglossum sp.	Microseris lanceolata	27	0.01	0.04	0.02	0.04
0	1	0.57	1	t	1	Lasioglossum sp.	Hypochoeris radicata	27	0.01	0.04	0.04	0.04
0	0	0.32	1	cv	1	Sapromyza sp.	Prasophyllum alpinum	27	0.02	0.04	0.00	0.03
0	0	0.33	1	t	1	Leptorhynchos squamatus	Oreixenica orichora	27	0.00	0.04	0.02	0.04
0	0	0.41	1	t	1	Exoneura sp. (female)	Celmisia longifolia	27	0.01	0.04	0.03	0.04
0	0	0.46	1	t	1	Exoneura sp. (female)	Epilobium gunnianum	27	0.01	0.04	0.03	0.03
1	1	0.63	1	cv	1	Calliphora hilli	Phebalium ovatifolium	27	0.04	0.03	0.02	0.04
0	0	0.44	1	t	1	Helipterum albicans	Senostoma sp. B	27	0.01	0.03	0.03	0.04
0	1	0.53	1	t	1	Helipterum albicans	Melangyna sp.	27	0.01	0.03	0.04	0.04
0	0	0.26	1	t	1	Helipterum albicans	Prosena sp. C	27	0.01	0.03	0.01	0.04
0	0	0.38	1	cv	1	Helipterum albicans	Oreixenica orichora	27	0.01	0.03	0.02	0.04
0	0	0.33	1	t	1	Helipterum albicans	Empis sp. E	27	0.01	0.03	0.02	0.03

0	0	0.25	1	t	1	Helipterum albicans	Anthoboscinae	27	0.01	0.03	0.00	0.03
0	0	0.37	1	t	1	Euryinae sp. 1	Celmisia longifolia	27	0.00	0.04	0.03	0.04
0	0	0.22	1	cv	1	Euryinae sp. 1	Epacris petrophila	27	0.00	0.04	0.01	0.03
0	0	0.48	1	t	1	Euryinae sp. 1	Euphrasia collina diversicolor	27	0.00	0.04	0.04	0.04
0	0	0.35	1	t	1	Euryinae sp. 1	Wahlenbergia ceracea	27	0.00	0.04	0.02	0.04
0	0	0.30	1	t	1	Euryinae sp. 1	Phebalium ovatifolium	27	0.00	0.04	0.02	0.04
0	0	0.19	1	cv	1	Euryinae sp. 1	Aciphylla glacialis	27	0.00	0.04	0.00	0.04
0	0	0.19	1	t	1	Euryinae sp. 1	Leucopogon montanus	27	0.00	0.04	0.00	0.04
0	0	0.48	1	t	1	Euryinae sp. 1	Hypochoeris radicata	27	0.00	0.04	0.04	0.04
0	0	0.48	1	t	1	Euryinae sp. 3	Euphrasia collina diversicolor	27	0.00	0.04	0.04	0.04
0	0	0.20	1	cv	1	Euryinae sp. 3	Aciphylla glacialis	27	0.00	0.04	0.00	0.04
0	1	0.52	1	t	1	Euryinae sp. 2	Euphrasia collina diversicolor	27	0.01	0.04	0.04	0.04
1	0	0.25	1	t	1	Hippotion scofa	Epacris paludosa	27	0.00	0.02	0.00	0.03
1	0	0.29	1	t	1	Lasioglossum (Parasphecodes) sp. 2	Achillea millefolium	27	0.01	0.03	0.01	0.03
0	0	0.21	1	cv	1	Eristalis tenax	Pemelea ligustrina	27	0.01	0.04	0.00	0.04
0	0	0.28	1	t	1	Alophera sp.	Achillea millefolium	27	0.01	0.04	0.01	0.03
1	1	0.50	1	t	1	Epacris paludosa	Agrostis infusa	27	0.00	0.03	0.03	0.03
0	0	0.25	1	t	1	Unidentified Family Stratiomyidae	Epacris petrophila	27	0.00	0.03	0.01	0.03
1	1	0.69	1	cv	1	Leioproctus sp. 1 (male)	Wahlenbergia ceracea	27	0.04	0.04	0.02	0.04
1	1	0.60	1	t	1	Leioproctus sp. 1 (male)	Microseris lanceolata	27	0.04	0.04	0.02	0.04
0	0	0.40	1	t	1	Celmisia longifolia	Musca vetustissima (females)	27	0.03	0.04	0.01	0.04

0	0	0.37	1	t	1	Celmisia longifolia	Lasioglossum (Parasphecodes) sp. 1 (female)	27	0.03	0.04	0.00	0.04
0	0	0.41	1	cv	1	Celmisia longifolia	Prosena sp. C	27	0.03	0.04	0.01	0.04
0	1	0.55	1	t	1	Celmisia longifolia	Oreixenica orichora	27	0.03	0.04	0.02	0.04
0	0	0.36	1	t	1	Epacris petrophila	Helina sp. A	27	0.01	0.03	0.02	0.04
0	0	0.25	1	t	1	Epacris petrophila	Musca vetustissima (females)	27	0.01	0.03	0.01	0.04
0	1	0.60	1	cv	1	Epacris petrophila	Tabanus froggatti	27	0.01	0.03	0.04	0.03
0	0	0.36	1	t	1	Epacris petrophila	Pelecorhychus rubidus	27	0.01	0.03	0.02	0.03
0	0	0.33	1	t	1	Epacris petrophila	Empis sp. E	27	0.01	0.03	0.02	0.03
0	1	0.65	1	t	1	Euphrasia collina diversicolor	Oreixenica orichora	27	0.04	0.04	0.02	0.04
0	1	0.54	1	cv	1	Euphrasia collina diversicolor	Poecilohetaerus sp.	27	0.04	0.04	0.01	0.04
1	1	0.67	1	t	1	Wahlenbergia ceracea	Melangyna sp.	27	0.02	0.04	0.04	0.04
0	0	0.47	1	t	1	Wahlenbergia ceracea	Empis sp. E	27	0.02	0.04	0.02	0.03
0	0	0.47	1	t	1	Wahlenbergia ceracea	Empis sp. C	27	0.02	0.04	0.02	0.03
0	0	0.48	1	cv	1	Brachycome scapigera	Melangyna sp.	27	0.00	0.04	0.04	0.04
0	0	0.33	1	t	1	Brachycome scapigera	Oreixenica orichora	27	0.00	0.04	0.02	0.04
0	0	0.21	1	t	1	Musca vetustissima (females)	Aciphylla glacialis	27	0.01	0.04	0.00	0.04
1	1	0.61	1	t	1	Melangyna sp.	Phebalium ovatifolium	27	0.04	0.04	0.02	0.04
0	0	0.27	1	cv	1	Microseris lanceolata	Hylaeus semipersonatus	27	0.02	0.04	0.00	0.04
0	0	0.28	1	t	1	Phebalium ovatifolium	Leioproctus sp. 3 (female)	27	0.02	0.04	0.00	0.04
0	0	0.30	1	t	1	Phebalium ovatifolium	Empis sp. F	27	0.02	0.04	0.00	0.04
0	0	0.31	1	t	1	Pemelea ligustrina	Pelecorhychus rubidus	27	0.00	0.04	0.02	0.03
0	0	0.23	1	cv	1	Prasophyllum alpinum	Prosena sp. C	27	0.00	0.03	0.01	0.04

0	0	0.34	1	t	1	Prasophyllum alpinum	Oreixenica orichora	27	0.00	0.03	0.02	0.04
0	0	0.25	1	t	1	Prasophyllum alpinum	Poecilohetaerus sp.	27	0.00	0.03	0.01	0.04
0	0	0.26	1	t	1	Aciphylla glacialis	Hydrellia sp.	27	0.00	0.04	0.01	0.03
0	0	0.25	1	cv	1	Aciphylla glacialis	Poecilohetaerus sp.	27	0.00	0.04	0.01	0.04
0	1	0.64	1	t	1	Lasioglossum (Parasphecodes) sp. 1 (male)	Hypochoeris radicata	27	0.02	0.04	0.04	0.04
0	0	0.49	1	t	1	Lasioglossum (Parasphecodes) sp. 1	Hypochoeris radicata	27	0.00	0.04	0.04	0.04
0	0	0.26	1	t	1	Prosena sp. C	Achillea millefolium	27	0.01	0.04	0.01	0.03
0	0	0.24	1	cv	1	Prosena sp. C	Brachycome sp.	27	0.01	0.04	0.00	0.03
0	0	0.25	1	t	1	Leucopogon montanus	Poecilohetaerus sp.	27	0.00	0.04	0.01	0.04
0	1	0.65	1	t	1	Pelecorhychus rubidus	Hypochoeris radicata	27	0.02	0.03	0.04	0.04
1	1	0.74	0	t	1	Euphrasia collina diversicolor	Epilobium gunnianum	27	0.04	0.04	0.03	0.03
1	0	0.41	0	cv	1	Calliphora sp.	Kunzea muelleri	27	0.00	0.05	0.03	0.04
1	1	0.67	0	t	1	Musca vetustissima (males)	Megachile sp.	27	0.03	0.03	0.03	0.02
0	0	0.20	0	t	1	Syrphus sp.	Senostoma sp. A	27	0.00	0.05	0.01	0.04
0	1	0.56	0	t	1	Richea continentis	Prohardya carinata	27	0.02	0.03	0.02	0.04
1	1	0.55	0	cv	1	Unidentified no. 1	Megachile sp.	27	0.02	0.03	0.03	0.02
1	1	0.55	0	t	1	Lasioglossum sp. 4	Senostoma sp. D	27	0.01	0.04	0.03	0.03
0	0	0.37	0	t	1	Lasioglossum sp. 3	Comptosia sylvana	27	0.03	0.04	0.00	0.04
0	0	0.30	0	t	1	Calliphora sp. B	Diostracus sp. 1	27	0.00	0.04	0.02	0.02
0	0	0.37	0	cv	1	Scaptia alpina	Prohardya carinata	27	0.00	0.03	0.02	0.04
0	0	0.36	0	t	1	Leptorhynchos squamatus	Wahlenbergia ceracea	27	0.00	0.04	0.02	0.04
0	0	0.29	0	t	1	Unidentified no. 1	Prasophyllum alpinum	27	0.02	0.03	0.00	0.03
0	0	0.21	0	t	1	Tapeigaster nigricornis	Prasophyllum alpinum	27	0.00	0.03	0.00	0.03

0	0	0.23	0	cv	1	Olearia phlogopappa	Pemelea ligustrina	27	0.01	0.03	0.00	0.04
0	1	0.51	0	t	1	Lasioglossum sp. 5	Anthoboscinae	27	0.04	0.04	0.00	0.03
0	0	0.39	0	t	1	Helichrysum alpinum	Empis sp. E	27	0.02	0.04	0.02	0.03
0	0	0.33	0	t	1	Oreixenica orichora	Leucopogon montanus	27	0.02	0.04	0.00	0.04
0	0	0.18	0	cv	1	Limnophora sp.	Leioproctus sp. 1 (female)	27	0.00	0.04	0.00	0.04
0	0	0.29	0	t	1	Syrphus sp.	Diostracus sp. 1	27	0.00	0.05	0.02	0.02
0	0	0.35	0	t	1	Unidentified no. 2	Prohardya carinata	27	0.01	0.04	0.02	0.04
0	0	0.30	0	t	1	Euryglossa sp.	Hylaeus sp.	27	0.00	0.03	0.01	0.03
0	0	0.25	0	cv	1	Asperula gunnii	Oxylobium ellipticum	27	0.01	0.03	0.00	0.03
0	0	0.47	0	t	1	Lasioglossum sp. 3	Lasioglossum sp. 4	27	0.03	0.04	0.01	0.04
1	1	0.56	0	t	1	Musca vetustissima (males)	Euryglossa sp. 1	27	0.03	0.03	0.02	0.03
0	0	0.25	0	t	1	Villa sp.	Eristalis tenax	27	0.01	0.03	0.01	0.04
0	0	0.31	0	cv	1	Rutilia sp.	Prohardya carinata	27	0.00	0.05	0.02	0.04
0	0	0.19	0	t	1	Incurviseta sp. K	Exoneura sp. (male)	27	0.00	0.03	0.00	0.05
0	0	0.22	0	t	1	Epacris microphylla	Prasophyllum alpinum	27	0.00	0.03	0.00	0.03
1	1	0.82	0	t	1	Pentachondra pumila	Calliphora hilli	27	0.04	0.03	0.04	0.03
0	0	0.40	0	cv	1	Coenosia sp. B	Helina sp. B	27	0.01	0.03	0.02	0.03
0	0	0.37	0	t	1	Helichrysum scorpioides	Lasioglossum (Parasphecodes) sp. 1 (male)	27	0.01	0.04	0.02	0.04
0	1	0.52	0	t	1	Senostoma sp. D	Epacris petrophila	27	0.03	0.03	0.01	0.03
0	0	0.29	0	t	1	Neopaxia australasica	Prasophyllum alpinum	27	0.01	0.02	0.00	0.03
0	0	0.26	0	cv	1	Lasioglossum (Parasphecodes) sp. 2 (male)	Euryinae sp. 2	27	0.01	0.03	0.01	0.04
0	0	0.27	0	t	1	Euryglossa sp.	Senecio lautus	27	0.00	0.03	0.01	0.04
0	0	0.26	0	t	1	Villa sp.	Prosena sp. C	27	0.01	0.03	0.01	0.04
0	0	0.37	0	t	1	Empis ap. B	Anthoboscinae	27	0.02	0.04	0.00	0.03
0	0	0.41	0	cv	1	Epacris petrophila	Wahlenbergia ceracea	27	0.01	0.03	0.02	0.04

0	1	0.64	0	t	1	Incurviseta sp. D	Exoneura sp. (male)	27	0.06	0.04	0.00	0.05
0	0	0.30	0	t	1	Lasioglossum (Parasphecodes) sp. 1 (male)	Lasioglossum (Parasphecodes) sp. 1 (female)	27	0.02	0.04	0.00	0.04
0	0	0.26	0	t	1	Oxylobium ellipticum	Lasioglossum (Parasphecodes) sp. 2 (female)	27	0.00	0.03	0.01	0.03
0	0	0.25	0	cv	1	Prostanthera cuneata	Euryinae sp. 3	27	0.01	0.04	0.00	0.04
1	0	0.27	0	t	1	Olearia phlogopappa	Coenosia sp. A	27	0.01	0.03	0.00	0.02
0	0	0.44	0	t	1	Gentianella diemensis	Euryglossa sp. 2	27	0.03	0.04	0.01	0.04
0	1	0.54	0	t	1	Epilobium gunnianum	Empis sp. E	27	0.03	0.03	0.02	0.03
0	0	0.31	0	cv	1	Calliphora sp. B	Euryglossa sp. 1	27	0.00	0.04	0.02	0.03
0	0	0.26	0	t	1	Unidentified Family Dolichopodida	Empis sp. C	27	0.00	0.05	0.02	0.03
0	1	0.53	0	t	1	Unidentified no. 4	Leioproctus sp. 3 (male)	27	0.01	0.03	0.04	0.04
0	0	0.21	0	t	1	Eristalis tenax	Lasioglossum (Parasphecodes) sp. 1 (female)	27	0.01	0.04	0.00	0.04
0	0	0.28	0	cv	1	Empis sp. D	Lasioglossum (Parasphecodes) sp. 2 (male)	27	0.01	0.03	0.01	0.03
0	1	0.57	0	t	1	Empis ap. B	Senostoma sp. B	27	0.02	0.04	0.03	0.04
0	0	0.37	0	t	1	Senostoma sp. B	Hylaeus semipersonatus	27	0.03	0.04	0.00	0.04
0	0	0.45	0	t	1	Prasophyllum alpinum	Kunzea muelleri	27	0.00	0.03	0.03	0.04
0	0	0.41	0	cv	1	Lasioglossum sp.	Helina sp. B	27	0.01	0.04	0.02	0.03
0	0	0.37	0	t	1	Helichrysum alpinum	Hylaeus sp.	27	0.02	0.04	0.01	0.03
0	1	0.58	0	t	1	Tapeigaster nigricornis	Apis mellifera	27	0.00	0.03	0.04	0.04
0	0	0.29	0	t	1	Villa sp.	Alophera sp.	27	0.01	0.03	0.01	0.04
1	0	0.36	0	cv	1	Coenosia sp. A	Aciphylla simplicifolia	27	0.00	0.02	0.01	0.01
0	0	0.20	0	t	1	Chaetophthalamus dorsalis	Rivellia sp. F	27	0.00	0.04	0.00	0.04

0	0	0.25	0	t	1	Lasioglossum sp. 4	Brachycome scapigera	27	0.01	0.04	0.00	0.04
0	1	0.59	0	t	1	Apis mellifera	Achillea millefolium	27	0.04	0.04	0.01	0.03
0	0	0.23	0	cv	1	Calliphora fulvicoxa	Hippotion scofa	27	0.00	0.04	0.00	0.02
0	0	0.21	0	t	1	Hyphesma sp.	Brachycome sp.	27	0.01	0.05	0.00	0.03
0	1	0.54	0	t	1	Lasioglossum (Parasphecodes) sp. 2 (male)	Euphrasia collina diversicolor	27	0.01	0.03	0.04	0.04
0	1	0.51	0	t	1	Calliphora hilli	Musca vetustissima (females)	27	0.04	0.03	0.01	0.04
0	1	0.52	0	cv	1	Hippotion scofa	Agrostis infusa	27	0.00	0.02	0.03	0.03
0	0	0.25	0	t	1	Calliphora sp. A	Lasioglossum sp. 2	27	0.01	0.04	0.01	0.03
0	0	0.46	0	t	1	Calliphora sp. B	Senostoma sp. D	27	0.00	0.04	0.03	0.03
0	1	0.62	0	t	1	Apis mellifera	Senecio lautus	27	0.04	0.04	0.01	0.04
0	0	0.49	0	cv	1	Prasophyllum alpinum	Phytoliriomyza sp.	27	0.00	0.03	0.03	0.03
0	0	0.37	0	t	1	Chloromerus sp.	Epacris petrophila	27	0.02	0.03	0.01	0.03
0	1	0.55	0	t	1	Unidentified no. 4	Senostoma sp. D	27	0.01	0.03	0.03	0.03
0	0	0.39	0	t	1	Senecio gunnii	Empis ap. B	27	0.01	0.04	0.02	0.04
0	0	0.27	0	cv	1	Lasioglossum sp. 1	Senecio lautus	27	0.00	0.03	0.01	0.04
0	0	0.33	0	t	1	Musca vetustissima (females)	Lasioglossum (Parasphecodes) sp. 1 (male)	27	0.01	0.04	0.02	0.04
0	0	0.22	0	t	1	Stylidium graminifolium	Helipterum anthemoides	27	0.00	0.03	0.00	0.03
0	0	0.26	0	t	1	Unidentified no. 2	Alophera sp.	27	0.01	0.04	0.01	0.04
1	0	0.33	0	cv	1	Lasioglossum sp. 6	Empis sp. C	27	0.00	0.03	0.02	0.03
1	1	0.75	0	t	1	Lasioglossum sp. 3	Dilophus sp.	27	0.03	0.04	0.04	0.04
0	0	0.47	0	t	1	Senecio pectinatus	Kunzea muelleri	27	0.01	0.04	0.03	0.04
0	1	0.54	0	t	1	Unidentified no. 4	Agrostis infusa	27	0.01	0.03	0.03	0.03
0	0	0.41	0	cv	1	Lasioglossum sp. 3	Tapeigaster nigricornis	27	0.03	0.04	0.00	0.03

0	0	0.44	0	t	1	Leioproctus sp. 3 (male)	Lasioglossum (Parasphecodes) sp. 1 (female)	27	0.04	0.04	0.00	0.04
0	0	0.22	0	t	1	Calliphora sp.	Rivellia sp. F	27	0.01	0.04	0.00	0.04
0	0	0.37	0	t	1	Epacris paludosa	Oreixenica orichora	27	0.00	0.03	0.02	0.04
1	1	0.62	0	cv	1	Leioproctus sp. 3 (male)	Oreixenica orichora	27	0.04	0.04	0.02	0.04
0	0	0.24	0	t	1	Euryinae sp. 2	Senostoma sp. A	27	0.01	0.04	0.01	0.04
0	0	0.28	0	t	1	Unidentified no. 3	Hydrellia sp.	27	0.01	0.04	0.01	0.03
0	0	0.20	0	t	1	Oxylobium ellipticum	Leucopogon montanus	27	0.00	0.03	0.00	0.04
1	0	0.28	0	cv	1	Prostanthera cuneata	Lasioglossum (Parasphecodes) sp. 3	27	0.01	0.04	0.00	0.02
0	0	0.26	0	t	1	Lasioglossum sp.	Calliphora stygia	27	0.01	0.04	0.01	0.05
0	0	0.29	0	t	1	Tephritis sp. A	Unidentified Family Stratiomyidae	27	0.02	0.04	0.00	0.03
0	0	0.41	0	t	1	Helichrysum alpinum	Phebalium ovatifolium	27	0.02	0.04	0.02	0.04
0	0	0.16	0	cv	1	Rutilia sp.	Calliphora sp. B	27	0.00	0.05	0.00	0.04
0	0	0.17	0	t	1	Calliphora sp.	Pemelea ligustrina	27	0.00	0.05	0.00	0.04
0	0	0.29	0	t	1	Calliphora stygia	Empis sp. E	27	0.01	0.05	0.02	0.03
0	0	0.45	0	t	1	Senostoma sp. B	Poecilohetaerus sp.	27	0.03	0.04	0.01	0.04
0	0	0.26	0	cv	1	Alophera sp.	Helipterum anthemoides	27	0.01	0.04	0.00	0.03
0	0	0.31	0	t	1	Asperula gunnii	Lasioglossum (Parasphecodes) sp. 2 (female)	27	0.01	0.03	0.01	0.03
0	0	0.25	0	t	1	Unidentified no. 3	Hippotion scofa	27	0.01	0.04	0.00	0.02
0	0	0.21	0	t	1	Unidentified Family Therevidae	Pemelea ligustrina	27	0.00	0.03	0.00	0.04
0	1	0.74	0	cv	1	Unidentified no. 1	Craspedia sp.	27	0.02	0.03	0.05	0.04
0	0	0.23	0	t	1	Helichrysum scorpioides	Leioproctus sp. 3 (female)	27	0.01	0.04	0.00	0.04
0	1	0.61	0	t	1	Coenosia sp. B	Pentachondra pumila	27	0.01	0.03	0.04	0.03

0	1	0.78	0	t	1	Dilophus sp.	Leioproctus sp. 3 (male)	27	0.04	0.04	0.04	0.04
0	0	0.18	0	cv	1	Calliphora sp. B	Aciphylla glacialis	27	0.00	0.04	0.00	0.04
0	0	0.22	0	t	1	Tephritis sp. B	Brachycome stolonifera	27	0.01	0.04	0.00	0.04
0	0	0.21	0	t	1	Limnophora sp.	Musca vetustissima (females)	27	0.00	0.04	0.01	0.04
1	1	0.70	0	t	1	Incurviseta sp. D	Prosena sp. C	27	0.06	0.04	0.01	0.04
0	0	0.28	0	cv	1	Rutilia sp.	Helina sp. A	27	0.00	0.05	0.02	0.04
0	0	0.42	0	t	1	Musca vetustissima (males)	Lasioglossum sp. 6	27	0.03	0.03	0.00	0.03
0	0	0.41	0	t	1	Euryglossa sp.	Senostoma sp. B	27	0.00	0.03	0.03	0.04
1	1	0.89	0	t	1	Craspedia sp.	Pentachondra pumila	27	0.05	0.04	0.04	0.03
0	0	0.30	0	cv	1	Prasophyllum alpinum	Empis sp. C	27	0.00	0.03	0.02	0.03
0	0	0.33	0	t	1	Leptorhynchos squamatus	Chloromerus sp.	27	0.00	0.04	0.02	0.03
0	1	0.52	0	t	1	Incurviseta sp. K	Calliphora hilli	27	0.00	0.03	0.04	0.03
0	1	0.55	0	t	1	Lasioglossum sp. 6	Leioproctus sp. 1 (male)	27	0.00	0.03	0.04	0.04
1	1	0.50	0	cv	1	Euryglossa sp. 1	Oreixenica orichora	27	0.02	0.03	0.02	0.04
1	0	0.31	0	t	1	Epacris microphylla	Empis sp. C	27	0.00	0.03	0.02	0.03
0	1	0.51	0	t	1	Agrostis infusa	Achillea millefolium	27	0.03	0.03	0.01	0.03
0	0	0.24	0	t	1	Senecio gunnii	Unidentified Family Stratiomyidae	27	0.01	0.04	0.00	0.03
0	0	0.20	0	cv	1	Calliphora fulvicoxa	Empis sp. F	27	0.00	0.04	0.00	0.04
0	0	0.25	0	t	1	Lasioglossum sp. 4	Empis sp. F	27	0.01	0.04	0.00	0.04
0	1	0.80	0	t	1	Incurviseta sp. D	Euryglossa sp. 1	27	0.06	0.04	0.02	0.03
0	1	0.59	0	t	1	Lasioglossum sp. 1	Apis mellifera	27	0.00	0.03	0.04	0.04
0	0	0.32	0	cv	1	Helichrysum alpinum	Epacris petrophila	27	0.02	0.04	0.01	0.03
0	0	0.21	0	t	1	Lasioglossum sp. 6	Unidentified Family Dolichopodidae	27	0.00	0.03	0.00	0.05

0	0	0.36	0	t	1	Comptosia sylvana	Celmisia longifolia	27	0.00	0.04	0.03	0.04
0	0	0.21	0	t	1	Musca vetustissima (females)	Lasioglossum (Parasphecodes) sp. 1 (female)	27	0.01	0.04	0.00	0.04
0	0	0.21	0	cv	1	Chaetophthalamus dorsalis	Hyphesma sp.	27	0.00	0.04	0.01	0.05
0	0	0.34	0	t	1	Hylaeus sp.	Prostanthera cuneata	27	0.01	0.03	0.01	0.04
1	1	0.61	0	t	1	Melangyna sp.	Lasioglossum (Parasphecodes) sp. 1 (male)	27	0.04	0.04	0.02	0.04
0	0	0.33	0	t	1	Aciphylla simplicifolia	Brachycome sp.	27	0.01	0.01	0.00	0.03
0	0	0.38	0	cv	1	Lasioglossum (Parasphecodes) sp. 1 (male)	Hydrellia sp.	27	0.02	0.04	0.01	0.03
0	0	0.26	0	t	1	Unidentified no. 1	Leioproctus sp. 3 (female)	27	0.02	0.03	0.00	0.04
1	0	0.30	0	t	1	Brachycome scapiformis	Lasioglossum sp. 1	27	0.01	0.03	0.00	0.03
0	0	0.47	0	t	1	Lasioglossum sp. 3	Poecilohetaerus sp.	27	0.03	0.04	0.01	0.04
1	1	0.53	0	cv	1	Exoneura sp. (female)	Calliphora hilli	27	0.01	0.04	0.04	0.03
0	0	0.30	0	t	1	Neopaxia australasica	Tapeigaster nigricornis	27	0.01	0.02	0.00	0.03
0	0	0.23	0	t	1	Eristalis tenax	Unidentified Family Stratiomyidae	27	0.01	0.04	0.00	0.03
1	1	0.81	0	t	1	Lasioglossum sp. 5	Dilophus sp.	27	0.04	0.04	0.04	0.04
0	0	0.38	0	cv	1	Richea continentis	Brachycome scapigera	27	0.02	0.03	0.00	0.04
0	0	0.45	0	t	1	Empis sp. D	Celmisia longifolia	27	0.01	0.03	0.03	0.04
0	1	0.54	0	t	1	Aciphylla simplicifolia	Celmisia longifolia	27	0.01	0.01	0.03	0.04
0	0	0.23	0	t	1	Unidentified Family Dolichopodida	Lasioglossum sp.	27	0.00	0.05	0.01	0.04
1	1	0.74	0	cv	1	Megachile sp.	Phytoliriomyza sp.	27	0.03	0.02	0.03	0.03
0	0	0.23	0	t	1	Leioproctus sp. 3 (female)	Poecilohetaerus sp.	27	0.00	0.04	0.01	0.04
0	1	0.61	0	t	1	Sphenella marginata	Hypochoeris radicata	27	0.00	0.01	0.04	0.04

0	0	0.22	0	t	1	Limnophora sp.	Achillea millefolium	27	0.00	0.04	0.01	0.03
0	1	0.66	0	cv	1	Celmisia longifolia	Kunzea muelleri	27	0.03	0.04	0.03	0.04
0	0	0.22	0	t	1	Coenosia sp. B	Leioproctus sp. 3 (female)	27	0.01	0.03	0.00	0.04
0	0	0.22	0	t	1	Lasioglossum (Parasphecodes) sp. 1	Prosena sp. C	27	0.00	0.04	0.01	0.04
0	0	0.24	0	t	1	Calliphora sp. A	Eristalis tenax	27	0.01	0.04	0.01	0.04
0	0	0.19	0	cv	1	Comptosia sylvana	Helipterum anthemoides	27	0.00	0.04	0.00	0.03
0	0	0.22	0	t	1	Lasioglossum (Parasphecodes) sp. 2 (male)	Pemelea ligustrina	27	0.01	0.03	0.00	0.04
0	0	0.38	0	t	1	Poecilohetaerus sp.	Pelecorhychus rubidus	27	0.01	0.04	0.02	0.03
0	0	0.23	0	t	1	Leucopogon montanus	Achillea millefolium	27	0.00	0.04	0.01	0.03
0	0	0.26	0	cv	1	Scaptomyza australis	Euryinae sp. 1	27	0.01	0.02	0.00	0.04
0	0	0.34	0	t	1	Lasioglossum sp. 1	Helina sp. A	27	0.00	0.03	0.02	0.04
0	0	0.16	0	t	1	Comptosia sylvana	Leioproctus sp. 3 (female)	27	0.00	0.04	0.00	0.04
0	1	0.54	0	t	1	Villa sp.	Euphrasia collina diversicolor	27	0.01	0.03	0.04	0.04
0	0	0.24	0	cv	1	Empis sp. D	Leioproctus sp. 1 (female)	27	0.01	0.03	0.00	0.04
1	0	0.31	0	t	1	Syrphus sp.	Oreixenica orichora	27	0.00	0.05	0.02	0.04
0	0	0.21	0	t	1	Calliphora fulvicoxa	Euryglossa sp.	27	0.00	0.04	0.00	0.03
0	0	0.48	0	t	1	Aciphylla simplicifolia	Chloromerus sp.	27	0.01	0.01	0.02	0.03
0	0	0.49	0	cv	1	Calliphora fulvicoxa	Euphrasia collina diversicolor	27	0.00	0.04	0.04	0.04
0	0	0.26	0	t	1	Calliphora sp. A	Coenosia sp. A	27	0.01	0.04	0.00	0.02
0	0	0.30	0	t	1	Unidentified Family Therevidae	Microseris lanceolata	27	0.00	0.03	0.02	0.04
0	0	0.43	0	t	1	Aciphylla simplicifolia	Empis sp. C	27	0.01	0.01	0.02	0.03
1	1	0.73	0	cv	1	Lasioglossum sp. 3	Hypochoeris radicata	27	0.03	0.04	0.04	0.04

0	0	0.24	0	t	1	Alophera sp.	Hylaeus semipersonatus	27	0.01	0.04	0.00	0.04
0	0	0.27	0	t	1	Incurviseta sp. K	Lasioglossum (Parasphecodes) sp. 2 (female)	27	0.00	0.03	0.01	0.03
0	0	0.36	0	t	1	Chloromerus	Prosena sp. C	27	0.02	0.03	0.01	0.04
1	1	0.56	0	cv	1	Alophera sp.	Hypochoeris radicata	27	0.01	0.04	0.04	0.04
0	0	0.22	0	t	1	Euryglossa sp. 2	Epacris microphylla	27	0.01	0.04	0.00	0.03
0	0	0.30	0	t	1	Calliphora sp.	Euryglossa sp. 1	27	0.00	0.05	0.02	0.03
0	0	0.21	0	t	1	Epacris paludosa	Pemelea ligustrina	27	0.00	0.03	0.00	0.04
0	1	0.70	0	cv	1	Musca vetustissima (males)	Melangyna sp.	27	0.03	0.03	0.04	0.04
0	0	0.27	0	t	1	Empis sp. D	Prosena sp. C	27	0.01	0.03	0.01	0.04
0	0	0.20	0	t	1	Rutilia sp.	Euryinae sp. 2	27	0.00	0.05	0.01	0.04
1	1	0.68	0	t	1	Empis ap. B	Hypochoeris radicata	27	0.02	0.04	0.04	0.04
0	0	0.30	0	cv	1	Phebalium ovatifolium	Leucopogon montanus	27	0.02	0.04	0.00	0.04
0	1	0.74	0	t	1	Craspedia sp.	Aciphylla simplicifolia	27	0.05	0.04	0.01	0.01
1	0	0.42	0	t	1	Chloromerus sp.	Sphenella marginata	27	0.02	0.03	0.00	0.01
0	0	0.24	0	t	1	Lasioglossum sp. 4	Leioproctus sp. 1 (female)	27	0.01	0.04	0.00	0.04
1	0	0.25	0	cv	1	Calliphora sp. A	Lasioglossum (Parasphecodes) sp. 3	27	0.01	0.04	0.00	0.02
0	0	0.42	0	t	1	Calliphora sp. A	Celmisia longifolia	27	0.01	0.04	0.03	0.04
0	0	0.41	0	t	1	Hylaeus sp.	Phebalium ovatifolium	27	0.01	0.03	0.02	0.04
0	0	0.30	0	t	1	Asperula	Empis sp. D	27	0.01	0.03	0.01	0.03
0	0	0.20	0	cv	1	Calliphora sp. B	Senostoma sp. A	27	0.00	0.04	0.01	0.04
0	0	0.21	0	t	1	Lasioglossum sp. 6	Comptosia sylvana	27	0.00	0.03	0.00	0.04
0	0	0.20	0	t	1	Limnophora sp.	Unidentified Family Therevidae	27	0.00	0.04	0.00	0.03
0	0	0.21	0	t	1	Calliphora sp.	Prosena sp. C	27	0.00	0.04	0.01	0.04
0	0	0.37	0	cv	1	Lasioglossum sp. 6	Sapromyza sp.	27	0.00	0.03	0.02	0.04

0	0	0.47	0	t	1	Euryinae sp. 1	Melangyna sp.	27	0.00	0.04	0.04	0.04
1	0	0.36	0	t	1	Euryglossa sp.	Euryglossa sp. 1	27	0.00	0.03	0.02	0.03
0	0	0.17	0	t	1	Syrphus sp.	Leptorhynchos squamatus	27	0.00	0.05	0.00	0.04
0	0	0.30	0	cv	1	Hylaeus sp.	Euryinae sp. 2	27	0.01	0.03	0.01	0.04
0	0	0.32	0	t	1	Lasioglossum (Parasphecodes) sp. 2 (male)	Microseris lanceolata	27	0.01	0.03	0.02	0.04
0	0	0.18	0	t	1	Syrphus sp.	Euryglossa sp. 2	27	0.00	0.05	0.01	0.04
0	0	0.41	0	t	1	Leioproctus sp. 3 (female)	Kunzea muelleri	27	0.00	0.04	0.03	0.04
0	0	0.44	0	cv	1	Hylaeus sp.	Helina sp. B	27	0.01	0.03	0.02	0.03
1	0	0.29	0	t	1	Scaptomyza australis	Anthoboscinae	27	0.01	0.02	0.00	0.03
0	0	0.35	0	t	1	Unidentified no. 1	Alophera sp.	27	0.02	0.03	0.01	0.04
0	0	0.20	0	t	1	Prasophyllum alpinum	Hylaeus semipersonatus	27	0.00	0.03	0.00	0.04
0	0	0.21	0	cv	1	Olearia phlogopappa	Syrphus sp.	27	0.01	0.03	0.00	0.05
0	0	0.39	0	t	1	Baeckia gunniana	Phebalium ovatifolium	27	0.01	0.03	0.02	0.04
0	0	0.19	0	t	1	Leioproctus sp.2	Calliphora fulvicoxa	27	0.00	0.04	0.00	0.04
0	0	0.47	0	t	1	Musca vetustissima (males)	Prostanthera cuneata	27	0.03	0.03	0.01	0.04
0	0	0.27	0	cv	1	Unidentified Family	Limnophora sp.	27	0.02	0.03	0.00	0.04
0	0	0.26	0	t	1	Rutilia sp.	Unidentified no. 1	27	0.00	0.05	0.02	0.03
1	0	0.46	0	t	1	Epacris microphylla	Kunzea muelleri	27	0.00	0.03	0.03	0.04
0	0	0.41	0	t	1	Helipterum albicans	Wahlenbergia ceracea	27	0.01	0.03	0.02	0.04
0	0	0.35	0	cv	1	Unidentified Family	Hydrellia sp.	27	0.02	0.03	0.01	0.03
1	1	0.71	0	t	1	Helina sp. B	Tabanus froggatti	27	0.02	0.03	0.04	0.03
0	0	0.21	0	t	1	Unidentified Family Therevidae	Brachycome scapigera	27	0.00	0.03	0.00	0.04
0	0	0.42	0	t	1	Unidentified no. 3	Richea continentis	27	0.01	0.04	0.02	0.03

1	0	0.29	0	cv	1	Unidentified Family Therevidae	Sphenella marginata	27	0.00	0.03	0.00	0.01
0	1	0.69	0	t	1	Craspedia sp.	Senecio lautus	27	0.05	0.04	0.01	0.04
0	0	0.47	0	t	1	Leptorhynchos squamatus	Phytoliriomyza sp.	27	0.00	0.04	0.03	0.03
0	0	0.22	0	t	1	Lasioglossum sp. 6	Brachycome scapigera	27	0.00	0.03	0.00	0.04
1	0	0.38	0	cv	1	Lasioglossum (Parasphecodes) sp. 2 (female)	Lasioglossum (Parasphecodes) sp. 1 (male)	27	0.01	0.03	0.02	0.04
0	1	0.59	0	t	1	Apis mellifera	Lasioglossum (Parasphecodes) sp. 2 (male)	27	0.04	0.04	0.01	0.03
0	1	0.68	0	t	1	Incurviseta sp. D	Calliphora stygia	27	0.06	0.04	0.01	0.05
0	1	0.64	0	t	1	Unidentified no. 1	Dilophus sp.	27	0.02	0.03	0.04	0.04
0	0	0.47	0	cv	1	Calliphora sp.	Calliphora hilli	27	0.00	0.05	0.04	0.03
0	0	0.30	0	t	1	Syrphus sp.	Euryglossa sp. 1	27	0.00	0.05	0.02	0.03
0	0	0.25	0	t	1	Euryglossa sp. 2	Poecilohetaerus sp.	27	0.01	0.04	0.01	0.04
0	0	0.20	0	t	1	Pemelea ligustrina	Brachycome sp.	27	0.00	0.04	0.00	0.03
0	0	0.45	0	cv	1	Unidentified no. 2	Epilobium gunnianum	27	0.01	0.04	0.03	0.03
1	0	0.31	0	t	1	Brachycome scapiformis	Prosena sp. C	27	0.01	0.03	0.01	0.04
0	1	0.61	0	t	1	Unidentified no. 1	Hypochoeris radicata	27	0.02	0.03	0.04	0.04
0	0	0.27	0	t	1	Helichrysum scorpioides	Lasioglossum sp. 6	27	0.01	0.04	0.00	0.03
0	0	0.32	0	cv	1	Prostanthera cuneata	Hydrellia sp.	27	0.01	0.04	0.01	0.03
0	0	0.30	0	t	1	Syrphus sp.	Sapromyza sp.	27	0.00	0.05	0.02	0.04
1	1	0.66	0	t	1	Calliphora sp.	Incurviseta sp. D	27	0.00	0.05	0.06	0.04
0	0	0.41	0	t	1	Lasioglossum sp. 3	Hyphesma sp.	27	0.03	0.04	0.01	0.05
0	0	0.25	0	cv	1	Olearia phlogopappa	Unidentified Family Therevidae	27	0.01	0.03	0.00	0.03
0	1	0.65	0	t	1	Aciphylla simplicifolia	Calliphora hilli	27	0.01	0.01	0.04	0.03

0	0	0.33	0	t	1	Helina sp. A	Anthoboscinae	27	0.02	0.04	0.00	0.03
0	0	0.22	0	t	1	Unidentified no. 3	Stylidium graminifolium1	27	0.01	0.04	0.00	0.03
0	0	0.24	0	cv	1	Helichrysum scorpioides	Leptorhynchos squamatus	27	0.01	0.04	0.00	0.04
0	1	0.57	0	t	1	Apis mellifera	Exoneura sp. (female)	27	0.04	0.04	0.01	0.04
0	0	0.31	0	t	1	Empis sp. E	Anthoboscinae	27	0.02	0.03	0.00	0.03
0	0	0.28	0	t	1	Lasioglossum sp. 4	Euryinae sp. 2	27	0.01	0.04	0.01	0.04
0	1	0.75	0	cv	1	Neopaxia australasica	Craspedia sp.	27	0.01	0.02	0.05	0.04
0	1	0.58	0	t	1	Unidentified no. 4	Lasioglossum sp. 5	27	0.01	0.03	0.04	0.04
0	0	0.23	0	t	1	Lasioglossum sp. 2	Leucopogon montanus	27	0.01	0.03	0.00	0.04
0	0	0.42	0	t	1	Epacris paludosa	Celmisia longifolia	27	0.00	0.03	0.03	0.04
0	0	0.32	0	cv	1	Leioproctus sp.2	Pelecorhychus rubidus	27	0.00	0.04	0.02	0.03
0	0	0.25	0	t	1	Leptorhynchos squamatus	Hydrellia sp.	27	0.00	0.04	0.01	0.03
1	0	0.23	0	t	1	Epacris microphylla	Anthoboscinae	27	0.00	0.03	0.00	0.03
0	0	0.30	0	t	1	Hippotion	Hydrellia sp.	27	0.00	0.02	0.01	0.03
0	1	0.54	0	cv	1	Apis mellifera	Aciphylla glacialis	27	0.04	0.04	0.00	0.04
1	0	0.31	0	t	1	Brachycome scapiformis	Calliphora sp. A	27	0.01	0.03	0.01	0.04
0	0	0.50	0	t	1	Unidentified no. 3	Agrostis infusa	27	0.01	0.04	0.03	0.03
0	0	0.47	0	t	1	Unidentified no. 3	Gentianella diemensis	27	0.01	0.04	0.03	0.04
0	0	0.24	0	cv	1	Euryglossa sp.	Lasioglossum sp. 6	27	0.00	0.03	0.00	0.03
0	0	0.39	0	t	1	Olearia phlogopappa	Helina sp. B	27	0.01	0.03	0.02	0.03
0	0	0.32	0	t	1	Rutilia sp.	Empis ap. B	27	0.00	0.05	0.02	0.04
0	0	0.27	0	t	1	Hylaeus sp.	Brachycome stolonifera	27	0.01	0.03	0.00	0.04
0	0	0.24	0	cv	1	Coenosia sp. B	Empis sp. F	27	0.01	0.03	0.00	0.04
0	1	0.50	0	t	1	Euryglossa sp. 1	Prohardya carinata	27	0.02	0.03	0.02	0.04
0	1	0.70	0	t	1	Diostracus sp. 1	Pentachondra pumila	27	0.02	0.02	0.04	0.03
0	1	0.67	0	t	1	Neopaxia australasica	Tabanus froggatti	27	0.01	0.02	0.04	0.03

0	0	0.22	0	cv	1	Senecio pectinatus	Stylidium graminifolium1	27	0.01	0.04	0.00	0.03
0	0	0.45	0	t	1	Unidentified Family	Helina sp. B	27	0.02	0.03	0.02	0.03
0	0	0.38	0	t	1	Lasioglossum sp. 6	Oreixenica orichora	27	0.00	0.03	0.02	0.04
0	0	0.38	0	t	1	Richea continentis	Rivellia sp. F	27	0.02	0.03	0.00	0.04
1	0	0.43	0	cv	1	Euryglossa sp.	Hylaeus sp.	27	0.02	0.03	0.01	0.03
0	1	0.69	0	t	1	Senostoma sp. D	Celmisia longifolia	27	0.03	0.03	0.03	0.04
1	0	0.36	0	t	1	Hylaeus sp.	Sphenella marginata	27	0.01	0.03	0.00	0.01
0	0	0.30	0	t	1	Leptorhynchos squamatus	Phebalium ovatifolium	27	0.00	0.04	0.02	0.04
0	0	0.35	0	cv	1	Tephritis sp. A	Sphenella marginata	27	0.02	0.04	0.00	0.01
0	0	0.37	0	t	1	Lasioglossum (Parasphecodes) sp. 2 (male)	Sapromyza sp.	27	0.01	0.03	0.02	0.04
0	1	0.60	0	t	1	Neopaxia australasica	Melangyna sp.	27	0.01	0.02	0.04	0.04
0	0	0.24	0	t	1	Tephritis sp. B	Anthoboscinae	27	0.01	0.04	0.00	0.03
1	1	0.74	0	cv	1	Gentianella diemensis	Melangyna sp.	27	0.03	0.04	0.04	0.04
0	0	0.19	0	t	1	Unidentified Family Dolichopodida	Exoneura sp. (female)	27	0.00	0.05	0.01	0.04
0	0	0.27	0	t	1	Coenosia sp. B	Eristalis tenax	27	0.01	0.03	0.01	0.04
0	0	0.18	0	t	1	Leioproctus sp. 3 (female)	Brachycome stolonifera	27	0.00	0.04	0.00	0.04
0	1	0.60	1	cv	1	Phoebis sennae	Gossypium barbadense	28	0.05	0.05	0.01	0.05
1	1	0.57	1	t	1	Phoebis	Cassia sp.	28	0.05	0.05	0.01	0.04
1	1	0.78	1	t	1	Phoebis	Cassia picta	28	0.05	0.05	0.03	0.05
1	1	0.68	1	t	1	Phoebis sennae	Tournefortia rufo-sericea	28	0.05	0.05	0.02	0.04
1	1	0.57	1	cv	1	Phoebis sennae	Sida rhombifolia	28	0.05	0.05	0.01	0.04
0	1	0.59	1	t	1	Phoebis sennae	Justicia galapagana	28	0.05	0.05	0.01	0.04
0	1	0.57	1	t	1	Phoebis sennae	Catharanthus roseus	28	0.05	0.05	0.01	0.04
0	1	0.60	1	t	1	Phoebis	Bidens pilosa	28	0.05	0.05	0.01	0.05
0	1	0.68	1	cv	1	Phoebis	Diodia radula	28	0.05	0.05	0.02	0.04
1	1	0.96	1	t	1	Phoebis	Cordia lutea	28	0.05	0.05	0.07	0.05
0	1	0.57	1	t	1	Phoebis sennae	Bougainvillea spectabilis	28	0.05	0.05	0.01	0.04

1	1	0.57	1	t	1	Phoebis sennae	Gossypium sp.	28	0.05	0.05	0.01	0.04
1	1	0.58	1	cv	1	Phoebis sennae	Plumbago scandens	28	0.05	0.05	0.01	0.04
0	1	0.57	1	t	1	Phoebis	Opuntia sp.	28	0.05	0.05	0.01	0.04
0	0	0.37	1	t	1	Cardiospermu m	Xylocopa darwini	28	0.04	0.05	0.01	0.09
0	0	0.23	1	t	1	Damsel bug nymph	Justicia galapagana	28	0.01	0.03	0.01	0.04
1	1	0.76	1	cv	1	Beetles	Cordia lutea	28	0.01	0.05	0.07	0.05
0	0	0.08	1	t	1	Sesuvium portulacastru	Xylocopa darwini	28	0.01	0.08	0.01	0.09
0	0	0.14	1	t	1	Gossypium barbadense	Xylocopa darwini	28	0.01	0.05	0.01	0.09
1	0	0.17	1	t	1	Hawk moth (Sphing.)	Clerodendru m molle	28	0.01	0.03	0.01	0.06
0	0	0.11	1	cv	1	Ipomoea linearifolia	Xylocopa darwini	28	0.01	0.07	0.01	0.09
0	0	0.13	1	t	1	Commicarpus tuberosus	Xylocopa darwini	28	0.01	0.04	0.01	0.09
0	1	0.50	1	t	1	Commicarpus tuberosus	Leptotes parrhasioides	28	0.01	0.04	0.04	0.03
0	0	0.17	1	t	1	Mentzelia aspera	Xylocopa darwini	28	0.02	0.06	0.01	0.09
1	0	0.39	1	cv	1	Agrius cingulatus	Opuntia sp. 2	28	0.01	0.01	0.01	0.01
1	0	0.44	1	t	1	Agrius cingulatus	Carica papaya	28	0.01	0.01	0.01	0.01
1	0	0.39	1	t	1	Agrius cingulatus	Ipomoea sp.	28	0.01	0.01	0.01	0.01
1	0	0.39	1	t	1	Agrius cingulatus	Cacabus miersii	28	0.01	0.01	0.01	0.01
0	0	0.08	1	cv	1	Scalesia helleri	Xylocopa darwini	28	0.01	0.08	0.01	0.09
0	0	0.20	1	t	1	Galvezia leucantha pubesc.	Xylocopa darwini	28	0.02	0.06	0.01	0.09
0	0	0.16	1	t	1	Cassia sp.	Green winged hemerobiid	28	0.01	0.04	0.01	0.05
0	0	0.20	1	t	1	Cassia sp.	Oxacis sp.	28	0.01	0.04	0.01	0.05
0	0	0.41	1	cv	1	Cassia sp.	Atteva hysginiella	28	0.01	0.04	0.03	0.05
0	0	0.23	1	t	1	Cassia sp.	Euchrombius ocellus	28	0.01	0.04	0.02	0.05
0	0	0.29	1	t	1	Rhynchosia minima	Xylocopa darwini	28	0.03	0.05	0.01	0.09
0	0	0.11	1	t	1	Lantana pecucularis	Xylocopa darwini	28	0.01	0.07	0.01	0.09

1	0	0.29	1	cv	1	Avicennia germinans	Tapinoma melanocephalum	28	0.01	0.03	0.01	0.03
0	0	0.20	1	t	1	Avicennia germinans	Paratrechina longicornis	28	0.01	0.03	0.01	0.05
0	0	0.27	1	t	1	Cassia picta	Xylocopa darwini	28	0.03	0.05	0.01	0.09
0	0	0.32	1	t	1	Tournefortia rufo-sericea	Disclisioprocta stellata	28	0.02	0.04	0.01	0.04
0	0	0.25	1	cv	1	Tournefortia rufo-sericea	Wasmannia auropunctata	28	0.02	0.04	0.01	0.05
0	0	0.36	1	t	1	Tournefortia rufo-sericea	Utetheisa galapagensis	28	0.02	0.04	0.02	0.05
0	0	0.27	1	t	1	Tournefortia rufo-sericea	Frankliniella sp.	28	0.02	0.04	0.01	0.04
0	0	0.27	1	t	1	Tournefortia rufo-sericea	Mordellistena galapagensis	28	0.02	0.04	0.01	0.04
0	0	0.27	1	cv	1	Tournefortia rufo-sericea	Leafhopper (Cicadellidae)	28	0.02	0.04	0.01	0.04
1	1	0.76	1	t	1	Green winged hemerobiid	Cordia lutea	28	0.01	0.05	0.07	0.05
0	0	0.16	1	t	1	Green winged hemerobiid	Gossypium sp.	28	0.01	0.05	0.01	0.04
0	0	0.12	1	t	1	Acacia insulae-iacobi	Xylocopa darwini	28	0.01	0.06	0.01	0.09
0	0	0.16	1	cv	1	Acacia insulae-iacobi	Urbanus dorantes galap.	28	0.01	0.06	0.01	0.05
0	0	0.10	1	t	1	Clerodendrum molle	Xylocopa darwini	28	0.01	0.06	0.01	0.09
0	0	0.08	1	t	1	Abelmoschus manihot	Xylocopa darwini	28	0.01	0.08	0.01	0.09
0	0	0.08	1	t	1	Vallesia glabra	Xylocopa darwini	28	0.01	0.08	0.01	0.09
0	0	0.08	1	cv	1	Bastardia viscosa	Xylocopa darwini	28	0.01	0.08	0.01	0.09
1	0	0.26	1	t	1	Orthoperus sp.	Darwiniothamnus tenuifolius	28	0.01	0.03	0.01	0.03
0	0	0.14	1	t	1	Brassica campestris	Xylocopa darwini	28	0.02	0.06	0.01	0.09
0	0	0.25	1	t	1	Mormordica charantia	Wasmannia auropunctata	28	0.02	0.04	0.01	0.05
0	0	0.18	1	cv	1	Mormordica charantia	Xylocopa darwini	28	0.02	0.04	0.01	0.09
0	1	0.60	1	t	1	Mormordica charantia	Leptotes parrhasioides	28	0.02	0.04	0.04	0.03
1	1	0.80	1	t	1	Oxacis sp.	Cordia lutea	28	0.01	0.05	0.07	0.05
0	0	0.19	1	t	1	Oxacis sp.	Gossypium sp.	28	0.01	0.05	0.01	0.04

1	1	0.76	1	cv	1	Camponotus planus	Cordia lutea	28	0.01	0.05	0.07	0.05
0	0	0.15	1	t	1	Camponotus planus	Cryptocarpus pyriformis	28	0.01	0.05	0.01	0.06
0	0	0.22	1	t	1	Naucles sp.	Plumbago scandens	28	0.01	0.03	0.01	0.04
0	0	0.11	1	t	1	Passiflora foetida	Xylocopa darwini	28	0.01	0.07	0.01	0.09
1	0	0.41	1	cv	1	Moth (Pyralidae)	Tournefortia psilostachya	28	0.02	0.02	0.01	0.03
0	0	0.35	1	t	1	Moth (Pyralidae)	Scalesia beurii	28	0.02	0.02	0.01	0.03
0	0	0.18	1	t	1	Clerodendrum molle var.	Manduca rustica	28	0.01	0.05	0.01	0.05
0	0	0.14	1	t	1	Clerodendrum molle var.	Xylocopa darwini	28	0.01	0.05	0.01	0.09
0	0	0.29	1	cv	1	Clerodendrum molle var.	Psedoplusia includens	28	0.01	0.05	0.01	0.03
0	0	0.22	1	t	1	Disclisioprocta stellata	Cordia leucophlyctis	28	0.01	0.04	0.01	0.05
1	0	0.20	1	t	1	Opuntia echios	Ammophorus sp.	28	0.01	0.06	0.01	0.04
0	0	0.13	1	t	1	Opuntia echios	Xylocopa darwini	28	0.01	0.06	0.01	0.09
0	1	0.52	1	cv	1	Waltheria ovata	Atteva hysginiella	28	0.02	0.05	0.03	0.05
0	0	0.19	1	t	1	Waltheria ovata	Xylocopa darwini	28	0.02	0.05	0.01	0.09
0	0	0.13	1	t	1	Sida rhombifolia	Xylocopa darwini	28	0.01	0.04	0.01	0.09
0	0	0.27	1	t	1	Sida rhombifolia	Utetheisa galapagensis	28	0.01	0.04	0.02	0.05
0	0	0.50	1	cv	1	Sida rhombifolia	Leptotes parrhasioides	28	0.01	0.04	0.04	0.03
0	1	0.90	1	t	1	Opuntia echios gig.	Xylocopa darwini	28	0.14	0.14	0.01	0.09
0	0	0.20	1	t	1	Monomorium floricola	Opuntia helleri	28	0.01	0.05	0.01	0.02
1	1	0.76	1	t	1	Monomorium floricola	Cordia lutea	28	0.01	0.05	0.07	0.05
1	0	0.45	1	cv	1	Lycopersicon cheesmanii minor	Leaf bug (Hem., Miridae)	28	0.01	0.01	0.02	0.01
0	0	0.08	1	t	1	Abutilon depauperatum	Xylocopa darwini	28	0.01	0.08	0.01	0.09
1	1	0.51	1	t	1	Polygonum opelousanum	Toxomerus crockeri	28	0.01	0.03	0.03	0.03
1	0	0.43	1	t	1	Cordia sp.	Psedoplusia includens	28	0.01	0.01	0.01	0.03

1	0	0.39	1	cv	1	Prosopis juliflora	Tapinoma melanocephalum	28	0.02	0.05	0.01	0.03
0	0	0.23	1	t	1	Prosopis juliflora	Xylocopa darwini	28	0.02	0.05	0.01	0.09
1	0	0.32	1	t	1	Opuntia helleri	Catterpillar	28	0.01	0.02	0.01	0.03
0	0	0.21	1	t	1	Opuntia helleri	Manduca rustica	28	0.01	0.02	0.01	0.05
1	0	0.30	1	cv	1	Opuntia helleri	Cricket	28	0.01	0.02	0.01	0.02
1	0	0.30	1	t	1	Opuntia helleri	Tetramorium guineense	28	0.01	0.02	0.01	0.02
0	0	0.28	1	t	1	Justicia galapagana	Short-horned grasshopper nymph	28	0.01	0.04	0.01	0.03
0	0	0.19	1	t	1	Justicia galapagana	Wasmannia auropunctata	28	0.01	0.04	0.01	0.05
0	0	0.14	1	cv	1	Justicia galapagana	Xylocopa darwini	28	0.01	0.04	0.01	0.09
0	0	0.46	1	t	1	Justicia galapagana	Toxomerus crockeri	28	0.01	0.04	0.03	0.03
0	1	0.52	1	t	1	Justicia galapagana	Leptotes parrhasioides	28	0.01	0.04	0.04	0.03
0	0	0.18	1	t	1	Justicia galapagana	Urbanus dorantes galap.	28	0.01	0.04	0.01	0.05
1	1	0.51	1	cv	1	Jaegeria gracilis	Toxomerus crockeri	28	0.01	0.03	0.03	0.03
0	0	0.08	1	t	1	Euphorbia cyathophora	Xylocopa darwini	28	0.01	0.08	0.01	0.09
1	0	0.43	1	t	1	Heliothis cystiphora	Encelia hispida	28	0.01	0.01	0.01	0.01
1	0	0.31	1	t	1	Olcella sp.	Darwiniothamnus tenuifolius	28	0.01	0.02	0.01	0.03
1	0	0.28	1	cv	1	Catterpillar	Croton scouleri	28	0.01	0.03	0.01	0.04
1	1	0.76	1	t	1	Paratrechina vaga	Cordia lutea	28	0.01	0.05	0.07	0.05
0	0	0.17	1	t	1	Paratrechina vaga	Plumbago scandens	28	0.01	0.05	0.01	0.04
0	0	0.31	1	t	1	Cardiocondyla nuda	Plumbago scandens	28	0.02	0.03	0.01	0.04
1	1	0.76	1	cv	1	Manduca rustica	Cordia lutea	28	0.01	0.05	0.07	0.05
0	0	0.20	1	t	1	Setaria geniculta	Wasmannia auropunctata	28	0.01	0.04	0.01	0.05
0	0	0.08	1	t	1	Canna sp.	Xylocopa darwini	28	0.01	0.08	0.01	0.09
0	0	0.08	1	t	1	Scaveola plumieri	Xylocopa darwini	28	0.01	0.08	0.01	0.09

0	0	0.08	1	cv	1	Sonchus oleraceus	Xylocopa darwini	28	0.01	0.08	0.01	0.09
0	0	0.14	1	t	1	Bidens pilosa	Xylocopa darwini	28	0.01	0.05	0.01	0.09
0	0	0.08	1	t	1	Malvastrum coromandelia num	Xylocopa darwini	28	0.01	0.08	0.01	0.09
0	1	0.56	1	t	1	Diodia radula	Toxomerus crockeri	28	0.02	0.04	0.03	0.03
0	0	0.25	1	cv	1	Diodia radula	Urbanus dorantes galap.	28	0.02	0.04	0.01	0.05
0	0	0.08	1	t	1	Tetramerium nervosum	Xylocopa darwini	28	0.01	0.08	0.01	0.09
0	0	0.08	1	t	1	Sida acuta	Xylocopa darwini	28	0.01	0.08	0.01	0.09
1	0	0.32	1	t	1	Lepidanthrax tinctus	Darwiniotha mnus tenuifolius	28	0.02	0.03	0.01	0.03
0	0	0.30	1	cv	1	Lepidanthrax tinctus	Plumbago scandens	28	0.02	0.03	0.01	0.04
1	0	0.33	1	t	1	Lepidanthrax tinctus	Scalesia beurii	28	0.02	0.03	0.01	0.03
0	0	0.08	1	t	1	Persea maericana	Xylocopa darwini	28	0.01	0.08	0.01	0.09
0	0	0.08	1	t	1	Inga edulis	Xylocopa darwini	28	0.01	0.08	0.01	0.09
0	0	0.08	1	cv	1	Stachtarpheta cayanensis	Xylocopa darwini	28	0.01	0.08	0.01	0.09
1	1	0.76	1	t	1	Enyo lugubris delanoi	Cordia lutea	28	0.01	0.05	0.07	0.05
0	0	0.08	1	t	1	Acacia macracantha	Xylocopa darwini	28	0.01	0.08	0.01	0.09
0	0	0.16	1	t	1	Solanum americanum	Xylocopa darwini	28	0.01	0.04	0.01	0.09
0	1	0.50	1	cv	1	Solanum americanum	Toxomerus crockeri	28	0.01	0.04	0.03	0.03
1	1	0.91	1	t	1	Atteva hysginiella	Cordia lutea	28	0.03	0.05	0.07	0.05
1	0	0.39	1	t	1	Atteva hysginiella	Gossypium sp.	28	0.03	0.05	0.01	0.04
1	0	0.37	1	t	1	Atteva hysginiella	Cordia leucophlyctis	28	0.03	0.05	0.01	0.05
0	0	0.42	1	cv	1	Atteva hysginiella	Darwiniotha mnus tenuifolius	28	0.03	0.05	0.01	0.03
1	0	0.41	1	t	1	Atteva hysginiella	Croton scouleri	28	0.03	0.05	0.01	0.04
0	0	0.43	1	t	1	Atteva hysginiella	Scalesia beurii	28	0.03	0.05	0.01	0.03

0	1	0.73	1	t	1	Cordia lutea	Perepitragus fascipes	28	0.07	0.05	0.01	0.05
0	1	0.73	1	cv	1	Cordia lutea	Chrysopa sp.	28	0.07	0.05	0.01	0.05
0	1	0.73	1	t	1	Cordia lutea	Hypasclera sp.	28	0.07	0.05	0.01	0.05
1	1	0.75	1	t	1	Cordia lutea	Wasmannia auropunctata	28	0.07	0.05	0.01	0.05
0	1	0.73	1	t	1	Cordia lutea	Melipotis indomita	28	0.07	0.05	0.01	0.05
0	1	0.67	1	cv	1	Cordia lutea	Xylocopa darwini	28	0.07	0.05	0.01	0.09
1	1	0.81	1	t	1	Cordia lutea	Euchrombius ocellus	28	0.07	0.05	0.02	0.05
1	1	0.84	1	t	1	Cordia lutea	Utetheisa galapagensis	28	0.07	0.05	0.02	0.05
0	1	0.73	1	t	1	Cordia lutea	Metacanthus galapagensis	28	0.07	0.05	0.01	0.05
1	1	0.73	1	cv	1	Cordia lutea	Paratrechina longicornis	28	0.07	0.05	0.01	0.05
0	1	0.73	1	t	1	Cordia lutea	Amblycerus piurae	28	0.07	0.05	0.01	0.05
0	1	0.73	1	t	1	Cordia lutea	Hypasclera collenettei	28	0.07	0.05	0.01	0.05
0	1	0.74	1	t	1	Cordia lutea	Urbanus dorantes galap.	28	0.07	0.05	0.01	0.05
1	1	0.51	1	cv	1	Ageratum conyzoides	Toxomerus crockeri	28	0.01	0.03	0.03	0.03
0	0	0.22	1	t	1	Ornebius erraticus	Plumbago scandens	28	0.01	0.03	0.01	0.04
1	0	0.26	1	t	1	Darwinysius marginalis	Darwiniothamnus tenuifolius	28	0.01	0.03	0.01	0.03
0	0	0.42	1	t	1	Wasmannia auropunctata	Capsicum frutescens	28	0.01	0.05	0.03	0.03
0	0	0.18	1	cv	1	Wasmannia auropunctata	Plumbago scandens	28	0.01	0.05	0.01	0.04
0	0	0.26	1	t	1	Scalesia sp.	Xylocopa darwini	28	0.03	0.05	0.01	0.09
0	0	0.08	1	t	1	Caesalpinia pulcherrima	Xylocopa darwini	28	0.01	0.08	0.01	0.09
0	0	0.08	1	t	1	Hibiscus teliaceus	Xylocopa darwini	28	0.01	0.08	0.01	0.09
0	0	0.23	1	cv	1	Gossypium sp.	Euchrombius ocellus	28	0.01	0.04	0.02	0.05
1	0	0.26	1	t	1	Mythenteles sp.	Scalesia beurii	28	0.01	0.03	0.01	0.03
1	0	0.26	1	t	1	Moth (Gelechioidea)	Darwiniothamnus tenuifolius	28	0.01	0.03	0.01	0.03

0	0	0.08	1	t	1	Chicocca alba	Xylocopa darwini	28	0.01	0.08	0.01	0.09
0	0	0.08	1	cv	1	Parkinsonia aculeata	Xylocopa darwini	28	0.01	0.08	0.01	0.09
0	0	0.12	1	t	1	Cordia leucophlyctis	Xylocopa darwini	28	0.01	0.05	0.01	0.09
0	0	0.14	1	t	1	Castela galapageia	Xylocopa darwini	28	0.02	0.06	0.01	0.09
0	0	0.13	1	t	1	Vigna luteola	Xylocopa darwini	28	0.01	0.04	0.01	0.09
0	0	0.27	1	cv	1	Vigna luteola	Utetheisa galapagensis	28	0.01	0.04	0.02	0.05
0	0	0.50	1	t	1	Vigna luteola	Leptotes parrhasioides	28	0.01	0.04	0.04	0.03
0	0	0.08	1	t	1	Opuntia megasperma	Xylocopa darwini	28	0.01	0.08	0.01	0.09
0	0	0.08	1	t	1	Psychotria rufipes	Xylocopa darwini	28	0.01	0.08	0.01	0.09
0	0	0.08	1	cv	1	Xylocopa darwini	Clerodendru m molle glabr.	28	0.01	0.09	0.01	0.08
0	0	0.08	1	t	1	Xylocopa darwini	Galactea striata	28	0.01	0.09	0.01	0.08
0	0	0.08	1	t	1	Xylocopa darwini	Geoffroea spinosa	28	0.01	0.09	0.01	0.08
0	0	0.19	1	t	1	Xylocopa darwini	Sida spinosa	28	0.01	0.09	0.02	0.06
0	0	0.08	1	cv	1	Xylocopa darwini	Ipomoea pes-caprae	28	0.01	0.09	0.01	0.08
0	0	0.08	1	t	1	Xylocopa darwini	Psidium guajava	28	0.01	0.09	0.01	0.08
0	0	0.15	1	t	1	Xylocopa darwini	Tribulus cistoides	28	0.01	0.09	0.01	0.04
0	0	0.08	1	t	1	Xylocopa darwini	Macraea laricifolia	28	0.01	0.09	0.01	0.08
0	0	0.08	1	cv	1	Xylocopa darwini	Scalesia affinis	28	0.01	0.09	0.01	0.08
0	0	0.08	1	t	1	Xylocopa darwini	Cucurbita pepo	28	0.01	0.09	0.01	0.08
0	0	0.11	1	t	1	Xylocopa darwini	Cryptocarpus pyriformis	28	0.01	0.09	0.01	0.06
0	0	0.08	1	t	1	Xylocopa darwini	Cassia occidentalis	28	0.01	0.09	0.01	0.08
0	0	0.13	1	cv	1	Xylocopa darwini	Darwiniotha mnus tenuifolius	28	0.01	0.09	0.01	0.03
0	0	0.08	1	t	1	Xylocopa darwini	Cuphea racemosa	28	0.01	0.09	0.01	0.08
0	0	0.08	1	t	1	Xylocopa darwini	Piscidia carthagenesis	28	0.01	0.09	0.01	0.08

0	0	0.11	1	t	1	Xylocopa darwini	Galvezia leucantha	28	0.01	0.09	0.01	0.07
0	0	0.08	1	cv	1	Xylocopa darwini	Crotalaria incana	28	0.01	0.09	0.01	0.08
0	0	0.08	1	t	1	Xylocopa darwini	Mirabilis jalapa	28	0.01	0.09	0.01	0.08
0	0	0.08	1	t	1	Xylocopa darwini	Miconia robinsoniana	28	0.01	0.09	0.01	0.08
0	0	0.08	1	t	1	Xylocopa darwini	Bacopa monniera	28	0.01	0.09	0.01	0.08
0	0	0.08	1	cv	1	Xylocopa darwini	Nolana galapagensis	28	0.01	0.09	0.01	0.08
0	0	0.12	1	t	1	Xylocopa darwini	Plumbago scandens	28	0.01	0.09	0.01	0.04
0	0	0.08	1	t	1	Xylocopa darwini	Portulaca oleracea	28	0.01	0.09	0.01	0.08
0	0	0.19	1	t	1	Xylocopa darwini	Scalesia pedunculata	28	0.01	0.09	0.02	0.06
0	0	0.08	1	cv	1	Xylocopa darwini	Bursera graveolens	28	0.01	0.09	0.01	0.08
0	0	0.08	1	t	1	Xylocopa darwini	Physalis pubescens	28	0.01	0.09	0.01	0.08
0	0	0.08	1	t	1	Xylocopa darwini	Coffea arabica	28	0.01	0.09	0.01	0.08
0	0	0.08	1	t	1	Xylocopa darwini	Sida paniculata	28	0.01	0.09	0.01	0.08
0	0	0.17	1	cv	1	Xylocopa darwini	Lycopersicon cheesmanii	28	0.01	0.09	0.02	0.04
0	0	0.47	1	t	1	Toxomerus crockeri	Darwiniothamnus tenuifolius	28	0.03	0.03	0.01	0.03
1	0	0.26	1	t	1	Goniozus sp.	Darwiniothamnus tenuifolius	28	0.01	0.03	0.01	0.03
0	1	0.55	1	t	1	Tribulus cistoides	Leptotes parrhasioides	28	0.01	0.04	0.04	0.03
1	1	0.59	1	cv	1	Leptotes parrhasioides	Tournefortia psilostachya	28	0.04	0.03	0.01	0.03
1	1	0.81	1	t	1	Leptotes parrhasioides	Phyla sp.	28	0.04	0.03	0.04	0.03
0	1	0.59	1	t	1	Leptotes parrhasioides	Lycopersicon cheesmanii	28	0.04	0.03	0.02	0.04
0	0	0.19	1	t	1	Darwiniothamnus tenuifolius	Urbanus dorantes galap.	28	0.01	0.03	0.01	0.05
1	0	0.25	1	cv	1	Darwiniothamnus tenuifolius	Moth (Tortricidae)	28	0.01	0.03	0.01	0.03
0	0	0.18	1	t	1	Plumbago scandens	Urbanus dorantes galap.	28	0.01	0.04	0.01	0.05
0	0	0.26	1	t	1	Scalesia beurii	Rhinacloa sp.	28	0.01	0.03	0.01	0.03

0	0	0.24	1	t	1	Urbanus dorantes	Lycopersicon cheesmanii	28	0.01	0.05	0.02	0.04
0	0	0.17	0	cv	1	Mentzelia aspera	Canna sp.	28	0.02	0.06	0.01	0.08
0	0	0.27	0	t	1	Lepidanthrax tinctus	Amblycerus piurae	28	0.02	0.03	0.01	0.05
1	0	0.26	0	t	1	Polygonum opelousanum	Ornebius erraticus	28	0.01	0.03	0.01	0.03
0	0	0.17	0	t	1	Opuntia echios	Manduca rustica	28	0.01	0.06	0.01	0.05
0	0	0.21	0	cv	1	Opuntia helleri	Hypasclera collenettei	28	0.01	0.02	0.01	0.05
0	0	0.21	0	t	1	Brassica campestris	Oxacis sp.	28	0.02	0.06	0.01	0.05
0	0	0.40	0	t	1	Beetles	Capsicum frutescens	28	0.01	0.05	0.03	0.03
0	0	0.20	0	t	1	Disclisioprocta stellata	Enyo lugubris delanoi	28	0.01	0.04	0.01	0.05
0	0	0.27	0	cv	1	Brassica campestris	Lycopersicon cheesmanii minor	28	0.02	0.06	0.01	0.01
0	0	0.17	0	t	1	Wasmannia auropunctata	Cryptocarpus pyriformis	28	0.01	0.05	0.01	0.06
0	0	0.11	0	t	1	Cryptocarpus pyriformis	Mirabilis jalapa	28	0.01	0.06	0.01	0.08
0	0	0.50	0	t	1	Catharanthus roseus	Leptotes parrhasioides	28	0.01	0.04	0.04	0.03
0	0	0.14	0	cv	1	Clerodendrum molle var.	Mirabilis jalapa	28	0.01	0.05	0.01	0.08
0	0	0.21	0	t	1	Beetles	Cricket	28	0.01	0.05	0.01	0.02
0	0	0.19	0	t	1	Bidens pilosa	Urbanus dorantes galap.	28	0.01	0.05	0.01	0.05
0	0	0.13	0	t	1	Bougainvillea spectabilis	Clerodendrum molle glabr.	28	0.01	0.04	0.01	0.08
0	0	0.21	0	cv	1	Goniozus sp.	Galvezia leucantha	28	0.01	0.03	0.01	0.07
0	0	0.19	0	t	1	Damsel bug nymph	Chrysopa sp.	28	0.01	0.03	0.01	0.05
0	0	0.20	0	t	1	Euphorbia cyathophora	Lepidanthrax tinctus	28	0.01	0.08	0.02	0.03
0	0	0.14	0	t	1	Beetles	Chrysopa sp.	28	0.01	0.05	0.01	0.05
0	0	0.18	0	cv	1	Mormordica charantia	Cucurbita pepo	28	0.02	0.04	0.01	0.08
0	0	0.17	0	t	1	Hypasclera sp.	Plumbago scandens	28	0.01	0.05	0.01	0.04
0	1	0.85	0	t	1	Mormordica charantia	Cordia lutea	28	0.02	0.04	0.07	0.05
0	0	0.12	0	t	1	Sesuvium portulacastru	Catharanthus roseus	28	0.01	0.08	0.01	0.04

1	1	0.55	0	cv	1	Catterpillar	Capsicum frutescens	28	0.01	0.03	0.03	0.03
0	1	0.77	0	t	1	Cordia lutea	Croton scouleri	28	0.07	0.05	0.01	0.04
0	1	0.51	0	t	1	Galvezia leucantha pubesc.	Cassia picta	28	0.02	0.06	0.03	0.05
0	0	0.08	0	t	1	Psidium guajava	Portulaca oleracea	28	0.01	0.08	0.01	0.08
0	0	0.28	0	cv	1	Opuntia helleri	Goniozus sp.	28	0.01	0.02	0.01	0.03
0	0	0.08	0	t	1	Scaveola plumieri	Piscidia carthagenesis	28	0.01	0.08	0.01	0.08
1	0	0.47	0	t	1	Ammophorus sp.	Toxomerus crockeri	28	0.01	0.04	0.03	0.03
0	0	0.23	0	t	1	Ipomoea linearifolia	Lycopersicon cheesmanii	28	0.01	0.07	0.02	0.04
0	0	0.17	0	cv	1	Frankliniella sp.	Amblycerus piurae	28	0.01	0.04	0.01	0.05
0	0	0.22	0	t	1	Tapinoma melanocephal um	Hypasclera collenettei	28	0.01	0.03	0.01	0.05
0	0	0.19	0	t	1	Diodia radula	Piscidia carthagenesis	28	0.02	0.04	0.01	0.08
0	0	0.11	0	t	1	Beetles	Crotalaria incana	28	0.01	0.05	0.01	0.08
0	0	0.25	0	cv	1	Bidens pilosa	Rhinacloa sp.	28	0.01	0.05	0.01	0.03
0	0	0.15	0	t	1	Scaveola plumieri	Moth (Gelechioidea)	28	0.01	0.08	0.01	0.03
0	0	0.11	0	t	1	Hypasclera sp.	Cuphea racemosa	28	0.01	0.05	0.01	0.08
1	0	0.23	0	t	1	Ageratum conyzoides	Vigna luteola	28	0.01	0.03	0.01	0.04
0	0	0.11	0	cv	1	Sesuvium portulacastru	Metacanthus galapagensis	28	0.01	0.08	0.01	0.05
0	0	0.16	0	t	1	Stachtarpheta cayanensis	Tetramorium guineense	28	0.01	0.08	0.01	0.02
0	1	0.51	0	t	1	Rhynchosia minima	Olcella sp.	28	0.03	0.05	0.01	0.02
0	0	0.48	0	t	1	Ornebius erraticus	Toxomerus crockeri	28	0.01	0.03	0.03	0.03
0	0	0.24	0	cv	1	Darwinysius marginalis	Plumbago scandens	28	0.01	0.03	0.01	0.04
0	0	0.35	0	t	1	Rhynchosia minima	Amblycerus piurae	28	0.03	0.05	0.01	0.05
0	0	0.27	0	t	1	Lycopersicon cheesmanii minor	Plumbago scandens	28	0.01	0.01	0.01	0.04

0	1	0.53	0	t	1	Cardiocondyla nuda	Scalesia sp.	28	0.02	0.03	0.03	0.05
0	0	0.32	0	cv	1	Lepidanthrax tinctus	Ammophorus sp.	28	0.02	0.03	0.01	0.04
0	0	0.14	0	t	1	Croton scouleri	Bursera graveolens	28	0.01	0.04	0.01	0.08
0	0	0.34	0	t	1	Scalesia sp.	Cordia leucophlyctis	28	0.03	0.05	0.01	0.05
0	0	0.21	0	t	1	Disclisioprocta stellata	Monomorium floricola	28	0.01	0.04	0.01	0.05
1	0	0.34	0	cv	1	Agrius cingulatus	Darwinysius marginalis	28	0.01	0.01	0.01	0.03
0	0	0.11	0	t	1	Ipomoea linearifolia	Canna sp.	28	0.01	0.07	0.01	0.08
0	0	0.08	0	t	1	Stachtarpheta cayanensis	Portulaca oleracea	28	0.01	0.08	0.01	0.08
0	0	0.08	0	t	1	Canna sp.	Scaveola plumieri	28	0.01	0.08	0.01	0.08
0	0	0.15	0	cv	1	Hibiscus teliaceus	Moth (Tortricidae)	28	0.01	0.08	0.01	0.03
0	0	0.32	0	t	1	Ipomoea sp.	Castela galapageia	28	0.01	0.01	0.02	0.06
0	0	0.13	0	t	1	Cassia sp.	Malvastrum coromandelia num	28	0.01	0.04	0.01	0.08
0	0	0.19	0	t	1	Tournefortia rufo-sericea	Xylocopa darwini	28	0.02	0.04	0.01	0.09
0	0	0.20	0	cv	1	Euphorbia cyathophora	Scalesia pedunculata	28	0.01	0.08	0.02	0.06
0	1	0.97	0	t	1	Avicennia germinans	Opuntia echios gig.	28	0.01	0.03	0.14	0.14
0	0	0.15	0	t	1	Canna sp.	Darwinysius marginalis	28	0.01	0.08	0.01	0.03
1	0	0.28	0	t	1	Polygonum opelousanum	Darwinysius marginalis	28	0.01	0.03	0.01	0.03
0	0	0.13	0	cv	1	Opuntia echios	Portulaca oleracea	28	0.01	0.06	0.01	0.08
0	0	0.21	0	t	1	Orthoperus sp.	Urbanus dorantes galap.	28	0.01	0.03	0.01	0.05
0	0	0.24	0	t	1	Beetles	Psedoplusia includens	28	0.01	0.05	0.01	0.03
0	0	0.25	0	t	1	Disclisioprocta stellata	Darwiniotha mnus tenuifolius	28	0.01	0.04	0.01	0.03
0	0	0.08	0	cv	1	Cuphea racemosa	Physalis pubescens	28	0.01	0.08	0.01	0.08
0	0	0.16	0	t	1	Catharanthus roseus	Amblycerus piurae	28	0.01	0.04	0.01	0.05
0	0	0.16	0	t	1	Disclisioprocta stellata	Parkinsonia aculeata	28	0.01	0.04	0.01	0.08

0	1	0.67	0	t	1	Cordia lutea	Portulaca oleracea	28	0.07	0.05	0.01	0.08
0	0	0.23	0	cv	1	Mordellistena galapagensis	Moth (Tortricidae)	28	0.01	0.04	0.01	0.03
0	0	0.20	0	t	1	Galvezia leucantha pubesc.	Scalesia affinis	28	0.02	0.06	0.01	0.08
0	0	0.08	0	t	1	Malvastrum coromandelia num	Coffea arabica	28	0.01	0.08	0.01	0.08
0	0	0.08	0	t	1	Vallesia glabra	Psychotria rufipes	28	0.01	0.08	0.01	0.08
0	0	0.11	0	cv	1	Camponotus planus	Chicocca alba	28	0.01	0.05	0.01	0.08
0	0	0.46	0	t	1	Phoebis sennae	Malvastrum coromandelia num	28	0.05	0.05	0.01	0.08
0	1	0.61	0	t	1	Moth (Pyralidae)	Capsicum frutescens	28	0.02	0.02	0.03	0.03
0	0	0.08	0	t	1	Sida acuta	Sida paniculata	28	0.01	0.08	0.01	0.08
0	0	0.31	0	cv	1	Waltheria ovata	Opuntia helleri	28	0.02	0.05	0.01	0.02
0	0	0.15	0	t	1	Perepitragus fascipes	Cryptocarpus pyriformis	28	0.01	0.05	0.01	0.06
0	0	0.15	0	t	1	Euphorbia cyathophora	Moth (Tortricidae)	28	0.01	0.08	0.01	0.03
0	0	0.29	0	t	1	Cordia sp.	Enyo lugubris delanoi	28	0.01	0.01	0.01	0.05
0	0	0.18	0	cv	1	Monomorium floricola	Castela galapageia	28	0.01	0.05	0.02	0.06
0	0	0.23	0	t	1	Vigna luteola	Rhinacloa sp.	28	0.01	0.04	0.01	0.03
0	0	0.19	0	t	1	Lycopersicon cheesmanii minor	Chicocca alba	28	0.01	0.01	0.01	0.08
0	0	0.17	0	t	1	Commicarpus tuberosus	Paratrechina vaga	28	0.01	0.04	0.01	0.05
0	0	0.41	0	cv	1	Tournefortia rufo-sericea	Encelia hispida	28	0.02	0.04	0.01	0.01
0	0	0.40	0	t	1	Enyo lugubris delanoi	Toxomerus crockeri	28	0.01	0.05	0.03	0.03
1	0	0.37	0	t	1	Orthoperus sp.	Moth (Pyralidae)	28	0.01	0.03	0.02	0.02
0	0	0.40	0	t	1	Rhynchosia minima	Croton scouleri	28	0.03	0.05	0.01	0.04
0	0	0.14	0	cv	1	Ammophorus sp.	Opuntia megasperma	28	0.01	0.04	0.01	0.08
1	0	0.23	0	t	1	Ammophorus sp.	Castela galapageia	28	0.01	0.04	0.02	0.06
0	0	0.24	0	t	1	Jaegeria gracilis	Plumbago scandens	28	0.01	0.03	0.01	0.04

0	0	0.11	0	t	1	Scalesia helleri	Acacia insulae-iacobi	28	0.01	0.08	0.01	0.06
0	0	0.29	0	cv	1	Encelia hispida	Chrysopa sp.	28	0.01	0.01	0.01	0.05
0	0	0.23	0	t	1	Lantana pedecularis	Short-horned grasshopper nymph	28	0.01	0.07	0.01	0.03
0	0	0.37	0	t	1	Leptotes parrhasioides	Bursera graveolens	28	0.04	0.03	0.01	0.08
0	0	0.12	0	t	1	Wasmannia auropunctata	Chicocca alba	28	0.01	0.05	0.01	0.08
0	0	0.11	0	cv	1	Lantana pedecularis	Abutilon depauperatu m	28	0.01	0.07	0.01	0.08
0	1	0.54	0	t	1	Phoebis sennae	Manduca rustica	28	0.05	0.05	0.01	0.05
0	0	0.18	0	t	1	Portulaca oleracea	Cacabus miersii	28	0.01	0.08	0.01	0.01
0	0	0.15	0	t	1	Sida acuta	Moth (Gelechioidea)	28	0.01	0.08	0.01	0.03
0	0	0.13	0	cv	1	Sida rhombifolia	Cuphea racemosa	28	0.01	0.04	0.01	0.08
0	0	0.17	0	t	1	Opuntia echios	Cordia leucophlyctis	28	0.01	0.06	0.01	0.05
0	0	0.11	0	t	1	Green winged hemerobiid	Opuntia megasperma	28	0.01	0.05	0.01	0.08
0	0	0.29	0	t	1	Carica papaya	Perepitragus fascipes	28	0.01	0.01	0.01	0.05
0	0	0.20	0	cv	1	Opuntia sp. 2	Cassia occidentalis	28	0.01	0.01	0.01	0.08
0	0	0.26	0	t	1	Mormordica charantia	Plumbago scandens	28	0.02	0.04	0.01	0.04
0	0	0.20	0	t	1	Brassica campestris	Plumbago scandens	28	0.02	0.06	0.01	0.04
0	0	0.11	0	t	1	Miconia robinsoniana	Urbanus dorantes galap.	28	0.01	0.08	0.01	0.05
0	0	0.24	0	cv	1	Manduca rustica	Psedoplusia includens	28	0.01	0.05	0.01	0.03
0	0	0.39	0	t	1	Phyla sp.	Portulaca oleracea	28	0.04	0.03	0.01	0.08
0	0	0.25	0	t	1	Acacia insulae-iacobi	Ipomoea sp.	28	0.01	0.06	0.01	0.01
0	0	0.22	0	t	1	Bidens pilosa	Ammophorus sp.	28	0.01	0.05	0.01	0.04
0	0	0.08	0	cv	1	Inga edulis	Bursera graveolens	28	0.01	0.08	0.01	0.08
0	0	0.20	0	t	1	Ipomoea pes-caprae	Scalesia pedunculata	28	0.01	0.08	0.02	0.06

0	0	0.26	0	t	1	Ammophorus sp.	Cricket	28	0.01	0.04	0.01	0.02
0	0	0.19	0	t	1	Bougainvillea spectabilis	Vigna luteola	28	0.01	0.04	0.01	0.04
0	0	0.40	0	cv	1	Lycopersicon cheesmanii minor	Scalesia pedunculata	28	0.01	0.01	0.02	0.06
0	0	0.27	0	t	1	Catharanthus roseus	Ipomoea sp.	28	0.01	0.04	0.01	0.01
0	0	0.44	0	t	1	Rhynchosia minima	Goniozus sp.	28	0.03	0.05	0.01	0.03
0	0	0.13	0	t	1	Commicarpus tuberosus	Bastardia viscosa	28	0.01	0.04	0.01	0.08
0	0	0.21	0	cv	1	Mentzelia aspera	Paratrechina vaga	28	0.02	0.06	0.01	0.05
0	0	0.39	0	t	1	Olcella sp.	Diodia radula	28	0.01	0.02	0.02	0.04
0	0	0.08	0	t	1	Parkinsonia aculeata	Scalesia affinis	28	0.01	0.08	0.01	0.08
0	0	0.18	0	t	1	Clerodendrum molle var.	Perepitragus fascipes	28	0.01	0.05	0.01	0.05
1	0	0.39	0	cv	1	Opuntia sp. 2	Heliothis cystiphora	28	0.01	0.01	0.01	0.01
0	0	0.17	0	t	1	Camponotus planus	Frankliniella sp.	28	0.01	0.05	0.01	0.04
0	0	0.30	0	t	1	Heliothis cystiphora	Setaria geniculta	28	0.01	0.01	0.01	0.04
0	1	0.53	0	t	1	Justicia galapagana	Phyla sp.	28	0.01	0.04	0.04	0.03
0	0	0.08	0	cv	1	Sesuvium portulacastru	Persea maericana	28	0.01	0.08	0.01	0.08
0	0	0.23	0	t	1	Cordia sp.	Clerodendrum molle glabr.	28	0.01	0.01	0.01	0.08
0	0	0.15	0	t	1	Damsel bug nymph	Bastardia viscosa	28	0.01	0.03	0.01	0.08
0	0	0.19	0	t	1	Lycopersicon cheesmanii minor	Nolana galapagensis	28	0.01	0.01	0.01	0.08
0	0	0.13	0	cv	1	Oxacis sp.	Clerodendrum molle glabr.	28	0.01	0.05	0.01	0.08
0	0	0.24	0	t	1	Justicia galapagana	Rhinacloa sp.	28	0.01	0.04	0.01	0.03
0	0	0.18	0	t	1	Oxacis sp.	Urbanus dorantes galap.	28	0.01	0.05	0.01	0.05
0	0	0.16	0	t	1	Darwinysius marginalis	Crotalaria incana	28	0.01	0.03	0.01	0.08
0	0	0.15	0	cv	1	Naucles sp.	Nolana galapagensis	28	0.01	0.03	0.01	0.08

0	0	0.15	0	t	1	Acacia insulae-iacobi	Passiflora foetida	28	0.01	0.06	0.01	0.07
1	0	0.26	0	t	1	Damsel bug nymph	Polygonum opelousanum	28	0.01	0.03	0.01	0.03
0	0	0.15	0	t	1	Damsel bug nymph	Scaveola plumieri	28	0.01	0.03	0.01	0.08
0	0	0.20	0	cv	1	Heliothis cystiphora	Inga edulis	28	0.01	0.01	0.01	0.08
0	0	0.08	0	t	1	Sonchus oleraceus	Chicocca alba	28	0.01	0.08	0.01	0.08
0	0	0.17	0	t	1	Malvastrum coromandelia num	Tournefortia psilostachya	28	0.01	0.08	0.01	0.03
0	0	0.31	0	t	1	Prosopis juliflora	Bougainvillea spectabilis	28	0.02	0.05	0.01	0.04
0	0	0.08	0	cv	1	Canna sp.	Miconia robinsoniana	28	0.01	0.08	0.01	0.08
0	0	0.11	0	t	1	Cucurbita pepo	Urbanus dorantes galap.	28	0.01	0.08	0.01	0.05
0	0	0.43	0	t	1	Galvezia leucantha pubesc.	Cordia sp.	28	0.02	0.06	0.01	0.01
0	0	0.16	0	t	1	Tetramerium nervosum	Cricket	28	0.01	0.08	0.01	0.02
0	1	0.57	0	cv	1	Disclisioprocta stellata	Phyla sp.	28	0.01	0.04	0.04	0.03
0	0	0.18	0	t	1	Tetramerium nervosum	Cacabus miersii	28	0.01	0.08	0.01	0.01
0	0	0.18	0	t	1	Ipomoea linearifolia	Castela galapageia	28	0.01	0.07	0.02	0.06
0	0	0.16	0	t	1	Mythenteles sp.	Opuntia megasperma	28	0.01	0.03	0.01	0.08
0	0	0.22	0	cv	1	Ipomoea linearifolia	Lycopersicon cheesmanii minor	28	0.01	0.07	0.01	0.01
0	0	0.11	0	t	1	Enyo lugubris delanoi	Hibiscus teliaceus	28	0.01	0.05	0.01	0.08
0	0	0.44	0	t	1	Clerodendrum molle	Phyla sp.	28	0.01	0.06	0.04	0.03
0	0	0.11	0	t	1	Passiflora foetida	Physalis pubescens	28	0.01	0.07	0.01	0.08
0	0	0.14	0	cv	1	Clerodendrum molle var.	Acacia macracantha	28	0.01	0.05	0.01	0.08
0	1	0.96	0	t	1	Opuntia echios gig.	Olcella sp.	28	0.14	0.14	0.01	0.02
0	0	0.26	0	t	1	Psedoplusia includens	Cryptocarpus pyriformis	28	0.01	0.03	0.01	0.06
0	0	0.19	0	t	1	Lycopersicon cheesmanii minor	Sonchus oleraceus	28	0.01	0.01	0.01	0.08

0	0	0.24	0	cv	1	Goniozus sp.	Croton scouleri	28	0.01	0.03	0.01	0.04
0	0	0.11	0	t	1	Abutilon depauperatum	Cordia leucophlyctis	28	0.01	0.08	0.01	0.05
0	0	0.20	0	t	1	Monomorium floricola	Mythenteles sp.	28	0.01	0.05	0.01	0.03
0	0	0.17	0	t	1	Abelmoschus manihot	Tapinoma melanocephalum	28	0.01	0.08	0.01	0.03
0	0	0.16	0	cv	1	Opuntia helleri	Sida acuta	28	0.01	0.02	0.01	0.08
0	0	0.11	0	t	1	Manduca rustica	Persea maericana	28	0.01	0.05	0.01	0.08
0	0	0.26	0	t	1	Ammophorus sp.	Tetramorium guineense	28	0.01	0.04	0.01	0.02
0	0	0.33	0	t	1	Scalesia pedunculata	Moth (Tortricidae)	28	0.02	0.06	0.01	0.03
1	0	0.32	0	cv	1	Hawk moth (Sphing.)	Sida spinosa	28	0.01	0.03	0.02	0.06
0	0	0.20	0	t	1	Opuntia sp. 2	Euphorbia cyathophora	28	0.01	0.01	0.01	0.08
0	0	0.15	0	t	1	Persea maericana	Tribulus cistoides	28	0.01	0.08	0.01	0.04
0	0	0.08	0	t	1	Bastardia viscosa	Geoffroea spinosa	28	0.01	0.08	0.01	0.08
0	0	0.13	0	cv	1	Oxacis sp.	Acacia macracantha	28	0.01	0.05	0.01	0.08
0	0	0.14	0	t	1	Scalesia helleri	Castela galapageia	28	0.01	0.08	0.02	0.06
0	0	0.11	0	t	1	Perepitragus fascipes	Geoffroea spinosa	28	0.01	0.05	0.01	0.08
0	0	0.23	0	t	1	Brassica campestris	Scalesia beurii	28	0.02	0.06	0.01	0.03
0	0	0.14	0	cv	1	Galactea striata	Darwiniothamnus tenuifolius	28	0.01	0.08	0.01	0.03
0	0	0.24	0	t	1	Waltheria ovata	Manduca rustica	28	0.02	0.05	0.01	0.05
0	0	0.42	0	t	1	Acacia insulae-iacobi	Toxomerus crockeri	28	0.01	0.06	0.03	0.03
0	0	0.36	0	t	1	Prosopis juliflora	Mythenteles sp.	28	0.02	0.05	0.01	0.03
0	0	0.18	0	cv	1	Mormordica charantia	Macraea laricifolia	28	0.02	0.04	0.01	0.08
0	0	0.20	0	t	1	Heliothis cystiphora	Clerodendrum molle glabr.	28	0.01	0.01	0.01	0.08
0	1	0.85	0	t	1	Phoebis sennae	Cardiospermum galapageium	28	0.05	0.05	0.04	0.05
0	0	0.13	0	t	1	Scalesia helleri	Croton scouleri	28	0.01	0.08	0.01	0.04

0	0	0.23	0	cv	1	Cordia sp.	Scaveola plumieri	28	0.01	0.01	0.01	0.08
0	0	0.11	0	t	1	Camponotus planus	Canna sp.	28	0.01	0.05	0.01	0.08
0	0	0.22	0	t	1	Solanum americanum	Gossypium sp.	28	0.01	0.04	0.01	0.04
0	0	0.27	0	t	1	Avicennia germinans	Opuntia helleri	28	0.01	0.03	0.01	0.02
0	0	0.23	0	cv	1	Encelia hispida	Acacia macracantha	28	0.01	0.01	0.01	0.08
0	0	0.11	0	t	1	Hypasclera sp.	Geoffroea spinosa	28	0.01	0.05	0.01	0.08
0	0	0.26	0	t	1	Damsel bug nymph	Moth (Gelechioidea)	28	0.01	0.03	0.01	0.03
1	0	0.34	0	t	1	Ipomoea sp.	Darwinysius marginalis	28	0.01	0.01	0.01	0.03
0	0	0.12	0	cv	1	Persea maericana	Bougainvillea spectabilis	28	0.01	0.08	0.01	0.04
0	0	0.29	0	t	1	Brassica campestris	Diodia radula	28	0.02	0.06	0.02	0.04
0	0	0.29	0	t	1	Lycopersicon cheesmanii minor	Setaria geniculta	28	0.01	0.01	0.01	0.04
0	0	0.24	0	t	1	Justicia galapagana	Solanum americanum	28	0.01	0.04	0.01	0.04
0	0	0.11	0	cv	1	Bastardia viscosa	Manduca rustica	28	0.01	0.08	0.01	0.05
0	0	0.20	0	t	1	Avicennia germinans	Amblycerus piurae	28	0.01	0.03	0.01	0.05
0	0	0.23	0	t	1	Avicennia germinans	Opuntia echios	28	0.01	0.03	0.01	0.06
0	0	0.32	0	t	1	Capsicum frutescens	Crotalaria incana	28	0.03	0.03	0.01	0.08
1	0	0.44	1	cv	1	Agrotis ipsilon	Discaria nana Clos. Weberb.	29	0.01	0.02	0.01	0.02
1	1	0.72	1	t	1	Adesmia haemisphaeric a Hauman	Megachile sp2	29	0.05	0.04	0.02	0.04
0	1	0.63	1	t	1	Adesmia haemisphaeric a Hauman	Phulia sp2	29	0.05	0.04	0.01	0.04
1	1	0.92	1	t	1	Adesmia haemisphaeric a Hauman	Megachile sp1	29	0.05	0.04	0.06	0.04
1	1	0.72	1	cv	1	Adesmia haemisphaeric a Hauman	Tachinidae sp7	29	0.05	0.04	0.02	0.04

1	1	0.79	1	t	1	Adesmia haemisphaerica Hauman	Anthidium chubuti	29	0.05	0.04	0.03	0.04
1	0	0.44	1	t	1	Hypodinerus sp2	Discaria nana Clos. Weberb.	29	0.01	0.02	0.01	0.02
1	1	0.56	1	t	1	Hypodinerus sp1	Calandrinia caespitosa Gill. ex Arn.	29	0.02	0.02	0.01	0.02
1	1	0.54	1	cv	1	Hypodinerus sp1	Discaria nana Clos. Weberb.	29	0.02	0.02	0.01	0.02
0	0	0.44	1	t	1	Azorella monanthos Clos in Gay	Liphantus sp2	29	0.02	0.03	0.01	0.04
1	1	0.73	1	t	1	Azorella monanthos Clos in Gay	Ichneumonidae sp.	29	0.02	0.03	0.04	0.03
0	0	0.46	1	t	1	Azorella monanthos Clos in Gay	Thyridanthrax sp3-2	29	0.02	0.03	0.01	0.03
0	0	0.47	1	cv	1	Azorella monanthos Clos in Gay	Microcerella rusca	29	0.02	0.03	0.01	0.03
0	1	0.53	1	t	1	Azorella monanthos Clos in Gay	Tachinidae sp7	29	0.02	0.03	0.02	0.04
0	1	0.58	1	t	1	Azorella monanthos Clos in Gay	Thyridanthrax sp2	29	0.02	0.03	0.02	0.03
0	0	0.46	1	t	1	Azorella monanthos Clos in Gay	Podagritus sp.	29	0.02	0.03	0.01	0.03
0	1	0.58	1	cv	1	Azorella monanthos Clos in Gay	Chlorobrachycoma versicolor	29	0.02	0.03	0.02	0.03
0	1	0.55	1	t	1	Azorella monanthos Clos in Gay	Anthomyiidae sp1	29	0.02	0.03	0.02	0.04
1	1	0.77	1	t	1	Azorella monanthos Clos in Gay	Coccinela anchoralis	29	0.02	0.03	0.05	0.04
0	1	0.55	1	t	1	Azorella monanthos Clos in Gay	cf. Mosillus sp	29	0.02	0.03	0.02	0.04
1	1	0.71	1	cv	1	Azorella monanthos Clos in Gay	Tachinidae sp1	29	0.02	0.03	0.04	0.04
0	1	0.77	1	t	1	Menonvillea hookeri Rollins	Thysanoptera sp1	29	0.08	0.08	0.01	0.07

1	1	0.73	1	t	1	Liphantus sp2	Jaborosa laciniata Miers A.T. Hunziker & G. Barboza	29	0.01	0.04	0.05	0.04
0	1	0.67	1	t	1	Megachile sp2	Sisyrinchium junceum ssp. junceum E. Meyer ex Presl.	29	0.02	0.04	0.04	0.04
0	0	0.39	1	cv	1	Megachile sp2	Astragalus nivicola Gmez- Sosa	29	0.02	0.04	0.01	0.04
0	1	0.78	1	t	1	Megachile sp2	Senecio looseri Cabr.	29	0.02	0.04	0.05	0.05
0	1	0.60	1	t	1	Ichneumonidae sp.	Senecio tricephalus OK.	29	0.04	0.03	0.01	0.04
1	1	0.82	1	t	1	Tarasa humilis Gill.	Megachile sp1	29	0.02	0.04	0.06	0.04
0	0	0.48	1	cv	1	Tarasa humilis Gill.	Spinoliella sp.	29	0.02	0.04	0.02	0.04
1	0	0.44	1	t	1	Phulia sp1	Discaria nana Clos. Weberb.	29	0.01	0.02	0.01	0.02
1	0	0.44	1	t	1	Issoria lathonioides	Discaria nana Clos. Weberb.	29	0.01	0.02	0.01	0.02
1	1	0.55	1	t	1	Calliphoridae sp1	Cerastium arvense L.	29	0.01	0.02	0.02	0.02
0	0	0.43	1	cv	1	Unknow sp.	Phacelia cf. secunda Gmelin	29	0.01	0.04	0.02	0.04
0	1	0.68	1	t	1	Liphantus sp1	Sisyrinchium junceum ssp. junceum E. Meyer ex Presl.	29	0.02	0.04	0.04	0.04
0	0	0.43	1	t	1	Liphantus sp1	Leuceria candidissima Don	29	0.02	0.04	0.01	0.03
1	0	0.44	1	t	1	Pseudoleucania nobilis	Discaria nana Clos. Weberb.	29	0.01	0.02	0.01	0.02
1	0	0.44	1	cv	1	Dolichogyna sp.	Discaria nana Clos. Weberb.	29	0.01	0.02	0.01	0.02
0	1	0.50	1	t	1	Loasa incurva Crespo & Prez-Moreau	Anthidium chubuti	29	0.01	0.04	0.03	0.04

1	1	0.90	1	t	1	Megachile sp1	Sisyrinchium junceum ssp. junceum E. Meyer ex Presl.	29	0.06	0.04	0.04	0.04
1	1	0.82	1	t	1	Megachile sp1	Phacelia cf. secunda Gmelin	29	0.06	0.04	0.02	0.04
0	1	0.74	1	cv	1	Megachile sp1	Astragalus nivicola Gmez-Sosa	29	0.06	0.04	0.01	0.04
1	1	0.94	1	t	1	Megachile sp1	Senecio looseri Cabr.	29	0.06	0.04	0.05	0.05
1	1	0.86	1	t	1	Megachile sp1	Astragalus cruckshanksii Hook. et Arn. Grisebach	29	0.06	0.04	0.03	0.05
1	0	0.44	1	t	1	Tachinidae sp2	Discaria nana Clos. Weberb.	29	0.01	0.02	0.01	0.02
1	1	0.65	1	cv	1	Cerastium arvense L.	Tropidia notata	29	0.02	0.02	0.02	0.02
0	1	0.55	1	t	1	Chironomidae sp.	Phacelia cf. secunda Gmelin	29	0.02	0.03	0.02	0.04
1	0	0.50	1	t	1	Chironomidae sp.	Discaria nana Clos. Weberb.	29	0.02	0.03	0.01	0.02
1	1	0.87	1	t	1	Alloscirtetica sp.	Sisyrinchium junceum ssp. junceum E. Meyer ex Presl.	29	0.05	0.04	0.04	0.04
1	1	0.66	1	cv	1	Sisyrinchium junceum ssp. junceum E. Meyer ex	Alloscirtetica antarctica	29	0.04	0.04	0.02	0.04
1	1	0.74	1	t	1	Sisyrinchium junceum ssp. junceum E. Meyer ex	Anthidium chubuti	29	0.04	0.04	0.03	0.04
1	1	0.55	1	t	1	Montiopsis gilliesii Hook. et Arn. Ford	Chilimelissa sp.	29	0.01	0.04	0.04	0.05
1	1	0.55	1	t	1	Montiopsis gilliesii Hook. et Arn. Ford	Geron sp.	29	0.01	0.04	0.03	0.03
1	1	0.79	1	cv	1	Jaborosa laciniata Miers A.T. Hunziker & G. Barboza	Anthomyiida e sp1	29	0.05	0.04	0.02	0.04

1	1	0.79	1	t	1	Jaborosa laciniata Miers A.T. Hunziker & G. Barboza	cf. Mosillus sp	29	0.05	0.04	0.02	0.04		
1	1	0.71	1	t	1	Jaborosa laciniata Miers A.T. Hunziker & G. Barboza	Tachinidae sp3	29	0.05	0.04	0.01	0.04		
1	1	0.89	1	t	1	Jaborosa laciniata Miers A.T. Hunziker & G. Barboza	Tachinidae sp1	29	0.05	0.04	0.04	0.04		
0	1	0.61	1	cv	1	Thysanoptera sp1	Senecio looseri Cabr.	29	0.01	0.07	0.05	0.05		
0	0	0.23	1	t	1	Thysanoptera sp1	Senecio tricephalus OK.	29	0.01	0.07	0.01	0.04		
1	1	0.70	1	t	1	Phacelia cf. secunda	Chilimelissa farellones	29	0.02	0.04	0.04	0.04		
0	1	0.51	1	t	1	Phacelia cf. secunda	Tachinidae sp7	29	0.02	0.04	0.02	0.04		
1	1	0.70	1	cv	1	Phacelia cf. secunda	Tachinidae sp1	29	0.02	0.04	0.04	0.04		
0	1	0.54	1	t	1	Astragalus nivicola Gmez-Sosa	Geron sp.	29	0.01	0.04	0.03	0.03		
1	0	0.41	1	t	1	Microcerella rusca	Discaria nana Clos. Weberb.	29	0.01	0.03	0.01	0.02		
1	0	0.44	1	t	1	Dasybaris chillan	Discaria nana Clos. Weberb.	29	0.01	0.02	0.01	0.02		
0	1	0.89	1	cv	1	Astylus sp.	Oxalis erythrorhiza Gillies	29	0.11	0.11	0.02	0.10		
0	1	0.82	1	t	1	Astylus sp.	Hypochoeris montana Phil. Reiche	29	0.11	0.11	0.01	0.11		
0	1	0.78	1	t	1	Tachinidae sp7	Senecio looseri Cabr.	29	0.02	0.04	0.05	0.05		
1	1	0.52	1	t	1	Discaria nana Clos. Weberb.	Thyridanthrax sp2	29	0.01	0.02	0.02	0.03		
1	1	0.52	1	cv	1	Discaria nana Clos. Weberb.	Chlorobrachycoma versicolor	29	0.01	0.02	0.02	0.03		
1	0	0.44	1	t	1	Discaria nana Clos. Weberb.	Thyridanthrax sp1	29	0.01	0.02	0.01	0.02		
1	0	0.44	1	t	1	Discaria nana Clos. Weberb.	Tamseuxoa ingoufii	29	0.01	0.02	0.01	0.02		
1	1	0.61	1	t	1	Discaria nana Clos. Weberb.	Geron sp.	29	0.01	0.02	0.03	0.03		

1	0	0.44	1	cv	1	Discaria nana Clos. Weberb.	Pseudoleucania sp.	29	0.01	0.02	0.01	0.02
1	0	0.44	1	t	1	Discaria nana Clos. Weberb.	Peridroma saucia	29	0.01	0.02	0.01	0.02
0	1	0.66	1	t	1	Discaria nana Clos. Weberb.	Tachinidae sp1	29	0.01	0.02	0.04	0.04
1	1	0.54	1	t	1	Discaria nana Clos. Weberb.	Tropidia notata	29	0.01	0.02	0.02	0.02
1	0	0.44	1	cv	1	Discaria nana Clos. Weberb.	Thyridanthrax sp3	29	0.01	0.02	0.01	0.02
0	1	0.77	1	t	1	Spinoliella sp.	Senecio looseri Cabr.	29	0.02	0.04	0.05	0.05
0	1	0.62	1	t	1	Chilimelissa sp.	Epilobium nivale Meyen	29	0.04	0.05	0.02	0.04
0	0	0.43	1	t	1	Chilimelissa sp.	Oxalis erythrorhiza Gillies	29	0.04	0.05	0.02	0.10
0	1	0.64	1	cv	1	Chilimelissa sp.	Perezia pilifera Don. Hook. et Arnott	29	0.04	0.05	0.02	0.04
0	1	0.65	1	t	1	Geron sp.	Perezia pilifera Don. Hook. et Arnott	29	0.03	0.03	0.02	0.04
0	1	0.67	1	t	1	Senecio looseri Cabr.	Pyrgus cf. fides	29	0.05	0.05	0.01	0.05
0	1	0.81	0	t	1	Hypodinerus sp1	Coccinela anchoralis	29	0.02	0.02	0.05	0.04
0	0	0.30	0	cv	1	Phulia sp2	Astragalus nivicola Gmez-Sosa	29	0.01	0.04	0.01	0.04
1	1	0.69	0	t	1	Ichneumonidae sp.	Spinoliella sp.	29	0.04	0.03	0.02	0.04
0	0	0.45	0	t	1	Astragalus nivicola Gmez-Sosa	Chlorobrachycoma versicolor	29	0.01	0.04	0.02	0.03
0	1	0.96	0	t	1	Calliphoridae sp1	Astylus sp.	29	0.01	0.02	0.11	0.11
0	0	0.42	0	cv	1	Microcerella rusca	Peridroma saucia	29	0.01	0.03	0.01	0.02
0	0	0.39	0	t	1	Unknow sp.	Pseudoleucania nobilis	29	0.01	0.04	0.01	0.02
0	1	0.95	0	t	1	Astylus sp.	Perezia pilifera Don. Hook. et Arnott	29	0.11	0.11	0.02	0.04
0	0	0.31	0	t	1	Unknow sp.	Pyrgus cf. fides	29	0.01	0.04	0.01	0.05
0	1	0.68	0	cv	1	Spinoliella sp.	Tachinidae sp1	29	0.02	0.04	0.04	0.04

0	1	0.71	0	t	1	Cerastium arvense L.	Chilimelissa sp.	29	0.02	0.02	0.04	0.05
0	0	0.31	0	t	1	Tarasa humilis Gill.	Thysanoptera sp1	29	0.02	0.04	0.01	0.07
0	1	0.66	0	t	1	Dolichogyna sp.	Sisyrinchium junceum ssp. junceum E. Meyer ex Presl.	29	0.01	0.02	0.04	0.04
0	0	0.47	0	cv	1	Epilobium nivale Meyen	Pseudoleucania sp.	29	0.02	0.04	0.01	0.02
0	1	0.70	0	t	1	Adesmia haemisphaerica Hauman	Tachinidae sp2	29	0.05	0.04	0.01	0.02
0	0	0.48	0	t	1	Calandrinia caespitosa Gill. ex Arn.	Spinoliella sp.	29	0.01	0.02	0.02	0.04
0	0	0.50	0	t	1	Thysanoptera sp1	Tachinidae sp1	29	0.01	0.07	0.04	0.04
1	0	0.46	0	cv	1	Phulia sp1	Dasybaris chillan	29	0.01	0.02	0.01	0.02
0	1	0.58	0	t	1	Alloscirtetica antarctica	Tropidia notata	29	0.02	0.04	0.02	0.02
0	0	0.46	0	t	1	Spinoliella sp.	Thyridanthrax sp3	29	0.02	0.04	0.01	0.02
1	0	0.45	0	t	1	Hypodinerus sp2	Calliphoridae sp1	29	0.01	0.02	0.01	0.02
0	1	0.57	0	cv	1	Thyridanthrax sp2	cf. Mosillus sp	29	0.02	0.03	0.02	0.04
1	1	0.63	0	t	1	Hypodinerus sp1	Chlorobrachycoma versicolor	29	0.02	0.02	0.02	0.03
1	1	0.76	0	t	1	Alloscirtetica sp.	Spinoliella sp.	29	0.05	0.04	0.02	0.04
0	1	0.55	0	t	1	Alloscirtetica antarctica	Chlorobrachycoma versicolor	29	0.02	0.04	0.02	0.03
0	1	0.63	0	cv	1	Spinoliella sp.	Chilimelissa sp.	29	0.02	0.04	0.04	0.05
0	0	0.48	0	t	1	Phacelia cf. secunda	Peridroma saucia	29	0.02	0.04	0.01	0.02
0	1	0.78	0	t	1	Megachile sp2	Alloscirtetica sp.	29	0.02	0.04	0.05	0.04
0	1	0.97	0	t	1	Astylus sp.	Tachinidae sp1	29	0.11	0.11	0.04	0.04
0	1	0.63	0	cv	1	Adesmia haemisphaerica Hauman	Loasa incurva Crespo & Prez-Moreau	29	0.05	0.04	0.01	0.04

0	0	0.36	0	t	1	Thyridanthrax sp3-2	Leuceria candidissima Don	29	0.01	0.03	0.01	0.03
0	1	0.51	0	t	1	Megachile sp2	Liphantus sp1	29	0.02	0.04	0.02	0.04
0	1	0.74	0	t	1	Tamseuxoa ingoufii	Coccinela anchoralis	29	0.01	0.02	0.05	0.04
1	0	0.40	0	cv	1	Discaria nana Clos. Weberb.	Podagritus sp.	29	0.01	0.02	0.01	0.03
0	1	0.86	0	t	1	Menonvillea hookeri Rollins	Calliphoridae sp1	29	0.08	0.08	0.01	0.02
0	0	0.36	0	t	1	Podagritus sp.	Leuceria candidissima Don	29	0.01	0.03	0.01	0.03
0	1	0.73	0	t	1	Megachile sp1	Pyrgus cf. fides	29	0.06	0.04	0.01	0.05
0	1	0.60	0	cv	1	Cerastium arvense L.	Perezia pilifera Don. Hook. et Arnott	29	0.02	0.02	0.02	0.04
0	1	0.77	0	t	1	Sisyrinchium junceum ssp. junceum E. Meyer ex	Geron sp.	29	0.04	0.04	0.03	0.03
0	0	0.27	0	t	1	Thyridanthrax sp3-2	Thysanoptera sp1	29	0.01	0.03	0.01	0.07
0	0	0.47	0	t	1	Chlorobrachycoma versicolor	Leuceria candidissima Don	29	0.02	0.03	0.01	0.03
0	0	0.31	0	cv	1	Dolichogyna sp.	Thysanoptera sp1	29	0.01	0.02	0.01	0.07
1	1	0.93	0	t	1	Megachile sp1	Coccinela anchoralis	29	0.06	0.04	0.05	0.04
0	1	0.51	0	t	1	Alloscirtetica antarctica	Phacelia cf. secunda Gmelin	29	0.02	0.04	0.02	0.04
0	0	0.47	0	t	1	Epilobium nivale Meyen	Peridroma saucia	29	0.02	0.04	0.01	0.02
1	1	0.56	0	cv	1	Hypodinerus sp1	Dasybaris chillan	29	0.02	0.02	0.01	0.02
0	0	0.43	0	t	1	Hypodinerus sp2	Microcerella rusca	29	0.01	0.02	0.01	0.03
0	1	0.93	0	t	1	Astylus sp.	Discaria nana Clos. Weberb.	29	0.11	0.11	0.01	0.02
0	1	0.96	0	t	1	Pseudoleucania nobilis	Astylus sp.	29	0.01	0.02	0.11	0.11
0	1	0.78	0	cv	1	Calliphoridae sp1	Jaborosa laciniata Miers A.T. Hunziker & G. Barboza	29	0.01	0.02	0.05	0.04

0	0	0.36	0	t	1	Oxalis erythrorhiza Gillies	Tropidia notata	29	0.02	0.10	0.02	0.02
0	0	0.34	0	t	1	Sisyrinchium junceum ssp. junceum E. Meyer ex	Hypochoeris montana Phil. Reiche	29	0.04	0.04	0.01	0.11
0	0	0.30	0	t	1	Issoria lathonioides	Oxalis erythrorhiza Gillies	29	0.01	0.02	0.02	0.10
0	0	0.22	0	cv	1	Phacelia cf. secunda Gmelin	Hypochoeris montana Phil. Reiche	29	0.02	0.04	0.01	0.11
0	0	0.49	0	t	1	Thyridanthrax sp3-2	Thyridanthra x sp2	29	0.01	0.03	0.02	0.03
0	0	0.40	0	t	1	Phulia sp2	Epilobium nivale Meyen	29	0.01	0.04	0.02	0.04
0	0	0.47	0	t	1	Senecio looseri Cabr.	Hypochoeris montana Phil. Reiche	29	0.05	0.05	0.01	0.11
0	1	0.50	0	cv	1	Calandrinia caespitosa Gill. ex Arn.	cf. Mosillus sp	29	0.01	0.02	0.02	0.04
0	0	0.43	0	t	1	Azorella monanthos Clos in Gay	Montiopsis gilliesii Hook. et Arn. Ford	29	0.02	0.03	0.01	0.04
0	0	0.43	0	t	1	Agrotis ipsilon	Microcerella rusca	29	0.01	0.02	0.01	0.03
0	0	0.47	0	t	1	Tachinidae sp7	Pseudoleucan ia sp.	29	0.02	0.04	0.01	0.02
0	0	0.48	0	cv	1	Calliphoridae sp1	Alloscirtetica antarctica	29	0.01	0.02	0.02	0.04
0	1	0.58	0	t	1	Phulia sp1	Anthidium chubuti	29	0.01	0.02	0.03	0.04
0	1	0.69	0	t	1	Jaborosa laciniata Miers A.T. Hunziker & G. Barboza	Pyrgus cf. fides	29	0.05	0.04	0.01	0.05
0	0	0.35	0	t	1	Hypochoeris montana Phil. Reiche	Tachinidae sp1	29	0.01	0.11	0.04	0.04
0	0	0.48	0	cv	1	Discaria nana Clos. Weberb.	cf. Mosillus sp	29	0.01	0.02	0.02	0.04
0	1	0.68	0	t	1	Chilimelissa farellones	Tachinidae sp7	29	0.04	0.04	0.02	0.04
0	1	0.94	0	t	1	Astylus sp.	Pseudoleucan ia sp.	29	0.11	0.11	0.01	0.02
0	1	0.53	0	t	1	Microcerella rusca	Astragalus cruckshanksii Hook. et Arn. Grisebach	29	0.01	0.03	0.03	0.05

0	1	0.52	0	cv	1	Thyridanthrax sp3-2	Astragalus cruckshanksii Hook. et Arn. Grisebach	29	0.01	0.03	0.03	0.05
0	1	0.57	0	t	1	Sisyrinchium junceum ssp. junceum E. Meyer ex	Senecio tricephalus OK.	29	0.04	0.04	0.01	0.04
0	1	0.96	0	t	1	Menonvillea hookeri Rollins	Alloscirtetica sp.	29	0.08	0.08	0.05	0.04
0	0	0.25	0	t	1	Thyridanthrax sp2	Hypochoeris montana Phil. Reiche	29	0.02	0.03	0.01	0.11
0	1	0.98	0	cv	1	Sisyrinchium junceum ssp. junceum E. Meyer ex	Astylus sp.	29	0.04	0.04	0.11	0.11
0	0	0.48	0	t	1	Loasa incurva Crespo & Prez-Moreau	Tropidia notata	29	0.01	0.04	0.02	0.02
0	0	0.32	0	t	1	Loasa incurva Crespo & Prez-Moreau	Montiopsis gilliesii Hook. et Arn. Ford	29	0.01	0.04	0.01	0.04
0	1	0.79	0	t	1	Dolichogyna sp.	Jaborosa laciniata Miers A.T. Hunziker & G. Barboza	29	0.01	0.02	0.05	0.04
0	0	0.38	0	cv	1	Oxalis erythrorhiza Gillies	Anthidium chubuti	29	0.02	0.10	0.03	0.04
0	0	0.39	0	t	1	Issoria lathonioides	Astragalus nivicola Gmez-Sosa	29	0.01	0.02	0.01	0.04
0	0	0.39	0	t	1	Pseudoleucania nobilis	Loasa incurva Crespo & Prez-Moreau	29	0.01	0.02	0.01	0.04
1	1	0.76	0	t	1	Phacelia cf. secunda	Coccinela anchoralis	29	0.02	0.04	0.05	0.04
0	0	0.42	0	cv	1	Issoria lathonioides	Podagritus sp.	29	0.01	0.02	0.01	0.03
1	0	0.46	0	t	1	Phulia sp1	Pseudoleucania sp.	29	0.01	0.02	0.01	0.02
1	0	0.38	1	t	1	Senecio subulatus Don ex Hook. &	Ammophila sp.	30	0.03	0.05	0.01	0.05
0	1	0.50	1	t	1	Senecio subulatus Don ex Hook. &	Tachinidae sp5	30	0.03	0.05	0.02	0.04

0	0	0.39	1	cv	1	Senecio subulatus Don ex Hook. &	Scaeva occidentalis	30	0.03	0.05	0.02	0.08
0	0	0.34	1	t	1	Senecio subulatus Don ex Hook. &	Copestylum aricia	30	0.03	0.05	0.01	0.07
0	1	0.50	1	t	1	Senecio subulatus Don ex Hook. &	Sarcophagidae sp.	30	0.03	0.05	0.03	0.08
1	0	0.25	1	t	1	Oxalis sp.	Centris sp.	30	0.02	0.05	0.02	0.07
1	0	0.34	1	cv	1	Oxalis sp.	Megachile sp4	30	0.02	0.05	0.02	0.06
0	0	0.14	1	t	1	Empididae sp.	Grindelia chiloensis (Corn.) Cabrera	30	0.01	0.07	0.02	0.08
1	1	0.55	1	t	1	Tatochila sp.	Junellia cf. tonini (Kuntze) Moldenke	30	0.02	0.02	0.02	0.02
1	0	0.46	1	t	1	Tatochila sp.	Arjona patagonica Hombron & Jacquinot	30	0.02	0.02	0.01	0.02
1	1	0.88	1	cv	1	Geometridae sp.	Discaria trinervis (Hook. et Arn.) Reiche	30	0.01	0.09	0.11	0.07
0	0	0.15	1	t	1	Bibionidae sp2	Grindelia chiloensis (Corn.) Cabrera	30	0.02	0.09	0.02	0.08
1	1	0.91	1	t	1	Bibionidae sp2	Discaria trinervis (Hook. et Arn.) Reiche	30	0.02	0.09	0.11	0.07
0	0	0.15	1	t	1	Bibionidae sp1	Grindelia chiloensis (Corn.) Cabrera	30	0.02	0.09	0.02	0.08
1	1	0.91	1	cv	1	Bibionidae sp1	Discaria trinervis (Hook. et Arn.) Reiche	30	0.02	0.09	0.11	0.07
1	0	0.28	1	t	1	Lecanophora heterophylla (Cav.) Krapovickas	Megachile sp3	30	0.01	0.03	0.01	0.04

1	1	0.88	1	t	1	Ephydridae sp.	Discaria trinervis (Hook. et Arn.) Reiche	30	0.01	0.09	0.11	0.07
1	0	0.28	1	t	1	Mimulus sp.	Megachile sp4	30	0.01	0.05	0.02	0.06
0	0	0.32	1	cv	1	Arhysosage bifasciata	Pyrrhocactus cf. strausianum (K. Schum.) Berger	30	0.02	0.07	0.02	0.05
0	0	0.23	1	t	1	Arhysosage bifasciata	Opuntia corrugata S-D.	30	0.02	0.07	0.03	0.10
1	1	0.58	1	t	1	Tachinidae sp5	Rosa rubiginosa L.	30	0.02	0.04	0.03	0.03
0	0	0.14	1	t	1	Tachinidae sp4	Grindelia chiloensis (Corn.) Cabrera	30	0.01	0.07	0.02	0.08
0	0	0.18	1	cv	1	Centris sp.	Lathyrus sp.	30	0.02	0.07	0.02	0.10
1	1	0.88	1	t	1	Tachinidae sp6	Discaria trinervis (Hook. et Arn.) Reiche	30	0.01	0.09	0.11	0.07
1	0	0.34	1	t	1	Pyrrhocactus cf. strausianum	Megachile sp3	30	0.02	0.05	0.01	0.04
1	0	0.34	1	t	1	Pyrrhocactus cf. strausianum	Psaenythia rufipes	30	0.02	0.05	0.02	0.06
0	0	0.22	1	cv	1	Prionyx sp.	Baccharis pingraea DC.	30	0.01	0.06	0.02	0.06
0	0	0.30	1	t	1	Rapistrum rugosum (L.) Allioni	Scaeva occidentalis	30	0.02	0.05	0.02	0.08
0	0	0.26	1	t	1	Rapistrum rugosum (L.) Allioni	Copestylum aricia	30	0.02	0.05	0.01	0.07
1	0	0.33	1	t	1	Rapistrum rugosum (L.) Allioni	Eristalis bogotensis	30	0.02	0.05	0.01	0.04
0	0	0.14	1	cv	1	Curculionidae sp.	Grindelia chiloensis (Corn.) Cabrera	30	0.01	0.07	0.02	0.08
0	0	0.36	1	t	1	Junellia cf. tonini (Kuntze) Moldenke	Vanessa cf. cardui	30	0.02	0.02	0.01	0.05

1	1	0.88	1	t	1	Thysanoptera sp2	Discaria trinervis (Hook. et Arn.) Reiche	30	0.01	0.09	0.11	0.07
1	0	0.34	1	t	1	Colletes lycii	Polygala stenophylla A. Gray	30	0.01	0.03	0.01	0.02
1	1	0.57	1	cv	1	Colletes lycii	Larrea divaricata Cav.	30	0.01	0.03	0.03	0.03
1	1	0.60	1	t	1	Sappho sparganura	Mutisia decurrens Cav.	30	0.02	0.02	0.02	0.02
1	0	0.46	1	t	1	Sappho sparganura	Oenothera mendocinensis Gilles ex Hook. et Arn.	30	0.02	0.02	0.01	0.02
1	1	0.91	1	t	1	Alipumilio sp.	Discaria trinervis (Hook. et Arn.) Reiche	30	0.02	0.09	0.11	0.07
1	1	0.56	1	cv	1	Epicauta sp.	Larrea divaricata Cav.	30	0.01	0.03	0.03	0.03
1	1	0.88	1	t	1	Calliphoridae sp2	Discaria trinervis (Hook. et Arn.) Reiche	30	0.01	0.09	0.11	0.07
0	0	0.29	1	t	1	Arjona patagonica Hombron & Jacquinot	Vanessa cf. cardui	30	0.01	0.02	0.01	0.05
0	0	0.12	1	t	1	Grindelia chiloensis (Corn.)	Mordellidae sp.	30	0.02	0.08	0.01	0.09
0	0	0.18	1	cv	1	Grindelia chiloensis (Corn.)	Callonychium mandibulare	30	0.02	0.08	0.01	0.05
0	0	0.19	1	t	1	Grindelia chiloensis (Corn.)	Vanessa cf. cardui	30	0.02	0.08	0.01	0.05
0	0	0.28	1	t	1	Grindelia chiloensis (Corn.)	Sphecidae sp.	30	0.02	0.08	0.02	0.05
0	0	0.19	1	t	1	Grindelia chiloensis (Corn.)	Copestylum sp2	30	0.02	0.08	0.01	0.05
0	0	0.17	1	cv	1	Grindelia chiloensis (Corn.)	Scaeva occidentalis	30	0.02	0.08	0.02	0.08

0	0	0.19	1	t	1	Grindelia chiloensis (Corn.)	Megachile sp3	30	0.02	0.08	0.01	0.04
0	0	0.20	1	t	1	Grindelia chiloensis (Corn.)	Dialictus sp1	30	0.02	0.08	0.02	0.08
0	1	0.93	1	t	1	Grindelia chiloensis (Corn.)	Dialictus sp2	30	0.02	0.08	0.12	0.10
0	0	0.18	1	cv	1	Grindelia chiloensis (Corn.)	Buprestidae sp.	30	0.02	0.08	0.01	0.05
0	0	0.24	1	t	1	Grindelia chiloensis (Corn.)	Svastrides zebra	30	0.02	0.08	0.02	0.05
0	0	0.20	1	t	1	Grindelia chiloensis (Corn.)	Psaenythia rufipes	30	0.02	0.08	0.02	0.06
0	0	0.32	1	t	1	Grindelia chiloensis (Corn.)	Alloscirtetica herbsti	30	0.02	0.08	0.03	0.06
0	0	0.12	1	cv	1	Grindelia chiloensis (Corn.)	Coccinelidae sp1	30	0.02	0.08	0.01	0.09
0	0	0.14	1	t	1	Grindelia chiloensis (Corn.)	Copestylum aricia	30	0.02	0.08	0.01	0.07
0	0	0.14	1	t	1	Grindelia chiloensis (Corn.)	Muscidae sp3	30	0.02	0.08	0.01	0.07
0	0	0.14	1	t	1	Grindelia chiloensis (Corn.)	Muscidae sp1	30	0.02	0.08	0.01	0.07
0	0	0.14	1	cv	1	Grindelia chiloensis (Corn.)	cf. Entypus sp.	30	0.02	0.08	0.01	0.07
0	0	0.15	1	t	1	Grindelia chiloensis (Corn.)	Muscidae sp4	30	0.02	0.08	0.02	0.09
0	0	0.14	1	t	1	Grindelia chiloensis (Corn.)	Eriopis cf. conexa	30	0.02	0.08	0.01	0.07
0	0	0.15	1	t	1	Grindelia chiloensis (Corn.)	Compsomyiops fulvicrura	30	0.02	0.08	0.02	0.09
0	0	0.20	1	cv	1	Grindelia chiloensis (Corn.)	Carposcalis punctulata	30	0.02	0.08	0.02	0.09
0	0	0.14	1	t	1	Grindelia chiloensis (Corn.)	Hemiptera sp2	30	0.02	0.08	0.01	0.07

0	0	0.25	1	t	1	Grindelia chiloensis (Corn.)	Sarcophagidae sp.	30	0.02	0.08	0.03	0.08
0	0	0.14	1	t	1	Grindelia chiloensis (Corn.)	Diadasia patagonica	30	0.02	0.08	0.01	0.07
0	0	0.24	1	cv	1	Grindelia chiloensis (Corn.)	Apis mellifera	30	0.02	0.08	0.02	0.05
0	0	0.14	1	t	1	Grindelia chiloensis (Corn.)	Cantharidae sp.	30	0.02	0.08	0.01	0.07
0	0	0.14	1	t	1	Grindelia chiloensis (Corn.)	Tephritidae sp.	30	0.02	0.08	0.01	0.07
0	0	0.14	1	t	1	Grindelia chiloensis (Corn.)	Chalepogenus caeruleus	30	0.02	0.08	0.01	0.07
1	1	0.54	1	cv	1	Eumenidae sp.	Senecio filaginoides DC.	30	0.02	0.03	0.02	0.03
1	1	0.52	1	t	1	Phacelia cf. secunda	Anthidium rubriceps	30	0.01	0.02	0.02	0.02
1	1	0.88	1	t	1	Mordellidae sp.	Discaria trinervis (Hook. et Arn.) Reiche	30	0.01	0.09	0.11	0.07
1	1	0.91	1	t	1	Thyridanthrax sp4	Discaria trinervis (Hook. et Arn.) Reiche	30	0.02	0.09	0.11	0.07
1	1	0.88	1	cv	1	Anthomyiidae sp2	Discaria trinervis (Hook. et Arn.) Reiche	30	0.01	0.09	0.11	0.07
0	0	0.39	1	t	1	Callonychium mandibulare	Hysterionica jasionoides Willd.	30	0.01	0.05	0.03	0.05
0	0	0.37	1	t	1	Callonychium mandibulare	Thelesperma megapotamicum (Spreng.) O. Kuntze	30	0.01	0.05	0.02	0.04
0	0	0.35	1	t	1	Sphecidae sp.	Baccharis pingraea DC.	30	0.02	0.05	0.02	0.06
0	0	0.49	1	cv	1	Sphecidae sp.	Thelesperma megapotamicum (Spreng.) O. Kuntze	30	0.02	0.05	0.02	0.04
0	0	0.50	1	t	1	Copestylum sp2	Larrea divaricata Cav.	30	0.01	0.05	0.03	0.03

0	1	0.64	1	t	1	Baccharis pingraea DC.	Syrphidae sp3	30	0.02	0.06	0.06	0.08
0	0	0.22	1	t	1	Baccharis pingraea DC.	Scaeva occidentalis	30	0.02	0.06	0.02	0.08
0	0	0.26	1	cv	1	Baccharis pingraea DC.	Dialictus sp1	30	0.02	0.06	0.02	0.08
0	1	0.55	1	t	1	Baccharis pingraea DC.	Oxybelus sp.	30	0.02	0.06	0.04	0.04
0	0	0.22	1	t	1	Baccharis pingraea DC.	Muscidae sp2	30	0.02	0.06	0.01	0.06
0	0	0.25	1	t	1	Baccharis pingraea DC.	Carposcalis punctulata	30	0.02	0.06	0.02	0.09
0	0	0.31	1	cv	1	Baccharis pingraea DC.	Sarcophagidae sp.	30	0.02	0.06	0.03	0.08
0	0	0.22	1	t	1	Baccharis pingraea DC.	cf. Notiphila sp.	30	0.02	0.06	0.01	0.06
0	0	0.22	1	t	1	Baccharis pingraea DC.	Caenohalictus sp1	30	0.02	0.06	0.01	0.06
1	1	0.99	1	t	1	Syrphidae sp3	Discaria trinervis (Hook. et Arn.) Reiche	30	0.06	0.08	0.11	0.07
1	0	0.35	1	cv	1	Scaeva occidentalis	Hysterionica jasionoides Willd.	30	0.02	0.08	0.03	0.05
1	1	0.92	1	t	1	Scaeva occidentalis	Discaria trinervis (Hook. et Arn.) Reiche	30	0.02	0.08	0.11	0.07
1	1	0.96	1	t	1	Hypodinerus sp3	Discaria trinervis (Hook. et Arn.) Reiche	30	0.04	0.09	0.11	0.07
0	0	0.24	1	t	1	Dialictus sp1	Opuntia corrugata S-D.	30	0.02	0.08	0.03	0.10
0	1	0.91	1	cv	1	Dialictus sp2	Lathyrus sp.	30	0.12	0.10	0.02	0.10
0	1	0.93	1	t	1	Dialictus sp2	Opuntia corrugata S-D.	30	0.12	0.10	0.03	0.10
1	0	0.27	1	t	1	Megachile sp4	Adesmia retrofracta Hook. et Arn.	30	0.02	0.06	0.01	0.05
0	0	0.25	1	t	1	Megachile sp4	Lathyrus sp.	30	0.02	0.06	0.02	0.10
0	0	0.40	1	cv	1	Hysterionica jasionoides Willd.	Psaenythia rufipes	30	0.03	0.05	0.02	0.06
0	0	0.32	1	t	1	Hysterionica jasionoides Willd.	Copestylum aricia	30	0.03	0.05	0.01	0.07

1	0	0.41	1	t	1	Hysterionica jasionoides Willd.	Hemiptera sp1	30	0.03	0.05	0.01	0.04
0	1	0.57	1	t	1	Larrea divaricata Cav.	Svastrides zebra	30	0.03	0.03	0.02	0.05
0	0	0.42	1	cv	1	Larrea divaricata Cav.	Copestylum aricia	30	0.03	0.03	0.01	0.07
1	1	0.55	1	t	1	Larrea divaricata Cav.	Centris muralis	30	0.03	0.03	0.01	0.03
1	1	0.88	1	t	1	Eristalis tenax	Discaria trinervis (Hook. et Arn.) Reiche	30	0.01	0.09	0.11	0.07
0	0	0.49	1	t	1	Buprestidae sp.	Rosa rubiginosa L.	30	0.01	0.05	0.03	0.03
0	0	0.24	1	cv	1	Psaenythia rufipes	Opuntia corrugata S-D.	30	0.02	0.06	0.03	0.10
1	1	0.88	1	t	1	Coccinelidae sp1	Discaria trinervis (Hook. et Arn.) Reiche	30	0.01	0.09	0.11	0.07
1	1	0.91	1	t	1	Coccinelidae sp2	Discaria trinervis (Hook. et Arn.) Reiche	30	0.02	0.09	0.11	0.07
1	1	0.88	1	t	1	Coccinelidae sp3	Discaria trinervis (Hook. et Arn.) Reiche	30	0.01	0.09	0.11	0.07
0	0	0.25	1	cv	1	Copestylum aricia	Senecio filaginoides DC.	30	0.01	0.07	0.02	0.03
0	0	0.29	1	t	1	Copestylum aricia	Thelesperma megapotamicum (Spreng.) O. Kuntze	30	0.01	0.07	0.02	0.04
0	0	0.41	1	t	1	Copestylum aricia	Rosa rubiginosa L.	30	0.01	0.07	0.03	0.03
1	1	0.91	1	t	1	Copestylum aricia	Discaria trinervis (Hook. et Arn.) Reiche	30	0.01	0.07	0.11	0.07
1	1	0.98	1	cv	1	cf. Microcerella sp.	Discaria trinervis (Hook. et Arn.) Reiche	30	0.05	0.08	0.11	0.07
1	1	0.56	1	t	1	cf. Toxomerus sp.	Rosa rubiginosa L.	30	0.01	0.03	0.03	0.03

1	1	0.91	1	t	1	Muscidae sp4	Discaria trinervis (Hook. et Arn.) Reiche	30	0.02	0.09	0.11	0.07
1	1	0.91	1	t	1	Compsomyiops fulvicrura	Discaria trinervis (Hook. et Arn.) Reiche	30	0.02	0.09	0.11	0.07
1	1	0.93	1	cv	1	Carposcalis punctulata	Discaria trinervis (Hook. et Arn.) Reiche	30	0.02	0.09	0.11	0.07
0	1	0.57	1	t	1	Rosa rubiginosa L.	Apis mellifera	30	0.03	0.03	0.02	0.05
1	1	0.95	1	t	1	Sarcophagidae sp.	Discaria trinervis (Hook. et Arn.) Reiche	30	0.03	0.08	0.11	0.07
0	1	0.86	1	t	1	Discaria trinervis (Hook. et Arn.)	Augochloropsis sp.	30	0.11	0.07	0.01	0.09
0	1	0.86	1	cv	1	Discaria trinervis (Hook. et Arn.)	Caenohalictus sp2	30	0.11	0.07	0.01	0.09
0	1	0.86	1	t	1	Discaria trinervis (Hook. et Arn.)	Carposcalis saltana	30	0.11	0.07	0.01	0.09
0	1	0.86	1	t	1	Discaria trinervis (Hook. et Arn.)	Ocyptamus meridionalis	30	0.11	0.07	0.01	0.09
0	0	0.21	0	t	1	Oxalis sp.	Thyridanthrax sp4	30	0.02	0.05	0.02	0.09
0	0	0.19	0	cv	1	Arhysosage bifasciata	Grindelia chiloensis (Corn.) Cabrera	30	0.02	0.07	0.02	0.08
0	0	0.49	0	t	1	Adesmia retrofracta Hook. et Arn.	Rosa rubiginosa L.	30	0.01	0.05	0.03	0.03
0	0	0.38	0	t	1	Alloscirtetica herbsti	Opuntia corrugata S-D.	30	0.03	0.06	0.03	0.10
0	0	0.35	0	t	1	Pyrrhocactus cf. strausianum	Dialictus sp1	30	0.02	0.05	0.02	0.08
0	0	0.29	0	cv	1	Vanessa cf. cardui	Oenothera mendocinensis Gilles ex Hook. et Arn.	30	0.01	0.05	0.01	0.02
0	1	0.97	0	t	1	Hypodinerus sp3	Dialictus sp2	30	0.04	0.09	0.12	0.10

0	0	0.49	0	t	1	Eumenidae sp.	Svastrides zebra	30	0.02	0.03	0.02	0.05
1	0	0.27	0	t	1	Lecanophora heterophylla (Cav.) Krapovickas	Mimulus sp.	30	0.01	0.03	0.01	0.05
0	0	0.20	0	cv	1	Psaenythia rufipes	Muscidae sp2	30	0.02	0.06	0.01	0.06
0	0	0.34	0	t	1	Anthidium rubriceps	Hemiptera sp2	30	0.02	0.02	0.01	0.07
0	0	0.15	0	t	1	Prionyx sp.	Copestylum aricia	30	0.01	0.06	0.01	0.07
1	0	0.19	0	t	1	Adesmia retrofracta Hook. et Arn.	Muscidae sp2	30	0.01	0.05	0.01	0.06
1	0	0.47	0	cv	1	Lecanophora heterophylla (Cav.) Krapovickas	Hysterionica jasionoides Willd.	30	0.01	0.03	0.03	0.05
0	0	0.21	0	t	1	Rapistrum rugosum (L.) Allioni	Caenohalictus sp2	30	0.02	0.05	0.01	0.09
0	0	0.17	0	t	1	Ephydridae sp.	Polygala stenophylla A. Gray	30	0.01	0.09	0.01	0.02
0	1	0.98	0	t	1	Dialictus sp2	Rosa rubiginosa L.	30	0.12	0.10	0.03	0.03
1	0	0.25	0	cv	1	Buprestidae sp.	cf. Toxomerus sp.	30	0.01	0.05	0.01	0.03
0	0	0.25	0	t	1	Colletes lycii	Grindelia chiloensis (Corn.) Cabrera	30	0.01	0.03	0.02	0.08
0	0	0.42	0	t	1	Syrphidae sp3	Caenohalictus sp2	30	0.06	0.08	0.01	0.09
0	0	0.21	0	t	1	Rapistrum rugosum (L.) Allioni	Ocyptamus meridionalis	30	0.02	0.05	0.01	0.09
0	0	0.29	0	cv	1	Svastrides zebra	Psaenythia rufipes	30	0.02	0.05	0.02	0.06
0	0	0.18	0	t	1	Callonychium mandibulare	Compsomyiops fulvicrura	30	0.01	0.05	0.02	0.09
0	0	0.43	0	t	1	Larrea divaricata Cav.	Compsomyiops fulvicrura	30	0.03	0.03	0.02	0.09
0	0	0.22	0	t	1	Epicauta sp.	Coccinelidae sp2	30	0.01	0.03	0.02	0.09
0	0	0.29	0	cv	1	Bibionidae sp1	Alloscirtetica herbsti	30	0.02	0.09	0.03	0.06
0	0	0.41	0	t	1	Thyridanthrax sp4	Rosa rubiginosa L.	30	0.02	0.09	0.03	0.03

0	0	0.34	0	t	1	Thyridanthrax sp4	Anthidium rubriceps	30	0.02	0.09	0.02	0.02
1	0	0.09	0	t	1	Ephydridae sp.	cf. Entypus sp.	30	0.01	0.09	0.01	0.07
0	0	0.21	0	cv	1	Lecanophora heterophylla (Cav.) Krapovickas	Eriopis cf. conexa	30	0.01	0.03	0.01	0.07
0	1	0.58	0	t	1	Larrea divaricata Cav.	Sarcophagidae sp.	30	0.03	0.03	0.03	0.08
0	1	0.50	0	t	1	cf. Microcerella	Hemiptera sp1	30	0.05	0.08	0.01	0.04
1	0	0.12	0	t	1	Bibionidae sp1	Cantharidae sp.	30	0.02	0.09	0.01	0.07
0	0	0.22	0	cv	1	Senecio filaginoides	Augochloropsis sp.	30	0.02	0.03	0.01	0.09
0	1	0.96	0	t	1	Senecio filaginoides DC.	Discaria trinervis (Hook. et Arn.) Reiche	30	0.02	0.03	0.11	0.07
0	0	0.32	0	t	1	Tatochila sp.	Alipumilio sp.	30	0.02	0.02	0.02	0.09
0	0	0.22	0	t	1	Pyrrhocactus cf. strausianum	Ocyptamus meridionalis	30	0.02	0.05	0.01	0.09
0	0	0.15	0	cv	1	Baccharis pingraea DC.	Augochloropsis sp.	30	0.02	0.06	0.01	0.09
0	0	0.13	0	t	1	Lathyrus sp.	Muscidae sp3	30	0.02	0.10	0.01	0.07
0	1	0.95	0	t	1	Lecanophora heterophylla (Cav.) Krapovickas	Discaria trinervis (Hook. et Arn.) Reiche	30	0.01	0.03	0.11	0.07
1	0	0.34	0	t	1	Colletes lycii	Apis mellifera	30	0.01	0.03	0.02	0.05
0	0	0.38	0	cv	1	Prionyx sp.	Anthidium rubriceps	30	0.01	0.06	0.02	0.02
0	0	0.18	0	t	1	Bibionidae sp2	Carposcalis punctulata	30	0.02	0.09	0.02	0.09
0	0	0.41	0	t	1	Tatochila sp.	Opuntia corrugata S-D.	30	0.02	0.02	0.03	0.10
0	1	0.95	0	t	1	Svastrides zebra	Discaria trinervis (Hook. et Arn.) Reiche	30	0.02	0.05	0.11	0.07
0	0	0.10	0	cv	1	Geometridae sp.	Copestylum aricia	30	0.01	0.09	0.01	0.07
0	0	0.10	0	t	1	Muscidae sp4	Augochloropsis sp.	30	0.02	0.09	0.01	0.09
0	0	0.27	0	t	1	Tachinidae sp6	Anthidium rubriceps	30	0.01	0.09	0.02	0.02
0	1	0.64	0	t	1	Syrphidae sp3	Apis mellifera	30	0.06	0.08	0.02	0.05

0	0	0.31	0	cv	1	Baccharis pingraea DC.	Polygala stenophylla A. Gray	30	0.02	0.06	0.01	0.02
0	0	0.37	0	t	1	Mutisia decurrens Cav.	Muscidae sp4	30	0.02	0.02	0.02	0.09
0	0	0.38	0	t	1	Sappho sparganura	Psaenythia rufipes	30	0.02	0.02	0.02	0.06
0	0	0.11	0	t	1	Muscidae sp3	Cantharidae sp.	30	0.01	0.07	0.01	0.07
0	0	0.08	0	cv	1	Geometridae sp.	Caenohalictus sp2	30	0.01	0.09	0.01	0.09
0	0	0.41	0	t	1	Oxybelus sp.	Muscidae sp3	30	0.04	0.04	0.01	0.07
0	0	0.47	0	t	1	Grindelia chiloensis (Corn.)	Oxybelus sp.	30	0.02	0.08	0.04	0.04
1	0	0.45	0	t	1	Lecanophora heterophylla (Cav.) Krapovickas	Thelesperma megapotamicum (Spreng.) O. Kuntze	30	0.01	0.03	0.02	0.04
0	0	0.41	0	cv	1	Rapistrum rugosum (L.) Allioni	Oenothera mendocinensis Gilles ex Hook. et Arn.	30	0.02	0.05	0.01	0.02
0	1	0.73	0	t	1	Eumenidae sp.	cf. Microcerella sp.	30	0.02	0.03	0.05	0.08
0	0	0.27	0	t	1	Arjona patagonica Hombron & Jacquinot	Grindelia chiloensis (Corn.) Cabrera	30	0.01	0.02	0.02	0.08
0	0	0.29	0	t	1	Hypodinerus sp3	Compsomyiops fulvicrura	30	0.04	0.09	0.02	0.09
0	0	0.27	0	cv	1	Calliphoridae sp2	Anthidium rubriceps	30	0.01	0.09	0.02	0.02
0	0	0.08	0	t	1	Mordellidae sp.	Carposcalis saltana	30	0.01	0.09	0.01	0.09
1	0	0.49	0	t	1	Oxybelus sp.	Buprestidae sp.	30	0.04	0.04	0.01	0.05
0	0	0.12	0	t	1	Geometridae sp.	Mimulus sp.	30	0.01	0.09	0.01	0.05
0	0	0.37	0	cv	1	Psaenythia rufipes	Thelesperma megapotamicum (Spreng.) O. Kuntze	30	0.02	0.06	0.02	0.04
0	0	0.41	0	t	1	Rapistrum rugosum (L.) Allioni	Polygala stenophylla A. Gray	30	0.02	0.05	0.01	0.02
1	0	0.47	0	t	1	Tatochila sp.	Oenothera mendocinensis Gilles ex Hook. et Arn.	30	0.02	0.02	0.01	0.02

1	1	0.71	0	t	1	Eumenidae sp.	Larrea divaricata Cav.	30	0.02	0.03	0.03	0.03
0	0	0.09	0	cv	1	Tachinidae sp4	Coccinelidae sp3	30	0.01	0.07	0.01	0.09
1	0	0.19	0	t	1	Bibionidae sp1	Baccharis pingraea DC.	30	0.02	0.09	0.02	0.06
1	0	0.12	0	t	1	Muscidae sp4	Hemiptera sp2	30	0.02	0.09	0.01	0.07
0	0	0.30	0	t	1	Alipumilio sp.	Hypodinerus sp3	30	0.02	0.09	0.04	0.09
0	1	0.78	0	cv	1	Larrea divaricata Cav.	Rosa rubiginosa L.	30	0.03	0.03	0.03	0.03
0	0	0.40	0	t	1	Eumenidae sp.	Buprestidae sp.	30	0.02	0.03	0.01	0.05
1	0	0.49	0	t	1	Eumenidae sp.	Phacelia cf. secunda Gmelin	30	0.02	0.03	0.01	0.02
0	1	0.55	0	t	1	Senecio subulatus Don ex Hook. &	Senecio filaginoides DC.	30	0.03	0.05	0.02	0.03
0	0	0.09	0	cv	1	Hemiptera sp2	Ocyptamus meridionalis	30	0.01	0.07	0.01	0.09
0	0	0.24	0	t	1	Coccinelidae sp1	Thelesperma megapotamicum (Spreng.) O. Kuntze	30	0.01	0.09	0.02	0.04
0	0	0.11	0	t	1	Muscidae sp1	cf. Entypus sp.	30	0.01	0.07	0.01	0.07
0	0	0.20	0	t	1	Thyridanthrax sp4	cf. Toxomerus sp.	30	0.02	0.09	0.01	0.03
0	0	0.16	0	cv	1	Thyridanthrax sp4	Callonychium mandibulare	30	0.02	0.09	0.01	0.05
0	0	0.33	0	t	1	Senecio subulatus Don ex Hook. &	Chalepogenus caeruleus	30	0.03	0.05	0.01	0.07
0	0	0.13	0	t	1	Adesmia retrofracta Hook. et Arn.	Ocyptamus meridionalis	30	0.01	0.05	0.01	0.09
1	0	0.23	0	t	1	Callonychium mandibulare	Hemiptera sp1	30	0.01	0.05	0.01	0.04
0	0	0.20	0	cv	1	cf. Toxomerus sp.	Muscidae sp3	30	0.01	0.03	0.01	0.07
1	0	0.09	0	t	1	Mordellidae sp.	Tephritidae sp.	30	0.01	0.09	0.01	0.07
0	0	0.23	0	t	1	Phacelia cf. secunda	Eriopis cf. conexa	30	0.01	0.02	0.01	0.07
0	1	0.94	0	t	1	Callonychium mandibulare	Dialictus sp2	30	0.01	0.05	0.12	0.10

0	0	0.50	0	cv	1	Lecanophora heterophylla (Cav.) Krapovickas	Anthidium rubriceps	30	0.01	0.03	0.02	0.02
1	1	0.52	0	t	1	Tatochila sp.	Senecio filaginoides DC.	30	0.02	0.02	0.02	0.03
0	0	0.32	0	t	1	Polygala stenophylla A. Gray	Opuntia corrugata S-D.	30	0.01	0.02	0.03	0.10
1	0	0.09	0	t	1	Ephydridae sp.	Tachinidae sp4	30	0.01	0.09	0.01	0.07
0	0	0.40	0	cv	1	Tachinidae sp4	Rosa rubiginosa L.	30	0.01	0.07	0.03	0.03
1	0	0.22	0	t	1	Buprestidae sp.	Eristalis bogotensis	30	0.01	0.05	0.01	0.04
0	1	0.52	0	t	1	Hysterionica jasionoides Willd.	Senecio filaginoides DC.	30	0.03	0.05	0.02	0.03
0	0	0.21	0	t	1	Lecanophora heterophylla (Cav.) Krapovickas	Cantharidae sp.	30	0.01	0.03	0.01	0.07
1	1	0.68	0	cv	1	Oxybelus sp.	Thelesperma megapotamicum (Spreng.) O. Kuntze	30	0.04	0.04	0.02	0.04
0	0	0.17	0	t	1	Bibionidae sp2	Megachile sp3	30	0.02	0.09	0.01	0.04
0	0	0.36	0	t	1	Apis mellifera	Oenothera mendocinensis Gilles ex Hook. et Arn.	30	0.02	0.05	0.01	0.02
1	0	0.49	0	t	1	Colletes lycii	Mutisia decurrens Cav.	30	0.01	0.03	0.02	0.02
0	0	0.15	0	cv	1	Coccinelidae sp2	Muscidae sp2	30	0.02	0.09	0.01	0.06
1	0	0.09	0	t	1	Geometridae sp.	Curculionidae sp.	30	0.01	0.09	0.01	0.07
0	0	0.21	0	t	1	Empididae sp.	Polygala stenophylla A. Gray	30	0.01	0.07	0.01	0.02
0	0	0.42	0	t	1	Eumenidae sp.	Carposcalis punctulata	30	0.02	0.03	0.02	0.09
0	0	0.16	0	cv	1	Bibionidae sp2	Callonychium mandibulare	30	0.02	0.09	0.01	0.05
0	0	0.20	0	t	1	Muscidae sp1	Apis mellifera	30	0.01	0.07	0.02	0.05
0	0	0.17	0	t	1	Prionyx sp.	Scaeva occidentalis	30	0.01	0.06	0.02	0.08

0	0	0.10	0	t	1	Tachinidae sp4	Mordellidae sp.	30	0.01	0.07	0.01	0.09
0	0	0.38	0	cv	1	Colletes lycii	Sphecidae sp.	30	0.01	0.03	0.02	0.05
0	0	0.16	0	t	1	Oxalis sp.	Carposcalis saltana	30	0.02	0.05	0.01	0.09
0	0	0.17	0	t	1	Tachinidae sp4	Hemiptera sp1	30	0.01	0.07	0.01	0.04
0	0	0.15	0	t	1	Arhysosage bifasciata	Muscidae sp1	30	0.02	0.07	0.01	0.07
0	0	0.43	0	cv	1	Mutisia decurrens Cav.	Megachile sp3	30	0.02	0.02	0.01	0.04
0	0	0.26	0	t	1	Psaenythia rufipes	Centris muralis	30	0.02	0.06	0.01	0.03
0	1	0.74	0	t	1	Anthidium rubriceps	cf. Microcerella sp.	30	0.02	0.02	0.05	0.08
0	0	0.31	0	t	1	Hemiptera sp1	Sarcophagidae sp.	30	0.01	0.04	0.03	0.08
0	1	0.67	0	cv	1	Epicauta sp.	Syrphidae sp3	30	0.01	0.03	0.06	0.08
0	0	0.21	0	t	1	Callonychium mandibulare	Buprestidae sp.	30	0.01	0.05	0.01	0.05
0	0	0.41	0	t	1	Oxybelus sp.	Cantharidae sp.	30	0.04	0.04	0.01	0.07
0	0	0.34	0	t	1	Coccinelidae sp3	Rosa rubiginosa L.	30	0.01	0.09	0.03	0.03
0	0	0.15	0	cv	1	Coccinelidae sp3	Centris muralis	30	0.01	0.09	0.01	0.03
0	0	0.45	0	t	1	Mordellidae sp.	Syrphidae sp3	30	0.01	0.09	0.06	0.08
0	0	0.30	0	t	1	Rapistrum rugosum (L.) Allioni	Grindelia chiloensis (Corn.) Cabrera	30	0.02	0.05	0.02	0.08
0	1	0.95	0	t	1	Tachinidae sp5	Discaria trinervis (Hook. et Arn.) Reiche	30	0.02	0.04	0.11	0.07
1	1	0.85	1	cv	1	24	Heteractis magnifica	31	0.00	0.06	0.09	0.04
0	0	0.33	1	t	1	25	Entacmaea quadricolor	31	0.00	0.05	0.03	0.04
1	1	0.67	1	t	1	26	Stichodactyla mertensii	31	0.01	0.06	0.06	0.04
1	1	0.87	1	t	1	26	Heteractis magnifica	31	0.01	0.06	0.09	0.04
1	1	0.57	1	cv	1	27	86	31	0.00	0.05	0.05	0.04
1	1	0.89	1	t	1	20	Heteractis magnifica	31	0.01	0.06	0.09	0.04
1	1	0.62	1	t	1	20	90	31	0.01	0.06	0.05	0.04
1	1	0.67	1	t	1	20	92	31	0.01	0.06	0.05	0.04

1	1	0.51	1	cv	1	21	81	31	0.01	0.06	0.04	0.04
1	1	0.83	1	t	1	21	100	31	0.01	0.06	0.07	0.04
1	1	0.90	1	t	1	21	Heteractis magnifica	31	0.01	0.06	0.09	0.04
1	0	0.39	1	t	1	21	97	31	0.01	0.06	0.03	0.04
1	1	0.85	1	cv	1	22	Heteractis magnifica	31	0.00	0.06	0.09	0.04
1	1	0.64	1	t	1	23	81	31	0.03	0.06	0.04	0.04
1	1	0.77	1	t	1	23	86	31	0.03	0.06	0.05	0.04
1	1	0.82	1	t	1	23	Stichodactyla mertensii	31	0.03	0.06	0.06	0.04
1	1	0.55	1	cv	1	23	Entacmaea quadricolor	31	0.03	0.06	0.03	0.04
1	1	0.89	1	t	1	23	100	31	0.03	0.06	0.07	0.04
1	1	0.62	1	t	1	23	Heteractis aurora	31	0.03	0.06	0.03	0.04
1	1	0.94	1	t	1	23	Heteractis magnifica	31	0.03	0.06	0.09	0.04
1	1	0.62	1	cv	1	23	91	31	0.03	0.06	0.03	0.04
1	1	0.80	1	t	1	23	92	31	0.03	0.06	0.05	0.04
1	0	0.48	1	t	1	28	81	31	0.01	0.06	0.04	0.04
1	1	0.81	1	t	1	28	100	31	0.01	0.06	0.07	0.04
1	0	0.41	1	cv	1	28	99	31	0.01	0.06	0.03	0.04
1	1	0.76	1	t	1	29	100	31	0.00	0.06	0.07	0.04
1	1	0.76	1	t	1	4	100	31	0.00	0.06	0.07	0.04
1	1	0.65	1	t	1	8	86	31	0.01	0.06	0.05	0.04
1	1	0.73	1	cv	1	8	Stichodactyla mertensii	31	0.01	0.06	0.06	0.04
1	1	0.90	1	t	1	8	Heteractis magnifica	31	0.01	0.06	0.09	0.04
1	1	0.70	1	t	1	8	92	31	0.01	0.06	0.05	0.04
1	1	0.85	1	t	1	59	Heteractis magnifica	31	0.00	0.06	0.09	0.04
1	1	0.52	1	cv	1	58	88	31	0.03	0.06	0.02	0.04
1	1	0.67	1	t	1	58	81	31	0.03	0.06	0.04	0.04
1	0	0.33	1	t	1	58	84	31	0.03	0.06	0.00	0.04
1	1	0.84	1	t	1	58	Stichodactyla mertensii	31	0.03	0.06	0.06	0.04
1	1	0.57	1	cv	1	58	Entacmaea quadricolor	31	0.03	0.06	0.03	0.04
1	1	0.90	1	t	1	58	100	31	0.03	0.06	0.07	0.04
1	1	0.95	1	t	1	58	Heteractis magnifica	31	0.03	0.06	0.09	0.04
1	1	0.74	1	t	1	58	98	31	0.03	0.06	0.04	0.04
1	1	0.78	1	cv	1	58	90	31	0.03	0.06	0.05	0.04
1	1	0.82	1	t	1	58	92	31	0.03	0.06	0.05	0.04
1	1	0.83	1	t	1	55	100	31	0.01	0.06	0.07	0.04
1	1	0.90	1	t	1	55	Heteractis magnifica	31	0.01	0.06	0.09	0.04
1	0	0.44	1	cv	1	55	99	31	0.01	0.06	0.03	0.04
1	1	0.65	1	t	1	55	90	31	0.01	0.06	0.05	0.04

1	1	0.85	1	t	1	54	Heteractis magnifica	31	0.00	0.06	0.09	0.04
1	1	0.67	1	t	1	57	88	31	0.04	0.06	0.02	0.04
1	1	0.55	1	cv	1	57	89	31	0.04	0.06	0.01	0.04
1	1	0.52	1	t	1	57	83	31	0.04	0.06	0.01	0.04
1	1	0.69	1	t	1	57	80	31	0.04	0.06	0.03	0.04
1	1	0.79	1	t	1	57	81	31	0.04	0.06	0.04	0.04
1	1	0.87	1	cv	1	57	86	31	0.04	0.06	0.05	0.04
1	1	0.74	1	t	1	57	85	31	0.04	0.06	0.03	0.04
1	1	0.91	1	t	1	57	Stichodactyla mertensii	31	0.04	0.06	0.06	0.04
1	1	0.95	1	t	1	57	100	31	0.04	0.06	0.07	0.04
1	1	0.77	1	cv	1	57	Heteractis aurora	31	0.04	0.06	0.03	0.04
1	1	0.97	1	t	1	57	Heteractis magnifica	31	0.04	0.06	0.09	0.04
1	1	0.74	1	t	1	57	99	31	0.04	0.06	0.03	0.04
1	1	0.84	1	t	1	57	98	31	0.04	0.06	0.04	0.04
1	1	0.87	1	cv	1	57	90	31	0.04	0.06	0.05	0.04
1	1	0.89	1	t	1	57	92	31	0.04	0.06	0.05	0.04
1	1	0.59	1	t	1	57	94	31	0.04	0.06	0.01	0.04
1	1	0.69	1	t	1	57	97	31	0.04	0.06	0.03	0.04
1	1	0.61	1	cv	1	56	92	31	0.00	0.06	0.05	0.04
1	0	0.36	1	t	1	51	80	31	0.01	0.06	0.03	0.04
1	0	0.47	1	t	1	51	81	31	0.01	0.06	0.04	0.04
1	1	0.89	1	t	1	51	Heteractis magnifica	31	0.01	0.06	0.09	0.04
1	1	0.70	1	cv	1	50	Stichodactyla mertensii	31	0.01	0.06	0.06	0.04
1	1	0.89	1	t	1	50	Heteractis magnifica	31	0.01	0.06	0.09	0.04
1	0	0.45	1	t	1	50	91	31	0.01	0.06	0.03	0.04
1	0	0.35	1	t	1	53	80	31	0.01	0.06	0.03	0.04
1	1	0.89	1	cv	1	53	Heteractis magnifica	31	0.01	0.06	0.09	0.04
1	0	0.22	1	t	1	53	93	31	0.01	0.06	0.01	0.04
1	0	0.25	1	t	1	52	87	31	0.01	0.06	0.01	0.04
1	1	0.81	1	t	1	52	100	31	0.01	0.06	0.07	0.04
1	1	0.89	1	cv	1	52	Heteractis magnifica	31	0.01	0.06	0.09	0.04
1	1	0.63	1	t	1	88	31	31	0.02	0.04	0.04	0.06
1	1	0.71	1	t	1	88	34	31	0.02	0.04	0.05	0.06
1	1	0.51	1	t	1	88	48	31	0.02	0.04	0.03	0.06
1	1	0.63	1	cv	1	88	43	31	0.02	0.04	0.04	0.06
1	1	0.59	1	t	1	88	72	31	0.02	0.04	0.03	0.06
1	1	0.52	1	t	1	89	43	31	0.01	0.04	0.04	0.06
1	0	0.47	1	t	1	89	72	31	0.01	0.04	0.03	0.06
0	0	0.29	1	cv	1	82	64	31	0.01	0.03	0.02	0.05
0	0	0.24	1	t	1	82	73	31	0.01	0.03	0.01	0.05
1	0	0.44	1	t	1	83	72	31	0.01	0.04	0.03	0.06
1	1	0.65	1	t	1	80	31	31	0.03	0.04	0.04	0.06

0	0	0.40	1	cv	1	80	35	31	0.03	0.04	0.01	0.06
1	1	0.73	1	t	1	80	34	31	0.03	0.04	0.05	0.06
1	1	0.54	1	t	1	80	48	31	0.03	0.04	0.03	0.06
1	1	0.65	1	t	1	80	43	31	0.03	0.04	0.04	0.06
1	0	0.44	1	cv	1	81	7	31	0.04	0.04	0.01	0.06
1	1	0.75	1	t	1	81	31	31	0.04	0.04	0.04	0.06
1	1	0.81	1	t	1	81	34	31	0.04	0.04	0.05	0.06
1	1	0.60	1	t	1	81	45	31	0.04	0.04	0.02	0.06
1	1	0.57	1	cv	1	81	42	31	0.04	0.04	0.02	0.06
0	0	0.45	1	t	1	81	75	31	0.04	0.04	0.01	0.05
1	1	0.71	1	t	1	81	72	31	0.04	0.04	0.03	0.06
1	1	0.84	1	t	1	86	31	31	0.05	0.04	0.04	0.06
1	1	0.88	1	cv	1	86	34	31	0.05	0.04	0.05	0.06
1	1	0.64	1	t	1	86	62	31	0.05	0.04	0.01	0.06
1	1	0.68	1	t	1	86	64	31	0.05	0.04	0.02	0.05
1	1	0.59	1	t	1	86	65	31	0.05	0.04	0.01	0.05
1	1	0.59	1	cv	1	86	66	31	0.05	0.04	0.01	0.05
0	1	0.55	1	t	1	86	67	31	0.05	0.04	0.00	0.05
1	1	0.58	1	t	1	86	14	31	0.05	0.04	0.01	0.05
1	1	0.74	1	t	1	86	18	31	0.05	0.04	0.03	0.06
1	1	0.65	1	cv	1	86	46	31	0.05	0.04	0.01	0.06
1	1	0.72	1	t	1	86	45	31	0.05	0.04	0.02	0.06
1	1	0.84	1	t	1	86	43	31	0.05	0.04	0.04	0.06
1	1	0.62	1	t	1	86	74	31	0.05	0.04	0.01	0.05
0	1	0.63	1	cv	1	86	73	31	0.05	0.04	0.01	0.05
1	1	0.81	1	t	1	86	72	31	0.05	0.04	0.03	0.06
1	1	0.62	1	t	1	86	71	31	0.05	0.04	0.01	0.05
1	1	0.54	1	t	1	87	31	31	0.01	0.04	0.04	0.06
1	0	0.39	1	cv	1	87	68	31	0.01	0.04	0.03	0.06
1	0	0.29	1	t	1	87	46	31	0.01	0.04	0.01	0.06
0	0	0.38	1	t	1	85	3	31	0.03	0.04	0.01	0.06
1	1	0.70	1	t	1	85	31	31	0.03	0.04	0.04	0.06
0	0	0.44	1	cv	1	85	35	31	0.03	0.04	0.01	0.06
1	1	0.76	1	t	1	85	34	31	0.03	0.04	0.05	0.06
0	0	0.38	1	t	1	85	6	31	0.03	0.04	0.01	0.05
1	1	0.58	1	t	1	85	48	31	0.03	0.04	0.03	0.06
1	1	0.70	1	cv	1	85	43	31	0.03	0.04	0.04	0.06
0	0	0.38	1	t	1	85	75	31	0.03	0.04	0.01	0.05
1	1	0.66	1	t	1	85	72	31	0.03	0.04	0.03	0.06
1	1	0.79	1	t	1	3	100	31	0.01	0.06	0.07	0.04
1	1	0.79	1	cv	1	7	100	31	0.01	0.06	0.07	0.04
1	1	0.80	1	t	1	Cryptodendrum adhaesivum	33	31	0.06	0.04	0.03	0.06
1	1	0.88	1	t	1	Cryptodendrum adhaesivum	31	31	0.06	0.04	0.04	0.06
1	1	0.91	1	t	1	Cryptodendrum adhaesivum	34	31	0.06	0.04	0.05	0.06
1	1	0.65	1	cv	1	Cryptodendrum adhaesivum	60	31	0.06	0.04	0.01	0.06
1	1	0.80	1	t	1	Cryptodendrum adhaesivum	68	31	0.06	0.04	0.03	0.06

1	1	0.62	1	t	1	Cryptodendrum adhaesivum	69	31	0.06	0.04	0.00	0.06
1	1	0.66	1	t	1	Cryptodendrum adhaesivum	6	31	0.06	0.04	0.01	0.05
1	1	0.68	1	cv	1	Cryptodendrum adhaesivum	13	31	0.06	0.04	0.01	0.06
1	1	0.76	1	t	1	Cryptodendrum adhaesivum	15	31	0.06	0.04	0.02	0.06
1	1	0.66	1	t	1	Cryptodendrum adhaesivum	14	31	0.06	0.04	0.01	0.05
1	1	0.68	1	t	1	Cryptodendrum adhaesivum	16	31	0.06	0.04	0.01	0.06
1	1	0.74	1	cv	1	Cryptodendrum adhaesivum	19	31	0.06	0.04	0.02	0.06
1	1	0.82	1	t	1	Cryptodendrum adhaesivum	48	31	0.06	0.04	0.03	0.06
1	1	0.78	1	t	1	Cryptodendrum adhaesivum	49	31	0.06	0.04	0.02	0.06
1	1	0.72	1	t	1	Cryptodendrum adhaesivum	46	31	0.06	0.04	0.01	0.06
1	1	0.74	1	cv	1	Cryptodendrum adhaesivum	44	31	0.06	0.04	0.02	0.06
1	1	0.88	1	t	1	Cryptodendrum adhaesivum	43	31	0.06	0.04	0.04	0.06
1	1	0.69	1	t	1	Cryptodendrum adhaesivum	71	31	0.06	0.04	0.01	0.05
1	1	0.78	1	t	1	Cryptodendrum adhaesivum	70	31	0.06	0.04	0.02	0.06
0	0	0.32	1	cv	1	Premnas biaculeatus	37	31	0.03	0.04	0.00	0.05
1	1	0.74	1	t	1	Premnas biaculeatus	34	31	0.03	0.04	0.05	0.06
1	1	0.53	1	t	1	Premnas biaculeatus	68	31	0.03	0.04	0.03	0.06
1	0	0.47	1	t	1	Premnas biaculeatus	42	31	0.03	0.04	0.02	0.06
1	0	0.41	1	cv	1	Premnas biaculeatus	40	31	0.03	0.04	0.01	0.06
1	1	0.50	1	t	1	Premnas biaculeatus	70	31	0.03	0.04	0.02	0.06
1	1	0.87	1	t	1	100	33	31	0.07	0.04	0.03	0.06
1	1	0.93	1	t	1	100	31	31	0.07	0.04	0.04	0.06
1	1	0.82	1	cv	1	100	35	31	0.07	0.04	0.01	0.06
1	1	0.95	1	t	1	100	34	31	0.07	0.04	0.05	0.06
1	1	0.87	1	t	1	100	68	31	0.07	0.04	0.03	0.06
1	1	0.73	1	t	1	100	2	31	0.07	0.04	0.00	0.06
1	1	0.76	1	cv	1	100	12	31	0.07	0.04	0.01	0.06
1	1	0.85	1	t	1	100	15	31	0.07	0.04	0.02	0.06
1	1	0.79	1	t	1	100	17	31	0.07	0.04	0.01	0.06
1	1	0.88	1	t	1	100	18	31	0.07	0.04	0.03	0.06
1	1	0.86	1	cv	1	100	49	31	0.07	0.04	0.02	0.06

1	1	0.82	1	t	1	100	46	31	0.07	0.04	0.01	0.06
1	1	0.83	1	t	1	100	44	31	0.07	0.04	0.02	0.06
1	1	0.86	1	t	1	100	45	31	0.07	0.04	0.02	0.06
1	1	0.85	1	cv	1	100	42	31	0.07	0.04	0.02	0.06
1	1	0.93	1	t	1	100	43	31	0.07	0.04	0.04	0.06
1	1	0.81	1	t	1	100	40	31	0.07	0.04	0.01	0.06
1	1	0.82	1	t	1	100	1	31	0.07	0.04	0.01	0.06
1	1	0.73	1	cv	1	100	5	31	0.07	0.04	0.00	0.06
1	1	0.76	1	t	1	100	76	31	0.07	0.04	0.01	0.06
1	1	0.91	1	t	1	100	72	31	0.07	0.04	0.03	0.06
1	1	0.86	1	t	1	100	70	31	0.07	0.04	0.02	0.06
1	1	0.79	1	cv	1	100	79	31	0.07	0.04	0.01	0.06
1	1	0.60	1	t	1	Amphiprion bicinctus	33	31	0.03	0.04	0.03	0.06
1	0	0.49	1	t	1	Amphiprion bicinctus	35	31	0.03	0.04	0.01	0.06
1	1	0.80	1	t	1	Amphiprion bicinctus	34	31	0.03	0.04	0.05	0.06
1	1	0.60	1	cv	1	Amphiprion bicinctus	68	31	0.03	0.04	0.03	0.06
1	1	0.54	1	t	1	Amphiprion bicinctus	15	31	0.03	0.04	0.02	0.06
1	1	0.60	1	t	1	Amphiprion bicinctus	18	31	0.03	0.04	0.03	0.06
1	1	0.63	1	t	1	Amphiprion bicinctus	48	31	0.03	0.04	0.03	0.06
1	1	0.57	1	cv	1	Amphiprion bicinctus	49	31	0.03	0.04	0.02	0.06
1	1	0.70	1	t	1	Amphiprion bicinctus	72	31	0.03	0.04	0.03	0.06
1	1	0.57	1	t	1	Amphiprion bicinctus	70	31	0.03	0.04	0.02	0.06
1	1	0.83	1	t	1	Stichodactyla haddoni	39	31	0.09	0.04	0.00	0.06
1	1	0.83	1	cv	1	Stichodactyla haddoni	38	31	0.09	0.04	0.00	0.06
1	1	0.93	1	t	1	Stichodactyla haddoni	33	31	0.09	0.04	0.03	0.06
1	1	0.83	1	t	1	Stichodactyla haddoni	32	31	0.09	0.04	0.00	0.06
1	1	0.96	1	t	1	Stichodactyla haddoni	31	31	0.09	0.04	0.04	0.06
1	1	0.85	1	cv	1	Stichodactyla haddoni	30	31	0.09	0.04	0.01	0.06
1	1	0.97	1	t	1	Stichodactyla haddoni	34	31	0.09	0.04	0.05	0.06
1	1	0.85	1	t	1	Stichodactyla haddoni	60	31	0.09	0.04	0.01	0.06
1	1	0.83	1	t	1	Stichodactyla haddoni	61	31	0.09	0.04	0.00	0.06

1	1	0.89	1	cv	1	Stichodactyla haddoni	62	31	0.09	0.04	0.01	0.06
1	1	0.93	1	t	1	Stichodactyla haddoni	68	31	0.09	0.04	0.03	0.06
1	1	0.83	1	t	1	Stichodactyla haddoni	10	31	0.09	0.04	0.00	0.06
1	1	0.87	1	t	1	Stichodactyla haddoni	13	31	0.09	0.04	0.01	0.06
1	1	0.85	1	cv	1	Stichodactyla haddoni	12	31	0.09	0.04	0.01	0.06
1	1	0.91	1	t	1	Stichodactyla haddoni	15	31	0.09	0.04	0.02	0.06
1	1	0.87	1	t	1	Stichodactyla haddoni	17	31	0.09	0.04	0.01	0.06
1	1	0.87	1	t	1	Stichodactyla haddoni	16	31	0.09	0.04	0.01	0.06
1	1	0.90	1	cv	1	Stichodactyla haddoni	19	31	0.09	0.04	0.02	0.06
1	1	0.93	1	t	1	Stichodactyla haddoni	18	31	0.09	0.04	0.03	0.06
1	1	0.93	1	t	1	Stichodactyla haddoni	48	31	0.09	0.04	0.03	0.06
1	1	0.92	1	t	1	Stichodactyla haddoni	49	31	0.09	0.04	0.02	0.06
1	1	0.92	1	cv	1	Stichodactyla haddoni	45	31	0.09	0.04	0.02	0.06
1	1	0.91	1	t	1	Stichodactyla haddoni	42	31	0.09	0.04	0.02	0.06
1	1	0.96	1	t	1	Stichodactyla haddoni	43	31	0.09	0.04	0.04	0.06
1	1	0.89	1	t	1	Stichodactyla haddoni	40	31	0.09	0.04	0.01	0.06
1	1	0.83	1	cv	1	Stichodactyla haddoni	41	31	0.09	0.04	0.00	0.06
1	1	0.86	1	t	1	Stichodactyla haddoni	77	31	0.09	0.04	0.01	0.06
1	1	0.85	1	t	1	Stichodactyla haddoni	76	31	0.09	0.04	0.01	0.06
1	1	0.95	1	t	1	Stichodactyla haddoni	72	31	0.09	0.04	0.03	0.06
1	1	0.92	1	cv	1	Stichodactyla haddoni	70	31	0.09	0.04	0.02	0.06
1	1	0.55	1	t	1	33	99	31	0.03	0.06	0.03	0.04
1	1	0.60	1	t	1	33	91	31	0.03	0.06	0.03	0.04
1	0	0.50	1	t	1	33	97	31	0.03	0.06	0.03	0.04
1	0	0.38	1	cv	1	33	96	31	0.03	0.06	0.01	0.04
1	1	0.68	1	t	1	31	99	31	0.04	0.06	0.03	0.04
1	1	0.81	1	t	1	31	98	31	0.04	0.06	0.04	0.04
1	1	0.72	1	t	1	31	91	31	0.04	0.06	0.03	0.04
1	1	0.84	1	cv	1	31	90	31	0.04	0.06	0.05	0.04
1	1	0.87	1	t	1	31	92	31	0.04	0.06	0.05	0.04

1	0	0.41	1	t	1	30	91	31	0.01	0.06	0.03	0.04
1	1	0.61	1	t	1	36	92	31	0.00	0.06	0.05	0.04
1	1	0.75	1	cv	1	34	99	31	0.05	0.06	0.03	0.04
1	1	0.85	1	t	1	34	98	31	0.05	0.06	0.04	0.04
1	1	0.78	1	t	1	34	91	31	0.05	0.06	0.03	0.04
1	1	0.88	1	t	1	34	90	31	0.05	0.06	0.05	0.04
1	1	0.90	1	cv	1	34	92	31	0.05	0.06	0.05	0.04
1	1	0.61	1	t	1	34	94	31	0.05	0.06	0.01	0.04
1	1	0.71	1	t	1	34	97	31	0.05	0.06	0.03	0.04
1	1	0.60	1	t	1	34	96	31	0.05	0.06	0.01	0.04
1	0	0.44	1	cv	1	62	99	31	0.01	0.06	0.03	0.04
1	1	0.60	1	t	1	62	98	31	0.01	0.06	0.04	0.04
1	1	0.55	1	t	1	63	98	31	0.01	0.05	0.04	0.04
1	1	0.60	1	t	1	63	90	31	0.01	0.05	0.05	0.04
1	1	0.64	1	cv	1	64	98	31	0.02	0.05	0.04	0.04
1	1	0.70	1	t	1	64	90	31	0.02	0.05	0.05	0.04
1	0	0.27	1	t	1	64	95	31	0.02	0.05	0.01	0.04
1	1	0.60	1	t	1	65	90	31	0.01	0.05	0.05	0.04
1	1	0.60	1	cv	1	66	90	31	0.01	0.05	0.05	0.04
1	1	0.79	1	t	1	68	92	31	0.03	0.06	0.05	0.04
1	0	0.38	1	t	1	68	96	31	0.03	0.06	0.01	0.04
1	0	0.47	1	t	1	99	19	31	0.03	0.04	0.02	0.06
1	1	0.53	1	cv	1	99	49	31	0.03	0.04	0.02	0.06
1	1	0.66	1	t	1	99	72	31	0.03	0.04	0.03	0.06
1	1	0.70	1	t	1	98	18	31	0.04	0.04	0.03	0.06
1	1	0.67	1	t	1	98	49	31	0.04	0.04	0.02	0.06
1	1	0.62	1	cv	1	98	44	31	0.04	0.04	0.02	0.06
1	1	0.67	1	t	1	98	45	31	0.04	0.04	0.02	0.06
1	1	0.81	1	t	1	98	43	31	0.04	0.04	0.04	0.06
1	1	0.59	1	t	1	98	1	31	0.04	0.04	0.01	0.06
0	0	0.49	1	cv	1	98	9	31	0.04	0.04	0.00	0.05
0	1	0.57	1	t	1	98	73	31	0.04	0.04	0.01	0.05
1	1	0.78	1	t	1	98	72	31	0.04	0.04	0.03	0.06
0	0	0.49	1	t	1	98	78	31	0.04	0.04	0.00	0.05
1	0	0.44	1	cv	1	91	16	31	0.03	0.04	0.01	0.06
1	1	0.51	1	t	1	91	19	31	0.03	0.04	0.02	0.06
1	1	0.60	1	t	1	91	18	31	0.03	0.04	0.03	0.06
1	1	0.63	1	t	1	91	48	31	0.03	0.04	0.03	0.06
1	1	0.73	1	cv	1	91	43	31	0.03	0.04	0.04	0.06
1	1	0.57	1	t	1	91	70	31	0.03	0.04	0.02	0.06
1	1	0.61	1	t	1	90	17	31	0.05	0.04	0.01	0.06
1	1	0.74	1	t	1	90	18	31	0.05	0.04	0.03	0.06
1	1	0.67	1	cv	1	90	44	31	0.05	0.04	0.02	0.06
1	1	0.72	1	t	1	90	45	31	0.05	0.04	0.02	0.06
1	1	0.70	1	t	1	90	42	31	0.05	0.04	0.02	0.06
1	1	0.65	1	t	1	90	1	31	0.05	0.04	0.01	0.06
1	1	0.57	1	cv	1	90	77	31	0.05	0.04	0.01	0.06
1	1	0.62	1	t	1	90	74	31	0.05	0.04	0.01	0.05
1	1	0.72	1	t	1	90	70	31	0.05	0.04	0.02	0.06
1	1	0.61	1	t	1	90	79	31	0.05	0.04	0.01	0.06
1	0	0.39	1	cv	1	93	48	31	0.01	0.04	0.03	0.06

1	0	0.33	1	t	1	93	49	31	0.01	0.04	0.02	0.06
1	1	0.59	1	t	1	92	11	31	0.05	0.04	0.00	0.06
1	1	0.65	1	t	1	92	13	31	0.05	0.04	0.01	0.06
1	1	0.71	1	cv	1	92	19	31	0.05	0.04	0.02	0.06
1	1	0.78	1	t	1	92	18	31	0.05	0.04	0.03	0.06
1	1	0.59	1	t	1	92	47	31	0.05	0.04	0.00	0.06
1	1	0.72	1	t	1	92	44	31	0.05	0.04	0.02	0.06
1	1	0.76	1	cv	1	92	45	31	0.05	0.04	0.02	0.06
1	1	0.74	1	t	1	92	42	31	0.05	0.04	0.02	0.06
1	1	0.87	1	t	1	92	43	31	0.05	0.04	0.04	0.06
1	1	0.68	1	t	1	92	40	31	0.05	0.04	0.01	0.06
1	1	0.67	1	cv	1	92	74	31	0.05	0.04	0.01	0.05
1	1	0.66	1	t	1	92	71	31	0.05	0.04	0.01	0.05
1	1	0.66	1	t	1	92	79	31	0.05	0.04	0.01	0.06
1	0	0.29	1	t	1	95	15	31	0.01	0.04	0.02	0.06
1	1	0.55	1	cv	1	94	43	31	0.01	0.04	0.04	0.06
0	0	0.30	1	t	1	94	1	31	0.01	0.04	0.01	0.06
1	0	0.45	1	t	1	97	15	31	0.03	0.04	0.02	0.06
1	1	0.53	1	t	1	97	48	31	0.03	0.04	0.03	0.06
1	1	0.65	1	cv	1	97	43	31	0.03	0.04	0.04	0.06
1	1	0.61	1	t	1	97	72	31	0.03	0.04	0.03	0.06
1	1	0.55	1	t	1	96	43	31	0.01	0.04	0.04	0.06
1	0	0.36	0	t	1	76	72	31	0.01	0.06	0.03	0.06
0	1	0.92	0	cv	1	98	92	31	0.04	0.04	0.05	0.04
0	0	0.30	0	t	1	51	68	31	0.01	0.06	0.03	0.06
1	0	0.29	0	t	1	6	18	31	0.01	0.05	0.03	0.06
1	1	0.53	0	t	1	30	98	31	0.01	0.06	0.04	0.04
0	0	0.15	0	cv	1	52	9	31	0.01	0.06	0.00	0.05
0	0	0.21	0	t	1	20	21	31	0.01	0.06	0.01	0.06
0	0	0.26	0	t	1	21	19	31	0.01	0.06	0.02	0.06
1	0	0.37	0	t	1	26	99	31	0.01	0.06	0.03	0.04
0	0	0.21	0	cv	1	82	66	31	0.01	0.03	0.01	0.05
0	1	0.76	0	t	1	81	Heteractis aurora	31	0.04	0.04	0.03	0.04
1	0	0.31	0	t	1	65	48	31	0.01	0.05	0.03	0.06
0	0	0.19	0	t	1	50	71	31	0.01	0.06	0.01	0.05
0	0	0.37	0	cv	1	94	49	31	0.01	0.04	0.02	0.06
1	1	0.62	0	t	1	92	77	31	0.05	0.04	0.01	0.06
0	0	0.21	0	t	1	8	52	31	0.01	0.06	0.01	0.06
0	0	0.15	0	t	1	28	29	31	0.01	0.06	0.00	0.06
1	0	0.12	0	cv	1	25	36	31	0.00	0.05	0.00	0.06
0	0	0.18	0	t	1	77	73	31	0.01	0.06	0.01	0.05
0	0	0.27	0	t	1	11	48	31	0.00	0.06	0.03	0.06
1	0	0.26	0	t	1	20	96	31	0.01	0.06	0.01	0.04
1	1	0.65	0	cv	1	Cryptodendrum adhaesivum	76	31	0.06	0.04	0.01	0.06
1	0	0.44	0	t	1	85	1	31	0.03	0.04	0.01	0.06
1	0	0.38	0	t	1	29	91	31	0.00	0.06	0.03	0.04
1	0	0.40	0	t	1	51	99	31	0.01	0.06	0.03	0.04
1	0	0.22	0	cv	1	55	73	31	0.01	0.06	0.01	0.05
0	0	0.26	0	t	1	13	15	31	0.01	0.06	0.02	0.06

0	1	0.94	0	t	1	Cryptodendrum adhaesivum	90	31	0.06	0.04	0.05	0.04
1	1	0.50	0	t	1	81	62	31	0.04	0.04	0.01	0.06
0	0	0.17	0	cv	1	4	55	31	0.00	0.06	0.01	0.06
1	1	0.51	0	t	1	44	43	31	0.02	0.06	0.04	0.06
1	1	0.62	0	t	1	50	90	31	0.01	0.06	0.05	0.04
1	1	0.53	0	t	1	99	70	31	0.03	0.04	0.02	0.06
0	1	0.58	0	cv	1	82	34	31	0.01	0.03	0.05	0.06
1	0	0.21	0	t	1	87	2	31	0.01	0.04	0.00	0.06
1	1	0.86	0	t	1	56	Heteractis magnifica	31	0.00	0.06	0.09	0.04
0	0	0.39	0	t	1	37	91	31	0.00	0.05	0.03	0.04
0	0	0.17	0	cv	1	62	11	31	0.01	0.06	0.00	0.06
1	0	0.33	0	t	1	85	5	31	0.03	0.04	0.00	0.06
0	0	0.17	0	t	1	26	71	31	0.01	0.06	0.01	0.05
0	0	0.13	0	t	1	22	63	31	0.00	0.06	0.01	0.05
0	1	0.67	0	cv	1	82	86	31	0.01	0.03	0.05	0.04
1	1	0.56	0	t	1	53	98	31	0.01	0.06	0.04	0.04
1	1	0.81	0	t	1	90	72	31	0.05	0.04	0.03	0.06
0	0	0.26	0	t	1	10	48	31	0.00	0.06	0.03	0.06
1	1	0.58	0	cv	1	31	68	31	0.04	0.06	0.03	0.06
1	0	0.21	0	t	1	93	76	31	0.01	0.04	0.01	0.06
0	0	0.14	0	t	1	6	9	31	0.01	0.05	0.00	0.05
1	0	0.36	0	t	1	91	10	31	0.03	0.04	0.00	0.06
0	0	0.15	0	cv	1	4	52	31	0.00	0.06	0.01	0.06
0	0	0.16	0	t	1	8	54	31	0.01	0.06	0.00	0.06
1	0	0.16	0	t	1	22	83	31	0.00	0.06	0.01	0.04
1	0	0.34	0	t	1	29	85	31	0.00	0.06	0.03	0.04
0	0	0.17	0	cv	1	22	1	31	0.00	0.06	0.01	0.06
0	0	0.27	0	t	1	89	62	31	0.01	0.04	0.01	0.06
0	0	0.17	0	t	1	50	3	31	0.01	0.06	0.01	0.06
0	0	0.15	0	t	1	10	74	31	0.00	0.06	0.01	0.05
0	0	0.15	0	cv	1	7	76	31	0.01	0.06	0.01	0.06
1	0	0.26	0	t	1	45	75	31	0.02	0.06	0.01	0.05
1	0	0.33	0	t	1	18	1	31	0.03	0.06	0.01	0.06
0	0	0.11	0	t	1	56	39	31	0.00	0.06	0.00	0.06
0	0	0.23	0	cv	1	17	19	31	0.01	0.06	0.02	0.06
1	0	0.21	0	t	1	3	93	31	0.01	0.06	0.01	0.04
0	0	0.21	0	t	1	89	76	31	0.01	0.04	0.01	0.06
1	1	0.62	0	t	1	50	86	31	0.01	0.06	0.05	0.04
1	0	0.48	0	cv	1	97	70	31	0.03	0.04	0.02	0.06
1	0	0.21	0	t	1	93	77	31	0.01	0.04	0.01	0.06
0	0	0.28	0	t	1	20	45	31	0.01	0.06	0.02	0.06
1	0	0.41	0	t	1	Amphiprion bicinctus	76	31	0.03	0.04	0.01	0.06
0	1	0.61	0	cv	1	99	97	31	0.03	0.04	0.03	0.04
1	1	0.52	0	t	1	86	32	31	0.05	0.04	0.00	0.06
1	0	0.50	0	t	1	82	31	31	0.01	0.03	0.04	0.06
0	0	0.37	0	t	1	80	74	31	0.03	0.04	0.01	0.05
1	0	0.19	0	cv	1	25	19	31	0.00	0.05	0.02	0.06
0	0	0.26	0	t	1	94	16	31	0.01	0.04	0.01	0.06

1	0	0.36	0	t	1	27	85	31	0.00	0.05	0.03	0.04
1	0	0.18	0	t	1	25	1	31	0.00	0.05	0.01	0.06
0	0	0.12	0	cv	1	38	78	31	0.00	0.06	0.00	0.05
1	1	0.57	0	t	1	67	90	31	0.00	0.05	0.05	0.04
1	1	0.54	0	t	1	22	86	31	0.00	0.06	0.05	0.04
1	0	0.50	0	t	1	68	97	31	0.03	0.06	0.03	0.04
1	0	0.40	0	cv	1	53	85	31	0.01	0.06	0.03	0.04
0	1	0.85	0	t	1	99	92	31	0.03	0.04	0.05	0.04
1	0	0.45	0	t	1	20	91	31	0.01	0.06	0.03	0.04
1	0	0.14	0	t	1	11	75	31	0.00	0.06	0.01	0.05
1	1	0.73	0	cv	1	Amphiprion bicinctus	43	31	0.03	0.04	0.04	0.06
0	1	0.86	0	t	1	25	Heteractis magnifica	31	0.00	0.05	0.09	0.04
0	0	0.13	0	t	1	27	67	31	0.00	0.05	0.00	0.05
0	0	0.14	0	t	1	52	61	31	0.01	0.06	0.00	0.06
0	0	0.11	0	cv	1	10	41	31	0.00	0.06	0.00	0.06
1	0	0.23	0	t	1	64	75	31	0.02	0.05	0.01	0.05
0	0	0.48	0	t	1	89	85	31	0.01	0.04	0.03	0.04
1	0	0.18	0	t	1	53	84	31	0.01	0.06	0.00	0.04
1	0	0.35	0	cv	1	Premnas biaculeatus	14	31	0.03	0.04	0.01	0.05
0	0	0.15	0	t	1	5	71	31	0.00	0.06	0.01	0.05
1	0	0.12	0	t	1	25	37	31	0.00	0.05	0.00	0.05
0	0	0.17	0	t	1	26	28	31	0.01	0.06	0.01	0.06
1	0	0.37	0	cv	1	30	99	31	0.01	0.06	0.03	0.04
1	0	0.45	0	t	1	27	57	31	0.00	0.05	0.04	0.06
1	0	0.35	0	t	1	64	49	31	0.02	0.05	0.02	0.06
0	0	0.22	0	t	1	89	63	31	0.01	0.04	0.01	0.05
0	1	0.85	0	cv	1	Cryptodendrum adhaesivum	Entacmaea quadricolor	31	0.06	0.04	0.03	0.04
0	0	0.33	0	t	1	20	48	31	0.01	0.06	0.03	0.06
0	0	0.13	0	t	1	22	60	31	0.00	0.06	0.01	0.06
1	0	0.32	0	t	1	29	Entacmaea quadricolor	31	0.00	0.06	0.03	0.04
0	1	0.69	0	cv	1	89	86	31	0.01	0.04	0.05	0.04
0	1	0.51	0	t	1	37	98	31	0.00	0.05	0.04	0.04
1	0	0.40	0	t	1	88	44	31	0.02	0.04	0.02	0.06
1	0	0.19	0	t	1	67	93	31	0.00	0.05	0.01	0.04
1	0	0.48	0	cv	1	34	63	31	0.05	0.06	0.01	0.05
0	0	0.18	0	t	1	77	74	31	0.01	0.06	0.01	0.05
0	0	0.21	0	t	1	16	40	31	0.01	0.06	0.01	0.06
0	0	0.28	0	t	1	23	76	31	0.03	0.06	0.01	0.06
0	0	0.38	0	cv	1	82	48	31	0.01	0.03	0.03	0.06
0	0	0.16	0	t	1	62	10	31	0.01	0.06	0.00	0.06
1	0	0.38	0	t	1	53	Entacmaea quadricolor	31	0.01	0.06	0.03	0.04
1	0	0.21	0	t	1	53	82	31	0.01	0.06	0.01	0.03
0	0	0.12	0	cv	1	56	2	31	0.00	0.06	0.00	0.06
1	0	0.16	0	t	1	26	84	31	0.01	0.06	0.00	0.04
1	0	0.28	0	t	1	58	69	31	0.03	0.06	0.00	0.06

0	0	0.21	0	t	1	21	52	31	0.01	0.06	0.01	0.06
0	0	0.14	0	cv	1	7	47	31	0.01	0.06	0.00	0.06
0	0	0.22	0	t	1	96	9	31	0.01	0.04	0.00	0.05
0	0	0.15	0	t	1	54	74	31	0.00	0.06	0.01	0.05
1	0	0.15	0	t	1	27	84	31	0.00	0.05	0.00	0.04
0	1	0.97	0	cv	1	86	100	31	0.05	0.04	0.07	0.04
1	1	0.55	0	t	1	58	80	31	0.03	0.06	0.03	0.04
1	1	0.55	0	t	1	4	90	31	0.00	0.06	0.05	0.04
1	1	0.72	0	t	1	86	49	31	0.05	0.04	0.02	0.06
1	0	0.38	0	cv	1	23	89	31	0.03	0.06	0.01	0.04
0	0	0.20	0	t	1	83	7	31	0.01	0.04	0.01	0.06
1	1	0.54	0	t	1	21	57	31	0.01	0.06	0.04	0.06
1	0	0.40	0	t	1	50	72	31	0.01	0.06	0.03	0.06
0	1	0.86	0	cv	1	81	86	31	0.04	0.04	0.05	0.04
0	0	0.12	0	t	1	54	78	31	0.00	0.06	0.00	0.05
1	0	0.34	0	t	1	89	49	31	0.01	0.04	0.02	0.06
0	0	0.14	0	t	1	29	63	31	0.00	0.06	0.01	0.05
0	0	0.14	0	cv	1	56	63	31	0.00	0.06	0.01	0.05
0	0	0.16	0	t	1	21	39	31	0.01	0.06	0.00	0.06
0	0	0.16	0	t	1	69	71	31	0.00	0.06	0.01	0.05
0	0	0.17	0	t	1	17	76	31	0.01	0.06	0.01	0.06
0	0	0.12	0	cv	1	30	10	31	0.01	0.06	0.00	0.06
1	0	0.49	0	t	1	15	72	31	0.02	0.06	0.03	0.06
1	0	0.25	0	t	1	28	64	31	0.01	0.06	0.02	0.05
0	0	0.13	0	t	1	38	66	31	0.00	0.06	0.01	0.05
1	1	0.50	0	cv	1	56	98	31	0.00	0.06	0.04	0.04
0	0	0.12	0	t	1	29	36	31	0.00	0.06	0.00	0.06
0	0	0.12	0	t	1	36	9	31	0.00	0.06	0.00	0.05
1	0	0.33	0	t	1	85	41	31	0.03	0.04	0.00	0.06
1	1	0.60	0	cv	1	34	42	31	0.05	0.06	0.02	0.06
0	0	0.15	0	t	1	53	5	31	0.01	0.06	0.00	0.06
0	0	0.15	0	t	1	39	74	31	0.00	0.06	0.01	0.05
1	0	0.42	0	t	1	Premnas biaculeatus	46	31	0.03	0.04	0.01	0.06
0	0	0.15	0	cv	1	84	11	31	0.00	0.04	0.00	0.06
0	0	0.14	0	t	1	22	52	31	0.00	0.06	0.01	0.06
0	0	0.11	0	t	1	29	41	31	0.00	0.06	0.00	0.06
0	0	0.17	0	t	1	25	21	31	0.00	0.05	0.01	0.06
1	0	0.14	0	cv	1	7	37	31	0.01	0.06	0.00	0.05
0	0	0.11	0	t	1	54	10	31	0.00	0.06	0.00	0.06
0	0	0.30	0	t	1	96	46	31	0.01	0.04	0.01	0.06
0	1	0.64	0	t	1	89	98	31	0.01	0.04	0.04	0.04
1	0	0.31	0	cv	1	45	1	31	0.02	0.06	0.01	0.06
1	1	0.53	0	t	1	23	80	31	0.03	0.06	0.03	0.04
1	0	0.17	0	t	1	4	83	31	0.00	0.06	0.01	0.04
0	0	0.20	0	t	1	54	42	31	0.00	0.06	0.02	0.06
1	0	0.17	0	cv	1	29	83	31	0.00	0.06	0.01	0.04
1	0	0.33	0	t	1	22	99	31	0.00	0.06	0.03	0.04
0	0	0.12	0	t	1	67	11	31	0.00	0.05	0.00	0.06
0	1	0.90	0	t	1	86	98	31	0.05	0.04	0.04	0.04
0	0	0.11	0	cv	1	2	10	31	0.00	0.06	0.00	0.06

0	0	0.22	0	t	1	28	35	31	0.01	0.06	0.01	0.06
1	1	0.86	0	t	1	Stichodactyla haddoni	14	31	0.09	0.04	0.01	0.05
0	1	0.74	0	t	1	100	9	31	0.07	0.04	0.00	0.05
0	1	0.90	0	cv	1	81	Stichodactyla mertensii	31	0.04	0.04	0.06	0.04
1	0	0.48	0	t	1	98	5	31	0.04	0.04	0.00	0.06
1	0	0.48	0	t	1	8	43	31	0.01	0.06	0.04	0.06
0	0	0.15	0	t	1	30	76	31	0.01	0.06	0.01	0.06
0	0	0.49	0	cv	1	83	Heteractis aurora	31	0.01	0.04	0.03	0.04
0	0	0.46	0	t	1	Premnas biaculeatus	64	31	0.03	0.04	0.02	0.05
1	1	0.63	0	t	1	34	70	31	0.05	0.06	0.02	0.06
0	0	0.11	0	t	1	69	41	31	0.00	0.06	0.00	0.06
0	0	0.43	0	cv	1	Premnas biaculeatus	93	31	0.03	0.04	0.01	0.04
1	0	0.46	0	t	1	Amphiprion bicinctus	71	31	0.03	0.04	0.01	0.05
1	1	0.69	0	t	1	90	15	31	0.05	0.04	0.02	0.06
0	0	0.17	0	t	1	56	83	31	0.00	0.06	0.01	0.04
1	0	0.35	0	cv	1	27	72	31	0.00	0.05	0.03	0.06
1	1	0.59	0	t	1	68	43	31	0.03	0.06	0.04	0.06
0	0	0.12	0	t	1	29	47	31	0.00	0.06	0.00	0.06
0	0	0.11	0	t	1	59	47	31	0.00	0.06	0.00	0.06
1	0	0.18	0	cv	1	93	10	31	0.01	0.04	0.00	0.06
1	0	0.46	0	t	1	88	49	31	0.02	0.04	0.02	0.06
0	0	0.34	0	t	1	80	66	31	0.03	0.04	0.01	0.05
1	0	0.23	0	t	1	26	94	31	0.01	0.06	0.01	0.04
1	0	0.49	0	cv	1	35	91	31	0.01	0.06	0.03	0.04
0	0	0.23	0	t	1	60	15	31	0.01	0.06	0.02	0.06
1	0	0.49	0	t	1	30	34	31	0.01	0.06	0.05	0.06
1	0	0.36	0	t	1	35	48	31	0.01	0.06	0.03	0.06
0	1	0.67	0	cv	1	84	92	31	0.00	0.04	0.05	0.04
1	0	0.44	0	t	1	53	31	31	0.01	0.06	0.04	0.06
1	0	0.35	0	t	1	23	35	31	0.03	0.06	0.01	0.06
0	1	0.86	0	t	1	87	100	31	0.01	0.04	0.07	0.04
0	0	0.14	0	cv	1	6	78	31	0.01	0.05	0.00	0.05
1	0	0.38	0	t	1	69	43	31	0.00	0.06	0.04	0.06
0	0	0.31	0	t	1	28	68	31	0.01	0.06	0.03	0.06
0	0	0.21	0	t	1	55	13	31	0.01	0.06	0.01	0.06
0	0	0.20	0	cv	1	24	42	31	0.00	0.06	0.02	0.06
1	1	0.63	0	t	1	Cryptodendrum adhaesivum	67	31	0.06	0.04	0.00	0.05
0	0	0.17	0	t	1	69	1	31	0.00	0.06	0.01	0.06
0	0	0.36	0	t	1	30	72	31	0.01	0.06	0.03	0.06
0	0	0.12	0	cv	1	36	5	31	0.00	0.06	0.00	0.06
0	0	0.15	0	t	1	20	4	31	0.01	0.06	0.00	0.06
1	1	0.74	0	t	1	100	36	31	0.07	0.04	0.00	0.06
0	0	0.33	0	t	1	21	68	31	0.01	0.06	0.03	0.06
0	0	0.11	0	cv	1	41	5	31	0.00	0.06	0.00	0.06

1	0	0.23	0	t	1	63	44	31	0.01	0.05	0.02	0.06
1	0	0.32	0	t	1	58	75	31	0.03	0.06	0.01	0.05
1	0	0.16	0	t	1	65	14	31	0.01	0.05	0.01	0.05
1	0	0.39	0	cv	1	81	38	31	0.04	0.04	0.00	0.06
0	0	0.31	0	t	1	55	70	31	0.01	0.06	0.02	0.06
1	0	0.46	0	t	1	36	34	31	0.00	0.06	0.05	0.06
1	0	0.14	0	t	1	66	69	31	0.01	0.05	0.00	0.06
1	1	0.65	0	cv	1	27	Stichodactyla mertensii	31	0.00	0.05	0.06	0.04
0	0	0.17	0	t	1	82	41	31	0.01	0.03	0.00	0.06
0	0	0.17	0	t	1	82	10	31	0.01	0.03	0.00	0.06
1	0	0.44	0	t	1	20	43	31	0.01	0.06	0.04	0.06
1	0	0.24	0	cv	1	87	3	31	0.01	0.04	0.01	0.06
0	0	0.15	0	t	1	24	28	31	0.00	0.06	0.01	0.06
1	1	0.73	0	t	1	100	38	31	0.07	0.04	0.00	0.06
1	0	0.30	0	t	1	88	77	31	0.02	0.04	0.01	0.06
0	0	0.15	0	cv	1	28	11	31	0.01	0.06	0.00	0.06
0	0	0.16	0	t	1	46	41	31	0.01	0.06	0.00	0.06
0	0	0.14	0	t	1	27	30	31	0.00	0.05	0.01	0.06
0	0	0.24	0	t	1	27	49	31	0.00	0.05	0.02	0.06
1	0	0.20	0	cv	1	63	95	31	0.01	0.05	0.01	0.04
0	0	0.12	0	t	1	27	36	31	0.00	0.05	0.00	0.06
0	0	0.16	0	t	1	11	73	31	0.00	0.06	0.01	0.05
0	0	0.13	0	t	1	4	7	31	0.00	0.06	0.01	0.06
0	0	0.11	0	cv	1	59	10	31	0.00	0.06	0.00	0.06
1	0	0.48	0	t	1	98	47	31	0.04	0.04	0.00	0.06
1	0	0.12	0	t	1	56	37	31	0.00	0.06	0.00	0.05
0	0	0.26	0	t	1	68	77	31	0.03	0.06	0.01	0.06
1	0	0.14	0	cv	1	66	78	31	0.01	0.05	0.00	0.05
1	0	0.31	0	t	1	46	45	31	0.01	0.06	0.02	0.06
0	0	0.15	0	t	1	27	14	31	0.00	0.05	0.01	0.05
1	0	0.26	0	t	1	23	69	31	0.03	0.06	0.00	0.06
0	0	0.27	0	cv	1	62	44	31	0.01	0.06	0.02	0.06
1	0	0.32	0	t	1	97	77	31	0.03	0.04	0.01	0.06
0	0	0.15	0	t	1	56	51	31	0.00	0.06	0.01	0.06
0	0	0.50	0	t	1	82	43	31	0.01	0.03	0.04	0.06
0	0	0.24	0	cv	1	68	2	31	0.03	0.06	0.00	0.06
0	0	0.11	0	t	1	54	38	31	0.00	0.06	0.00	0.06
0	0	0.15	0	t	1	13	47	31	0.01	0.06	0.00	0.06
1	0	0.20	0	t	1	74	71	31	0.01	0.05	0.01	0.05
0	0	0.17	0	cv	1	21	11	31	0.01	0.06	0.00	0.06
0	0	0.13	0	t	1	10	77	31	0.00	0.06	0.01	0.06
0	0	0.13	0	t	1	59	3	31	0.00	0.06	0.01	0.06
1	0	0.22	0	t	1	64	77	31	0.02	0.05	0.01	0.06
0	0	0.19	0	cv	1	83	12	31	0.01	0.04	0.01	0.06
1	0	0.32	0	t	1	33	95	31	0.03	0.06	0.01	0.04
0	0	0.17	0	t	1	8	69	31	0.01	0.06	0.00	0.06
0	0	0.25	0	t	1	29	18	31	0.00	0.06	0.03	0.06
1	0	0.32	0	cv	1	60	97	31	0.01	0.06	0.03	0.04
0	0	0.22	0	t	1	21	74	31	0.01	0.06	0.01	0.05
0	0	0.16	0	t	1	83	32	31	0.01	0.04	0.00	0.06

1	0	0.18	0	t	1	24	89	31	0.00	0.06	0.01	0.04
0	0	0.34	0	cv	1	58	52	31	0.03	0.06	0.01	0.06
0	0	0.15	0	t	1	25	53	31	0.00	0.05	0.01	0.06
1	1	0.56	0	t	1	69	90	31	0.00	0.06	0.05	0.04
0	0	0.39	0	t	1	Amphiprion bicinctus	9	31	0.03	0.04	0.00	0.05
1	0	0.43	0	cv	1	34	2	31	0.05	0.06	0.00	0.06
0	0	0.15	0	t	1	53	78	31	0.01	0.06	0.00	0.05
0	0	0.13	0	t	1	10	75	31	0.00	0.06	0.01	0.05
0	0	0.19	0	t	1	28	51	31	0.01	0.06	0.01	0.06
0	0	0.13	0	cv	1	3	10	31	0.01	0.06	0.00	0.06
0	0	0.34	0	t	1	85	47	31	0.03	0.04	0.00	0.06
0	0	0.27	0	t	1	69	48	31	0.00	0.06	0.03	0.06
0	0	0.14	0	t	1	59	51	31	0.00	0.06	0.01	0.06
1	1	0.54	0	cv	1	55	57	31	0.01	0.06	0.04	0.06
0	0	0.12	0	t	1	22	37	31	0.00	0.06	0.00	0.05
0	1	0.66	0	t	1	90	93	31	0.05	0.04	0.01	0.04
1	0	0.47	0	t	1	18	48	31	0.03	0.06	0.03	0.06
0	0	0.18	0	cv	1	11	1	31	0.00	0.06	0.01	0.06
0	0	0.14	0	t	1	37	77	31	0.00	0.05	0.01	0.06
1	0	0.17	0	t	1	9	73	31	0.00	0.05	0.01	0.05
1	0	0.23	0	t	1	87	60	31	0.01	0.04	0.01	0.06
1	0	0.26	0	cv	1	87	13	31	0.01	0.04	0.01	0.06
0	0	0.13	0	t	1	26	4	31	0.01	0.06	0.00	0.06
1	1	0.57	0	t	1	86	76	31	0.05	0.04	0.01	0.06
1	0	0.20	0	t	1	54	87	31	0.00	0.06	0.01	0.04
0	0	0.17	0	cv	1	13	14	31	0.01	0.06	0.01	0.05
1	0	0.22	0	t	1	1	71	31	0.01	0.06	0.01	0.05
0	0	0.11	0	t	1	38	32	31	0.00	0.06	0.00	0.06
0	0	0.24	0	t	1	8	62	31	0.01	0.06	0.01	0.06
1	0	0.24	0	cv	1	9	70	31	0.00	0.05	0.02	0.06
0	0	0.17	0	t	1	24	1	31	0.00	0.06	0.01	0.06
0	0	0.15	0	t	1	20	36	31	0.01	0.06	0.00	0.06
0	0	0.13	0	t	1	26	56	31	0.01	0.06	0.00	0.06
1	0	0.31	0	cv	1	22	Entacmaea quadricolor	31	0.00	0.06	0.03	0.04
1	1	0.76	0	t	1	57	31	31	0.04	0.06	0.04	0.06
0	1	0.93	1	t	1	Caltha palustris	Spilogona sanctipauli	32	0.08	0.08	0.06	0.08
0	1	0.88	1	t	1	Caltha palustris	Spilogona melanosoma	32	0.08	0.08	0.04	0.09
0	1	0.78	1	cv	1	Caltha palustris	Spilogona hurdiana	32	0.08	0.08	0.02	0.08
0	1	0.99	1	t	1	Caltha palustris	Spilogona almquistii	32	0.08	0.08	0.10	0.08
0	1	0.88	1	t	1	Caltha palustris	Spilogona dorsata	32	0.08	0.08	0.04	0.09
0	1	0.57	1	t	1	Pogonomyiodes segnis	Dryas integrifolia	32	0.06	0.09	0.02	0.08
1	1	0.98	1	cv	1	Pogonomyiodes segnis	Potentilla vahliana	32	0.06	0.09	0.11	0.08

0	1	0.93	1	t	1	Pogonomyiodes segnis	Papaver radicatum	32	0.06	0.09	0.08	0.08
0	1	0.60	1	t	1	Dryas integrifolia	Spilogona sanctipauli	32	0.02	0.08	0.06	0.08
0	0	0.32	1	t	1	Dryas integrifolia	Spilogona projecta	32	0.02	0.08	0.02	0.07
0	1	0.90	1	cv	1	Dryas integrifolia	Spilogona almquistii	32	0.02	0.08	0.10	0.08
0	1	0.86	1	t	1	Dryas integrifolia	Boloria improba	32	0.02	0.08	0.09	0.08
0	1	0.59	1	t	1	Dryas integrifolia	Eupogonomyia probilofensis	32	0.02	0.08	0.06	0.09
0	0	0.49	1	t	1	Spilogona aesturium	Taraxacum sp. 2	32	0.02	0.07	0.04	0.08
1	1	0.97	1	cv	1	Boreellus atriceps	Potentilla vahliana	32	0.04	0.09	0.11	0.08
0	1	0.62	1	t	1	Boreellus atriceps	Taraxacum sp. 2	32	0.04	0.09	0.04	0.08
0	0	0.49	1	t	1	Rhamphomyia sp.	Taraxacum sp. 2	32	0.02	0.07	0.04	0.08
1	1	0.98	1	t	1	Potentilla vahliana	Spilogona sanctipauli	32	0.11	0.08	0.06	0.08
0	1	0.92	1	cv	1	Potentilla vahliana	Spilogona latilamina	32	0.11	0.08	0.02	0.09
0	1	0.96	1	t	1	Potentilla vahliana	Spilogona melanosoma	32	0.11	0.08	0.04	0.09
1	1	1.00	1	t	1	Potentilla vahliana	Spilogona almquistii	32	0.11	0.08	0.10	0.08
0	1	0.92	1	t	1	Potentilla vahliana	Protocalliphora sp.	32	0.11	0.08	0.02	0.09
1	1	0.98	1	cv	1	Potentilla vahliana	Eupogonomyia probilofensis	32	0.11	0.08	0.06	0.09
0	1	0.96	1	t	1	Potentilla vahliana	Spilogona dorsata	32	0.11	0.08	0.04	0.09
0	1	0.84	1	t	1	Arnica alpina	Spilogona sanctipauli	32	0.06	0.08	0.06	0.08
0	1	0.97	1	t	1	Arnica alpina	Spilogona almquistii	32	0.06	0.08	0.10	0.08
0	1	0.84	1	cv	1	Arnica alpina	Eupogonomyia probilofensis	32	0.06	0.08	0.06	0.09
0	1	0.75	1	t	1	Spilogona sanctipauli	Taraxacum sp. 2	32	0.06	0.08	0.04	0.08
0	1	0.77	1	t	1	Spilogona sanctipauli	Cerastium alpinum	32	0.06	0.08	0.04	0.07
0	0	0.38	1	t	1	Astragalus alpinus	Colias hecla	32	0.02	0.07	0.02	0.06
0	1	0.89	1	cv	1	Astragalus alpinus	Colias nastes	32	0.02	0.07	0.09	0.08

0	1	0.60	1	t	1	Astragalus alpinus	Bombus polaris	32	0.02	0.07	0.04	0.05
0	1	0.95	1	t	1	Geum rossii	Spilogona almquistii	32	0.04	0.08	0.10	0.08
1	1	0.99	1	t	1	Papaver radicatum	Spilogona almquistii	32	0.08	0.08	0.10	0.08
0	1	0.95	1	cv	1	Taraxacum sp. 2	Spilogona almquistii	32	0.04	0.08	0.10	0.08
0	1	0.54	1	t	1	Spilogona projecta	Cerastium alpinum	32	0.02	0.07	0.04	0.07
0	1	0.68	1	t	1	Bombus polaris	Petasites frigidus	32	0.04	0.05	0.02	0.04
1	1	0.66	1	t	1	Anarta richardsoni	Oxytropis arctica	32	0.02	0.02	0.02	0.02
0	1	0.95	0	cv	1	Dryas integrifolia	Potentilla vahliana	32	0.02	0.08	0.11	0.08
0	1	0.96	0	t	1	Cerastium alpinum	Spilogona almquistii	32	0.04	0.07	0.10	0.08
0	1	0.88	0	t	1	Caltha	Geum rossii	32	0.08	0.08	0.04	0.08
0	1	0.82	0	t	1	Arnica alpina	Bombus polaris	32	0.06	0.08	0.04	0.05
0	0	0.42	0	cv	1	Rhamphomyia sp.	Petasites frigidus	32	0.02	0.07	0.02	0.04
0	0	0.47	0	t	1	Spilogona aesturium	Geum rossii	32	0.02	0.07	0.04	0.08
0	0	0.40	0	t	1	Dryas integrifolia	Petasites frigidus	32	0.02	0.08	0.02	0.04
0	0	0.48	0	t	1	Spilogona projecta	Spilogona dorsata	32	0.02	0.07	0.04	0.09
0	1	0.92	0	cv	1	Colias nastes	Oxytropis arctica	32	0.09	0.08	0.02	0.02
0	1	0.60	0	t	1	Pogonomyiodes segnis	Astragalus alpinus	32	0.06	0.09	0.02	0.07
0	1	0.74	0	t	1	Arnica alpina	Spilogona dorsata	32	0.06	0.08	0.04	0.09
0	1	0.53	0	t	1	Spilogona latilamina	Bombus polaris	32	0.02	0.09	0.04	0.05
0	1	0.63	0	cv	1	Arnica alpina	Spilogona projecta	32	0.06	0.08	0.02	0.07
0	0	0.48	0	t	1	Spilogona hurdiana	Cerastium alpinum	32	0.02	0.08	0.04	0.07
0	0	0.34	0	t	1	Dryas integrifolia	Colias hecla	32	0.02	0.08	0.02	0.06
0	1	0.75	0	t	1	Arnica alpina	Anarta richardsoni	32	0.06	0.08	0.02	0.02
0	1	0.63	0	cv	1	Astragalus alpinus	Eupogonomyia probilofensis	32	0.02	0.07	0.06	0.09
0	0	0.34	0	t	1	Colias hecla	Spilogona hurdiana	32	0.02	0.06	0.02	0.08

0	1	0.56	0	t	1	Dryas integrifolia	Bombus polaris	32	0.02	0.08	0.04	0.05
0	1	0.77	0	t	1	Oxytropis arctica	Eupogonomyia probilofensis	32	0.02	0.02	0.06	0.09
0	1	0.93	0	cv	1	Spilogona sanctipauli	Papaver radicatum	32	0.06	0.08	0.08	0.08
0	1	0.89	0	t	1	Spilogona hurdiana	Spilogona almquistii	32	0.02	0.08	0.10	0.08
0	1	0.85	0	t	1	Spilogona latilamina	Boloria improba	32	0.02	0.09	0.09	0.08
0	1	0.95	0	t	1	Potentilla vahliana	Colias hecla	32	0.11	0.08	0.02	0.06
0	0	0.39	0	cv	1	Petasites frigidus	Protocalliphora sp.	32	0.02	0.04	0.02	0.09
0	0	0.46	0	t	1	Spilogona aesturium	Spilogona dorsata	32	0.02	0.07	0.04	0.09
0	0	0.34	0	t	1	Rhamphomyia sp.	Spilogona projecta	32	0.02	0.07	0.02	0.07
0	1	0.79	0	t	1	Caltha palustris	Dryas integrifolia	32	0.08	0.08	0.02	0.08
0	1	0.93	0	cv	1	Taraxacum sp.	Colias nastes	32	0.04	0.08	0.09	0.08
0	1	0.98	0	t	1	Potentilla vahliana	Bombus polaris	32	0.11	0.08	0.04	0.05
0	1	0.68	0	t	1	Pogonomyiodes segnis	Petasites frigidus	32	0.06	0.09	0.02	0.04
0	1	0.54	0	t	1	Bombus polaris	Protocalliphora sp.	32	0.04	0.05	0.02	0.09
0	1	0.87	0	cv	1	Papaver radicatum	Spilogona dorsata	32	0.08	0.08	0.04	0.09
0	0	0.40	0	t	1	Boreellus atriceps	Protocalliphora sp.	32	0.04	0.09	0.02	0.09
0	1	0.94	0	t	1	Anarta richardsoni	Boloria improba	32	0.02	0.02	0.09	0.08
0	1	0.50	0	t	1	Astragalus alpinus	Taraxacum sp. 2	32	0.02	0.07	0.04	0.08
0	1	0.90	0	cv	1	Colias hecla	Boloria improba	32	0.02	0.06	0.09	0.08
0	1	0.88	0	t	1	Astragalus alpinus	Boloria improba	32	0.02	0.07	0.09	0.08
0	0	0.28	1	t	1	Nomada (Heminomada) luteola	Stellaria pubera	33	0.01	0.05	0.02	0.05
1	1	0.67	1	t	1	Osmia atriventris	Viola papilionacea	33	0.02	0.03	0.04	0.03
0	0	0.44	1	cv	1	Osmia atriventris	Stellaria pubera	33	0.02	0.03	0.02	0.05
1	1	0.73	1	t	1	halictid sp.	Thalictrum thalictroides	33	0.03	0.03	0.04	0.04
0	0	0.50	1	t	1	halictid sp.	Hepatica americana	33	0.03	0.03	0.02	0.05

0	1	0.51	1	t	1	halictid sp.	Claytonia virginica	33	0.03	0.03	0.02	0.05
0	1	0.58	1	cv	1	Viola papilionacea	Bombylius major	33	0.04	0.03	0.02	0.04
0	1	0.51	1	t	1	Viola papilionacea	Osmia conjucta	33	0.04	0.03	0.01	0.04
1	1	0.56	1	t	1	Viola papilionacea	Osmia simillima	33	0.04	0.03	0.01	0.03
1	1	0.63	1	t	1	Viola papilionacea	Andrena (Tomelissa) violae	33	0.04	0.03	0.02	0.03
0	1	0.60	1	cv	1	Ephemoropsis laboriosa	Tiarella cordifolia collina	33	0.05	0.04	0.01	0.05
1	1	0.87	1	t	1	Ephemoropsis laboriosa	Aesculus sylvatica	33	0.05	0.04	0.04	0.04
1	1	0.63	1	t	1	Uvularia sessilifolia	Dialictus abanci	33	0.01	0.04	0.05	0.04
1	0	0.42	1	t	1	Uvularia sessilifolia	Andrena (Euandrena) nigrihirta	33	0.01	0.04	0.03	0.05
1	0	0.42	1	cv	1	Uvularia sessilifolia	Andrena (Andrena) tridens	33	0.01	0.04	0.03	0.05
1	1	0.51	1	t	1	Uvularia sessilifolia	Andrena (Micrandrena) ziziaeformis	33	0.01	0.04	0.04	0.03
1	0	0.30	1	t	1	Uvularia sessilifolia	Evylaeus macoupinens is	33	0.01	0.04	0.02	0.04
1	1	0.84	1	t	1	Dialictus abanci	Thalictrum thalictroides	33	0.05	0.04	0.04	0.04
0	1	0.65	1	cv	1	Dialictus abanci	Hepatica americana	33	0.05	0.04	0.02	0.05
0	1	0.66	1	t	1	Dialictus abanci	Claytonia virginica	33	0.05	0.04	0.02	0.05
0	1	0.69	1	t	1	Dialictus abanci	Stellaria pubera	33	0.05	0.04	0.02	0.05
0	1	0.79	1	t	1	Dialictus abanci	Erythronium umbilicatum	33	0.05	0.04	0.03	0.05
1	1	0.55	1	cv	1	Thalictrum thalictroides	Bombylius major	33	0.04	0.04	0.02	0.04
1	1	0.61	1	t	1	Thalictrum thalictroides	Platycheirus obscurus	33	0.04	0.04	0.02	0.04
1	1	0.61	1	t	1	Thalictrum thalictroides	Nomada sp.	33	0.04	0.04	0.02	0.04
1	1	0.66	1	t	1	Thalictrum thalictroides	Osmia sp.	33	0.04	0.04	0.03	0.04
1	1	0.66	1	cv	1	Thalictrum thalictroides	Gonia sp.	33	0.04	0.04	0.03	0.04

1	1	0.57	1	t	1	Thalictrum thalictroides	Evylaeus macoupinensis	33	0.04	0.04	0.02	0.04
1	0	0.49	1	t	1	Thalictrum thalictroides	Ceratina calcarata	33	0.04	0.04	0.01	0.04
1	1	0.66	1	t	1	Thalictrum thalictroides	Andrena (Ptilandrena) erigeniae	33	0.04	0.04	0.03	0.04
0	0	0.38	1	cv	1	Hylema platura	Hepatica americana	33	0.02	0.04	0.02	0.05
0	0	0.39	1	t	1	Hylema platura	Claytonia virginica	33	0.02	0.04	0.02	0.05
0	0	0.44	1	t	1	Bombylius major	Cardamine angustata angustata	33	0.02	0.04	0.03	0.06
0	0	0.32	1	t	1	Bombylius major	Hepatica americana	33	0.02	0.04	0.02	0.05
0	0	0.25	1	cv	1	Bombylius major	Tiarella cordifolia collina	33	0.02	0.04	0.01	0.05
0	0	0.32	1	t	1	Bombylius major	Claytonia virginica	33	0.02	0.04	0.02	0.05
0	0	0.36	1	t	1	Bombylius major	Stellaria pubera	33	0.02	0.04	0.02	0.05
0	0	0.48	1	t	1	Bombylius major	Erythronium umbilicatum	33	0.02	0.04	0.03	0.05
0	0	0.37	1	cv	1	Bombylius major	Sangiunaria canadensis	33	0.02	0.04	0.02	0.05
0	0	0.37	1	t	1	Xylocopa virginica virginica	Cardamine angustata angustata	33	0.01	0.04	0.03	0.06
0	1	0.53	1	t	1	Xylocopa virginica virginica	Podophyllum peltatum	33	0.01	0.04	0.04	0.04
0	0	0.26	1	t	1	Xylocopa virginica virginica	Claytonia virginica	33	0.01	0.04	0.02	0.05
0	0	0.20	1	cv	1	Xylocopa virginica virginica	Trillium catesbei	33	0.01	0.04	0.01	0.05
0	0	0.40	1	t	1	Xylocopa virginica virginica	Erythronium umbilicatum	33	0.01	0.04	0.03	0.05
0	0	0.30	1	t	1	Xylocopa virginica virginica	Sangiunaria canadensis	33	0.01	0.04	0.02	0.05
0	0	0.35	1	t	1	Nomada (Nomada) pygmaea	Cardamine angustata angustata	33	0.01	0.05	0.03	0.06

0	0	0.24	1	cv	1	Nomada (Nomada) pygmaea	Claytonia virginica	33	0.01	0.05	0.02	0.05
0	0	0.27	1	t	1	Nomada (Nomada) pygmaea	Stellaria pubera	33	0.01	0.05	0.02	0.05
0	0	0.39	1	t	1	unknown sp.	Cardamine angustata angustata	33	0.02	0.05	0.03	0.06
0	1	0.88	1	t	1	Andrena (Micrandrena) personata	Stellaria pubera	33	0.09	0.08	0.02	0.05
0	1	0.81	1	cv	1	Euchlo creusa lotta	Cardamine angustata angustata	33	0.06	0.05	0.03	0.06
0	1	0.71	1	t	1	Euchlo creusa lotta	Claytonia virginica	33	0.06	0.05	0.02	0.05
0	0	0.48	1	t	1	Cardamine angustata angustata	Platycheirus obscurus	33	0.03	0.06	0.02	0.04
0	0	0.48	1	t	1	Cardamine angustata angustata	Nomada sp.	33	0.03	0.06	0.02	0.04
0	0	0.39	1	cv	1	Cardamine angustata angustata	Andrena (Simandrena) nasonii	33	0.03	0.06	0.02	0.05
0	1	0.58	1	t	1	Cardamine angustata angustata	Andrena (Euandrena) nigrihirta	33	0.03	0.06	0.03	0.05
0	1	0.54	1	t	1	Cardamine angustata angustata	Osmia sp.	33	0.03	0.06	0.03	0.04
0	0	0.38	1	t	1	Cardamine angustata angustata	Andrena (Scaphandrena) arabis	33	0.03	0.06	0.02	0.05
0	1	0.58	1	cv	1	Cardamine angustata angustata	Augochlora pura	33	0.03	0.06	0.03	0.05
0	1	0.58	1	t	1	Cardamine angustata angustata	Andrena (Andrena) tridens	33	0.03	0.06	0.03	0.05
0	1	0.95	1	t	1	Cardamine angustata angustata	Andrena (Melandrena) dunningi	33	0.03	0.06	0.10	0.09
0	1	0.54	1	t	1	Cardamine angustata angustata	Gonia sp.	33	0.03	0.06	0.03	0.04
0	1	0.62	1	cv	1	Cardamine angustata angustata	Nomada (Gnathias) perplexa	33	0.03	0.06	0.04	0.05

1	1	0.93	1	t	1	Cardamine angustata angustata	Apis mellifera	33	0.03	0.06	0.08	0.05
0	1	0.86	1	t	1	Cardamine angustata angustata	Andrena (Melandrena) carlini	33	0.03	0.06	0.06	0.05
0	0	0.35	1	t	1	Cardamine angustata angustata	Osmia conjucta	33	0.03	0.06	0.01	0.04
0	1	0.68	1	cv	1	Cardamine angustata angustata	Bombus bimaculatus	33	0.03	0.06	0.04	0.04
0	0	0.47	1	t	1	Cardamine angustata angustata	bombus pennsylvanic us	33	0.03	0.06	0.02	0.05
0	0	0.33	1	t	1	Cardamine angustata angustata	Osmia lignaria	33	0.03	0.06	0.01	0.05
0	1	0.50	1	t	1	Cardamine angustata angustata	Lassioglossu m fuscipenne	33	0.03	0.06	0.03	0.05
0	1	0.54	1	cv	1	Cardamine angustata angustata	Andrena (Ptilandrena) erigeniae	33	0.03	0.06	0.03	0.04
1	0	0.37	1	t	1	Hepatica americana	Nomada sp.	33	0.02	0.05	0.02	0.04
1	0	0.46	1	t	1	Hepatica americana	Andrena (Euandrena) nigrihirta	33	0.02	0.05	0.03	0.05
1	0	0.46	1	t	1	Hepatica americana	Andrena (Andrena) tridens	33	0.02	0.05	0.03	0.05
1	0	0.42	1	cv	1	Hepatica americana	Gonia sp.	33	0.02	0.05	0.03	0.04
0	1	0.89	1	t	1	Hepatica americana	Apis mellifera	33	0.02	0.05	0.08	0.05
1	1	0.79	1	t	1	Hepatica americana	Andrena (Melandrena) carlini	33	0.02	0.05	0.06	0.05
1	0	0.33	1	t	1	Hepatica americana	Evylaeus macoupinens is	33	0.02	0.05	0.02	0.04
1	1	0.57	1	cv	1	Hepatica americana	Bombus bimaculatus	33	0.02	0.05	0.04	0.04
0	1	0.55	1	t	1	Andrena (Trachandrena) ceanothi	Claytonia virginica	33	0.04	0.04	0.02	0.05
0	0	0.37	1	t	1	Platycheirus obscurus	Claytonia virginica	33	0.02	0.04	0.02	0.05
0	0	0.41	1	t	1	Platycheirus obscurus	Stellaria pubera	33	0.02	0.04	0.02	0.05

0	0	0.37	1	cv	1	Nomada sp.	Claytonia virginica	33	0.02	0.04	0.02	0.05
0	0	0.41	1	t	1	Nomada sp.	Stellaria pubera	33	0.02	0.04	0.02	0.05
0	1	0.53	1	t	1	Nomada sp.	Erythronium umbilicatum	33	0.02	0.04	0.03	0.05
0	0	0.29	1	t	1	Andrena (Simandrena) nasonii	Claytonia virginica	33	0.02	0.05	0.02	0.05
0	0	0.33	1	cv	1	Andrena (Simandrena) nasonii	Stellaria pubera	33	0.02	0.05	0.02	0.05
0	1	0.52	1	t	1	Toxomerous geminatus	Claytonia virginica	33	0.04	0.04	0.02	0.05
0	1	0.55	1	t	1	Toxomerous geminatus	Stellaria pubera	33	0.04	0.04	0.02	0.05
0	1	0.56	1	t	1	Toxomerous geminatus	Sangiunaria canadensis	33	0.04	0.04	0.02	0.05
1	1	0.80	1	cv	1	Podophyllum peltatum	Bombus bimaculatus	33	0.04	0.04	0.04	0.04
0	0	0.46	1	t	1	Andrena (Euandrena) nigrihirta	Claytonia virginica	33	0.03	0.05	0.02	0.05
0	0	0.50	1	t	1	Andrena (Euandrena) nigrihirta	Stellaria pubera	33	0.03	0.05	0.02	0.05
0	1	0.62	1	t	1	Andrena (Euandrena) nigrihirta	Erythronium umbilicatum	33	0.03	0.05	0.03	0.05
0	1	0.86	1	cv	1	Tiarella cordifolia collina	Apis mellifera	33	0.01	0.05	0.08	0.05
1	0	0.29	1	t	1	Tiarella cordifolia collina	Dialictus cressonii	33	0.01	0.05	0.02	0.04
0	0	0.27	1	t	1	Tiarella cordifolia collina	Evylaeus macoupinensis	33	0.01	0.05	0.02	0.04
0	0	0.20	1	t	1	Tiarella cordifolia collina	Osmia conjucta	33	0.01	0.05	0.01	0.04
1	0	0.49	1	cv	1	Tiarella cordifolia collina	Bombus bimaculatus	33	0.01	0.05	0.04	0.04
0	0	0.42	1	t	1	Osmia sp.	Claytonia virginica	33	0.03	0.04	0.02	0.05
0	0	0.46	1	t	1	Osmia sp.	Stellaria pubera	33	0.03	0.04	0.02	0.05
0	1	0.58	1	t	1	Osmia sp.	Erythronium umbilicatum	33	0.03	0.04	0.03	0.05

0	0	0.45	1	cv	1	Augochlora pura	Claytonia virginica	33	0.03	0.05	0.02	0.05
0	0	0.49	1	t	1	Augochlora pura	Stellaria pubera	33	0.03	0.05	0.02	0.05
0	1	0.62	1	t	1	Augochlora pura	Erythronium umbilicatum	33	0.03	0.05	0.03	0.05
0	0	0.26	1	t	1	Augochorella striata	Claytonia virginica	33	0.01	0.04	0.02	0.05
0	0	0.41	1	cv	1	Augochorella striata	Erythronium umbilicatum	33	0.01	0.04	0.03	0.05
0	0	0.46	1	t	1	Andrena (Andrena) tridens	Claytonia virginica	33	0.03	0.05	0.02	0.05
0	1	0.62	1	t	1	Andrena (Andrena) tridens	Erythronium umbilicatum	33	0.03	0.05	0.03	0.05
0	0	0.42	1	t	1	Gonia sp.	Claytonia virginica	33	0.03	0.04	0.02	0.05
0	0	0.46	1	cv	1	Gonia sp.	Stellaria pubera	33	0.03	0.04	0.02	0.05
1	1	0.51	1	t	1	Claytonia virginica	Nomada (Gnathias) perplexa	33	0.02	0.05	0.04	0.05
1	1	0.56	1	t	1	Claytonia virginica	Andrena (Micrandrena) ziziaeformis	33	0.02	0.05	0.04	0.03
0	1	0.89	1	t	1	Claytonia virginica	Apis mellifera	33	0.02	0.05	0.08	0.05
1	0	0.37	1	cv	1	Claytonia virginica	Dialictus cressonii	33	0.02	0.05	0.02	0.04
1	1	0.79	1	t	1	Claytonia virginica	Andrena (Melandrena) carlini	33	0.02	0.05	0.06	0.05
1	0	0.34	1	t	1	Claytonia virginica	Evylaeus macoupinens is	33	0.02	0.05	0.02	0.04
0	0	0.27	1	t	1	Claytonia virginica	Andrena (Scrapopteris) imatrix	33	0.02	0.05	0.01	0.04
1	0	0.27	1	cv	1	Claytonia virginica	Ceratina calcarata	33	0.02	0.05	0.01	0.04
1	0	0.26	1	t	1	Claytonia virginica	Osmia conjucta	33	0.02	0.05	0.01	0.04
1	0	0.24	1	t	1	Claytonia virginica	Osmia lignaria	33	0.02	0.05	0.01	0.05
0	0	0.27	1	t	1	Claytonia virginica	Lycaenopsis argiolus	33	0.02	0.05	0.01	0.04
1	0	0.43	1	cv	1	Claytonia virginica	Andrena (Ptilandrena) erigeniae	33	0.02	0.05	0.03	0.04

0	1	0.54	1	t	1	Nomada (Gnathias) perplexa	Stellaria pubera	33	0.04	0.05	0.02	0.05
0	1	0.66	1	t	1	Nomada (Gnathias) perplexa	Erythronium umbilicatum	33	0.04	0.05	0.03	0.05
1	1	0.93	1	t	1	Apis mellifera	Erythronium umbilicatum	33	0.08	0.05	0.03	0.05
1	1	0.89	1	cv	1	Apis mellifera	Sangiunaria canadensis	33	0.08	0.05	0.02	0.05
1	1	0.81	1	t	1	Stellaria pubera	Andrena (Melandrena) carlini	33	0.02	0.05	0.06	0.05
0	0	0.40	1	t	1	Stellaria pubera	Nomada (Heminomad a) bishopii	33	0.02	0.05	0.02	0.04
0	0	0.30	1	t	1	Stellaria pubera	Ceratina calcarata	33	0.02	0.05	0.01	0.04
0	0	0.28	1	cv	1	Stellaria pubera	Osmia conjucta	33	0.02	0.05	0.01	0.04
0	1	0.61	1	t	1	Stellaria pubera	Nomada (Nomada) sayi	33	0.02	0.05	0.04	0.04
0	0	0.46	1	t	1	Stellaria pubera	Andrena (Ptilandrena) erigeniae	33	0.02	0.05	0.03	0.04
0	0	0.41	1	t	1	Dialictus cressonii	Sangiunaria canadensis	33	0.02	0.04	0.02	0.05
1	1	0.80	1	cv	1	Aesculus sylvatica	Bombus bimaculatus	33	0.04	0.04	0.04	0.04
0	1	0.62	1	t	1	Aesculus sylvatica	bombus pennsylvanic us	33	0.04	0.04	0.02	0.05
1	1	0.74	1	t	1	Trillium catesbei	Andrena (Melandrena) carlini	33	0.01	0.05	0.06	0.05
1	0	0.50	1	t	1	Trillium catesbei	Bombus bimaculatus	33	0.01	0.05	0.04	0.04
1	1	0.87	1	cv	1	Andrena (Melandrena) carlini	Erythronium umbilicatum	33	0.06	0.05	0.03	0.05
0	1	0.80	1	t	1	Andrena (Melandrena) carlini	Sangiunaria canadensis	33	0.06	0.05	0.02	0.05
1	0	0.39	1	t	1	Erythronium umbilicatum	Osmia conjucta	33	0.03	0.05	0.01	0.04
1	0	0.37	1	t	1	Erythronium umbilicatum	Osmia lignaria	33	0.03	0.05	0.01	0.05
0	1	0.61	1	cv	1	Bombus bimaculatus	Sangiunaria canadensis	33	0.04	0.04	0.02	0.05

0	1	0.61	0	t	1	Podophyllum peltatum	Evylaeus macoupinensis	33	0.04	0.04	0.02	0.04
0	0	0.35	0	t	1	unknown sp.	Platycheirus obscurus	33	0.02	0.05	0.02	0.04
0	0	0.34	0	t	1	Andrena (Scaphandrena) arabis	Dialictus cressonii	33	0.02	0.05	0.02	0.04
0	0	0.47	0	cv	1	Hylema platura	Nomada (Heminomada) bishopii	33	0.02	0.04	0.02	0.04
0	1	0.90	0	t	1	Apis mellifera	Dialictus cressonii	33	0.08	0.05	0.02	0.04
0	0	0.27	0	t	1	Hepatica americana	Ceratina calcarata	33	0.02	0.05	0.01	0.04
0	1	0.74	0	t	1	Nomada (Nomada) pygmaea	Andrena (Melandrena) carlini	33	0.01	0.05	0.06	0.05
0	1	0.85	0	cv	1	Dialictus abanci	Andrena (Micrandrena) ziziaeformis	33	0.05	0.04	0.04	0.03
0	1	0.80	0	t	1	Andrena (Trachandrena) ceanothi	Podophyllum peltatum	33	0.04	0.04	0.04	0.04
0	1	0.67	0	t	1	Hylema platura	Nomada (Nomada) sayi	33	0.02	0.04	0.04	0.04
0	0	0.25	0	t	1	Nomada (Heminomada) luteola	Hepatica americana	33	0.01	0.05	0.02	0.05
1	0	0.22	0	cv	1	Uvularia sessilifolia	Osmia conjucta	33	0.01	0.04	0.01	0.04
0	1	0.62	0	t	1	Bombus bimaculatus	bombus pennsylvanicus	33	0.04	0.04	0.02	0.05
0	0	0.45	0	t	1	Platycheirus obscurus	Nomada sp.	33	0.02	0.04	0.02	0.04
1	0	0.37	0	t	1	Hepatica americana	Platycheirus obscurus	33	0.02	0.05	0.02	0.04
0	1	0.79	0	cv	1	Euchlo creusa lotta	Lassioglossum fuscipenne	33	0.06	0.05	0.03	0.05
0	1	0.77	0	t	1	Dialictus	Gonia sp.	33	0.05	0.04	0.03	0.04
0	1	0.65	0	t	1	Thalictrum thalictroides	Cardamine angustata angustata	33	0.04	0.04	0.03	0.06
0	0	0.49	0	t	1	Hylema platura	Lassioglossum fuscipenne	33	0.02	0.04	0.03	0.05
0	0	0.36	0	cv	1	Platycheirus obscurus	Andrena (Simandrena) nasonii	33	0.02	0.04	0.02	0.05

0	1	0.70	0	t	1	Dialictus abanci	Sangiunaria canadensis	33	0.05	0.04	0.02	0.05
0	0	0.30	0	t	1	Augochorella striata	Sangiunaria canadensis	33	0.01	0.04	0.02	0.05
0	0	0.43	0	t	1	Nomada sp.	bombus pennsylvanicus	33	0.02	0.04	0.02	0.05
0	0	0.38	0	cv	1	Augochorella striata	Andrena (Ptilandrena) erigeniae	33	0.01	0.04	0.03	0.04
0	1	0.64	0	t	1	Nomada sp.	Andrena (Micrandrena) ziziaeformis	33	0.02	0.04	0.04	0.03
0	0	0.47	0	t	1	halictid sp.	Lycaenopsis argiolus	33	0.03	0.03	0.01	0.04
0	1	0.69	0	t	1	Xylocopa virginica virginica	Euchlo creusa lotta	33	0.01	0.04	0.06	0.05
0	1	0.68	0	cv	1	Osmia atriventris	Nomada (Nomada) sayi	33	0.02	0.03	0.04	0.04
0	1	0.95	0	t	1	halictid sp.	Apis mellifera	33	0.03	0.03	0.08	0.05
1	0	0.39	0	t	1	Hepatica americana	Lassioglossum fuscipenne	33	0.02	0.05	0.03	0.05
0	1	0.84	0	t	1	Ephemoropsis laboriosa	Toxomerous geminatus	33	0.05	0.04	0.04	0.04
0	0	0.36	0	cv	1	Claytonia virginica	Andrena (Tomelissa) violae	33	0.02	0.05	0.02	0.03
1	0	0.24	0	t	1	Uvularia sessilifolia	Ceratina calcarata	33	0.01	0.04	0.01	0.04
0	1	0.76	0	t	1	Nomada (Gnathias) perplexa	Bombus bimaculatus	33	0.04	0.05	0.04	0.04
0	1	0.50	0	t	1	Viola papilionacea	Xylocopa virginica virginica	33	0.04	0.03	0.01	0.04
0	1	0.51	0	cv	1	Xylocopa virginica virginica	Andrena (Micrandrena) ziziaeformis	33	0.01	0.04	0.04	0.03
0	0	0.41	0	t	1	Andrena (Andrena) tridens	Osmia conjucta	33	0.03	0.05	0.01	0.04
0	0	0.47	0	t	1	Augochorella striata	Nomada (Gnathias) perplexa	33	0.01	0.04	0.04	0.05
1	0	0.43	0	t	1	Tiarella cordifolia collina	Nomada (Gnathias) perplexa	33	0.01	0.05	0.04	0.05

0	1	0.63	0	cv	1	Andrena (Micrandrena) ziziaeformis	Dialictus cressonii	33	0.04	0.03	0.02	0.04
0	0	0.27	0	t	1	Andrena (Simandrena) nasonii	Ceratina calcarata	33	0.02	0.05	0.01	0.04
0	1	0.54	0	t	1	Andrena (Euandrena) nigrihirta	Nomada (Heminomada) bishopii	33	0.03	0.05	0.02	0.04
0	0	0.32	0	t	1	Nomada sp.	Augochorella striata	33	0.02	0.04	0.01	0.04
0	1	0.80	0	cv	1	Hylema platura	Euchlo creusa lotta	33	0.02	0.04	0.06	0.05
0	1	0.54	0	t	1	unknown sp.	Aesculus sylvatica	33	0.02	0.05	0.04	0.04
0	1	0.60	0	t	1	Platycheirus obscurus	Toxomerous geminatus	33	0.02	0.04	0.04	0.04
1	0	0.38	0	t	1	Uvularia sessilifolia	Gonia sp.	33	0.01	0.04	0.03	0.04
0	1	0.66	0	cv	1	Aesculus sylvatica	Lassioglossum fuscipenne	33	0.04	0.04	0.03	0.05
0	1	0.87	0	t	1	Osmia sp.	Andrena (Melandrena) carlini	33	0.03	0.04	0.06	0.05
0	1	0.55	0	t	1	Platycheirus obscurus	Andrena (Euandrena) nigrihirta	33	0.02	0.04	0.03	0.05
0	1	0.81	0	t	1	Osmia atriventris	Euchlo creusa lotta	33	0.02	0.03	0.06	0.05
0	1	0.87	0	cv	1	Andrena (Melandrena) dunningi	Trillium catesbei	33	0.10	0.09	0.01	0.05
0	0	0.47	0	t	1	Cardamine angustata angustata	Dialictus cressonii	33	0.03	0.06	0.02	0.04
0	0	0.34	0	t	1	Platycheirus obscurus	Andrena (Scrapopteris) imatrix	33	0.02	0.04	0.01	0.04
0	0	0.45	0	t	1	Hylema platura	bombus pennsylvanicus	33	0.02	0.04	0.02	0.05
1	1	0.85	0	cv	1	Ephemoropsis laboriosa	Thalictrum thalictroides	33	0.05	0.04	0.04	0.04
0	1	0.77	0	t	1	Toxomerous geminatus	Bombus bimaculatus	33	0.04	0.04	0.04	0.04
0	0	0.50	0	t	1	Andrena (Trachandrena) ceanothi	Augochorella striata	33	0.04	0.04	0.01	0.04
0	1	0.55	0	t	1	Andrena (Scaphandrena) arabis	Bombus bimaculatus	33	0.02	0.05	0.04	0.04

0	0	0.22	0	cv	1	Andrena (Scaphandrena) arabis	Trillium catesbei	33	0.02	0.05	0.01	0.05
0	1	0.68	0	t	1	Toxomerous geminatus	Augochlora pura	33	0.04	0.04	0.03	0.05
0	1	0.80	0	t	1	Andrena (Micrandrena) ziziaeformis	Bombus bimaculatus	33	0.04	0.03	0.04	0.04
0	0	0.43	0	t	1	Osmia atriventris	Bombylius major	33	0.02	0.03	0.02	0.04
0	0	0.38	0	cv	1	Bombylius major	bombus pennsylvanicus	33	0.02	0.04	0.02	0.05
0	0	0.37	0	t	1	Gonia sp.	Osmia conjucta	33	0.03	0.04	0.01	0.04
0	0	0.27	0	t	1	Tiarella cordifolia collina	Sangiunaria canadensis	33	0.01	0.05	0.02	0.05
0	1	0.60	0	t	1	Andrena (Euandrena) nigrihirta	Gonia sp.	33	0.03	0.05	0.03	0.04
1	1	0.53	0	cv	1	Thalictrum thalictroides	Claytonia virginica	33	0.04	0.04	0.02	0.05
0	0	0.24	0	t	1	unknown sp.	Osmia conjucta	33	0.02	0.05	0.01	0.04
0	0	0.44	0	t	1	Toxomerous geminatus	Osmia lignaria	33	0.04	0.04	0.01	0.05
1	0	0.35	0	t	1	Hepatica americana	bombus pennsylvanicus	33	0.02	0.05	0.02	0.05
0	0	0.44	0	cv	1	Evylaeus macoupinensi	Lassioglossum fuscipenne	33	0.02	0.04	0.03	0.05
0	1	0.76	0	t	1	Dialictus abanci	Cardamine angustata angustata	33	0.05	0.04	0.03	0.06
0	1	0.55	0	t	1	unknown sp.	Bombus bimaculatus	33	0.02	0.05	0.04	0.04
0	1	0.65	0	t	1	Nomada sp.	Podophyllum peltatum	33	0.02	0.04	0.04	0.04
0	1	0.51	0	cv	1	Hylema platura	Cardamine angustata angustata	33	0.02	0.04	0.03	0.06
0	1	0.65	0	t	1	Platycheirus obscurus	Nomada (Nomada) sayi	33	0.02	0.04	0.04	0.04
0	1	0.98	0	t	1	Ephemoropsis laboriosa	Andrena (Micrandrena) personata	33	0.05	0.04	0.09	0.08
0	0	0.48	0	t	1	Andrena (Micrandrena) ziziaeformis	Osmia lignaria	33	0.04	0.03	0.01	0.05

0	0	0.25	0	cv	1	Andrena (Scaphandrena) arabis	Ceratina calcarata	33	0.02	0.05	0.01	0.04
0	0	0.47	0	t	1	halictid sp.	Andrena (Scrapopteris) imatrix	33	0.03	0.03	0.01	0.04
0	1	0.77	0	t	1	Andrena (Melandrena) carlini	Osmia simillima	33	0.06	0.05	0.01	0.03
0	0	0.46	0	t	1	Andrena (Simandrena) nasonii	Augochlora pura	33	0.02	0.05	0.03	0.05
0	1	0.64	0	cv	1	Dialictus cressonii	Aesculus sylvatica	33	0.02	0.04	0.04	0.04
0	1	0.65	0	t	1	Nomada sp.	Aesculus sylvatica	33	0.02	0.04	0.04	0.04
0	1	0.69	0	t	1	Osmia atriventris	Podophyllum peltatum	33	0.02	0.03	0.04	0.04
0	1	0.72	0	t	1	Andrena (Euandrena) nigrihirta	Andrena (Micrandrena) ziziaeformis	33	0.03	0.05	0.04	0.03
0	0	0.38	0	cv	1	Hylema platura	Osmia simillima	33	0.02	0.04	0.01	0.03
1	0	0.34	0	t	1	Uvularia sessilifolia	Hylema platura	33	0.01	0.04	0.02	0.04
0	1	0.80	0	t	1	Dialictus abanci	Andrena (Euandrena) nigrihirta	33	0.05	0.04	0.03	0.05
0	1	0.67	0	t	1	Ephemoropsis laboriosa	Andrena (Simandrena) nasonii	33	0.05	0.04	0.02	0.05
0	1	0.71	0	cv	1	unknown sp.	Euchlo creusa lotta	33	0.02	0.05	0.06	0.05
0	0	0.39	0	t	1	Osmia sp.	Ceratina calcarata	33	0.03	0.04	0.01	0.04
0	1	0.74	0	t	1	Ephemoropsis laboriosa	Platycheirus obscurus	33	0.05	0.04	0.02	0.04
0	0	0.25	0	t	1	Xylocopa virginica virginica	Andrena (Simandrena) nasonii	33	0.01	0.04	0.02	0.05
0	1	0.96	0	cv	1	Apis mellifera	Aesculus sylvatica	33	0.08	0.05	0.04	0.04
0	1	0.53	0	t	1	Augochorella striata	Aesculus sylvatica	33	0.01	0.04	0.04	0.04
0	0	0.34	0	t	1	Nomada (Heminomada) luteola	Lassioglossum fuscipenne	33	0.01	0.05	0.03	0.05
0	1	0.63	0	t	1	Ephemoropsis laboriosa	Osmia conjucta	33	0.05	0.04	0.01	0.04
0	1	0.69	0	cv	1	Osmia sp.	Bombus bimaculatus	33	0.03	0.04	0.04	0.04

0	0	0.25	0	t	1	Nomada (Heminomada) luteola	Claytonia virginica	33	0.01	0.05	0.02	0.05
0	0	0.30	0	t	1	Trillium catesbei	Andrena (Tomelissa) violae	33	0.01	0.05	0.02	0.03
0	1	0.86	0	t	1	Dialictus abanci	Aesculus sylvatica	33	0.05	0.04	0.04	0.04
0	1	0.64	0	cv	1	Gonia sp.	Nomada (Gnathias) perplexa	33	0.03	0.04	0.04	0.05
0	1	0.96	0	t	1	Osmia sp.	Andrena (Melandrena) dunningi	33	0.03	0.04	0.10	0.09
0	1	0.59	0	t	1	Andrena (Trachandrena) ceanothi	Stellaria pubera	33	0.04	0.04	0.02	0.05
1	0	0.47	0	t	1	Osmia atriventris	Andrena (Tomelissa) violae	33	0.02	0.03	0.02	0.03
0	0	0.32	0	cv	1	Andrena (Scrapopteris) imatrix	Sangiunaria canadensis	33	0.01	0.04	0.02	0.05
0	0	0.25	0	t	1	Andrena (Simandrena) nasonii	Augochorella striata	33	0.02	0.05	0.01	0.04
0	1	0.95	0	t	1	Andrena (Micrandrena) personata	Andrena (Trachandrena) ceanothi	33	0.09	0.08	0.04	0.04
1	1	0.78	0	t	1	Thalictrum thalictroides	Podophyllum peltatum	33	0.04	0.04	0.04	0.04
0	0	0.48	0	cv	1	Tiarella cordifolia collina	Andrena (Micrandrena) ziziaeformis	33	0.01	0.05	0.04	0.03
0	0	0.23	0	t	1	Xylocopa virginica virginica	Lycaenopsis argiolus	33	0.01	0.04	0.01	0.04
0	0	0.29	0	t	1	Nomada (Heminomada) luteola	Sangiunaria canadensis	33	0.01	0.05	0.02	0.05
1	0	0.31	0	t	1	Trillium catesbei	Nomada (Heminomada) bishopii	33	0.01	0.05	0.02	0.04
0	0	0.24	0	cv	1	Uvularia sessilifolia	Andrena (Scaphandrena) arabis	33	0.01	0.04	0.02	0.05
0	0	0.24	0	t	1	Claytonia virginica	Trillium catesbei	33	0.02	0.05	0.01	0.05
0	1	0.64	0	t	1	Viola papilionacea	Nomada (Heminomada) bishopii	33	0.04	0.03	0.02	0.04

0	0	0.48	0	t	1	Osmia atriventris	Dialictus cressonii	33	0.02	0.03	0.02	0.04
0	0	0.41	0	cv	1	Nomada sp.	Evylaeus macoupinensis	33	0.02	0.04	0.02	0.04
0	0	0.28	0	t	1	unknown sp.	Osmia simillima	33	0.02	0.05	0.01	0.03
0	1	0.59	0	t	1	Bombylius major	Andrena (Micrandrena) ziziaeformis	33	0.02	0.04	0.04	0.03
0	1	0.95	0	t	1	Osmia atriventris	Andrena (Melandrena) dunningi	33	0.02	0.03	0.10	0.09
0	1	0.83	0	cv	1	Euchlo creusa lotta	Augochlora pura	33	0.06	0.05	0.03	0.05
0	1	0.95	0	t	1	Andrena (Micrandrena) personata	Andrena (Micrandrena) ziziaeformis	33	0.09	0.08	0.04	0.03
0	1	0.81	0	t	1	Ephemoropsis laboriosa	Augochlora pura	33	0.05	0.04	0.03	0.05
0	1	0.60	0	t	1	Bombylius major	Aesculus sylvatica	33	0.02	0.04	0.04	0.04
0	1	0.50	0	cv	1	Nomada sp.	Osmia sp.	33	0.02	0.04	0.03	0.04
0	1	0.86	0	t	1	Andrena (Micrandrena) personata	Hepatica americana	33	0.09	0.08	0.02	0.05
0	0	0.20	0	t	1	Nomada (Heminomada) luteola	Osmia lignaria	33	0.01	0.05	0.01	0.05
0	1	0.90	0	t	1	Andrena (Micrandrena) personata	Nomada sp.	33	0.09	0.08	0.02	0.04
0	1	0.89	0	cv	1	Andrena (Micrandrena) personata	Andrena (Tomelissa) violae	33	0.09	0.08	0.02	0.03
0	0	0.24	0	t	1	Andrena (Scaphandrena) arabis	Osmia conjucta	33	0.02	0.05	0.01	0.04
0	0	0.33	0	t	1	Andrena (Scrapopteris) imatrix	bombus pennsylvanicus	33	0.01	0.04	0.02	0.05
0	1	0.54	0	t	1	Osmia atriventris	Andrena (Ptilandrena) erigeniae	33	0.02	0.03	0.03	0.04
0	1	0.67	0	cv	1	Hylema platura	Podophyllum peltatum	33	0.02	0.04	0.04	0.04
0	1	0.86	0	t	1	Nomada (Heminomada) luteola	Andrena (Micrandrena) personata	33	0.01	0.05	0.09	0.08
1	1	0.52	0	t	1	Thalictrum thalictroides	Osmia simillima	33	0.04	0.04	0.01	0.03

0	0	0.50	0	t	1	unknown sp.	Toxomerous geminatus	33	0.02	0.05	0.04	0.04
0	1	0.88	0	cv	1	Andrena (Andrena) tridens	Andrena (Melandrena) carlini	33	0.03	0.05	0.06	0.05
0	0	0.24	0	t	1	Augochorella striata	Ceratina calcarata	33	0.01	0.04	0.01	0.04
0	1	0.66	0	t	1	Cardamine angustata angustata	Andrena (Trachandren a) ceanothi	33	0.03	0.06	0.04	0.04
0	0	0.34	0	t	1	Hylema platura	Augochorella striata	33	0.02	0.04	0.01	0.04
1	1	0.97	1	cv	1	Solidago sempervivens	Halictus sp.	34	0.05	0.08	0.10	0.08
0	1	0.60	1	t	1	Solidago sempervivens	Agrotis ipsilon	34	0.05	0.08	0.03	0.07
1	1	0.97	1	t	1	Reseda luteola	Halictus sp.	34	0.05	0.08	0.10	0.08
1	1	0.87	1	t	1	Reseda luteola	Apis mellifera	34	0.05	0.08	0.07	0.08
0	1	0.92	1	cv	1	Colias crocea	Azorina vidalii	34	0.07	0.08	0.07	0.08
0	1	0.84	1	t	1	Colias crocea	Freesia refracta	34	0.07	0.08	0.05	0.09
0	1	0.92	1	t	1	Colias crocea	Lotus corniculatus	34	0.07	0.08	0.07	0.09
0	1	0.96	1	t	1	Sepsis thoracica	Azorina vidalii	34	0.08	0.07	0.07	0.08
0	1	0.93	1	cv	1	Sepsis thoracica	Silene vulgaris	34	0.08	0.07	0.05	0.08
0	1	0.98	1	t	1	Sepsis thoracica	Crithmum maritimum	34	0.08	0.07	0.08	0.08
0	1	0.84	1	t	1	Sepsis	Beta vulgaris	34	0.08	0.07	0.03	0.08
0	1	0.87	1	t	1	Eristalix tenax	Crithmum maritimum	34	0.03	0.07	0.08	0.08
1	1	0.99	1	cv	1	Chamomilla suaveolens	Halictus sp.	34	0.07	0.08	0.10	0.08
0	1	0.74	1	t	1	Chamomilla suaveolens	Anothomyia pluvialis	34	0.07	0.08	0.03	0.07
1	1	0.96	1	t	1	Chamomilla suaveolens	Musca domestica	34	0.07	0.08	0.08	0.08
1	1	0.98	1	t	1	Halictus sp.	Azorina vidalii	34	0.10	0.08	0.07	0.08
1	1	0.98	1	cv	1	Halictus sp.	Daucus carota	34	0.10	0.08	0.07	0.08
1	1	0.97	1	t	1	Halictus sp.	Silene vulgaris	34	0.10	0.08	0.05	0.08
1	1	0.99	1	t	1	Azorina vidalii	Bombus ruderatus	34	0.07	0.08	0.12	0.09
1	1	0.72	1	t	1	Azorina vidalii	Lasius niger	34	0.07	0.08	0.03	0.08
1	1	0.92	1	cv	1	Azorina vidalii	Apis mellifera	34	0.07	0.08	0.07	0.08
1	1	0.72	1	t	1	Azorina vidalii	Calliphora vemitoria	34	0.07	0.08	0.03	0.08

1	1	0.96	1	t	1	Azorina vidalii	Musca domestica	34	0.07	0.08	0.08	0.08
0	1	0.98	1	t	1	Bombus ruderatus	Freesia refracta	34	0.12	0.09	0.05	0.09
1	1	0.99	1	cv	1	Bombus ruderatus	Lotus corniculatus	34	0.12	0.09	0.07	0.09
0	1	0.88	1	t	1	Daucus carota	Lucilia sericata	34	0.07	0.08	0.05	0.07
1	1	0.96	1	t	1	Daucus carota	Musca domestica	34	0.07	0.08	0.08	0.08
1	1	0.93	1	t	1	Crithmum maritimum	Lucilia sericata	34	0.08	0.08	0.05	0.07
1	1	0.98	1	cv	1	Crithmum maritimum	Musca domestica	34	0.08	0.08	0.08	0.08
0	1	0.92	1	t	1	Apis mellifera	Lotus corniculatus	34	0.07	0.08	0.07	0.09
0	1	0.62	0	t	1	Lasius niger	Lucilia sericata	34	0.03	0.08	0.05	0.07
0	1	0.86	0	t	1	Solidago sempervivens	Lotus corniculatus	34	0.05	0.08	0.07	0.09
0	1	0.72	0	cv	1	Colias crocea	Calliphora vemitoria	34	0.07	0.08	0.03	0.08
0	1	0.96	0	t	1	Apis mellifera	Musca domestica	34	0.07	0.08	0.08	0.08
0	1	1.00	0	t	1	Halictus sp.	Bombus ruderatus	34	0.10	0.08	0.12	0.09
0	1	0.75	0	t	1	Daucus carota	Agrotis ipsilon	34	0.07	0.08	0.03	0.07
0	0	0.41	0	cv	1	Anothomyia pluvialis	Agrotis ipsilon	34	0.03	0.07	0.03	0.07
0	1	0.96	0	t	1	Colias crocea	Musca domestica	34	0.07	0.08	0.08	0.08
0	1	0.74	0	t	1	Daucus carota	Anothomyia pluvialis	34	0.07	0.08	0.03	0.07
0	1	0.78	0	t	1	Solidago sempervivens	Lucilia sericata	34	0.05	0.08	0.05	0.07
1	1	0.93	0	cv	1	Daucus carota	Apis mellifera	34	0.07	0.08	0.07	0.08
0	1	0.95	0	t	1	Bombus ruderatus	Lasius niger	34	0.12	0.09	0.03	0.08
0	0	0.42	0	t	1	Eristalix tenax	Beta vulgaris	34	0.03	0.07	0.03	0.08
0	1	0.93	0	t	1	Halictus sp.	Beta vulgaris	34	0.10	0.08	0.03	0.08
0	1	0.98	0	cv	1	Halictus sp.	Lotus corniculatus	34	0.10	0.08	0.07	0.09
0	1	0.72	0	t	1	Lasius niger	Lotus corniculatus	34	0.03	0.08	0.07	0.09
0	1	0.74	0	t	1	Colias crocea	Eristalix tenax	34	0.07	0.08	0.03	0.07
0	1	0.98	0	t	1	Halictus sp.	Apis mellifera	34	0.10	0.08	0.07	0.08
0	1	0.55	0	cv	1	Lasius niger	Freesia refracta	34	0.03	0.08	0.05	0.09

0	1	0.92	0	t	1	Daucus carota	Lotus corniculatus	34	0.07	0.08	0.07	0.09
0	0	0.40	0	t	1	Anothomyia pluvialis	Beta vulgaris	34	0.03	0.07	0.03	0.08
1	1	0.87	0	t	1	Solidago sempervivens	Apis mellifera	34	0.05	0.08	0.07	0.08
0	1	0.86	0	cv	1	Azorina vidalii	Silene vulgaris	34	0.07	0.08	0.05	0.08
0	1	0.59	0	t	1	Solidago sempervivens	Beta vulgaris	34	0.05	0.08	0.03	0.08
0	0	0.43	0	t	1	Eristalix tenax	Agrotis ipsilon	34	0.03	0.07	0.03	0.07
0	1	0.72	0	t	1	Calliphora vemitoria	Lotus corniculatus	34	0.03	0.08	0.07	0.09
0	1	0.87	0	cv	1	Colias crocea	Lucilia sericata	34	0.07	0.08	0.05	0.07
0	1	0.73	0	t	1	Colias crocea	Beta vulgaris	34	0.07	0.08	0.03	0.08
0	1	0.58	0	t	1	Silene vulgaris	Calliphora vemitoria	34	0.05	0.08	0.03	0.08
0	1	0.99	0	t	1	Bombus ruderatus	Apis mellifera	34	0.12	0.09	0.07	0.08
0	1	0.92	1	cv	1	Phelsuma ornata	Argusia argentea	35	0.10	0.08	0.03	0.08
0	1	0.87	1	t	1	Phelsuma ornata	Passiflora suberosa	35	0.10	0.08	0.02	0.08
0	1	0.88	1	t	1	Phelsuma ornata	Pemphis acidula	35	0.10	0.08	0.02	0.07
0	1	0.95	1	t	1	Phelsuma ornata	Dracaena concinna	35	0.10	0.08	0.04	0.08
0	1	0.95	1	cv	1	Phelsuma ornata	Leucaena leucocephala	35	0.10	0.08	0.04	0.08
1	1	0.99	1	t	1	Phelsuma ornata	Morinda citrifolia	35	0.10	0.08	0.07	0.08
1	1	0.99	1	t	1	Phelsuma ornata	Scaevola sericea	35	0.10	0.08	0.09	0.08
1	1	1.00	1	t	1	Phelsuma ornata	Gastonia mauritiana	35	0.10	0.08	0.11	0.08
1	1	0.97	1	cv	1	Phelsuma ornata	Hibiscus tiliaceus	35	0.10	0.08	0.05	0.08
0	1	0.93	1	t	1	Phelsuma ornata	Thespesia populnea	35	0.10	0.08	0.03	0.07
0	0	0.32	1	t	1	Argusia argentea	Syrphid sp.	35	0.03	0.08	0.02	0.07
0	0	0.45	1	t	1	Argusia argentea	Apis mellifera	35	0.03	0.08	0.03	0.08
0	0	0.43	1	cv	1	Argusia argentea	Musca domestica	35	0.03	0.08	0.03	0.08
0	0	0.31	1	t	1	Passiflora suberosa	Apis mellifera	35	0.02	0.08	0.03	0.08
1	1	0.97	1	t	1	Protaetia aurichalcea	Gastonia mauritiana	35	0.04	0.09	0.11	0.08

0	1	0.73	1	t	1	Suriana maritima	Megachile sp.	35	0.07	0.07	0.02	0.07
0	1	0.82	1	cv	1	Suriana maritima	Apis mellifera	35	0.07	0.07	0.03	0.08
0	1	0.93	1	t	1	Suriana maritima	Leptotes pirithous	35	0.07	0.07	0.05	0.08
0	1	0.57	1	t	1	Pemphis acidula	Stratiomydae sp.	35	0.02	0.07	0.05	0.08
0	0	0.33	1	t	1	Pemphis	Apis mellifera	35	0.02	0.07	0.03	0.08
0	1	0.70	1	cv	1	Pemphis acidula	Phalantha phalantha	35	0.02	0.07	0.06	0.06
0	1	0.57	1	t	1	Pemphis acidula	Leptotes pirithous	35	0.02	0.07	0.05	0.08
0	0	0.47	1	t	1	Pemphis acidula	Xylocopa fenestrata	35	0.02	0.07	0.04	0.07
1	1	0.97	1	t	1	Ornidia obesa	Gastonia mauritiana	35	0.04	0.09	0.11	0.08
0	0	0.44	1	cv	1	Dracaena concinna	Syrphid sp.	35	0.04	0.08	0.02	0.07
0	1	0.57	1	t	1	Dracaena concinna	Apis mellifera	35	0.04	0.08	0.03	0.08
0	1	0.84	1	t	1	Megachile sp.	Scaevola sericea	35	0.02	0.07	0.09	0.08
0	1	0.61	1	t	1	Megachile sp.	Turnera angustifolia	35	0.02	0.07	0.05	0.07
1	1	0.97	1	cv	1	Lucilia sp.	Gastonia mauritiana	35	0.04	0.09	0.11	0.08
0	1	0.70	1	t	1	Syrphid sp.	Stachytaphet a jamaicencis	35	0.02	0.07	0.06	0.07
0	1	0.62	1	t	1	Ipomoea macrantha	Apis mellifera	35	0.04	0.06	0.03	0.08
0	1	0.78	1	t	1	Leucaena leucocephala	Stratiomydae sp.	35	0.04	0.08	0.05	0.08
0	1	0.57	1	cv	1	Leucaena leucocephala	Apis mellifera	35	0.04	0.08	0.03	0.08
0	1	0.81	1	t	1	Morinda citrifolia	Apis mellifera	35	0.07	0.08	0.03	0.08
0	1	0.92	1	t	1	Morinda citrifolia	Leptotes pirithous	35	0.07	0.08	0.05	0.08
1	1	0.98	1	t	1	Stratiomydae sp.	Gastonia mauritiana	35	0.05	0.08	0.11	0.08
0	1	0.69	1	cv	1	Stratiomydae sp.	Thespesia populnea	35	0.05	0.08	0.03	0.07
0	1	0.91	1	t	1	Stratiomydae sp.	Stachytaphet a jamaicencis	35	0.05	0.08	0.06	0.07
0	1	0.87	1	t	1	Stratiomydae sp.	Turnera angustifolia	35	0.05	0.08	0.05	0.07
0	1	0.88	1	t	1	Scaevola sericea	Apis mellifera	35	0.09	0.08	0.03	0.08
0	1	0.93	1	cv	1	Scaevola sericea	Xylocopa fenestrata	35	0.09	0.08	0.04	0.07

0	1	0.95	1	t	1	Gastonia mauritiana	Apis mellifera	35	0.11	0.08	0.03	0.08
0	1	0.95	1	t	1	Gastonia mauritiana	Musca domestica	35	0.11	0.08	0.03	0.08
1	1	0.98	1	t	1	Gastonia mauritiana	Leptotes pirithous	35	0.11	0.08	0.05	0.08
0	1	0.68	1	cv	1	Apis mellifera	Hibiscus tiliaceus	35	0.03	0.08	0.05	0.08
0	0	0.46	1	t	1	Apis mellifera	Thespesia populnea	35	0.03	0.08	0.03	0.07
0	1	0.64	1	t	1	Thespesia populnea	Borbo borbonica	35	0.03	0.07	0.04	0.07
1	1	0.95	1	t	1	Phalantha phalantha	Stachytaphet a jamaicencis	35	0.06	0.06	0.06	0.07
1	1	0.88	1	cv	1	Borbo borbonica	Stachytaphet a jamaicencis	35	0.04	0.07	0.06	0.07
0	1	0.84	1	t	1	Borbo borbonica	Turnera angustifolia	35	0.04	0.07	0.05	0.07
0	1	0.91	1	t	1	Stachytapheta jamaicencis	Leptotes pirithous	35	0.06	0.07	0.05	0.08
0	1	0.82	1	t	1	Xylocopa fenestrata	Turnera angustifolia	35	0.04	0.07	0.05	0.07
0	1	0.77	0	cv	1	Dracaena concinna	Hibiscus tiliaceus	35	0.04	0.08	0.05	0.08
0	1	0.66	0	t	1	Musca domestica	Leptotes pirithous	35	0.03	0.08	0.05	0.08
0	1	0.93	0	t	1	Suriana maritima	Stratiomydae sp.	35	0.07	0.07	0.05	0.08
0	1	0.79	0	t	1	Argusia argentea	Phalantha phalantha	35	0.03	0.08	0.06	0.06
0	1	0.99	0	cv	1	Gastonia mauritiana	Stachytaphet a jamaicencis	35	0.11	0.08	0.06	0.07
0	1	0.76	0	t	1	Ornidia obesa	Leptotes pirithous	35	0.04	0.09	0.05	0.08
0	1	0.59	0	t	1	Leucaena leucocephala	Thespesia populnea	35	0.04	0.08	0.03	0.07
0	1	0.68	0	t	1	Hibiscus tiliaceus	Thespesia populnea	35	0.05	0.08	0.03	0.07
0	1	0.97	0	cv	1	Gastonia mauritiana	Borbo borbonica	35	0.11	0.08	0.04	0.07
0	1	0.61	0	t	1	Ipomoea macrantha	Musca domestica	35	0.04	0.06	0.03	0.08
0	1	0.73	0	t	1	Suriana maritima	Pemphis acidula	35	0.07	0.07	0.02	0.07
0	0	0.43	0	t	1	Passiflora suberosa	Leucaena leucocephala	35	0.02	0.08	0.04	0.08
0	0	0.48	0	cv	1	Passiflora suberosa	Ipomoea macrantha	35	0.02	0.08	0.04	0.06
0	1	0.89	0	t	1	Morinda citrifolia	Borbo borbonica	35	0.07	0.08	0.04	0.07

0	1	0.73	0	t	1	Suriana maritima	Syrphid sp.	35	0.07	0.07	0.02	0.07
0	1	0.74	0	t	1	Megachile sp.	Morinda citrifolia	35	0.02	0.07	0.07	0.08
0	1	0.96	0	cv	1	Suriana maritima	Phalantha phalantha	35	0.07	0.07	0.06	0.06
0	1	0.71	0	t	1	Protaetia aurichalcea	Borbo borbonica	35	0.04	0.09	0.04	0.07
0	1	0.65	0	t	1	Protaetia aurichalcea	Ornidia obesa	35	0.04	0.09	0.04	0.09
0	1	0.68	0	t	1	Protaetia aurichalcea	Xylocopa fenestrata	35	0.04	0.09	0.04	0.07
0	1	0.70	0	cv	1	Thespesia populnea	Leptotes pirithous	35	0.03	0.07	0.05	0.08
0	1	0.95	0	t	1	Ipomoea macrantha	Scaevola sericea	35	0.04	0.06	0.09	0.08
0	1	0.60	0	t	1	Borbo borbonica	Musca domestica	35	0.04	0.07	0.03	0.08
0	1	0.76	0	t	1	Protaetia aurichalcea	Stratiomydae sp.	35	0.04	0.09	0.05	0.08
1	1	0.97	0	cv	1	Phelsuma ornata	Stratiomydae sp.	35	0.10	0.08	0.05	0.08
0	1	0.87	0	t	1	Protaetia aurichalcea	Morinda citrifolia	35	0.04	0.09	0.07	0.08
0	1	0.81	0	t	1	Ipomoea macrantha	Hibiscus tiliaceus	35	0.04	0.06	0.05	0.08
0	1	0.90	0	t	1	Ipomoea macrantha	Morinda citrifolia	35	0.04	0.06	0.07	0.08
0	1	0.81	0	cv	1	Thespesia populnea	Phalantha phalantha	35	0.03	0.07	0.06	0.06
0	1	0.79	0	t	1	Leptotes pirithous	Xylocopa fenestrata	35	0.05	0.08	0.04	0.07
0	0	0.41	0	t	1	Passiflora suberosa	Ornidia obesa	35	0.02	0.08	0.04	0.09
0	1	0.56	0	t	1	Megachile sp.	Hibiscus tiliaceus	35	0.02	0.07	0.05	0.08
0	1	0.83	0	cv	1	Argusia argentea	Suriana maritima	35	0.03	0.08	0.07	0.07
0	1	0.88	0	t	1	Morinda citrifolia	Xylocopa fenestrata	35	0.07	0.08	0.04	0.07
0	1	0.95	0	t	1	Scaevola sericea	Hibiscus tiliaceus	35	0.09	0.08	0.05	0.08
0	0	0.23	0	t	1	Pemphis	Syrphid sp.	35	0.02	0.07	0.02	0.07
0	1	0.94	0	cv	1	Suriana maritima	Turnera angustifolia	35	0.07	0.07	0.05	0.07
0	1	0.61	0	t	1	Thespesia populnea	Xylocopa fenestrata	35	0.03	0.07	0.04	0.07
0	1	0.85	0	t	1	Protaetia aurichalcea	Phalantha phalantha	35	0.04	0.09	0.06	0.06
0	1	0.97	0	t	1	Gastonia mauritiana	Xylocopa fenestrata	35	0.11	0.08	0.04	0.07

0	1	0.54	0	cv	1	Ornidia obesa	Apis mellifera	35	0.04	0.09	0.03	0.08
0	1	0.50	0	t	1	Syrphid sp.	Borbo borbonica	35	0.02	0.07	0.04	0.07
0	1	0.57	0	t	1	Argusia argentea	Dracaena concinna	35	0.03	0.08	0.04	0.08
0	0	0.45	0	t	1	Thespesia populnea	Musca domestica	35	0.03	0.07	0.03	0.08
1	1	1.00	0	cv	1	Scaevola sericea	Gastonia mauritiana	35	0.09	0.08	0.11	0.08
0	0	0.32	0	t	1	Syrphid sp.	Musca domestica	35	0.02	0.07	0.03	0.08
0	1	0.89	0	t	1	Suriana maritima	Xylocopa fenestrata	35	0.07	0.07	0.04	0.07
0	0	0.46	0	t	1	Syrphid sp.	Leucaena leucocephala	35	0.02	0.07	0.04	0.08
1	1	0.97	0	cv	1	Phelsuma ornata	Leptotes pirithous	35	0.10	0.08	0.05	0.08
0	1	0.94	0	t	1	Scaevola sericea	Borbo borbonica	35	0.09	0.08	0.04	0.07
0	1	0.67	0	t	1	Protaetia aurichalcea	Leucaena leucocephala	35	0.04	0.09	0.04	0.08
0	1	0.83	0	t	1	Passiflora suberosa	Scaevola sericea	35	0.02	0.08	0.09	0.08
0	0	0.30	1	cv	1	Calliphoridae genus 3	Xysmalobium gerrardii	36	0.01	0.03	0.01	0.04
0	0	0.19	1	t	1	Other wasps	Aspidonepsis diploglossa	36	0.01	0.06	0.01	0.06
0	0	0.22	1	t	1	Other wasps	Xysmalobium gerrardii	36	0.01	0.06	0.01	0.04
0	0	0.09	1	t	1	Other wasps	Asclepias woodii	36	0.01	0.06	0.01	0.12
0	0	0.32	1	cv	1	Other wasps	Xysmalobium involucratum	36	0.01	0.06	0.02	0.03
0	0	0.43	1	t	1	Other Diptera	Xysmalobium gerrardii	36	0.03	0.06	0.01	0.04
0	0	0.21	1	t	1	Other Diptera	Asclepias woodii	36	0.03	0.06	0.01	0.12
0	1	0.69	1	t	1	Other Diptera	Pachycarpus natalensis	36	0.03	0.06	0.04	0.05
0	0	0.43	1	cv	1	Other Diptera	Miraglossum verticillare	36	0.03	0.06	0.01	0.04
0	0	0.30	1	t	1	Bibionidae	Xysmalobium gerrardii	36	0.01	0.03	0.01	0.04
0	0	0.05	1	t	1	Curculionidae sp. 2	Asclepias woodii	36	0.01	0.10	0.01	0.12
1	0	0.32	1	t	1	Hemipepsis hilaris	Sisyranthus trichostomus	36	0.01	0.04	0.01	0.03
0	0	0.28	1	cv	1	Hemipepsis hilaris	Xysmalobium gerrardii	36	0.01	0.04	0.01	0.04
0	1	0.54	1	t	1	Hemipepsis hilaris	Pachycarpus natalensis	36	0.01	0.04	0.04	0.05

1	0	0.39	1	t	1	Hemipepsis hilaris	Xysmalobium involucratum	36	0.01	0.04	0.02	0.03
0	0	0.32	1	t	1	Hemipepsis hilaris	Miraglossum pilosum	36	0.01	0.04	0.01	0.03
0	0	0.29	1	cv	1	Hemipepsis hilaris	Miraglossum verticillare	36	0.01	0.04	0.01	0.04
0	1	0.61	1	t	1	Cantharidae sp.	Xysmalobium gerrardii	36	0.04	0.04	0.01	0.04
1	0	0.45	1	t	1	Sisyranthus trichostomus	Calliphoridae genus 2	36	0.01	0.03	0.02	0.03
1	0	0.38	1	t	1	Sisyranthus trichostomus	Arge sp.	36	0.01	0.03	0.01	0.02
1	0	0.32	1	cv	1	Sisyranthus trichostomus	Aspilocoryph us fasciativentris	36	0.01	0.03	0.01	0.04
0	0	0.26	1	t	1	Sisyranthus trichostomus	Cyrtothyrea marginalis	36	0.01	0.03	0.01	0.06
1	0	0.42	1	t	1	Sisyranthus trichostomus	Apis mellifera	36	0.01	0.03	0.02	0.03
1	1	0.60	1	t	1	Sisyranthus trichostomus	Unidentified butterfly	36	0.01	0.03	0.03	0.03
1	0	0.36	1	cv	1	Sisyranthus trichostomus	Curculionidae sp. 1	36	0.01	0.03	0.01	0.02
0	0	0.38	1	t	1	Sisyranthus trichostomus	Atrichelaphin is tigrina	36	0.01	0.03	0.02	0.05
1	0	0.38	1	t	1	Sisyranthus trichostomus	Diptera sp. 3	36	0.01	0.03	0.01	0.02
1	0	0.36	1	t	1	Sisyranthus trichostomus	Chrysomelida e sp. 2	36	0.01	0.03	0.01	0.02
1	0	0.38	1	cv	1	Sisyranthus trichostomus	Coleoptera sp. 8	36	0.01	0.03	0.01	0.02
0	0	0.32	1	t	1	Scarabaeinae sp. 2	Xysmalobium gerrardii	36	0.01	0.03	0.01	0.04
1	0	0.43	1	t	1	Scarabaeinae sp. 2	Xysmalobium involucratum	36	0.01	0.03	0.02	0.03
0	1	0.77	1	t	1	Empididae sp. 2	Xysmalobium gerrardii	36	0.07	0.06	0.01	0.04
0	0	0.40	1	cv	1	Calliphoridae genus 2	Xysmalobium gerrardii	36	0.02	0.03	0.01	0.04
0	0	0.24	1	t	1	Aspidonepsis diploglossa	Other solitary bees	36	0.01	0.06	0.02	0.06
0	0	0.45	1	t	1	Aspidonepsis diploglossa	Chloropidae	36	0.01	0.06	0.03	0.04
0	1	0.78	1	t	1	Aspidonepsis diploglossa	Halictidae sp. 1	36	0.01	0.06	0.08	0.07
0	0	0.32	1	cv	1	Aspidonepsis diploglossa	Apis mellifera	36	0.01	0.06	0.02	0.03
0	0	0.30	1	t	1	Aspidonepsis diploglossa	Musca domestica	36	0.01	0.06	0.02	0.04

0	0	0.22	1	t	1	Aspidonepsis diploglossa	Muscidae genus 2	36	0.01	0.06	0.01	0.04
0	1	0.67	1	t	1	Xysmalobium gerrardii	Calliphoridae genus 1	36	0.01	0.04	0.05	0.04
0	0	0.30	1	cv	1	Xysmalobium gerrardii	Coleoptera sp. 3	36	0.01	0.04	0.01	0.03
0	0	0.30	1	t	1	Xysmalobium gerrardii	Other Heteroptera	36	0.01	0.04	0.01	0.03
0	1	0.53	1	t	1	Xysmalobium gerrardii	Pentatomoidea sp.	36	0.01	0.04	0.03	0.03
0	0	0.30	1	t	1	Xysmalobium gerrardii	Microphthalma sp. 2	36	0.01	0.04	0.01	0.03
0	0	0.28	1	cv	1	Xysmalobium gerrardii	Aspilocoryphus fasciativentris	36	0.01	0.04	0.01	0.04
0	1	0.70	1	t	1	Xysmalobium gerrardii	Tachinidae genus 2	36	0.01	0.04	0.05	0.05
0	0	0.23	1	t	1	Xysmalobium gerrardii	Cyrtothyrea marginalis	36	0.01	0.04	0.01	0.06
0	0	0.24	1	t	1	Xysmalobium gerrardii	Sarcophaga sp.	36	0.01	0.04	0.01	0.05
0	0	0.50	1	cv	1	Xysmalobium gerrardii	Lygaeidae sp. 2	36	0.01	0.04	0.03	0.04
1	1	0.51	1	t	1	Xysmalobium gerrardii	Chloropidae	36	0.01	0.04	0.03	0.04
0	0	0.37	1	t	1	Xysmalobium gerrardii	Apis mellifera	36	0.01	0.04	0.02	0.03
0	0	0.30	1	t	1	Xysmalobium gerrardii	Pompilidae sp. 2	36	0.01	0.04	0.01	0.03
0	0	0.30	1	cv	1	Xysmalobium gerrardii	Coreidae sp.	36	0.01	0.04	0.01	0.03
0	0	0.46	1	t	1	Xysmalobium gerrardii	Lycidae sp.	36	0.01	0.04	0.03	0.03
0	0	0.31	1	t	1	Xysmalobium gerrardii	Curculionidae sp. 1	36	0.01	0.04	0.01	0.02
0	0	0.31	1	t	1	Xysmalobium gerrardii	Other Coleoptera	36	0.01	0.04	0.01	0.03
0	0	0.30	1	cv	1	Xysmalobium gerrardii	Scarabaeinae sp. 1	36	0.01	0.04	0.01	0.03
0	0	0.33	1	t	1	Xysmalobium gerrardii	Atrichelaphinis tigrina	36	0.01	0.04	0.02	0.05
0	0	0.40	1	t	1	Xysmalobium gerrardii	Other bees	36	0.01	0.04	0.02	0.03
0	0	0.39	1	t	1	Xysmalobium gerrardii	Actea sp.	36	0.01	0.04	0.02	0.03
0	0	0.31	1	cv	1	Xysmalobium gerrardii	Sepsidae sp. 1	36	0.01	0.04	0.01	0.03
0	0	0.39	1	t	1	Xysmalobium gerrardii	Tachinidae subfamily Goniinae	36	0.01	0.04	0.02	0.03

0	0	0.36	1	t	1	Xysmalobium gerrardii	Musca domestica	36	0.01	0.04	0.02	0.04
0	0	0.31	1	t	1	Xysmalobium gerrardii	Chrysomelidae sp. 2	36	0.01	0.04	0.01	0.02
0	0	0.30	1	cv	1	Xysmalobium gerrardii	Scarabaeinae sp. 3	36	0.01	0.04	0.01	0.03
1	0	0.33	1	t	1	Unidentified micromoth	Asclepias cucullata	36	0.01	0.03	0.01	0.04
0	1	0.86	1	t	1	Diptera sp. 22	Pachycarpus natalensis	36	0.05	0.05	0.04	0.05
0	1	0.84	1	t	1	Tiphia sp.	Asclepias woodii	36	0.13	0.13	0.01	0.12
0	0	0.11	1	cv	1	Asclepias woodii	Other solitary bees	36	0.01	0.12	0.02	0.06
0	0	0.08	1	t	1	Asclepias woodii	Cyrtothyrea marginalis	36	0.01	0.12	0.01	0.06
0	0	0.09	1	t	1	Asclepias woodii	Sarcophaga sp.	36	0.01	0.12	0.01	0.05
0	0	0.13	1	t	1	Asclepias woodii	Atrichelaphinis tigrina	36	0.01	0.12	0.02	0.05
0	1	0.94	1	cv	1	Dacus sp.	Pachycarpus natalensis	36	0.08	0.06	0.04	0.05
0	1	0.53	1	t	1	Aspilocoryphus	Pachycarpus natalensis	36	0.01	0.04	0.04	0.05
1	0	0.39	1	t	1	Aspilocoryphus	Xysmalobium involucratum	36	0.01	0.04	0.02	0.03
0	0	0.29	1	t	1	Aspilocoryphus	Asclepias cucullata	36	0.01	0.04	0.01	0.04
0	0	0.28	1	cv	1	Aspilocoryphus	Miraglossum verticillare	36	0.01	0.04	0.01	0.04
0	1	0.53	1	t	1	Other solitary bees	Pachycarpus natalensis	36	0.02	0.06	0.04	0.05
0	1	0.80	1	t	1	Microphthalma sp. 1	Pachycarpus natalensis	36	0.04	0.05	0.04	0.05
1	0	0.45	1	t	1	Sepsidae sp. 2	Xysmalobium involucratum	36	0.01	0.02	0.02	0.03
0	0	0.23	1	cv	1	Cyrtothyrea marginalis	Miraglossum verticillare	36	0.01	0.06	0.01	0.04
0	0	0.48	1	t	1	Sarcophaga sp.	Pachycarpus natalensis	36	0.01	0.05	0.04	0.05
0	0	0.34	1	t	1	Sarcophaga sp.	Xysmalobium involucratum	36	0.01	0.05	0.02	0.03
0	0	0.24	1	t	1	Sarcophaga sp.	Miraglossum verticillare	36	0.01	0.05	0.01	0.04
0	1	0.74	1	cv	1	Lygaeidae sp. 2	Pachycarpus natalensis	36	0.03	0.04	0.04	0.05
1	1	0.61	1	t	1	Lygaeidae sp. 2	Xysmalobium involucratum	36	0.03	0.04	0.02	0.03
1	0	0.49	1	t	1	Lygaeidae sp. 2	Miraglossum verticillare	36	0.03	0.04	0.01	0.04

0	0	0.47	1	t	1	Pachycarpus natalensis	Spilostethus rivularis	36	0.04	0.05	0.01	0.05
0	1	0.65	1	cv	1	Pachycarpus natalensis	Empididae sp. 1	36	0.04	0.05	0.03	0.05
0	1	0.66	1	t	1	Pachycarpus natalensis	Chrysomelidae sp. 1	36	0.04	0.05	0.03	0.04
0	0	0.47	1	t	1	Pachycarpus natalensis	Homoecerus annulatus	36	0.04	0.05	0.01	0.05
0	0	0.41	1	t	1	Empididae sp. 1	Asclepias cucullata	36	0.03	0.05	0.01	0.04
1	1	0.54	1	cv	1	Xysmalobium involucratum	Chrysomelidae sp. 1	36	0.02	0.03	0.03	0.04
1	0	0.44	1	t	1	Xysmalobium involucratum	Elateridae sp.	36	0.02	0.03	0.01	0.02
1	0	0.43	1	t	1	Xysmalobium involucratum	Curculionidae sp. 1	36	0.02	0.03	0.01	0.02
1	0	0.42	1	t	1	Xysmalobium involucratum	Other Coleoptera	36	0.02	0.03	0.01	0.03
0	0	0.44	1	cv	1	Xysmalobium involucratum	Atrichelaphinis tigrina	36	0.02	0.03	0.02	0.05
1	1	0.52	1	t	1	Xysmalobium involucratum	Other bees	36	0.02	0.03	0.02	0.03
1	0	0.42	1	t	1	Xysmalobium involucratum	Sepsidae sp. 1	36	0.02	0.03	0.01	0.03
1	0	0.43	1	t	1	Xysmalobium involucratum	Chrysomelidae sp. 2	36	0.02	0.03	0.01	0.02
0	1	0.61	1	cv	1	Asclepias cucullata	Halictidae sp. 2	36	0.01	0.04	0.04	0.03
0	0	0.33	1	t	1	Atrichelaphinis tigrina	Miraglossum verticillare	36	0.02	0.05	0.01	0.04
0	0	0.48	1	t	1	Sepsidae sp. 3	Miraglossum verticillare	36	0.03	0.02	0.01	0.04
0	1	0.96	0	t	1	Dacus sp.	Microphthalma sp. 1	36	0.08	0.06	0.04	0.05
0	0	0.16	0	cv	1	Asclepias woodii	Xysmalobium involucratum	36	0.01	0.12	0.02	0.03
0	1	0.94	0	t	1	Tiphia sp.	Microphthalma sp. 2	36	0.13	0.13	0.01	0.03
0	1	0.54	0	t	1	Lygaeidae sp. 2	Curculionidae sp. 1	36	0.03	0.04	0.01	0.02
1	1	0.66	0	t	1	Pentatomoidea sp.	Other bees	36	0.03	0.03	0.02	0.03
0	1	0.98	0	cv	1	Tiphia sp.	Halictidae sp. 2	36	0.13	0.13	0.04	0.03
0	0	0.37	0	t	1	Coleoptera sp. 3	Atrichelaphinis tigrina	36	0.01	0.03	0.02	0.05
0	0	0.37	0	t	1	Coleoptera sp. 3	Diptera sp. 3	36	0.01	0.03	0.01	0.02
0	1	0.93	0	t	1	Empididae sp. 2	Microphthalma sp. 1	36	0.07	0.06	0.04	0.05
0	1	0.70	0	cv	1	Diptera sp. 22	Coreidae sp.	36	0.05	0.05	0.01	0.03

0	1	0.52	0	t	1	Lygaeidae sp. 2	Scarabaeinae sp. 1	36	0.03	0.04	0.01	0.03
0	1	0.81	0	t	1	Calliphoridae genus 2	Tachinidae genus 2	36	0.02	0.03	0.05	0.05
0	0	0.43	0	t	1	Sepsidae sp. 2	Musca domestica	36	0.01	0.02	0.02	0.04
1	0	0.33	0	cv	1	Asclepias cucullata	Other Coleoptera	36	0.01	0.04	0.01	0.03
0	0	0.43	0	t	1	Calliphoridae genus 2	Coleoptera sp. 3	36	0.02	0.03	0.01	0.03
1	0	0.46	0	t	1	Other Coleoptera	Other bees	36	0.01	0.03	0.02	0.03
0	1	0.62	0	t	1	Unidentified butterfly	Atrichelaphinis tigrina	36	0.03	0.03	0.02	0.05
0	1	0.73	0	cv	1	Calliphoridae genus 1	Musca domestica	36	0.05	0.04	0.02	0.04
0	0	0.41	0	t	1	Other Coleoptera	Musca domestica	36	0.01	0.03	0.02	0.04
0	0	0.27	0	t	1	Aspidonepsis diploglossa	Sepsidae sp. 2	36	0.01	0.06	0.01	0.02
0	0	0.40	0	t	1	Elateridae sp.	Atrichelaphinis tigrina	36	0.01	0.02	0.02	0.05
0	0	0.43	0	cv	1	Calliphoridae genus 2	Scarabaeinae sp. 3	36	0.02	0.03	0.01	0.03
0	0	0.28	0	t	1	Microphthalma sp. 2	Homoecerus annulatus	36	0.01	0.03	0.01	0.05
0	1	0.70	0	t	1	Bibionidae	Calliphoridae genus 1	36	0.01	0.03	0.05	0.04
0	0	0.41	0	t	1	Coleoptera sp. 3	Xysmalobium involucratum	36	0.01	0.03	0.02	0.03
0	1	0.65	0	cv	1	Other wasps	Tachinidae genus 2	36	0.01	0.06	0.05	0.05
0	0	0.20	0	t	1	Aspidonepsis diploglossa	Homoecerus annulatus	36	0.01	0.06	0.01	0.05
0	0	0.50	0	t	1	Other Diptera	Elateridae sp.	36	0.03	0.06	0.01	0.02
0	1	0.57	0	t	1	Chloropidae	Atrichelaphinis tigrina	36	0.03	0.04	0.02	0.05
0	1	0.99	0	cv	1	Diptera sp. 22	Tiphia sp.	36	0.05	0.05	0.13	0.13
0	1	0.56	0	t	1	Calliphoridae genus 3	Pachycarpus natalensis	36	0.01	0.03	0.04	0.05
0	1	0.96	0	t	1	Calliphoridae genus 1	Halictidae sp. 1	36	0.05	0.04	0.08	0.07
0	0	0.35	0	t	1	Calliphoridae genus 3	Curculionidae sp. 1	36	0.01	0.03	0.01	0.02
1	0	0.40	0	cv	1	Arge sp.	Sepsidae sp. 1	36	0.01	0.02	0.01	0.03
1	1	0.57	0	t	1	Pentatomoidea sp.	Other Coleoptera	36	0.03	0.03	0.01	0.03
0	0	0.45	0	t	1	Cyrtothyrea marginalis	Chloropidae	36	0.01	0.06	0.03	0.04

0	0	0.28	0	t	1	Sisyranthus trichostomus	Spilostethus rivularis	36	0.01	0.03	0.01	0.05
0	1	0.58	0	cv	1	Cantharidae sp.	Homoecerus annulatus	36	0.04	0.04	0.01	0.05
0	1	0.96	0	t	1	Tiphia sp.	Tachinidae subfamily Goniinae	36	0.13	0.13	0.02	0.03
0	0	0.31	0	t	1	Hemipepsis hilaris	Microphthalma sp. 2	36	0.01	0.04	0.01	0.03
0	0	0.13	0	t	1	Curculionidae sp. 2	Xysmalobium gerrardii	36	0.01	0.10	0.01	0.04
0	1	0.54	0	cv	1	Lygaeidae sp. 2	Other Coleoptera	36	0.03	0.04	0.01	0.03
0	0	0.41	0	t	1	Xysmalobium gerrardii	Empididae sp. 1	36	0.01	0.04	0.03	0.05
0	1	0.66	0	t	1	Cantharidae sp.	Other Coleoptera	36	0.04	0.04	0.01	0.03
0	0	0.49	0	t	1	Other solitary bees	Sepsidae sp. 3	36	0.02	0.06	0.03	0.02
0	0	0.33	0	cv	1	Calliphoridae genus 3	Scarabaeinae sp. 1	36	0.01	0.03	0.01	0.03
0	1	0.58	0	t	1	Unidentified micromoth	Pachycarpus natalensis	36	0.01	0.03	0.04	0.05
0	0	0.39	0	t	1	Apis mellifera	Asclepias cucullata	36	0.02	0.03	0.01	0.04
0	0	0.36	0	t	1	Other wasps	Chrysomelidae sp. 1	36	0.01	0.06	0.03	0.04
1	0	0.34	0	cv	1	Calliphoridae genus 3	Sisyranthus trichostomus	36	0.01	0.03	0.01	0.03
0	0	0.38	0	t	1	Curculionidae sp. 2	Microphthalma sp. 1	36	0.01	0.10	0.04	0.05
0	0	0.12	0	t	1	Asclepias woodii	Miraglossum pilosum	36	0.01	0.12	0.01	0.03
0	0	0.45	0	t	1	Chrysomelidae sp. 1	Miraglossum pilosum	36	0.03	0.04	0.01	0.03
0	0	0.41	0	cv	1	Coleoptera sp. 3	Apis mellifera	36	0.01	0.03	0.02	0.03
0	0	0.12	0	t	1	Curculionidae sp. 2	Sarcophaga sp.	36	0.01	0.10	0.01	0.05
0	0	0.40	0	t	1	Sepsidae sp. 2	Atrichelaphinis tigrina	36	0.01	0.02	0.02	0.05
0	1	0.55	0	t	1	Unidentified butterfly	Miraglossum verticillare	36	0.03	0.03	0.01	0.04
0	1	0.79	0	cv	1	Microphthalma sp. 1	Chloropidae	36	0.04	0.05	0.03	0.04
0	0	0.32	0	t	1	Hemipepsis hilaris	Other Coleoptera	36	0.01	0.04	0.01	0.03
0	0	0.31	0	t	1	Spilostethus rivularis	Coleoptera sp. 8	36	0.01	0.05	0.01	0.02
0	0	0.37	0	t	1	Coleoptera sp. 3	Arge sp.	36	0.01	0.03	0.01	0.02

0	0	0.36	0	cv	1	Microphthalma sp. 2	Elateridae sp.	36	0.01	0.03	0.01	0.02
0	1	0.90	0	t	1	Empididae sp.	Chloropidae	36	0.07	0.06	0.03	0.04
0	0	0.40	0	t	1	Empididae sp. 1	Miraglossum verticillare	36	0.03	0.05	0.01	0.04
0	1	0.95	0	t	1	Dacus sp.	Halictidae sp. 2	36	0.08	0.06	0.04	0.03
0	0	0.33	0	cv	1	Hemipepsis hilaris	Unidentified micromoth	36	0.01	0.04	0.01	0.03
0	0	0.42	0	t	1	Hemipepsis hilaris	Empididae sp. 1	36	0.01	0.04	0.03	0.05
0	1	0.67	0	t	1	Diptera sp. 22	Miraglossum verticillare	36	0.05	0.05	0.01	0.04
0	1	0.92	0	t	1	Chloropidae	Halictidae sp. 1	36	0.03	0.04	0.08	0.07
0	1	0.70	0	cv	1	Xysmalobium gerrardii	Diptera sp. 22	36	0.01	0.04	0.05	0.05
0	1	0.80	0	t	1	Empididae sp. 2	Sepsidae sp. 1	36	0.07	0.06	0.01	0.03
0	0	0.13	0	t	1	Microphthalma sp. 2	Asclepias woodii	36	0.01	0.03	0.01	0.12
0	0	0.29	0	t	1	Microphthalma sp. 2	Muscidae genus 2	36	0.01	0.03	0.01	0.04
0	1	0.85	0	cv	1	Scarabaeinae sp. 2	Halictidae sp. 1	36	0.01	0.03	0.08	0.07
0	1	0.55	0	t	1	Pentatomoidea sp.	Coreidae sp.	36	0.03	0.03	0.01	0.03
0	1	0.81	0	t	1	Pentatomoidea sp.	Microphthalma sp. 1	36	0.03	0.03	0.04	0.05
0	1	0.55	0	t	1	Chloropidae	Other Coleoptera	36	0.03	0.04	0.01	0.03
0	0	0.32	0	cv	1	Other Coleoptera	Miraglossum verticillare	36	0.01	0.03	0.01	0.04
0	1	0.66	0	t	1	Cantharidae sp.	Unidentified micromoth	36	0.04	0.04	0.01	0.03
0	0	0.27	0	t	1	Other wasps	Curculionidae sp. 1	36	0.01	0.06	0.01	0.02
1	1	0.58	0	t	1	Sepsidae sp. 2	Lygaeidae sp. 2	36	0.01	0.02	0.03	0.04
1	1	0.62	0	cv	1	Tachinidae subfamily Goniinae	Sepsidae sp. 3	36	0.02	0.03	0.03	0.02
1	1	0.89	0	t	1	Diptera sp. 22	Microphthalma sp. 1	36	0.05	0.05	0.04	0.05
0	1	0.60	0	t	1	Chloropidae	Musca domestica	36	0.03	0.04	0.02	0.04
1	1	0.84	0	t	1	Calliphoridae genus 1	Chloropidae	36	0.05	0.04	0.03	0.04
0	1	0.69	0	cv	1	Apis mellifera	Halictidae sp. 2	36	0.02	0.03	0.04	0.03

0	1	0.95	0	t	1	Tiphia sp.	Sepsidae sp. 2	36	0.13	0.13	0.01	0.02
0	1	0.90	0	t	1	Empididae sp. 2	Lygaeidae sp. 2	36	0.07	0.06	0.03	0.04
0	0	0.32	0	t	1	Calliphoridae genus 3	Asclepias cucullata	36	0.01	0.03	0.01	0.04
0	0	0.35	0	cv	1	Unidentified micromoth	Microphthalma sp. 2	36	0.01	0.03	0.01	0.03
0	1	0.96	0	t	1	Tiphia sp.	Apis mellifera	36	0.13	0.13	0.02	0.03
0	1	0.80	0	t	1	Other Diptera	Calliphoridae genus 1	36	0.03	0.06	0.05	0.04
0	0	0.49	0	t	1	Pachycarpus natalensis	Muscidae genus 2	36	0.04	0.05	0.01	0.04
0	1	0.70	0	cv	1	Calliphoridae genus 1	Elateridae sp.	36	0.05	0.04	0.01	0.02
0	0	0.43	0	t	1	Calliphoridae genus 2	Scarabaeinae sp. 1	36	0.02	0.03	0.01	0.03
0	1	0.79	0	t	1	Empididae sp.	Coreidae sp.	36	0.07	0.06	0.01	0.03
0	1	0.50	0	t	1	Coreidae sp.	Lycidae sp.	36	0.01	0.03	0.03	0.03
0	1	0.64	0	cv	1	Unidentified micromoth	Halictidae sp. 2	36	0.01	0.03	0.04	0.03
0	1	0.82	0	t	1	Halictidae sp. 1	Other Coleoptera	36	0.08	0.07	0.01	0.03
0	1	0.77	0	t	1	Chloropidae	Unidentified butterfly	36	0.03	0.04	0.03	0.03
0	0	0.37	0	t	1	Calliphoridae genus 3	Arge sp.	36	0.01	0.03	0.01	0.02
1	1	0.67	0	cv	1	Unidentified butterfly	Actea sp.	36	0.03	0.03	0.02	0.03
0	0	0.45	0	t	1	Coleoptera sp. 3	Empididae sp. 1	36	0.01	0.03	0.03	0.05
0	1	0.58	1	t	1	Aeschynomene pratensis	Megachile sp.	37	0.03	0.04	0.02	0.04
1	0	0.48	1	t	1	Aeschynomene pratensis	Eumenes sp.	37	0.03	0.04	0.01	0.04
0	0	0.48	1	cv	1	Aeschynomene pratensis	Exomalopsis sp.	37	0.03	0.04	0.01	0.04
0	1	0.66	1	t	1	Aeschynomene pratensis	Polybia occidentalis	37	0.03	0.04	0.03	0.06
1	0	0.43	1	t	1	Dialictus sp.	Sauvagesia rubiginosa	37	0.03	0.06	0.02	0.04
1	0	0.35	1	t	1	Dialictus sp.	Pterolepis glomerata	37	0.03	0.06	0.01	0.04
1	1	0.92	1	cv	1	Dialictus sp.	Ludwigia nervosa	37	0.03	0.06	0.08	0.05
1	1	0.85	1	t	1	Pereirapis sp. 2	Synqonanthus caulescens	37	0.03	0.04	0.06	0.05
1	1	0.77	1	t	1	Pereirapis sp. 2	Saggitaria quyanensis	37	0.03	0.04	0.05	0.04
1	1	0.65	1	t	1	Pereirapis sp. 2	Ludwigia decurrens	37	0.03	0.04	0.03	0.03

1	1	0.55	1	cv	1	Cyclocephala gravis	Montricharida arborescens	37	0.01	0.02	0.03	0.02
1	1	0.65	1	t	1	Rhynchanthera serrulata	Palpada pusio	37	0.02	0.02	0.03	0.03
1	1	0.59	1	t	1	Rhynchanthera serrulata	Melipona favosa	37	0.02	0.02	0.03	0.03
1	1	0.54	1	t	1	Rhynchanthera serrulata	Pompeius amblyspoila	37	0.02	0.02	0.02	0.02
1	0	0.27	1	cv	1	Helicobia morionella	Tyttnera scabra	37	0.01	0.06	0.02	0.04
1	1	0.85	1	t	1	Helicobia morionella	Ludwigia nervosa	37	0.01	0.06	0.08	0.05
1	1	0.80	1	t	1	Euhibus sp.	Synqonanthus caulescens	37	0.02	0.05	0.06	0.05
1	1	0.58	1	t	1	Campsomeris dorsata	Desmodium barbatum	37	0.01	0.04	0.04	0.04
0	0	0.29	1	cv	1	Campsomeris dorsata	Cuphea o'donellii	37	0.01	0.04	0.01	0.03
1	1	0.55	1	t	1	Phaenica sp.	Clidemia capitellata	37	0.01	0.02	0.03	0.02
1	1	0.52	1	t	1	Palpada pusio	Hyptis dilatata	37	0.03	0.03	0.01	0.04
0	1	0.53	1	t	1	Palpada pusio	Besmoscelis villosa	37	0.03	0.03	0.01	0.03
1	1	0.67	1	cv	1	Palpada pusio	Croton hirtus	37	0.03	0.03	0.03	0.04
1	0	0.40	1	t	1	Euglossa cordata	Besmoscelis villosa	37	0.02	0.03	0.01	0.03
1	0	0.39	1	t	1	Euglossa cordata	Pterolepis glomerata	37	0.02	0.03	0.01	0.04
1	1	0.67	1	t	1	Sarcophagula occidua	Caperonia pallustris	37	0.01	0.05	0.05	0.04
1	0	0.41	1	cv	1	Urbanus dorantes dorantes	Cuphea o'donellii	37	0.03	0.06	0.01	0.03
1	0	0.37	1	t	1	Urbanus dorantes dorantes	Diodia multiflora	37	0.03	0.06	0.01	0.05
1	0	0.47	1	t	1	Urbanus dorantes dorantes	Melochia villosa	37	0.03	0.06	0.02	0.05
1	1	0.93	1	t	1	Urbanus dorantes dorantes	Ludwigia nervosa	37	0.03	0.06	0.08	0.05
1	0	0.31	1	cv	1	Cogia calchas	Cuphea o'donellii	37	0.01	0.03	0.01	0.03
1	0	0.22	1	t	1	Eurema nise	Cuphea o'donellii	37	0.01	0.06	0.01	0.03
1	1	0.85	1	t	1	Eurema nise	Ludwigia nervosa	37	0.01	0.06	0.08	0.05

1	0	0.33	1	t	1	Augochlora sp.	Besmoscelis villosa	37	0.01	0.03	0.01	0.03
0	1	0.52	1	cv	1	Toxomerus marginatus	Tyttnera scabra	37	0.03	0.03	0.02	0.04
1	0	0.44	1	t	1	Toxomerus marginatus	Pterolepis glomerata	37	0.03	0.03	0.01	0.04
1	1	0.68	1	t	1	Toxomerus marginatus	Ludwigia decurrens	37	0.03	0.03	0.03	0.03
0	0	0.45	1	t	1	Melipona favosa	Mimosa camporum	37	0.03	0.03	0.01	0.04
0	0	0.47	1	cv	1	Melipona favosa	Besmoscelis villosa	37	0.03	0.03	0.01	0.03
1	1	0.80	1	t	1	Desmodium barbatum	Auhochloropsis vesta	37	0.04	0.04	0.04	0.04
1	1	0.72	1	t	1	Desmodium barbatum	Anthidiellum sp.	37	0.04	0.04	0.03	0.04
0	0	0.30	1	t	1	Hyptis conferta	Polistes subsericeus	37	0.01	0.03	0.02	0.06
0	0	0.47	1	cv	1	Hyptis	Cerceris sp.	37	0.01	0.03	0.03	0.03
0	0	0.39	1	t	1	Hyptis	Apis mellifera	37	0.01	0.03	0.03	0.06
0	0	0.48	1	t	1	Hyptis	Ceratina sp.	37	0.01	0.03	0.03	0.03
0	0	0.32	1	t	1	Hyptis conferta	Omicrom ruficolle	37	0.01	0.03	0.01	0.03
1	1	0.89	1	cv	1	Augochlora sp. 3	Ludwigia nervosa	37	0.02	0.06	0.08	0.05
1	0	0.28	1	t	1	Eumenes sp.	Shultesia brachyptera	37	0.01	0.04	0.01	0.04
1	1	0.87	1	t	1	Dialictus sp. 7	Saggitaria quyanensis	37	0.04	0.04	0.05	0.04
1	1	0.54	1	t	1	Thalia geniculata	Eulaema nigrita	37	0.03	0.02	0.01	0.02
0	0	0.40	1	cv	1	Cuphea o'donellii	Junonia evarete	37	0.01	0.03	0.02	0.03
0	1	0.53	1	t	1	Cuphea o'donellii	Pyrgus oileus orcus	37	0.01	0.03	0.03	0.03
0	0	0.29	1	t	1	Shultesia brachyptera	Polybia ignobilis	37	0.01	0.04	0.01	0.04
0	0	0.37	1	t	1	Shultesia brachyptera	Apis mellifera	37	0.01	0.04	0.03	0.06
1	1	0.93	1	cv	1	Xylocopa fibriata	Ludwigia nervosa	37	0.03	0.06	0.08	0.05
0	0	0.41	1	t	1	Tyttnera scabra	Toxomerus floralis	37	0.02	0.04	0.02	0.05
0	0	0.36	1	t	1	Tyttnera scabra	Oxyssarcodexia peltata	37	0.02	0.04	0.02	0.06
0	0	0.28	1	t	1	Polistes subsericeus	Hyptis dilatata	37	0.02	0.06	0.01	0.04
1	1	0.89	1	cv	1	Polistes subsericeus	Ludwigia nervosa	37	0.02	0.06	0.08	0.05
0	0	0.33	1	t	1	Diodia multiflora	Apis mellifera	37	0.01	0.05	0.03	0.06

1	0	0.40	1	t	1	Amazilia tovasi	Heliconia psittacorum	37	0.01	0.02	0.01	0.02
1	0	0.48	1	t	1	Amazilia tovasi	Sinningia sp.	37	0.01	0.02	0.02	0.02
0	1	0.61	1	cv	1	Parancistrocerus sp.	Hyptis dilatata	37	0.04	0.04	0.01	0.04
0	1	0.53	1	t	1	Xyris laxifolia	Toxomerus floralis	37	0.03	0.04	0.02	0.05
0	1	0.57	1	t	1	Xyris laxifolia	Augochlorensis callichroa	37	0.03	0.04	0.03	0.06
1	1	0.81	1	t	1	Toxomerus floralis	Synqonanthus caulescens	37	0.02	0.05	0.06	0.05
1	0	0.41	1	cv	1	Toxomerus floralis	Sauvagesia rubiginosa	37	0.02	0.05	0.02	0.04
1	1	0.71	1	t	1	Toxomerus floralis	Saggitaria quyanensis	37	0.02	0.05	0.05	0.04
1	0	0.30	1	t	1	Toxomerus floralis	Xyris savanensis	37	0.02	0.05	0.01	0.04
1	1	0.82	1	t	1	Synqonanthus caulescens	Apis mellifera	37	0.06	0.05	0.03	0.06
0	0	0.28	1	cv	1	Sphex melanopus	Hyptis dilatata	37	0.02	0.06	0.01	0.04
1	1	0.89	1	t	1	Sphex melanopus	Ludwigia nervosa	37	0.02	0.06	0.08	0.05
0	0	0.47	1	t	1	Hyptis dilatata	Cerceris sp.	37	0.01	0.04	0.03	0.03
0	1	0.63	1	t	1	Hyptis dilatata	Pepsis sp.	37	0.01	0.04	0.04	0.04
0	0	0.29	1	cv	1	Hyptis dilatata	Polybia ignobilis	37	0.01	0.04	0.01	0.04
1	1	0.53	1	t	1	Hyptis dilatata	Pyrgus oileus orcus	37	0.01	0.04	0.03	0.03
0	0	0.38	1	t	1	Hyptis dilatata	Augochlorensis callichroa	37	0.01	0.04	0.03	0.06
0	0	0.31	1	t	1	Hyptis dilatata	Extemnius sp.	37	0.01	0.04	0.01	0.03
1	1	0.67	1	cv	1	Miconia stephananther	Urbanus simplicius	37	0.03	0.04	0.03	0.03
1	1	0.76	1	t	1	Miconia stephananther	Auhochloropsis vesta	37	0.03	0.04	0.04	0.04
0	1	0.61	1	t	1	Sauvagesia rubiginosa	Pyrgus oileus orcus	37	0.02	0.04	0.03	0.03
0	0	0.34	1	t	1	Sauvagesia rubiginosa	Heliopetes arsalte	37	0.02	0.04	0.01	0.04
1	1	0.63	1	cv	1	Clidemia capitellata	Augochloropsis sp.	37	0.03	0.02	0.02	0.02
0	0	0.42	1	t	1	Melochia villosa	Augochlorensis callichroa	37	0.02	0.05	0.03	0.06
0	0	0.31	1	t	1	Melochia villosa	Heliopetes arsalte	37	0.02	0.05	0.01	0.04
0	0	0.36	1	t	1	Mimosa camporum	Apis mellifera	37	0.01	0.04	0.03	0.06
0	0	0.43	1	cv	1	Mimosa camporum	Polybia occidentalis	37	0.01	0.04	0.03	0.06

0	0	0.39	1	t	1	Besmoscelis villosa	Apis mellifera	37	0.01	0.03	0.03	0.06
0	0	0.39	1	t	1	Besmoscelis villosa	Augochlorensis callichroa	37	0.01	0.03	0.03	0.06
1	1	0.87	1	t	1	Caperonia pallustris	Auhochloropsis vesta	37	0.05	0.04	0.04	0.04
1	1	0.81	1	cv	1	Caperonia pallustris	Polybia occidentalis	37	0.05	0.04	0.03	0.06
1	1	0.92	1	t	1	Apis mellifera	Ludwigia nervosa	37	0.03	0.06	0.08	0.05
1	0	0.33	1	t	1	Apis mellifera	Xyris savanensis	37	0.03	0.06	0.01	0.04
1	1	0.70	1	t	1	Auhochloropsis vesta	Croton hirtus	37	0.04	0.04	0.03	0.04
0	0	0.38	1	cv	1	Pterolepis glomerata	Augochlorensis callichroa	37	0.01	0.04	0.03	0.06
0	0	0.31	1	t	1	Pterolepis glomerata	Pachodunerus brevithorax	37	0.01	0.04	0.01	0.03
1	1	0.92	1	t	1	Augochlorensis callichroa	Ludwigia nervosa	37	0.03	0.06	0.08	0.05
1	0	0.33	1	t	1	Augochlorensis callichroa	Xyris savanensis	37	0.03	0.06	0.01	0.04
1	1	0.63	1	cv	1	Chrysagria duodecimpuntata	Ludwigia decurrens	37	0.02	0.03	0.03	0.03
1	1	0.89	1	t	1	Athidium sp.	Ludwigia nervosa	37	0.02	0.06	0.08	0.05
1	0	0.25	1	t	1	Athidium sp.	Xyris savanensis	37	0.02	0.06	0.01	0.04
1	1	0.89	1	t	1	Tabanus sp.	Ludwigia nervosa	37	0.02	0.06	0.08	0.05
1	1	0.56	1	cv	1	Heliconia psittacorum	Amazilia fibriata	37	0.01	0.02	0.03	0.02
1	1	0.87	1	t	1	Ludwigia nervosa	Ornidia obesa	37	0.08	0.05	0.02	0.06
1	1	0.82	1	t	1	Ludwigia nervosa	Trigona angustula	37	0.08	0.05	0.01	0.06
1	1	0.93	1	t	1	Ludwigia nervosa	Polybia occidentalis	37	0.08	0.05	0.03	0.06
1	1	0.87	1	cv	1	Ludwigia nervosa	Oxyssarcodexia peltata	37	0.08	0.05	0.02	0.06
0	0	0.43	1	t	1	Ornidia obesa	Croton hirtus	37	0.02	0.06	0.03	0.04
1	1	0.64	1	t	1	Sinningia sp.	Amazilia fibriata	37	0.02	0.02	0.03	0.02
1	0	0.48	0	t	1	Augochlora sp.	Toxomerus marginatus	37	0.01	0.03	0.03	0.03
0	0	0.35	0	cv	1	Helicobia morionella	Polybia occidentalis	37	0.01	0.06	0.03	0.06
0	1	0.50	0	t	1	Rhynchanthera serrulata	Augochlorensis callichroa	37	0.02	0.02	0.03	0.06

0	0	0.28	0	t	1	Athidium sp.	Ornidia obesa	37	0.02	0.06	0.02	0.06
1	1	0.81	0	t	1	Miconia stephananther	Pepsis sp.	37	0.03	0.04	0.04	0.04
0	0	0.48	0	cv	1	Xyris laxifolia	Athidium sp.	37	0.03	0.04	0.02	0.06
0	0	0.33	0	t	1	Eumenes sp.	Heliconia psittacorum	37	0.01	0.04	0.01	0.02
0	0	0.49	0	t	1	Montricharida arborescens	Athidium sp.	37	0.03	0.02	0.02	0.06
0	1	0.50	0	t	1	Cuphea o'donellii	Amazilia fibriata	37	0.01	0.03	0.03	0.02
0	1	0.62	0	cv	1	Urbanus dorantes dorantes	Montricharid a arborescens	37	0.03	0.06	0.03	0.02
0	0	0.41	0	t	1	Sarcophagula occidua	Urbanus simplicius	37	0.01	0.05	0.03	0.03
0	1	0.51	0	t	1	Euhibus sp.	Ceratina sp.	37	0.02	0.05	0.03	0.03
0	1	0.65	0	t	1	Xyris laxifolia	Polybia occidentalis	37	0.03	0.04	0.03	0.06
1	0	0.27	0	cv	1	Sphex melanopus	Mimosa camporum	37	0.02	0.06	0.01	0.04
0	0	0.43	0	t	1	Besmoscelis villosa	Augochlorops is sp.	37	0.01	0.03	0.02	0.02
0	1	0.94	0	t	1	Ludwigia nervosa	Amazilia fibriata	37	0.08	0.05	0.03	0.02
0	0	0.34	0	t	1	Campsomeris dorsata	Amazilia tovasi	37	0.01	0.04	0.01	0.02
0	1	0.74	0	cv	1	Diodia multiflora	Synqonanthu s caulescens	37	0.01	0.05	0.06	0.05
1	0	0.25	0	t	1	Tabanus sp.	Xyris savanensis	37	0.02	0.06	0.01	0.04
0	0	0.49	0	t	1	Campsomeris dorsata	Montricharid a arborescens	37	0.01	0.04	0.03	0.02
0	0	0.35	0	t	1	Shultesia brachyptera	Tyttnera scabra	37	0.01	0.04	0.02	0.04
0	0	0.40	0	cv	1	Sarcophagula occidua	Croton hirtus	37	0.01	0.05	0.03	0.04
0	0	0.36	0	t	1	Dialictus sp.	Cogia calchas	37	0.03	0.06	0.01	0.03
0	1	0.59	0	t	1	Amazilia tovasi	Ludwigia decurrens	37	0.01	0.02	0.03	0.03
0	1	0.51	0	t	1	Tabanus sp.	Ludwigia decurrens	37	0.02	0.06	0.03	0.03
0	1	0.56	0	cv	1	Desmodium barbatum	Augochlora sp. 3	37	0.04	0.04	0.02	0.06
0	1	0.55	0	t	1	Euglossa cordata	Polybia occidentalis	37	0.02	0.03	0.03	0.06
0	0	0.26	0	t	1	Shultesia brachyptera	Heliopetes arsalte	37	0.01	0.04	0.01	0.04
0	1	0.50	0	t	1	Clidemia capitellata	Pachoduneru s brevithorax	37	0.03	0.02	0.01	0.03

0	0	0.48	0	cv	1	Euhibus sp.	Polybia occidentalis	37	0.02	0.05	0.03	0.06
0	0	0.23	0	t	1	Sarcophagula occidua	Diodia multiflora	37	0.01	0.05	0.01	0.05
0	0	0.35	0	t	1	Euglossa cordata	Sarcophagula occidua	37	0.02	0.03	0.01	0.05
0	1	0.57	0	t	1	Xylocopa fibriata	Polybia occidentalis	37	0.03	0.06	0.03	0.06
0	0	0.28	0	cv	1	Hyptis conferta	Heliopetes arsalte	37	0.01	0.03	0.01	0.04
0	0	0.42	0	t	1	Sauvagesia rubiginosa	Melochia villosa	37	0.02	0.04	0.02	0.05
0	0	0.27	0	t	1	Sarcophagula occidua	Extemnius sp.	37	0.01	0.05	0.01	0.03
0	1	0.56	0	t	1	Pyrgus oileus orcus	Heliconia psittacorum	37	0.03	0.03	0.01	0.02
0	0	0.42	0	cv	1	Polybia	Sinningia sp.	37	0.01	0.04	0.02	0.02
0	1	0.70	0	t	1	Montricharida arborescens	Xyris laxifolia	37	0.03	0.02	0.03	0.04
0	0	0.30	0	t	1	Euglossa cordata	Eurema nise	37	0.02	0.03	0.01	0.06
0	0	0.30	0	t	1	Campsomeris dorsata	Hyptis conferta	37	0.01	0.04	0.01	0.03
1	0	0.32	0	cv	1	Augochlora sp. 3	Melochia villosa	37	0.02	0.06	0.02	0.05
0	1	0.80	0	t	1	Clidemia capitellata	Pepsis sp.	37	0.03	0.02	0.04	0.04
0	0	0.28	0	t	1	Tabanus sp.	Oxyssarcodexia peltata	37	0.02	0.06	0.02	0.06
1	1	0.62	0	t	1	Palpada pusio	Chrysagria duodecimpuntata	37	0.03	0.03	0.02	0.03
0	1	0.61	0	cv	1	Euhibus sp.	Desmodium barbatum	37	0.02	0.05	0.04	0.04
0	1	0.51	0	t	1	Athidium sp.	Ludwigia decurrens	37	0.02	0.06	0.03	0.03
1	0	0.39	0	t	1	Phaenica sp.	Amazilia tovasi	37	0.01	0.02	0.01	0.02
0	1	0.64	0	t	1	Desmodium barbatum	Tyttnera scabra	37	0.04	0.04	0.02	0.04
0	0	0.34	0	cv	1	Melochia villosa	Pterolepis glomerata	37	0.02	0.05	0.01	0.04
0	0	0.45	0	t	1	Diodia multiflora	Amazilia fibriata	37	0.01	0.05	0.03	0.02
0	0	0.37	0	t	1	Eurema nise	Ceratina sp.	37	0.01	0.06	0.03	0.03
0	0	0.46	0	t	1	Melipona favosa	Sphex melanopus	37	0.03	0.03	0.02	0.06
1	1	0.51	0	cv	1	Toxomerus floralis	Cerceris sp.	37	0.02	0.05	0.03	0.03
1	1	0.70	0	t	1	Thalia geniculata	Amazilia fibriata	37	0.03	0.02	0.03	0.02

0	1	0.96	0	t	1	Palpada pusio	Ludwigia nervosa	37	0.03	0.03	0.08	0.05
0	0	0.35	0	t	1	Cuphea o'donellii	Melochia villosa	37	0.01	0.03	0.02	0.05
0	0	0.44	0	cv	1	Dialictus sp.	Augochlorensis callichroa	37	0.03	0.06	0.03	0.06
0	1	0.51	0	t	1	Pyrgus oileus orcus	Oxyssarcodexia peltata	37	0.03	0.03	0.02	0.06
0	0	0.49	0	t	1	Dialictus sp.	Sinningia sp.	37	0.03	0.06	0.02	0.02
0	0	0.35	0	t	1	Phaenica sp.	Ornidia obesa	37	0.01	0.02	0.02	0.06
1	0	0.39	0	cv	1	Pereirapis sp. 2	Mimosa camporum	37	0.03	0.04	0.01	0.04
0	1	0.55	0	t	1	Euhibus sp.	Miconia stephananthera	37	0.02	0.05	0.03	0.04
0	0	0.41	0	t	1	Rhynchanthera serrulata	Pterolepis glomerata	37	0.02	0.02	0.01	0.04
0	0	0.43	0	t	1	Pterolepis glomerata	Sinningia sp.	37	0.01	0.04	0.02	0.02
1	1	0.57	0	cv	1	Eurema nise	Saggitaria quyanensis	37	0.01	0.06	0.05	0.04
1	1	0.77	0	t	1	Polistes subsericeus	Synqonanthus caulescens	37	0.02	0.06	0.06	0.05
0	0	0.40	0	t	1	Mimosa camporum	Pompeius amblyspoila	37	0.01	0.04	0.02	0.02
1	0	0.33	0	t	1	Eumenes sp.	Melochia villosa	37	0.01	0.04	0.02	0.05
0	0	0.28	0	cv	1	Cuphea o'donellii	Mimosa camporum	37	0.01	0.03	0.01	0.04
0	1	0.56	0	t	1	Miconia stephananther	Heliconia psittacorum	37	0.03	0.04	0.01	0.02
0	1	0.55	0	t	1	Aeschynomene pratensis	Amazilia tovasi	37	0.03	0.04	0.01	0.02
0	1	0.78	0	t	1	Melipona favosa	Pepsis sp.	37	0.03	0.03	0.04	0.04
0	1	0.63	0	cv	1	Cuphea o'donellii	Pepsis sp.	37	0.01	0.03	0.04	0.04
1	1	0.68	0	t	1	Urbanus simplicius	Pyrgus oileus orcus	37	0.03	0.03	0.03	0.03
0	1	0.82	0	t	1	Clidemia capitellata	Saggitaria quyanensis	37	0.03	0.02	0.05	0.04
1	1	0.64	0	t	1	Pyrgus oileus orcus	Pompeius amblyspoila	37	0.03	0.03	0.02	0.02
0	0	0.30	0	cv	1	Cogia calchas	Shultesia brachyptera	37	0.01	0.03	0.01	0.04
0	1	0.89	0	t	1	Exomalopsis sp.	Ludwigia nervosa	37	0.01	0.04	0.08	0.05
0	0	0.50	0	t	1	Rhynchanthera serrulata	Sauvagesia rubiginosa	37	0.02	0.02	0.02	0.04

0	0	0.36	0	t	1	Helicobia morionella	Cerceris sp.	37	0.01	0.06	0.03	0.03
0	1	0.57	0	cv	1	Aeschynomene pratensis	Tyttnera scabra	37	0.03	0.04	0.02	0.04
0	1	0.55	0	t	1	Urbanus dorantes dorantes	Sinningia sp.	37	0.03	0.06	0.02	0.02
0	1	0.90	0	t	1	Besmoscelis villosa	Ludwigia nervosa	37	0.01	0.03	0.08	0.05
0	1	0.68	0	t	1	Cogia calchas	Saggitaria quyanensis	37	0.01	0.03	0.05	0.04
0	1	0.66	0	cv	1	Aeschynomene pratensis	Toxomerus marginatus	37	0.03	0.04	0.03	0.03
0	0	0.19	0	t	1	Sarcophagula occidua	Trigona angustula	37	0.01	0.05	0.01	0.06
0	0	0.32	0	t	1	Sphex melanopus	Heliconia psittacorum	37	0.02	0.06	0.01	0.02
0	0	0.34	0	t	1	Euhibus sp.	Augochlora sp.	37	0.02	0.05	0.01	0.03
1	1	0.63	0	cv	1	Montricharida arborescens	Sinningia sp.	37	0.03	0.02	0.02	0.02
1	1	0.81	0	t	1	Synqonanthus caulescens	Augochlorensis callichroa	37	0.06	0.05	0.03	0.06
1	1	0.69	0	t	1	Palpada pusio	Cerceris sp.	37	0.03	0.03	0.03	0.03
0	1	0.57	0	t	1	Hyptis conferta	Auhochloropsis vesta	37	0.01	0.03	0.04	0.04
0	0	0.37	0	cv	1	Hyptis dilatata	Sauvagesia rubiginosa	37	0.01	0.04	0.02	0.04
1	1	0.63	0	t	1	Clidemia capitellata	Sinningia sp.	37	0.03	0.02	0.02	0.02
0	0	0.33	0	t	1	Euhibus sp.	Pachodunerus brevithorax	37	0.02	0.05	0.01	0.03
1	0	0.41	0	t	1	Rhynchanthera serrulata	Hyptis dilatata	37	0.02	0.02	0.01	0.04
0	0	0.33	0	cv	1	Helicobia morionella	Sinningia sp.	37	0.01	0.06	0.02	0.02
0	0	0.49	0	t	1	Thalia geniculata	Oxyssarcodexia peltata	37	0.03	0.02	0.02	0.06
1	0	0.20	0	t	1	Helicobia morionella	Xyris savanensis	37	0.01	0.06	0.01	0.04
0	0	0.34	0	t	1	Megachile sp.	Xyris savanensis	37	0.02	0.04	0.01	0.04
1	0	0.48	0	cv	1	Ceratina sp.	Pachodunerus brevithorax	37	0.03	0.03	0.01	0.03
0	1	0.51	0	t	1	Thalia geniculata	Omicrom ruficolle	37	0.03	0.02	0.01	0.03
0	0	0.39	0	t	1	Xyris laxifolia	Trigona angustula	37	0.03	0.04	0.01	0.06
0	0	0.49	0	t	1	Exomalopsis sp.	Amazilia fibriata	37	0.01	0.04	0.03	0.02

0	0	0.22	0	cv	1	Helicobia morionella	Pachoduneru s brevithorax	37	0.01	0.06	0.01	0.03
0	1	0.50	0	t	1	Urbanus dorantes dorantes	Apis mellifera	37	0.03	0.06	0.03	0.06
0	0	0.36	0	t	1	Sphex melanopus	Apis mellifera	37	0.02	0.06	0.03	0.06
1	1	0.56	0	t	1	Junonia	Croton hirtus	37	0.02	0.03	0.03	0.04
0	0	0.40	1	cv	1	Erylaeus macroupinensi	Isopyrum biternatum	39	0.02	0.11	0.06	0.10
0	0	0.40	1	t	1	Augochlora pura	Isopyrum biternatum	39	0.02	0.11	0.06	0.10
0	0	0.18	1	t	1	Bombus greseocollis	Dicentra canadensis	39	0.02	0.11	0.03	0.08
0	0	0.10	1	t	1	Bombus greseocollis	Dicentra cucullaria	39	0.02	0.11	0.02	0.09
0	1	0.70	1	cv	1	Bombus greseocollis	Dentaria laciniata	39	0.02	0.11	0.09	0.10
1	1	0.95	1	t	1	Bombus greseocollis	Claytonia virginica	39	0.02	0.11	0.14	0.11
1	1	0.98	1	t	1	Dialictus obscurus	Claytonia virginica	39	0.04	0.12	0.14	0.11
0	1	0.60	1	t	1	Surphus torvus	Isopyrum biternatum	39	0.04	0.11	0.06	0.10
0	1	0.86	1	cv	1	Surphus torvus	Dentaria laciniata	39	0.04	0.11	0.09	0.10
1	1	0.98	1	t	1	Surphus torvus	Claytonia virginica	39	0.04	0.11	0.14	0.11
0	0	0.36	1	t	1	Andrena forbesii	Isopyrum biternatum	39	0.02	0.12	0.06	0.10
0	0	0.08	1	t	1	Andrena forbesii	Erythronium albidum	39	0.02	0.12	0.02	0.10
1	1	0.94	1	cv	1	Andrena forbesii	Claytonia virginica	39	0.02	0.12	0.14	0.11
0	1	0.69	1	t	1	Andrena miserabilis	Isopyrum biternatum	39	0.05	0.11	0.06	0.10
1	1	0.99	1	t	1	Andrena miserabilis	Claytonia virginica	39	0.05	0.11	0.14	0.11
1	1	0.98	1	t	1	Andrena irgeniae	Isopyrum biternatum	39	0.13	0.11	0.06	0.10
0	1	0.91	1	cv	1	Andrena irgeniae	Erythronium albidum	39	0.13	0.11	0.02	0.10
1	1	1.00	1	t	1	Andrena irgeniae	Dentaria laciniata	39	0.13	0.11	0.09	0.10
1	1	1.00	1	t	1	Andrena irgeniae	Claytonia virginica	39	0.13	0.11	0.14	0.11
0	0	0.35	1	t	1	Isopyrum biternatum	Andrena rugosa	39	0.06	0.10	0.02	0.12
0	0	0.38	1	cv	1	Isopyrum biternatum	Andrena personata	39	0.06	0.10	0.02	0.11

0	0	0.38	1	t	1	Isopyrum biternatum	Xylota nemorum	39	0.06	0.10	0.02	0.11
0	0	0.38	1	t	1	Isopyrum biternatum	Dialictus imitatus	39	0.06	0.10	0.02	0.11
0	1	0.60	1	t	1	Isopyrum biternatum	Andrena mansonii	39	0.06	0.10	0.04	0.11
0	1	0.61	1	cv	1	Isopyrum biternatum	Metasyrphus americanus	39	0.06	0.10	0.04	0.11
0	0	0.48	1	t	1	Isopyrum biternatum	Andrena cressoni	39	0.06	0.10	0.03	0.11
0	0	0.38	1	t	1	Isopyrum biternatum	Dialictus oblongus	39	0.06	0.10	0.02	0.11
0	1	0.70	1	t	1	Isopyrum biternatum	Andrena carlini	39	0.06	0.10	0.05	0.11
0	0	0.38	1	cv	1	Isopyrum biternatum	Augochlorella striata	39	0.06	0.10	0.02	0.11
0	0	0.38	1	t	1	Isopyrum biternatum	Dialictus zephyrus	39	0.06	0.10	0.02	0.11
0	0	0.35	1	t	1	Isopyrum biternatum	Helophilus fasciatus	39	0.06	0.10	0.02	0.12
0	1	0.60	1	t	1	Isopyrum biternatum	Melanostoma sp.	39	0.06	0.10	0.04	0.11
0	0	0.20	1	cv	1	Erythronium albidum	Andrena mansonii	39	0.02	0.10	0.04	0.11
1	0	0.10	1	t	1	Erythronium albidum	Andrena erythronii	39	0.02	0.10	0.02	0.10
0	0	0.28	1	t	1	Erythronium albidum	Andrena carlini	39	0.02	0.10	0.05	0.11
1	1	0.94	1	t	1	Lasioglossum forbesii	Claytonia virginica	39	0.02	0.12	0.14	0.11
0	0	0.45	1	cv	1	Sanguinaria canadensis	Metasyrphus americanus	39	0.04	0.09	0.04	0.11
0	1	0.69	1	t	1	Andrena rugosa	Dentaria laciniata	39	0.02	0.12	0.09	0.10
1	1	0.94	1	t	1	Andrena rugosa	Claytonia virginica	39	0.02	0.12	0.14	0.11
1	1	0.98	1	t	1	Ceratina metallica	Claytonia virginica	39	0.04	0.12	0.14	0.11
0	1	0.79	1	cv	1	Eristalis dimidiatus	Dentaria laciniata	39	0.03	0.12	0.09	0.10
1	1	0.97	1	t	1	Eristalis dimidiatus	Claytonia virginica	39	0.03	0.12	0.14	0.11
1	1	0.98	1	t	1	Andrena mansonii	Claytonia virginica	39	0.04	0.11	0.14	0.11
0	1	0.79	1	t	1	Syrphus sp.	Dentaria laciniata	39	0.03	0.12	0.09	0.10
1	1	0.97	1	cv	1	Syrphus sp.	Claytonia virginica	39	0.03	0.12	0.14	0.11
1	1	0.98	1	t	1	Metasyrphus americanus	Claytonia virginica	39	0.04	0.11	0.14	0.11

0	1	0.70	1	t	1	Halictus confusus	Dentaria laciniata	39	0.02	0.11	0.09	0.10
1	1	0.99	1	t	1	Carposcalis obscura	Claytonia virginica	39	0.05	0.12	0.14	0.11
1	1	0.97	1	cv	1	Andrena cressoni	Claytonia virginica	39	0.03	0.11	0.14	0.11
0	1	0.90	1	t	1	Andrena carlini	Dentaria laciniata	39	0.05	0.11	0.09	0.10
1	1	0.99	1	t	1	Andrena carlini	Claytonia virginica	39	0.05	0.11	0.14	0.11
1	1	0.94	1	t	1	nomada luteola	Claytonia virginica	39	0.02	0.12	0.14	0.11
0	1	0.69	1	cv	1	Helophilus fasciatus	Dentaria laciniata	39	0.02	0.12	0.09	0.10
1	1	0.94	1	t	1	Helophilus fasciatus	Claytonia virginica	39	0.02	0.12	0.14	0.11
0	1	0.86	1	t	1	Melanostoma sp.	Dentaria laciniata	39	0.04	0.11	0.09	0.10
1	1	0.98	1	t	1	Melanostoma sp.	Claytonia virginica	39	0.04	0.11	0.14	0.11
1	1	0.94	1	cv	1	Mesograpta marginata	Claytonia virginica	39	0.02	0.12	0.14	0.11
1	1	0.94	1	t	1	Andrena sp.	Claytonia virginica	39	0.02	0.12	0.14	0.11
1	1	0.94	1	t	1	Dialictus coeruleus	Claytonia virginica	39	0.02	0.12	0.14	0.11
0	0	0.09	0	t	1	Andrena erythronii	Helophilus fasciatus	39	0.02	0.10	0.02	0.12
0	1	0.95	0	cv	1	Andrena personata	Claytonia virginica	39	0.02	0.11	0.14	0.11
0	0	0.24	0	t	1	Lasioglossum forbesii	Andrena carlini	39	0.02	0.12	0.05	0.11
0	0	0.07	0	t	1	Andrena forbesii	Dialictus coeruleus	39	0.02	0.12	0.02	0.12
0	0	0.10	0	t	1	Dicentra cucullaria	nomada luteola	39	0.02	0.09	0.02	0.12
0	0	0.18	0	cv	1	Dicentra canadensis	Helophilus fasciatus	39	0.03	0.08	0.02	0.12
0	0	0.26	0	t	1	Syrphus sp.	Metasyrphus americanus	39	0.03	0.12	0.04	0.11
0	0	0.39	0	t	1	Isopyrum biternatum	Erythronium albidum	39	0.06	0.10	0.02	0.10
0	0	0.16	0	t	1	Melanostoma sp.	Mesograpta marginata	39	0.04	0.11	0.02	0.12
0	0	0.22	0	cv	1	Dialictus obscurus	Dicentra cucullaria	39	0.04	0.12	0.02	0.09
0	0	0.18	0	t	1	Dialictus obscurus	Xylota nemorum	39	0.04	0.12	0.02	0.11
0	1	0.74	0	t	1	Andrena erythronii	Dentaria laciniata	39	0.02	0.10	0.09	0.10

0	1	0.60	0	t	1	Dialictus obscurus	Isopyrum biternatum	39	0.04	0.12	0.06	0.10
0	0	0.23	0	cv	1	Andrena miserabilis	Andrena rugosa	39	0.05	0.11	0.02	0.12
0	0	0.26	0	t	1	Eristalis dimidiatus	Metasyrphus americanus	39	0.03	0.12	0.04	0.11
0	0	0.19	0	t	1	Augochlora pura	Melanostoma sp.	39	0.02	0.11	0.04	0.11
0	0	0.07	0	t	1	Andrena forbesii	Lasioglossum forbesii	39	0.02	0.12	0.02	0.12
0	0	0.25	0	cv	1	Carposcalis obscura	Augochlorella striata	39	0.05	0.12	0.02	0.11
0	0	0.25	0	t	1	Halictus confusus	Andrena carlini	39	0.02	0.11	0.05	0.11
0	0	0.08	0	t	1	Erylaeus macroupinensi	Andrena sp.	39	0.02	0.11	0.02	0.12
1	0	0.08	0	t	1	Andrena rugosa	Andrena erythronii	39	0.02	0.12	0.02	0.10
0	0	0.09	0	cv	1	Dialictus imitatus	Dialictus oblongus	39	0.02	0.11	0.02	0.11
0	0	0.26	0	t	1	Surphus torvus	Andrena cressoni	39	0.04	0.11	0.03	0.11
0	0	0.38	0	t	1	Dicentra canadensis	Ceratina metallica	39	0.03	0.08	0.04	0.12
0	0	0.27	0	t	1	Dialictus oblongus	Andrena carlini	39	0.02	0.11	0.05	0.11
0	0	0.22	0	cv	1	Andrena	Andrena sp.	39	0.05	0.11	0.02	0.12
0	0	0.36	0	t	1	Dialictus obscurus	Metasyrphus americanus	39	0.04	0.12	0.04	0.11
0	0	0.16	0	t	1	Ceratina metallica	Andrena sp.	39	0.04	0.12	0.02	0.12
0	0	0.16	0	t	1	Andrena mansonii	Dialictus coeruleus	39	0.04	0.11	0.02	0.12
0	0	0.08	0	cv	1	Erylaeus macroupinensi	Helophilus fasciatus	39	0.02	0.11	0.02	0.12
0	0	0.11	0	t	1	Andrena cressoni	nomada luteola	39	0.03	0.11	0.02	0.12
0	0	0.08	0	t	1	Erylaeus macroupinensi	Mesograpta marginata	39	0.02	0.11	0.02	0.12
1	0	0.08	0	t	1	Andrena forbesii	Andrena erythronii	39	0.02	0.12	0.02	0.10
0	0	0.17	0	cv	1	Andrena forbesii	Andrena mansonii	39	0.02	0.12	0.04	0.11
0	0	0.17	0	t	1	Ceratina metallica	Halictus confusus	39	0.04	0.12	0.02	0.11
0	1	0.90	0	t	1	Andrena irgeniae	Xylota nemorum	39	0.13	0.11	0.02	0.11
0	0	0.19	0	t	1	Andrena personata	Andrena mansonii	39	0.02	0.11	0.04	0.11
0	0	0.16	0	cv	1	Surphus torvus	Lasioglossum forbesii	39	0.04	0.11	0.02	0.12

0	0	0.11	0	t	1	Andrena rugosa	Andrena cressoni	39	0.02	0.12	0.03	0.11		
0	0	0.12	0	t	1	Syrphus sp.	Dialictus zephyrus	39	0.03	0.12	0.02	0.11		
0	0	0.45	0	t	1	Dialictus obscurus	Andrena miserabilis	39	0.04	0.12	0.05	0.11		
0	0	0.48	0	cv	1	Dicentra canadensis	Carposcalis obscura	39	0.03	0.08	0.05	0.12		
0	0	0.09	0	t	1	Andrena personata	Augochlorella striata	39	0.02	0.11	0.02	0.11		
0	0	0.45	0	t	1	Dialictus obscurus	Andrena carlini	39	0.04	0.12	0.05	0.11		
0	0	0.34	0	t	1	Isopyrum biternatum	Andrena sp.	39	0.06	0.10	0.02	0.12		
0	0	0.18	0	cv	1	Eristalis dimidiatus	Andrena cressoni	39	0.03	0.12	0.03	0.11		
0	0	0.08	0	t	1	Andrena personata	nomada luteola	39	0.02	0.11	0.02	0.12		
0	1	0.96	0	t	1	Andrena irgeniae	Ceratina metallica	39	0.13	0.11	0.04	0.12		
0	0	0.08	0	t	1	Erylaeus macroupinensi	Bombus greseocollis	39	0.02	0.11	0.02	0.11		
0	0	0.11	0	cv	1	Eristalis dimidiatus	Andrena sp.	39	0.03	0.12	0.02	0.12		
0	0	0.10	0	t	1	Andrena personata	Andrena erythronii	39	0.02	0.11	0.02	0.10		
0	0	0.18	0	t	1	Ceratina metallica	Dialictus zephyrus	39	0.04	0.12	0.02	0.11		
0	0	0.23	0	t	1	Andrena miserabilis	Helophilus fasciatus	39	0.05	0.11	0.02	0.12		
0	0	0.24	0	cv	1	Sanguinaria canadensis	Andrena personata	39	0.04	0.09	0.02	0.11		
0	0	0.29	0	t	1	Andrena erythronii	Andrena carlini	39	0.02	0.10	0.05	0.11		
0	0	0.25	0	t	1	Carposcalis obscura	Dialictus zephyrus	39	0.05	0.12	0.02	0.11		
0	1	0.73	0	t	1	Xylota nemorum	Dentaria laciniata	39	0.02	0.11	0.09	0.10		
0	0	0.07	0	cv	1	Bombus greseocollis	nomada luteola	39	0.02	0.11	0.02	0.12		
0	0	0.11	0	t	1	Syrphus sp.	Dialictus coeruleus	39	0.03	0.12	0.02	0.12		
0	1	0.67	1	t	1	Calopogon pulchellus	Thymelicus lineola	40	0.03	0.03	0.03	0.04		
1	1	0.65	1	t	1	Calopogon pulchellus	Toxomerus marginatus	40	0.03	0.03	0.02	0.03		
0	0	0.19	1	cv	1	Sercomyia transversa	Kalmia angustifolia	40	0.01	0.05	0.01	0.04		
0	0	0.37	1	t	1	Sercomyia transversa	Vaccinium myrtilloides	40	0.01	0.05	0.03	0.05		

0	0	0.33	1	t	1	Sercomyia transversa	Gaylussacia baccata	40	0.01	0.05	0.03	0.05
0	1	0.68	1	t	1	Sercomyia transversa	Ledum groenlandicum	40	0.01	0.05	0.06	0.05
0	1	0.64	1	cv	1	Sercomyia transversa	Kalmia polifolia	40	0.01	0.05	0.06	0.05
0	1	0.54	1	t	1	Dilophus caurinus	Nemopanthus mucronata	40	0.02	0.05	0.03	0.05
0	1	0.67	1	t	1	Helophilus laetus	Spiraea alba	40	0.03	0.05	0.04	0.05
0	1	0.58	1	t	1	Helophilus laetus	Aronia melanocarpa	40	0.03	0.05	0.03	0.05
1	1	0.82	1	cv	1	Helophilus laetus	Ledum groenlandicum	40	0.03	0.05	0.06	0.05
0	1	0.56	1	t	1	Andromeda glaucophylla	Pyrobombus impatiens	40	0.03	0.05	0.03	0.05
0	1	0.77	1	t	1	Andromeda glaucophylla	Pyrobombus ternarius	40	0.03	0.05	0.05	0.05
0	1	0.80	1	t	1	Pyrobombus perplexus	Spiraea alba	40	0.04	0.05	0.04	0.05
0	1	0.73	1	cv	1	Pyrobombus perplexus	Vaccinium myrtilloides	40	0.04	0.05	0.03	0.05
1	1	0.91	1	t	1	Pyrobombus perplexus	Salix fragilis	40	0.04	0.05	0.06	0.05
0	1	0.73	1	t	1	Pyrobombus perplexus	Aronia melanocarpa	40	0.04	0.05	0.03	0.05
1	1	0.89	1	t	1	Pyrobombus perplexus	Kalmia polifolia	40	0.04	0.05	0.06	0.05
0	0	0.47	1	cv	1	Kalmia angustifolia	Syritta pipiens	40	0.01	0.04	0.04	0.04
0	0	0.26	1	t	1	Kalmia angustifolia	Hybomitra typhus	40	0.01	0.04	0.02	0.05
0	0	0.41	1	t	1	Kalmia angustifolia	Pyrophaena rosarum	40	0.01	0.04	0.03	0.05
0	0	0.34	1	t	1	Kalmia angustifolia	Helophilus fasciatus	40	0.01	0.04	0.03	0.05
0	1	0.58	1	cv	1	Kalmia angustifolia	Pyrobombus ternarius	40	0.01	0.04	0.05	0.05
0	0	0.19	1	t	1	Kalmia angustifolia	Pyrobombus sandersoni	40	0.01	0.04	0.01	0.05
0	0	0.29	1	t	1	Kalmia angustifolia	Andrena mandibularis	40	0.01	0.04	0.02	0.05
1	1	0.94	1	t	1	Adela	Salix fragilis	40	0.06	0.06	0.06	0.05
0	1	0.65	1	cv	1	Spiraea alba	Pyrobombus impatiens	40	0.04	0.05	0.03	0.05
0	1	0.92	1	t	1	Spiraea alba	Hybomitra minuscula	40	0.04	0.05	0.06	0.05
0	1	0.78	1	t	1	Spiraea alba	Bombus terricola	40	0.04	0.05	0.04	0.05

0	0	0.48	1	t	1	Spiraea alba	Macrodactlyus subspinosus	40	0.04	0.05	0.01	0.05
0	1	0.76	1	cv	1	Spiraea alba	Syritta pipiens	40	0.04	0.05	0.04	0.04
0	1	0.69	1	t	1	Spiraea alba	Thymelicus lineola	40	0.04	0.05	0.03	0.04
0	1	0.71	1	t	1	Spiraea alba	Eristalis dimidiata	40	0.04	0.05	0.03	0.05
0	1	0.72	1	t	1	Spiraea alba	Pyrophaena rosarum	40	0.04	0.05	0.03	0.05
0	1	0.83	1	cv	1	Spiraea alba	Pyrobombus ternarius	40	0.04	0.05	0.05	0.05
0	0	0.46	1	t	1	Spiraea alba	Pyrobombus sandersoni	40	0.04	0.05	0.01	0.05
0	1	0.56	1	t	1	Pyrobombus impatiens	Vaccinium myrtilloides	40	0.03	0.05	0.03	0.05
0	1	0.51	1	t	1	Pyrobombus impatiens	Gaylussacia baccata	40	0.03	0.05	0.03	0.05
1	1	0.90	1	cv	1	Pyrobombus impatiens	Chamaedaphne calyculata	40	0.03	0.05	0.08	0.05
1	1	0.81	1	t	1	Pyrobombus impatiens	Ledum groenlandicum	40	0.03	0.05	0.06	0.05
1	1	0.79	1	t	1	Pyrobombus impatiens	Kalmia polifolia	40	0.03	0.05	0.06	0.05
0	1	0.74	1	t	1	Spilogona fatima	Salix fragilis	40	0.02	0.05	0.06	0.05
0	0	0.44	1	cv	1	Spilogona fatima	Nemopanthus mucronata	40	0.02	0.05	0.03	0.05
1	1	0.85	1	t	1	Spilogona fatima	Chamaedaphne calyculata	40	0.02	0.05	0.08	0.05
0	0	0.46	1	t	1	Spilogona fatima	Smilacina trifolia	40	0.02	0.05	0.03	0.04
0	0	0.37	1	t	1	Andrena vicina	Vaccinium myrtilloides	40	0.01	0.05	0.03	0.05
0	1	0.69	1	cv	1	Andrena vicina	Salix fragilis	40	0.01	0.05	0.06	0.05
0	0	0.32	1	t	1	Andrena vicina	Gaylussacia baccata	40	0.01	0.05	0.03	0.05
0	0	0.38	1	t	1	Andrena vicina	Nemopanthus mucronata	40	0.01	0.05	0.03	0.05
0	0	0.38	1	t	1	Andrena vicina	Aronia melanocarpa	40	0.01	0.05	0.03	0.05
1	1	0.81	1	cv	1	Andrena vicina	Chamaedaphne calyculata	40	0.01	0.05	0.08	0.05
1	1	0.67	1	t	1	Andrena vicina	Ledum groenlandicum	40	0.01	0.05	0.06	0.05
0	1	0.63	1	t	1	Andrena vicina	Kalmia polifolia	40	0.01	0.05	0.06	0.05

0	1	0.70	1	t	1	Vaccinium myrtilloides	Bombus terricola	40	0.03	0.05	0.04	0.05
0	1	0.73	1	cv	1	Vaccinium myrtilloides	Andrena regularis	40	0.03	0.05	0.04	0.05
0	0	0.43	1	t	1	Vaccinium myrtilloides	Andrena carolina	40	0.03	0.05	0.02	0.05
0	1	0.86	1	t	1	Vaccinium myrtilloides	Colletes inaequalis	40	0.03	0.05	0.06	0.05
0	1	0.90	1	t	1	Vaccinium myrtilloides	Apis mellifera	40	0.03	0.05	0.07	0.05
0	0	0.43	1	cv	1	Vaccinium myrtilloides	Andrena carlini	40	0.03	0.05	0.02	0.05
0	1	0.77	1	t	1	Vaccinium myrtilloides	Pyrobombus ternarius	40	0.03	0.05	0.05	0.05
0	0	0.36	1	t	1	Vaccinium myrtilloides	Pyrobombus sandersoni	40	0.03	0.05	0.01	0.05
0	0	0.49	1	t	1	Vaccinium myrtilloides	Andrena mandibularis	40	0.03	0.05	0.02	0.05
0	1	0.60	1	cv	1	Vaccinium myrtilloides	Andrena bradleyi	40	0.03	0.05	0.03	0.05
1	1	0.89	1	t	1	Bombus terricola	Salix fragilis	40	0.04	0.05	0.06	0.05
0	1	0.65	1	t	1	Bombus terricola	Gaylussacia baccata	40	0.04	0.05	0.03	0.05
1	1	0.89	1	t	1	Bombus terricola	Ledum groenlandicu m	40	0.04	0.05	0.06	0.05
1	1	0.68	1	cv	1	Andrena regularis	Gaylussacia baccata	40	0.04	0.05	0.03	0.05
1	1	0.73	1	t	1	Andrena regularis	Nemopanthu s mucronata	40	0.04	0.05	0.03	0.05
1	1	0.73	1	t	1	Andrena regularis	Aronia melanocarpa	40	0.04	0.05	0.03	0.05
1	1	0.95	1	t	1	Andrena regularis	Chamaedaph ne calyculata	40	0.04	0.05	0.08	0.05
1	1	0.90	1	cv	1	Andrena regularis	Ledum groenlandicu m	40	0.04	0.05	0.06	0.05
1	1	0.89	1	t	1	Andrena regularis	Kalmia polifolia	40	0.04	0.05	0.06	0.05
0	0	0.46	1	t	1	Syrphus torvus	Nemopanthu s mucronata	40	0.02	0.05	0.03	0.05
0	0	0.46	1	t	1	Syrphus torvus	Aronia melanocarpa	40	0.02	0.05	0.03	0.05
1	1	0.96	1	cv	1	Salix fragilis	Cyphon variabilis	40	0.06	0.05	0.06	0.06
0	1	0.72	1	t	1	Salix fragilis	Andrena carolina	40	0.06	0.05	0.02	0.05
1	1	0.95	1	t	1	Salix fragilis	Colletes inaequalis	40	0.06	0.05	0.06	0.05
1	1	0.97	1	t	1	Salix fragilis	Apis mellifera	40	0.06	0.05	0.07	0.05

0	1	0.72	1	cv	1	Salix fragilis	Andrena carlini	40	0.06	0.05	0.02	0.05
1	1	0.92	1	t	1	Salix fragilis	Pyrobombus ternarius	40	0.06	0.05	0.05	0.05
0	1	0.66	1	t	1	Salix fragilis	Pyrobombus sandersoni	40	0.06	0.05	0.01	0.05
0	1	0.84	1	t	1	Salix fragilis	Andrena bradleyi	40	0.06	0.05	0.03	0.05
1	1	0.69	1	cv	1	Syritta pipiens	Aronia melanocarpa	40	0.04	0.04	0.03	0.05
0	1	0.70	1	t	1	Syritta pipiens	Smilacina trifolia	40	0.04	0.04	0.03	0.04
0	0	0.38	1	t	1	Gaylussacia baccata	Andrena carolina	40	0.03	0.05	0.02	0.05
0	0	0.40	1	t	1	Gaylussacia baccata	Hybomitra typhus	40	0.03	0.05	0.02	0.05
0	1	0.57	1	cv	1	Gaylussacia baccata	Eristalis dimidiata	40	0.03	0.05	0.03	0.05
0	1	0.88	1	t	1	Gaylussacia baccata	Apis mellifera	40	0.03	0.05	0.07	0.05
0	1	0.51	1	t	1	Gaylussacia baccata	Helophilus fasciatus	40	0.03	0.05	0.03	0.05
0	1	0.73	1	t	1	Gaylussacia baccata	Pyrobombus ternarius	40	0.03	0.05	0.05	0.05
0	0	0.32	1	cv	1	Gaylussacia baccata	Pyrobombus sandersoni	40	0.03	0.05	0.01	0.05
0	1	0.84	1	t	1	Gaylussacia baccata	Helophilus latifrons	40	0.03	0.05	0.06	0.05
0	1	0.55	1	t	1	Gaylussacia baccata	Andrena bradleyi	40	0.03	0.05	0.03	0.05
0	1	0.73	1	t	1	Andrena carolina	Ledum groenlandicum	40	0.02	0.05	0.06	0.05
0	1	0.63	1	cv	1	Eristalis dimidiata	Aronia melanocarpa	40	0.03	0.05	0.03	0.05
1	1	0.85	1	t	1	Eristalis dimidiata	Ledum groenlandicum	40	0.03	0.05	0.06	0.05
1	1	0.85	1	t	1	Colletes inaequalis	Aronia melanocarpa	40	0.06	0.05	0.03	0.05
1	1	0.98	1	t	1	Colletes inaequalis	Chamaedaphne calyculata	40	0.06	0.05	0.08	0.05
1	1	0.95	1	cv	1	Colletes inaequalis	Ledum groenlandicum	40	0.06	0.05	0.06	0.05
0	1	0.63	1	t	1	Nemopanthus mucronata	Pyrophaena rosarum	40	0.03	0.05	0.03	0.05
0	1	0.90	1	t	1	Nemopanthus mucronata	Apis mellifera	40	0.03	0.05	0.07	0.05
0	0	0.43	1	t	1	Nemopanthus mucronata	Andrena carlini	40	0.03	0.05	0.02	0.05

0	1	0.62	1	cv	1	Nemopanthus mucronata	Andrena alleghaniensis	40	0.03	0.05	0.03	0.05
0	1	0.51	1	t	1	Nemopanthus mucronata	Melanostoma sp.	40	0.03	0.05	0.02	0.05
0	1	0.57	1	t	1	Nemopanthus mucronata	Dialictus pilosus	40	0.03	0.05	0.03	0.05
0	1	0.77	1	t	1	Nemopanthus mucronata	Pyrobombus ternarius	40	0.03	0.05	0.05	0.05
0	1	0.50	1	cv	1	Nemopanthus mucronata	Andrena mandibularis	40	0.03	0.05	0.02	0.05
1	1	0.85	1	t	1	Pyrophaena rosarum	Ledum groenlandicum	40	0.03	0.05	0.06	0.05
0	1	0.65	1	t	1	Pyrophaena rosarum	Smilacina trifolia	40	0.03	0.05	0.03	0.04
1	1	0.96	1	t	1	Apis mellifera	Kalmia polifolia	40	0.07	0.05	0.06	0.05
0	0	0.37	1	cv	1	Aronia melanocarpa	Shaerophoria sp.	40	0.03	0.05	0.01	0.05
0	1	0.63	1	t	1	Aronia melanocarpa	Andrena alleghaniensis	40	0.03	0.05	0.03	0.05
0	1	0.56	1	t	1	Aronia melanocarpa	Helophilus fasciatus	40	0.03	0.05	0.03	0.05
0	1	0.51	1	t	1	Aronia melanocarpa	Melanostoma sp.	40	0.03	0.05	0.02	0.05
0	1	0.57	1	cv	1	Aronia melanocarpa	Dialictus pilosus	40	0.03	0.05	0.03	0.05
0	1	0.77	1	t	1	Aronia melanocarpa	Pyrobombus ternarius	40	0.03	0.05	0.05	0.05
0	0	0.37	1	t	1	Aronia melanocarpa	Pyrobombus sandersoni	40	0.03	0.05	0.01	0.05
0	1	0.87	1	t	1	Aronia melanocarpa	Helophilus latifrons	40	0.03	0.05	0.06	0.05
1	1	0.85	1	cv	1	Andrena carlini	Chamaedaphne calyculata	40	0.02	0.05	0.08	0.05
1	1	0.73	1	t	1	Andrena carlini	Ledum groenlandicum	40	0.02	0.05	0.06	0.05
1	1	0.82	1	t	1	Shaerophoria sp.	Chamaedaphne calyculata	40	0.01	0.05	0.08	0.05
1	1	0.68	1	t	1	Shaerophoria sp.	Ledum groenlandicum	40	0.01	0.05	0.06	0.05
1	0	0.40	1	cv	1	Shaerophoria sp.	Smilacina trifolia	40	0.01	0.05	0.03	0.04
1	1	0.85	1	t	1	Andrena alleghaniensis	Ledum groenlandicum	40	0.03	0.05	0.06	0.05

1	1	0.90	1	t	1	Helophilus fasciatus	Chamaedaphne calyculata	40	0.03	0.05	0.08	0.05
1	1	0.81	1	t	1	Helophilus fasciatus	Ledum groenlandicum	40	0.03	0.05	0.06	0.05
1	1	0.88	1	cv	1	Melanostoma sp.	Chamaedaphne calyculata	40	0.02	0.05	0.08	0.05
1	1	0.53	1	t	1	Melanostoma sp.	Smilacina trifolia	40	0.02	0.05	0.03	0.04
0	1	0.79	1	t	1	Dialictus pilosus	Kalmia polifolia	40	0.03	0.05	0.06	0.05
1	1	0.96	1	t	1	Chamaedaphne calyculata	Pyrobombus ternarius	40	0.08	0.05	0.05	0.05
0	1	0.79	1	cv	1	Chamaedaphne calyculata	Pyrobombus sandersoni	40	0.08	0.05	0.01	0.05
0	1	0.87	1	t	1	Chamaedaphne calyculata	Andrena mandibularis	40	0.08	0.05	0.02	0.05
1	1	0.91	1	t	1	Chamaedaphne calyculata	Andrena bradleyi	40	0.08	0.05	0.03	0.05
0	1	0.91	1	t	1	Ledum groenlandicu	Pyrobombus ternarius	40	0.06	0.05	0.05	0.05
0	1	0.65	1	cv	1	Ledum groenlandicu	Pyrobombus sandersoni	40	0.06	0.05	0.01	0.05
0	1	0.76	1	t	1	Ledum groenlandicu	Andrena mandibularis	40	0.06	0.05	0.02	0.05
1	1	0.95	1	t	1	Ledum groenlandicu	Helophilus latifrons	40	0.06	0.05	0.06	0.05
1	1	0.90	1	t	1	Pyrobombus ternarius	Kalmia polifolia	40	0.05	0.05	0.06	0.05
0	1	0.63	1	cv	1	Pyrobombus sandersoni	Kalmia polifolia	40	0.01	0.05	0.06	0.05
1	1	0.74	1	t	1	Andrena mandibularis	Kalmia polifolia	40	0.02	0.05	0.06	0.05
0	1	0.81	1	t	1	Kalmia polifolia	Andrena bradleyi	40	0.06	0.05	0.03	0.05
0	0	0.38	0	t	1	Andrena vicina	Pyrophaena rosarum	40	0.01	0.05	0.03	0.05
0	1	0.55	0	cv	1	Eristalis dimidiata	Helophilus fasciatus	40	0.03	0.05	0.03	0.05
0	1	0.90	0	t	1	Calopogon pulchellus	Cyphon variabilis	40	0.03	0.03	0.06	0.06
1	1	0.84	0	t	1	Helophilus latifrons	Andrena bradleyi	40	0.06	0.05	0.03	0.05
0	1	0.82	0	t	1	Gaylussacia baccata	Ledum groenlandicum	40	0.03	0.05	0.06	0.05
0	1	0.60	0	cv	1	Andromeda glaucophylla	Thymelicus lineola	40	0.03	0.05	0.03	0.04
0	1	0.91	0	t	1	Spiraea alba	Helophilus latifrons	40	0.04	0.05	0.06	0.05

0	0	0.29	0	t	1	Sercomyia transversa	Dilophus caurinus	40	0.01	0.05	0.02	0.05
0	1	0.80	0	t	1	Adela purpurea	Eristalis dimidiata	40	0.06	0.06	0.03	0.05
0	1	0.57	0	cv	1	Pyrobombus perplexus	Hybomitra typhus	40	0.04	0.05	0.02	0.05
0	1	0.60	0	t	1	Eristalis dimidiata	Andrena bradleyi	40	0.03	0.05	0.03	0.05
1	1	0.96	0	t	1	Hybomitra minuscula	Salix fragilis	40	0.06	0.05	0.06	0.05
0	0	0.34	0	t	1	Dilophus caurinus	Andrena carlini	40	0.02	0.05	0.02	0.05
0	1	0.77	0	cv	1	Dilophus caurinus	Kalmia polifolia	40	0.02	0.05	0.06	0.05
0	1	0.90	0	t	1	Syritta pipiens	Cyphon variabilis	40	0.04	0.04	0.06	0.06
0	1	0.58	0	t	1	Bombus terricola	Andrena mandibularis	40	0.04	0.05	0.02	0.05
0	1	0.86	0	t	1	Salix fragilis	Smilacina trifolia	40	0.06	0.05	0.03	0.04
0	1	0.70	0	cv	1	Andromeda glaucophylla	Bombus terricola	40	0.03	0.05	0.04	0.05
0	1	0.55	0	t	1	Dilophus caurinus	Smilacina trifolia	40	0.02	0.05	0.03	0.04
0	1	0.81	0	t	1	Salix fragilis	Helophilus fasciatus	40	0.06	0.05	0.03	0.05
0	1	0.65	0	t	1	Adela purpurea	Andrena carolina	40	0.06	0.06	0.02	0.05
0	0	0.36	0	cv	1	Vaccinium myrtilloides	Shaerophoria sp.	40	0.03	0.05	0.01	0.05
0	1	0.53	0	t	1	Dilophus caurinus	Andromeda glaucophylla	40	0.02	0.05	0.03	0.05
0	1	0.66	0	t	1	Spiraea alba	Dialictus pilosus	40	0.04	0.05	0.03	0.05
0	0	0.47	0	t	1	Hybomitra typhus	Nemopanthus mucronata	40	0.02	0.05	0.03	0.05
0	1	0.87	0	cv	1	Nemopanthus mucronata	Helophilus latifrons	40	0.03	0.05	0.06	0.05
0	1	0.52	0	t	1	Syritta pipiens	Hybomitra typhus	40	0.04	0.04	0.02	0.05
0	0	0.37	0	t	1	Andromeda glaucophylla	Pyrobombus sandersoni	40	0.03	0.05	0.01	0.05
0	0	0.34	0	t	1	Dilophus caurinus	Andrena carolina	40	0.02	0.05	0.02	0.05
0	0	0.28	0	cv	1	Dilophus caurinus	Pyrobombus sandersoni	40	0.02	0.05	0.01	0.05
0	1	0.92	0	t	1	Andrena alleghaniensis	Chamaedaphne calyculata	40	0.03	0.05	0.08	0.05
0	1	0.73	0	t	1	Helophilus laetus	Pyrobombus ternarius	40	0.03	0.05	0.05	0.05

1	1	0.94	0	t	1	Adela purpurea	Cyphon variabilis	40	0.06	0.06	0.06	0.06
0	1	0.76	0	cv	1	Syrphus torvus	Colletes inaequalis	40	0.02	0.05	0.06	0.05
0	1	0.70	0	t	1	Hybomitra minuscula	Pyrobombus sandersoni	40	0.06	0.05	0.01	0.05
0	0	0.49	0	t	1	Syritta pipiens	Andrena carlini	40	0.04	0.04	0.02	0.05
0	1	0.89	0	t	1	Bombus terricola	Colletes inaequalis	40	0.04	0.05	0.06	0.05
1	1	0.68	0	cv	1	Andrena regularis	Dialictus pilosus	40	0.04	0.05	0.03	0.05
0	0	0.42	0	t	1	Andrena carlini	Andrena bradleyi	40	0.02	0.05	0.03	0.05
1	1	0.94	0	t	1	Adela purpurea	Helophilus latifrons	40	0.06	0.06	0.06	0.05
1	1	0.87	0	t	1	Apis mellifera	Dialictus pilosus	40	0.07	0.05	0.03	0.05
1	1	0.82	0	cv	1	Andrena alleghaniensis	Kalmia polifolia	40	0.03	0.05	0.06	0.05
0	0	0.49	0	t	1	Syritta pipiens	Andrena carolina	40	0.04	0.04	0.02	0.05
0	1	0.70	0	t	1	Bombus terricola	Aronia melanocarpa	40	0.04	0.05	0.03	0.05
0	0	0.47	0	t	1	Dilophus caurinus	Helophilus laetus	40	0.02	0.05	0.03	0.05
0	1	0.51	0	cv	1	Dilophus caurinus	Andrena bradleyi	40	0.02	0.05	0.03	0.05
0	1	0.52	0	t	1	Pyrobombus impatiens	Toxomerus marginatus	40	0.03	0.05	0.02	0.03
0	1	0.63	0	t	1	Calopogon pulchellus	Pyrobombus impatiens	40	0.03	0.03	0.03	0.05
0	0	0.40	0	t	1	Syrphus torvus	Gaylussacia baccata	40	0.02	0.05	0.03	0.05
0	1	0.64	0	cv	1	Eristalis dimidiata	Smilacina trifolia	40	0.03	0.05	0.03	0.04
0	1	0.72	0	t	1	Colletes inaequalis	Andrena carlini	40	0.06	0.05	0.02	0.05
1	1	0.97	0	t	1	Adela purpurea	Chamaedaphne calyculata	40	0.06	0.06	0.08	0.05
0	0	0.43	0	t	1	Eristalis dimidiata	Andrena carlini	40	0.03	0.05	0.02	0.05
0	0	0.30	0	cv	1	Pyrobombus impatiens	Andrena vicina	40	0.03	0.05	0.01	0.05
0	1	0.83	0	t	1	Dilophus caurinus	Hybomitra minuscula	40	0.02	0.05	0.06	0.05
0	1	0.84	0	t	1	Kalmia angustifolia	Chamaedaphne calyculata	40	0.01	0.04	0.08	0.05
0	1	0.59	0	t	1	Dialictus pilosus	Smilacina trifolia	40	0.03	0.05	0.03	0.04

0	1	0.69	0	cv	1	Calopogon pulchellus	Pyrophaena rosarum	40	0.03	0.03	0.03	0.05
0	0	0.28	0	t	1	Dilophus caurinus	Shaerophoria sp.	40	0.02	0.05	0.01	0.05
1	1	0.96	0	t	1	Hybomitra minuscula	Cyphon variabilis	40	0.06	0.05	0.06	0.06
0	1	0.81	0	t	1	Colletes inaequalis	Helophilus fasciatus	40	0.06	0.05	0.03	0.05
0	0	0.46	0	cv	1	Dilophus caurinus	Pyrobombus impatiens	40	0.02	0.05	0.03	0.05
0	1	0.72	0	t	1	Macrodactlyus subspinosus	Cyphon variabilis	40	0.01	0.05	0.06	0.06
0	1	0.58	0	t	1	Gaylussacia baccata	Aronia melanocarpa	40	0.03	0.05	0.03	0.05
0	1	0.54	0	t	1	Toxomerus marginatus	Dialictus pilosus	40	0.02	0.03	0.03	0.05
1	1	0.83	0	cv	1	Apis mellifera	Melanostoma sp.	40	0.07	0.05	0.02	0.05
0	0	0.21	0	t	1	Andrena vicina	Andrena carolina	40	0.01	0.05	0.02	0.05
0	1	0.91	0	t	1	Spiraea alba	Cyphon variabilis	40	0.04	0.05	0.06	0.06
0	1	0.72	0	t	1	Helophilus fasciatus	Pyrobombus ternarius	40	0.03	0.05	0.05	0.05
0	1	0.72	0	cv	1	Andromeda glaucophylla	Spiraea alba	40	0.03	0.05	0.04	0.05
0	1	0.50	0	t	1	Pyrophaena rosarum	Andrena mandibularis	40	0.03	0.05	0.02	0.05
0	0	0.40	0	t	1	Macrodactlyus subspinosus	Nemopanthus mucronata	40	0.01	0.05	0.03	0.05
0	0	0.38	0	t	1	Macrodactlyus subspinosus	Thymelicus lineola	40	0.01	0.05	0.03	0.04
1	1	0.76	0	cv	1	Hybomitra minuscula	Syrphus torvus	40	0.06	0.05	0.02	0.05
0	1	0.65	0	t	1	Andromeda glaucophylla	Smilacina trifolia	40	0.03	0.05	0.03	0.04
0	1	0.75	0	t	1	Pyrobombus perplexus	Smilacina trifolia	40	0.04	0.05	0.03	0.04
0	1	0.73	0	t	1	Pyrobombus perplexus	Eristalis dimidiata	40	0.04	0.05	0.03	0.05
0	0	0.43	0	cv	1	Pyrophaena rosarum	Andrena carlini	40	0.03	0.05	0.02	0.05
0	0	0.37	0	t	1	Pyrophaena rosarum	Pyrobombus sandersoni	40	0.03	0.05	0.01	0.05
0	1	0.50	0	t	1	Spilogona fatima	Syritta pipiens	40	0.02	0.05	0.04	0.04
0	1	0.84	0	t	1	Salix fragilis	Thymelicus lineola	40	0.06	0.05	0.03	0.04
0	0	0.21	0	cv	1	Andrena carolina	Pyrobombus sandersoni	40	0.02	0.05	0.01	0.05

0	1	0.85	0	t	1	Salix fragilis	Eristalis dimidiata	40	0.06	0.05	0.03	0.05
0	0	0.40	0	t	1	Macrodactlyus subspinosus	Aronia melanocarpa	40	0.01	0.05	0.03	0.05
1	1	0.82	0	t	1	Pyrobombus perplexus	Andrena regularis	40	0.04	0.05	0.04	0.05
0	1	0.92	0	cv	1	Toxomerus marginatus	Chamaedaphne calyculata	40	0.02	0.03	0.08	0.05
0	1	0.78	0	t	1	Pyrobombus ternarius	Smilacina trifolia	40	0.05	0.05	0.03	0.04
0	1	0.75	0	t	1	Hybomitra minuscula	Andrena carlini	40	0.06	0.05	0.02	0.05
0	1	0.71	0	t	1	Adela purpurea	Andrena mandibularis	40	0.06	0.06	0.02	0.05
0	0	0.38	0	cv	1	Pyrobombus impatiens	Syrphus torvus	40	0.03	0.05	0.02	0.05
0	1	0.78	0	t	1	Pyrobombus perplexus	Syritta pipiens	40	0.04	0.05	0.04	0.04
0	0	0.48	0	t	1	Pyrobombus perplexus	Andrena vicina	40	0.04	0.05	0.01	0.05
0	1	0.57	0	t	1	Calopogon pulchellus	Melanostoma sp.	40	0.03	0.03	0.02	0.05
1	1	0.91	0	cv	1	Andrena regularis	Cyphon variabilis	40	0.04	0.05	0.06	0.06
0	0	0.48	0	t	1	Andrena mandibularis	Andrena bradleyi	40	0.02	0.05	0.03	0.05
0	0	0.49	0	t	1	Eristalis dimidiata	Andrena mandibularis	40	0.03	0.05	0.02	0.05
0	1	0.70	0	t	1	Hybomitra minuscula	Shaerophoria sp.	40	0.06	0.05	0.01	0.05
0	1	0.77	0	cv	1	Andrena alleghaniensis	Pyrobombus ternarius	40	0.03	0.05	0.05	0.05
0	1	0.66	0	t	1	Syritta pipiens	Thymelicus lineola	40	0.04	0.04	0.03	0.04
0	0	0.40	0	t	1	Pyrobombus sandersoni	Smilacina trifolia	40	0.01	0.05	0.03	0.04
0	0	0.38	0	t	1	Vaccinium myrtilloides	Macrodactlyus subspinosus	40	0.03	0.05	0.01	0.05
0	1	0.85	0	cv	1	Helophilus laetus	Hybomitra minuscula	40	0.03	0.05	0.06	0.05
0	1	0.59	0	t	1	Thymelicus lineola	Andrena bradleyi	40	0.03	0.04	0.03	0.05
0	1	0.89	0	t	1	Calopogon pulchellus	Colletes inaequalis	40	0.03	0.03	0.06	0.05
0	0	0.44	0	t	1	Sercomyia transversa	Syritta pipiens	40	0.01	0.05	0.04	0.04
0	1	0.75	0	cv	1	Andrena carlini	Helophilus latifrons	40	0.02	0.05	0.06	0.05
0	0	0.43	0	t	1	Andromeda glaucophylla	Andrena carolina	40	0.03	0.05	0.02	0.05

0	0	0.27	0	t	1	Shaerophoria sp.	Melanostoma sp.	40	0.01	0.05	0.02	0.05
0	0	0.28	0	t	1	Syrphus torvus	Andrena carolina	40	0.02	0.05	0.02	0.05
0	1	0.77	0	cv	1	Salix fragilis	Andrena mandibularis	40	0.06	0.05	0.02	0.05
0	0	0.36	0	t	1	Eristalis dimidiata	Shaerophoria sp.	40	0.03	0.05	0.01	0.05
0	0	0.46	0	t	1	Hybomitra typhus	Andrena alleghaniensis	40	0.02	0.05	0.03	0.05
0	1	0.79	0	t	1	Kalmia angustifolia	Apis mellifera	40	0.01	0.04	0.07	0.05
1	1	0.83	0	cv	1	Pyrobombus impatiens	Salix fragilis	40	0.03	0.05	0.06	0.05
0	0	0.38	0	t	1	Sercomyia transversa	Aronia melanocarpa	40	0.01	0.05	0.03	0.05
1	1	0.94	0	t	1	Colletes inaequalis	Kalmia polifolia	40	0.06	0.05	0.06	0.05
0	0	0.30	0	t	1	Pyrobombus impatiens	Pyrobombus sandersoni	40	0.03	0.05	0.01	0.05
0	0	0.44	0	cv	1	Bombus terricola	Shaerophoria sp.	40	0.04	0.05	0.01	0.05
1	1	0.83	0	t	1	Eristalis dimidiata	Kalmia polifolia	40	0.03	0.05	0.06	0.05
0	0	0.23	0	t	1	Macrodactlyus subspinosus	Andrena carolina	40	0.01	0.05	0.02	0.05
0	1	0.92	0	t	1	Adela purpurea	Kalmia polifolia	40	0.06	0.06	0.06	0.05
0	1	0.80	0	cv	1	Adela purpurea	Pyrophaena rosarum	40	0.06	0.06	0.03	0.05
0	0	0.19	0	t	1	Sercomyia transversa	Macrodactlyus subspinosus	40	0.01	0.05	0.01	0.05
0	1	0.54	0	t	1	Helophilus fasciatus	Andrena bradleyi	40	0.03	0.05	0.03	0.05
0	1	0.57	0	t	1	Macrodactlyus subspinosus	Pyrobombus ternarius	40	0.01	0.05	0.05	0.05
0	1	0.93	0	cv	1	Spiraea alba	Apis mellifera	40	0.04	0.05	0.07	0.05
0	0	0.44	0	t	1	Andrena carolina	Eristalis dimidiata	40	0.02	0.05	0.03	0.05
0	1	0.67	0	t	1	Calopogon pulchellus	Andrena bradleyi	40	0.03	0.03	0.03	0.05
0	1	0.73	0	t	1	Kalmia angustifolia	Cyphon variabilis	40	0.01	0.04	0.06	0.06
0	0	0.37	0	cv	1	Pyrobombus impatiens	Spilogona fatima	40	0.03	0.05	0.02	0.05
0	1	0.91	0	t	1	Calopogon pulchellus	Hybomitra minuscula	40	0.03	0.03	0.06	0.05
0	0	0.32	0	t	1	Spilogona fatima	Andrena mandibularis	40	0.02	0.05	0.02	0.05

0	1	0.74	0	t	1	Spilogona fatima	Colletes inaequalis	40	0.02	0.05	0.06	0.05
0	1	0.83	0	cv	1	Gaylussacia baccata	Colletes inaequalis	40	0.03	0.05	0.06	0.05
0	1	0.50	0	t	1	Calopogon pulchellus	Spilogona fatima	40	0.03	0.03	0.02	0.05
0	1	0.55	0	t	1	Spiraea alba	Syrphus torvus	40	0.04	0.05	0.02	0.05
0	1	0.55	0	t	1	Sercomyia transversa	Pyrobombus ternarius	40	0.01	0.05	0.05	0.05
1	1	0.83	0	cv	1	Apis mellifera	Andrena mandibularis	40	0.07	0.05	0.02	0.05
0	0	0.23	0	t	1	Macrodactlyus subspinosus	Andrena carlini	40	0.01	0.05	0.02	0.05
0	0	0.21	0	t	1	Andrena vicina	Andrena carlini	40	0.01	0.05	0.02	0.05
0	1	0.55	0	t	1	Helophilus laetus	Andrena bradleyi	40	0.03	0.05	0.03	0.05
0	0	0.16	1	cv	1	24	95	41	0.01	0.08	0.02	0.06
1	1	0.51	1	t	1	25	98	41	0.01	0.05	0.04	0.05
0	0	0.17	1	t	1	26	Stichodactyla mertensii	41	0.01	0.07	0.02	0.06
1	1	0.54	1	t	1	27	Heteractis aurora	41	0.02	0.09	0.05	0.07
1	1	0.97	1	cv	1	27	92	41	0.02	0.09	0.13	0.08
1	0	0.32	1	t	1	27	97	41	0.02	0.09	0.03	0.07
1	1	0.80	1	t	1	27	96	41	0.02	0.09	0.08	0.07
1	0	0.38	1	t	1	20	99	41	0.02	0.09	0.03	0.05
1	1	0.52	1	cv	1	20	98	41	0.02	0.09	0.04	0.05
1	1	0.97	1	t	1	20	92	41	0.02	0.09	0.13	0.08
1	1	0.80	1	t	1	20	96	41	0.02	0.09	0.08	0.07
1	1	0.85	1	t	1	21	93	41	0.02	0.09	0.09	0.06
1	1	0.96	1	cv	1	21	92	41	0.02	0.09	0.13	0.08
1	1	0.77	1	t	1	21	96	41	0.02	0.09	0.08	0.07
1	1	0.93	1	t	1	22	92	41	0.01	0.10	0.13	0.08
1	0	0.29	1	t	1	23	91	41	0.01	0.08	0.03	0.06
1	1	0.85	1	cv	1	23	93	41	0.01	0.08	0.09	0.06
1	1	0.51	1	t	1	28	98	41	0.01	0.05	0.04	0.05
1	1	0.93	1	t	1	29	92	41	0.01	0.10	0.13	0.08
1	0	0.33	1	t	1	4	100	41	0.02	0.08	0.02	0.06
1	0	0.42	1	cv	1	4	99	41	0.02	0.08	0.03	0.05
1	1	0.90	1	t	1	4	93	41	0.02	0.08	0.09	0.06
1	1	0.60	1	t	1	4	94	41	0.02	0.08	0.05	0.06
1	0	0.49	1	t	1	8	Heteractis aurora	41	0.02	0.10	0.05	0.07
1	1	0.96	1	cv	1	8	92	41	0.02	0.10	0.13	0.08
1	0	0.48	1	t	1	8	94	41	0.02	0.10	0.05	0.06
1	1	0.95	1	t	1	59	92	41	0.01	0.10	0.13	0.08
1	1	0.71	1	t	1	59	96	41	0.01	0.10	0.08	0.07
1	1	0.69	1	cv	1	58	96	41	0.01	0.09	0.08	0.07
1	1	0.95	1	t	1	55	92	41	0.01	0.10	0.13	0.08

1	1	0.71	1	t	1	55	96	41	0.01	0.10	0.08	0.07
1	1	0.81	1	t	1	54	93	41	0.01	0.08	0.09	0.06
1	1	0.93	1	cv	1	57	92	41	0.01	0.10	0.13	0.08
1	0	0.48	1	t	1	56	Heteractis aurora	41	0.01	0.08	0.05	0.07
1	1	0.84	1	t	1	56	93	41	0.01	0.08	0.09	0.06
1	1	0.81	1	t	1	51	93	41	0.01	0.08	0.09	0.06
1	1	0.69	1	cv	1	50	96	41	0.01	0.09	0.08	0.07
1	0	0.25	1	t	1	53	91	41	0.01	0.08	0.03	0.06
1	1	0.81	1	t	1	52	93	41	0.01	0.08	0.09	0.06
1	1	0.81	1	t	1	88	93	41	0.01	0.08	0.09	0.06
1	1	0.93	1	cv	1	89	92	41	0.01	0.10	0.13	0.08
1	0	0.41	1	t	1	82	Heteractis aurora	41	0.01	0.09	0.05	0.07
1	1	0.93	1	t	1	83	92	41	0.01	0.10	0.13	0.08
1	1	0.93	1	t	1	80	92	41	0.01	0.10	0.13	0.08
1	1	0.81	1	cv	1	81	93	41	0.01	0.08	0.09	0.06
1	1	0.51	1	t	1	86	98	41	0.01	0.05	0.04	0.05
1	1	0.93	1	t	1	87	92	41	0.01	0.10	0.13	0.08
1	1	0.69	1	t	1	84	96	41	0.01	0.09	0.08	0.07
1	1	0.69	1	cv	1	85	96	41	0.01	0.09	0.08	0.07
1	0	0.43	1	t	1	3	91	41	0.03	0.09	0.03	0.06
1	1	0.91	1	t	1	3	93	41	0.03	0.09	0.09	0.06
1	1	0.98	1	t	1	3	92	41	0.03	0.09	0.13	0.08
1	1	0.63	1	cv	1	3	94	41	0.03	0.09	0.05	0.06
1	0	0.42	1	t	1	3	97	41	0.03	0.09	0.03	0.07
1	1	0.86	1	t	1	3	96	41	0.03	0.09	0.08	0.07
1	1	0.87	1	t	1	7	93	41	0.02	0.09	0.09	0.06
1	1	0.97	1	cv	1	7	92	41	0.02	0.09	0.13	0.08
1	1	0.55	1	t	1	7	94	41	0.02	0.09	0.05	0.06
1	1	0.80	1	t	1	7	96	41	0.02	0.09	0.08	0.07
1	0	0.48	1	t	1	Cryptodendrum adhaesivum	2	41	0.02	0.06	0.05	0.09
0	0	0.37	1	cv	1	Cryptodendrum adhaesivum	9	41	0.02	0.06	0.04	0.09
1	0	0.46	1	t	1	Premnas biaculeatus	2	41	0.02	0.07	0.05	0.09
0	0	0.23	1	t	1	Premnas biaculeatus	15	41	0.02	0.07	0.02	0.09
0	0	0.18	1	t	1	Premnas biaculeatus	42	41	0.02	0.07	0.02	0.10
1	1	0.55	1	cv	1	100	2	41	0.02	0.06	0.05	0.09
0	0	0.37	1	t	1	100	6	41	0.02	0.06	0.02	0.07
0	0	0.43	1	t	1	100	9	41	0.02	0.06	0.04	0.09
1	0	0.48	1	t	1	Amphiprion bicinctus	30	41	0.05	0.07	0.02	0.09
1	0	0.42	1	cv	1	Amphiprion bicinctus	36	41	0.05	0.07	0.01	0.10
1	1	0.78	1	t	1	Amphiprion bicinctus	2	41	0.05	0.07	0.05	0.09

1	0	0.42	1	t	1	Amphiprion bicinctus	11	41	0.05	0.07	0.01	0.10
1	0	0.48	1	t	1	Amphiprion bicinctus	13	41	0.05	0.07	0.02	0.09
1	0	0.48	1	cv	1	Amphiprion bicinctus	17	41	0.05	0.07	0.02	0.09
1	0	0.42	1	t	1	Amphiprion bicinctus	16	41	0.05	0.07	0.01	0.10
1	0	0.48	1	t	1	Amphiprion bicinctus	42	41	0.05	0.07	0.02	0.10
0	0	0.08	1	t	1	Stichodactyla haddoni	37	41	0.01	0.08	0.01	0.10
1	0	0.28	1	cv	1	39	91	41	0.02	0.10	0.03	0.06
1	1	0.96	1	t	1	39	92	41	0.02	0.10	0.13	0.08
1	0	0.27	1	t	1	39	97	41	0.02	0.10	0.03	0.07
1	1	0.95	1	t	1	38	92	41	0.01	0.10	0.13	0.08
1	0	0.23	1	cv	1	38	97	41	0.01	0.10	0.03	0.07
1	1	0.93	1	t	1	33	92	41	0.01	0.10	0.13	0.08
1	1	0.81	1	t	1	32	93	41	0.01	0.08	0.09	0.06
1	0	0.48	1	t	1	31	98	41	0.01	0.07	0.04	0.05
1	1	0.86	1	cv	1	31	93	41	0.01	0.07	0.09	0.06
1	1	0.96	1	t	1	30	92	41	0.02	0.09	0.13	0.08
1	1	0.76	1	t	1	30	96	41	0.02	0.09	0.08	0.07
1	1	0.94	1	t	1	37	92	41	0.01	0.10	0.13	0.08
1	1	0.95	1	cv	1	36	92	41	0.01	0.10	0.13	0.08
1	1	0.93	1	t	1	35	92	41	0.01	0.10	0.13	0.08
1	1	0.95	1	t	1	34	92	41	0.01	0.10	0.13	0.08
1	1	0.71	1	t	1	34	96	41	0.01	0.10	0.08	0.07
1	1	0.93	1	cv	1	60	92	41	0.01	0.10	0.13	0.08
1	1	0.81	1	t	1	61	93	41	0.01	0.08	0.09	0.06
1	1	0.93	1	t	1	62	92	41	0.01	0.10	0.13	0.08
1	1	0.93	1	t	1	63	92	41	0.01	0.10	0.13	0.08
1	1	0.93	1	cv	1	64	92	41	0.01	0.10	0.13	0.08
1	1	0.81	1	t	1	65	93	41	0.01	0.08	0.09	0.06
1	1	0.51	1	t	1	66	98	41	0.01	0.05	0.04	0.05
1	1	0.81	1	t	1	67	93	41	0.01	0.08	0.09	0.06
1	1	0.81	1	cv	1	68	93	41	0.01	0.08	0.09	0.06
1	1	0.93	1	t	1	69	92	41	0.01	0.10	0.13	0.08
1	1	0.95	1	t	1	2	93	41	0.05	0.09	0.09	0.06
1	1	0.99	1	t	1	2	92	41	0.05	0.09	0.13	0.08
1	0	0.44	1	cv	1	2	95	41	0.05	0.09	0.02	0.06
1	1	0.76	1	t	1	2	94	41	0.05	0.09	0.05	0.06
1	1	0.57	1	t	1	2	97	41	0.05	0.09	0.03	0.07
1	1	0.92	1	t	1	2	96	41	0.05	0.09	0.08	0.07
1	0	0.46	1	cv	1	6	99	41	0.02	0.07	0.03	0.05
1	1	0.61	1	t	1	6	98	41	0.02	0.07	0.04	0.05
1	1	0.63	1	t	1	6	94	41	0.02	0.07	0.05	0.06
1	0	0.39	1	t	1	99	19	41	0.03	0.05	0.01	0.06
1	0	0.40	1	cv	1	99	43	41	0.03	0.05	0.02	0.07
1	1	0.53	1	t	1	99	9	41	0.03	0.05	0.04	0.09
1	1	0.53	1	t	1	98	19	41	0.04	0.05	0.01	0.06

0	1	0.54	1	t	1	98	43	41	0.04	0.05	0.02	0.07
1	0	0.40	1	cv	1	91	14	41	0.03	0.06	0.03	0.09
1	0	0.29	1	t	1	91	1	41	0.03	0.06	0.01	0.08
1	1	0.93	1	t	1	90	92	41	0.01	0.10	0.13	0.08
1	1	0.86	1	t	1	93	15	41	0.09	0.06	0.02	0.09
1	1	0.88	1	cv	1	93	14	41	0.09	0.06	0.03	0.09
1	1	0.78	1	t	1	93	18	41	0.09	0.06	0.01	0.08
1	1	0.78	1	t	1	93	49	41	0.09	0.06	0.01	0.08
1	1	0.87	1	t	1	93	43	41	0.09	0.06	0.02	0.07
1	1	0.82	1	cv	1	93	1	41	0.09	0.06	0.01	0.08
1	1	0.92	1	t	1	93	9	41	0.09	0.06	0.04	0.09
1	1	0.78	1	t	1	93	75	41	0.09	0.06	0.01	0.08
1	1	0.78	1	t	1	93	72	41	0.09	0.06	0.01	0.08
1	1	0.93	1	cv	1	92	11	41	0.13	0.08	0.01	0.10
1	1	0.93	1	t	1	92	10	41	0.13	0.08	0.01	0.10
1	1	0.95	1	t	1	92	13	41	0.13	0.08	0.02	0.09
1	1	0.96	1	t	1	92	15	41	0.13	0.08	0.02	0.09
1	1	0.97	1	cv	1	92	14	41	0.13	0.08	0.03	0.09
1	1	0.95	1	t	1	92	17	41	0.13	0.08	0.02	0.09
1	1	0.93	1	t	1	92	16	41	0.13	0.08	0.01	0.10
1	1	0.91	1	t	1	92	47	41	0.13	0.08	0.01	0.10
1	1	0.91	1	cv	1	92	44	41	0.13	0.08	0.01	0.10
1	1	0.91	1	t	1	92	45	41	0.13	0.08	0.01	0.10
1	1	0.95	1	t	1	92	42	41	0.13	0.08	0.02	0.10
1	1	0.91	1	t	1	92	40	41	0.13	0.08	0.01	0.10
1	1	0.91	1	cv	1	92	41	41	0.13	0.08	0.01	0.10
1	1	0.95	1	t	1	92	5	41	0.13	0.08	0.02	0.10
1	1	0.98	1	t	1	92	9	41	0.13	0.08	0.04	0.09
1	1	0.91	1	t	1	92	77	41	0.13	0.08	0.01	0.10
1	1	0.91	1	cv	1	92	74	41	0.13	0.08	0.01	0.10
1	1	0.91	1	t	1	92	71	41	0.13	0.08	0.01	0.10
1	1	0.91	1	t	1	92	70	41	0.13	0.08	0.01	0.10
1	1	0.91	1	t	1	92	79	41	0.13	0.08	0.01	0.10
1	1	0.91	1	cv	1	92	78	41	0.13	0.08	0.01	0.10
0	0	0.19	1	t	1	95	5	41	0.02	0.06	0.02	0.10
0	0	0.41	1	t	1	94	12	41	0.05	0.06	0.01	0.08
1	1	0.54	1	t	1	94	15	41	0.05	0.06	0.02	0.09
1	1	0.59	1	cv	1	94	14	41	0.05	0.06	0.03	0.09
0	0	0.41	1	t	1	94	48	41	0.05	0.06	0.01	0.08
1	1	0.68	1	t	1	94	9	41	0.05	0.06	0.04	0.09
0	0	0.27	1	t	1	97	5	41	0.03	0.07	0.02	0.10
1	1	0.69	1	cv	1	96	10	41	0.08	0.07	0.01	0.10
1	1	0.74	1	t	1	96	13	41	0.08	0.07	0.02	0.09
1	1	0.82	1	t	1	96	14	41	0.08	0.07	0.03	0.09
1	1	0.74	1	t	1	96	17	41	0.08	0.07	0.02	0.09
1	1	0.66	1	cv	1	96	46	41	0.08	0.07	0.01	0.09
1	1	0.87	1	t	1	96	9	41	0.08	0.07	0.04	0.09
1	1	0.66	1	t	1	96	76	41	0.08	0.07	0.01	0.09
1	1	0.66	1	t	1	96	73	41	0.08	0.07	0.01	0.09
0	0	0.05	0	cv	1	44	77	41	0.01	0.10	0.01	0.10
1	1	0.68	0	t	1	96	49	41	0.08	0.07	0.01	0.08

1	1	0.79	0	t	1	93	16	41	0.09	0.06	0.01	0.10
0	0	0.09	0	t	1	25	62	41	0.01	0.05	0.01	0.10
0	0	0.06	0	cv	1	60	10	41	0.01	0.10	0.01	0.10
0	0	0.05	0	t	1	22	69	41	0.01	0.10	0.01	0.10
0	0	0.05	0	t	1	60	74	41	0.01	0.10	0.01	0.10
0	0	0.06	0	t	1	51	90	41	0.01	0.08	0.01	0.10
0	0	0.05	0	cv	1	74	79	41	0.01	0.10	0.01	0.10
0	0	0.09	0	t	1	39	46	41	0.02	0.10	0.01	0.09
1	1	0.93	0	t	1	92	49	41	0.13	0.08	0.01	0.08
0	0	0.06	0	t	1	80	34	41	0.01	0.10	0.01	0.10
0	0	0.25	0	cv	1	99	46	41	0.03	0.05	0.01	0.09
0	0	0.15	0	t	1	27	23	41	0.02	0.09	0.01	0.08
0	0	0.13	0	t	1	51	7	41	0.01	0.08	0.02	0.09
0	0	0.13	0	t	1	6	47	41	0.02	0.07	0.01	0.10
0	0	0.11	0	cv	1	31	12	41	0.01	0.07	0.01	0.08
1	0	0.17	0	t	1	19	1	41	0.01	0.06	0.01	0.08
0	0	0.08	0	t	1	90	13	41	0.01	0.10	0.02	0.09
0	1	0.85	0	t	1	6	96	41	0.02	0.07	0.08	0.07
0	0	0.17	0	cv	1	52	6	41	0.01	0.08	0.02	0.07
1	0	0.22	0	t	1	86	Stichodactyla mertensii	41	0.01	0.05	0.02	0.06
1	0	0.41	0	t	1	Amphiprion bicinctus	32	41	0.05	0.07	0.01	0.08
0	0	0.16	0	t	1	95	11	41	0.02	0.06	0.01	0.10
0	0	0.08	0	cv	1	54	72	41	0.01	0.08	0.01	0.08
0	0	0.13	0	t	1	59	15	41	0.01	0.10	0.02	0.09
0	0	0.10	0	t	1	8	67	41	0.02	0.10	0.01	0.08
0	0	0.06	0	t	1	88	44	41	0.01	0.08	0.01	0.10
0	0	0.09	0	cv	1	42	73	41	0.02	0.10	0.01	0.09
0	0	0.12	0	t	1	35	14	41	0.01	0.10	0.03	0.09
0	0	0.06	0	t	1	Stichodactyla haddoni	79	41	0.01	0.08	0.01	0.10
0	0	0.15	0	t	1	25	5	41	0.01	0.05	0.02	0.10
0	0	0.06	0	cv	1	35	11	41	0.01	0.10	0.01	0.10
1	0	0.16	0	t	1	25	31	41	0.01	0.05	0.01	0.07
1	0	0.10	0	t	1	Stichodactyla haddoni	13	41	0.01	0.08	0.02	0.09
0	1	0.95	0	t	1	Stichodactyla haddoni	92	41	0.01	0.08	0.13	0.08
1	0	0.16	0	cv	1	53	Entacmaea quadricolor	41	0.01	0.08	0.02	0.07
0	0	0.13	0	t	1	88	15	41	0.01	0.08	0.02	0.09
0	0	0.14	0	t	1	4	82	41	0.02	0.08	0.01	0.09
0	0	0.06	0	t	1	80	65	41	0.01	0.10	0.01	0.08
0	0	0.15	0	cv	1	Cryptodendrum adhaesivum	Heteractis magnifica	41	0.02	0.06	0.01	0.08
0	0	0.08	0	t	1	54	34	41	0.01	0.08	0.01	0.10
0	0	0.14	0	t	1	3	35	41	0.03	0.09	0.01	0.10
0	0	0.11	0	t	1	21	55	41	0.02	0.09	0.01	0.10
0	0	0.06	0	cv	1	52	45	41	0.01	0.08	0.01	0.10
0	0	0.09	0	t	1	39	76	41	0.02	0.10	0.01	0.09

0	0	0.06	0	t	1	82	71	41	0.01	0.09	0.01	0.10
1	0	0.18	0	t	1	87	97	41	0.01	0.10	0.03	0.07
0	0	0.18	0	cv	1	97	70	41	0.03	0.07	0.01	0.10
0	0	0.06	0	t	1	83	38	41	0.01	0.10	0.01	0.10
0	0	0.09	0	t	1	28	87	41	0.01	0.05	0.01	0.10
1	1	0.71	0	t	1	88	96	41	0.01	0.08	0.08	0.07
0	0	0.10	0	cv	1	8	81	41	0.02	0.10	0.01	0.08
0	0	0.09	0	t	1	27	57	41	0.02	0.09	0.01	0.10
0	0	0.10	0	t	1	69	43	41	0.01	0.10	0.02	0.07
0	0	0.07	0	t	1	22	56	41	0.01	0.10	0.01	0.08
0	0	0.12	0	cv	1	50	7	41	0.01	0.09	0.02	0.09
0	0	0.34	0	t	1	94	71	41	0.05	0.06	0.01	0.10
1	0	0.21	0	t	1	29	99	41	0.01	0.10	0.03	0.05
0	0	0.08	0	t	1	10	16	41	0.01	0.10	0.01	0.10
0	0	0.10	0	cv	1	67	1	41	0.01	0.08	0.01	0.08
1	1	0.96	0	t	1	92	19	41	0.13	0.08	0.01	0.06
0	0	0.19	0	t	1	91	40	41	0.03	0.06	0.01	0.10
1	0	0.27	0	t	1	61	99	41	0.01	0.08	0.03	0.05
0	0	0.10	0	cv	1	54	1	41	0.01	0.08	0.01	0.08
0	0	0.08	0	t	1	23	29	41	0.01	0.08	0.01	0.10
0	0	0.06	0	t	1	88	41	41	0.01	0.08	0.01	0.10
0	0	0.06	0	t	1	85	70	41	0.01	0.09	0.01	0.10
1	0	0.07	0	cv	1	12	73	41	0.01	0.08	0.01	0.09
0	0	0.05	0	t	1	89	78	41	0.01	0.10	0.01	0.10
1	0	0.29	0	t	1	20	100	41	0.02	0.09	0.02	0.06
1	0	0.21	0	t	1	87	99	41	0.01	0.10	0.03	0.05
0	0	0.06	0	cv	1	54	80	41	0.01	0.08	0.01	0.10
0	0	0.15	0	t	1	28	39	41	0.01	0.05	0.02	0.10
0	0	0.26	0	t	1	23	9	41	0.01	0.08	0.04	0.09
0	0	0.05	0	t	1	47	40	41	0.01	0.10	0.01	0.10
0	0	0.07	0	cv	1	85	11	41	0.01	0.09	0.01	0.10
0	0	0.10	0	t	1	8	18	41	0.02	0.10	0.01	0.08
0	0	0.09	0	t	1	50	1	41	0.01	0.09	0.01	0.08
1	0	0.41	0	t	1	58	Heteractis aurora	41	0.01	0.09	0.05	0.07
0	0	0.07	0	cv	1	55	46	41	0.01	0.10	0.01	0.09
0	0	0.05	0	t	1	29	74	41	0.01	0.10	0.01	0.10
1	0	0.12	0	t	1	89	Stichodactyla mertensii	41	0.01	0.10	0.02	0.06
0	0	0.12	0	t	1	27	65	41	0.02	0.09	0.01	0.08
0	0	0.28	0	cv	1	2	10	41	0.05	0.09	0.01	0.10
0	0	0.08	0	t	1	60	1	41	0.01	0.10	0.01	0.08
0	0	0.12	0	t	1	25	51	41	0.01	0.05	0.01	0.08
0	0	0.08	0	t	1	51	52	41	0.01	0.08	0.01	0.08
0	0	0.18	0	cv	1	25	27	41	0.01	0.05	0.02	0.09
0	0	0.19	0	t	1	95	17	41	0.02	0.06	0.02	0.09
0	0	0.06	0	t	1	84	90	41	0.01	0.09	0.01	0.10
0	0	0.07	0	t	1	52	84	41	0.01	0.08	0.01	0.09
0	0	0.07	0	cv	1	53	83	41	0.01	0.08	0.01	0.10
0	0	0.06	0	t	1	62	48	41	0.01	0.10	0.01	0.08
0	0	0.25	0	t	1	91	10	41	0.03	0.06	0.01	0.10

0	0	0.15	0	t	1	27	39	41	0.02	0.09	0.02	0.10
0	0	0.27	0	cv	1	Cryptodendrum adhaesivum	Entacmaea quadricolor	41	0.02	0.06	0.02	0.07
1	0	0.15	0	t	1	59	Entacmaea quadricolor	41	0.01	0.10	0.02	0.07
0	0	0.27	0	t	1	99	12	41	0.03	0.05	0.01	0.08
0	0	0.08	0	t	1	59	11	41	0.01	0.10	0.01	0.10
0	0	0.15	0	cv	1	Premnas biaculeatus	16	41	0.02	0.07	0.01	0.10
0	0	0.08	0	t	1	8	41	41	0.02	0.10	0.01	0.10
1	0	0.34	0	t	1	7	91	41	0.02	0.09	0.03	0.06
0	0	0.16	0	t	1	42	43	41	0.02	0.10	0.02	0.07
0	0	0.13	0	cv	1	18	43	41	0.01	0.08	0.02	0.07
1	0	0.20	0	t	1	55	100	41	0.01	0.10	0.02	0.06
0	0	0.08	0	t	1	88	49	41	0.01	0.08	0.01	0.08
0	0	0.06	0	t	1	83	75	41	0.01	0.10	0.01	0.08
0	0	0.10	0	cv	1	39	10	41	0.02	0.10	0.01	0.10
0	0	0.10	0	t	1	83	7	41	0.01	0.10	0.02	0.09
0	0	0.21	0	t	1	88	9	41	0.01	0.08	0.04	0.09
0	0	0.06	0	t	1	44	72	41	0.01	0.10	0.01	0.08
0	0	0.11	0	cv	1	59	66	41	0.01	0.10	0.01	0.05
0	1	0.73	0	t	1	93	44	41	0.09	0.06	0.01	0.10
0	0	0.07	0	t	1	58	37	41	0.01	0.09	0.01	0.10
0	0	0.08	0	t	1	40	1	41	0.01	0.10	0.01	0.08
0	0	0.12	0	cv	1	4	80	41	0.02	0.08	0.01	0.10
1	0	0.11	0	t	1	Stichodactyla haddoni	31	41	0.01	0.08	0.01	0.07
0	0	0.11	0	t	1	19	44	41	0.01	0.06	0.01	0.10
0	0	0.06	0	t	1	64	18	41	0.01	0.10	0.01	0.08
0	0	0.41	0	cv	1	Stichodactyla haddoni	94	41	0.01	0.08	0.05	0.06
0	1	0.90	0	t	1	97	96	41	0.03	0.07	0.08	0.07
0	0	0.16	0	t	1	27	86	41	0.02	0.09	0.01	0.05
0	0	0.10	0	t	1	23	67	41	0.01	0.08	0.01	0.08
0	0	0.10	0	cv	1	57	7	41	0.01	0.10	0.02	0.09
1	0	0.48	0	t	1	94	13	41	0.05	0.06	0.02	0.09
0	0	0.10	0	t	1	56	67	41	0.01	0.08	0.01	0.08
1	0	0.15	0	t	1	84	6	41	0.01	0.09	0.02	0.07
0	0	0.14	0	cv	1	28	56	41	0.01	0.05	0.01	0.08
0	0	0.06	0	t	1	50	45	41	0.01	0.09	0.01	0.10
0	0	0.08	0	t	1	88	34	41	0.01	0.08	0.01	0.10
0	0	0.14	0	t	1	95	46	41	0.02	0.06	0.01	0.09
0	0	0.07	0	cv	1	18	46	41	0.01	0.08	0.01	0.09
0	0	0.15	0	t	1	4	61	41	0.02	0.08	0.01	0.08
0	0	0.11	0	t	1	28	84	41	0.01	0.05	0.01	0.09
1	0	0.50	0	t	1	23	Heteractis aurora	41	0.01	0.08	0.05	0.07
0	0	0.08	0	cv	1	63	5	41	0.01	0.10	0.02	0.10
0	0	0.05	0	t	1	80	63	41	0.01	0.10	0.01	0.10
0	0	0.16	0	t	1	100	45	41	0.02	0.06	0.01	0.10
0	0	0.06	0	t	1	80	81	41	0.01	0.10	0.01	0.08

0	0	0.16	0	cv	1	100	71	41	0.02	0.06	0.01	0.10
0	0	0.05	0	t	1	29	64	41	0.01	0.10	0.01	0.10
1	0	0.26	0	t	1	6	19	41	0.02	0.07	0.01	0.06
0	0	0.12	0	t	1	28	18	41	0.01	0.05	0.01	0.08
0	0	0.06	0	cv	1	22	12	41	0.01	0.10	0.01	0.08
0	0	0.07	0	t	1	5	77	41	0.02	0.10	0.01	0.10
0	0	0.13	0	t	1	8	30	41	0.02	0.10	0.02	0.09
0	0	0.11	0	t	1	19	45	41	0.01	0.06	0.01	0.10
0	0	0.06	0	cv	1	38	63	41	0.01	0.10	0.01	0.10
0	0	0.05	0	t	1	22	64	41	0.01	0.10	0.01	0.10
0	0	0.10	0	t	1	38	42	41	0.01	0.10	0.02	0.10
0	0	0.08	0	t	1	39	35	41	0.02	0.10	0.01	0.10
0	0	0.19	0	cv	1	14	17	41	0.03	0.09	0.02	0.09
0	1	0.70	0	t	1	98	97	41	0.04	0.05	0.03	0.07
0	1	0.93	0	t	1	93	97	41	0.09	0.06	0.03	0.07
1	0	0.12	0	t	1	82	19	41	0.01	0.09	0.01	0.06
0	0	0.16	0	cv	1	9	74	41	0.04	0.09	0.01	0.10
1	0	0.09	0	t	1	82	1	41	0.01	0.09	0.01	0.08
0	0	0.08	0	t	1	55	88	41	0.01	0.10	0.01	0.08
0	0	0.05	0	t	1	62	90	41	0.01	0.10	0.01	0.10
0	0	0.05	0	cv	1	60	69	41	0.01	0.10	0.01	0.10
0	0	0.10	0	t	1	21	67	41	0.02	0.09	0.01	0.08
0	1	0.87	0	t	1	28	93	41	0.01	0.05	0.09	0.06
0	0	0.09	0	t	1	5	72	41	0.02	0.10	0.01	0.08
0	1	0.78	1	cv	1	Magnificent Riflebird	Cissus hypoglauca	42	0.04	0.06	0.05	0.07
0	1	0.59	1	t	1	Magnificent Riflebird	Schefflera	42	0.04	0.06	0.03	0.07
1	1	0.87	1	t	1	Magnificent Riflebird	F. odoardi	42	0.04	0.06	0.07	0.07
0	0	0.36	1	t	1	Magnificent Riflebird	Gastonia	42	0.04	0.06	0.01	0.07
0	1	0.64	1	cv	1	Magnificent Riflebird	Homalanthus	42	0.04	0.06	0.04	0.07
0	1	0.81	1	t	1	Magnificent Riflebird	Chisocheton	42	0.04	0.06	0.05	0.07
1	1	0.97	1	t	1	Ficus #371	Trumpet Manucode	42	0.05	0.07	0.09	0.06
1	1	0.88	1	t	1	Ficus #371	Blue Bird of Paradise	42	0.05	0.07	0.06	0.06
1	1	0.73	1	cv	1	Ficus #371	Superb Bird of Paradise	42	0.05	0.07	0.04	0.06
1	0	0.42	1	t	1	Ficus #371	Raggiana Bird of Paradise	42	0.05	0.07	0.01	0.06
1	1	0.85	1	t	1	Ficus #371	Blackbilled Sicklebill	42	0.05	0.07	0.06	0.06
1	1	0.95	1	t	1	Ficus #371	Crinkle-collared Manucode	42	0.05	0.07	0.08	0.06

1	1	0.77	1	cv	1	Ficus #371	Lawes' Six-wired Bird of Paradise	42	0.05	0.07	0.05	0.06
1	1	0.96	1	t	1	Elmerrillia	Trumpet Manucode	42	0.05	0.07	0.09	0.06
1	1	0.96	1	t	1	Elmerrillia	Magnificent Bird of Paradise	42	0.05	0.07	0.09	0.06
1	1	0.73	1	t	1	Elmerrillia	Superb Bird of Paradise	42	0.05	0.07	0.04	0.06
1	0	0.41	1	cv	1	Elmerrillia	Raggiana Bird of Paradise	42	0.05	0.07	0.01	0.06
1	1	0.85	1	t	1	Elmerrillia	Blackbilled Sicklebill	42	0.05	0.07	0.06	0.06
1	1	0.95	1	t	1	Elmerrillia	Crinkle-collared Manucode	42	0.05	0.07	0.08	0.06
1	1	0.77	1	t	1	Elmerrillia	Lawes' Six-wired Bird of Paradise	42	0.05	0.07	0.05	0.06
1	1	0.86	1	cv	1	Piper	Trumpet Manucode	42	0.01	0.08	0.09	0.06
1	1	0.86	1	t	1	Glochidion	Magnificent Bird of Paradise	42	0.01	0.08	0.09	0.06
0	1	0.83	1	t	1	Trumpet Manucode	Uvaria	42	0.09	0.06	0.01	0.08
1	1	0.97	1	t	1	Trumpet Manucode	Cissus hypoglauca	42	0.09	0.06	0.05	0.07
1	1	0.96	1	cv	1	Trumpet Manucode	Ficus #275	42	0.09	0.06	0.05	0.07
1	1	0.92	1	t	1	Trumpet Manucode	Myristica	42	0.09	0.06	0.03	0.08
0	1	0.83	1	t	1	Trumpet Manucode	Ficus drupacea	42	0.09	0.06	0.01	0.08
1	1	0.91	1	t	1	Trumpet Manucode	Syzygium	42	0.09	0.06	0.02	0.07
1	1	0.90	1	cv	1	Trumpet Manucode	Ficus #217	42	0.09	0.06	0.02	0.08
1	1	0.96	1	t	1	Trumpet Manucode	Endospermum	42	0.09	0.06	0.05	0.07
1	1	0.98	1	t	1	Trumpet Manucode	F. odoardi	42	0.09	0.06	0.07	0.07
0	1	0.84	1	t	1	Trumpet Manucode	Gastonia	42	0.09	0.06	0.01	0.07
1	1	0.99	1	cv	1	Trumpet Manucode	Ficus #246	42	0.09	0.06	0.08	0.07
1	1	0.93	1	t	1	Trumpet Manucode	Ficus gul	42	0.09	0.06	0.03	0.08
1	1	0.92	1	t	1	Trumpet Manucode	Ficus #202	42	0.09	0.06	0.03	0.08

1	1	0.94	1	t	1	Trumpet Manucode	Homalanthus	42	0.09	0.06	0.04	0.07
1	1	0.90	1	cv	1	Trumpet Manucode	S. sogerensis	42	0.09	0.06	0.02	0.08
1	1	0.96	1	t	1	Trumpet Manucode	Ficus #181	42	0.09	0.06	0.05	0.07
1	1	0.97	1	t	1	Trumpet Manucode	Chisocheton	42	0.09	0.06	0.05	0.07
1	1	0.92	1	t	1	Trumpet Manucode	Dysoxylum	42	0.09	0.06	0.03	0.08
0	0	0.39	1	cv	1	Aporusa	Superb Bird of Paradise	42	0.01	0.07	0.04	0.06
1	1	0.86	1	t	1	Canthium	Magnificent Bird of Paradise	42	0.01	0.08	0.09	0.06
1	1	0.97	1	t	1	Cissus hypoglauca	Magnificent Bird of Paradise	42	0.05	0.07	0.09	0.06
1	1	0.90	1	t	1	Cissus hypoglauca	Blue Bird of Paradise	42	0.05	0.07	0.06	0.06
1	1	0.77	1	cv	1	Cissus hypoglauca	Superb Bird of Paradise	42	0.05	0.07	0.04	0.06
1	0	0.47	1	t	1	Cissus hypoglauca	Raggiana Bird of Paradise	42	0.05	0.07	0.01	0.06
1	1	0.88	1	t	1	Cissus hypoglauca	Blackbilled Sicklebill	42	0.05	0.07	0.06	0.06
1	1	0.81	1	t	1	Cissus hypoglauca	Lawes' Six-wired Bird of Paradise	42	0.05	0.07	0.05	0.06
1	1	0.96	1	cv	1	Magnificent Bird of	Ficus #275	42	0.09	0.06	0.05	0.07
1	1	0.92	1	t	1	Magnificent Bird of	Myristica	42	0.09	0.06	0.03	0.08
0	1	0.87	1	t	1	Magnificent Bird of	C. aristata	42	0.09	0.06	0.02	0.08
1	1	0.93	1	t	1	Magnificent Bird of	Schefflera	42	0.09	0.06	0.03	0.07
0	1	0.87	1	cv	1	Magnificent Bird of	Zingiberaceae	42	0.09	0.06	0.02	0.08
1	1	0.96	1	t	1	Magnificent Bird of	Endospermum	42	0.09	0.06	0.05	0.07
1	1	0.98	1	t	1	Magnificent Bird of	F. odoardi	42	0.09	0.06	0.07	0.07
0	1	0.83	1	t	1	Magnificent Bird of	Gastonia	42	0.09	0.06	0.01	0.07
1	1	0.90	1	cv	1	Magnificent Bird of	Sloanea aberrans	42	0.09	0.06	0.02	0.08
1	1	0.99	1	t	1	Magnificent Bird of	Ficus #246	42	0.09	0.06	0.08	0.07
1	1	0.92	1	t	1	Magnificent Bird of	Ficus gul	42	0.09	0.06	0.03	0.08

1	1	0.91	1	t	1	Magnificent Bird of	Pandanus	42	0.09	0.06	0.02	0.07
1	1	0.92	1	cv	1	Magnificent Bird of	Ficus #202	42	0.09	0.06	0.03	0.08
1	1	0.94	1	t	1	Magnificent Bird of	Homalanthus	42	0.09	0.06	0.04	0.07
1	1	0.90	1	t	1	Magnificent Bird of	S. sogerensis	42	0.09	0.06	0.02	0.08
1	1	0.97	1	t	1	Magnificent Bird of	Chisocheton	42	0.09	0.06	0.05	0.07
1	1	0.92	1	cv	1	Magnificent Bird of	Dysoxylum	42	0.09	0.06	0.03	0.08
1	1	0.87	1	t	1	Ficus #275	Blue Bird of Paradise	42	0.05	0.07	0.06	0.06
1	1	0.73	1	t	1	Ficus #275	Superb Bird of Paradise	42	0.05	0.07	0.04	0.06
1	0	0.41	1	t	1	Ficus #275	Raggiana Bird of Paradise	42	0.05	0.07	0.01	0.06
1	1	0.95	1	cv	1	Ficus #275	Crinkle-collared Manucode	42	0.05	0.07	0.08	0.06
1	1	0.77	1	t	1	Ficus #275	Lawes' Six-wired Bird of Paradise	42	0.05	0.07	0.05	0.06
1	0	0.27	1	t	1	Myristica	Raggiana Bird of Paradise	42	0.03	0.08	0.01	0.06
1	1	0.63	1	t	1	Myristica	Lawes' Six-wired Bird of Paradise	42	0.03	0.08	0.05	0.06
1	1	0.85	1	cv	1	C. aristata	Crinkle-collared Manucode	42	0.02	0.08	0.08	0.06
1	1	0.79	1	t	1	Schefflera	Blue Bird of Paradise	42	0.03	0.07	0.06	0.06
1	1	0.64	1	t	1	Schefflera	Lawes' Six-wired Bird of Paradise	42	0.03	0.07	0.05	0.06
0	0	0.22	1	t	1	Syzygium	Raggiana Bird of Paradise	42	0.02	0.07	0.01	0.06
1	1	0.58	1	cv	1	Syzygium	Lawes' Six-wired Bird of Paradise	42	0.02	0.07	0.05	0.06
0	1	0.66	1	t	1	Blue Bird of Paradise	Zingiberacea e	42	0.06	0.06	0.02	0.08
0	1	0.59	1	t	1	Blue Bird of Paradise	Gastonia	42	0.06	0.06	0.01	0.07
0	1	0.78	1	t	1	Blue Bird of Paradise	Ficus gul	42	0.06	0.06	0.03	0.08
0	1	0.73	1	cv	1	Blue Bird of Paradise	Pandanus	42	0.06	0.06	0.02	0.07

0	1	0.82	1	t	1	Blue Bird of Paradise	Homalanthus	42	0.06	0.06	0.04	0.07
1	1	0.88	1	t	1	Blue Bird of Paradise	Ficus #181	42	0.06	0.06	0.05	0.07
1	1	0.91	1	t	1	Blue Bird of Paradise	Chisocheton	42	0.06	0.06	0.05	0.07
1	1	0.85	1	cv	1	Zingiberaceae	Crinkle-collared Manucode	42	0.02	0.08	0.08	0.06
0	0	0.36	1	t	1	Aglaia	Raggiana Bird of Paradise	42	0.04	0.06	0.01	0.06
0	1	0.73	1	t	1	Aglaia	Lawes' Six-wired Bird of Paradise	42	0.04	0.06	0.05	0.06
1	1	0.73	1	t	1	Endospermum	Superb Bird of Paradise	42	0.05	0.07	0.04	0.06
1	0	0.41	1	cv	1	Endospermum	Raggiana Bird of Paradise	42	0.05	0.07	0.01	0.06
1	1	0.77	1	t	1	Endospermum	Lawes' Six-wired Bird of Paradise	42	0.05	0.07	0.05	0.06
1	1	0.86	1	t	1	F. odoardi	Superb Bird of Paradise	42	0.07	0.07	0.04	0.06
1	1	0.63	1	t	1	F. odoardi	Raggiana Bird of Paradise	42	0.07	0.07	0.01	0.06
1	1	0.98	1	cv	1	F. odoardi	Crinkle-collared Manucode	42	0.07	0.07	0.08	0.06
1	1	0.89	1	t	1	F. odoardi	Lawes' Six-wired Bird of Paradise	42	0.07	0.07	0.05	0.06
0	1	0.58	1	t	1	Superb Bird of Paradise	Ficus gul	42	0.04	0.06	0.03	0.08
0	1	0.52	1	t	1	Superb Bird of Paradise	Pandanus	42	0.04	0.06	0.02	0.07
0	1	0.64	1	cv	1	Superb Bird of Paradise	Homalanthus	42	0.04	0.06	0.04	0.07
0	1	0.52	1	t	1	Superb Bird of Paradise	S. sogerensis	42	0.04	0.06	0.02	0.08
0	1	0.81	1	t	1	Superb Bird of Paradise	Chisocheton	42	0.04	0.06	0.05	0.07
0	0	0.14	1	t	1	Gastonia	Raggiana Bird of Paradise	42	0.01	0.07	0.01	0.06
1	0	0.43	1	cv	1	Gastonia	Lawes' Six-wired Bird of Paradise	42	0.01	0.07	0.05	0.06
1	1	0.99	1	t	1	Ficus #246	Crinkle-collared Manucode	42	0.08	0.07	0.08	0.06
0	0	0.29	1	t	1	Raggiana Bird of Paradise	Ficus gul	42	0.01	0.06	0.03	0.08

0	0	0.24	1	t	1	Raggiana Bird of Paradise	Pandanus	42	0.01	0.06	0.02	0.07
0	0	0.34	1	cv	1	Raggiana Bird of Paradise	Homalanthus	42	0.01	0.06	0.04	0.07
0	0	0.23	1	t	1	Raggiana Bird of Paradise	S. sogerensis	42	0.01	0.06	0.02	0.08
0	1	0.54	1	t	1	Raggiana Bird of Paradise	Chisocheton	42	0.01	0.06	0.05	0.07
0	0	0.28	1	t	1	Raggiana Bird of Paradise	Dysoxylum	42	0.01	0.06	0.03	0.08
1	1	0.90	1	cv	1	Ficus gul	Crinkle-collared Manucode	42	0.03	0.08	0.08	0.06
1	1	0.64	1	t	1	Ficus gul	Lawes' Six-wired Bird of Paradise	42	0.03	0.08	0.05	0.06
1	1	0.90	1	t	1	Ficus #202	Crinkle-collared Manucode	42	0.03	0.08	0.08	0.06
1	1	0.90	1	t	1	Blackbilled Sicklebill	Chisocheton	42	0.06	0.06	0.05	0.07
1	1	0.96	1	cv	1	Crinkle-collared	Chisocheton	42	0.08	0.06	0.05	0.07
0	1	0.69	1	t	1	Lawes' Six-wired Bird of Paradise	Homalanthus	42	0.05	0.06	0.04	0.07
0	1	0.57	1	t	1	Lawes' Six-wired Bird of Paradise	S. sogerensis	42	0.05	0.06	0.02	0.08
0	1	0.78	1	t	1	Lawes' Six-wired Bird of Paradise	Ficus #181	42	0.05	0.06	0.05	0.07
0	0	0.43	1	cv	1	Lawes' Six-wired Bird of Paradise	Sterculia	42	0.05	0.06	0.01	0.07
0	1	0.84	1	t	1	Lawes' Six-wired Bird of Paradise	Chisocheton	42	0.05	0.06	0.05	0.07
0	1	0.63	1	t	1	Lawes' Six-wired Bird of Paradise	Dysoxylum	42	0.05	0.06	0.03	0.08
0	0	0.23	0	t	1	Glochidion	Schefflera	42	0.01	0.08	0.03	0.07
0	0	0.23	0	cv	1	Uvaria	Myristica	42	0.01	0.08	0.03	0.08
0	0	0.23	0	t	1	Canthium	Ficus gul	42	0.01	0.08	0.03	0.08
0	0	0.37	0	t	1	Elmerrillia	Aporusa	42	0.05	0.07	0.01	0.07
1	1	0.75	0	t	1	Ficus #202	Blackbilled Sicklebill	42	0.03	0.08	0.06	0.06
0	0	0.41	0	cv	1	Myristica	Ficus gul	42	0.03	0.08	0.03	0.08
0	0	0.39	0	t	1	Aporusa	Endospermum	42	0.01	0.07	0.05	0.07

0	1	0.79	0	t	1	Blackbilled Sicklebill	Homalanthus	42	0.06	0.06	0.04	0.07
0	0	0.45	0	t	1	Aglaia	Pandanus	42	0.04	0.06	0.02	0.07
0	1	0.92	0	cv	1	Ficus #371	Ficus #246	42	0.05	0.07	0.08	0.07
1	1	0.90	0	t	1	Magnificent Bird of	Syzygium	42	0.09	0.06	0.02	0.07
0	0	0.28	0	t	1	C. aristata	Ficus #202	42	0.02	0.08	0.03	0.08
0	1	0.59	0	t	1	Piper	F. odoardi	42	0.01	0.08	0.07	0.07
0	1	0.57	0	cv	1	Ficus #371	Ficus #202	42	0.05	0.07	0.03	0.08
0	0	0.24	0	t	1	Aporusa	Dysoxylum	42	0.01	0.07	0.03	0.08
0	0	0.23	0	t	1	Piper	Schefflera	42	0.01	0.08	0.03	0.07
1	0	0.37	0	t	1	Piper	Superb Bird of Paradise	42	0.01	0.08	0.04	0.06
0	1	0.76	0	cv	1	F. odoardi	Ficus #202	42	0.07	0.07	0.03	0.08
0	1	0.62	0	t	1	Sloanea aberrans	Chisocheton	42	0.02	0.08	0.05	0.07
1	1	0.86	0	t	1	Piper	Magnificent Bird of Paradise	42	0.01	0.08	0.09	0.06
0	0	0.38	0	t	1	Magnificent Riflebird	Sterculia	42	0.04	0.06	0.01	0.07
0	0	0.34	0	cv	1	C. aristata	Homalanthus	42	0.02	0.08	0.04	0.07
0	1	0.62	0	t	1	Cissus hypoglauca	Myristica	42	0.05	0.07	0.03	0.08
1	1	0.88	0	t	1	Ficus #217	Crinkle-collared Manucode	42	0.02	0.08	0.08	0.06
0	1	0.58	0	t	1	Aglaia	Homalanthus	42	0.04	0.06	0.04	0.07
0	0	0.35	0	cv	1	Elmerrillia	Glochidion	42	0.05	0.07	0.01	0.08
0	1	0.92	0	t	1	Endospermum	Ficus #246	42	0.05	0.07	0.08	0.07
0	0	0.36	0	t	1	Magnificent Riflebird	Ficus drupacea	42	0.04	0.06	0.01	0.08
0	0	0.14	0	t	1	Zingiberaceae	Gastonia	42	0.02	0.08	0.01	0.07
0	1	0.60	0	cv	1	Blue Bird of Paradise	Sterculia	42	0.06	0.06	0.01	0.07
0	1	0.68	0	t	1	Aglaia	Ficus #181	42	0.04	0.06	0.05	0.07
0	0	0.37	0	t	1	Elmerrillia	Sterculia	42	0.05	0.07	0.01	0.07
0	0	0.37	0	t	1	Canthium	Ficus #275	42	0.01	0.08	0.05	0.07
0	1	0.51	0	cv	1	Magnificent Riflebird	Ficus #217	42	0.04	0.06	0.02	0.08
0	0	0.15	0	t	1	Raggiana Bird of Paradise	Sterculia	42	0.01	0.06	0.01	0.07
1	0	0.43	0	t	1	Uvaria	Lawes' Six-wired Bird of Paradise	42	0.01	0.08	0.05	0.06
0	1	0.87	0	t	1	Ficus #371	F. odoardi	42	0.05	0.07	0.07	0.07
0	0	0.19	0	cv	1	Ficus #217	Sterculia	42	0.02	0.08	0.01	0.07
1	1	0.67	0	t	1	Ficus #275	Aglaia	42	0.05	0.07	0.04	0.06
0	0	0.48	0	t	1	Cissus hypoglauca	C. aristata	42	0.05	0.07	0.02	0.08
0	0	0.35	0	t	1	Syzygium	Dysoxylum	42	0.02	0.07	0.03	0.08

0	1	0.88	0	cv	1	Ficus #246	Homalanthus	42	0.08	0.07	0.04	0.07
0	0	0.29	0	t	1	Sloanea aberrans	S. sogerensis	42	0.02	0.08	0.02	0.08
0	1	0.94	0	t	1	Cissus hypoglauca	Ficus #246	42	0.05	0.07	0.08	0.07
0	0	0.31	0	t	1	Glochidion	Aglaia	42	0.01	0.08	0.04	0.06
0	0	0.38	0	cv	1	Gastonia	Ficus #181	42	0.01	0.07	0.05	0.07
0	0	0.11	0	t	1	Glochidion	Ficus drupacea	42	0.01	0.08	0.01	0.08
1	1	0.71	0	t	1	Cissus hypoglauca	Aglaia	42	0.05	0.07	0.04	0.06
0	1	0.84	0	t	1	Trumpet Manucode	Sterculia	42	0.09	0.06	0.01	0.07
1	1	0.89	0	cv	1	Crinkle-collared	Dysoxylum	42	0.08	0.06	0.03	0.08
0	1	0.57	0	t	1	Endospermum	Dysoxylum	42	0.05	0.07	0.03	0.08
0	0	0.28	0	t	1	Raggiana Bird of Paradise	Ficus #202	42	0.01	0.06	0.03	0.08
0	1	0.67	0	t	1	Myristica	Chisocheton	42	0.03	0.08	0.05	0.07
0	0	0.22	0	cv	1	Glochidion	Ficus #202	42	0.01	0.08	0.03	0.08
1	0	0.27	0	t	1	Schefflera	Raggiana Bird of Paradise	42	0.03	0.07	0.01	0.06
0	1	0.61	0	t	1	Aporusa	F. odoardi	42	0.01	0.07	0.07	0.07
0	0	0.28	0	t	1	C. aristata	Dysoxylum	42	0.02	0.08	0.03	0.08
0	1	0.77	0	cv	1	Elmerrillia	Cissus hypoglauca	42	0.05	0.07	0.05	0.07
1	1	0.63	0	t	1	C. aristata	Blackbilled Sicklebill	42	0.02	0.08	0.06	0.06
0	0	0.11	0	t	1	Glochidion	Gastonia	42	0.01	0.08	0.01	0.07
0	1	0.67	0	t	1	Ficus #202	Chisocheton	42	0.03	0.08	0.05	0.07
0	1	0.67	0	cv	1	Ficus gul	Chisocheton	42	0.03	0.08	0.05	0.07
0	0	0.41	0	t	1	Syzygium	Homalanthus	42	0.02	0.07	0.04	0.07
0	0	0.36	0	t	1	Schefflera	Syzygium	42	0.03	0.07	0.02	0.07
0	0	0.14	0	t	1	C. aristata	Ficus drupacea	42	0.02	0.08	0.01	0.08
0	1	0.57	0	cv	1	Elmerrillia	Ficus #202	42	0.05	0.07	0.03	0.08
0	0	0.37	0	t	1	Piper	Ficus #275	42	0.01	0.08	0.05	0.07
0	1	0.87	0	t	1	Blue Bird of Paradise	Superb Bird of Paradise	42	0.06	0.06	0.04	0.06
0	0	0.15	0	t	1	Zingiberaceae	Sterculia	42	0.02	0.08	0.01	0.07
0	1	0.60	0	cv	1	Schefflera	Ficus #181	42	0.03	0.07	0.05	0.07
0	1	0.90	0	t	1	Aglaia	Ficus #246	42	0.04	0.06	0.08	0.07
0	1	0.62	0	t	1	Pandanus	Chisocheton	42	0.02	0.07	0.05	0.07
0	0	0.38	0	t	1	C. aristata	Aglaia	42	0.02	0.08	0.04	0.06
0	1	0.57	0	cv	1	Elmerrillia	Ficus gul	42	0.05	0.07	0.03	0.08
0	1	0.85	0	t	1	Ficus #246	Ficus gul	42	0.08	0.07	0.03	0.08
1	0	0.37	0	t	1	Uvaria	Superb Bird of Paradise	42	0.01	0.08	0.04	0.06
1	0	0.45	0	t	1	Zingiberaceae	Superb Bird of Paradise	42	0.02	0.08	0.04	0.06
0	0	0.47	0	cv	1	Myristica	Homalanthus	42	0.03	0.08	0.04	0.07

1	1	0.61	0	t	1	Glochidion	Blue Bird of Paradise	42	0.01	0.08	0.06	0.06
0	0	0.11	0	t	1	Glochidion	Sterculia	42	0.01	0.08	0.01	0.07
0	0	0.47	0	t	1	Ficus gul	Homalanthus	42	0.03	0.08	0.04	0.07
0	0	0.29	0	cv	1	Zingiberaceae	Ficus #202	42	0.02	0.08	0.03	0.08
0	0	0.18	0	t	1	Uvaria	S. sogerensis	42	0.01	0.08	0.02	0.08
0	0	0.42	0	t	1	Canthium	Cissus hypoglauca	42	0.01	0.08	0.05	0.07
0	1	0.74	0	t	1	Ficus #371	Ficus #181	42	0.05	0.07	0.05	0.07
0	0	0.28	0	cv	1	Piper	Homalanthus	42	0.01	0.08	0.04	0.07
0	0	0.23	0	t	1	Canthium	Dysoxylum	42	0.01	0.08	0.03	0.08
0	0	0.37	0	t	1	Canthium	Endospermu m	42	0.01	0.08	0.05	0.07
0	0	0.45	0	t	1	Ficus #217	Aglaia	42	0.02	0.08	0.04	0.06
0	1	0.58	0	cv	1	Myristica	Endospermu m	42	0.03	0.08	0.05	0.07
0	0	0.32	0	t	1	Aglaia	Sterculia	42	0.04	0.06	0.01	0.07
0	1	0.57	0	t	1	Endospermum	Ficus #202	42	0.05	0.07	0.03	0.08
0	1	0.73	0	t	1	Magnificent Riflebird	Elmerrillia	42	0.04	0.06	0.05	0.07
0	1	0.58	0	cv	1	Elmerrillia	Schefflera	42	0.05	0.07	0.03	0.07
0	0	0.11	0	t	1	Aporusa	Canthium	42	0.01	0.07	0.01	0.08
0	0	0.35	0	t	1	Pandanus	Dysoxylum	42	0.02	0.07	0.03	0.08
0	0	0.23	0	t	1	Ficus drupacea	Dysoxylum	42	0.01	0.08	0.03	0.08
0	0	0.42	0	cv	1	Pandanus	Homalanthus	42	0.02	0.07	0.04	0.07
0	1	0.98	0	t	1	Blackbilled Sicklebill	Crinkle-collared Manucode	42	0.06	0.06	0.08	0.06
1	1	0.93	0	t	1	Trumpet Manucode	Schefflera	42	0.09	0.06	0.03	0.07
0	0	0.19	0	t	1	Uvaria	Syzygium	42	0.01	0.08	0.02	0.07
0	0	0.45	0	cv	1	Syzygium	Aglaia	42	0.02	0.07	0.04	0.06
0	0	0.36	0	t	1	Magnificent Riflebird	Canthium	42	0.04	0.06	0.01	0.08
0	0	0.23	0	t	1	Glochidion	Ficus gul	42	0.01	0.08	0.03	0.08
1	1	0.58	0	t	1	Ficus #217	Lawes' Six-wired Bird of Paradise	42	0.02	0.08	0.05	0.06
0	1	0.68	0	cv	1	Aglaia	Endospermu m	42	0.04	0.06	0.05	0.07
0	0	0.11	0	t	1	Canthium	Gastonia	42	0.01	0.08	0.01	0.07
0	1	0.58	0	t	1	Magnificent Riflebird	Myristica	42	0.04	0.06	0.03	0.08
0	0	0.29	0	t	1	Ficus #217	Sloanea aberrans	42	0.02	0.08	0.02	0.08
0	1	0.50	0	cv	1	Raggiana Bird of Paradise	Lawes' Six-wired Bird of Paradise	42	0.01	0.06	0.05	0.06
0	0	0.45	0	t	1	Aglaia	Sloanea aberrans	42	0.04	0.06	0.02	0.08
0	0	0.11	0	t	1	Uvaria	Canthium	42	0.01	0.08	0.01	0.08

0	0	0.18	0	t	1	Glochidion	S. sogerensis	42	0.01	0.08	0.02	0.08
0	1	0.63	0	cv	1	Endospermum	Homalanthus	42	0.05	0.07	0.04	0.07
0	0	0.47	0	t	1	Piper	Chisocheton	42	0.01	0.08	0.05	0.07
0	0	0.29	0	t	1	Ficus #217	S. sogerensis	42	0.02	0.08	0.02	0.08
0	0	0.45	0	t	1	Aporusa	Lawes' Six-wired Bird of Paradise	42	0.01	0.07	0.05	0.06
0	1	0.73	0	cv	1	Ficus #371	Elmerrillia	42	0.05	0.07	0.05	0.07
0	1	0.87	0	t	1	Crinkle-collared	S. sogerensis	42	0.08	0.06	0.02	0.08
0	1	0.74	1	t	1	Schiffornis turdinus	Psychotria marginata	43	0.03	0.06	0.06	0.07
0	1	0.69	1	t	1	Schiffornis turdinus	Miconia impetiolaris	43	0.03	0.06	0.05	0.07
0	1	0.74	1	cv	1	Schiffornis turdinus	Psychotria hoffmannseggiana	43	0.03	0.06	0.06	0.07
0	1	0.82	1	t	1	Ramphocelus dimidiatus	Miconia argentea	43	0.01	0.07	0.08	0.07
1	1	0.95	1	t	1	Psychotria acuminata	Pipra coronata	43	0.04	0.07	0.09	0.07
1	1	0.94	1	t	1	Psychotria acuminata	C. ustulatus	43	0.04	0.07	0.08	0.07
1	1	0.80	1	cv	1	Psychotria acuminata	C. minimus	43	0.04	0.07	0.05	0.06
1	1	0.80	1	t	1	Psychotria acuminata	C. fuscescens	43	0.04	0.07	0.05	0.06
1	1	0.96	1	t	1	Psychotria acuminata	P. mentalis	43	0.04	0.07	0.09	0.07
1	1	0.89	1	t	1	Psychotria pittieri	Pipra coronata	43	0.02	0.07	0.09	0.07
1	1	0.79	1	cv	1	Psychotria pittieri	Manacus vitellinus	43	0.02	0.07	0.07	0.07
0	1	0.60	1	t	1	Eucometis penicillata	Miconia impetiolaris	43	0.02	0.07	0.05	0.07
0	1	0.87	1	t	1	Eucometis penicillata	Miconia argentea	43	0.02	0.07	0.08	0.07
0	1	0.91	1	t	1	Habia fuscicauda	Miconia argentea	43	0.03	0.07	0.08	0.07
0	1	0.54	1	cv	1	Habia fuscicauda	Psychotria deflexa	43	0.03	0.07	0.04	0.07
0	1	0.61	1	t	1	Habia fuscicauda	Psychotria poeppigiana	43	0.03	0.07	0.04	0.07
0	1	0.82	1	t	1	D. striata	Miconia argentea	43	0.01	0.07	0.08	0.07
1	1	0.92	1	t	1	Psychotria racemosa	Pipra coronata	43	0.03	0.07	0.09	0.07
1	1	0.84	1	cv	1	Psychotria racemosa	Manacus vitellinus	43	0.03	0.07	0.07	0.07
1	1	0.93	1	t	1	Psychotria racemosa	P. mentalis	43	0.03	0.07	0.09	0.07

1	1	0.98	1	t	1	Pipra coronata	Miconia lacera	43	0.09	0.07	0.07	0.07
0	1	0.91	1	t	1	Pipra coronata	Psychotria brachybotrya	43	0.09	0.07	0.03	0.07
0	1	0.87	1	cv	1	Pipra coronata	Psychotria psychotrifolia	43	0.09	0.07	0.02	0.08
1	1	0.97	1	t	1	Pipra coronata	Psychotria marginata	43	0.09	0.07	0.06	0.07
0	1	0.96	1	t	1	Pipra coronata	Miconia impetiolaris	43	0.09	0.07	0.05	0.07
1	1	0.95	1	t	1	Pipra coronata	Miconia minutiflora	43	0.09	0.07	0.04	0.07
1	1	0.99	1	cv	1	Pipra coronata	Miconia argentea	43	0.09	0.07	0.08	0.07
1	1	0.98	1	t	1	Pipra coronata	Psychotria horizontalis	43	0.09	0.07	0.06	0.07
0	1	0.93	1	t	1	Pipra coronata	Psychotria deflexa	43	0.09	0.07	0.04	0.07
1	1	0.97	1	t	1	Pipra coronata	Psychotria hoffmannseggiana	43	0.09	0.07	0.06	0.07
0	1	0.95	1	cv	1	Pipra coronata	Psychotria poeppigiana	43	0.09	0.07	0.04	0.07
1	1	0.97	1	t	1	C. ustulatus	Miconia lacera	43	0.08	0.07	0.07	0.07
0	1	0.88	1	t	1	C. ustulatus	Psychotria brachybotrya	43	0.08	0.07	0.03	0.07
0	1	0.91	1	t	1	C. ustulatus	Psychotria limonensis	43	0.08	0.07	0.04	0.07
1	1	0.96	1	cv	1	C. ustulatus	Psychotria marginata	43	0.08	0.07	0.06	0.07
0	1	0.93	1	t	1	C. ustulatus	Miconia minutiflora	43	0.08	0.07	0.04	0.07
1	1	0.99	1	t	1	C. ustulatus	Miconia argentea	43	0.08	0.07	0.08	0.07
1	1	0.97	1	t	1	C. ustulatus	Psychotria horizontalis	43	0.08	0.07	0.06	0.07
0	1	0.91	1	cv	1	C. ustulatus	Psychotria deflexa	43	0.08	0.07	0.04	0.07
1	1	0.96	1	t	1	C. ustulatus	Psychotria hoffmannseggiana	43	0.08	0.07	0.06	0.07
0	1	0.84	1	t	1	C. ustulatus	Psychotria emetica	43	0.08	0.07	0.02	0.07
0	1	0.93	1	t	1	C. ustulatus	Psychotria poeppigiana	43	0.08	0.07	0.04	0.07
0	1	0.65	1	cv	1	Miconia lacera	Seiurus aurocapillus	43	0.07	0.07	0.01	0.06
0	1	0.65	1	t	1	Miconia lacera	Euphonia fulvicrissa	43	0.07	0.07	0.01	0.06

1	1	0.91	1	t	1	Miconia lacera	C. minimus	43	0.07	0.07	0.05	0.06
1	1	0.96	1	t	1	Miconia lacera	Manacus vitellinus	43	0.07	0.07	0.07	0.07
1	1	0.74	1	cv	1	Miconia lacera	Empidonax virescens	43	0.07	0.07	0.02	0.06
1	1	0.91	1	t	1	Miconia lacera	C. fuscescens	43	0.07	0.07	0.05	0.06
1	1	0.98	1	t	1	Miconia lacera	P. mentalis	43	0.07	0.07	0.09	0.07
0	1	0.82	1	t	1	Leptotila cassinii	Miconia argentea	43	0.01	0.07	0.08	0.07
1	1	0.93	1	cv	1	Psychotria brachybotrya	P. mentalis	43	0.03	0.07	0.09	0.07
1	1	0.90	1	t	1	Psychotria psychotrifolia	P. mentalis	43	0.02	0.08	0.09	0.07
1	1	0.88	1	t	1	Psychotria limonensis	Manacus vitellinus	43	0.04	0.07	0.07	0.07
1	1	0.75	1	t	1	Psychotria limonensis	C. fuscescens	43	0.04	0.07	0.05	0.06
1	1	0.95	1	cv	1	Psychotria limonensis	P. mentalis	43	0.04	0.07	0.09	0.07
0	1	0.82	1	t	1	Dendroica pensylvanica	Miconia argentea	43	0.01	0.07	0.08	0.07
0	1	0.80	1	t	1	C. minimus	Miconia minutiflora	43	0.05	0.06	0.04	0.07
0	1	0.89	1	t	1	C. minimus	Psychotria horizontalis	43	0.05	0.06	0.06	0.07
0	1	0.87	1	cv	1	C. minimus	Psychotria hoffmannseggiana	43	0.05	0.06	0.06	0.07
0	1	0.59	1	t	1	C. minimus	Psychotria emetica	43	0.05	0.06	0.02	0.07
1	1	0.54	1	t	1	Psychotria marginata	Trogon rufus	43	0.06	0.07	0.01	0.06
1	1	0.94	1	t	1	Psychotria marginata	Manacus vitellinus	43	0.06	0.07	0.07	0.07
1	1	0.87	1	cv	1	Psychotria marginata	C. fuscescens	43	0.06	0.07	0.05	0.06
1	1	0.98	1	t	1	Psychotria marginata	P. mentalis	43	0.06	0.07	0.09	0.07
1	1	0.93	1	t	1	Miconia impetiolaris	Manacus vitellinus	43	0.05	0.07	0.07	0.07
1	1	0.97	1	t	1	Miconia impetiolaris	P. mentalis	43	0.05	0.07	0.09	0.07
0	1	0.73	1	cv	1	Miconia impetiolaris	Mionectes oleagineus	43	0.05	0.07	0.04	0.07
0	1	0.82	1	t	1	Tachyphonus luctuosus	Miconia argentea	43	0.01	0.07	0.08	0.07
0	1	0.98	1	t	1	Manacus vitellinus	Miconia argentea	43	0.07	0.07	0.08	0.07
1	1	0.95	1	t	1	Manacus vitellinus	Psychotria horizontalis	43	0.07	0.07	0.06	0.07

0	1	0.94	1	cv	1	Manacus vitellinus	Psychotria hoffmannseggiana	43	0.07	0.07	0.06	0.07
0	1	0.90	1	t	1	Manacus vitellinus	Psychotria poeppigiana	43	0.07	0.07	0.04	0.07
1	1	0.96	1	t	1	Miconia minutiflora	P. mentalis	43	0.04	0.07	0.09	0.07
0	1	0.68	1	t	1	Miconia minutiflora	Mionectes oleagineus	43	0.04	0.07	0.04	0.07
1	1	0.99	1	cv	1	Miconia argentea	P. mentalis	43	0.08	0.07	0.09	0.07
0	1	0.79	1	t	1	Miconia argentea	Oryzoborus angolensis	43	0.08	0.07	0.01	0.07
1	1	0.92	1	t	1	Miconia argentea	Mionectes oleagineus	43	0.08	0.07	0.04	0.07
1	1	0.70	1	t	1	Psychotria horizontalis	Empidonax virescens	43	0.06	0.07	0.02	0.06
1	1	0.89	1	cv	1	Psychotria horizontalis	C. fuscescens	43	0.06	0.07	0.05	0.06
1	1	0.98	1	t	1	Psychotria horizontalis	P. mentalis	43	0.06	0.07	0.09	0.07
1	1	0.82	1	t	1	Psychotria horizontalis	Mionectes oleagineus	43	0.06	0.07	0.04	0.07
1	1	0.95	1	t	1	Psychotria deflexa	P. mentalis	43	0.04	0.07	0.09	0.07
0	1	0.87	1	cv	1	C. fuscescens	Psychotria hoffmannseggiana	43	0.05	0.06	0.06	0.07
1	1	0.98	1	t	1	Psychotria hoffmannseggiana	P. mentalis	43	0.06	0.07	0.09	0.07
1	1	0.86	1	t	1	Psychotria grandis	P. mentalis	43	0.01	0.08	0.09	0.07
1	1	0.96	1	t	1	P. mentalis	Psychotria poeppigiana	43	0.09	0.07	0.04	0.07
0	1	0.81	0	cv	1	Ramphocelus dimidiatus	C. ustulatus	43	0.01	0.07	0.08	0.07
0	1	0.86	0	t	1	Habia fuscicauda	Manacus vitellinus	43	0.03	0.07	0.07	0.07
0	0	0.46	0	t	1	Empidonax virescens	Psychotria deflexa	43	0.02	0.06	0.04	0.07
0	0	0.23	0	t	1	Trogon rufus	Psychotria emetica	43	0.01	0.06	0.02	0.07
0	0	0.24	0	cv	1	Ramphocelus dimidiatus	Empidonax virescens	43	0.01	0.07	0.02	0.06
0	1	0.79	0	t	1	C. ustulatus	Seiurus aurocapillus	43	0.08	0.07	0.01	0.06
0	1	0.86	0	t	1	Psychotria pittieri	Miconia argentea	43	0.02	0.07	0.08	0.07
0	1	0.65	0	t	1	Psychotria brachybotrya	Miconia impetiolaris	43	0.03	0.07	0.05	0.07

0	0	0.43	0	cv	1	Leptotila cassinii	Miconia minutiflora	43	0.01	0.07	0.04	0.07
0	0	0.16	0	t	1	D. striata	Tachyphonus luctuosus	43	0.01	0.07	0.01	0.07
0	1	0.60	0	t	1	Psychotria pittieri	C. minimus	43	0.02	0.07	0.05	0.06
0	1	0.68	0	t	1	Habia fuscicauda	Miconia impetiolaris	43	0.03	0.07	0.05	0.07
0	0	0.30	0	cv	1	Habia fuscicauda	D. striata	43	0.03	0.07	0.01	0.07
0	0	0.30	0	t	1	Habia fuscicauda	Euphonia fulvicrissa	43	0.03	0.07	0.01	0.06
0	0	0.45	0	t	1	Eucometis penicillata	Psychotria deflexa	43	0.02	0.07	0.04	0.07
0	1	0.63	0	t	1	Eucometis penicillata	C. minimus	43	0.02	0.07	0.05	0.06
0	1	0.71	0	cv	1	Schiffornis turdinus	C. minimus	43	0.03	0.06	0.05	0.06
0	0	0.15	0	t	1	Tachyphonus luctuosus	Psychotria grandis	43	0.01	0.07	0.01	0.08
0	0	0.18	0	t	1	Seiurus aurocapillus	Euphonia fulvicrissa	43	0.01	0.06	0.01	0.06
0	1	0.57	0	t	1	Euphonia fulvicrissa	Psychotria marginata	43	0.01	0.06	0.06	0.07
0	0	0.21	0	cv	1	Psychotria pittieri	Oryzoborus angolensis	43	0.02	0.07	0.01	0.07
1	1	0.65	0	t	1	Miconia lacera	Trogon rufus	43	0.07	0.07	0.01	0.06
0	1	0.59	0	t	1	Psychotria limonensis	Psychotria deflexa	43	0.04	0.07	0.04	0.07
0	1	0.69	0	t	1	Psychotria psychotrifolia	Psychotria horizontalis	43	0.02	0.08	0.06	0.07
0	1	0.62	0	cv	1	Schiffornis turdinus	Psychotria acuminata	43	0.03	0.06	0.04	0.07
0	1	0.76	0	t	1	C. fuscescens	Mionectes oleagineus	43	0.05	0.06	0.04	0.07
0	1	0.89	0	t	1	Pipra coronata	Empidonax virescens	43	0.09	0.07	0.02	0.06
0	1	0.72	0	t	1	Miconia minutiflora	Psychotria poeppigiana	43	0.04	0.07	0.04	0.07
0	1	0.61	0	cv	1	Habia fuscicauda	Miconia minutiflora	43	0.03	0.07	0.04	0.07
0	0	0.30	0	t	1	Eucometis penicillata	Psychotria emetica	43	0.02	0.07	0.02	0.07
0	1	0.77	0	t	1	Psychotria limonensis	Psychotria marginata	43	0.04	0.07	0.06	0.07
1	1	0.68	0	t	1	Psychotria brachybotrya	C. minimus	43	0.03	0.07	0.05	0.06
0	1	0.86	0	cv	1	Schiffornis turdinus	Manacus vitellinus	43	0.03	0.06	0.07	0.07
0	0	0.47	0	t	1	Schiffornis turdinus	Psychotria brachybotrya	43	0.03	0.06	0.03	0.07

0	0	0.42	0	t	1	Psychotria acuminata	Seiurus aurocapillus	43	0.04	0.07	0.01	0.06
0	0	0.38	0	t	1	Ramphocelus dimidiatus	Mionectes oleagineus	43	0.01	0.07	0.04	0.07
0	0	0.41	0	cv	1	Psychotria acuminata	Oryzoborus angolensis	43	0.04	0.07	0.01	0.07
0	1	0.87	0	t	1	Miconia lacera	Psychotria poeppigiana	43	0.07	0.07	0.04	0.07
0	0	0.43	0	t	1	Ramphocelus dimidiatus	Psychotria acuminata	43	0.01	0.07	0.04	0.07
0	0	0.44	0	t	1	Seiurus aurocapillus	Miconia minutiflora	43	0.01	0.06	0.04	0.07
0	0	0.49	0	cv	1	Empidonax virescens	Mionectes oleagineus	43	0.02	0.06	0.04	0.07
0	1	0.50	0	t	1	Psychotria pittieri	Miconia minutiflora	43	0.02	0.07	0.04	0.07
0	1	0.53	0	t	1	D. striata	C. fuscescens	43	0.01	0.07	0.05	0.06
1	1	0.94	0	t	1	Psychotria acuminata	Miconia argentea	43	0.04	0.07	0.08	0.07
0	0	0.17	0	cv	1	D. striata	Euphonia fulvicrissa	43	0.01	0.07	0.01	0.06
0	0	0.28	0	t	1	Ramphocelus dimidiatus	Psychotria brachybotrya	43	0.01	0.07	0.03	0.07
0	0	0.30	0	t	1	Habia fuscicauda	Oryzoborus angolensis	43	0.03	0.07	0.01	0.07
0	0	0.50	0	t	1	D. striata	Miconia impetiolaris	43	0.01	0.07	0.05	0.07
1	1	0.92	0	cv	1	Miconia lacera	Psychotria hoffmannseg giana	43	0.07	0.07	0.06	0.07
0	0	0.28	0	t	1	D. striata	Psychotria brachybotrya	43	0.01	0.07	0.03	0.07
0	1	0.60	0	t	1	Psychotria acuminata	Habia fuscicauda	43	0.04	0.07	0.03	0.07
0	0	0.17	0	t	1	Ramphocelus dimidiatus	Euphonia fulvicrissa	43	0.01	0.07	0.01	0.06
0	0	0.16	0	cv	1	Dendroica pensylvanica	Tachyphonus luctuosus	43	0.01	0.07	0.01	0.07
0	0	0.16	0	t	1	Trogon rufus	Psychotria grandis	43	0.01	0.06	0.01	0.08
0	0	0.35	0	t	1	Psychotria limonensis	Trogon rufus	43	0.04	0.07	0.01	0.06
0	1	0.71	0	t	1	Psychotria racemosa	Psychotria marginata	43	0.03	0.07	0.06	0.07
0	1	0.62	0	cv	1	Psychotria limonensis	Mionectes oleagineus	43	0.04	0.07	0.04	0.07
0	1	0.75	0	t	1	Trogon rufus	Manacus vitellinus	43	0.01	0.06	0.07	0.07
0	1	0.63	0	t	1	Psychotria psychotrifolia	Psychotria marginata	43	0.02	0.08	0.06	0.07

0	0	0.46	0	t	1	Eucometis penicillata	Psychotria limonensis	43	0.02	0.07	0.04	0.07
0	0	0.49	0	cv	1	Schiffornis turdinus	Habia fuscicauda	43	0.03	0.06	0.03	0.07
0	1	0.74	0	t	1	D. striata	Manacus vitellinus	43	0.01	0.07	0.07	0.07
0	1	0.56	0	t	1	Leptotila cassinii	Psychotria marginata	43	0.01	0.07	0.06	0.07
1	1	0.90	0	t	1	Miconia lacera	Miconia impetiolaris	43	0.07	0.07	0.05	0.07
0	0	0.22	0	cv	1	Psychotria psychotrifolia	Trogon rufus	43	0.02	0.08	0.01	0.06
0	0	0.17	0	t	1	Leptotila cassinii	Trogon rufus	43	0.01	0.07	0.01	0.06
0	0	0.43	0	t	1	Dendroica pensylvanica	Psychotria poeppigiana	43	0.01	0.07	0.04	0.07
0	1	0.91	0	t	1	Eucometis penicillata	P. mentalis	43	0.02	0.07	0.09	0.07
0	1	0.83	0	cv	1	Miconia lacera	Psychotria limonensis	43	0.07	0.07	0.04	0.07
0	1	0.75	0	t	1	Seiurus aurocapillus	Manacus vitellinus	43	0.01	0.06	0.07	0.07
0	0	0.24	0	t	1	Eucometis penicillata	Trogon rufus	43	0.02	0.07	0.01	0.06
0	0	0.24	0	t	1	Eucometis penicillata	Seiurus aurocapillus	43	0.02	0.07	0.01	0.06
0	1	0.55	0	cv	1	Schiffornis turdinus	Psychotria limonensis	43	0.03	0.06	0.04	0.07
0	0	0.39	0	t	1	Euphonia fulvicrissa	Mionectes oleagineus	43	0.01	0.06	0.04	0.07
0	0	0.16	0	t	1	Ramphocelus dimidiatus	D. striata	43	0.01	0.07	0.01	0.07
0	1	0.56	0	t	1	Miconia impetiolaris	Psychotria emetica	43	0.05	0.07	0.02	0.07
0	0	0.46	0	cv	1	Psychotria emetica	Mionectes oleagineus	43	0.02	0.07	0.04	0.07
0	0	0.17	0	t	1	Leptotila cassinii	Seiurus aurocapillus	43	0.01	0.07	0.01	0.06
0	0	0.42	0	t	1	Psychotria psychotrifolia	Psychotria deflexa	43	0.02	0.08	0.04	0.07
0	0	0.43	0	t	1	D. striata	Miconia minutiflora	43	0.01	0.07	0.04	0.07
0	1	0.65	0	cv	1	Miconia minutiflora	Psychotria deflexa	43	0.04	0.07	0.04	0.07
0	0	0.28	0	t	1	Psychotria brachybotrya	Trogon rufus	43	0.03	0.07	0.01	0.06
0	0	0.19	0	t	1	Psychotria psychotrifolia	Psychotria grandis	43	0.02	0.08	0.01	0.08
0	1	0.86	0	t	1	Ramphocelus dimidiatus	Pipra coronata	43	0.01	0.07	0.09	0.07

0	0	0.28	0	cv	1	Ramphocelus dimidiatus	Psychotria racemosa	43	0.01	0.07	0.03	0.07
0	1	0.71	0	t	1	Schiffornis turdinus	C. fuscescens	43	0.03	0.06	0.05	0.06
0	1	0.70	1	t	1	Solanum sp.	Ramphocelus carbo	44	0.06	0.05	0.01	0.04
0	1	0.80	1	t	1	Solanum sp.	Tangara gyrola	44	0.06	0.05	0.02	0.04
1	1	0.72	1	cv	1	Tangara mexicana	Calycolpus glaber	44	0.03	0.03	0.03	0.03
0	1	0.60	1	t	1	Tangara mexicana	Cecropia peltata	44	0.03	0.03	0.02	0.03
0	0	0.45	1	t	1	Tangara mexicana	Cordia bicolor	44	0.03	0.03	0.01	0.03
0	1	0.63	1	t	1	Tangara mexicana	Aegiphila integrifolia	44	0.03	0.03	0.03	0.03
0	1	0.53	1	cv	1	Tangara mexicana	Lacistema aggregatum	44	0.03	0.03	0.02	0.03
0	0	0.42	1	t	1	Tangara mexicana	Chiococca alba	44	0.03	0.03	0.01	0.04
1	1	0.67	1	t	1	Tangara mexicana	Psidium guajava	44	0.03	0.03	0.03	0.03
1	1	0.51	1	t	1	Tangara mexicana	Eugenia baileyi	44	0.03	0.03	0.01	0.03
0	0	0.40	1	cv	1	Tangara mexicana	Ilex sp.	44	0.03	0.03	0.01	0.04
0	0	0.45	1	t	1	Tangara mexicana	Laetia procera	44	0.03	0.03	0.01	0.03
0	1	0.52	1	t	1	Tangara mexicana	Protium heptaphyllum	44	0.03	0.03	0.02	0.05
1	1	0.75	1	t	1	Tangara mexicana	Myrcia sp.	44	0.03	0.03	0.04	0.03
0	1	0.50	1	cv	1	Tangara mexicana	Ficus citrifolia	44	0.03	0.03	0.02	0.04
0	0	0.49	1	t	1	Tangara mexicana	Didymopanax morototoni	44	0.03	0.03	0.01	0.03
0	0	0.46	1	t	1	Tangara mexicana	Ficus tobagensis	44	0.03	0.03	0.01	0.04
1	1	0.68	1	t	1	Tangara mexicana	Hieronyma caribaea	44	0.03	0.03	0.03	0.03
0	1	0.61	1	cv	1	Tangara mexicana	div. spp.	44	0.03	0.03	0.03	0.04
0	0	0.39	1	t	1	Tangara mexicana	Trema micrantha	44	0.03	0.03	0.01	0.04
0	0	0.50	1	t	1	Tangara mexicana	Miconia spp.	44	0.03	0.03	0.02	0.04
0	0	0.45	1	t	1	Tangara mexicana	Cordia curassavica	44	0.03	0.03	0.01	0.03
0	0	0.43	1	cv	1	Tangara mexicana	Rhipsalis sp.	44	0.03	0.03	0.01	0.04

0	0	0.44	1	t	1	Tangara mexicana	Ficus clusiifolia	44	0.03	0.03	0.01	0.04
0	1	0.61	1	t	1	Alchornea glandulosa	Cyanerpes caeruleus	44	0.02	0.03	0.03	0.04
0	0	0.28	1	t	1	Alchornea glandulosa	Thraupis episcopus	44	0.02	0.03	0.01	0.05
0	1	0.50	1	cv	1	Alchornea glandulosa	Thraupis palmarum	44	0.02	0.03	0.02	0.03
0	1	0.61	1	t	1	Alchornea glandulosa	Tangara guttata	44	0.02	0.03	0.03	0.03
0	0	0.35	1	t	1	Alchornea glandulosa	Dacnis cayana	44	0.02	0.03	0.01	0.03
0	0	0.30	1	t	1	Alchornea glandulosa	Chlorophanes spiza	44	0.02	0.03	0.01	0.03
0	0	0.44	1	cv	1	Alchornea glandulosa	Tangara gyrola	44	0.02	0.03	0.02	0.04
0	0	0.48	1	t	1	Clusia spp.	Cyanerpes caeruleus	44	0.01	0.04	0.03	0.04
0	0	0.24	1	t	1	Clusia spp.	Dacnis cayana	44	0.01	0.04	0.01	0.03
0	0	0.19	1	t	1	Clusia spp.	Cyanerpes cyaneus	44	0.01	0.04	0.01	0.04
0	0	0.21	1	cv	1	Clusia spp.	Chlorophanes spiza	44	0.01	0.04	0.01	0.03
0	0	0.35	1	t	1	Anacardium occidentale	Ramphocelus carbo	44	0.02	0.03	0.01	0.04
0	0	0.31	1	t	1	Coussarea paniculata	Thraupis episcopus	44	0.02	0.04	0.01	0.05
0	1	0.54	1	t	1	Coussarea paniculata	Thraupis palmarum	44	0.02	0.04	0.02	0.03
0	0	0.36	1	cv	1	Coussarea paniculata	Tachyphonus rufus	44	0.02	0.04	0.01	0.04
0	0	0.35	1	t	1	Coussarea paniculata	Ramphocelus carbo	44	0.02	0.04	0.01	0.04
1	1	0.78	1	t	1	Coussarea paniculata	Euphonia violacea	44	0.02	0.04	0.05	0.04
0	0	0.46	1	t	1	Calycolpus glaber	Thraupis episcopus	44	0.03	0.03	0.01	0.05
1	1	0.59	1	cv	1	Calycolpus glaber	Coereba flaveola	44	0.03	0.03	0.01	0.03
1	1	0.69	1	t	1	Calycolpus glaber	Thraupis palmarum	44	0.03	0.03	0.02	0.03
1	1	0.54	1	t	1	Calycolpus glaber	Dacnis cayana	44	0.03	0.03	0.01	0.03
0	1	0.51	1	t	1	Calycolpus glaber	Ramphocelus carbo	44	0.03	0.03	0.01	0.04
0	0	0.34	1	cv	1	Cecropia peltata	Thraupis episcopus	44	0.02	0.03	0.01	0.05
0	0	0.46	1	t	1	Cecropia peltata	Coereba flaveola	44	0.02	0.03	0.01	0.03

0	1	0.57	1	t	1	Cecropia peltata	Thraupis palmarum	44	0.02	0.03	0.02	0.03
0	0	0.39	1	t	1	Cecropia peltata	Tachyphonus rufus	44	0.02	0.03	0.01	0.04
0	1	0.68	1	cv	1	Cecropia peltata	Tangara guttata	44	0.02	0.03	0.03	0.03
0	0	0.41	1	t	1	Cecropia peltata	Dacnis cayana	44	0.02	0.03	0.01	0.03
0	0	0.37	1	t	1	Cecropia peltata	Chlorophanes spiza	44	0.02	0.03	0.01	0.03
0	0	0.38	1	t	1	Cecropia peltata	Ramphocelus carbo	44	0.02	0.03	0.01	0.04
0	1	0.51	1	cv	1	Cecropia peltata	Tangara gyrola	44	0.02	0.03	0.02	0.04
0	0	0.22	1	t	1	Cordia bicolor	Thraupis episcopus	44	0.01	0.03	0.01	0.05
0	0	0.33	1	t	1	Cordia bicolor	Coereba flaveola	44	0.01	0.03	0.01	0.03
0	0	0.43	1	t	1	Cordia bicolor	Thraupis palmarum	44	0.01	0.03	0.02	0.03
0	0	0.27	1	cv	1	Cordia bicolor	Tachyphonus rufus	44	0.01	0.03	0.01	0.04
0	0	0.37	1	t	1	Cordia bicolor	Tangara gyrola	44	0.01	0.03	0.02	0.04
0	0	0.19	1	t	1	Piper sp.	Thraupis episcopus	44	0.01	0.05	0.01	0.05
0	1	0.66	1	t	1	Piper sp.	Euphonia violacea	44	0.01	0.05	0.05	0.04
0	1	0.54	1	cv	1	Aegiphila integrifolia	Tangara gyrola	44	0.03	0.03	0.02	0.04
0	0	0.28	1	t	1	Lacistema aggregatum	Thraupis episcopus	44	0.02	0.03	0.01	0.05
0	1	0.51	1	t	1	Lacistema aggregatum	Thraupis palmarum	44	0.02	0.03	0.02	0.03
0	0	0.35	1	t	1	Lacistema aggregatum	Dacnis cayana	44	0.02	0.03	0.01	0.03
0	0	0.31	1	cv	1	Lacistema aggregatum	Chlorophanes spiza	44	0.02	0.03	0.01	0.03
0	0	0.32	1	t	1	Lacistema aggregatum	Ramphocelus carbo	44	0.02	0.03	0.01	0.04
0	0	0.44	1	t	1	Lacistema aggregatum	Tangara gyrola	44	0.02	0.03	0.02	0.04
0	0	0.23	1	t	1	Chiococca alba	Chlorophanes spiza	44	0.01	0.04	0.01	0.03
0	0	0.34	1	cv	1	Chiococca alba	Tangara gyrola	44	0.01	0.04	0.02	0.04
0	1	0.67	1	t	1	Chiococca alba	Euphonia violacea	44	0.01	0.04	0.05	0.04
1	1	0.89	1	t	1	Cyanerpes caeruleus	Wulffia baccata	44	0.03	0.04	0.06	0.04

1	1	0.80	1	t	1	Cyanerpes caeruleus	Myrcia sp.	44	0.03	0.04	0.04	0.03
1	1	0.74	1	cv	1	Cyanerpes caeruleus	Hieronyma caribaea	44	0.03	0.04	0.03	0.03
1	1	0.68	1	t	1	Cyanerpes caeruleus	div. spp.	44	0.03	0.04	0.03	0.04
1	1	0.81	1	t	1	Cyanerpes caeruleus	Sapium aucuparium	44	0.03	0.04	0.04	0.03
0	0	0.46	1	t	1	Cyanerpes caeruleus	Trema micrantha	44	0.03	0.04	0.01	0.04
0	1	0.57	1	cv	1	Cyanerpes caeruleus	Miconia spp.	44	0.03	0.04	0.02	0.04
0	1	0.51	1	t	1	Cyanerpes caeruleus	Ficus clusiifolia	44	0.03	0.04	0.01	0.04
0	0	0.25	1	t	1	unid. spp.	Tachyphonus rufus	44	0.01	0.04	0.01	0.04
0	0	0.24	1	t	1	unid. spp.	Ramphocelus carbo	44	0.01	0.04	0.01	0.04
0	0	0.45	1	cv	1	Psidium guajava	Ramphocelus carbo	44	0.03	0.03	0.01	0.04
0	0	0.35	1	t	1	Marcgravia sp.	Tangara gyrola	44	0.01	0.04	0.02	0.04
0	0	0.17	1	t	1	Sloanea stipitata	Thraupis episcopus	44	0.01	0.04	0.01	0.05
0	0	0.25	1	t	1	Sloanea stipitata	Coereba flaveola	44	0.01	0.04	0.01	0.03
0	0	0.35	1	cv	1	Sloanea stipitata	Thraupis palmarum	44	0.01	0.04	0.02	0.03
0	0	0.20	1	t	1	Sloanea stipitata	Tachyphonus rufus	44	0.01	0.04	0.01	0.04
0	1	0.77	1	t	1	Sloanea stipitata	Tachyphonus luctuosus	44	0.01	0.04	0.07	0.05
0	0	0.45	1	t	1	Sloanea stipitata	Tangara guttata	44	0.01	0.04	0.03	0.03
0	0	0.22	1	cv	1	Sloanea stipitata	Dacnis cayana	44	0.01	0.04	0.01	0.03
0	0	0.17	1	t	1	Sloanea stipitata	Cyanerpes cyaneus	44	0.01	0.04	0.01	0.04
0	0	0.19	1	t	1	Sloanea stipitata	Ramphocelus carbo	44	0.01	0.04	0.01	0.04
0	0	0.29	1	t	1	Sloanea stipitata	Tangara gyrola	44	0.01	0.04	0.02	0.04
1	1	0.85	1	cv	1	Podocarpus spp.	Euphonia violacea	44	0.03	0.05	0.05	0.04
0	0	0.26	1	t	1	Thraupis episcopus	Eugenia baileyi	44	0.01	0.05	0.01	0.03
0	0	0.19	1	t	1	Thraupis episcopus	Doliocarpus dentatus	44	0.01	0.05	0.01	0.03
0	0	0.22	1	t	1	Thraupis episcopus	Laetia procera	44	0.01	0.05	0.01	0.03

0	0	0.27	1	cv	1	Thraupis episcopus	Protium heptaphyllum	44	0.01	0.05	0.02	0.05
0	1	0.75	1	t	1	Thraupis episcopus	Castilloa elastica	44	0.01	0.05	0.07	0.06
0	0	0.25	1	t	1	Thraupis episcopus	Didymopanax morototoni	44	0.01	0.05	0.01	0.03
0	0	0.27	1	t	1	Thraupis episcopus	Artocarpus incisa	44	0.01	0.05	0.01	0.03
0	0	0.18	1	cv	1	Thraupis episcopus	Trema micrantha	44	0.01	0.05	0.01	0.04
0	1	0.57	1	t	1	Thraupis episcopus	Vismia spp.	44	0.01	0.05	0.05	0.05
0	0	0.25	1	t	1	Thraupis episcopus	Miconia spp.	44	0.01	0.05	0.02	0.04
0	0	0.22	1	t	1	Thraupis episcopus	Cordia curassavica	44	0.01	0.05	0.01	0.03
0	0	0.21	1	cv	1	Thraupis episcopus	Ficus clusiifolia	44	0.01	0.05	0.01	0.04
1	1	0.72	1	t	1	Wulffia baccata	Dacnis cayana	44	0.06	0.04	0.01	0.03
0	1	0.69	1	t	1	Wulffia baccata	Ramphocelus carbo	44	0.06	0.04	0.01	0.04
1	1	0.79	1	t	1	Wulffia baccata	Tangara gyrola	44	0.06	0.04	0.02	0.04
1	0	0.49	1	cv	1	Eugenia baileyi	Thraupis palmarum	44	0.01	0.03	0.02	0.03
0	0	0.48	1	t	1	Davilla aspera	Thraupis palmarum	44	0.01	0.03	0.02	0.03
0	0	0.30	1	t	1	Davilla aspera	Tachyphonus rufus	44	0.01	0.03	0.01	0.04
0	0	0.29	1	t	1	Davilla aspera	Ramphocelus carbo	44	0.01	0.03	0.01	0.04
0	1	0.51	1	cv	1	Phytolacca rivinoides	Thraupis palmarum	44	0.02	0.03	0.02	0.03
0	0	0.34	1	t	1	Phytolacca rivinoides	Tachyphonus rufus	44	0.02	0.03	0.01	0.04
0	0	0.32	1	t	1	Phytolacca rivinoides	Ramphocelus carbo	44	0.02	0.03	0.01	0.04
0	0	0.38	1	t	1	Byrsonima spicata	Dacnis cayana	44	0.02	0.04	0.01	0.03
0	0	0.47	1	cv	1	Byrsonima spicata	Tangara gyrola	44	0.02	0.04	0.02	0.04
1	0	0.42	1	t	1	Coereba flaveola	Cupania sp.	44	0.01	0.03	0.02	0.03
0	0	0.37	1	t	1	Coereba flaveola	Didymopanax morototoni	44	0.01	0.03	0.01	0.03
0	0	0.31	1	t	1	Coereba flaveola	Epiphyllum hookeri	44	0.01	0.03	0.01	0.03
1	1	0.56	1	cv	1	Coereba flaveola	Hieronyma caribaea	44	0.01	0.03	0.03	0.03

0	0	0.48	1	t	1	Coereba flaveola	div. spp.	44	0.01	0.03	0.03	0.04
1	1	0.66	1	t	1	Coereba flaveola	Sapium aucuparium	44	0.01	0.03	0.04	0.03
0	0	0.28	1	t	1	Coereba flaveola	Trema micrantha	44	0.01	0.03	0.01	0.04
0	0	0.37	1	cv	1	Coereba flaveola	Miconia spp.	44	0.01	0.03	0.02	0.04
0	0	0.33	1	t	1	Coereba flaveola	Cordia curassavica	44	0.01	0.03	0.01	0.03
0	0	0.31	1	t	1	Coereba flaveola	Rhipsalis sp.	44	0.01	0.03	0.01	0.04
1	0	0.42	1	t	1	Coereba flaveola	Rourea surinamensis	44	0.01	0.03	0.02	0.03
0	0	0.32	1	cv	1	Coereba flaveola	Ficus clusiifolia	44	0.01	0.03	0.01	0.04
0	0	0.23	1	t	1	Pisonia eggersiana	Tachyphonus rufus	44	0.01	0.04	0.01	0.04
0	0	0.22	1	t	1	Ilex sp.	Ramphocelus carbo	44	0.01	0.04	0.01	0.04
0	0	0.32	1	t	1	Ilex sp.	Tangara gyrola	44	0.01	0.04	0.02	0.04
0	0	0.30	1	cv	1	Cupania sp.	Cyanerpes cyaneus	44	0.02	0.03	0.01	0.04
0	1	0.60	1	t	1	Matayba guianensis	Cyanerpes cyaneus	44	0.06	0.05	0.01	0.04
0	0	0.40	1	t	1	Doliocarpus dentatus	Thraupis palmarum	44	0.01	0.03	0.02	0.03
0	0	0.25	1	t	1	Doliocarpus dentatus	Dacnis cayana	44	0.01	0.03	0.01	0.03
0	0	0.20	1	cv	1	Doliocarpus dentatus	Cyanerpes cyaneus	44	0.01	0.03	0.01	0.04
0	0	0.43	1	t	1	Thraupis palmarum	Laetia procera	44	0.02	0.03	0.01	0.03
0	0	0.50	1	t	1	Thraupis palmarum	Protium heptaphyllum	44	0.02	0.03	0.02	0.05
1	0	0.40	1	t	1	Thraupis palmarum	Roystonea oleracea	44	0.02	0.03	0.01	0.03
0	0	0.47	1	cv	1	Thraupis palmarum	Didymopanax morototoni	44	0.02	0.03	0.01	0.03
1	1	0.66	1	t	1	Thraupis palmarum	Pinzona calineoides	44	0.02	0.03	0.03	0.03
0	0	0.37	1	t	1	Thraupis palmarum	Trema micrantha	44	0.02	0.03	0.01	0.04
0	0	0.47	1	t	1	Thraupis palmarum	Miconia spp.	44	0.02	0.03	0.02	0.04
0	0	0.41	1	cv	1	Thraupis palmarum	Rhipsalis sp.	44	0.02	0.03	0.01	0.04
0	0	0.41	1	t	1	Thraupis palmarum	Ficus clusiifolia	44	0.02	0.03	0.01	0.04

0	0	0.26	1	t	1	Anthurium gracile	Tachyphonus rufus	44	0.01	0.05	0.01	0.04
0	0	0.25	1	t	1	Anthurium gracile	Ramphocelus carbo	44	0.01	0.05	0.01	0.04
0	1	0.70	1	cv	1	Anthurium gracile	Euphonia violacea	44	0.01	0.05	0.05	0.04
0	0	0.27	1	t	1	Laetia procera	Tachyphonus rufus	44	0.01	0.03	0.01	0.04
0	0	0.29	1	t	1	Laetia procera	Dacnis cayana	44	0.01	0.03	0.01	0.03
0	0	0.26	1	t	1	Laetia procera	Ramphocelus carbo	44	0.01	0.03	0.01	0.04
0	0	0.28	1	cv	1	Henriettea sp.	Tachyphonus rufus	44	0.01	0.04	0.01	0.04
0	0	0.27	1	t	1	Henriettea sp.	Ramphocelus carbo	44	0.01	0.04	0.01	0.04
0	0	0.38	1	t	1	Henriettea sp.	Tangara gyrola	44	0.01	0.04	0.02	0.04
0	1	0.58	1	t	1	Myrcia leptoclada	Tangara gyrola	44	0.03	0.04	0.02	0.04
1	1	0.85	1	cv	1	Myrcia leptoclada	Euphonia violacea	44	0.03	0.04	0.05	0.04
0	0	0.32	1	t	1	Protium heptaphyllum	Tachyphonus rufus	44	0.02	0.05	0.01	0.04
0	1	0.86	1	t	1	Protium heptaphyllum	Tachyphonus luctuosus	44	0.02	0.05	0.07	0.05
0	0	0.34	1	t	1	Protium heptaphyllum	Dacnis cayana	44	0.02	0.05	0.01	0.03
0	0	0.30	1	cv	1	Protium heptaphyllum	Chlorophanes spiza	44	0.02	0.05	0.01	0.03
0	0	0.31	1	t	1	Protium heptaphyllum	Ramphocelus carbo	44	0.02	0.05	0.01	0.04
0	0	0.43	1	t	1	Protium heptaphyllum	Tangara gyrola	44	0.02	0.05	0.02	0.04
1	1	0.75	1	t	1	Protium heptaphyllum	Euphonia violacea	44	0.02	0.05	0.05	0.04
0	0	0.31	1	cv	1	Geophila herbacea	Tachyphonus rufus	44	0.01	0.03	0.01	0.04
1	1	0.80	1	t	1	Myrcia sp.	Tangara guttata	44	0.04	0.03	0.03	0.03
0	0	0.31	1	t	1	Tachyphonus rufus	Ficus citrifolia	44	0.01	0.04	0.02	0.04
0	1	0.81	1	t	1	Tachyphonus rufus	Clidemia spp.	44	0.01	0.04	0.07	0.05
0	0	0.25	1	cv	1	Tachyphonus rufus	Aechmaea mertensii	44	0.01	0.04	0.01	0.04
0	0	0.25	1	t	1	Tachyphonus rufus	Gravisia aquilega	44	0.01	0.04	0.01	0.04
0	0	0.24	1	t	1	Tachyphonus rufus	Aechmaea nudicaulis	44	0.01	0.04	0.01	0.05

0	0	0.19	1	t	1	Tachyphonus rufus	Anthurium scandens	44	0.01	0.04	0.01	0.05
0	1	0.71	1	cv	1	Tachyphonus rufus	Isertia parviflora	44	0.01	0.04	0.06	0.05
0	0	0.46	1	t	1	Tachyphonus rufus	Aechmaea dichlamydea	44	0.01	0.04	0.03	0.03
0	0	0.49	1	t	1	Tachyphonus rufus	Hieronyma caribaea	44	0.01	0.04	0.03	0.03
0	0	0.41	1	t	1	Tachyphonus rufus	div. spp.	44	0.01	0.04	0.03	0.04
0	0	0.22	1	cv	1	Tachyphonus rufus	Trema micrantha	44	0.01	0.04	0.01	0.04
0	0	0.30	1	t	1	Tachyphonus rufus	Miconia spp.	44	0.01	0.04	0.02	0.04
0	0	0.27	1	t	1	Tachyphonus rufus	Cordia curassavica	44	0.01	0.04	0.01	0.03
0	0	0.23	1	t	1	Tachyphonus rufus	Aechmaea porteoides	44	0.01	0.04	0.01	0.04
0	0	0.26	1	cv	1	Tachyphonus rufus	Ficus clusiifolia	44	0.01	0.04	0.01	0.04
0	0	0.41	1	t	1	Ficus citrifolia	Tangara gyrola	44	0.02	0.04	0.02	0.04
0	1	0.74	1	t	1	Ficus citrifolia	Euphonia violacea	44	0.02	0.04	0.05	0.04
0	1	0.70	1	t	1	Tachyphonus luctuosus	Eugenia malaccensis	44	0.07	0.05	0.01	0.06
0	1	0.72	1	cv	1	Tachyphonus luctuosus	Anthurium scandens	44	0.07	0.05	0.01	0.05
0	1	0.82	1	t	1	Tachyphonus luctuosus	Miconia spp.	44	0.07	0.05	0.02	0.04
0	0	0.27	1	t	1	Norantea guianensis	Dacnis cayana	44	0.01	0.04	0.01	0.03
0	0	0.36	1	t	1	Norantea guianensis	Tangara gyrola	44	0.01	0.04	0.02	0.04
1	1	0.77	1	cv	1	Clidemia spp.	Ramphocelus carbo	44	0.07	0.05	0.01	0.04
1	1	0.85	1	t	1	Clidemia spp.	Tangara gyrola	44	0.07	0.05	0.02	0.04
1	1	0.96	1	t	1	Clidemia spp.	Euphonia violacea	44	0.07	0.05	0.05	0.04
0	0	0.24	1	t	1	Aechmaea mertensii	Ramphocelus carbo	44	0.01	0.04	0.01	0.04
0	1	0.75	1	cv	1	Castilloa elastica	Ramphocelus carbo	44	0.07	0.06	0.01	0.04
1	1	0.75	1	t	1	Tangara guttata	Hieronyma caribaea	44	0.03	0.03	0.03	0.03
1	1	0.82	1	t	1	Tangara guttata	Sapium aucuparium	44	0.03	0.03	0.04	0.03
0	0	0.47	1	t	1	Tangara guttata	Trema micrantha	44	0.03	0.03	0.01	0.04

1	1	0.83	1	cv	1	Tangara guttata	Psychotria spp.	44	0.03	0.03	0.04	0.04
0	1	0.57	1	t	1	Tangara	Miconia spp.	44	0.03	0.03	0.02	0.04
1	1	0.51	1	t	1	Tangara guttata	Ficus clusiifolia	44	0.03	0.03	0.01	0.04
0	0	0.24	1	t	1	Gravisia aquilega	Ramphocelus carbo	44	0.01	0.04	0.01	0.04
0	0	0.32	1	cv	1	Didymopanax morototoni	Dacnis cayana	44	0.01	0.03	0.01	0.03
0	0	0.28	1	t	1	Didymopanax morototoni	Chlorophanes spiza	44	0.01	0.03	0.01	0.03
0	0	0.29	1	t	1	Didymopanax morototoni	Ramphocelus carbo	44	0.01	0.03	0.01	0.04
0	0	0.41	1	t	1	Didymopanax morototoni	Tangara gyrola	44	0.01	0.03	0.02	0.04
0	1	0.66	1	cv	1	Aechmaea nudicaulis	Euphonia violacea	44	0.01	0.05	0.05	0.04
0	0	0.20	1	t	1	Ficus religiosa	Ramphocelus carbo	44	0.01	0.04	0.01	0.04
0	0	0.25	1	t	1	Ficus tobagensis	Chlorophanes spiza	44	0.01	0.04	0.01	0.03
0	0	0.26	1	t	1	Ficus tobagensis	Ramphocelus carbo	44	0.01	0.04	0.01	0.04
0	0	0.38	1	cv	1	Ficus tobagensis	Tangara gyrola	44	0.01	0.04	0.02	0.04
0	1	0.71	1	t	1	Ficus tobagensis	Euphonia violacea	44	0.01	0.04	0.05	0.04
0	1	0.60	1	t	1	Anthurium scandens	Euphonia violacea	44	0.01	0.05	0.05	0.04
0	1	0.66	1	t	1	Isertia parviflora	Ramphocelus carbo	44	0.06	0.05	0.01	0.04
1	1	0.51	1	cv	1	Dacnis cayana	Hieronyma caribaea	44	0.01	0.03	0.03	0.03
0	0	0.43	1	t	1	Dacnis cayana	div. spp.	44	0.01	0.03	0.03	0.04
1	1	0.62	1	t	1	Dacnis cayana	Sapium aucuparium	44	0.01	0.03	0.04	0.03
0	0	0.24	1	t	1	Dacnis cayana	Trema micrantha	44	0.01	0.03	0.01	0.04
0	0	0.32	1	cv	1	Dacnis cayana	Miconia spp.	44	0.01	0.03	0.02	0.04
1	0	0.37	1	t	1	Dacnis cayana	Rourea surinamensis	44	0.01	0.03	0.02	0.03
0	0	0.27	1	t	1	Dacnis cayana	Ficus clusiifolia	44	0.01	0.03	0.01	0.04
0	0	0.43	1	t	1	Aechmaea dichlamydea	Ramphocelus carbo	44	0.03	0.03	0.01	0.04
0	0	0.45	1	cv	1	Hieronyma caribaea	Chlorophanes spiza	44	0.03	0.03	0.01	0.03
0	1	0.60	1	t	1	Hieronyma caribaea	Tangara gyrola	44	0.03	0.03	0.02	0.04
0	1	0.54	1	t	1	Cyanerpes cyaneus	Sapium aucuparium	44	0.01	0.04	0.04	0.03

0	0	0.18	1	t	1	Cyanerpes cyaneus	Trema micrantha	44	0.01	0.04	0.01	0.04
0	0	0.26	1	cv	1	Cyanerpes cyaneus	Miconia spp.	44	0.01	0.04	0.02	0.04
0	0	0.22	1	t	1	Cyanerpes cyaneus	Ficus clusiifolia	44	0.01	0.04	0.01	0.04
0	0	0.38	1	t	1	div. spp.	Chlorophanes spiza	44	0.03	0.04	0.01	0.03
0	1	0.52	1	t	1	div. spp.	Tangara gyrola	44	0.03	0.04	0.02	0.04
0	1	0.81	1	cv	1	div. spp.	Euphonia violacea	44	0.03	0.04	0.05	0.04
0	1	0.55	1	t	1	Sapium aucuparium	Chlorophanes spiza	44	0.04	0.03	0.01	0.03
0	1	0.69	1	t	1	Sapium aucuparium	Tangara gyrola	44	0.04	0.03	0.02	0.04
0	0	0.20	1	t	1	Trema micrantha	Chlorophanes spiza	44	0.01	0.04	0.01	0.03
0	0	0.21	1	cv	1	Trema micrantha	Ramphocelus carbo	44	0.01	0.04	0.01	0.04
0	0	0.32	1	t	1	Trema micrantha	Tangara gyrola	44	0.01	0.04	0.02	0.04
0	1	0.65	1	t	1	Trema micrantha	Euphonia violacea	44	0.01	0.04	0.05	0.04
1	1	0.90	1	t	1	Rollinia exsucca	Euphonia violacea	44	0.05	0.05	0.05	0.04
0	0	0.29	1	cv	1	Chlorophanes spiza	Miconia spp.	44	0.01	0.03	0.02	0.04
0	0	0.33	1	t	1	Chlorophanes spiza	Rourea surinamensis	44	0.01	0.03	0.02	0.03
0	0	0.24	1	t	1	Chlorophanes spiza	Ficus clusiifolia	44	0.01	0.03	0.01	0.04
0	0	0.29	1	t	1	Ramphocelus carbo	Miconia spp.	44	0.01	0.04	0.02	0.04
0	0	0.25	1	cv	1	Ramphocelus carbo	Cordia curassavica	44	0.01	0.04	0.01	0.03
1	1	0.70	1	t	1	Psychotria spp.	Tangara gyrola	44	0.04	0.04	0.02	0.04
0	0	0.41	1	t	1	Miconia spp.	Tangara gyrola	44	0.02	0.04	0.02	0.04
0	1	0.73	1	t	1	Miconia spp.	Euphonia violacea	44	0.02	0.04	0.05	0.04
0	0	0.35	1	cv	1	Tangara gyrola	Ficus clusiifolia	44	0.02	0.04	0.01	0.04
0	1	0.68	1	t	1	Rhipsalis sp.	Euphonia violacea	44	0.01	0.04	0.05	0.04
0	1	0.66	1	t	1	Euphonia violacea	Ficus clusiifolia	44	0.05	0.04	0.01	0.04
0	0	0.22	0	t	1	Piper sp.	Ramphocelus carbo	44	0.01	0.05	0.01	0.04

0	1	0.73	0	cv	1	Solanum sp.	Cordia curassavica	44	0.06	0.05	0.01	0.03
0	0	0.40	0	t	1	Lacistema aggregatum	Coereba flaveola	44	0.02	0.03	0.01	0.03
0	1	0.64	0	t	1	Byrsonima spicata	Tangara guttata	44	0.02	0.04	0.03	0.03
0	1	0.94	0	t	1	Castilloa elastica	Vismia spp.	44	0.07	0.06	0.05	0.05
0	1	0.84	0	cv	1	Geophila herbacea	Castilloa elastica	44	0.01	0.03	0.07	0.06
1	1	0.94	0	t	1	Solanum sp.	Euphonia violacea	44	0.06	0.05	0.05	0.04
1	1	0.52	0	t	1	Calycolpus glaber	Roystonea oleracea	44	0.03	0.03	0.01	0.03
0	0	0.36	0	t	1	Byrsonima spicata	Epiphyllum hookeri	44	0.02	0.04	0.01	0.03
0	1	0.64	0	cv	1	Cordia bicolor	Psychotria spp.	44	0.01	0.03	0.04	0.04
0	0	0.43	0	t	1	Aegiphila integrifolia	Aechmaea mertensii	44	0.03	0.03	0.01	0.04
0	0	0.43	0	t	1	Aegiphila integrifolia	Marcgravia sp.	44	0.03	0.03	0.01	0.04
0	0	0.27	0	t	1	Aechmaea mertensii	Dacnis cayana	44	0.01	0.04	0.01	0.03
0	0	0.31	0	cv	1	Tangara gyrola	Aechmaea porteoides	44	0.02	0.04	0.01	0.04
0	1	0.75	0	t	1	Cecropia peltata	Rollinia exsucca	44	0.02	0.03	0.05	0.05
0	1	0.88	0	t	1	Solanum sp.	Pinzona calineoides	44	0.06	0.05	0.03	0.03
0	1	0.57	0	t	1	Chiococca alba	Myrcia sp.	44	0.01	0.04	0.04	0.03
0	0	0.36	0	cv	1	Byrsonima spicata	Rhipsalis sp.	44	0.02	0.04	0.01	0.04
0	0	0.46	0	t	1	Aechmaea dichlamydea	Ficus clusiifolia	44	0.03	0.03	0.01	0.04
0	0	0.31	0	t	1	Alchornea glandulosa	Doliocarpus dentatus	44	0.02	0.03	0.01	0.03
0	1	0.55	0	t	1	Ilex sp.	Myrcia sp.	44	0.01	0.04	0.04	0.03
0	1	0.72	0	cv	1	Norantea guianensis	Isertia parviflora	44	0.01	0.04	0.06	0.05
0	1	0.58	0	t	1	Cecropia peltata	div. spp.	44	0.02	0.03	0.03	0.04
0	0	0.13	0	t	1	Eugenia malaccensis	Anthurium scandens	44	0.01	0.06	0.01	0.05
0	1	0.57	0	t	1	Tangara mexicana	Coussarea paniculata	44	0.03	0.03	0.02	0.04
0	0	0.32	0	cv	1	Lacistema aggregatum	Aechmaea nudicaulis	44	0.02	0.03	0.01	0.05
0	0	0.38	0	t	1	Cecropia peltata	Piper sp.	44	0.02	0.03	0.01	0.05
0	0	0.27	0	t	1	Cordia bicolor	unid. spp.	44	0.01	0.03	0.01	0.04

1	1	0.80	0	t	1	Podocarpus spp.	Rollinia exsucca	44	0.03	0.05	0.05	0.05
0	1	0.65	0	cv	1	Sloanea stipitata	Isertia parviflora	44	0.01	0.04	0.06	0.05
0	1	0.51	0	t	1	Myrcia sp.	Ficus religiosa	44	0.04	0.03	0.01	0.04
0	0	0.47	0	t	1	Myrcia leptoclada	Rhipsalis sp.	44	0.03	0.04	0.01	0.04
1	1	0.64	0	t	1	Calycolpus glaber	Rourea surinamensis	44	0.03	0.03	0.02	0.03
1	1	0.71	0	cv	1	Wulffia baccata	Ficus clusiifolia	44	0.06	0.04	0.01	0.04
0	0	0.35	0	t	1	Anacardium occidentale	Doliocarpus dentatus	44	0.02	0.03	0.01	0.03
0	1	0.50	0	t	1	Myrcia sp.	Cyanerpes cyaneus	44	0.04	0.03	0.01	0.04
0	0	0.38	0	t	1	Cupania sp.	Cordia curassavica	44	0.02	0.03	0.01	0.03
0	0	0.41	0	cv	1	Lacistema aggregatum	Geophila herbacea	44	0.02	0.03	0.01	0.03
1	0	0.33	0	t	1	Alchornea glandulosa	Roystonea oleracea	44	0.02	0.03	0.01	0.03
0	1	0.72	0	t	1	Alchornea glandulosa	Vismia spp.	44	0.02	0.03	0.05	0.05
0	0	0.50	0	t	1	Cordia bicolor	Psidium guajava	44	0.01	0.03	0.03	0.03
0	0	0.46	0	cv	1	Chiococca alba	Aechmaea dichlamydea	44	0.01	0.04	0.03	0.03
0	0	0.25	0	t	1	Cordia bicolor	Ilex sp.	44	0.01	0.03	0.01	0.04
1	1	0.68	0	t	1	Tangara mexicana	Pinzona calineoides	44	0.03	0.03	0.03	0.03
0	0	0.47	0	t	1	Aegiphila integrifolia	Ficus tobagensis	44	0.03	0.03	0.01	0.04
0	0	0.25	0	cv	1	Piper sp.	Laetia procera	44	0.01	0.05	0.01	0.03
0	1	0.76	0	t	1	Phytolacca rivinoides	Euphonia violacea	44	0.02	0.03	0.05	0.04
0	0	0.37	0	t	1	Cordia bicolor	Phytolacca rivinoides	44	0.01	0.03	0.02	0.03
0	0	0.19	0	t	1	Anthurium scandens	Ficus clusiifolia	44	0.01	0.05	0.01	0.04
0	1	0.60	0	cv	1	Trema micrantha	Vismia spp.	44	0.01	0.04	0.05	0.05
0	1	0.75	0	t	1	Matayba guianensis	Tangara gyrola	44	0.06	0.05	0.02	0.04
0	0	0.28	0	t	1	Pisonia eggersiana	Miconia spp.	44	0.01	0.04	0.02	0.04
0	0	0.21	0	t	1	Marcgravia sp.	Thraupis episcopus	44	0.01	0.04	0.01	0.05
0	1	0.97	0	cv	1	Solanum sp.	Castilloa elastica	44	0.06	0.05	0.07	0.06

0	0	0.19	0	t	1	Aechmaea mertensii	Anthurium scandens	44	0.01	0.04	0.01	0.05
0	1	0.71	0	t	1	Cupania sp.	Sapium aucuparium	44	0.02	0.03	0.04	0.03
0	0	0.36	0	t	1	Eugenia baileyi	Henriettea sp.	44	0.01	0.03	0.01	0.04
1	1	0.92	0	cv	1	Isertia parviflora	Vismia spp.	44	0.06	0.05	0.05	0.05
0	0	0.23	0	t	1	Piper sp.	Chiococca alba	44	0.01	0.05	0.01	0.04
0	0	0.40	0	t	1	Byrsonima spicata	Henriettea sp.	44	0.02	0.04	0.01	0.04
0	0	0.33	0	t	1	Pisonia eggersiana	Rourea surinamensis	44	0.01	0.04	0.02	0.03
0	1	0.58	0	cv	1	Ficus religiosa	Vismia spp.	44	0.01	0.04	0.05	0.05
0	0	0.16	0	t	1	Eugenia malaccensis	Trema micrantha	44	0.01	0.06	0.01	0.04
0	0	0.34	0	t	1	Lacistema aggregatum	Marcgravia sp.	44	0.02	0.03	0.01	0.04
0	0	0.23	0	t	1	Anthurium gracile	Ficus religiosa	44	0.01	0.05	0.01	0.04
0	1	0.82	0	cv	1	Norantea guianensis	Clidemia spp.	44	0.01	0.04	0.07	0.05
0	1	0.50	0	t	1	Cyanerpes caeruleus	Rhipsalis sp.	44	0.03	0.04	0.01	0.04
0	0	0.23	0	t	1	Doliocarpus dentatus	Aechmaea nudicaulis	44	0.01	0.03	0.01	0.05
1	0	0.33	0	t	1	Geophila herbacea	Dacnis cayana	44	0.01	0.03	0.01	0.03
0	0	0.34	0	cv	1	Anacardium occidentale	Pisonia eggersiana	44	0.02	0.03	0.01	0.04
0	1	0.73	0	t	1	Coussarea paniculata	Rollinia exsucca	44	0.02	0.04	0.05	0.05
0	1	0.71	0	t	1	Cordia bicolor	Matayba guianensis	44	0.01	0.03	0.06	0.05
1	1	0.57	0	t	1	Cyanerpes caeruleus	Geophila herbacea	44	0.03	0.04	0.01	0.03
0	0	0.28	0	cv	1	Trema micrantha	Miconia spp.	44	0.01	0.04	0.02	0.04
0	0	0.26	0	t	1	Norantea guianensis	Rhipsalis sp.	44	0.01	0.04	0.01	0.04
0	0	0.36	0	t	1	Coussarea paniculata	Roystonea oleracea	44	0.02	0.04	0.01	0.03
0	0	0.24	0	t	1	Chlorophanes spiza	Rhipsalis sp.	44	0.01	0.03	0.01	0.04
0	1	0.70	0	cv	1	Solanum sp.	Piper sp.	44	0.06	0.05	0.01	0.05
0	0	0.23	0	t	1	Sloanea stipitata	Ficus tobagensis	44	0.01	0.04	0.01	0.04
0	1	0.60	0	t	1	Coussarea paniculata	Aechmaea dichlamydea	44	0.02	0.04	0.03	0.03

0	1	0.68	0	t	1	Solanum sp.	Aechmaea porteoides	44	0.06	0.05	0.01	0.04
0	0	0.26	0	cv	1	Henriettea sp.	Chlorophanes spiza	44	0.01	0.04	0.01	0.03
0	0	0.20	0	t	1	Chiococca alba	Sloanea stipitata	44	0.01	0.04	0.01	0.04
0	0	0.28	0	t	1	Henriettea sp.	Epiphyllum hookeri	44	0.01	0.04	0.01	0.03
0	1	0.53	0	t	1	div. spp.	Rourea surinamensis	44	0.03	0.04	0.02	0.03
0	0	0.46	0	cv	1	Ilex sp.	Myrcia leptoclada	44	0.01	0.04	0.03	0.04
0	0	0.17	0	t	1	Clusia spp.	Anthurium scandens	44	0.01	0.04	0.01	0.05
0	1	0.90	0	t	1	Matayba guianensis	Sapium aucuparium	44	0.06	0.05	0.04	0.03
0	0	0.25	0	t	1	Norantea guianensis	Aechmaea nudicaulis	44	0.01	0.04	0.01	0.05
0	0	0.42	0	cv	1	Marcgravia sp.	div. spp.	44	0.01	0.04	0.03	0.04
0	0	0.30	0	t	1	Piper sp.	Artocarpus incisa	44	0.01	0.05	0.01	0.03
1	0	0.44	0	t	1	Artocarpus incisa	Rourea surinamensis	44	0.01	0.03	0.02	0.03
0	0	0.41	0	t	1	Coussarea paniculata	Henriettea sp.	44	0.02	0.04	0.01	0.04
0	0	0.15	0	cv	1	Thraupis episcopus	Anthurium scandens	44	0.01	0.05	0.01	0.05
0	0	0.28	0	t	1	Norantea guianensis	Cordia curassavica	44	0.01	0.04	0.01	0.03
0	0	0.50	0	t	1	Marcgravia sp.	Hieronyma caribaea	44	0.01	0.04	0.03	0.03
0	0	0.23	0	t	1	Thraupis episcopus	Ficus tobagensis	44	0.01	0.05	0.01	0.04
0	1	0.53	0	cv	1	Calycolpus glaber	Aechmaea mertensii	44	0.03	0.03	0.01	0.04
0	1	0.74	0	t	1	Henriettea sp.	Isertia parviflora	44	0.01	0.04	0.06	0.05
0	0	0.43	0	t	1	Tangara mexicana	unid. spp.	44	0.03	0.03	0.01	0.04
0	0	0.20	0	t	1	Eugenia malaccensis	Ficus tobagensis	44	0.01	0.06	0.01	0.04
0	1	0.59	0	cv	1	Sapium aucuparium	Rhipsalis sp.	44	0.04	0.03	0.01	0.04
0	0	0.40	0	t	1	Sloanea stipitata	Aechmaea dichlamydea	44	0.01	0.04	0.03	0.03
0	1	0.78	0	t	1	Piper sp.	Castilloa elastica	44	0.01	0.05	0.07	0.06
0	1	0.88	0	t	1	Tachyphonus luctuosus	div. spp.	44	0.07	0.05	0.03	0.04
0	0	0.23	0	cv	1	Clusia spp.	Rhipsalis sp.	44	0.01	0.04	0.01	0.04

0	0	0.26	0	t	1	Alchornea glandulosa	Anthurium scandens	44	0.02	0.03	0.01	0.05
0	1	0.62	0	t	1	Coussarea paniculata	Myrcia leptoclada	44	0.02	0.04	0.03	0.04
0	0	0.46	0	t	1	Coussarea paniculata	Protium heptaphyllum	44	0.02	0.04	0.02	0.05
0	1	0.67	0	cv	1	Cecropia peltata	Cyanerpes caeruleus	44	0.02	0.03	0.03	0.04
0	0	0.30	0	t	1	Aechmaea nudicaulis	Artocarpus incisa	44	0.01	0.05	0.01	0.03
0	1	0.66	0	t	1	Psychotria	Miconia spp.	44	0.04	0.04	0.02	0.04
0	1	0.93	0	t	1	Cyanerpes caeruleus	Tachyphonus luctuosus	44	0.03	0.04	0.07	0.05
0	0	0.44	0	cv	1	Laetia procera	div. spp.	44	0.01	0.03	0.03	0.04
0	0	0.22	0	t	1	Sloanea stipitata	Cordia curassavica	44	0.01	0.04	0.01	0.03
1	1	0.64	0	t	1	Myrcia sp.	Artocarpus incisa	44	0.04	0.03	0.01	0.03
0	1	0.59	0	t	1	Myrcia leptoclada	Rourea surinamensis	44	0.03	0.04	0.02	0.03
0	1	0.86	0	cv	1	Isertia parviflora	Pinzona calineoides	44	0.06	0.05	0.03	0.03
0	1	0.62	0	t	1	Gravisia aquilega	Psychotria spp.	44	0.01	0.04	0.04	0.04
0	1	0.61	0	t	1	Pisonia eggersiana	Vismia spp.	44	0.01	0.04	0.05	0.05
0	1	0.92	0	t	1	Cyanerpes caeruleus	Castilloa elastica	44	0.03	0.04	0.07	0.06
0	0	0.18	0	cv	1	Eugenia malaccensis	Rhipsalis sp.	44	0.01	0.06	0.01	0.04
0	0	0.41	0	t	1	Lacistema aggregatum	Eugenia baileyi	44	0.02	0.03	0.01	0.03
0	0	0.43	0	t	1	Ilex sp.	Aechmaea dichlamydea	44	0.01	0.04	0.03	0.03
1	1	0.92	0	t	1	Wulffia baccata	Psychotria spp.	44	0.06	0.04	0.04	0.04
0	0	0.39	0	cv	1	Piper sp.	div. spp.	44	0.01	0.05	0.03	0.04
0	1	0.83	0	t	1	Henriettea sp.	Tachyphonus luctuosus	44	0.01	0.04	0.07	0.05
0	0	0.34	0	t	1	Phytolacca rivinoides	Aechmaea mertensii	44	0.02	0.03	0.01	0.04
0	0	0.26	0	t	1	Phytolacca rivinoides	Eugenia malaccensis	44	0.02	0.03	0.01	0.06
0	0	0.35	0	cv	1	Chiococca alba	Rourea surinamensis	44	0.01	0.04	0.02	0.03
0	0	0.17	0	t	1	Thraupis episcopus	Ficus religiosa	44	0.01	0.05	0.01	0.04
0	0	0.23	0	t	1	Marcgravia sp.	Aechmaea porteoides	44	0.01	0.04	0.01	0.04
0	1	0.78	0	t	1	Tachyphonus luctuosus	Epiphyllum hookeri	44	0.07	0.05	0.01	0.03

0	0	0.36	0	cv	1	Rhipsalis sp.	Rourea surinamensis	44	0.01	0.04	0.02	0.03
0	0	0.29	0	t	1	Aechmaea nudicaulis	Miconia spp.	44	0.01	0.05	0.02	0.04
0	0	0.26	0	t	1	Aechmaea mertensii	Rhipsalis sp.	44	0.01	0.04	0.01	0.04
0	0	0.36	0	t	1	Coussarea paniculata	Chiococca alba	44	0.02	0.04	0.01	0.04
0	1	0.57	0	cv	1	Phytolacca rivinoides	Aechmaea dichlamydea	44	0.02	0.03	0.03	0.03
0	1	0.76	0	t	1	Ficus citrifolia	Isertia parviflora	44	0.02	0.04	0.06	0.05
0	1	0.65	0	t	1	Cecropia peltata	Myrcia leptoclada	44	0.02	0.03	0.03	0.04
0	1	0.57	0	t	1	Eugenia baileyi	Myrcia leptoclada	44	0.01	0.03	0.03	0.04
0	0	0.29	0	cv	1	Henriettea sp.	Rhipsalis sp.	44	0.01	0.04	0.01	0.04
0	0	0.47	0	t	1	Anacardium occidentale	Phytolacca rivinoides	44	0.02	0.03	0.02	0.03
1	0	0.32	0	t	1	Geophila herbacea	Rhipsalis sp.	44	0.01	0.03	0.01	0.04
0	1	0.54	0	t	1	Myrcia leptoclada	Ficus citrifolia	44	0.03	0.04	0.02	0.04
1	1	0.91	0	cv	1	Wulffia baccata	Sapium aucuparium	44	0.06	0.04	0.04	0.03
0	0	0.31	0	t	1	Ficus citrifolia	Ficus clusiifolia	44	0.02	0.04	0.01	0.04
0	0	0.21	0	t	1	Gravisia aquilega	Cyanerpes cyaneus	44	0.01	0.04	0.01	0.04
0	1	0.69	0	t	1	Isertia parviflora	Ficus clusiifolia	44	0.06	0.05	0.01	0.04
0	0	0.42	0	cv	1	Tangara mexicana	Epiphyllum hookeri	44	0.03	0.03	0.01	0.03
0	0	0.31	0	t	1	Marcgravia sp.	Didymopanax morototoni	44	0.01	0.04	0.01	0.03
0	0	0.38	0	t	1	Thraupis palmarum	Aechmaea nudicaulis	44	0.02	0.03	0.01	0.05
1	0	0.36	0	t	1	Anacardium occidentale	Roystonea oleracea	44	0.02	0.03	0.01	0.03
0	1	0.63	0	cv	1	Norantea guianensis	Psychotria spp.	44	0.01	0.04	0.04	0.04
0	1	0.58	0	t	1	Pisonia eggersiana	Rollinia exsucca	44	0.01	0.04	0.05	0.05
0	0	0.22	0	t	1	Roystonea oleracea	Ficus religiosa	44	0.01	0.03	0.01	0.04
0	1	0.61	0	t	1	Chiococca alba	Psychotria spp.	44	0.01	0.04	0.04	0.04
1	0	0.26	0	cv	1	Roystonea oleracea	Ficus clusiifolia	44	0.01	0.03	0.01	0.04
1	1	0.77	0	t	1	Aegiphila integrifolia	Sapium aucuparium	44	0.03	0.03	0.04	0.03

1	1	0.60	0	t	1	Psychotria	Rhipsalis sp.	44	0.04	0.04	0.01	0.04
0	0	0.20	0	t	1	Anthurium gracile	Anthurium scandens	44	0.01	0.05	0.01	0.05
0	0	0.23	0	cv	1	Norantea guianensis	Trema micrantha	44	0.01	0.04	0.01	0.04
0	0	0.31	0	t	1	Phytolacca rivinoides	Chlorophanes spiza	44	0.02	0.03	0.01	0.03
0	0	0.22	0	t	1	Anthurium gracile	Cyanerpes cyaneus	44	0.01	0.05	0.01	0.04
0	0	0.49	0	t	1	Clusia spp.	Tangara guttata	44	0.01	0.04	0.03	0.03
0	1	0.50	0	cv	1	Cyanerpes caeruleus	Marcgravia sp.	44	0.03	0.04	0.01	0.04
0	0	0.18	0	t	1	Chiococca alba	Eugenia malaccensis	44	0.01	0.04	0.01	0.06
0	1	0.73	0	t	1	Cordia bicolor	Isertia parviflora	44	0.01	0.03	0.06	0.05
0	1	0.68	0	t	1	Aegiphila integrifolia	Podocarpus spp.	44	0.03	0.03	0.03	0.05
1	1	0.77	0	cv	1	Calycolpus glaber	Cyanerpes caeruleus	44	0.03	0.03	0.03	0.04
0	0	0.46	0	t	1	Calycolpus glaber	Cyanerpes cyaneus	44	0.03	0.03	0.01	0.04
0	0	0.28	0	t	1	Coereba flaveola	Aechmaea porteoides	44	0.01	0.03	0.01	0.04
0	1	0.86	0	t	1	Alchornea glandulosa	Tachyphonus luctuosus	44	0.02	0.03	0.07	0.05
0	1	0.52	0	cv	1	Lacistema aggregatum	div. spp.	44	0.02	0.03	0.03	0.04
0	1	0.90	0	t	1	Matayba guianensis	Psychotria spp.	44	0.06	0.05	0.04	0.04
0	0	0.27	0	t	1	Geophila herbacea	Ficus religiosa	44	0.01	0.03	0.01	0.04
0	0	0.22	0	t	1	Piper sp.	Aechmaea nudicaulis	44	0.01	0.05	0.01	0.05
0	1	0.50	0	cv	1	Tangara guttata	Epiphyllum hookeri	44	0.03	0.03	0.01	0.03
0	0	0.46	0	t	1	Aechmaea mertensii	Aechmaea dichlamydea	44	0.01	0.04	0.03	0.03
0	0	0.40	0	t	1	Lacistema aggregatum	Miconia spp.	44	0.02	0.03	0.02	0.04
0	0	0.36	0	t	1	Alchornea glandulosa	Ficus tobagensis	44	0.02	0.03	0.01	0.04
0	0	0.25	0	cv	1	Artocarpus incisa	Anthurium scandens	44	0.01	0.03	0.01	0.05
0	0	0.32	0	t	1	Lacistema aggregatum	Doliocarpus dentatus	44	0.02	0.03	0.01	0.03
0	0	0.40	0	t	1	Aegiphila integrifolia	Pisonia eggersiana	44	0.03	0.03	0.01	0.04
0	0	0.40	0	t	1	Podocarpus spp.	Thraupis episcopus	44	0.03	0.05	0.01	0.05

0	1	0.73	0	cv	1	Chiococca alba	Wulffia baccata	44	0.01	0.04	0.06	0.04
0	0	0.33	0	t	1	Davilla aspera	Laetia procera	44	0.01	0.03	0.01	0.03
0	1	0.65	0	t	1	Ficus tobagensis	Psychotria spp.	44	0.01	0.04	0.04	0.04
0	0	0.40	0	t	1	Alchornea glandulosa	Coereba flaveola	44	0.02	0.03	0.01	0.03
0	1	0.92	0	cv	1	Tachyphonus luctuosus	Tangara guttata	44	0.07	0.05	0.03	0.03
0	1	0.97	0	t	1	Wulffia baccata	Castilloa elastica	44	0.06	0.04	0.07	0.06
0	1	0.85	0	t	1	Coereba flaveola	Tachyphonus luctuosus	44	0.01	0.03	0.07	0.05
0	1	0.60	0	t	1	unid. spp.	Sapium aucuparium	44	0.01	0.04	0.04	0.03
1	1	0.81	0	cv	1	Psidium guajava	Psychotria spp.	44	0.03	0.03	0.04	0.04
0	0	0.41	0	t	1	Tangara mexicana	Piper sp.	44	0.03	0.03	0.01	0.05
0	0	0.27	0	t	1	Anthurium gracile	Ficus clusiifolia	44	0.01	0.05	0.01	0.04
0	1	0.69	0	t	1	Matayba guianensis	Ficus tobagensis	44	0.06	0.05	0.01	0.04
0	0	0.28	0	cv	1	Davilla aspera	Aechmaea porteoides	44	0.01	0.03	0.01	0.04
0	0	0.45	0	t	1	Psidium guajava	Aechmaea nudicaulis	44	0.03	0.03	0.01	0.05
0	0	0.38	0	t	1	Cordia bicolor	Cupania sp.	44	0.01	0.03	0.02	0.03
0	0	0.37	0	t	1	Anacardium occidentale	Tachyphonus rufus	44	0.02	0.03	0.01	0.04
0	0	0.29	0	cv	1	unid. spp.	Henriettea sp.	44	0.01	0.04	0.01	0.04
0	0	0.22	0	t	1	Dacnis cayana	Cyanerpes cyaneus	44	0.01	0.03	0.01	0.04
0	1	0.54	0	t	1	Anthurium gracile	Tangara guttata	44	0.01	0.05	0.03	0.03
0	1	0.89	0	t	1	Cecropia peltata	Clidemia spp.	44	0.02	0.03	0.07	0.05
0	0	0.21	0	cv	1	Aechmaea mertensii	Ficus religiosa	44	0.01	0.04	0.01	0.04
0	0	0.25	0	t	1	Clusia spp.	Cordia curassavica	44	0.01	0.04	0.01	0.03
0	0	0.48	0	t	1	Byrsonima spicata	Cupania sp.	44	0.02	0.04	0.02	0.03
0	0	0.31	0	t	1	Doliocarpus dentatus	Artocarpus incisa	44	0.01	0.03	0.01	0.03
0	0	0.38	0	cv	1	Ilex sp.	Thraupis palmarum	44	0.01	0.04	0.02	0.03
0	0	0.37	0	t	1	Aegiphila integrifolia	Sloanea stipitata	44	0.03	0.03	0.01	0.04

0	1	0.68	0	t	1	Ficus citrifolia	Psychotria spp.	44	0.02	0.04	0.04	0.04
0	0	0.28	0	t	1	Laetia procera	Norantea guianensis	44	0.01	0.03	0.01	0.04
0	0	0.37	0	cv	1	Ficus citrifolia	Didymopanax morototoni	44	0.02	0.04	0.01	0.03
0	1	0.55	0	t	1	Rollinia exsucca	Chlorophanes spiza	44	0.05	0.05	0.01	0.03
0	0	0.30	0	t	1	Ficus citrifolia	Roystonea oleracea	44	0.02	0.04	0.01	0.03
0	0	0.25	0	t	1	Clusia spp.	Laetia procera	44	0.01	0.04	0.01	0.03
0	1	0.77	0	cv	1	Solanum sp.	Eugenia baileyi	44	0.06	0.05	0.01	0.03
0	1	0.81	0	t	1	Pinzona calineoides	Rollinia exsucca	44	0.03	0.03	0.05	0.05
0	1	0.71	0	t	1	Epiphyllum hookeri	Isertia parviflora	44	0.01	0.03	0.06	0.05
0	1	0.98	0	t	1	Tachyphonus luctuosus	Castilloa elastica	44	0.07	0.05	0.07	0.06
0	0	0.33	0	cv	1	Protium heptaphyllum	Norantea guianensis	44	0.02	0.05	0.01	0.04
0	0	0.38	0	t	1	Aegiphila integrifolia	Ficus religiosa	44	0.03	0.03	0.01	0.04
0	0	0.16	0	t	1	Eugenia malaccensis	Chlorophanes spiza	44	0.01	0.06	0.01	0.03
0	0	0.46	0	t	1	Hieronyma caribaea	Ramphocelus carbo	44	0.03	0.03	0.01	0.04
0	0	0.25	0	cv	1	Cordia bicolor	Chlorophanes spiza	44	0.01	0.03	0.01	0.03
0	0	0.26	0	t	1	Didymopanax morototoni	Cyanerpes cyaneus	44	0.01	0.03	0.01	0.04
0	1	0.51	0	t	1	Anthurium gracile	Pinzona calineoides	44	0.01	0.05	0.03	0.03
1	1	0.60	0	t	1	Calycolpus glaber	Eugenia baileyi	44	0.03	0.03	0.01	0.03
0	0	0.49	0	cv	1	Marcgravia sp.	Myrcia leptoclada	44	0.01	0.04	0.03	0.04
0	1	0.64	0	t	1	unid. spp.	Vismia spp.	44	0.01	0.04	0.05	0.05
0	1	0.73	0	t	1	Ficus tobagensis	Isertia parviflora	44	0.01	0.04	0.06	0.05
0	1	0.55	0	t	1	Thraupis episcopus	Psychotria spp.	44	0.01	0.05	0.04	0.04
1	1	0.52	0	cv	1	Tangara guttata	Dacnis cayana	44	0.03	0.03	0.01	0.03
0	0	0.24	0	t	1	Eugenia baileyi	Eugenia malaccensis	44	0.01	0.03	0.01	0.06
0	0	0.31	0	t	1	Pisonia eggersiana	Protium heptaphyllum	44	0.01	0.04	0.02	0.05
0	0	0.42	0	t	1	Tangara guttata	Anthurium scandens	44	0.03	0.03	0.01	0.05

0	0	0.25	0	cv	1	Cordia bicolor	Aechmaea porteoides	44	0.01	0.03	0.01	0.04
0	1	0.70	0	t	1	Coereba flaveola	Vismia spp.	44	0.01	0.03	0.05	0.05
0	1	0.55	0	t	1	Cordia bicolor	Tangara guttata	44	0.01	0.03	0.03	0.03
0	0	0.49	0	t	1	Calycolpus glaber	Pisonia eggersiana	44	0.03	0.03	0.01	0.04
0	0	0.24	0	cv	1	Norantea guianensis	Chlorophanes spiza	44	0.01	0.04	0.01	0.03
0	1	0.64	0	t	1	Roystonea oleracea	Vismia spp.	44	0.01	0.03	0.05	0.05
0	1	0.62	0	t	1	Henriettea sp.	Myrcia sp.	44	0.01	0.04	0.04	0.03
0	0	0.24	0	t	1	Piper sp.	Rhipsalis sp.	44	0.01	0.05	0.01	0.04
0	1	0.69	0	cv	1	Phytolacca rivinoides	Sapium aucuparium	44	0.02	0.03	0.04	0.03
0	1	0.79	0	t	1	Tachyphonus luctuosus	Dacnis cayana	44	0.07	0.05	0.01	0.03
0	0	0.39	0	t	1	Coussarea paniculata	Anthurium gracile	44	0.02	0.04	0.01	0.05
0	0	0.43	0	t	1	Podocarpus spp.	Aechmaea porteoides	44	0.03	0.05	0.01	0.04
1	1	0.64	1	cv	1	Malus	Turdus pilaris	45	0.03	0.06	0.03	0.04
1	1	0.92	1	t	1	Malus sylvestris	Erithacus rubecula	45	0.03	0.06	0.07	0.05
1	1	0.72	1	t	1	Malus sylvestris	Turdus philomelos	45	0.03	0.06	0.04	0.04
1	1	0.78	1	t	1	Malus	Turdus iliacus	45	0.03	0.06	0.05	0.04
1	1	0.93	1	cv	1	Malus sylvestris	Turdus merula	45	0.03	0.06	0.08	0.05
1	1	0.85	1	t	1	Malus sylvestris	Turdus viscivorus	45	0.03	0.06	0.06	0.04
1	1	0.82	1	t	1	Malus sylvestris	Corvus corone	45	0.03	0.06	0.05	0.04
1	1	0.61	1	t	1	Malus sylvestris	Sturnus vulgaris	45	0.03	0.06	0.03	0.04
1	1	0.56	1	cv	1	Prunus avium	Jay	45	0.04	0.06	0.02	0.04
1	1	0.69	1	t	1	Prunus avium	Sylvia borin	45	0.04	0.06	0.03	0.04
1	1	0.72	1	t	1	Prunus avium	Magpie	45	0.04	0.06	0.04	0.04
1	1	0.77	1	t	1	Prunus avium	Turdus philomelos	45	0.04	0.06	0.04	0.04
1	1	0.95	1	cv	1	Prunus avium	Turdus merula	45	0.04	0.06	0.08	0.05
1	1	0.88	1	t	1	Prunus avium	Turdus viscivorus	45	0.04	0.06	0.06	0.04
1	1	0.92	1	t	1	Prunus avium	Sylvia atricapilla	45	0.04	0.06	0.07	0.04
1	1	0.56	1	t	1	Prunus avium	Columba palumbus	45	0.04	0.06	0.02	0.04
1	1	0.85	1	cv	1	Prunus avium	Corvus corone	45	0.04	0.06	0.05	0.04

1	1	0.67	1	t	1	Prunus avium	Sturnus vulgaris	45	0.04	0.06	0.03	0.04
0	0	0.32	1	t	1	Jay	Sorbus aucuparia	45	0.02	0.04	0.02	0.06
0	0	0.44	1	t	1	Jay	Sambucus nigra	45	0.02	0.04	0.03	0.06
1	0	0.44	1	cv	1	Jay	Sorbus aria	45	0.02	0.04	0.03	0.06
1	1	0.67	1	t	1	Sylvia borin	Rubus fruticosus	45	0.03	0.04	0.04	0.06
1	1	0.53	1	t	1	Sylvia borin	Ribes rubrum	45	0.03	0.04	0.02	0.06
1	1	0.60	1	t	1	Sylvia borin	Prunus padus	45	0.03	0.04	0.03	0.06
0	0	0.44	1	cv	1	Sylvia borin	Sorbus aucuparia	45	0.03	0.04	0.02	0.06
1	1	0.57	1	t	1	Sylvia borin	Viburnum lantana	45	0.03	0.04	0.03	0.06
1	1	0.57	1	t	1	Sylvia borin	Sambucus nigra	45	0.03	0.04	0.03	0.06
1	1	0.80	1	t	1	Sylvia borin	Bryonia dioica	45	0.03	0.04	0.05	0.06
1	0	0.42	1	cv	1	Turdus torquatus	Taxus baccata	45	0.01	0.04	0.03	0.06
1	0	0.45	1	t	1	Turdus torquatus	Hedera helix	45	0.01	0.04	0.04	0.06
1	1	0.92	1	t	1	Taxus baccata	Erithacus rubecula	45	0.03	0.06	0.07	0.05
1	1	0.72	1	t	1	Taxus baccata	Turdus philomelos	45	0.03	0.06	0.04	0.04
1	1	0.78	1	cv	1	Taxus baccata	Turdus iliacus	45	0.03	0.06	0.05	0.04
1	1	0.93	1	t	1	Taxus baccata	Turdus merula	45	0.03	0.06	0.08	0.05
1	1	0.84	1	t	1	Taxus baccata	Turdus viscivorus	45	0.03	0.06	0.06	0.04
1	1	0.90	1	t	1	Taxus baccata	Sylvia atricapilla	45	0.03	0.06	0.07	0.04
1	1	0.60	1	cv	1	Taxus baccata	Sturnus vulgaris	45	0.03	0.06	0.03	0.04
1	1	0.73	1	t	1	Magpie	Cornus sanguinea	45	0.04	0.04	0.04	0.06
1	1	0.67	1	t	1	Magpie	Ligustrum vulgare	45	0.04	0.04	0.03	0.06
1	1	0.64	1	t	1	Magpie	Prunus padus	45	0.04	0.04	0.03	0.06
1	0	0.48	1	cv	1	Magpie	Sorbus aucuparia	45	0.04	0.04	0.02	0.06
1	1	0.70	1	t	1	Magpie	Prunus spinosa	45	0.04	0.04	0.04	0.06
1	1	0.61	1	t	1	Magpie	Sambucus nigra	45	0.04	0.04	0.03	0.06
1	1	0.56	1	t	1	Magpie	Rubus idaeus	45	0.04	0.04	0.02	0.06
1	1	0.61	1	cv	1	Magpie	Sorbus aria	45	0.04	0.04	0.03	0.06
1	1	0.93	1	t	1	Rubus fruticosus	Erithacus rubecula	45	0.04	0.06	0.07	0.05

1	1	0.75	1	t	1	Rubus fruticosus	Turdus philomelos	45	0.04	0.06	0.04	0.04
1	1	0.94	1	t	1	Rubus fruticosus	Turdus merula	45	0.04	0.06	0.08	0.05
1	0	0.47	1	cv	1	Rubus fruticosus	Sylvia communis	45	0.04	0.06	0.02	0.04
1	1	0.91	1	t	1	Rubus fruticosus	Sylvia atricapilla	45	0.04	0.06	0.07	0.04
1	1	0.72	1	t	1	Rubus fruticosus	Gallinula chloropus	45	0.04	0.06	0.04	0.04
1	1	0.59	1	t	1	Rubus fruticosus	Sylvia curruca	45	0.04	0.06	0.03	0.04
1	1	0.63	1	cv	1	Rubus fruticosus	Sturnus vulgaris	45	0.04	0.06	0.03	0.04
1	1	0.64	1	t	1	Turdus pilaris	Rhamnus catharticus	45	0.03	0.04	0.03	0.06
1	1	0.64	1	t	1	Turdus pilaris	Ligustrum vulgare	45	0.03	0.04	0.03	0.06
1	1	0.67	1	t	1	Turdus pilaris	Prunus spinosa	45	0.03	0.04	0.04	0.06
1	1	0.64	1	cv	1	Turdus pilaris	Crataegus spp.	45	0.03	0.04	0.03	0.06
1	1	0.57	1	t	1	Turdus pilaris	Ilex aquifolium	45	0.03	0.04	0.03	0.06
1	1	0.67	1	t	1	Turdus pilaris	Hedera helix	45	0.03	0.04	0.04	0.06
1	1	0.64	1	t	1	Turdus pilaris	Rosa canina	45	0.03	0.04	0.03	0.06
1	1	0.58	1	cv	1	Turdus pilaris	Sambucus nigra	45	0.03	0.04	0.03	0.06
1	1	0.57	1	t	1	Turdus pilaris	Tamus communis	45	0.03	0.04	0.03	0.06
1	1	0.58	1	t	1	Turdus pilaris	Sorbus aria	45	0.03	0.04	0.03	0.06
1	1	0.94	1	t	1	Juniperus communis	Erithacus rubecula	45	0.04	0.06	0.07	0.05
1	1	0.95	1	cv	1	Juniperus communis	Turdus merula	45	0.04	0.06	0.08	0.05
1	1	0.89	1	t	1	Juniperus communis	Turdus viscivorus	45	0.04	0.06	0.06	0.04
1	1	0.99	1	t	1	Arum maculatum	Erithacus rubecula	45	0.08	0.06	0.07	0.05
1	1	0.99	1	t	1	Arum maculatum	Turdus merula	45	0.08	0.06	0.08	0.05
1	1	0.91	1	cv	1	Erithacus rubecula	Rhamnus catharticus	45	0.07	0.05	0.03	0.06
1	1	0.93	1	t	1	Erithacus rubecula	Cornus sanguinea	45	0.07	0.05	0.04	0.06
1	1	0.91	1	t	1	Erithacus rubecula	Ligustrum vulgare	45	0.07	0.05	0.03	0.06
1	1	0.86	1	t	1	Erithacus rubecula	Ribes rubrum	45	0.07	0.05	0.02	0.06
1	1	0.90	1	cv	1	Erithacus rubecula	Prunus padus	45	0.07	0.05	0.03	0.06

1	1	0.82	1	t	1	Erithacus rubecula	Sorbus aucuparia	45	0.07	0.05	0.02	0.06
1	1	0.84	1	t	1	Erithacus rubecula	Honeysuckle (perfoliate)	45	0.07	0.05	0.02	0.06
1	1	0.79	1	t	1	Erithacus rubecula	Viscum album	45	0.07	0.05	0.01	0.06
1	1	0.92	1	cv	1	Erithacus rubecula	Prunus spinosa	45	0.07	0.05	0.04	0.06
1	1	0.84	1	t	1	Erithacus rubecula	Euonymus europaeus	45	0.07	0.05	0.02	0.06
1	1	0.88	1	t	1	Erithacus rubecula	Viburnum opulus	45	0.07	0.05	0.03	0.06
1	1	0.79	1	t	1	Erithacus rubecula	Solanum dulcamara	45	0.07	0.05	0.01	0.06
1	1	0.91	1	cv	1	Erithacus rubecula	Crataegus spp.	45	0.07	0.05	0.03	0.06
1	1	0.84	1	t	1	Erithacus rubecula	Lonicera spp.	45	0.07	0.05	0.02	0.06
1	1	0.88	1	t	1	Erithacus rubecula	Ilex aquifolium	45	0.07	0.05	0.03	0.06
1	1	0.92	1	t	1	Erithacus rubecula	Hedera helix	45	0.07	0.05	0.04	0.06
1	1	0.91	1	cv	1	Erithacus rubecula	Rosa canina	45	0.07	0.05	0.03	0.06
1	1	0.88	1	t	1	Erithacus rubecula	Viburnum lantana	45	0.07	0.05	0.03	0.06
1	1	0.88	1	t	1	Erithacus rubecula	Sambucus nigra	45	0.07	0.05	0.03	0.06
1	1	0.86	1	t	1	Erithacus rubecula	Rubus idaeus	45	0.07	0.05	0.02	0.06
1	1	0.88	1	cv	1	Erithacus rubecula	Tamus communis	45	0.07	0.05	0.03	0.06
1	1	0.88	1	t	1	Erithacus rubecula	Sorbus aria	45	0.07	0.05	0.03	0.06
1	1	0.96	1	t	1	Erithacus rubecula	Bryonia dioica	45	0.07	0.05	0.05	0.06
1	1	0.72	1	t	1	Turdus philomelos	Rhamnus catharticus	45	0.04	0.04	0.03	0.06
1	1	0.77	1	cv	1	Turdus philomelos	Cornus sanguinea	45	0.04	0.04	0.04	0.06
1	1	0.72	1	t	1	Turdus philomelos	Ligustrum vulgare	45	0.04	0.04	0.03	0.06
1	1	0.62	1	t	1	Turdus philomelos	Ribes rubrum	45	0.04	0.04	0.02	0.06
1	1	0.69	1	t	1	Turdus philomelos	Prunus padus	45	0.04	0.04	0.03	0.06
1	1	0.54	1	cv	1	Turdus philomelos	Sorbus aucuparia	45	0.04	0.04	0.02	0.06
1	1	0.58	1	t	1	Turdus philomelos	Honeysuckle (perfoliate)	45	0.04	0.04	0.02	0.06

1	0	0.49	1	t	1	Turdus philomelos	Viscum album	45	0.04	0.04	0.01	0.06
1	1	0.75	1	t	1	Turdus philomelos	Prunus spinosa	45	0.04	0.04	0.04	0.06
1	1	0.58	1	cv	1	Turdus philomelos	Euonymus europaeus	45	0.04	0.04	0.02	0.06
1	1	0.66	1	t	1	Turdus philomelos	Viburnum opulus	45	0.04	0.04	0.03	0.06
1	0	0.49	1	t	1	Turdus philomelos	Solanum dulcamara	45	0.04	0.04	0.01	0.06
1	1	0.72	1	t	1	Turdus philomelos	Crataegus spp.	45	0.04	0.04	0.03	0.06
1	1	0.58	1	cv	1	Turdus philomelos	Lonicera spp.	45	0.04	0.04	0.02	0.06
1	1	0.66	1	t	1	Turdus philomelos	Ilex aquifolium	45	0.04	0.04	0.03	0.06
1	1	0.75	1	t	1	Turdus philomelos	Hedera helix	45	0.04	0.04	0.04	0.06
1	1	0.72	1	t	1	Turdus philomelos	Rosa canina	45	0.04	0.04	0.03	0.06
1	1	0.66	1	cv	1	Turdus philomelos	Viburnum lantana	45	0.04	0.04	0.03	0.06
1	1	0.66	1	t	1	Turdus philomelos	Sambucus nigra	45	0.04	0.04	0.03	0.06
1	1	0.66	1	t	1	Turdus philomelos	Tamus communis	45	0.04	0.04	0.03	0.06
1	1	0.66	1	t	1	Turdus philomelos	Sorbus aria	45	0.04	0.04	0.03	0.06
1	1	0.86	1	cv	1	Turdus philomelos	Bryonia dioica	45	0.04	0.04	0.05	0.06
1	1	0.78	1	t	1	Rhamnus catharticus	Turdus iliacus	45	0.03	0.06	0.05	0.04
1	1	0.93	1	t	1	Rhamnus catharticus	Turdus merula	45	0.03	0.06	0.08	0.05
1	1	0.84	1	t	1	Rhamnus catharticus	Turdus viscivorus	45	0.03	0.06	0.06	0.04
1	1	0.90	1	cv	1	Rhamnus catharticus	Sylvia atricapilla	45	0.03	0.06	0.07	0.04
1	1	0.60	1	t	1	Rhamnus catharticus	Sturnus vulgaris	45	0.03	0.06	0.03	0.04
1	1	0.82	1	t	1	Cornus sanguinea	Turdus iliacus	45	0.04	0.06	0.05	0.04
1	1	0.95	1	t	1	Cornus sanguinea	Turdus merula	45	0.04	0.06	0.08	0.05
1	1	0.88	1	cv	1	Cornus sanguinea	Turdus viscivorus	45	0.04	0.06	0.06	0.04
1	1	0.92	1	t	1	Cornus sanguinea	Sylvia atricapilla	45	0.04	0.06	0.07	0.04
1	0	0.41	1	t	1	Cornus sanguinea	Picus viridus	45	0.04	0.06	0.01	0.04

1	1	0.85	1	t	1	Cornus sanguinea	Corvus corone	45	0.04	0.06	0.05	0.04
1	1	0.66	1	cv	1	Cornus sanguinea	Sturnus vulgaris	45	0.04	0.06	0.03	0.04
1	1	0.78	1	t	1	Turdus iliacus	Ligustrum vulgare	45	0.05	0.04	0.03	0.06
1	1	0.57	1	t	1	Turdus iliacus	Viscum album	45	0.05	0.04	0.01	0.06
1	1	0.81	1	t	1	Turdus iliacus	Prunus spinosa	45	0.05	0.04	0.04	0.06
1	1	0.73	1	cv	1	Turdus iliacus	Viburnum opulus	45	0.05	0.04	0.03	0.06
1	1	0.78	1	t	1	Turdus iliacus	Crataegus spp.	45	0.05	0.04	0.03	0.06
1	1	0.73	1	t	1	Turdus iliacus	Ilex aquifolium	45	0.05	0.04	0.03	0.06
1	1	0.80	1	t	1	Turdus iliacus	Hedera helix	45	0.05	0.04	0.04	0.06
1	1	0.78	1	cv	1	Turdus iliacus	Rosa canina	45	0.05	0.04	0.03	0.06
1	1	0.73	1	t	1	Turdus iliacus	Sambucus nigra	45	0.05	0.04	0.03	0.06
1	1	0.73	1	t	1	Turdus iliacus	Sorbus aria	45	0.05	0.04	0.03	0.06
1	1	0.93	1	t	1	Ligustrum vulgare	Turdus merula	45	0.03	0.06	0.08	0.05
1	1	0.85	1	cv	1	Ligustrum vulgare	Turdus viscivorus	45	0.03	0.06	0.06	0.04
1	1	0.90	1	t	1	Ligustrum vulgare	Sylvia atricapilla	45	0.03	0.06	0.07	0.04
1	1	0.89	1	t	1	Turdus merula	Ribes rubrum	45	0.08	0.05	0.02	0.06
1	1	0.92	1	t	1	Turdus merula	Prunus padus	45	0.08	0.05	0.03	0.06
1	1	0.85	1	cv	1	Turdus merula	Sorbus aucuparia	45	0.08	0.05	0.02	0.06
1	1	0.87	1	t	1	Turdus merula	Honeysuckle (perfoliate)	45	0.08	0.05	0.02	0.06
1	1	0.82	1	t	1	Turdus merula	Viscum album	45	0.08	0.05	0.01	0.06
1	1	0.94	1	t	1	Turdus merula	Prunus spinosa	45	0.08	0.05	0.04	0.06
1	1	0.87	1	cv	1	Turdus merula	Euonymus europaeus	45	0.08	0.05	0.02	0.06
1	1	0.90	1	t	1	Turdus merula	Viburnum opulus	45	0.08	0.05	0.03	0.06
1	1	0.82	1	t	1	Turdus merula	Solanum dulcamara	45	0.08	0.05	0.01	0.06
1	1	0.93	1	t	1	Turdus merula	Crataegus spp.	45	0.08	0.05	0.03	0.06
1	1	0.87	1	cv	1	Turdus merula	Lonicera spp.	45	0.08	0.05	0.02	0.06
1	1	0.90	1	t	1	Turdus merula	Ilex aquifolium	45	0.08	0.05	0.03	0.06
1	1	0.93	1	t	1	Turdus merula	Hedera helix	45	0.08	0.05	0.04	0.06
1	1	0.93	1	t	1	Turdus merula	Rosa canina	45	0.08	0.05	0.03	0.06

1	1	0.90	1	cv	1	Turdus merula	Viburnum lantana	45	0.08	0.05	0.03	0.06
1	1	0.91	1	t	1	Turdus merula	Sambucus nigra	45	0.08	0.05	0.03	0.06
1	1	0.89	1	t	1	Turdus merula	Rubus idaeus	45	0.08	0.05	0.02	0.06
1	1	0.90	1	t	1	Turdus merula	Tamus communis	45	0.08	0.05	0.03	0.06
1	1	0.90	1	cv	1	Turdus merula	Sorbus aria	45	0.08	0.05	0.03	0.06
1	1	0.97	1	t	1	Turdus merula	Bryonia dioica	45	0.08	0.05	0.05	0.06
1	1	0.85	1	t	1	Ribes rubrum	Sylvia atricapilla	45	0.02	0.06	0.07	0.04
1	1	0.82	1	t	1	Turdus viscivorus	Prunus padus	45	0.06	0.04	0.03	0.06
1	1	0.71	1	cv	1	Turdus viscivorus	Sorbus aucuparia	45	0.06	0.04	0.02	0.06
1	1	0.66	1	t	1	Turdus viscivorus	Viscum album	45	0.06	0.04	0.01	0.06
1	1	0.86	1	t	1	Turdus viscivorus	Prunus spinosa	45	0.06	0.04	0.04	0.06
1	1	0.80	1	t	1	Turdus viscivorus	Viburnum opulus	45	0.06	0.04	0.03	0.06
1	1	0.84	1	cv	1	Turdus viscivorus	Crataegus spp.	45	0.06	0.04	0.03	0.06
1	1	0.80	1	t	1	Turdus viscivorus	Ilex aquifolium	45	0.06	0.04	0.03	0.06
1	1	0.86	1	t	1	Turdus viscivorus	Hedera helix	45	0.06	0.04	0.04	0.06
1	1	0.84	1	t	1	Turdus viscivorus	Rosa canina	45	0.06	0.04	0.03	0.06
1	1	0.80	1	cv	1	Turdus viscivorus	Sambucus nigra	45	0.06	0.04	0.03	0.06
1	1	0.80	1	t	1	Turdus viscivorus	Sorbus aria	45	0.06	0.04	0.03	0.06
1	1	0.88	1	t	1	Prunus padus	Sylvia atricapilla	45	0.03	0.06	0.07	0.04
0	0	0.39	1	t	1	Sylvia communis	Sambucus nigra	45	0.02	0.04	0.03	0.06
1	1	0.66	1	cv	1	Sylvia communis	Bryonia dioica	45	0.02	0.04	0.05	0.06
1	1	0.80	1	t	1	Sorbus aucuparia	Sylvia atricapilla	45	0.02	0.06	0.07	0.04
1	0	0.38	1	t	1	Sorbus aucuparia	Sylvia curruca	45	0.02	0.06	0.03	0.04
1	0	0.42	1	t	1	Sorbus aucuparia	Sturnus vulgaris	45	0.02	0.06	0.03	0.04
1	1	0.81	1	cv	1	Sylvia atricapilla	Honeysuckle (perfoliate)	45	0.07	0.04	0.02	0.06
1	1	0.75	1	t	1	Sylvia atricapilla	Viscum album	45	0.07	0.04	0.01	0.06

1	1	0.81	1	t	1	Sylvia atricapilla	Euonymus europaeus	45	0.07	0.04	0.02	0.06
1	1	0.86	1	t	1	Sylvia atricapilla	Viburnum opulus	45	0.07	0.04	0.03	0.06
1	1	0.75	1	cv	1	Sylvia atricapilla	Solanum dulcamara	45	0.07	0.04	0.01	0.06
1	1	0.86	1	t	1	Sylvia atricapilla	Ilex aquifolium	45	0.07	0.04	0.03	0.06
1	1	0.90	1	t	1	Sylvia	Hedera helix	45	0.07	0.04	0.04	0.06
1	1	0.89	1	t	1	Sylvia	Rosa canina	45	0.07	0.04	0.03	0.06
1	1	0.86	1	cv	1	Sylvia atricapilla	Viburnum lantana	45	0.07	0.04	0.03	0.06
1	1	0.86	1	t	1	Sylvia atricapilla	Sambucus nigra	45	0.07	0.04	0.03	0.06
1	1	0.84	1	t	1	Sylvia	Rubus idaeus	45	0.07	0.04	0.02	0.06
1	1	0.86	1	t	1	Sylvia atricapilla	Tamus communis	45	0.07	0.04	0.03	0.06
1	1	0.86	1	cv	1	Sylvia	Sorbus aria	45	0.07	0.04	0.03	0.06
1	1	0.95	1	t	1	Sylvia atricapilla	Bryonia dioica	45	0.07	0.04	0.05	0.06
1	0	0.42	1	t	1	Honeysuckle (perfoliate)	Sylvia curruca	45	0.02	0.06	0.03	0.04
1	0	0.46	1	t	1	Honeysuckle (perfoliate)	Sturnus vulgaris	45	0.02	0.06	0.03	0.04
1	1	0.84	1	cv	1	Prunus spinosa	Corvus corone	45	0.04	0.06	0.05	0.04
1	1	0.64	1	t	1	Prunus spinosa	Sturnus vulgaris	45	0.04	0.06	0.03	0.04
1	1	0.50	1	t	1	Columba palumbus	Crataegus spp.	45	0.02	0.04	0.03	0.06
1	0	0.43	1	t	1	Columba palumbus	Ilex aquifolium	45	0.02	0.04	0.03	0.06
1	1	0.50	1	cv	1	Columba palumbus	Rosa canina	45	0.02	0.04	0.03	0.06
1	0	0.21	1	t	1	Solanum dulcamara	Musciapa striata	45	0.01	0.06	0.01	0.04
1	0	0.37	1	t	1	Solanum dulcamara	Sturnus vulgaris	45	0.01	0.06	0.03	0.04
1	1	0.63	1	t	1	Gallinula chloropus	Sambucus nigra	45	0.04	0.04	0.03	0.06
1	1	0.60	1	cv	1	Crataegus spp.	Sturnus vulgaris	45	0.03	0.06	0.03	0.04
1	0	0.46	1	t	1	Lonicera spp.	Sturnus vulgaris	45	0.02	0.06	0.03	0.04
1	0	0.50	1	t	1	Sylvia curruca	Viburnum lantana	45	0.03	0.04	0.03	0.06
1	1	0.50	1	t	1	Sylvia curruca	Sambucus nigra	45	0.03	0.04	0.03	0.06
1	1	0.76	1	cv	1	Sylvia curruca	Bryonia dioica	45	0.03	0.04	0.05	0.06

0	0	0.36	1	t	1	Musciapa striata	Sambucus nigra	45	0.01	0.04	0.03	0.06
1	1	0.63	1	t	1	Hedera helix	Sturnus vulgaris	45	0.04	0.06	0.03	0.04
1	1	0.77	1	t	1	Corvus corone	Sambucus nigra	45	0.05	0.04	0.03	0.06
1	1	0.77	1	cv	1	Corvus corone	Sorbus aria	45	0.05	0.04	0.03	0.06
1	1	0.54	1	t	1	Sambucus nigra	Sturnus vulgaris	45	0.03	0.06	0.03	0.04
1	1	0.54	1	t	1	Sorbus aria	Sturnus vulgaris	45	0.03	0.06	0.03	0.04
1	1	0.55	0	t	1	Prunus spinosa	Sambucus nigra	45	0.04	0.06	0.03	0.06
1	1	0.60	0	cv	1	Magpie	Tamus communis	45	0.04	0.04	0.03	0.06
0	0	0.43	0	t	1	Euonymus europaeus	Rosa canina	45	0.02	0.06	0.03	0.06
1	0	0.35	0	t	1	Taxus baccata	Picus viridus	45	0.03	0.06	0.01	0.04
1	1	0.61	0	t	1	Prunus spinosa	Crataegus spp.	45	0.04	0.06	0.03	0.06
0	0	0.50	0	cv	1	Rubus fruticosus	Rubus idaeus	45	0.04	0.06	0.02	0.06
0	0	0.37	0	t	1	Honeysuckle (perfoliate)	Sorbus aria	45	0.02	0.06	0.03	0.06
0	0	0.36	0	t	1	Honeysuckle (perfoliate)	Ilex aquifolium	45	0.02	0.06	0.03	0.06
0	1	0.63	0	t	1	Jay	Gallinula chloropus	45	0.02	0.04	0.04	0.04
1	0	0.47	0	cv	1	Viscum album	Gallinula chloropus	45	0.01	0.06	0.04	0.04
1	0	0.32	0	t	1	Prunus padus	Picus viridus	45	0.03	0.06	0.01	0.04
0	0	0.32	0	t	1	Lonicera spp.	Rubus idaeus	45	0.02	0.06	0.02	0.06
1	1	0.54	0	t	1	Rhamnus catharticus	Prunus padus	45	0.03	0.06	0.03	0.06
1	1	0.81	0	cv	1	Cornus sanguinea	Bryonia dioica	45	0.04	0.06	0.05	0.06
0	1	0.80	0	t	1	Sylvia atricapilla	Musciapa striata	45	0.07	0.04	0.01	0.04
0	0	0.29	0	t	1	Sylvia communis	Musciapa striata	45	0.02	0.04	0.01	0.04
1	0	0.46	0	t	1	Cornus sanguinea	Musciapa striata	45	0.04	0.06	0.01	0.04
1	0	0.45	0	cv	1	Turdus torquatus	Prunus spinosa	45	0.01	0.04	0.04	0.06
0	0	0.36	0	t	1	Lonicera spp.	Viburnum lantana	45	0.02	0.06	0.03	0.06
1	1	0.58	0	t	1	Ligustrum vulgare	Crataegus spp.	45	0.03	0.06	0.03	0.06
0	1	0.60	0	t	1	Sorbus aucuparia	Bryonia dioica	45	0.02	0.06	0.05	0.06

0	0	0.39	0	cv	1	Columba palumbus	Rubus idaeus	45	0.02	0.04	0.02	0.06
0	1	0.93	0	t	1	Turdus merula	Sturnus vulgaris	45	0.08	0.05	0.03	0.04
0	1	0.54	0	t	1	Hedera helix	Tamus communis	45	0.04	0.06	0.03	0.06
0	0	0.39	0	t	1	Malus sylvestris	Sorbus aucuparia	45	0.03	0.06	0.02	0.06
0	0	0.40	0	cv	1	Viburnum opulus	Rubus idaeus	45	0.03	0.06	0.02	0.06
1	1	0.61	0	t	1	Taxus baccata	Prunus spinosa	45	0.03	0.06	0.04	0.06
0	1	0.85	0	t	1	Arum maculatum	Rubus idaeus	45	0.08	0.06	0.02	0.06
1	1	0.59	0	t	1	Gallinula chloropus	Rubus idaeus	45	0.04	0.04	0.02	0.06
1	0	0.45	0	cv	1	Musciapa striata	Hedera helix	45	0.01	0.04	0.04	0.06
1	0	0.45	0	t	1	Ribes rubrum	Sylvia curruca	45	0.02	0.06	0.03	0.04
1	1	0.66	0	t	1	Turdus viscivorus	Solanum dulcamara	45	0.06	0.04	0.01	0.06
0	0	0.43	0	t	1	Lonicera spp.	Rosa canina	45	0.02	0.06	0.03	0.06
1	1	0.74	0	cv	1	Prunus padus	Bryonia dioica	45	0.03	0.06	0.05	0.06
0	0	0.35	0	t	1	Solanum dulcamara	Crataegus spp.	45	0.01	0.06	0.03	0.06
0	0	0.35	0	t	1	Turdus torquatus	Viburnum lantana	45	0.01	0.04	0.03	0.06
0	0	0.33	0	t	1	Jay	Turdus torquatus	45	0.02	0.04	0.01	0.04
0	1	0.77	0	cv	1	Turdus pilaris	Turdus philomelos	45	0.03	0.04	0.04	0.04
1	1	0.51	0	t	1	Prunus avium	Sylvia communis	45	0.04	0.06	0.02	0.04
0	1	0.69	0	t	1	Magpie	Sturnus vulgaris	45	0.04	0.04	0.03	0.04
1	1	0.61	0	t	1	Malus sylvestris	Prunus spinosa	45	0.03	0.06	0.04	0.06
0	0	0.42	0	cv	1	Taxus baccata	Honeysuckle (perfoliate)	45	0.03	0.06	0.02	0.06
0	1	0.60	0	t	1	Juniperus communis	Sambucus nigra	45	0.04	0.06	0.03	0.06
1	1	0.90	0	t	1	Sylvia atricapilla	Prunus spinosa	45	0.07	0.04	0.04	0.06
0	0	0.40	0	t	1	Ribes rubrum	Sorbus aria	45	0.02	0.06	0.03	0.06
1	0	0.45	0	cv	1	Sambucus	Sorbus aria	45	0.03	0.06	0.03	0.06
0	0	0.35	0	t	1	Prunus padus	Sorbus aucuparia	45	0.03	0.06	0.02	0.06
0	1	0.75	0	t	1	Turdus viscivorus	Sylvia communis	45	0.06	0.04	0.02	0.04

1	1	0.54	0	t	1	Rubus fruticosus	Sorbus aria	45	0.04	0.06	0.03	0.06
1	1	0.60	0	cv	1	Rosa canina	Sturnus vulgaris	45	0.03	0.06	0.03	0.04
0	1	0.90	0	t	1	Turdus merula	Columba palumbus	45	0.08	0.05	0.02	0.04
1	1	0.58	0	t	1	Prunus avium	Sambucus nigra	45	0.04	0.06	0.03	0.06
0	1	0.83	0	t	1	Turdus viscivorus	Sylvia curruca	45	0.06	0.04	0.03	0.04
0	1	0.73	0	cv	1	Prunus avium	Juniperus communis	45	0.04	0.06	0.04	0.06
0	1	0.72	0	t	1	Magpie	Turdus pilaris	45	0.04	0.04	0.03	0.04
1	1	0.53	0	t	1	Tamus communis	Sturnus vulgaris	45	0.03	0.06	0.03	0.04
1	1	0.61	0	t	1	Rubus fruticosus	Crataegus spp.	45	0.04	0.06	0.03	0.06
1	1	0.51	0	cv	1	Viburnum opulus	Crataegus spp.	45	0.03	0.06	0.03	0.06
1	1	0.67	0	t	1	Magpie	Crataegus spp.	45	0.04	0.04	0.03	0.06
0	0	0.48	0	t	1	Turdus torquatus	Turdus pilaris	45	0.01	0.04	0.03	0.04
1	1	0.52	0	t	1	Prunus spinosa	Columba palumbus	45	0.04	0.06	0.02	0.04
0	1	0.91	0	cv	1	Arum maculatum	Sturnus vulgaris	45	0.08	0.06	0.03	0.04
1	1	0.78	0	t	1	Arum maculatum	Picus viridus	45	0.08	0.06	0.01	0.04
0	1	0.90	0	t	1	Arum maculatum	Ligustrum vulgare	45	0.08	0.06	0.03	0.06
1	0	0.40	0	t	1	Malus sylvestris	Musciapa striata	45	0.03	0.06	0.01	0.04
0	1	0.55	0	cv	1	Juniperus communis	Ribes rubrum	45	0.04	0.06	0.02	0.06
0	0	0.29	0	t	1	Solanum dulcamara	Ilex aquifolium	45	0.01	0.06	0.03	0.06
1	1	0.74	0	t	1	Prunus avium	Gallinula chloropus	45	0.04	0.06	0.04	0.04
0	1	0.74	0	t	1	Turdus philomelos	Sturnus vulgaris	45	0.04	0.04	0.03	0.04
1	1	0.58	0	cv	1	Malus sylvestris	Crataegus spp.	45	0.03	0.06	0.03	0.06
0	0	0.47	0	t	1	Prunus padus	Ilex aquifolium	45	0.03	0.06	0.03	0.06
0	1	0.71	0	t	1	Gallinula chloropus	Sturnus vulgaris	45	0.04	0.04	0.03	0.04
1	1	0.58	0	t	1	Crataegus spp.	Rosa canina	45	0.03	0.06	0.03	0.06
1	1	0.63	0	cv	1	Turdus torquatus	Bryonia dioica	45	0.01	0.04	0.05	0.06

0	0	0.39	0	t	1	Prunus padus	Honeysuckle (perfoliate)	45	0.03	0.06	0.02	0.06
1	1	0.79	0	t	1	Juniperus communis	Turdus philomelos	45	0.04	0.06	0.04	0.04
0	0	0.40	0	t	1	Ribes rubrum	Viburnum lantana	45	0.02	0.06	0.03	0.06
0	0	0.37	0	cv	1	Lonicera spp.	Sambucus nigra	45	0.02	0.06	0.03	0.06
0	0	0.34	0	t	1	Malus sylvestris	Solanum dulcamara	45	0.03	0.06	0.01	0.06
0	0	0.40	0	t	1	Turdus torquatus	Sylvia curruca	45	0.01	0.04	0.03	0.04
0	1	0.87	0	t	1	Arum maculatum	Viburnum opulus	45	0.08	0.06	0.03	0.06
0	0	0.29	0	cv	1	Viscum album	Viburnum lantana	45	0.01	0.06	0.03	0.06
0	0	0.34	0	t	1	Ligustrum vulgare	Viscum album	45	0.03	0.06	0.01	0.06
1	1	0.68	0	t	1	Prunus avium	Prunus spinosa	45	0.04	0.06	0.04	0.06
1	1	0.66	0	t	1	Prunus padus	Gallinula chloropus	45	0.03	0.06	0.04	0.04
1	1	0.52	0	cv	1	Magpie	Lonicera spp.	45	0.04	0.04	0.02	0.06
1	1	0.54	0	t	1	Prunus	Sorbus aria	45	0.04	0.06	0.03	0.06
0	0	0.33	0	t	1	Sorbus aucuparia	Sambucus nigra	45	0.02	0.06	0.03	0.06
1	0	0.26	0	t	1	Ribes rubrum	Picus viridus	45	0.02	0.06	0.01	0.04
0	0	0.40	0	cv	1	Prunus avium	Viscum album	45	0.04	0.06	0.01	0.06
0	1	0.88	0	t	1	Turdus pilaris	Turdus viscivorus	45	0.03	0.04	0.06	0.04
0	0	0.40	0	t	1	Ribes rubrum	Ilex aquifolium	45	0.02	0.06	0.03	0.06
0	0	0.34	0	t	1	Rhamnus catharticus	Viscum album	45	0.03	0.06	0.01	0.06
1	0	0.40	0	cv	1	Malus sylvestris	Turdus torquatus	45	0.03	0.06	0.01	0.04
1	1	0.64	0	t	1	Rubus fruticosus	Hedera helix	45	0.04	0.06	0.04	0.06
1	1	0.60	0	t	1	Ligustrum vulgare	Sturnus vulgaris	45	0.03	0.06	0.03	0.04
1	0	0.38	0	t	1	Turdus torquatus	Prunus padus	45	0.01	0.04	0.03	0.06
0	1	0.71	0	cv	1	Turdus iliacus	Columba palumbus	45	0.05	0.04	0.02	0.04
0	1	0.87	0	t	1	Arum maculatum	Columba palumbus	45	0.08	0.06	0.02	0.04
1	1	0.52	0	t	1	Rubus fruticosus	Columba palumbus	45	0.04	0.06	0.02	0.04
1	1	0.57	0	t	1	Sylvia borin	Ilex aquifolium	45	0.03	0.04	0.03	0.06

1	0	0.27	0	cv	1	Lonicera spp.	Musciapa striata	45	0.02	0.06	0.01	0.04
0	1	0.85	0	t	1	Erithacus rubecula	Sylvia communis	45	0.07	0.05	0.02	0.04
0	1	0.96	0	t	1	Turdus philomelos	Turdus merula	45	0.04	0.04	0.08	0.05
1	1	0.61	0	t	1	Ligustrum vulgare	Prunus spinosa	45	0.03	0.06	0.04	0.06
0	0	0.36	0	cv	1	Euonymus europaeus	Tamus communis	45	0.02	0.06	0.03	0.06
1	1	0.71	0	t	1	Honeysuckle (perfoliate)	Corvus corone	45	0.02	0.06	0.05	0.04
1	0	0.43	0	t	1	Columba palumbus	Viburnum opulus	45	0.02	0.04	0.03	0.06
1	0	0.40	0	t	1	Taxus baccata	Musciapa striata	45	0.03	0.06	0.01	0.04
0	0	0.42	0	cv	1	Crataegus spp.	Lonicera spp.	45	0.03	0.06	0.02	0.06
0	0	0.46	0	t	1	Prunus spinosa	Euonymus europaeus	45	0.04	0.06	0.02	0.06
0	1	0.87	0	t	1	Arum maculatum	Ilex aquifolium	45	0.08	0.06	0.03	0.06
0	1	0.54	0	t	1	Prunus spinosa	Viburnum lantana	45	0.04	0.06	0.03	0.06
1	1	0.57	0	cv	1	Sylvia borin	Sorbus aria	45	0.03	0.04	0.03	0.06
1	1	0.56	0	t	1	Malus sylvestris	Sylvia curruca	45	0.03	0.06	0.03	0.04
0	0	0.41	0	t	1	Turdus pilaris	Picus viridus	45	0.03	0.04	0.01	0.04
0	0	0.44	0	t	1	Turdus torquatus	Sturnus vulgaris	45	0.01	0.04	0.03	0.04
1	1	0.67	0	cv	1	Magpie	Rhamnus catharticus	45	0.04	0.04	0.03	0.06
1	1	0.84	0	t	1	Gallinula chloropus	Bryonia dioica	45	0.04	0.04	0.05	0.06
0	1	0.68	0	t	1	Gallinula chloropus	Sylvia curruca	45	0.04	0.04	0.03	0.04
0	1	0.77	0	t	1	Turdus iliacus	Sylvia curruca	45	0.05	0.04	0.03	0.04
0	1	0.95	0	cv	1	Turdus pilaris	Turdus merula	45	0.03	0.04	0.08	0.05
1	0	0.41	0	t	1	Lonicera spp.	Sylvia curruca	45	0.02	0.06	0.03	0.04
0	1	0.84	0	t	1	Sylvia communis	Sylvia atricapilla	45	0.02	0.04	0.07	0.04
1	0	0.44	0	t	1	Ilex aquifolium	Sambucus nigra	45	0.03	0.06	0.03	0.06
1	1	0.51	0	cv	1	Jay	Ligustrum vulgare	45	0.02	0.04	0.03	0.06
1	1	0.82	0	t	1	Crataegus spp.	Corvus corone	45	0.03	0.06	0.05	0.04
0	1	0.73	0	t	1	Jay	Turdus iliacus	45	0.02	0.04	0.05	0.04
0	1	0.82	0	t	1	Sylvia borin	Turdus iliacus	45	0.03	0.04	0.05	0.04

1	0	0.43	0	cv	1	Juniperus communis	Picus viridus	45	0.04	0.06	0.01	0.04
1	1	0.58	0	t	1	Rhamnus catharticus	Rosa canina	45	0.03	0.06	0.03	0.06
1	1	0.51	0	t	1	Malus	Sorbus aria	45	0.03	0.06	0.03	0.06
0	1	0.53	0	t	1	Jay	Sturnus vulgaris	45	0.02	0.04	0.03	0.04
1	1	0.68	0	cv	1	Ribes rubrum	Bryonia dioica	45	0.02	0.06	0.05	0.06
1	1	0.61	0	t	1	Rubus fruticosus	Ligustrum vulgare	45	0.04	0.06	0.03	0.06
1	0	0.37	0	t	1	Prunus padus	Musciapa striata	45	0.03	0.06	0.01	0.04
0	1	0.94	0	t	1	Magpie	Erithacus rubecula	45	0.04	0.04	0.07	0.05
0	1	0.87	0	cv	1	Arum maculatum	Viburnum lantana	45	0.08	0.06	0.03	0.06
0	0	0.47	0	t	1	Turdus pilaris	Musciapa striata	45	0.03	0.04	0.01	0.04
1	1	0.65	0	t	1	Juniperus communis	Sylvia curruca	45	0.04	0.06	0.03	0.04
1	1	0.64	0	t	1	Sylvia borin	Rhamnus catharticus	45	0.03	0.04	0.03	0.06
1	1	0.72	0	cv	1	Gallinula chloropus	Hedera helix	45	0.04	0.04	0.04	0.06
1	0	0.35	0	t	1	Ligustrum vulgare	Picus viridus	45	0.03	0.06	0.01	0.04
0	0	0.42	0	t	1	Rhamnus catharticus	Euonymus europaeus	45	0.03	0.06	0.02	0.06
0	0	0.44	0	t	1	Viburnum opulus	Viburnum lantana	45	0.03	0.06	0.03	0.06
0	0	0.36	0	cv	1	Jay	Honeysuckle (perfoliate)	45	0.02	0.04	0.02	0.06
0	0	0.36	0	t	1	Lonicera spp.	Ilex aquifolium	45	0.02	0.06	0.03	0.06
1	1	0.71	0	t	1	Tamus communis	Bryonia dioica	45	0.03	0.06	0.05	0.06
1	1	0.67	0	t	1	Rubus fruticosus	Turdus pilaris	45	0.04	0.06	0.03	0.04
0	0	0.20	0	cv	1	Sorbus aucuparia	Solanum dulcamara	45	0.02	0.06	0.01	0.06
0	1	0.87	0	t	1	Jay	Sylvia atricapilla	45	0.02	0.04	0.07	0.04
0	0	0.39	0	t	1	Sylvia communis	Sorbus aria	45	0.02	0.04	0.03	0.06
0	0	0.47	0	t	1	Honeysuckle (perfoliate)	Hedera helix	45	0.02	0.06	0.04	0.06
0	0	0.38	0	cv	1	Ligustrum vulgare	Sorbus aucuparia	45	0.03	0.06	0.02	0.06
0	1	0.93	0	t	1	Turdus pilaris	Arum maculatum	45	0.03	0.04	0.08	0.06

1	1	0.54	0	t	1	Columba palumbus	Hedera helix	45	0.02	0.04	0.04	0.06
0	1	0.51	0	t	1	Juniperus communis	Lonicera spp.	45	0.04	0.06	0.02	0.06
1	1	0.51	0	cv	1	Turdus torquatus	Juniperus communis	45	0.01	0.04	0.04	0.06
1	0	0.38	0	t	1	Rubus fruticosus	Picus viridus	45	0.04	0.06	0.01	0.04
0	0	0.28	0	t	1	Jay	Solanum dulcamara	45	0.02	0.04	0.01	0.06
1	1	0.66	0	t	1	Turdus iliacus	Lonicera spp.	45	0.05	0.04	0.02	0.06
1	1	0.82	0	cv	1	Juniperus communis	Bryonia dioica	45	0.04	0.06	0.05	0.06
0	0	0.21	0	t	1	Turdus torquatus	Viscum album	45	0.01	0.04	0.01	0.06
1	1	0.81	0	t	1	Sylvia	Lonicera spp.	45	0.07	0.04	0.02	0.06
1	0	0.40	0	t	1	Crataegus spp.	Musciapa striata	45	0.03	0.06	0.01	0.04
0	0	0.27	0	cv	1	Columba palumbus	Solanum dulcamara	45	0.02	0.04	0.01	0.06
1	0	0.43	0	t	1	Prunus spinosa	Musciapa striata	45	0.04	0.06	0.01	0.04
0	0	0.49	0	t	1	Sylvia communis	Prunus spinosa	45	0.02	0.04	0.04	0.06
0	1	0.96	0	t	1	Sylvia atricapilla	Corvus corone	45	0.07	0.04	0.05	0.04
1	0	0.49	0	cv	1	Malus	Jay	45	0.03	0.06	0.02	0.04
0	1	0.89	0	t	1	Turdus philomelos	Corvus corone	45	0.04	0.04	0.05	0.04
1	0	0.23	0	t	1	Euonymus europaeus	Picus viridus	45	0.02	0.06	0.01	0.04
0	1	0.89	0	t	1	Turdus viscivorus	Gallinula chloropus	45	0.06	0.04	0.04	0.04
0	0	0.40	0	cv	1	Prunus avium	Solanum dulcamara	45	0.04	0.06	0.01	0.06
0	1	0.92	0	t	1	Turdus pilaris	Sylvia atricapilla	45	0.03	0.04	0.07	0.04
0	0	0.25	0	t	1	Sylvia communis	Picus viridus	45	0.02	0.04	0.01	0.04
0	1	0.93	0	t	1	Prunus avium	Arum maculatum	45	0.04	0.06	0.08	0.06
0	0	0.47	0	cv	1	Malus	Ribes rubrum	45	0.03	0.06	0.02	0.06
1	0	0.41	0	t	1	Prunus padus	Sylvia communis	45	0.03	0.06	0.02	0.04
1	1	0.86	0	t	1	Rubus fruticosus	Turdus viscivorus	45	0.04	0.06	0.06	0.04
1	1	0.51	0	t	1	Rosa canina	Sorbus aria	45	0.03	0.06	0.03	0.06
0	0	0.31	0	cv	1	Prunus padus	Viscum album	45	0.03	0.06	0.01	0.06
1	1	0.72	0	t	1	Juniperus communis	Cornus sanguinea	45	0.04	0.06	0.04	0.06

0	1	0.73	0	t	1	Sylvia communis	Corvus corone	45	0.02	0.04	0.05	0.04
1	1	0.81	0	t	1	Turdus pilaris	Bryonia dioica	45	0.03	0.04	0.05	0.06
0	1	0.92	0	cv	1	Rubus fruticosus	Arum maculatum	45	0.04	0.06	0.08	0.06
0	0	0.39	0	t	1	Sylvia communis	Viburnum lantana	45	0.02	0.04	0.03	0.06
1	1	0.51	0	t	1	Ligustrum vulgare	Sorbus aria	45	0.03	0.06	0.03	0.06
0	1	0.72	0	t	1	Turdus viscivorus	Musciapa striata	45	0.06	0.04	0.01	0.04
0	0	0.46	0	cv	1	Prunus	Lonicera spp.	45	0.04	0.06	0.02	0.06
0	1	0.50	0	t	1	Ligustrum vulgare	Viburnum lantana	45	0.03	0.06	0.03	0.06
0	0	0.28	0	t	1	Turdus torquatus	Euonymus europaeus	45	0.01	0.04	0.02	0.06
1	0	0.49	0	t	1	Viburnum opulus	Sylvia curruca	45	0.03	0.06	0.03	0.04
1	0	0.29	0	cv	1	Viburnum opulus	Picus viridus	45	0.03	0.06	0.01	0.04
1	0	0.49	0	t	1	Rhamnus catharticus	Columba palumbus	45	0.03	0.06	0.02	0.04
0	0	0.29	0	t	1	Euonymus europaeus	Lonicera spp.	45	0.02	0.06	0.02	0.06
1	1	0.82	0	t	1	Rosa canina	Corvus corone	45	0.03	0.06	0.05	0.04
0	0	0.43	0	cv	1	Viburnum lantana	Tamus communis	45	0.03	0.06	0.03	0.06
1	0	0.35	0	t	1	Crataegus spp.	Picus viridus	45	0.03	0.06	0.01	0.04
1	0	0.34	0	t	1	Ilex aquifolium	Musciapa striata	45	0.03	0.06	0.01	0.04
0	0	0.29	0	t	1	Jay	Picus viridus	45	0.02	0.04	0.01	0.04
1	1	0.58	0	cv	1	Malus sylvestris	Rhamnus catharticus	45	0.03	0.06	0.03	0.06
1	0	0.33	0	t	1	Solanum dulcamara	Sylvia curruca	45	0.01	0.06	0.03	0.04
1	1	0.61	0	t	1	Rhamnus catharticus	Hedera helix	45	0.03	0.06	0.04	0.06
1	1	0.58	0	t	1	Rhamnus catharticus	Crataegus spp.	45	0.03	0.06	0.03	0.06
0	0	0.44	0	cv	1	Ilex aquifolium	Tamus communis	45	0.03	0.06	0.03	0.06
0	1	0.67	0	t	1	Turdus viscivorus	Picus viridus	45	0.06	0.04	0.01	0.04
1	0	0.17	0	t	1	Solanum dulcamara	Picus viridus	45	0.01	0.06	0.01	0.04
0	1	0.77	0	t	1	Magpie	Gallinula chloropus	45	0.04	0.04	0.04	0.04
0	0	0.46	0	cv	1	Lonicera spp.	Hedera helix	45	0.02	0.06	0.04	0.06

0	1	0.50	0	t	1	Crataegus spp.	Tamus communis	45	0.03	0.06	0.03	0.06
0	1	0.88	1	t	1	Cyanistes caeruleus	Rubus fruetiosa	46	0.06	0.09	0.06	0.08
0	1	0.82	1	t	1	Cyanistes caeruleus	Lonicera perielymenum	46	0.06	0.09	0.05	0.08
1	1	0.99	1	cv	1	Cyanistes caeruleus	Sambucus niger	46	0.06	0.09	0.12	0.08
1	1	0.88	1	t	1	Cyanistes caeruleus	Bryonia dioecia	46	0.06	0.09	0.06	0.08
1	1	0.88	1	t	1	Turdus philomelos	Rubus fruetiosa	46	0.06	0.08	0.06	0.08
1	1	0.94	1	t	1	Turdus philomelos	Crategus monogyna	46	0.06	0.08	0.08	0.08
1	1	0.99	1	cv	1	Turdus philomelos	Sambucus niger	46	0.06	0.08	0.12	0.08
1	1	0.92	1	t	1	Turdus philomelos	Hedera helix	46	0.06	0.08	0.07	0.08
0	1	0.87	1	t	1	Turdus pilaris	Crategus monogyna	46	0.04	0.08	0.08	0.08
0	1	0.66	1	t	1	Turdus pilaris	Prunus spinosa	46	0.04	0.08	0.05	0.08
0	1	0.96	1	cv	1	Rubus fruetiosa	Turdus merula	46	0.06	0.08	0.09	0.08
0	1	0.96	1	t	1	Rubus fruetiosa	Poecile palustris/Poecile montanus	46	0.06	0.08	0.09	0.08
1	1	0.97	1	t	1	Erithacus rubecula	Sambucus niger	46	0.04	0.09	0.12	0.08
1	1	0.81	1	t	1	Erithacus rubecula	Hedera helix	46	0.04	0.09	0.07	0.08
1	1	0.91	1	cv	1	Turdus iliacus	Crategus monogyna	46	0.05	0.08	0.08	0.08
0	1	0.75	1	t	1	Turdus iliacus	Prunus spinosa	46	0.05	0.08	0.05	0.08
1	1	0.98	1	t	1	Turdus iliacus	Sambucus niger	46	0.05	0.08	0.12	0.08
0	1	0.98	1	t	1	Crategus monogyna	Turdus merula	46	0.08	0.08	0.09	0.08
0	1	0.96	1	cv	1	Crategus monogyna	Pyrrhula pyrrula	46	0.08	0.08	0.07	0.08
0	1	0.85	1	t	1	Crategus monogyna	Colurnba palumbus	46	0.08	0.08	0.04	0.09
1	1	0.94	1	t	1	Turdus merula	Prunus spinosa	46	0.09	0.08	0.05	0.08
1	1	0.93	1	t	1	Turdus merula	Euonymus europaeus	46	0.09	0.08	0.05	0.08
1	1	1.00	1	cv	1	Turdus merula	Sambucus niger	46	0.09	0.08	0.12	0.08

1	1	0.89	1	t	1	Turdus merula	Rosa canina	46	0.09	0.08	0.04	0.08
1	1	0.97	1	t	1	Turdus merula	Hedera helix	46	0.09	0.08	0.07	0.08
1	1	0.97	1	t	1	Parus major	Sambucus niger	46	0.04	0.09	0.12	0.08
0	1	0.73	1	cv	1	Parus major	Bryonia dioecia	46	0.04	0.09	0.06	0.08
0	1	0.94	1	t	1	Lonicera perielymenum	Poecile palustris/Poecile montanus	46	0.05	0.08	0.09	0.08
0	1	0.88	1	t	1	Lonicera perielymenum	Pyrrhula pyrrula	46	0.05	0.08	0.07	0.08
0	1	0.94	1	t	1	Euonymus europaeus	Poecile palustris/Poecile montanus	46	0.05	0.08	0.09	0.08
0	0	0.49	1	cv	1	Euonymus europaeus	Aegithalos caudatus	46	0.05	0.08	0.02	0.08
1	1	1.00	1	t	1	Poecile palustris/Poecile montanus	Sambucus niger	46	0.09	0.08	0.12	0.08
1	1	0.96	1	t	1	Poecile palustris/Poecile montanus	Bryonia dioecia	46	0.09	0.08	0.06	0.08
0	1	0.80	1	t	1	Poecile palustris/Poecile montanus	Tamus communis	46	0.09	0.08	0.02	0.08
1	1	0.93	1	cv	1	Poecile palustris/Poecile montanus	Woody nightshade	46	0.09	0.08	0.05	0.08
1	1	0.97	1	t	1	Fringilla coelebs	Sambucus niger	46	0.04	0.09	0.12	0.08
0	1	0.73	1	t	1	Fringilla coelebs	Bryonia dioecia	46	0.04	0.09	0.06	0.08
0	1	0.96	1	t	1	Sambucus niger	Sitta europaea	46	0.12	0.08	0.04	0.09
1	1	0.99	1	cv	1	Sambucus niger	Pyrrhula pyrrula	46	0.12	0.08	0.07	0.08
0	1	0.97	1	t	1	Sambucus niger	Colurnba palumbus	46	0.12	0.08	0.04	0.09
1	1	0.98	1	t	1	Sambucus niger	Sylvia atricapilla	46	0.12	0.08	0.05	0.09
0	1	0.62	1	t	1	Sitta europaea	Woody nightshade	46	0.04	0.09	0.05	0.08
0	1	0.81	1	cv	1	Rosa canina	Pyrrhula pyrrula	46	0.04	0.08	0.07	0.08
1	1	0.94	1	t	1	Pyrrhula	Hedera helix	46	0.07	0.08	0.07	0.08
0	1	0.87	1	t	1	Hedera helix	Sylvia atricapilla	46	0.07	0.08	0.05	0.09
0	1	0.74	1	t	1	Woody nightshade	Sylvia atricapilla	46	0.05	0.08	0.05	0.09

0	0	0.50	0	cv	1	Rosa canina	Colurnba palumbus	46	0.04	0.08	0.04	0.09
0	1	0.85	0	t	1	Crategus monogyna	Fringilla coelebs	46	0.08	0.08	0.04	0.09
0	1	0.82	0	t	1	Rubus fruetiosa	Sylvia atricapilla	46	0.06	0.08	0.05	0.09
0	1	0.62	0	t	1	Turdus pilaris	Sylvia atricapilla	46	0.04	0.08	0.05	0.09
0	1	0.82	0	cv	1	Turdus pilaris	Hedera helix	46	0.04	0.08	0.07	0.08
0	1	0.84	0	t	1	Rubus fruetiosa	Prunus spinosa	46	0.06	0.08	0.05	0.08
0	1	0.72	0	t	1	Cyanistes caeruleus	Turdus pilaris	46	0.06	0.09	0.04	0.08
0	1	0.88	0	t	1	Hedera helix	Woody nightshade	46	0.07	0.08	0.05	0.08
0	1	0.69	0	cv	1	Hedera helix	Tamus communis	46	0.07	0.08	0.02	0.08
0	1	0.80	0	t	1	Cyanistes caeruleus	Sylvia atricapilla	46	0.06	0.09	0.05	0.09
0	0	0.49	0	t	1	Turdus pilaris	Fringilla coelebs	46	0.04	0.08	0.04	0.09
1	1	0.97	0	t	1	Turdus merula	Pyrrhula pyrrula	46	0.09	0.08	0.07	0.08
0	0	0.46	0	cv	1	Erithacus rubecula	Sitta europaea	46	0.04	0.09	0.04	0.09
0	1	0.70	0	t	1	Cyanistes caeruleus	Fringilla coelebs	46	0.06	0.09	0.04	0.09
0	0	0.37	0	t	1	Rosa canina	Tamus communis	46	0.04	0.08	0.02	0.08
1	1	0.96	0	t	1	Cyanistes caeruleus	Poecile palustris/Poe cile montanus	46	0.06	0.09	0.09	0.08
0	0	0.46	0	cv	1	Parus major	Sitta europaea	46	0.04	0.09	0.04	0.09
0	1	0.92	0	t	1	Rubus fruetiosa	Pyrrhula pyrrula	46	0.06	0.08	0.07	0.08
1	1	0.82	0	t	1	Turdus philomelos	Woody nightshade	46	0.06	0.08	0.05	0.08
0	1	0.85	0	t	1	Crategus monogyna	Parus major	46	0.08	0.08	0.04	0.09
0	1	0.73	0	cv	1	Sitta europaea	Bryonia dioecia	46	0.04	0.09	0.06	0.08
0	1	0.63	0	t	1	Prunus spinosa	Fringilla coelebs	46	0.05	0.08	0.04	0.09
0	0	0.49	0	t	1	Turdus pilaris	Colurnba palumbus	46	0.04	0.08	0.04	0.09
0	1	0.65	0	t	1	Turdus pilaris	Euonymus europaeus	46	0.04	0.08	0.05	0.08

1	1	0.99	0	cv	1	Turdus merula	Poecile palustris/Poecile montanus	46	0.09	0.08	0.09	0.08
0	0	0.50	0	t	1	Euonymus europaeus	Tamus communis	46	0.05	0.08	0.02	0.08
0	1	0.99	0	t	1	Sambucus niger	Bryonia dioecia	46	0.12	0.08	0.06	0.08
0	1	0.75	0	t	1	Turdus iliacus	Lonicera perielymenum	46	0.05	0.08	0.05	0.08
0	1	0.88	0	cv	1	Turdus merula	Sitta europaea	46	0.09	0.08	0.04	0.09
0	1	0.63	0	t	1	Prunus spinosa	Colurnba palumbus	46	0.05	0.08	0.04	0.09
0	1	0.60	0	t	1	Rubus fruetiosa	Tamus communis	46	0.06	0.08	0.02	0.08
0	1	0.70	0	t	1	Turdus philomelos	Erithacus rubecula	46	0.06	0.08	0.04	0.09
0	1	0.88	0	cv	1	Euonymus europaeus	Pyrrhula pyrrula	46	0.05	0.08	0.07	0.08
0	1	0.91	0	t	1	Cyanistes caeruleus	Pyrrhula pyrrula	46	0.06	0.09	0.07	0.08
0	1	0.64	0	t	1	Rosa canina	Sylvia atricapilla	46	0.04	0.08	0.05	0.09
0	1	0.82	0	t	1	Turdus philomelos	Lonicera perielymenum	46	0.06	0.08	0.05	0.08
1	1	0.87	0	cv	1	Pyrrhula pyrrula	Woody nightshade	46	0.07	0.08	0.05	0.08
0	0	0.33	0	t	1	Erithacus rubecula	Tamus communis	46	0.04	0.09	0.02	0.08
0	1	0.60	0	t	1	Turdus iliacus	Colurnba palumbus	46	0.05	0.08	0.04	0.09
0	1	0.82	0	t	1	Bryonia dioecia	Sylvia atricapilla	46	0.06	0.08	0.05	0.09
1	1	0.57	0	cv	1	Turdus philomelos	Aegithalos caudatus	46	0.06	0.08	0.02	0.08
0	1	0.98	0	t	1	Sambucus niger	Woody nightshade	46	0.12	0.08	0.05	0.08
0	0	0.48	0	t	1	Turdus pilaris	Erithacus rubecula	46	0.04	0.08	0.04	0.09
0	1	0.62	0	t	1	Fringilla coelebs	Woody nightshade	46	0.04	0.09	0.05	0.08
1	1	0.92	0	cv	1	Cyanistes caeruleus	Hedera helix	46	0.06	0.09	0.07	0.08
0	1	0.85	0	t	1	Crategus monogyna	Sitta europaea	46	0.08	0.08	0.04	0.09
1	0	0.25	1	t	1	Chironomini	Tabellaria flocculosa	48	0.02	0.05	0.01	0.05

1	1	0.90	1	t	1	Chironomini	Unidentified detritus	48	0.02	0.05	0.08	0.05
1	0	0.25	1	cv	1	Chironomini	Melosira varians	48	0.02	0.05	0.01	0.05
1	1	0.67	1	t	1	Chironomini	Navicula avenacea	48	0.02	0.05	0.05	0.05
1	0	0.30	1	t	1	Chironomini	Staurostratu m	48	0.02	0.05	0.01	0.05
0	0	0.35	1	t	1	Gomphonema sp. unk	Helicopsyche	48	0.02	0.04	0.01	0.05
0	0	0.29	1	cv	1	Tabellaria flocculosa	Cricotopus sp I	48	0.01	0.05	0.02	0.05
0	0	0.23	1	t	1	Oligo II	Plant materials	48	0.01	0.06	0.01	0.05
0	0	0.19	1	t	1	Oligo II	Ameletopsis perscitus	48	0.01	0.06	0.01	0.05
1	1	0.85	1	t	1	Oligo II	Unidentified detritus	48	0.01	0.06	0.08	0.05
1	0	0.18	1	cv	1	Oligo II	Calothrix	48	0.01	0.06	0.01	0.05
0	0	0.26	1	t	1	Oligo II	Ulothrix	48	0.01	0.06	0.02	0.05
1	0	0.17	1	t	1	Oligo II	Polyplectropu s	48	0.01	0.06	0.01	0.05
0	0	0.21	1	t	1	Oligo II	Staurostratu m	48	0.01	0.06	0.01	0.05
0	0	0.30	1	cv	1	Aoteapsyche	Surirella elegans	48	0.03	0.06	0.01	0.05
0	0	0.39	1	t	1	Aoteapsyche	Plant materials	48	0.03	0.06	0.01	0.05
0	0	0.33	1	t	1	Aoteapsyche	Anguilla dieffenbachii	48	0.03	0.06	0.01	0.05
1	1	0.92	1	t	1	Aoteapsyche	Unidentified detritus	48	0.03	0.06	0.08	0.05
0	0	0.50	1	cv	1	Aoteapsyche	Fragilaria vaucheriae	48	0.03	0.06	0.02	0.05
0	1	0.60	1	t	1	Aoteapsyche	Achnanthes lanceolata	48	0.03	0.06	0.03	0.05
1	1	0.73	1	t	1	Aoteapsyche	Navicula avenacea	48	0.03	0.06	0.05	0.05
0	0	0.35	1	t	1	Aoteapsyche	Staurostratu m	48	0.03	0.06	0.01	0.05
1	1	0.89	1	cv	1	Aoteapsyche	Meiofauna	48	0.03	0.06	0.07	0.06
0	1	0.72	1	t	1	Gomphonema truncatum	Paucispiniger a approximata	48	0.03	0.05	0.04	0.05
0	1	0.73	1	t	1	Surirella	Deleatidium	48	0.01	0.05	0.07	0.05
0	1	0.66	1	t	1	Surirella	Zelandoperla	48	0.01	0.05	0.06	0.05
0	1	0.81	1	cv	1	Plant	Deleatidium	48	0.01	0.05	0.07	0.05
0	1	0.72	1	t	1	Plant materials	Hudsonema aliena	48	0.01	0.05	0.06	0.05
0	0	0.20	1	t	1	Plant materials	Paracalliope pale	48	0.01	0.05	0.01	0.05

0	0	0.26	1	t	1	Plant materials	Hydora nitida (l)	48	0.01	0.05	0.01	0.05
0	0	0.33	1	cv	1	Plant materials	Hudsonema amabilis	48	0.01	0.05	0.01	0.03
0	0	0.41	1	t	1	Plant	Polypedellum	48	0.01	0.05	0.03	0.05
0	0	0.27	1	t	1	Plant	Cristaperla	48	0.01	0.05	0.01	0.05
0	0	0.25	1	t	1	Plant	Helicopsyche	48	0.01	0.05	0.01	0.05
0	1	0.50	1	cv	1	Plant materials	Paracalliope fluviatalus	48	0.01	0.05	0.04	0.05
0	0	0.27	1	t	1	Plant	Scirtid	48	0.01	0.05	0.01	0.06
0	0	0.49	1	t	1	Plant	Coloburiscus	48	0.01	0.05	0.03	0.05
0	0	0.32	1	t	1	Plant	Zelandobius	48	0.01	0.05	0.02	0.05
0	0	0.31	1	cv	1	Plant	Oligo skinny	48	0.01	0.05	0.02	0.05
0	0	0.37	1	t	1	Plant	Eriopterini	48	0.01	0.05	0.02	0.05
0	0	0.21	1	t	1	Plant materials	Neozephlebia scita	48	0.01	0.05	0.01	0.05
0	0	0.44	1	t	1	Plant materials	Cricotopus sp II	48	0.01	0.05	0.03	0.05
0	1	0.52	1	cv	1	Plant materials	Hydora nitida (ad)	48	0.01	0.05	0.04	0.05
0	0	0.35	1	t	1	Plant materials	Austrosimulium	48	0.01	0.05	0.02	0.05
0	0	0.24	1	t	1	Plant materials	Austroperla cyrene	48	0.01	0.05	0.01	0.05
0	0	0.21	1	t	1	Plant	Mischoderus	48	0.01	0.05	0.01	0.05
0	1	0.53	1	cv	1	Plant materials	Paucispinigera approximata	48	0.01	0.05	0.04	0.05
0	1	0.75	1	t	1	Plant	Zelandoperla	48	0.01	0.05	0.06	0.05
0	1	0.77	1	t	1	Deleatidium	Achnanthes minutissima	48	0.07	0.05	0.01	0.05
0	1	0.73	1	t	1	Deleatidium	Ameletopsis perscitus	48	0.07	0.05	0.01	0.05
0	1	0.73	1	cv	1	Deleatidium	Anguilla dieffenbachii	48	0.07	0.05	0.01	0.05
0	1	0.83	1	t	1	Deleatidium	Achnanthes linearis	48	0.07	0.05	0.02	0.05
1	1	0.99	1	t	1	Deleatidium	Unidentified detritus	48	0.07	0.05	0.08	0.05
0	1	0.69	1	t	1	Deleatidium	Costachorema	48	0.07	0.05	0.01	0.05
0	1	0.80	1	cv	1	Deleatidium	Ulothrix	48	0.07	0.05	0.02	0.05
0	1	0.80	1	t	1	Deleatidium	Rhoicosphenia curvata	48	0.07	0.05	0.02	0.05
1	1	0.94	1	t	1	Deleatidium	Blue-green algae	48	0.07	0.05	0.05	0.05
1	1	0.89	1	t	1	Deleatidium	Achnanthes lanceolata	48	0.07	0.05	0.03	0.05
1	1	0.92	1	cv	1	Deleatidium	Cocconeis placentula	48	0.07	0.05	0.04	0.05

0	1	0.74	1	t	1	Deleatidium	Gomphoneis herculeana	48	0.07	0.05	0.01	0.05
0	1	0.84	1	t	1	Deleatidium	Stenoperla prasinia	48	0.07	0.05	0.02	0.05
0	1	0.94	1	t	1	Deleatidium	Navicula avenacea	48	0.07	0.05	0.05	0.05
1	1	0.91	1	cv	1	Deleatidium	Hydrobiosis parumbripen nis	48	0.07	0.05	0.04	0.05
0	1	0.83	1	t	1	Deleatidium	Gomphonem a angustatum	48	0.07	0.05	0.02	0.05
1	1	0.91	1	t	1	Hudsonema aliena	Blue-green algae	48	0.06	0.05	0.05	0.05
0	1	0.66	1	t	1	Hudsonema aliena	Staurostratu m	48	0.06	0.05	0.01	0.05
1	0	0.38	1	cv	1	Sphaerid	Staurostratu m	48	0.02	0.03	0.01	0.05
0	1	0.74	1	t	1	Achnanthes minutissima	Zelandoperla	48	0.01	0.05	0.06	0.05
1	1	0.82	1	t	1	Stictocladius	Unidentified detritus	48	0.01	0.06	0.08	0.05
1	0	0.17	1	t	1	Stictocladius	Staurostratu m	48	0.01	0.06	0.01	0.05
0	0	0.19	1	cv	1	Ameletopsis perscitus	Anguilla dieffenbachii	48	0.01	0.05	0.01	0.05
1	1	0.85	1	t	1	Ameletopsis perscitus	Unidentified detritus	48	0.01	0.05	0.08	0.05
0	0	0.42	1	t	1	Ameletopsis perscitus	Coloburiscus	48	0.01	0.05	0.03	0.05
0	0	0.25	1	t	1	Ameletopsis perscitus	Oligo skinny	48	0.01	0.05	0.02	0.05
0	0	0.37	1	cv	1	Ameletopsis perscitus	Cricotopus sp II	48	0.01	0.05	0.03	0.05
0	1	0.79	1	t	1	Ameletopsis perscitus	Meiofauna	48	0.01	0.05	0.07	0.06
1	1	0.93	1	t	1	Paralimnophil a skuseii	Unidentified detritus	48	0.03	0.06	0.08	0.05
1	0	0.38	1	t	1	Paralimnophil a skuseii	Staurostratu m	48	0.03	0.06	0.01	0.05
1	1	0.85	1	cv	1	Anguilla dieffenbachii	Unidentified detritus	48	0.01	0.05	0.08	0.05
0	0	0.42	1	t	1	Anguilla dieffenbachii	Coloburiscus	48	0.01	0.05	0.03	0.05
0	0	0.46	1	t	1	Anguilla dieffenbachii	Hydrobiosis parumbripen nis	48	0.01	0.05	0.04	0.05
1	1	0.91	1	t	1	Cricotopus sp I	Unidentified detritus	48	0.02	0.05	0.08	0.05
1	0	0.28	1	cv	1	Cricotopus sp I	Melosira varians	48	0.02	0.05	0.01	0.05

1	1	0.70	1	t	1	Cricotopus sp I	Navicula avenacea	48	0.02	0.05	0.05	0.05
1	0	0.33	1	t	1	Cricotopus sp I	Staurostratu m	48	0.02	0.05	0.01	0.05
0	1	0.59	1	t	1	Achnanthes linearis	Hydora nitida (ad)	48	0.02	0.05	0.04	0.05
1	1	0.80	1	cv	1	Achnanthes linearis	Zelandoperla	48	0.02	0.05	0.06	0.05
0	0	0.21	1	t	1	Pinnularia spp.	Oligo Lumbri pink	48	0.01	0.03	0.01	0.05
0	1	0.71	1	t	1	Synedra ulna	Zelandoperla	48	0.01	0.05	0.06	0.05
0	0	0.41	1	t	1	Gomphonema accuminatum	Paracalliope fluviatalus	48	0.01	0.05	0.04	0.05
0	0	0.23	1	cv	1	Paracalliope pale	Frustulia rhomboides	48	0.01	0.05	0.02	0.05
1	1	0.82	1	t	1	Paracalliope pale	Unidentified detritus	48	0.01	0.05	0.08	0.05
0	0	0.23	1	t	1	Paracalliope pale	Rhoicospheni a curvata	48	0.01	0.05	0.02	0.05
0	0	0.18	1	t	1	Paracalliope pale	Batrachosper mum	48	0.01	0.05	0.01	0.05
0	1	0.54	1	cv	1	Paracalliope pale	Blue-green algae	48	0.01	0.05	0.05	0.05
0	0	0.17	1	t	1	Paracalliope pale	Gomphoneis herculeana	48	0.01	0.05	0.01	0.05
0	1	0.52	1	t	1	Paracalliope pale	Navicula avenacea	48	0.01	0.05	0.05	0.05
1	1	0.85	1	t	1	Achnanthes inflata	Zelandoperla	48	0.03	0.05	0.06	0.05
0	0	0.35	1	cv	1	Gyrosigma	Helicopsyche	48	0.02	0.04	0.01	0.05
0	1	0.54	1	t	1	Frustulia rhomboides	Paracalliope fluviatalus	48	0.02	0.05	0.04	0.05
0	0	0.23	1	t	1	Frustulia rhomboides	Neozephlebia scita	48	0.02	0.05	0.01	0.05
0	1	0.77	1	t	1	Frustulia rhomboides	Zelandoperla	48	0.02	0.05	0.06	0.05
0	0	0.23	1	cv	1	Hydora nitida (l)	Staurostratu m	48	0.01	0.05	0.01	0.05
1	1	0.53	1	t	1	Hudsonema amabilis	Diatoma heimale	48	0.01	0.03	0.02	0.02
0	0	0.30	1	t	1	Hudsonema amabilis	Staurostratu m	48	0.01	0.03	0.01	0.05
0	0	0.49	1	t	1	Eunotia pectinalis	Hydora nitida (ad)	48	0.01	0.04	0.04	0.05
0	0	0.33	1	cv	1	Eunotia pectinalis	Austrosimuliu m	48	0.01	0.04	0.02	0.05
1	1	0.92	1	t	1	Polypedellum	Unidentified detritus	48	0.03	0.05	0.08	0.05
0	1	0.61	1	t	1	Polypedellum	Achnanthes lanceolata	48	0.03	0.05	0.03	0.05

1	1	0.69	1	t	1	Polypedellum	Cocconeis placentula	48	0.03	0.05	0.04	0.05
1	0	0.36	1	cv	1	Polypedellum	Staurostratum	48	0.03	0.05	0.01	0.05
1	0	0.32	1	t	1	Polypedellum	Nitzschia dissipata	48	0.03	0.05	0.01	0.05
1	0	0.31	1	t	1	Polypedellum	Cymbella kappii	48	0.03	0.05	0.01	0.05
1	1	0.87	1	t	1	Cristaperla	Unidentified detritus	48	0.01	0.05	0.08	0.05
0	1	0.63	1	cv	1	Cristaperla	Blue-green algae	48	0.01	0.05	0.05	0.05
0	1	0.56	1	t	1	Cristaperla	Cocconeis placentula	48	0.01	0.05	0.04	0.05
0	0	0.35	1	t	1	Cristaperla	Stenoperla prasinia	48	0.01	0.05	0.02	0.05
0	0	0.24	1	t	1	Cristaperla	Staurostratum	48	0.01	0.05	0.01	0.05
1	1	0.83	1	cv	1	Unidentified detritus	Helicopsyche	48	0.08	0.05	0.01	0.05
1	1	0.79	1	t	1	Unidentified detritus	Costachorema	48	0.08	0.05	0.01	0.05
1	1	0.94	1	t	1	Unidentified detritus	Paracalliope fluviatalus	48	0.08	0.05	0.04	0.05
1	1	0.85	1	t	1	Unidentified detritus	Scirtid	48	0.08	0.05	0.01	0.06
0	1	0.80	1	cv	1	Unidentified detritus	Oligo Lumbri pink	48	0.08	0.05	0.01	0.05
1	1	0.87	1	t	1	Unidentified detritus	Oligo skinny	48	0.08	0.05	0.02	0.05
1	1	0.90	1	t	1	Unidentified detritus	Eriopterini	48	0.08	0.05	0.02	0.05
1	1	0.89	1	t	1	Unidentified detritus	Zelandotipula	48	0.08	0.05	0.02	0.05
0	1	0.78	1	cv	1	Unidentified detritus	Nannochorista phillpotti	48	0.08	0.05	0.01	0.06
0	1	0.80	1	t	1	Unidentified detritus	Neozephlebia scita	48	0.08	0.05	0.01	0.05
1	1	0.92	1	t	1	Unidentified detritus	Cricotopus sp II	48	0.08	0.05	0.03	0.05
1	1	0.85	1	t	1	Unidentified detritus	Eye forward chiron	48	0.08	0.05	0.01	0.06
1	1	0.89	1	cv	1	Unidentified detritus	Austrosimulium	48	0.08	0.05	0.02	0.05
1	1	0.87	1	t	1	Unidentified detritus	Hydrobiosella stenocerca	48	0.08	0.05	0.02	0.05
1	1	0.82	1	t	1	Unidentified detritus	Austroperla cyrene	48	0.08	0.05	0.01	0.05
1	1	0.94	1	t	1	Unidentified detritus	Hydrobiosis parumbripennis	48	0.08	0.05	0.04	0.05

1	1	0.87	1	cv	1	Unidentified detritus	Polypedellum II	48	0.08	0.05	0.02	0.05
1	1	0.95	1	t	1	Unidentified detritus	Paucispiniger a approximata	48	0.08	0.05	0.04	0.05
1	1	0.98	1	t	1	Unidentified detritus	Zelandoperla	48	0.08	0.05	0.06	0.05
0	0	0.28	1	t	1	Fragilaria vaucheriae	Oligo Lumbri pink	48	0.02	0.05	0.01	0.05
0	0	0.29	1	cv	1	Fragilaria vaucheriae	Mischoderus	48	0.02	0.05	0.01	0.05
0	1	0.82	1	t	1	Fragilaria vaucheriae	Zelandoperla	48	0.02	0.05	0.06	0.05
0	0	0.16	1	t	1	Calothrix	Oligo Lumbri pink	48	0.01	0.05	0.01	0.05
0	0	0.25	1	t	1	Calothrix	Zelandobius	48	0.01	0.05	0.02	0.05
0	0	0.30	1	cv	1	Calothrix	Eriopterini	48	0.01	0.05	0.02	0.05
0	0	0.27	1	t	1	Calothrix	Zelandotipula	48	0.01	0.05	0.02	0.05
0	0	0.24	1	t	1	Calothrix	Polypedellum II	48	0.01	0.05	0.02	0.05
0	0	0.20	1	t	1	Melosira varians	Eye forward chiron	48	0.01	0.05	0.01	0.06
0	0	0.28	1	cv	1	Melosira varians	Austrosimuliu m	48	0.01	0.05	0.02	0.05
0	1	0.52	1	t	1	Helicopsyche	Cocconeis placentula	48	0.01	0.05	0.04	0.05
0	0	0.17	1	t	1	Gomphonema intricatum	Oligo Lumbri pink	48	0.01	0.04	0.01	0.05
0	1	0.74	1	t	1	Cymbella mulleri	Zelandoperla	48	0.01	0.05	0.06	0.05
0	0	0.47	1	cv	1	Ulothrix	Cricotopus sp II	48	0.02	0.05	0.03	0.05
0	1	0.52	1	t	1	Paracalliope fluviatalus	Rhoicospheni a curvata	48	0.04	0.05	0.02	0.05
1	0	0.44	1	t	1	Paracalliope fluviatalus	Batrachosper mum	48	0.04	0.05	0.01	0.05
1	1	0.81	1	t	1	Paracalliope fluviatalus	Blue-green algae	48	0.04	0.05	0.05	0.05
0	0	0.43	1	cv	1	Paracalliope fluviatalus	Gomphoneis herculeana	48	0.04	0.05	0.01	0.05
1	1	0.80	1	t	1	Paracalliope fluviatalus	Navicula avenacea	48	0.04	0.05	0.05	0.05
1	0	0.39	1	t	1	Paracalliope fluviatalus	Cymbella kappi	48	0.04	0.05	0.01	0.05
0	0	0.38	1	t	1	Rhoicosphenia curvata	Austrosimuliu m	48	0.02	0.05	0.02	0.05
0	1	0.77	1	cv	1	Rhoicosphenia curvata	Zelandoperla	48	0.02	0.05	0.06	0.05
1	0	0.24	1	t	1	Scirtid	Staurostratu m	48	0.01	0.06	0.01	0.05

0	1	0.68	1	t	1	Coloburiscus	Achnanthes lanceolata	48	0.03	0.05	0.03	0.05
0	1	0.75	1	t	1	Coloburiscus	Cocconeis placentula	48	0.03	0.05	0.04	0.05
0	0	0.49	1	cv	1	Coloburiscus	Hydrobiosella stenocerca	48	0.03	0.05	0.02	0.05
0	1	0.57	1	t	1	Coloburiscus	Stenoperla prasinia	48	0.03	0.05	0.02	0.05
0	0	0.44	1	t	1	Coloburiscus	Staurostratu m	48	0.03	0.05	0.01	0.05
0	1	0.56	1	t	1	Coloburiscus	Green algae	48	0.03	0.05	0.02	0.05
0	1	0.55	1	cv	1	Oligo Lumbri pink	Blue-green algae	48	0.01	0.05	0.05	0.05
0	0	0.38	1	t	1	Oligo Lumbri pink	Achnanthes lanceolata	48	0.01	0.05	0.03	0.05
0	0	0.47	1	t	1	Oligo Lumbri pink	Cocconeis placentula	48	0.01	0.05	0.04	0.05
0	0	0.18	1	t	1	Oligo Lumbri pink	Staurostratu m	48	0.01	0.05	0.01	0.05
0	1	0.62	1	cv	1	Zelandobius	Cocconeis placentula	48	0.02	0.05	0.04	0.05
0	0	0.29	1	t	1	Zelandobius	Staurostratu m	48	0.02	0.05	0.01	0.05
1	1	0.67	1	t	1	Oligo skinny	Blue-green algae	48	0.02	0.05	0.05	0.05
1	1	0.64	1	t	1	Oligo skinny	Navicula avenacea	48	0.02	0.05	0.05	0.05
1	0	0.27	1	cv	1	Oligo skinny	Staurostratu m	48	0.02	0.05	0.01	0.05
1	0	0.33	1	t	1	Eriopterini	Staurostratu m	48	0.02	0.05	0.01	0.05
0	0	0.44	1	t	1	Eriopterini	Green algae	48	0.02	0.05	0.02	0.05
1	1	0.70	1	t	1	Zelandotipula	Blue-green algae	48	0.02	0.05	0.05	0.05
0	0	0.29	1	cv	1	Zelandotipula	Gomphoneis herculeana	48	0.02	0.05	0.01	0.05
1	1	0.67	1	t	1	Zelandotipula	Navicula avenacea	48	0.02	0.05	0.05	0.05
1	1	0.74	1	t	1	Nannochorista phillpotti	Meiofauna	48	0.01	0.06	0.07	0.06
0	1	0.55	1	t	1	Nitzschia linearis	Austrosimuliu m	48	0.03	0.04	0.02	0.05
0	1	0.52	1	cv	1	Blue-green algae	Neozephlebia scita	48	0.05	0.05	0.01	0.05
0	1	0.77	1	t	1	Blue-green algae	Cricotopus sp II	48	0.05	0.05	0.03	0.05
0	1	0.82	1	t	1	Blue-green algae	Hydora nitida (ad)	48	0.05	0.05	0.04	0.05
0	1	0.57	1	t	1	Blue-green algae	Austroperla cyrene	48	0.05	0.05	0.01	0.05

0	1	0.65	1	cv	1	Blue-green algae	Polypedellum II	48	0.05	0.05	0.02	0.05
0	1	0.83	1	t	1	Blue-green algae	Paucispiniger a approximata	48	0.05	0.05	0.04	0.05
0	1	0.93	1	t	1	Blue-green algae	Zelandoperla	48	0.05	0.05	0.06	0.05
0	0	0.38	1	t	1	Neozephlebia scita	Achnanthes lanceolata	48	0.01	0.05	0.03	0.05
0	0	0.47	1	cv	1	Neozephlebia scita	Cocconeis placentula	48	0.01	0.05	0.04	0.05
0	1	0.52	1	t	1	Neozephlebia scita	Navicula avenacea	48	0.01	0.05	0.05	0.05
0	0	0.18	1	t	1	Neozephlebia scita	Staurostratu m	48	0.01	0.05	0.01	0.05
0	1	0.77	1	t	1	Ankitodesmus sp.	Zelandoperla	48	0.02	0.05	0.06	0.05
0	1	0.64	1	cv	1	Achnanthes lanceolata	Cricotopus sp II	48	0.03	0.05	0.03	0.05
0	1	0.71	1	t	1	Achnanthes lanceolata	Hydora nitida (ad)	48	0.03	0.05	0.04	0.05
0	1	0.55	1	t	1	Achnanthes lanceolata	Austrosimuliu m	48	0.03	0.05	0.02	0.05
0	0	0.38	1	t	1	Achnanthes lanceolata	Mischoderus	48	0.03	0.05	0.01	0.05
0	1	0.87	1	cv	1	Achnanthes lanceolata	Zelandoperla	48	0.03	0.05	0.06	0.05
0	1	0.77	1	t	1	Cocconeis placentula	Hydora nitida (ad)	48	0.04	0.05	0.04	0.05
0	1	0.63	1	t	1	Cocconeis placentula	Austrosimuliu m	48	0.04	0.05	0.02	0.05
0	1	0.58	1	t	1	Cocconeis placentula	Polypedellum II	48	0.04	0.05	0.02	0.05
1	1	0.90	1	cv	1	Cocconeis placentula	Zelandoperla	48	0.04	0.05	0.06	0.05
0	0	0.39	1	t	1	Cricotopus sp II	Staurostratu m	48	0.03	0.05	0.01	0.05
0	0	0.33	1	t	1	Cricotopus sp II	Epithemia sorex	48	0.03	0.05	0.01	0.05
0	1	0.81	1	t	1	Hydora nitida (ad)	Navicula avenacea	48	0.04	0.05	0.05	0.05
0	0	0.46	1	cv	1	Hydora nitida (ad)	Staurostratu m	48	0.04	0.05	0.01	0.05
0	1	0.58	1	t	1	Hydora nitida (ad)	Green algae	48	0.04	0.05	0.02	0.05
0	1	0.58	1	t	1	Hydora nitida (ad)	Gomphonem a angustatum	48	0.04	0.05	0.02	0.05
0	1	0.67	1	t	1	Nitzschia	Zelandoperla	48	0.01	0.05	0.06	0.05
0	0	0.32	1	cv	1	Austrosimuliu m	Gomphonem a parvulum	48	0.02	0.05	0.01	0.04

1	1	0.69	1	t	1	Austrosimulium	Navicula avenacea	48	0.02	0.05	0.05	0.05
0	0	0.31	1	t	1	Austrosimulium	Staurostratum	48	0.02	0.05	0.01	0.05
0	0	0.41	1	t	1	Austrosimulium	Gomphonema sp. III	48	0.02	0.05	0.02	0.04
0	0	0.27	1	cv	1	Austrosimulium	Nitzschia dissipata	48	0.02	0.05	0.01	0.05
0	0	0.42	1	t	1	Austrosimulium	Gomphonema angustatum	48	0.02	0.05	0.02	0.05
0	0	0.20	1	t	1	Austroperla cyrene	Gomphoneis herculeana	48	0.01	0.05	0.01	0.05
0	0	0.21	1	t	1	Austroperla cyrene	Staurostratum	48	0.01	0.05	0.01	0.05
0	0	0.20	1	cv	1	Polyplectropus	Staurostratum	48	0.01	0.05	0.01	0.05
0	1	0.71	1	t	1	Gomphoneis herculeana	Zelandoperla	48	0.01	0.05	0.06	0.05
1	1	0.87	1	t	1	Stenoperla prasinia	Meiofauna	48	0.02	0.05	0.07	0.06
0	0	0.28	1	t	1	Staurostratum	Polypedellum II	48	0.01	0.05	0.02	0.05
0	0	0.19	1	cv	1	Staurostratum	Mischoderus	48	0.01	0.05	0.01	0.05
0	0	0.50	1	t	1	Staurostratum	Paucispinigera approximata	48	0.01	0.05	0.04	0.05
0	1	0.80	1	t	1	Green algae	Zelandoperla	48	0.02	0.05	0.06	0.05
0	1	0.67	1	t	1	Terrestrial invertebrates	Zelandoperla	48	0.01	0.05	0.06	0.05
0	1	0.78	1	cv	1	Zelandoperla	Gomphonema angustatum	48	0.06	0.05	0.02	0.05
0	0	0.32	0	t	1	Achnanthes linearis	Scirtid	48	0.02	0.05	0.01	0.06
0	0	0.22	0	t	1	Cymbella mulleri	Nitzschia dissipata	48	0.01	0.05	0.01	0.05
0	1	0.57	0	t	1	Hydrobiosis parumbripenn	Gomphonema sp. III	48	0.04	0.05	0.02	0.04
0	0	0.30	0	cv	1	Paracalliope pale	Polypedellum	48	0.01	0.05	0.03	0.05
0	0	0.28	0	t	1	Surirella	Eriopterini	48	0.01	0.05	0.02	0.05
0	0	0.25	0	t	1	Achnanthes linearis	Neozephlebia scita	48	0.02	0.05	0.01	0.05
0	0	0.42	0	t	1	Gomphonema sp. III	Gomphonema angustatum	48	0.02	0.04	0.02	0.05
0	1	0.52	0	cv	1	Hydora nitida (ad)	Hydrobiosella stenocerca	48	0.04	0.05	0.02	0.05
0	1	0.55	0	t	1	Blue-green algae	Nitzschia dissipata	48	0.05	0.05	0.01	0.05
0	0	0.29	0	t	1	Austrosimulium	Austroperla cyrene	48	0.02	0.05	0.01	0.05

0	0	0.35	0	t	1	Eunotia pectinalis	Eriopterini	48	0.01	0.04	0.02	0.05
0	1	0.70	0	cv	1	Deleatidium	Mischoderus	48	0.07	0.05	0.01	0.05
1	1	0.79	0	t	1	Austroperla cyrene	Meiofauna	48	0.01	0.05	0.07	0.06
0	0	0.37	0	t	1	Hudsonema amabilis	Zelandobius	48	0.01	0.03	0.02	0.05
0	0	0.33	0	t	1	Cricotopus sp I	Eye forward chiron	48	0.02	0.05	0.01	0.06
0	0	0.26	0	cv	1	Helicopsyche	Hydrobiosella stenocerca	48	0.01	0.05	0.02	0.05
0	0	0.30	0	t	1	Aoteapsyche	Cymbella kappii	48	0.03	0.06	0.01	0.05
0	0	0.23	0	t	1	Pinnularia spp.	Nitzschia dissipata	48	0.01	0.03	0.01	0.05
0	1	0.54	0	t	1	Hydrobiosella stenocerca	Paucispiniger a approximata	48	0.02	0.05	0.04	0.05
0	0	0.21	0	cv	1	Rhoicosphenia curvata	Nannochorist a phillpotti	48	0.02	0.05	0.01	0.06
0	1	0.51	0	t	1	Ameletopsis perscitus	Cocconeis placentula	48	0.01	0.05	0.04	0.05
0	0	0.23	0	t	1	Hydora nitida (l)	Batrachosper mum	48	0.01	0.05	0.01	0.05
0	0	0.24	0	t	1	Frustulia rhomboides	Mischoderus	48	0.02	0.05	0.01	0.05
0	1	0.50	0	cv	1	Ulothrix	Diatoma heimale	48	0.02	0.05	0.02	0.02
1	1	0.87	0	t	1	Achnanthes linearis	Meiofauna	48	0.02	0.05	0.07	0.06
0	0	0.33	0	t	1	Pinnularia spp.	Ankitodesmu s sp.	48	0.01	0.03	0.02	0.05
0	0	0.21	0	t	1	Eunotia pectinalis	Gomphonem a intricatum	48	0.01	0.04	0.01	0.04
0	0	0.39	0	cv	1	Surirella elegans	Achnanthes lanceolata	48	0.01	0.05	0.03	0.05
1	1	0.61	0	t	1	Sphaerid	Diatoma heimale	48	0.02	0.03	0.02	0.02
0	1	0.64	0	t	1	Gomphonema truncatum	Cricotopus sp II	48	0.03	0.05	0.03	0.05
0	0	0.47	0	t	1	Hydora nitida (ad)	Eye forward chiron	48	0.04	0.05	0.01	0.06
0	0	0.39	0	cv	1	Achnanthes lanceolata	Nitzschia dissipata	48	0.03	0.05	0.01	0.05
0	1	0.56	0	t	1	Gyrosigma	Cricotopus sp II	48	0.02	0.04	0.03	0.05
0	0	0.17	0	t	1	Batrachosper mum	Nannochorist a phillpotti	48	0.01	0.05	0.01	0.06
0	1	0.65	0	t	1	Plant materials	Blue-green algae	48	0.01	0.05	0.05	0.05

0	1	0.59	0	cv	1	Sphaerid	Achnanthes inflata	48	0.02	0.03	0.03	0.05
0	0	0.44	0	t	1	Fragilaria vaucheriae	Gomphonema sp. III	48	0.02	0.05	0.02	0.04
0	0	0.24	0	t	1	Plant materials	Ameletopsis perscitus	48	0.01	0.05	0.01	0.05
0	0	0.31	0	t	1	Frustulia rhomboides	Cymbella mulleri	48	0.02	0.05	0.01	0.05
0	0	0.44	0	cv	1	Polypedellum	Zelandobius	48	0.03	0.05	0.02	0.05
0	0	0.32	0	t	1	Surirella	Sphaerid	48	0.01	0.05	0.02	0.03
0	0	0.40	0	t	1	Zelandobius	Green algae	48	0.02	0.05	0.02	0.05
0	0	0.18	0	t	1	Tabellaria flocculosa	Anguilla dieffenbachii	48	0.01	0.05	0.01	0.05
0	0	0.17	0	cv	1	Ameletopsis perscitus	Oligo Lumbri pink	48	0.01	0.05	0.01	0.05
0	0	0.25	0	t	1	Melosira varians	Ankitodesmus sp.	48	0.01	0.05	0.02	0.05
0	1	0.50	0	t	1	Sphaerid	Stenoperla prasinia	48	0.02	0.03	0.02	0.05
0	1	0.61	0	t	1	Gyrosigma	Nitzschia linearis	48	0.02	0.04	0.03	0.04
0	0	0.40	0	cv	1	Plant	Gyrosigma	48	0.01	0.05	0.02	0.04
0	1	0.76	0	t	1	Gomphonema truncatum	Cocconeis placentula	48	0.03	0.05	0.04	0.05
0	0	0.17	0	t	1	Calothrix	Nitzschia dissipata	48	0.01	0.05	0.01	0.05
1	0	0.28	0	t	1	Cricotopus sp I	Cymbella kappii	48	0.02	0.05	0.01	0.05
0	0	0.44	0	cv	1	Achnanthes inflata	Cymbella mulleri	48	0.03	0.05	0.01	0.05
0	0	0.36	0	t	1	Chironomini	Ankitodesmus sp.	48	0.02	0.05	0.02	0.05
0	0	0.37	0	t	1	Oligo skinny	Green algae	48	0.02	0.05	0.02	0.05
0	0	0.32	0	t	1	Chironomini	Achnanthes minutissima	48	0.02	0.05	0.01	0.05
0	0	0.22	0	cv	1	Anguilla dieffenbachii	Scirtid	48	0.01	0.05	0.01	0.06
0	0	0.49	0	t	1	Hudsonema amabilis	Cricotopus sp II	48	0.01	0.03	0.03	0.05
0	1	0.51	0	t	1	Pinnularia spp.	Paracalliope fluviatalus	48	0.01	0.03	0.04	0.05
1	0	0.36	0	t	1	Hydrobiosella stenocerca	Gomphonema sp. III	48	0.02	0.05	0.02	0.04
0	0	0.25	0	cv	1	Costachorema	Austrosimulium	48	0.01	0.05	0.02	0.05
0	0	0.22	0	t	1	Gomphonema intricatum	Eye forward chiron	48	0.01	0.04	0.01	0.06
1	1	0.76	0	t	1	Hydrobiosella stenocerca	Zelandoperla	48	0.02	0.05	0.06	0.05
0	0	0.40	0	t	1	Paracalliope pale	Hydora nitida (ad)	48	0.01	0.05	0.04	0.05

1	0	0.42	0	cv	1	Oligo II	Coloburiscus	48	0.01	0.06	0.03	0.05
0	1	0.75	0	t	1	Meiofauna	Nitzschia dissipata	48	0.07	0.06	0.01	0.05
0	0	0.28	0	t	1	Scirtid	Hydrobiosella stenocerca	48	0.01	0.06	0.02	0.05
0	0	0.28	0	t	1	Polyplectropus	Green algae	48	0.01	0.05	0.02	0.05
0	0	0.29	0	cv	1	Aoteapsyche	Oligo Lumbri pink	48	0.03	0.06	0.01	0.05
0	0	0.22	0	t	1	Oligo skinny	Epithemia sorex	48	0.02	0.05	0.01	0.05
0	1	0.59	0	t	1	Ameletopsis perscitus	Blue-green algae	48	0.01	0.05	0.05	0.05
0	1	0.61	0	t	1	Rhoicosphenia curvata	Cocconeis placentula	48	0.02	0.05	0.04	0.05
0	0	0.45	0	cv	1	Hydora nitida (l)	Nitzschia linearis	48	0.01	0.05	0.03	0.04
0	0	0.32	0	t	1	Eriopterini	Gomphoneis herculeana	48	0.02	0.05	0.01	0.05
0	0	0.33	0	t	1	Achnanthes minutissima	Gomphonema sp. III	48	0.01	0.05	0.02	0.04
0	0	0.37	0	t	1	Plant materials	Stenoperla prasinia	48	0.01	0.05	0.02	0.05
0	0	0.44	0	cv	1	Eriopterini	Gomphonema angustatum	48	0.02	0.05	0.02	0.05
0	0	0.45	0	t	1	Paralimnophila skuseii	Rhoicosphenia curvata	48	0.03	0.06	0.02	0.05
0	0	0.46	0	t	1	Chironomini	Polypedellum	48	0.02	0.05	0.03	0.05
0	1	0.83	0	t	1	Unidentified detritus	Gomphoneis herculeana	48	0.08	0.05	0.01	0.05
0	0	0.43	0	cv	1	Sphaerid	Oligo skinny	48	0.02	0.03	0.02	0.05
0	1	0.70	0	t	1	Blue-green algae	Gomphonema angustatum	48	0.05	0.05	0.02	0.05
0	1	0.79	0	t	1	Coloburiscus	Navicula avenacea	48	0.03	0.05	0.05	0.05
0	0	0.19	0	t	1	Oligo Lumbri pink	Gomphonema parvulum	48	0.01	0.05	0.01	0.04
0	1	0.59	0	cv	1	Fragilaria vaucheriae	Coloburiscus	48	0.02	0.05	0.03	0.05
0	0	0.46	0	t	1	Paracalliope fluviatalus	Scirtid	48	0.04	0.05	0.01	0.06
1	0	0.42	0	t	1	Hydrobiosis parumbripenn	Cymbella kappii	48	0.04	0.05	0.01	0.05
0	0	0.29	0	t	1	Plant materials	Pinnularia spp.	48	0.01	0.05	0.01	0.03
0	1	0.57	0	cv	1	Coloburiscus	Eriopterini	48	0.03	0.05	0.02	0.05
0	0	0.17	0	t	1	Anguilla dieffenbachii	Epithemia sorex	48	0.01	0.05	0.01	0.05
0	0	0.29	0	t	1	Rhoicosphenia curvata	Gomphonema parvulum	48	0.02	0.05	0.01	0.04
0	0	0.37	0	t	1	Hydrobiosella stenocerca	Green algae	48	0.02	0.05	0.02	0.05

0	0	0.35	0	cv	1	Tabellaria flocculosa	Paralimnophila skuseii	48	0.01	0.05	0.03	0.06
0	0	0.17	0	t	1	Paracalliope pale	Helicopsyche	48	0.01	0.05	0.01	0.05
0	1	0.61	0	t	1	Gomphonema truncatum	Polypedellum	48	0.03	0.05	0.03	0.05
0	1	0.87	0	t	1	Eunotia pectinalis	Unidentified detritus	48	0.01	0.04	0.08	0.05
0	0	0.26	0	cv	1	Plant materials	Staurostratum	48	0.01	0.05	0.01	0.05
0	0	0.31	0	t	1	Pinnularia spp.	Polypedellum II	48	0.01	0.03	0.02	0.05
0	0	0.22	0	t	1	Anguilla dieffenbachii	Eye forward chiron	48	0.01	0.05	0.01	0.06
0	0	0.22	0	t	1	Pinnularia spp.	Cymbella kappi	48	0.01	0.03	0.01	0.05
0	1	0.65	0	cv	1	Cocconeis placentula	Stenoperla prasinia	48	0.04	0.05	0.02	0.05
0	1	0.72	0	t	1	Paracalliope fluviatalus	Hydrobiosis parumbripennis	48	0.04	0.05	0.04	0.05
0	0	0.31	0	t	1	Chironomini	Scirtid	48	0.02	0.05	0.01	0.06
0	0	0.25	0	t	1	Zelandotipula	Cymbella kappi	48	0.02	0.05	0.01	0.05
1	1	0.60	0	cv	1	Aoteapsyche	Coloburiscus	48	0.03	0.06	0.03	0.05
0	0	0.20	0	t	1	Gomphonema parvulum	Mischoderus	48	0.01	0.04	0.01	0.05
0	0	0.17	0	t	1	Ameletopsis perscitus	Terrestrial invertebrates	48	0.01	0.05	0.01	0.05
0	0	0.19	0	t	1	Eunotia pectinalis	Neozephlebia scita	48	0.01	0.04	0.01	0.05
0	0	0.21	0	cv	1	Stictocladius	Polypedellum II	48	0.01	0.06	0.02	0.05
1	0	0.38	0	t	1	Paralimnophila skuseii	Batrachospermum	48	0.03	0.06	0.01	0.05
0	1	0.68	0	t	1	Achnanthes inflata	Hydora nitida (ad)	48	0.03	0.05	0.04	0.05
0	1	0.57	0	t	1	Gomphonema truncatum	Stenoperla prasinia	48	0.03	0.05	0.02	0.05
0	0	0.28	0	cv	1	Eunotia pectinalis	Polypedellum II	48	0.01	0.04	0.02	0.05
0	1	0.64	0	t	1	Achnanthes inflata	Diatoma heimale	48	0.03	0.05	0.02	0.02
0	0	0.41	0	t	1	Chironomini	Cricotopus sp I	48	0.02	0.05	0.02	0.05
0	0	0.15	0	t	1	Surirella elegans	Oligo Lumbri pink	48	0.01	0.05	0.01	0.05
0	0	0.26	0	cv	1	Oligo II	Frustulia rhomboides	48	0.01	0.06	0.02	0.05
0	1	0.91	0	t	1	Unidentified detritus	Fragilaria vaucheriae	48	0.08	0.05	0.02	0.05

0	0	0.30	0	t	1	Gyrosigma	Neozephlebia scita	48	0.02	0.04	0.01	0.05
0	0	0.31	0	t	1	Oligo II	Cricotopus sp I	48	0.01	0.06	0.02	0.05
0	0	0.28	0	cv	1	Batrachospermum	Ankitodesmus sp.	48	0.01	0.05	0.02	0.05
0	0	0.24	0	t	1	Gomphonema parvulum	Staurostratum	48	0.01	0.04	0.01	0.05
0	0	0.38	0	t	1	Paracalliope pale	Achnanthes lanceolata	48	0.01	0.05	0.03	0.05
0	0	0.37	0	t	1	Ulothrix	Gomphonema sp. III	48	0.02	0.05	0.02	0.04
1	1	0.79	0	cv	1	Hudsonema aliena	Polypedellum	48	0.06	0.05	0.03	0.05
0	0	0.42	0	t	1	Cristaperla	Cricotopus sp II	48	0.01	0.05	0.03	0.05
0	0	0.42	0	t	1	Achnanthes minutissima	Paralimnophila skuseii	48	0.01	0.05	0.03	0.06
0	0	0.24	0	t	1	Plant materials	Anguilla dieffenbachii	48	0.01	0.05	0.01	0.05
0	0	0.27	0	cv	1	Chironomini	Ameletopsis perscitus	48	0.02	0.05	0.01	0.05
0	0	0.36	0	t	1	Oligo II	Paralimnophila skuseii	48	0.01	0.06	0.03	0.06
0	0	0.22	0	t	1	Pinnularia spp.	Epithemia sorex	48	0.01	0.03	0.01	0.05
0	1	0.77	0	t	1	Achnanthes inflata	Navicula avenacea	48	0.03	0.05	0.05	0.05
0	0	0.36	0	cv	1	Chironomini	Ulothrix	48	0.02	0.05	0.02	0.05
0	0	0.19	0	t	1	Staurostratum	Cymbella kappi	48	0.01	0.05	0.01	0.05
0	1	0.75	0	t	1	Gomphonema sp. unk	Blue-green algae	48	0.02	0.04	0.05	0.05
0	1	0.87	0	t	1	Diatoma heimale	Zelandoperla	48	0.02	0.02	0.06	0.05
0	0	0.20	0	cv	1	Helicopsyche	Austroperla cyrene	48	0.01	0.05	0.01	0.05
1	0	0.27	0	t	1	Cricotopus sp I	Mischoderus	48	0.02	0.05	0.01	0.05
0	0	0.37	0	t	1	Paralimnophila skuseii	Helicopsyche	48	0.03	0.06	0.01	0.05
0	0	0.42	0	t	1	Gomphonema sp. unk	Oligo skinny	48	0.02	0.04	0.02	0.05
0	0	0.32	0	cv	1	Eye forward chiron	Gomphonema angustatum	48	0.01	0.06	0.02	0.05
0	0	0.46	0	t	1	Achnanthes minutissima	Diatoma heimale	48	0.01	0.05	0.02	0.02
1	1	0.90	0	t	1	Cricotopus sp	Meiofauna	48	0.03	0.05	0.07	0.06
1	0	0.28	0	t	1	Cricotopus sp I	Nitzschia dissipata	48	0.02	0.05	0.01	0.05
0	1	0.98	0	cv	1	Hudsonema aliena	Unidentified detritus	48	0.06	0.05	0.08	0.05

0	1	0.55	0	t	1	Frustulia rhomboides	Hydora nitida (ad)	48	0.02	0.05	0.04	0.05
0	0	0.18	0	t	1	Ameletopsis perscitus	Nitzschia dissipata	48	0.01	0.05	0.01	0.05
0	1	0.61	0	t	1	Gomphonema parvulum	Navicula avenacea	48	0.01	0.04	0.05	0.05
0	1	0.51	0	cv	1	Navicula avenacea	Terrestrial invertebrates	48	0.05	0.05	0.01	0.05
0	0	0.38	0	t	1	Hydora nitida	Polypedellum	48	0.01	0.05	0.03	0.05
0	0	0.48	0	t	1	Achnanthes minutissima	Nitzschia linearis	48	0.01	0.05	0.03	0.04
0	0	0.16	0	t	1	Gomphonema accuminatum	Epithemia sorex	48	0.01	0.05	0.01	0.05
0	0	0.15	0	cv	1	Paracalliope pale	Terrestrial invertebrates	48	0.01	0.05	0.01	0.05
0	0	0.16	0	t	1	Epithemia sorex	Cymbella kappii	48	0.01	0.05	0.01	0.05
0	1	0.87	0	t	1	Unidentified detritus	Ulothrix	48	0.08	0.05	0.02	0.05
0	0	0.38	0	t	1	Ulothrix	Austrosimulium	48	0.02	0.05	0.02	0.05
0	0	0.47	0	cv	1	Gomphonema truncatum	Achnanthes minutissima	48	0.03	0.05	0.01	0.05
0	0	0.34	0	t	1	Aoteapsyche	Helicopsyche	48	0.03	0.06	0.01	0.05
0	0	0.19	0	t	1	Scirtid	Terrestrial invertebrates	48	0.01	0.06	0.01	0.05
0	0	0.18	0	t	1	Gomphonema intricatum	Epithemia sorex	48	0.01	0.04	0.01	0.05
0	1	0.90	0	cv	1	Hudsonema amabilis	Unidentified detritus	48	0.01	0.03	0.08	0.05
0	0	0.27	0	t	1	Achnanthes linearis	Cymbella kappi	48	0.02	0.05	0.01	0.05
0	0	0.21	0	t	1	Cymbella mulleri	Cymbella kappii	48	0.01	0.05	0.01	0.05
0	0	0.26	0	t	1	Pinnularia spp.	Synedra ulna	48	0.01	0.03	0.01	0.05
0	0	0.18	0	cv	1	Gomphonema intricatum	Terrestrial invertebrates	48	0.01	0.04	0.01	0.05
0	0	0.36	0	t	1	Sphaerid	Synedra ulna	48	0.02	0.03	0.01	0.05
0	1	0.75	0	t	1	Sphaerid	Navicula avenacea	48	0.02	0.03	0.05	0.05
0	0	0.37	0	t	1	Rhoicosphenia curvata	Zelandotipula	48	0.02	0.05	0.02	0.05
0	0	0.19	0	cv	1	Oligo Lumbri pink	Eye forward chiron	48	0.01	0.05	0.01	0.06
1	1	0.65	0	t	1	Paracalliope pale	Zelandoperla	48	0.01	0.05	0.06	0.05
0	1	0.69	0	t	1	Ameletopsis perscitus	Zelandoperla	48	0.01	0.05	0.06	0.05
0	0	0.19	0	t	1	Paracalliope pale	Scirtid	48	0.01	0.05	0.01	0.06
0	0	0.20	0	cv	1	Oligo II	Helicopsyche	48	0.01	0.06	0.01	0.05

0	0	0.42	0	t	1	Anguilla dieffenbachii	Nitzschia linearis	48	0.01	0.05	0.03	0.04
0	1	0.68	0	t	1	Deleatidium	Stictocladius	48	0.07	0.05	0.01	0.06
0	0	0.47	0	t	1	Aoteapsyche	Green algae	48	0.03	0.06	0.02	0.05
0	0	0.42	0	cv	1	Achnanthes inflata	Gomphonem a parvulum	48	0.03	0.05	0.01	0.04
0	0	0.37	0	t	1	Sphaerid	Batrachosper mum	48	0.02	0.03	0.01	0.05
1	0	0.15	0	t	1	Costachorema	Nitzschia dissipata	48	0.01	0.05	0.01	0.05
1	0	0.39	0	t	1	Chironomini	Gomphonem a sp. III	48	0.02	0.05	0.02	0.04
0	0	0.40	0	cv	1	Chironomini	Gomphonem a angustatum	48	0.02	0.05	0.02	0.05
1	1	0.64	0	t	1	Hydrobiosella stenocerca	Navicula avenacea	48	0.02	0.05	0.05	0.05
0	1	0.59	0	t	1	Anguilla dieffenbachii	Blue-green algae	48	0.01	0.05	0.05	0.05
1	1	0.55	0	t	1	Paracalliope fluviatalus	Gomphonem a sp. III	48	0.04	0.05	0.02	0.04
0	0	0.21	0	cv	1	Synedra ulna	Gomphoneis herculeana	48	0.01	0.05	0.01	0.05
0	1	0.88	0	t	1	Plant materials	Unidentified detritus	48	0.01	0.05	0.08	0.05
0	1	0.85	0	t	1	Meiofauna	Green algae	48	0.07	0.06	0.02	0.05
0	0	0.37	0	t	1	Rhoicosphenia curvata	Gomphonem a sp. III	48	0.02	0.05	0.02	0.04
0	0	0.42	0	cv	1	Calothrix	Paracalliope fluviatalus	48	0.01	0.05	0.04	0.05
0	1	0.68	0	t	1	Nitzschia linearis	Diatoma heimale	48	0.03	0.04	0.02	0.02
0	0	0.36	0	t	1	Gyrosigma	Hydora nitida (l)	48	0.02	0.04	0.01	0.05
0	0	0.20	0	t	1	Paracalliope pale	Cymbella mulleri	48	0.01	0.05	0.01	0.05
0	0	0.34	0	cv	1	Aoteapsyche	Gomphoneis herculeana	48	0.03	0.06	0.01	0.05
0	0	0.16	0	t	1	Cymbella kappi	Terrestrial invertebrates	48	0.01	0.05	0.01	0.05
0	1	0.52	0	t	1	Ulothrix	Coloburiscus	48	0.02	0.05	0.03	0.05
0	1	0.51	0	t	1	Oligo II	Cocconeis placentula	48	0.01	0.06	0.04	0.05
0	1	0.67	0	cv	1	Hudsonema aliena	Eye forward chiron	48	0.06	0.05	0.01	0.06
0	1	0.81	0	t	1	Unidentified detritus	Cymbella kappi	48	0.08	0.05	0.01	0.05
0	0	0.30	0	t	1	Gomphonema intricatum	Green algae	48	0.01	0.04	0.02	0.05
0	0	0.38	0	t	1	Paracalliope fluviatalus	Oligo Lumbri pink	48	0.04	0.05	0.01	0.05

1	1	0.75	0	cv	1	Paralimnophila skuseii	Navicula avenacea	48	0.03	0.06	0.05	0.05
0	0	0.18	0	t	1	Costachorema	Staurostratum	48	0.01	0.05	0.01	0.05
0	0	0.28	0	t	1	Stenoperla prasinia	Epithemia sorex	48	0.02	0.05	0.01	0.05
0	0	0.29	0	t	1	Frustulia rhomboides	Staurostratum	48	0.02	0.05	0.01	0.05
0	0	0.37	0	cv	1	Gyrosigma	Gomphonema parvulum	48	0.02	0.04	0.01	0.04
0	0	0.44	0	t	1	Cricotopus sp II	Hydrobiosella stenocerca	48	0.03	0.05	0.02	0.05
0	1	0.52	0	t	1	Navicula avenacea	Cymbella kappii	48	0.05	0.05	0.01	0.05
0	0	0.15	0	t	1	Paracalliope pale	Calothrix	48	0.01	0.05	0.01	0.05
0	0	0.13	0	cv	1	Paracalliope pale	Nannochorista phillpotti	48	0.01	0.05	0.01	0.06
0	0	0.27	0	t	1	Terrestrial invertebrates	Gomphonema angustatum	48	0.01	0.05	0.02	0.05
0	0	0.33	0	t	1	Fragilaria vaucheriae	Gomphoneis herculeana	48	0.02	0.05	0.01	0.05
0	0	0.46	0	t	1	Gomphonema truncatum	Cristaperla	48	0.03	0.05	0.01	0.05
0	1	0.81	0	cv	1	Deleatidium	Ankitodesmus sp.	48	0.07	0.05	0.02	0.05
0	0	0.22	0	t	1	Pinnularia spp.	Cymbella kappii	48	0.01	0.03	0.01	0.05
0	0	0.45	0	t	1	Hydora nitida	Coloburiscus	48	0.01	0.05	0.03	0.05
1	0	0.32	0	t	1	Polypedellum	Melosira varians	48	0.03	0.05	0.01	0.05
0	1	0.84	0	cv	1	Cocconeis placentula	Navicula avenacea	48	0.04	0.05	0.05	0.05
0	0	0.39	0	t	1	Diatoma heimale	Nitzschia dissipata	48	0.02	0.02	0.01	0.05
0	0	0.15	0	t	1	Costachorema	Epithemia sorex	48	0.01	0.05	0.01	0.05
0	1	0.72	0	t	1	Diatoma heimale	Hydrobiosis parumbripennis	48	0.02	0.02	0.04	0.05
0	0	0.16	0	cv	1	Mischoderus	Cymbella kappii	48	0.01	0.05	0.01	0.05
1	1	0.53	0	t	1	Paracalliope fluviatalus	Zelandobius	48	0.04	0.05	0.02	0.05
1	0	0.23	0	t	1	Eye forward chiron	Staurostratum	48	0.01	0.06	0.01	0.05
0	1	0.52	0	t	1	Sphaerid	Fragilaria vaucheriae	48	0.02	0.03	0.02	0.05
0	0	0.35	0	cv	1	Rhoicosphenia curvata	Zelandobius	48	0.02	0.05	0.02	0.05

0	1	0.71	0	t	1	Deleatidium	Cymbella kappii	48	0.07	0.05	0.01	0.05
0	0	0.23	0	t	1	Frustulia rhomboides	Costachorem a	48	0.02	0.05	0.01	0.05
0	0	0.36	0	t	1	Cricotopus sp I	Cymbella mulleri	48	0.02	0.05	0.01	0.05
0	0	0.46	1	cv	1	Larsia sp.	Oligochaeta I	49	0.02	0.08	0.04	0.08
0	0	0.18	1	t	1	Larsia sp.	Oligochaeta II	49	0.02	0.08	0.01	0.08
1	1	0.95	1	t	1	Larsia sp.	Unidentified detritus	49	0.02	0.08	0.11	0.07
0	0	0.24	1	t	1	Hydora nitida l	Plant material	49	0.03	0.08	0.01	0.07
1	1	0.96	1	cv	1	Hydora nitida l	Unidentified detritus	49	0.03	0.08	0.11	0.07
0	0	0.44	1	t	1	Hydora nitida l	Cocconeis placentula	49	0.03	0.08	0.03	0.07
1	1	0.73	1	t	1	Hydora nitida l	Stenoperla prasinia	49	0.03	0.08	0.06	0.07
0	0	0.48	1	t	1	Oligochaeta I	Ameletopsis perscitus	49	0.04	0.08	0.02	0.07
0	0	0.33	1	cv	1	Oligochaeta I	Plant material	49	0.04	0.08	0.01	0.07
1	1	0.97	1	t	1	Oligochaeta I	Unidentified detritus	49	0.04	0.08	0.11	0.07
0	0	0.32	1	t	1	Oligochaeta I	Gomphonem a truncatum	49	0.04	0.08	0.01	0.07
0	1	0.52	1	t	1	Oligochaeta I	Hydrobiosella stenocerca	49	0.04	0.08	0.03	0.08
0	0	0.17	1	cv	1	Tabellaria flocculosa	Paracalliope fluviatalus	49	0.01	0.06	0.02	0.08
1	1	0.95	1	t	1	Coloburiscus humeralis	Unidentified detritus	49	0.02	0.08	0.11	0.07
0	1	0.80	1	t	1	Coloburiscus humeralis	Crayfish	49	0.02	0.08	0.07	0.07
1	1	0.69	1	t	1	Coloburiscus humeralis	Stenoperla prasinia	49	0.02	0.08	0.06	0.07
0	1	0.60	1	cv	1	Pinnularia gibba	Oligochaeta lumbr blue	49	0.01	0.07	0.06	0.07
0	1	0.69	1	t	1	Surirella	Zelanoperla	49	0.01	0.06	0.07	0.07
0	1	0.72	1	t	1	Deleatidium	Achnanthes minutissima	49	0.07	0.07	0.02	0.07
1	1	0.85	1	t	1	Deleatidium	Rhoicospheni a curvata	49	0.07	0.07	0.04	0.07
0	1	0.77	1	cv	1	Deleatidium	Pinnularia divergens	49	0.07	0.07	0.02	0.07
1	1	0.64	1	t	1	Deleatidium	Plant material	49	0.07	0.07	0.01	0.07
1	1	0.99	1	t	1	Deleatidium	Unidentified detritus	49	0.07	0.07	0.11	0.07

0	1	0.72	1	t	1	Deleatidium	Eunotia pectinalis	49	0.07	0.07	0.02	0.06
1	1	0.94	1	cv	1	Deleatidium	Achnanthes lanceolata	49	0.07	0.07	0.06	0.07
1	1	0.82	1	t	1	Deleatidium	Cocconeis placentula	49	0.07	0.07	0.03	0.07
1	1	0.97	1	t	1	Deleatidium	Crayfish	49	0.07	0.07	0.07	0.07
0	1	0.64	1	t	1	Deleatidium	Gomphoneis herculeana	49	0.07	0.07	0.01	0.07
1	1	0.94	1	cv	1	Deleatidium	Stenoperla prasinia	49	0.07	0.07	0.06	0.07
0	1	0.77	1	t	1	Deleatidium	Navicula avenacea	49	0.07	0.07	0.02	0.07
0	1	0.77	1	t	1	Deleatidium	Gomphonema angustatum	49	0.07	0.07	0.02	0.06
0	1	0.68	1	t	1	Achnanthes minutissima	Oligochaeta lumbr blue	49	0.02	0.07	0.06	0.07
0	1	0.75	1	cv	1	Achnanthes minutissima	Zelanoperla	49	0.02	0.07	0.07	0.07
0	0	0.20	1	t	1	Ameletopsis perscitus	Oligochaeta II	49	0.02	0.07	0.01	0.08
0	0	0.38	1	t	1	Ameletopsis perscitus	Hydrobiosella stenocerca	49	0.02	0.07	0.03	0.08
0	0	0.26	1	t	1	Ameletopsis perscitus	Austroperla cyrene	49	0.02	0.07	0.02	0.08
0	0	0.40	1	cv	1	Ameletopsis perscitus	Terrestrial invertebrates	49	0.02	0.07	0.03	0.07
0	0	0.29	1	t	1	Rhoicosphenia curvata	Paralimnophila	49	0.04	0.07	0.01	0.08
0	1	0.86	1	t	1	Rhoicosphenia curvata	Zelanoperla	49	0.04	0.07	0.07	0.07
0	1	0.73	1	t	1	Pinnularia viridis	Oligochaeta lumbr blue	49	0.02	0.07	0.06	0.07
1	1	0.91	1	cv	1	Oligochaeta II	Unidentified detritus	49	0.01	0.08	0.11	0.07
0	1	0.68	1	t	1	Oligochaeta II	Crayfish	49	0.01	0.08	0.07	0.07
0	0	0.23	1	t	1	Oligochaeta II	Hydrobiosella stenocerca	49	0.01	0.08	0.03	0.08
0	1	0.55	1	t	1	Oligochaeta II	Stenoperla prasinia	49	0.01	0.08	0.06	0.07
1	1	0.97	1	cv	1	Tanytarsus verspertinus	Unidentified detritus	49	0.04	0.08	0.11	0.07
0	1	0.69	1	t	1	Achnanthes inflata	Zelanoperla	49	0.01	0.06	0.07	0.07
0	0	0.11	1	t	1	Hydora adult	Plant material	49	0.01	0.08	0.01	0.07
1	1	0.90	1	t	1	Hydora adult	Unidentified detritus	49	0.01	0.08	0.11	0.07
0	0	0.25	1	cv	1	Hydora adult	Cocconeis placentula	49	0.01	0.08	0.03	0.07

0	0	0.30	1	t	1	Plant material	Patatanytarsus nr grimmi	49	0.01	0.07	0.04	0.08
0	0	0.16	1	t	1	Plant material	Paracalliope fluviatalus	49	0.01	0.07	0.02	0.08
0	0	0.16	1	t	1	Plant material	Eriopterini	49	0.01	0.07	0.02	0.08
0	0	0.12	1	cv	1	Plant material	Tanytarsus spp.	49	0.01	0.07	0.01	0.08
0	0	0.30	1	t	1	Plant material	Naonella	49	0.01	0.07	0.04	0.08
0	1	0.63	1	t	1	Plant material	Cristaperla	49	0.01	0.07	0.07	0.08
0	1	0.60	1	t	1	Plant material	Oligochaeta lumbr blue	49	0.01	0.07	0.06	0.07
0	1	0.71	1	cv	1	Plant material	Crayfish	49	0.01	0.07	0.07	0.07
0	0	0.12	1	t	1	Plant material	Paralimnophila	49	0.01	0.07	0.01	0.08
0	0	0.20	1	t	1	Plant material	Paucispinigera approximata	49	0.01	0.07	0.02	0.08
1	1	0.98	1	t	1	Stictoclcaius	Unidentified detritus	49	0.05	0.08	0.11	0.07
0	1	0.88	1	cv	1	Unidentified detritus	Forcipomyiinae	49	0.11	0.07	0.01	0.08
1	1	0.96	1	t	1	Unidentified detritus	Patatanytarsus nr grimmi	49	0.11	0.07	0.04	0.08
1	1	0.92	1	t	1	Unidentified detritus	Paracalliope fluviatalus	49	0.11	0.07	0.02	0.08
0	1	0.91	1	t	1	Unidentified detritus	Eriopterini	49	0.11	0.07	0.02	0.08
0	1	0.88	1	cv	1	Unidentified detritus	Tanytarsus spp.	49	0.11	0.07	0.01	0.08
1	1	0.96	1	t	1	Unidentified detritus	Naonella	49	0.11	0.07	0.04	0.08
1	1	0.99	1	t	1	Unidentified detritus	Cristaperla	49	0.11	0.07	0.07	0.08
1	1	0.99	1	t	1	Unidentified detritus	Oligochaeta lumbr blue	49	0.11	0.07	0.06	0.07
0	1	0.99	1	cv	1	Unidentified detritus	Crayfish	49	0.11	0.07	0.07	0.07
1	1	0.95	1	t	1	Unidentified detritus	Polypedellum green type	49	0.11	0.07	0.03	0.08
0	1	0.88	1	t	1	Unidentified detritus	Paralimnophila	49	0.11	0.07	0.01	0.08
0	1	0.95	1	t	1	Unidentified detritus	Hydrobiosella stenocerca	49	0.11	0.07	0.03	0.08
0	1	0.91	1	cv	1	Unidentified detritus	Austroperla cyrene	49	0.11	0.07	0.02	0.08
1	1	0.93	1	t	1	Unidentified detritus	Cricotopus II	49	0.11	0.07	0.02	0.08
0	1	0.91	1	t	1	Unidentified detritus	Eukiefiriella	49	0.11	0.07	0.02	0.08
1	1	0.99	1	t	1	Unidentified detritus	Zelanoperla	49	0.11	0.07	0.07	0.07

1	1	0.93	1	cv	1	Unidentified detritus	Paucispiniger a approximata	49	0.11	0.07	0.02	0.08
0	1	0.63	1	t	1	Calothrix	Cristaperla	49	0.01	0.07	0.07	0.08
0	0	0.26	1	t	1	Melosira varians	Polypedellum green type	49	0.01	0.06	0.03	0.08
0	1	0.75	1	t	1	Eunotia pectinalis	Zelanoperla	49	0.02	0.06	0.07	0.07
0	1	0.67	1	cv	1	Blue green algae	Oligochaeta lumbr blue	49	0.02	0.07	0.06	0.07
0	1	0.77	1	t	1	Blue green algae	Crayfish	49	0.02	0.07	0.07	0.07
0	0	0.32	1	t	1	Blue green algae	Polypedellum green type	49	0.02	0.07	0.03	0.08
1	0	0.42	1	t	1	Paracalliope fluviatalus	Cymbella aspera	49	0.02	0.08	0.04	0.05
0	0	0.32	1	cv	1	Paracalliope fluviatalus	Cocconeis placentula	49	0.02	0.08	0.03	0.07
0	1	0.77	1	t	1	Achnanthes lanceolata	Naonella	49	0.06	0.07	0.04	0.08
0	1	0.73	1	t	1	Achnanthes lanceolata	Polypedellum green type	49	0.06	0.07	0.03	0.08
0	1	0.52	1	t	1	Achnanthes lanceolata	Paralimnophil a	49	0.06	0.07	0.01	0.08
0	1	0.94	1	cv	1	Achnanthes lanceolata	Zelanoperla	49	0.06	0.07	0.07	0.07
0	1	0.51	1	t	1	Cocconeis placentula	Naonella	49	0.03	0.07	0.04	0.08
0	1	0.80	1	t	1	Cocconeis placentula	Cristaperla	49	0.03	0.07	0.07	0.08
0	1	0.78	1	t	1	Cocconeis placentula	Oligochaeta lumbr blue	49	0.03	0.07	0.06	0.07
0	0	0.45	1	cv	1	Cocconeis placentula	Polypedellum green type	49	0.03	0.07	0.03	0.08
0	0	0.31	1	t	1	Cocconeis placentula	Austroperla cyrene	49	0.03	0.07	0.02	0.08
0	1	0.83	1	t	1	Cocconeis placentula	Zelanoperla	49	0.03	0.07	0.07	0.07
1	1	0.93	1	t	1	Cristaperla	Stenoperla prasinia	49	0.07	0.08	0.06	0.07
1	1	0.95	1	cv	1	Oligochaeta lumbr blue	Crayfish	49	0.06	0.07	0.07	0.07
1	1	0.80	1	t	1	Oligochaeta lumbr blue	Surirella striatula	49	0.06	0.07	0.04	0.07
0	1	0.56	1	t	1	Oligochaeta lumbr blue	Cymbella kappi	49	0.06	0.07	0.01	0.07
0	1	0.71	1	t	1	Oligochaeta lumbr blue	Unknown green algal cells	49	0.06	0.07	0.02	0.07
0	1	0.64	1	cv	1	Oligochaeta lumbr blue	Pinnularia sp.	49	0.06	0.07	0.02	0.07

0	1	0.82	1	t	1	Crayfish	Hydrobiosella stenocerca	49	0.07	0.07	0.03	0.08
0	1	0.83	1	t	1	Crayfish	Terrestrial invertebrates	49	0.07	0.07	0.03	0.07
0	1	0.80	1	t	1	Crayfish	Navicula avenacea	49	0.07	0.07	0.02	0.07
0	1	0.74	1	cv	1	Crayfish	Pinnularia sp.	49	0.07	0.07	0.02	0.07
0	0	0.38	1	t	1	Polypedellum green type	Unknown green algal cells	49	0.03	0.08	0.02	0.07
0	0	0.38	1	t	1	Polypedellum green type	Gomphonema angustatum	49	0.03	0.08	0.02	0.06
1	1	0.62	1	t	1	Austroperla cyrene	Stenoperla prasinia	49	0.02	0.08	0.06	0.07
0	0	0.21	1	cv	1	Gomphoneis herculeana	Cricotopus II	49	0.01	0.07	0.02	0.08
0	1	0.75	1	t	1	Stenoperla prasinia	Terrestrial invertebrates	49	0.06	0.07	0.03	0.07
0	1	0.67	1	t	1	Stenoperla prasinia	Cricotopus II	49	0.06	0.07	0.02	0.08
0	1	0.60	1	t	1	Stenoperla prasinia	Eukiefiriella	49	0.06	0.07	0.02	0.08
0	1	0.94	1	cv	1	Stenoperla prasinia	Zelanoperla	49	0.06	0.07	0.07	0.07
0	1	0.82	1	t	1	Terrestrial invertebrates	Zelanoperla	49	0.03	0.07	0.07	0.07
0	1	0.77	1	t	1	Zelanoperla	Navicula avenacea	49	0.07	0.07	0.02	0.07
1	1	0.77	1	t	1	Zelanoperla	Unknown green algal cells	49	0.07	0.07	0.02	0.07
0	1	0.77	1	cv	1	Zelanoperla	Gomphonema angustatum	49	0.07	0.07	0.02	0.06
0	0	0.49	0	t	1	Oligochaeta I	Unknown green algal cells	49	0.04	0.08	0.02	0.07
0	1	0.81	0	t	1	Rhoicosphenia curvata	Stenoperla prasinia	49	0.04	0.07	0.06	0.07
0	1	0.86	0	t	1	Cymbella aspera	Cristaperla	49	0.04	0.05	0.07	0.08
0	0	0.32	0	cv	1	Pinnularia divergens	Cricotopus II	49	0.02	0.07	0.02	0.08
0	0	0.10	0	t	1	Tanytarsus spp.	Paralimnophila	49	0.01	0.08	0.01	0.08
0	0	0.37	0	t	1	Stictoclcaius	Cymbella kappi	49	0.05	0.08	0.01	0.07
0	0	0.19	0	t	1	Larsia sp.	Gomphonema truncatum	49	0.02	0.08	0.01	0.07
0	1	0.54	0	cv	1	Cocconeis placentula	Surirella striatula	49	0.03	0.07	0.04	0.07

1	1	0.85	0	t	1	Deleatidium	Surirella striatula	49	0.07	0.07	0.04	0.07
0	1	0.84	0	t	1	Cymbella aspera	Oligochaeta lumbr blue	49	0.04	0.05	0.06	0.07
0	1	0.64	0	t	1	Tanytarsus verspertinus	Cymbella aspera	49	0.04	0.08	0.04	0.05
0	0	0.25	0	cv	1	Coloburiscus humeralis	Pinnularia sp.	49	0.02	0.08	0.02	0.07
0	0	0.41	0	t	1	Pinnularia divergens	Terrestrial invertebrates	49	0.02	0.07	0.03	0.07
0	0	0.26	0	t	1	Forcipomyiina	Naonella	49	0.01	0.08	0.04	0.08
0	0	0.23	0	t	1	Larsia sp.	Eukiefiriella	49	0.02	0.08	0.02	0.08
0	1	0.88	0	cv	1	Cymbella aspera	Zelanoperla	49	0.04	0.05	0.07	0.07
0	0	0.23	0	t	1	Coloburiscus humeralis	Eukiefiriella	49	0.02	0.08	0.02	0.08
0	1	0.60	0	t	1	Achnanthes lanceolata	Eukiefiriella	49	0.06	0.07	0.02	0.08
0	0	0.32	0	t	1	Cricotopus II	Gomphonema angustatum	49	0.02	0.08	0.02	0.06
0	1	0.65	0	cv	1	Oligochaeta I	Cymbella aspera	49	0.04	0.08	0.04	0.05
0	0	0.19	0	t	1	Gomphonema truncatum	Paucispinigera approximata	49	0.01	0.07	0.02	0.08
0	0	0.37	0	t	1	Rhoicosphenia curvata	Paracalliope fluviatalus	49	0.04	0.07	0.02	0.08
0	0	0.16	0	t	1	Eunotia pectinalis	Tanytarsus spp.	49	0.02	0.06	0.01	0.08
0	1	0.64	0	cv	1	Zelanoperla	Cymbella kappi	49	0.07	0.07	0.01	0.07
0	0	0.23	0	t	1	Pinnularia divergens	Cymbella kappi	49	0.02	0.07	0.01	0.07
1	1	0.65	0	t	1	Stictoclcaius	Surirella striatula	49	0.05	0.08	0.04	0.07
0	0	0.34	0	t	1	Achnanthes inflata	Surirella striatula	49	0.01	0.06	0.04	0.07
0	0	0.30	0	cv	1	Eriopterini	Terrestrial invertebrates	49	0.02	0.08	0.03	0.07
0	1	0.63	0	t	1	Deleatidium	Gomphonema truncatum	49	0.07	0.07	0.01	0.07
0	1	0.67	0	t	1	Calothrix	Zelanoperla	49	0.01	0.07	0.07	0.07
0	0	0.36	0	t	1	Tanytarsus verspertinus	Eriopterini	49	0.04	0.08	0.02	0.08
0	0	0.26	0	cv	1	Ameletopsis perscitus	Paracalliope fluviatalus	49	0.02	0.07	0.02	0.08
0	0	0.38	0	t	1	Patatanytarsus nr grimmi	Paucispinigera approximata	49	0.04	0.08	0.02	0.08
0	1	0.68	0	t	1	Gomphoneis herculeana	Zelanoperla	49	0.01	0.07	0.07	0.07

0	0	0.41	0	t	1	Pinnularia viridis	Terrestrial invertebrates	49	0.02	0.07	0.03	0.07
0	0	0.20	0	cv	1	Tanytarsus spp.	Gomphonema angustatum	49	0.01	0.08	0.02	0.06
0	0	0.28	0	t	1	Plant material	Cocconeis placentula	49	0.01	0.07	0.03	0.07
0	0	0.48	0	t	1	Patatanytarsus nr grimmi	Terrestrial invertebrates	49	0.04	0.08	0.03	0.07
0	1	0.79	0	t	1	Ameletopsis perscitus	Zelanoperla	49	0.02	0.07	0.07	0.07
0	0	0.13	0	cv	1	Calothrix	Gomphonema truncatum	49	0.01	0.07	0.01	0.07
0	1	0.53	0	t	1	Oligochaeta lumbr blue	Paralimnophila	49	0.06	0.07	0.01	0.08
0	0	0.31	0	t	1	Ameletopsis perscitus	Cricotopus II	49	0.02	0.07	0.02	0.08
0	1	0.61	0	t	1	Oligochaeta lumbr blue	Austroperla cyrene	49	0.06	0.07	0.02	0.08
0	0	0.41	0	cv	1	Eunotia pectinalis	Surirella striatula	49	0.02	0.06	0.04	0.07
0	1	0.80	0	t	1	Crayfish	Gomphonema angustatum	49	0.07	0.07	0.02	0.06
0	0	0.31	0	t	1	Hydora nitida l	Achnanthes minutissima	49	0.03	0.08	0.02	0.07
0	0	0.17	0	t	1	Calothrix	Pinnularia sp.	49	0.01	0.07	0.02	0.07
0	1	0.71	0	cv	1	Achnanthes minutissima	Cristaperla	49	0.02	0.07	0.07	0.08
0	0	0.22	0	t	1	Ameletopsis perscitus	Gomphoneis herculeana	49	0.02	0.07	0.01	0.07
0	0	0.22	0	t	1	Achnanthes minutissima	Paracalliope fluviatalus	49	0.02	0.07	0.02	0.08
0	0	0.25	0	t	1	Gomphonema truncatum	Polypedellum green type	49	0.01	0.07	0.03	0.08
0	1	0.95	0	cv	1	Pinnularia divergens	Unidentified detritus	49	0.02	0.07	0.11	0.07
0	0	0.24	0	t	1	Melosira varians	Unknown green algal cells	49	0.01	0.06	0.02	0.07
0	1	0.97	0	t	1	Unidentified detritus	Surirella striatula	49	0.11	0.07	0.04	0.07
0	1	0.99	0	t	1	Unidentified detritus	Achnanthes lanceolata	49	0.11	0.07	0.06	0.07
0	1	0.68	0	cv	1	Plant material	Zelanoperla	49	0.01	0.07	0.07	0.07
0	0	0.35	0	t	1	Pinnularia divergens	Navicula avenacea	49	0.02	0.07	0.02	0.07
0	0	0.18	0	t	1	Oligochaeta II	Cricotopus II	49	0.01	0.08	0.02	0.08
0	0	0.32	0	t	1	Larsia sp.	Pinnularia divergens	49	0.02	0.08	0.02	0.07
0	0	0.21	0	cv	1	Paralimnophila	Gomphonema angustatum	49	0.01	0.08	0.02	0.06

0	0	0.14	0	t	1	Gomphoneis herculeana	Cymbella kappi	49	0.01	0.07	0.01	0.07
0	0	0.18	0	t	1	Achnanthes minutissima	Cymbella kappi	49	0.02	0.07	0.01	0.07
0	1	0.77	0	t	1	Deleatidium	Ameletopsis perscitus	49	0.07	0.07	0.02	0.07
0	0	0.40	0	cv	1	Larsia sp.	Patatanytarsus nr grimmi	49	0.02	0.08	0.04	0.08
0	0	0.17	0	t	1	Achnanthes minutissima	Oligochaeta II	49	0.02	0.07	0.01	0.08
0	0	0.23	0	t	1	Cricotopus II	Eukiefiriella	49	0.02	0.08	0.02	0.08
0	0	0.17	0	t	1	Tabellaria flocculosa	Austroperla cyrene	49	0.01	0.06	0.02	0.08
0	0	0.29	0	cv	1	Naonella	Cymbella kappi	49	0.04	0.08	0.01	0.07
0	1	0.90	0	t	1	Unidentified detritus	Cymbella kappi	49	0.11	0.07	0.01	0.07
0	0	0.40	0	t	1	Tanytarsus verspertinus	Pinnularia sp.	49	0.04	0.08	0.02	0.07
0	0	0.44	0	t	1	Pinnularia	Naonella	49	0.02	0.07	0.04	0.08
0	0	0.32	0	cv	1	Paucispinigera approximata	Unknown green algal cells	49	0.02	0.08	0.02	0.07
1	1	0.74	0	t	1	Cristaperla	Unknown green algal cells	49	0.07	0.08	0.02	0.07
0	1	0.92	0	t	1	Tabellaria flocculosa	Unidentified detritus	49	0.01	0.06	0.11	0.07
0	1	0.80	0	t	1	Achnanthes lanceolata	Surirella striatula	49	0.06	0.07	0.04	0.07
0	0	0.34	0	cv	1	Blue green algae	Terrestrial invertebrates	49	0.02	0.07	0.03	0.07
0	0	0.14	0	t	1	Pinnularia gibba	Melosira varians	49	0.01	0.07	0.01	0.06
0	0	0.21	0	t	1	Paralimnophila	Unknown green algal cells	49	0.01	0.08	0.02	0.07
1	0	0.33	0	t	1	Tanytarsus verspertinus	Melosira varians	49	0.04	0.08	0.01	0.06
0	0	0.37	0	cv	1	Stictoclcaius	Gomphoneis herculeana	49	0.05	0.08	0.01	0.07
0	1	0.57	0	t	1	Hydora nitida l	Stictoclcaius	49	0.03	0.08	0.05	0.08
0	0	0.13	0	t	1	Plant material	Gomphonema truncatum	49	0.01	0.07	0.01	0.07
0	0	0.47	0	t	1	Rhoicosphenia curvata	Navicula avenacea	49	0.04	0.07	0.02	0.07
0	0	0.14	0	cv	1	Plant material	Melosira varians	49	0.01	0.07	0.01	0.06
0	0	0.39	0	t	1	Pinnularia divergens	Polypedellum green type	49	0.02	0.07	0.03	0.08

0	0	0.26	0	t	1	Eriopterini	Gomphonema angustatum	49	0.02	0.08	0.02	0.06
0	0	0.12	0	t	1	Oligochaeta II	Melosira varians	49	0.01	0.08	0.01	0.06
0	0	0.29	0	cv	1	Rhoicosphenia curvata	Hydora adult	49	0.04	0.07	0.01	0.08
0	1	0.72	0	t	1	Pinnularia viridis	Achnanthes lanceolata	49	0.02	0.07	0.06	0.07
0	0	0.20	0	t	1	Tanytarsus spp.	Unknown green algal cells	49	0.01	0.08	0.02	0.07
0	0	0.24	0	t	1	Tabellaria flocculosa	Pinnularia viridis	49	0.01	0.06	0.02	0.07
0	0	0.14	0	cv	1	Pinnularia gibba	Achnanthes inflata	49	0.01	0.07	0.01	0.06
0	0	0.49	0	t	1	Naonella	Terrestrial invertebrates	49	0.04	0.08	0.03	0.07
0	0	0.37	0	t	1	Tanytarsus verspertinus	Eukiefiriella	49	0.04	0.08	0.02	0.08
0	0	0.30	0	t	1	Hydora nitida l	Blue green algae	49	0.03	0.08	0.02	0.07
0	0	0.29	0	cv	1	Oligochaeta I	Tanytarsus spp.	49	0.04	0.08	0.01	0.08
0	0	0.26	0	t	1	Gomphonema truncatum	Terrestrial invertebrates	49	0.01	0.07	0.03	0.07
0	1	0.62	0	t	1	Gomphonema truncatum	Cristaperla	49	0.01	0.07	0.07	0.08
0	1	0.60	0	t	1	Rhoicosphenia curvata	Surirella striatula	49	0.04	0.07	0.04	0.07
0	0	0.26	0	cv	1	Pinnularia	Eukiefiriella	49	0.02	0.07	0.02	0.08
1	1	0.61	0	t	1	Eriopterini	Stenoperla prasinia	49	0.02	0.08	0.06	0.07
0	1	0.71	0	t	1	Deleatidium	Blue green algae	49	0.07	0.07	0.02	0.07
0	0	0.34	0	t	1	Paracalliope fluviatalus	Naonella	49	0.02	0.08	0.04	0.08
0	0	0.27	0	cv	1	Calothrix	Terrestrial invertebrates	49	0.01	0.07	0.03	0.07
1	1	0.63	0	t	1	Paracalliope fluviatalus	Achnanthes lanceolata	49	0.02	0.08	0.06	0.07
0	1	0.68	0	t	1	Melosira varians	Zelanoperla	49	0.01	0.06	0.07	0.07
0	0	0.20	0	t	1	Cymbella kappi	Paucispiniger a approximata	49	0.01	0.07	0.02	0.08
0	1	0.70	0	cv	1	Coloburiscus humeralis	Oligochaeta lumbr blue	49	0.02	0.08	0.06	0.07
0	0	0.33	0	t	1	Naonella	Austroperla cyrene	49	0.04	0.08	0.02	0.08
0	0	0.26	0	t	1	Pinnularia divergens	Eukiefiriella	49	0.02	0.07	0.02	0.08

0	0	0.19	0	t	1	Pinnularia gibba	Eunotia pectinalis	49	0.01	0.07	0.02	0.06
0	0	0.37	0	cv	1	Terrestrial invertebrates	Cricotopus II	49	0.03	0.07	0.02	0.08
0	0	0.13	0	t	1	Gomphonema truncatum	Gomphoneis herculeana	49	0.01	0.07	0.01	0.07
0	1	0.77	0	t	1	Crayfish	Cricotopus II	49	0.07	0.07	0.02	0.08
0	0	0.29	0	t	1	Hydora adult	Surirella striatula	49	0.01	0.08	0.04	0.07
0	0	0.33	0	cv	1	Pinnularia gibba	Surirella striatula	49	0.01	0.07	0.04	0.07
1	1	0.55	0	t	1	Tanytarsus verspertinus	Cocconeis placentula	49	0.04	0.08	0.03	0.07
1	1	0.64	1	t	1	Blue green algae	Stictocladius	50	0.03	0.06	0.05	0.08
1	1	0.52	1	t	1	Blue green algae	Isopoda	50	0.03	0.06	0.04	0.08
1	1	0.64	1	cv	1	Blue green algae	Cristaperla	50	0.03	0.06	0.05	0.08
0	0	0.25	1	t	1	Blue green algae	Paracalliope purple	50	0.03	0.06	0.01	0.08
1	1	0.75	1	t	1	Hydora nitida l	Rhoicospheni a curvata	50	0.02	0.09	0.07	0.06
1	1	0.94	1	t	1	Hydora nitida l	Unidentified detritus	50	0.02	0.09	0.11	0.07
1	0	0.38	1	cv	1	Hydora nitida l	Cocconeis placentula	50	0.02	0.09	0.04	0.06
1	1	0.92	1	t	1	Hydora nitida l	Plant material	50	0.02	0.09	0.10	0.07
1	0	0.20	1	t	1	Hydora nitida l	Hydrobiosis silvicola	50	0.02	0.09	0.02	0.06
1	1	0.87	1	t	1	Deleatidium	Rhoicospheni a curvata	50	0.04	0.08	0.07	0.06
1	1	0.97	1	cv	1	Deleatidium	Unidentified detritus	50	0.04	0.08	0.11	0.07
1	1	0.83	1	t	1	Deleatidium	Galaxias	50	0.04	0.08	0.07	0.06
1	0	0.46	1	t	1	Deleatidium	Achnanthes lanceolata	50	0.04	0.08	0.03	0.06
1	1	0.59	1	t	1	Deleatidium	Cocconeis placentula	50	0.04	0.08	0.04	0.06
1	1	0.96	1	cv	1	Deleatidium	Plant material	50	0.04	0.08	0.10	0.07
0	0	0.48	1	t	1	Deleatidium	Stenoperla prasinia	50	0.04	0.08	0.03	0.07
1	1	0.58	1	t	1	Deleatidium	Unknown green algae	50	0.04	0.08	0.04	0.06
1	1	0.99	1	t	1	Pycnocentria	Unidentified detritus	50	0.05	0.09	0.11	0.07
1	1	0.98	1	cv	1	Pycnocentria	Plant material	50	0.05	0.09	0.10	0.07

1	1	0.94	1	t	1	Hydora nitida a	Unidentified detritus	50	0.02	0.09	0.11	0.07
1	1	0.91	1	t	1	Hydora nitida a	Plant material	50	0.02	0.09	0.10	0.07
1	0	0.31	1	t	1	Calothrix	Isopoda	50	0.01	0.06	0.04	0.08
1	1	0.92	1	cv	1	Scirtid brd	Unidentified detritus	50	0.01	0.09	0.11	0.07
1	1	0.91	1	t	1	Rhoicosphenia curvata	Stictocladius	50	0.07	0.06	0.05	0.08
1	1	0.89	1	t	1	Rhoicosphenia curvata	Nothodixa	50	0.07	0.06	0.04	0.08
0	1	0.89	1	t	1	Rhoicosphenia curvata	Oligo I	50	0.07	0.06	0.04	0.08
1	1	0.83	1	cv	1	Rhoicosphenia curvata	Zelandobius	50	0.07	0.06	0.03	0.09
1	1	0.73	1	t	1	Rhoicosphenia curvata	Eriopterini	50	0.07	0.06	0.02	0.08
1	1	0.78	1	t	1	Rhoicosphenia curvata	Naonella	50	0.07	0.06	0.03	0.09
1	1	0.91	1	t	1	Rhoicosphenia curvata	Cristaperla	50	0.07	0.06	0.05	0.08
1	1	0.66	1	cv	1	Rhoicosphenia curvata	Paracalliope purple	50	0.07	0.06	0.01	0.08
1	1	0.83	1	t	1	Rhoicosphenia curvata	Spaniocerca	50	0.07	0.06	0.03	0.08
1	1	0.73	1	t	1	Rhoicosphenia curvata	Podonomous	50	0.07	0.06	0.02	0.09
1	1	0.83	1	t	1	Rhoicosphenia curvata	Zelandoperla	50	0.07	0.06	0.03	0.08
1	1	0.98	1	cv	1	Stictocladius	Unidentified detritus	50	0.05	0.08	0.11	0.07
1	1	0.56	1	t	1	Stictocladius	Achnanthes lanceolata	50	0.05	0.08	0.03	0.06
1	1	0.97	1	t	1	Stictocladius	Plant material	50	0.05	0.08	0.10	0.07
1	0	0.48	1	t	1	Stictocladius	Navicula avenacea	50	0.05	0.08	0.02	0.06
1	1	0.68	1	cv	1	Stictocladius	Unknown green algae	50	0.05	0.08	0.04	0.06
1	0	0.31	1	t	1	Synedra ulna	Austrosimuliu m	50	0.01	0.06	0.04	0.09
1	1	0.96	1	t	1	Polypedellum	Unidentified detritus	50	0.03	0.09	0.11	0.07
1	1	0.74	1	t	1	Polypedellum	Galaxias	50	0.03	0.09	0.07	0.06
1	1	0.94	1	cv	1	Polypedellum	Plant material	50	0.03	0.09	0.10	0.07
1	0	0.37	1	t	1	Navicula pusio	Nothodixa	50	0.01	0.06	0.04	0.08
1	1	0.92	1	t	1	Psychodid	Unidentified detritus	50	0.01	0.09	0.11	0.07
1	1	0.98	1	t	1	Nothodixa	Unidentified detritus	50	0.04	0.08	0.11	0.07

1	1	0.64	1	cv	1	Nothodixa	Cocconeis placentula	50	0.04	0.08	0.04	0.06
1	1	0.97	1	t	1	Nothodixa	Plant material	50	0.04	0.08	0.10	0.07
1	1	0.63	1	t	1	Nothodixa	Unknown green algae	50	0.04	0.08	0.04	0.06
1	1	0.98	1	t	1	Oligo I	Unidentified detritus	50	0.04	0.08	0.11	0.07
0	0	0.37	1	cv	1	Oligo I	Aotepsyche	50	0.04	0.08	0.02	0.08
1	1	0.86	1	t	1	Oligo I	Galaxias	50	0.04	0.08	0.07	0.06
0	1	0.51	1	t	1	Oligo I	Crayfish	50	0.04	0.08	0.03	0.08
0	1	0.54	1	t	1	Oligo I	Stenoperla prasinia	50	0.04	0.08	0.03	0.07
1	1	0.93	1	cv	1	Unidentified detritus	Aotepsyche	50	0.11	0.07	0.02	0.08
1	1	0.93	1	t	1	Unidentified detritus	Eukiefidrella	50	0.11	0.07	0.02	0.09
1	1	0.93	1	t	1	Unidentified detritus	Aspectrotany pus	50	0.11	0.07	0.02	0.09
1	1	0.90	1	t	1	Unidentified detritus	Cricotopus I	50	0.11	0.07	0.01	0.09
1	1	0.97	1	cv	1	Unidentified detritus	Isopoda	50	0.11	0.07	0.04	0.08
1	1	0.96	1	t	1	Unidentified detritus	Zelandobius	50	0.11	0.07	0.03	0.09
1	1	0.95	1	t	1	Unidentified detritus	Naonella	50	0.11	0.07	0.03	0.09
1	1	0.98	1	t	1	Unidentified detritus	Cristaperla	50	0.11	0.07	0.05	0.08
1	1	0.96	1	cv	1	Unidentified detritus	Crayfish	50	0.11	0.07	0.03	0.08
1	1	0.93	1	t	1	Unidentified detritus	Paralimnophil a	50	0.11	0.07	0.02	0.09
1	1	0.97	1	t	1	Unidentified detritus	Austrosimuliu m	50	0.11	0.07	0.04	0.09
1	1	0.90	1	t	1	Unidentified detritus	Paracalliope purple	50	0.11	0.07	0.01	0.08
1	1	0.90	1	cv	1	Unidentified detritus	Cricotopus II	50	0.11	0.07	0.01	0.09
1	1	0.96	1	t	1	Unidentified detritus	Spaniocerca	50	0.11	0.07	0.03	0.08
1	1	0.95	1	t	1	Unidentified detritus	Paracalliope other	50	0.11	0.07	0.03	0.09
1	1	0.93	1	t	1	Unidentified detritus	Podonomous	50	0.11	0.07	0.02	0.09
1	1	0.98	1	cv	1	Unidentified detritus	Lumbriculiid pink	50	0.11	0.07	0.04	0.08
0	0	0.43	1	t	1	Aotepsyche	Cristaperla	50	0.02	0.08	0.05	0.08
1	1	0.92	1	t	1	Aotepsyche	Plant material	50	0.02	0.08	0.10	0.07

0	0	0.38	1	t	1	Aotepsyche	Lumbriculiid pink	50	0.02	0.08	0.04	0.08
1	1	0.82	1	cv	1	Galaxias	Isopoda	50	0.07	0.06	0.04	0.08
0	1	0.90	1	t	1	Galaxias	Hydrobiosella stenocerca	50	0.07	0.06	0.05	0.07
1	1	0.82	1	t	1	Galaxias	Austrosimulium	50	0.07	0.06	0.04	0.09
1	1	0.58	1	t	1	Galaxias	Paracalliope purple	50	0.07	0.06	0.01	0.08
1	1	0.78	1	cv	1	Galaxias	Spaniocerca	50	0.07	0.06	0.03	0.08
1	1	0.73	1	t	1	Galaxias	Paracalliope other	50	0.07	0.06	0.03	0.09
0	1	0.70	1	t	1	Galaxias	Terrestrial invertebrates	50	0.07	0.06	0.02	0.07
1	1	0.86	1	t	1	Galaxias	Lumbriculiid pink	50	0.07	0.06	0.04	0.08
1	0	0.38	1	cv	1	Eukiefidrella	Unknown green algae	50	0.02	0.09	0.04	0.06
1	1	0.91	1	t	1	Aspectrotanypus	Plant material	50	0.02	0.09	0.10	0.07
1	1	0.96	1	t	1	Isopoda	Plant material	50	0.04	0.08	0.10	0.07
1	1	0.95	1	t	1	Zelandobius	Plant material	50	0.03	0.09	0.10	0.07
1	1	0.52	1	cv	1	Zelandobius	Unknown green algae	50	0.03	0.09	0.04	0.06
1	1	0.92	1	t	1	Eriopterini	Plant material	50	0.02	0.08	0.10	0.07
0	0	0.42	1	t	1	Achnanthes lanceolata	Zelandoperla	50	0.03	0.06	0.03	0.08
0	0	0.39	1	t	1	Cocconeis placentula	Austroperla cyrene	50	0.04	0.06	0.02	0.08
1	1	0.54	1	cv	1	Cocconeis placentula	Zelandoperla	50	0.04	0.06	0.03	0.08
1	0	0.26	1	t	1	Naonella	Navicula avenacea	50	0.03	0.09	0.02	0.06
1	1	0.97	1	t	1	Cristaperla	Plant material	50	0.05	0.08	0.10	0.07
0	1	0.58	1	t	1	Cristaperla	Stenoperla prasinia	50	0.05	0.08	0.03	0.07
1	0	0.47	1	cv	1	Cristaperla	Hydrobiosis silvicola	50	0.05	0.08	0.02	0.06
0	0	0.35	1	t	1	Auodinella	Paracalliope purple	50	0.04	0.05	0.01	0.08
1	1	0.95	1	t	1	Crayfish	Plant material	50	0.03	0.08	0.10	0.07
0	0	0.28	1	t	1	Crayfish	Terrestrial invertebrates	50	0.03	0.08	0.02	0.07
1	1	0.90	1	cv	1	Plant material	Paralimnophila	50	0.10	0.07	0.02	0.09

1	1	0.96	1	t	1	Plant material	Austrosimulium	50	0.10	0.07	0.04	0.09
1	1	0.87	1	t	1	Plant material	Paracalliope purple	50	0.10	0.07	0.01	0.08
1	1	0.90	1	t	1	Plant material	Austroperla cyrene	50	0.10	0.07	0.02	0.08
0	1	0.95	1	cv	1	Plant material	Stenoperla prasinia	50	0.10	0.07	0.03	0.07
1	1	0.94	1	t	1	Plant material	Spaniocerca	50	0.10	0.07	0.03	0.08
1	1	0.92	1	t	1	Plant material	Paracalliope other	50	0.10	0.07	0.03	0.09
1	1	0.97	1	t	1	Plant material	Lumbriculiid pink	50	0.10	0.07	0.04	0.08
1	1	0.94	1	cv	1	Plant material	Zelandoperla	50	0.10	0.07	0.03	0.08
1	0	0.12	1	t	1	Paracalliope purple	Psilachorema	50	0.01	0.08	0.01	0.06
0	1	0.54	1	t	1	Stenoperla prasinia	Lumbriculiid pink	50	0.03	0.07	0.04	0.08
0	0	0.37	1	t	1	Psilachorema	Lumbriculiid pink	50	0.01	0.06	0.04	0.08
0	0	0.42	0	cv	1	Cocconeis placentula	Terrestrial invertebrates	50	0.04	0.06	0.02	0.07
0	0	0.34	0	t	1	Navicula avenacea	Zelandoperla	50	0.02	0.06	0.03	0.08
0	0	0.12	0	t	1	Hydora nitida l	Paracalliope purple	50	0.02	0.09	0.01	0.08
0	1	0.90	0	t	1	Plant material	Psilachorema	50	0.10	0.07	0.01	0.06
1	0	0.21	0	cv	1	Eukiefidrella	Navicula avenacea	50	0.02	0.09	0.02	0.06
0	0	0.30	0	t	1	Isopoda	Podonomous	50	0.04	0.08	0.02	0.09
0	0	0.11	0	t	1	Synedra ulna	Cricotopus I	50	0.01	0.06	0.01	0.09
1	0	0.46	0	t	1	Cocconeis placentula	Paracalliope other	50	0.04	0.06	0.03	0.09
0	0	0.26	0	cv	1	Crayfish	Austroperla cyrene	50	0.03	0.08	0.02	0.08
0	0	0.39	0	t	1	Blue green algae	Polypedellum	50	0.03	0.06	0.03	0.09
0	0	0.46	0	t	1	Cocconeis placentula	Navicula avenacea	50	0.04	0.06	0.02	0.06
0	1	0.50	0	t	1	Lumbriculiid pink	Zelandoperla	50	0.04	0.08	0.03	0.08
1	0	0.42	0	cv	1	Calothrix	Stictocladius	50	0.01	0.06	0.05	0.08
0	1	0.94	0	t	1	Calothrix	Unidentified detritus	50	0.01	0.06	0.11	0.07
0	0	0.20	0	t	1	Synedra ulna	Hydrobiosis silvicola	50	0.01	0.06	0.02	0.06
0	1	0.64	0	t	1	Hydrobiosella stenocerca	Stenoperla prasinia	50	0.05	0.07	0.03	0.07
0	0	0.44	0	cv	1	Deleatidium	Spaniocerca	50	0.04	0.08	0.03	0.08
1	0	0.25	0	t	1	Synedra ulna	Spaniocerca	50	0.01	0.06	0.03	0.08

0	1	0.99	0	t	1	Galaxias	Plant material	50	0.07	0.06	0.10	0.07
0	0	0.15	0	t	1	Cricotopus I	Paracalliope other	50	0.01	0.09	0.03	0.09
0	0	0.20	0	cv	1	Synedra ulna	Naonella	50	0.01	0.06	0.03	0.09
0	0	0.36	0	t	1	Eukiefidrella	Lumbriculiid pink	50	0.02	0.09	0.04	0.08
0	0	0.33	0	t	1	Cricotopus I	Cristaperla	50	0.01	0.09	0.05	0.08
0	0	0.27	0	t	1	Hydrobiosis silvicola	Paracalliope other	50	0.02	0.06	0.03	0.09
1	0	0.20	0	cv	1	Psychodid	Achnanthes lanceolata	50	0.01	0.09	0.03	0.06
1	1	0.72	0	t	1	Rhoicosphenia curvata	Eukiefidrella	50	0.07	0.06	0.02	0.09
0	0	0.31	0	t	1	Stictocladius	Cricotopus II	50	0.05	0.08	0.01	0.09
1	0	0.44	0	t	1	Stictocladius	Terrestrial invertebrates	50	0.05	0.08	0.02	0.07
0	0	0.15	0	cv	1	Hydora nitida	Eukiefidrella	50	0.02	0.09	0.02	0.09
0	0	0.43	0	t	1	Auodinella	Podonomous	50	0.04	0.05	0.02	0.09
0	0	0.27	0	t	1	Aotepsyche	Zelandobius	50	0.02	0.08	0.03	0.09
1	0	0.22	0	t	1	Aotepsyche	Navicula avenacea	50	0.02	0.08	0.02	0.06
0	0	0.28	0	cv	1	Aotepsyche	Zelandoperla	50	0.02	0.08	0.03	0.08
0	1	0.94	0	t	1	Synedra ulna	Unidentified detritus	50	0.01	0.06	0.11	0.07
0	1	0.50	0	t	1	Oligo I	Zelandobius	50	0.04	0.08	0.03	0.09
0	1	0.56	0	t	1	Nothodixa	Isopoda	50	0.04	0.08	0.04	0.08
0	0	0.39	0	cv	1	Stictocladius	Aspectrotany pus	50	0.05	0.08	0.02	0.09
1	1	0.88	0	t	1	Psychodid	Plant material	50	0.01	0.09	0.10	0.07
0	0	0.38	0	t	1	Zelandobius	Zelandoperla	50	0.03	0.09	0.03	0.08
0	0	0.38	0	t	1	Unknown green algae	Podonomous	50	0.04	0.06	0.02	0.09
0	0	0.30	0	cv	1	Hydora nitida	Isopoda	50	0.02	0.09	0.04	0.08
0	0	0.36	0	t	1	Nothodixa	Aotepsyche	50	0.04	0.08	0.02	0.08
0	0	0.21	0	t	1	Psilachorema	Navicula avenacea	50	0.01	0.06	0.02	0.06
0	0	0.17	0	t	1	Navicula pusio	Eriopterini	50	0.01	0.06	0.02	0.08
1	0	0.38	0	cv	1	Deleatidium	Navicula avenacea	50	0.04	0.08	0.02	0.06
0	0	0.27	0	t	1	Stenoperla prasinia	Psilachorema	50	0.03	0.07	0.01	0.06
0	0	0.28	0	t	1	Cricotopus II	Lumbriculiid pink	50	0.01	0.09	0.04	0.08
0	0	0.33	0	t	1	Navicula avenacea	Spaniocerca	50	0.02	0.06	0.03	0.08
0	0	0.11	0	cv	1	Paralimnophil	Cricotopus II	50	0.02	0.09	0.01	0.09
0	0	0.15	0	t	1	Hydora nitida a	Paralimnophil a	50	0.02	0.09	0.02	0.09

0	0	0.17	0	t	1	Aotepsyche	Paralimnophila	50	0.02	0.08	0.02	0.09
1	1	0.66	0	t	1	Galaxias	Paralimnophila	50	0.07	0.06	0.02	0.09
1	0	0.45	0	cv	1	Polypedellum	Cocconeis placentula	50	0.03	0.09	0.04	0.06
0	0	0.44	0	t	1	Crayfish	Austrosimulium	50	0.03	0.08	0.04	0.09
0	0	0.11	0	t	1	Hydora nitida l	Cricotopus I	50	0.02	0.09	0.01	0.09
0	0	0.28	0	t	1	Achnanthes lanceolata	Austroperla cyrene	50	0.03	0.06	0.02	0.08
1	1	0.88	0	cv	1	Galaxias	Cristaperla	50	0.07	0.06	0.05	0.08
0	1	0.72	0	t	1	Rhoicosphenia curvata	Psilachorema	50	0.07	0.06	0.01	0.06
0	1	0.70	0	t	1	Pycnocentria	Nothodixa	50	0.05	0.09	0.04	0.08
0	0	0.33	0	t	1	Blue green algae	Aotepsyche	50	0.03	0.06	0.02	0.08
0	0	0.29	0	cv	1	Calothrix	Stenoperla prasinia	50	0.01	0.06	0.03	0.07
0	0	0.20	0	t	1	Aspectrotanypus	Naonella	50	0.02	0.09	0.03	0.09
0	1	0.65	0	t	1	Galaxias	Psilachorema	50	0.07	0.06	0.01	0.06
0	0	0.20	0	t	1	Polypedellum	Aspectrotanypus	50	0.03	0.09	0.02	0.09
0	0	0.26	0	cv	1	Paralimnophil	Zelandoperla	50	0.02	0.09	0.03	0.08
0	0	0.41	0	t	1	Hydora nitida l	Stictocladius	50	0.02	0.09	0.05	0.08
0	0	0.21	0	t	1	Synedra ulna	Navicula avenacea	50	0.01	0.06	0.02	0.06
0	0	0.32	0	t	1	Deleatidium	Aotepsyche	50	0.04	0.08	0.02	0.08
0	0	0.18	0	cv	1	Hydora nitida a	Terrestrial invertebrates	50	0.02	0.09	0.02	0.07
1	1	0.58	0	t	1	Austrosimulium	Unknown green algae	50	0.04	0.09	0.04	0.06
0	1	0.66	0	t	1	Stictocladius	Nothodixa	50	0.05	0.08	0.04	0.08
0	0	0.24	0	t	1	Navicula avenacea	Terrestrial invertebrates	50	0.02	0.06	0.02	0.07
0	0	0.39	0	cv	1	Blue green algae	Hydrobiosis silvicola	50	0.03	0.06	0.02	0.06
0	1	0.85	0	t	1	Rhoicosphenia curvata	Stenoperla prasinia	50	0.07	0.06	0.03	0.07
0	0	0.34	0	t	1	Achnanthes lanceolata	Hydrobiosis silvicola	50	0.03	0.06	0.02	0.06
0	1	0.55	0	t	1	Cristaperla	Zelandoperla	50	0.05	0.08	0.03	0.08
1	0	0.39	0	cv	1	Zelandobius	Achnanthes lanceolata	50	0.03	0.09	0.03	0.06
1	0	0.28	0	t	1	Aotepsyche	Achnanthes lanceolata	50	0.02	0.08	0.03	0.06
0	0	0.36	0	t	1	Podonomous	Lumbriculiid pink	50	0.02	0.09	0.04	0.08
0	0	0.27	0	t	1	Nothodixa	Cricotopus II	50	0.04	0.08	0.01	0.09
0	0	0.11	0	cv	1	Hydora nitida	Cricotopus II	50	0.02	0.09	0.01	0.09

0	0	0.12	0	t	1	Scirtid brd	Austroperla cyrene	50	0.01	0.09	0.02	0.08
0	0	0.12	0	t	1	Cricotopus I	Austroperla cyrene	50	0.01	0.09	0.02	0.08
0	0	0.25	0	t	1	Hydora nitida	Crayfish	50	0.02	0.09	0.03	0.08
0	0	0.11	0	cv	1	Hydora nitida	Psychodid	50	0.02	0.09	0.01	0.09
1	1	0.74	0	t	1	Hydora nitida a	Rhoicospheni a curvata	50	0.02	0.09	0.07	0.06
0	0	0.27	0	t	1	Calothrix	Achnanthes lanceolata	50	0.01	0.06	0.03	0.06
0	0	0.46	0	t	1	Hydrobiosella stenocerca	Eriopterini	50	0.05	0.07	0.02	0.08
1	1	0.72	0	cv	1	Stictocladius	Auodinella	50	0.05	0.08	0.04	0.05
0	0	0.19	0	t	1	Psychodid	Crayfish	50	0.01	0.09	0.03	0.08
0	1	0.51	0	t	1	Auodinella	Navicula avenacea	50	0.04	0.05	0.02	0.06
0	1	0.97	0	t	1	Plant material	Unknown green algae	50	0.10	0.07	0.04	0.06
0	0	0.28	0	cv	1	Cricotopus I	Lumbriculiid pink	50	0.01	0.09	0.04	0.08
0	0	0.22	0	t	1	Psychodid	Stenoperla prasinia	50	0.01	0.09	0.03	0.07
0	0	0.29	0	t	1	Eriopterini	Stenoperla prasinia	50	0.02	0.08	0.03	0.07
0	0	0.25	0	t	1	Hydora nitida	Zelandobius	50	0.02	0.09	0.03	0.09
0	0	0.09	0	cv	1	Paracalliope purple	Cricotopus II	50	0.01	0.08	0.01	0.09
0	1	0.56	0	t	1	Isopoda	Lumbriculiid pink	50	0.04	0.08	0.04	0.08
1	0	0.15	0	t	1	Hydora nitida	Synedra ulna	50	0.02	0.09	0.01	0.06
0	0	0.18	0	t	1	Psilachorema	Terrestrial invertebrates	50	0.01	0.06	0.02	0.07
0	1	0.95	0	cv	1	Unidentified detritus	Hydrobiosis silvicola	50	0.11	0.07	0.02	0.06
0	1	0.53	0	t	1	Hydrobiosella stenocerca	Naonella	50	0.05	0.07	0.03	0.09
0	0	0.15	0	t	1	Synedra ulna	Psilachorema	50	0.01	0.06	0.01	0.06
0	0	0.37	0	t	1	Cocconeis placentula	Psilachorema	50	0.04	0.06	0.01	0.06
0	1	0.59	0	cv	1	Achnanthes lanceolata	Cristaperla	50	0.03	0.06	0.05	0.08
0	0	0.29	0	t	1	Scirtid brd	Oligo I	50	0.01	0.09	0.04	0.08
1	0	0.23	0	t	1	Polypedellum	Terrestrial invertebrates	50	0.03	0.09	0.02	0.07
0	0	0.37	0	t	1	Polypedellum	Austrosimuliu m	50	0.03	0.09	0.04	0.09
1	1	0.69	0	cv	1	Oligo I	Auodinella	50	0.04	0.08	0.04	0.05
1	0	0.44	0	t	1	Hydrobiosella stenocerca	Psilachorema	50	0.05	0.07	0.01	0.06
0	0	0.27	1	t	1	Brundin	Unidentified detritus	51	0.01	0.04	0.01	0.04

0	1	0.53	1	t	1	Hydora nitida l	Surirella elegans	51	0.03	0.04	0.01	0.04
0	1	0.66	1	cv	1	Hydora nitida l	Rhoicospheni a curvata	51	0.03	0.04	0.03	0.04
0	1	0.52	1	t	1	Hydora nitida l	Coccoenis placentula	51	0.03	0.04	0.01	0.04
0	1	0.52	1	t	1	Hydora nitida l	Unidentified detritus	51	0.03	0.04	0.01	0.04
1	1	0.76	1	t	1	Hydora nitida l	Ulothrix	51	0.03	0.04	0.04	0.04
0	1	0.53	1	cv	1	Hydora nitida l	Gomphonem a angustatum	51	0.03	0.04	0.01	0.04
1	1	0.81	1	t	1	Hydora nitida l	Plant material	51	0.03	0.04	0.04	0.04
0	1	0.69	1	t	1	Hydora nitida l	Achnanthes lanceolata	51	0.03	0.04	0.03	0.04
1	1	0.85	1	t	1	Hydora nitida l	Navicula avenacea	51	0.03	0.04	0.05	0.04
1	1	0.61	1	cv	1	Tabellaria flocculosa	Aphrophila neozelandiae	51	0.01	0.04	0.05	0.04
0	0	0.43	1	t	1	Ceratopogonid	Unidentified detritus	51	0.02	0.03	0.01	0.04
0	0	0.35	1	t	1	Coloburiscus humeralis	Gomphonem a truncatum	51	0.01	0.04	0.02	0.04
0	0	0.46	1	t	1	Coloburiscus humeralis	Rhoicospheni a curvata	51	0.01	0.04	0.03	0.04
0	0	0.33	1	cv	1	Coloburiscus humeralis	Coccoenis placentula	51	0.01	0.04	0.01	0.04
0	0	0.33	1	t	1	Coloburiscus humeralis	Unidentified detritus	51	0.01	0.04	0.01	0.04
0	1	0.72	1	t	1	Coloburiscus humeralis	Navicula avenacea	51	0.01	0.04	0.05	0.04
0	0	0.27	1	t	1	Gomphonema truncatum	Eukiefidrella	51	0.02	0.04	0.01	0.04
1	1	0.83	1	cv	1	Gomphonema truncatum	Pirara matakiri	51	0.02	0.04	0.06	0.04
1	1	0.57	1	t	1	Surirella	Scirtid skinny	51	0.01	0.04	0.04	0.04
1	1	0.59	1	t	1	Surirella elegans	Zelandoperla agnetis	51	0.01	0.04	0.04	0.04
1	1	0.69	1	t	1	Surirella elegans	Aphrophila neozelandiae	51	0.01	0.04	0.05	0.04
0	0	0.19	1	cv	1	Stauroneis vacheriae	Eukiefidrella	51	0.01	0.04	0.01	0.04
1	1	0.75	1	t	1	Stauroneis vacheriae	Pirara matakiri	51	0.01	0.04	0.06	0.04
1	0	0.49	1	t	1	Stauroneis vacheriae	Zelandoperla agnetis	51	0.01	0.04	0.04	0.04
0	0	0.49	1	t	1	Nitzschia palea	Deleatidium lilli	51	0.01	0.05	0.04	0.05
0	0	0.48	1	cv	1	Hudsonema aliena	Rhoicospheni a curvata	51	0.01	0.04	0.03	0.04

0	0	0.34	1	t	1	Hudsonema aliena	Unidentified detritus	51	0.01	0.04	0.01	0.04
0	1	0.52	1	t	1	Orchymontia	Unidentified detritus	51	0.03	0.04	0.01	0.04
1	1	0.76	1	t	1	Orchymontia	Ulothrix	51	0.03	0.04	0.04	0.04
0	1	0.69	1	cv	1	Orchymontia	Achnanthes lanceolata	51	0.03	0.04	0.03	0.04
0	0	0.43	1	t	1	Austrosimulium australense	Rhoicosphenia curvata	51	0.01	0.04	0.03	0.04
0	0	0.30	1	t	1	Austrosimulium australense	Coccoenis placentula	51	0.01	0.04	0.01	0.04
0	0	0.42	1	t	1	Austrosimulium australense	Frustulia rhomboides	51	0.01	0.04	0.03	0.04
0	0	0.30	1	cv	1	Austrosimulium australense	Unidentified detritus	51	0.01	0.04	0.01	0.04
0	0	0.30	1	t	1	Austrosimulium australense	Calothrix	51	0.01	0.04	0.01	0.04
0	1	0.55	1	t	1	Austrosimulium australense	Ulothrix	51	0.01	0.04	0.04	0.04
0	1	0.54	1	t	1	Austrosimulium australense	Diatoma elongatum	51	0.01	0.04	0.04	0.04
0	0	0.30	1	cv	1	Austrosimulium australense	Gomphonema angustatum	51	0.01	0.04	0.01	0.04
0	1	0.69	1	t	1	Austrosimulium australense	Navicula avenacea	51	0.01	0.04	0.05	0.04
0	0	0.42	1	t	1	Stigeoclonium	Oeconesus	51	0.02	0.04	0.01	0.04
0	1	0.65	1	t	1	Stigeoclonium	Pycnocentrodes	51	0.02	0.04	0.04	0.04
1	1	0.87	1	cv	1	Paralimnophila skuseii	Oligo skinny	51	0.06	0.04	0.03	0.04
1	1	0.87	1	t	1	Paralimnophila skuseii	Frustulia rhomboides	51	0.06	0.04	0.03	0.04
1	1	0.79	1	t	1	Paralimnophila skuseii	Unidentified detritus	51	0.06	0.04	0.01	0.04
1	1	0.92	1	t	1	Paralimnophila skuseii	Ulothrix	51	0.06	0.04	0.04	0.04
1	1	0.78	1	cv	1	Paralimnophila skuseii	Unknown green algae spp.	51	0.06	0.04	0.01	0.04
1	1	0.66	1	t	1	Rhoicosphenia curvata	Eriopterini	51	0.03	0.04	0.03	0.04
0	1	0.69	1	t	1	Rhoicosphenia curvata	Scirtid skinny	51	0.03	0.04	0.04	0.04
1	1	0.72	1	t	1	Rhoicosphenia curvata	Phreatoicus prasinia	51	0.03	0.04	0.04	0.04
0	0	0.37	1	cv	1	Rhoicosphenia curvata	Eukiefidrella	51	0.03	0.04	0.01	0.04
0	1	0.88	1	t	1	Rhoicosphenia curvata	Pirara matakiri	51	0.03	0.04	0.06	0.04
0	1	0.58	1	t	1	Rhoicosphenia curvata	Paracalliope fluviatalus	51	0.03	0.04	0.02	0.04

0	0	0.46	1	t	1	Rhoicosphenia curvata	Stictocladius	51	0.03	0.04	0.01	0.04
0	0	0.37	1	cv	1	Rhoicosphenia curvata	Hydrophiliid beetle	51	0.03	0.04	0.01	0.04
0	0	0.50	1	t	1	Rhoicosphenia curvata	Stratiomyid I	51	0.03	0.04	0.02	0.04
1	1	0.71	1	t	1	Rhoicosphenia curvata	Zelandoperla agnetis	51	0.03	0.04	0.04	0.04
0	0	0.46	1	t	1	Rhoicosphenia curvata	Oeconesus	51	0.03	0.04	0.01	0.04
0	1	0.53	1	cv	1	Rhoicosphenia curvata	Austroperla cyrene	51	0.03	0.04	0.02	0.04
0	0	0.42	1	t	1	Rhoicosphenia curvata	Naonella	51	0.03	0.04	0.01	0.04
0	0	0.37	1	t	1	Rhoicosphenia curvata	Heliodidae beetle	51	0.03	0.04	0.01	0.04
1	1	0.79	1	t	1	Rhoicosphenia curvata	Aphrophila neozelandiae	51	0.03	0.04	0.05	0.04
1	1	0.69	1	cv	1	Rhoicosphenia curvata	Pycnocentrodes	51	0.03	0.04	0.04	0.04
0	1	0.70	1	t	1	Rhoicosphenia curvata	Deleatidium lilli	51	0.03	0.04	0.04	0.05
0	0	0.47	1	t	1	Rhoicosphenia curvata	Ameletopsis perscitus	51	0.03	0.04	0.01	0.04
0	0	0.46	1	t	1	Rhoicosphenia curvata	Paucispinigera approximata	51	0.03	0.04	0.01	0.04
0	1	0.53	1	cv	1	Coccoenis placentula	Eriopterini	51	0.01	0.04	0.03	0.04
0	0	0.34	1	t	1	Coccoenis placentula	Stictocladius	51	0.01	0.04	0.01	0.04
0	1	0.69	1	t	1	Coccoenis placentula	Aphrophila neozelandiae	51	0.01	0.04	0.05	0.04
0	0	0.33	1	t	1	Coccoenis placentula	Paucispinigera approximata	51	0.01	0.04	0.01	0.04
0	0	0.49	1	cv	1	Oligo skinny	Gomphoneis herculeana var. robusta	51	0.03	0.04	0.02	0.04
0	0	0.46	1	t	1	Oligo skinny	Unidentified detritus	51	0.03	0.04	0.01	0.04
1	1	0.69	1	t	1	Oligo skinny	Costachorema xanthoptera	51	0.03	0.04	0.04	0.04
1	1	0.71	1	t	1	Oligo skinny	Ulothrix	51	0.03	0.04	0.04	0.04
1	1	0.77	1	cv	1	Oligo skinny	Plant material	51	0.03	0.04	0.04	0.04
0	0	0.45	1	t	1	Oligo skinny	Hydrobiosella stenocerca	51	0.03	0.04	0.01	0.04
0	0	0.42	1	t	1	Oligo skinny	Polyplectropus puerilis	51	0.03	0.04	0.01	0.04

1	1	0.79	1	t	1	Oligo skinny	Aphrophila neozelandiae	51	0.03	0.04	0.05	0.04
0	0	0.49	1	cv	1	Oligo skinny	Unknown blue green algae	51	0.03	0.04	0.02	0.04
0	0	0.42	1	t	1	Oligo skinny	Salmo trutta	51	0.03	0.04	0.01	0.04
0	1	0.51	1	t	1	Oligo skinny	Nannochorista philpotti	51	0.03	0.04	0.02	0.04
1	1	0.82	1	t	1	Oligo skinny	Navicula avenacea	51	0.03	0.04	0.05	0.04
1	1	0.57	1	cv	1	Synedra ulna	Scirtid skinny	51	0.01	0.04	0.04	0.04
1	1	0.60	1	t	1	Synedra ulna	Phreatoicus prasinia	51	0.01	0.04	0.04	0.04
0	0	0.34	1	t	1	Synedra ulna	Stictocladius	51	0.01	0.04	0.01	0.04
0	0	0.26	1	t	1	Synedra ulna	Hydrophiliid beetle	51	0.01	0.04	0.01	0.04
0	0	0.41	1	cv	1	Synedra ulna	Austroperla cyrene	51	0.01	0.04	0.02	0.04
0	0	0.30	1	t	1	Synedra ulna	Naonella	51	0.01	0.04	0.01	0.04
1	1	0.69	1	t	1	Synedra ulna	Aphrophila neozelandiae	51	0.01	0.04	0.05	0.04
0	0	0.24	1	t	1	Pinnularia	Eukiefidrella	51	0.01	0.04	0.01	0.04
1	1	0.80	1	cv	1	Pinnularia viridis	Pirara matakiri	51	0.01	0.04	0.06	0.04
0	0	0.24	1	t	1	Pinnularia viridis	Hydrophiliid beetle	51	0.01	0.04	0.01	0.04
0	1	0.54	1	t	1	Gomphonema accuminatum	Eriopterini	51	0.01	0.04	0.03	0.04
0	0	0.34	1	t	1	Gomphonema accuminatum	Oeconesus	51	0.01	0.04	0.01	0.04
0	1	0.57	1	cv	1	Gomphonema accuminatum	Pycnocentrodes	51	0.01	0.04	0.04	0.04
0	0	0.46	1	t	1	Molophilus	Unidentified detritus	51	0.03	0.04	0.01	0.04
0	1	0.71	1	t	1	Molophilus	Ulothrix	51	0.03	0.04	0.04	0.04
1	1	0.77	1	t	1	Molophilus	Plant material	51	0.03	0.04	0.04	0.04
0	1	0.65	1	cv	1	Eriopterini	Frustulia rhomboides	51	0.03	0.04	0.03	0.04
0	1	0.52	1	t	1	Eriopterini	Unidentified detritus	51	0.03	0.04	0.01	0.04
1	1	0.76	1	t	1	Eriopterini	Ulothrix	51	0.03	0.04	0.04	0.04
0	1	0.52	1	t	1	Eriopterini	Gomphonema angustatum	51	0.03	0.04	0.01	0.04
1	1	0.81	1	cv	1	Eriopterini	Plant material	51	0.03	0.04	0.04	0.04
0	1	0.55	1	t	1	Eriopterini	Unknown blue green algae	51	0.03	0.04	0.02	0.04
1	1	0.85	1	t	1	Eriopterini	Navicula avenacea	51	0.03	0.04	0.05	0.04

0	1	0.62	1	t	1	Gomphoneis herculeana var. robusta	Phreatoicus prasinia	51	0.02	0.04	0.04	0.04
0	0	0.27	1	cv	1	Gomphoneis herculeana var. robusta	Eukiefidrella	51	0.02	0.04	0.01	0.04
1	1	0.83	1	t	1	Gomphoneis herculeana var. robusta	Pirara matakiri	51	0.02	0.04	0.06	0.04
0	0	0.47	1	t	1	Gomphoneis herculeana var. robusta	Paracalliope fluviatalus	51	0.02	0.04	0.02	0.04
0	1	0.61	1	t	1	Gomphoneis herculeana var. robusta	Zelandoperla agnetis	51	0.02	0.04	0.04	0.04
0	0	0.43	1	cv	1	Gomphoneis herculeana var. robusta	Austroperla cyrene	51	0.02	0.04	0.02	0.04
0	0	0.32	1	t	1	Gomphoneis herculeana var. robusta	Naonella	51	0.02	0.04	0.01	0.04
0	1	0.60	1	t	1	Gomphoneis herculeana var. robusta	Deleatidium lilli	51	0.02	0.04	0.04	0.05
1	1	0.70	1	t	1	Frustulia rhomboides	Zelandoperla agnetis	51	0.03	0.04	0.04	0.04
1	1	0.78	1	cv	1	Scirtid skinny	Ulothrix	51	0.04	0.04	0.04	0.04
1	1	0.81	1	t	1	Scirtid skinny	Navicula rhynocephala	51	0.04	0.04	0.04	0.04
0	1	0.56	1	t	1	Scirtid skinny	Gomphonema angustatum	51	0.04	0.04	0.01	0.04
0	1	0.58	1	t	1	Scirtid skinny	Unknown blue green algae	51	0.04	0.04	0.02	0.04
0	0	0.33	1	cv	1	Surirella linearis	Ameletopsis perscitus	51	0.01	0.03	0.01	0.04
0	1	0.94	1	t	1	Eunotia pectinalis	Deleatidium lilli	51	0.08	0.06	0.04	0.05
0	0	0.40	1	t	1	Unidentified detritus	Hexatomini	51	0.01	0.04	0.02	0.04
0	1	0.60	1	t	1	Unidentified detritus	Phreatoicus prasinia	51	0.01	0.04	0.04	0.04
0	0	0.25	1	cv	1	Unidentified detritus	Eukiefidrella	51	0.01	0.04	0.01	0.04
0	0	0.26	1	t	1	Unidentified detritus	Aspectrotanypus	51	0.01	0.04	0.01	0.04
0	1	0.81	1	t	1	Unidentified detritus	Pirara matakiri	51	0.01	0.04	0.06	0.04
0	0	0.45	1	t	1	Unidentified detritus	Paracalliope fluviatalus	51	0.01	0.04	0.02	0.04

0	0	0.34	1	cv	1	Unidentified detritus	Stictocladius	51	0.01	0.04	0.01	0.04
1	1	0.64	1	t	1	Unidentified detritus	Hydrobiosis frater	51	0.01	0.04	0.04	0.04
0	0	0.25	1	t	1	Unidentified detritus	Hydrophiliid beetle	51	0.01	0.04	0.01	0.04
0	0	0.36	1	t	1	Unidentified detritus	Stratiomyid I	51	0.01	0.04	0.02	0.04
1	1	0.59	1	cv	1	Unidentified detritus	Zelandoperla agnetis	51	0.01	0.04	0.04	0.04
0	0	0.34	1	t	1	Unidentified detritus	Oeconesus	51	0.01	0.04	0.01	0.04
0	0	0.40	1	t	1	Unidentified detritus	Austroperla cyrene	51	0.01	0.04	0.02	0.04
0	0	0.30	1	t	1	Unidentified detritus	Naonella	51	0.01	0.04	0.01	0.04
1	1	0.52	1	cv	1	Unidentified detritus	Megaleptoperla diminuata	51	0.01	0.04	0.03	0.03
0	0	0.25	1	t	1	Unidentified detritus	Heliodidae beetle	51	0.01	0.04	0.01	0.04
1	1	0.69	1	t	1	Unidentified detritus	Aphrophila neozelandiae	51	0.01	0.04	0.05	0.04
0	1	0.57	1	t	1	Unidentified detritus	Pycnocentrodes	51	0.01	0.04	0.04	0.04
0	0	0.37	1	cv	1	Unidentified detritus	Nannochorista philpotti	51	0.01	0.04	0.02	0.04
0	1	0.58	1	t	1	Unidentified detritus	Deleatidium lilli	51	0.01	0.04	0.04	0.05
0	0	0.34	1	t	1	Unidentified detritus	Ameletopsis perscitus	51	0.01	0.04	0.01	0.04
0	0	0.33	1	t	1	Unidentified detritus	Paucispinigera approximata	51	0.01	0.04	0.01	0.04
0	0	0.35	1	cv	1	Unidentified detritus	Megaleptoperla grandis	51	0.01	0.04	0.01	0.03
0	1	0.53	1	t	1	Unidentified detritus	Paranephrops zelandicus	51	0.01	0.04	0.03	0.04
0	1	0.66	1	t	1	Hexatomini	Ulothrix	51	0.02	0.04	0.04	0.04
0	1	0.57	1	t	1	Hexatomini	Achnanthes lanceolata	51	0.02	0.04	0.03	0.04
1	1	0.60	1	cv	1	Calothrix	Phreatoicus prasinia	51	0.01	0.04	0.04	0.04
0	0	0.45	1	t	1	Calothrix	Paracalliope fluviatalus	51	0.01	0.04	0.02	0.04
0	0	0.34	1	t	1	Calothrix	Stictocladius	51	0.01	0.04	0.01	0.04
1	1	0.69	1	t	1	Calothrix	Aphrophila neozelandiae	51	0.01	0.04	0.05	0.04
1	1	0.80	1	cv	1	Phreatoicus prasinia	Ulothrix	51	0.04	0.04	0.04	0.04
1	1	0.79	1	t	1	Phreatoicus prasinia	Diatoma elongatum	51	0.04	0.04	0.04	0.04

1	1	0.77	1	t	1	Phreatoicus prasinia	Fragilaria vaucheriae	51	0.04	0.04	0.03	0.04
0	1	0.50	1	t	1	Phreatoicus prasinia	Nitzschia dubia	51	0.04	0.04	0.01	0.04
0	1	0.74	1	cv	1	Phreatoicus prasinia	Achnanthes lanceolata	51	0.04	0.04	0.03	0.04
1	1	0.88	1	t	1	Phreatoicus prasinia	Navicula avenacea	51	0.04	0.04	0.05	0.04
0	0	0.49	1	t	1	Eukiefidrella	Ulothrix	51	0.01	0.04	0.04	0.04
0	1	0.58	1	t	1	Eukiefidrella	Plant material	51	0.01	0.04	0.04	0.04
0	1	0.64	1	cv	1	Eukiefidrella	Navicula avenacea	51	0.01	0.04	0.05	0.04
0	0	0.28	1	t	1	Eukiefidrella	Pinnularia sp. A	51	0.01	0.04	0.02	0.04
0	1	0.52	1	t	1	Costachorema xanthoptera	Naonella	51	0.04	0.04	0.01	0.04
0	1	0.52	1	t	1	Costachorema xanthoptera	Salmo trutta	51	0.04	0.04	0.01	0.04
1	1	0.92	1	cv	1	Pirara matakiri	Ulothrix	51	0.06	0.04	0.04	0.04
1	1	0.94	1	t	1	Pirara matakiri	Plant material	51	0.06	0.04	0.04	0.04
1	1	0.95	1	t	1	Pirara matakiri	Navicula avenacea	51	0.06	0.04	0.05	0.04
1	1	0.81	1	t	1	Pirara matakiri	Pinnularia sp. A	51	0.06	0.04	0.02	0.04
1	1	0.68	1	cv	1	Ulothrix	Paracalliope fluviatalus	51	0.04	0.04	0.02	0.04
0	1	0.58	1	t	1	Ulothrix	Stictocladius	51	0.04	0.04	0.01	0.04
0	0	0.48	1	t	1	Ulothrix	Hydrophiliid beetle	51	0.04	0.04	0.01	0.04
1	1	0.61	1	t	1	Ulothrix	Stratiomyid I	51	0.04	0.04	0.02	0.04
1	1	0.79	1	cv	1	Ulothrix	Zelandoperla agnetis	51	0.04	0.04	0.04	0.04
1	1	0.64	1	t	1	Ulothrix	Austroperla cyrene	51	0.04	0.04	0.02	0.04
0	1	0.53	1	t	1	Ulothrix	Naonella	51	0.04	0.04	0.01	0.04
0	0	0.48	1	t	1	Ulothrix	Heliodidae beetle	51	0.04	0.04	0.01	0.04
1	1	0.86	1	cv	1	Ulothrix	Aphrophila neozelandiae	51	0.04	0.04	0.05	0.04
0	1	0.79	1	t	1	Ulothrix	Deleatidium lilli	51	0.04	0.04	0.04	0.05
1	1	0.58	1	t	1	Ulothrix	Ameletopsis perscitus	51	0.04	0.04	0.01	0.04
0	1	0.68	1	t	1	Paracalliope fluviatalus	Diatoma elongatum	51	0.02	0.04	0.04	0.04
0	1	0.66	1	cv	1	Paracalliope fluviatalus	Fragilaria vaucheriae	51	0.02	0.04	0.03	0.04
0	0	0.36	1	t	1	Paracalliope fluviatalus	Nitzschia dubia	51	0.02	0.04	0.01	0.04

0	0	0.40	1	t	1	Paracalliope fluviatalus	Salmo trutta	51	0.02	0.04	0.01	0.04
0	0	0.37	1	t	1	Paracalliope fluviatalus	Philorheithrus agilis	51	0.02	0.04	0.01	0.03
0	0	0.49	1	cv	1	Paracalliope fluviatalus	Nannochorista philpotti	51	0.02	0.04	0.02	0.04
0	1	0.65	1	t	1	Hydrophiliid beetle	Navicula avenacea	51	0.01	0.04	0.05	0.04
0	0	0.20	1	t	1	Hydrophiliid beetle	Spirogyra	51	0.01	0.04	0.01	0.04
0	1	0.75	1	t	1	Stratiomyid I	Navicula avenacea	51	0.02	0.04	0.05	0.04
0	0	0.38	1	cv	1	Unknown green algae	Austroperla cyrene	51	0.01	0.04	0.02	0.04
0	0	0.24	1	t	1	Unknown green algae	Heliodidae beetle	51	0.01	0.04	0.01	0.04
1	1	0.59	1	t	1	Pinnularia borealis	Oeconesus	51	0.04	0.04	0.01	0.04
1	1	0.86	1	t	1	Pinnularia borealis	Aphrophila neozelandiae	51	0.04	0.04	0.05	0.04
1	1	0.79	1	cv	1	Pinnularia borealis	Pycnocentrodes	51	0.04	0.04	0.04	0.04
0	1	0.58	1	t	1	Zelandoperla agnetis	Gomphonema angustatum	51	0.04	0.04	0.01	0.04
0	1	0.73	1	t	1	Zelandoperla agnetis	Achnanthes lanceolata	51	0.04	0.04	0.03	0.04
0	1	0.54	1	t	1	Zelandoperla agnetis	Salmo trutta	51	0.04	0.04	0.01	0.04
1	1	0.88	1	cv	1	Zelandoperla agnetis	Navicula avenacea	51	0.04	0.04	0.05	0.04
0	1	0.63	1	t	1	Oeconesus	Navicula rhynocephala	51	0.01	0.04	0.04	0.04
0	0	0.34	1	t	1	Oeconesus	Gomphonema angustatum	51	0.01	0.04	0.01	0.04
0	1	0.67	1	t	1	Oeconesus	Plant material	51	0.01	0.04	0.04	0.04
0	0	0.30	1	cv	1	Oeconesus	Navicula placentula	51	0.01	0.04	0.01	0.04
0	0	0.30	1	t	1	Oeconesus	Salmo trutta	51	0.01	0.04	0.01	0.04
0	1	0.61	1	t	1	Oeconesus	Epithemia sorex	51	0.01	0.04	0.04	0.04
0	0	0.40	1	t	1	Austroperla cyrene	Gomphonema angustatum	51	0.02	0.04	0.01	0.04
0	1	0.72	1	cv	1	Austroperla cyrene	Plant material	51	0.02	0.04	0.04	0.04
0	1	0.78	1	t	1	Austroperla cyrene	Navicula avenacea	51	0.02	0.04	0.05	0.04
1	1	0.81	1	t	1	Navicula rhynocephala	Pycnocentrodes	51	0.04	0.04	0.04	0.04
0	0	0.32	1	t	1	Naonella	Stenoperla prasinia	51	0.01	0.04	0.01	0.03

0	0	0.46	1	cv	1	Naonella	Achnanthes lanceolata	51	0.01	0.04	0.03	0.04
0	0	0.24	1	t	1	Naonella	Philorheithrus agilis	51	0.01	0.04	0.01	0.03
0	0	0.34	1	t	1	Naonella	Nannochorista philpotti	51	0.01	0.04	0.02	0.04
0	1	0.69	1	t	1	Naonella	Navicula avenacea	51	0.01	0.04	0.05	0.04
0	0	0.32	1	cv	1	Naonella	Pinnularia sp. A	51	0.01	0.04	0.02	0.04
1	1	0.69	1	t	1	Gomphonema angustatum	Aphrophila neozelandiae	51	0.01	0.04	0.05	0.04
0	1	0.57	1	t	1	Gomphonema angustatum	Pycnocentrodes	51	0.01	0.04	0.04	0.04
0	1	0.50	1	t	1	Megaleptoperla diminuata	Hydrobiosella stenocerca	51	0.03	0.03	0.01	0.04
1	0	0.47	1	cv	1	Megaleptoperla diminuata	Salmo trutta	51	0.03	0.03	0.01	0.04
0	1	0.58	1	t	1	Heliodidae beetle	Plant material	51	0.01	0.04	0.04	0.04
0	0	0.21	1	t	1	Heliodidae beetle	Rhopaloidea musculus	51	0.01	0.04	0.01	0.03
0	1	0.65	1	t	1	Heliodidae beetle	Navicula avenacea	51	0.01	0.04	0.05	0.04
0	0	0.20	1	cv	1	Heliodidae beetle	Spirogyra	51	0.01	0.04	0.01	0.04
1	1	0.89	1	t	1	Plant material	Aphrophila neozelandiae	51	0.04	0.04	0.05	0.04
1	1	0.83	1	t	1	Plant material	Pycnocentrodes	51	0.04	0.04	0.04	0.04
1	1	0.61	1	t	1	Plant material	Salmo trutta	51	0.04	0.04	0.01	0.04
1	1	0.65	1	cv	1	Plant material	Paucispinigera approximata	51	0.04	0.04	0.01	0.04
1	1	0.81	1	t	1	Plant material	Paranephrops zelandicus	51	0.04	0.04	0.03	0.04
0	0	0.32	1	t	1	Stenoperla prasinia	Salmo trutta	51	0.01	0.03	0.01	0.04
0	1	0.57	1	t	1	Hydrobiosella stenocerca	Deleatidium lilli	51	0.01	0.04	0.04	0.05
1	1	0.81	1	cv	1	Achnanthes lanceolata	Aphrophila neozelandiae	51	0.03	0.04	0.05	0.04
0	1	0.72	1	t	1	Achnanthes lanceolata	Deleatidium lilli	51	0.03	0.04	0.04	0.05
0	0	0.27	1	t	1	Polyplectropus puerilis	Salmo trutta	51	0.01	0.04	0.01	0.04
1	1	0.66	1	t	1	Navicula placentula	Aphrophila neozelandiae	51	0.01	0.04	0.05	0.04
0	1	0.53	1	cv	1	Navicula placentula	Pycnocentrodes	51	0.01	0.04	0.04	0.04

1	1	0.91	1	t	1	Aphrophila neozelandiae	Navicula avenacea	51	0.05	0.04	0.05	0.04
0	1	0.60	1	t	1	Unknown blue green algae	Deleatidium lilli	51	0.02	0.04	0.04	0.05
0	1	0.79	1	t	1	Pycnocentrodes	Epithemia sorex	51	0.04	0.04	0.04	0.04
0	1	0.54	1	cv	1	Salmo trutta	Deleatidium lilli	51	0.01	0.04	0.04	0.05
0	0	0.29	1	t	1	Salmo trutta	Terrestrial invertebrates	51	0.01	0.04	0.01	0.03
0	0	0.50	1	t	1	Salmo trutta	Paranephrops zelandicus	51	0.01	0.04	0.03	0.04
0	1	0.87	1	t	1	Deleatidium lilli	Navicula avenacea	51	0.04	0.05	0.05	0.04
0	1	0.79	1	cv	1	Deleatidium lilli	Epithemia sorex	51	0.04	0.05	0.04	0.04
1	1	0.70	1	t	1	Navicula avenacea	Paucispinigera approximata	51	0.05	0.04	0.01	0.04
0	0	0.31	0	t	1	Hexatomini	Eukiefidrella	51	0.02	0.04	0.01	0.04
0	1	0.54	0	t	1	Megaleptoperla diminuata	Stenoperla prasinia	51	0.03	0.03	0.01	0.03
0	1	0.64	0	cv	1	Plant material	Hydrobiosella stenocerca	51	0.04	0.04	0.01	0.04
0	1	0.64	0	t	1	Coloburiscus humeralis	Hydrobiosis frater	51	0.01	0.04	0.04	0.04
0	1	0.66	0	t	1	Aphrophila neozelandiae	Terrestrial invertebrates	51	0.05	0.04	0.01	0.03
0	0	0.30	0	t	1	Gomphonema accuminatum	Navicula placentula	51	0.01	0.04	0.01	0.04
0	0	0.25	0	cv	1	Tabellaria flocculosa	Pinnularia viridis	51	0.01	0.04	0.01	0.04
0	1	0.81	0	t	1	Eunotia pectinalis	Eukiefidrella	51	0.08	0.06	0.01	0.04
1	1	0.63	0	t	1	Rhoicosphenia curvata	Achnanthes lanceolata	51	0.03	0.04	0.03	0.04
1	1	0.55	0	t	1	Synedra ulna	Fragilaria vaucheriae	51	0.01	0.04	0.03	0.04
0	0	0.28	0	cv	1	Gomphoneis herculeana var. robusta	Heliodidae beetle	51	0.02	0.04	0.01	0.04
0	0	0.40	0	t	1	Coloburiscus humeralis	Austroperla cyrene	51	0.01	0.04	0.02	0.04
0	0	0.45	0	t	1	Frustulia rhomboides	Unidentified detritus	51	0.03	0.04	0.01	0.04
0	0	0.41	0	t	1	Frustulia rhomboides	Navicula placentula	51	0.03	0.04	0.01	0.04
0	0	0.50	0	cv	1	Ulothrix	Rhopaloidea musculus	51	0.04	0.04	0.01	0.03
0	0	0.34	0	t	1	Coccoenis placentula	Synedra ulna	51	0.01	0.04	0.01	0.04

0	0	0.30	0	t	1	Gomphonema accuminatum	Polyplectropus puerilis	51	0.01	0.04	0.01	0.04
0	0	0.35	0	t	1	Coccoenis placentula	Pinnularia sp. A	51	0.01	0.04	0.02	0.04
0	0	0.37	0	cv	1	Frustulia rhomboides	Aspectrotanypus	51	0.03	0.04	0.01	0.04
0	0	0.37	0	t	1	Stigeoclonium	Polyplectropus puerilis	51	0.02	0.04	0.01	0.04
0	0	0.37	0	t	1	Coloburiscus humeralis	Nannochorista philpotti	51	0.01	0.04	0.02	0.04
1	1	0.75	0	t	1	Diatoma elongatum	Paranephrops zelandicus	51	0.04	0.04	0.03	0.04
1	1	0.78	0	cv	1	Costachorema xanthoptera	Diatoma elongatum	51	0.04	0.04	0.04	0.04
0	1	0.84	0	t	1	Tabellaria flocculosa	Eunotia pectinalis	51	0.01	0.04	0.08	0.06
0	1	0.61	0	t	1	Heliodidae beetle	Aphrophila neozelandiae	51	0.01	0.04	0.05	0.04
0	0	0.26	0	t	1	Nitzschia	Synedra ulna	51	0.01	0.05	0.01	0.04
0	1	0.93	0	cv	1	Frustulia rhomboides	Eunotia pectinalis	51	0.03	0.04	0.08	0.06
0	0	0.32	0	t	1	Naonella	Unknown blue green algae	51	0.01	0.04	0.02	0.04
0	1	0.50	0	t	1	Eukiefidrella	Pinnularia borealis	51	0.01	0.04	0.04	0.04
0	1	0.50	0	t	1	Nitzschia dubia	Deleatidium lilli	51	0.01	0.04	0.04	0.05
0	0	0.32	0	cv	1	Pinnularia viridis	Paucispinigera approximata	51	0.01	0.04	0.01	0.04
0	1	0.74	0	t	1	Pirara matakiri	Rhopaloidea musculus	51	0.06	0.04	0.01	0.03
0	1	0.87	0	t	1	Eunotia pectinalis	Megaleptoperla grandis	51	0.08	0.06	0.01	0.03
0	1	0.92	0	t	1	Scirtid skinny	Pirara matakiri	51	0.04	0.04	0.06	0.04
0	0	0.27	0	cv	1	Stauroneis vacheriae	Pinnularia sp. A	51	0.01	0.04	0.02	0.04
0	1	0.55	0	t	1	Orchymontia	Unknown blue green algae	51	0.03	0.04	0.02	0.04
0	1	0.55	0	t	1	Gomphoneis herculeana var. robusta	Megaleptoperla diminuata	51	0.02	0.04	0.03	0.03
0	0	0.26	0	t	1	Aspectrotanypus	Hydrobiosella stenocerca	51	0.01	0.04	0.01	0.04
0	1	0.63	0	cv	1	Austrosimulium australense	Plant material	51	0.01	0.04	0.04	0.04
0	1	0.58	0	t	1	Surirella linearis	Zelandoperla agnetis	51	0.01	0.03	0.04	0.04

0	0	0.23	0	t	1	Heliodidae beetle	Polyplectropus puerilis	51	0.01	0.04	0.01	0.04
1	1	0.88	0	t	1	Oligo skinny	Pirara matakiri	51	0.03	0.04	0.06	0.04
0	0	0.36	0	cv	1	Gomphonema accuminatum	Pinnularia sp. A	51	0.01	0.04	0.02	0.04
0	0	0.30	0	t	1	Naonella	Paucispinigera approximata	51	0.01	0.04	0.01	0.04
0	0	0.28	0	t	1	Hudsonema aliena	Philorheithrus agilis	51	0.01	0.04	0.01	0.03
0	0	0.34	0	t	1	Pinnularia viridis	Unknown blue green algae	51	0.01	0.04	0.02	0.04
0	0	0.25	0	cv	1	Stauroneis vacheriae	Paucispinigera approximata	51	0.01	0.04	0.01	0.04
0	1	0.61	0	t	1	Orchymontia	Stigeoclonium	51	0.03	0.04	0.02	0.04
0	0	0.45	0	t	1	Ceratopogonid	Gomphonema truncatum	51	0.02	0.03	0.02	0.04
0	1	0.58	0	t	1	Stigeoclonium	Achnanthes lanceolata	51	0.02	0.04	0.03	0.04
0	0	0.48	0	cv	1	Gomphoneis herculeana var. robusta	Frustulia rhomboides	51	0.02	0.04	0.03	0.04
0	0	0.23	0	t	1	Tabellaria flocculosa	Polyplectropus puerilis	51	0.01	0.04	0.01	0.04
0	1	0.65	0	t	1	Gomphonema accuminatum	Hydrobiosis frater	51	0.01	0.04	0.04	0.04
0	1	0.77	0	t	1	Scirtid skinny	Costachorema xanthoptera	51	0.04	0.04	0.04	0.04
0	1	0.69	0	cv	1	Navicula placentula	Navicula avenacea	51	0.01	0.04	0.05	0.04
0	0	0.30	0	t	1	Stenoperla prasinia	Rhopaloidea musculus	51	0.01	0.03	0.01	0.03
1	1	0.81	0	t	1	Pinnularia borealis	Epithemia sorex	51	0.04	0.04	0.04	0.04
0	1	0.55	0	t	1	Hydora nitida l	Gomphoneis herculeana var. robusta	51	0.03	0.04	0.02	0.04
0	1	0.77	0	cv	1	Orchymontia	Epithemia sorex	51	0.03	0.04	0.04	0.04
0	1	0.65	0	t	1	Pinnularia viridis	Plant material	51	0.01	0.04	0.04	0.04
0	0	0.36	0	t	1	Nitzschia palea	Paracalliope fluviatalus	51	0.01	0.05	0.02	0.04
0	0	0.42	0	t	1	Rhoicosphenia curvata	Salmo trutta	51	0.03	0.04	0.01	0.04

0	0	0.26	0	cv	1	Hudsonema aliena	Heliodidae beetle	51	0.01	0.04	0.01	0.04
0	1	0.55	0	t	1	Hydora nitida l	Gomphonema truncatum	51	0.03	0.04	0.02	0.04
0	0	0.25	0	t	1	Unknown green algae	Spirogyra	51	0.01	0.04	0.01	0.04
0	0	0.29	0	t	1	Austrosimulium australense	Unknown green algae spp.	51	0.01	0.04	0.01	0.04
0	1	0.73	0	cv	1	Paralimnophila skuseii	Spirogyra	51	0.06	0.04	0.01	0.04
0	0	0.36	0	t	1	Gomphoneis herculeana var. robusta	Oeconesus	51	0.02	0.04	0.01	0.04
0	1	0.77	0	t	1	Zelandoperla agnetis	Fragilaria vaucheriae	51	0.04	0.04	0.03	0.04
0	0	0.26	0	t	1	Coloburiscus humeralis	Aspectrotanypus	51	0.01	0.04	0.01	0.04
0	0	0.34	0	cv	1	Stictocladius	Ameletopsis perscitus	51	0.01	0.04	0.01	0.04
0	0	0.30	0	t	1	Hydrophiliid beetle	Nannochorista philpotti	51	0.01	0.04	0.02	0.04
0	0	0.42	0	t	1	Brundin	Achnanthes lanceolata	51	0.01	0.04	0.03	0.04
0	0	0.44	0	t	1	Frustulia rhomboides	Terrestrial invertebrates	51	0.03	0.04	0.01	0.03
1	1	0.64	0	cv	1	Diatoma elongatum	Austroperla cyrene	51	0.04	0.04	0.02	0.04
0	0	0.41	0	t	1	Frustulia rhomboides	Polyplectropus puerilis	51	0.03	0.04	0.01	0.04
0	0	0.45	0	t	1	Molophilus	Surirella linearis	51	0.03	0.04	0.01	0.03
0	0	0.34	0	t	1	Unknown blue green algae	Terrestrial invertebrates	51	0.02	0.04	0.01	0.03
0	0	0.34	0	cv	1	Calothrix	Gomphonema angustatum	51	0.01	0.04	0.01	0.04
0	0	0.34	0	t	1	Coloburiscus humeralis	Hudsonema aliena	51	0.01	0.04	0.01	0.04
0	0	0.47	0	t	1	Brundin	Fragilaria vaucheriae	51	0.01	0.04	0.03	0.04
1	1	0.87	0	t	1	Diatoma elongatum	Navicula avenacea	51	0.04	0.04	0.05	0.04
1	1	0.83	0	cv	1	Hydrobiosis frater	Pinnularia borealis	51	0.04	0.04	0.04	0.04
0	0	0.39	0	t	1	Stratiomyid l	Megaleptoperla grandis	51	0.02	0.04	0.01	0.03
0	1	0.59	0	t	1	Surirella	Ulothrix	51	0.01	0.04	0.04	0.04
1	1	0.81	0	t	1	Diatoma elongatum	Navicula rhynocephala	51	0.04	0.04	0.04	0.04
0	0	0.37	0	cv	1	Stratiomyid l	Ameletopsis perscitus	51	0.02	0.04	0.01	0.04

0	0	0.33	0	t	1	Oeconesus	Hydrobiosella stenocerca	51	0.01	0.04	0.01	0.04
0	0	0.27	0	t	1	Gomphonema accuminatum	Philorheithrus agilis	51	0.01	0.04	0.01	0.03
0	0	0.35	0	t	1	Unidentified detritus	Unknown blue green algae	51	0.01	0.04	0.02	0.04
1	1	0.71	0	cv	1	Pinnularia viridis	Navicula avenacea	51	0.01	0.04	0.05	0.04
0	0	0.34	0	t	1	Ceratopogonid	Eukiefidrella	51	0.02	0.03	0.01	0.04
1	1	0.58	0	t	1	Navicula rhynocephala	Naonella	51	0.04	0.04	0.01	0.04
0	0	0.32	0	t	1	Surirella linearis	Paucispinigera approximata	51	0.01	0.03	0.01	0.04
0	0	0.28	0	cv	1	Aspectrotanypus	Ameletopsis perscitus	51	0.01	0.04	0.01	0.04
1	1	0.66	0	t	1	Oligo skinny	Paranephrops zelandicus	51	0.03	0.04	0.03	0.04
1	1	0.80	0	t	1	Paralimnophila skuseii	Oeconesus	51	0.06	0.04	0.01	0.04
0	1	0.82	0	t	1	Costachorema xanthoptera	Hydrobiosis frater	51	0.04	0.04	0.04	0.04
0	0	0.31	0	cv	1	Navicula placentula	Ameletopsis perscitus	51	0.01	0.04	0.01	0.04
0	0	0.42	0	t	1	Gomphonema truncatum	Austroperla cyrene	51	0.02	0.04	0.02	0.04
0	0	0.38	0	t	1	Stictocladius	Nannochorista philpotti	51	0.01	0.04	0.02	0.04
0	1	0.51	0	t	1	Hydora nitida l	Surirella linearis	51	0.03	0.04	0.01	0.03
0	0	0.34	0	cv	1	Surirella elegans	Hudsonema aliena	51	0.01	0.04	0.01	0.04
0	0	0.34	0	t	1	Hudsonema aliena	Calothrix	51	0.01	0.04	0.01	0.04
0	1	0.53	0	t	1	Terrestrial invertebrates	Paranephrops zelandicus	51	0.01	0.03	0.03	0.04
0	0	0.27	0	t	1	Stauroneis vacheriae	Unknown blue green algae	51	0.01	0.04	0.02	0.04
0	0	0.34	0	cv	1	Ceratopogonid	Heliodidae beetle	51	0.02	0.03	0.01	0.04
0	0	0.46	0	t	1	Nitzschia palea	Fragilaria vaucheriae	51	0.01	0.05	0.03	0.04
0	0	0.33	0	t	1	Stigeoclonium	Nitzschia dubia	51	0.02	0.04	0.01	0.04
0	0	0.32	0	t	1	Pinnularia viridis	Ameletopsis perscitus	51	0.01	0.04	0.01	0.04
0	1	0.77	0	cv	1	Orchymontia	Pinnularia borealis	51	0.03	0.04	0.04	0.04

0	0	0.29	0	t	1	Brundin	Pinnularia sp. A	51	0.01	0.04	0.02	0.04
0	0	0.36	0	t	1	Unknown blue green algae	Ameletopsis perscitus	51	0.02	0.04	0.01	0.04
0	1	0.67	0	t	1	Stictocladius	Plant material	51	0.01	0.04	0.04	0.04
0	1	0.53	0	cv	1	Gomphonema accuminatum	Megaleptoperla diminuata	51	0.01	0.04	0.03	0.03
0	1	0.59	0	t	1	Hydrobiosis frater	Naonella	51	0.04	0.04	0.01	0.04
1	1	0.82	0	t	1	Hydrobiosis frater	Pycnocentrodes	51	0.04	0.04	0.04	0.04
0	0	0.43	0	t	1	Paracalliope fluviatalus	Hydrobiosella stenocerca	51	0.02	0.04	0.01	0.04
0	0	0.48	0	cv	1	Gomphonema truncatum	Frustulia rhomboides	51	0.02	0.04	0.03	0.04
0	0	0.36	0	t	1	Gomphonema truncatum	Hudsonema aliena	51	0.02	0.04	0.01	0.04
0	1	0.81	0	t	1	Paracalliope fluviatalus	Navicula avenacea	51	0.02	0.04	0.05	0.04
0	0	0.34	0	t	1	Pinnularia viridis	Pinnularia sp. A	51	0.01	0.04	0.02	0.04
0	0	0.34	0	cv	1	Hexatomini	Rhopaloidea musculus	51	0.02	0.04	0.01	0.03
0	0	0.23	0	t	1	Austrosimulium australense	Eukiefidrella	51	0.01	0.04	0.01	0.04
0	0	0.27	0	t	1	Brundin	Synedra ulna	51	0.01	0.04	0.01	0.04
0	0	0.29	0	t	1	Navicula placentula	Terrestrial invertebrates	51	0.01	0.04	0.01	0.03
0	0	0.37	0	cv	1	Stictocladius	Stratiomyid I	51	0.01	0.04	0.02	0.04
0	0	0.38	0	t	1	Frustulia rhomboides	Rhopaloidea musculus	51	0.03	0.04	0.01	0.03
0	1	0.78	0	t	1	Hexatomini	Navicula avenacea	51	0.02	0.04	0.05	0.04
0	0	0.33	0	t	1	Stictocladius	Hydrobiosella stenocerca	51	0.01	0.04	0.01	0.04
0	1	0.54	0	cv	1	Synedra ulna	Paranephrops zelandicus	51	0.01	0.04	0.03	0.04
0	1	0.58	0	t	1	Gomphonema accuminatum	Diatoma elongatum	51	0.01	0.04	0.04	0.04
0	0	0.46	0	t	1	Scirtid skinny	Eukiefidrella	51	0.04	0.04	0.01	0.04
0	1	0.56	0	t	1	Stratiomyid I	Megaleptoperla diminuata	51	0.02	0.04	0.03	0.03
0	1	0.56	0	cv	1	Hydrobiosella stenocerca	Pycnocentrodes	51	0.01	0.04	0.04	0.04
0	0	0.37	0	t	1	Stauroneis vacheriae	Molophilus	51	0.01	0.04	0.03	0.04
0	1	0.86	0	t	1	Zelandoperla agnetis	Aphrophila neozelandiae	51	0.04	0.04	0.05	0.04
1	1	0.65	0	t	1	Calothrix	Hydrobiosis frater	51	0.01	0.04	0.04	0.04

0	1	0.53	0	cv	1	Hudsonema aliena	Megaleptoperla diminuata	51	0.01	0.04	0.03	0.03
0	0	0.19	0	t	1	Stauroneis vacheriae	Spirogyra	51	0.01	0.04	0.01	0.04
0	1	0.56	0	t	1	Eukiefidrella	Hydrobiosis frater	51	0.01	0.04	0.04	0.04
0	0	0.19	0	t	1	Eukiefidrella	Heliodidae beetle	51	0.01	0.04	0.01	0.04
0	1	0.58	0	cv	1	Costachorema xanthoptera	Pinnularia sp. A	51	0.04	0.04	0.02	0.04
0	1	0.54	0	t	1	Gomphonema angustatum	Paranephrops zelandicus	51	0.01	0.04	0.03	0.04
0	0	0.34	0	t	1	Brundin	Hexatomini	51	0.01	0.04	0.02	0.04
0	0	0.49	0	t	1	Orchymontia	Navicula placentula	51	0.03	0.04	0.01	0.04
0	0	0.42	0	cv	1	Molophilus	Salmo trutta	51	0.03	0.04	0.01	0.04
0	1	0.62	0	t	1	Stigeoclonium	Paranephrops zelandicus	51	0.02	0.04	0.03	0.04
0	1	0.66	0	t	1	Hydora nitida l	Oligo skinny	51	0.03	0.04	0.03	0.04
0	1	0.67	0	t	1	Gomphonema angustatum	Plant material	51	0.01	0.04	0.04	0.04
0	1	0.55	0	cv	1	Pinnularia viridis	Costachorema xanthoptera	51	0.01	0.04	0.04	0.04
0	0	0.36	0	t	1	Gomphoneis herculeana var. robusta	Ameletopsis perscitus	51	0.02	0.04	0.01	0.04
1	1	0.72	0	t	1	Ulothrix	Achnanthes lanceolata	51	0.04	0.04	0.03	0.04
1	1	0.65	0	t	1	Surirella elegans	Hydrobiosis frater	51	0.01	0.04	0.04	0.04
0	0	0.24	0	cv	1	Nitzschia palea	Unknown green algae spp.	51	0.01	0.05	0.01	0.04
0	0	0.23	0	t	1	Eukiefidrella	Navicula placentula	51	0.01	0.04	0.01	0.04
0	1	0.67	0	t	1	Ceratopogonid	Costachorema xanthoptera	51	0.02	0.03	0.04	0.04
0	1	0.56	0	t	1	Ceratopogonid	Frustulia rhomboides	51	0.02	0.03	0.03	0.04
0	0	0.28	0	cv	1	Heliodidae beetle	Megaleptoperla grandis	51	0.01	0.04	0.01	0.03
0	0	0.32	0	t	1	Pinnularia	Calothrix	51	0.01	0.04	0.01	0.04
1	1	0.91	0	t	1	Paralimnophila skuseii	Pycnocentrodes	51	0.06	0.04	0.04	0.04
0	1	0.58	0	t	1	Unknown green algae	Epithemia sorex	51	0.01	0.04	0.04	0.04
0	0	0.42	0	cv	1	Rhoicosphenia curvata	Navicula placentula	51	0.03	0.04	0.01	0.04

0	0	0.48	0	t	1	Austrosimulium australense	Megaleptoperla diminuata	51	0.01	0.04	0.03	0.03
0	0	0.30	0	t	1	Rhopaloidea musculus	Pinnularia sp. A	51	0.01	0.03	0.02	0.04
0	1	0.84	0	t	1	Eunotia pectinalis	Polyplectropus puerilis	51	0.08	0.06	0.01	0.04
0	1	0.59	0	cv	1	Surirella linearis	Epithemia sorex	51	0.01	0.03	0.04	0.04
1	1	0.66	0	t	1	Unidentified detritus	Plant material	51	0.01	0.04	0.04	0.04
0	1	0.76	0	t	1	Fragilaria vaucheriae	Deleatidium lilli	51	0.03	0.04	0.04	0.05
0	1	0.54	0	t	1	Philorheithrus agilis	Epithemia sorex	51	0.01	0.03	0.04	0.04
0	1	0.59	0	cv	1	Stictocladius	Deleatidium lilli	51	0.01	0.04	0.04	0.05
0	0	0.44	0	t	1	Megaleptoperla diminuata	Philorheithrus agilis	51	0.03	0.03	0.01	0.03
0	1	0.70	0	t	1	Molophilus	Deleatidium lilli	51	0.03	0.04	0.04	0.05
0	1	0.92	0	t	1	Ceratopogonid	Eunotia pectinalis	51	0.02	0.03	0.08	0.06
0	0	0.33	0	cv	1	Synedra ulna	Terrestrial invertebrates	51	0.01	0.04	0.01	0.03
0	0	0.28	0	t	1	Gomphonema truncatum	Philorheithrus agilis	51	0.02	0.04	0.01	0.03
0	1	0.74	0	t	1	Paralimnophila skuseii	Philorheithrus agilis	51	0.06	0.04	0.01	0.03
1	1	0.71	0	t	1	Rhoicosphenia curvata	Ulothrix	51	0.03	0.04	0.04	0.04
1	1	0.80	0	cv	1	Pinnularia borealis	Zelandoperla agnetis	51	0.04	0.04	0.04	0.04
0	0	0.45	0	t	1	Coloburiscus humeralis	Paracalliope fluviatalus	51	0.01	0.04	0.02	0.04
0	0	0.43	0	t	1	Hexatomini	Unknown blue green algae	51	0.02	0.04	0.02	0.04
0	0	0.34	0	t	1	Surirella elegans	Gomphonema accuminatum	51	0.01	0.04	0.01	0.04
0	0	0.31	0	cv	1	Unknown green algae	Hydrobiosella stenocerca	51	0.01	0.04	0.01	0.04
1	1	0.57	0	t	1	Synedra ulna	Pycnocentrodes	51	0.01	0.04	0.04	0.04
0	0	0.48	0	t	1	Nitzschia	Scirtid skinny	51	0.01	0.05	0.04	0.04
0	0	0.28	0	t	1	Hudsonema aliena	Rhopaloidea musculus	51	0.01	0.04	0.01	0.03
0	0	0.48	0	cv	1	Zelandoperla agnetis	Heliodidae beetle	51	0.04	0.04	0.01	0.04
0	0	0.34	0	t	1	Gomphonema truncatum	Terrestrial invertebrates	51	0.02	0.04	0.01	0.03

0	0	0.36	0	t	1	Unknown green algae	Nannochorista philpotti	51	0.01	0.04	0.02	0.04
0	0	0.28	0	t	1	Unknown green algae	Navicula placentula	51	0.01	0.04	0.01	0.04
0	1	0.61	0	cv	1	Aphrophila neozelandiae	Rhopaloidea musculus	51	0.05	0.04	0.01	0.03
0	0	0.38	0	t	1	Oligo skinny	Aspectrotanypus	51	0.03	0.04	0.01	0.04
0	0	0.43	0	t	1	Ceratopogonid	Surirella elegans	51	0.02	0.03	0.01	0.04
0	1	0.61	0	t	1	Hydrobiosis frater	Unknown green algae spp.	51	0.04	0.04	0.01	0.04
0	0	0.34	0	cv	1	Ameletopsis perscitus	Paucispinigera approximata	51	0.01	0.04	0.01	0.04
0	0	0.25	0	t	1	Hydrophiliid beetle	Hydrobiosella stenocerca	51	0.01	0.04	0.01	0.04
0	0	0.30	0	t	1	Stictocladius	Naonella	51	0.01	0.04	0.01	0.04
0	1	0.77	0	t	1	Ceratopogonid	Aphrophila neozelandiae	51	0.02	0.03	0.05	0.04
0	1	0.58	0	cv	1	Hudsonema aliena	Pycnocentrodes	51	0.01	0.04	0.04	0.04
0	0	0.30	0	t	1	Calothrix	Salmo trutta	51	0.01	0.04	0.01	0.04
0	0	0.25	0	t	1	Heliodidae beetle	Terrestrial invertebrates	51	0.01	0.04	0.01	0.03
0	0	0.36	0	t	1	Synedra ulna	Megaleptoperla grandis	51	0.01	0.04	0.01	0.03
0	1	0.69	0	cv	1	Austroperla cyrene	Navicula rhynocephala	51	0.02	0.04	0.04	0.04
0	0	0.34	0	t	1	Synedra ulna	Gomphonema angustatum	51	0.01	0.04	0.01	0.04
0	1	0.72	0	t	1	Stigeoclonium	Hydrobiosis frater	51	0.02	0.04	0.04	0.04
0	1	0.75	0	t	1	Orchymontia	Costachorema xanthoptera	51	0.03	0.04	0.04	0.04
0	0	0.19	0	cv	1	Tabellaria flocculosa	Stauroneis vacheriae	51	0.01	0.04	0.01	0.04
0	0	0.42	0	t	1	Achnanthes lanceolata	Rhopaloidea musculus	51	0.03	0.04	0.01	0.03
0	0	0.34	0	t	1	Calothrix	Paucispinigera approximata	51	0.01	0.04	0.01	0.04
1	1	0.71	0	t	1	Hydora nitida l	Megaleptoperla diminuata	51	0.03	0.04	0.03	0.03
0	0	0.44	0	cv	1	Stratiomyid l	Austroperla cyrene	51	0.02	0.04	0.02	0.04
0	1	0.86	0	t	1	Eunotia pectinalis	Calothrix	51	0.08	0.06	0.01	0.04

0	1	0.58	0	t	1	Gomphonema angustatum	Deleatidium lilli	51	0.01	0.04	0.04	0.05
1	1	0.77	0	t	1	Frustulia rhomboides	Plant material	51	0.03	0.04	0.04	0.04
0	1	0.59	0	cv	1	Synedra ulna	Ulothrix	51	0.01	0.04	0.04	0.04
0	1	0.67	0	t	1	Hexatomini	Phreatoicus prasinia	51	0.02	0.04	0.04	0.04
0	0	0.50	0	t	1	Gomphonema angustatum	Achnanthes lanceolata	51	0.01	0.04	0.03	0.04
1	1	0.56	0	t	1	Fragilaria vaucheriae	Megaleptoperla grandis	51	0.03	0.04	0.01	0.03
0	0	0.20	0	cv	1	Hydrophiliid beetle	Nitzschia dubia	51	0.01	0.04	0.01	0.04
0	1	0.59	0	t	1	Coccoenis placentula	Zelandoperla agnetis	51	0.01	0.04	0.04	0.04
0	0	0.32	0	t	1	Unidentified detritus	Unknown green algae spp.	51	0.01	0.04	0.01	0.04
0	0	0.44	0	t	1	Stigeoclonium	Unknown blue green algae	51	0.02	0.04	0.02	0.04
0	1	0.54	0	cv	1	Phreatoicus prasinia	Polyplectropus puerilis	51	0.04	0.04	0.01	0.04
0	1	0.84	0	t	1	Nitzschia palea	Eunotia pectinalis	51	0.01	0.05	0.08	0.06
1	1	0.69	0	t	1	Oligo skinny	Pycnocentrodes	51	0.03	0.04	0.04	0.04
0	1	0.58	0	t	1	Unknown green algae	Pinnularia borealis	51	0.01	0.04	0.04	0.04
0	0	0.24	0	cv	1	Aspectrotanypus	Salmo trutta	51	0.01	0.04	0.01	0.04
0	0	0.29	0	t	1	Unknown blue green algae	Rhopaloidea musculus	51	0.02	0.04	0.01	0.03
0	1	0.78	0	t	1	Scirtid skinny	Deleatidium lilli	51	0.04	0.04	0.04	0.05
0	0	0.43	0	t	1	Pinnularia viridis	Paracalliope fluviatalus	51	0.01	0.04	0.02	0.04
0	1	0.84	0	cv	1	Eunotia pectinalis	Naonella	51	0.08	0.06	0.01	0.04
1	1	0.79	0	t	1	Hydora nitida l	Navicula rhynocephala	51	0.03	0.04	0.04	0.04
0	0	0.20	0	t	1	Hydrophiliid beetle	Heliodidae beetle	51	0.01	0.04	0.01	0.04
0	0	0.38	0	t	1	Nitzschia	Oligo skinny	51	0.01	0.05	0.03	0.04
0	0	0.27	0	cv	1	Unidentified detritus	Rhopaloidea musculus	51	0.01	0.04	0.01	0.03
0	1	0.68	0	t	1	Stigeoclonium	Phreatoicus prasinia	51	0.02	0.04	0.04	0.04
0	0	0.43	0	t	1	Stigeoclonium	Pinnularia sp. A	51	0.02	0.04	0.02	0.04
0	1	0.53	0	t	1	Hydora nitida l	Synedra ulna	51	0.03	0.04	0.01	0.04

0	0	0.45	0	cv	1	Pinnularia	Molophilus	51	0.01	0.04	0.03	0.04
0	1	0.56	0	t	1	Megaleptoperla grandis	Paranephrops zelandicus	51	0.01	0.03	0.03	0.04
0	0	0.32	1	t	1	Aoteapsyche raruraru	Terrestrial invertebrate remains	52	0.02	0.03	0.01	0.03
0	0	0.45	1	t	1	Aoteapsyche raruraru	Salmo trutta	52	0.02	0.03	0.02	0.04
0	0	0.31	1	cv	1	Aoteapsyche raruraru	Deleatidum lilli	52	0.02	0.03	0.01	0.04
0	0	0.41	1	t	1	Tanytarsini sp.	Gomphonema subclavatum	52	0.03	0.04	0.01	0.05
0	1	0.70	1	t	1	Tanytarsini sp.	Tiphobiosis montana	52	0.03	0.04	0.04	0.03
0	1	0.69	1	t	1	Tanytarsini sp.	Unidentified organic detritus	52	0.03	0.04	0.04	0.05
0	1	0.55	1	cv	1	Tanytarsini sp.	Terrestrial plant material	52	0.03	0.04	0.03	0.04
0	1	0.54	1	t	1	Tanytarsini sp.	Fragilaria vaucheriae	52	0.03	0.04	0.02	0.04
0	1	0.50	1	t	1	Tanytarsini sp.	Navicula avenacea	52	0.03	0.04	0.02	0.05
0	1	0.68	1	t	1	Zelandoperla fenestrata	Tabellaria flocculosa	52	0.05	0.04	0.02	0.05
0	1	0.60	1	cv	1	Zelandoperla fenestrata	Gomphonema truncatum	52	0.05	0.04	0.01	0.04
0	1	0.60	1	t	1	Zelandoperla fenestrata	Surirella elegans	52	0.05	0.04	0.01	0.04
0	1	0.54	1	t	1	Zelandoperla fenestrata	Cymbella aspera	52	0.05	0.04	0.01	0.04
1	1	0.91	1	t	1	Zelandoperla fenestrata	Rhoicosphenia curvata	52	0.05	0.04	0.06	0.05
0	1	0.60	1	cv	1	Zelandoperla fenestrata	Cricotopus sp. Type 'pale'	52	0.05	0.04	0.01	0.04
0	1	0.83	1	t	1	Zelandoperla fenestrata	Unidentified organic detritus	52	0.05	0.04	0.04	0.05
0	1	0.73	1	t	1	Zelandoperla fenestrata	Terrestrial plant material	52	0.05	0.04	0.03	0.04
0	1	0.60	1	t	1	Zelandoperla fenestrata	Cocconeis placentual	52	0.05	0.04	0.01	0.05
0	1	0.69	1	cv	1	Zelandoperla fenestrata	Navicula avenacea	52	0.05	0.04	0.02	0.05
0	1	0.70	1	t	1	Zelandoperla fenestrata	Salmo trutta	52	0.05	0.04	0.02	0.04

0	1	0.82	1	t	1	Hydraena homolaena	Rhoicospheni a curvata	52	0.03	0.04	0.06	0.05
0	0	0.33	1	t	1	Hydraena homolaena	Gomphonem a sp. II	52	0.03	0.04	0.01	0.04
0	0	0.38	1	cv	1	Hydraena homolaena	Gomphonem a subclavatum	52	0.03	0.04	0.01	0.05
0	1	0.67	1	t	1	Hydraena homolaena	Unidentified organic detritus	52	0.03	0.04	0.04	0.05
0	0	0.48	1	t	1	Hydraena homolaena	Navicula avenacea	52	0.03	0.04	0.02	0.05
0	0	0.35	1	t	1	Tabellaria flocculosa	Amphipoda type 'purple'	52	0.02	0.05	0.02	0.06
1	1	0.87	1	cv	1	Tabellaria flocculosa	Austroperla cyrene	52	0.02	0.05	0.07	0.05
0	0	0.44	1	t	1	Tabellaria flocculosa	Aphrophila neozelandicu s	52	0.02	0.05	0.02	0.04
0	1	0.55	1	t	1	Hudsonema amabilis	Unidentified organic detritus	52	0.01	0.04	0.04	0.05
0	0	0.40	1	t	1	Hudsonema amabilis	Terrestrial plant material	52	0.01	0.04	0.03	0.04
1	1	0.84	1	cv	1	Maoridiamesi a sp.	Rhoicospheni a curvata	52	0.03	0.04	0.06	0.05
0	1	0.72	1	t	1	Maoridiamesi a sp.	Unidentified organic detritus	52	0.03	0.04	0.04	0.05
0	1	0.70	1	t	1	Maoridiamesi a sp.	Ulothrix spp.	52	0.03	0.04	0.04	0.04
0	1	0.50	1	t	1	Maoridiamesi a sp.	Nitzschia linearis	52	0.03	0.04	0.02	0.04
0	0	0.43	1	cv	1	Maoridiamesi a sp.	Cocconeis placentual	52	0.03	0.04	0.01	0.05
0	1	0.53	1	t	1	Maoridiamesi a sp.	Navicula avenacea	52	0.03	0.04	0.02	0.05
0	0	0.37	1	t	1	Gomphonema truncatum	Aphrophila neozelandicu s	52	0.01	0.04	0.02	0.04
0	0	0.48	1	t	1	Surirella	Hydora nitida	52	0.01	0.04	0.03	0.04
0	0	0.47	1	cv	1	Lumbriculiid type 'II'	Ameletopsis perscitus	52	0.03	0.04	0.01	0.04
0	1	0.86	1	t	1	Lumbriculiid type 'II'	Rhoicospheni a curvata	52	0.03	0.04	0.06	0.05
0	0	0.41	1	t	1	Lumbriculiid type 'II'	Blue-green algae	52	0.03	0.04	0.01	0.04
0	1	0.74	1	t	1	Lumbriculiid type 'II'	Unidentified organic detritus	52	0.03	0.04	0.04	0.05

0	1	0.61	1	cv	1	Lumbriculiid type 'II'	Terrestrial plant material	52	0.03	0.04	0.03	0.04
0	0	0.47	1	t	1	Lumbriculiid type 'II'	Stenoperla prasinia	52	0.03	0.04	0.01	0.04
0	1	0.56	1	t	1	Lumbriculiid type 'II'	Navicula avenacea	52	0.03	0.04	0.02	0.05
0	1	0.86	1	t	1	Hydora nitida	Rhoicospheni a curvata	52	0.03	0.04	0.06	0.05
0	0	0.46	1	cv	1	Hydora nitida	Gomphonem a subclavatum	52	0.03	0.04	0.01	0.05
0	1	0.74	1	t	1	Hydora nitida	Unidentified organic detritus	52	0.03	0.04	0.04	0.05
0	1	0.61	1	t	1	Hydora nitida	Terrestrial plant material	52	0.03	0.04	0.03	0.04
0	0	0.47	1	t	1	Hydora nitida	Stenoperla prasinia	52	0.03	0.04	0.01	0.04
0	1	0.58	1	cv	1	Hydora nitida	Salmo trutta	52	0.03	0.04	0.02	0.04
0	0	0.40	1	t	1	Cyclotella sp.	Paracalliope fluviatalus	52	0.01	0.04	0.03	0.04
0	0	0.24	1	t	1	Ameletopsis perscitus	Deleatidum lilli	52	0.01	0.04	0.01	0.04
1	1	0.71	1	t	1	Rhoicosphenia curvata	Austrosimuliu m australense	52	0.06	0.05	0.01	0.04
1	1	0.83	1	cv	1	Rhoicosphenia curvata	Paracalliope fluviatalus	52	0.06	0.05	0.03	0.04
1	1	0.97	1	t	1	Rhoicosphenia curvata	Austroperla cyrene	52	0.06	0.05	0.07	0.05
1	1	0.74	1	t	1	Rhoicosphenia curvata	Naonella forsythii	52	0.06	0.05	0.02	0.04
1	1	0.75	1	t	1	Rhoicosphenia curvata	Potamopyrgu s antipodarum	52	0.06	0.05	0.02	0.04
1	1	0.83	1	cv	1	Rhoicosphenia curvata	Isopoda	52	0.06	0.05	0.03	0.04
1	1	0.96	1	t	1	Rhoicosphenia curvata	Stictocladius sp.	52	0.06	0.05	0.06	0.05
1	1	0.75	1	t	1	Rhoicosphenia curvata	Zelandobius confusus	52	0.06	0.05	0.02	0.04
1	1	0.66	1	t	1	Rhoicosphenia curvata	Deleatidum lilli	52	0.06	0.05	0.01	0.04
1	1	0.93	1	cv	1	Rhoicosphenia curvata	Pycnocentria evecta	52	0.06	0.05	0.05	0.04
0	0	0.25	1	t	1	Austrosimuliu m australense	Frustulia rhomboides	52	0.01	0.04	0.01	0.04
0	0	0.31	1	t	1	Austrosimuliu m australense	Gomphonem a angustatum	52	0.01	0.04	0.01	0.04

0	1	0.56	1	t	1	Austrosimulium australense	Synedra rumpens	52	0.01	0.04	0.04	0.04
0	1	0.54	1	cv	1	Austrosimulium australense	Ulothrix spp.	52	0.01	0.04	0.04	0.04
0	0	0.42	1	t	1	Austrosimulium australense	Terrestrial plant material	52	0.01	0.04	0.03	0.04
0	0	0.49	1	t	1	Austrosimulium australense	Gomphonema parvulum	52	0.01	0.04	0.03	0.04
0	0	0.37	1	t	1	Austrosimulium australense	Navicula avenacea	52	0.01	0.04	0.02	0.05
0	1	0.63	1	cv	1	Batrachospermum spp.	Scirtidae type 'broad'	52	0.04	0.04	0.02	0.04
0	1	0.53	1	t	1	Batrachospermum spp.	Amphipoda type 'purple'	52	0.04	0.04	0.02	0.06
0	0	0.39	1	t	1	Synedra ulna	Scirtidae type 'broad'	52	0.01	0.04	0.02	0.04
0	0	0.35	1	t	1	Synedra ulna	Potamopyrgus antipodarum	52	0.01	0.04	0.02	0.04
0	0	0.39	1	cv	1	Synedra ulna	Aphrophila neozelandicus	52	0.01	0.04	0.02	0.04
0	0	0.26	1	t	1	Synedra ulna	Deleatidum lilli	52	0.01	0.04	0.01	0.04
0	1	0.64	1	t	1	Podaena sp.	Unidentified organic detritus	52	0.02	0.04	0.04	0.05
0	0	0.49	1	t	1	Podaena sp.	Terrestrial plant material	52	0.02	0.04	0.03	0.04
0	1	0.66	1	cv	1	Scirtidae type 'broad'	Unidentified organic detritus	52	0.02	0.04	0.04	0.05
0	1	0.51	1	t	1	Scirtidae type 'broad'	Terrestrial plant material	52	0.02	0.04	0.03	0.04
1	1	0.92	1	t	1	Eukiefliidriella brundini	Unidentified organic detritus	52	0.07	0.05	0.04	0.05
1	1	0.84	1	t	1	Eukiefliidriella brundini	Navicula avenacea	52	0.07	0.05	0.02	0.05
1	1	0.86	1	cv	1	Eukiefliidriella brundini	Megaleptoperla grandis	52	0.07	0.05	0.03	0.05
0	0	0.50	1	t	1	Unknown green algae	Cricotopus sp. Type 'pale'	52	0.03	0.03	0.01	0.04
0	1	0.57	1	t	1	Unknown green algae	Potamopyrgus antipodarum	52	0.03	0.03	0.02	0.04

0	0	0.46	1	t	1	Unknown green algae	Deleatidum lilli	52	0.03	0.03	0.01	0.04
0	0	0.24	1	cv	1	Frustulia rhomboides	Cricotopus sp. Type 'pale'	52	0.01	0.04	0.01	0.04
0	0	0.28	1	t	1	Frustulia rhomboides	Naonella forsythii	52	0.01	0.04	0.02	0.04
0	0	0.29	1	t	1	Frustulia rhomboides	Potamopyrgus antipodarum	52	0.01	0.04	0.02	0.04
0	0	0.32	1	t	1	Frustulia rhomboides	Aphrophila neozelandicus	52	0.01	0.04	0.02	0.04
0	0	0.28	1	cv	1	Frustulia rhomboides	Zelandobius confusus	52	0.01	0.04	0.02	0.04
0	0	0.21	1	t	1	Frustulia rhomboides	Deleatidum lilli	52	0.01	0.04	0.01	0.04
0	1	0.60	1	t	1	Cristaperla fimbria	Unidentified organic detritus	52	0.02	0.04	0.04	0.05
0	0	0.35	1	t	1	Gomphonema angustatum	Potamopyrgus antipodarum	52	0.01	0.04	0.02	0.04
0	0	0.26	1	cv	1	Gomphonema angustatum	Deleatidum lilli	52	0.01	0.04	0.01	0.04
1	1	0.85	1	t	1	Epithemis turgida	Austroperla cyrene	52	0.02	0.05	0.07	0.05
0	0	0.40	1	t	1	Epithemis turgida	Aphrophila neozelandicus	52	0.02	0.05	0.02	0.04
0	0	0.23	1	t	1	Amphipoda type 'purple'	Blue-green algae	52	0.02	0.06	0.01	0.04
0	1	0.85	1	cv	1	Amphipoda type 'purple'	Oedogonium spp.	52	0.02	0.06	0.08	0.07
0	1	0.55	1	t	1	Amphipoda type 'purple'	Unidentified organic detritus	52	0.02	0.06	0.04	0.05
0	1	0.53	1	t	1	Amphipoda type 'purple'	Ulothrix spp.	52	0.02	0.06	0.04	0.04
0	0	0.40	1	t	1	Amphipoda type 'purple'	Terrestrial plant material	52	0.02	0.06	0.03	0.04
0	0	0.37	1	cv	1	Amphipoda type 'purple'	Aphrophila neozelandicus	52	0.02	0.06	0.02	0.04
0	1	0.55	1	t	1	Rhabdomastrix sp.	Unidentified organic detritus	52	0.01	0.04	0.04	0.05
0	0	0.40	1	t	1	Rhabdomastrix sp.	Terrestrial plant material	52	0.01	0.04	0.03	0.04

0	1	0.55	1	t	1	Cricotopus sp. Type 'pale'	Unidentified organic detritus	52	0.01	0.04	0.04	0.05
0	0	0.40	1	cv	1	Cricotopus sp. Type 'pale'	Terrestrial plant material	52	0.01	0.04	0.03	0.04
0	0	0.27	1	t	1	Cricotopus sp. Type 'pale'	Stenoperla prasinia	52	0.01	0.04	0.01	0.04
0	1	0.76	1	t	1	Cricotopus sp. Type 'pale'	Navicula placentula	52	0.01	0.04	0.06	0.05
0	0	0.35	1	t	1	Cricotopus sp. Type 'pale'	Navicula avenacea	52	0.01	0.04	0.02	0.05
0	0	0.39	1	cv	1	Cricotopus sp. Type 'pale'	Megaleptoperla grandis	52	0.01	0.04	0.03	0.05
0	1	0.59	1	t	1	Synedra rumpens	Zelandobius confusus	52	0.04	0.04	0.02	0.04
0	1	0.83	1	t	1	Gomphonema subclavatum	Austroperla cyrene	52	0.01	0.05	0.07	0.05
0	0	0.31	1	t	1	Gomphonema subclavatum	Naonella forsythii	52	0.01	0.05	0.02	0.04
0	0	0.32	1	cv	1	Gomphonema subclavatum	Potamopyrgus antipodarum	52	0.01	0.05	0.02	0.04
0	0	0.36	1	t	1	Gomphonema subclavatum	Aphrophila neozelandicus	52	0.01	0.05	0.02	0.04
0	0	0.23	1	t	1	Gomphonema subclavatum	Deleatidum lilli	52	0.01	0.05	0.01	0.04
0	0	0.26	1	t	1	Terrestrial invertebrate remains	Stenoperla prasinia	52	0.01	0.03	0.01	0.04
0	0	0.25	1	cv	1	Terrestrial invertebrate remains	Hydrobiosella stenocerca	52	0.01	0.03	0.01	0.03
0	0	0.35	1	t	1	Terrestrial invertebrate remains	Aphrophila neozelandicus	52	0.01	0.03	0.02	0.04
0	0	0.35	1	t	1	Terrestrial invertebrate remains	Salmo trutta	52	0.01	0.03	0.02	0.04
0	0	0.45	1	t	1	Terrestrial invertebrate remains	Paranephrops zelandicus	52	0.01	0.03	0.03	0.04
0	1	0.70	1	cv	1	Paracalliope fluviatalus	Pinnularia borealis	52	0.03	0.04	0.04	0.04
0	1	0.72	1	t	1	Paracalliope fluviatalus	Unidentified organic detritus	52	0.03	0.04	0.04	0.05
0	1	0.58	1	t	1	Paracalliope fluviatalus	Terrestrial plant material	52	0.03	0.04	0.03	0.04

1	1	0.84	1	t	1	Paracalliope fluviatalus	Galaxias brevipennis	52	0.03	0.04	0.05	0.04
0	1	0.53	1	cv	1	Paracalliope fluviatalus	Navicula avenacea	52	0.03	0.04	0.02	0.05
0	1	0.55	1	t	1	Paracalliope fluviatalus	Salmo trutta	52	0.03	0.04	0.02	0.04
0	1	0.68	1	t	1	Coloburiscus humeralis	Unidentified organic detritus	52	0.03	0.04	0.04	0.05
0	1	0.54	1	t	1	Coloburiscus humeralis	Terrestrial plant material	52	0.03	0.04	0.03	0.04
0	1	0.81	1	cv	1	Coloburiscus humeralis	Galaxias brevipennis	52	0.03	0.04	0.05	0.04
0	0	0.49	1	t	1	Coloburiscus humeralis	Navicula avenacea	52	0.03	0.04	0.02	0.05
0	1	0.51	1	t	1	Coloburiscus humeralis	Salmo trutta	52	0.03	0.04	0.02	0.04
0	1	0.63	1	t	1	Pinnularia borealis	Aphrophila neozelandicu s	52	0.04	0.04	0.02	0.04
0	0	0.28	1	cv	1	Blue-green algae	Potamopyrgu s antipodarum	52	0.01	0.04	0.02	0.04
0	0	0.27	1	t	1	Blue-green algae	Zelandobius confusus	52	0.01	0.04	0.02	0.04
0	0	0.20	1	t	1	Blue-green algae	Deleatidum lilli	52	0.01	0.04	0.01	0.04
1	1	0.94	1	t	1	Unidentified organic	Austroperla cyrene	52	0.04	0.05	0.07	0.05
0	1	0.58	1	cv	1	Unidentified organic	Naonella forsythii	52	0.04	0.05	0.02	0.04
0	1	0.60	1	t	1	Unidentified organic detritus	Potamopyrgu s antipodarum	52	0.04	0.05	0.02	0.04
1	1	0.70	1	t	1	Unidentified organic	Isopoda	52	0.04	0.05	0.03	0.04
0	1	0.64	1	t	1	Unidentified organic detritus	Aphrophila neozelandicu s	52	0.04	0.05	0.02	0.04
1	1	0.92	1	cv	1	Unidentified organic	Stictocladius sp.	52	0.04	0.05	0.06	0.05
0	1	0.59	1	t	1	Unidentified organic	Zelandobius confusus	52	0.04	0.05	0.02	0.04
0	0	0.49	1	t	1	Unidentified organic	Deleatidum lilli	52	0.04	0.05	0.01	0.04
1	1	0.86	1	t	1	Unidentified organic	Pycnocentria evecta	52	0.04	0.05	0.05	0.04
0	0	0.47	1	cv	1	Unidentified organic	Hydrophilida e	52	0.04	0.05	0.01	0.04

0	1	0.62	1	t	1	Ulothrix spp.	Aphrophila neozelandicus	52	0.04	0.04	0.02	0.04
1	1	0.88	1	t	1	Austroperla cyrene	Terrestrial plant material	52	0.07	0.05	0.03	0.04
1	1	0.80	1	t	1	Austroperla cyrene	Cocconeis placentual	52	0.07	0.05	0.01	0.05
1	1	0.86	1	cv	1	Austroperla cyrene	Navicula avenacea	52	0.07	0.05	0.02	0.05
0	0	0.45	1	t	1	Terrestrial plant material	Potamopyrgus antipodarum	52	0.03	0.04	0.02	0.04
0	0	0.50	1	t	1	Terrestrial plant material	Aphrophila neozelandicus	52	0.03	0.04	0.02	0.04
0	1	0.86	1	t	1	Terrestrial plant material	Stictocladius sp.	52	0.03	0.04	0.06	0.05
0	1	0.60	1	cv	1	Terrestrial plant material	Paranephrops zelandicus	52	0.03	0.04	0.03	0.04
0	0	0.45	1	t	1	Terrestrial plant material	Zelandobius confusus	52	0.03	0.04	0.02	0.04
0	0	0.35	1	t	1	Terrestrial plant material	Deleatidum lilli	52	0.03	0.04	0.01	0.04
1	1	0.78	1	t	1	Terrestrial plant material	Pycnocentria evecta	52	0.03	0.04	0.05	0.04
0	0	0.39	1	cv	1	Naonella forsythii	Navicula avenacea	52	0.02	0.04	0.02	0.05
0	0	0.39	1	t	1	Nitzschia linearis	Potamopyrgus antipodarum	52	0.02	0.04	0.02	0.04
0	0	0.29	1	t	1	Nitzschia linearis	Deleatidum lilli	52	0.02	0.04	0.01	0.04
0	0	0.45	1	t	1	Fragilaria vaucheriae	Potamopyrgus antipodarum	52	0.02	0.04	0.02	0.04
0	0	0.34	1	cv	1	Fragilaria vaucheriae	Deleatidum lilli	52	0.02	0.04	0.01	0.04
1	1	0.65	1	t	1	Galaxias brevipennis	Deleatidum lilli	52	0.05	0.04	0.01	0.04
0	0	0.32	1	t	1	Potamopyrgus antipodarum	Cocconeis placentual	52	0.02	0.04	0.01	0.05
0	0	0.41	1	t	1	Potamopyrgus antipodarum	Navicula avenacea	52	0.02	0.04	0.02	0.05
0	0	0.43	1	cv	1	Potamopyrgus antipodarum	Salmo trutta	52	0.02	0.04	0.02	0.04
0	1	0.52	1	t	1	Isopoda	Navicula avenacea	52	0.03	0.04	0.02	0.05
0	0	0.37	1	t	1	Stenoperla prasinia	Salmo trutta	52	0.01	0.04	0.02	0.04

0	0	0.39	1	t	1	Stenoperla prasinia	Megaleptoperla grandis	52	0.01	0.04	0.03	0.05
0	0	0.24	1	cv	1	Hydrobiosella stenocerca	Deleatidum lilli	52	0.01	0.03	0.01	0.04
0	0	0.40	1	t	1	Hydrobiosis	Salmo trutta	52	0.01	0.03	0.02	0.04
0	1	0.57	1	t	1	Gomphonema parvulum	Aphrophila neozelandicus	52	0.03	0.04	0.02	0.04
0	0	0.36	1	t	1	Cocconeis placentual	Aphrophila neozelandicus	52	0.01	0.05	0.02	0.04
0	0	0.23	1	cv	1	Cocconeis placentual	Deleatidum lilli	52	0.01	0.05	0.01	0.04
1	1	0.81	1	t	1	Navicula placentula	Aphrophila neozelandicus	52	0.06	0.05	0.02	0.04
0	0	0.45	1	t	1	Aphrophila neozelandicus	Navicula avenacea	52	0.02	0.04	0.02	0.05
0	1	0.61	1	t	1	Aphrophila neozelandicus	Melosira granulata	52	0.02	0.04	0.03	0.03
1	1	0.82	1	cv	1	Stictocladius sp.	Navicula avenacea	52	0.06	0.05	0.02	0.05
0	0	0.40	1	t	1	Navicula avenacea	Zelandobius confusus	52	0.02	0.05	0.02	0.04
0	0	0.31	1	t	1	Navicula avenacea	Deleatidum lilli	52	0.02	0.05	0.01	0.04
0	1	0.57	1	t	1	Salmo trutta	Paranephrops zelandicus	52	0.02	0.04	0.03	0.04
0	0	0.32	1	cv	1	Salmo trutta	Deleatidum lilli	52	0.02	0.04	0.01	0.04
0	1	0.76	1	t	1	Salmo trutta	Pycnocentria evecta	52	0.02	0.04	0.05	0.04
0	0	0.49	1	t	1	Salmo trutta	Megaleptoperla grandis	52	0.02	0.04	0.03	0.05
0	0	0.41	1	t	1	Paranephrops zelandicus	Deleatidum lilli	52	0.03	0.04	0.01	0.04
0	0	0.35	1	cv	1	Deleatidum lilli	Megaleptoperla grandis	52	0.01	0.04	0.03	0.05
1	1	0.78	0	t	1	Rhoicosphenia curvata	Aphrophila neozelandicus	52	0.06	0.05	0.02	0.04
0	0	0.33	0	t	1	Rhabdomastrix sp.	Potamopyrgus antipodarum	52	0.01	0.04	0.02	0.04
0	0	0.23	0	t	1	Frustulia rhomboides	Hydrobiosella stenocerca	52	0.01	0.04	0.01	0.03
0	0	0.41	0	cv	1	Hydraena homolaena	Austrosimulium australense	52	0.03	0.04	0.01	0.04
0	1	0.78	0	t	1	Cymbella aspera	Eukiefldriella brundini	52	0.01	0.04	0.07	0.05

1	1	0.74	0	t	1	Tabellaria flocculosa	Pycnocentria evecta	52	0.02	0.05	0.05	0.04
0	1	0.79	0	t	1	Zelandoperla fenestrata	Lumbriculiid type 'II'	52	0.05	0.04	0.03	0.04
0	1	0.83	0	cv	1	Nitzschia linearis	Stictocladius sp.	52	0.02	0.04	0.06	0.05
0	1	0.71	0	t	1	Batrachospermum spp.	Paranephrops zelandicus	52	0.04	0.04	0.03	0.04
0	0	0.46	0	t	1	Maoridiamesia sp.	Synedra ulna	52	0.03	0.04	0.01	0.04
0	1	0.63	0	t	1	Tabellaria flocculosa	Unidentified organic detritus	52	0.02	0.05	0.04	0.05
0	1	0.65	0	cv	1	Batrachospermum spp.	Megaleptoperla grandis	52	0.04	0.04	0.03	0.05
0	0	0.45	0	t	1	Hydraena homolaena	Zelandobius confusus	52	0.03	0.04	0.02	0.04
0	0	0.23	0	t	1	Gomphonema truncatum	Blue-green algae	52	0.01	0.04	0.01	0.04
0	0	0.22	0	t	1	Blue-green algae	Hydrobiosella stenocerca	52	0.01	0.04	0.01	0.03
1	1	0.70	0	cv	1	Rhoicosphenia curvata	Rhabdomastrix sp.	52	0.06	0.05	0.01	0.04
0	1	0.89	0	t	1	Gomphonema parvulum	Stictocladius sp.	52	0.03	0.04	0.06	0.05
0	1	0.76	0	t	1	Podaena sp.	Pycnocentria evecta	52	0.02	0.04	0.05	0.04
0	0	0.30	0	t	1	Podaena sp.	Hydrophilidae	52	0.02	0.04	0.01	0.04
0	1	0.55	0	cv	1	Surirella elegans	Unidentified organic detritus	52	0.01	0.04	0.04	0.05
1	1	0.87	0	t	1	Rhoicosphenia curvata	Ulothrix spp.	52	0.06	0.05	0.04	0.04
0	0	0.29	0	t	1	Epithemis turgida	Gomphonema subclavatum	52	0.02	0.05	0.01	0.05
0	0	0.25	0	t	1	Hydrobiosis sp.	Hydrophilidae	52	0.01	0.03	0.01	0.04
1	1	0.74	0	cv	1	Unknown green algae	Melosira granulata	52	0.03	0.03	0.03	0.03
0	1	0.81	0	t	1	Surirella elegans	Eukiefliidriella brundini	52	0.01	0.04	0.07	0.05
0	1	0.62	0	t	1	Aoteapsyche raruraru	Pinnularia borealis	52	0.02	0.03	0.04	0.04
0	0	0.39	0	t	1	Hydrobiosella stenocerca	Megaleptoperla grandis	52	0.01	0.03	0.03	0.05
0	1	0.70	0	cv	1	Paracalliope fluviatalus	Ulothrix spp.	52	0.03	0.04	0.04	0.04
1	1	0.91	0	t	1	Unidentified organic	Navicula placentula	52	0.04	0.05	0.06	0.05

0	0	0.37	0	t	1	Aoteapsyche raruraru	Gomphonema angustatum	52	0.02	0.03	0.01	0.04
0	0	0.23	0	t	1	Cymbella aspera	Cricotopus sp. Type 'pale'	52	0.01	0.04	0.01	0.04
0	0	0.35	0	cv	1	Stenoperla prasinia	Navicula avenacea	52	0.01	0.04	0.02	0.05
1	1	0.97	0	t	1	Austroperla cyrene	Navicula placentula	52	0.07	0.05	0.06	0.05
0	0	0.50	0	t	1	Aoteapsyche raruraru	Coloburiscus humeralis	52	0.02	0.03	0.03	0.04
0	1	0.80	0	t	1	Blue-green algae	Austroperla cyrene	52	0.01	0.04	0.07	0.05
0	0	0.26	0	cv	1	Naonella forsythii	Hydrophilidae	52	0.02	0.04	0.01	0.04
0	0	0.44	0	t	1	Ameletopsis perscitus	Isopoda	52	0.01	0.04	0.03	0.04
0	1	0.55	0	t	1	Batrachospermum spp.	Epithemis turgida	52	0.04	0.04	0.02	0.05
0	0	0.41	0	t	1	Hudsonema amabilis	Coloburiscus humeralis	52	0.01	0.04	0.03	0.04
0	1	0.57	0	cv	1	Gomphonema parvulum	Salmo trutta	52	0.03	0.04	0.02	0.04
1	1	0.70	0	t	1	Rhoicosphenia curvata	Stenoperla prasinia	52	0.06	0.05	0.01	0.04
0	1	0.58	0	t	1	Batrachospermum spp.	Potamopyrgus antipodarum	52	0.04	0.04	0.02	0.04
0	1	0.54	0	t	1	Ulothrix spp.	Hydrobiosis sp.	52	0.04	0.04	0.01	0.03
0	1	0.58	0	cv	1	Coloburiscus humeralis	Isopoda	52	0.03	0.04	0.03	0.04
0	1	0.65	0	t	1	Pinnularia borealis	Megaleptoperla grandis	52	0.04	0.04	0.03	0.05
0	1	0.74	0	t	1	Eukiefidriella brundini	Hydrophilidae	52	0.07	0.05	0.01	0.04
0	0	0.27	0	t	1	Gomphonema angustatum	Terrestrial invertebrate remains	52	0.01	0.04	0.01	0.03
0	0	0.41	0	cv	1	Gomphonema sp. II	Gomphonema parvulum	52	0.01	0.04	0.03	0.04
0	0	0.48	0	t	1	Hudsonema amabilis	Hydora nitida	52	0.01	0.04	0.03	0.04
0	1	0.82	0	t	1	Cristaperla fimbria	Stictocladius sp.	52	0.02	0.04	0.06	0.05
0	0	0.35	0	t	1	Hudsonema amabilis	Navicula avenacea	52	0.01	0.04	0.02	0.05
0	0	0.34	0	cv	1	Aoteapsyche raruraru	Cocconeis placentual	52	0.02	0.03	0.01	0.05
1	1	0.81	0	t	1	Navicula placentula	Salmo trutta	52	0.06	0.05	0.02	0.04

0	1	0.78	0	t	1	Synedra rumpens	Pinnularia borealis	52	0.04	0.04	0.04	0.04
0	1	0.64	0	t	1	Deleatidum lilli	Pycnocentria evecta	52	0.01	0.04	0.05	0.04
0	1	0.85	0	cv	1	Aphrophila neozelandicus	Stictocladius sp.	52	0.02	0.04	0.06	0.05
0	1	0.79	0	t	1	Scirtidae type 'broad'	Galaxias brevipennis	52	0.02	0.04	0.05	0.04
0	1	0.62	0	t	1	Tabellaria flocculosa	Synedra rumpens	52	0.02	0.05	0.04	0.04
0	0	0.35	0	t	1	Hydrobiosis sp.	Zelandobius confusus	52	0.01	0.03	0.02	0.04
0	0	0.36	0	cv	1	Epithemis turgida	Nitzschia linearis	52	0.02	0.05	0.02	0.04
0	0	0.25	0	t	1	Blue-green algae	Hydrobiosis sp.	52	0.01	0.04	0.01	0.03
0	1	0.71	0	t	1	Epithemis turgida	Pycnocentria evecta	52	0.02	0.05	0.05	0.04
0	1	0.64	0	t	1	Podaena sp.	Synedra rumpens	52	0.02	0.04	0.04	0.04
0	1	0.78	0	cv	1	Batrachosper mum spp.	Unidentified organic detritus	52	0.04	0.04	0.04	0.05
0	0	0.41	0	t	1	Ameletopsis perscitus	Terrestrial plant material	52	0.01	0.04	0.03	0.04
0	0	0.47	0	t	1	Synedra rumpens	Hydrophilida e	52	0.04	0.04	0.01	0.04
0	0	0.46	0	t	1	Podaena sp.	Salmo trutta	52	0.02	0.04	0.02	0.04
0	1	0.85	0	cv	1	Lumbriculiid type 'II'	Galaxias brevipennis	52	0.03	0.04	0.05	0.04
0	1	0.55	0	t	1	Batrachosper mum spp.	Hydrobiosis sp.	52	0.04	0.04	0.01	0.03
1	1	0.81	0	t	1	Epithemis turgida	Stictocladius sp.	52	0.02	0.05	0.06	0.05
0	0	0.45	0	t	1	Hydraena homolaena	Potamopyrgu s antipodarum	52	0.03	0.04	0.02	0.04
0	1	0.97	0	cv	1	Oedogonium spp.	Galaxias brevipennis	52	0.08	0.07	0.05	0.04
0	1	0.62	0	t	1	Lumbriculiid type 'II'	Coloburiscus humeralis	52	0.03	0.04	0.03	0.04
0	0	0.25	0	t	1	Gomphonema sp. II	Hydrobiosis sp.	52	0.01	0.04	0.01	0.03
0	1	0.62	0	t	1	Maoridiamesi a sp.	Isopoda	52	0.03	0.04	0.03	0.04
0	0	0.22	0	cv	1	Blue-green algae	Cocconeis placentual	52	0.01	0.04	0.01	0.05
0	0	0.33	0	t	1	Gomphonema sp. II	Megaleptope rla grandis	52	0.01	0.04	0.03	0.05

0	1	0.82	0	t	1	Potamopyrgus antipodarum	Stictocladius sp.	52	0.02	0.04	0.06	0.05
0	0	0.28	0	t	1	Gomphonema truncatum	Surirella elegans	52	0.01	0.04	0.01	0.04
1	1	0.81	0	cv	1	Rhoicosphenia curvata	Coloburiscus humeralis	52	0.06	0.05	0.03	0.04
0	1	0.53	0	t	1	Austrosimulium australense	Melosira granulata	52	0.01	0.04	0.03	0.03
0	1	0.58	0	t	1	Lumbriculiid type 'II'	Aphrophila neozelandicus	52	0.03	0.04	0.02	0.04
0	0	0.30	0	t	1	Epithemis turgida	Cricotopus sp. Type 'pale'	52	0.02	0.05	0.01	0.04
0	1	0.91	0	cv	1	Austroperla cyrene	Gomphonema parvulum	52	0.07	0.05	0.03	0.04
0	1	0.75	0	t	1	Frustulia rhomboides	Stictocladius sp.	52	0.01	0.04	0.06	0.05
0	1	0.53	0	t	1	Hudsonema amabilis	Batrachospermum spp.	52	0.01	0.04	0.04	0.04
0	1	0.79	0	t	1	Eukiefildriella brundini	Rhabdomastrix sp.	52	0.07	0.05	0.01	0.04
0	0	0.48	0	cv	1	Epithemis turgida	Paracalliope fluviatalus	52	0.02	0.05	0.03	0.04
0	0	0.40	0	t	1	Cristaperla fimbria	Navicula avenacea	52	0.02	0.04	0.02	0.05
0	0	0.38	0	t	1	Cricotopus sp. Type 'pale'	Fragilaria vaucheriae	52	0.01	0.04	0.02	0.04
0	1	0.50	0	t	1	Unknown green algae	Rhabdomastrix sp.	52	0.03	0.03	0.01	0.04
0	0	0.40	0	cv	1	Frustulia rhomboides	Paracalliope fluviatalus	52	0.01	0.04	0.03	0.04
0	1	0.59	0	t	1	Hydora nitida	Scirtidae type 'broad'	52	0.03	0.04	0.02	0.04
0	0	0.39	0	t	1	Hudsonema amabilis	Megaleptoperla grandis	52	0.01	0.04	0.03	0.05
0	0	0.33	0	t	1	Surirella elegans	Nitzschia linearis	52	0.01	0.04	0.02	0.04
0	0	0.33	0	cv	1	Nitzschia linearis	Stenoperla prasinia	52	0.02	0.04	0.01	0.04
0	1	0.83	0	t	1	Paranephrops zelandicus	Pycnocentria evecta	52	0.03	0.04	0.05	0.04
0	0	0.34	0	t	1	Coloburiscus humeralis	Hydrophilidae	52	0.03	0.04	0.01	0.04
0	0	0.27	0	t	1	Zelandobius confusus	Hydrophilidae	52	0.02	0.04	0.01	0.04
0	1	0.57	0	cv	1	Maoridiamesia sp.	Megaleptoperla grandis	52	0.03	0.04	0.03	0.05
0	0	0.35	0	t	1	Potamopyrgus antipodarum	Hydrobiosis sp.	52	0.02	0.04	0.01	0.03

0	0	0.28	0	t	1	Epithemis turgida	Terrestrial invertebrate remains	52	0.02	0.05	0.01	0.03
0	1	0.53	0	t	1	Potamopyrgus antipodarum	Gomphonema parvulum	52	0.02	0.04	0.03	0.04
0	0	0.45	0	cv	1	Nitzschia linearis	Fragilaria vaucheriae	52	0.02	0.04	0.02	0.04
0	1	0.86	0	t	1	Epithemis turgida	Oedogonium spp.	52	0.02	0.05	0.08	0.07
0	1	0.62	0	t	1	Batrachospermum spp.	Aphrophila neozelandicus	52	0.04	0.04	0.02	0.04
0	0	0.35	0	t	1	Frustulia rhomboides	Megaleptoperla grandis	52	0.01	0.04	0.03	0.05
0	0	0.30	0	cv	1	Epithemis turgida	Rhabdomastrix sp.	52	0.02	0.05	0.01	0.04
0	0	0.35	0	t	1	Aoteapsyche raruraru	Hudsonema amabilis	52	0.02	0.03	0.01	0.04
0	0	0.32	0	t	1	Surirella elegans	Zelandobius confusus	52	0.01	0.04	0.02	0.04
0	0	0.33	0	t	1	Cyclotella sp.	Scirtidae type 'broad'	52	0.01	0.04	0.02	0.04
0	1	0.71	0	cv	1	Hydora nitida	Unknown green algae	52	0.03	0.04	0.03	0.03
0	1	0.91	0	t	1	Tanytarsini sp.	Oedogonium spp.	52	0.03	0.04	0.08	0.07
0	0	0.49	0	t	1	Gomphonema truncatum	Hydora nitida	52	0.01	0.04	0.03	0.04
0	0	0.45	0	t	1	Austrosimulium australense	Isopoda	52	0.01	0.04	0.03	0.04
0	0	0.46	0	cv	1	Cyclotella sp.	Melosira granulata	52	0.01	0.04	0.03	0.03
0	1	0.72	0	t	1	Zelandobius confusus	Pycnocentria evecta	52	0.02	0.04	0.05	0.04
0	1	0.85	0	t	1	Scirtidae type 'broad'	Stictocladius sp.	52	0.02	0.04	0.06	0.05
0	0	0.31	0	t	1	Cocconeis placentual	Zelandobius confusus	52	0.01	0.05	0.02	0.04
0	1	0.87	0	cv	1	Paracalliope fluviatalus	Navicula placentula	52	0.03	0.04	0.06	0.05
0	0	0.36	0	t	1	Aoteapsyche raruraru	Ameletopsis perscitus	52	0.02	0.03	0.01	0.04
0	1	0.68	0	t	1	Synedra rumpens	Coloburiscus humeralis	52	0.04	0.04	0.03	0.04
0	1	0.88	0	t	1	Austroperla cyrene	Fragilaria vaucheriae	52	0.07	0.05	0.02	0.04
0	1	0.72	0	cv	1	Cyclotella sp.	Navicula placentula	52	0.01	0.04	0.06	0.05
0	0	0.29	0	t	1	Surirella	Synedra ulna	52	0.01	0.04	0.01	0.04

0	0	0.35	0	t	1	Blue-green algae	Terrestrial plant material	52	0.01	0.04	0.03	0.04
0	0	0.41	0	t	1	Lumbriculiid type 'II'	Hydrophilidae	52	0.03	0.04	0.01	0.04
0	1	0.54	0	cv	1	Maoridiamesia sp.	Podaena sp.	52	0.03	0.04	0.02	0.04
1	1	0.68	0	t	1	Tiphobiosis montana	Coloburiscus humeralis	52	0.04	0.03	0.03	0.04
0	1	0.59	0	t	1	Paracalliope fluviatalus	Coloburiscus humeralis	52	0.03	0.04	0.03	0.04
0	1	0.79	0	t	1	Synedra rumpens	Unidentified organic detritus	52	0.04	0.04	0.04	0.05
0	0	0.28	0	cv	1	Gomphonema sp. II	Potamopyrgus antipodarum	52	0.01	0.04	0.02	0.04
0	1	0.57	0	t	1	Cristaperla fimbria	Ulothrix spp.	52	0.02	0.04	0.04	0.04
0	0	0.24	0	t	1	Gomphonema sp. II	Amphipoda type 'purple'	52	0.01	0.04	0.02	0.06
0	0	0.42	0	t	1	Frustulia rhomboides	Paranephrops zelandicus	52	0.01	0.04	0.03	0.04
0	0	0.46	0	cv	1	Cricotopus sp. Type 'pale'	Paranephrops zelandicus	52	0.01	0.04	0.03	0.04
0	0	0.33	0	t	1	Ameletopsis perscitus	Zelandobius confusus	52	0.01	0.04	0.02	0.04
0	0	0.33	0	t	1	Scirtidae type 'broad'	Deleatidum lilli	52	0.02	0.04	0.01	0.04
0	0	0.36	0	t	1	Frustulia rhomboides	Terrestrial plant material	52	0.01	0.04	0.03	0.04
0	0	0.35	0	cv	1	Gomphonema angustatum	Zelandobius confusus	52	0.01	0.04	0.02	0.04
0	0	0.50	0	t	1	Cyclotella sp.	Unidentified organic detritus	52	0.01	0.04	0.04	0.05
0	1	0.50	0	t	1	Nitzschia linearis	Isopoda	52	0.02	0.04	0.03	0.04
0	0	0.45	0	t	1	Nitzschia linearis	Megaleptoperla grandis	52	0.02	0.04	0.03	0.05
0	1	0.55	0	cv	1	Tabellaria flocculosa	Paranephrops zelandicus	52	0.02	0.05	0.03	0.04
0	0	0.31	0	t	1	Rhabdomastrix sp.	Naonella forsythii	52	0.01	0.04	0.02	0.04
0	1	0.92	0	t	1	Eukieflidriella brundini	Pinnularia borealis	52	0.07	0.05	0.04	0.04
0	0	0.41	0	t	1	Gomphonema truncatum	Coloburiscus humeralis	52	0.01	0.04	0.03	0.04
0	0	0.31	0	cv	1	Gomphonema angustatum	Amphipoda type 'purple'	52	0.01	0.04	0.02	0.06

0	1	0.65	0	t	1	Coloburiscus humeralis	Melosira granulata	52	0.03	0.04	0.03	0.03
0	1	0.83	0	t	1	Eukiefliidriella brundini	Nitzschia linearis	52	0.07	0.05	0.02	0.04
0	1	0.78	0	t	1	Eukiefliidriella brundini	Hydrobiosella stenocerca	52	0.07	0.05	0.01	0.03
0	0	0.38	0	cv	1	Podaena sp.	Hydrobiosis sp.	52	0.02	0.04	0.01	0.03
0	1	0.72	0	t	1	Hydora nitida	Ulothrix spp.	52	0.03	0.04	0.04	0.04
1	1	0.86	0	t	1	Galaxias brevipennis	Melosira granulata	52	0.05	0.04	0.03	0.03
0	1	0.83	0	t	1	Austroperla cyrene	Hydrobiosis sp.	52	0.07	0.05	0.01	0.03
0	1	0.90	0	cv	1	Unknown green algae	Navicula placentula	52	0.03	0.03	0.06	0.05
0	1	0.55	0	t	1	Zelandoperla fenestrata	Frustulia rhomboides	52	0.05	0.04	0.01	0.04
0	0	0.41	0	t	1	Blue-green algae	Paranephrops zelandicus	52	0.01	0.04	0.03	0.04
0	0	0.31	0	t	1	Gomphonema sp. II	Salmo trutta	52	0.01	0.04	0.02	0.04
0	0	0.35	0	cv	1	Tanytarsini sp.	Gomphonema sp. II	52	0.03	0.04	0.01	0.04
0	0	0.47	0	t	1	Batrachospermum spp.	Frustulia rhomboides	52	0.04	0.04	0.01	0.04
0	1	0.58	0	t	1	Synedra rumpens	Naonella forsythii	52	0.04	0.04	0.02	0.04
0	0	0.25	0	t	1	Cyclotella sp.	Gomphonema angustatum	52	0.01	0.04	0.01	0.04
0	0	0.42	0	cv	1	Hydrobiosis sp.	Megaleptoperla grandis	52	0.01	0.03	0.03	0.05
0	1	0.56	0	t	1	Zelandoperla fenestrata	Deleatidum lilli	52	0.05	0.04	0.01	0.04
0	0	0.29	0	t	1	Hudsonema amabilis	Austrosimulium australense	52	0.01	0.04	0.01	0.04
0	0	0.35	0	t	1	Podaena sp.	Gomphonema subclavatum	52	0.02	0.04	0.01	0.05
0	0	0.23	0	cv	1	Gomphonema truncatum	Cymbella aspera	52	0.01	0.04	0.01	0.04
0	0	0.45	0	t	1	Gomphonema truncatum	Paracalliope fluviatalus	52	0.01	0.04	0.03	0.04
0	1	0.65	0	t	1	Paracalliope fluviatalus	Gomphonema parvulum	52	0.03	0.04	0.03	0.04
0	1	0.68	0	t	1	Ulothrix spp.	Isopoda	52	0.04	0.04	0.03	0.04
0	0	0.25	0	cv	1	Synedra ulna	Frustulia rhomboides	52	0.01	0.04	0.01	0.04
0	0	0.28	0	t	1	Austrosimulium australense	Hydrobiosella stenocerca	52	0.01	0.04	0.01	0.03

0	1	0.60	0	t	1	Zelandoperla fenestrata	Hudsonema amabilis	52	0.05	0.04	0.01	0.04
0	0	0.23	0	t	1	Surirella elegans	Hydrophilidae	52	0.01	0.04	0.01	0.04
0	1	0.74	0	cv	1	Eukiefliedriella brundini	Gomphonema sp. II	52	0.07	0.05	0.01	0.04
0	1	0.78	0	t	1	Gomphonema subclavatum	Stictocladius sp.	52	0.01	0.05	0.06	0.05
0	1	0.54	0	t	1	Hydraena homolaena	Coloburiscus humeralis	52	0.03	0.04	0.03	0.04
0	1	0.57	0	t	1	Batrachospermum spp.	Cristaperla fimbria	52	0.04	0.04	0.02	0.04
0	0	0.22	0	cv	1	Cyclotella sp.	Terrestrial invertebrate remains	52	0.01	0.04	0.01	0.03
0	1	0.79	0	t	1	Eukiefliedriella brundini	Stenoperla prasinia	52	0.07	0.05	0.01	0.04
0	1	0.59	0	t	1	Unknown green algae	Navicula avenacea	52	0.03	0.03	0.02	0.05
0	1	0.54	0	t	1	Gomphonema truncatum	Pinnularia borealis	52	0.01	0.04	0.04	0.04
0	0	0.44	0	cv	1	Epithemis turgida	Coloburiscus humeralis	52	0.02	0.05	0.03	0.04
0	0	0.33	0	t	1	Cymbella aspera	Fragilaria vaucheriae	52	0.01	0.04	0.02	0.04
0	1	0.83	0	t	1	Rhabdomastrix sp.	Austroperla cyrene	52	0.01	0.04	0.07	0.05
0	0	0.47	0	t	1	Ameletopsis perscitus	Paranephrops zelandicus	52	0.01	0.04	0.03	0.04
0	0	0.26	1	cv	1	Scirtid narrow	Unidentified detritus	53	0.01	0.07	0.02	0.07
0	1	0.62	1	t	1	Scirtid narrow	Plant material	53	0.01	0.07	0.06	0.07
1	1	0.58	1	t	1	Surirella biserrata	Deleatidium	53	0.01	0.04	0.04	0.04
0	0	0.46	1	t	1	Surirella biserrata	Potamopyrgus	53	0.01	0.04	0.03	0.05
0	1	0.95	1	cv	1	Corynoneura	Unidentified detritus	53	0.11	0.09	0.02	0.07
1	1	0.99	1	t	1	Corynoneura	Plant material	53	0.11	0.09	0.06	0.07
0	0	0.27	1	t	1	Tabellaria flocculosa	Austroperla	53	0.01	0.04	0.01	0.06
0	1	0.51	1	t	1	Tabellaria flocculosa	Hudsonema aliena	53	0.01	0.04	0.03	0.04
0	0	0.37	1	cv	1	Tabellaria flocculosa	Unknown green algal cells	53	0.01	0.04	0.02	0.05
0	0	0.28	1	t	1	Oligo II	Nannochorista	53	0.02	0.06	0.01	0.05
0	0	0.23	1	t	1	Oligo II	Nothodixa	53	0.02	0.06	0.01	0.05

0	0	0.33	1	t	1	Oligo II	Unidentified detritus	53	0.02	0.06	0.02	0.07
0	0	0.44	1	cv	1	Oligo II	Polyplectropu s	53	0.02	0.06	0.03	0.05
0	0	0.23	1	t	1	Oligo II	Stenoperla	53	0.02	0.06	0.01	0.05
0	0	0.29	1	t	1	Oligo II	Crayfish	53	0.02	0.06	0.02	0.07
0	1	0.70	1	t	1	Oligo II	Plant material	53	0.02	0.06	0.06	0.07
0	0	0.29	1	cv	1	Oligo II	Cristaperla	53	0.02	0.06	0.02	0.07
1	1	0.82	1	t	1	Blue green algae	Deleatidium	53	0.04	0.04	0.04	0.04
1	1	0.73	1	t	1	Blue green algae	Potamopyrgu s	53	0.04	0.04	0.03	0.05
0	0	0.37	1	t	1	Austroperla	Rhoicospheni a curvata	53	0.01	0.06	0.03	0.05
0	0	0.41	1	cv	1	Austroperla	Pinnularia mesolepta	53	0.01	0.06	0.03	0.04
0	0	0.29	1	t	1	Austroperla	Unidentified detritus	53	0.01	0.06	0.02	0.07
0	0	0.20	1	t	1	Austroperla	Frustulia rhomboides	53	0.01	0.06	0.01	0.05
0	1	0.66	1	t	1	Austroperla	Plant material	53	0.01	0.06	0.06	0.07
0	0	0.33	1	cv	1	Austroperla	Unknown green algal cells	53	0.01	0.06	0.02	0.05
0	1	0.68	1	t	1	Deleatidium	Rhoicospheni a curvata	53	0.04	0.04	0.03	0.05
0	1	0.52	1	t	1	Deleatidium	Synedra ulna	53	0.04	0.04	0.01	0.06
1	1	0.73	1	t	1	Deleatidium	Galaxias	53	0.04	0.04	0.03	0.04
0	1	0.55	1	cv	1	Deleatidium	Navicula avenacea	53	0.04	0.04	0.01	0.05
1	1	0.64	1	t	1	Deleatidium	Gomphonem a sp.3	53	0.04	0.04	0.02	0.04
0	1	0.59	1	t	1	Deleatidium	Unidentified detritus	53	0.04	0.04	0.02	0.07
0	1	0.55	1	t	1	Deleatidium	Calothrix	53	0.04	0.04	0.01	0.05
0	1	0.68	1	cv	1	Deleatidium	Eunotia pectinalis	53	0.04	0.04	0.02	0.04
0	1	0.70	1	t	1	Deleatidium	Polyplectropu s	53	0.04	0.04	0.03	0.05
0	1	0.77	1	t	1	Deleatidium	Diploneis ovalis	53	0.04	0.04	0.03	0.04
0	0	0.46	1	t	1	Deleatidium	Stenoperla	53	0.04	0.04	0.01	0.05
0	0	0.45	1	cv	1	Deleatidium	Achnanthes minutissima	53	0.04	0.04	0.01	0.06
0	1	0.51	1	t	1	Deleatidium	Cocconeis placentula	53	0.04	0.04	0.01	0.06
0	1	0.63	1	t	1	Deleatidium	Unknown green algal cells	53	0.04	0.04	0.02	0.05

0	0	0.47	1	t	1	Deleatidium	Pinnularia viridis	53	0.04	0.04	0.01	0.05
0	1	0.71	1	cv	1	Deleatidium	Navicula rhynocephala	53	0.04	0.04	0.03	0.04
0	1	0.86	1	t	1	Hydora nitida	Rhoicospheni a curvata	53	0.07	0.06	0.03	0.05
0	1	0.76	1	t	1	Hydora nitida	Synedra ulna	53	0.07	0.06	0.01	0.06
0	1	0.81	1	t	1	Hydora nitida	Unidentified detritus	53	0.07	0.06	0.02	0.07
0	1	0.71	1	cv	1	Hydora nitida	Achnanthes minutissima	53	0.07	0.06	0.01	0.06
0	1	0.76	1	t	1	Hydora nitida	Cocconeis placentula	53	0.07	0.06	0.01	0.06
0	1	0.95	1	t	1	Hydora nitida	Plant material	53	0.07	0.06	0.06	0.07
0	1	0.53	1	t	1	Hudsonema aliena	Unidentified detritus	53	0.03	0.04	0.02	0.07
0	1	0.74	1	cv	1	Hudsonema aliena	Achnanthes lanceolata	53	0.03	0.04	0.04	0.04
0	0	0.37	1	t	1	Mischoderus	Unidentified detritus	53	0.02	0.05	0.02	0.07
0	0	0.49	1	t	1	Mischoderus	Polypedellum II	53	0.02	0.05	0.03	0.05
0	0	0.31	1	t	1	Nannochorista	Unidentified detritus	53	0.01	0.05	0.02	0.07
0	0	0.37	1	cv	1	Nannochorista	Paracalliope fluv.	53	0.01	0.05	0.03	0.06
0	0	0.19	1	t	1	Nannochorista	Paracalliope purple	53	0.01	0.05	0.01	0.06
0	0	0.42	1	t	1	Nannochorista	Polypedellum II	53	0.01	0.05	0.03	0.05
0	0	0.45	1	t	1	Hydra	Unidentified detritus	53	0.03	0.05	0.02	0.07
0	0	0.23	1	cv	1	Zelandobius	Unidentified detritus	53	0.01	0.06	0.02	0.07
0	0	0.35	1	t	1	Rhoicosphenia curvata	Paucispiniger a	53	0.03	0.05	0.01	0.06
0	0	0.49	1	t	1	Rhoicosphenia curvata	Unknown green algal cells	53	0.03	0.05	0.02	0.05
0	0	0.49	1	t	1	Epithemia 'suqare ended	Neozephlebia	53	0.03	0.04	0.02	0.05
0	0	0.24	1	cv	1	Stictocladius I	Navicula avenacea	53	0.01	0.06	0.01	0.05
0	0	0.27	1	t	1	Stictocladius I	Unidentified detritus	53	0.01	0.06	0.02	0.07
0	0	0.24	1	t	1	Stictocladius I	Calothrix	53	0.01	0.06	0.01	0.05
0	1	0.64	1	t	1	Stictocladius I	Plant material	53	0.01	0.06	0.06	0.07
0	0	0.19	1	cv	1	Stictocladius I	Pinnularia viridis	53	0.01	0.06	0.01	0.05

0	0	0.40	1	t	1	Molophilus	Unidentified detritus	53	0.03	0.07	0.02	0.07
0	0	0.26	1	t	1	Molophilus	Ulothrix	53	0.03	0.07	0.01	0.06
0	1	0.76	1	t	1	Molophilus	Plant material	53	0.03	0.07	0.06	0.07
0	0	0.26	1	cv	1	Molophilus	Gomphoneis herculeana	53	0.03	0.07	0.01	0.06
0	0	0.25	1	t	1	Molophilus	Oligo smooth	53	0.03	0.07	0.01	0.07
0	0	0.39	1	t	1	Galaxias	Nothodixa	53	0.03	0.04	0.01	0.05
0	1	0.61	1	t	1	Pinnularia mesolepta	Potamopyrgu s	53	0.03	0.04	0.03	0.05
0	0	0.24	1	cv	1	Navicula avenacea	Lumbr Pinkk	53	0.01	0.05	0.01	0.07
0	0	0.44	1	t	1	Navicula avenacea	Potamopyrgu s	53	0.01	0.05	0.03	0.05
0	0	0.26	1	t	1	Nothodixa	Unidentified detritus	53	0.01	0.05	0.02	0.07
0	0	0.30	1	t	1	Nothodixa	Oligo I	53	0.01	0.05	0.03	0.06
0	0	0.39	1	cv	1	Hydrobiosis parumbripenn	Unidentified detritus	53	0.02	0.06	0.02	0.07
0	0	0.20	1	t	1	Unidentified detritus	Hexatomini	53	0.02	0.07	0.01	0.07
0	0	0.26	1	t	1	Unidentified detritus	Paucispiniger a	53	0.02	0.07	0.01	0.06
0	0	0.33	1	t	1	Unidentified detritus	Forcipomyiin ae	53	0.02	0.07	0.02	0.06
0	0	0.22	1	cv	1	Unidentified detritus	Stictocladius III	53	0.02	0.07	0.01	0.06
0	0	0.42	1	t	1	Unidentified detritus	Paracalliope fluv.	53	0.02	0.07	0.03	0.06
0	0	0.30	1	t	1	Unidentified detritus	Eriopterini	53	0.02	0.07	0.02	0.07
0	0	0.40	1	t	1	Unidentified detritus	Zelandotipula	53	0.02	0.07	0.03	0.07
0	0	0.34	1	cv	1	Unidentified detritus	Neozephlebia	53	0.02	0.07	0.02	0.05
0	0	0.24	1	t	1	Unidentified detritus	Podonomid type hairy	53	0.02	0.07	0.01	0.05
0	0	0.20	1	t	1	Unidentified detritus	Naonella	53	0.02	0.07	0.01	0.07
0	0	0.26	1	t	1	Unidentified detritus	Lumbr Pinkk	53	0.02	0.07	0.01	0.07
0	1	0.91	1	cv	1	Unidentified detritus	Psychoda	53	0.02	0.07	0.09	0.07
0	0	0.31	1	t	1	Unidentified detritus	Crayfish	53	0.02	0.07	0.02	0.07
0	0	0.26	1	t	1	Unidentified detritus	Paralimnophil a	53	0.02	0.07	0.01	0.07
0	0	0.47	1	t	1	Unidentified detritus	Potamopyrgu s	53	0.02	0.07	0.03	0.05

0	0	0.25	1	cv	1	Unidentified detritus	Tanytarsus	53	0.02	0.07	0.01	0.07
0	0	0.22	1	t	1	Unidentified detritus	Paracalliope purple	53	0.02	0.07	0.01	0.06
0	0	0.39	1	t	1	Unidentified detritus	Unknown green algal cells	53	0.02	0.07	0.02	0.05
0	0	0.22	1	t	1	Unidentified detritus	Cricotopus II	53	0.02	0.07	0.01	0.06
0	0	0.33	1	cv	1	Unidentified detritus	Eukiefiriella	53	0.02	0.07	0.02	0.06
0	0	0.47	1	t	1	Unidentified detritus	Polypedellum II	53	0.02	0.07	0.03	0.05
0	0	0.41	1	t	1	Unidentified detritus	Oligo I	53	0.02	0.07	0.03	0.06
0	0	0.38	1	t	1	Unidentified detritus	Hydora adult	53	0.02	0.07	0.02	0.06
0	0	0.21	1	cv	1	Unidentified detritus	Oligo smooth	53	0.02	0.07	0.01	0.07
0	0	0.30	1	t	1	Unidentified detritus	Cristaperla	53	0.02	0.07	0.02	0.07
0	1	0.56	1	t	1	Hexatomini	Plant material	53	0.01	0.07	0.06	0.07
0	0	0.30	1	t	1	Calothrix	Forcipomyiinae	53	0.01	0.05	0.02	0.06
0	0	0.22	1	cv	1	Paucispinigera	Cocconeis placentula	53	0.01	0.06	0.01	0.06
0	1	0.64	1	t	1	Paucispinigera	Plant material	53	0.01	0.06	0.06	0.07
0	1	0.70	1	t	1	Forcipomyiinae	Plant material	53	0.02	0.06	0.06	0.07
0	0	0.24	1	t	1	Forcipomyiinae	Pinnularia viridis	53	0.02	0.06	0.01	0.05
0	0	0.46	1	cv	1	Forcipomyiinae	Navicula rhynocephala	53	0.02	0.06	0.03	0.04
0	0	0.50	1	t	1	Eunotia pectinalis	Unknown green algal cells	53	0.02	0.04	0.02	0.05
0	0	0.45	1	t	1	Gomphonema intricatum	Potamopyrgus	53	0.01	0.04	0.03	0.05
0	0	0.45	1	t	1	Diatoma heimale	Potamopyrgus	53	0.01	0.04	0.03	0.05
0	1	0.55	1	cv	1	Polyplectropus	Paracalliope fluv.	53	0.03	0.05	0.03	0.06
0	0	0.32	1	t	1	Polyplectropus	Paracalliope purple	53	0.03	0.05	0.01	0.06
0	0	0.40	1	t	1	Polyplectropus	Terrestrial invertebrates	53	0.03	0.05	0.01	0.03
0	1	0.51	1	t	1	Polyplectropus	Hydora adult	53	0.03	0.05	0.02	0.06
0	0	0.30	1	cv	1	Paracalliope fluv.	Stenoperla	53	0.03	0.06	0.01	0.05

0	1	0.78	1	t	1	Paracalliope fluv.	Plant material	53	0.03	0.06	0.06	0.07
0	0	0.15	1	t	1	Stenoperla	Paracalliope purple	53	0.01	0.05	0.01	0.06
0	0	0.30	1	t	1	Stenoperla	Oligo I	53	0.01	0.05	0.03	0.06
0	0	0.25	1	cv	1	Eriopterini	Cocconeis placentula	53	0.02	0.07	0.01	0.06
0	1	0.68	1	t	1	Eriopterini	Plant material	53	0.02	0.07	0.06	0.07
0	1	0.75	1	t	1	Zelandotipula	Plant material	53	0.03	0.07	0.06	0.07
0	1	0.57	1	t	1	Neozephlebia	Achnanthes lanceolata	53	0.02	0.05	0.04	0.04
0	1	0.72	1	cv	1	Neozephlebia	Plant material	53	0.02	0.05	0.06	0.07
0	0	0.48	1	t	1	Neozephlebia	Navicula rhynocephala	53	0.02	0.05	0.03	0.04
0	0	0.19	1	t	1	Podonomid type hairy	Cocconeis placentula	53	0.01	0.05	0.01	0.06
0	0	0.28	1	t	1	Podonomid type hairy	Unknown green algal cells	53	0.01	0.05	0.02	0.05
0	1	0.57	1	cv	1	Fragilaria vaucheriae	Potamopyrgu s	53	0.02	0.04	0.03	0.05
1	1	0.68	1	t	1	Achnanthes lanceolata	Potamopyrgu s	53	0.04	0.04	0.03	0.05
0	1	0.61	1	t	1	Achnanthes lanceolata	Hydora adult	53	0.04	0.04	0.02	0.06
0	0	0.21	1	t	1	Cocconeis placentula	Paralimnophil a	53	0.01	0.06	0.01	0.07
0	0	0.40	1	cv	1	Cocconeis placentula	Potamopyrgu s	53	0.01	0.06	0.03	0.05
0	1	0.56	1	t	1	Naonella	Plant material	53	0.01	0.07	0.06	0.07
0	0	0.30	1	t	1	Frustulia rhomboides	Unknown green algal cells	53	0.01	0.05	0.02	0.05
0	1	0.63	1	t	1	Lumbr Pinkk	Plant material	53	0.01	0.07	0.06	0.07
1	1	0.98	1	cv	1	Psychoda	Plant material	53	0.09	0.07	0.06	0.07
0	1	0.68	1	t	1	Crayfish	Plant material	53	0.02	0.07	0.06	0.07
0	0	0.37	1	t	1	Crayfish	Oligo I	53	0.02	0.07	0.03	0.06
0	0	0.18	1	t	1	Crayfish	Oligo smooth	53	0.02	0.07	0.01	0.07
0	1	0.60	1	cv	1	Plant material	Paralimnophil a	53	0.06	0.07	0.01	0.07
0	1	0.79	1	t	1	Plant material	Potamopyrgu s	53	0.06	0.07	0.03	0.05
0	1	0.59	1	t	1	Plant material	Tanytarsus	53	0.06	0.07	0.01	0.07

0	1	0.55	1	t	1	Plant material	Paracalliope purple	53	0.06	0.07	0.01	0.06
0	1	0.74	1	cv	1	Plant material	Unknown green algal cells	53	0.06	0.07	0.02	0.05
0	1	0.75	1	t	1	Plant material	Oligo I	53	0.06	0.07	0.03	0.06
0	1	0.73	1	t	1	Plant material	Hydora adult	53	0.06	0.07	0.02	0.06
0	1	0.53	1	t	1	Plant material	Oligo smooth	53	0.06	0.07	0.01	0.07
0	1	0.65	1	cv	1	Plant material	Cristaperla	53	0.06	0.07	0.02	0.07
0	0	0.37	1	t	1	Potamopyrgus	Stauroneis sp.	53	0.03	0.05	0.01	0.04
0	1	0.60	1	t	1	Potamopyrgus	Navicula rhynocephala	53	0.03	0.05	0.03	0.04
0	1	0.82	1	t	1	Potamopyrgus	Nitzschia dissipata	53	0.03	0.05	0.05	0.05
0	0	0.29	1	cv	1	Unknown green algal	Pinnularia viridis	53	0.02	0.05	0.01	0.05
0	0	0.15	1	t	1	Pinnularia	Oligo smooth	53	0.01	0.05	0.01	0.07
0	0	0.35	0	t	1	Epithemia 'suqare ended	Ulothrix	53	0.03	0.04	0.01	0.06
0	0	0.30	0	t	1	Hexatomini	Polyplectropu s	53	0.01	0.07	0.03	0.05
0	0	0.23	0	cv	1	Navicula avenacea	Frustulia rhomboides	53	0.01	0.05	0.01	0.05
0	1	0.57	0	t	1	Blue green algae	Nannochorist a	53	0.04	0.04	0.01	0.05
0	0	0.27	0	t	1	Gomphonema sp.3	Stictocladius III	53	0.02	0.04	0.01	0.06
0	0	0.31	0	t	1	Oligo II	Eukiefiriella	53	0.02	0.06	0.02	0.06
0	0	0.47	0	cv	1	Polyplectropus	Neozephlebia	53	0.03	0.05	0.02	0.05
0	0	0.19	0	t	1	Scirtid narrow	Paucispiniger a	53	0.01	0.07	0.01	0.06
1	0	0.39	0	t	1	Gomphonema sp.3	Gomphonem a intricatum	53	0.02	0.04	0.01	0.04
0	1	0.52	0	t	1	Hydora adult	Navicula rhynocephala	53	0.02	0.06	0.03	0.04
0	0	0.25	0	cv	1	Tabellaria flocculosa	Stictocladius I	53	0.01	0.04	0.01	0.06
0	1	0.93	0	t	1	Corynoneura	Austroperla	53	0.11	0.09	0.01	0.06
0	0	0.23	0	t	1	Scirtid narrow	Forcipomyiin ae	53	0.01	0.07	0.02	0.06
0	1	0.94	0	t	1	Corynoneura	Gomphonem a intricatum	53	0.11	0.09	0.01	0.04
0	0	0.17	0	cv	1	Paucispinigera	Paracalliope purple	53	0.01	0.06	0.01	0.06
0	0	0.38	0	t	1	Hydrobiosis parumbripenn	Eukiefiriella	53	0.02	0.06	0.02	0.06
0	1	0.67	0	t	1	Fragilaria vaucheriae	Achnanthes lanceolata	53	0.02	0.04	0.04	0.04
0	0	0.26	0	t	1	Mischoderus	Nothodixa	53	0.02	0.05	0.01	0.05

0	0	0.22	0	cv	1	Surirella biserrata	Paracalliope purple	53	0.01	0.04	0.01	0.06
0	0	0.45	0	t	1	Oligo II	Navicula rhynocephala	53	0.02	0.06	0.03	0.04
0	0	0.31	0	t	1	Polypedellum	Oligo smooth	53	0.03	0.05	0.01	0.07
0	0	0.19	0	t	1	Nothodixa	Paucispinigera	53	0.01	0.05	0.01	0.06
0	0	0.39	0	cv	1	Diatoma heimale	Oligo I	53	0.01	0.04	0.03	0.06
0	0	0.27	0	t	1	Synedra ulna	Forcipomyiinae	53	0.01	0.06	0.02	0.06
0	1	0.79	0	t	1	Austroperla	Hydora nitida	53	0.01	0.06	0.07	0.06
0	0	0.34	0	t	1	Polyplectropus	Podonomid type hairy	53	0.03	0.05	0.01	0.05
0	0	0.31	0	cv	1	Stictocladius I	Hydrobiosis parumbripennis	53	0.01	0.06	0.02	0.06
0	1	0.86	0	t	1	Diploneis ovalis	Plant material	53	0.03	0.04	0.06	0.07
0	0	0.21	0	t	1	Tabellaria flocculosa	Stictocladius III	53	0.01	0.04	0.01	0.06
0	0	0.32	0	t	1	Hydrobiosis parumbripenn	Cocconeis placentula	53	0.02	0.06	0.01	0.06
0	0	0.24	0	cv	1	Tabellaria flocculosa	Lumbr Pinkk	53	0.01	0.04	0.01	0.07
0	0	0.36	0	t	1	Molophilus	Calothrix	53	0.03	0.07	0.01	0.05
0	0	0.40	0	t	1	Galaxias	Tanytarsus	53	0.03	0.04	0.01	0.07
0	1	0.94	0	t	1	Corynoneura	Crayfish	53	0.11	0.09	0.02	0.07
0	1	0.51	0	cv	1	Hydrobiosis parumbripenn	Polyplectropus	53	0.02	0.06	0.03	0.05
0	0	0.49	0	t	1	Molophilus	Eunotia pectinalis	53	0.03	0.07	0.02	0.04
0	0	0.34	0	t	1	Mischoderus	Calothrix	53	0.02	0.05	0.01	0.05
0	0	0.42	0	t	1	Diatoma heimale	Fragilaria vaucheriae	53	0.01	0.04	0.02	0.04
0	1	0.87	0	cv	1	Lumbr Pinkk	Psychoda	53	0.01	0.07	0.09	0.07
0	0	0.48	0	t	1	Paucispinigera	Achnanthes lanceolata	53	0.01	0.06	0.04	0.04
0	0	0.37	0	t	1	Rhoicosphenia curvata	Cocconeis placentula	53	0.03	0.05	0.01	0.06
0	0	0.27	0	t	1	Ulothrix	Unknown green algal cells	53	0.01	0.06	0.02	0.05
0	0	0.22	0	cv	1	Paucispinigera	Terrestrial invertebrates	53	0.01	0.06	0.01	0.03
0	1	0.71	0	t	1	Gomphonema intricatum	Plant material	53	0.01	0.04	0.06	0.07
1	1	0.50	0	t	1	Achnanthes lanceolata	Terrestrial invertebrates	53	0.04	0.04	0.01	0.03
0	0	0.20	0	t	1	Eriopterini	Achnanthes minutissima	53	0.02	0.07	0.01	0.06

0	0	0.35	0	cv	1	Epithemia 'suqare ended	Gomphoneis herculeana	53	0.03	0.04	0.01	0.06
0	1	0.91	0	t	1	Tabellaria flocculosa	Psychoda	53	0.01	0.04	0.09	0.07
0	1	0.58	0	t	1	Hudsonema aliena	Zelandotipula	53	0.03	0.04	0.03	0.07
0	0	0.32	0	t	1	Stictocladius I	Molophilus	53	0.01	0.06	0.03	0.07
0	1	0.78	0	cv	1	Molophilus	Nitzschia dissipata	53	0.03	0.07	0.05	0.05
0	0	0.34	0	t	1	Paucispinigera	Paracalliope fluv.	53	0.01	0.06	0.03	0.06
0	0	0.30	0	t	1	Forcipomyiinae	Gomphonema intricatum	53	0.02	0.06	0.01	0.04
0	0	0.25	0	t	1	Gomphonema intricatum	Paralimnophila	53	0.01	0.04	0.01	0.07
0	0	0.16	0	cv	1	Stenoperla	Podonomid type hairy	53	0.01	0.05	0.01	0.05
0	0	0.30	0	t	1	Hydrobiosis parumbripenn	Stauroneis sp.	53	0.02	0.06	0.01	0.04
0	1	0.74	0	t	1	Neozephlebia	Nitzschia dissipata	53	0.02	0.05	0.05	0.05
0	0	0.34	0	t	1	Nannochorista	Hydrobiosis parumbripennis	53	0.01	0.05	0.02	0.06
0	1	0.69	0	cv	1	Hydora nitida	Stictocladius III	53	0.07	0.06	0.01	0.06
0	0	0.17	0	t	1	Tanytarsus	Pinnularia viridis	53	0.01	0.07	0.01	0.05
0	0	0.33	0	t	1	Zelandotipula	Terrestrial invertebrates	53	0.03	0.07	0.01	0.03
0	1	0.80	0	t	1	Eunotia pectinalis	Plant material	53	0.02	0.04	0.06	0.07
0	0	0.20	0	cv	1	Calothrix	Stictocladius III	53	0.01	0.05	0.01	0.06
0	1	0.66	0	t	1	Paucispinigera	Nitzschia dissipata	53	0.01	0.06	0.05	0.05
0	0	0.22	0	t	1	Surirella biserrata	Zelandobius	53	0.01	0.04	0.01	0.06
0	1	0.92	0	t	1	Corynoneura	Lumbr Pinkk	53	0.11	0.09	0.01	0.07
0	0	0.20	0	cv	1	Tabellaria flocculosa	Oligo smooth	53	0.01	0.04	0.01	0.07
0	0	0.33	0	t	1	Hydra	Tanytarsus	53	0.03	0.05	0.01	0.07
0	0	0.24	0	t	1	Calothrix	Stauroneis sp.	53	0.01	0.05	0.01	0.04
0	0	0.41	0	t	1	Navicula avenacea	Fragilaria vaucheriae	53	0.01	0.05	0.02	0.04
0	0	0.24	0	cv	1	Tanytarsus	Eukiefiriella	53	0.01	0.07	0.02	0.06
0	0	0.27	0	t	1	Oligo II	Cocconeis placentula	53	0.02	0.06	0.01	0.06
0	0	0.41	0	t	1	Eunotia pectinalis	Crayfish	53	0.02	0.04	0.02	0.07

0	1	0.60	0	t	1	Rhoicosphenia curvata	Galaxias	53	0.03	0.05	0.03	0.04
0	0	0.14	0	cv	1	Achnanthes minutissima	Cricotopus II	53	0.01	0.06	0.01	0.06
0	1	0.54	0	t	1	Pinnularia mesolepta	Zelandotipula	53	0.03	0.04	0.03	0.07
0	1	0.93	0	t	1	Corynoneura	Cocconeis placentula	53	0.11	0.09	0.01	0.06
0	0	0.19	0	t	1	Austroperla	Achnanthes minutissima	53	0.01	0.06	0.01	0.06
0	0	0.39	0	cv	1	Hydrobiosis parumbripenn	Neozephlebia	53	0.02	0.06	0.02	0.05
0	0	0.22	0	t	1	Frustulia rhomboides	Crayfish	53	0.01	0.05	0.02	0.07
0	0	0.32	0	t	1	Nothodixa	Paracalliope fluv.	53	0.01	0.05	0.03	0.06
0	0	0.29	0	t	1	Oligo II	Navicula avenacea	53	0.02	0.06	0.01	0.05
0	0	0.24	0	cv	1	Zelandotipula	Oligo smooth	53	0.03	0.07	0.01	0.07
0	1	0.88	0	t	1	Hydora nitida	Polyplectropu s	53	0.07	0.06	0.03	0.05
0	0	0.14	0	t	1	Gomphoneis herculeana	Cricotopus II	53	0.01	0.06	0.01	0.06
0	1	0.65	0	t	1	Pinnularia viridis	Nitzschia dissipata	53	0.01	0.05	0.05	0.05
0	0	0.14	0	cv	1	Scirtid narrow	Naonella	53	0.01	0.07	0.01	0.07
0	0	0.23	0	t	1	Crayfish	Stauroneis sp.	53	0.02	0.07	0.01	0.04
0	0	0.24	0	t	1	Surirella biserrata	Stenoperla	53	0.01	0.04	0.01	0.05
0	0	0.16	0	t	1	Stauroneis sp.	Oligo smooth	53	0.01	0.04	0.01	0.07
0	1	0.94	0	cv	1	Blue green algae	Hydora nitida	53	0.04	0.04	0.07	0.06
0	1	0.86	0	t	1	Ulothrix	Psychoda	53	0.01	0.06	0.09	0.07
0	0	0.28	0	t	1	Diatoma heimale	Terrestrial invertebrates	53	0.01	0.04	0.01	0.03
0	0	0.12	0	t	1	Hexatomini	Ulothrix	53	0.01	0.07	0.01	0.06
0	0	0.47	0	cv	1	Tabellaria flocculosa	Epithemia 'suqare ended	53	0.01	0.04	0.03	0.04
0	0	0.20	0	t	1	Ulothrix	Crayfish	53	0.01	0.06	0.02	0.07
0	1	0.53	0	t	1	Epithemia 'suqare ended	Hydora adult	53	0.03	0.04	0.02	0.06
0	0	0.21	0	t	1	Forcipomyiina	Ulothrix	53	0.02	0.06	0.01	0.06
0	0	0.28	0	cv	1	Tanytarsus	Hydora adult	53	0.01	0.07	0.02	0.06
0	1	0.91	0	t	1	Hydora nitida	Diploneis ovalis	53	0.07	0.06	0.03	0.04
0	0	0.22	0	t	1	Mischoderus	Naonella	53	0.02	0.05	0.01	0.07
0	0	0.31	0	t	1	Paracalliope fluv.	Frustulia rhomboides	53	0.03	0.06	0.01	0.05
0	0	0.22	0	cv	1	Scirtid narrow	Crayfish	53	0.01	0.07	0.02	0.07

0	0	0.21	0	t	1	Neozephlebia	Oligo smooth	53	0.02	0.05	0.01	0.07
0	0	0.19	0	t	1	Crayfish	Paracalliope purple	53	0.02	0.07	0.01	0.06
0	0	0.48	0	t	1	Paracalliope fluv.	Oligo I	53	0.03	0.06	0.03	0.06
0	0	0.18	0	cv	1	Hexatomini	Eriopterini	53	0.01	0.07	0.02	0.07
0	1	0.57	0	t	1	Eunotia pectinalis	Polypedellum II	53	0.02	0.04	0.03	0.05
0	0	0.44	0	t	1	Calothrix	Polypedellum II	53	0.01	0.05	0.03	0.05
0	0	0.47	0	t	1	Blue green algae	Paracalliope purple	53	0.04	0.04	0.01	0.06
0	0	0.18	0	cv	1	Stictocladius III	Cocconeis placentula	53	0.01	0.06	0.01	0.06
0	0	0.15	0	t	1	Zelandobius	Tanytarsus	53	0.01	0.06	0.01	0.07
0	1	0.57	0	t	1	Unidentified detritus	Achnanthes lanceolata	53	0.02	0.07	0.04	0.04
0	0	0.23	0	t	1	Forcipomyiina	Stenoperla	53	0.02	0.06	0.01	0.05
0	0	0.43	0	cv	1	Deleatidium	Zelandobius	53	0.04	0.04	0.01	0.06
0	0	0.31	0	t	1	Paralimnophil	Oligo I	53	0.01	0.07	0.03	0.06
0	1	0.50	0	t	1	Blue green algae	Stenoperla	53	0.04	0.04	0.01	0.05
0	0	0.34	0	t	1	Fragilaria vaucheriae	Tanytarsus	53	0.02	0.04	0.01	0.07
0	0	0.29	0	cv	1	Eukiefiriella	Cristaperla	53	0.02	0.06	0.02	0.07
0	0	0.31	0	t	1	Navicula avenacea	Eukiefiriella	53	0.01	0.05	0.02	0.06
0	0	0.28	0	t	1	Nothodixa	Hydora adult	53	0.01	0.05	0.02	0.06
1	1	0.65	0	t	1	Diploneis ovalis	Fragilaria vaucheriae	53	0.03	0.04	0.02	0.04
0	0	0.31	0	cv	1	Surirella biserrata	Calothrix	53	0.01	0.04	0.01	0.05
0	0	0.20	0	t	1	Achnanthes minutissima	Terrestrial invertebrates	53	0.01	0.06	0.01	0.03
0	0	0.29	0	t	1	Hydra	Cricotopus II	53	0.03	0.05	0.01	0.06
0	0	0.39	0	t	1	Epithemia 'suqare ended	Paralimnophil a	53	0.03	0.04	0.01	0.07
0	0	0.29	0	cv	1	Eriopterini	Eukiefiriella	53	0.02	0.07	0.02	0.06
0	0	0.18	0	t	1	Nothodixa	Pinnularia viridis	53	0.01	0.05	0.01	0.05
0	0	0.32	0	t	1	Surirella biserrata	Forcipomyiin ae	53	0.01	0.04	0.02	0.06
0	0	0.33	0	t	1	Cricotopus II	Navicula rhynocephala	53	0.01	0.06	0.03	0.04
0	0	0.26	0	cv	1	Synedra ulna	Calothrix	53	0.01	0.06	0.01	0.05
0	1	0.93	0	t	1	Corynoneura	Nannochorist a	53	0.11	0.09	0.01	0.05
0	1	0.72	0	t	1	Unidentified detritus	Plant material	53	0.02	0.07	0.06	0.07
0	0	0.45	0	t	1	Epithemia 'suqare ended	Crayfish	53	0.03	0.04	0.02	0.07

0	0	0.50	0	cv	1	Fragilaria vaucheriae	Unknown green algal cells	53	0.02	0.04	0.02	0.05
0	0	0.27	0	t	1	Mischoderus	Pinnularia viridis	53	0.02	0.05	0.01	0.05
0	0	0.44	0	t	1	Surirella biserrata	Hydra	53	0.01	0.04	0.03	0.05
0	1	0.63	0	t	1	Epithemia 'suqare ended	Pinnularia mesolepta	53	0.03	0.04	0.03	0.04
0	1	0.64	0	cv	1	Diploneis ovalis	Paracalliope fluv.	53	0.03	0.04	0.03	0.06
0	1	0.63	0	t	1	Podonomid type hairy	Nitzschia dissipata	53	0.01	0.05	0.05	0.05
0	1	0.51	0	t	1	Zelandotipula	Polypedellum II	53	0.03	0.07	0.03	0.05
0	0	0.28	0	t	1	Rhoicosphenia curvata	Oligo smooth	53	0.03	0.05	0.01	0.07
0	0	0.18	0	cv	1	Nothodixa	Tanytarsus	53	0.01	0.05	0.01	0.07
0	1	0.58	0	t	1	Hudsonema aliena	Unknown green algal cells	53	0.03	0.04	0.02	0.05
0	1	0.82	0	t	1	Psychoda	Cricotopus II	53	0.09	0.07	0.01	0.06
0	1	0.91	0	t	1	Psychoda	Unknown green algal cells	53	0.09	0.07	0.02	0.05
0	1	0.94	0	cv	1	Hydra	Psychoda	53	0.03	0.05	0.09	0.07
0	0	0.32	0	t	1	Oligo II	Neozephlebia	53	0.02	0.06	0.02	0.05
0	0	0.13	0	t	1	Cricotopus II	Oligo smooth	53	0.01	0.06	0.01	0.07
0	0	0.17	0	t	1	Paucispinigera	Gomphoneis herculeana	53	0.01	0.06	0.01	0.06
0	0	0.23	0	cv	1	Oligo II	Pinnularia viridis	53	0.02	0.06	0.01	0.05
0	0	0.35	0	t	1	Nannochorista	Zelandotipula	53	0.01	0.05	0.03	0.07
0	1	0.61	0	t	1	Achnanthes minutissima	Plant material	53	0.01	0.06	0.06	0.07
0	0	0.44	0	t	1	Surirella biserrata	Rhoicospheni a curvata	53	0.01	0.04	0.03	0.05
0	0	0.35	1	cv	1	Hudsonema amabilis	Achnanthes lanceoloata	54	0.02	0.04	0.01	0.04
0	0	0.32	1	t	1	Hudsonema amabilis	Pinnularia sp. (large)	54	0.02	0.04	0.01	0.04
1	1	0.75	1	t	1	Hudsonema amabilis	Salmo trutta	54	0.02	0.04	0.05	0.04
0	0	0.41	1	t	1	Hudsonema amabilis	Cyclotella sp.	54	0.02	0.04	0.01	0.04
1	1	0.85	1	cv	1	Hudsonema amabilis	Unidentified detritus	54	0.02	0.04	0.06	0.04
0	0	0.47	1	t	1	Hudsonema amabilis	Audouinella	54	0.02	0.04	0.02	0.04

0	1	0.54	1	t	1	Hudsonema amabilis	Navicula avenacea	54	0.02	0.04	0.03	0.04
1	1	0.70	1	t	1	Hudsonema amabilis	Cocconeis sp.	54	0.02	0.04	0.04	0.04
0	0	0.40	1	cv	1	Achnanthes lanceoloata	Philorheithrus agilis	54	0.01	0.04	0.03	0.04
0	1	0.54	1	t	1	Achnanthes lanceoloata	Pycnocentrodes evecta	54	0.01	0.04	0.04	0.04
0	0	0.42	1	t	1	Achnanthes lanceoloata	Austroperla cyrene	54	0.01	0.04	0.03	0.04
0	0	0.20	1	t	1	Achnanthes lanceoloata	Deleatidium spp.	54	0.01	0.04	0.00	0.04
0	0	0.50	1	cv	1	Achnanthes lanceoloata	Hydora nitida larva	54	0.01	0.04	0.03	0.04
1	1	0.66	1	t	1	Achnanthes lanceoloata	Naonella	54	0.01	0.04	0.05	0.04
0	0	0.50	1	t	1	Achnanthes lanceoloata	Potamopyrgus antipodarum	54	0.01	0.04	0.03	0.04
1	1	0.62	1	t	1	Achnanthes lanceoloata	Coloburiscus humeralis	54	0.01	0.04	0.05	0.04
1	1	0.70	1	cv	1	Achnanthes lanceoloata	Zealandoperla/Acroperla sp.	54	0.01	0.04	0.05	0.04
1	1	0.64	1	t	1	Achnanthes lanceoloata	Austrosimulium australense	54	0.01	0.04	0.05	0.04
0	0	0.44	1	t	1	Achnanthes lanceoloata	Oligochaete Type I	54	0.01	0.04	0.03	0.04
1	1	0.65	1	t	1	Achnanthes lanceoloata	Aphrophila noevaezelandiae	54	0.01	0.04	0.05	0.04
1	1	0.65	1	cv	1	Achnanthes lanceoloata	Aoteapsyche sp.	54	0.01	0.04	0.05	0.04
0	0	0.43	1	t	1	Aulacoseira	Pirara	54	0.03	0.04	0.01	0.04
1	1	0.69	1	t	1	Aulacoseira sp.	Maoridiamesea	54	0.03	0.04	0.03	0.04
1	1	0.65	1	t	1	Aulacoseira sp.	Austroperla cyrene	54	0.03	0.04	0.03	0.04
0	1	0.52	1	cv	1	Aulacoseira	Stictocladius	54	0.03	0.04	0.01	0.04
1	1	0.84	1	t	1	Aulacoseira	Naonella	54	0.03	0.04	0.05	0.04
1	1	0.72	1	t	1	Aulacoseira sp.	Potamopyrgus antipodarum	54	0.03	0.04	0.03	0.04
1	1	0.81	1	t	1	Aulacoseira sp.	Coloburiscus humeralis	54	0.03	0.04	0.05	0.04
1	1	0.86	1	cv	1	Aulacoseira sp.	Zealandoperla/Acroperla sp.	54	0.03	0.04	0.05	0.04

1	1	0.82	1	t	1	Aulacoseira sp.	Austrosimulium australense	54	0.03	0.04	0.05	0.04
1	1	0.67	1	t	1	Aulacoseira sp.	Oligochaete Type I	54	0.03	0.04	0.03	0.04
0	1	0.61	1	t	1	Aulacoseira	Eukiefieriella	54	0.03	0.04	0.02	0.04
1	1	0.83	1	cv	1	Aulacoseira sp.	Aphrophila noevaezelandiae	54	0.03	0.04	0.05	0.04
1	1	0.83	1	t	1	Aulacoseira sp.	Aoteapsyche sp.	54	0.03	0.04	0.05	0.04
0	1	0.74	1	t	1	Rhoicosphenia curvata	Pycnocentrodes evecta	54	0.03	0.04	0.04	0.04
0	1	0.51	1	t	1	Rhoicosphenia curvata	Pelicypod sp.	54	0.03	0.04	0.01	0.04
1	1	0.64	1	cv	1	Cymbella	Naonella	54	0.01	0.04	0.05	0.04
1	1	0.61	1	t	1	Cymbella kappii	Austrosimulium australense	54	0.01	0.04	0.05	0.04
1	1	0.62	1	t	1	Cymbella kappii	Aphrophila noevaezelandiae	54	0.01	0.04	0.05	0.04
0	0	0.32	1	t	1	Pycnocentria sp.	Rhopalodia novae zelandicae	54	0.01	0.04	0.02	0.04
0	0	0.29	1	cv	1	Pycnocentria sp.	Macrophyte	54	0.01	0.04	0.01	0.04
0	0	0.27	1	t	1	Pycnocentria sp.	moss cells	54	0.01	0.04	0.01	0.04
0	0	0.30	1	t	1	Pycnocentria sp.	Cyclotella sp.	54	0.01	0.04	0.01	0.04
1	1	0.78	1	t	1	Pycnocentria sp.	Unidentified detritus	54	0.01	0.04	0.06	0.04
1	1	0.59	1	cv	1	Pycnocentria sp.	Cocconeis sp.	54	0.01	0.04	0.04	0.04
1	1	0.65	1	t	1	Polyplectropus puerilis	Pycnocentrodes evecta	54	0.02	0.04	0.04	0.04
0	0	0.30	1	t	1	Polyplectropus puerilis	Deleatidium spp.	54	0.02	0.04	0.00	0.04
1	1	0.75	1	t	1	Polyplectropus puerilis	Salmo trutta	54	0.02	0.04	0.05	0.04
1	1	0.85	1	cv	1	Polyplectropus puerilis	Unidentified detritus	54	0.02	0.04	0.06	0.04
1	1	0.73	1	t	1	Polyplectropus puerilis	Coloburiscus humeralis	54	0.02	0.04	0.05	0.04
0	0	0.40	1	t	1	Polyplectropus puerilis	Terrestrial invertebrates	54	0.02	0.04	0.01	0.04
0	1	0.62	1	t	1	Rhopalodia novae zelandicae	Pycnocentrodes evecta	54	0.02	0.04	0.04	0.04
1	1	0.73	1	cv	1	Oedogonium	Naonella	54	0.02	0.04	0.05	0.04

1	1	0.76	1	t	1	Oedogonium	Zealandoperla/Acroperla sp.	54	0.02	0.04	0.05	0.04
1	1	0.71	1	t	1	Oedogonium	Aphrophila noevaezelandiae	54	0.02	0.04	0.05	0.04
0	0	0.37	1	t	1	Pirara	Achnanthes linearis	54	0.01	0.04	0.03	0.04
0	0	0.25	1	cv	1	Pirara	Fragilaria vaucheriae	54	0.01	0.04	0.01	0.04
0	0	0.41	1	t	1	Pirara	Gomphonema parvulum	54	0.01	0.04	0.03	0.04
1	1	0.76	1	t	1	Pirara	Unidentified detritus	54	0.01	0.04	0.06	0.04
0	1	0.56	1	t	1	Pirara	Cocconeis sp.	54	0.01	0.04	0.04	0.04
0	1	0.63	1	cv	1	Pirara	Aoteapsyche sp.	54	0.01	0.04	0.05	0.04
0	0	0.31	1	t	1	Pirara	Costachorema xanthoptera	54	0.01	0.04	0.02	0.04
1	1	0.74	1	t	1	Diatoma heimale	Oligochaete Type II	54	0.04	0.04	0.03	0.04
0	1	0.67	1	t	1	Diatoma heimale	Orchymontia sp.	54	0.04	0.04	0.02	0.04
0	0	0.36	1	cv	1	Achnanthes linearis	Hydora nitida adult	54	0.03	0.04	0.01	0.04
0	0	0.33	1	t	1	Achnanthes linearis	Deleatidium spp.	54	0.03	0.04	0.00	0.04
0	1	0.52	1	t	1	Achnanthes linearis	Orchymontia sp.	54	0.03	0.04	0.02	0.04
1	1	0.66	1	t	1	Achnanthes linearis	Hydora nitida larva	54	0.03	0.04	0.03	0.04
1	1	0.79	1	cv	1	Achnanthes linearis	Naonella	54	0.03	0.04	0.05	0.04
1	1	0.76	1	t	1	Achnanthes linearis	Coloburiscus humeralis	54	0.03	0.04	0.05	0.04
1	1	0.82	1	t	1	Achnanthes linearis	Zealandoperla/Acroperla sp.	54	0.03	0.04	0.05	0.04
0	0	0.44	1	t	1	Achnanthes linearis	Pelicypod sp.	54	0.03	0.04	0.01	0.04
0	1	0.54	1	cv	1	Achnanthes linearis	Eukiefieriella	54	0.03	0.04	0.02	0.04
0	0	0.39	1	t	1	Hydora nitida adult	Meridion circulare	54	0.01	0.04	0.03	0.04
0	0	0.25	1	t	1	Hydora nitida adult	Fragilaria vaucheriae	54	0.01	0.04	0.01	0.04
0	0	0.41	1	t	1	Hydora nitida adult	Gomphonema parvulum	54	0.01	0.04	0.03	0.04

0	1	0.62	1	cv	1	Hydora nitida adult	Salmo trutta	54	0.01	0.04	0.05	0.04
0	1	0.76	1	t	1	Hydora nitida adult	Unidentified detritus	54	0.01	0.04	0.06	0.04
0	0	0.33	1	t	1	Hydora nitida adult	Gomphonema cf. olivaceum	54	0.01	0.04	0.02	0.04
0	0	0.31	1	t	1	Hydora nitida adult	Stephanodiscus sp.	54	0.01	0.04	0.02	0.04
0	0	0.39	1	cv	1	Hydora nitida adult	Navicula avenacea	54	0.01	0.04	0.03	0.04
0	1	0.56	1	t	1	Hydora nitida adult	Cocconeis sp.	54	0.01	0.04	0.04	0.04
0	0	0.36	1	t	1	Philorheithrus agilis	Pinnularia sp. (large)	54	0.03	0.04	0.01	0.04
0	0	0.45	1	t	1	Philorheithrus agilis	Cyclotella sp.	54	0.03	0.04	0.01	0.04
1	1	0.87	1	cv	1	Philorheithrus agilis	Unidentified detritus	54	0.03	0.04	0.06	0.04
0	1	0.52	1	t	1	Philorheithrus agilis	Audouinella	54	0.03	0.04	0.02	0.04
0	1	0.58	1	t	1	Philorheithrus agilis	Navicula avenacea	54	0.03	0.04	0.03	0.04
1	1	0.73	1	t	1	Philorheithrus agilis	Cocconeis sp.	54	0.03	0.04	0.04	0.04
1	1	0.86	1	cv	1	Fragilaria spp.	Zealandoperla/Acroperla sp.	54	0.03	0.04	0.05	0.04
0	1	0.65	1	t	1	Helicopsyche albescens	Caloneis sp. ?	54	0.02	0.04	0.04	0.04
0	0	0.31	1	t	1	Helicopsyche albescens	Pinnularia (small - mesolepta??)	54	0.02	0.04	0.01	0.04
0	0	0.39	1	t	1	Helicopsyche albescens	Cyclotella sp.	54	0.02	0.04	0.01	0.04
0	0	0.45	1	cv	1	Helicopsyche albescens	Tabellaria flocculosa	54	0.02	0.04	0.02	0.04
0	0	0.43	1	t	1	Helicopsyche albescens	Frustulia rhomboides	54	0.02	0.04	0.02	0.04
0	1	0.84	1	t	1	Helicopsyche albescens	Unidentified detritus	54	0.02	0.04	0.06	0.04
0	0	0.45	1	t	1	Helicopsyche albescens	Gomphonema cf. olivaceum	54	0.02	0.04	0.02	0.04
0	0	0.43	1	cv	1	Helicopsyche albescens	Nitzschia sp. (large)	54	0.02	0.04	0.02	0.04
0	0	0.41	1	t	1	Helicopsyche albescens	Cymbella minuta	54	0.02	0.04	0.02	0.04
0	0	0.50	1	t	1	Helicopsyche albescens	Synedra ulna	54	0.02	0.04	0.03	0.04

0	0	0.31	1	t	1	Helicopsyche albescens	Psilachorema bidens	54	0.02	0.04	0.01	0.04
0	1	0.52	1	cv	1	Helicopsyche albescens	Navicula avenacea	54	0.02	0.04	0.03	0.04
0	1	0.68	1	t	1	Helicopsyche albescens	Cocconeis sp.	54	0.02	0.04	0.04	0.04
1	1	0.56	1	t	1	Stauroneis sp.	Coloburiscus humeralis	54	0.00	0.04	0.05	0.04
0	0	0.47	1	t	1	Maoridiamese a	Fragilaria vaucheriae	54	0.03	0.04	0.01	0.04
0	1	0.61	1	cv	1	Maoridiamese a	Navicula rhynchoceph ala	54	0.03	0.04	0.03	0.04
1	1	0.82	1	t	1	Maoridiamese	Salmo trutta	54	0.03	0.04	0.05	0.04
1	1	0.89	1	t	1	Maoridiamese a	Unidentified detritus	54	0.03	0.04	0.06	0.04
1	1	0.72	1	t	1	Maoridiamese	Eunotia sp.	54	0.03	0.04	0.04	0.04
1	1	0.85	1	cv	1	Caloneis sp. ?	Coloburiscus humeralis	54	0.04	0.04	0.05	0.04
0	1	0.61	1	t	1	Nitzschia sp. (small)	Pycnocentrod es evecta	54	0.02	0.04	0.04	0.04
0	1	0.58	1	t	1	Nitzschia sp. (small)	Hydora nitida larva	54	0.02	0.04	0.03	0.04
1	1	0.69	1	t	1	Nitzschia sp. (small)	Coloburiscus humeralis	54	0.02	0.04	0.05	0.04
0	0	0.41	1	cv	1	Nitzschia sp. (small)	Tanytarsini	54	0.02	0.04	0.02	0.04
1	1	0.72	1	t	1	Nitzschia sp. (small)	Aphrophila noevaezeland iae	54	0.02	0.04	0.05	0.04
0	0	0.39	1	t	1	Cymbella aspera	Austroperla cyrene	54	0.01	0.04	0.03	0.04
1	1	0.64	1	t	1	Cymbella aspera	Naonella	54	0.01	0.04	0.05	0.04
0	0	0.47	1	cv	1	Cymbella aspera	Potamopyrgu s antipodarum	54	0.01	0.04	0.03	0.04
1	1	0.59	1	t	1	Cymbella aspera	Coloburiscus humeralis	54	0.01	0.04	0.05	0.04
1	1	0.61	1	t	1	Cymbella aspera	Austrosimuliu m australense	54	0.01	0.04	0.05	0.04
1	1	0.62	1	t	1	Cymbella aspera	Aoteapsyche sp.	54	0.01	0.04	0.05	0.04
1	1	0.74	1	cv	1	Surirella (small)	Zealandoperl a/Acroperla sp.	54	0.01	0.04	0.05	0.04
1	1	0.69	1	t	1	Surirella (small)	Aphrophila noevaezeland iae	54	0.01	0.04	0.05	0.04

0	1	0.51	1	t	1	Nothodixa sp.	Gomphonema parvulum	54	0.02	0.04	0.03	0.04
1	1	0.83	1	t	1	Nothodixa sp.	Unidentified detritus	54	0.02	0.04	0.06	0.04
0	0	0.47	1	cv	1	Nothodixa sp.	Synedra ulna	54	0.02	0.04	0.03	0.04
0	0	0.41	1	t	1	Nothodixa sp.	Stephanodiscus sp.	54	0.02	0.04	0.02	0.04
1	1	0.66	1	t	1	Nothodixa sp.	Cocconeis sp.	54	0.02	0.04	0.04	0.04
0	0	0.41	1	t	1	Pinnularia sp. (large)	Oligochaete Type II	54	0.01	0.04	0.03	0.04
0	0	0.19	1	cv	1	Pinnularia sp. (large)	Deleatidium spp.	54	0.01	0.04	0.00	0.04
0	0	0.27	1	t	1	Pinnularia sp. (large)	Olinga feredayii	54	0.01	0.04	0.01	0.04
0	0	0.47	1	t	1	Pinnularia sp. (large)	Potamopyrgus antipodarum	54	0.01	0.04	0.03	0.04
0	1	0.59	1	t	1	Pinnularia sp. (large)	Coloburiscus humeralis	54	0.01	0.04	0.05	0.04
0	1	0.61	1	cv	1	Pinnularia sp. (large)	Austrosimulium australense	54	0.01	0.04	0.05	0.04
0	0	0.27	1	t	1	Pinnularia sp. (large)	Pelicypod sp.	54	0.01	0.04	0.01	0.04
0	0	0.41	1	t	1	Pinnularia sp. (large)	Oligochaete Type I	54	0.01	0.04	0.03	0.04
0	1	0.62	1	t	1	Pinnularia sp. (large)	Aphrophila noevaezelandiae	54	0.01	0.04	0.05	0.04
0	0	0.49	1	cv	1	Macrophyte	Zealolessica cheira	54	0.01	0.04	0.03	0.04
0	0	0.47	1	t	1	Macrophyte	Austroperla cyrene	54	0.01	0.04	0.03	0.04
0	1	0.55	1	t	1	Macrophyte	Hydora nitida larva	54	0.01	0.04	0.03	0.04
0	0	0.34	1	t	1	Macrophyte	Olinga feredayii	54	0.01	0.04	0.01	0.04
0	0	0.45	1	cv	1	Macrophyte	Amphipoda spp.	54	0.01	0.04	0.03	0.04
1	1	0.71	1	t	1	Macrophyte	Naonella	54	0.01	0.04	0.05	0.04
0	1	0.55	1	t	1	Macrophyte	Potamopyrgus antipodarum	54	0.01	0.04	0.03	0.04
1	1	0.74	1	t	1	Macrophyte	Zealandoperla/Acroperla sp.	54	0.01	0.04	0.05	0.04
0	0	0.49	1	cv	1	Macrophyte	Oligochaete Type I	54	0.01	0.04	0.03	0.04

1	1	0.69	1	t	1	Macrophyte	Aphrophila noevaezelandiae	54	0.01	0.04	0.05	0.04
1	1	0.69	1	t	1	Macrophyte	Aoteapsyche sp.	54	0.01	0.04	0.05	0.04
0	1	0.62	1	t	1	Meridion circulare	Oligochaete Type II	54	0.03	0.04	0.03	0.04
0	1	0.60	1	cv	1	Meridion circulare	Austroperla cyrene	54	0.03	0.04	0.03	0.04
0	0	0.35	1	t	1	Meridion circulare	Deleatidium spp.	54	0.03	0.04	0.00	0.04
0	0	0.41	1	t	1	Meridion circulare	Scirtid sp. (Broad type)	54	0.03	0.04	0.01	0.04
1	1	0.80	1	t	1	Meridion circulare	Naonella	54	0.03	0.04	0.05	0.04
1	1	0.67	1	cv	1	Meridion circulare	Potamopyrgus antipodarum	54	0.03	0.04	0.03	0.04
1	1	0.83	1	t	1	Meridion circulare	Zealandoperla/Acroperla sp.	54	0.03	0.04	0.05	0.04
1	1	0.78	1	t	1	Meridion circulare	Austrosimulium australense	54	0.03	0.04	0.05	0.04
1	1	0.62	1	t	1	Meridion circulare	Oligochaete Type I	54	0.03	0.04	0.03	0.04
0	1	0.51	1	cv	1	Meridion circulare	Tanytarsini	54	0.03	0.04	0.02	0.04
1	1	0.79	1	t	1	Meridion circulare	Aphrophila noevaezelandiae	54	0.03	0.04	0.05	0.04
0	0	0.45	1	t	1	Oligochaete Type II	Fragilaria vaucheriae	54	0.03	0.04	0.01	0.04
1	0	0.40	1	t	1	Oligochaete Type II	Navicula sp.	54	0.03	0.04	0.01	0.04
1	1	0.89	1	cv	1	Oligochaete Type II	Unidentified detritus	54	0.03	0.04	0.06	0.04
1	0	0.43	1	t	1	Oligochaete Type II	Melosira varians	54	0.03	0.04	0.01	0.04
1	1	0.70	1	t	1	Oligochaete Type II	Eunotia sp.	54	0.03	0.04	0.04	0.04
1	1	0.53	1	t	1	Oligochaete Type II	Nitzschia sp. (large)	54	0.03	0.04	0.02	0.04
1	1	0.84	1	cv	1	Oligochaete Type II	Zealandoperla/Acroperla sp.	54	0.03	0.04	0.05	0.04
1	1	0.60	1	t	1	Oligochaete Type II	Synedra ulna	54	0.03	0.04	0.03	0.04
1	0	0.37	1	t	1	Oligochaete Type II	Eunotia sp. large	54	0.03	0.04	0.00	0.04

1	1	0.81	1	t	1	Oligochaete Type II	Aoteapsyche sp.	54	0.03	0.04	0.05	0.04
0	0	0.40	1	cv	1	Oligochaete Type II	Megaleptoperla grandis	54	0.03	0.04	0.01	0.04
1	1	0.89	1	t	1	Zealolessica cheira	Unidentified detritus	54	0.03	0.04	0.06	0.04
0	1	0.53	1	t	1	Zealolessica cheira	Nitzschia sp. (large)	54	0.03	0.04	0.02	0.04
1	1	0.76	1	t	1	Zealolessica cheira	Cocconeis sp.	54	0.03	0.04	0.04	0.04
0	1	0.56	1	cv	1	Fragilaria vaucheriae	Pycnocentrodes evecta	54	0.01	0.04	0.04	0.04
0	0	0.22	1	t	1	Fragilaria vaucheriae	Deleatidium spp.	54	0.01	0.04	0.00	0.04
0	1	0.52	1	t	1	Fragilaria vaucheriae	Hydora nitida larva	54	0.01	0.04	0.03	0.04
0	0	0.31	1	t	1	Fragilaria vaucheriae	Stictocladius	54	0.01	0.04	0.01	0.04
0	0	0.42	1	cv	1	Fragilaria vaucheriae	Amphipoda spp.	54	0.01	0.04	0.03	0.04
1	1	0.69	1	t	1	Fragilaria vaucheriae	Naonella	54	0.01	0.04	0.05	0.04
0	1	0.52	1	t	1	Fragilaria vaucheriae	Potamopyrgus antipodarum	54	0.01	0.04	0.03	0.04
0	1	0.64	1	t	1	Fragilaria vaucheriae	Coloburiscus humeralis	54	0.01	0.04	0.05	0.04
1	1	0.73	1	cv	1	Fragilaria vaucheriae	Zealandoperla/Acroperla sp.	54	0.01	0.04	0.05	0.04
1	1	0.66	1	t	1	Fragilaria vaucheriae	Austrosimulium australense	54	0.01	0.04	0.05	0.04
0	0	0.40	1	t	1	Fragilaria vaucheriae	Eukiefieriella	54	0.01	0.04	0.02	0.04
0	0	0.36	1	t	1	Fragilaria vaucheriae	Tanytarsini	54	0.01	0.04	0.02	0.04
1	1	0.67	1	cv	1	Fragilaria vaucheriae	Aphrophila noevaezelandiae	54	0.01	0.04	0.05	0.04
1	1	0.67	1	t	1	Fragilaria vaucheriae	Aoteapsyche sp.	54	0.01	0.04	0.05	0.04
0	1	0.56	1	t	1	moss cells	Pycnocentrodes evecta	54	0.01	0.04	0.04	0.04
0	0	0.45	1	t	1	moss cells	Austroperla cyrene	54	0.01	0.04	0.03	0.04
0	1	0.67	1	cv	1	moss cells	Aphrophila noevaezelandiae	54	0.01	0.04	0.05	0.04
1	1	0.86	1	t	1	Pycnocentrodes evecta	Salmo trutta	54	0.04	0.04	0.05	0.04

0	1	0.58	1	t	1	Pycnocentrodes evecta	Cyclotella sp.	54	0.04	0.04	0.01	0.04
0	1	0.62	1	t	1	Pycnocentrodes evecta	Frustulia rhomboides	54	0.04	0.04	0.02	0.04
1	1	0.92	1	cv	1	Pycnocentrodes evecta	Unidentified detritus	54	0.04	0.04	0.06	0.04
0	1	0.62	1	t	1	Pycnocentrodes evecta	Nitzschia sp. (large)	54	0.04	0.04	0.02	0.04
0	1	0.68	1	t	1	Pycnocentrodes evecta	Synedra ulna	54	0.04	0.04	0.03	0.04
0	1	0.64	1	t	1	Pycnocentrodes evecta	Audouinella	54	0.04	0.04	0.02	0.04
1	1	0.70	1	cv	1	Pycnocentrodes evecta	Navicula avenacea	54	0.04	0.04	0.03	0.04
1	1	0.82	1	t	1	Pycnocentrodes evecta	Cocconeis sp.	54	0.04	0.04	0.04	0.04
0	1	0.60	1	t	1	Pycnocentrodes evecta	Hydrobiosid sp.	54	0.04	0.04	0.02	0.04
0	1	0.54	1	t	1	Austroperla cyrene	Tabellaria flocculosa	54	0.03	0.04	0.02	0.04
1	1	0.88	1	cv	1	Austroperla cyrene	Unidentified detritus	54	0.03	0.04	0.06	0.04
0	1	0.69	1	t	1	Austroperla cyrene	Eunotia sp.	54	0.03	0.04	0.04	0.04
0	0	0.35	1	t	1	Austroperla cyrene	Unknown green algae filaments	54	0.03	0.04	0.00	0.04
1	1	0.75	1	t	1	Austroperla cyrene	Cocconeis sp.	54	0.03	0.04	0.04	0.04
0	0	0.38	1	cv	1	Deleatidium spp.	Gomphonema parvulum	54	0.00	0.04	0.03	0.04
0	0	0.19	1	t	1	Deleatidium spp.	Cymbella cuspidata	54	0.00	0.04	0.01	0.04
0	1	0.59	1	t	1	Deleatidium spp.	Salmo trutta	54	0.00	0.04	0.05	0.04
0	1	0.73	1	t	1	Deleatidium spp.	Unidentified detritus	54	0.00	0.04	0.06	0.04
0	0	0.21	1	cv	1	Deleatidium spp.	Melosira varians	54	0.00	0.04	0.01	0.04
0	0	0.24	1	t	1	Deleatidium spp.	Achnanthes saxonica	54	0.00	0.04	0.01	0.04
0	0	0.46	1	t	1	Deleatidium spp.	Eunotia sp.	54	0.00	0.04	0.04	0.04
0	0	0.34	1	t	1	Deleatidium spp.	Synedra ulna	54	0.00	0.04	0.03	0.04
0	0	0.21	1	cv	1	Deleatidium spp.	Gomphoneis herculeana	54	0.00	0.04	0.01	0.04
0	1	0.53	1	t	1	Deleatidium spp.	Cocconeis sp.	54	0.00	0.04	0.04	0.04

0	0	0.28	1	t	1	Deleatidium spp.	Costachorema xanthoptera	54	0.00	0.04	0.02	0.04
0	0	0.27	1	t	1	Deleatidium spp.	Hydrobiosid sp.	54	0.00	0.04	0.02	0.04
1	1	0.66	1	cv	1	Navicula rhynchocephal	Hydora nitida larva	54	0.03	0.04	0.03	0.04
0	0	0.44	1	t	1	Navicula rhynchocephal	Stictocladius	54	0.03	0.04	0.01	0.04
1	1	0.79	1	t	1	Navicula rhynchocephal	Naonella	54	0.03	0.04	0.05	0.04
1	1	0.66	1	t	1	Navicula rhynchocephala	Potamopyrgus antipodarum	54	0.03	0.04	0.03	0.04
1	1	0.76	1	cv	1	Navicula rhynchocephal	Coloburiscus humeralis	54	0.03	0.04	0.05	0.04
1	1	0.82	1	t	1	Navicula rhynchocephala	Zealandoperla/Acroperla sp.	54	0.03	0.04	0.05	0.04
1	1	0.77	1	t	1	Navicula rhynchocephala	Austrosimulium australense	54	0.03	0.04	0.05	0.04
0	1	0.60	1	t	1	Navicula rhynchocephal	Oligochaete Type I	54	0.03	0.04	0.03	0.04
0	0	0.49	1	cv	1	Navicula rhynchocephal	Tanytarsini	54	0.03	0.04	0.02	0.04
1	1	0.69	1	t	1	Gomphonema parvulum	Hydora nitida larva	54	0.03	0.04	0.03	0.04
1	1	0.82	1	t	1	Gomphonema parvulum	Naonella	54	0.03	0.04	0.05	0.04
1	1	0.78	1	t	1	Gomphonema parvulum	Coloburiscus humeralis	54	0.03	0.04	0.05	0.04
1	1	0.84	1	cv	1	Gomphonema parvulum	Zealandoperla/Acroperla sp.	54	0.03	0.04	0.05	0.04
1	1	0.80	1	t	1	Gomphonema parvulum	Austrosimulium australense	54	0.03	0.04	0.05	0.04
0	1	0.57	1	t	1	Gomphonema parvulum	Eukiefieriella	54	0.03	0.04	0.02	0.04
1	1	0.81	1	t	1	Gomphonema parvulum	Aphrophila noevaezelandiae	54	0.03	0.04	0.05	0.04
1	1	0.81	1	cv	1	Gomphonema parvulum	Aoteapsyche sp.	54	0.03	0.04	0.05	0.04
0	0	0.48	1	t	1	Orchymontia sp.	Tabellaria flocculosa	54	0.02	0.04	0.02	0.04
1	1	0.85	1	t	1	Orchymontia sp.	Unidentified detritus	54	0.02	0.04	0.06	0.04

0	0	0.48	1	t	1	Orchymontia sp.	Gomphonema cf. olivaceum	54	0.02	0.04	0.02	0.04
0	0	0.43	1	cv	1	Orchymontia sp.	Cymbella minuta	54	0.02	0.04	0.02	0.04
0	1	0.54	1	t	1	Orchymontia sp.	Navicula avenacea	54	0.02	0.04	0.03	0.04
1	1	0.70	1	t	1	Orchymontia sp.	Cocconeis sp.	54	0.02	0.04	0.04	0.04
0	1	0.61	1	t	1	Pinnularia (small - mesolepta??)	Austrosimulium australense	54	0.01	0.04	0.05	0.04
1	1	0.84	1	cv	1	Hydora nitida larva	Salmo trutta	54	0.03	0.04	0.05	0.04
0	0	0.46	1	t	1	Hydora nitida larva	Navicula sp.	54	0.03	0.04	0.01	0.04
1	1	0.91	1	t	1	Hydora nitida larva	Unidentified detritus	54	0.03	0.04	0.06	0.04
0	1	0.54	1	t	1	Hydora nitida larva	Achnanthes saxonica	54	0.03	0.04	0.01	0.04
0	1	0.51	1	cv	1	Hydora nitida larva	Gomphonema subclavatum	54	0.03	0.04	0.01	0.04
0	0	0.48	1	t	1	Hydora nitida larva	Gomphoneis herculeana	54	0.03	0.04	0.01	0.04
0	1	0.67	1	t	1	Hydora nitida larva	Navicula avenacea	54	0.03	0.04	0.03	0.04
1	1	0.80	1	t	1	Hydora nitida larva	Cocconeis sp.	54	0.03	0.04	0.04	0.04
1	1	0.64	1	cv	1	Cymbella cuspidata	Naonella	54	0.01	0.04	0.05	0.04
1	1	0.95	1	t	1	Salmo trutta	Unidentified detritus	54	0.05	0.04	0.06	0.04
1	1	0.77	1	t	1	Salmo trutta	Amphipoda spp.	54	0.05	0.04	0.03	0.04
1	1	0.91	1	t	1	Salmo trutta	Naonella	54	0.05	0.04	0.05	0.04
1	1	0.83	1	cv	1	Salmo trutta	Potamopyrgus antipodarum	54	0.05	0.04	0.03	0.04
1	1	0.89	1	t	1	Salmo trutta	Coloburiscus humeralis	54	0.05	0.04	0.05	0.04
1	1	0.92	1	t	1	Salmo trutta	Zealandoperla/Acroperla sp.	54	0.05	0.04	0.05	0.04
1	1	0.90	1	t	1	Salmo trutta	Austrosimulium australense	54	0.05	0.04	0.05	0.04
1	1	0.67	1	cv	1	Salmo trutta	Terrestrial invertebrates	54	0.05	0.04	0.01	0.04
1	1	0.60	1	t	1	Salmo trutta	Psilachorema bidens	54	0.05	0.04	0.01	0.04

1	1	0.90	1	t	1	Salmo trutta	Aphrophila noevaezelandiae	54	0.05	0.04	0.05	0.04
1	1	0.90	1	t	1	Salmo trutta	Aoteapsyche sp.	54	0.05	0.04	0.05	0.04
1	1	0.71	1	cv	1	Salmo trutta	Costachorema xanthoptera	54	0.05	0.04	0.02	0.04
1	1	0.69	1	t	1	Salmo trutta	Hydrobiosid sp.	54	0.05	0.04	0.02	0.04
1	1	0.78	1	t	1	Scirtid sp. (Broad type)	Unidentified detritus	54	0.01	0.04	0.06	0.04
0	1	0.52	1	t	1	Scirtid sp. (Broad type)	Eunotia sp.	54	0.01	0.04	0.04	0.04
1	1	0.81	1	cv	1	Olinga feredayii	Unidentified detritus	54	0.01	0.04	0.06	0.04
0	0	0.40	1	t	1	Olinga	Audouinella	54	0.01	0.04	0.02	0.04
1	1	0.64	1	t	1	Olinga feredayii	Cocconeis sp.	54	0.01	0.04	0.04	0.04
1	1	0.81	1	t	1	Stictocladius	Unidentified detritus	54	0.01	0.04	0.06	0.04
0	1	0.57	1	cv	1	Stictocladius	Eunotia sp.	54	0.01	0.04	0.04	0.04
1	1	0.73	1	t	1	Tabellaria flocculosa	Coloburiscus humeralis	54	0.02	0.04	0.05	0.04
1	1	0.79	1	t	1	Tabellaria flocculosa	Zealandoperla/Acroperla sp.	54	0.02	0.04	0.05	0.04
1	1	0.74	1	t	1	Tabellaria flocculosa	Austrosimulium australense	54	0.02	0.04	0.05	0.04
0	0	0.45	1	cv	1	Tabellaria flocculosa	Tanytarsini	54	0.02	0.04	0.02	0.04
1	1	0.75	1	t	1	Tabellaria flocculosa	Aoteapsyche sp.	54	0.02	0.04	0.05	0.04
1	1	0.75	1	t	1	Frustulia rhomboides	Naonella	54	0.02	0.04	0.05	0.04
1	1	0.78	1	t	1	Frustulia rhomboides	Zealandoperla/Acroperla sp.	54	0.02	0.04	0.05	0.04
1	1	0.72	1	cv	1	Frustulia rhomboides	Austrosimulium australense	54	0.02	0.04	0.05	0.04
1	1	0.54	1	t	1	Frustulia rhomboides	Oligochaete Type I	54	0.02	0.04	0.03	0.04
1	1	0.73	1	t	1	Frustulia rhomboides	Aphrophila noevaezelandiae	54	0.02	0.04	0.05	0.04
0	0	0.45	1	t	1	Microspora sp.	Amphipoda spp.	54	0.01	0.04	0.03	0.04

1	1	0.74	1	cv	1	Microspora sp.	Zealandoperla/Acroperla sp.	54	0.01	0.04	0.05	0.04
1	1	0.68	1	t	1	Microspora sp.	Austrosimulium australense	54	0.01	0.04	0.05	0.04
1	1	0.69	1	t	1	Microspora sp.	Aphrophila noevaezelandiae	54	0.01	0.04	0.05	0.04
1	1	0.69	1	t	1	Microspora sp.	Aoteapsyche sp.	54	0.01	0.04	0.05	0.04
1	1	0.86	1	cv	1	Unidentified detritus	Amphipoda spp.	54	0.06	0.04	0.03	0.04
1	1	0.95	1	t	1	Unidentified detritus	Naonella	54	0.06	0.04	0.05	0.04
1	1	0.90	1	t	1	Unidentified detritus	Potamopyrgus antipodarum	54	0.06	0.04	0.03	0.04
1	1	0.94	1	t	1	Unidentified detritus	Coloburiscus humeralis	54	0.06	0.04	0.05	0.04
1	1	0.96	1	cv	1	Unidentified detritus	Zealandoperla/Acroperla sp.	54	0.06	0.04	0.05	0.04
1	1	0.94	1	t	1	Unidentified detritus	Austrosimulium australense	54	0.06	0.04	0.05	0.04
1	1	0.79	1	t	1	Unidentified detritus	Pelicypod sp.	54	0.06	0.04	0.01	0.04
1	1	0.88	1	t	1	Unidentified detritus	Oligochaete Type I	54	0.06	0.04	0.03	0.04
1	1	0.85	1	cv	1	Unidentified detritus	Eukiefieriella	54	0.06	0.04	0.02	0.04
1	1	0.73	1	t	1	Unidentified detritus	Stratiomyid sp.	54	0.06	0.04	0.01	0.04
1	1	0.82	1	t	1	Unidentified detritus	Tanytarsini	54	0.06	0.04	0.02	0.04
1	1	0.95	1	t	1	Unidentified detritus	Aphrophila noevaezelandiae	54	0.06	0.04	0.05	0.04
1	1	0.95	1	cv	1	Unidentified detritus	Aoteapsyche sp.	54	0.06	0.04	0.05	0.04
1	1	0.82	1	t	1	Unidentified detritus	Costachorema xanthoptera	54	0.06	0.04	0.02	0.04
1	1	0.81	1	t	1	Unidentified detritus	Hydrobiosid sp.	54	0.06	0.04	0.02	0.04
0	0	0.45	1	t	1	Melosira varians	Oligochaete Type I	54	0.01	0.04	0.03	0.04
1	1	0.74	1	cv	1	Achnanthes saxonica	Zealandoperla/Acroperla sp.	54	0.01	0.04	0.05	0.04

1	1	0.68	1	t	1	Achnanthes saxonica	Austrosimulium australense	54	0.01	0.04	0.05	0.04
1	1	0.69	1	t	1	Achnanthes saxonica	Aoteapsyche sp.	54	0.01	0.04	0.05	0.04
1	1	0.69	1	t	1	Gomphonema subclavatum	Naonella	54	0.01	0.04	0.05	0.04
1	1	0.64	1	cv	1	Gomphonema subclavatum	Coloburiscus humeralis	54	0.01	0.04	0.05	0.04
1	1	0.67	1	t	1	Gomphonema subclavatum	Aoteapsyche sp.	54	0.01	0.04	0.05	0.04
1	1	0.67	1	t	1	Amphipoda	Eunotia sp.	54	0.03	0.04	0.04	0.04
0	0	0.33	1	t	1	Amphipoda spp.	Navicula bicephala	54	0.03	0.04	0.00	0.04
1	1	0.73	1	cv	1	Amphipoda spp.	Cocconeis sp.	54	0.03	0.04	0.04	0.04
1	1	0.78	1	t	1	Amphipoda spp.	Aoteapsyche sp.	54	0.03	0.04	0.05	0.04
0	0	0.33	1	t	1	Amphipoda spp.	Achnanthes sp. (small)	54	0.03	0.04	0.00	0.04
1	1	0.76	1	t	1	Gomphonema cf. olivaceum	Naonella	54	0.02	0.04	0.05	0.04
1	1	0.62	1	cv	1	Gomphonema cf. olivaceum	Potamopyrgus antipodarum	54	0.02	0.04	0.03	0.04
1	1	0.74	1	t	1	Gomphonema cf. olivaceum	Austrosimulium australense	54	0.02	0.04	0.05	0.04
1	1	0.75	1	t	1	Gomphonema cf. olivaceum	Aphrophila noevaezelandiae	54	0.02	0.04	0.05	0.04
1	1	0.75	1	t	1	Gomphonema cf. olivaceum	Aoteapsyche sp.	54	0.02	0.04	0.05	0.04
1	1	0.85	1	cv	1	Naonella	Eunotia sp.	54	0.05	0.04	0.04	0.04
1	1	0.69	1	t	1	Naonella	Navicula cryptocephala	54	0.05	0.04	0.01	0.04
0	1	0.73	1	t	1	Naonella	Nitzschia sp. (large)	54	0.05	0.04	0.02	0.04
1	1	0.71	1	t	1	Naonella	Cymbella minuta	54	0.05	0.04	0.02	0.04
1	1	0.75	1	cv	1	Naonella	Audouinella	54	0.05	0.04	0.02	0.04
1	1	0.80	1	t	1	Naonella	Navicula avenacea	54	0.05	0.04	0.03	0.04
1	1	0.88	1	t	1	Naonella	Cocconeis sp.	54	0.05	0.04	0.04	0.04
0	1	0.73	1	t	1	Naonella	Costachorema xanthoptera	54	0.05	0.04	0.02	0.04

1	1	0.75	1	cv	1	Eunotia sp.	Potamopyrgus antipodarum	54	0.04	0.04	0.03	0.04
1	1	0.83	1	t	1	Eunotia sp.	Coloburiscus humeralis	54	0.04	0.04	0.05	0.04
1	1	0.88	1	t	1	Eunotia sp.	Zealandoperla/Acroperla sp.	54	0.04	0.04	0.05	0.04
1	1	0.84	1	t	1	Eunotia sp.	Austrosimulium australense	54	0.04	0.04	0.05	0.04
1	1	0.70	1	cv	1	Eunotia sp.	Oligochaete Type I	54	0.04	0.04	0.03	0.04
0	1	0.60	1	t	1	Eunotia sp.	Tanytarsini	54	0.04	0.04	0.02	0.04
1	1	0.85	1	t	1	Eunotia sp.	Aphrophila noevaezelandiae	54	0.04	0.04	0.05	0.04
1	1	0.85	1	t	1	Eunotia sp.	Aoteapsyche sp.	54	0.04	0.04	0.05	0.04
1	1	0.59	1	cv	1	Spirogyra	Aoteapsyche sp.	54	0.00	0.04	0.05	0.04
1	1	0.74	1	t	1	Navicula cryptocephala	Zealandoperla/Acroperla sp.	54	0.01	0.04	0.05	0.04
1	1	0.68	1	t	1	Navicula cryptocephala	Austrosimulium australense	54	0.01	0.04	0.05	0.04
1	1	0.69	1	t	1	Navicula cryptocephala	Aphrophila noevaezelandiae	54	0.01	0.04	0.05	0.04
1	1	0.69	1	cv	1	Navicula cryptocephala	Aoteapsyche sp.	54	0.01	0.04	0.05	0.04
0	1	0.65	1	t	1	Potamopyrgus antipodarum	Synedra ulna	54	0.03	0.04	0.03	0.04
0	1	0.59	1	t	1	Potamopyrgus antipodarum	Stephanodiscus sp.	54	0.03	0.04	0.02	0.04
1	1	0.80	1	t	1	Potamopyrgus antipodarum	Cocconeis sp.	54	0.03	0.04	0.04	0.04
0	1	0.72	1	cv	1	Nitzschia sp. (large)	Austrosimulium australense	54	0.02	0.04	0.05	0.04
0	1	0.54	1	t	1	Nitzschia sp. (large)	Oligochaete Type I	54	0.02	0.04	0.03	0.04
0	1	0.67	1	t	1	Coloburiscus humeralis	Cymbella minuta	54	0.05	0.04	0.02	0.04
1	1	0.75	1	t	1	Coloburiscus humeralis	Synedra ulna	54	0.05	0.04	0.03	0.04
0	1	0.69	1	cv	1	Coloburiscus humeralis	Stephanodiscus sp.	54	0.05	0.04	0.02	0.04
1	1	0.87	1	t	1	Coloburiscus humeralis	Cocconeis sp.	54	0.05	0.04	0.04	0.04

0	1	0.68	1	t	1	Coloburiscus humeralis	Hydrobiosid sp.	54	0.05	0.04	0.02	0.04
1	1	0.76	1	t	1	Cymbella minuta	Zealandoperla/Acroperla sp.	54	0.02	0.04	0.05	0.04
1	1	0.71	1	cv	1	Cymbella minuta	Aphrophila noevaezelandiae	54	0.02	0.04	0.05	0.04
1	1	0.81	1	t	1	Zealandoperla/Acroperla sp.	Synedra ulna	54	0.05	0.04	0.03	0.04
0	1	0.76	1	t	1	Zealandoperla/Acroperla sp.	Stephanodiscus sp.	54	0.05	0.04	0.02	0.04
1	1	0.78	1	t	1	Zealandoperla/Acroperla sp.	Audouinella	54	0.05	0.04	0.02	0.04
0	1	0.68	1	cv	1	Zealandoperla/Acroperla sp.	Gomphoneis herculeana	54	0.05	0.04	0.01	0.04
1	1	0.82	1	t	1	Zealandoperla/Acroperla sp.	Navicula avenacea	54	0.05	0.04	0.03	0.04
1	1	0.90	1	t	1	Zealandoperla/Acroperla sp.	Cocconeis sp.	54	0.05	0.04	0.04	0.04
0	1	0.65	1	t	1	Zealandoperla/Acroperla sp.	Megaleptoperla grandis	54	0.05	0.04	0.01	0.04
1	1	0.76	1	cv	1	Austrosimulium australense	Synedra ulna	54	0.05	0.04	0.03	0.04
1	1	0.78	1	t	1	Austrosimulium australense	Navicula avenacea	54	0.05	0.04	0.03	0.04
1	1	0.87	1	t	1	Austrosimulium australense	Cocconeis sp.	54	0.05	0.04	0.04	0.04
1	1	0.78	1	t	1	Synedra ulna	Aoteapsyche sp.	54	0.03	0.04	0.05	0.04
0	0	0.38	1	cv	1	Stephanodiscus sp.	Pelicypod sp.	54	0.02	0.04	0.01	0.04
0	0	0.30	1	t	1	Stephanodiscus sp.	Stratiomyid sp.	54	0.02	0.04	0.01	0.04
1	1	0.75	1	t	1	Audouinella	Aphrophila noevaezelandiae	54	0.02	0.04	0.05	0.04
1	1	0.75	1	t	1	Audouinella	Aoteapsyche sp.	54	0.02	0.04	0.05	0.04
1	1	0.76	1	cv	1	Oligochaete Type I	Cocconeis sp.	54	0.03	0.04	0.04	0.04
0	1	0.70	1	t	1	Terrestrial invertebrates	Aoteapsyche sp.	54	0.01	0.04	0.05	0.04
0	0	0.38	1	t	1	Terrestrial invertebrates	Costachorema xanthoptera	54	0.01	0.04	0.02	0.04
0	0	0.36	1	t	1	Terrestrial invertebrates	Hydrobiosid sp.	54	0.01	0.04	0.02	0.04
1	1	0.77	1	cv	1	Eukiefieriella	Aphrophila noevaezelandiae	54	0.02	0.04	0.05	0.04

1	1	0.71	1	t	1	Eukiefieriella	Cocconeis sp.	54	0.02	0.04	0.04	0.04
1	1	0.77	1	t	1	Eukiefieriella	Aoteapsyche sp.	54	0.02	0.04	0.05	0.04
0	0	0.47	1	t	1	Eukiefieriella	Costachorema xanthoptera	54	0.02	0.04	0.02	0.04
1	1	0.79	1	cv	1	Aphrophila noevaezelandiae	Navicula avenacea	54	0.05	0.04	0.03	0.04
1	1	0.88	1	t	1	Aphrophila noevaezelandiae	Cocconeis sp.	54	0.05	0.04	0.04	0.04
1	1	0.88	1	t	1	Cocconeis sp.	Aoteapsyche sp.	54	0.04	0.04	0.05	0.04
0	1	0.56	0	t	1	Polyplectropus puerilis	Oligochaete Type I	54	0.02	0.04	0.03	0.04
0	0	0.29	0	cv	1	Cymbella kappii	Nothodixa sp.	54	0.01	0.04	0.02	0.04
0	1	0.60	0	t	1	Austroperla cyrene	Navicula avenacea	54	0.03	0.04	0.03	0.04
0	1	0.53	0	t	1	Unknown green algae	Cocconeis sp.	54	0.00	0.04	0.04	0.04
0	1	0.62	0	t	1	Diatoma heimale	Nothodixa sp.	54	0.04	0.04	0.02	0.04
0	1	0.58	0	cv	1	Rhoicosphenia curvata	Gomphonema cf. olivaceum	54	0.03	0.04	0.02	0.04
0	0	0.36	0	t	1	Pycnocentria sp.	Tabellaria flocculosa	54	0.01	0.04	0.02	0.04
0	1	0.62	0	t	1	Orchymontia sp.	Hydora nitida larva	54	0.02	0.04	0.03	0.04
1	0	0.40	0	t	1	Fragilaria spp.	Unknown green algae filaments	54	0.03	0.04	0.00	0.04
0	0	0.26	0	cv	1	Terrestrial invertebrates	Megaleptoperla grandis	54	0.01	0.04	0.01	0.04
0	0	0.29	0	t	1	Pirara	Nitzschia sp. (small)	54	0.01	0.04	0.02	0.04
0	0	0.28	0	t	1	Helicopsyche albescens	Stauroneis sp.	54	0.02	0.04	0.00	0.04
0	0	0.30	0	t	1	Scirtid sp. (Broad type)	Cyclotella sp.	54	0.01	0.04	0.01	0.04
0	0	0.40	0	cv	1	Oedogonium	Nitzschia sp. (large)	54	0.02	0.04	0.02	0.04
0	1	0.70	0	t	1	Cyclotella sp.	Aoteapsyche sp.	54	0.01	0.04	0.05	0.04
0	0	0.44	0	t	1	Synedra ulna	Terrestrial invertebrates	54	0.03	0.04	0.01	0.04

0	1	0.89	0	t	1	Coloburiscus humeralis	Austrosimulium australense	54	0.05	0.04	0.05	0.04
0	1	0.51	0	cv	1	Zealolessica cheira	Cymbella minuta	54	0.03	0.04	0.02	0.04
1	1	0.70	0	t	1	Unidentified detritus	Spirogyra	54	0.06	0.04	0.00	0.04
0	0	0.28	0	t	1	Microspora sp.	Gomphoneis herculeana	54	0.01	0.04	0.01	0.04
0	0	0.39	0	t	1	Cymbella aspera	Meridion circulare	54	0.01	0.04	0.03	0.04
0	0	0.36	0	cv	1	Nothodixa sp.	Achnanthes saxonica	54	0.02	0.04	0.01	0.04
0	0	0.36	0	t	1	Olinga feredayii	Cymbella minuta	54	0.01	0.04	0.02	0.04
0	0	0.24	0	t	1	Stauroneis sp.	Macrophyte	54	0.00	0.04	0.01	0.04
1	1	0.64	0	t	1	Pycnocentria sp.	Austrosimulium australense	54	0.01	0.04	0.05	0.04
0	0	0.31	0	cv	1	Pirara	Helicopsyche albescens	54	0.01	0.04	0.02	0.04
0	0	0.22	0	t	1	moss cells	Achnanthes sp. (small)	54	0.01	0.04	0.00	0.04
0	0	0.29	0	t	1	Rhopalodia novae zelandicae	Megaleptoperla grandis	54	0.02	0.04	0.01	0.04
0	0	0.47	0	t	1	Pirara	Potamopyrgus antipodarum	54	0.01	0.04	0.03	0.04
0	0	0.29	0	cv	1	moss cells	Gomphonema subclavatum	54	0.01	0.04	0.01	0.04
0	0	0.24	0	t	1	Cyclotella sp.	Navicula bicephala	54	0.01	0.04	0.00	0.04
1	1	0.71	0	t	1	Nitzschia sp. (small)	Salmo trutta	54	0.02	0.04	0.05	0.04
0	0	0.19	0	t	1	Stauroneis sp.	Psilachorema bidens	54	0.00	0.04	0.01	0.04
0	0	0.39	0	cv	1	Cymbella kappii	Austroperla cyrene	54	0.01	0.04	0.03	0.04
0	0	0.45	0	t	1	Rhoicosphenia curvata	Gomphoneis herculeana	54	0.03	0.04	0.01	0.04
0	0	0.38	0	t	1	Navicula bicephala	Oligochaete Type I	54	0.00	0.04	0.03	0.04
0	1	0.61	0	t	1	Austrosimulium australense	Gomphoneis herculeana	54	0.05	0.04	0.01	0.04
1	1	0.68	0	cv	1	Eunotia sp.	Navicula avenacea	54	0.04	0.04	0.03	0.04
0	0	0.36	0	t	1	Stauroneis sp.	Austroperla cyrene	54	0.00	0.04	0.03	0.04

0	1	0.66	0	t	1	Diatoma heimale	Tabellaria flocculosa	54	0.04	0.04	0.02	0.04
0	1	0.50	0	t	1	Rhoicosphenia curvata	Surirella (small)	54	0.03	0.04	0.01	0.04
0	0	0.30	0	cv	1	Cymbella kappii	Stephanodiscus sp.	54	0.01	0.04	0.02	0.04
0	1	0.56	0	t	1	Navicula rhynchocephal	Synedra ulna	54	0.03	0.04	0.03	0.04
1	1	0.73	0	t	1	Frustulia rhomboides	Aoteapsyche sp.	54	0.02	0.04	0.05	0.04
0	0	0.31	0	t	1	Cymbella cuspidata	Frustulia rhomboides	54	0.01	0.04	0.02	0.04
0	0	0.30	0	cv	1	Navicula bicephala	Audouinella	54	0.00	0.04	0.02	0.04
0	0	0.23	0	t	1	Achnanthes lanceoloata	Hydora nitida adult	54	0.01	0.04	0.01	0.04
0	0	0.24	0	t	1	Surirella (small)	Deleatidium spp.	54	0.01	0.04	0.00	0.04
0	0	0.31	0	t	1	Pinnularia sp. (large)	Nitzschia sp. (large)	54	0.01	0.04	0.02	0.04
0	0	0.33	0	cv	1	Scirtid sp. (Broad type)	Frustulia rhomboides	54	0.01	0.04	0.02	0.04
0	0	0.35	0	t	1	Navicula sp.	Eukiefieriella	54	0.01	0.04	0.02	0.04
1	1	0.60	0	t	1	Synedra ulna	Oligochaete Type I	54	0.03	0.04	0.03	0.04
0	0	0.35	0	t	1	Austroperla cyrene	Spirogyra	54	0.03	0.04	0.00	0.04
0	0	0.39	0	cv	1	Cymbella kappii	Meridion circulare	54	0.01	0.04	0.03	0.04
0	1	0.51	0	t	1	Achnanthes lanceoloata	Eunotia sp.	54	0.01	0.04	0.04	0.04
0	0	0.37	0	t	1	Oligochaete Type I	Eunotia sp. large	54	0.03	0.04	0.00	0.04
0	1	0.60	0	t	1	Hudsonema amabilis	Fragilaria spp.	54	0.02	0.04	0.03	0.04
0	1	0.78	0	cv	1	Coloburiscus humeralis	Oligochaete Type I	54	0.05	0.04	0.03	0.04
0	0	0.33	0	t	1	Pinnularia sp. (large)	Audouinella	54	0.01	0.04	0.02	0.04
1	1	0.74	0	t	1	Maoridiamesea	Pycnocentrodes evecta	54	0.03	0.04	0.04	0.04
0	1	0.52	0	t	1	Fragilaria spp.	Olinga feredayii	54	0.03	0.04	0.01	0.04
0	0	0.41	0	cv	1	Orchymontia sp.	Cyclotella sp.	54	0.02	0.04	0.01	0.04
0	0	0.47	0	t	1	Oedogonium	Amphipoda spp.	54	0.02	0.04	0.03	0.04
0	0	0.21	0	t	1	Melosira varians	Achnanthes sp. (small)	54	0.01	0.04	0.00	0.04
0	0	0.20	0	t	1	Achnanthes lanceoloata	Spirogyra	54	0.01	0.04	0.00	0.04

0	1	0.52	0	cv	1	Nothodixa sp.	Oligochaete Type I	54	0.02	0.04	0.03	0.04
0	0	0.33	0	t	1	Pinnularia sp. (large)	Tabellaria flocculosa	54	0.01	0.04	0.02	0.04
1	1	0.72	0	t	1	Fragilaria spp.	Hydora nitida larva	54	0.03	0.04	0.03	0.04
0	0	0.27	0	t	1	moss cells	Melosira varians	54	0.01	0.04	0.01	0.04
0	0	0.41	0	cv	1	Achnanthes linearis	Fragilaria vaucheriae	54	0.03	0.04	0.01	0.04
0	1	0.58	0	t	1	Nothodixa sp.	Hydora nitida larva	54	0.02	0.04	0.03	0.04
0	0	0.26	0	t	1	Nothodixa sp.	Navicula bicephala	54	0.02	0.04	0.00	0.04
0	1	0.55	0	t	1	Oligochaete Type II	Tabellaria flocculosa	54	0.03	0.04	0.02	0.04
0	0	0.44	0	cv	1	Surirella	Synedra ulna	54	0.01	0.04	0.03	0.04
0	0	0.49	0	t	1	Nitzschia sp. (small)	Navicula avenacea	54	0.02	0.04	0.03	0.04
0	0	0.44	0	t	1	Gomphonema cf. olivaceum	Stephanodiscus sp.	54	0.02	0.04	0.02	0.04
0	0	0.50	0	t	1	Cocconeis sp.	Achnanthes sp. (small)	54	0.04	0.04	0.00	0.04
0	1	0.50	0	cv	1	Pycnocentria sp.	Hydora nitida larva	54	0.01	0.04	0.03	0.04
0	1	0.51	0	t	1	Aulacoseira sp.	Navicula cryptocephala	54	0.03	0.04	0.01	0.04
0	0	0.45	0	t	1	Frustulia rhomboides	Gomphonema cf. olivaceum	54	0.02	0.04	0.02	0.04
0	0	0.49	0	t	1	Synedra ulna	Tanytarsini	54	0.03	0.04	0.02	0.04
0	0	0.49	0	cv	1	Aulacoseira sp.	Fragilaria vaucheriae	54	0.03	0.04	0.01	0.04
0	1	0.73	0	t	1	Rhopalodia novae zelandicae	Naonella	54	0.02	0.04	0.05	0.04
0	0	0.25	0	t	1	Pirara	moss cells	54	0.01	0.04	0.01	0.04
1	0	0.29	0	t	1	Microspora sp.	Melosira varians	54	0.01	0.04	0.01	0.04
0	1	0.72	0	cv	1	Aphrophila noevaezelandiae	Costachorema xanthoptera	54	0.05	0.04	0.02	0.04
0	0	0.25	0	t	1	Pinnularia sp. (large)	moss cells	54	0.01	0.04	0.01	0.04
0	1	0.56	0	t	1	Fragilaria spp.	Costachorema xanthoptera	54	0.03	0.04	0.02	0.04
0	0	0.27	0	t	1	Pirara	Olinga feredayii	54	0.01	0.04	0.01	0.04

0	0	0.34	0	cv	1	Rhopalodia novae zelandicae	moss cells	54	0.02	0.04	0.01	0.04
0	0	0.40	0	t	1	Nitzschia sp. (small)	Costachorema xanthoptera	54	0.02	0.04	0.02	0.04
1	1	0.55	0	t	1	Navicula cryptocephala	Potamopyrgus antipodarum	54	0.01	0.04	0.03	0.04
0	1	0.62	0	t	1	Pycnocentrodes evecta	Stephanodiscus sp.	54	0.04	0.04	0.02	0.04
0	0	0.33	0	cv	1	Gomphonema subclavatum	Hydrobiosid sp.	54	0.01	0.04	0.02	0.04
0	0	0.32	0	t	1	Unknown green algae	Eukiefieriella	54	0.00	0.04	0.02	0.04
0	0	0.38	0	t	1	Nitzschia sp. (large)	Terrestrial invertebrates	54	0.02	0.04	0.01	0.04
0	0	0.35	0	t	1	Meridion circulare	Unknown green algae filaments	54	0.03	0.04	0.00	0.04
0	0	0.18	0	cv	1	Cymbella kappii	Stauroneis sp.	54	0.01	0.04	0.00	0.04
0	0	0.27	0	t	1	Pycnocentria sp.	Fragilaria vaucheriae	54	0.01	0.04	0.01	0.04
0	1	0.61	0	t	1	Pirara	Austrosimulium australense	54	0.01	0.04	0.05	0.04
0	0	0.47	0	t	1	Gomphonema parvulum	Microspora sp.	54	0.03	0.04	0.01	0.04
1	1	0.81	0	cv	1	Fragilaria spp.	Coloburiscus humeralis	54	0.03	0.04	0.05	0.04
1	1	0.65	0	t	1	Pycnocentria sp.	Aphrophila noevaezelandiae	54	0.01	0.04	0.05	0.04
0	0	0.36	0	t	1	Achnanthes linearis	Navicula sp.	54	0.03	0.04	0.01	0.04
0	0	0.29	0	t	1	Polyplectropus puerilis	Unknown green algae filaments	54	0.02	0.04	0.00	0.04
0	0	0.20	0	cv	1	Unknown green algae	Gomphoneis herculeana	54	0.00	0.04	0.01	0.04
0	0	0.24	0	t	1	Achnanthes saxonica	Eunotia sp. large	54	0.01	0.04	0.00	0.04
0	0	0.24	0	t	1	Fragilaria vaucheriae	Stratiomyid sp.	54	0.01	0.04	0.01	0.04
1	1	0.82	0	t	1	Nitzschia sp. (small)	Unidentified detritus	54	0.02	0.04	0.06	0.04
0	0	0.19	0	cv	1	Hydora nitida adult	Eunotia sp. large	54	0.01	0.04	0.00	0.04

0	0	0.43	0	t	1	Fragilaria spp.	Pinnularia (small - mesolepta??)	54	0.03	0.04	0.01	0.04
0	0	0.24	0	t	1	Stictocladius	Unknown green algae filaments	54	0.01	0.04	0.00	0.04
0	0	0.42	0	t	1	Rhoicosphenia curvata	Pirara	54	0.03	0.04	0.01	0.04
0	1	0.64	0	cv	1	Helicopsyche albescens	Pycnocentrodes evecta	54	0.02	0.04	0.04	0.04
0	1	0.59	0	t	1	Diatoma heimale	Microspora sp.	54	0.04	0.04	0.01	0.04
0	1	0.51	0	t	1	Nitzschia sp. (small)	Zealolessica cheira	54	0.02	0.04	0.03	0.04
0	0	0.43	0	t	1	Orchymontia sp.	Hydrobiosid sp.	54	0.02	0.04	0.02	0.04
0	1	0.66	0	cv	1	Cocconeis sp.	Costachorema xanthoptera	54	0.04	0.04	0.02	0.04
1	1	0.69	0	t	1	Nothodixa sp.	Coloburiscus humeralis	54	0.02	0.04	0.05	0.04
0	0	0.26	0	t	1	Cymbella aspera	Surirella (small)	54	0.01	0.04	0.01	0.04
0	0	0.45	0	t	1	Nothodixa sp.	Eukiefieriella	54	0.02	0.04	0.02	0.04
1	1	0.76	0	cv	1	Amphipoda spp.	Coloburiscus humeralis	54	0.03	0.04	0.05	0.04
0	1	0.59	0	t	1	Navicula bicephala	Aphrophila noevaezelandiae	54	0.00	0.04	0.05	0.04
0	0	0.41	0	t	1	Helicopsyche albescens	Hydrobiosid sp.	54	0.02	0.04	0.02	0.04
0	0	0.35	0	t	1	Achnanthes lanceoloata	Polyplectropus puerilis	54	0.01	0.04	0.02	0.04
0	0	0.33	0	cv	1	Cymbella	Audouinella	54	0.01	0.04	0.02	0.04
1	1	0.92	0	t	1	Caloneis sp. ?	Unidentified detritus	54	0.04	0.04	0.06	0.04
0	0	0.39	0	t	1	Stratiomyid sp.	Navicula avenacea	54	0.01	0.04	0.03	0.04
0	1	0.52	0	t	1	Rhopalodia novae zelandicae	Zealolessica cheira	54	0.02	0.04	0.03	0.04
0	0	0.29	0	cv	1	Cymbella cuspidata	Hydrobiosid sp.	54	0.01	0.04	0.02	0.04
0	1	0.56	0	t	1	Caloneis sp. ?	Fragilaria vaucheriae	54	0.04	0.04	0.01	0.04
0	0	0.29	0	t	1	Rhopalodia novae zelandicae	Pirara	54	0.02	0.04	0.01	0.04
1	1	0.69	0	t	1	Gomphonema parvulum	Potamopyrgus antipodarum	54	0.03	0.04	0.03	0.04

0	1	0.61	0	cv	1	Philorheithrus agilis	Oligochaete Type I	54	0.03	0.04	0.03	0.04
0	1	0.62	0	t	1	Pinnularia sp. (large)	Aoteapsyche sp.	54	0.01	0.04	0.05	0.04
1	1	0.62	0	t	1	Oedogonium	Caloneis sp. ?	54	0.02	0.04	0.04	0.04
0	0	0.19	0	t	1	Deleatidium spp.	Megaleptoperla grandis	54	0.00	0.04	0.01	0.04
0	0	0.34	0	cv	1	Eukiefieriella	Megaleptoperla grandis	54	0.02	0.04	0.01	0.04
1	1	0.62	0	t	1	Scirtid sp. (Broad type)	Coloburiscus humeralis	54	0.01	0.04	0.05	0.04
0	0	0.21	0	t	1	Psilachorema bidens	Stratiomyid sp.	54	0.01	0.04	0.01	0.04
0	0	0.34	0	t	1	moss cells	Cymbella minuta	54	0.01	0.04	0.02	0.04
0	1	0.83	0	cv	1	Potamopyrgus antipodarum	Austrosimulium australense	54	0.03	0.04	0.05	0.04
0	0	0.46	0	t	1	Achnanthes linearis	Cymbella minuta	54	0.03	0.04	0.02	0.04
0	0	0.26	0	t	1	Cymbella kappii	Navicula cryptocephala	54	0.01	0.04	0.01	0.04
0	0	0.32	0	t	1	Polyplectropus puerilis	Navicula sp.	54	0.02	0.04	0.01	0.04
0	1	0.66	0	cv	1	Rhoicosphenia curvata	Oligochaete Type I	54	0.03	0.04	0.03	0.04
0	1	0.64	0	t	1	Diatoma heimale	Nitzschia sp. (large)	54	0.04	0.04	0.02	0.04
0	1	0.54	0	t	1	Diatoma heimale	Scirtid sp. (Broad type)	54	0.04	0.04	0.01	0.04
0	0	0.39	0	t	1	Achnanthes lanceoloata	Achnanthes linearis	54	0.01	0.04	0.03	0.04
0	0	0.39	0	cv	1	Rhoicosphenia curvata	Stauroneis sp.	54	0.03	0.04	0.00	0.04
0	0	0.48	0	t	1	Oligochaete Type I	Terrestrial invertebrates	54	0.03	0.04	0.01	0.04
0	0	0.40	0	t	1	Nitzschia sp. (large)	Hydrobiosid sp.	54	0.02	0.04	0.02	0.04
0	0	0.44	0	t	1	Philorheithrus agilis	Terrestrial invertebrates	54	0.03	0.04	0.01	0.04
0	0	0.17	0	cv	1	Unknown green algae	Achnanthes sp. (small)	54	0.00	0.04	0.00	0.04
0	0	0.19	0	t	1	Navicula sp.	Spirogyra	54	0.01	0.04	0.00	0.04
0	1	0.51	0	t	1	Fragilaria spp.	Navicula cryptocephala	54	0.03	0.04	0.01	0.04
0	0	0.40	0	t	1	Surirella (small)	Tabellaria flocculosa	54	0.01	0.04	0.02	0.04
1	1	0.79	0	cv	1	Meridion circulare	Salmo trutta	54	0.03	0.04	0.05	0.04

0	0	0.33	0	t	1	Macrophyte	Achnanthes saxonica	54	0.01	0.04	0.01	0.04
0	1	0.54	0	t	1	Coloburiscus humeralis	Unknown green algae filaments	54	0.05	0.04	0.00	0.04
0	1	0.54	0	t	1	Austroperla cyrene	Gomphonem a cf. olivaceum	54	0.03	0.04	0.02	0.04
0	0	0.43	0	cv	1	Aulacoseira sp.	Stratiomyid sp.	54	0.03	0.04	0.01	0.04
0	0	0.36	0	t	1	Helicopsyche albescens	moss cells	54	0.02	0.04	0.01	0.04
0	0	0.45	0	t	1	Audouinella	Costachorem a xanthoptera	54	0.02	0.04	0.02	0.04
0	0	0.39	0	t	1	Amphipoda spp.	Gomphoneis herculeana	54	0.03	0.04	0.01	0.04
0	0	0.32	0	cv	1	Pycnocentria sp.	Nitzschia sp. (small)	54	0.01	0.04	0.02	0.04
0	0	0.46	0	t	1	Austroperla cyrene	Terrestrial invertebrates	54	0.03	0.04	0.01	0.04
0	1	0.58	0	t	1	Naonella	Unknown green algae filaments	54	0.05	0.04	0.00	0.04
0	0	0.30	0	t	1	Oedogonium	Gomphoneis herculeana	54	0.02	0.04	0.01	0.04
1	1	0.78	0	cv	1	Achnanthes lanceoloata	Unidentified detritus	54	0.01	0.04	0.06	0.04
0	0	0.39	0	t	1	Aulacoseira	Spirogyra	54	0.03	0.04	0.00	0.04
0	0	0.31	0	t	1	moss cells	Achnanthes saxonica	54	0.01	0.04	0.01	0.04
0	0	0.46	0	t	1	Pycnocentrod es evecta	Unknown green algae filaments	54	0.04	0.04	0.00	0.04
0	1	0.87	0	cv	1	Diatoma heimale	Aoteapsyche sp.	54	0.04	0.04	0.05	0.04
0	0	0.38	0	t	1	Achnanthes saxonica	Stephanodisc us sp.	54	0.01	0.04	0.02	0.04
0	0	0.36	0	t	1	Navicula rhynchocephal	Psilachorema bidens	54	0.03	0.04	0.01	0.04
0	1	0.75	0	t	1	Cyclotella sp.	Zealandoperl a/Acroperla sp.	54	0.01	0.04	0.05	0.04
0	1	0.77	0	cv	1	Naonella	Eukiefieriella	54	0.05	0.04	0.02	0.04
0	1	0.69	0	t	1	Potamopyrgus antipodarum	Oligochaete Type I	54	0.03	0.04	0.03	0.04
0	1	0.51	0	t	1	Aulacoseira sp.	Achnanthes saxonica	54	0.03	0.04	0.01	0.04
0	0	0.21	0	t	1	Melosira varians	Unknown green algae filaments	54	0.01	0.04	0.00	0.04

1	1	0.80	0	cv	1	Gomphonema parvulum	Salmo trutta	54	0.03	0.04	0.05	0.04
0	0	0.33	0	t	1	Philorheithrus agilis	Navicula bicephala	54	0.03	0.04	0.00	0.04
0	0	0.34	0	t	1	Philorheithrus agilis	Eunotia sp. large	54	0.03	0.04	0.00	0.04
1	1	0.56	0	t	1	Achnanthes saxonica	Eunotia sp.	54	0.01	0.04	0.04	0.04
0	0	0.47	0	cv	1	Polyplectropus puerilis	Audouinella	54	0.02	0.04	0.02	0.04
0	0	0.40	0	t	1	Pycnocentria sp.	Amphipoda spp.	54	0.01	0.04	0.03	0.04
0	0	0.45	0	t	1	Gomphonema cf. olivaceum	Tanytarsini	54	0.02	0.04	0.02	0.04
0	1	0.51	0	t	1	Aulacoseira sp.	Surirella (small)	54	0.03	0.04	0.01	0.04
0	1	0.60	0	cv	1	Aphrophila noevaezelandiae	Megaleptoperla grandis	54	0.05	0.04	0.01	0.04
0	0	0.37	0	t	1	Pinnularia (small -	Synedra ulna	54	0.01	0.04	0.03	0.04
1	1	0.64	0	t	1	Oligochaete Type II	Gomphonema parvulum	54	0.03	0.04	0.03	0.04
0	0	0.48	0	t	1	Potamopyrgus antipodarum	Gomphoneis herculeana	54	0.03	0.04	0.01	0.04
0	0	0.44	0	cv	1	Navicula rhynchocephal	Cyclotella sp.	54	0.03	0.04	0.01	0.04
0	0	0.28	0	t	1	Deleatidium spp.	Frustulia rhomboides	54	0.00	0.04	0.02	0.04
0	0	0.29	0	t	1	Deleatidium spp.	Nitzschia sp. (large)	54	0.00	0.04	0.02	0.04
1	1	0.68	0	t	1	Pinnularia (small - mesolepta??)	Zealandoperla/Acroperla sp.	54	0.01	0.04	0.05	0.04
0	1	0.55	0	cv	1	Zealolessica cheira	Tabellaria flocculosa	54	0.03	0.04	0.02	0.04
0	0	0.43	0	t	1	Eunotia sp.	Achnanthes sp. (small)	54	0.04	0.04	0.00	0.04
0	0	0.40	0	t	1	Orchymontia sp.	Navicula cryptocephala	54	0.02	0.04	0.01	0.04
0	0	0.30	0	t	1	Frustulia rhomboides	Megaleptoperla grandis	54	0.02	0.04	0.01	0.04
0	0	0.22	0	cv	1	Achnanthes lanceoloata	Cymbella cuspidata	54	0.01	0.04	0.01	0.04
0	0	0.47	0	t	1	Stephanodiscus sp.	Eukiefieriella	54	0.02	0.04	0.02	0.04
1	1	0.81	0	t	1	Microspora sp.	Unidentified detritus	54	0.01	0.04	0.06	0.04
0	0	0.46	0	t	1	Aulacoseira sp.	Scirtid sp. (Broad type)	54	0.03	0.04	0.01	0.04

0	0	0.41	0	cv	1	Rhopalodia novae zelandicae	Stephanodiscus sp.	54	0.02	0.04	0.02	0.04
0	0	0.24	0	t	1	Olinga feredayii	Unknown green algae filaments	54	0.01	0.04	0.00	0.04
0	0	0.24	0	t	1	Macrophyte	Unknown green algae filaments	54	0.01	0.04	0.00	0.04
1	1	0.78	0	t	1	Philorheithrus agilis	Aoteapsyche sp.	54	0.03	0.04	0.05	0.04
1	0	0.43	0	cv	1	Zealolessica cheira	Melosira varians	54	0.03	0.04	0.01	0.04
0	1	0.72	0	t	1	Diatoma heimale	Meridion circulare	54	0.04	0.04	0.03	0.04
0	0	0.39	0	t	1	Achnanthes linearis	Melosira varians	54	0.03	0.04	0.01	0.04
0	0	0.48	0	t	1	Rhoicosphenia curvata	Fragilaria vaucheriae	54	0.03	0.04	0.01	0.04
1	1	0.82	0	cv	1	Fragilaria spp.	Salmo trutta	54	0.03	0.04	0.05	0.04
0	0	0.48	0	t	1	Diatoma heimale	Navicula bicephala	54	0.04	0.04	0.00	0.04
0	0	0.31	0	t	1	Nitzschia sp. (small)	Scirtid sp. (Broad type)	54	0.02	0.04	0.01	0.04
0	0	0.49	0	t	1	Synedra ulna	Costachorema xanthoptera	54	0.03	0.04	0.02	0.04
1	0	0.25	0	cv	1	Oedogonium	Navicula bicephala	54	0.02	0.04	0.00	0.04
0	0	0.31	0	t	1	Achnanthes lanceoloata	Nothodixa sp.	54	0.01	0.04	0.02	0.04
0	0	0.35	0	t	1	Surirella (small)	Cymbella minuta	54	0.01	0.04	0.02	0.04
0	0	0.40	0	t	1	Hudsonema amabilis	Achnanthes saxonica	54	0.02	0.04	0.01	0.04
0	0	0.47	0	cv	1	Polyplectropus puerilis	Tabellaria flocculosa	54	0.02	0.04	0.02	0.04
0	0	0.26	0	t	1	Nitzschia sp. (small)	Achnanthes sp. (small)	54	0.02	0.04	0.00	0.04
0	0	0.23	0	t	1	Pycnocentria sp.	Cymbella cuspidata	54	0.01	0.04	0.01	0.04
0	1	0.64	0	t	1	Maoridiamesea	Meridion circulare	54	0.03	0.04	0.03	0.04
0	0	0.41	0	cv	1	Cyclotella sp.	Audouinella	54	0.01	0.04	0.02	0.04
1	1	0.83	0	t	1	Austroperla cyrene	Zealandoperla/Acroperla sp.	54	0.03	0.04	0.05	0.04
0	0	0.48	0	t	1	Pinnularia sp. (large)	Eunotia sp.	54	0.01	0.04	0.04	0.04
0	0	0.21	0	t	1	Pirara	Cymbella cuspidata	54	0.01	0.04	0.01	0.04

0	0	0.49	0	cv	1	Oedogonium	Meridion circulare	54	0.02	0.04	0.03	0.04
0	0	0.33	0	t	1	Achnanthes saxonica	Terrestrial invertebrates	54	0.01	0.04	0.01	0.04
1	1	0.61	0	t	1	Gomphonema subclavatum	Cocconeis sp.	54	0.01	0.04	0.04	0.04
0	0	0.43	0	t	1	Rhoicosphenia curvata	Hydora nitida adult	54	0.03	0.04	0.01	0.04
0	0	0.38	0	cv	1	Synedra ulna	Gomphoneis herculeana	54	0.03	0.04	0.01	0.04
0	0	0.18	0	t	1	Stauroneis sp.	Cymbella aspera	54	0.00	0.04	0.01	0.04
1	1	0.87	0	t	1	Achnanthes linearis	Unidentified detritus	54	0.03	0.04	0.06	0.04
0	0	0.45	0	t	1	Amphipoda	Pelicypod sp.	54	0.03	0.04	0.01	0.04
0	1	0.71	0	cv	1	Rhoicosphenia curvata	Hydora nitida larva	54	0.03	0.04	0.03	0.04
0	1	0.60	0	t	1	Eunotia sp. large	Aoteapsyche sp.	54	0.00	0.04	0.05	0.04
0	0	0.37	0	t	1	Cymbella cuspidata	Synedra ulna	54	0.01	0.04	0.03	0.04
1	0	0.29	0	t	1	Surirella (small)	Melosira varians	54	0.01	0.04	0.01	0.04
0	1	0.58	0	cv	1	Naonella	Achnanthes sp. (small)	54	0.05	0.04	0.00	0.04
0	0	0.19	0	t	1	Stauroneis sp.	Stratiomyid sp.	54	0.00	0.04	0.01	0.04
1	1	0.90	0	t	1	Austrosimulium australense	Aphrophila noevaezelandiae	54	0.05	0.04	0.05	0.04
0	0	0.25	0	t	1	Oedogonium	Spirogyra	54	0.02	0.04	0.00	0.04
0	0	0.39	0	cv	1	Pinnularia sp. (large)	Austroperla cyrene	54	0.01	0.04	0.03	0.04
0	0	0.31	0	t	1	Surirella (small)	Gomphonema subclavatum	54	0.01	0.04	0.01	0.04
1	1	0.60	0	t	1	Frustulia rhomboides	Potamopyrgus antipodarum	54	0.02	0.04	0.03	0.04
0	0	0.36	0	t	1	Nitzschia sp. (small)	Olinga feredayii	54	0.02	0.04	0.01	0.04
0	0	0.27	0	cv	1	Frustulia rhomboides	Spirogyra	54	0.02	0.04	0.00	0.04
0	1	0.64	0	t	1	Naonella	Gomphoneis herculeana	54	0.05	0.04	0.01	0.04
0	0	0.25	0	t	1	Scirtid sp. (Broad type)	Gomphoneis herculeana	54	0.01	0.04	0.01	0.04
0	0	0.49	0	t	1	Achnanthes linearis	Nitzschia sp. (large)	54	0.03	0.04	0.02	0.04

0	0	0.41	0	cv	1	Rhopalodia novae zelandicae	Helicopsyche albescens	54	0.02	0.04	0.02	0.04
0	0	0.27	0	t	1	Pinnularia sp. (large)	Navicula cryptocephala	54	0.01	0.04	0.01	0.04
0	0	0.32	0	t	1	moss cells	Cyclotella sp.	54	0.01	0.04	0.01	0.04
0	0	0.34	0	t	1	Amphipoda spp.	Eunotia sp. large	54	0.03	0.04	0.00	0.04
0	0	0.38	0	cv	1	Microspora sp.	Stephanodiscus sp.	54	0.01	0.04	0.02	0.04
0	1	0.54	0	t	1	Austroperla cyrene	Audouinella	54	0.03	0.04	0.02	0.04
0	1	0.64	0	t	1	Achnanthes lanceoloata	Salmo trutta	54	0.01	0.04	0.05	0.04
0	0	0.49	0	t	1	Surirella (small)	Oligochaete Type II	54	0.01	0.04	0.03	0.04
0	0	0.32	0	cv	1	Tabellaria flocculosa	Stratiomyid sp.	54	0.02	0.04	0.01	0.04
0	0	0.32	0	t	1	Hudsonema amabilis	Megaleptoperla grandis	54	0.02	0.04	0.01	0.04
1	1	0.55	0	t	1	Aulacoseira sp.	Rhopalodia novae zelandicae	54	0.03	0.04	0.02	0.04
0	0	0.48	0	t	1	Oligochaete Type II	Achnanthes saxonica	54	0.03	0.04	0.01	0.04
0	0	0.34	0	cv	1	Stictocladius	Microspora sp.	54	0.01	0.04	0.01	0.04
0	0	0.32	0	t	1	Stauroneis sp.	Eukiefieriella	54	0.00	0.04	0.02	0.04
0	0	0.46	0	t	1	Achnanthes lanceoloata	Rhoicosphenia curvata	54	0.01	0.04	0.03	0.04
0	1	0.66	0	t	1	Rhoicosphenia curvata	Zealolessica cheira	54	0.03	0.04	0.03	0.04
0	0	0.36	0	cv	1	Achnanthes saxonica	Hydrobiosid sp.	54	0.01	0.04	0.02	0.04
0	0	0.23	0	t	1	Cymbella cuspidata	Melosira varians	54	0.01	0.04	0.01	0.04
0	0	0.23	0	t	1	Cymbella aspera	Melosira varians	54	0.01	0.04	0.01	0.04
0	1	0.50	0	t	1	Maoridiamese	Cyclotella sp.	54	0.03	0.04	0.01	0.04
0	1	0.51	0	cv	1	Austroperla cyrene	Frustulia rhomboides	54	0.03	0.04	0.02	0.04
0	0	0.41	0	t	1	Navicula rhynchocephala	Gomphonema subclavatum	54	0.03	0.04	0.01	0.04
0	0	0.34	0	t	1	Olinga feredayii	Terrestrial invertebrates	54	0.01	0.04	0.01	0.04
0	0	0.23	0	t	1	Psilachorema bidens	Gomphoneis herculeana	54	0.01	0.04	0.01	0.04

1	1	0.78	0	cv	1	Synedra ulna	Aphrophila noevaezelandiae	54	0.03	0.04	0.05	0.04
0	1	0.75	0	t	1	Diatoma heimale	Maoridiamesea	54	0.04	0.04	0.03	0.04
0	0	0.31	0	t	1	Helicopsyche albescens	Navicula sp.	54	0.02	0.04	0.01	0.04
0	1	0.55	0	t	1	Gomphonema parvulum	Orchymontia sp.	54	0.03	0.04	0.02	0.04
0	0	0.40	0	cv	1	Tabellaria flocculosa	Achnanthes saxonica	54	0.02	0.04	0.01	0.04
0	0	0.17	0	t	1	Unknown green algae	Eunotia sp. large	54	0.00	0.04	0.00	0.04
0	1	0.55	0	t	1	Achnanthes lanceoloata	Caloneis sp. ?	54	0.01	0.04	0.04	0.04
0	0	0.33	0	t	1	Surirella (small)	Terrestrial invertebrates	54	0.01	0.04	0.01	0.04
0	0	0.34	0	cv	1	Gomphonema cf. olivaceum	Gomphoneis herculeana	54	0.02	0.04	0.01	0.04
0	0	0.44	0	t	1	Nothodixa sp.	Orchymontia sp.	54	0.02	0.04	0.02	0.04
0	0	0.37	0	t	1	Cymbella aspera	Synedra ulna	54	0.01	0.04	0.03	0.04
0	0	0.26	0	t	1	Surirella (small)	Pinnularia (small - mesolepta??)	54	0.01	0.04	0.01	0.04
0	1	0.59	0	cv	1	Hydora nitida larva	Tanytarsini	54	0.03	0.04	0.02	0.04
0	1	0.58	0	t	1	Rhoicosphenia curvata	Tabellaria flocculosa	54	0.03	0.04	0.02	0.04
0	1	0.51	0	t	1	Fragilaria spp.	Macrophyte	54	0.03	0.04	0.01	0.04
0	0	0.48	0	t	1	Orchymontia sp.	Audouinella	54	0.02	0.04	0.02	0.04
0	1	0.54	0	cv	1	Stephanodiscus sp.	Oligochaete Type I	54	0.02	0.04	0.03	0.04
1	1	0.78	0	t	1	Achnanthes linearis	Aphrophila noevaezelandiae	54	0.03	0.04	0.05	0.04
0	0	0.41	0	t	1	Cymbella aspera	Oligochaete Type I	54	0.01	0.04	0.03	0.04
0	0	0.38	0	t	1	Maoridiamesea	Achnanthes sp. (small)	54	0.03	0.04	0.00	0.04
0	0	0.40	0	cv	1	Gomphonema cf. olivaceum	Pelicypod sp.	54	0.02	0.04	0.01	0.04
1	1	0.59	0	t	1	Stauroneis sp.	Aphrophila noevaezelandiae	54	0.00	0.04	0.05	0.04
0	0	0.26	0	t	1	Gomphonema subclavatum	Gomphoneis herculeana	54	0.01	0.04	0.01	0.04

0	1	0.73	0	t	1	Helicopsyche albescens	Austrosimulium australense	54	0.02	0.04	0.05	0.04
1	1	0.84	0	cv	1	Zealolessica cheira	Zealandoperla/Acroperla sp.	54	0.03	0.04	0.05	0.04
0	1	0.51	0	t	1	Zealolessica cheira	Hydrobiosid sp.	54	0.03	0.04	0.02	0.04
0	0	0.46	0	t	1	Oligochaete Type II	moss cells	54	0.03	0.04	0.01	0.04
0	0	0.45	0	t	1	Pirara	Fragilaria spp.	54	0.01	0.04	0.03	0.04
0	1	0.51	0	cv	1	Amphipoda spp.	Gomphonema cf. olivaceum	54	0.03	0.04	0.02	0.04
0	0	0.29	0	t	1	Pinnularia (small -	Cymbella minuta	54	0.01	0.04	0.02	0.04
0	0	0.21	0	t	1	Stratiomyid sp.	Megaleptoperla grandis	54	0.01	0.04	0.01	0.04
1	1	0.87	0	t	1	Unidentified detritus	Navicula avenacea	54	0.06	0.04	0.03	0.04
0	0	0.47	0	cv	1	Cyclotella sp.	Navicula avenacea	54	0.01	0.04	0.03	0.04
0	0	0.49	0	t	1	Achnanthes linearis	Costachorema xanthoptera	54	0.03	0.04	0.02	0.04
0	1	0.58	0	t	1	Meridion circulare	Amphipoda spp.	54	0.03	0.04	0.03	0.04
0	1	0.59	0	t	1	Pinnularia (small -	Coloburiscus humeralis	54	0.01	0.04	0.05	0.04
0	0	0.27	0	cv	1	Hydora nitida adult	Terrestrial invertebrates	54	0.01	0.04	0.01	0.04
0	1	0.54	0	t	1	Potamopyrgus antipodarum	Pelicypod sp.	54	0.03	0.04	0.01	0.04
0	0	0.33	0	t	1	Amphipoda	Spirogyra	54	0.03	0.04	0.00	0.04
0	0	0.43	0	t	1	Hydora nitida larva	Eunotia sp. large	54	0.03	0.04	0.00	0.04
0	0	0.33	0	cv	1	Microspora sp.	Navicula cryptocephala	54	0.01	0.04	0.01	0.04
0	0	0.37	0	t	1	Tabellaria flocculosa	Gomphonema subclavatum	54	0.02	0.04	0.01	0.04
0	0	0.24	0	t	1	Gomphonema subclavatum	Stratiomyid sp.	54	0.01	0.04	0.01	0.04
0	0	0.21	0	t	1	Cymbella cuspidata	Psilachorema bidens	54	0.01	0.04	0.01	0.04
0	0	0.26	0	cv	1	Nitzschia sp. (small)	Unknown green algae filaments	54	0.02	0.04	0.00	0.04

0	0	0.43	0	t	1	Oligochaete Type I	Gomphoneis herculeana	54	0.03	0.04	0.01	0.04
0	0	0.36	0	t	1	Nothodixa sp.	Macrophyte	54	0.02	0.04	0.01	0.04
0	0	0.28	0	t	1	Stauroneis sp.	Stephanodiscus sp.	54	0.00	0.04	0.02	0.04
0	0	0.18	0	cv	1	Cymbella aspera	Unknown green algae filaments	54	0.01	0.04	0.00	0.04
0	1	0.62	0	t	1	Achnanthes linearis	Maoridiamesea	54	0.03	0.04	0.03	0.04
0	0	0.29	0	t	1	Stratiomyid sp.	Hydrobiosid sp.	54	0.01	0.04	0.02	0.04
0	0	0.29	0	t	1	Polyplectropus puerilis	Spirogyra	54	0.02	0.04	0.00	0.04
0	0	0.36	0	cv	1	Navicula rhynchocephal	Cymbella cuspidata	54	0.03	0.04	0.01	0.04
0	0	0.49	0	t	1	Frustulia rhomboides	Amphipoda spp.	54	0.02	0.04	0.03	0.04
0	0	0.27	0	t	1	Pelicypod sp.	Psilachorema bidens	54	0.01	0.04	0.01	0.04
0	1	0.87	0	t	1	Diatoma heimale	Aphrophila noevaezelandiae	54	0.04	0.04	0.05	0.04
1	1	0.69	0	cv	1	Philorheithrus agilis	Pycnocentrodes evecta	54	0.03	0.04	0.04	0.04
0	0	0.44	0	t	1	Achnanthes linearis	Macrophyte	54	0.03	0.04	0.01	0.04
0	0	0.49	0	t	1	Nitzschia sp. (small)	Meridion circulare	54	0.02	0.04	0.03	0.04
0	0	0.46	0	t	1	Navicula rhynchocephal	Hydrobiosid sp.	54	0.03	0.04	0.02	0.04
0	0	0.47	0	cv	1	Achnanthes lanceoloata	Aulacoseira sp.	54	0.01	0.04	0.03	0.04
0	0	0.27	0	t	1	Olinga feredayii	Megaleptoperla grandis	54	0.01	0.04	0.01	0.04
0	0	0.40	0	t	1	Zealolessica cheira	Pinnularia (small - mesolepta??)	54	0.03	0.04	0.01	0.04
0	1	0.53	0	t	1	Zealolessica cheira	Tanytarsini	54	0.03	0.04	0.02	0.04
0	0	0.43	0	cv	1	Nitzschia sp. (small)	Orchymontia sp.	54	0.02	0.04	0.02	0.04
0	1	0.52	0	t	1	Nothodixa sp.	Oligochaete Type II	54	0.02	0.04	0.03	0.04
0	0	0.46	0	t	1	Pycnocentrodes evecta	Deleatidium spp.	54	0.04	0.04	0.00	0.04
0	1	0.59	0	t	1	Macrophyte	Pycnocentrodes evecta	54	0.01	0.04	0.04	0.04
0	0	0.34	0	cv	1	Cyclotella sp.	Terrestrial invertebrates	54	0.01	0.04	0.01	0.04

0	0	0.37	0	t	1	Oligochaete Type II	Unknown green algae filaments	54	0.03	0.04	0.00	0.04
1	1	0.92	0	t	1	Coloburiscus humeralis	Zealandoperla/Acroperla sp.	54	0.05	0.04	0.05	0.04
0	0	0.36	0	t	1	Gomphonema parvulum	Spirogyra	54	0.03	0.04	0.00	0.04
0	0	0.27	0	cv	1	Cyclotella sp.	Psilachorema bidens	54	0.01	0.04	0.01	0.04
0	0	0.41	0	t	1	Rhopalodia novae zelandicae	Frustulia rhomboides	54	0.02	0.04	0.02	0.04
0	0	0.27	0	t	1	Pirara	Terrestrial invertebrates	54	0.01	0.04	0.01	0.04
0	0	0.18	0	t	1	Pirara	Spirogyra	54	0.01	0.04	0.00	0.04
1	1	0.89	0	cv	1	Caloneis sp. ?	Zealandoperla/Acroperla sp.	54	0.04	0.04	0.05	0.04
0	0	0.42	0	t	1	Maoridiamesea	Megaleptoperla grandis	54	0.03	0.04	0.01	0.04
0	0	0.31	0	t	1	Surirella (small)	Fragilaria vaucheriae	54	0.01	0.04	0.01	0.04
0	0	0.40	0	t	1	Aulacoseira sp.	Deleatidium spp.	54	0.03	0.04	0.00	0.04
1	1	0.73	0	cv	1	Zealolessica cheira	Pycnocentrodes evecta	54	0.03	0.04	0.04	0.04
0	0	0.36	0	t	1	Cyclotella sp.	Hydrobiosid sp.	54	0.01	0.04	0.02	0.04
0	0	0.47	0	t	1	Oedogonium	Achnanthes linearis	54	0.02	0.04	0.03	0.04
0	0	0.43	0	t	1	Hudsonema amabilis	Hydrobiosid sp.	54	0.02	0.04	0.02	0.04
0	0	0.40	0	cv	1	Cymbella minuta	Costachorema xanthoptera	54	0.02	0.04	0.02	0.04
0	0	0.43	0	t	1	Nitzschia sp. (small)	Gomphonema cf. olivaceum	54	0.02	0.04	0.02	0.04
1	0	0.43	0	t	1	Fragilaria spp.	Navicula sp.	54	0.03	0.04	0.01	0.04
0	1	0.62	0	t	1	Philorheithrus agilis	Maoridiamesea	54	0.03	0.04	0.03	0.04
0	0	0.38	0	cv	1	Stauroneis sp.	Zealolessica cheira	54	0.00	0.04	0.03	0.04
0	1	0.76	0	t	1	Unidentified detritus	Melosira varians	54	0.06	0.04	0.01	0.04
1	1	0.56	0	t	1	Microspora sp.	Eunotia sp.	54	0.01	0.04	0.04	0.04
0	0	0.33	0	t	1	Surirella (small)	Microspora sp.	54	0.01	0.04	0.01	0.04
0	0	0.44	0	cv	1	Philorheithrus agilis	Achnanthes saxonica	54	0.03	0.04	0.01	0.04

1	0	0.43	0	t	1	Aulacoseira	Navicula sp.	54	0.03	0.04	0.01	0.04
1	1	0.73	0	t	1	Nitzschia sp. (small)	Naonella	54	0.02	0.04	0.05	0.04
0	0	0.44	0	t	1	Philorheithrus agilis	Surirella (small)	54	0.03	0.04	0.01	0.04
1	1	0.84	0	cv	1	Fragilaria spp.	Naonella	54	0.03	0.04	0.05	0.04
1	1	0.62	0	t	1	Salmo trutta	Gomphoneis herculeana	54	0.05	0.04	0.01	0.04
1	0	0.46	1	t	1	Pycnocentria spp.	Gomphonem a parvulum	55	0.01	0.03	0.02	0.03
0	0	0.43	1	t	1	Pycnocentria spp.	Cyclotella sp.	55	0.01	0.03	0.02	0.03
1	1	0.64	1	cv	1	Pycnocentria spp.	Cocconeis sp.	55	0.01	0.03	0.04	0.03
1	1	0.67	1	t	1	Pycnocentria spp.	Unidentified detritus	55	0.01	0.03	0.04	0.03
0	0	0.41	1	t	1	Pycnocentria spp.	Achnanthes saxonica	55	0.01	0.03	0.02	0.03
1	1	0.60	1	t	1	Pycnocentria spp.	Cymbella minuta	55	0.01	0.03	0.03	0.03
0	0	0.27	1	cv	1	Pycnocentria spp.	Fragilaria vaucheriae	55	0.01	0.03	0.00	0.03
1	1	0.57	1	t	1	Closterium sp.	Aphrophila noevaezeland iae	55	0.00	0.03	0.04	0.03
1	1	0.57	1	t	1	Gomphonema sp. (skinny - tenue??)	Aphrophila noevaezeland iae	55	0.00	0.03	0.04	0.03
1	0	0.49	1	t	1	Achnanthes lanceoloata	Polypedellum	55	0.04	0.03	0.00	0.03
1	1	0.83	1	cv	1	Achnanthes lanceoloata	Pirara	55	0.04	0.03	0.04	0.03
1	1	0.71	1	t	1	Achnanthes lanceoloata	Oligochaeta type I	55	0.04	0.03	0.02	0.03
1	1	0.75	1	t	1	Achnanthes lanceoloata	Hydora nitida adult	55	0.04	0.03	0.03	0.03
1	0	0.49	1	t	1	Achnanthes lanceoloata	Helicopsyche albescens	55	0.04	0.03	0.00	0.03
1	1	0.53	1	cv	1	Achnanthes lanceoloata	Maoridiames ea	55	0.04	0.03	0.01	0.03
1	1	0.59	1	t	1	Achnanthes lanceoloata	Hudsonema aliena	55	0.04	0.03	0.01	0.03
1	1	0.59	1	t	1	Achnanthes lanceoloata	Oligochaeta type II	55	0.04	0.03	0.01	0.03
1	1	0.82	1	t	1	Achnanthes lanceoloata	Austroperla cyrene	55	0.04	0.03	0.04	0.03
1	0	0.49	1	cv	1	Achnanthes lanceoloata	Potamopyrgu s antipodarum	55	0.04	0.03	0.00	0.03
1	1	0.77	1	t	1	Achnanthes lanceoloata	Deleatidium spp.	55	0.04	0.03	0.03	0.03

1	0	0.49	1	t	1	Achnanthes lanceoloata	Coloburiscus humeralis	55	0.04	0.03	0.00	0.03
1	1	0.69	1	t	1	Achnanthes lanceoloata	Oxyethira albiceps	55	0.04	0.03	0.02	0.03
1	1	0.77	1	cv	1	Achnanthes lanceoloata	Amphipoda spp.	55	0.04	0.03	0.03	0.03
1	1	0.74	1	t	1	Achnanthes lanceoloata	Naonella	55	0.04	0.03	0.03	0.03
1	0	0.49	1	t	1	Achnanthes lanceoloata	Hydora nitida larvae	55	0.04	0.03	0.00	0.03
1	1	0.80	1	t	1	Achnanthes lanceoloata	Oligochaeta type weak-long-short	55	0.04	0.03	0.03	0.03
1	1	0.76	1	cv	1	Achnanthes lanceoloata	Unknown orthoclad	55	0.04	0.03	0.03	0.03
1	1	0.79	1	t	1	Achnanthes lanceoloata	Pycnocentrodes evecta	55	0.04	0.03	0.03	0.03
1	1	0.76	1	t	1	Achnanthes lanceoloata	Podonomus	55	0.04	0.03	0.03	0.03
1	1	0.84	1	t	1	Achnanthes lanceoloata	Autrosimilium australense	55	0.04	0.03	0.04	0.03
1	1	0.75	1	cv	1	Achnanthes lanceoloata	Tanytarsini	55	0.04	0.03	0.03	0.03
1	1	0.85	1	t	1	Achnanthes lanceoloata	Aphrophila noevaezelandiae	55	0.04	0.03	0.04	0.03
1	1	0.75	1	t	1	Achnanthes lanceoloata	Eukiefiriella	55	0.04	0.03	0.03	0.03
0	0	0.40	1	t	1	Aulacoseira	Polypedellum	55	0.03	0.03	0.00	0.03
1	1	0.63	1	cv	1	Aulacoseira sp.	Oligochaeta type I	55	0.03	0.03	0.02	0.03
1	1	0.68	1	t	1	Aulacoseira sp.	Hydora nitida adult	55	0.03	0.03	0.03	0.03
1	1	0.81	1	t	1	Aulacoseira sp.	Olinga feredayi	55	0.03	0.03	0.04	0.03
1	1	0.51	1	t	1	Aulacoseira sp.	Hudsonema aliena	55	0.03	0.03	0.01	0.03
1	1	0.51	1	cv	1	Aulacoseira sp.	Oligochaeta type II	55	0.03	0.03	0.01	0.03
1	1	0.76	1	t	1	Aulacoseira sp.	Austroperla cyrene	55	0.03	0.03	0.04	0.03
0	0	0.40	1	t	1	Aulacoseira sp.	Potamopyrgus antipodarum	55	0.03	0.03	0.00	0.03
1	1	0.71	1	t	1	Aulacoseira sp.	Deleatidium spp.	55	0.03	0.03	0.03	0.03
1	1	0.62	1	cv	1	Aulacoseira sp.	Oxyethira albiceps	55	0.03	0.03	0.02	0.03
1	1	0.70	1	t	1	Aulacoseira sp.	Amphipoda spp.	55	0.03	0.03	0.03	0.03

0	0	0.40	1	t	1	Aulacoseira sp.	Hydora nitida larvae	55	0.03	0.03	0.00	0.03
1	1	0.50	1	t	1	Aulacoseira sp.	Zelandoperla sp	55	0.03	0.03	0.01	0.03
1	1	0.73	1	cv	1	Aulacoseira sp.	Oligochaeta type weak-long-short	55	0.03	0.03	0.03	0.03
1	1	0.69	1	t	1	Aulacoseira sp.	Unknown orthoclad	55	0.03	0.03	0.03	0.03
1	1	0.69	1	t	1	Aulacoseira	Podonomus	55	0.03	0.03	0.03	0.03
1	1	0.68	1	t	1	Aulacoseira	Tanytarsini	55	0.03	0.03	0.03	0.03
0	0	0.27	1	cv	1	Rhoicosphenia curvata	Polypedellum	55	0.01	0.03	0.00	0.03
1	1	0.65	1	t	1	Rhoicosphenia curvata	Pirara	55	0.01	0.03	0.04	0.03
0	0	0.27	1	t	1	Rhoicosphenia curvata	Potamopyrgus antipodarum	55	0.01	0.03	0.00	0.03
0	0	0.27	1	t	1	Rhoicosphenia curvata	Hydora nitida larvae	55	0.01	0.03	0.00	0.03
1	1	0.61	1	cv	1	Rhoicosphenia curvata	Oligochaeta type weak-long-short	55	0.01	0.03	0.03	0.03
1	1	0.60	1	t	1	Rhoicosphenia curvata	Pycnocentrodes evecta	55	0.01	0.03	0.03	0.03
1	1	0.54	1	t	1	Rhoicosphenia curvata	Tanytarsini	55	0.01	0.03	0.03	0.03
1	1	0.60	1	t	1	Cymbella	Pirara	55	0.01	0.03	0.04	0.03
1	0	0.49	1	cv	1	Cymbella kappii	Hydora nitida adult	55	0.01	0.03	0.03	0.03
1	1	0.52	1	t	1	Cymbella kappii	Deleatidium spp.	55	0.01	0.03	0.03	0.03
1	1	0.52	1	t	1	Cymbella kappii	Amphipoda spp.	55	0.01	0.03	0.03	0.03
0	0	0.24	1	t	1	Polyplectropus puerilis	Maoridiamesea	55	0.00	0.03	0.01	0.03
1	0	0.38	1	cv	1	Polyplectropus puerilis	Hydrobiosid spp.	55	0.00	0.03	0.02	0.03
1	0	0.48	1	t	1	Polyplectropus puerilis	Deleatidium spp.	55	0.00	0.03	0.03	0.03
0	0	0.22	1	t	1	Polyplectropus puerilis	Salmo trutta	55	0.00	0.03	0.00	0.03
1	0	0.45	1	t	1	Polyplectropus puerilis	Naonella	55	0.00	0.03	0.03	0.03
1	1	0.59	1	cv	1	Polyplectropus puerilis	Autrosimilium australense	55	0.00	0.03	0.04	0.03
1	0	0.46	1	t	1	Polyplectropus puerilis	Eukiefiriella	55	0.00	0.03	0.03	0.03

0	1	0.56	1	t	1	Rhopalodia novae zelandicae	Podonomus	55	0.01	0.03	0.03	0.03
0	0	0.22	1	t	1	Polypedellum	Blue-green algae	55	0.00	0.03	0.01	0.03
0	0	0.48	1	cv	1	Polypedellum	Achnanthes linearis	55	0.00	0.03	0.03	0.03
0	0	0.49	1	t	1	Polypedellum	Nitzschia sp. (small)	55	0.00	0.03	0.03	0.03
0	0	0.28	1	t	1	Polypedellum	Meridion circulare	55	0.00	0.03	0.01	0.03
0	0	0.21	1	t	1	Polypedellum	Navicula rhynchocephala	55	0.00	0.03	0.00	0.03
0	0	0.35	1	cv	1	Polypedellum	Gomphonema parvulum	55	0.00	0.03	0.02	0.03
0	0	0.24	1	t	1	Polypedellum	Pinnularia (small - mesolepta??)	55	0.00	0.03	0.01	0.03
0	0	0.32	1	t	1	Polypedellum	Cyclotella sp.	55	0.00	0.03	0.02	0.03
0	1	0.53	1	t	1	Polypedellum	Cocconeis sp.	55	0.00	0.03	0.04	0.03
0	0	0.38	1	cv	1	Polypedellum	Microspora sp.	55	0.00	0.03	0.02	0.03
1	1	0.56	1	t	1	Polypedellum	Unidentified detritus	55	0.00	0.03	0.04	0.03
0	0	0.46	1	t	1	Polypedellum	Gomphonema cf. olivaceum	55	0.00	0.03	0.03	0.03
1	1	0.56	1	t	1	Polypedellum	Eunotia sp.	55	0.00	0.03	0.04	0.03
0	0	0.46	1	cv	1	Polypedellum	Navicula cryptocephala	55	0.00	0.03	0.03	0.03
0	0	0.27	1	t	1	Polypedellum	Navicula avenacea	55	0.00	0.03	0.01	0.03
0	0	0.48	1	t	1	Polypedellum	Cymbella minuta	55	0.00	0.03	0.03	0.03
0	0	0.36	1	t	1	Polypedellum	Synedra ulna	55	0.00	0.03	0.02	0.03
0	0	0.19	1	cv	1	Polypedellum	Fragilaria vaucheriae	55	0.00	0.03	0.00	0.03
0	0	0.36	1	t	1	Polypedellum	Tabellaria flocculosa	55	0.00	0.03	0.02	0.03
0	0	0.42	1	t	1	Polypedellum	Diatoma heimale	55	0.00	0.03	0.03	0.03
1	1	0.55	1	t	1	Archichauliodes diversus	Deleatidium spp.	55	0.01	0.03	0.03	0.03
0	0	0.28	1	cv	1	Archichauliodes diversus	Terrestrial invertebrates	55	0.01	0.03	0.00	0.03
1	0	0.27	1	t	1	Archichauliodes diversus	Salmo trutta	55	0.01	0.03	0.00	0.03

1	0	0.47	1	t	1	Blue-green algae	Hydora nitida adult	55	0.01	0.03	0.03	0.03
1	1	0.50	1	t	1	Blue-green algae	Deleatidium spp.	55	0.01	0.03	0.03	0.03
1	1	0.54	1	cv	1	Blue-green algae	Oligochaeta type weak-long-short	55	0.01	0.03	0.03	0.03
1	0	0.49	1	t	1	Blue-green algae	Unknown orthoclad	55	0.01	0.03	0.03	0.03
1	1	0.53	1	t	1	Blue-green algae	Pycnocentrodes evecta	55	0.01	0.03	0.03	0.03
1	1	0.67	1	t	1	Eunotia sp. large	Aphrophila noevaezelandiae	55	0.01	0.03	0.04	0.03
1	1	0.81	1	cv	1	Pirara	Achnanthes linearis	55	0.04	0.03	0.03	0.03
1	1	0.77	1	t	1	Pirara	Philorheithrus agilis	55	0.04	0.03	0.03	0.03
1	1	0.82	1	t	1	Pirara	unknown red algal spp.	55	0.04	0.03	0.03	0.03
1	1	0.71	1	t	1	Pirara	Gomphonema parvulum	55	0.04	0.03	0.02	0.03
1	1	0.68	1	cv	1	Pirara	Cyclotella sp.	55	0.04	0.03	0.02	0.03
1	1	0.85	1	t	1	Pirara	Unidentified detritus	55	0.04	0.03	0.04	0.03
1	1	0.62	1	t	1	Pirara	Navicula avenacea	55	0.04	0.03	0.01	0.03
1	1	0.52	1	t	1	Pirara	Fragilaria vaucheriae	55	0.04	0.03	0.00	0.03
1	1	0.72	1	cv	1	Pirara	Tabellaria flocculosa	55	0.04	0.03	0.02	0.03
1	1	0.64	1	t	1	Pirara	Psilachorema bidens	55	0.04	0.03	0.01	0.03
1	1	0.77	1	t	1	Pirara	Diatoma heimale	55	0.04	0.03	0.03	0.03
1	1	0.69	1	t	1	Oligochaeta type I	Achnanthes linearis	55	0.02	0.03	0.03	0.03
1	1	0.70	1	cv	1	Oligochaeta type I	Nitzschia sp. (small)	55	0.02	0.03	0.03	0.03
1	1	0.70	1	t	1	Oligochaeta type I	Pinnularia sp. (large)	55	0.02	0.03	0.03	0.03
0	0	0.39	1	t	1	Oligochaeta type I	Navicula rhynchocephala	55	0.02	0.03	0.00	0.03
1	1	0.57	1	t	1	Oligochaeta type I	Gomphonema parvulum	55	0.02	0.03	0.02	0.03
0	0	0.43	1	cv	1	Oligochaeta type I	Pinnularia (small - mesolepta??)	55	0.02	0.03	0.01	0.03
1	1	0.53	1	t	1	Oligochaeta type I	Cyclotella sp.	55	0.02	0.03	0.02	0.03

1	1	0.73	1	t	1	Oligochaeta type I	Cocconeis sp.	55	0.02	0.03	0.04	0.03
1	1	0.57	1	t	1	Oligochaeta type I	Frustulia rhomboides	55	0.02	0.03	0.02	0.03
1	1	0.75	1	cv	1	Oligochaeta type I	Unidentified detritus	55	0.02	0.03	0.04	0.03
1	1	0.52	1	t	1	Oligochaeta type I	Achnanthes saxonica	55	0.02	0.03	0.02	0.03
1	1	0.59	1	t	1	Oligochaeta type I	Gomphonem a subclavatum	55	0.02	0.03	0.02	0.03
1	1	0.67	1	t	1	Oligochaeta type I	Gomphonem a cf. olivaceum	55	0.02	0.03	0.03	0.03
1	1	0.75	1	cv	1	Oligochaeta type I	Eunotia sp.	55	0.02	0.03	0.04	0.03
1	0	0.47	1	t	1	Oligochaeta type I	Navicula avenacea	55	0.02	0.03	0.01	0.03
1	1	0.69	1	t	1	Oligochaeta type I	Cymbella minuta	55	0.02	0.03	0.03	0.03
1	1	0.58	1	t	1	Oligochaeta type I	Synedra ulna	55	0.02	0.03	0.02	0.03
0	0	0.36	1	cv	1	Oligochaeta type I	Fragilaria vaucheriae	55	0.02	0.03	0.00	0.03
1	1	0.58	1	t	1	Oligochaeta type I	Tabellaria flocculosa	55	0.02	0.03	0.02	0.03
1	1	0.63	1	t	1	Oligochaeta type I	Diatoma heimale	55	0.02	0.03	0.03	0.03
1	1	0.73	1	t	1	Achnanthes linearis	Hydora nitida adult	55	0.03	0.03	0.03	0.03
1	0	0.46	1	cv	1	Achnanthes linearis	Helicopsyche albescens	55	0.03	0.03	0.00	0.03
1	1	0.51	1	t	1	Achnanthes linearis	Maoridiames ea	55	0.03	0.03	0.01	0.03
1	1	0.57	1	t	1	Achnanthes linearis	Hudsonema aliena	55	0.03	0.03	0.01	0.03
1	1	0.57	1	t	1	Achnanthes linearis	Oligochaeta type II	55	0.03	0.03	0.01	0.03
1	1	0.80	1	cv	1	Achnanthes linearis	Austroperla cyrene	55	0.03	0.03	0.04	0.03
1	0	0.46	1	t	1	Achnanthes linearis	Potamopyrgu s antipodarum	55	0.03	0.03	0.00	0.03
1	1	0.75	1	t	1	Achnanthes linearis	Deleatidium spp.	55	0.03	0.03	0.03	0.03
1	0	0.46	1	t	1	Achnanthes linearis	Hydora nitida larvae	55	0.03	0.03	0.00	0.03
1	1	0.78	1	cv	1	Achnanthes linearis	Oligochaeta type weak-long-short	55	0.03	0.03	0.03	0.03

1	1	0.77	1	t	1	Achnanthes linearis	Pycnocentrodes evecta	55	0.03	0.03	0.03	0.03
1	1	0.74	1	t	1	Achnanthes linearis	Podonomus	55	0.03	0.03	0.03	0.03
1	1	0.83	1	t	1	Achnanthes linearis	Autrosimilium australense	55	0.03	0.03	0.04	0.03
1	1	0.73	1	cv	1	Achnanthes linearis	Tanytarsini	55	0.03	0.03	0.03	0.03
1	1	0.83	1	t	1	Achnanthes linearis	Aphrophila noevaezelandiae	55	0.03	0.03	0.04	0.03
1	1	0.75	1	t	1	Hydora nitida adult	Nitzschia sp. (small)	55	0.03	0.03	0.03	0.03
1	1	0.54	1	t	1	Hydora nitida adult	Meridion circulare	55	0.03	0.03	0.01	0.03
1	1	0.62	1	cv	1	Hydora nitida adult	Gomphonema parvulum	55	0.03	0.03	0.02	0.03
1	0	0.48	1	t	1	Hydora nitida adult	Pinnularia (small - mesolepta??)	55	0.03	0.03	0.01	0.03
1	1	0.59	1	t	1	Hydora nitida adult	Cyclotella sp.	55	0.03	0.03	0.02	0.03
1	1	0.64	1	t	1	Hydora nitida adult	Microspora sp.	55	0.03	0.03	0.02	0.03
1	1	0.57	1	cv	1	Hydora nitida adult	Achnanthes saxonica	55	0.03	0.03	0.02	0.03
1	1	0.64	1	t	1	Hydora nitida adult	Gomphonema subclavatum	55	0.03	0.03	0.02	0.03
1	1	0.72	1	t	1	Hydora nitida adult	Gomphonema cf. olivaceum	55	0.03	0.03	0.03	0.03
1	1	0.79	1	t	1	Hydora nitida adult	Eunotia sp.	55	0.03	0.03	0.04	0.03
1	1	0.74	1	cv	1	Hydora nitida adult	Cymbella minuta	55	0.03	0.03	0.03	0.03
1	1	0.63	1	t	1	Hydora nitida adult	Tabellaria flocculosa	55	0.03	0.03	0.02	0.03
1	1	0.51	1	t	1	Philorheithrus agilis	Hudsonema aliena	55	0.03	0.03	0.01	0.03
1	0	0.44	1	t	1	Philorheithrus agilis	Terrestrial invertebrates	55	0.03	0.03	0.00	0.03
1	1	0.68	1	cv	1	Philorheithrus agilis	Naonella	55	0.03	0.03	0.03	0.03
1	0	0.29	1	t	1	Fragilaria spp.	Maoridiamesea	55	0.01	0.02	0.01	0.03
0	0	0.50	1	t	1	Helicopsyche albescens	Nitzschia sp. (small)	55	0.00	0.03	0.03	0.03
0	0	0.31	1	t	1	Helicopsyche albescens	Macrophyte	55	0.00	0.03	0.02	0.03

0	0	0.28	1	cv	1	Helicopsyche albescens	Meridion circulare	55	0.00	0.03	0.01	0.03
0	0	0.38	1	t	1	Helicopsyche albescens	Navicula sp.	55	0.00	0.03	0.02	0.03
0	0	0.21	1	t	1	Helicopsyche albescens	Navicula rhynchocephala	55	0.00	0.03	0.00	0.03
0	0	0.24	1	t	1	Helicopsyche albescens	Pinnularia (small - mesolepta??)	55	0.00	0.03	0.01	0.03
0	0	0.21	1	cv	1	Helicopsyche albescens	Salmo trutta	55	0.00	0.03	0.00	0.03
0	0	0.32	1	t	1	Helicopsyche albescens	Cyclotella sp.	55	0.00	0.03	0.02	0.03
0	1	0.53	1	t	1	Helicopsyche albescens	Cocconeis sp.	55	0.00	0.03	0.04	0.03
0	0	0.35	1	t	1	Helicopsyche albescens	Frustulia rhomboides	55	0.00	0.03	0.02	0.03
0	1	0.56	1	cv	1	Helicopsyche albescens	Unidentified detritus	55	0.00	0.03	0.04	0.03
0	0	0.38	1	t	1	Helicopsyche albescens	Gomphonema subclavatum	55	0.00	0.03	0.02	0.03
0	0	0.21	1	t	1	Helicopsyche albescens	Navicula cf. radiosa	55	0.00	0.03	0.00	0.03
0	0	0.46	1	t	1	Helicopsyche albescens	Gomphonema cf. olivaceum	55	0.00	0.03	0.03	0.03
1	1	0.56	1	cv	1	Helicopsyche albescens	Eunotia sp.	55	0.00	0.03	0.04	0.03
0	0	0.27	1	t	1	Helicopsyche albescens	Navicula avenacea	55	0.00	0.03	0.01	0.03
0	0	0.28	1	t	1	Helicopsyche albescens	Nitzschia sp. (large)	55	0.00	0.03	0.01	0.03
0	0	0.22	1	t	1	Helicopsyche albescens	Surirella tenera ??	55	0.00	0.03	0.01	0.03
0	0	0.48	1	cv	1	Helicopsyche albescens	Cymbella minuta	55	0.00	0.03	0.03	0.03
0	0	0.36	1	t	1	Helicopsyche albescens	Synedra ulna	55	0.00	0.03	0.02	0.03
0	0	0.27	1	t	1	Helicopsyche albescens	moss cells	55	0.00	0.03	0.01	0.03
0	0	0.19	1	t	1	Helicopsyche albescens	Fragilaria vaucheriae	55	0.00	0.03	0.00	0.03
0	0	0.36	1	cv	1	Helicopsyche albescens	Tabellaria flocculosa	55	0.00	0.03	0.02	0.03
0	0	0.38	1	t	1	Helicopsyche albescens	Epithemia sorex	55	0.00	0.03	0.02	0.03
0	0	0.42	1	t	1	Helicopsyche albescens	Diatoma heimale	55	0.00	0.03	0.03	0.03

1	1	0.62	1	t	1	Stauroneis sp.	Oligochaeta type II	55	0.04	0.03	0.01	0.03
1	1	0.62	1	cv	1	Stauroneis sp.	Zelandoperla sp	55	0.04	0.03	0.01	0.03
1	1	0.75	1	t	1	Olinga feredayi	Gomphonema parvulum	55	0.04	0.03	0.02	0.03
1	1	0.57	1	t	1	Olinga feredayi	Fragilaria vaucheriae	55	0.04	0.03	0.00	0.03
1	1	0.54	1	t	1	Maoridiamesea	Nitzschia sp. (small)	55	0.01	0.03	0.03	0.03
0	0	0.32	1	cv	1	Maoridiamesea	Meridion circulare	55	0.01	0.03	0.01	0.03
0	0	0.24	1	t	1	Maoridiamesea	Navicula rhynchocephala	55	0.01	0.03	0.00	0.03
0	0	0.39	1	t	1	Maoridiamesea	Gomphonema parvulum	55	0.01	0.03	0.02	0.03
0	0	0.31	1	t	1	Maoridiamesea	Nannochorista philpotti	55	0.01	0.03	0.01	0.03
0	0	0.36	1	cv	1	Maoridiamese	Cyclotella sp.	55	0.01	0.03	0.02	0.03
1	1	0.58	1	t	1	Maoridiamesea	Cocconeis sp.	55	0.01	0.03	0.04	0.03
0	0	0.39	1	t	1	Maoridiamesea	Frustulia rhomboides	55	0.01	0.03	0.02	0.03
1	1	0.60	1	t	1	Maoridiamesea	Unidentified detritus	55	0.01	0.03	0.04	0.03
0	0	0.42	1	cv	1	Maoridiamesea	Gomphonema subclavatum	55	0.01	0.03	0.02	0.03
1	1	0.50	1	t	1	Maoridiamesea	Gomphonema cf. olivaceum	55	0.01	0.03	0.03	0.03
1	1	0.60	1	t	1	Maoridiamese	Eunotia sp.	55	0.01	0.03	0.04	0.03
1	1	0.53	1	t	1	Maoridiamesea	Cymbella minuta	55	0.01	0.03	0.03	0.03
0	0	0.41	1	cv	1	Maoridiamese	Synedra ulna	55	0.01	0.03	0.02	0.03
0	0	0.22	1	t	1	Maoridiamesea	Fragilaria vaucheriae	55	0.01	0.03	0.00	0.03
0	0	0.23	1	t	1	Maoridiamesea	Gomphoneis herculeana	55	0.01	0.03	0.00	0.03
0	0	0.41	1	t	1	Maoridiamesea	Tabellaria flocculosa	55	0.01	0.03	0.02	0.03
0	0	0.32	1	cv	1	Maoridiamesea	Costachorema xanthoptera	55	0.01	0.03	0.01	0.03
0	0	0.46	1	t	1	Maoridiamesea	Diatoma heimale	55	0.01	0.03	0.03	0.03
1	0	0.47	1	t	1	Nitzschia sp. (small)	Potamopyrgus antipodarum	55	0.03	0.03	0.00	0.03

1	1	0.77	1	t	1	Nitzschia sp. (small)	Scirtid sp. (Broad type)	55	0.03	0.03	0.03	0.03
1	1	0.68	1	cv	1	Nitzschia sp. (small)	Oxyethira albiceps	55	0.03	0.03	0.02	0.03
1	1	0.76	1	t	1	Nitzschia sp. (small)	Amphipoda spp.	55	0.03	0.03	0.03	0.03
1	1	0.79	1	t	1	Nitzschia sp. (small)	Oligochaeta type weak-long-short	55	0.03	0.03	0.03	0.03
1	1	0.75	1	t	1	Nitzschia sp. (small)	Podonomus	55	0.03	0.03	0.03	0.03
1	1	0.83	1	cv	1	Nitzschia sp. (small)	Autrosimilium australense	55	0.03	0.03	0.04	0.03
1	1	0.61	1	t	1	Nitzschia sp. (small)	Scirtid sp. (Skinny type)	55	0.03	0.03	0.02	0.03
1	1	0.74	1	t	1	Nitzschia sp. (small)	Tanytarsini	55	0.03	0.03	0.03	0.03
1	1	0.84	1	t	1	Nitzschia sp. (small)	Aphrophila noevaezelandiae	55	0.03	0.03	0.04	0.03
1	1	0.74	1	cv	1	Nitzschia sp. (small)	Eukiefiriella	55	0.03	0.03	0.03	0.03
1	0	0.49	1	t	1	Cymbella aspera	Hudsonema aliena	55	0.03	0.03	0.01	0.03
1	1	0.70	1	t	1	Cymbella aspera	Deleatidium spp.	55	0.03	0.03	0.03	0.03
1	0	0.39	1	t	1	Cymbella aspera	Coloburiscus humeralis	55	0.03	0.03	0.00	0.03
1	0	0.39	1	cv	1	Cymbella aspera	Hydora nitida larvae	55	0.03	0.03	0.00	0.03
1	1	0.78	1	t	1	Cymbella aspera	Autrosimilium australense	55	0.03	0.03	0.04	0.03
1	1	0.79	1	t	1	Cymbella aspera	Aphrophila noevaezelandiae	55	0.03	0.03	0.04	0.03
0	0	0.40	1	t	1	Hudsonema aliena	Macrophyte	55	0.01	0.03	0.02	0.03
0	0	0.28	1	cv	1	Hudsonema aliena	Navicula rhynchocephala	55	0.01	0.03	0.00	0.03
0	0	0.45	1	t	1	Hudsonema aliena	Gomphonema parvulum	55	0.01	0.03	0.02	0.03
0	0	0.32	1	t	1	Hudsonema aliena	Pinnularia (small - mesolepta??)	55	0.01	0.03	0.01	0.03
1	0	0.50	1	t	1	Hudsonema aliena	Cymbella cuspidata	55	0.01	0.03	0.02	0.03
0	0	0.28	1	cv	1	Hudsonema aliena	Salmo trutta	55	0.01	0.03	0.00	0.03

1	1	0.63	1	t	1	Hudsonema aliena	Cocconeis sp.	55	0.01	0.03	0.04	0.03
1	0	0.47	1	t	1	Hudsonema aliena	Microspora sp.	55	0.01	0.03	0.02	0.03
1	1	0.65	1	t	1	Hudsonema aliena	Unidentified detritus	55	0.01	0.03	0.04	0.03
0	0	0.40	1	cv	1	Hudsonema aliena	Achnanthes saxonica	55	0.01	0.03	0.02	0.03
1	0	0.48	1	t	1	Hudsonema aliena	Gomphonema subclavatum	55	0.01	0.03	0.02	0.03
1	1	0.56	1	t	1	Hudsonema aliena	Gomphonema cf. olivaceum	55	0.01	0.03	0.03	0.03
1	1	0.65	1	t	1	Hudsonema aliena	Eunotia sp.	55	0.01	0.03	0.04	0.03
0	0	0.35	1	cv	1	Hudsonema aliena	Navicula avenacea	55	0.01	0.03	0.01	0.03
0	0	0.46	1	t	1	Hudsonema aliena	Synedra ulna	55	0.01	0.03	0.02	0.03
0	0	0.26	1	t	1	Hudsonema aliena	Fragilaria vaucheriae	55	0.01	0.03	0.00	0.03
0	0	0.46	1	t	1	Hudsonema aliena	Tabellaria flocculosa	55	0.01	0.03	0.02	0.03
1	1	0.52	1	cv	1	Hudsonema aliena	Diatoma heimale	55	0.01	0.03	0.03	0.03
1	1	0.58	1	t	1	Pinnularia sp. (large)	Oligochaeta type II	55	0.03	0.03	0.01	0.03
1	0	0.47	1	t	1	Pinnularia sp. (large)	Potamopyrgus antipodarum	55	0.03	0.03	0.00	0.03
1	1	0.76	1	t	1	Pinnularia sp. (large)	Deleatidium spp.	55	0.03	0.03	0.03	0.03
1	1	0.79	1	cv	1	Pinnularia sp. (large)	Oligochaeta type weak-long-short	55	0.03	0.03	0.03	0.03
1	1	0.75	1	t	1	Pinnularia sp. (large)	Unknown orthoclad	55	0.03	0.03	0.03	0.03
1	1	0.78	1	t	1	Pinnularia sp. (large)	Pycnocentrodes evecta	55	0.03	0.03	0.03	0.03
1	1	0.83	1	t	1	Pinnularia sp. (large)	Autrosimilium australense	55	0.03	0.03	0.04	0.03
1	1	0.84	1	cv	1	Pinnularia sp. (large)	Aphrophila noevaezelandiae	55	0.03	0.03	0.04	0.03
1	0	0.40	1	t	1	Macrophyte	Oligochaeta type II	55	0.02	0.03	0.01	0.03
1	1	0.67	1	t	1	Macrophyte	Austroperla cyrene	55	0.02	0.03	0.04	0.03

0	0	0.30	1	t	1	Macrophyte	Potamopyrgus antipodarum	55	0.02	0.03	0.00	0.03
1	0	0.46	1	cv	1	Macrophyte	Tanyderid sp.	55	0.02	0.03	0.02	0.03
1	1	0.60	1	t	1	Macrophyte	Amphipoda spp.	55	0.02	0.03	0.03	0.03
0	0	0.30	1	t	1	Macrophyte	Hydora nitida larvae	55	0.02	0.03	0.00	0.03
1	0	0.39	1	t	1	Macrophyte	Zelandoperla sp	55	0.02	0.03	0.01	0.03
1	1	0.59	1	cv	1	Macrophyte	Unknown orthoclad	55	0.02	0.03	0.03	0.03
1	1	0.63	1	t	1	Macrophyte	Pycnocentrodes evecta	55	0.02	0.03	0.03	0.03
1	1	0.71	1	t	1	Macrophyte	Aphrophila noevaezelandiae	55	0.02	0.03	0.04	0.03
1	0	0.45	1	t	1	Hydrobiosid spp.	Oligochaeta type II	55	0.02	0.03	0.01	0.03
1	0	0.37	1	cv	1	Hydrobiosid spp.	Salmo trutta	55	0.02	0.03	0.00	0.03
0	1	0.62	1	t	1	Hydrobiosid spp.	Naonella	55	0.02	0.03	0.03	0.03
1	0	0.47	1	t	1	Hydrobiosid spp.	Costachorema xanthoptera	55	0.02	0.03	0.01	0.03
0	0	0.37	1	t	1	Meridion circulare	Oligochaeta type II	55	0.01	0.03	0.01	0.03
0	0	0.27	1	cv	1	Meridion circulare	Potamopyrgus antipodarum	55	0.01	0.03	0.00	0.03
1	1	0.57	1	t	1	Meridion circulare	Deleatidium spp.	55	0.01	0.03	0.03	0.03
1	1	0.57	1	t	1	Meridion circulare	Amphipoda spp.	55	0.01	0.03	0.03	0.03
1	1	0.54	1	t	1	Meridion circulare	Naonella	55	0.01	0.03	0.03	0.03
1	1	0.61	1	cv	1	Meridion circulare	Oligochaeta type weak-long-short	55	0.01	0.03	0.03	0.03
1	1	0.56	1	t	1	Meridion circulare	Unknown orthoclad	55	0.01	0.03	0.03	0.03
1	1	0.56	1	t	1	Meridion circulare	Podonomus	55	0.01	0.03	0.03	0.03
1	1	0.67	1	t	1	Meridion circulare	Autrosimilium australense	55	0.01	0.03	0.04	0.03
1	1	0.68	1	cv	1	Meridion circulare	Aphrophila noevaezelandiae	55	0.01	0.03	0.04	0.03

1	1	0.67	1	t	1	Navicula sp.	Amphipoda spp.	55	0.02	0.03	0.03	0.03
1	0	0.36	1	t	1	Navicula sp.	Hydora nitida larvae	55	0.02	0.03	0.00	0.03
1	0	0.46	1	t	1	Navicula sp.	Zelandoperla sp	55	0.02	0.03	0.01	0.03
1	1	0.76	1	cv	1	Navicula sp.	Autrosimilium australense	55	0.02	0.03	0.04	0.03
1	1	0.77	1	t	1	Navicula sp.	Aphrophila noevaezelandiae	55	0.02	0.03	0.04	0.03
1	1	0.74	1	t	1	unknown red algal spp.	Tanytarsini	55	0.03	0.03	0.03	0.03
0	0	0.28	1	t	1	Oligochaeta type II	Navicula rhynchocephala	55	0.01	0.03	0.00	0.03
0	0	0.44	1	cv	1	Oligochaeta type II	Gomphonema parvulum	55	0.01	0.03	0.02	0.03
0	0	0.32	1	t	1	Oligochaeta type II	Pinnularia (small - mesolepta??)	55	0.01	0.03	0.01	0.03
0	0	0.41	1	t	1	Oligochaeta type II	Cyclotella sp.	55	0.01	0.03	0.02	0.03
1	1	0.63	1	t	1	Oligochaeta type II	Cocconeis sp.	55	0.01	0.03	0.04	0.03
1	0	0.47	1	cv	1	Oligochaeta type II	Microspora sp.	55	0.01	0.03	0.02	0.03
1	1	0.65	1	t	1	Oligochaeta type II	Unidentified detritus	55	0.01	0.03	0.04	0.03
0	0	0.26	1	t	1	Oligochaeta type II	Melosira varians	55	0.01	0.03	0.00	0.03
1	1	0.56	1	t	1	Oligochaeta type II	Gomphonema cf. olivaceum	55	0.01	0.03	0.03	0.03
1	1	0.65	1	cv	1	Oligochaeta type II	Eunotia sp.	55	0.01	0.03	0.04	0.03
1	1	0.56	1	t	1	Oligochaeta type II	Navicula cryptocephala	55	0.01	0.03	0.03	0.03
1	1	0.58	1	t	1	Oligochaeta type II	Cymbella minuta	55	0.01	0.03	0.03	0.03
0	0	0.33	1	t	1	Oligochaeta type II	Gomphonema truncatum	55	0.01	0.03	0.01	0.03
1	0	0.46	1	cv	1	Oligochaeta type II	Synedra ulna	55	0.01	0.03	0.02	0.03
0	0	0.26	1	t	1	Oligochaeta type II	Fragilaria vaucheriae	55	0.01	0.03	0.00	0.03
1	0	0.46	1	t	1	Oligochaeta type II	Tabellaria flocculosa	55	0.01	0.03	0.02	0.03

0	0	0.37	1	t	1	Oligochaeta type II	Psilachorema bidens	55	0.01	0.03	0.01	0.03
1	1	0.52	1	cv	1	Oligochaeta type II	Diatoma heimale	55	0.01	0.03	0.03	0.03
1	1	0.53	1	t	1	Austroperla cyrene	Navicula rhynchocephala	55	0.04	0.03	0.00	0.03
1	1	0.58	1	t	1	Austroperla cyrene	Pinnularia (small - mesolepta??)	55	0.04	0.03	0.01	0.03
1	1	0.68	1	t	1	Austroperla cyrene	Cyclotella sp.	55	0.04	0.03	0.02	0.03
1	1	0.70	1	cv	1	Austroperla cyrene	Frustulia rhomboides	55	0.04	0.03	0.02	0.03
1	1	0.73	1	t	1	Austroperla cyrene	Microspora sp.	55	0.04	0.03	0.02	0.03
1	1	0.85	1	t	1	Austroperla cyrene	Unidentified detritus	55	0.04	0.03	0.04	0.03
1	1	0.79	1	t	1	Austroperla cyrene	Gomphonema cf. olivaceum	55	0.04	0.03	0.03	0.03
1	1	0.85	1	cv	1	Austroperla cyrene	Eunotia sp.	55	0.04	0.03	0.04	0.03
1	1	0.61	1	t	1	Austroperla cyrene	moss cells	55	0.04	0.03	0.01	0.03
1	1	0.51	1	t	1	Austroperla cyrene	Fragilaria vaucheriae	55	0.04	0.03	0.00	0.03
1	1	0.71	1	t	1	Austroperla cyrene	Tabellaria flocculosa	55	0.04	0.03	0.02	0.03
1	1	0.76	1	cv	1	Austroperla cyrene	Diatoma heimale	55	0.04	0.03	0.03	0.03
1	1	0.70	1	t	1	Asterionella sp.	Deleatidium spp.	55	0.03	0.03	0.03	0.03
0	0	0.35	1	t	1	Potamopyrgus antipodarum	Gomphonema parvulum	55	0.00	0.03	0.02	0.03
0	0	0.32	1	t	1	Potamopyrgus antipodarum	Cyclotella sp.	55	0.00	0.03	0.02	0.03
1	1	0.56	1	cv	1	Potamopyrgus antipodarum	Unidentified detritus	55	0.00	0.03	0.04	0.03
0	0	0.46	1	t	1	Potamopyrgus antipodarum	Gomphonema cf. olivaceum	55	0.00	0.03	0.03	0.03
1	1	0.56	1	t	1	Potamopyrgus antipodarum	Eunotia sp.	55	0.00	0.03	0.04	0.03
0	0	0.27	1	t	1	Potamopyrgus antipodarum	Navicula avenacea	55	0.00	0.03	0.01	0.03
0	0	0.22	1	cv	1	Potamopyrgus antipodarum	Surirella tenera ??	55	0.00	0.03	0.01	0.03
0	0	0.48	1	t	1	Potamopyrgus antipodarum	Cymbella minuta	55	0.00	0.03	0.03	0.03

0	0	0.25	1	t	1	Potamopyrgus antipodarum	Gomphonema truncatum	55	0.00	0.03	0.01	0.03
0	0	0.19	1	t	1	Potamopyrgus antipodarum	Fragilaria vaucheriae	55	0.00	0.03	0.00	0.03
0	0	0.36	1	cv	1	Potamopyrgus antipodarum	Tabellaria flocculosa	55	0.00	0.03	0.02	0.03
0	0	0.38	1	t	1	Potamopyrgus antipodarum	Epithemia sorex	55	0.00	0.03	0.02	0.03
1	0	0.46	1	t	1	Deleatidium spp.	Navicula rhynchocephala	55	0.03	0.03	0.00	0.03
1	1	0.64	1	t	1	Deleatidium spp.	Gomphonema parvulum	55	0.03	0.03	0.02	0.03
0	1	0.51	1	cv	1	Deleatidium spp.	Pinnularia (small - mesolepta??)	55	0.03	0.03	0.01	0.03
1	0	0.47	1	t	1	Deleatidium spp.	Salmo trutta	55	0.03	0.03	0.00	0.03
1	1	0.61	1	t	1	Deleatidium spp.	Cyclotella sp.	55	0.03	0.03	0.02	0.03
1	1	0.79	1	t	1	Deleatidium spp.	Cocconeis sp.	55	0.03	0.03	0.04	0.03
1	1	0.67	1	cv	1	Deleatidium spp.	Microspora sp.	55	0.03	0.03	0.02	0.03
1	1	0.60	1	t	1	Deleatidium spp.	Achnanthes saxonica	55	0.03	0.03	0.02	0.03
1	1	0.67	1	t	1	Deleatidium spp.	Gomphonema subclavatum	55	0.03	0.03	0.02	0.03
1	1	0.74	1	t	1	Deleatidium spp.	Gomphonema cf. olivaceum	55	0.03	0.03	0.03	0.03
1	1	0.81	1	cv	1	Deleatidium spp.	Eunotia sp.	55	0.03	0.03	0.04	0.03
1	1	0.55	1	t	1	Deleatidium spp.	Navicula avenacea	55	0.03	0.03	0.01	0.03
1	1	0.56	1	t	1	Deleatidium spp.	Nitzschia sp. (large)	55	0.03	0.03	0.01	0.03
1	1	0.76	1	t	1	Deleatidium spp.	Cymbella minuta	55	0.03	0.03	0.03	0.03
1	1	0.66	1	cv	1	Deleatidium spp.	Synedra ulna	55	0.03	0.03	0.02	0.03
1	1	0.55	1	t	1	Deleatidium spp.	moss cells	55	0.03	0.03	0.01	0.03
0	0	0.44	1	t	1	Deleatidium spp.	Fragilaria vaucheriae	55	0.03	0.03	0.00	0.03
1	1	0.66	1	t	1	Deleatidium spp.	Tabellaria flocculosa	55	0.03	0.03	0.02	0.03
1	1	0.67	1	cv	1	Deleatidium spp.	Epithemia sorex	55	0.03	0.03	0.02	0.03

1	1	0.57	1	t	1	Deleatidium spp.	Costachorema xanthoptera	55	0.03	0.03	0.01	0.03
1	1	0.57	1	t	1	Deleatidium spp.	Psilachorema bidens	55	0.03	0.03	0.01	0.03
1	1	0.52	1	t	1	Navicula rhynchocephala	Oligochaeta type weak-long-short	55	0.00	0.03	0.03	0.03
1	0	0.47	1	cv	1	Navicula rhynchocephal	Unknown orthoclad	55	0.00	0.03	0.03	0.03
1	1	0.59	1	t	1	Navicula rhynchocephala	Autrosimilium australense	55	0.00	0.03	0.04	0.03
1	1	0.60	1	t	1	Navicula rhynchocephala	Aphrophila noevaezelandiae	55	0.00	0.03	0.04	0.03
1	1	0.66	1	t	1	Gomphonema parvulum	Scirtid sp. (Broad type)	55	0.02	0.03	0.03	0.03
1	1	0.55	1	cv	1	Gomphonema parvulum	Oxyethira albiceps	55	0.02	0.03	0.02	0.03
1	0	0.47	1	t	1	Gomphonema parvulum	Hydraena homalaena sp. (type H2)	55	0.02	0.03	0.02	0.03
1	1	0.65	1	t	1	Gomphonema parvulum	Amphipoda spp.	55	0.02	0.03	0.03	0.03
1	1	0.61	1	t	1	Gomphonema parvulum	Naonella	55	0.02	0.03	0.03	0.03
1	0	0.44	1	cv	1	Gomphonema parvulum	Zelandoperla sp	55	0.02	0.03	0.01	0.03
1	1	0.68	1	t	1	Gomphonema parvulum	Oligochaeta type weak-long-short	55	0.02	0.03	0.03	0.03
1	1	0.67	1	t	1	Gomphonema parvulum	Pycnocentrodes evecta	55	0.02	0.03	0.03	0.03
1	1	0.63	1	t	1	Gomphonema parvulum	Podonomus	55	0.02	0.03	0.03	0.03
1	1	0.74	1	cv	1	Gomphonema parvulum	Autrosimilium australense	55	0.02	0.03	0.04	0.03
1	0	0.47	1	t	1	Gomphonema parvulum	Scirtid sp. (Skinny type)	55	0.02	0.03	0.02	0.03
1	1	0.75	1	t	1	Gomphonema parvulum	Aphrophila noevaezelandiae	55	0.02	0.03	0.04	0.03
1	1	0.62	1	t	1	Gomphonema parvulum	Eukiefiriella	55	0.02	0.03	0.03	0.03
1	1	0.84	1	cv	1	Synedra rumpens	Autrosimilium australense	55	0.04	0.03	0.04	0.03
1	1	0.75	1	t	1	Synedra rumpens	Tanytarsini	55	0.04	0.03	0.03	0.03

0	0	0.30	1	t	1	Terrestrial invertebrates	Nannochorist a philpotti	55	0.00	0.03	0.01	0.03
0	0	0.23	1	t	1	Terrestrial invertebrates	Salmo trutta	55	0.00	0.03	0.00	0.03
0	0	0.24	1	cv	1	Terrestrial invertebrates	Larsia II	55	0.00	0.03	0.00	0.03
0	0	0.31	1	t	1	Terrestrial invertebrates	Psilachorema bidens	55	0.00	0.03	0.01	0.03
1	1	0.53	1	t	1	Nannochorista philpotti	Naonella	55	0.01	0.03	0.03	0.03
0	0	0.23	1	t	1	Pinnularia (small -	Hydora nitida larvae	55	0.01	0.03	0.00	0.03
1	1	0.55	1	cv	1	Pinnularia (small -	Pycnocentrod es evecta	55	0.01	0.03	0.03	0.03
1	1	0.51	1	t	1	Pinnularia (small -	Podonomus	55	0.01	0.03	0.03	0.03
1	0	0.49	1	t	1	Pinnularia (small -	Tanytarsini	55	0.01	0.03	0.03	0.03
1	1	0.64	1	t	1	Pinnularia (small - mesolepta??)	Aphrophila noevaezeland iae	55	0.01	0.03	0.04	0.03
1	0	0.49	1	cv	1	Pinnularia (small -	Eukiefiriella	55	0.01	0.03	0.03	0.03
1	0	0.39	1	t	1	Cymbella cuspidata	Hydora nitida larvae	55	0.02	0.03	0.00	0.03
1	0	0.49	1	t	1	Salmo trutta	Scirtid sp. (Broad type)	55	0.00	0.03	0.03	0.03
1	1	0.51	1	t	1	Salmo trutta	Pycnocentrod es evecta	55	0.00	0.03	0.03	0.03
1	1	0.59	1	cv	1	Salmo trutta	Autrosimiliu m australense	55	0.00	0.03	0.04	0.03
1	0	0.45	1	t	1	Salmo trutta	Tanytarsini	55	0.00	0.03	0.03	0.03
1	1	0.60	1	t	1	Salmo trutta	Aphrophila noevaezeland iae	55	0.00	0.03	0.04	0.03
0	0	0.30	1	t	1	Salmo trutta	Costachorem a xanthoptera	55	0.00	0.03	0.01	0.03
0	0	0.30	1	cv	1	Salmo trutta	Psilachorema bidens	55	0.00	0.03	0.01	0.03
1	1	0.62	1	t	1	Scirtid sp. (Broad type)	Cyclotella sp.	55	0.03	0.03	0.02	0.03
1	1	0.80	1	t	1	Scirtid sp. (Broad type)	Cocconeis sp.	55	0.03	0.03	0.04	0.03
1	1	0.81	1	t	1	Scirtid sp. (Broad type)	Unidentified detritus	55	0.03	0.03	0.04	0.03
1	1	0.76	1	cv	1	Scirtid sp. (Broad type)	Cymbella minuta	55	0.03	0.03	0.03	0.03
1	0	0.45	1	t	1	Scirtid sp. (Broad type)	Fragilaria vaucheriae	55	0.03	0.03	0.00	0.03

1	1	0.71	1	t	1	Scirtid sp. (Broad type)	Diatoma heimale	55	0.03	0.03	0.03	0.03
0	0	0.32	1	t	1	Coloburiscus humeralis	Cyclotella sp.	55	0.00	0.03	0.02	0.03
0	0	0.38	1	cv	1	Coloburiscus humeralis	Microspora sp.	55	0.00	0.03	0.02	0.03
1	1	0.56	1	t	1	Coloburiscus humeralis	Unidentified detritus	55	0.00	0.03	0.04	0.03
0	0	0.46	1	t	1	Coloburiscus humeralis	Gomphonem a cf. olivaceum	55	0.00	0.03	0.03	0.03
1	1	0.52	1	t	1	Cyclotella sp.	Oxyethira albiceps	55	0.02	0.03	0.02	0.03
1	0	0.44	1	cv	1	Cyclotella sp.	Hydraena homalaena sp. (type H2)	55	0.02	0.03	0.02	0.03
1	1	0.58	1	t	1	Cyclotella sp.	Naonella	55	0.02	0.03	0.03	0.03
0	0	0.31	1	t	1	Cyclotella sp.	Hydora nitida larvae	55	0.02	0.03	0.00	0.03
1	1	0.65	1	t	1	Cyclotella sp.	Oligochaeta type weak-long-short	55	0.02	0.03	0.03	0.03
1	1	0.61	1	cv	1	Cyclotella sp.	Unknown orthoclad	55	0.02	0.03	0.03	0.03
1	1	0.64	1	t	1	Cyclotella sp.	Pycnocentrod es evecta	55	0.02	0.03	0.03	0.03
1	1	0.61	1	t	1	Cyclotella sp.	Podonomus	55	0.02	0.03	0.03	0.03
1	1	0.71	1	t	1	Cyclotella sp.	Autrosimiliu m australense	55	0.02	0.03	0.04	0.03
1	0	0.44	1	cv	1	Cyclotella sp.	Scirtid sp. (Skinny type)	55	0.02	0.03	0.02	0.03
1	1	0.59	1	t	1	Cyclotella sp.	Tanytarsini	55	0.02	0.03	0.03	0.03
1	1	0.72	1	t	1	Cyclotella sp.	Aphrophila noevaezeland iae	55	0.02	0.03	0.04	0.03
1	1	0.59	1	t	1	Cyclotella sp.	Eukiefiriella	55	0.02	0.03	0.03	0.03
1	1	0.71	1	cv	1	Cocconeis sp.	Oxyethira albiceps	55	0.04	0.03	0.02	0.03
1	1	0.79	1	t	1	Cocconeis sp.	Amphipoda spp.	55	0.04	0.03	0.03	0.03
1	1	0.51	1	t	1	Cocconeis sp.	Hydora nitida larvae	55	0.04	0.03	0.00	0.03
1	1	0.61	1	t	1	Cocconeis sp.	Zelandoperla sp	55	0.04	0.03	0.01	0.03
1	1	0.81	1	cv	1	Cocconeis sp.	Oligochaeta type weak-long-short	55	0.04	0.03	0.03	0.03
1	1	0.78	1	t	1	Cocconeis sp.	Unknown orthoclad	55	0.04	0.03	0.03	0.03

1	1	0.80	1	t	1	Cocconeis sp.	Pycnocentrodes evecta	55	0.04	0.03	0.03	0.03
1	1	0.78	1	t	1	Cocconeis sp.	Podonomus	55	0.04	0.03	0.03	0.03
1	1	0.64	1	cv	1	Cocconeis sp.	Scirtid sp. (Skinny type)	55	0.04	0.03	0.02	0.03
1	1	0.77	1	t	1	Cocconeis sp.	Tanytarsini	55	0.04	0.03	0.03	0.03
1	1	0.86	1	t	1	Cocconeis sp.	Aphrophila noevaezelandiae	55	0.04	0.03	0.04	0.03
1	1	0.77	1	t	1	Cocconeis sp.	Eukiefiriella	55	0.04	0.03	0.03	0.03
1	1	0.55	1	cv	1	Oxyethira albiceps	Frustulia rhomboides	55	0.02	0.03	0.02	0.03
1	1	0.58	1	t	1	Oxyethira albiceps	Microspora sp.	55	0.02	0.03	0.02	0.03
1	1	0.74	1	t	1	Oxyethira albiceps	Unidentified detritus	55	0.02	0.03	0.04	0.03
1	1	0.66	1	t	1	Oxyethira albiceps	Gomphonema cf. olivaceum	55	0.02	0.03	0.03	0.03
1	1	0.74	1	cv	1	Oxyethira albiceps	Eunotia sp.	55	0.02	0.03	0.04	0.03
0	0	0.35	1	t	1	Oxyethira albiceps	Fragilaria vaucheriae	55	0.02	0.03	0.00	0.03
1	1	0.57	1	t	1	Oxyethira albiceps	Tabellaria flocculosa	55	0.02	0.03	0.02	0.03
1	0	0.45	1	t	1	Larsia II	Naonella	55	0.00	0.03	0.03	0.03
1	0	0.48	1	cv	1	Amphora sp.	Pycnocentrodes evecta	55	0.00	0.03	0.03	0.03
1	1	0.64	1	t	1	Frustulia rhomboides	Amphipoda spp.	55	0.02	0.03	0.03	0.03
0	0	0.34	1	t	1	Frustulia rhomboides	Hydora nitida larvae	55	0.02	0.03	0.00	0.03
1	1	0.68	1	t	1	Frustulia rhomboides	Oligochaeta type weak-long-short	55	0.02	0.03	0.03	0.03
1	1	0.63	1	cv	1	Frustulia rhomboides	Unknown orthoclad	55	0.02	0.03	0.03	0.03
1	1	0.67	1	t	1	Frustulia rhomboides	Pycnocentrodes evecta	55	0.02	0.03	0.03	0.03
1	1	0.74	1	t	1	Frustulia rhomboides	Autrosimilium australense	55	0.02	0.03	0.04	0.03
1	1	0.75	1	t	1	Frustulia rhomboides	Aphrophila noevaezelandiae	55	0.02	0.03	0.04	0.03
1	0	0.36	1	cv	1	Microspora sp.	Hydora nitida larvae	55	0.02	0.03	0.00	0.03
1	0	0.46	1	t	1	Microspora sp.	Zelandoperla sp	55	0.02	0.03	0.01	0.03
1	1	0.66	1	t	1	Microspora sp.	Unknown orthoclad	55	0.02	0.03	0.03	0.03

1	1	0.69	1	t	1	Microspora sp.	Pycnocentrodes evecta	55	0.02	0.03	0.03	0.03
1	1	0.76	1	cv	1	Microspora sp.	Autrosimilium australense	55	0.02	0.03	0.04	0.03
1	1	0.77	1	t	1	Microspora sp.	Aphrophila noevaezelandiae	55	0.02	0.03	0.04	0.03
1	1	0.67	1	t	1	Unidentified detritus	Hydraena homalaena sp. (type H2)	55	0.04	0.03	0.02	0.03
1	1	0.53	1	t	1	Unidentified detritus	Hydraena orchymontia	55	0.04	0.03	0.00	0.03
1	1	0.80	1	cv	1	Unidentified detritus	Amphipoda spp.	55	0.04	0.03	0.03	0.03
1	1	0.78	1	t	1	Unidentified detritus	Naonella	55	0.04	0.03	0.03	0.03
1	1	0.53	1	t	1	Unidentified detritus	Hydora nitida larvae	55	0.04	0.03	0.00	0.03
1	1	0.63	1	t	1	Unidentified detritus	Zelandoperla sp	55	0.04	0.03	0.01	0.03
1	1	0.82	1	cv	1	Unidentified detritus	Oligochaeta type weak-long-short	55	0.04	0.03	0.03	0.03
1	1	0.79	1	t	1	Unidentified detritus	Unknown orthoclad	55	0.04	0.03	0.03	0.03
1	1	0.82	1	t	1	Unidentified detritus	Pycnocentrodes evecta	55	0.04	0.03	0.03	0.03
1	1	0.79	1	t	1	Unidentified detritus	Podonomus	55	0.04	0.03	0.03	0.03
1	1	0.86	1	cv	1	Unidentified detritus	Autrosimilium australense	55	0.04	0.03	0.04	0.03
1	1	0.66	1	t	1	Unidentified detritus	Scirtid sp. (Skinny type)	55	0.04	0.03	0.02	0.03
1	1	0.78	1	t	1	Unidentified detritus	Tanytarsini	55	0.04	0.03	0.03	0.03
1	1	0.87	1	t	1	Unidentified detritus	Aphrophila noevaezelandiae	55	0.04	0.03	0.04	0.03
1	1	0.78	1	cv	1	Unidentified detritus	Eukiefiriella	55	0.04	0.03	0.03	0.03
0	0	0.38	1	t	1	Hydraena homalaena sp. (type H2)	Navicula avenacea	55	0.02	0.03	0.01	0.03
1	1	0.61	1	t	1	Hydraena homalaena sp. (type H2)	Cymbella minuta	55	0.02	0.03	0.03	0.03
0	0	0.49	1	t	1	Hydraena homalaena sp. (type H2)	Synedra ulna	55	0.02	0.03	0.02	0.03

0	0	0.29	1	cv	1	Hydraena homalaena sp. (type H2)	Fragilaria vaucheriae	55	0.02	0.03	0.00	0.03
1	0	0.49	1	t	1	Hydraena homalaena sp. (type H2)	Tabellaria flocculosa	55	0.02	0.03	0.02	0.03
1	1	0.59	1	t	1	Achnanthes saxonica	Podonomus	55	0.02	0.03	0.03	0.03
1	1	0.70	1	t	1	Achnanthes saxonica	Autrosimilium australense	55	0.02	0.03	0.04	0.03
1	1	0.71	1	cv	1	Achnanthes saxonica	Aphrophila noevaezelandiae	55	0.02	0.03	0.04	0.03
1	1	0.58	1	t	1	Achnanthes saxonica	Eukiefiriella	55	0.02	0.03	0.03	0.03
1	1	0.67	1	t	1	Gomphonema subclavatum	Amphipoda spp.	55	0.02	0.03	0.03	0.03
1	1	0.64	1	t	1	Gomphonema subclavatum	Naonella	55	0.02	0.03	0.03	0.03
1	0	0.37	1	cv	1	Gomphonema subclavatum	Hydora nitida larvae	55	0.02	0.03	0.00	0.03
1	1	0.70	1	t	1	Gomphonema subclavatum	Oligochaeta type weak-long-short	55	0.02	0.03	0.03	0.03
1	1	0.69	1	t	1	Gomphonema subclavatum	Pycnocentrodes evecta	55	0.02	0.03	0.03	0.03
1	1	0.76	1	t	1	Gomphonema subclavatum	Autrosimilium australense	55	0.02	0.03	0.04	0.03
1	1	0.77	1	cv	1	Gomphonema subclavatum	Aphrophila noevaezelandiae	55	0.02	0.03	0.04	0.03
1	1	0.65	1	t	1	Gomphonema subclavatum	Eukiefiriella	55	0.02	0.03	0.03	0.03
0	0	0.46	1	t	1	Ulothrix	Amphipoda spp.	55	0.00	0.03	0.03	0.03
1	1	0.74	1	t	1	Amphipoda spp.	Gomphonema cf. olivaceum	55	0.03	0.03	0.03	0.03
1	1	0.81	1	cv	1	Amphipoda	Eunotia sp.	55	0.03	0.03	0.04	0.03
1	1	0.74	1	t	1	Amphipoda spp.	Navicula cryptocephala	55	0.03	0.03	0.03	0.03
1	1	0.76	1	t	1	Amphipoda spp.	Cymbella minuta	55	0.03	0.03	0.03	0.03
1	1	0.54	1	t	1	Amphipoda	moss cells	55	0.03	0.03	0.01	0.03
0	0	0.44	1	cv	1	Amphipoda spp.	Fragilaria vaucheriae	55	0.03	0.03	0.00	0.03
1	1	0.56	1	t	1	Amphipoda spp.	Psilachorema bidens	55	0.03	0.03	0.01	0.03

1	0	0.44	1	t	1	Gomphonema cf. olivaceum	Hydora nitida larvae	55	0.03	0.03	0.00	0.03
1	1	0.76	1	t	1	Gomphonema cf. olivaceum	Oligochaeta type weak-long-short	55	0.03	0.03	0.03	0.03
1	1	0.75	1	cv	1	Gomphonema cf. olivaceum	Pycnocentrodes evecta	55	0.03	0.03	0.03	0.03
1	1	0.72	1	t	1	Gomphonema cf. olivaceum	Podonomus	55	0.03	0.03	0.03	0.03
1	1	0.81	1	t	1	Gomphonema cf. olivaceum	Autrosimilium australense	55	0.03	0.03	0.04	0.03
1	1	0.71	1	t	1	Gomphonema cf. olivaceum	Tanytarsini	55	0.03	0.03	0.03	0.03
1	1	0.82	1	cv	1	Gomphonema cf. olivaceum	Aphrophila noevaezelandiae	55	0.03	0.03	0.04	0.03
1	1	0.73	1	t	1	Naonella	Cymbella minuta	55	0.03	0.03	0.03	0.03
1	1	0.62	1	t	1	Naonella	Synedra ulna	55	0.03	0.03	0.02	0.03
0	0	0.41	1	t	1	Naonella	Fragilaria vaucheriae	55	0.03	0.03	0.00	0.03
1	1	0.62	1	cv	1	Naonella	Tabellaria flocculosa	55	0.03	0.03	0.02	0.03
1	1	0.53	1	t	1	Naonella	Costachorema xanthoptera	55	0.03	0.03	0.01	0.03
1	1	0.53	1	t	1	Naonella	Psilachorema bidens	55	0.03	0.03	0.01	0.03
1	0	0.45	1	t	1	Surirella (small)	Hydora nitida larvae	55	0.03	0.03	0.00	0.03
0	1	0.56	1	cv	1	Hydora nitida larvae	Eunotia sp.	55	0.00	0.03	0.04	0.03
0	0	0.46	1	t	1	Hydora nitida larvae	Navicula cryptocephala	55	0.00	0.03	0.03	0.03
0	0	0.27	1	t	1	Hydora nitida larvae	Navicula avenacea	55	0.00	0.03	0.01	0.03
0	0	0.28	1	t	1	Hydora nitida larvae	Nitzschia sp. (large)	55	0.00	0.03	0.01	0.03
0	0	0.48	1	cv	1	Hydora nitida larvae	Cymbella minuta	55	0.00	0.03	0.03	0.03
0	0	0.25	1	t	1	Hydora nitida larvae	Gomphonema truncatum	55	0.00	0.03	0.01	0.03
0	0	0.27	1	t	1	Hydora nitida larvae	moss cells	55	0.00	0.03	0.01	0.03
0	0	0.19	1	t	1	Hydora nitida larvae	Fragilaria vaucheriae	55	0.00	0.03	0.00	0.03
0	0	0.36	1	cv	1	Hydora nitida larvae	Tabellaria flocculosa	55	0.00	0.03	0.02	0.03

0	0	0.38	1	t	1	Hydora nitida larvae	Epithemia sorex	55	0.00	0.03	0.02	0.03
0	0	0.42	1	t	1	Hydora nitida larvae	Diatoma heimale	55	0.00	0.03	0.03	0.03
1	1	0.82	1	t	1	Eunotia sp.	Oligochaeta type weak-long-short	55	0.04	0.03	0.03	0.03
1	1	0.82	1	cv	1	Eunotia sp.	Pycnocentrodes evecta	55	0.04	0.03	0.03	0.03
1	1	0.79	1	t	1	Eunotia sp.	Podonomus	55	0.04	0.03	0.03	0.03
1	1	0.86	1	t	1	Eunotia sp.	Autrosimilium australense	55	0.04	0.03	0.04	0.03
1	1	0.78	1	t	1	Eunotia sp.	Tanytarsini	55	0.04	0.03	0.03	0.03
1	1	0.87	1	cv	1	Eunotia sp.	Aphrophila noevaezelandiae	55	0.04	0.03	0.04	0.03
1	1	0.78	1	t	1	Eunotia sp.	Eukiefiriella	55	0.04	0.03	0.03	0.03
1	1	0.54	1	t	1	Navicula cryptocephala	Zelandoperla sp	55	0.03	0.03	0.01	0.03
1	1	0.82	1	t	1	Navicula cryptocephala	Aphrophila noevaezelandiae	55	0.03	0.03	0.04	0.03
1	1	0.59	1	cv	1	Navicula avenacea	Oligochaeta type weak-long-short	55	0.01	0.03	0.03	0.03
1	1	0.54	1	t	1	Navicula avenacea	Unknown orthoclad	55	0.01	0.03	0.03	0.03
1	1	0.54	1	t	1	Navicula avenacea	Podonomus	55	0.01	0.03	0.03	0.03
1	1	0.53	1	t	1	Navicula avenacea	Eukiefiriella	55	0.01	0.03	0.03	0.03
1	1	0.61	1	cv	1	Nitzschia sp. (large)	Oligochaeta type weak-long-short	55	0.01	0.03	0.03	0.03
1	1	0.56	1	t	1	Nitzschia sp. (large)	Unknown orthoclad	55	0.01	0.03	0.03	0.03
1	1	0.56	1	t	1	Nitzschia sp. (large)	Podonomus	55	0.01	0.03	0.03	0.03
1	1	0.67	1	t	1	Nitzschia sp. (large)	Autrosimilium australense	55	0.01	0.03	0.04	0.03
1	1	0.66	1	cv	1	Zelandoperla sp	Autrosimilium australense	55	0.01	0.03	0.04	0.03
0	1	0.54	1	t	1	Surirella tenera ??	Oligochaeta type weak-long-short	55	0.01	0.03	0.03	0.03
0	0	0.47	1	t	1	Surirella tenera ??	Tanytarsini	55	0.01	0.03	0.03	0.03

1	1	0.74	1	t	1	Cymbella minuta	Unknown orthoclad	55	0.03	0.03	0.03	0.03
1	1	0.77	1	cv	1	Cymbella minuta	Pycnocentrodes evecta	55	0.03	0.03	0.03	0.03
1	1	0.74	1	t	1	Cymbella minuta	Podonomus	55	0.03	0.03	0.03	0.03
1	1	0.83	1	t	1	Cymbella minuta	Autrosimilium australense	55	0.03	0.03	0.04	0.03
1	1	0.60	1	t	1	Cymbella minuta	Scirtid sp. (Skinny type)	55	0.03	0.03	0.02	0.03
1	1	0.73	1	cv	1	Cymbella minuta	Tanytarsini	55	0.03	0.03	0.03	0.03
1	1	0.83	1	t	1	Cymbella minuta	Aphrophila noevaezelandiae	55	0.03	0.03	0.04	0.03
1	1	0.73	1	t	1	Cymbella minuta	Eukiefiriella	55	0.03	0.03	0.03	0.03
1	1	0.53	1	t	1	Gomphonema truncatum	Unknown orthoclad	55	0.01	0.03	0.03	0.03
1	1	0.64	1	cv	1	Gomphonema truncatum	Autrosimilium australense	55	0.01	0.03	0.04	0.03
1	1	0.69	1	t	1	Oligochaeta type weak-long-short	Synedra ulna	55	0.03	0.03	0.02	0.03
1	0	0.47	1	t	1	Oligochaeta type weak-long-short	Fragilaria vaucheriae	55	0.03	0.03	0.00	0.03
1	1	0.73	1	t	1	Oligochaeta type weak-long-short	Diatoma heimale	55	0.03	0.03	0.03	0.03
1	1	0.65	1	cv	1	Synedra ulna	Podonomus	55	0.02	0.03	0.03	0.03
1	1	0.75	1	t	1	Synedra ulna	Autrosimilium australense	55	0.02	0.03	0.04	0.03
1	1	0.64	1	t	1	Synedra ulna	Eukiefiriella	55	0.02	0.03	0.03	0.03
1	1	0.67	1	t	1	moss cells	Aphrophila noevaezelandiae	55	0.01	0.03	0.04	0.03
0	0	0.43	1	cv	1	Unknown orthoclad	Fragilaria vaucheriae	55	0.03	0.03	0.00	0.03
1	1	0.64	1	t	1	Unknown orthoclad	Tabellaria flocculosa	55	0.03	0.03	0.02	0.03
1	1	0.66	1	t	1	Unknown orthoclad	Epithemia sorex	55	0.03	0.03	0.02	0.03
1	0	0.46	1	t	1	Pycnocentrodes evecta	Fragilaria vaucheriae	55	0.03	0.03	0.00	0.03
1	1	0.68	1	cv	1	Pycnocentrodes evecta	Tabellaria flocculosa	55	0.03	0.03	0.02	0.03

1	1	0.72	1	t	1	Pycnocentrodes evecta	Diatoma heimale	55	0.03	0.03	0.03	0.03
0	0	0.43	1	t	1	Podonomus	Fragilaria vaucheriae	55	0.03	0.03	0.00	0.03
1	1	0.54	1	t	1	Autrosimilium australense	Fragilaria vaucheriae	55	0.04	0.03	0.00	0.03
1	1	0.74	1	cv	1	Autrosimilium australense	Tabellaria flocculosa	55	0.04	0.03	0.02	0.03
1	1	0.66	1	t	1	Autrosimilium australense	Costachorema xanthoptera	55	0.04	0.03	0.01	0.03
1	1	0.78	1	t	1	Autrosimilium australense	Diatoma heimale	55	0.04	0.03	0.03	0.03
0	0	0.29	1	t	1	Scirtid sp. (Skinny type)	Fragilaria vaucheriae	55	0.02	0.03	0.00	0.03
1	1	0.55	1	cv	1	Scirtid sp. (Skinny type)	Diatoma heimale	55	0.02	0.03	0.03	0.03
0	0	0.43	1	t	1	Fragilaria vaucheriae	Tanytarsini	55	0.00	0.03	0.03	0.03
1	1	0.58	1	t	1	Fragilaria vaucheriae	Aphrophila noevaezelandiae	55	0.00	0.03	0.04	0.03
0	0	0.43	1	t	1	Fragilaria vaucheriae	Eukiefiriella	55	0.00	0.03	0.03	0.03
1	1	0.68	1	cv	1	Tanytarsini	Diatoma heimale	55	0.03	0.03	0.03	0.03
1	1	0.75	1	t	1	Aphrophila noevaezelandiae	Tabellaria flocculosa	55	0.04	0.03	0.02	0.03
1	1	0.79	1	t	1	Aphrophila noevaezelandiae	Diatoma heimale	55	0.04	0.03	0.03	0.03
1	1	0.63	1	t	1	Tabellaria flocculosa	Eukiefiriella	55	0.02	0.03	0.03	0.03
1	1	0.54	1	cv	1	Eukiefiriella	Costachorema xanthoptera	55	0.03	0.03	0.01	0.03
1	1	0.68	1	t	1	Eukiefiriella	Diatoma heimale	55	0.03	0.03	0.03	0.03
1	1	0.56	0	t	1	Gomphonema sp. (skinny - tenue??)	Autrosimilium australense	55	0.00	0.03	0.04	0.03
0	1	0.62	0	t	1	Tanyderid sp.	Amphipoda spp.	55	0.02	0.03	0.03	0.03
0	1	0.55	0	cv	1	Hudsonema aliena	Podonomus	55	0.01	0.03	0.03	0.03
0	0	0.27	0	t	1	Polypedellum	Hudsonema aliena	55	0.00	0.03	0.01	0.03
1	1	0.72	0	t	1	Hydora nitida adult	Amphipoda spp.	55	0.03	0.03	0.03	0.03

1	1	0.77	0	t	1	Unidentified detritus	Diatoma heimale	55	0.04	0.03	0.03	0.03
1	0	0.41	0	cv	1	Macrophyte	Meridion circulare	55	0.02	0.03	0.01	0.03
1	0	0.47	0	t	1	Philorheithrus agilis	Pinnularia (small - mesolepta??)	55	0.03	0.03	0.01	0.03
1	0	0.49	0	t	1	Meridion circulare	Microspora sp.	55	0.01	0.03	0.02	0.03
1	0	0.47	0	t	1	Rhoicosphenia curvata	Tabellaria flocculosa	55	0.01	0.03	0.02	0.03
1	0	0.45	0	cv	1	Oxyethira albiceps	moss cells	55	0.02	0.03	0.01	0.03
0	0	0.30	0	t	1	Macrophyte	Gomphoneis herculeana	55	0.02	0.03	0.00	0.03
1	1	0.66	0	t	1	Eunotia sp.	Scirtid sp. (Skinny type)	55	0.04	0.03	0.02	0.03
1	1	0.50	0	t	1	Navicula rhynchocephal	Pycnocentrodes evecta	55	0.00	0.03	0.03	0.03
0	0	0.30	0	cv	1	Navicula cf. radiosa	Psilachorema bidens	55	0.00	0.03	0.01	0.03
1	1	0.50	0	t	1	Gomphonema parvulum	Cyclotella sp.	55	0.02	0.03	0.02	0.03
1	1	0.55	0	t	1	Gomphonema parvulum	Synedra ulna	55	0.02	0.03	0.02	0.03
1	0	0.39	0	t	1	Nannochorista philpotti	Hydraena homalaena sp. (type H2)	55	0.01	0.03	0.02	0.03
1	1	0.82	0	cv	1	Olinga	Podonomus	55	0.04	0.03	0.03	0.03
1	1	0.79	0	t	1	Achnanthes lanceoloata	Achnanthes linearis	55	0.04	0.03	0.03	0.03
0	0	0.25	0	t	1	Eunotia sp. large	Amphora sp.	55	0.01	0.03	0.00	0.03
1	1	0.87	0	t	1	Olinga feredayi	Austroperla cyrene	55	0.04	0.03	0.04	0.03
1	0	0.49	0	cv	1	Pycnocentria spp.	Gomphonema subclavatum	55	0.01	0.03	0.02	0.03
1	1	0.62	0	t	1	Asterionella sp.	Microspora sp.	55	0.03	0.03	0.02	0.03
1	1	0.71	0	t	1	Aulacoseira sp.	Scirtid sp. (Broad type)	55	0.03	0.03	0.03	0.03
1	1	0.69	0	t	1	Synedra rumpens	Synedra ulna	55	0.04	0.03	0.02	0.03
1	0	0.50	0	cv	1	Gomphonema sp. (skinny - tenue??)	Synedra rumpens	55	0.00	0.03	0.04	0.03
1	1	0.54	0	t	1	Blue-green algae	Synedra rumpens	55	0.01	0.03	0.04	0.03
0	0	0.27	0	t	1	Closterium sp.	Rhoicosphenia curvata	55	0.00	0.03	0.01	0.03

1	0	0.33	0	t	1	Achnanthes saxonica	Surirella tenera ??	55	0.02	0.03	0.01	0.03
1	0	0.35	0	cv	1	Gomphonema sp. (skinny - tenue??)	Hydrobiosid spp.	55	0.00	0.03	0.02	0.03
1	1	0.78	0	t	1	Hydora nitida adult	Austroperla cyrene	55	0.03	0.03	0.04	0.03
1	1	0.67	0	t	1	Olinga feredayi	Navicula avenacea	55	0.04	0.03	0.01	0.03
0	1	0.58	0	t	1	Polypedellum	Aphrophila noevaezelandiae	55	0.00	0.03	0.04	0.03
1	1	0.79	0	cv	1	Synedra rumpens	Oligochaeta type weak-long-short	55	0.04	0.03	0.03	0.03
1	0	0.47	0	t	1	unknown red algal spp.	Hydora nitida larvae	55	0.03	0.03	0.00	0.03
0	0	0.45	0	t	1	Terrestrial invertebrates	Diatoma heimale	55	0.00	0.03	0.03	0.03
1	0	0.38	0	t	1	Navicula sp.	Navicula rhynchocephala	55	0.02	0.03	0.00	0.03
1	1	0.72	0	cv	1	Achnanthes lanceoloata	Cymbella cuspidata	55	0.04	0.03	0.02	0.03
1	1	0.62	0	t	1	Navicula sp.	Asterionella sp.	55	0.02	0.03	0.03	0.03
1	1	0.82	0	t	1	Olinga feredayi	Navicula cryptocephala	55	0.04	0.03	0.03	0.03
1	0	0.50	0	t	1	Achnanthes linearis	Terrestrial invertebrates	55	0.03	0.03	0.00	0.03
1	1	0.77	0	cv	1	Olinga feredayi	Microspora sp.	55	0.04	0.03	0.02	0.03
0	0	0.28	0	t	1	Amphora sp.	Psilachorema bidens	55	0.00	0.03	0.01	0.03
0	0	0.42	0	t	1	Coloburiscus humeralis	Diatoma heimale	55	0.00	0.03	0.03	0.03
0	0	0.43	0	t	1	Potamopyrgus antipodarum	Tanytarsini	55	0.00	0.03	0.03	0.03
1	1	0.60	0	cv	1	Rhoicosphenia curvata	unknown red algal spp.	55	0.01	0.03	0.03	0.03
1	1	0.79	0	t	1	Pinnularia sp. (large)	Synedra rumpens	55	0.03	0.03	0.04	0.03
1	1	0.53	0	t	1	Eunotia sp. large	Eukiefiriella	55	0.01	0.03	0.03	0.03
0	0	0.20	0	t	1	Gomphonema sp. (skinny - tenue??)	Navicula cf. radiosa	55	0.00	0.03	0.00	0.03
1	1	0.68	0	cv	1	Asterionella	Eukiefiriella	55	0.03	0.03	0.03	0.03
1	1	0.60	0	t	1	Philorheithrus agilis	Gomphonema parvulum	55	0.03	0.03	0.02	0.03

0	0	0.22	0	t	1	Potamopyrgus antipodarum	Terrestrial invertebrates	55	0.00	0.03	0.00	0.03
1	1	0.65	0	t	1	Stauroneis sp.	Scirtid sp. (Skinny type)	55	0.04	0.03	0.02	0.03
1	1	0.60	0	cv	1	Cymbella cuspidata	Tabellaria flocculosa	55	0.02	0.03	0.02	0.03
1	1	0.67	0	t	1	Rhoicosphenia curvata	Autrosimiliu m australense	55	0.01	0.03	0.04	0.03
0	0	0.26	0	t	1	Zelandoperla sp	Fragilaria vaucheriae	55	0.01	0.03	0.00	0.03
1	0	0.34	0	t	1	Blue-green algae	Macrophyte	55	0.01	0.03	0.02	0.03
0	0	0.22	0	cv	1	Maoridiamese a	Melosira varians	55	0.01	0.03	0.00	0.03
1	0	0.48	0	t	1	Asterionella sp.	Gomphonem a truncatum	55	0.03	0.03	0.01	0.03
1	1	0.67	0	t	1	Nitzschia sp. (small)	Frustulia rhomboides	55	0.03	0.03	0.02	0.03
0	1	0.54	0	t	1	Fragilaria spp.	Gomphonem a cf. olivaceum	55	0.01	0.02	0.03	0.03
1	1	0.72	0	cv	1	Hydora nitida adult	Navicula cryptocephal a	55	0.03	0.03	0.03	0.03
1	1	0.68	0	t	1	Olinga feredayi	Nitzschia sp. (large)	55	0.04	0.03	0.01	0.03
0	0	0.48	0	t	1	Navicula cf. radiosa	Surirella (small)	55	0.00	0.03	0.03	0.03
0	0	0.19	0	t	1	Polypedellum	Amphora sp.	55	0.00	0.03	0.00	0.03
0	0	0.26	0	cv	1	Amphora sp.	Navicula avenacea	55	0.00	0.03	0.01	0.03
1	1	0.68	0	t	1	Hydrobiosid spp.	Cymbella minuta	55	0.02	0.03	0.03	0.03
0	0	0.26	0	t	1	Amphora sp.	Zelandoperla sp	55	0.00	0.03	0.01	0.03
1	1	0.55	0	t	1	Meridion circulare	Eukiefiriella	55	0.01	0.03	0.03	0.03
0	0	0.25	0	cv	1	Maoridiamese a	Surirella tenera ??	55	0.01	0.03	0.01	0.03
1	1	0.60	0	t	1	Nitzschia sp. (small)	Costachorem a xanthoptera	55	0.03	0.03	0.01	0.03
1	1	0.50	0	t	1	Oxyethira albiceps	Achnanthes saxonica	55	0.02	0.03	0.02	0.03
1	1	0.56	0	t	1	Oligochaeta type II	Surirella (small)	55	0.01	0.03	0.03	0.03
1	0	0.47	0	cv	1	Rhoicosphenia curvata	Hydrobiosid spp.	55	0.01	0.03	0.02	0.03
1	1	0.85	0	t	1	Pirara	Eunotia sp.	55	0.04	0.03	0.04	0.03

1	0	0.27	0	t	1	Larsia II	Gomphonema truncatum	55	0.00	0.03	0.01	0.03
1	1	0.52	0	t	1	Macrophyte	Navicula sp.	55	0.02	0.03	0.02	0.03
0	0	0.38	0	cv	1	Pycnocentria spp.	Rhopalodia novae zelandicae	55	0.01	0.03	0.01	0.03
1	1	0.52	0	t	1	Aulacoseira sp.	Rhoicosphenia curvata	55	0.03	0.03	0.01	0.03
0	0	0.42	0	t	1	Polypedellum	Philorheithrus agilis	55	0.00	0.03	0.03	0.03
1	1	0.66	0	t	1	Achnanthes linearis	Gomphonema parvulum	55	0.03	0.03	0.02	0.03
0	0	0.26	0	cv	1	Eunotia sp. large	Coloburiscus humeralis	55	0.01	0.03	0.00	0.03
1	1	0.57	0	t	1	Navicula rhynchocephal	Unidentified detritus	55	0.00	0.03	0.04	0.03
1	1	0.74	0	t	1	Pycnocentrodes evecta	Eukiefiriella	55	0.03	0.03	0.03	0.03
0	0	0.23	0	t	1	Polyplectropus puerilis	Terrestrial invertebrates	55	0.00	0.03	0.00	0.03
1	1	0.80	0	cv	1	Pirara	Amphipoda spp.	55	0.04	0.03	0.03	0.03
1	1	0.67	0	t	1	Cymbella aspera	Tanytarsini	55	0.03	0.03	0.03	0.03
1	0	0.47	0	t	1	Pycnocentria spp.	Hydrobiosid spp.	55	0.01	0.03	0.02	0.03
1	1	0.57	0	t	1	Navicula sp.	Tabellaria flocculosa	55	0.02	0.03	0.02	0.03
0	1	0.51	0	cv	1	Maoridiamesea	Deleatidium spp.	55	0.01	0.03	0.03	0.03
0	0	0.46	0	t	1	Hudsonema aliena	Oxyethira albiceps	55	0.01	0.03	0.02	0.03
1	1	0.74	0	t	1	unknown red algal spp.	Eukiefiriella	55	0.03	0.03	0.03	0.03
1	1	0.55	0	t	1	Cymbella cuspidata	Cyclotella sp.	55	0.02	0.03	0.02	0.03
1	1	0.64	0	cv	1	Meridion circulare	Austroperla cyrene	55	0.01	0.03	0.04	0.03
0	0	0.48	0	t	1	Maoridiamese	Eukiefiriella	55	0.01	0.03	0.03	0.03
0	0	0.39	0	t	1	Oligochaeta type II	Hydraena homalaena sp. (type H2)	55	0.01	0.03	0.02	0.03
1	1	0.62	0	t	1	Austroperla cyrene	Nannochorista philpotti	55	0.04	0.03	0.01	0.03
1	1	0.74	0	cv	1	Naonella	Oligochaeta type weak-long-short	55	0.03	0.03	0.03	0.03
1	1	0.65	0	t	1	Gomphonema cf. olivaceum	Synedra ulna	55	0.03	0.03	0.02	0.03
0	0	0.21	0	t	1	Larsia II	Amphora sp.	55	0.00	0.03	0.00	0.03

0	1	0.58	0	t	1	Fragilaria spp.	Nitzschia sp. (small)	55	0.01	0.02	0.03	0.03
0	0	0.19	0	cv	1	Closterium sp.	Polypedellum	55	0.00	0.03	0.00	0.03
0	0	0.20	0	t	1	Closterium sp.	Salmo trutta	55	0.00	0.03	0.00	0.03
1	1	0.58	0	t	1	Navicula sp.	Synedra ulna	55	0.02	0.03	0.02	0.03
1	1	0.71	0	t	1	Achnanthes linearis	Asterionella sp.	55	0.03	0.03	0.03	0.03
1	1	0.52	0	cv	1	Aulacoseira sp.	Meridion circulare	55	0.03	0.03	0.01	0.03
1	1	0.63	0	t	1	Oligochaeta type I	Asterionella sp.	55	0.02	0.03	0.03	0.03
1	1	0.57	0	t	1	Achnanthes linearis	Zelandoperla sp	55	0.03	0.03	0.01	0.03
0	1	0.62	0	t	1	Tanyderid sp.	Surirella (small)	55	0.02	0.03	0.03	0.03
0	0	0.28	0	cv	1	Potamopyrgus antipodarum	Psilachorema bidens	55	0.00	0.03	0.01	0.03
1	1	0.67	0	t	1	Gomphonema cf. olivaceum	Epithemia sorex	55	0.03	0.03	0.02	0.03
1	1	0.52	0	t	1	Achnanthes linearis	Pinnularia (small - mesolepta??)	55	0.03	0.03	0.01	0.03
1	1	0.52	0	t	1	Cyclotella sp.	Tabellaria flocculosa	55	0.02	0.03	0.02	0.03
1	0	0.50	0	cv	1	Navicula sp.	Scirtid sp. (Skinny type)	55	0.02	0.03	0.02	0.03
0	0	0.33	0	t	1	Blue-green algae	Scirtid sp. (Skinny type)	55	0.01	0.03	0.02	0.03
1	1	0.74	0	t	1	Oligochaeta type weak-long-short	Tanytarsini	55	0.03	0.03	0.03	0.03
0	0	0.45	0	t	1	Hydora nitida larvae	Podonomus	55	0.00	0.03	0.03	0.03
0	0	0.49	0	cv	1	Potamopyrgus antipodarum	Pycnocentrodes evecta	55	0.00	0.03	0.03	0.03
1	1	0.53	0	t	1	Gomphonema sp. (skinny - tenue??)	Austroperla cyrene	55	0.00	0.03	0.04	0.03
1	1	0.58	0	t	1	Salmo trutta	Eunotia sp.	55	0.00	0.03	0.04	0.03
1	1	0.68	0	t	1	Pirara	Tanyderid sp.	55	0.04	0.03	0.02	0.03
0	0	0.32	0	cv	1	Cyclotella sp.	Gomphoneis herculeana	55	0.02	0.03	0.00	0.03
0	0	0.23	0	t	1	Cymbella kappii	Coloburiscus humeralis	55	0.01	0.03	0.00	0.03
1	1	0.89	0	t	1	Olinga feredayi	Aphrophila noevaezelandiae	55	0.04	0.03	0.04	0.03
1	1	0.64	0	t	1	Rhopalodia novae zelandicae	Cocconeis sp.	55	0.01	0.03	0.04	0.03

0	0	0.29	0	cv	1	Hydraena homalaena sp. (type H2)	Hydraena orchymontia	55	0.02	0.03	0.00	0.03
0	0	0.30	0	t	1	Maoridiamesea	Navicula avenacea	55	0.01	0.03	0.01	0.03
1	1	0.64	0	t	1	Navicula sp.	Tanytarsini	55	0.02	0.03	0.03	0.03
0	0	0.30	0	t	1	Polyplectropus puerilis	Meridion circulare	55	0.00	0.03	0.01	0.03
0	0	0.31	0	cv	1	Cyclotella sp.	Hydraena orchymontia	55	0.02	0.03	0.00	0.03
1	1	0.64	0	t	1	Rhoicosphenia curvata	Austroperla cyrene	55	0.01	0.03	0.04	0.03
1	1	0.60	0	t	1	Achnanthes linearis	Scirtid sp. (Skinny type)	55	0.03	0.03	0.02	0.03
1	0	0.44	0	t	1	Cymbella kappii	Epithemia sorex	55	0.01	0.03	0.02	0.03
1	1	0.76	0	cv	1	Asterionella sp.	Cocconeis sp.	55	0.03	0.03	0.04	0.03
0	0	0.31	0	t	1	Cyclotella sp.	Ulothrix	55	0.02	0.03	0.00	0.03
1	1	0.55	0	t	1	Hydraena orchymontia	Eunotia sp.	55	0.00	0.03	0.04	0.03
0	0	0.24	0	t	1	Terrestrial invertebrates	Surirella tenera ??	55	0.00	0.03	0.01	0.03
1	1	0.82	0	cv	1	Synedra rumpens	Cocconeis sp.	55	0.04	0.03	0.04	0.03
0	1	0.61	0	t	1	Larsia II	Aphrophila noevaezelandiae	55	0.00	0.03	0.04	0.03
1	0	0.48	0	t	1	Scirtid sp. (Broad type)	Larsia II	55	0.03	0.03	0.00	0.03
1	1	0.80	0	t	1	Pirara	Deleatidium spp.	55	0.04	0.03	0.03	0.03
0	0	0.24	0	cv	1	Potamopyrgus antipodarum	Pinnularia (small - mesolepta??)	55	0.00	0.03	0.01	0.03
0	0	0.45	0	t	1	Helicopsyche albescens	Unknown orthoclad	55	0.00	0.03	0.03	0.03
1	1	0.52	0	t	1	Navicula rhynchocephal	Synedra rumpens	55	0.00	0.03	0.04	0.03
0	1	0.52	0	t	1	Pirara	Hydora nitida larvae	55	0.04	0.03	0.00	0.03
0	0	0.27	0	cv	1	Rhoicosphenia curvata	Coloburiscus humeralis	55	0.01	0.03	0.00	0.03
1	0	0.37	0	t	1	Closterium sp.	Epithemia sorex	55	0.00	0.03	0.02	0.03
0	0	0.45	0	t	1	Hydrobiosid spp.	Nannochorista philpotti	55	0.02	0.03	0.01	0.03
1	0	0.43	0	t	1	Gomphonema parvulum	Navicula avenacea	55	0.02	0.03	0.01	0.03
1	1	0.65	0	cv	1	Navicula sp.	Eukiefiriella	55	0.02	0.03	0.03	0.03

1	1	0.65	0	t	1	Aulacoseira sp.	Cymbella aspera	55	0.03	0.03	0.03	0.03
1	1	0.80	0	t	1	Stauroneis sp.	Deleatidium spp.	55	0.04	0.03	0.03	0.03
1	1	0.64	0	t	1	Microspora sp.	Naonella	55	0.02	0.03	0.03	0.03
1	1	0.57	0	cv	1	Olinga feredayi	Hydraena orchymontia	55	0.04	0.03	0.00	0.03
1	1	0.64	0	t	1	Navicula sp.	Naonella	55	0.02	0.03	0.03	0.03
0	0	0.22	0	t	1	Closterium sp.	Surirella tenera ??	55	0.00	0.03	0.01	0.03
1	1	0.54	0	t	1	Philorheithrus agilis	Scirtid sp. (Skinny type)	55	0.03	0.03	0.02	0.03
1	1	0.53	0	cv	1	Eunotia sp.	Fragilaria vaucheriae	55	0.04	0.03	0.00	0.03
0	0	0.26	0	t	1	Melosira varians	moss cells	55	0.00	0.03	0.01	0.03
1	1	0.66	0	t	1	Gomphonema subclavatum	Unknown orthoclad	55	0.02	0.03	0.03	0.03
0	0	0.24	0	t	1	Maoridiamese	Salmo trutta	55	0.01	0.03	0.00	0.03
1	1	0.64	0	cv	1	Meridion circulare	Cocconeis sp.	55	0.01	0.03	0.04	0.03
0	0	0.35	0	t	1	Oligochaeta type II	Zelandoperla sp	55	0.01	0.03	0.01	0.03
1	1	0.66	0	t	1	Cymbella aspera	Naonella	55	0.03	0.03	0.03	0.03
1	1	0.65	0	t	1	Synedra rumpens	Tanyderid sp.	55	0.04	0.03	0.02	0.03
0	0	0.41	0	cv	1	Asterionella sp.	Navicula cf. radiosa	55	0.03	0.03	0.00	0.03
0	0	0.47	0	t	1	Oligochaeta type I	Oligochaeta type II	55	0.02	0.03	0.01	0.03
0	0	0.19	0	t	1	Closterium sp.	Potamopyrgus antipodarum	55	0.00	0.03	0.00	0.03
0	0	0.26	0	t	1	Hudsonema aliena	Melosira varians	55	0.01	0.03	0.00	0.03
1	1	0.73	0	cv	1	Frustulia rhomboides	Eunotia sp.	55	0.02	0.03	0.04	0.03
1	1	0.70	0	t	1	Cyclotella sp.	Eunotia sp.	55	0.02	0.03	0.04	0.03
0	0	0.37	0	t	1	Hydora nitida larvae	Synedra ulna	55	0.00	0.03	0.02	0.03
0	0	0.22	0	t	1	Polypedellum	Terrestrial invertebrates	55	0.00	0.03	0.00	0.03
0	1	0.59	0	cv	1	Hydora nitida adult	Tanyderid sp.	55	0.03	0.03	0.02	0.03
1	1	0.79	0	t	1	Stauroneis sp.	Podonomus	55	0.04	0.03	0.03	0.03
0	1	0.60	0	t	1	Polypedellum	Olinga feredayi	55	0.00	0.03	0.04	0.03
1	1	0.55	0	t	1	Amphipoda spp.	Zelandoperla sp	55	0.03	0.03	0.01	0.03

0	0	0.30	0	cv	1	Hydraena orchymontia	Scirtid sp. (Skinny type)	55	0.00	0.03	0.02	0.03
1	1	0.82	0	t	1	unknown red algal spp.	Eunotia sp.	55	0.03	0.03	0.04	0.03
0	0	0.27	0	t	1	Helicopsyche albescens	Zelandoperla sp	55	0.00	0.03	0.01	0.03
1	1	0.56	0	t	1	Asterionella	Cyclotella sp.	55	0.03	0.03	0.02	0.03
1	0	0.45	0	cv	1	Archichauliodes diversus	Oxyethira albiceps	55	0.01	0.03	0.02	0.03
0	1	0.51	0	t	1	Terrestrial invertebrates	Scirtid sp. (Broad type)	55	0.00	0.03	0.03	0.03
0	0	0.46	0	t	1	Fragilaria spp.	Navicula sp.	55	0.01	0.02	0.02	0.03
1	1	0.64	0	t	1	Nitzschia sp. (small)	Tanyderid sp.	55	0.03	0.03	0.02	0.03
0	0	0.28	0	cv	1	Gomphonema sp. (skinny - tenue??)	Psilachorema bidens	55	0.00	0.03	0.01	0.03
1	1	0.55	0	t	1	Philorheithrus agilis	Hydraena homalaena sp. (type H2)	55	0.03	0.03	0.02	0.03
1	1	0.63	0	t	1	Cocconeis sp.	Nitzschia sp. (large)	55	0.04	0.03	0.01	0.03
1	0	0.32	0	t	1	Archichauliodes diversus	Fragilaria spp.	55	0.01	0.03	0.01	0.02
1	0	0.34	0	cv	1	Archichauliodes diversus	Navicula avenacea	55	0.01	0.03	0.01	0.03
0	0	0.30	0	t	1	Oligochaeta type II	Surirella tenera ??	55	0.01	0.03	0.01	0.03
0	0	0.19	0	t	1	Melosira varians	Ulothrix	55	0.00	0.03	0.00	0.03
1	1	0.74	0	t	1	Eunotia sp.	Epithemia sorex	55	0.04	0.03	0.02	0.03
0	0	0.30	0	cv	1	Macrophyte	Coloburiscus humeralis	55	0.02	0.03	0.00	0.03
1	1	0.56	0	t	1	Pinnularia (small - mesolepta??)	Oligochaeta type weak-long-short	55	0.01	0.03	0.03	0.03
0	0	0.43	0	t	1	Helicopsyche albescens	Tanytarsini	55	0.00	0.03	0.03	0.03
1	1	0.54	0	t	1	Cocconeis sp.	Larsia II	55	0.04	0.03	0.00	0.03
0	1	0.68	0	cv	1	Hydrobiosid spp.	Pycnocentrodes evecta	55	0.02	0.03	0.03	0.03
0	0	0.49	0	t	1	Pinnularia sp. (large)	Navicula cf. radiosa	55	0.03	0.03	0.00	0.03
0	0	0.31	0	t	1	Rhoicosphenia curvata	Maoridiamesea	55	0.01	0.03	0.01	0.03
0	0	0.24	0	t	1	Blue-green algae	Larsia II	55	0.01	0.03	0.00	0.03
0	0	0.28	0	cv	1	Rhopalodia novae zelandicae	Helicopsyche albescens	55	0.01	0.03	0.00	0.03

1	1	0.79	0	t	1	Naonella	Autrosimilium australense	55	0.03	0.03	0.04	0.03
1	1	0.71	0	t	1	Aulacoseira sp.	Surirella (small)	55	0.03	0.03	0.03	0.03
0	0	0.42	0	t	1	Pycnocentria spp.	Macrophyte	55	0.01	0.03	0.02	0.03
1	1	0.56	0	cv	1	Cymbella kappii	Synedra rumpens	55	0.01	0.03	0.04	0.03
1	1	0.64	0	t	1	Macrophyte	Synedra rumpens	55	0.02	0.03	0.04	0.03
0	0	0.43	0	t	1	Amphora sp.	Eukiefiriella	55	0.00	0.03	0.03	0.03
1	0	0.46	0	t	1	Cyclotella sp.	Achnanthes saxonica	55	0.02	0.03	0.02	0.03
1	0	0.49	0	cv	1	Nitzschia sp. (small)	Navicula rhynchocephala	55	0.03	0.03	0.00	0.03
1	1	0.53	0	t	1	Achnanthes lanceoloata	Surirella tenera ??	55	0.04	0.03	0.01	0.03
1	1	0.52	0	t	1	Pirara	Coloburiscus humeralis	55	0.04	0.03	0.00	0.03
1	1	0.51	0	t	1	Eunotia sp. large	Diatoma heimale	55	0.01	0.03	0.03	0.03
0	0	0.42	0	cv	1	Melosira varians	Diatoma heimale	55	0.00	0.03	0.03	0.03
1	1	0.68	0	t	1	Hydraena homalaena sp. (type H2)	Eunotia sp.	55	0.02	0.03	0.04	0.03
1	1	0.72	0	t	1	Unknown orthoclad	Podonomus	55	0.03	0.03	0.03	0.03
1	1	0.75	0	t	1	Nitzschia sp. (small)	Unknown orthoclad	55	0.03	0.03	0.03	0.03
1	1	0.79	0	cv	1	Pirara	Gomphonema cf. olivaceum	55	0.04	0.03	0.03	0.03
1	1	0.63	0	t	1	Philorheithrus agilis	Navicula sp.	55	0.03	0.03	0.02	0.03
0	0	0.36	0	t	1	Hudsonema aliena	Oligochaeta type II	55	0.01	0.03	0.01	0.03
1	1	0.68	0	t	1	Pinnularia sp. (large)	Hydrobiosid spp.	55	0.03	0.03	0.02	0.03
0	0	0.25	0	cv	1	Navicula rhynchocephala	Pinnularia (small - mesolepta??)	55	0.00	0.03	0.01	0.03
0	0	0.19	0	t	1	Helicopsyche albescens	Amphora sp.	55	0.00	0.03	0.00	0.03
1	0	0.27	0	t	1	Nannochorista philpotti	Ulothrix	55	0.01	0.03	0.00	0.03
1	0	0.50	0	t	1	Asterionella sp.	Zelandoperla sp	55	0.03	0.03	0.01	0.03
1	1	0.51	0	cv	1	Cymbella	Podonomus	55	0.01	0.03	0.03	0.03

1	0	0.46	0	t	1	Achnanthes linearis	Ulothrix	55	0.03	0.03	0.00	0.03
1	1	0.64	0	t	1	Microspora sp.	Tanytarsini	55	0.02	0.03	0.03	0.03
1	1	0.70	0	t	1	Gomphonema cf. olivaceum	Diatoma heimale	55	0.03	0.03	0.03	0.03
1	0	0.44	0	cv	1	Navicula rhynchocephal	Naonella	55	0.00	0.03	0.03	0.03
1	1	0.65	0	t	1	Eunotia sp.	Nitzschia sp. (large)	55	0.04	0.03	0.01	0.03
1	1	0.74	0	t	1	Philorheithrus agilis	Nitzschia sp. (small)	55	0.03	0.03	0.03	0.03
0	0	0.30	0	t	1	Blue-green algae	Hudsonema aliena	55	0.01	0.03	0.01	0.03
1	1	0.55	0	cv	1	Pirara	Salmo trutta	55	0.04	0.03	0.00	0.03
0	0	0.41	0	t	1	Helicopsyche albescens	Asterionella sp.	55	0.00	0.03	0.03	0.03
1	1	0.59	0	t	1	Gomphonema subclavatum	Epithemia sorex	55	0.02	0.03	0.02	0.03
0	0	0.38	0	t	1	Hydraena homalaena sp. (type H2)	Zelandoperla sp	55	0.02	0.03	0.01	0.03
1	0	0.49	0	cv	1	Cymbella	Tanytarsini	55	0.01	0.03	0.03	0.03
1	1	0.59	0	t	1	Navicula sp.	Epithemia sorex	55	0.02	0.03	0.02	0.03
1	1	0.58	0	t	1	Archichauliode s diversus	Oligochaeta type weak-long-short	55	0.01	0.03	0.03	0.03
0	0	0.27	0	t	1	Gomphonema sp. (skinny - tenue??)	Rhoicospheni a curvata	55	0.00	0.03	0.01	0.03
1	1	0.55	0	cv	1	Oligochaeta type II	Podonomus	55	0.01	0.03	0.03	0.03
1	0	0.30	0	t	1	Rhopalodia novae zelandicae	Larsia II	55	0.01	0.03	0.00	0.03
1	0	0.43	0	t	1	Blue-green algae	Cymbella cuspidata	55	0.01	0.03	0.02	0.03
1	1	0.73	0	t	1	Pirara	Microspora sp.	55	0.04	0.03	0.02	0.03
1	1	0.82	0	cv	1	Deleatidium spp.	Autrosimiliu m australense	55	0.03	0.03	0.04	0.03
0	0	0.39	0	t	1	Oligochaeta type II	Scirtid sp. (Skinny type)	55	0.01	0.03	0.02	0.03
1	1	0.53	0	t	1	unknown red algal spp.	Pinnularia (small - mesolepta??)	55	0.03	0.03	0.01	0.03
1	1	0.63	0	t	1	Microspora sp.	Diatoma heimale	55	0.02	0.03	0.03	0.03
1	1	0.73	0	cv	1	Navicula sp.	Cocconeis sp.	55	0.02	0.03	0.04	0.03

1	1	0.59	0	t	1	Nitzschia sp. (small)	Nitzschia sp. (large)	55	0.03	0.03	0.01	0.03
1	0	0.34	0	t	1	Meridion circulare	Gomphonema truncatum	55	0.01	0.03	0.01	0.03
1	1	0.62	0	t	1	unknown red algal spp.	Achnanthes saxonica	55	0.03	0.03	0.02	0.03
0	0	0.47	0	cv	1	Helicopsyche albescens	Scirtid sp. (Broad type)	55	0.00	0.03	0.03	0.03
0	1	0.62	0	t	1	Rhopalodia novae zelandicae	Synedra rumpens	55	0.01	0.03	0.04	0.03
1	1	0.76	0	t	1	Cocconeis sp.	Diatoma heimale	55	0.04	0.03	0.03	0.03
0	1	0.64	0	t	1	Fragilaria spp.	Unidentified detritus	55	0.01	0.02	0.04	0.03
0	0	0.36	0	cv	1	Gomphonema truncatum	Scirtid sp. (Skinny type)	55	0.01	0.03	0.02	0.03
1	1	0.56	0	t	1	Scirtid sp. (Broad type)	moss cells	55	0.03	0.03	0.01	0.03
0	0	0.32	0	t	1	Polypedellum	Tanyderid sp.	55	0.00	0.03	0.02	0.03
1	1	0.56	0	t	1	Amphipoda spp.	Nitzschia sp. (large)	55	0.03	0.03	0.01	0.03
1	1	0.69	0	cv	1	Microspora sp.	Cymbella minuta	55	0.02	0.03	0.03	0.03
1	1	0.73	0	t	1	Nitzschia sp. (small)	Naonella	55	0.03	0.03	0.03	0.03
0	0	0.26	0	t	1	Closterium sp.	Oligochaeta type II	55	0.00	0.03	0.01	0.03
1	1	0.68	0	t	1	Achnanthes linearis	Navicula sp.	55	0.03	0.03	0.02	0.03
1	1	0.64	0	cv	1	Stauroneis sp.	Meridion circulare	55	0.04	0.03	0.01	0.03
1	0	0.38	0	t	1	Nitzschia sp. (large)	Psilachorema bidens	55	0.01	0.03	0.01	0.03
1	1	0.53	0	t	1	Achnanthes lanceoloata	Blue-green algae	55	0.04	0.03	0.01	0.03
1	1	0.82	0	t	1	Stauroneis sp.	Synedra rumpens	55	0.04	0.03	0.04	0.03
1	1	0.79	0	cv	1	Achnanthes lanceoloata	Nitzschia sp. (small)	55	0.04	0.03	0.03	0.03
1	0	0.45	0	t	1	Eunotia sp. large	Hydrobiosid spp.	55	0.01	0.03	0.02	0.03
1	0	0.43	0	t	1	Macrophyte	Scirtid sp. (Skinny type)	55	0.02	0.03	0.02	0.03
1	1	0.62	0	t	1	Pycnocentria spp.	Synedra rumpens	55	0.01	0.03	0.04	0.03
0	0	0.37	0	cv	1	Pycnocentria spp.	Hudsonema aliena	55	0.01	0.03	0.01	0.03
0	0	0.38	0	t	1	Melosira varians	Gomphonema subclavatum	55	0.00	0.03	0.02	0.03

0	0	0.35	0	t	1	Oligochaeta type II	Navicula avenacea	55	0.01	0.03	0.01	0.03
0	1	0.54	0	t	1	Rhopalodia novae zelandicae	Naonella	55	0.01	0.03	0.03	0.03
1	1	0.82	0	cv	1	Pirara	Pinnularia sp. (large)	55	0.04	0.03	0.03	0.03
1	1	0.59	0	t	1	Asterionella sp.	Frustulia rhomboides	55	0.03	0.03	0.02	0.03
1	1	0.76	0	t	1	Pinnularia sp. (large)	Navicula cryptocephal a	55	0.03	0.03	0.03	0.03
0	0	0.29	0	t	1	Amphora sp.	Scirtid sp. (Skinny type)	55	0.00	0.03	0.02	0.03
0	0	0.34	0	cv	1	Frustulia rhomboides	Hydraena orchymontia	55	0.02	0.03	0.00	0.03
1	1	0.50	0	t	1	Achnanthes linearis	Surirella tenera ??	55	0.03	0.03	0.01	0.03
1	0	0.36	0	t	1	moss cells	Psilachorema bidens	55	0.01	0.03	0.01	0.03
1	0	0.46	0	t	1	Achnanthes linearis	Amphora sp.	55	0.03	0.03	0.00	0.03
1	1	0.67	0	cv	1	Aulacoseira sp.	Diatoma heimale	55	0.03	0.03	0.03	0.03
1	1	0.51	0	t	1	Cymbella cuspidata	Costachorem a xanthoptera	55	0.02	0.03	0.01	0.03
1	0	0.48	0	t	1	Gomphonema sp. (skinny - tenue??)	Achnanthes linearis	55	0.00	0.03	0.03	0.03
1	1	0.71	0	t	1	Naonella	Surirella (small)	55	0.03	0.03	0.03	0.03
1	0	0.50	0	cv	1	Gomphonema subclavatum	Scirtid sp. (Skinny type)	55	0.02	0.03	0.02	0.03
1	1	0.72	0	t	1	Nitzschia sp. (small)	Cymbella aspera	55	0.03	0.03	0.03	0.03
0	0	0.23	0	t	1	Cymbella	Amphora sp.	55	0.01	0.03	0.00	0.03
1	0	0.49	0	t	1	Closterium sp.	Oligochaeta type weak-long-short	55	0.00	0.03	0.03	0.03
1	1	0.76	0	cv	1	Aulacoseira sp.	Cocconeis sp.	55	0.03	0.03	0.04	0.03
0	0	0.27	0	t	1	Coloburiscus humeralis	Zelandoperla sp	55	0.00	0.03	0.01	0.03
1	1	0.62	0	t	1	Cyclotella sp.	Surirella (small)	55	0.02	0.03	0.03	0.03
0	0	0.29	0	t	1	Meridion circulare	Salmo trutta	55	0.01	0.03	0.00	0.03
0	0	0.21	0	cv	1	Navicula rhynchocephal	Coloburiscus humeralis	55	0.00	0.03	0.00	0.03

0	0	0.27	0	t	1	Polypedellum	Zelandoperla sp	55	0.00	0.03	0.01	0.03
1	1	0.58	0	t	1	Pinnularia sp. (large)	Zelandoperla sp	55	0.03	0.03	0.01	0.03
0	1	0.62	0	t	1	Hydraena homalaena sp. (type H2)	Pycnocentrodes evecta	55	0.02	0.03	0.03	0.03
1	1	0.63	0	cv	1	Eunotia sp.	Zelandoperla sp	55	0.04	0.03	0.01	0.03
1	1	0.62	0	t	1	Archichauliodes diversus	Austroperla cyrene	55	0.01	0.03	0.04	0.03
1	1	0.52	0	t	1	Macrophyte	Gomphonema subclavatum	55	0.02	0.03	0.02	0.03
0	0	0.37	0	t	1	Amphora sp.	Microspora sp.	55	0.00	0.03	0.02	0.03
1	1	0.67	0	cv	1	Navicula cryptocephala	Epithemia sorex	55	0.03	0.03	0.02	0.03
0	0	0.30	0	t	1	Eunotia sp. large	Maoridiamesea	55	0.01	0.03	0.01	0.03
0	0	0.30	0	t	1	Pycnocentria spp.	Salmo trutta	55	0.01	0.03	0.00	0.03
0	0	0.29	0	t	1	Rhopalodia novae zelandicae	Navicula rhynchocephala	55	0.01	0.03	0.00	0.03
1	1	0.78	0	cv	1	Pirara	Eukiefiriella	55	0.04	0.03	0.03	0.03
0	0	0.24	0	t	1	Polyplectropus puerilis	Blue-green algae	55	0.00	0.03	0.01	0.03
1	0	0.33	0	t	1	Frustulia rhomboides	Ulothrix	55	0.02	0.03	0.00	0.03
1	1	0.63	0	t	1	Hydora nitida adult	Hydrobiosid spp.	55	0.03	0.03	0.02	0.03
0	1	0.58	0	cv	1	Nannochorista philpotti	Scirtid sp. (Broad type)	55	0.01	0.03	0.03	0.03
0	0	0.42	0	t	1	Maoridiamesea	Microspora sp.	55	0.01	0.03	0.02	0.03
0	0	0.28	0	t	1	Navicula rhynchocephal	moss cells	55	0.00	0.03	0.01	0.03
1	1	0.54	0	t	1	Eunotia sp. large	Unknown orthoclad	55	0.01	0.03	0.03	0.03
0	0	0.29	0	cv	1	Terrestrial invertebrates	Navicula avenacea	55	0.00	0.03	0.01	0.03
0	0	0.27	0	t	1	Polyplectropus puerilis	Gomphonema truncatum	55	0.00	0.03	0.01	0.03
0	0	0.33	0	t	1	Eunotia sp. large	Gomphonema truncatum	55	0.01	0.03	0.01	0.03
1	1	0.76	0	t	1	Cocconeis sp.	Naonella	55	0.04	0.03	0.03	0.03
1	1	0.61	0	cv	1	Rhopalodia novae zelandicae	unknown red algal spp.	55	0.01	0.03	0.03	0.03

1	1	0.50	0	t	1	Nitzschia sp. (small)	Larsia II	55	0.03	0.03	0.00	0.03
1	1	0.53	0	t	1	Rhoicosphenia curvata	Philorheithrus agilis	55	0.01	0.03	0.03	0.03
1	1	0.79	0	t	1	Pirara	Podonomus	55	0.04	0.03	0.03	0.03
1	1	0.68	0	cv	1	Olinga feredayi	Psilachorema bidens	55	0.04	0.03	0.01	0.03
1	1	0.62	0	t	1	Asterionella sp.	Gomphonema subclavatum	55	0.03	0.03	0.02	0.03
1	1	0.58	0	t	1	Pinnularia sp. (large)	Nannochorista philpotti	55	0.03	0.03	0.01	0.03
1	1	0.55	0	t	1	Gomphonema sp. (skinny - tenue??)	Eunotia sp.	55	0.00	0.03	0.04	0.03
1	0	0.47	0	cv	1	Pinnularia sp. (large)	Amphora sp.	55	0.03	0.03	0.00	0.03
1	1	0.53	0	t	1	Unidentified detritus	Fragilaria vaucheriae	55	0.04	0.03	0.00	0.03
1	1	0.53	0	t	1	Cyclotella sp.	Gomphonema subclavatum	55	0.02	0.03	0.02	0.03
0	1	0.73	0	t	1	Deleatidium spp.	Unknown orthoclad	55	0.03	0.03	0.03	0.03
0	0	0.34	0	cv	1	Gomphonema parvulum	Melosira varians	55	0.02	0.03	0.00	0.03
1	1	0.71	0	t	1	Podonomus	Tanytarsini	55	0.03	0.03	0.03	0.03
0	1	0.64	0	t	1	Hydrobiosid spp.	Unknown orthoclad	55	0.02	0.03	0.03	0.03
1	1	0.54	0	t	1	Cymbella kappii	Cymbella minuta	55	0.01	0.03	0.03	0.03
1	0	0.49	0	cv	1	Hydraena orchymontia	Pycnocentrodes evecta	55	0.00	0.03	0.03	0.03
1	1	0.61	0	t	1	Unidentified detritus	Gomphonema truncatum	55	0.04	0.03	0.01	0.03
1	1	0.76	0	t	1	unknown red algal spp.	Gomphonema cf. olivaceum	55	0.03	0.03	0.03	0.03
0	0	0.49	0	t	1	Maoridiamese	Podonomus	55	0.01	0.03	0.03	0.03
0	0	0.50	0	cv	1	Helicopsyche albescens	Oligochaeta type weak-long-short	55	0.00	0.03	0.03	0.03
0	0	0.26	0	t	1	Oligochaeta type II	Ulothrix	55	0.01	0.03	0.00	0.03
1	1	0.82	0	t	1	Olinga feredayi	Unknown orthoclad	55	0.04	0.03	0.03	0.03
0	0	0.20	0	t	1	Navicula rhynchocephal	Fragilaria vaucheriae	55	0.00	0.03	0.00	0.03
0	0	0.29	0	cv	1	Navicula rhynchocephal	Nitzschia sp. (large)	55	0.00	0.03	0.01	0.03

1	1	0.61	0	t	1	Pycnocentria spp.	unknown red algal spp.	55	0.01	0.03	0.03	0.03
1	1	0.63	0	t	1	Eunotia sp. large	Austroperla cyrene	55	0.01	0.03	0.04	0.03
0	1	0.57	0	t	1	Olinga feredayi	Gomphoneis herculeana	55	0.04	0.03	0.00	0.03
1	1	0.51	0	cv	1	Larsia II	Cymbella minuta	55	0.00	0.03	0.03	0.03
1	1	0.62	0	t	1	Oligochaeta type I	Cymbella aspera	55	0.02	0.03	0.03	0.03
1	0	0.30	0	t	1	Larsia II	Psilachorema bidens	55	0.00	0.03	0.01	0.03
0	1	0.58	0	t	1	Fragilaria spp.	Pinnularia sp. (large)	55	0.01	0.02	0.03	0.03
0	0	0.32	0	cv	1	Helicopsyche albescens	Tanyderid sp.	55	0.00	0.03	0.02	0.03
0	0	0.23	0	t	1	Cymbella kappii	Helicopsyche albescens	55	0.01	0.03	0.00	0.03
0	0	0.31	0	t	1	Blue-green algae	Meridion circulare	55	0.01	0.03	0.01	0.03
0	0	0.29	0	t	1	Hydraena homalaena sp. (type H2)	Gomphoneis herculeana	55	0.02	0.03	0.00	0.03
0	1	0.64	0	cv	1	Hydrobiosid spp.	Podonomus	55	0.02	0.03	0.03	0.03
0	1	0.67	0	t	1	Hudsonema aliena	Aphrophila noevaezeland iae	55	0.01	0.03	0.04	0.03
1	1	0.53	0	t	1	Hydora nitida adult	Nannochorist a philpotti	55	0.03	0.03	0.01	0.03
0	0	0.27	0	t	1	Helicopsyche albescens	Hudsonema aliena	55	0.00	0.03	0.01	0.03
1	1	0.76	0	cv	1	Amphipoda spp.	Pycnocentrod es evecta	55	0.03	0.03	0.03	0.03
0	0	0.30	0	t	1	Achnanthes saxonica	Gomphoneis herculeana	55	0.02	0.03	0.00	0.03
0	0	0.26	0	t	1	Polypedellum	Eunotia sp. large	55	0.00	0.03	0.01	0.03
0	0	0.47	0	t	1	Fragilaria spp.	Epithemia sorex	55	0.01	0.02	0.02	0.03
0	1	0.60	0	cv	1	Olinga feredayi	Terrestrial invertebrates	55	0.04	0.03	0.00	0.03
1	1	0.82	0	t	1	Eunotia sp.	Cymbella minuta	55	0.04	0.03	0.03	0.03
1	1	0.59	0	t	1	Rhoicosphenia curvata	Cymbella minuta	55	0.01	0.03	0.03	0.03
1	0	0.36	0	t	1	Navicula sp.	Melosira varians	55	0.02	0.03	0.00	0.03
1	1	0.57	0	cv	1	Rhoicosphenia curvata	Gomphonem a cf. olivaceum	55	0.01	0.03	0.03	0.03

1	1	0.79	0	t	1	Stauroneis sp.	Gomphonema cf. olivaceum	55	0.04	0.03	0.03	0.03
0	0	0.19	0	t	1	Polypedellum	Helicopsyche albescens	55	0.00	0.03	0.00	0.03
0	0	0.20	0	t	1	Closterium sp.	Polyplectropus puerilis	55	0.00	0.03	0.00	0.03
0	0	0.31	0	cv	1	Hydraena homalaena sp. (type H2)	Navicula cf. radiosa	55	0.02	0.03	0.00	0.03
0	0	0.21	0	t	1	Larsia II	Hydora nitida larvae	55	0.00	0.03	0.00	0.03
1	0	0.36	0	t	1	Fragilaria spp.	Costachorema xanthoptera	55	0.01	0.02	0.01	0.03
1	0	0.49	0	t	1	Oligochaeta type I	Costachorema xanthoptera	55	0.02	0.03	0.01	0.03
1	1	0.59	0	cv	1	Hydraena homalaena sp. (type H2)	Surirella (small)	55	0.02	0.03	0.03	0.03
0	1	0.59	0	t	1	Terrestrial invertebrates	Eunotia sp.	55	0.00	0.03	0.04	0.03
1	1	0.58	0	t	1	Larsia II	Unidentified detritus	55	0.00	0.03	0.04	0.03
0	0	0.28	0	t	1	Salmo trutta	Navicula avenacea	55	0.00	0.03	0.01	0.03
1	1	0.70	0	cv	1	Navicula cryptocephala	Diatoma heimale	55	0.03	0.03	0.03	0.03
1	1	0.53	0	t	1	Pycnocentria spp.	Diatoma heimale	55	0.01	0.03	0.03	0.03
1	1	0.72	0	t	1	Asterionella sp.	Cymbella minuta	55	0.03	0.03	0.03	0.03
1	0	0.43	0	t	1	Philorheithrus agilis	Salmo trutta	55	0.03	0.03	0.00	0.03
1	1	0.63	0	cv	1	Achnanthes saxonica	Pycnocentrodes evecta	55	0.02	0.03	0.03	0.03
0	0	0.34	0	t	1	Eunotia sp. large	Navicula avenacea	55	0.01	0.03	0.01	0.03
0	0	0.25	0	t	1	Gomphonema sp. (skinny - tenue??)	Gomphonema truncatum	55	0.00	0.03	0.01	0.03
1	1	0.70	0	t	1	Pycnocentria spp.	Olinga feredayi	55	0.01	0.03	0.04	0.03
1	0	0.50	0	cv	1	Pinnularia sp. (large)	Salmo trutta	55	0.03	0.03	0.00	0.03
0	0	0.22	0	t	1	Blue-green algae	Hydora nitida larvae	55	0.01	0.03	0.00	0.03
0	1	0.76	0	t	1	Hydrobiosid spp.	Aphrophila noevaezelandiae	55	0.02	0.03	0.04	0.03

1	0	0.48	0	t	1	Achnanthes lanceoloata	Ulothrix	55	0.04	0.03	0.00	0.03
1	0	0.49	0	cv	1	Cymbella cuspidata	Zelandoperla sp	55	0.02	0.03	0.01	0.03
0	0	0.28	0	t	1	Archichauliodes diversus	Surirella tenera ??	55	0.01	0.03	0.01	0.03
1	1	0.65	0	t	1	Microspora sp.	Eukiefiriella	55	0.02	0.03	0.03	0.03
0	0	0.33	0	t	1	Surirella tenera ??	Scirtid sp. (Skinny type)	55	0.01	0.03	0.02	0.03
1	1	0.72	0	cv	1	Surirella	Eukiefiriella	55	0.03	0.03	0.03	0.03
1	0	0.41	0	t	1	Rhopalodia novae zelandicae	Achnanthes saxonica	55	0.01	0.03	0.02	0.03
1	1	0.57	0	t	1	Hydrobiosid spp.	Microspora sp.	55	0.02	0.03	0.02	0.03
1	1	0.61	0	t	1	Cocconeis sp.	Navicula avenacea	55	0.04	0.03	0.01	0.03
1	1	0.82	0	cv	1	Pinnularia sp. (large)	Unidentified detritus	55	0.03	0.03	0.04	0.03
1	1	0.69	0	t	1	Cymbella cuspidata	Navicula cryptocephala	55	0.02	0.03	0.03	0.03
0	0	0.23	0	t	1	Cymbella	Polypedellum	55	0.01	0.03	0.00	0.03
1	1	0.78	0	t	1	Synedra rumpens	Scirtid sp. (Broad type)	55	0.04	0.03	0.03	0.03
1	0	0.43	0	cv	1	Cymbella	Navicula sp.	55	0.01	0.03	0.02	0.03
1	0	0.46	0	t	1	Aulacoseira sp.	Cymbella kappii	55	0.03	0.03	0.01	0.03
1	0	0.49	0	t	1	Blue-green algae	Podonomus	55	0.01	0.03	0.03	0.03
0	1	0.50	0	t	1	Potamopyrgus antipodarum	Synedra rumpens	55	0.00	0.03	0.04	0.03
1	0	0.46	0	cv	1	Achnanthes linearis	Fragilaria vaucheriae	55	0.03	0.03	0.00	0.03
0	1	0.62	0	t	1	Fragilaria spp.	Cocconeis sp.	55	0.01	0.02	0.04	0.03
1	0	0.47	0	t	1	unknown red algal spp.	Hydraena orchymontia	55	0.03	0.03	0.00	0.03
1	1	0.62	0	t	1	Cymbella aspera	Microspora sp.	55	0.03	0.03	0.02	0.03
0	0	0.28	0	cv	1	Melosira varians	Nitzschia sp. (large)	55	0.00	0.03	0.01	0.03
1	1	0.51	0	t	1	Eunotia sp. large	Philorheithrus agilis	55	0.01	0.03	0.03	0.03
0	0	0.28	0	t	1	Closterium sp.	Rhopalodia novae zelandicae	55	0.00	0.03	0.01	0.03
1	1	0.78	0	t	1	Naonella	Eunotia sp.	55	0.03	0.03	0.04	0.03
1	1	0.52	0	cv	1	Polyplectropus puerilis	Pinnularia sp. (large)	55	0.00	0.03	0.03	0.03

1	1	0.61	0	t	1	Pinnularia sp. (large)	Hydraena homalaena sp. (type H2)	55	0.03	0.03	0.02	0.03
1	1	0.65	0	t	1	Philorheithrus agilis	Cymbella cuspidata	55	0.03	0.03	0.02	0.03
1	0	0.43	0	t	1	Rhopalodia novae zelandicae	Cyclotella sp.	55	0.01	0.03	0.02	0.03
0	0	0.29	0	cv	1	Pycnocentria spp.	Navicula rhynchocephala	55	0.01	0.03	0.00	0.03
1	1	0.55	0	t	1	Amphora sp.	Unidentified detritus	55	0.00	0.03	0.04	0.03
0	0	0.29	0	t	1	Ulothrix	Scirtid sp. (Skinny type)	55	0.00	0.03	0.02	0.03
1	0	0.35	0	t	1	Tanyderid sp.	Surirella tenera ??	55	0.02	0.03	0.01	0.03
1	0	0.49	0	cv	1	Cymbella aspera	Navicula avenacea	55	0.03	0.03	0.01	0.03
1	1	0.57	0	t	1	Stauroneis sp.	Maoridiamesea	55	0.04	0.03	0.01	0.03
1	1	0.77	0	t	1	Scirtid sp. (Broad type)	Pycnocentrodes evecta	55	0.03	0.03	0.03	0.03
0	1	0.57	0	t	1	Olinga feredayi	Melosira varians	55	0.04	0.03	0.00	0.03
1	1	0.55	0	cv	1	Polyplectropus puerilis	Cocconeis sp.	55	0.00	0.03	0.04	0.03
1	1	0.79	0	t	1	Olinga feredayi	Cymbella aspera	55	0.04	0.03	0.03	0.03
0	0	0.33	0	t	1	Pinnularia (small -	Nitzschia sp. (large)	55	0.01	0.03	0.01	0.03
0	0	0.24	0	t	1	Larsia II	Surirella tenera ??	55	0.00	0.03	0.01	0.03
0	0	0.45	0	cv	1	Naonella	Surirella tenera ??	55	0.03	0.03	0.01	0.03
1	0	0.46	0	t	1	Blue-green algae	Philorheithrus agilis	55	0.01	0.03	0.03	0.03
0	0	0.38	0	t	1	Polypedellum	Gomphonema subclavatum	55	0.00	0.03	0.02	0.03
1	1	0.51	0	t	1	Austroperla cyrene	Potamopyrgus antipodarum	55	0.04	0.03	0.00	0.03
0	0	0.19	0	cv	1	Closterium sp.	Gomphonema sp. (skinny - tenue??)	55	0.00	0.03	0.00	0.03
1	0	0.47	0	t	1	Eunotia sp. large	Epithemia sorex	55	0.01	0.03	0.02	0.03
0	0	0.42	0	t	1	Hydora nitida adult	Potamopyrgus antipodarum	55	0.03	0.03	0.00	0.03

1	1	0.68	0	t	1	Oxyethira albiceps	Cymbella minuta	55	0.02	0.03	0.03	0.03
1	0	0.49	0	cv	1	Closterium sp.	unknown red algal spp.	55	0.00	0.03	0.03	0.03
1	1	0.57	0	t	1	Unidentified detritus	Surirella tenera ??	55	0.04	0.03	0.01	0.03
0	1	0.57	0	t	1	Fragilaria spp.	Cymbella minuta	55	0.01	0.02	0.03	0.03
1	1	0.60	0	t	1	Eunotia sp. large	Synedra rumpens	55	0.01	0.03	0.04	0.03
1	1	0.51	0	cv	1	Zelandoperla sp	Diatoma heimale	55	0.01	0.03	0.03	0.03
1	0	0.44	0	t	1	Salmo trutta	Diatoma heimale	55	0.00	0.03	0.03	0.03
1	0	0.48	0	t	1	Synedra rumpens	Hydora nitida larvae	55	0.04	0.03	0.00	0.03
1	1	0.67	0	t	1	Pycnocentria spp.	Eunotia sp.	55	0.01	0.03	0.04	0.03
1	0	0.42	0	cv	1	Closterium sp.	Naonella	55	0.00	0.03	0.03	0.03
0	0	0.27	0	t	1	Rhoicosphenia curvata	Melosira varians	55	0.01	0.03	0.00	0.03
0	1	0.51	0	t	1	Austroperla cyrene	Hydora nitida larvae	55	0.04	0.03	0.00	0.03
0	1	0.58	0	t	1	Deleatidium spp.	Hydraena homalaena sp. (type H2)	55	0.03	0.03	0.02	0.03
1	1	0.70	0	cv	1	Oligochaeta type I	unknown red algal spp.	55	0.02	0.03	0.03	0.03
0	0	0.50	0	t	1	Hydora nitida larvae	Oligochaeta type weak-long-short	55	0.00	0.03	0.03	0.03
0	0	0.36	0	t	1	Amphora sp.	Synedra ulna	55	0.00	0.03	0.02	0.03
0	0	0.28	0	t	1	Amphora sp.	Nitzschia sp. (large)	55	0.00	0.03	0.01	0.03
1	1	0.55	0	cv	1	Eunotia sp. large	Deleatidium spp.	55	0.01	0.03	0.03	0.03
0	0	0.22	0	t	1	Polyplectropus puerilis	Navicula rhynchoceph ala	55	0.00	0.03	0.00	0.03
1	0	0.41	0	t	1	Tanyderid sp.	moss cells	55	0.02	0.03	0.01	0.03
1	1	0.51	0	t	1	Naonella	moss cells	55	0.03	0.03	0.01	0.03
1	1	0.57	0	cv	1	Surirella (small)	Costachorem a xanthoptera	55	0.03	0.03	0.01	0.03
0	0	0.37	0	t	1	Gomphonema parvulum	Terrestrial invertebrates	55	0.02	0.03	0.00	0.03
1	0	0.40	0	t	1	Meridion circulare	Hydraena homalaena sp. (type H2)	55	0.01	0.03	0.02	0.03
0	0	0.19	0	t	1	Helicopsyche albescens	Melosira varians	55	0.00	0.03	0.00	0.03

1	0	0.45	0	cv	1	Navicula rhynchocephal	Eukiefiriella	55	0.00	0.03	0.03	0.03
1	1	0.56	0	t	1	Pycnocentria spp.	Podonomus	55	0.01	0.03	0.03	0.03
1	1	0.59	0	t	1	Blue-green algae	Eunotia sp.	55	0.01	0.03	0.04	0.03
1	0	0.48	0	t	1	Meridion circulare	Oxyethira albiceps	55	0.01	0.03	0.02	0.03
1	0	0.41	0	cv	1	Philorheithrus agilis	Potamopyrgu s antipodarum	55	0.03	0.03	0.00	0.03
0	1	0.69	0	t	1	Hydraena homalaena sp. (type H2)	Autrosimiliu m australense	55	0.02	0.03	0.04	0.03
1	0	0.46	0	t	1	Asterionella sp.	Pinnularia (small - mesolepta??)	55	0.03	0.03	0.01	0.03
0	0	0.27	0	t	1	Nannochorista philpotti	Gomphoneis herculeana	55	0.01	0.03	0.00	0.03
1	0	0.37	0	cv	1	Gomphonema sp. (skinny - tenue??)	Epithemia sorex	55	0.00	0.03	0.02	0.03
0	0	0.46	0	t	1	Coloburiscus humeralis	Surirella (small)	55	0.00	0.03	0.03	0.03
1	1	0.52	0	t	1	Asterionella sp.	Costachorem a xanthoptera	55	0.03	0.03	0.01	0.03
1	0	0.43	0	t	1	Tanyderid sp.	Nitzschia sp. (large)	55	0.02	0.03	0.01	0.03
1	1	0.68	0	cv	1	unknown red algal spp.	Oxyethira albiceps	55	0.03	0.03	0.02	0.03
1	1	0.60	0	t	1	Cymbella cuspidata	Oxyethira albiceps	55	0.02	0.03	0.02	0.03
1	1	0.73	0	t	1	Pinnularia sp. (large)	Diatoma heimale	55	0.03	0.03	0.03	0.03
1	0	0.49	0	t	1	Surirella (small)	Surirella tenera ??	55	0.03	0.03	0.01	0.03
1	0	0.46	0	cv	1	Cymbella kappii	Cymbella aspera	55	0.01	0.03	0.03	0.03
1	0	0.44	0	t	1	Microspora sp.	Gomphonem a truncatum	55	0.02	0.03	0.01	0.03
1	0	0.36	0	t	1	moss cells	Costachorem a xanthoptera	55	0.01	0.03	0.01	0.03
0	0	0.36	0	t	1	Fragilaria spp.	Meridion circulare	55	0.01	0.02	0.01	0.03
1	0	0.46	0	cv	1	Hydrobiosid spp.	Meridion circulare	55	0.02	0.03	0.01	0.03
0	0	0.35	0	t	1	Pycnocentria spp.	Archichauliod es diversus	55	0.01	0.03	0.01	0.03

1	1	0.80	0	t	1	Hydora nitida adult	Autrosimilium australense	55	0.03	0.03	0.04	0.03
0	0	0.43	0	t	1	Polypedellum	Hydora nitida adult	55	0.00	0.03	0.03	0.03
1	0	0.44	0	cv	1	Aulacoseira sp.	Blue-green algae	55	0.03	0.03	0.01	0.03
1	0	0.47	0	t	1	Navicula avenacea	Epithemia sorex	55	0.01	0.03	0.02	0.03
1	1	0.52	0	t	1	Achnanthes saxonica	Epithemia sorex	55	0.02	0.03	0.02	0.03
1	1	0.69	0	t	1	Cymbella minuta	Epithemia sorex	55	0.03	0.03	0.02	0.03
1	1	0.82	0	cv	1	Nitzschia sp. (small)	Eunotia sp.	55	0.03	0.03	0.04	0.03
0	0	0.29	0	t	1	Hydraena homalaena sp. (type H2)	Ulothrix	55	0.02	0.03	0.00	0.03
0	1	0.51	0	t	1	Hydrobiosid spp.	Tanyderid sp.	55	0.02	0.03	0.02	0.03
0	1	0.58	0	t	1	Rhopalodia novae zelandicae	Amphipoda spp.	55	0.01	0.03	0.03	0.03
0	0	0.46	0	cv	1	Polypedellum	Amphipoda spp.	55	0.00	0.03	0.03	0.03
0	0	0.28	0	t	1	Rhopalodia novae zelandicae	Coloburiscus humeralis	55	0.01	0.03	0.00	0.03
0	1	0.69	0	t	1	Tanyderid sp.	Cocconeis sp.	55	0.02	0.03	0.04	0.03
0	0	0.47	0	t	1	Terrestrial invertebrates	Tanytarsini	55	0.00	0.03	0.03	0.03
1	1	0.67	0	cv	1	Cymbella cuspidata	Tanytarsini	55	0.02	0.03	0.03	0.03
1	1	0.71	0	t	1	Achnanthes linearis	Cymbella aspera	55	0.03	0.03	0.03	0.03
1	0	0.47	0	t	1	Oxyethira albiceps	Psilachorema bidens	55	0.02	0.03	0.01	0.03
0	0	0.41	0	t	1	Naonella	Hydora nitida larvae	55	0.03	0.03	0.00	0.03
0	1	0.58	0	cv	1	Tanyderid sp.	Diatoma heimale	55	0.02	0.03	0.03	0.03
0	0	0.25	0	t	1	Gomphonema truncatum	Gomphoneis herculeana	55	0.01	0.03	0.00	0.03
1	0	0.38	0	t	1	Gomphonema parvulum	Surirella tenera ??	55	0.02	0.03	0.01	0.03
1	0	0.41	0	t	1	Cymbella aspera	Salmo trutta	55	0.03	0.03	0.00	0.03
0	1	0.53	0	cv	1	Unidentified detritus	Gomphoneis herculeana	55	0.04	0.03	0.00	0.03

0	0	0.36	0	t	1	Helicopsyche albescens	Hydrobiosid spp.	55	0.00	0.03	0.02	0.03
1	1	0.65	0	t	1	Oxyethira albiceps	Podonomus	55	0.02	0.03	0.03	0.03
0	0	0.42	0	t	1	Hydora nitida adult	Coloburiscus humeralis	55	0.03	0.03	0.00	0.03
0	0	0.31	0	cv	1	Maoridiamese a	Hudsonema aliena	55	0.01	0.03	0.01	0.03
1	1	0.71	0	t	1	Philorheithrus agilis	Surirella (small)	55	0.03	0.03	0.03	0.03
1	1	0.78	0	t	1	unknown red algal spp.	Pycnocentrod es evecta	55	0.03	0.03	0.03	0.03
1	1	0.82	0	t	1	Olinga feredayi	Gomphonem a cf. olivaceum	55	0.04	0.03	0.03	0.03
0	0	0.25	0	cv	1	Archichauliode s diversus	Helicopsyche albescens	55	0.01	0.03	0.00	0.03
0	0	0.29	0	t	1	Closterium sp.	Scirtid sp. (Skinny type)	55	0.00	0.03	0.02	0.03
0	0	0.29	0	t	1	Polyplectropus puerilis	Oligochaeta type II	55	0.00	0.03	0.01	0.03
1	1	0.67	0	t	1	Olinga feredayi	Oligochaeta type II	55	0.04	0.03	0.01	0.03
1	0	0.36	0	cv	1	Closterium sp.	Synedra ulna	55	0.00	0.03	0.02	0.03
1	0	0.38	0	t	1	Larsia II	Frustulia rhomboides	55	0.00	0.03	0.02	0.03
0	0	0.30	0	t	1	Pycnocentria spp.	Terrestrial invertebrates	55	0.01	0.03	0.00	0.03
0	0	0.49	0	t	1	Achnanthes lanceoloata	Gomphoneis herculeana	55	0.04	0.03	0.00	0.03
1	1	0.77	0	cv	1	Pinnularia sp. (large)	Scirtid sp. (Broad type)	55	0.03	0.03	0.03	0.03
1	1	0.83	0	t	1	Olinga feredayi	Surirella (small)	55	0.04	0.03	0.03	0.03
1	1	0.58	0	t	1	Navicula cryptocephala	Scirtid sp. (Skinny type)	55	0.03	0.03	0.02	0.03
0	0	0.26	0	t	1	Gomphonema sp. (skinny - tenue??)	Navicula avenacea	55	0.00	0.03	0.01	0.03
1	1	0.52	0	cv	1	Surirella tenera ??	Cymbella minuta	55	0.01	0.03	0.03	0.03
1	1	0.65	0	t	1	Cymbella cuspidata	Diatoma heimale	55	0.02	0.03	0.03	0.03
1	0	0.37	0	t	1	Closterium sp.	Microspora sp.	55	0.00	0.03	0.02	0.03
1	1	0.62	0	t	1	Hydora nitida adult	Frustulia rhomboides	55	0.03	0.03	0.02	0.03
0	1	0.63	0	cv	1	Hydraena homalaena sp. (type H2)	Oligochaeta type weak-long-short	55	0.02	0.03	0.03	0.03

1	1	0.67	0	t	1	Cymbella cuspidata	Eukiefiriella	55	0.02	0.03	0.03	0.03
1	0	0.37	0	t	1	Navicula avenacea	Costachorema xanthoptera	55	0.01	0.03	0.01	0.03
1	1	0.64	0	t	1	Pinnularia sp. (large)	Tanyderid sp.	55	0.03	0.03	0.02	0.03
0	0	0.31	0	cv	1	Cymbella kappii	Navicula avenacea	55	0.01	0.03	0.01	0.03
1	1	0.84	0	t	1	Olinga feredayi	Pycnocentrodes evecta	55	0.04	0.03	0.03	0.03
1	1	0.69	0	t	1	Synedra rumpens	Tabellaria flocculosa	55	0.04	0.03	0.02	0.03
0	0	0.28	0	t	1	Polyplectropus puerilis	moss cells	55	0.00	0.03	0.01	0.03
1	1	0.74	0	cv	1	Surirella (small)	Navicula cryptocephala	55	0.03	0.03	0.03	0.03
0	0	0.27	0	t	1	Helicopsyche albescens	Oligochaeta type II	55	0.00	0.03	0.01	0.03
0	0	0.21	0	t	1	Larsia II	Gomphoneis herculeana	55	0.00	0.03	0.00	0.03
0	0	0.45	0	t	1	Melosira varians	Unknown orthoclad	55	0.00	0.03	0.03	0.03
0	0	0.32	0	cv	1	Terrestrial invertebrates	Scirtid sp. (Skinny type)	55	0.00	0.03	0.02	0.03
1	1	0.52	0	t	1	Pinnularia (small - mesolepta??)	Gomphonema cf. olivaceum	55	0.01	0.03	0.03	0.03
0	0	0.40	0	t	1	Pycnocentria spp.	Hydraena homalaena sp. (type H2)	55	0.01	0.03	0.02	0.03
1	0	0.49	0	t	1	Synedra ulna	Scirtid sp. (Skinny type)	55	0.02	0.03	0.02	0.03
1	1	0.57	0	cv	1	Blue-green algae	Cocconeis sp.	55	0.01	0.03	0.04	0.03
1	1	0.77	0	t	1	Pirara	Naonella	55	0.04	0.03	0.03	0.03
0	0	0.50	0	t	1	Potamopyrgus antipodarum	Oligochaeta type weak-long-short	55	0.00	0.03	0.03	0.03
1	1	0.64	0	t	1	Eunotia sp. large	Eunotia sp.	55	0.01	0.03	0.04	0.03
0	0	0.20	0	cv	1	Amphora sp.	Navicula cf. radiosa	55	0.00	0.03	0.00	0.03
1	0	0.37	0	t	1	Polyplectropus puerilis	Gomphonema parvulum	55	0.00	0.03	0.02	0.03
0	0	0.27	0	t	1	Hydraena orchymontia	Zelandoperla sp	55	0.00	0.03	0.01	0.03
0	0	0.42	0	t	1	Hudsonema aliena	Cyclotella sp.	55	0.01	0.03	0.02	0.03

1	1	0.77	0	cv	1	Philorheithrus agilis	Stauroneis sp.	55	0.03	0.03	0.04	0.03
0	0	0.44	0	t	1	Deleatidium spp.	Melosira varians	55	0.03	0.03	0.00	0.03
1	0	0.19	0	t	1	Closterium sp.	Gomphoneis herculeana	55	0.00	0.03	0.00	0.03
1	0	0.43	0	t	1	Gomphonema sp. (skinny - tenue??)	Tanytarsini	55	0.00	0.03	0.03	0.03
1	1	0.73	0	cv	1	Pirara	Navicula sp.	55	0.04	0.03	0.02	0.03
0	0	0.22	0	t	1	Blue-green algae	Helicopsyche albescens	55	0.01	0.03	0.00	0.03
1	1	0.54	1	t	1	Oniscogaster sp.	Closterium sp.	56	0.04	0.03	0.01	0.03
1	1	0.74	1	t	1	Oniscogaster sp.	Achnanthes lanceoloata	56	0.04	0.03	0.03	0.03
1	1	0.79	1	cv	1	Oniscogaster sp.	Aulacoseira sp.	56	0.04	0.03	0.03	0.03
1	1	0.68	1	t	1	Oniscogaster sp.	Rhoicospheni a curvata	56	0.04	0.03	0.02	0.03
1	1	0.51	1	t	1	Oniscogaster sp.	Hydrobiosis parumbripen nis	56	0.04	0.03	0.00	0.03
1	1	0.63	1	t	1	Oniscogaster sp.	Polyplectropu s puerilis	56	0.04	0.03	0.01	0.03
1	1	0.78	1	cv	1	Oniscogaster sp.	Fragilaria vaucheriae	56	0.04	0.03	0.03	0.03
1	1	0.63	1	t	1	Oniscogaster sp.	Achnanthes linearis	56	0.04	0.03	0.01	0.03
1	1	0.68	1	t	1	Oniscogaster sp.	Cymbella aspera	56	0.04	0.03	0.02	0.03
1	1	0.75	1	t	1	Oniscogaster sp.	Pinnularia sp. (large)	56	0.04	0.03	0.03	0.03
1	1	0.54	1	cv	1	Oniscogaster sp.	Meridion circulare	56	0.04	0.03	0.01	0.03
1	1	0.74	1	t	1	Oniscogaster sp.	Navicula rhynchoceph ala	56	0.04	0.03	0.03	0.03
1	1	0.52	1	t	1	Oniscogaster sp.	Gomphonem a parvulum	56	0.04	0.03	0.00	0.03
1	1	0.65	1	t	1	Oniscogaster sp.	Pinnularia (small - mesolepta??)	56	0.04	0.03	0.02	0.03
1	1	0.50	1	cv	1	Oniscogaster sp.	Cyclotella sp.	56	0.04	0.03	0.00	0.03
1	1	0.69	1	t	1	Oniscogaster sp.	Nitzschia sp. (small)	56	0.04	0.03	0.02	0.03
1	1	0.89	1	t	1	Oniscogaster sp.	Frustulia rhomboides	56	0.04	0.03	0.05	0.03
1	1	0.50	1	t	1	Oniscogaster sp.	Microspora sp.	56	0.04	0.03	0.00	0.03

1	1	0.65	1	cv	1	Oniscogaster sp.	Unidentified detritus	56	0.04	0.03	0.02	0.03
1	1	0.52	1	t	1	Oniscogaster sp.	Achnanthes saxonica	56	0.04	0.03	0.00	0.03
1	1	0.68	1	t	1	Oniscogaster sp.	Gomphonema subclavatum	56	0.04	0.03	0.02	0.03
1	1	0.81	1	t	1	Oniscogaster sp.	Gomphonema cf. olivaceum	56	0.04	0.03	0.03	0.03
1	1	0.59	1	cv	1	Oniscogaster sp.	Eunotia sp.	56	0.04	0.03	0.01	0.03
1	1	0.76	1	t	1	Oniscogaster sp.	Nitzschia sp. (large)	56	0.04	0.03	0.03	0.03
1	1	0.78	1	t	1	Oniscogaster sp.	Cymbella kappii	56	0.04	0.03	0.03	0.03
1	1	0.85	1	t	1	Oniscogaster sp.	Cymbella minuta	56	0.04	0.03	0.04	0.03
1	1	0.69	1	cv	1	Oniscogaster sp.	Gomphonema truncatum	56	0.04	0.03	0.02	0.03
1	0	0.50	1	t	1	Oniscogaster sp.	Audouinella	56	0.04	0.03	0.00	0.03
1	1	0.79	1	t	1	Oniscogaster sp.	Navicula avenacea	56	0.04	0.03	0.03	0.03
1	1	0.77	1	t	1	Oniscogaster sp.	Diatoma heimale	56	0.04	0.03	0.03	0.03
1	1	0.63	1	cv	1	Cricotopus I	Aulacoseira sp.	56	0.02	0.03	0.03	0.03
1	0	0.34	1	t	1	Cricotopus I	moss cells	56	0.02	0.03	0.00	0.03
1	0	0.37	1	t	1	Cricotopus I	Tabellaria flocculosa	56	0.02	0.03	0.01	0.03
1	1	0.54	1	t	1	Cricotopus I	Macrophyte	56	0.02	0.03	0.02	0.03
1	0	0.36	1	cv	1	Cricotopus I	Meridion circulare	56	0.02	0.03	0.01	0.03
0	0	0.32	1	t	1	Cricotopus I	Surirella tenera ??	56	0.02	0.03	0.00	0.03
1	1	0.56	1	t	1	Cricotopus I	Cocconeis sp.	56	0.02	0.03	0.02	0.03
1	0	0.32	1	t	1	Cricotopus I	Microspora sp.	56	0.02	0.03	0.00	0.03
1	0	0.46	1	cv	1	Cricotopus I	Unidentified detritus	56	0.02	0.03	0.02	0.03
1	0	0.40	1	t	1	Cricotopus I	Eunotia sp.	56	0.02	0.03	0.01	0.03
0	0	0.46	1	t	1	Cricotopus I	Epithemia sorex	56	0.02	0.03	0.02	0.03
1	1	0.61	1	t	1	Cricotopus I	Diatoma heimale	56	0.02	0.03	0.03	0.03
1	1	0.69	1	cv	1	Hudsonema amabilis	Achnanthes lanceoloata	56	0.03	0.03	0.03	0.03
1	1	0.74	1	t	1	Hudsonema amabilis	Aulacoseira sp.	56	0.03	0.03	0.03	0.03

1	1	0.58	1	t	1	Hudsonema amabilis	Achnanthes linearis	56	0.03	0.03	0.01	0.03
1	0	0.47	1	t	1	Hudsonema amabilis	moss cells	56	0.03	0.03	0.00	0.03
1	0	0.44	1	cv	1	Hudsonema amabilis	Fragilaria spp.	56	0.03	0.03	0.00	0.03
1	0	0.50	1	t	1	Hudsonema amabilis	Tabellaria flocculosa	56	0.03	0.03	0.01	0.03
1	1	0.63	1	t	1	Hudsonema amabilis	Cymbella aspera	56	0.03	0.03	0.02	0.03
1	1	0.70	1	t	1	Hudsonema amabilis	Pinnularia sp. (large)	56	0.03	0.03	0.03	0.03
1	1	0.66	1	cv	1	Hudsonema amabilis	Macrophyte	56	0.03	0.03	0.02	0.03
1	0	0.48	1	t	1	Hudsonema amabilis	Meridion circulare	56	0.03	0.03	0.01	0.03
1	1	0.69	1	t	1	Hudsonema amabilis	Navicula rhynchocephala	56	0.03	0.03	0.03	0.03
1	0	0.46	1	t	1	Hudsonema amabilis	Gomphonema parvulum	56	0.03	0.03	0.00	0.03
1	0	0.45	1	cv	1	Hudsonema amabilis	Salmo trutta	56	0.03	0.03	0.00	0.03
1	1	0.64	1	t	1	Hudsonema amabilis	Nitzschia sp. (small)	56	0.03	0.03	0.02	0.03
1	1	0.87	1	t	1	Hudsonema amabilis	Frustulia rhomboides	56	0.03	0.03	0.05	0.03
1	1	0.63	1	t	1	Hudsonema amabilis	Gomphonema subclavatum	56	0.03	0.03	0.02	0.03
1	1	0.77	1	cv	1	Hudsonema amabilis	Gomphonema cf. olivaceum	56	0.03	0.03	0.03	0.03
1	1	0.53	1	t	1	Hudsonema amabilis	Eunotia sp.	56	0.03	0.03	0.01	0.03
1	1	0.63	1	t	1	Hudsonema amabilis	Navicula cryptocephala	56	0.03	0.03	0.02	0.03
1	0	0.44	1	t	1	Hudsonema amabilis	Audouinella	56	0.03	0.03	0.00	0.03
1	1	0.69	1	cv	1	Hudsonema amabilis	Oedogonium	56	0.03	0.03	0.03	0.03
1	1	0.74	1	t	1	Hudsonema amabilis	Navicula avenacea	56	0.03	0.03	0.03	0.03
1	1	0.59	1	t	1	Hudsonema amabilis	Epithemia sorex	56	0.03	0.03	0.02	0.03
1	1	0.73	1	t	1	Hudsonema amabilis	Diatoma heimale	56	0.03	0.03	0.03	0.03
1	0	0.38	1	cv	1	Closterium sp.	Coloburiscus humeralis	56	0.01	0.03	0.02	0.03

0	0	0.29	1	t	1	Closterium sp.	Deleatidium spp.	56	0.01	0.03	0.01	0.03
1	1	0.63	1	t	1	Achnanthes lanceoloata	Zealolessica cheira	56	0.03	0.03	0.02	0.03
1	1	0.71	1	t	1	Achnanthes lanceoloata	Maoridiemes ea	56	0.03	0.03	0.03	0.03
1	1	0.65	1	cv	1	Achnanthes lanceoloata	Polypedellum	56	0.03	0.03	0.03	0.03
1	0	0.43	1	t	1	Achnanthes lanceoloata	Cricotopus II	56	0.03	0.03	0.01	0.03
1	1	0.65	1	t	1	Achnanthes lanceoloata	Ceratopogoni d sp.	56	0.03	0.03	0.03	0.03
1	0	0.48	1	t	1	Achnanthes lanceoloata	Orchymontia sp.	56	0.03	0.03	0.01	0.03
1	1	0.59	1	cv	1	Achnanthes lanceoloata	Oxyethira albiceps	56	0.03	0.03	0.02	0.03
1	1	0.64	1	t	1	Achnanthes lanceoloata	Oeconesus sp.	56	0.03	0.03	0.02	0.03
1	1	0.65	1	t	1	Achnanthes lanceoloata	Austroclima jollyae	56	0.03	0.03	0.03	0.03
1	0	0.48	1	t	1	Achnanthes lanceoloata	Deleatidium spp.	56	0.03	0.03	0.01	0.03
1	1	0.72	1	cv	1	Achnanthes lanceoloata	Paranephrop s zealandicus	56	0.03	0.03	0.03	0.03
1	1	0.63	1	t	1	Achnanthes lanceoloata	Austrosimuliu m australense	56	0.03	0.03	0.02	0.03
1	0	0.50	1	t	1	Achnanthes lanceoloata	Pycnocentria sp.	56	0.03	0.03	0.01	0.03
1	0	0.41	1	t	1	Achnanthes lanceoloata	Aoteaphsych e sp.	56	0.03	0.03	0.00	0.03
1	0	0.39	1	cv	1	Achnanthes lanceoloata	Acroperla sp.	56	0.03	0.03	0.00	0.03
1	1	0.67	1	t	1	Achnanthes lanceoloata	Tanytarsini	56	0.03	0.03	0.03	0.03
1	1	0.77	1	t	1	Achnanthes lanceoloata	Aphrophila noevaezeland iae	56	0.03	0.03	0.04	0.03
1	1	0.76	1	t	1	Aulacoseira sp.	Maoridiemes ea	56	0.03	0.03	0.03	0.03
1	1	0.69	1	cv	1	Aulacoseira sp.	Oligochaeta type I	56	0.03	0.03	0.02	0.03
1	0	0.47	1	t	1	Aulacoseira sp.	Stratiomyiid sp.	56	0.03	0.03	0.00	0.03
1	0	0.49	1	t	1	Aulacoseira	Cricotopus II	56	0.03	0.03	0.01	0.03
1	1	0.54	1	t	1	Aulacoseira sp.	Oligochaeta type II	56	0.03	0.03	0.01	0.03
1	1	0.54	1	cv	1	Aulacoseira sp.	Nesameletus ornatus	56	0.03	0.03	0.01	0.03
1	1	0.53	1	t	1	Aulacoseira	Stictocladius	56	0.03	0.03	0.01	0.03

1	1	0.65	1	t	1	Aulacoseira sp.	Oxyethira albiceps	56	0.03	0.03	0.02	0.03
1	1	0.71	1	t	1	Aulacoseira sp.	Austroclima jollyae	56	0.03	0.03	0.03	0.03
1	1	0.72	1	cv	1	Aulacoseira sp.	Potamopurgus antipodarum	56	0.03	0.03	0.03	0.03
1	1	0.53	1	t	1	Aulacoseira sp.	Hydora nitida larvae	56	0.03	0.03	0.01	0.03
1	1	0.68	1	t	1	Aulacoseira sp.	Paralimnophora skusei	56	0.03	0.03	0.02	0.03
1	1	0.65	1	t	1	Aulacoseira sp.	Coloburiscus humeralis	56	0.03	0.03	0.02	0.03
1	1	0.54	1	cv	1	Aulacoseira sp.	Deleatidium spp.	56	0.03	0.03	0.01	0.03
1	1	0.77	1	t	1	Aulacoseira sp.	Paranephrops zealandicus	56	0.03	0.03	0.03	0.03
1	1	0.64	1	t	1	Aulacoseira sp.	Hexatomini sp.	56	0.03	0.03	0.02	0.03
1	1	0.69	1	t	1	Aulacoseira sp.	Austrosimulium australense	56	0.03	0.03	0.02	0.03
1	0	0.47	1	cv	1	Aulacoseira sp.	Aoteaphsyche sp.	56	0.03	0.03	0.00	0.03
1	0	0.45	1	t	1	Aulacoseira	Acroperla sp.	56	0.03	0.03	0.00	0.03
1	1	0.73	1	t	1	Aulacoseira	Tanytarsini	56	0.03	0.03	0.03	0.03
1	1	0.81	1	t	1	Aulacoseira sp.	Aphrophila noevaezelandiae	56	0.03	0.03	0.04	0.03
1	1	0.76	1	cv	1	Aulacoseira	Eukiefiriella	56	0.03	0.03	0.03	0.03
1	0	0.35	1	t	1	Rhoicosphenia curvata	Stratiomyiid sp.	56	0.02	0.03	0.00	0.03
1	1	0.59	1	t	1	Rhoicosphenia curvata	Ceratopogonid sp.	56	0.02	0.03	0.03	0.03
1	0	0.41	1	t	1	Rhoicosphenia curvata	Oligochaeta type II	56	0.02	0.03	0.01	0.03
1	0	0.41	1	cv	1	Rhoicosphenia curvata	Orchymontia sp.	56	0.02	0.03	0.01	0.03
0	0	0.33	1	t	1	Rhoicosphenia curvata	Amphipoda spp.	56	0.02	0.03	0.00	0.03
1	1	0.60	1	t	1	Rhoicosphenia curvata	Potamopurgus antipodarum	56	0.02	0.03	0.03	0.03
1	0	0.40	1	t	1	Rhoicosphenia curvata	Hydora nitida larvae	56	0.02	0.03	0.01	0.03
1	1	0.52	1	cv	1	Rhoicosphenia curvata	Coloburiscus humeralis	56	0.02	0.03	0.02	0.03
1	0	0.41	1	t	1	Rhoicosphenia curvata	Deleatidium spp.	56	0.02	0.03	0.01	0.03

1	1	0.57	1	t	1	Rhoicosphenia curvata	Austrosimulium australense	56	0.02	0.03	0.02	0.03
1	1	0.61	1	t	1	Rhoicosphenia curvata	Tanytarsini	56	0.02	0.03	0.03	0.03
1	1	0.65	1	cv	1	Rhoicosphenia curvata	Eukiefiriella	56	0.02	0.03	0.03	0.03
0	0	0.33	1	t	1	Hydrobiosid sp.	Unidentified detritus	56	0.00	0.03	0.02	0.03
0	0	0.23	1	t	1	Hydrobiosid sp.	Aoteaphsyche sp.	56	0.00	0.03	0.00	0.03
1	1	0.68	1	t	1	Zealolessica cheira	Fragilaria vaucheriae	56	0.02	0.03	0.03	0.03
1	1	0.51	1	cv	1	Zealolessica cheira	Achnanthes linearis	56	0.02	0.03	0.01	0.03
1	0	0.40	1	t	1	Zealolessica cheira	moss cells	56	0.02	0.03	0.00	0.03
1	1	0.56	1	t	1	Zealolessica cheira	Cymbella aspera	56	0.02	0.03	0.02	0.03
1	1	0.60	1	t	1	Zealolessica cheira	Macrophyte	56	0.02	0.03	0.02	0.03
1	0	0.40	1	cv	1	Zealolessica cheira	Gomphonema parvulum	56	0.02	0.03	0.00	0.03
1	1	0.59	1	t	1	Zealolessica cheira	Terrestrial invertebrates	56	0.02	0.03	0.02	0.03
1	0	0.38	1	t	1	Zealolessica cheira	Microspora sp.	56	0.02	0.03	0.00	0.03
1	1	0.52	1	t	1	Zealolessica cheira	Unidentified detritus	56	0.02	0.03	0.02	0.03
1	0	0.40	1	cv	1	Zealolessica cheira	Achnanthes saxonica	56	0.02	0.03	0.00	0.03
1	1	0.56	1	t	1	Zealolessica cheira	Gomphonema subclavatum	56	0.02	0.03	0.02	0.03
1	1	0.72	1	t	1	Zealolessica cheira	Gomphonema cf. olivaceum	56	0.02	0.03	0.03	0.03
1	0	0.46	1	t	1	Zealolessica cheira	Eunotia sp.	56	0.02	0.03	0.01	0.03
1	1	0.77	1	cv	1	Zealolessica cheira	Cymbella minuta	56	0.02	0.03	0.04	0.03
1	1	0.57	1	t	1	Zealolessica cheira	Gomphonema truncatum	56	0.02	0.03	0.02	0.03
1	1	0.52	1	t	1	Zealolessica cheira	Epithemia sorex	56	0.02	0.03	0.02	0.03
1	0	0.47	1	t	1	Maoridiemesea	Hydrobiosis parumbripennis	56	0.03	0.03	0.00	0.03
1	1	0.60	1	cv	1	Maoridiemesea	Polyplectropus puerilis	56	0.03	0.03	0.01	0.03
1	0	0.49	1	t	1	Maoridiemese	moss cells	56	0.03	0.03	0.00	0.03

1	1	0.52	1	t	1	Maoridiemesea	Tabellaria flocculosa	56	0.03	0.03	0.01	0.03
1	1	0.65	1	t	1	Maoridiemesea	Cymbella aspera	56	0.03	0.03	0.02	0.03
1	1	0.72	1	cv	1	Maoridiemesea	Pinnularia sp. (large)	56	0.03	0.03	0.03	0.03
1	1	0.68	1	t	1	Maoridiemese	Macrophyte	56	0.03	0.03	0.02	0.03
1	1	0.50	1	t	1	Maoridiemesea	Meridion circulare	56	0.03	0.03	0.01	0.03
1	1	0.71	1	t	1	Maoridiemesea	Navicula rhynchocephala	56	0.03	0.03	0.03	0.03
1	0	0.48	1	cv	1	Maoridiemesea	Gomphonema parvulum	56	0.03	0.03	0.00	0.03
1	1	0.62	1	t	1	Maoridiemesea	Pinnularia (small - mesolepta??)	56	0.03	0.03	0.02	0.03
1	1	0.61	1	t	1	Maoridiemesea	Cymbella cuspidata	56	0.03	0.03	0.02	0.03
1	0	0.47	1	t	1	Maoridiemese	Salmo trutta	56	0.03	0.03	0.00	0.03
1	0	0.47	1	cv	1	Maoridiemesea	Microspora sp.	56	0.03	0.03	0.00	0.03
1	1	0.61	1	t	1	Maoridiemesea	Unidentified detritus	56	0.03	0.03	0.02	0.03
1	0	0.49	1	t	1	Maoridiemesea	Achnanthes saxonica	56	0.03	0.03	0.00	0.03
1	1	0.65	1	t	1	Maoridiemesea	Gomphonema subclavatum	56	0.03	0.03	0.02	0.03
1	1	0.79	1	cv	1	Maoridiemesea	Gomphonema cf. olivaceum	56	0.03	0.03	0.03	0.03
1	1	0.55	1	t	1	Maoridiemese	Eunotia sp.	56	0.03	0.03	0.01	0.03
1	1	0.65	1	t	1	Maoridiemesea	Navicula cryptocephala	56	0.03	0.03	0.02	0.03
1	1	0.73	1	t	1	Maoridiemesea	Nitzschia sp. (large)	56	0.03	0.03	0.03	0.03
1	1	0.83	1	cv	1	Maoridiemesea	Cymbella minuta	56	0.03	0.03	0.04	0.03
1	1	0.66	1	t	1	Maoridiemesea	Gomphonema truncatum	56	0.03	0.03	0.02	0.03
1	1	0.55	1	t	1	Maoridiemesea	Deleatidium spp.	56	0.03	0.03	0.01	0.03
1	1	0.52	1	t	1	Larsia II	Unidentified detritus	56	0.02	0.02	0.02	0.03
1	0	0.41	1	cv	1	Hydrobiosis parumbripenn	Polypedellum	56	0.00	0.03	0.03	0.03
1	0	0.36	1	t	1	Hydrobiosis parumbripenn	Pirara	56	0.00	0.03	0.02	0.03

1	0	0.23	1	t	1	Hydrobiosis parumbripenn	Cricotopus II	56	0.00	0.03	0.01	0.03
1	0	0.31	1	t	1	Hydrobiosis parumbripenn	Megaleptoperla diminuta	56	0.00	0.03	0.01	0.02
1	0	0.38	1	cv	1	Hydrobiosis parumbripenn	Terrestrial invertebrates	56	0.00	0.03	0.02	0.03
1	0	0.26	1	t	1	Hydrobiosis parumbripenn	Orchymontia sp.	56	0.00	0.03	0.01	0.03
1	0	0.20	1	t	1	Hydrobiosis parumbripenn	Salmo trutta	56	0.00	0.03	0.00	0.03
1	0	0.32	1	t	1	Hydrobiosis parumbripenn	Unidentified detritus	56	0.00	0.03	0.02	0.03
1	0	0.33	1	cv	1	Hydrobiosis parumbripenn	Stenoperla prasinia	56	0.00	0.03	0.02	0.03
1	0	0.26	1	t	1	Hydrobiosis parumbripenn	Deleatidium spp.	56	0.00	0.03	0.01	0.03
0	0	0.22	1	t	1	Hydrobiosis parumbripenn	Hydrobiosella sp.	56	0.00	0.03	0.00	0.03
1	0	0.49	1	t	1	Hydrobiosis parumbripenn	Eukiefiriella	56	0.00	0.03	0.03	0.03
1	0	0.32	1	cv	1	Hydrobiosis parumbripenn	Psilachorema bidens	56	0.00	0.03	0.02	0.03
1	1	0.72	1	t	1	Eunotia sp. large	Aphrophila noevaezelandiae	56	0.02	0.03	0.04	0.03
1	0	0.43	1	t	1	Unknown green algal filaments	Potamopurgus antipodarum	56	0.00	0.03	0.03	0.03
1	1	0.54	1	t	1	Polyplectropus puerilis	Polypedellum	56	0.01	0.03	0.03	0.03
1	0	0.48	1	cv	1	Polyplectropus puerilis	Pirara	56	0.01	0.03	0.02	0.03
1	0	0.33	1	t	1	Polyplectropus puerilis	Cricotopus II	56	0.01	0.03	0.01	0.03
1	0	0.43	1	t	1	Polyplectropus puerilis	Megaleptoperla diminuta	56	0.01	0.03	0.01	0.02
1	0	0.50	1	t	1	Polyplectropus puerilis	Terrestrial invertebrates	56	0.01	0.03	0.02	0.03
1	0	0.37	1	cv	1	Polyplectropus puerilis	Orchymontia sp.	56	0.01	0.03	0.01	0.03
1	0	0.30	1	t	1	Polyplectropus puerilis	Salmo trutta	56	0.01	0.03	0.00	0.03
1	0	0.43	1	t	1	Polyplectropus puerilis	Unidentified detritus	56	0.01	0.03	0.02	0.03
1	0	0.45	1	t	1	Polyplectropus puerilis	Stenoperla prasinia	56	0.01	0.03	0.02	0.03
1	0	0.37	1	cv	1	Polyplectropus puerilis	Deleatidium spp.	56	0.01	0.03	0.01	0.03
1	1	0.61	1	t	1	Polyplectropus puerilis	Eukiefiriella	56	0.01	0.03	0.03	0.03

1	0	0.43	1	t	1	Polyplectropus puerilis	Psilachorema bidens	56	0.01	0.03	0.02	0.03
1	1	0.69	1	t	1	Rhopalodia novae zelandicae	Potamopurgu s antipodarum	56	0.03	0.03	0.03	0.03
1	1	0.69	1	cv	1	Polypedellum	Fragilaria vaucheriae	56	0.03	0.03	0.03	0.03
1	0	0.42	1	t	1	Polypedellum	moss cells	56	0.03	0.03	0.00	0.03
1	0	0.45	1	t	1	Polypedellum	Tabellaria flocculosa	56	0.03	0.03	0.01	0.03
1	1	0.61	1	t	1	Polypedellum	Macrophyte	56	0.03	0.03	0.02	0.03
1	1	0.65	1	cv	1	Polypedellum	Navicula rhynchoceph ala	56	0.03	0.03	0.03	0.03
1	1	0.55	1	t	1	Polypedellum	Pinnularia (small - mesolepta??)	56	0.03	0.03	0.02	0.03
1	0	0.40	1	t	1	Polypedellum	Cyclotella sp.	56	0.03	0.03	0.00	0.03
1	0	0.40	1	t	1	Polypedellum	Microspora sp.	56	0.03	0.03	0.00	0.03
1	1	0.54	1	cv	1	Polypedellum	Unidentified detritus	56	0.03	0.03	0.02	0.03
1	1	0.74	1	t	1	Polypedellum	Gomphonem a cf. olivaceum	56	0.03	0.03	0.03	0.03
1	0	0.48	1	t	1	Polypedellum	Eunotia sp.	56	0.03	0.03	0.01	0.03
1	1	0.58	1	t	1	Polypedellum	Navicula cryptocephal a	56	0.03	0.03	0.02	0.03
1	1	0.68	1	cv	1	Polypedellum	Nitzschia sp. (large)	56	0.03	0.03	0.03	0.03
1	1	0.68	1	t	1	Fragilaria vaucheriae	Oligochaeta type I	56	0.03	0.03	0.02	0.03
1	0	0.46	1	t	1	Fragilaria vaucheriae	Stratiomyiid sp.	56	0.03	0.03	0.00	0.03
1	0	0.48	1	t	1	Fragilaria vaucheriae	Cricotopus II	56	0.03	0.03	0.01	0.03
1	1	0.53	1	cv	1	Fragilaria vaucheriae	Oligochaeta type II	56	0.03	0.03	0.01	0.03
1	1	0.53	1	t	1	Fragilaria vaucheriae	Orchymontia sp.	56	0.03	0.03	0.01	0.03
1	1	0.52	1	t	1	Fragilaria vaucheriae	Stictocladius	56	0.03	0.03	0.01	0.03
1	1	0.64	1	t	1	Fragilaria vaucheriae	Oxyethira albiceps	56	0.03	0.03	0.02	0.03
1	0	0.44	1	cv	1	Fragilaria vaucheriae	Amphipoda spp.	56	0.03	0.03	0.00	0.03
1	1	0.70	1	t	1	Fragilaria vaucheriae	Austroclima jollyae	56	0.03	0.03	0.03	0.03

1	1	0.71	1	t	1	Fragilaria vaucheriae	Potamopurgus antipodarum	56	0.03	0.03	0.03	0.03
1	1	0.76	1	t	1	Fragilaria vaucheriae	Naonella	56	0.03	0.03	0.03	0.03
1	1	0.52	1	cv	1	Fragilaria vaucheriae	Hydora nitida larvae	56	0.03	0.03	0.01	0.03
1	1	0.53	1	t	1	Fragilaria vaucheriae	Deleatidium spp.	56	0.03	0.03	0.01	0.03
1	1	0.68	1	t	1	Fragilaria vaucheriae	Austrosimulium australense	56	0.03	0.03	0.02	0.03
1	1	0.55	1	t	1	Fragilaria vaucheriae	Pycnocentria sp.	56	0.03	0.03	0.01	0.03
1	0	0.46	1	cv	1	Fragilaria vaucheriae	Aoteaphsyche sp.	56	0.03	0.03	0.00	0.03
1	1	0.72	1	t	1	Fragilaria vaucheriae	Tanytarsini	56	0.03	0.03	0.03	0.03
1	1	0.80	1	t	1	Fragilaria vaucheriae	Aphrophila noevaezelandiae	56	0.03	0.03	0.04	0.03
1	1	0.75	1	t	1	Fragilaria vaucheriae	Eukiefiriella	56	0.03	0.03	0.03	0.03
1	0	0.39	1	cv	1	Pirara	Tabellaria flocculosa	56	0.02	0.03	0.01	0.03
1	1	0.60	1	t	1	Pirara	Surirella (small)	56	0.02	0.03	0.03	0.03
1	1	0.56	1	t	1	Pirara	Macrophyte	56	0.02	0.03	0.02	0.03
1	0	0.36	1	t	1	Pirara	Gomphonema parvulum	56	0.02	0.03	0.00	0.03
1	0	0.34	1	cv	1	Pirara	Cyclotella sp.	56	0.02	0.03	0.00	0.03
1	1	0.81	1	t	1	Pirara	Frustulia rhomboides	56	0.02	0.03	0.05	0.03
1	0	0.48	1	t	1	Pirara	Unidentified detritus	56	0.02	0.03	0.02	0.03
1	0	0.42	1	t	1	Pirara	Eunotia sp.	56	0.02	0.03	0.01	0.03
1	1	0.52	1	cv	1	Pirara	Navicula cryptocephala	56	0.02	0.03	0.02	0.03
1	1	0.74	1	t	1	Pirara	Cymbella minuta	56	0.02	0.03	0.04	0.03
1	1	0.53	1	t	1	Pirara	Gomphonema truncatum	56	0.02	0.03	0.02	0.03
1	1	0.73	1	t	1	Pirara	Synedra ulna	56	0.02	0.03	0.04	0.03
1	0	0.36	1	cv	1	Pirara	Aoteaphsyche sp.	56	0.02	0.03	0.00	0.03
1	1	0.65	1	t	1	Pirara	Navicula avenacea	56	0.02	0.03	0.03	0.03
1	0	0.48	1	t	1	Pirara	Epithemia sorex	56	0.02	0.03	0.02	0.03

1	0	0.44	1	t	1	Pirara	Costachorema xanthoptera	56	0.02	0.03	0.01	0.03
1	0	0.48	1	cv	1	Pirara	Psilachorema bidens	56	0.02	0.03	0.02	0.03
1	0	0.44	1	t	1	Oligochaeta type I	Tabellaria flocculosa	56	0.02	0.03	0.01	0.03
1	1	0.57	1	t	1	Oligochaeta type I	Cymbella aspera	56	0.02	0.03	0.02	0.03
1	1	0.64	1	t	1	Oligochaeta type I	Pinnularia sp. (large)	56	0.02	0.03	0.03	0.03
1	1	0.60	1	cv	1	Oligochaeta type I	Macrophyte	56	0.02	0.03	0.02	0.03
1	0	0.42	1	t	1	Oligochaeta type I	Meridion circulare	56	0.02	0.03	0.01	0.03
1	1	0.63	1	t	1	Oligochaeta type I	Navicula rhynchocephala	56	0.02	0.03	0.03	0.03
1	0	0.50	1	t	1	Oligochaeta type I	Synedra rumpens	56	0.02	0.03	0.01	0.03
1	0	0.38	1	cv	1	Oligochaeta type I	Surirella tenera ??	56	0.02	0.03	0.00	0.03
1	1	0.52	1	t	1	Oligochaeta type I	Cymbella cuspidata	56	0.02	0.03	0.02	0.03
1	0	0.38	1	t	1	Oligochaeta type I	Cyclotella sp.	56	0.02	0.03	0.00	0.03
1	1	0.58	1	t	1	Oligochaeta type I	Nitzschia sp. (small)	56	0.02	0.03	0.02	0.03
1	1	0.83	1	cv	1	Oligochaeta type I	Frustulia rhomboides	56	0.02	0.03	0.05	0.03
1	1	0.53	1	t	1	Oligochaeta type I	Unidentified detritus	56	0.02	0.03	0.02	0.03
1	1	0.56	1	t	1	Oligochaeta type I	Gomphonema subclavatum	56	0.02	0.03	0.02	0.03
1	0	0.47	1	t	1	Oligochaeta type I	Eunotia sp.	56	0.02	0.03	0.01	0.03
1	1	0.56	1	cv	1	Oligochaeta type I	Navicula cryptocephala	56	0.02	0.03	0.02	0.03
1	1	0.66	1	t	1	Oligochaeta type I	Nitzschia sp. (large)	56	0.02	0.03	0.03	0.03
1	1	0.68	1	t	1	Oligochaeta type I	Cymbella kappii	56	0.02	0.03	0.03	0.03
1	1	0.78	1	t	1	Oligochaeta type I	Cymbella minuta	56	0.02	0.03	0.04	0.03
1	0	0.45	1	cv	1	Oligochaeta type I	Gomphoneis herculeana	56	0.02	0.03	0.01	0.03
1	1	0.69	1	t	1	Oligochaeta type I	Navicula avenacea	56	0.02	0.03	0.03	0.03

1	1	0.67	1	t	1	Oligochaeta type I	Diatoma heimale	56	0.02	0.03	0.03	0.03
1	0	0.43	1	t	1	Stratiomyiid sp.	Surirella (small)	56	0.00	0.03	0.03	0.03
0	0	0.21	1	cv	1	Stratiomyiid	Cyclotella sp.	56	0.00	0.03	0.00	0.03
1	0	0.37	1	t	1	Stratiomyiid sp.	Nitzschia sp. (small)	56	0.00	0.03	0.02	0.03
1	1	0.68	1	t	1	Stratiomyiid sp.	Frustulia rhomboides	56	0.00	0.03	0.05	0.03
1	0	0.32	1	t	1	Stratiomyiid sp.	Unidentified detritus	56	0.00	0.03	0.02	0.03
1	0	0.46	1	cv	1	Stratiomyiid sp.	Nitzschia sp. (large)	56	0.00	0.03	0.03	0.03
1	1	0.59	1	t	1	Stratiomyiid sp.	Cymbella minuta	56	0.00	0.03	0.04	0.03
1	1	0.57	1	t	1	Stratiomyiid	Synedra ulna	56	0.00	0.03	0.04	0.03
0	0	0.21	1	t	1	Stratiomyiid	Audouinella	56	0.00	0.03	0.00	0.03
1	0	0.49	1	cv	1	Stratiomyiid sp.	Navicula avenacea	56	0.00	0.03	0.03	0.03
1	0	0.47	1	t	1	Stratiomyiid sp.	Diatoma heimale	56	0.00	0.03	0.03	0.03
0	0	0.32	1	t	1	Achnanthes linearis	Cricotopus II	56	0.01	0.03	0.01	0.03
1	0	0.37	1	t	1	Achnanthes linearis	Orchymontia sp.	56	0.01	0.03	0.01	0.03
1	0	0.37	1	cv	1	Achnanthes linearis	Nesameletus ornatus	56	0.01	0.03	0.01	0.03
0	0	0.35	1	t	1	Achnanthes linearis	Stictocladius	56	0.01	0.03	0.01	0.03
1	1	0.54	1	t	1	Achnanthes linearis	Austroclima jollyae	56	0.01	0.03	0.03	0.03
1	0	0.46	1	t	1	Achnanthes linearis	Podaena sp.	56	0.01	0.03	0.02	0.03
1	1	0.55	1	cv	1	Achnanthes linearis	Potamopurgus antipodarum	56	0.01	0.03	0.03	0.03
1	0	0.35	1	t	1	Achnanthes linearis	Hydora nitida larvae	56	0.01	0.03	0.01	0.03
1	0	0.47	1	t	1	Achnanthes linearis	Coloburiscus humeralis	56	0.01	0.03	0.02	0.03
1	0	0.37	1	t	1	Achnanthes linearis	Deleatidium spp.	56	0.01	0.03	0.01	0.03
1	0	0.38	1	cv	1	Achnanthes linearis	Pycnocentria sp.	56	0.01	0.03	0.01	0.03
0	0	0.31	1	t	1	Achnanthes linearis	Aoteaphsyche sp.	56	0.01	0.03	0.00	0.03
1	1	0.56	1	t	1	Achnanthes linearis	Tanytarsini	56	0.01	0.03	0.03	0.03
1	1	0.68	1	t	1	Achnanthes linearis	Aphrophila noevaezelandiae	56	0.01	0.03	0.04	0.03

1	1	0.60	1	cv	1	Achnanthes linearis	Eukiefiriella	56	0.01	0.03	0.03	0.03
1	1	0.73	1	t	1	Achnanthes minutissima	Ceratopogonid sp.	56	0.03	0.03	0.03	0.03
1	1	0.75	1	t	1	Achnanthes minutissima	Tanytarsini	56	0.03	0.03	0.03	0.03
1	1	0.52	1	t	1	moss cells	Paranephrops zealandicus	56	0.00	0.03	0.03	0.03
1	0	0.29	1	cv	1	moss cells	Pycnocentria sp.	56	0.00	0.03	0.01	0.03
0	0	0.23	1	t	1	moss cells	Aoteaphsyche sp.	56	0.00	0.03	0.00	0.03
0	0	0.21	1	t	1	moss cells	Acroperla sp.	56	0.00	0.03	0.00	0.03
0	0	0.26	1	t	1	Cricotopus II	Tabellaria flocculosa	56	0.01	0.03	0.01	0.03
1	0	0.37	1	cv	1	Cricotopus II	Cymbella aspera	56	0.01	0.03	0.02	0.03
0	0	0.25	1	t	1	Cricotopus II	Meridion circulare	56	0.01	0.03	0.01	0.03
1	0	0.44	1	t	1	Cricotopus II	Navicula rhynchocephala	56	0.01	0.03	0.03	0.03
0	0	0.24	1	t	1	Cricotopus II	Gomphonema parvulum	56	0.01	0.03	0.00	0.03
0	0	0.31	1	cv	1	Cricotopus II	Synedra rumpens	56	0.01	0.03	0.01	0.03
1	0	0.35	1	t	1	Cricotopus II	Pinnularia (small - mesolepta??)	56	0.01	0.03	0.02	0.03
0	0	0.22	1	t	1	Cricotopus II	Salmo trutta	56	0.01	0.03	0.00	0.03
0	0	0.22	1	t	1	Cricotopus II	Cyclotella sp.	56	0.01	0.03	0.00	0.03
1	0	0.43	1	cv	1	Cricotopus II	Cocconeis sp.	56	0.01	0.03	0.02	0.03
1	0	0.34	1	t	1	Cricotopus II	Unidentified detritus	56	0.01	0.03	0.02	0.03
1	0	0.37	1	t	1	Cricotopus II	Gomphonema subclavatum	56	0.01	0.03	0.02	0.03
1	1	0.55	1	t	1	Cricotopus II	Gomphonema cf. olivaceum	56	0.01	0.03	0.03	0.03
0	0	0.29	1	cv	1	Cricotopus II	Eunotia sp.	56	0.01	0.03	0.01	0.03
1	0	0.37	1	t	1	Cricotopus II	Navicula cryptocephala	56	0.01	0.03	0.02	0.03
1	0	0.48	1	t	1	Cricotopus II	Nitzschia sp. (large)	56	0.01	0.03	0.03	0.03
1	0	0.50	1	t	1	Cricotopus II	Cymbella kappii	56	0.01	0.03	0.03	0.03
1	1	0.61	1	cv	1	Cricotopus II	Cymbella minuta	56	0.01	0.03	0.04	0.03

1	0	0.39	1	t	1	Cricotopus II	Gomphonema truncatum	56	0.01	0.03	0.02	0.03
1	1	0.59	1	t	1	Cricotopus II	Synedra ulna	56	0.01	0.03	0.04	0.03
0	0	0.26	1	t	1	Cricotopus II	Diploneis sp.	56	0.01	0.03	0.01	0.03
1	1	0.51	1	cv	1	Cricotopus II	Navicula avenacea	56	0.01	0.03	0.03	0.03
1	0	0.49	1	t	1	Cricotopus II	Diatoma heimale	56	0.01	0.03	0.03	0.03
1	1	0.56	1	t	1	Fragilaria spp.	Aphrophila noevaezelandiae	56	0.00	0.03	0.04	0.03
1	1	0.57	1	t	1	Stauroneis sp.	Oxyethira albiceps	56	0.02	0.03	0.02	0.03
1	0	0.40	1	cv	1	Stauroneis sp.	Aoteaphsyche sp.	56	0.02	0.03	0.00	0.03
1	1	0.76	1	t	1	Stauroneis sp.	Aphrophila noevaezelandiae	56	0.02	0.03	0.04	0.03
0	0	0.29	1	t	1	Tabellaria flocculosa	Stictocladius	56	0.01	0.03	0.01	0.03
1	0	0.40	1	t	1	Tabellaria flocculosa	Oxyethira albiceps	56	0.01	0.03	0.02	0.03
1	0	0.47	1	cv	1	Tabellaria flocculosa	Austroclima jollyae	56	0.01	0.03	0.03	0.03
0	0	0.25	1	t	1	Tabellaria flocculosa	Aoteaphsyche sp.	56	0.01	0.03	0.00	0.03
0	0	0.26	1	t	1	Tabellaria flocculosa	Scirtid sp. (Skinny type)	56	0.01	0.03	0.01	0.03
1	0	0.49	1	t	1	Tabellaria flocculosa	Tanytarsini	56	0.01	0.03	0.03	0.03
1	1	0.54	1	cv	1	Tabellaria flocculosa	Eukiefiriella	56	0.01	0.03	0.03	0.03
1	0	0.41	1	t	1	Cymbella aspera	Oligochaeta type II	56	0.02	0.03	0.01	0.03
1	1	0.52	1	t	1	Cymbella aspera	Oxyethira albiceps	56	0.02	0.03	0.02	0.03
1	1	0.59	1	t	1	Cymbella aspera	Austroclima jollyae	56	0.02	0.03	0.03	0.03
1	1	0.60	1	cv	1	Cymbella aspera	Potamopurgus antipodarum	56	0.02	0.03	0.03	0.03
1	1	0.56	1	t	1	Cymbella aspera	Paralimnophora skusei	56	0.02	0.03	0.02	0.03
1	1	0.52	1	t	1	Cymbella aspera	Coloburiscus humeralis	56	0.02	0.03	0.02	0.03
1	0	0.41	1	t	1	Cymbella aspera	Deleatidium spp.	56	0.02	0.03	0.01	0.03
1	1	0.51	1	cv	1	Cymbella aspera	Hexatomini sp.	56	0.02	0.03	0.02	0.03

1	1	0.57	1	t	1	Cymbella aspera	Austrosimulium australense	56	0.02	0.03	0.02	0.03
1	0	0.43	1	t	1	Cymbella aspera	Pycnocentria sp.	56	0.02	0.03	0.01	0.03
1	0	0.35	1	t	1	Cymbella aspera	Aoteaphsyche sp.	56	0.02	0.03	0.00	0.03
1	1	0.72	1	cv	1	Cymbella aspera	Aphrophila noevaezelandiae	56	0.02	0.03	0.04	0.03
1	0	0.40	1	t	1	Surirella (small)	Amphipoda spp.	56	0.03	0.03	0.00	0.03
1	1	0.77	1	t	1	Surirella (small)	Aphrophila noevaezelandiae	56	0.03	0.03	0.04	0.03
1	1	0.60	1	t	1	Pinnularia sp. (large)	Oxyethira albiceps	56	0.03	0.03	0.02	0.03
1	1	0.66	1	cv	1	Pinnularia sp. (large)	Austroclima jollyae	56	0.03	0.03	0.03	0.03
1	1	0.67	1	t	1	Pinnularia sp. (large)	Potamopurgus antipodarum	56	0.03	0.03	0.03	0.03
1	0	0.47	1	t	1	Pinnularia sp. (large)	Hydora nitida larvae	56	0.03	0.03	0.01	0.03
1	0	0.49	1	t	1	Pinnularia sp. (large)	Deleatidium spp.	56	0.03	0.03	0.01	0.03
1	1	0.73	1	cv	1	Pinnularia sp. (large)	Paranephrops zealandicus	56	0.03	0.03	0.03	0.03
1	0	0.42	1	t	1	Pinnularia sp. (large)	Aoteaphsyche sp.	56	0.03	0.03	0.00	0.03
1	1	0.68	1	t	1	Pinnularia sp. (large)	Tanytarsini	56	0.03	0.03	0.03	0.03
1	1	0.72	1	t	1	Pinnularia sp. (large)	Eukiefiriella	56	0.03	0.03	0.03	0.03
1	0	0.45	1	cv	1	Macrophyte	Oligochaeta type II	56	0.02	0.03	0.01	0.03
1	1	0.52	1	t	1	Macrophyte	Austroperla cyrene	56	0.02	0.03	0.02	0.03
1	0	0.36	1	t	1	Macrophyte	Amphipoda spp.	56	0.02	0.03	0.00	0.03
1	1	0.62	1	t	1	Macrophyte	Austroclima jollyae	56	0.02	0.03	0.03	0.03
1	1	0.63	1	cv	1	Macrophyte	Potamopurgus antipodarum	56	0.02	0.03	0.03	0.03
1	1	0.53	1	t	1	Macrophyte	Stenoperla prasinia	56	0.02	0.03	0.02	0.03
1	1	0.69	1	t	1	Macrophyte	Paranephrops zealandicus	56	0.02	0.03	0.03	0.03
1	0	0.47	1	t	1	Macrophyte	Pycnocentria sp.	56	0.02	0.03	0.01	0.03

1	0	0.38	1	cv	1	Macrophyte	Aoteaphsyche sp.	56	0.02	0.03	0.00	0.03
1	0	0.36	1	t	1	Macrophyte	Acroperla sp.	56	0.02	0.03	0.00	0.03
1	1	0.75	1	t	1	Macrophyte	Aphrophila noevaezelandiae	56	0.02	0.03	0.04	0.03
1	1	0.68	1	t	1	Macrophyte	Eukiefiriella	56	0.02	0.03	0.03	0.03
1	0	0.39	1	cv	1	Macrophyte	Megaleptoperla grandis	56	0.02	0.03	0.00	0.03
0	0	0.29	1	t	1	Meridion circulare	Oligochaeta type II	56	0.01	0.03	0.01	0.03
0	0	0.35	1	t	1	Meridion circulare	Austroperla cyrene	56	0.01	0.03	0.02	0.03
0	0	0.28	1	t	1	Meridion circulare	Stictocladius	56	0.01	0.03	0.01	0.03
1	0	0.39	1	cv	1	Meridion circulare	Oxyethira albiceps	56	0.01	0.03	0.02	0.03
1	0	0.46	1	t	1	Meridion circulare	Potamopurgus antipodarum	56	0.01	0.03	0.03	0.03
1	1	0.53	1	t	1	Meridion circulare	Naonella	56	0.01	0.03	0.03	0.03
0	0	0.28	1	t	1	Meridion circulare	Hydora nitida larvae	56	0.01	0.03	0.01	0.03
1	0	0.38	1	cv	1	Meridion circulare	Coloburiscus humeralis	56	0.01	0.03	0.02	0.03
0	0	0.29	1	t	1	Meridion circulare	Deleatidium spp.	56	0.01	0.03	0.01	0.03
1	0	0.43	1	t	1	Meridion circulare	Austrosimulium australense	56	0.01	0.03	0.02	0.03
0	0	0.24	1	t	1	Meridion circulare	Aoteaphsyche sp.	56	0.01	0.03	0.00	0.03
1	0	0.48	1	cv	1	Meridion circulare	Tanytarsini	56	0.01	0.03	0.03	0.03
1	1	0.60	1	t	1	Meridion circulare	Aphrophila noevaezelandiae	56	0.01	0.03	0.04	0.03
1	1	0.52	1	t	1	Meridion circulare	Eukiefiriella	56	0.01	0.03	0.03	0.03
1	0	0.44	1	t	1	Megaleptoperla diminuta	Unidentified detritus	56	0.01	0.02	0.02	0.03
0	0	0.22	1	cv	1	Navicula sp.	Amphipoda spp.	56	0.01	0.03	0.00	0.03
1	0	0.38	1	t	1	Navicula sp.	Coloburiscus humeralis	56	0.01	0.03	0.02	0.03
1	1	0.52	1	t	1	Navicula sp.	Eukiefiriella	56	0.01	0.03	0.03	0.03
1	0	0.40	1	t	1	Ceratopogonid sp.	Cyclotella sp.	56	0.03	0.03	0.00	0.03
1	1	0.60	1	cv	1	Ceratopogonid sp.	Nitzschia sp. (small)	56	0.03	0.03	0.02	0.03

1	1	0.55	1	t	1	Ceratopogonid sp.	Gomphonema acuminatum	56	0.03	0.03	0.02	0.03
1	1	0.79	1	t	1	Ceratopogonid sp.	Cymbella minuta	56	0.03	0.03	0.04	0.03
1	1	0.71	1	t	1	Ceratopogonid sp.	Navicula avenacea	56	0.03	0.03	0.03	0.03
1	1	0.69	1	cv	1	Ceratopogonid sp.	Diatoma heimale	56	0.03	0.03	0.03	0.03
1	0	0.26	1	t	1	Oligochaeta type II	Surirella tenera ??	56	0.01	0.03	0.00	0.03
0	0	0.26	1	t	1	Oligochaeta type II	Cyclotella sp.	56	0.01	0.03	0.00	0.03
1	0	0.43	1	t	1	Oligochaeta type II	Nitzschia sp. (small)	56	0.01	0.03	0.02	0.03
1	1	0.74	1	cv	1	Oligochaeta type II	Frustulia rhomboides	56	0.01	0.03	0.05	0.03
1	0	0.38	1	t	1	Oligochaeta type II	Unidentified detritus	56	0.01	0.03	0.02	0.03
1	1	0.59	1	t	1	Oligochaeta type II	Gomphonema cf. olivaceum	56	0.01	0.03	0.03	0.03
1	0	0.33	1	t	1	Oligochaeta type II	Eunotia sp.	56	0.01	0.03	0.01	0.03
1	1	0.54	1	cv	1	Oligochaeta type II	Cymbella kappii	56	0.01	0.03	0.03	0.03
1	1	0.66	1	t	1	Oligochaeta type II	Cymbella minuta	56	0.01	0.03	0.04	0.03
1	1	0.64	1	t	1	Oligochaeta type II	Synedra ulna	56	0.01	0.03	0.04	0.03
1	1	0.55	1	t	1	Oligochaeta type II	Navicula avenacea	56	0.01	0.03	0.03	0.03
1	0	0.34	1	cv	1	Oligochaeta type II	Costachorema xanthoptera	56	0.01	0.03	0.01	0.03
1	0	0.38	1	t	1	Oligochaeta type II	Psilachorema bidens	56	0.01	0.03	0.02	0.03
1	1	0.53	1	t	1	Oligochaeta type II	Diatoma heimale	56	0.01	0.03	0.03	0.03
1	1	0.56	1	t	1	Austroperla cyrene	Navicula rhynchocephala	56	0.02	0.03	0.03	0.03
1	0	0.31	1	cv	1	Austroperla cyrene	Microspora sp.	56	0.02	0.03	0.00	0.03
1	0	0.49	1	t	1	Austroperla cyrene	Gomphonema subclavatum	56	0.02	0.03	0.02	0.03
1	1	0.66	1	t	1	Austroperla cyrene	Gomphonema cf. olivaceum	56	0.02	0.03	0.03	0.03

1	0	0.46	1	t	1	Navicula rhynchocephal	Hydora nitida larvae	56	0.03	0.03	0.01	0.03
1	0	0.48	1	cv	1	Navicula rhynchocephal	Deleatidium spp.	56	0.03	0.03	0.01	0.03
1	1	0.67	1	t	1	Navicula rhynchocephal	Tanytarsini	56	0.03	0.03	0.03	0.03
1	1	0.77	1	t	1	Navicula rhynchocephal a	Aphrophila noevaezeland iae	56	0.03	0.03	0.04	0.03
1	0	0.37	1	t	1	Gomphonema parvulum	Oxyethira albiceps	56	0.00	0.03	0.02	0.03
1	0	0.44	1	cv	1	Gomphonema parvulum	Austroclima jollyae	56	0.00	0.03	0.03	0.03
1	0	0.36	1	t	1	Gomphonema parvulum	Podaena sp.	56	0.00	0.03	0.02	0.03
1	1	0.51	1	t	1	Gomphonema parvulum	Naonella	56	0.00	0.03	0.03	0.03
1	0	0.37	1	t	1	Gomphonema parvulum	Coloburiscus humeralis	56	0.00	0.03	0.02	0.03
0	0	0.27	1	cv	1	Gomphonema parvulum	Deleatidium spp.	56	0.00	0.03	0.01	0.03
1	1	0.51	1	t	1	Gomphonema parvulum	Paranephrop s zealandicus	56	0.00	0.03	0.03	0.03
1	0	0.41	1	t	1	Gomphonema parvulum	Austrosimuliu m australense	56	0.00	0.03	0.02	0.03
0	0	0.29	1	t	1	Gomphonema parvulum	Pycnocentria sp.	56	0.00	0.03	0.01	0.03
0	0	0.22	1	cv	1	Gomphonema parvulum	Aoteaphsych e sp.	56	0.00	0.03	0.00	0.03
0	0	0.21	1	t	1	Gomphonema parvulum	Acroperla sp.	56	0.00	0.03	0.00	0.03
1	0	0.46	1	t	1	Gomphonema parvulum	Tanytarsini	56	0.00	0.03	0.03	0.03
1	1	0.58	1	t	1	Gomphonema parvulum	Aphrophila noevaezeland iae	56	0.00	0.03	0.04	0.03
1	0	0.37	1	cv	1	Terrestrial invertebrates	Salmo trutta	56	0.02	0.03	0.00	0.03
1	0	0.46	1	t	1	Terrestrial invertebrates	Pycnocentria sp.	56	0.02	0.03	0.01	0.03
1	0	0.38	1	t	1	Terrestrial invertebrates	Aoteaphsych e sp.	56	0.02	0.03	0.00	0.03
1	0	0.47	1	t	1	Terrestrial invertebrates	Costachorem a xanthoptera	56	0.02	0.03	0.01	0.03
1	1	0.51	1	cv	1	Terrestrial invertebrates	Psilachorema bidens	56	0.02	0.03	0.02	0.03
0	0	0.26	1	t	1	Orchymontia sp.	Cyclotella sp.	56	0.01	0.03	0.00	0.03

1	0	0.43	1	t	1	Orchymontia sp.	Nitzschia sp. (small)	56	0.01	0.03	0.02	0.03
1	0	0.42	1	t	1	Orchymontia sp.	Gomphonema subclavatum	56	0.01	0.03	0.02	0.03
1	0	0.33	1	cv	1	Orchymontia sp.	Eunotia sp.	56	0.01	0.03	0.01	0.03
1	1	0.54	1	t	1	Orchymontia sp.	Cymbella kappii	56	0.01	0.03	0.03	0.03
1	1	0.66	1	t	1	Orchymontia sp.	Cymbella minuta	56	0.01	0.03	0.04	0.03
1	1	0.55	1	t	1	Orchymontia sp.	Navicula avenacea	56	0.01	0.03	0.03	0.03
1	0	0.38	1	cv	1	Orchymontia sp.	Epithemia sorex	56	0.01	0.03	0.02	0.03
1	1	0.53	1	t	1	Orchymontia sp.	Diatoma heimale	56	0.01	0.03	0.03	0.03
0	0	0.25	1	t	1	Surirella tenera ??	Stictocladius	56	0.00	0.03	0.01	0.03
0	0	0.21	1	t	1	Surirella tenera ??	Aoteaphsyche sp.	56	0.00	0.03	0.00	0.03
1	0	0.48	1	cv	1	Surirella tenera ??	Eukiefiriella	56	0.00	0.03	0.03	0.03
1	1	0.51	1	t	1	Larsia I	Unidentified detritus	56	0.02	0.02	0.02	0.03
1	0	0.50	1	t	1	Pinnularia (small -	Oxyethira albiceps	56	0.02	0.03	0.02	0.03
1	1	0.58	1	t	1	Pinnularia (small - mesolepta??)	Potamopurgus antipodarum	56	0.02	0.03	0.03	0.03
1	0	0.49	1	cv	1	Pinnularia (small -	Coloburiscus humeralis	56	0.02	0.03	0.02	0.03
1	0	0.39	1	t	1	Pinnularia (small -	Deleatidium spp.	56	0.02	0.03	0.01	0.03
1	1	0.64	1	t	1	Pinnularia (small -	Paranephrops zealandicus	56	0.02	0.03	0.03	0.03
1	1	0.54	1	t	1	Pinnularia (small - mesolepta??)	Austrosimulium australense	56	0.02	0.03	0.02	0.03
1	0	0.33	1	cv	1	Pinnularia (small -	Aoteaphsyche sp.	56	0.02	0.03	0.00	0.03
1	1	0.59	1	t	1	Pinnularia (small -	Tanytarsini	56	0.02	0.03	0.03	0.03
1	1	0.70	1	t	1	Pinnularia (small - mesolepta??)	Aphrophila noevaezelandiae	56	0.02	0.03	0.04	0.03
1	1	0.63	1	t	1	Pinnularia (small -	Eukiefiriella	56	0.02	0.03	0.03	0.03
0	0	0.26	1	cv	1	Nesameletus ornatus	Cyclotella sp.	56	0.01	0.03	0.00	0.03

1	0	0.43	1	t	1	Nesameletus ornatus	Nitzschia sp. (small)	56	0.01	0.03	0.02	0.03
1	0	0.38	1	t	1	Nesameletus ornatus	Unidentified detritus	56	0.01	0.03	0.02	0.03
1	0	0.27	1	t	1	Nesameletus ornatus	Achnanthes saxonica	56	0.01	0.03	0.00	0.03
1	1	0.59	1	cv	1	Nesameletus ornatus	Gomphonema cf. olivaceum	56	0.01	0.03	0.03	0.03
1	1	0.66	1	t	1	Nesameletus ornatus	Cymbella minuta	56	0.01	0.03	0.04	0.03
0	0	0.25	1	t	1	Nesameletus ornatus	Audouinella	56	0.01	0.03	0.00	0.03
1	1	0.55	1	t	1	Nesameletus ornatus	Navicula avenacea	56	0.01	0.03	0.03	0.03
1	1	0.53	1	cv	1	Nesameletus ornatus	Diatoma heimale	56	0.01	0.03	0.03	0.03
1	1	0.56	1	t	1	Cymbella cuspidata	Potamopurgus antipodarum	56	0.02	0.03	0.03	0.03
0	0	0.31	1	t	1	Cymbella cuspidata	Aoteaphsyche sp.	56	0.02	0.03	0.00	0.03
1	1	0.57	1	t	1	Cymbella cuspidata	Tanytarsini	56	0.02	0.03	0.03	0.03
1	1	0.69	1	cv	1	Cymbella cuspidata	Aphrophila noevaezelandiae	56	0.02	0.03	0.04	0.03
1	0	0.31	1	t	1	Salmo trutta	Unidentified detritus	56	0.00	0.03	0.02	0.03
1	0	0.33	1	t	1	Salmo trutta	Stenoperla prasinia	56	0.00	0.03	0.02	0.03
1	0	0.26	1	t	1	Salmo trutta	Deleatidium spp.	56	0.00	0.03	0.01	0.03
1	1	0.56	1	cv	1	Salmo trutta	Aphrophila noevaezelandiae	56	0.00	0.03	0.04	0.03
0	0	0.25	1	t	1	cf. Batrachospermum??	Hydora nitida larvae	56	0.00	0.03	0.01	0.03
1	1	0.60	1	t	1	Tanyderid sp.	Unidentified detritus	56	0.03	0.03	0.02	0.03
0	0	0.25	1	t	1	Cyclotella sp.	Stictocladius	56	0.00	0.03	0.01	0.03
0	0	0.20	1	cv	1	Cyclotella sp.	Amphipoda spp.	56	0.00	0.03	0.00	0.03
1	0	0.42	1	t	1	Cyclotella sp.	Austroclima jollyae	56	0.00	0.03	0.03	0.03
0	0	0.34	1	t	1	Cyclotella sp.	Podaena sp.	56	0.00	0.03	0.02	0.03
1	0	0.43	1	t	1	Cyclotella sp.	Potamopurgus antipodarum	56	0.00	0.03	0.03	0.03

0	0	0.25	1	cv	1	Cyclotella sp.	Hydora nitida larvae	56	0.00	0.03	0.01	0.03
1	0	0.39	1	t	1	Cyclotella sp.	Paralimnophora skusei	56	0.00	0.03	0.02	0.03
0	0	0.35	1	t	1	Cyclotella sp.	Coloburiscus humeralis	56	0.00	0.03	0.02	0.03
0	0	0.26	1	t	1	Cyclotella sp.	Deleatidium spp.	56	0.00	0.03	0.01	0.03
0	0	0.34	1	cv	1	Cyclotella sp.	Hexatomini sp.	56	0.00	0.03	0.02	0.03
1	0	0.40	1	t	1	Cyclotella sp.	Austrosimulium australense	56	0.00	0.03	0.02	0.03
0	0	0.44	1	t	1	Cyclotella sp.	Tanytarsini	56	0.00	0.03	0.03	0.03
1	1	0.56	1	t	1	Cyclotella sp.	Aphrophila noevaezelandiae	56	0.00	0.03	0.04	0.03
1	0	0.48	1	cv	1	Cyclotella sp.	Eukiefiriella	56	0.00	0.03	0.03	0.03
1	0	0.46	1	t	1	Cocconeis sp.	Stictocladius	56	0.02	0.03	0.01	0.03
1	1	0.58	1	t	1	Cocconeis sp.	Oxyethira albiceps	56	0.02	0.03	0.02	0.03
1	1	0.64	1	t	1	Cocconeis sp.	Austroclima jollyae	56	0.02	0.03	0.03	0.03
1	1	0.71	1	cv	1	Cocconeis sp.	Naonella	56	0.02	0.03	0.03	0.03
1	0	0.45	1	t	1	Cocconeis sp.	Hydora nitida larvae	56	0.02	0.03	0.01	0.03
1	0	0.40	1	t	1	Cocconeis sp.	Aoteaphsyche sp.	56	0.02	0.03	0.00	0.03
1	1	0.76	1	t	1	Cocconeis sp.	Aphrophila noevaezelandiae	56	0.02	0.03	0.04	0.03
1	1	0.70	1	cv	1	Cocconeis sp.	Eukiefiriella	56	0.02	0.03	0.03	0.03
1	1	0.75	1	t	1	Mougeotia sp.	Aphrophila noevaezelandiae	56	0.02	0.03	0.04	0.03
1	0	0.37	1	t	1	Stictocladius	Unidentified detritus	56	0.01	0.03	0.02	0.03
1	0	0.41	1	t	1	Stictocladius	Gomphonema subclavatum	56	0.01	0.03	0.02	0.03
1	1	0.58	1	cv	1	Stictocladius	Gomphonema cf. olivaceum	56	0.01	0.03	0.03	0.03
1	0	0.32	1	t	1	Stictocladius	Eunotia sp.	56	0.01	0.03	0.01	0.03
1	1	0.51	1	t	1	Stictocladius	Nitzschia sp. (large)	56	0.01	0.03	0.03	0.03
1	0	0.42	1	t	1	Stictocladius	Gomphonema truncatum	56	0.01	0.03	0.02	0.03
0	0	0.28	1	cv	1	Stictocladius	Diploneis sp.	56	0.01	0.03	0.01	0.03
1	0	0.48	1	t	1	Stictocladius	Oedogonium	56	0.01	0.03	0.03	0.03

0	0	0.30	1	t	1	Stictocladius	Gomphoneis herculeana	56	0.01	0.03	0.01	0.03
1	1	0.54	1	t	1	Stictocladius	Navicula avenacea	56	0.01	0.03	0.03	0.03
1	0	0.36	1	cv	1	Oxyethira albiceps	Achnanthes saxonica	56	0.02	0.03	0.00	0.03
1	1	0.52	1	t	1	Oxyethira albiceps	Gomphonema subclavatum	56	0.02	0.03	0.02	0.03
1	1	0.69	1	t	1	Oxyethira albiceps	Gomphonema cf. olivaceum	56	0.02	0.03	0.03	0.03
1	0	0.43	1	t	1	Oxyethira albiceps	Eunotia sp.	56	0.02	0.03	0.01	0.03
1	1	0.74	1	cv	1	Oxyethira albiceps	Cymbella minuta	56	0.02	0.03	0.04	0.03
1	1	0.63	1	t	1	Oxyethira albiceps	Diatoma heimale	56	0.02	0.03	0.03	0.03
1	0	0.39	1	t	1	Oeconesus sp.	Microspora sp.	56	0.02	0.03	0.00	0.03
1	1	0.54	1	t	1	Oeconesus sp.	Unidentified detritus	56	0.02	0.03	0.02	0.03
1	0	0.48	1	cv	1	Oeconesus sp.	Eunotia sp.	56	0.02	0.03	0.01	0.03
1	1	0.65	1	t	1	Oeconesus sp.	Oedogonium	56	0.02	0.03	0.03	0.03
0	0	0.34	1	t	1	Nitzschia sp. (small)	Amphipoda spp.	56	0.02	0.03	0.00	0.03
1	1	0.60	1	t	1	Nitzschia sp. (small)	Austroclima jollyae	56	0.02	0.03	0.03	0.03
1	0	0.41	1	cv	1	Nitzschia sp. (small)	Hydora nitida larvae	56	0.02	0.03	0.01	0.03
1	1	0.53	1	t	1	Nitzschia sp. (small)	Coloburiscus humeralis	56	0.02	0.03	0.02	0.03
1	0	0.42	1	t	1	Nitzschia sp. (small)	Deleatidium spp.	56	0.02	0.03	0.01	0.03
1	1	0.58	1	t	1	Nitzschia sp. (small)	Austrosimulium australense	56	0.02	0.03	0.02	0.03
1	0	0.36	1	cv	1	Nitzschia sp. (small)	Aoteaphsyche sp.	56	0.02	0.03	0.00	0.03
0	0	0.36	1	t	1	Nitzschia sp. (small)	Unknown orthoclad	56	0.02	0.03	0.00	0.03
1	1	0.62	1	t	1	Nitzschia sp. (small)	Tanytarsini	56	0.02	0.03	0.03	0.03
1	1	0.73	1	t	1	Nitzschia sp. (small)	Aphrophila noevaezelandiae	56	0.02	0.03	0.04	0.03
1	1	0.66	1	cv	1	Nitzschia sp. (small)	Eukiefiriella	56	0.02	0.03	0.03	0.03
1	1	0.64	1	t	1	Frustulia rhomboides	Amphipoda spp.	56	0.05	0.03	0.00	0.03

1	1	0.84	1	t	1	Frustulia rhomboides	Austroclima jollyae	56	0.05	0.03	0.03	0.03
1	1	0.84	1	t	1	Frustulia rhomboides	Potamopurgus antipodarum	56	0.05	0.03	0.03	0.03
1	1	0.70	1	cv	1	Frustulia rhomboides	Hydora nitida larvae	56	0.05	0.03	0.01	0.03
1	1	0.80	1	t	1	Frustulia rhomboides	Coloburiscus humeralis	56	0.05	0.03	0.02	0.03
1	1	0.72	1	t	1	Frustulia rhomboides	Deleatidium spp.	56	0.05	0.03	0.01	0.03
1	1	0.83	1	t	1	Frustulia rhomboides	Austrosimulium australense	56	0.05	0.03	0.02	0.03
1	1	0.66	1	cv	1	Frustulia rhomboides	Aoteaphsyche sp.	56	0.05	0.03	0.00	0.03
1	1	0.64	1	t	1	Frustulia rhomboides	Acroperla sp.	56	0.05	0.03	0.00	0.03
1	1	0.85	1	t	1	Frustulia rhomboides	Tanytarsini	56	0.05	0.03	0.03	0.03
1	1	0.90	1	t	1	Frustulia rhomboides	Aphrophila noevaezelandiae	56	0.05	0.03	0.04	0.03
1	1	0.87	1	cv	1	Frustulia rhomboides	Eukiefiriella	56	0.05	0.03	0.03	0.03
1	0	0.42	1	t	1	Microspora sp.	Austroclima jollyae	56	0.00	0.03	0.03	0.03
1	0	0.35	1	t	1	Microspora sp.	Coloburiscus humeralis	56	0.00	0.03	0.02	0.03
0	0	0.26	1	t	1	Microspora sp.	Deleatidium spp.	56	0.00	0.03	0.01	0.03
1	0	0.50	1	cv	1	Microspora sp.	Paranephrops zealandicus	56	0.00	0.03	0.03	0.03
1	0	0.28	1	t	1	Microspora sp.	Pycnocentria sp.	56	0.00	0.03	0.01	0.03
0	0	0.21	1	t	1	Microspora sp.	Aoteaphsyche sp.	56	0.00	0.03	0.00	0.03
0	0	0.20	1	t	1	Microspora sp.	Acroperla sp.	56	0.00	0.03	0.00	0.03
1	0	0.30	1	cv	1	Unidentified detritus	Amphipoda spp.	56	0.02	0.03	0.00	0.03
1	1	0.56	1	t	1	Unidentified detritus	Austroclima jollyae	56	0.02	0.03	0.03	0.03
1	0	0.48	1	t	1	Unidentified detritus	Podaena sp.	56	0.02	0.03	0.02	0.03
1	1	0.57	1	t	1	Unidentified detritus	Potamopurgus antipodarum	56	0.02	0.03	0.03	0.03
1	1	0.63	1	cv	1	Unidentified detritus	Naonella	56	0.02	0.03	0.03	0.03
1	0	0.37	1	t	1	Unidentified detritus	Hydora nitida larvae	56	0.02	0.03	0.01	0.03

1	0	0.46	1	t	1	Unidentified detritus	Stenoperla prasinia	56	0.02	0.03	0.02	0.03
1	1	0.53	1	t	1	Unidentified detritus	Paralimnophora skusei	56	0.02	0.03	0.02	0.03
1	0	0.49	1	cv	1	Unidentified detritus	Coloburiscus humeralis	56	0.02	0.03	0.02	0.03
1	0	0.38	1	t	1	Unidentified detritus	Deleatidium spp.	56	0.02	0.03	0.01	0.03
1	1	0.63	1	t	1	Unidentified detritus	Paranephrops zealandicus	56	0.02	0.03	0.03	0.03
1	0	0.48	1	t	1	Unidentified detritus	Hexatomini sp.	56	0.02	0.03	0.02	0.03
1	1	0.54	1	cv	1	Unidentified detritus	Austrosimulium australense	56	0.02	0.03	0.02	0.03
1	0	0.40	1	t	1	Unidentified detritus	Pycnocentria sp.	56	0.02	0.03	0.01	0.03
1	0	0.32	1	t	1	Unidentified detritus	Aoteaphsyche sp.	56	0.02	0.03	0.00	0.03
0	0	0.32	1	t	1	Unidentified detritus	Unknown orthoclad	56	0.02	0.03	0.00	0.03
1	1	0.51	1	cv	1	Unidentified detritus	Zealandobius confusus type	56	0.02	0.03	0.02	0.02
1	0	0.30	1	t	1	Unidentified detritus	Acroperla sp.	56	0.02	0.03	0.00	0.03
1	0	0.34	1	t	1	Unidentified detritus	Scirtid sp. (Skinny type)	56	0.02	0.03	0.01	0.03
1	1	0.58	1	t	1	Unidentified detritus	Tanytarsini	56	0.02	0.03	0.03	0.03
0	0	0.33	1	cv	1	Unidentified detritus	Hydrobiosella sp.	56	0.02	0.03	0.00	0.03
1	1	0.69	1	t	1	Unidentified detritus	Aphrophila noevaezelandiae	56	0.02	0.03	0.04	0.03
1	1	0.62	1	t	1	Unidentified detritus	Eukiefiriella	56	0.02	0.03	0.03	0.03
1	0	0.40	1	t	1	Unidentified detritus	Costachorema xanthoptera	56	0.02	0.03	0.01	0.03
1	0	0.44	1	cv	1	Unidentified detritus	Psilachorema bidens	56	0.02	0.03	0.02	0.03
0	0	0.32	1	t	1	Unidentified detritus	Megaleptoperla grandis	56	0.02	0.03	0.00	0.03
1	1	0.62	1	t	1	Melosira varians	Austroclima jollyae	56	0.02	0.03	0.03	0.03
0	0	0.26	1	t	1	Achnanthes saxonica	Hydora nitida larvae	56	0.00	0.03	0.01	0.03
0	0	0.28	1	cv	1	Achnanthes saxonica	Deleatidium spp.	56	0.00	0.03	0.01	0.03

1	0	0.41	1	t	1	Achnanthes saxonica	Austrosimulium australense	56	0.00	0.03	0.02	0.03
0	0	0.29	1	t	1	Achnanthes saxonica	Pycnocentria sp.	56	0.00	0.03	0.01	0.03
1	1	0.58	1	t	1	Achnanthes saxonica	Aphrophila noevaezelandiae	56	0.00	0.03	0.04	0.03
1	1	0.59	1	cv	1	Gomphonema subclavatum	Austroclima jollyae	56	0.02	0.03	0.03	0.03
1	0	0.41	1	t	1	Gomphonema subclavatum	Deleatidium spp.	56	0.02	0.03	0.01	0.03
1	1	0.57	1	t	1	Gomphonema subclavatum	Austrosimulium australense	56	0.02	0.03	0.02	0.03
1	0	0.43	1	t	1	Gomphonema subclavatum	Pycnocentria sp.	56	0.02	0.03	0.01	0.03
1	0	0.35	1	cv	1	Gomphonema subclavatum	Aoteaphsyche sp.	56	0.02	0.03	0.00	0.03
1	1	0.72	1	t	1	Gomphonema subclavatum	Aphrophila noevaezelandiae	56	0.02	0.03	0.04	0.03
1	1	0.71	1	t	1	Navicula cf. radiosa	Austroclima jollyae	56	0.03	0.03	0.03	0.03
1	1	0.69	1	t	1	Navicula cf. radiosa	Austrosimulium australense	56	0.03	0.03	0.02	0.03
1	1	0.51	1	cv	1	Amphipoda spp.	Gomphonema cf. olivaceum	56	0.00	0.03	0.03	0.03
0	0	0.26	1	t	1	Amphipoda	Eunotia sp.	56	0.00	0.03	0.01	0.03
1	1	0.56	1	t	1	Amphipoda	Synedra ulna	56	0.00	0.03	0.04	0.03
1	0	0.47	1	t	1	Amphipoda spp.	Navicula avenacea	56	0.00	0.03	0.03	0.03
1	1	0.74	1	cv	1	Gomphonema cf. olivaceum	Austroclima jollyae	56	0.03	0.03	0.03	0.03
1	1	0.79	1	t	1	Gomphonema cf. olivaceum	Naonella	56	0.03	0.03	0.03	0.03
1	1	0.56	1	t	1	Gomphonema cf. olivaceum	Hydora nitida larvae	56	0.03	0.03	0.01	0.03
1	1	0.68	1	t	1	Gomphonema cf. olivaceum	Coloburiscus humeralis	56	0.03	0.03	0.02	0.03
1	1	0.58	1	cv	1	Gomphonema cf. olivaceum	Deleatidium spp.	56	0.03	0.03	0.01	0.03
1	1	0.79	1	t	1	Gomphonema cf. olivaceum	Paranephrops zealandicus	56	0.03	0.03	0.03	0.03
1	1	0.72	1	t	1	Gomphonema cf. olivaceum	Austrosimulium australense	56	0.03	0.03	0.02	0.03
1	1	0.60	1	t	1	Gomphonema cf. olivaceum	Pycnocentria sp.	56	0.03	0.03	0.01	0.03

1	1	0.51	1	cv	1	Gomphonema cf. olivaceum	Aoteaphsyche sp.	56	0.03	0.03	0.00	0.03
1	0	0.49	1	t	1	Gomphonema cf. olivaceum	Acroperla sp.	56	0.03	0.03	0.00	0.03
1	1	0.75	1	t	1	Gomphonema cf. olivaceum	Tanytarsini	56	0.03	0.03	0.03	0.03
1	1	0.83	1	t	1	Gomphonema cf. olivaceum	Aphrophila noevaezelandiae	56	0.03	0.03	0.04	0.03
1	1	0.79	1	cv	1	Gomphonema cf. olivaceum	Eukiefiriella	56	0.03	0.03	0.03	0.03
1	0	0.49	1	t	1	Austroclima jollyae	Eunotia sp.	56	0.03	0.03	0.01	0.03
1	1	0.58	1	t	1	Austroclima jollyae	Navicula cryptocephala	56	0.03	0.03	0.02	0.03
1	1	0.68	1	t	1	Austroclima jollyae	Nitzschia sp. (large)	56	0.03	0.03	0.03	0.03
1	1	0.70	1	cv	1	Austroclima jollyae	Cymbella kappii	56	0.03	0.03	0.03	0.03
1	1	0.79	1	t	1	Austroclima jollyae	Cymbella minuta	56	0.03	0.03	0.04	0.03
1	1	0.60	1	t	1	Austroclima jollyae	Gomphonema truncatum	56	0.03	0.03	0.02	0.03
1	1	0.78	1	t	1	Austroclima jollyae	Synedra ulna	56	0.03	0.03	0.04	0.03
1	1	0.71	1	cv	1	Austroclima jollyae	Navicula avenacea	56	0.03	0.03	0.03	0.03
1	1	0.55	1	t	1	Austroclima jollyae	Epithemia sorex	56	0.03	0.03	0.02	0.03
1	1	0.69	1	t	1	Austroclima jollyae	Diatoma heimale	56	0.03	0.03	0.03	0.03
1	1	0.61	1	t	1	Podaena sp.	Nitzschia sp. (large)	56	0.02	0.03	0.03	0.03
1	1	0.63	1	cv	1	Podaena sp.	Cymbella kappii	56	0.02	0.03	0.03	0.03
1	1	0.73	1	t	1	Podaena sp.	Cymbella minuta	56	0.02	0.03	0.04	0.03
1	1	0.52	1	t	1	Podaena sp.	Gomphonema truncatum	56	0.02	0.03	0.02	0.03
1	1	0.64	1	t	1	Podaena sp.	Navicula avenacea	56	0.02	0.03	0.03	0.03
1	1	0.62	1	cv	1	Podaena sp.	Diatoma heimale	56	0.02	0.03	0.03	0.03
1	1	0.69	1	t	1	Potamopurgus antipodarum	Nitzschia sp. (large)	56	0.03	0.03	0.03	0.03
1	1	0.71	1	t	1	Potamopurgus antipodarum	Cymbella kappii	56	0.03	0.03	0.03	0.03
1	1	0.80	1	t	1	Potamopurgus antipodarum	Cymbella minuta	56	0.03	0.03	0.04	0.03

1	1	0.72	1	cv	1	Potamopurgus antipodarum	Navicula avenacea	56	0.03	0.03	0.03	0.03
1	1	0.70	1	t	1	Potamopurgus antipodarum	Diatoma heimale	56	0.03	0.03	0.03	0.03
1	1	0.56	1	t	1	Naonella	Eunotia sp.	56	0.03	0.03	0.01	0.03
1	1	0.65	1	t	1	Naonella	Navicula cryptocephal a	56	0.03	0.03	0.02	0.03
1	1	0.74	1	cv	1	Naonella	Nitzschia sp. (large)	56	0.03	0.03	0.03	0.03
1	1	0.82	1	t	1	Naonella	Synedra ulna	56	0.03	0.03	0.04	0.03
1	1	0.54	1	t	1	Naonella	Gomphoneis herculeana	56	0.03	0.03	0.01	0.03
1	1	0.58	1	t	1	Naonella	Costachorem a xanthoptera	56	0.03	0.03	0.01	0.03
1	1	0.62	1	cv	1	Naonella	Psilachorema bidens	56	0.03	0.03	0.02	0.03
1	0	0.32	1	t	1	Hydora nitida larvae	Eunotia sp.	56	0.01	0.03	0.01	0.03
1	1	0.64	1	t	1	Hydora nitida larvae	Cymbella minuta	56	0.01	0.03	0.04	0.03
1	0	0.42	1	t	1	Hydora nitida larvae	Gomphonem a truncatum	56	0.01	0.03	0.02	0.03
0	0	0.24	1	cv	1	Hydora nitida larvae	Audouinella	56	0.01	0.03	0.00	0.03
1	1	0.54	1	t	1	Hydora nitida larvae	Navicula avenacea	56	0.01	0.03	0.03	0.03
1	1	0.52	1	t	1	Hydora nitida larvae	Diatoma heimale	56	0.01	0.03	0.03	0.03
1	0	0.43	1	t	1	Eunotia sp.	Coloburiscus humeralis	56	0.01	0.03	0.02	0.03
1	0	0.33	1	cv	1	Eunotia sp.	Deleatidium spp.	56	0.01	0.03	0.01	0.03
1	0	0.48	1	t	1	Eunotia sp.	Austrosimuliu m australense	56	0.01	0.03	0.02	0.03
1	0	0.35	1	t	1	Eunotia sp.	Pycnocentria sp.	56	0.01	0.03	0.01	0.03
0	0	0.27	1	t	1	Eunotia sp.	Aoteaphsych e sp.	56	0.01	0.03	0.00	0.03
0	0	0.26	1	cv	1	Eunotia sp.	Acroperla sp.	56	0.01	0.03	0.00	0.03
1	1	0.52	1	t	1	Eunotia sp.	Tanytarsini	56	0.01	0.03	0.03	0.03
1	1	0.64	1	t	1	Eunotia sp.	Aphrophila noevaezeland iae	56	0.01	0.03	0.04	0.03
1	1	0.57	1	t	1	Eunotia sp.	Eukiefiriella	56	0.01	0.03	0.03	0.03
1	1	0.69	1	cv	1	Paralimnopho ra skusei	Navicula avenacea	56	0.02	0.03	0.03	0.03
1	1	0.67	1	t	1	Paralimnopho ra skusei	Diatoma heimale	56	0.02	0.03	0.03	0.03

1	1	0.52	1	t	1	Navicula cryptocephala	Coloburiscus humeralis	56	0.02	0.03	0.02	0.03
1	0	0.35	1	t	1	Navicula cryptocephala	Aoteaphsyche sp.	56	0.02	0.03	0.00	0.03
1	1	0.72	1	cv	1	Navicula cryptocephala	Aphrophila noevaezelandiae	56	0.02	0.03	0.04	0.03
1	1	0.51	1	t	1	Nitzschia sp. (large)	Deleatidium spp.	56	0.03	0.03	0.01	0.03
1	1	0.74	1	t	1	Nitzschia sp. (large)	Paranephrops zealandicus	56	0.03	0.03	0.03	0.03
1	1	0.66	1	t	1	Nitzschia sp. (large)	Austrosimulium australense	56	0.03	0.03	0.02	0.03
1	1	0.70	1	cv	1	Nitzschia sp. (large)	Tanytarsini	56	0.03	0.03	0.03	0.03
1	1	0.79	1	t	1	Nitzschia sp. (large)	Aphrophila noevaezelandiae	56	0.03	0.03	0.04	0.03
1	1	0.74	1	t	1	Nitzschia sp. (large)	Eukiefiriella	56	0.03	0.03	0.03	0.03
1	1	0.64	1	t	1	Cymbella kappii	Coloburiscus humeralis	56	0.03	0.03	0.02	0.03
1	1	0.53	1	cv	1	Cymbella kappii	Deleatidium spp.	56	0.03	0.03	0.01	0.03
1	1	0.68	1	t	1	Cymbella kappii	Austrosimulium australense	56	0.03	0.03	0.02	0.03
1	0	0.46	1	t	1	Cymbella kappii	Aoteaphsyche sp.	56	0.03	0.03	0.00	0.03
1	1	0.75	1	t	1	Cymbella	Eukiefiriella	56	0.03	0.03	0.03	0.03
1	1	0.74	1	cv	1	Coloburiscus humeralis	Cymbella minuta	56	0.02	0.03	0.04	0.03
1	0	0.41	1	t	1	Coloburiscus humeralis	Gomphoneis herculeana	56	0.02	0.03	0.01	0.03
1	1	0.65	1	t	1	Coloburiscus humeralis	Navicula avenacea	56	0.02	0.03	0.03	0.03
1	1	0.63	1	t	1	Coloburiscus humeralis	Diatoma heimale	56	0.02	0.03	0.03	0.03
1	1	0.64	1	cv	1	Cymbella minuta	Deleatidium spp.	56	0.04	0.03	0.01	0.03
1	1	0.77	1	t	1	Cymbella minuta	Austrosimulium australense	56	0.04	0.03	0.02	0.03
1	1	0.66	1	t	1	Cymbella minuta	Pycnocentria sp.	56	0.04	0.03	0.01	0.03
1	1	0.57	1	t	1	Cymbella minuta	Aoteaphsyche sp.	56	0.04	0.03	0.00	0.03
1	1	0.55	1	cv	1	Cymbella minuta	Acroperla sp.	56	0.04	0.03	0.00	0.03

1	1	0.87	1	t	1	Cymbella minuta	Aphrophila noevaezelandiae	56	0.04	0.03	0.04	0.03
1	1	0.82	1	t	1	Cymbella minuta	Eukiefiriella	56	0.04	0.03	0.03	0.03
1	0	0.43	1	t	1	Gomphonema truncatum	Deleatidium spp.	56	0.02	0.03	0.01	0.03
1	1	0.58	1	cv	1	Gomphonema truncatum	Austrosimulium australense	56	0.02	0.03	0.02	0.03
1	0	0.44	1	t	1	Gomphonema truncatum	Pycnocentria sp.	56	0.02	0.03	0.01	0.03
1	0	0.34	1	t	1	Gomphonema truncatum	Acroperla sp.	56	0.02	0.03	0.00	0.03
1	1	0.66	1	t	1	Gomphonema truncatum	Eukiefiriella	56	0.02	0.03	0.03	0.03
1	1	0.57	1	cv	1	Deleatidium spp.	Paranephrops zealandicus	56	0.01	0.03	0.03	0.03
1	0	0.27	1	t	1	Deleatidium spp.	Aoteaphsyche sp.	56	0.01	0.03	0.00	0.03
0	0	0.25	1	t	1	Deleatidium spp.	Audouinella	56	0.01	0.03	0.00	0.03
1	1	0.56	1	t	1	Deleatidium spp.	Navicula avenacea	56	0.01	0.03	0.03	0.03
1	0	0.34	1	cv	1	Deleatidium spp.	Costachorema xanthoptera	56	0.01	0.03	0.01	0.03
1	0	0.38	1	t	1	Deleatidium spp.	Psilachorema bidens	56	0.01	0.03	0.02	0.03
1	1	0.53	1	t	1	Deleatidium spp.	Diatoma heimale	56	0.01	0.03	0.03	0.03
1	1	0.64	1	t	1	Hexatomini sp.	Navicula avenacea	56	0.02	0.03	0.03	0.03
1	1	0.62	1	cv	1	Hexatomini sp.	Diatoma heimale	56	0.02	0.03	0.03	0.03
1	1	0.76	1	t	1	Austrosimulium australense	Synedra ulna	56	0.02	0.03	0.04	0.03
1	1	0.69	1	t	1	Austrosimulium australense	Navicula avenacea	56	0.02	0.03	0.03	0.03
1	1	0.53	1	t	1	Austrosimulium australense	Epithemia sorex	56	0.02	0.03	0.02	0.03
1	0	0.49	1	cv	1	Austrosimulium australense	Costachorema xanthoptera	56	0.02	0.03	0.01	0.03
1	1	0.53	1	t	1	Austrosimulium australense	Psilachorema bidens	56	0.02	0.03	0.02	0.03
1	1	0.67	1	t	1	Austrosimulium australense	Diatoma heimale	56	0.02	0.03	0.03	0.03
1	1	0.55	1	t	1	Synedra ulna	Aoteaphsyche sp.	56	0.04	0.03	0.00	0.03
1	1	0.53	1	cv	1	Synedra ulna	Acroperla sp.	56	0.04	0.03	0.00	0.03

1	1	0.57	1	t	1	Synedra ulna	Scirtid sp. (Skinny type)	56	0.04	0.03	0.01	0.03
1	1	0.79	1	t	1	Synedra ulna	Tanytarsini	56	0.04	0.03	0.03	0.03
1	1	0.86	1	t	1	Synedra ulna	Aphrophila noevaezelandiae	56	0.04	0.03	0.04	0.03
1	0	0.40	1	cv	1	Pycnocentria sp.	Epithemia sorex	56	0.01	0.03	0.02	0.03
1	0	0.40	1	t	1	Pycnocentria sp.	Psilachorema bidens	56	0.01	0.03	0.02	0.03
1	0	0.49	1	t	1	Aoteaphsyche sp.	Navicula avenacea	56	0.00	0.03	0.03	0.03
1	0	0.32	1	t	1	Aoteaphsyche sp.	Epithemia sorex	56	0.00	0.03	0.02	0.03
0	0	0.21	1	cv	1	Aoteaphsyche sp.	Epithemia zebra	56	0.00	0.03	0.00	0.03
1	1	0.56	1	t	1	Audouinella	Aphrophila noevaezelandiae	56	0.00	0.03	0.04	0.03
1	0	0.47	1	t	1	Acroperla sp.	Navicula avenacea	56	0.00	0.03	0.03	0.03
1	1	0.71	1	t	1	Tanytarsini	Diatoma heimale	56	0.03	0.03	0.03	0.03
1	1	0.62	1	cv	1	Gomphoneis herculeana	Aphrophila noevaezelandiae	56	0.01	0.03	0.04	0.03
1	1	0.81	1	t	1	Aphrophila noevaezelandiae	Navicula avenacea	56	0.04	0.03	0.03	0.03
1	1	0.79	1	t	1	Aphrophila noevaezelandiae	Diatoma heimale	56	0.04	0.03	0.03	0.03
1	1	0.76	1	t	1	Navicula avenacea	Eukiefiriella	56	0.03	0.03	0.03	0.03
1	1	0.74	1	cv	1	Eukiefiriella	Diatoma heimale	56	0.03	0.03	0.03	0.03
1	0	0.36	0	t	1	Coloburiscus humeralis	Hydrobiosella sp.	56	0.02	0.03	0.00	0.03
1	1	0.60	0	t	1	Cocconeis sp.	Zealandobius confusus type	56	0.02	0.03	0.02	0.02
1	1	0.60	0	t	1	Navicula cf. radiosa	Gomphonema acuminatum	56	0.03	0.03	0.02	0.03
1	0	0.45	0	cv	1	Oligochaeta type I	Hydora nitida larvae	56	0.02	0.03	0.01	0.03
1	0	0.40	0	t	1	Stictocladius	Podaena sp.	56	0.01	0.03	0.02	0.03
1	0	0.33	0	t	1	Nesameletus ornatus	Deleatidium spp.	56	0.01	0.03	0.01	0.03
1	1	0.60	0	t	1	Cocconeis sp.	Melosira varians	56	0.02	0.03	0.02	0.03

1	0	0.39	0	cv	1	Unknown orthoclad	Zealandobius confusus type	56	0.00	0.03	0.02	0.02
0	0	0.24	0	t	1	Meridion circulare	Gomphonema parvulum	56	0.01	0.03	0.00	0.03
1	1	0.60	0	t	1	Achnanthes minutissima	Costachorema xanthoptera	56	0.03	0.03	0.01	0.03
1	0	0.30	0	t	1	Stictocladius	Hydora nitida larvae	56	0.01	0.03	0.01	0.03
0	0	0.35	0	cv	1	Navicula sp.	Pinnularia (small - mesolepta??)	56	0.01	0.03	0.02	0.03
1	1	0.68	0	t	1	Larsia II	Navicula avenacea	56	0.02	0.02	0.03	0.03
1	1	0.60	0	t	1	moss cells	Cymbella minuta	56	0.00	0.03	0.04	0.03
1	1	0.61	0	t	1	Megaleptoperla diminuta	Tanyderid sp.	56	0.01	0.02	0.03	0.03
1	1	0.53	0	cv	1	Cricotopus I	Zealandobius confusus type	56	0.02	0.03	0.02	0.02
1	1	0.62	0	t	1	Hudsonema amabilis	Podaena sp.	56	0.03	0.03	0.02	0.03
1	1	0.64	0	t	1	Deleatidium spp.	Synedra ulna	56	0.01	0.03	0.04	0.03
1	0	0.38	0	t	1	Orchymontia sp.	Unidentified detritus	56	0.01	0.03	0.02	0.03
0	1	0.51	0	cv	1	Melosira varians	Gomphonema acuminatum	56	0.02	0.03	0.02	0.03
0	0	0.34	0	t	1	Meridion circulare	Gomphonema acuminatum	56	0.01	0.03	0.02	0.03
0	0	0.39	0	t	1	Closterium sp.	Nitzschia sp. (small)	56	0.01	0.03	0.02	0.03
1	1	0.55	0	t	1	Oeconesus sp.	Stenoperla prasinia	56	0.02	0.03	0.02	0.03
1	1	0.63	0	cv	1	Stauroneis sp.	Oeconesus sp.	56	0.02	0.03	0.02	0.03
1	1	0.70	0	t	1	Oeconesus sp.	Navicula cf. radiosa	56	0.02	0.03	0.03	0.03
1	0	0.41	0	t	1	Acroperla sp.	Oedogonium	56	0.00	0.03	0.03	0.03
1	1	0.77	0	t	1	Maoridiemesea	Paranephrops zealandicus	56	0.03	0.03	0.03	0.03
1	1	0.54	0	cv	1	Cymbella aspera	Zealandobius confusus type	56	0.02	0.03	0.02	0.02
1	1	0.70	0	t	1	Achnanthes lanceoloata	Fragilaria vaucheriae	56	0.03	0.03	0.03	0.03

1	0	0.34	0	t	1	Cricotopus I	Stratiomyiid sp.	56	0.02	0.03	0.00	0.03
1	1	0.59	0	t	1	Potamopurgus antipodarum	Hexatomini sp.	56	0.03	0.03	0.02	0.03
1	0	0.48	0	cv	1	Stictocladius	Austroclima jollyae	56	0.01	0.03	0.03	0.03
1	0	0.47	0	t	1	Surirella (small)	Hydora nitida larvae	56	0.03	0.03	0.01	0.03
1	1	0.66	0	t	1	Podaena sp.	Paranephrops zealandicus	56	0.02	0.03	0.03	0.03
1	0	0.42	0	t	1	Stratiomyiid sp.	Navicula rhynchocephala	56	0.00	0.03	0.03	0.03
1	1	0.52	0	cv	1	Paralimnophora skusei	Psilachorema bidens	56	0.02	0.03	0.02	0.03
1	0	0.39	0	t	1	Larsia II	Gomphonema parvulum	56	0.02	0.02	0.00	0.03
0	0	0.33	0	t	1	Closterium sp.	Achnanthes linearis	56	0.01	0.03	0.01	0.03
1	1	0.56	0	t	1	Tanyderid sp.	Costachorema xanthoptera	56	0.03	0.03	0.01	0.03
1	0	0.40	0	cv	1	Ceratopogonid sp.	Acroperla sp.	56	0.03	0.03	0.00	0.03
1	0	0.37	0	t	1	Hydora nitida larvae	Psilachorema bidens	56	0.01	0.03	0.02	0.03
0	0	0.32	0	t	1	Achnanthes linearis	Navicula sp.	56	0.01	0.03	0.01	0.03
1	1	0.80	0	t	1	Cymbella minuta	Tanytarsini	56	0.04	0.03	0.03	0.03
1	1	0.51	0	cv	1	Achnanthes linearis	Mougeotia sp.	56	0.01	0.03	0.02	0.03
0	0	0.29	0	t	1	Achnanthes linearis	Surirella tenera ??	56	0.01	0.03	0.00	0.03
1	0	0.45	0	t	1	Eunotia sp.	Zealandobius confusus type	56	0.01	0.03	0.02	0.02
1	1	0.81	0	t	1	Nitzschia sp. (large)	Cymbella minuta	56	0.03	0.03	0.04	0.03
1	0	0.46	0	cv	1	Eunotia sp. large	Achnanthes linearis	56	0.02	0.03	0.01	0.03
0	0	0.37	0	t	1	Gomphonema parvulum	Nitzschia sp. (small)	56	0.00	0.03	0.02	0.03
0	0	0.25	0	t	1	cf. Batrachospermum??	Stictocladius	56	0.00	0.03	0.01	0.03
1	0	0.39	0	t	1	Oeconesus sp.	Audouinella	56	0.02	0.03	0.00	0.03
1	0	0.32	0	cv	1	Cricotopus I	Amphipoda spp.	56	0.02	0.03	0.00	0.03
0	0	0.45	0	t	1	Closterium sp.	Surirella (small)	56	0.01	0.03	0.03	0.03

1	0	0.39	0	t	1	Mougeotia sp.	Achnanthes saxonica	56	0.02	0.03	0.00	0.03
1	0	0.49	0	t	1	Nitzschia sp. (small)	Unidentified detritus	56	0.02	0.03	0.02	0.03
1	0	0.49	0	cv	1	Surirella (small)	Orchymontia sp.	56	0.03	0.03	0.01	0.03
0	1	0.65	0	t	1	Frustulia rhomboides	Epithemia zebra	56	0.05	0.03	0.00	0.03
1	0	0.33	0	t	1	Austroperla cyrene	Achnanthes saxonica	56	0.02	0.03	0.00	0.03
1	0	0.41	0	t	1	Fragilaria spp.	Ceratopogonid sp.	56	0.00	0.03	0.03	0.03
1	1	0.82	0	cv	1	Paranephrops zealandicus	Synedra ulna	56	0.03	0.03	0.04	0.03
1	0	0.41	0	t	1	Cymbella aspera	Orchymontia sp.	56	0.02	0.03	0.01	0.03
1	0	0.46	0	t	1	Achnanthes linearis	Gomphonema subclavatum	56	0.01	0.03	0.02	0.03
0	0	0.49	0	t	1	Synedra rumpens	Melosira varians	56	0.01	0.03	0.02	0.03
1	0	0.47	0	cv	1	Oligochaeta type I	Nesameletus ornatus	56	0.02	0.03	0.01	0.03
1	1	0.62	0	t	1	Larsia II	Oeconesus sp.	56	0.02	0.02	0.02	0.03
1	1	0.65	0	t	1	Hexatomini	Eukiefiriella	56	0.02	0.03	0.03	0.03
1	1	0.65	0	t	1	Tanyderid sp.	Gomphonema truncatum	56	0.03	0.03	0.02	0.03
0	1	0.51	0	cv	1	Unknown green algal	Achnanthes minutissima	56	0.00	0.03	0.03	0.03
1	1	0.63	0	t	1	Achnanthes lanceoloata	Stauroneis sp.	56	0.03	0.03	0.02	0.03
0	0	0.32	0	t	1	Megaleptoperla diminuta	Megaleptoperla grandis	56	0.01	0.02	0.00	0.03
1	1	0.74	0	t	1	Hudsonema amabilis	Navicula cf. radiosa	56	0.03	0.03	0.03	0.03
1	0	0.49	0	cv	1	Austroclima jollyae	Deleatidium spp.	56	0.03	0.03	0.01	0.03
1	1	0.74	0	t	1	Fragilaria vaucheriae	Navicula cf. radiosa	56	0.03	0.03	0.03	0.03
1	0	0.36	0	t	1	Salmo trutta	Nitzschia sp. (small)	56	0.00	0.03	0.02	0.03
1	1	0.71	0	t	1	Surirella	Tanyderid sp.	56	0.03	0.03	0.03	0.03
0	0	0.32	0	cv	1	Gomphonema parvulum	Cymbella cuspidata	56	0.00	0.03	0.02	0.03
1	1	0.66	0	t	1	Eunotia sp. large	Eukiefiriella	56	0.02	0.03	0.03	0.03
1	1	0.60	0	t	1	Polyplectropus puerilis	Navicula avenacea	56	0.01	0.03	0.03	0.03
0	0	0.24	0	t	1	Gomphonema parvulum	Scirtid sp. (Skinny type)	56	0.00	0.03	0.01	0.03

0	0	0.21	0	cv	1	moss cells	Fragilaria spp.	56	0.00	0.03	0.00	0.03
1	0	0.33	0	t	1	Cymbella aspera	Amphipoda spp.	56	0.02	0.03	0.00	0.03
0	0	0.22	0	t	1	Hydrobiosis parumbripenn	Gomphonema parvulum	56	0.00	0.03	0.00	0.03
1	1	0.77	0	t	1	Naonella	Eukiefiriella	56	0.03	0.03	0.03	0.03
1	0	0.46	0	cv	1	moss cells	Tanytarsini	56	0.00	0.03	0.03	0.03
1	0	0.35	0	t	1	Synedra rumpens	Nesameletus ornatus	56	0.01	0.03	0.01	0.03
1	0	0.36	0	t	1	Achnanthes saxonica	Hexatomini sp.	56	0.00	0.03	0.02	0.03
0	0	0.39	0	t	1	Tabellaria flocculosa	Gomphonema subclavatum	56	0.01	0.03	0.02	0.03
0	0	0.23	0	cv	1	Aoteaphsyche sp.	Megaleptoperla grandis	56	0.00	0.03	0.00	0.03
1	1	0.50	0	t	1	Oniscogaster sp.	Salmo trutta	56	0.04	0.03	0.00	0.03
0	0	0.28	0	t	1	Gomphonema parvulum	Eunotia sp.	56	0.00	0.03	0.01	0.03
1	0	0.48	0	t	1	Unidentified detritus	Gomphonema subclavatum	56	0.02	0.03	0.02	0.03
1	1	0.52	0	cv	1	Salmo trutta	Gomphonema cf. olivaceum	56	0.00	0.03	0.03	0.03
0	0	0.25	0	t	1	Closterium sp.	Cricotopus II	56	0.01	0.03	0.01	0.03
[truncated: 386,156 more chars]
